# Minor Spliceosomal 65K/RNPC3 Interacts with ANKRD11 and Mediates HDAC3‐Regulated Histone Deacetylation and Transcription

**DOI:** 10.1002/advs.202307804

**Published:** 2024-06-05

**Authors:** Chen‐Hui Li, Shao‐Bo Liang, Qi‐Wei Huang, Zhen‐Zhen Zhou, Zhan Ding, Ni Long, Kwang‐Chon Wi, Liang Li, Xi‐Ping Jiang, Yu‐Jie Fan, Yong‐Zhen Xu

**Affiliations:** ^1^ RNA Institute State Key Laboratory of Virology Hubei Key Laboratory of Cell Homeostasis College of Life Science TaiKang Center for Life and Medical Sciences Wuhan University Hubei 430072 China; ^2^ Key Laboratory of Insect Developmental and Evolutionary Biology Center for Excellence in Molecular Plant Sciences Chinese Academy of Sciences University of Chinese Academy of Sciences Shanghai 200032 China

**Keywords:** ANKRD11, HDAC3, histone deacetylation, minor splicing, U11/U12‐65K

## Abstract

RNA splicing is crucial in the multilayer regulatory networks for gene expression, making functional interactions with DNA‐ and other RNA‐processing machineries in the nucleus. However, these established couplings are all major spliceosome‐related; whether the minor spliceosome is involved remains unclear. Here, through affinity purification using *Drosophila* lysates, an interaction is identified between the minor spliceosomal 65K/RNPC3 and ANKRD11, a cofactor of histone deacetylase 3 (HDAC3). Using a CRISPR/Cas9 system, Deletion strains are constructed and found that both *Dm65K^Δ/Δ^* and *Dmankrd11^Δ/Δ^* mutants have reduced histone deacetylation at Lys9 of histone H3 (H3K9) and Lys5 of histone H4 (H4K5) in their heads, exhibiting various neural‐related defects. The 65K‐ANKRD11 interaction is also conserved in human cells, and the HsANKRD11 middle‐uncharacterized domain mediates Hs65K association with HDAC3. Cleavage under targets and tagmentation (CUT&Tag) assays revealed that HsANKRD11 is a bridging factor, which facilitates the synergistic common chromatin‐binding of HDAC3 and Hs65K. Knockdown (KD) of *HsANKRD11* simultaneously decreased their common binding, resulting in reduced deacetylation of nearby H3K9. Ultimately, this study demonstrates that expression changes of many genes caused by *HsANKRD11‐KD* are due to the decreased common chromatin‐binding of HDAC3 and Hs65K and subsequently reduced deacetylation of H3K9, illustrating a novel and conserved coupling mechanism that links the histone deacetylation with minor spliceosome for the regulation of gene expression.

## Graphical abstract

The interaction between ANKRD11 and the minor spliceosome‐specific protein 65K/RNPC3 enhances the chromatin‐binding of HDAC3 to further facilitate the deacetylation of histones, especially on H3K9 and H4K5. Caused by the deficiency of 65K or ANKRD11, the defective 65K‐ANKRD11 interaction weakens the chromatin‐binding of HDAC3 and decreases the deacetylation activity, resulting in changed gene expression. This regulatory mechanism is conserved in both *Drosophila* and human cells.

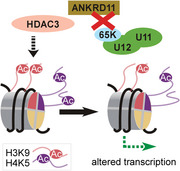

## 1 Introduction

Catalyzed by the spliceosome, pre‐mRNA splicing removes intronic sequences from precursor RNAs, which is critical for gene expression and regulation.^[^ ^1^ ^]^ The spliceosome is a macromolecular RNA‐protein machinery, consisting of five small nuclear RNAs (snRNAs) and dozens of proteins, forming dynamic complexes during the multiple stages of assembly, catalysis, and disassembly.^[^ ^2^ ^]^ Two distinct spliceosomes, the major and the minor spliceosomes, coexist in most metazoans and a few unicellular organisms.^[^ ^3^ ^]^ The major spliceosome recognizes and removes >99.5% of introns, named U2‐type or major introns; while the minor spliceosome catalyzes fewer introns, named U12‐type or minor introns.^[^ ^3^, ^4^ ^]^ However, the function and regulation of the minor spliceosome and minor‐intron‐containing genes (MIGs) are critical; mutations in the minor spliceosome or minor introns cause serious developmental defects and human diseases.^[^ ^5^ ^]^

In the composition of snRNAs, the two spliceosomes only share U5; the major has U1, U2, U4, and U6, and the minor has U11, U12, U4atac, and U6actac snRNAs.^[^ ^6^ ^]^ The two spliceosomes share many protein components but also have their own specific ones.^[^ ^3^ ^]^ For example, the human minor spliceosomal U11/U12 di‐small nuclear ribonucleoproteins (di‐snRNP) subcomplex does not have U1‐70K, U1‐A, or U1C from the U1 snRNP, or U2‐A', U2‐B', or SF3a subunits from the U2 snRNP, but has the complete SF3b complex like the major spliceosomal U2 snRNP. Instead, the U11/U12 di‐snRNP has 7 specific proteins, including 65K/RNPC3, 59K/PDCD7, 48K/SNRNP48, 35K/SNRNP35, 31K/ZCRB1, 25K/SNRNP25, and 20K/ZMAT5.^[^ ^7^ ^]^ In the human minor spliceosomal B^act^ complex, five minor‐specific proteins have been recently identified, including SCNM1, RBM48, ARMC7, PPIL2, and CRIPT.^[^ ^8^ ^]^ Most of the minor‐specific proteins have been identified in plants, fruit fly, zebrafish, and mouse, displaying a high evolutionary conservation across species; and their mutations cause deficient splicing of minor introns and aberrant alternative splicing (AS).^[^ ^9^ ^]^

Eukaryotic RNA processing includes 5′‐capping, splicing, 3′‐end polyadenylation, editing, and modifications on transcripts, which are carried out co‐transcriptionally in the nucleus.^[^ ^10^ ^]^ Chromatin remodeling, DNA and histone modifications, transcription, and RNA processing are also coupled with each other and form regulatory networks for accurate gene expression.^[^ ^11^ ^]^ RNA splicing is a crucial step in eukaryotes; many major spliceosome components have been found to interact with DNA‐ and RNA‐processing machineries. For example, the U2AF65‐Prp19 complex interacts with the C‐terminal domain of RNA polymerase II (CTD of RNAPII) to activate splicing;^[^ ^12^ ^]^ the Prp5‐U2 snRNP complex is recruited by the transcriptional Spt‐Ada‐Gcn5 acetyltransferase (SAGA) complex to modulate splicing fidelity^[^ ^13^ ^]^; and the assembly of the U2 snRNP, the splicing scaffold U5 snRNP and PRPF8 is facilitated by Ser5‐phosphorylated RNAPII.^[^ ^14^ ^]^ The chromatin remodeling factor CHD1 co‐purifies the U2 snRNP subcomplex SF3a, and the deficiency of CHD1 results in a decreased splicing rate due to impaired SF3a recruitment^[^ ^15^ ^]^; the chromatin remodeling factor BRG1 interacts with hnRNPL, hnRNPU, and SAM68 to modulate alternative splicing^[^ ^16^ ^]^; the chromatin‐binding protein HP1α/β and the splicing factors SRp20 and ASF/SF2 are interacting partners for trimethylations of H3K9^[^ ^17^ ^]^; and the DNA‐binding protein UHRF1 regulates alternative splicing by interacting with SF3B3 and U1/U2 snRNAs in an H3R2me‐involved manner.^[^ ^18^ ^]^ However, minor‐spliceosome‐coupled events and their regulatory functions in gene expression have not yet been investigated.

Methylation and acetylation are the two dominant histone post‐translational modifications,^[^ ^19^ ^]^ in which histone acetylation surrounding the transcription start sites (TSSs) stabilizes the binding of other chromatin remodeling factors and destabilizes nucleosome structure, leading to decreased nucleosome occupancy and enhanced transcription.^[^ ^20^ ^]^ Histone deacetylases (HDACs) are a group of enzymes that remove acetyl groups from histone lysines, allowing histones to package the chromatin more tightly, and thus silencing transcription.^[^ ^21^ ^]^ Class I HDACs (Rpd3‐like proteins) consists of four enzymes, HDAC1, HDAC2, HDAC3, and HDAC8, which are recruited to enhancers and promoters to modulate the epigenetics of chromatin and nearby gene expression.^[^ ^22^ ^]^ Defective HDAC3 results in increased levels of H3K9 acetylation (H3K9ac), H3K14 acetylation (H3K14ac), H4K5 acetylation (H4K5ac), and H4K12 acetylation (H4K12ac) in the late S phase of the cell cycle.^[^ ^23^ ^]^ Interacting with HDAC3, the ANKRD11 protein contains multiple regions called ankyrin domains and functions as a co‐factor of HDAC3, which shows specificity in brain nerve cells and may regulate genes involved in learning and memory.^[^ ^24^ ^]^

To identify minor‐spliceosome‐coupled events, we co‐purified Dm65K‐associated proteins from *Drosophila* and found that Dm65K interacts with DmANKRD11. Deletion of either *Dm65K* or *DmAnkrd11* leads to reduced deacetylation of H3K9 and H4K5 in the fly head. The 65K‐ANKRD11 interaction is conserved in human, and knockdown of the human *Hs65K* or *HsANKRD11* also increased H3K9ac and H4K5ac. Co‐immunoprecipitation (co‐IP) experiments revealed that HsANKRD11 mediates the Hs65K‐association with HDAC3, and CUT&Tag assays revealed that Hs65K and HDAC3 have largely overlapping chromatin binding and synergistically enhance each other's binding, which was weakened in the *HsANKRD11* knockdown cells. Furthermore, this 65K‐ANKRD11‐HDAC3 interaction influences the deacetylation of H3K9 surrounding TSS regions and gene expression, providing a novel regulatory strategy for gene expression through an interaction between machineries of histone modification and minor splicing.

## 2 Results

### 2.1 Identification of Dm65K‐Associated Proteins

**Figure 1:**
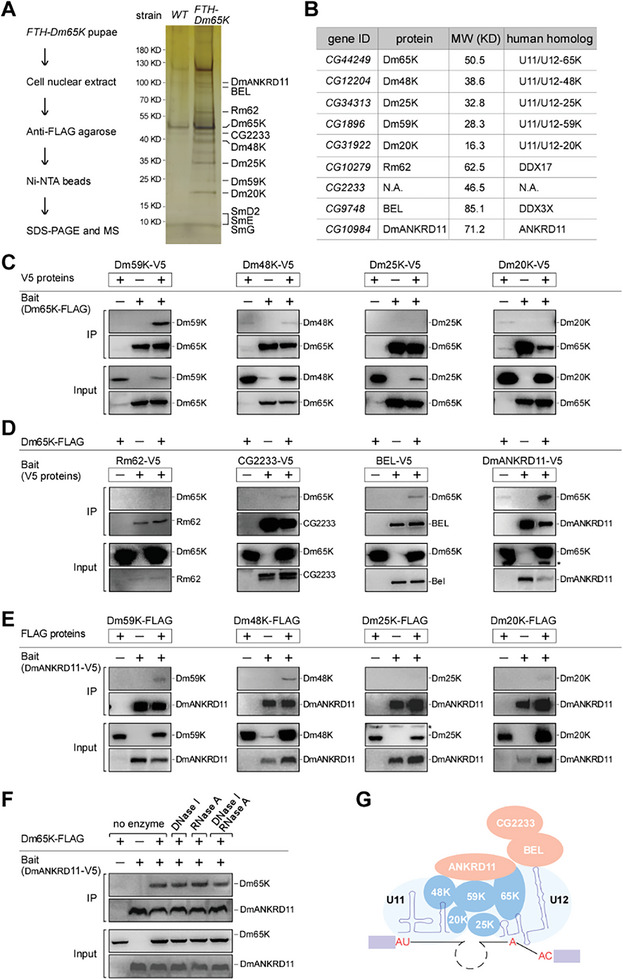
Dm65K interacts with the HDAC3‐cofactor DmAnkrd11 in *Drosophila*. A) The Dm65K‐associated proteins were co‐purified from the lysate of the FTH‐Dm65K pupae through a two‐step affinity purification and identified by mass spectrometry. Left, strategy for purification; right, silver staining of SDS‐polyacrylamide gel electrophoresis (SDS‐PAGE) gel for the co‐purified proteins. B) Information of the top identified proteins. C) FLAG‐tagged Dm65K effectively co‐IPed Dm59K, but not other three minor spliceosomal proteins. D) FLAG‐tagged Dm65K was effectively co‐IPed by DmANKRD11, but not by other three identified non‐minor spliceosomal proteins. E) Dm59K and Dm48K were ineffectively co‐IPed by DmANKRD11. F) The Dm65K‐DmANKRD11 interaction is DNA‐ and RNA‐independent in S2 cells. DNase I and/or RNase A were added during the co‐IPs. G) A proposed interaction network based on data from this figure and Figure S3 (Supporting Information). Asterisks, non‐specific bands.

Since only two minor spliceosome‐specific components, 65K/RNPC3 and 20K/ZMAT5, were experimentally confirmed in *Drosophila*,^[^ ^9^, ^25^ ^]^ we carried out a two‐step affinity purification using nuclear extract from the pupae of the FLAG‐Tev‐6×His (FTH)‐tagged Dm65K strain (**Figure** **1**A left). The top identified peptides of the associated proteins are from 11 *Drosophila* genes, including *Dm20K*, *Rm62*, *Bel, CG12204*, *CG34313*, *CG1896*, *CG2233*, and *CG10984*, as well as three Sm genes *SmD2*, *SmG*, and *SmE* (Figure 1A,B; Table S1, Supporting Information). Using *clustalw*,^[^ ^26^ ^]^ we identified CG12204, CG34313, and CG1896 as the homologs of human U11/U12‐48K; −25, and −59K, respectively; and Rm62, BEL, and CG10984 as homologs to human DDX17, DDX3X, and ANKRD11, respectively; *CG2233* is still an uncharacterized gene with unknown homologs (Figure 1B; Figure S1, Supporting Information).

Two sets of assays were then performed to distinguish the functions of the co‐purified proteins. First, their coding sequences with a FLAG‐tag were expressed in *Drosophila* S2 cells. Similar to Dm65K, the FLAG‐tagged Dm59K, Dm48K, Dm25K, and Dm20K co‐IPed U11 and U12 snRNAs, as well as pre‐mRNAs with minor introns, but neither the major U1 snRNA nor pre‐mRNAs with major introns (Figure S2, Supporting Information). In contrast, the FLAG‐tagged Rm62, CG2233, BEL, and DmANKRD11 co‐IPed none of the minor RNAs. Secondly, the splicing of minor introns was tested when those genes were knocked down (KD) in S2 cells. As with the *Dm65K‐KD*, KDs of *Dm59K, Dm48K*, *Dm25K*, and *Dm20K* resulted in notable retention of minor introns, whereas KDs of the other four genes did not inhibit minor splicing (Figure S2C,D, Supporting Information). These data demonstrate that *CG12204*, *CG34313*, and *CG1896* are homologs of the human U11/U12 di‐snRNP components in *Drosophila*, and thus we designate them as *Dm48K*, *Dm25K*, and *Dm59K*, respectively, while the other four proteins are not directly involved in minor splicing.

### 2.2 Dm65K Interacts with the Histone Deacetylation Cofactor DmANKRD11

To investigate protein‐protein interactions, we performed co‐IP assays using two separately expressed FLAG‐ and V5‐tagged proteins in S2 cells. First, Dm65K efficiently co‐IPed Dm59K, but not Dm48K, Dm25K, or Dm20K (Figure 1C); Dm59K efficiently co‐IPed Dm48K and Dm20K, and slightly Dm25K; Dm48K did not co‐IP either Dm25K or Dm20K, and Dm25K did not co‐IP Dm20K (Figure S3A–C, Supporting Information). These results suggest that the interactions of Dm65K‐Dm59K, Dm59K‐Dm48K, and Dm59K‐Dm20K are direct or at least strong. These interactions are independent of RNA, demonstrated by the observation of similar interactions remaining after treatment with Ribonuclease A (RNase A) (Figure S3A–D, Supporting Information). Second, Dm65K was efficiently co‐IPed by DmANKRD11, slightly by BEL and CG2233, and not detectably by Rm62 (Figure 1D), implying a strong Dm65K‐DmANKRD11 interaction. In contrast, DmANKRD11 slightly co‐IPed Dm59K and Dm48K but not others (Figure 1E), suggesting that interactions between DmANKRD11 with the minor spliceosome are mostly via Dm65K. The Dm65K‐DmANKRD11 interaction is also independent of both DNA and RNA (Figure 1F).

In summary, these results suggest a protein‐protein interaction network of the co‐purified proteins (Figure 1G). As the core U11/U12 di‐snRNP protein, Dm65K directly interacts with Dm59K and a non‐spliceosomal protein DmANKRD11, whose homolog in mammals was identified as a cofactor of the histone deacetylation enzyme HDAC3.^[^ ^24a^ ^]^

### 2.3 Histone Deacetylation is Reduced in the Head of *65k^Δ/Δ^* and *ankrd11^Δ/Δ^* Mutants

**Figure 2:**
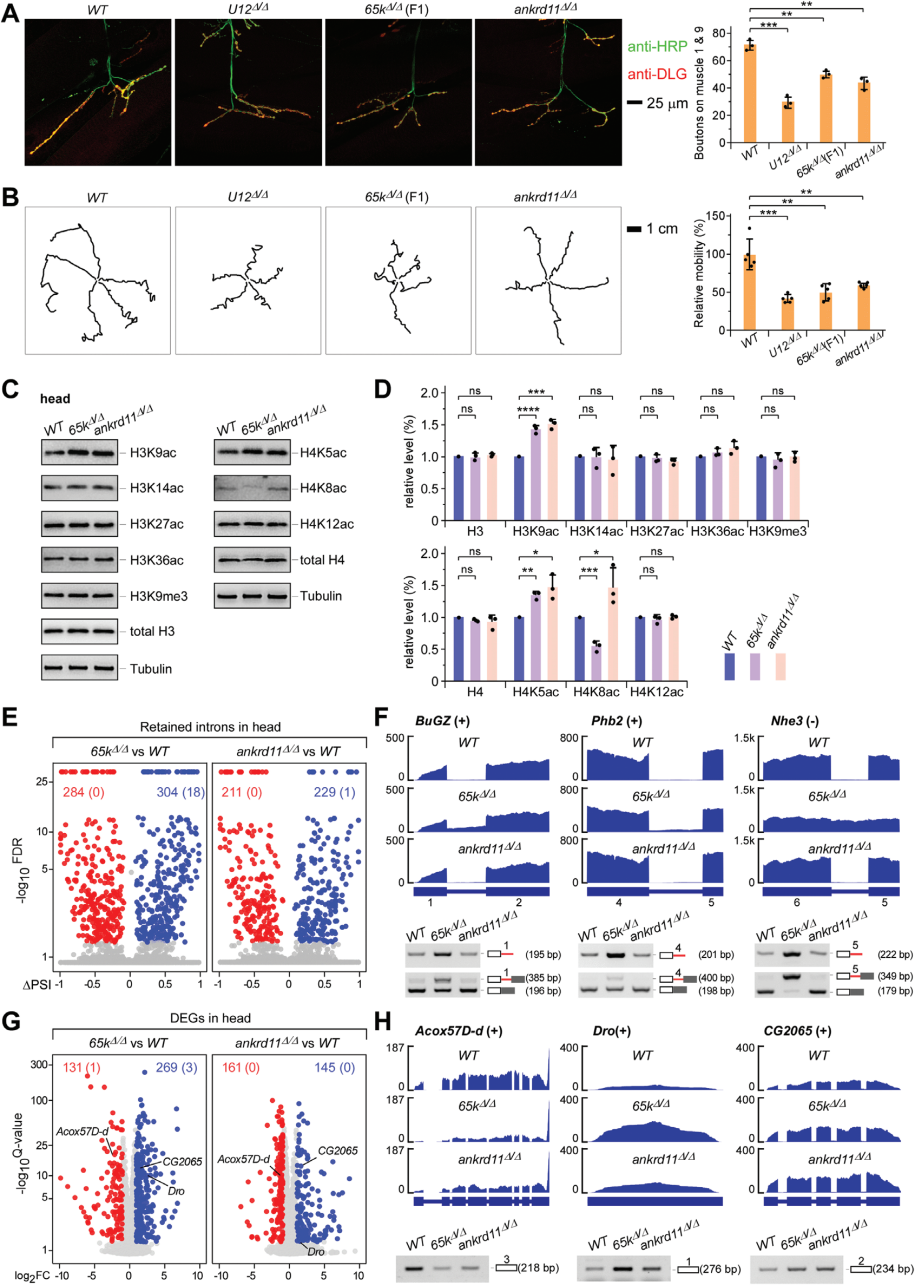
Both *65k^Δ/Δ^* and *ankrd11^Δ/Δ^* mutants exhibit enhanced levels of H3K9ac and H4K5ac in the head. A) The NMJ boutons are reduced in the two deletion strains, but less than the reduction in the U12‐deletion strain. Quantitation of the NMJ boutons is shown on the right. B) The larva locomotion is impaired in the two deletion strains. Quantitation of the relative mobility are shown on the right. C) H3K9ac and H4K5ac were enhanced in the head of the two deletion strains. D) Quantitative analyses of the changed levels of histone acetylations. E) Retained intron events in the head of the two deletion strains. Numbers in brackets are counting of minor introns. F) Validation of inhibited splicing of three minor introns in the two deletion mutants by RT‐PCR. Red lines, minor introns. G) Analyses of differentially expressed genes (DEGs) in the head of the two mutants. Numbers in brackets are counting of MIGs. H) Validation of three DEGs in the two deletion mutants by RT‐PCR. The length of PCR amplicons is indicated. Statistical data are shown as mean ± SD. p values were calculated using two‐sided unpaired t‐test; ^*^ *p* <0.05; ^**^ *p* <0.01; ^***^ *p* <0.001; ^****^ *p* <0.0001; ns, no significance.

Using the CRISPR/Cas9‐mediated system,^[^ ^27^ ^]^ we generated two deletion flies, *65k^Δ/Δ,^* and *ankrd11^Δ/Δ^* (Figure S4A,B, Supporting Information). Unlike the pupa‐stage lethality caused by the deletion of *U12* or *U6atac* snRNA,^[^ ^9d^ ^]^ the first generation of *65k^Δ/Δ^* (F1) was viable to mate and generate offspring; however, it exhibited significant defects in pupation, eclosion, and fecundity (Figure S4C–E, Supporting Information). The second generation of *65k^Δ/Δ^* (F2) was even more impaired, exhibiting more serious defects and no ability to cross (Figure S4C–F, Supporting Information). These results demonstrate that *Dm65K* is a critical gene for the survival and development of *Drosophila*. Deletion of *Ankrd11* resulted in relatively milder developmental defects (Figure S4C–F, Supporting Information). Importantly, both the *65k^Δ/Δ^* and *ankrd11^Δ/Δ^* flies showed milder spinal muscular atrophy (SMA) associated phenotypes^[^ ^9d^ ^]^ than the *U12^Δ/Δ^* did, fewer boutons of the neuromuscular junctions (NMJ) and less impaired larvae locomotion (**Figure** **2**A,B), suggesting that the two mutants have defects in the nerve systems.

Since the function of DmANKRD11 has not been experimentally investigated, we detected histone acetylation levels in mutants, including H3K9ac, H3K14ac, H3K27ac, H3K36ac, H4K5ac, K4K8ac, and H4K12ac that are substrates of HDAC3.^[^ ^24^, ^28^ ^]^ Compared to *WT* flies, neither *65k^Δ/Δ^* nor *ankrd11^Δ/Δ^* adults showed obvious changes in histone acetylation (Figure S5A, Supporting Information). Interestingly, H3K9ac and H4K5ac were increased in the heads of the two mutants, while other sites were not, except that H4K8ac was decreased in *65k^Δ/Δ^* and increased in *ankrd11^Δ/Δ^* (Figure 2C,D). These results demonstrate that histone deacetylation is reduced in the *Drosophila* brain when *Dm65K* or *DmANKRD11* is knocked out, and the data are consistent with findings in mammals that ANKRD11 controls histone acetylation during neural development.^[^ ^24a^ ^]^

We performed RNA‐sequencing (RNA‐seq) and identified more changed AS events in *65k^Δ/Δ^* than in *ankrd11^Δ/Δ^* (Figure S5B,C and Table S2, Supporting Information). Nearly all 19 *Drosophila* minor introns were retained in *65k^Δ/Δ^* (18 in head, 17 in adult), but only one was observed in *ankrd11^Δ/Δ^* (Figure 2E; Figure S5D, Supporting Information). Defective minor splicing in *65k^Δ/Δ^* was further validated by RT‐PCR, showing significantly increased retention of minor introns and decreased mRNAs from *BuGZ*, *Phb2*, and *Nhe3* genes (Figure 2F). Analyses of the differentially‐expressed genes (DEGs) showed that 269 and 145 genes were up‐regulated and 131 and 161 genes were down‐regulated in the head of *65k^Δ/Δ^* and *ankrd11^Δ/Δ^*, respectively (Figure 2G; Figure S5E and Table S2, Supporting Information). Including many neurodevelopment‐related genes, 71 DEGs were shared by the two mutants (Figure S5F, Supporting Information). For example, the *Acox57D‐d* was down‐regulated and *Drosocin* (*Dro*) was up‐regulated in both mutants (Figure 2G,H). Acox57D‐d is an acyl‐CoA oxidase involved in peroxisomal β‐oxidation, and disorders of peroxisome biogenesis result in neuronal dysfunction, muscle weakness, and locomotion problems.^[^ ^29^ ^]^ *Dro* is an antimicrobial peptide gene, *Dro*‐overexpression in *Drosophila* neurons leads to impaired locomotor activity.^[^ ^30^ ^]^ Since *65k^Δ/Δ^* and *ankrd11^Δ/Δ^* mutants showed defective NMJ and locomotion (Figure 2A,B), we performed RT‐PCR and confirmed that the expression of *Acox57D‐d* is down‐regulated and *Dro* is up‐regulated in the heads of both mutants (Figure 2H). Up‐regulation of *CG2065*, an NADP‐retinol dehydrogenase,^[^ ^31^ ^]^ was also validated by RT‐PCR. In addition, the splicing changes caused by the deletion of *Dm‐ankrd11* would be due to indirect effects. One of the possibilities could be the transcriptional‐coupled splicing, which has been extensively reported.^[^ ^10^, ^11^, ^32^ ^]^

### 2.4 The 65K‐ANKRD11 Interaction is Conserved in Human

**Figure 3:**
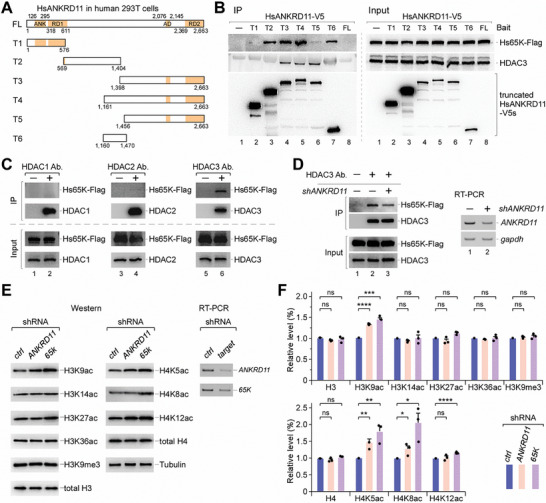
The 65K‐ANKRD11 interaction is conserved in human. A) Schematics for HsANKRD11‐truncated proteins that were expressed with V5‐tag in human 293T cells. Borders and domains are indicated. ANK, ankyrin repeat domain; RD, transcription repression domain; AD, transcription activation domain. B) Co‐IPs by the truncated HsANKRD11s indicated that T6, covers aa 1160 to 1470 in the long uncharacterized region, is sufficient for interaction with Hs65K. C) Hs65K was co‐IPed by an HDAC3 antibody, but not by an HDAC1 or HDAC2 antibody. D) Knockdown of *HsANKRD11* reduced the Hs65K association with HDAC3. E) H3K9ac, H4K5ac, and H4K8ac were enhanced in both *Hs65K‐KD* and *HsANKRD11‐KD* cells. KDs were performed using shRNAs, and the empty vector was used as a control. F) Quantitation of the histone acetylation levels in *Hs65K‐KD* and *HsANKRD11‐KD* cells. Statistical data are shown as mean ± SD. p values were calculated using two‐sided unpaired t‐test; ^*^ *p* <0.05; ^**^ *p* <0.01; ^***^ *p* <0.001; ^****^ *p* <0.0001; ns, no significance.

Human ANKRD11 (HsANKRD11) has 2663 aa with four identified domains^[^ ^33^ ^]^ and a long stretch of uncharacterized middle region (**Figure** **3**A). Lacking an obvious AD domain, the DmANKRD11 has three other domains and a much shorter middle region (Figure S1F, Supporting Information). To address the 65K‐ANKRD11 interaction in human, we cloned the full‐length (FL) and six truncations (T1‐T6) of HsANKRD11s and transfected them into 293T cells (Figure 3A). The FL protein was not detectably expressed due to its large size. Consistent with a previous report,^[^ ^34^ ^]^ the truncated HsANKRD11 with the C‐terminus (T3, T4, and T5) co‐IPed HDAC3, while other truncated HsANKRD11s did not (Figure 3B). Importantly, the truncated HsANKRD11 with the middle region (T2, T3, T4, and T6) co‐IPed different levels of Hs65K, in which T6, covering a shorter middle region (aa 1160‐1470), was sufficiently to co‐IP Hs65K. Taken together, we conclude that the 65K‐ANKRD11 interaction is also conserved in human, which relies on the stretched middle region of ANKRD11.

Using antibodies against HDACs, we found that Hs65K could be co‐IPed by HDAC3, but not by HDAC1 or HDAC2, the other two Class I HDAC enzymes (Figure 3C). Furthermore, fewer Hs65K was co‐IPed by HDAC3 when *HsANKRD11* was knocked down by shRNA (Figure 3D), but T6 and T2 still co‐IPed similar amounts of Hs65K when *HDAC3* was knocked down (Figure S6A, Supporting Information). These results suggest that HsANKRD11 mediates the Hs65K association with HDAC3. Importantly, H3K9ac and H4K5ac in 293T cells were also increased when *Hs65K* or *HsANKRD11* was knocked down (Figure 3E,F). Similarly, as in the two deletion flies, other histone acetylation sites were not changed except H4K8ac. The same changes of histone acetylation caused by deficiency of 65K and ANKRD11 in both human cells and *Drosophila* suggest a conserved regulatory manner for histone deacetylation, which is mediated by the 65K‐ANKRD11 interaction.

To address whether the 65K‐ANKRD11‐HDAC3 interaction is involved with other epigenetic factors, we performed additional co‐immunoprecipitations using an antibody against Hs65K. We found that Hs65K did not co‐IP SRC1, TIF2, or RAC3, three key components from the p160 coactivators/nuclear receptor complex, which has interaction with ANKRD11 (Figure S6B, Supporting Information).^[^ ^35^ ^]^ However, Hs65K strongly co‐IPed NOCR1, a component from the nuclear receptor corepressor & silencing mediator of retinoid and thyroid receptors complex, which has interaction with HDAC3 (Figure S6C, Supporting Information).^[^ ^36^ ^]^

### 2.5 ANKRD11 Mediates the Synergistic Chromatin Binding of 65K and HDAC3

**Figure 4:**
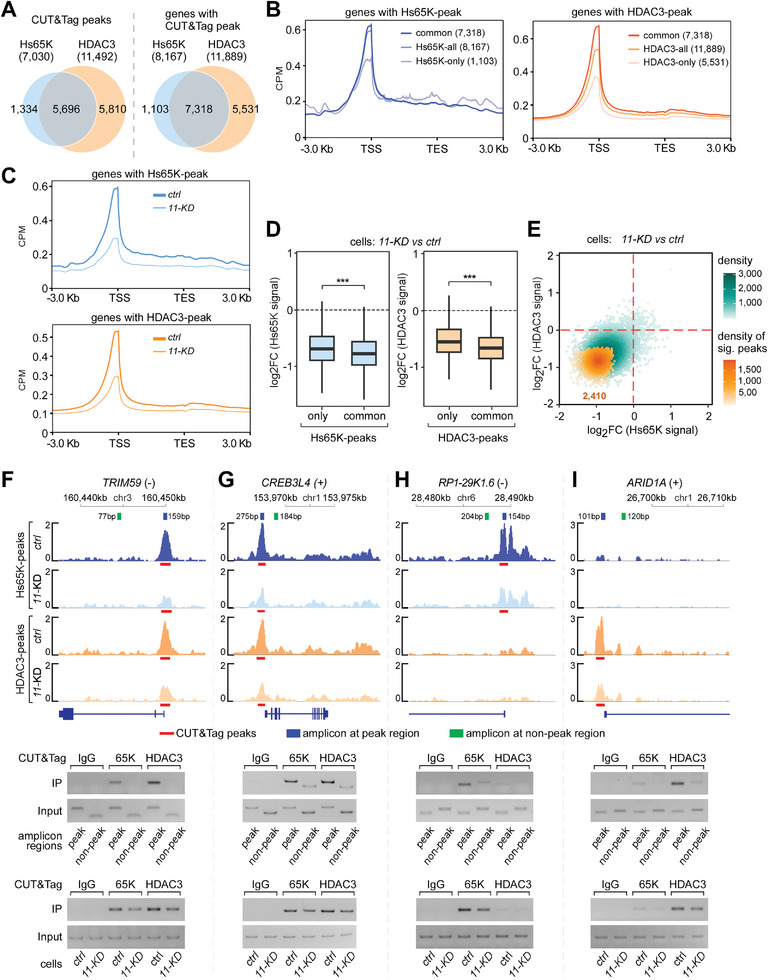
Hs65K and HDAC3 synergistically promote each other's chromatin binding. A) Analyses of chromatin‐binding sites of Hs65K and HDAC3 in 293T cells using CUT&Tag data. The majority of Hs65K‐binding sites overlap with HDAC3‐binding sites from the view of both peaks (left) and genes with peaks (right). B) Density distribution of Hs65K‐ and HDAC3‐binding signals on gene bodies. Peak‐containing genes are grouped as common‐, all‐, and only‐binding. TSS, transcription start site; TES, transcription end site. C) The chromatin‐bindings near the TSS region of Hs65K and HDAC3 were significantly decreased when *HsANKRD11* was knocked down by shRNA. *11‐KD*, *HsANKRD11‐KD*; *ctrl*, *control*. D) Peak signals of Hs65K and HDAC3 at the common‐binding sites were more seriously decreased than at the only‐binding sites in the *HsANKRD11‐KD* cells. p values were calculated using the Wilcoxon test; ^***^, p value < = 0.001. E) A large portion of the 65K‐ and HDAC3‐signals at the common‐binding sites were simultaneously decreased in the *HsANKRD11‐KD* cells. F–I) Experimental validation of the CUT&Tag analyses for chromatin‐binding of Hs65K and HDAC3 by individual PCRs. Four genes were selected for testing, including two common‐binding genes F) *TRIM59* and G) *CREB3L4*, H) an Hs65K‐only binding gene *RP1‐29K1.6*, and I) an HDAC3‐only binding gene *ARID1A*. The location of protein‐binding peaks and PCR amplification regions for peaks and non‐peaks are indicated.

To address whether Hs65K binds on chromatin along with HsANKRD11 and HDAC3, we carried out CUT&Tag assays^[^ ^37^ ^]^ to identify their chromatin binding sites. Antibodies against Hs65K and HDAC3 efficiently co‐IPed chromatin DNAs; unfortunately, the HsANKRD11‐antibody failed after multiple trials, showing no detectable DNA signal, similar to the IgG control (Figure S6D, Supporting Information). In total, we obtained 7030 and 11492 chromatin‐binding peaks of Hs65K and HDAC3, respectively. Their common peaks belong to 7318 genes, occupying ∼90% of the Hs65K‐binding genes and ∼62% of the HDAC3‐binding genes (**Figure** **4**A; Table S3, Supporting Information), indicating that most chromatin‐binding of Hs65K are along with HDAC3. Density analyses revealed that both the Hs65K‐ and HDAC3‐binding peaks were enriched surrounding TSSs (Figure 4B). Interestingly, Hs65K‐ and HDAC3‐peaks from the common‐binding genes were significantly higher, and peaks from the Hs65K‐only or HDAC3‐only binding genes were significantly lower (Figure 4B). These results suggest that Hs65K and HDAC3 promote each other's binding on chromatin, showing a synergistic enhancement effect.

Furthermore, the chromatin‐binding of Hs65K and HDAC3 near the TSS region were both dramatically decreased in the *HsANKRD11‐KD* (*11‐KD*) cells (Figure 4C; Figure S6E, Supporting Information), in which signals from the common‐binding were more decreased than signals from their respective only‐binding (Figure 4D). Individually, both Hs65K‐ and HDAC3‐signals from ∼90% of the common‐binding were simultaneously decreased in the *11‐KD* cells, in which 2410 were significantly dropped (Figure 4E), demonstrating that ANKRD11 is a bridging factor for maintaining the synergistic chromatin‐binding of Hs65K and HDAC3. In addition, the knockdown of HDAC3 also significantly decreases the chromatin‐binding of Hs65K, suggesting that the binding of Hs65K is also dependent on HDAC3 (Figure S6E, right, Supporting Information).

To validate, we inspected four genes by PCRs after co‐IPs. Consistent with the above analyses, Hs65K and HDAC3 efficiently co‐IPed DNAs from the peak regions but not or significantly less from the non‐peak regions of the common‐binding genes *TRIM59* and *CREB3L4* (Figure 4F,G, upper gels). Similarly, Hs65K co‐IPed the peak region DNA from the Hs65K‐only binding gene *RP1‐29K1.6* and HDAC3 co‐IPed the peak region DNA from the HDAC3‐only binding gene *ARID1A* (Figure 4H,I, upper gels). Importantly, those co‐IPed signals were decreased in the *11‐KD* cells (Figure 4F–I, lower gels). These results demonstrate that the 65K‐ANKRD11 interaction promotes Hs65K‐binding on chromatin along with HDAC3 and synergistically enhances HDAC3‐binding at the same chromatin domains.

### 2.6 65K‐ANKRD11 Interaction Modulates H3K9 Acetylation and Gene Expression

**Figure 5:**
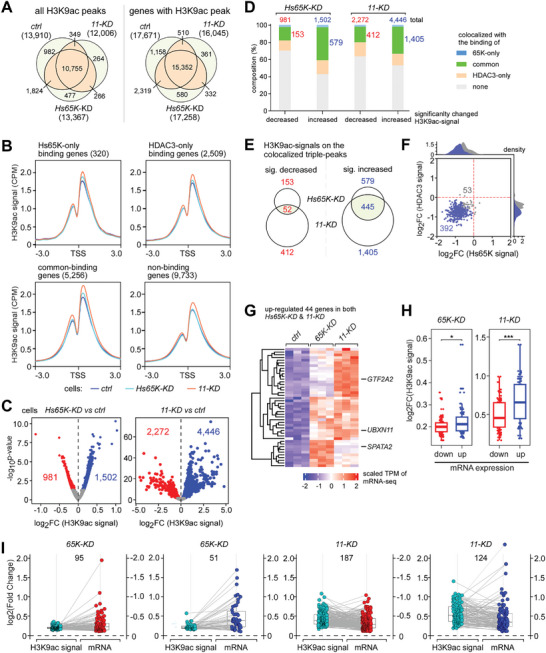
The 65K‐ANKRD11 interaction modulates H3K9 acetylation and gene expression. A) Statistics of H3K9ac ChIP‐peaks and peak‐containing genes in three human cells. The overlapping between the three cells is analyzed. *11‐KD*, *HsANKRD11‐KD*; *ctrl*, *control*. B) Density distribution of H3K9ac‐signals surrounding TSSs in human cells. All genes are divided into four groups according to their binding with Hs65K and HDAC3: Hs65K‐only binding, HDAC3‐only binding, common‐binding, and non‐binding. C) Fold changes of H3K9ac‐signals after knockdown of *Hs65K* (left) and *HsANKRD11* (right). D) Composition of the changed H3K9ac‐signals in the two KD cells. The H3K9ac‐signals are analyzed in four groups, each of them containing four parts according to the location relationship between the H3K9ac peaks and Hs65K, HDAC3 peaks. The numbers of decreased and increased H3K9ac‐signals at the chromatin domains with colocalized triple‐peaks are shown in red and blue, respectively. E) Overlapping of the changed H3K9ac‐signals at chromatin domains with the colocalized triple‐peaks between the two knockdown cells. The significantly increased H3K9ac‐signals show a larger overlapping between the two KD cells than the decreased H3K9ac‐signals. F) Most of the chromatin domains with colocalized triple‐peaks and increased H3K9ac‐signals in both two KD cells exhibit simultaneously decreased binding of Hs65K and HDAC3 in the *HsANKRD11‐KD* cells. G) Forty‐four genes are up‐regulated in both knockdown cells due to the decreased binding of Hs65K and HDAC3 and reduced deacetylation of H3K9 at their chromatin domains with the colocalized triple‐peaks. Three genes for further experimental validation are indicated. H,I) Compared with both down‐regulated 70 genes, both up‐regulated 44 genes have significantly more reduced deacetylation of H3K9 in both the *Hs65K‐KD* and *HsANKRD11‐KD* cells (H). p values were calculated using the Wilcoxon test; ^*^, p value <0.05, ^***^p value <0.001. The paired boxplot of increased H3K9ac sites and expression changes (I).

We then performed ChIP‐seq of acetylated H3K9 and H4K5. In total, we obtained 13910, 13367, and 12006 H3K9ac‐peaks in high quality from the control, *Hs65K‐KD*, and *11‐KD* cells, respectively (**Figure** **5**A; Table S4, Supporting Information), whereas the H4K5ac signals were of low quality and could not be further analyzed. The H3K9ac signals near the TSS region on the Hs65K‐ and HDAC3‐binding genes, especially on their common‐binding genes, were obviously increased in the two KD cells, but not on their non‐binding genes in the *Hs65K‐KD* cells (Figure 5B). Individually, 1502 and 4446 H3K9ac signals were significantly increased in the two KD cells respectively, about two‐fold to the decreased signals (Figure 5C). Composition analyses of the four H3K9ac‐signal changed groups (two directions in two KD cells in Figure 5C) revealed that the H3K9ac‐peaks colocalized with the common‐binding peaks of Hs65K and HDAC3 (i.e., colocalized triple‐peaks) were enriched in the increased groups (579 in *Hs65‐KD* and 1405 in *HsANKRD11‐KD*), while the H3K9ac‐peaks colocalized with the only‐binding peaks were not enriched (Figure 5D). Importantly, 77% of the increased H3K9ac‐signals from the colocalized triple‐peaks in *Hs65K‐KD* (445 out of 579) overlapped with their counterparts in *HsANKRD11‐KD*, whereas the ratio is only 34% between the two decreased groups (Figure 5E; Table S5, Supporting Information). Further analysis revealed that the binding of Hs65K and HDAC3 in *11‐KD* cells were simultaneously decreased on 392 out of the 445 colocalized triple‐peaks where H3K9ac‐signals were increased (Figure 5F). Taken together, these data indicate that the chromatin domain with common‐binding of Hs65K and HDAC3 displays a higher frequency of deacetylation of nearby H3K9 than the chromatin domain with HDAC3‐only binding. Knockdown of ANKRD11 severely disrupts the accessibilities of both 65K and HDAC3 to chromatin domains and results in reduced deacetylation of nearby H3K9 sites.

Sequencing of mRNAs was then performed to find genes whose expression is regulated by the 65K‐ANKRD11 interaction. First, expressions of 798 and 3446 genes were significantly changed in *Hs65K‐KD* and *HsANKRD* *11‐KD (11‐KD* in panels*)* cells, respectively (Figure S7A and Table S6, Supporting Information). Secondly, the splicing of many minor introns was significantly altered in *Hs65K‐KD* cells but was not in *11‐KD* cells (Figure S7B and Table S6, Supporting Information). These two lines of data are consistent with the fact that 65K is a splicing factor and ANKRD11 is a cofactor of HDAC3. Thirdly, we focused on the colocalized triple‐peaks containing genes that have decreased binding of 65K and/or HDAC3 and increased H3K9ac signals in *11‐KD* cells and found that 124 were up‐regulated and 310 were down‐regulated (Figure S7C, Supporting Information). In *Hs65K‐KD* cells, 44 of the 124 genes were also up‐regulated (Figure 5G), and 70 of the 310 genes were also down‐regulated (Figure S7D, Supporting Information), providing reliable evidence that expression changes of those 114 (44+70) genes are due to disruption of the 65K‐ANKRD11 interaction. We further analyzed the H3K9ac‐signal changes and found that in both KD cells, the commonly up‐regulated genes exhibited more reduced deacetylation of H3K9 than the commonly down‐regulated genes (Figure 5H,I), implying that effects of the changed H3K9ac caused by disruption of the 65K‐ANKRD11 interaction is to up‐regulate gene expression, whereas the down‐regulation of gene expression could be due to acetylation changes of other histone sites.

**Figure 6:**
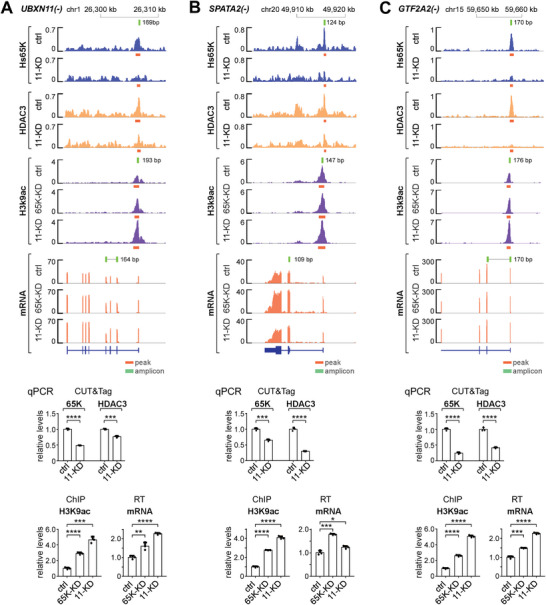
Experimental validation of three genes whose expressions are regulated by the 65K‐ANKRD11‐HDAC3 interaction. Chromatin domains of the three genes, A) *UBXN11*, B) *SPATA2*, and C) *GTF2A2*, have common‐bindings of Hs65K and HDAC3 and H3K9ac‐signals. These three genes are up‐regulated in both the *Hs65K‐KD* and *HsANKRD11‐KD* cells. Changes in the Hs65K and HDAC3 binding, and the H3K9ac‐signals were validated by co‐IPs and quantitative PCRs, gene expressions were validated by quantitative RT‐PCRs. Statistical data are shown as mean ± SD. p values were calculated using two‐sided unpaired t‐test; ^*^ *p* <0.05; ^**^ *p* <0.01; ^***^ *p* <0.001; ^****^ *p* <0.0001.

Further experimental confirmation was carried out for three commonly up‐regulated genes, *UBXN11*, *SPATA2*, and *GTF2A2*. Quantitative PCRs using CUT&Tag samples confirmed that common chromatin‐binding of Hs65K and HDAC3 surrounding TSS regions of these three genes were significantly decreased in *11‐KD* cells, quantitative PCRs using ChIP samples confirmed that the acetylation levels of their nearby H3K9s were increased in both the *Hs65K‐KD* and *11‐KD* cells, and quantitative RT‐PCRs using RNA samples confirmed that their expressions were up‐regulated in both KD cells (**Figure** **6**A–C).

## 3 Discussion

Studies on the regulatory mechanisms of pre‐mRNA splicing have been mostly focused on the major spliceosome, major introns, and major‐intron‐containing genes in the past four decades. Couplings between the histone acetylation machinery and the major spliceosome have been previously identified. For example, Gcn5 is a histone acetyltransferase in the SAGA complex associated with the acetylated H3, which interacts and recruits the U2 snRNP components to the exon,^[^ ^32^, ^38^ ^]^ and MRG15 is a subunit of the NuA4/TIP60 histone acetyltransferase complex, which recruits the PPyT‐binding protein PTB to H3K36me3 sites and represses inclusion of alternative exons.^[^ ^39^ ^]^ Recently, more attention has been paid to minor splicing due to the essentialness of minor‐intron‐containing genes^[^ ^40^ ^]^ and the development of techniques, including next‐generation sequencing, genome editing, and cryo‐electron microscopy.^[^ ^3^, ^8^, ^9^ ^]^ Distinct from the previously identified couplings, our finding of the 65K‐ANKRD11 interaction first reveals a coupling between the minor spliceosome and histone modification, which provides significant regulatory effects on the levels of H3K9ac and H4K5ac and subsequently on gene expression.

**Figure 7:**
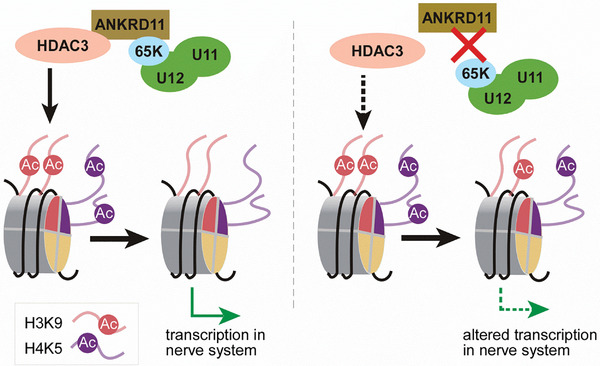
Minor spliceosomal component 65K interacts with ANKRD11 and facilitates HDAC3‐regulated histone deacetylation and gene expression. (Left) In the *WT* condition, HDAC3 deacetylates the histone proteins H3 and H4, which is facilitated by its cofactor ANKRD11. The interaction between ANKRD11 and the minor spliceosome‐specific protein 65K/RNPC3 enhances the chromatin‐binding of HDAC3 to further facilitate the deacetylation of histones, especially on H3K9 and H4K5, and results in regulated gene expression. (Right) Caused by the deficiency of 65K or ANKRD11, the defective 65K‐ANKRD11 interaction weakens the chromatin‐binding of HDAC3 and decreases the deacetylation activity, resulting in changed gene expression. This regulatory mechanism is conserved in both *Drosophila* and human cells.

In this study, we have identified an interaction between the minor spliceosomal 65K/RNPC3 and the histone deacetylation co‐factor ANKRD11 for the first time, which is conserved in both *Drosophila* and human cells. As a bridging factor, ANKRD11 interacts with 65K through its middle uncharacterized domain and mediates the association of 65K with HDAC3. Importantly, thousands of common chromatin‐binding sites of HDAC3 and 65K are synergistically enhanced due to this bridging, which facilitates the deacetylation of nearby histones (**Figure** **7** left). A deficiency of 65K or ANKRD11 causes a decreased 65K‐ANKRD11‐HDAC3 interaction and results in decreased chromatin binding of HDAC3, leading to reduced deacetylation at the nearby H3K9 and H4K5 sites and changed gene expression (Figure 7 right).

Histone acetylation weakens the histone‐DNA interaction, in which acetylations of H3K9, H3K14, H3K18, H3K27, and H4K16 are involved in transcriptional activation. Acetylation of H3K9 and H3K27 are often associated with the enhancers and promoters of active genes, allowing the binding of transcription factors to enhance gene expression^[^ ^41^ ^]^; however, acetylation of H4K5, H4K8, and H4K12 exhibit minor effects on transcription due to their lower presence at the promoter regions.^[^ ^42^ ^]^ Depending on cell types, HDAC3 has variable abilities to deacetylate H3K9ac, H3K18ac, H3K27ac, H4K5ac, H4K8ac, H4K12ac, and H4K16ac.^[^ ^43^ ^]^ Colocalization analyses indicate that about 27.4% of the Hs65K signal are colocalized with HDAC3, ≈6.5% of the Hs65K signal are colocalized with HDAC3 & H3K9ac together, significantly higher than its co‐localization with H3K27ac (3.6%) (Figure S8A–C, Supporting Information); this is consistent with the previous *Hs65K‐KD* results by western blotting (Figure 3E,F). This study demonstrates that the 65K‐ANKRD11 interaction facilitates HDAC3‐regulated deacetylation on H3K9 and H4K5 and that the modification of H3K9 regulates the expression of many genes.

In this study, we show in vivo evidence that the 65K‐ANKRD11 interaction is physiologically important in the brain and nervous systems in *Drosophila*, exhibiting reduced deacetylation of H3K9 and H4K5 in the heads of mutant animals. We retrieved the RNA‐seq data from the FlyBase and found that the expression of ANKRD11 is significantly higher in the brain and nervous systems, but the expression of 65K and HDAC3 are not obviously tissue‐specific (Figure S8D, Supporting Information). In mammals, it has been reported that maintenance of adequate amounts of ANKRD11 and 65K/RNPC3 are both essential for the development of the brain and bone. The heterozygous mutation of mouse ANKRD11 caused a decreased cortical precursor proliferation and perturbed genesis of neurons, leading to the increase of HDAC3‐regulated H4K5ac, H4K8ac, H3K9ac, and H4K16ac.^[^ ^24a^ ^]^ The phenotype of biallelic 65K/RNPC3 variants is mainly associated with growth hormone deficiency, delayed bone, intellectual disability, and brain anomalies.^[^ ^44^ ^]^ We also searched human cancer data (GEPIA2 database), and found that the expression of Hs65K, HsANKRD11, and HDAC3 is significantly changed in tumors versus normal tissues (Figure S8E, Supporting Information).

In this study, there are 145 up‐regulated DEGs in the head of *ankrd11^Δ/Δ^*, of which 21 are the central nervous system (CNS) related genes, and 6 of them are also up‐regulated in the head of *65k^Δ/Δ^* (Figure S8F, Supporting Information). Mutations in these genes are connected with brain diseases, including memory impairment, infantile muscular hypotonia, and cognitive impairment.^[^ ^45^ ^]^ Those shared CNS‐related DEGs and similar defective phenotypes from the *ankrd11^Δ/Δ^* and *65k^Δ/Δ^* animals suggest that the physiological functions of ANKRD11 and 65K in nervous systems are due to the 65K‐ANKRD11 interaction, a common molecular basis for the regulation of gene expression.

Post‐translational modifications may affect the binding strength of the 65K‐ANKRD11 interaction. It has been found that 65K has one site with O‐linked glycan and three phosphorylated serine residues (21, 108, and 381) in human cancer cell lines,^[^ ^46^ ^]^ and ANKRD11 has many modifications in its 65K‐interacting domain (aa1160‐1470), including the SUMOylated K1446,^[^ ^47^ ^]^ N‐glycosylated T1425,^[^ ^48^ ^]^ phosphorylated T1120, S1296, and T1419.^[^ ^49^ ^]^ Therefore, whether these modifications could affect the 65K‐ANKRD11 interaction and consequentially change their DNA‐binding strength is worth further investigation.

## 4 Experimental Section

### Fruitfly strains, *Drosophila* and Human Cell Lines

All *Drosophila* strains were maintained and cultured on a standard cornmeal agar medium. The *WT* was a *w1118* isogenic strain (BDSC 5905) and deletion strains were constructed using a CRISPR/Cas9‐medicated system.^[^ ^9d^ ^]^ Briefly, target sequences of two sgRNAs for each deletion were selected, and donor plasmid on the pMD18‐T vector was constructed with the insertion of the deletion region and the adjacent 2‐kb sequences as homologous arms. The gRNAs and donor plasmid were then co‐injected into embryos of a transgenic line *nanos‐Cas9* by UniHuaii Technology Company. Specific primers were used for genomic PCRs to screen for the desired alleles, which were further validated by sequencing. The obtained lines were then crossed for at least five generations with the *WT* to eliminate potential off‐target events. All the used primers and oligos are listed in Table S7 (Supporting Information). *Drosophila* S2 and human 293T cells were cultured with Schneider's insect medium (Sigma, S9895) and Dulbecco's modified Eagle's medium (Gibco, 11 965 118), respectively.

### Purification and Identification of Dm65K‐Associated Proteins

Ten grams of the Day_3 pupae from the *WT* and *65K‐FTH* strains^[^ ^9d^ ^]^ were collected and squished by a dounce with a loose pestle (Sigma, D9063). The obtained cells were washed with 1x PBS until the grease was removed, and their nuclear extract (NE) was prepared similarly as described.^[^ ^50^ ^]^ Briefly, pupae cells were resuspended and gently pipetted in three volumes of the hypotonic Buffer A [10 mm Tris‐HCl (pH 7.9), 10 mm KCl, 1.5 mm MgCl_2_, 0.5 mm Dithiothreitol, 0.5 mm Phenylmethanesulfonyl fluoride (PMSF), and 1x proteinase inhibitor cocktail (Roche, 04693159001)]. The swollen cells were transferred to a glass dounce homogenizer with a Type‐B pestle (Sigma, D9063) to rupture the cell membrane followed by centrifugation at 3,000 g. The nuclear pellets were resuspended in 0.5x volume of the low‐salt extraction Buffer B [20 mm Tris‐HCl (pH 7.9), 20 mm KCl, 1.5 mm MgCl_2_, 0.2 mm EDTA, 25% glycerol, 0.5 mm PMSF, and RNase and proteinase inhibitors], and then homogenized by a 15 mL dounce. Another 0.5x volume of the high‐salt extraction Buffer C (Buffer B with 1.2 m KCl) was added dropwise. The mixture was further homogenized using a glass dounce with a Type‐B pestle and tumbled for 30 min, followed by centrifugation at 18,000 g for 30 min. The supernatant was carefully transferred to a clean tube, and 1x volume of Buffer B was added to obtain the NE. For co‐purification, 4 mL of the NE was mixed with 100 µL of pre‐balanced ANTI‐FLAG M2 affinity gel (Sigma, A2220). After tumbling, the beads were rinsed by WB150 buffer [20 mm Tris‐HCl (pH 7.4), 150 mm NaCl, 0.1% CA‐630)] and the bound proteins were eluted with 150 ng mL^−1^ of 3x FLAG peptide (Sigma, F4799) three times. The combined elution was then mixed with Ni‐NTA beads (Merck, 70 666) in WB150 buffer with 20 mm imidazole for 2 h. The beads were then rinsed three times, and the associated proteins were eluted by 25 µL of WB150 buffer with 160 mm imidazole twice and applied for mass spectrometry by Q Exactive Focus (Thermo Scientific).

### Knockdown and Overexpression in *Drosophila* and Human Cells

Double‐stranded RNAs (dsRNAs) were constructed by the T7 Ribo‐MAX Express RNAi system (Promega, P1700) and absorbed by S2 cells to knock down the expression of *Drosophila* genes.^[^ ^51^ ^]^ Small hairpin RNAs (shRNAs) were designed by online software from Sigma and loaded into lentiviral vector pLKO.1 to knock down the expression of human genes in 293T cells.^[^ ^52^ ^]^

For expression of proteins with FLAG or V5 tags, coding sequence (CDS) of *Drosophila* genes were cloned into a pIZT‐V5 vector and transfected using Effectene reagent (Qiagen, 301 425) into S2 cells, and CDS of human genes or their truncated sequences were cloned into a pcDNA3.0 vector and transfected using Attractene (Qiagen, 301 005) into 293T cells.

### RT‐PCR and RNA‐seq

Total RNAs from the Drosophila and human cells and fruitfly samples were isolated by TRIzol (Sigma, T9424). For RT‐PCR, reverse transcription was performed using PrimeScript RT reagent Kit with gDNA Eraser (TaKaRa, RR047A), and the obtained cDNA was amplified by 2×Hieff PCR Master Mix (Yeasen, 10102ES03). For mRNA‐seq, the construction of cDNA libraries and sequencing were performed using Illumina Novaseq 6000‐PE150 by Novogene.

### Western Blotting and Antibodies

*Drosophila* samples, S2 cells, and 293T cells were collected and lysed using RIPA buffer for western blotting, in which total protein concentrations were determined by Enhanced BCA Protein Assay Kit (Beyotime, P0010). Western blot signals were visualized by antibodies against H3 (ab176842), H3K9ac (ab4441), H3K14ac (ab52946), H3K27ac (ab177178), H3K9me3 (ab8898), H4 (ab177840), H4K5ac (ab51997), H4K8ac (ab45166) and H4K12ac (ab177793) from Abcam; against Tubulin (AC030) and NCOR1 (A7046) from ABclonal; and against HDAC1 (10197‐1‐AP), HDAC2 (12922‐3‐AP), HDAC3 (10255‐1‐AP), and NCOR2 (29952‐1‐AP) from Proteintech. The immunofluorescence antibodies for 65K, HDAC3, H3K9ac, and H3K27ac were purchased from ThermoFisher Scientific (PA5‐65724), Abcam (ab32369), Active motif, (91 103), and Active motif (39 685) respectively. The anti‐HRP primary antibody and anti‐discs large primary antibody were purchased from Jackson Immuno Research (123‐545‐021), and Developmental Studies Hybridoma Bank (4F3) respectively. The secondary antibodies of immunofluorescence were purchased from Abcam; Goat Anti‐Rabbit IgG H&L (Alexa Fluor® 488), ab150077; Goat Anti‐Rabbit IgG H&L (Alexa Fluor® 405), ab175652; Goat Anti‐Mouse IgG H&L (Alexa Fluor® 594), ab150116.

### *Drosophila* Developmental Assays

The fecundity of *Drosophila WT* and mutants was measured as described.^[^ ^53^ ^]^ Briefly, ten individual mated females (adult 24–28_hr) from each strain were passed to new vials, and their laid eggs per vial were counted each day until Day_10. For hatching rates, 100 eggs from each mated strain were collected and the hatched eggs were counted under standard conditions. After hatching, thirty larvae of each strain were collected into new vials with standard food for detection of the pupation and eclosion rates, respectively. All the above tests were performed in triplets and counted in regular intervals.^[^ ^54^ ^]^ The statistical analyses were performed using GraphPad Prism 7 (San Diego) and the statistical differences were determined by *t*‐tests.

### Immunoprecipitation and Co‐Immunoprecipitation

Cell lysates were prepared from a 6‐well plate culture using 1 mL of IP buffer [20 mm Tris‐HCl (pH 8.0), 150 mm NaCl, 0.5% NP40, 1% Triton X‐100, 1 mm PMSF, and 1x proteinase inhibitor cocktail]. After removing debris, the lysates were first pre‐cleared by 25 µL of Protein A magnetic beads (Thermo, 88 846) for overnight incubation, and then applied to FLAG magnetic beads (Sigma, M8823) or V5 agarose beads (GNI, 4510‐V5) or pre‐conjugated 65K antibody (Proteintech, 25820‐1‐AP) with protein A‐G magnetic beads (Beyotime, P2108) for IP and co‐IP; Protein A magnetic beads were used as negative controls. For nuclease treatments, 20 U DNase I (Takara, 2270A) and/or 7 µL RNase A (Thermo, R1253) were added to the washed beads‐bound complexes in WB150 buffer, and the tubes were placed on a nutator at 28 °C for 30 min. The beads‐bound complexes were washed by WB150 buffer. The co‐purified proteins were separated on SDS‐PAGE and visualized by western blotting.

### CUT&Tag‐seq and ChIP‐seq

The CUT&Tag assay was performed as described.^[^ ^37^ ^]^ Briefly, the 293T cells were fixed and permeated by 0.1% formaldehyde and 0.05% digitonin, respectively. The target protein was sequentially bound with its primary antibody, secondary antibody Goat Anti‐Rabbit IgG (H+L) (ABclonal, AS070), and protein A/G Tn5 (ABclonal, RK20264). The purified DNAs were then tagmentationed and amplified for sequencing by Wuhan Biobank. Antibodies against 65K/RNPC3 (Proteintech, 25820‐1‐AP), HsANKRD11 (ThermoFisher Scientific, PA5‐65561), and HDAC3 (Proteintech, 10255‐1‐AP) were used in this assay.

The ChIP assay was performed as described.^[^ ^55^ ^]^ Briefly, the 293T cells were cross‐linked by 1% formaldehyde for 10 min and then the cell lysate was prepared by removing cytoplasm followed by sonication‐based chromatin fragmentation. Antibodies against H3K9ac (ab4441) and H4K5ac (ab51997) from Abcam were used for IP. The co‐IPed DNAs were applied for library construction by the Scale ssDNA‐seq Lib Prep Kit for Illumina V2 (ABclonal, RK20228) according to the manufacturer's instructions and then sequenced by Wuhan Biobank.

### Immunofluorescence Microscopy

293T cells were cultured on glass coverslips, washed with PBS, fixed with 4% paraformaldehyde, and permeabilized and blocked with 0.3% Triton X‐100, 5% goat serum in PBS. Cells were then incubated with primary antibody at 4 °C overnight. After washing with PBS, cells were incubated with the secondary antibody at 25 °C for 1 h. For colocalization, repeat the steps with other primary antibodies and secondary antibodies. Immunofluorescence images were captured by a structure illumination microscopy (CSR Biotech, P‐104WT).

For immunofluorescence of NMJ, the wandering stage larvae were dissected and stained. Neurons, postsynaptic, and presynaptic membrane were labeled with the indicated antibody. Immunofluorescence images were captured under a confocal laser scanning microscope (Leica SP8).

### Bioinformatic Analyses

Raw reads were cleaned with *trim‐galore* (v0.6.10) and mapped to the *Drosophila melanogaster* genome (dm6, FlyBase) and *Homo sapiens* genome (hg38, Ensembl) using *STAR* (v2.7.9a) for mRNA sequencing and *Bowtie2* (v2.3.5, –no‐discordant –no‐mixed ‐I 50 ‐X 600) for sequencing of CUT&Tag and ChIP assays.

For mRNA sequencing, the reads at the gene level were summarized by *featureCounts* (v2.0.1), followed by *DESeq2 R* package (1.30.1) for analysis of differential expression,^[^ ^56^ ^]^ and by *rMATS* (v4.1.2) for analysis of alternative splicing. Differentially‐expressed genes were identified when padj < 0.05 and foldchange > = 2. For CUT&Tag and ChIP sequencing, duplicate reads were removed by *picard* (v2.25), and peaks were called using *MACS2* (v2.2.7) with parameters (‐f BAMPE ‐q 0.001 –min‐length 200). Overlapping and colocalization between peaks were defined by *BEDTools* (v2.30.0, gene/peak: intersectBed –a genes (extended 3Kb laterally) –b peaks –F 0.2; peak/peak: windowBed –w 200). Differential peaks were analyzed by *DiffBind* (v3.0.15), the difference was considered significant when p‐value < 0.05.^[^ ^57^ ^]^ Data visualization was performed by *Deeptools* (3.5.0) for meta gene/region profile plots^[^ ^58^ ^]^ and by IGV (Integrative Genomics Viewer) for track plots, and R (v4.0.5) for the generation of other plots.

### Quantification and Statistical Analysis

For quantification of Western blot, ImageJ software was used to measure the relative intensity of each band, and the relative protein levels were normalized to levels of loading controls. Data were presented as the means ± SD from three independent experiments, and the differences between any two groups were compared by unpaired t‐test. For quantification of the boutons of NMJ, Types Ib and Is boutons at muscles 1 and 9 regions in abdominal segment A3 were counted. Data were presented as the means ± SD from three independent experiments, and the differences between any two groups were compared by unpaired t‐test. For quantification of the relative mobility, the larva locomotion paths were tracked as described.^[^ ^9d^ ^]^ For each strain, Data were presented as the means ± SD from five independent experiments. For quantification of the qPCR, data were presented as the means ± SD from three independent experiments, and the differences between any two groups were compared by unpaired t‐test. For quantification of the colocalization of the Hs65K/HDAC3 and the H3K9ac/H3K27ac signals, ImageJ software was used to measure the amount of protein, and then the colocalized amount of protein was compared to the total amount of 65K or HDAC3 as the relative colocalization. For each group, data were presented as the means ± SD from six independent counts.

### Accession numbers

All NGS data have been deposited to the Gene Expression Omnibus (accession number GSE243715).

## Conflict of Interest

The authors declare no conflict of interest.

## Author Contributions

C.‐H.L. and S.‐B.L. contributed equally to this work as co‐first authors. C.H.L., S.B.L., Y.J.F., and Y.Z.X. conceived the project and designed the experiments. C.H.L., Z.Z.Z., N.L., K.C.W., and L.L. performed the experiments. S.B.L., Q.W.H., and Z.D. performed the bioinformatic analyses. C.H.L., S.B.L., Y.J.F., and Y.Z.X. wrote the manuscript.

## Supporting information

Supporting Information

Supporting Information

## Data Availability

The data that support the findings of this study are available in the supporting information of this article.

## References

[1] M. E. Wilkinson , C. Charenton , K. Nagai , Annu. Rev. Biochem. 2020, 89, 359. 31794245 10.1146/annurev-biochem-091719-064225

[2] a) B. Kastner , C. L. Will , H. Stark , R. Luhrmann , Cold Spring Harbor Perspect. Biol. 2019, 11, a032417; 10.1101/cshperspect.a032417 PMC6824238 30765414

[3] Z. Ding , Y. R. Meng , Y. J. Fan , Y. Z. Xu , Wiley Interdiscip. Rev.: RNA. 2022, 14, e1761. 36056453 10.1002/wrna.1761

[4] a) G. E. Larue , M. Elias , S. W. Roy , Curr. Biol. 2021, 31, 3125; 34015249 10.1016/j.cub.2021.04.050

[5] a) R. A. Padgett , Trends Genet. 2012, 28, 147; 22397991 10.1016/j.tig.2012.01.001 PMC3319163

[6] W. Y. Tarn , J. A. Steitz , Cell 1996, 84, 801. 8625417 10.1016/s0092-8674(00)81057-0

[7] C. L. Will , C. Schneider , M. Hossbach , H. Urlaub , R. Rauhut , S. Elbashir , T. Tuschl , R. Luhrmann , RNA 2004, 10, 929. 15146077 10.1261/rna.7320604 PMC1370585

[8] R. Bai , R. Wan , L. Wang , K. Xu , Q. Zhang , J. Lei , Y. Shi , Science 2021, 371, abg0879. 10.1126/science.abg0879 33509932

[9] a) F. Bai , J. Corll , D. N. Shodja , R. Davenport , G. Feng , J. Mudunkothge , C. J. Brigolin , F. Martin , G. Spielbauer , C. W. Tseung , A. E. Siebert , W. B. Barbazuk , S. Lal , A. M. Settles , The Plant Cell 2019, 31, 715; 30760564 10.1105/tpc.18.00754 PMC6482629

[10] a) T. Alpert , L. Herzel , K. M. Neugebauer , Wiley Interdiscip. Rev.: RNA. 2017, 8, 1401; 10.1002/wrna.1401 PMC5355006 27873472

[11] a) S. Naftelberg , I. E. Schor , G. Ast , A. R. Kornblihtt , Annu. Rev. Biochem. 2015, 84, 165; 26034889 10.1146/annurev-biochem-060614-034242

[12] C. J. David , A. R. Boyne , S. R. Millhouse , J. L. Manley , Genes Dev. 2011, 25, 972. 21536736 10.1101/gad.2038011 PMC3084030

[13] W. Shao , Z. Ding , Z. Z. Zheng , J. J. Shen , Y. X. Shen , J. Pu , Y. J. Fan , C. C. Query , Y. Z. Xu , Nucleic Acids Res. 2020, 48, 5799. 32399566 10.1093/nar/gkaa311 PMC7293005

[14] T. Nojima , K. Rebelo , T. Gomes , A. R. Grosso , N. J. Proudfoot , M. Carmo‐Fonseca , Mol. Cell 2018, 72, 369. 30340024 10.1016/j.molcel.2018.09.004 PMC6201815

[15] a) M. Murawska , A. Brehm , Transcription 2011, 2, 244; 22223048 10.4161/trns.2.6.17840 PMC3265784

[16] A. Ganez‐Zapater , S. D. Mackowiak , Y. Guo , M. Tarbier , A. Jordan‐Pla , M. R. Friedlander , N. Visa , A. K. Ostlund Farrants , Mol. Genet. Genomics 2022, 297, 463. 35187582 10.1007/s00438-022-01863-9 PMC8960663

[17] a) R. J. Loomis , Y. Naoe , J. B. Parker , V. Savic , M. R. Bozovsky , T. Macfarlan , J. L. Manley , D. Chakravarti , Mol. Cell 2009, 33, 450; 19250906 10.1016/j.molcel.2009.02.003 PMC2667802

[18] P. Xu , L. Zhang , Y. Xiao , W. Li , Z. Hu , R. Zhang , J. Li , F. Wu , Y. Xi , Q. Zou , Z. Wang , R. Guo , H. Ma , S. Dong , M. Xiao , Z. Yang , X. Ren , C. Wei , W. Yu , Hum. Mol. Genet. 2021, 30, 2110. 34196368 10.1093/hmg/ddab178

[19] P. Tessarz , T. Kouzarides , Nat. Rev. Mol. Cell Biol. 2014, 15, 703. 25315270 10.1038/nrm3890

[20] a) H. Boeger , J. Griesenbeck , J. S. Strattan , R. D. Kornberg , Mol. Cell 2003, 11, 1587; 12820971 10.1016/s1097-2765(03)00231-4

[21] Z. Wang , C. Zang , K. Cui , D. E. Schones , A. Barski , W. Peng , K. Zhao , Cell 2009, 138, 1019. 19698979 10.1016/j.cell.2009.06.049 PMC2750862

[22] E. Seto , M. Yoshida , Cold Spring Harbor Perspect. Biol. 2014, 6, a018713. 10.1101/cshperspect.a018713 PMC3970420 24691964

[23] S. Bhaskara , S. K. Knutson , G. Jiang , M. B. Chandrasekharan , A. J. Wilson , S. Zheng , A. Yenamandra , K. Locke , J. L. Yuan , A. R. Bonine‐Summers , C. E. Wells , J. F. Kaiser , M. K. Washington , Z. Zhao , F. F. Wagner , Z. W. Sun , F. Xia , E. B. Holson , D. Khabele , S. W. Hiebert , Cancer Cell 2010, 18, 436. 21075309 10.1016/j.ccr.2010.10.022 PMC3004468

[24] a) D. Gallagher , A. Voronova , M. A. Zander , G. I. Cancino , A. Bramall , M. P. Krause , C. Abad , M. Tekin , P. M. Neilsen , D. F. Callen , S. W. Scherer , G. M. Keller , D. R. Kaplan , K. Walz , F. D. Miller , Dev. Cell 2015, 32, 31; 25556659 10.1016/j.devcel.2014.11.031

[25] C. Schneider , C. L. Will , J. Brosius , M. J. Frilander , R. Luhrmann , Proc. Natl. Acad. Sci. USA 2004, 101, 9584. 15210936 10.1073/pnas.0403400101 PMC470718

[26] R. C. Edgar , S. Batzoglou , Curr. Opin. Struct. Biol. 2006, 16, 368. 16679011 10.1016/j.sbi.2006.04.004

[27] B. Zhang , Z. Ding , L. Li , L. K. Xie , Y. J. Fan , Y. Z. Xu , PLoS Genet. 2021, 17, e1009861. 34723968 10.1371/journal.pgen.1009861 PMC8559932

[28] A. Rudenko , L. H. Tsai , Neuropharmacology 2014, 80, 70. 24495398 10.1016/j.neuropharm.2014.01.043

[29] J. E. Faust , A. Verma , C. Peng , J. A. McNew , Traffic 2012, 13, 1378. 22758915 10.1111/j.1600-0854.2012.01393.x PMC3443258

[30] W. Liu , W. Liang , X. P. Xiong , J. L. Li , R. Zhou , PLoS Genet. 2022, 18, e1010433. 36301831 10.1371/journal.pgen.1010433 PMC9612563

[31] O. V. Belyaeva , S. A. Lee , O. V. Kolupaev , N. Y. Kedishvili , Bioch. Biophys. Acta 2009, 1790, 1266. 10.1016/j.bbagen.2009.06.002 PMC2782731 19520149

[32] F. Q. Gunderson , T. L. Johnson , PLoS Genet. 2009, 5, e1000682. 19834536 10.1371/journal.pgen.1000682 PMC2752994

[33] A. Zhang , C. W. Li , J. D. Chen , Biochem. Biophys. Res. Commun. 2007, 358, 1034. 17521611 10.1016/j.bbrc.2007.05.017 PMC1950474

[34] A. Zhang , P. L. Yeung , C. W. Li , S. C. Tsai , G. K. Dinh , X. Wu , H. Li , J. D. Chen , J. Biol. Chem. 2004, 279, 33799. 15184363 10.1074/jbc.M403997200

[35] a) C. Leo , J. D. Chen , Gene 2000, 245, 1; 10713439 10.1016/s0378-1119(00)00024-x

[36] M. G. Guenther , O. Barak , M. A. Lazar , Mol. Cell. Biol. 2001, 21, 6091. 11509652 10.1128/MCB.21.18.6091-6101.2001 PMC87326

[37] H. S. Kaya‐Okur , S. J. Wu , C. A. Codomo , E. S. Pledger , T. D. Bryson , J. G. Henikoff , K. Ahmad , S. Henikoff , Nat. Commun. 2019, 10, 1930. 31036827 10.1038/s41467-019-09982-5 PMC6488672

[38] S. S. Li , M. A. Shogren‐Knaak , J. Biol. Chem. 2009, 284, 9411. 19218239 10.1074/jbc.M809617200 PMC2666593

[39] R. F. Luco , Q. Pan , K. Tominaga , B. J. Blencowe , O. M. Pereira‐Smith , T. Misteli , Science 2010, 327, 996. 20133523 10.1126/science.1184208 PMC2913848

[40] a) J. Argente , R. Flores , A. Gutierrez‐Arumi , B. Verma , G. A. Martos‐Moreno , I. Cusco , A. Oghabian , J. A. Chowen , M. J. Frilander , L. A. Perez‐Jurado , EMBO Mol. Med. 2014, 6, 299; 24480542 10.1002/emmm.201303573 PMC3958305

[41] S. Y. Roth , J. M. Denu , C. D. Allis , Annu. Rev. Biochem. 2001, 70, 81. 11395403 10.1146/annurev.biochem.70.1.81

[42] M. F. Dion , S. J. Altschuler , L. F. Wu , O. J. Rando , Proc. Natl. Acad. Sci. USA 2005, 102, 5501. 15795371 10.1073/pnas.0500136102 PMC555684

[43] a) X. Zhang , W. Wharton , Z. Yuan , S. C. Tsai , N. Olashaw , E. Seto , Mol. Cell. Biol. 2004, 24, 5106; 15169878 10.1128/MCB.24.12.5106-5118.2004 PMC419886

[44] E. A. Verberne , S. Faries , M. Mannens , A. V. Postma , M. M. van Haelst , Am. J. Med. Genet. Part A 2020, 182, 1952. 32462814 10.1002/ajmg.a.61632 PMC7496482

[45] L. Kolberg , U. Raudvere , I. Kuzmin , J. Vilo , H. Peterson , F1000Research 2020, 9, 709. 10.12688/f1000research.24956.1 PMC7859841 33564394

[46] a) H. J. Zhou , S. Di Palma , C. Preisinger , M. Peng , A. N. Polat , A. J. R. Heck , S. Mohammed , J. Proteome. Res. 2013, 12, 260; 23186163 10.1021/pr300630k

[47] I. A. Hendriks , D. Lyon , C. Young , L. J. Jensen , A. C. O. Vertegaal , M. L. Nielsen , Nat. Struct. Mol. Biol. 2017, 24, 325. 28112733 10.1038/nsmb.3366

[48] C. M. Woo , P. J. Lund , A. C. Huang , M. M. Davis , C. R. Bertozzi , S. J. Pitteri , Mol. Cell Proteomics 2018, 17, 764. 29351928 10.1074/mcp.RA117.000261 PMC5880114

[49] J. V. Olsen , B. Blagoev , F. Gnad , B. Macek , C. Kumar , P. Mortensen , M. Mann , Cell 2006, 127, 635. 17081983 10.1016/j.cell.2006.09.026

[50] T. W. Nilsen , Cold Spring Harb. Protoc. 2013, 2013, 579. 23734028 10.1101/pdb.prot075176

[51] C. Qiu , Y. Zhang , Y. J. Fan , T. L. Pang , Y. Su , S. Zhan , Y. Z. Xu , J. Mol. Cell Biol. 2019, 11, 170. 29750417 10.1093/jmcb/mjy029 PMC6734145

[52] S. C. Wei , L. Fattet , J. H. Tsai , Y. Guo , V. H. Pai , H. E. Majeski , A. C. Chen , R. L. Sah , S. S. Taylor , A. J. Engler , J. Yang , Nat. Cell Biol. 2015, 17, 678. 25893917 10.1038/ncb3157 PMC4452027

[53] I. Ostojic , W. Boll , M. J. Waterson , T. Chan , R. Chandra , S. D. Pletcher , J. Alcedo , Proc. Natl. Acad. Sci. USA 2014, 111, 8143. 24847072 10.1073/pnas.1315466111 PMC4050613

[54] S. Gronke , D. F. Clarke , S. Broughton , T. D. Andrews , L. Partridge , Plos. Genet. 2010, 6, e1000857. 20195512 10.1371/journal.pgen.1000857 PMC2829060

[55] D. U. Gorkin , I. Barozzi , Y. Zhao , Y. Zhang , H. Huang , A. Y. Lee , B. Li , J. Chiou , A. Wildberg , B. Ding , B. Zhang , M. Wang , J. S. Strattan , J. M. Davidson , Y. Qiu , V. Afzal , J. A. Akiyama , I. Plajzer‐Frick , C. S. Novak , M. Kato , T. H. Garvin , Q. T. Pham , A. N. Harrington , B. J. Mannion , E. A. Lee , Y. Fukuda‐Yuzawa , Y. He , S. Preissl , S. Chee , J. Y. Han , et al., Nature 2020, 583, 744. 32728240 10.1038/s41586-020-2093-3 PMC7398618

[56] M. I. Love , W. Huber , S. Anders , Genome Biol. 2014, 15, 550. 25516281 10.1186/s13059-014-0550-8 PMC4302049

[57] C. S. Ross‐Innes , R. Stark , A. E. Teschendorff , K. A. Holmes , H. R. Ali , M. J. Dunning , G. D. Brown , O. Gojis , I. O. Ellis , A. R. Green , S. Ali , S. F. Chin , C. Palmieri , C. Caldas , J. S. Carroll , Nature 2012, 481, 389. 22217937 10.1038/nature10730 PMC3272464

[58] F. Ramirez , D. P. Ryan , B. Gruning , V. Bhardwaj , F. Kilpert , A. S. Richter , S. Heyne , F. Dundar , T. Manke , Nucleic Acids Res. 2016, 44, W160. 27079975 10.1093/nar/gkw257 PMC4987876

