# Supplementary material for: Minor Spliceosomal 65K/RNPC3 Interacts with ANKRD11 and Mediates HDAC3‐Regulated Histone Deacetylation and Transcription
Source: Adv Sci (Weinh). 2024 Jun 5;11(29):2307804. doi: 10.1002/advs.202307804 (PMC11304329; doi:10.1002/advs.202307804)

ADVANCED SCIENCE
Open Access

Supporting Information

for *Adv. Sci.*, DOI 10.1002/advs.202307804

Minor Spliceosomal 65K/RNPC3 Interacts with ANKRD11 and Mediates HDAC3-Regulated Histone Deacetylation and Transcription

*Chen-Hui Li, Shao-Bo Liang, Qi-Wei Huang, Zhen-Zhen Zhou, Zhan Ding, Ni Long, Kwang-Chon Wi, Liang Li, Xi-Ping Jiang, Yu-Jie Fan and Yong-Zhen Xu**

Figure 1

Fig.1A

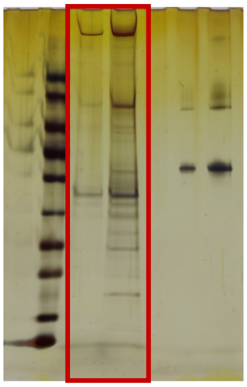

Fig.1C

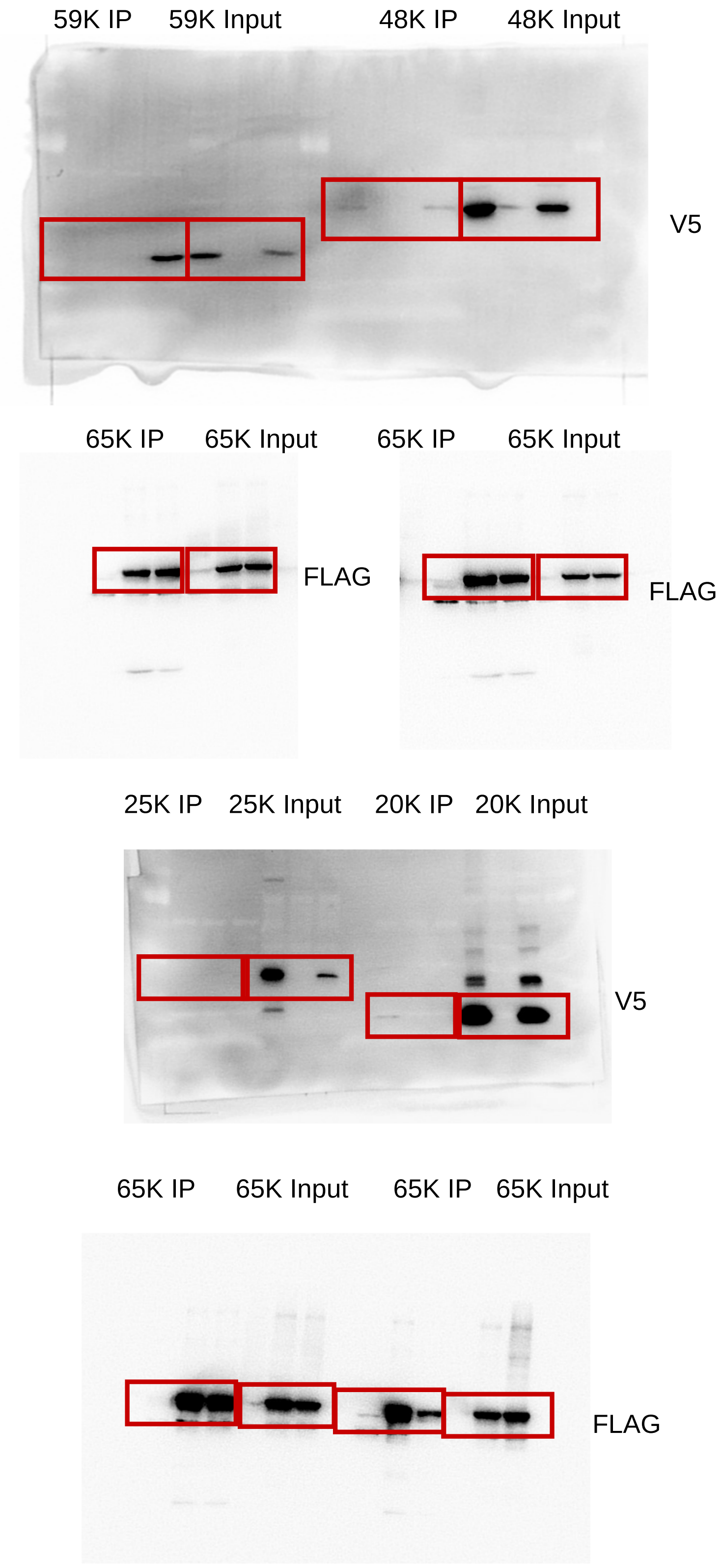

Fig.1D

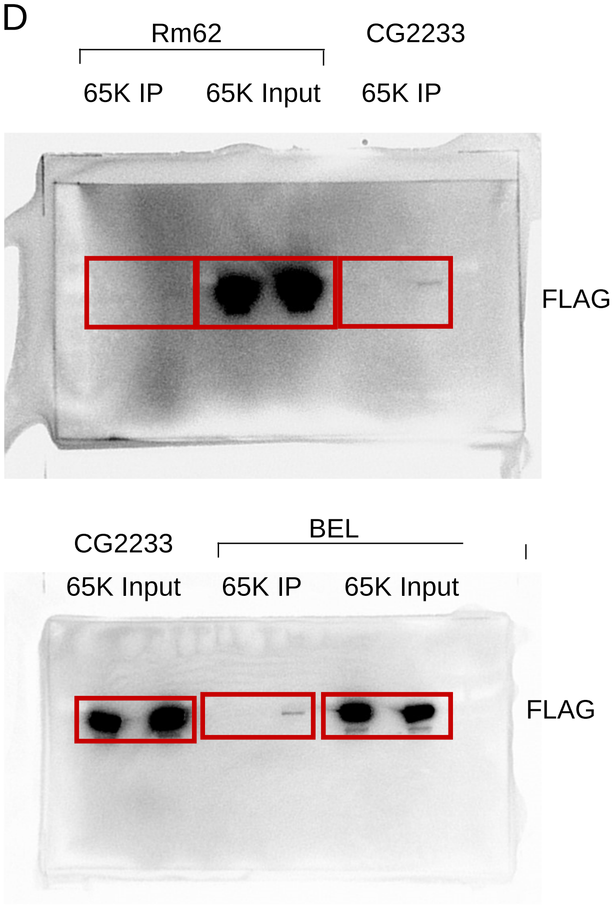

Figure 1 continued

Fig.1D

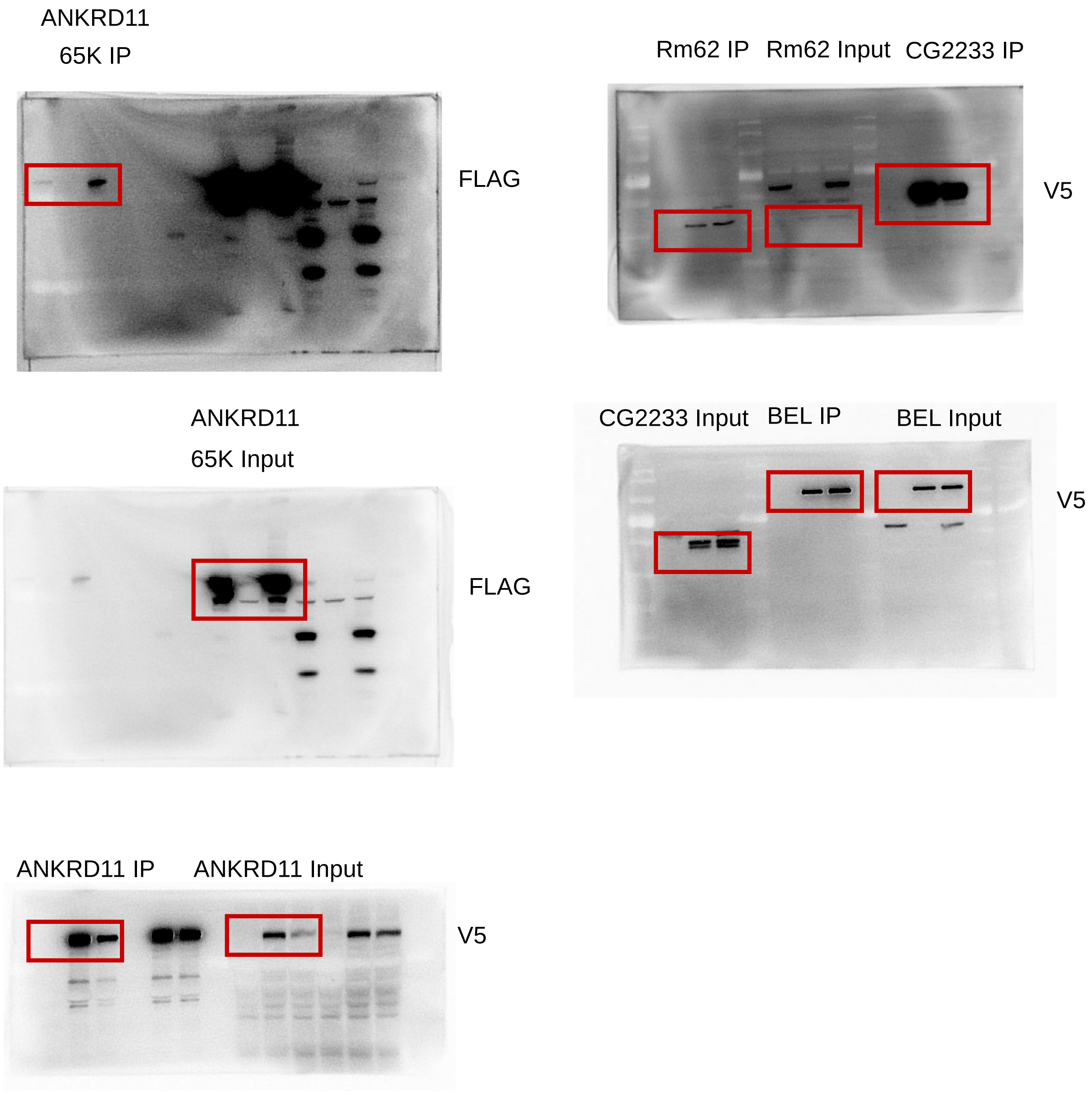

Figure 1 continued

Fig.1E

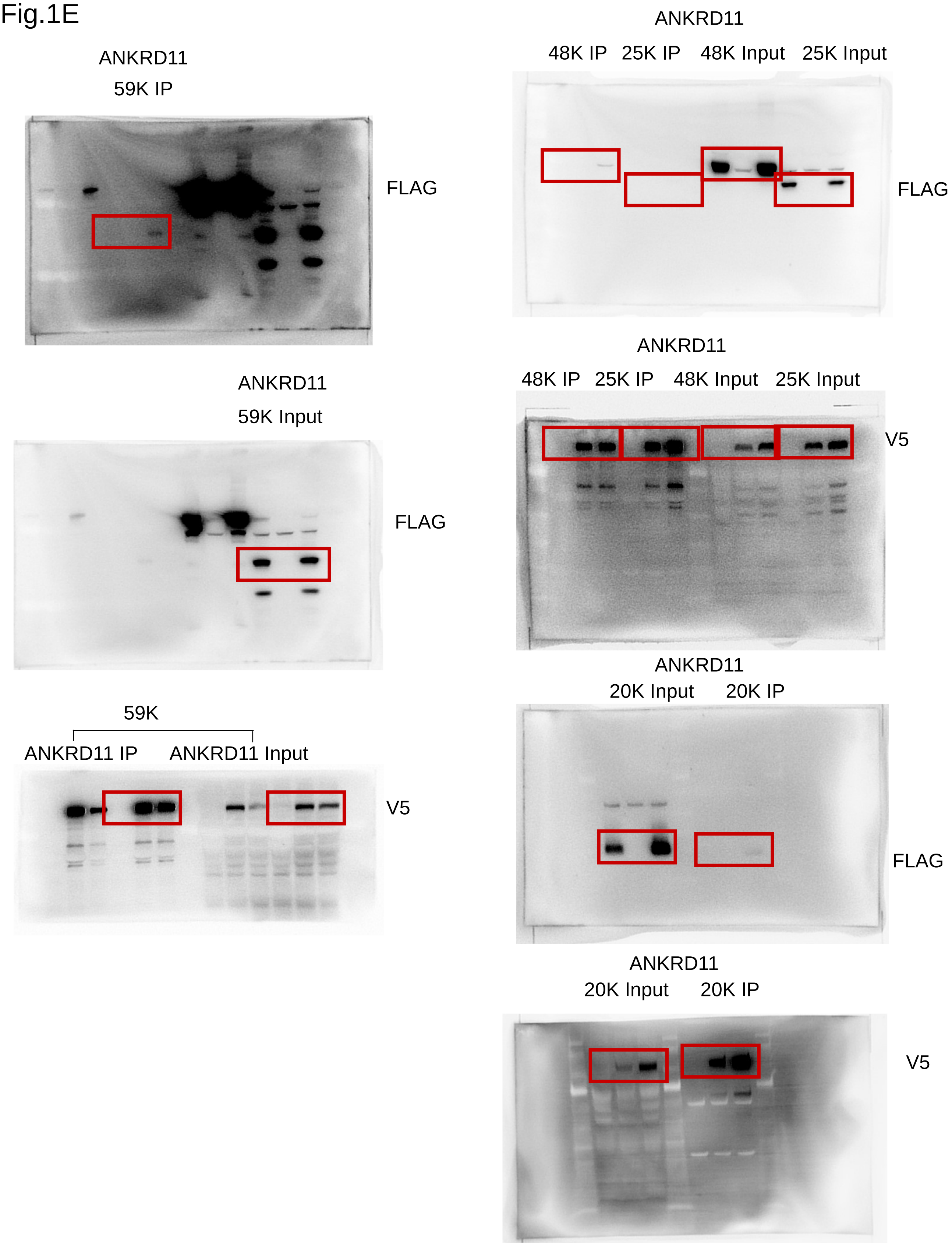

Figure 1 continued

Fig.1F

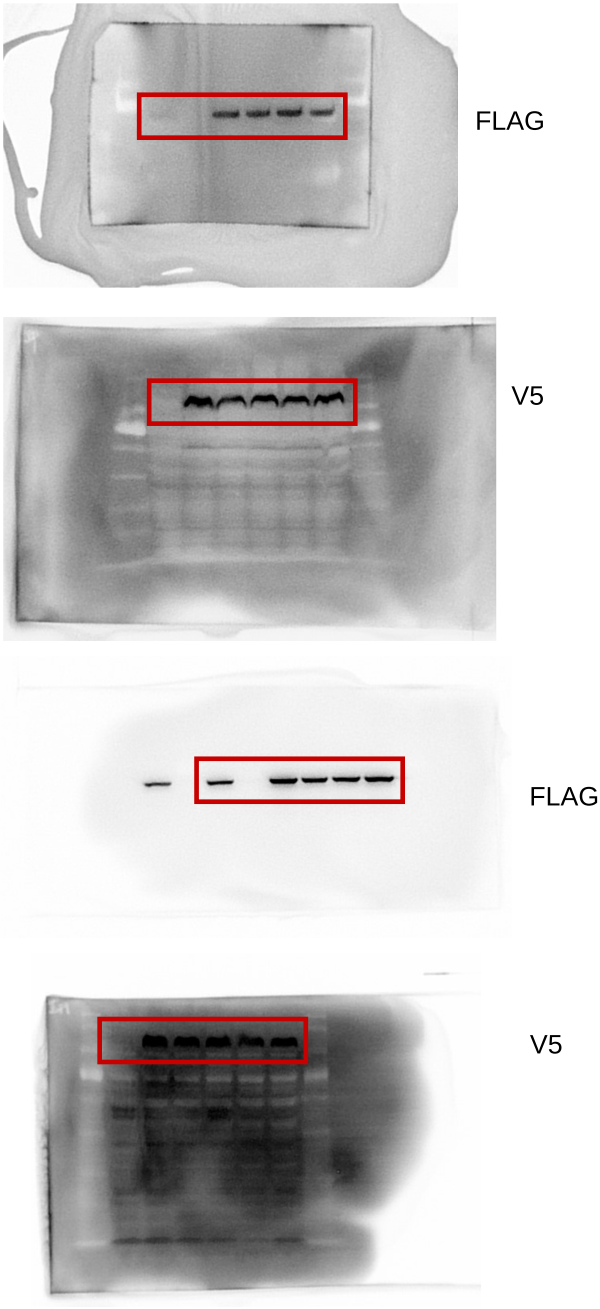

Fig.2A

	WT	*$U12^{\Delta/\Delta}$*	*$65K^{\Delta/\Delta}$*	*$ankrd11^{\Delta/\Delta}$*
EXP1	70	28	47	45
EXP2	75	34	52	47
EXP3	68	26	50	38

Fig.2B

	WT	*$U12^{\Delta/\Delta}$*	*$65K^{\Delta/\Delta}$*	*$ankrd11^{\Delta/\Delta}$*
EXP1	0.889119	0.43857	0.608877	0.582928
EXP2	1.355403	0.386389	0.388645	0.591696
EXP3	0.925887	0.499713	0.550551	0.620598
EXP4	0.979419	0.382328	0.594904	0.568722
EXP5	0.850171	0.434779	0.371111	0.627269

Fig.2C

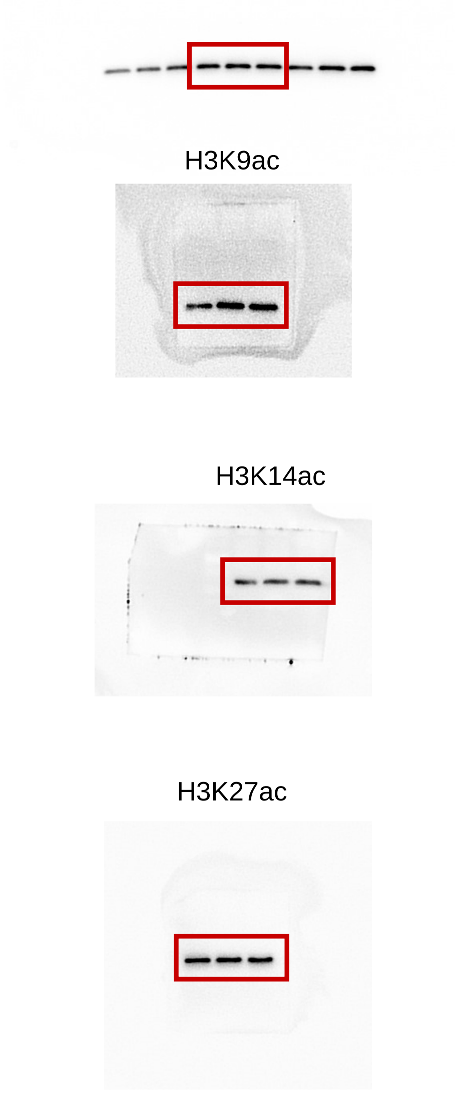

Figure 2 continued

Fig.2C

H3K36ac

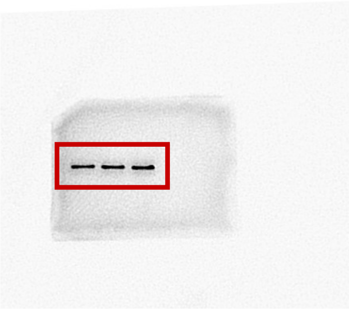

H3K9me3

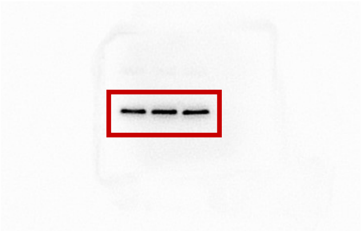

Tubulin

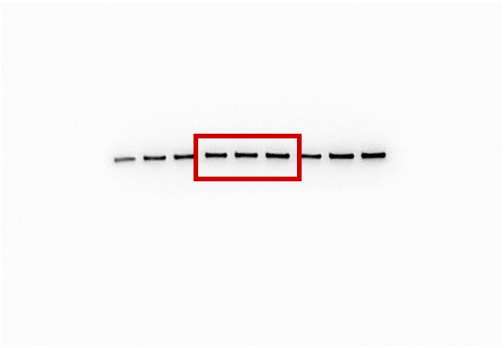

Tubulin

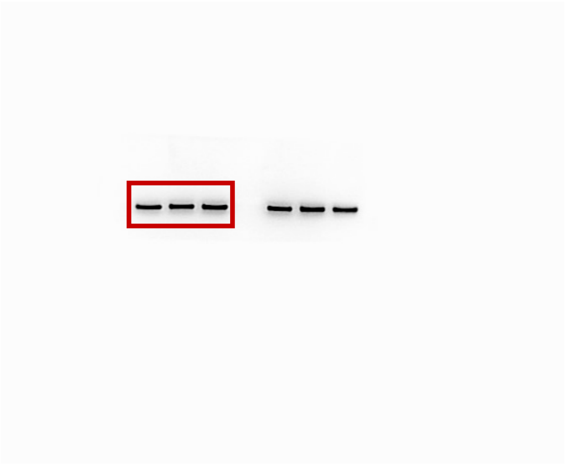

H4

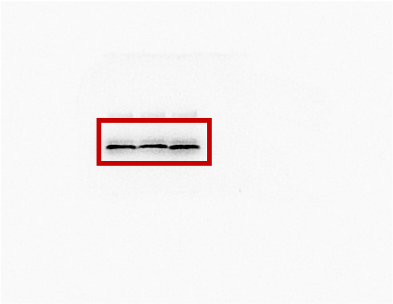

H4K5ac

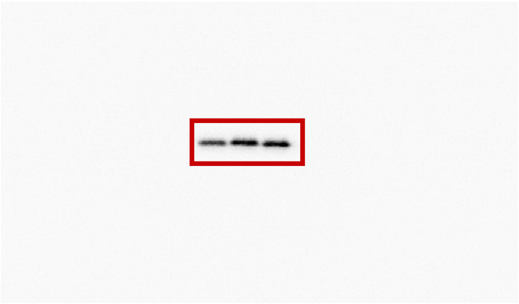

H4K8ac

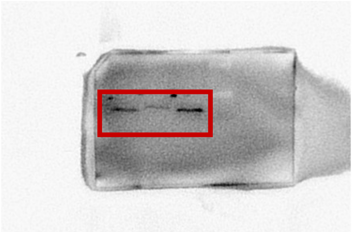

H4K12ac

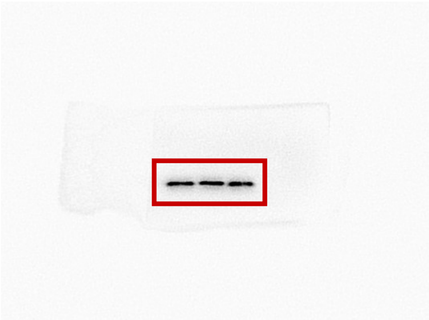

Figure 2 continued

Fig.2D

	WT	*65K*$^{\Delta/\Delta}$	*ankrd11*$^{\Delta/\Delta}$		*WT*	*65K*$^{\Delta/\Delta}$	*ankrd11*$^{\Delta/\Delta}$		*WT*	*65K*$^{\Delta/\Delta}$	*ankrd11*$^{\Delta/}$
H3 EXP 1	1	1.035084	0.994909	H3K9ac EXP 1	1	1.462582	1.553689	H3K14ac EXP 1	1	1.110377	1.144365
H3 EXP 2	1	1.012626	1.026687	H3K9ac EXP 2	1	1.430776	1.51717	H3K14ac EXP 2	1	1.032855	0.973835
H3 EXP 3	1	0.941267	1.011679	H3K9ac EXP 3	1	1.350199	1.415872	H3K14ac EXP 3	1	0.817064	0.716566
	WT	*65K*$^{\Delta/\Delta}$	*ankrd11*$^{\Delta/\Delta}$		*WT*	*65K*$^{\Delta/\Delta}$	*ankrd11*$^{\Delta/\Delta}$		*WT*	*65K*$^{\Delta/\Delta}$	*ankrd11*$^{\Delta/}$
H3K27ac EXP 1	1	1.031235	0.951492	H3K36ac EXP 1	1	1.022776	1.033422	H3K9me3 EXP 1	1	1.034138	0.96372
H3K27ac EXP 2	1	0.913868	0.859	H3K36ac EXP 2	1	1.010792	1.08558	H3K9me3 EXP 2	1	0.826271	0.93461
H3K27ac EXP 3	1	0.920939	0.94286	H3K36ac EXP 3	1	1.117007	1.235107	H3K9me3 EXP 3	1	0.953639	1.062616

	WT	*65K*$^{\Delta/\Delta}$	*ankrd11*$^{\Delta/\Delta}$		*WT*	*65K*$^{\Delta/\Delta}$	*ankrd11*$^{\Delta/}$
H4 EXP 1	1	0.933869	0.992657	H4K5ac EXP 1	1	1.39463	1.298547
H4 EXP 2	1	0.930872	0.967182	H4K5ac EXP 2	1	1.376926	1.568615
H4 EXP 3	1	0.953505	0.798644	H4K5ac EXP 3	1	1.270487	1.359795
	WT	*65K*$^{\Delta/\Delta}$	*ankrd11*$^{\Delta/\Delta}$		*WT*	*65K*$^{\Delta/\Delta}$	*ankrd11*$^{\Delta/}$
H4K8ac EXP 1	1	0.462961	1.354438	H4K12ac EXP 1	1	1.002154	0.973274
H4K8ac EXP 2	1	0.609231	1.222024	H4K12ac EXP 2	1	0.889988	0.991502
H4K8ac EXP 3	1	0.561819	1.803869	H4K12ac EXP 3	1	0.992928	1.028517

Figure 3

Fig.3B

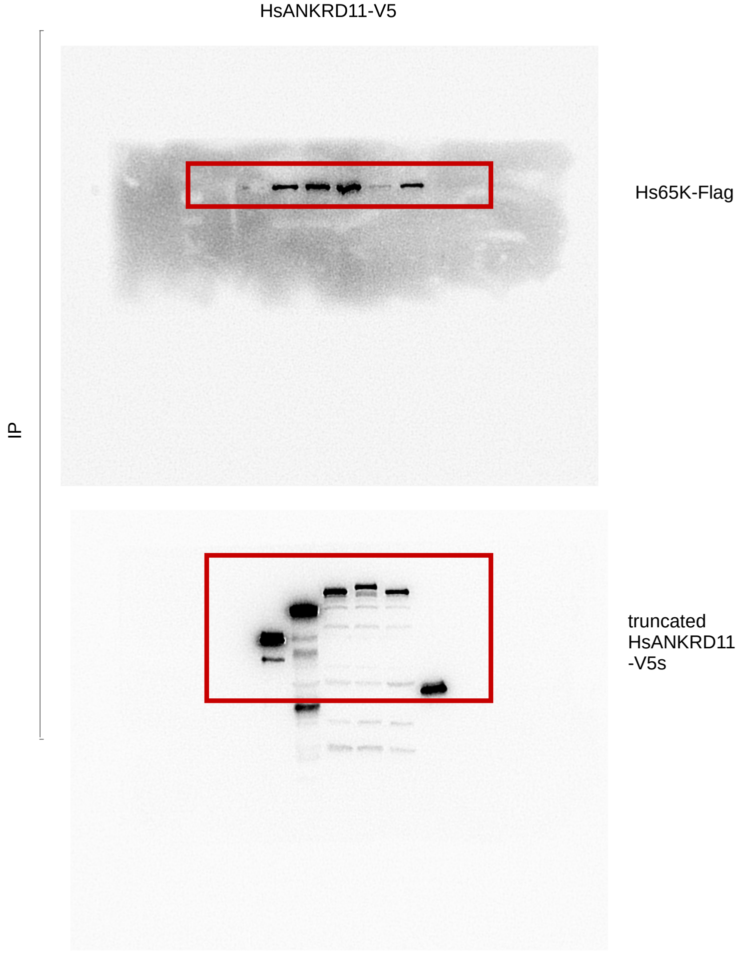

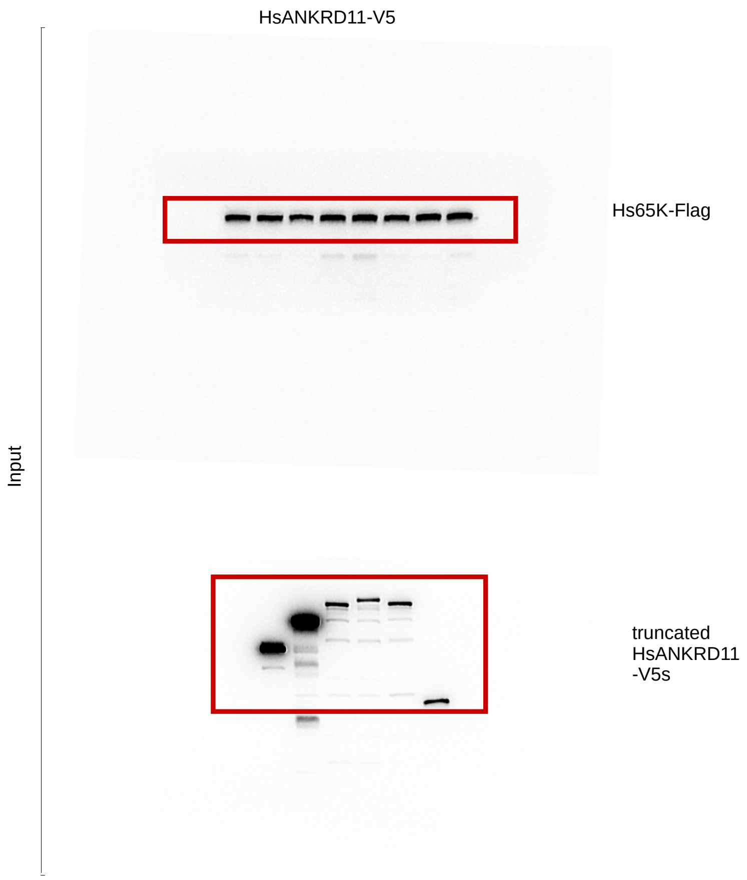

Figure 3 continued

Fig.3B

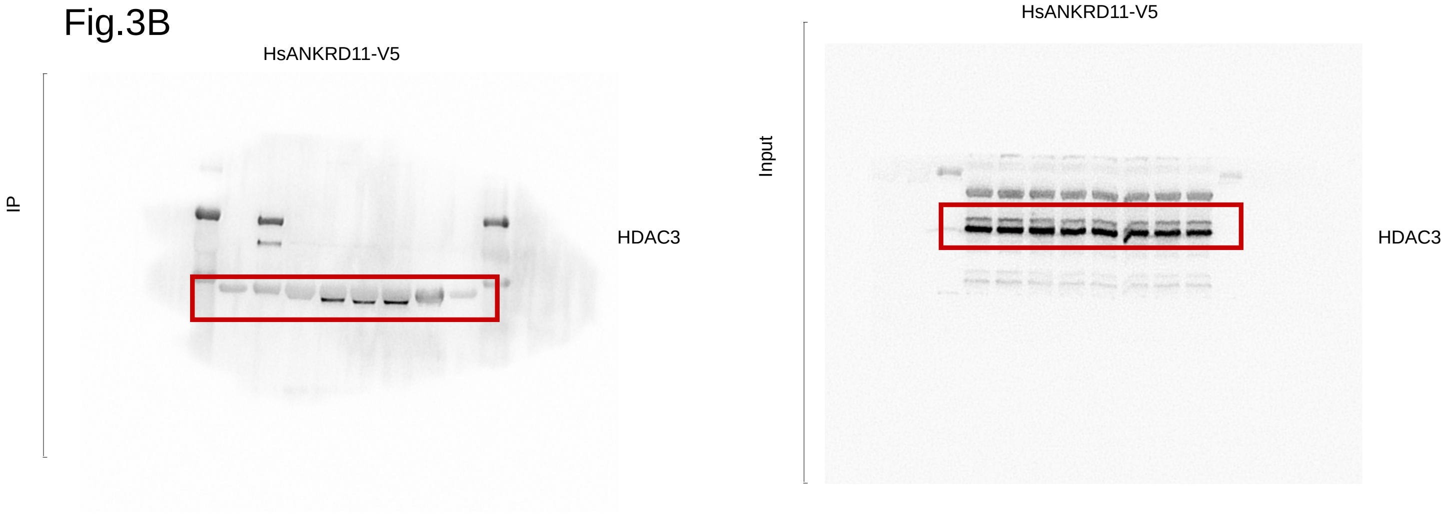

Fig.3C

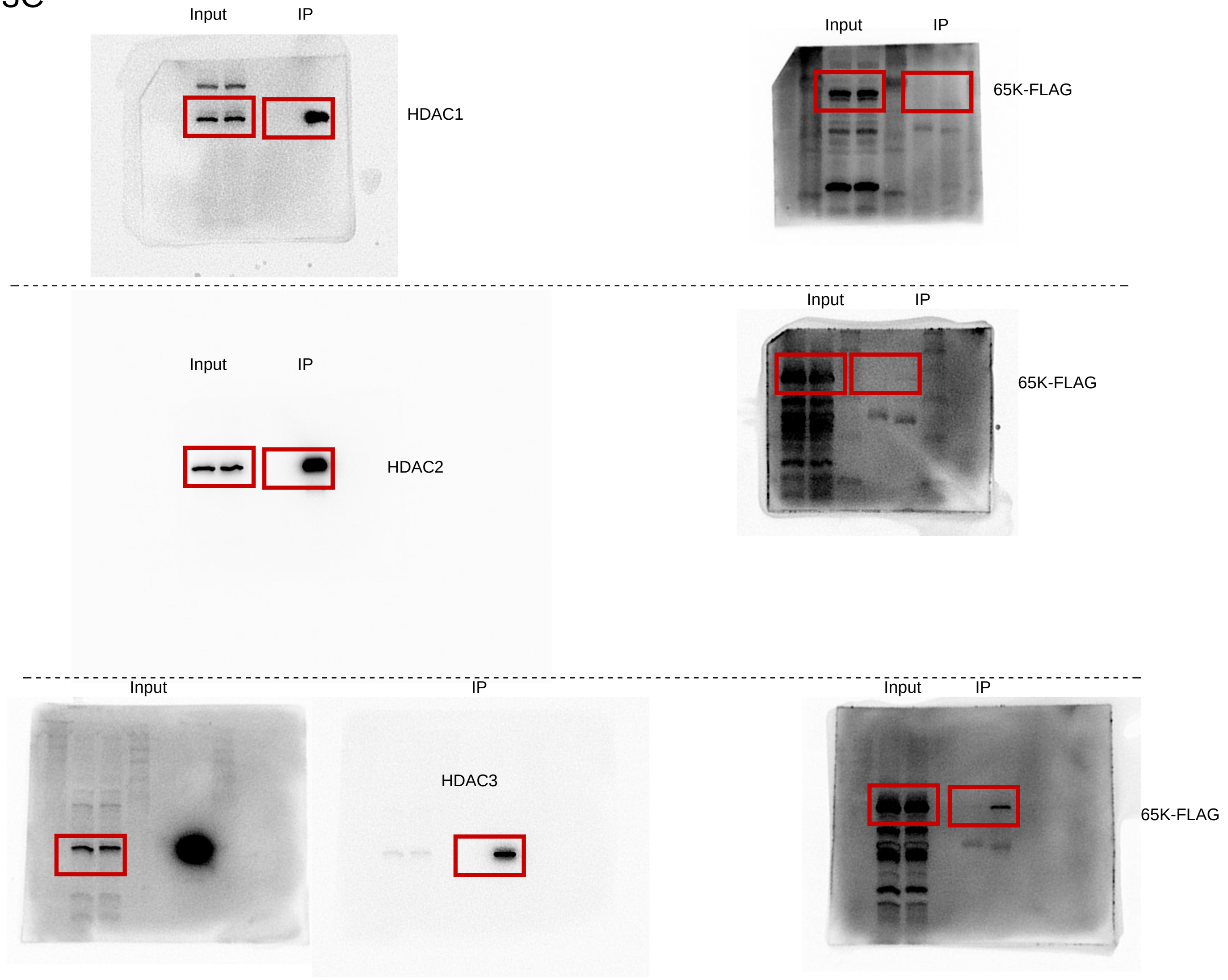

Figure3 continued

Fig.3D

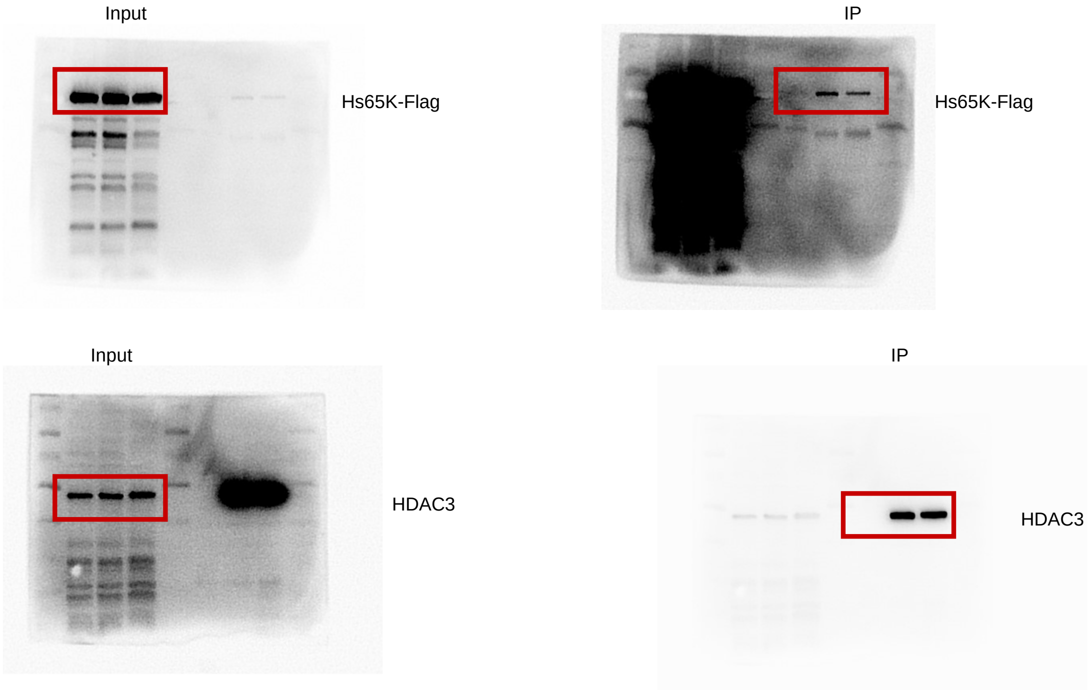

Fig.3E

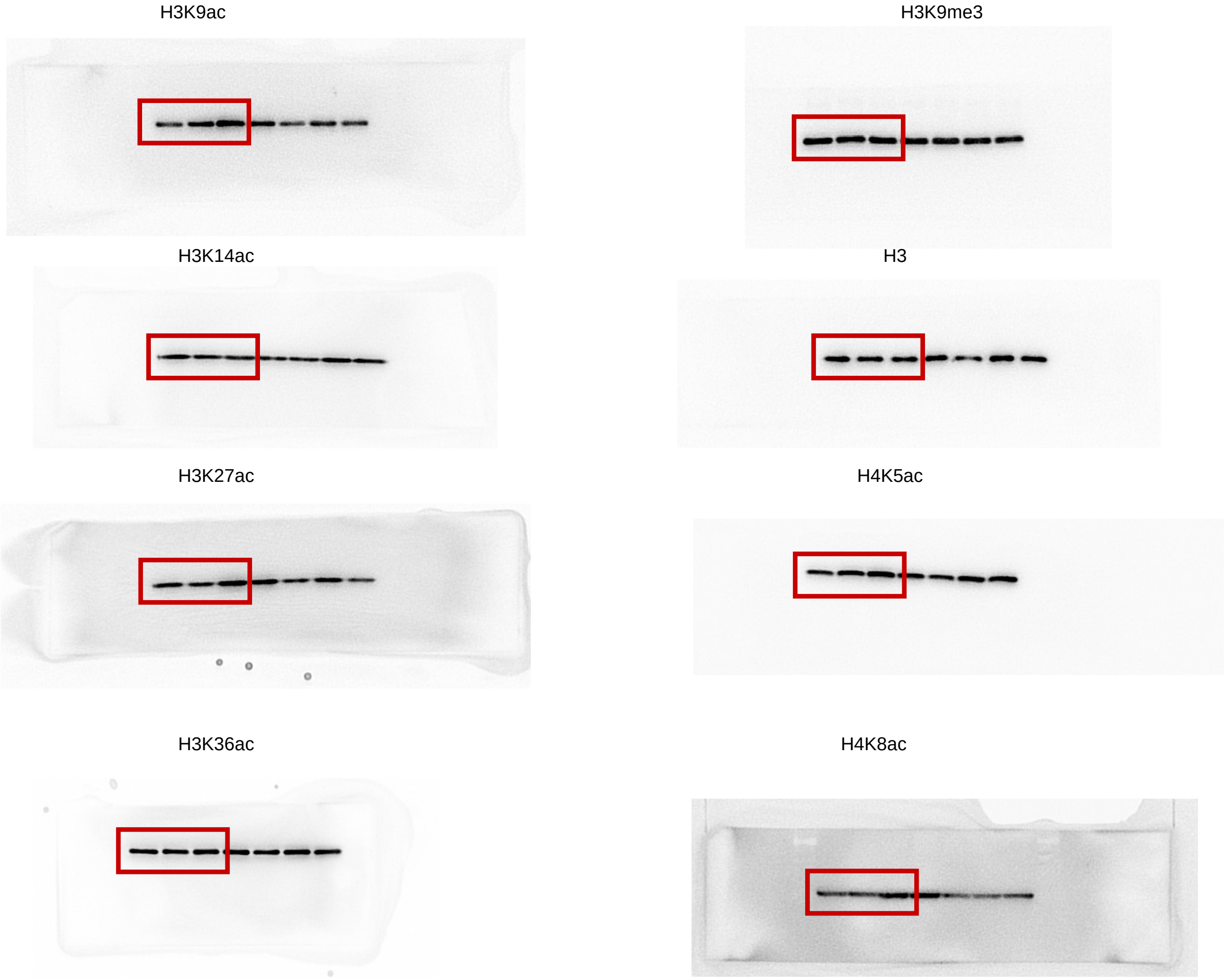

Figure3 continued

Fig.3E

H4K12ac

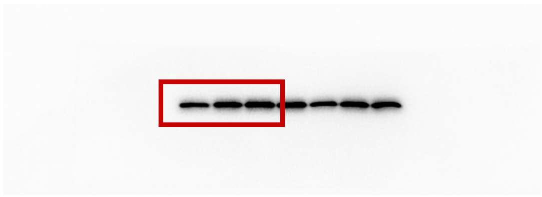

H4

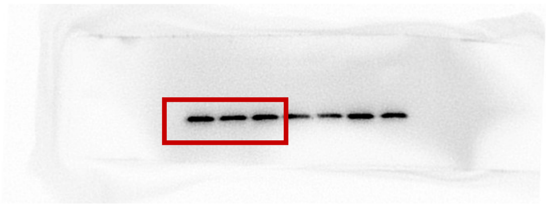

Tubulin

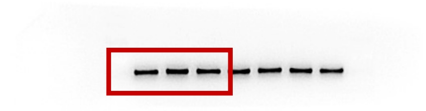

Fig.3F

	ctrl	*65K-KD*	*Ankrd11-KD*		*ctrl*	*65K-KD*	*Ankrd11-KD*		*ctrl*	*65K-KD*	*Ankrd11-KD*
H3 EXP 1	1	0.914649	0.888098587	H3K9ac EXP 1	1	1.328058	1.431758585	H3K14ac EXP 1	1	0.901672	0.880186487
H3 EXP 2	1	0.928809	0.978756712	H3K9ac EXP 2	1.000001	1.287756	1.391887073	H3K14ac EXP 2	0.999999	0.946931	1.137043437
H3 EXP 3	1	0.966065	1.020195879	H3K9ac EXP 3	1.000001	1.340038	1.524395593	H3K14ac EXP 3	1	0.942739	1.005929419
	ctrl	*65K-KD*	*Ankrd11-KD*		*ctrl*	*65K-KD*	*Ankrd11-KD*		*ctrl*	*65K-KD*	*Ankrd11-KD*
H3K27ac EXP 1	1	0.879472	1.128007454	H3K36ac EXP 1	1	0.925757	0.963044369	H3K9me3 EXP 1	1	0.985016	0.97712383
H3K27ac EXP 2	1	0.887968	1.046036332	H3K36ac EXP 2	1	0.977342	1.078651012	H3K9me3 EXP 2	1	0.983035	1.024039985
H3K27ac EXP 3	1	1.027786	1.135219189	H3K36ac EXP 3	1	0.95409	0.941651226	H3K9me3 EXP 3	1	1.036888	1.102321815

	ctrl	*65K-KD*	*Ankrd11-KD*		*ctrl*	*65K-KD*	*Ankrd11-KD*
H4 EXP 1	1	0.900818	0.978693	H4K5ac EXP 1	1	1.621422	1.938160506
H4 EXP 2	1	0.891107	0.986395	H4K5ac EXP 2	1.000001	1.26325	1.431710173
H4 EXP 3	1	1.008542	1.045243	H4K5ac EXP 3	1.000001	1.47813	1.97225848
	ctrl	*65K-KD*	*Ankrd11-KD*		*ctrl*	*65K-KD*	*Ankrd11-KD*
H4K8ac EXP 1	0.999999	1.066511	1.490638	H4K12ac EXP 1	1	0.903782	1.096902317
H4K8ac EXP 2	0.999999	1.309887	2.475352	H4K12ac EXP 2	1	1.012669	1.105374342
H4K8ac EXP 3	1.000001	1.420151	2.160946	H4K12ac EXP 3	1.000001	1.101715	1.190400354

Fig.6 A B C

		ctrl	*ANKRD11-KD*			*ctrl*	*ANKRD11-KD*			*ctrl*	*ANKRD11-KD*
	65K-EXP1	0.97513	0.508693959		65K-EXP1	0.958599	0.5145562		65K-EXP1	1.02054	0.210564383
	65K-EXP2	1.022901	0.492125745		65K-EXP2	1.011152	0.496149883		65K-EXP2	1.023374	0.247299861
CUT&Tag	65K-EXP3	1.002545	0.499118827	CUT&Tag	65K-EXP3	1.031683	0.489139142	CUT&Tag	65K-EXP3	0.957493	0.267634004
UBXN11	HDAC3-EXP1	0.985094	0.505192798	SPATA2	HDAC3-EXP1	0.946058	0.519048895	GTF2A2	HDAC3-EXP1	0.970186	0.424646226
	HDAC3-EXP2	1.019127	0.49341496		HDAC3-EXP2	1.047536	0.483793695		HDAC3-EXP2	0.961483	0.389402306
	HDAC3-EXP3	0.99608	0.501360461		HDAC3-EXP3	1.009052	0.496872763		HDAC3-EXP3	1.072021	0.424646226

		ctrl	*65K-KD*	*ANKRD11-KD*			*ctrl*	*65K-KD*	*ANKRD11-KD*			*ctrl*	*65K-KD*	*ANKRD11-KD*
H3K9ac	EXP 1	0.892753	3.038445	4.663242106	H3K9ac	EXP 1	0.936056	2.769579	4.024101422	H3K9ac	EXP 1	0.92959	2.697582	5.019927981
UBXN11	EXP 2	0.988514	2.894538	5.04667553	SPATA2	EXP 2	1.01654	2.788843	4.265357503	GTF2A2	EXP 2	1.05531	2.513446	5.214997881
	EXP 3	1.133146	2.740299	4.231981537		EXP 3	1.05093	2.746638	3.979719271		EXP 3	1.019362	2.600279	4.957685199

		ctrl	*65K-KD*	*ANKRD11-KD*			*ctrl*	*65K-KD*	*ANKRD11-KD*			*ctrl*	*65K-KD*	*ANKRD11-KD*
mRNA	EXP 1	1.017715	1.594753	2.211972322	mRNA	EXP 1	0.928302	1.769899	1.170128253	mRNA	EXP 1	0.930665	1.506639	2.307504295
UBXN11	EXP 2	1.083225	1.80667	2.26943526	SPATA2	EXP 2	0.976483	1.814619	1.291158995	GTF2A2	EXP 2	1.041984	1.472567	2.261583611
	EXP 3	0.9071	1.403796	2.304307631		EXP 3	1.103179	1.754022	1.235989654		EXP 3	1.031207	1.492089	2.227357806

Figure S3

Fig. S3A

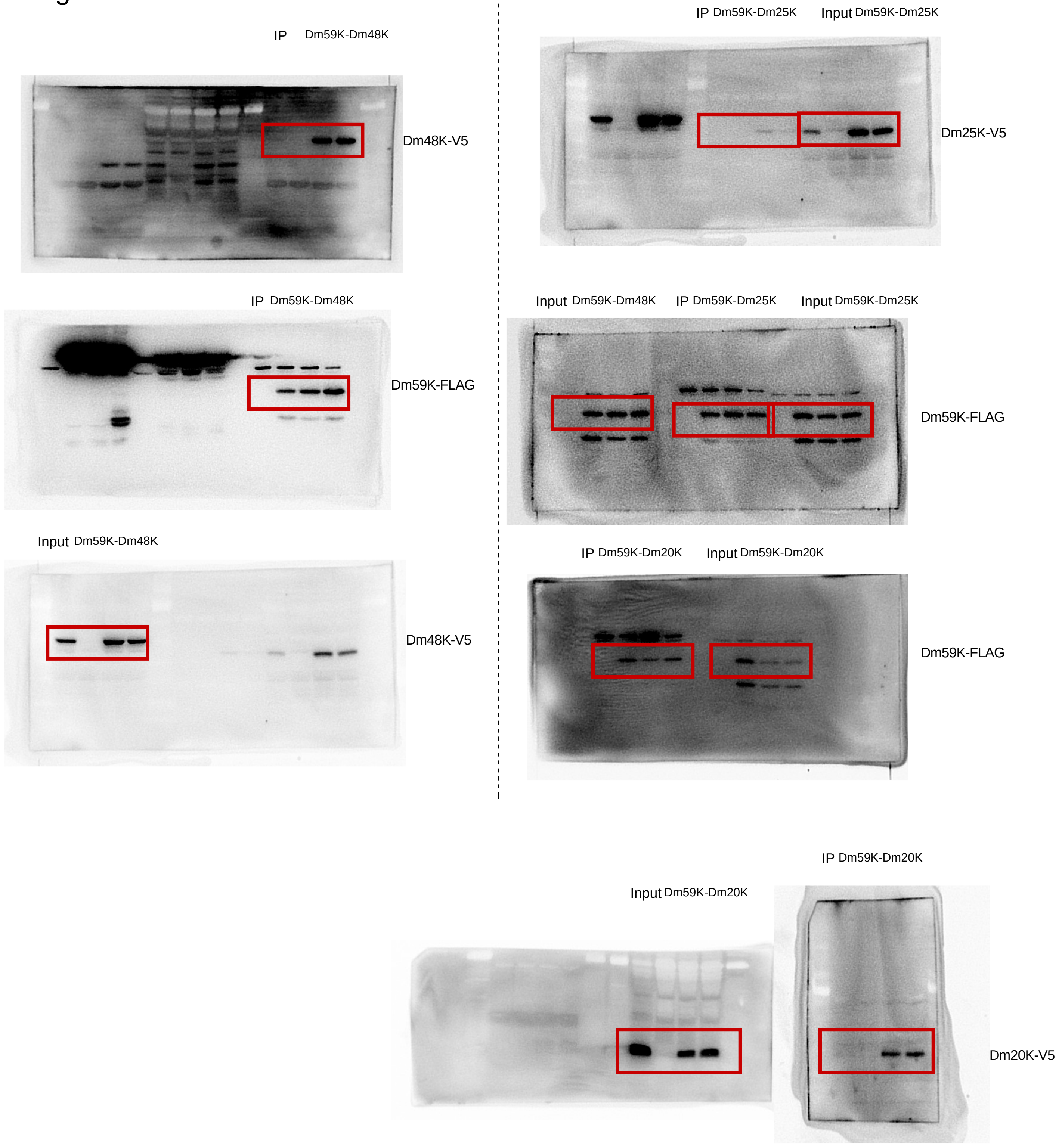

Figure S3 continue

Fig. S3B C

IP Dm48K-Dm25K Input Dm48K-Dm25K

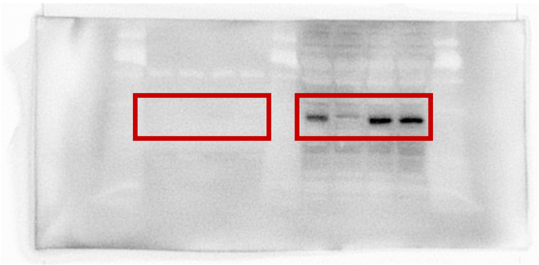

Dm25K-V5

IP Dm48K-Dm25K Input Dm48K-Dm25K

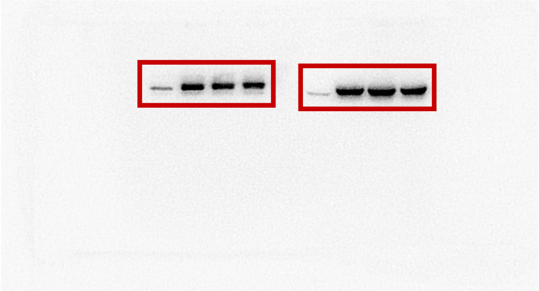

Dm48K-FLAG

IP Dm48K-Dm20K Input Dm48K-Dm20K

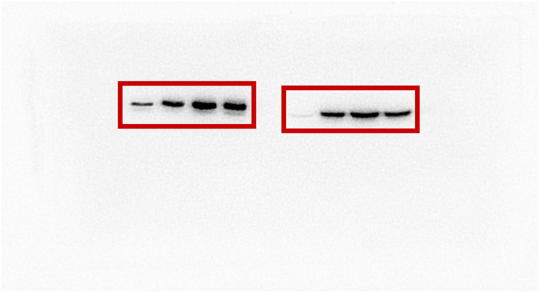

Dm48K-FLAG

IP Dm48K-Dm20K Input Dm48K-Dm20K

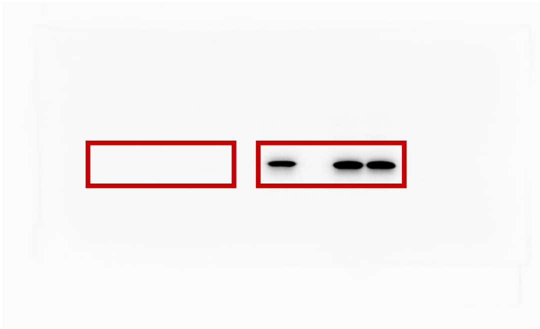

Dm20K-V5

IP Dm25K-Dm20K Input Dm25K-Dm20K

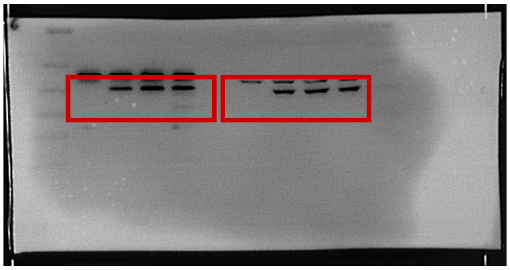

Dm25K-FLAG

IP Dm25K-Dm20K Input Dm25K-Dm20K

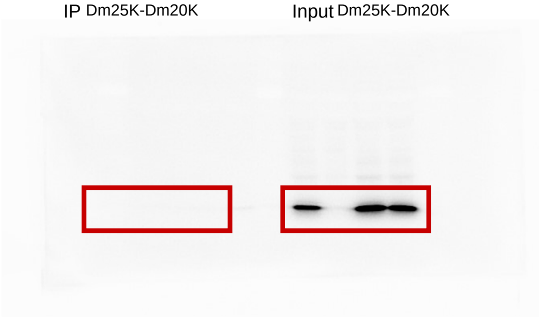

Dm20K-V5

Fig. S3D

IP Dm65K-Dm59K Input Dm65K-Dm59K

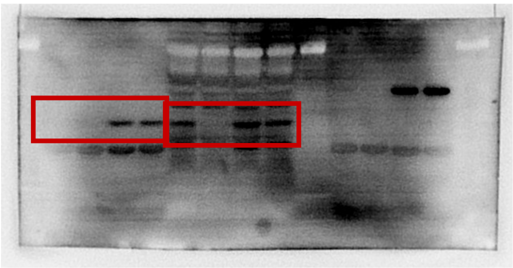

Dm59K-V5

IP Dm65K-Dm59K Input Dm65K-Dm59K

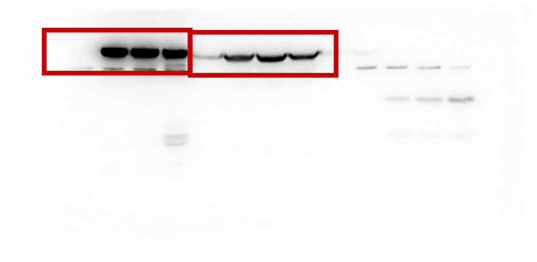

Dm65K-FLAG

Figure S5

Fig. S5A

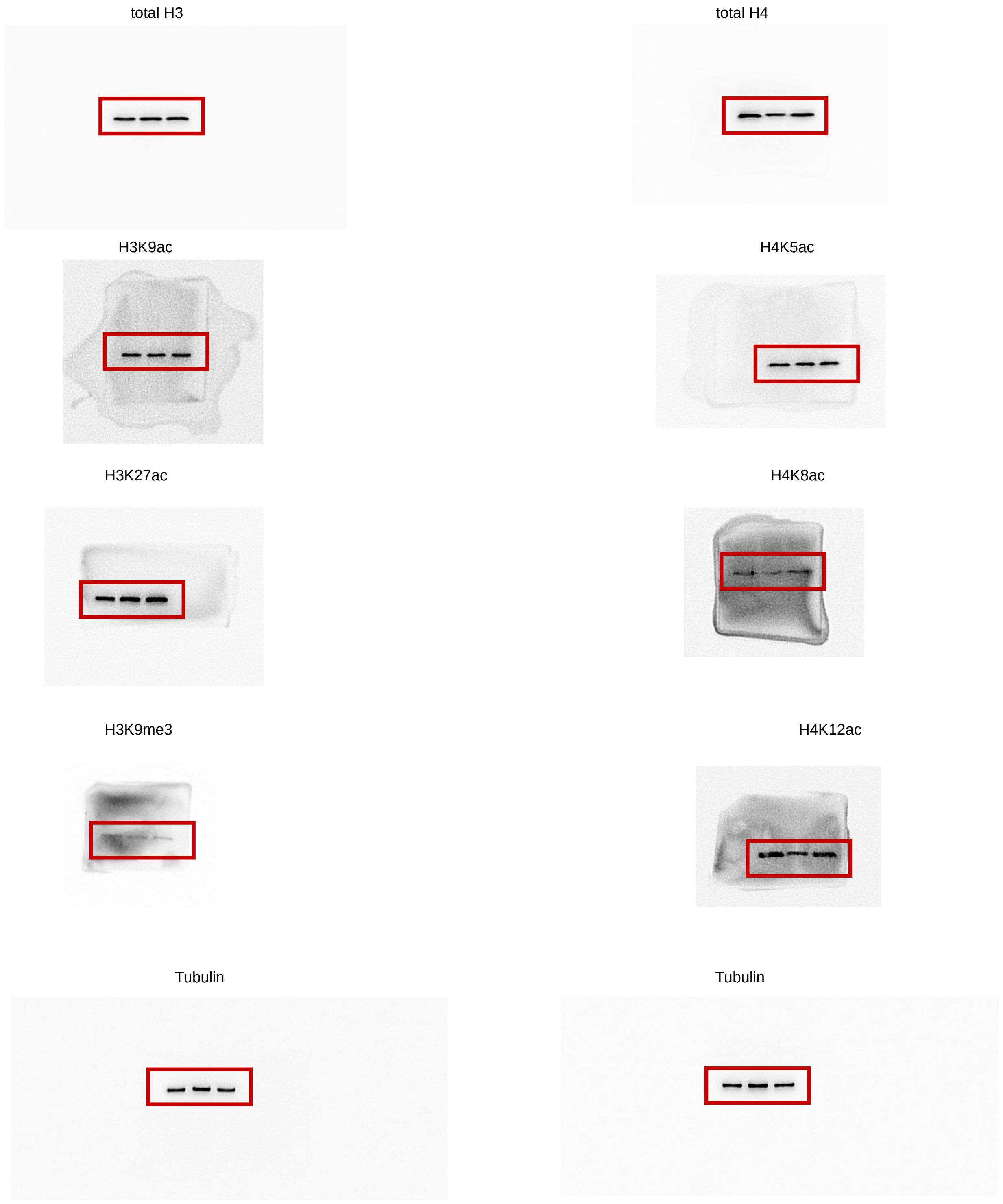

Figure S6

Fig. S6A

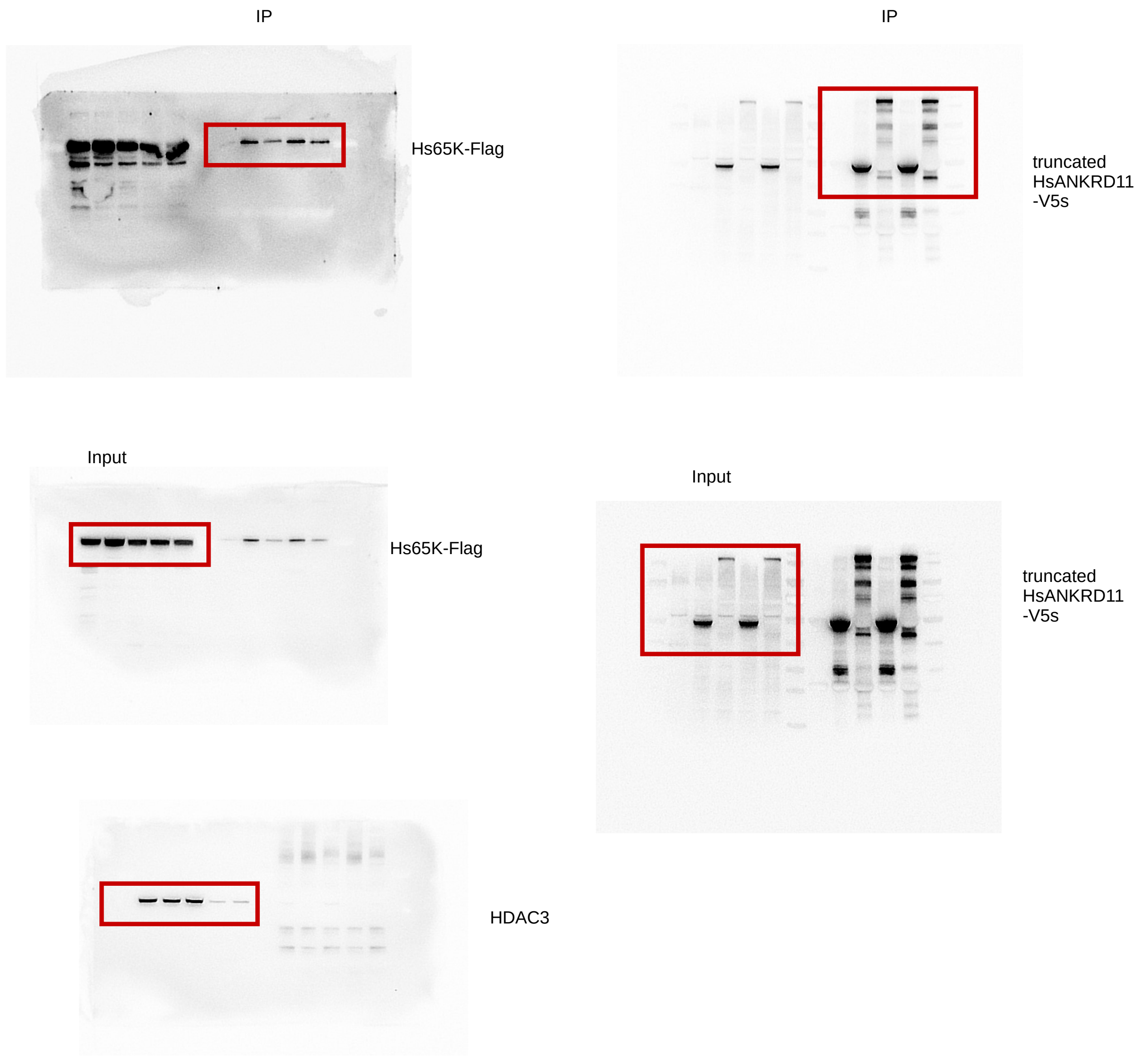

Fig. S6B

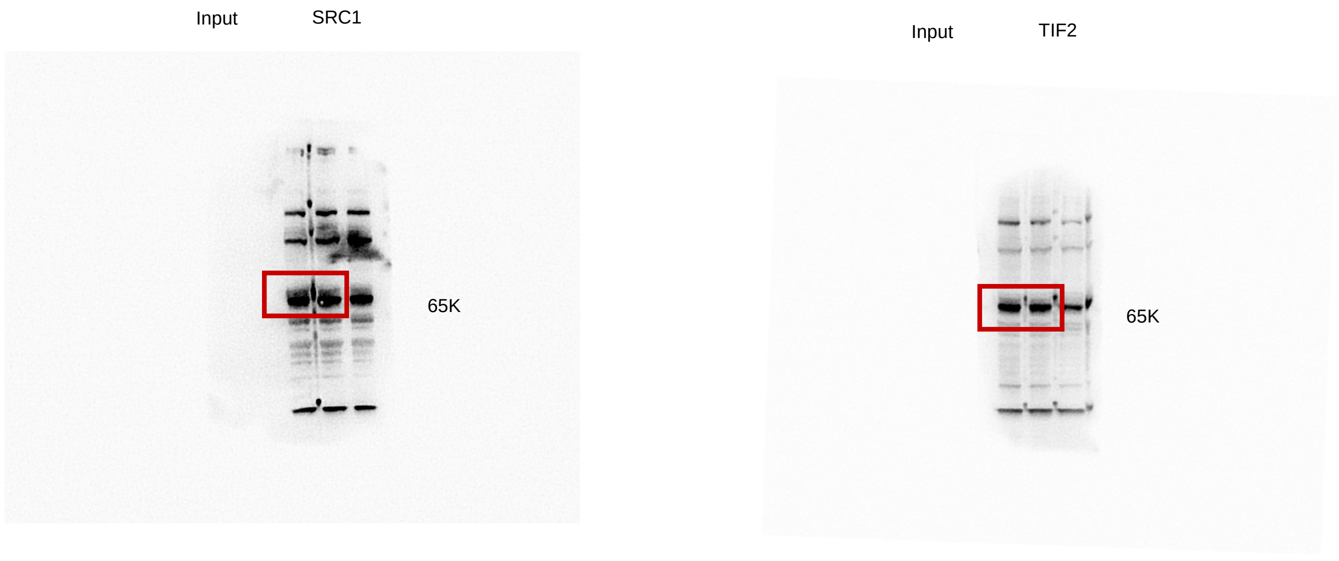

Figure S6B continue

Input RAC3

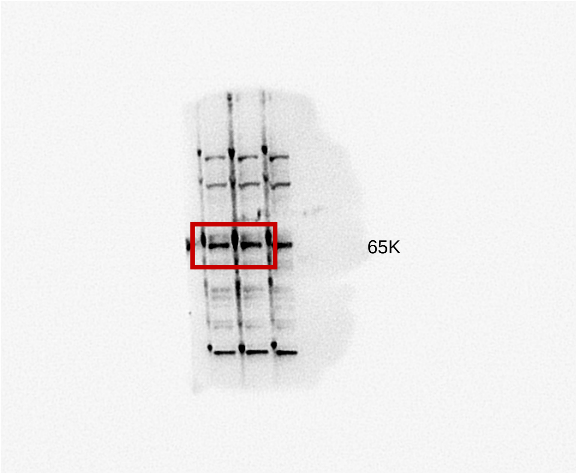

Input

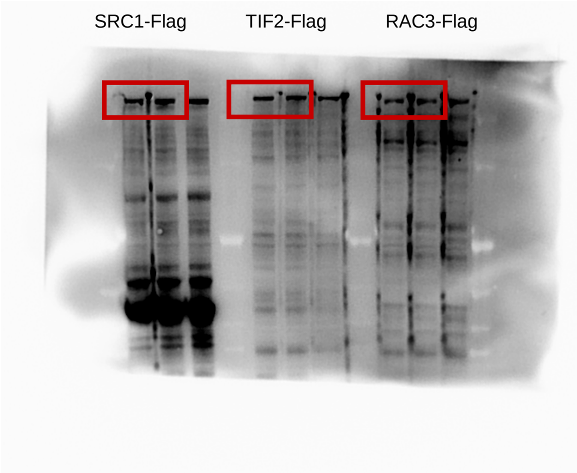

IP SRC1

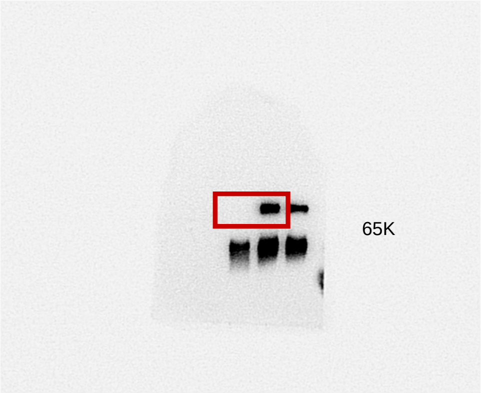

IP TIF2

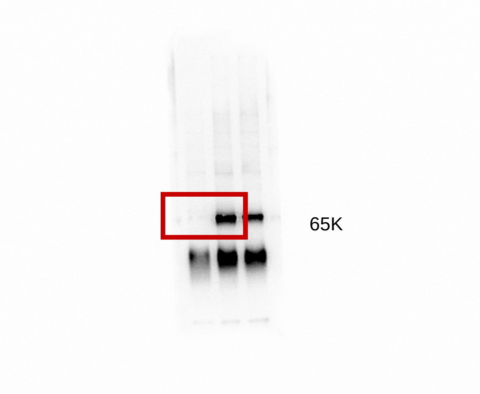

IP RAC3

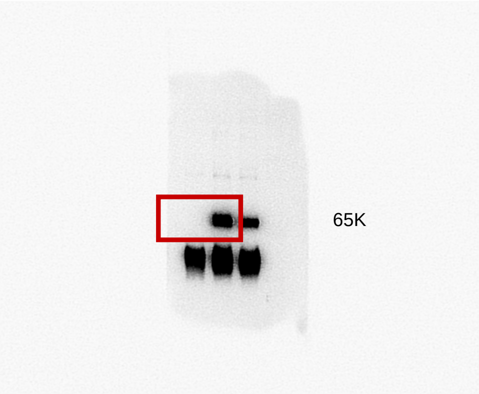

IP

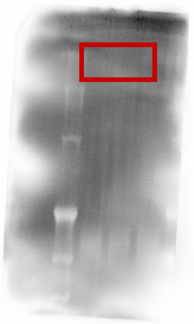

SRC1-Flag

Figure S6B continue

IP

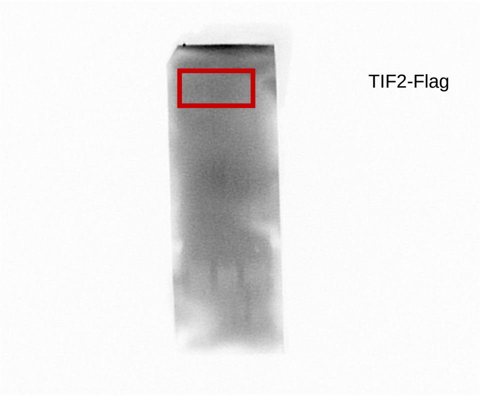

TIF2-Flag

IP

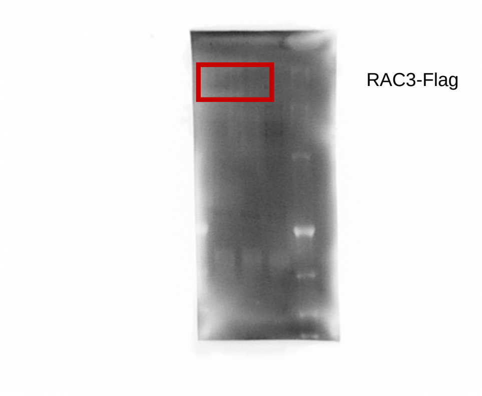

RAC3-Flag

Figure S6C

Input IP

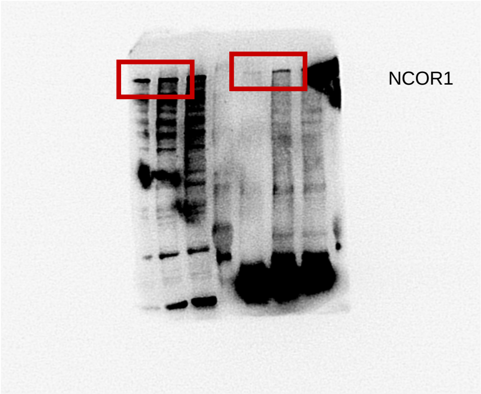

NCOR1

Input NCOR1

65K

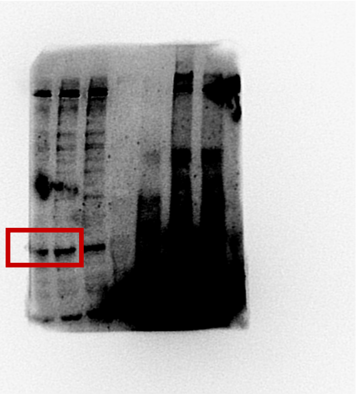

Figure S6C continue

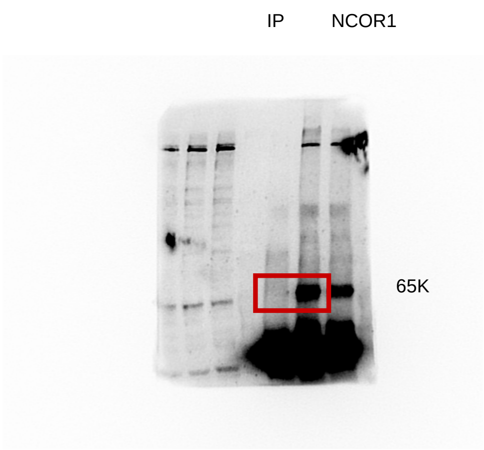

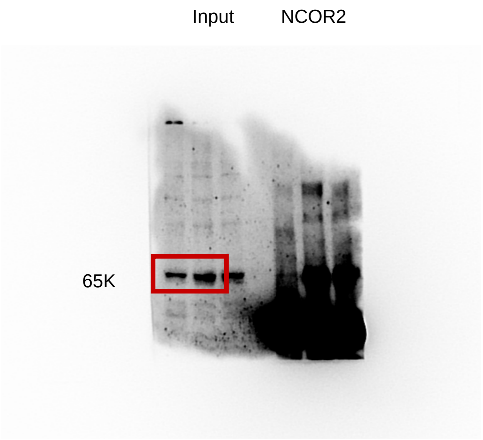

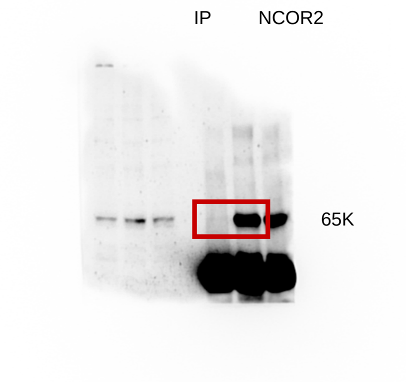

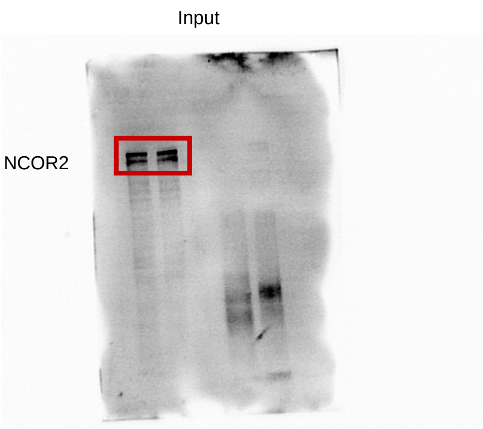

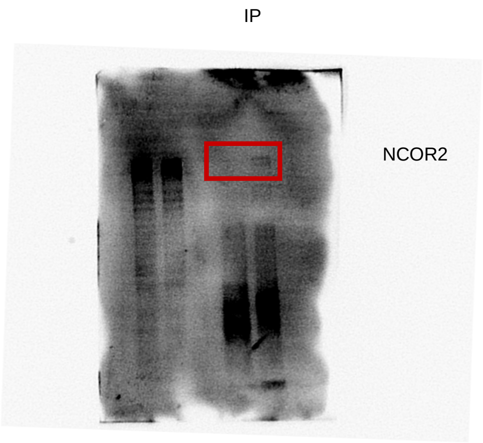

Supplement: Supplementary file 2 — Supporting Information [file ADVS-11-2307804-s001.pdf]
