# Supplementary material for: Minor Spliceosomal 65K/RNPC3 Interacts with ANKRD11 and Mediates HDAC3‐Regulated Histone Deacetylation and Transcription
Source: Adv Sci (Weinh). 2024 Jun 5;11(29):2307804. doi: 10.1002/advs.202307804 (PMC11304329; doi:10.1002/advs.202307804)
Supplement: Supplementary file 1 — Supporting Information [file ADVS-11-2307804-s002.pdf]

ADVANCED SCIENCE
Open Access

Supporting Information

for *Adv. Sci.*, DOI 10.1002/advs.202307804

Minor Spliceosomal 65K/RNPC3 Interacts with ANKRD11 and Mediates HDAC3-Regulated Histone Deacetylation and Transcription

*Chen-Hui Li, Shao-Bo Liang, Qi-Wei Huang, Zhen-Zhen Zhou, Zhan Ding, Ni Long, Kwang-Chon Wi, Liang Li, Xi-Ping Jiang, Yu-Jie Fan and Yong-Zhen Xu**

Minor Spliceosomal 65K/RNPC3 Interacts with ANKRD11 and Mediates HDAC3-regulated Histone Deacetylation and Transcription

Chen-Hui Li[1,#], Shao-Bo Liang[1,#], Qi-Wei Huang[1], Zhen-Zhen Zhou[1], Zhan Ding[1,2], Ni Long[1], Kwang-Chon Wi[1], Liang Li[1], Xi-Ping Jiang[1], Yu-Jie Fan[1] and Yong-Zhen Xu[1,*]

[1] RNA Institute, State Key Laboratory of Virology, Hubei Key Laboratory of Cell Homeostasis, College of Life Science, TaiKang Center for Life and Medical Sciences, Wuhan University, Hubei 430072, China

[2] University of Chinese Academy of Sciences, Key Laboratory of Insect Developmental and Evolutionary Biology, Center for Excellence in Molecular Plant Sciences, Chinese Academy of Sciences; Shanghai, China

[#] C. L., and S. L. contributed equally to this work.

[*] Correspondence: Yong-Zhen Xu, +86 (27) 68789348, Yongzhen.Xu@whu.edu.cn

Supplementary Figure S1-8

Li *et al*_Fig S1

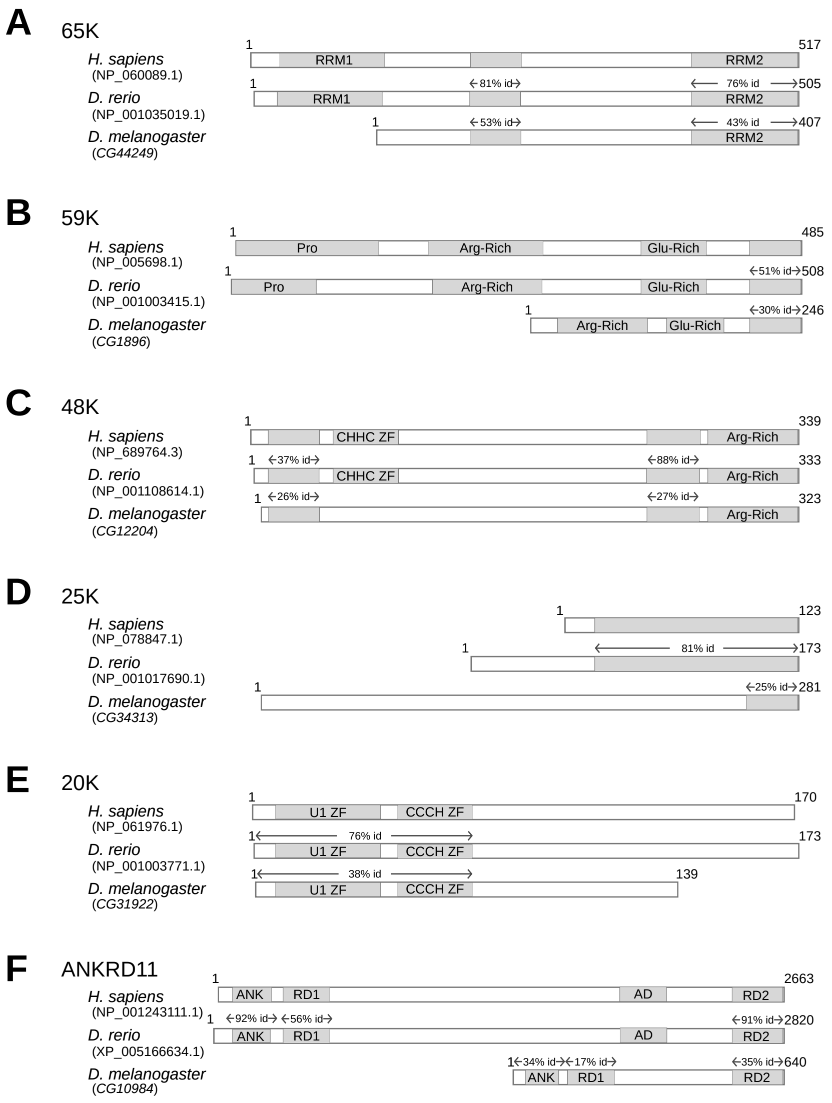

Figure S1. Analyses of homologous domains in the Dm65K co-purified proteins.
Domains are aligned for the top MS-identified proteins that are associated with Dm65K, including 65K (**A**), 59K (**B**), 48K (**C**), 25K (**D**), 20K (**E**) and ANKRD11 (**F**). Homologous proteins from three species (human, zebrafish and *Drosophila*) are analyzed for each gene.

Li *et al*_Fig S2

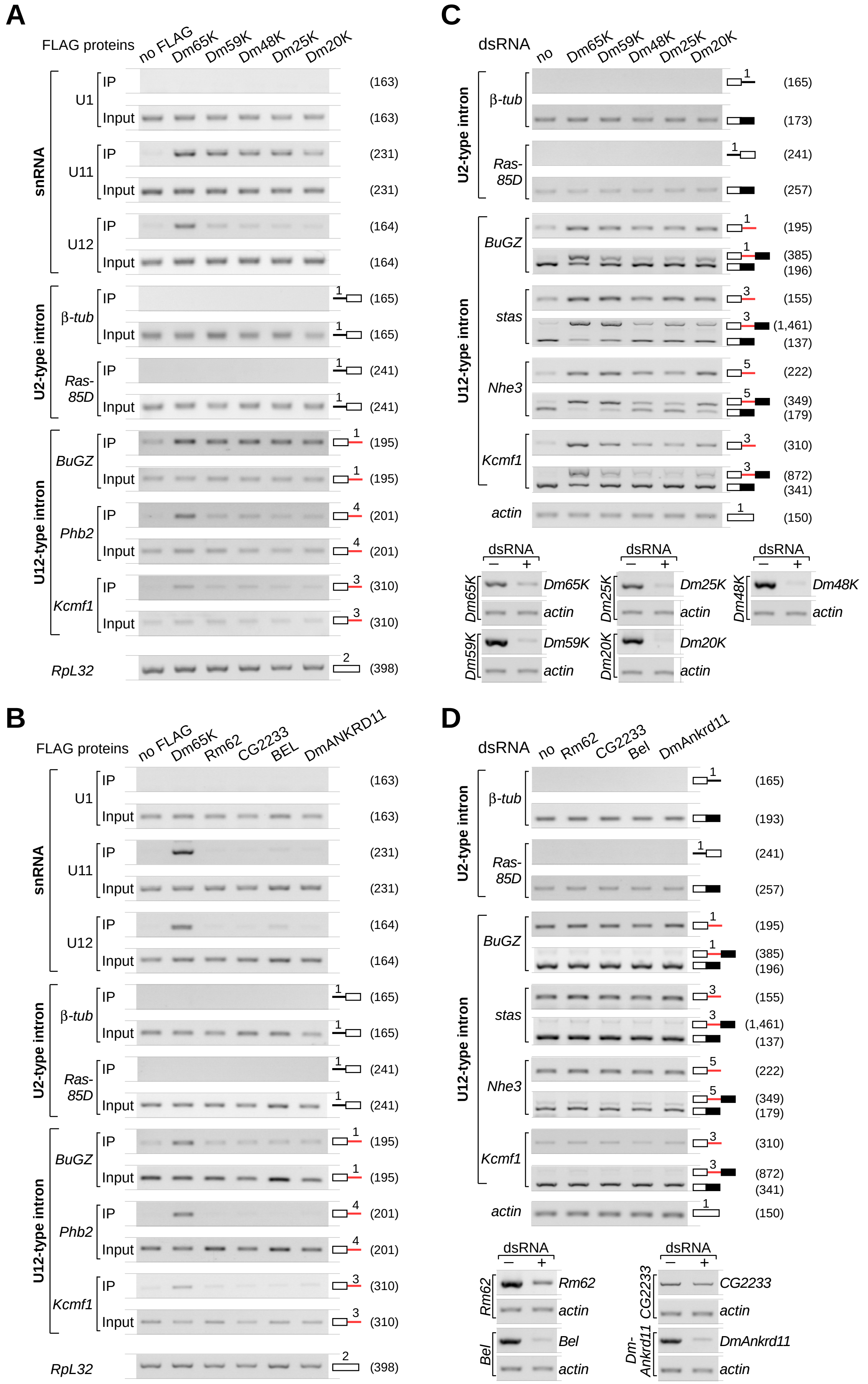

Figure S2. Three newly identified Dm65K-associated proteins are components of the *Drosophila* minor spliceosome.
(**A**) The newly identified three *Drosophila* minor spliceosomal proteins with FLAG tag co-IPed minor snRNAs and minor intron-containing pre-mRNAs, but not majors. (**B**) Four of the co-purified proteins did not co-IP minor snRNAs or pre-mRNAs from MIGs. These proteins were Flag-tagged and the FLAG epitope was used for co-IP. (**C**) Knockdown of the newly identified *Drosophila* minor spliceosomal proteins by dsRNA-mediated RNAi resulted in defective splicing of minor introns but not major introns. (**D**) Knockdown of the four proteins did not result in defective minor splicing. The length of PCR amplicons is indicated. Red lines, minor introns; black lines, major introns.

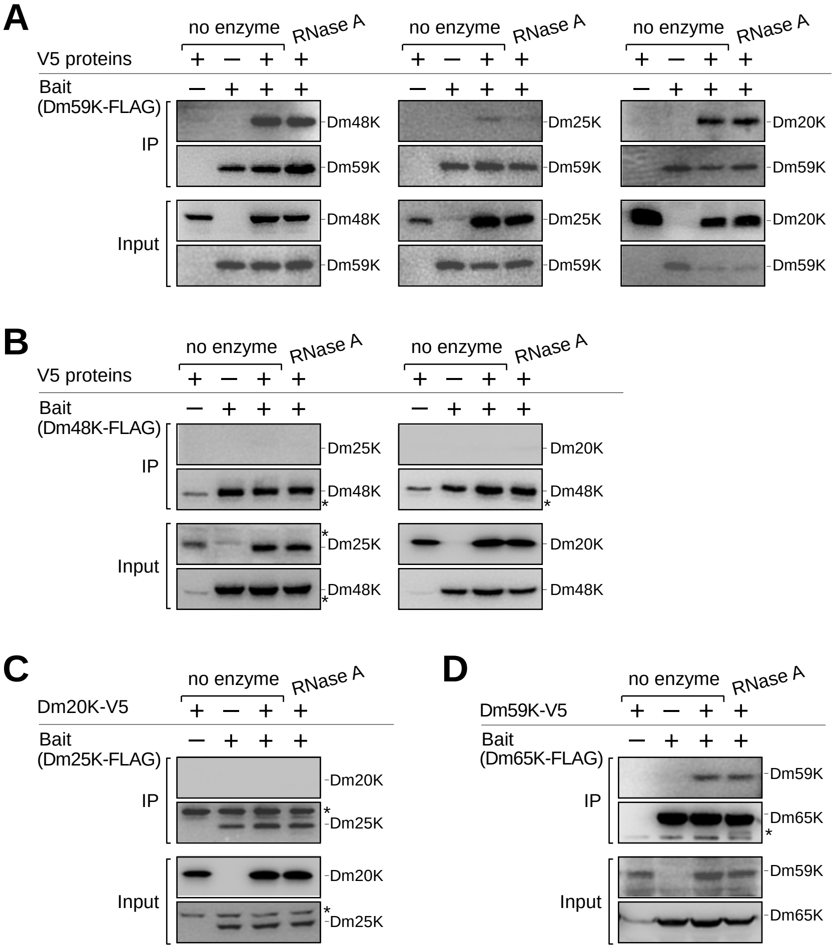

Figure S3. Protein-protein interactions between the minor spliceosomal components.
(**A**) Dm48K and Dm20K, but not Dm25K, were efficiently co-IPed by the FLAG-tagged Dm59K. (**B**) Dm25K and Dm20K were not co-IPed by the FLAG-tagged Dm48K. (**C**) Dm20K was not co-IPed by the FLAG-tagged Dm25K. (**D**) The Dm65K-Dm59K interaction is RNA-independent. Asterisks, non-specific signals.

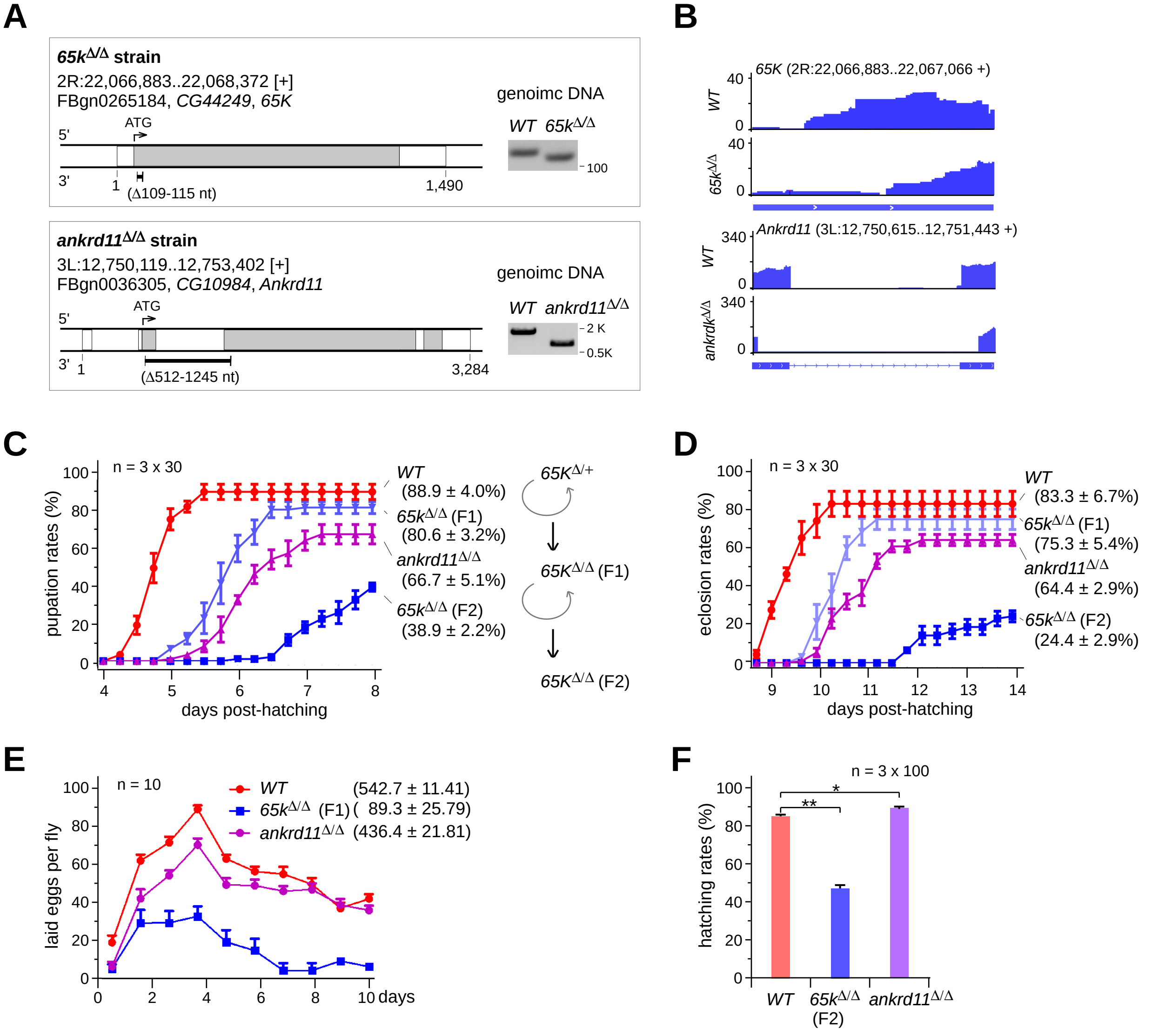

Figure S4. Deletion of *Dm65k* and *Dmankrd11* results in developmental defects of Drosophila.

(**A**) Schematics for construction of the Drosophila deletion strains *65k*$^{\Delta/\Delta}$ and *ankrd11*$^{\Delta/\Delta}$ using a CRISPR/Cas9-mediated system. Deletion regions of the two genes are indicated, and the strains are confirmed by PCR and sequencing of genomic DNAs. (**B**) Reads mapping from the RNA-seq data demonstrate that the *65k*$^{\Delta/\Delta}$ and *ankrd11*$^{\Delta/\Delta}$ mutants are successfully constructed. The *65k*$^{\Delta/\Delta}$ mutant exhibited significantly decreased rates of pupation (**C**), eclosion (**D**), impaired fecundity (**E**), and hatching rates (**F**). Statistical data are shown as mean ± SD. p values were calculated using two-sided unpaired t-test; *, $p < 0.05$; **, $p < 0.01$.

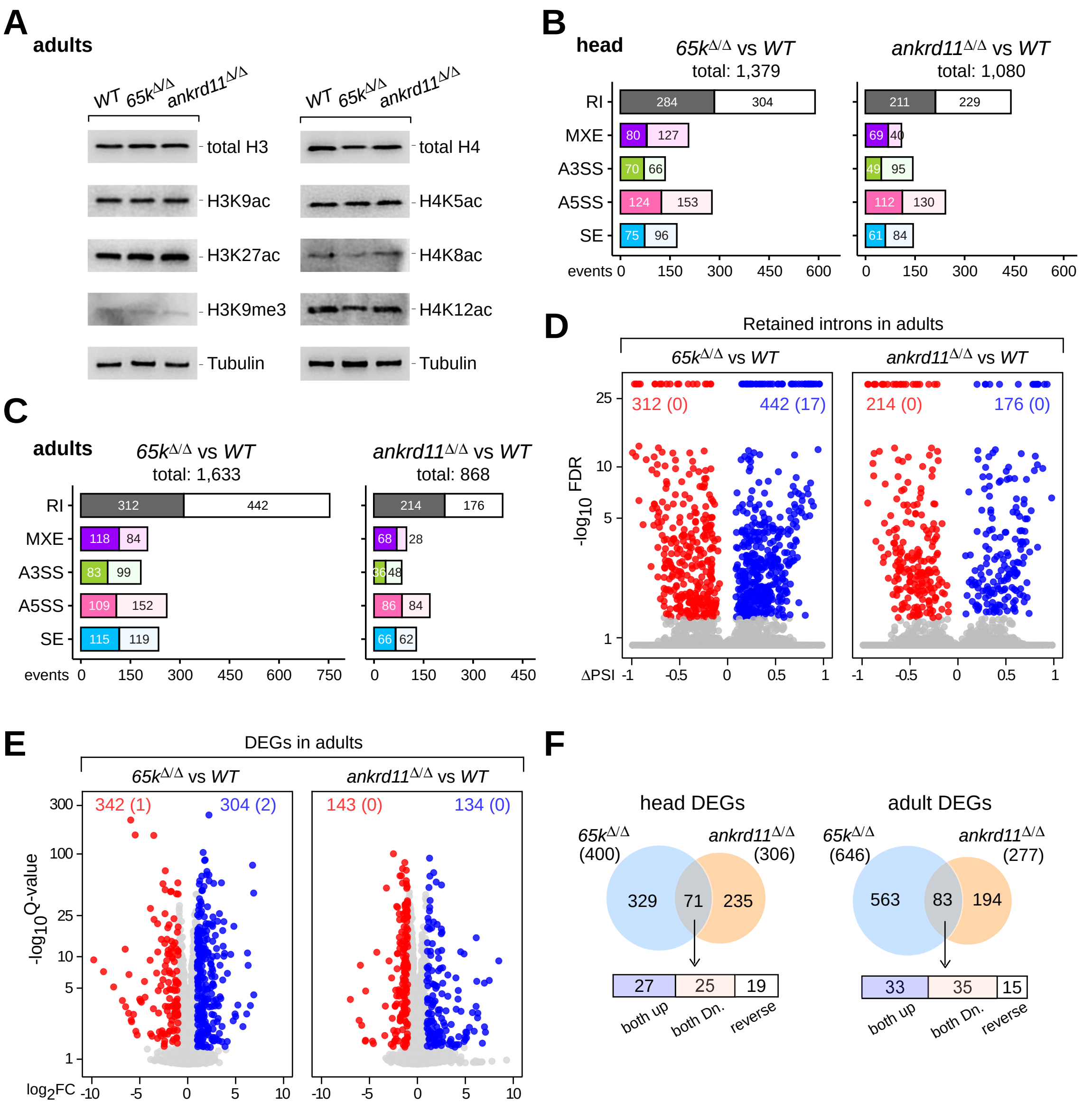

Figure S5. Alternative splicing changes and differentially-expressed genes in the *65k*$^{\Delta/\Delta}$ and *ankrd11*$^{\Delta/\Delta}$ mutants.
(**A**) Unlike in the head, histone acetylations are not changed in adults of the two *Drosophila* mutants. Statistics of the changed alternative splicing events in the head (**B**) and in the adults (**C**) of the two *Drosophila* mutants. Plot analyses of the percent of spliced-in (PSI) changes of the retained introns (**D**) and differentially-expressed genes (DEGs) (**E**) in the adults of the two mutants. (**F**) Overlapping of DEGs between the two mutants.

Li *et al*_Fig S6

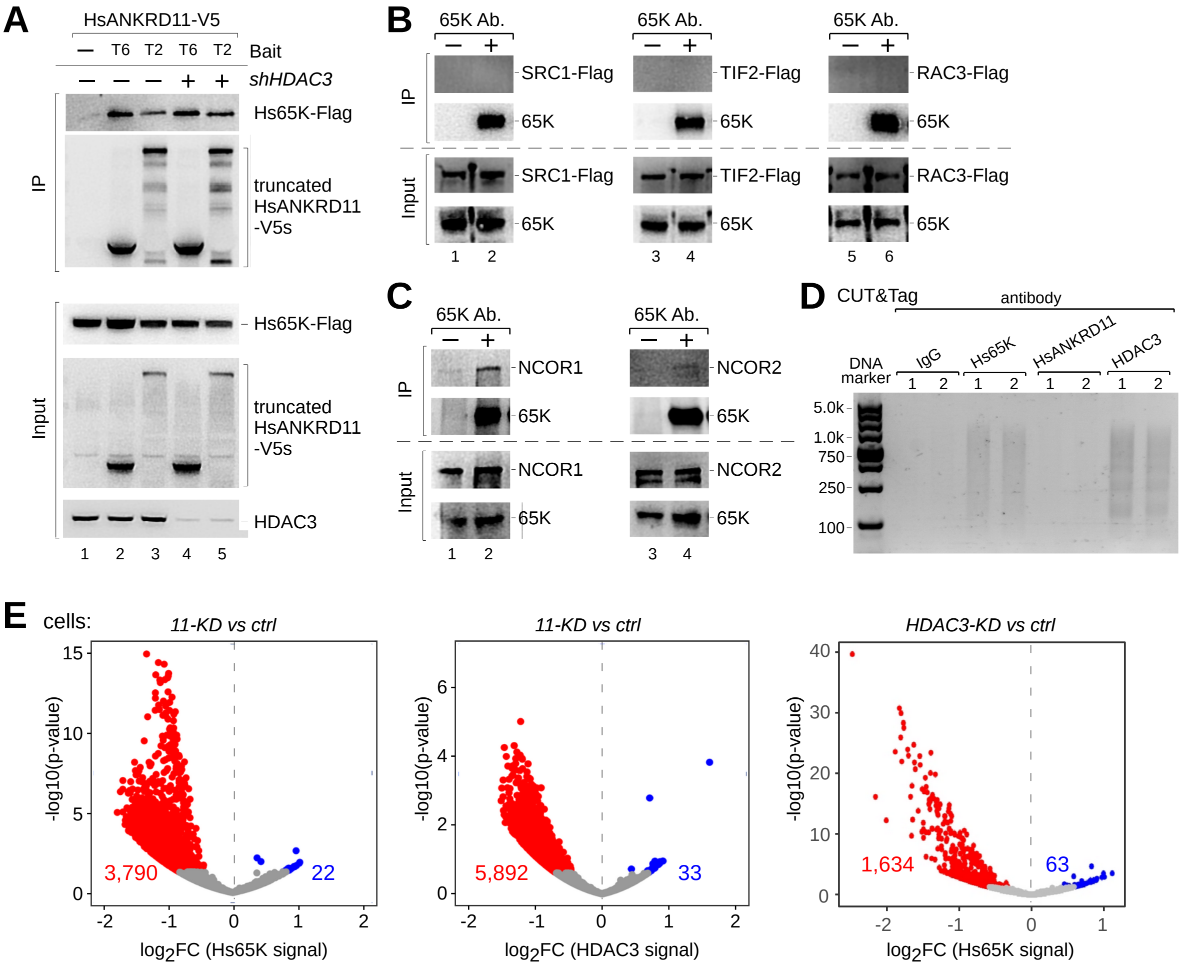

Figure S6. Knockdown of *HsANKRD11* significantly decreases the chromatin-bindings of Hs65K and HDAC3.
(**A**) Knockdown of *HDAC3* by shRNA did not affect the interaction between Hs65K and HsANKRD11. Truncated HsANKRD11 proteins T6 and T2 were used for co-IP of Hs65K in 293T cells. (**B**, **C**) Co-immunoprecipitation using the 65K antibody indicated that 65K did not interact with the tested three components (SRC1, TIF2 and RAC3) from the p160 coactivators/nuclear receptor complex (**B**), but interacts with NOCR1, a component from the NCOR&SMRT complex (**C**). (**D**) CUT&Tag assays were performed using three antibodies, in which the antibody against HsANKRD11 did not work. (**E**) In the *HsANKRD11* knockdown cells, many chromatin-bindings of Hs65K and HDAC3 were significantly decreased. Similarly, many chromatin-bindings of Hs65K were significantly decreased when *HDAC3* was knocked down.

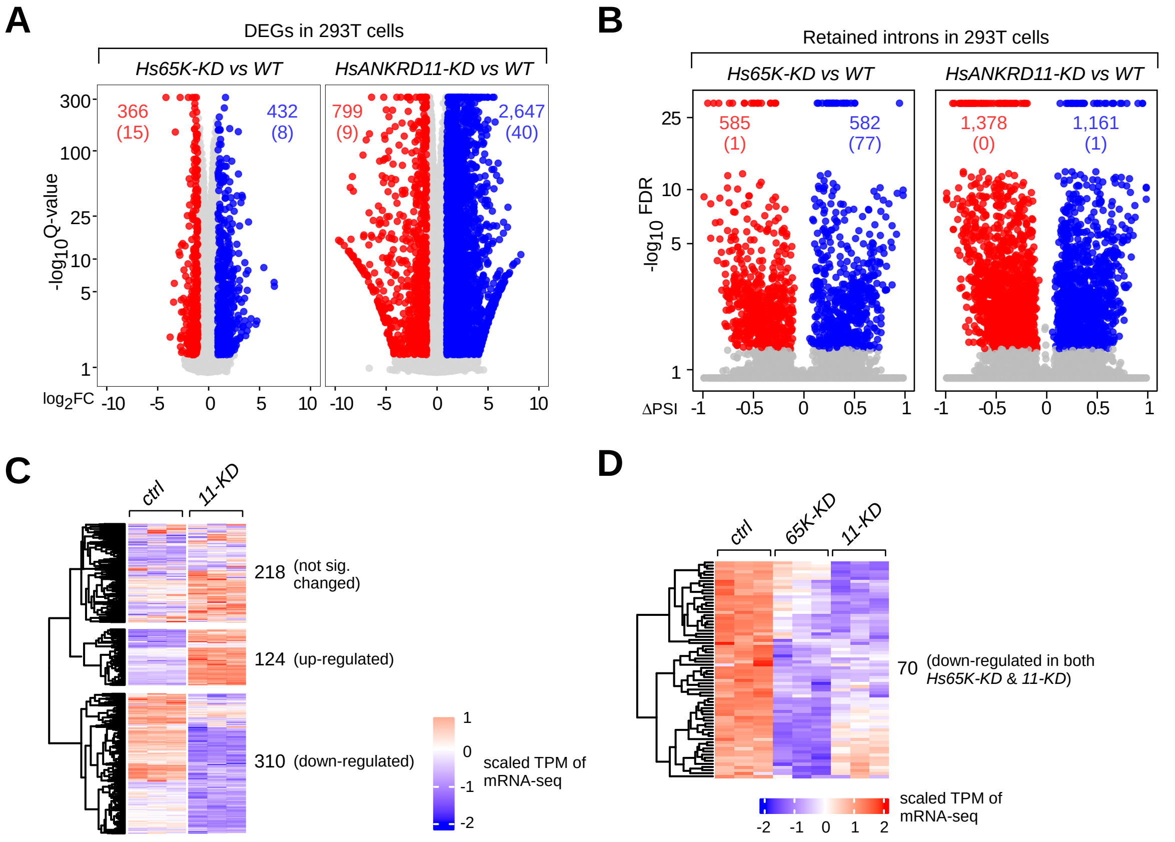

Figure S7. Differentially-expressed genes and changed alternative splicing events in the *Hs65K-KD* and *HsANKRD11* cells.
(**A**) Analyses of the differentially-expressed genes (DEGs) in the human *Hs65K-KD* and *HsANKRD11-KD* cells. Numbers in brackets are counting of MIGs. (**B**) Plot analyses of the PSI changes of the retained introns in the two KD cells. Numbers in brackets are counting of minor introns. (**C**) Expression changes of genes in the two KD cells, in which the gene should have at least one significantly decreased binding of 65K or HDAC3 and binding of the other one was not significantly increased in the *HsANKRD11-KD* cells. (**D**) Seventy genes are down-regulated in both knockdown cells due to the decreased binding of Hs65K and HDAC3 and reduced deacetylation of H3K9 and at their chromatin domains with the colocalized triple-peaks.

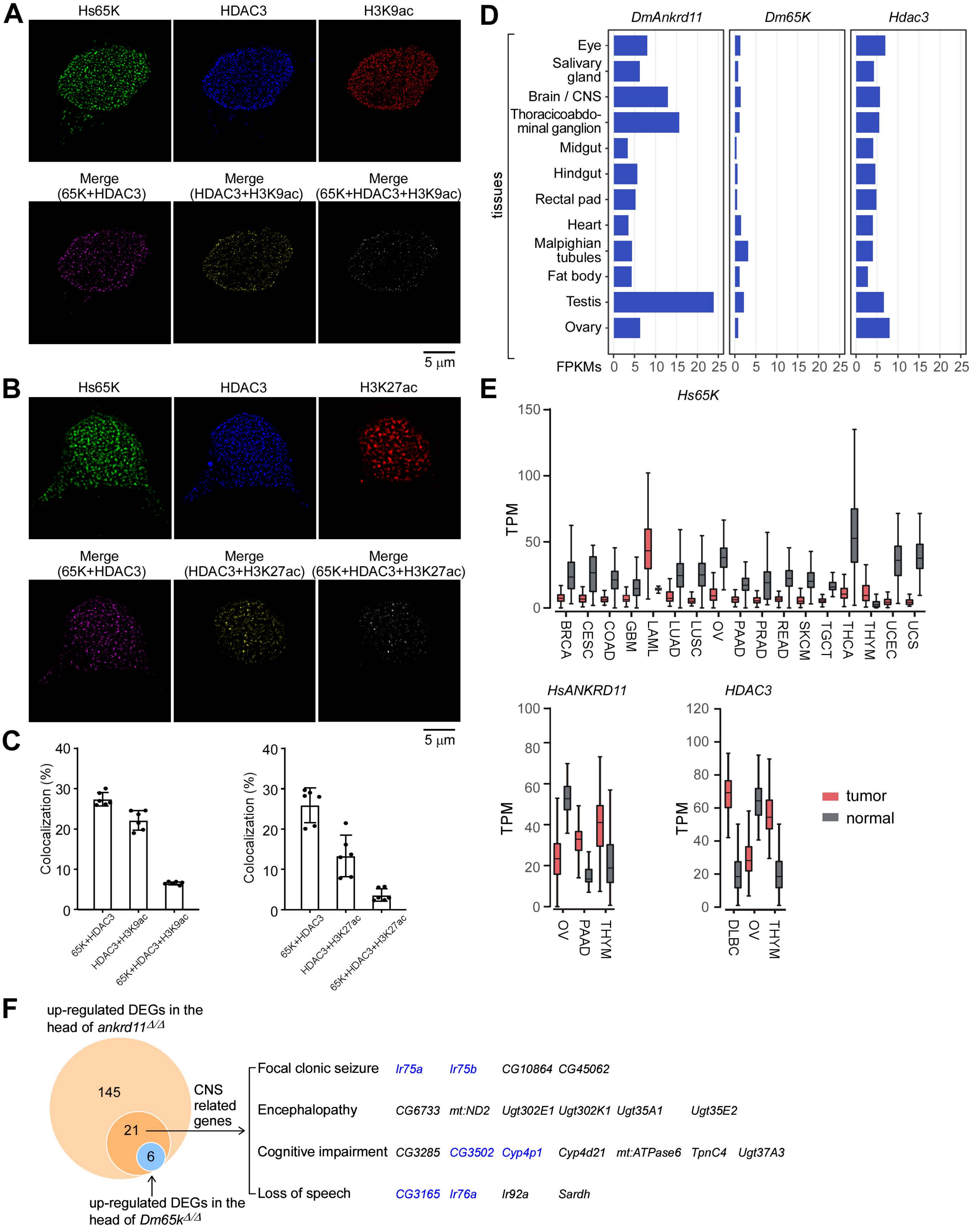

Figure S8. *Ankrd11* is highly expressed in nervous systems.
(**A**, **B, C**) Colocalization of the Hs65K/HDAC3 complex and the H3K9ac/H3K27ac signals in human cells. Immunostaining signals of 65K in green, HDAC3 in blue, and H3K9ac (**A**) or H3K27ac (**B**) in red are indicated. The percentages of colocalization are derived from the merged signals to the corresponding total Hs65K or HDAC3 signals (**C**). (**D**) The expression of *DmAnkrd11* is relatively higher in nervous systems than in other tissues. Expression data of the *DmAnkrd11*, *Dm65K*, and *DmHdac3* genes are retrieved from FlyAtlas 2. (**E**) The expressions of *Hs65K*, *HsANKRD11* and *HDAC3* are significantly changed in tumors *vs* normal tissues. Expression data are from GEPIA2. (**F**) Many of the up-regulated DEGs are shared in the head of *65k*$^{\Delta/\Delta}$ and *ankrd11*$^{\Delta/\Delta}$ mutants, of which 21 are CNS-related genes.

Supplementary Table S1-7

Table S1. Top MS-identified peptides from the Dm65K co-purified proteins

	gene	MW (KD)	protein	peptides	intensity
minor-specific proteins	CG44249	45.4	Dm65K	ALEQLQELSESLDKPLCISVFGPR, ESISKPESGASTSK, IGQSLQTNSHFYNQVLHLMNR, LLLAPASEEAR, LPETLVPPPIHPER, LSTSALEALPVYK, LTVQKPSFDAPK, MNLEPPFEK, MPCSLDELAQLIR, QHVGTQTEVIPSESESELESDDGEQK, QMLQQAAQLASHTKPK, SVDEQQLR, VLLEYADPEEAAK	5.1E+09
	CG1896	28.3	Dm59K	DAASILETFDLLEK, GWVLPPTKPTAQWIGYR, IQQVISSLGQLEQQSCGAGR, KVASLESQWK, PTAQWIGYR, RVQEETQAGHVK, STATLLDELDQVPGQQVVDEQK, STWDSYISYCGR, VASLESQWK, VQEETQAGHVK,	7.5E+08
	CG34313	32.8	Dm25K	AMLASTSEELRR, CFGLLNVDTNQPIDDQR, DGLSTLTVVLHQHEPTIAQLK, ELGIENAATLK, QPVAVASSTGELVHSAQR, RQPVAVASSTGELVHSAQR	3.5E+08
	CG12204	38.6	Dm48K	HPQFQDNTAFAEVVGETR, KHPQFQDNTAFAEVVGETR, LFVTASR, LLIYEHVLQHTK, LLPAASFDDLQAGLDASQR, SGEDVDEKR, SKPTLNEELHQLVSLQMQALEK,	2.5E+08
	CG31922	16.3	Dm20K	FTHYSGDNLR, GLPPSLQPINPEK, KGLPPSLQPINPEK, SYYCDYCCCFLK	1.1E+08
Common proteins in both spliceosomes	CG1249	13.5	SmD2	HCNMVLENVK, NNTQVLINCR, NPLATAAGK, QEEEEFNTGPLSVLTQSVK, SELTPEELAR	3.1E+08
	CG9742	8.5	SmG	AVTGILR	3.2E+07
	CG18591	11.1	SmE	GDNITLIQNVSPTK	1.9E+07
	CG6322	61.6	DmU4-U6-60K	AGRMVEMQKK	8330700
	CG3949 (hoip)	13.9	SNU13	SQITSIQQEIER	7142100
	CG4849	110.6	U5-116KD	GHVTQDAPVSGSPIYTIK	2606000
Others	Rm62	62.5	RNA helicase	EANQEINPALENLAR, ELAQQIQQVATEFGSSSYVR, FGGGGGFGDR, FGGGGGGGDYHGIR, GTSFAFFTK, SNILVATDVAAR, TLLSDIYDTSESPGK	5.4E+07
	CG2233	46.5		GTCELSIGSPAPVVISNFGSQR, SGTGQDEFVIIGHNSPFYR, YSALCYDLAAFQLK	3E+07
	CG9748 (bel)	85.0	DEAD-box helicase 3 X-linked	HVINFDLPSDVEEYVHR, VGSTSENITQTILWVYEPDKR	1.4E+07
	CG16901 (sqd)	33.1	HNRNPAB	IFVGGLTTEISDEEIK, VSAADEHIINSK	9878100
	CG13849 (Nop56)	54.8	NOP56	NLTQDQLEDLEK	9707100
	CG10984	71.2	Ankrd11	DLIEAHTVER, IVIPQQNCSIDTEGNVLR	8912600
	CG10206 (nop5)	57.0	NOP58	LIAHAGSLINLAK, SEVFTYQPEADNTLNVK	7864400
	CG8963	58.4	PAIP1	LSDEPVFYGPDGR	2228800
	CG6143 (Pep)	75.6	CDKN1A interacting zinc finger protein 1	EGAGDGEGAEGDEAEGEEAKEGEEAADETK	0

Table S2. Differential alternative splicing events and differentially-expressed genes in *Drosophila* mutants.

1) List of DASs in *Drosophila* heads of $Dm65k^{\Delta/\Delta}$ *vs WT*

2) List of DASs in *Drosophila* heads of $DmAnkrd11^{\Delta/\Delta}$ *vs WT*

3) List of DASs in *Drosophila* adults of $Dm65k^{\Delta/\Delta}$ *vs WT*

4) List of DASs in *Drosophila* adults of $DmAnkrd11^{\Delta/\Delta}$ *vs WT*

5) List of DEGs in *Drosophila* heads of $Dm65k^{\Delta/\Delta}$ *vs WT*

6) List of DEGs in *Drosophila* heads of $DmAnkrd11^{\Delta/\Delta}$ *vs WT*

7) List of DEGs in *Drosophila* adults of $Dm65k^{\Delta/\Delta}$ *vs WT*

8) List of DEGs in *Drosophila* adults of $DmAnkrd11^{\Delta/\Delta}$ *vs WT*

1) List of DASs in *Drosophila* heads of *Dm65k*$^{\Delta/\Delta}$ *vs* *WT*

A3SS											
	**FlyBase ID**	**chr**	**strand**	**longExonStart_0base**	**longExonEnd**	**shortES**	**shortEE**	**flankingES**	**flankingEE**	**FDR**	**ΔPSI**
1	FBgn0061492	chr3L	+	6095092	6095343	6095112	6095343	6094986	6095017	0.00351	0.484
2	FBgn0036324	chr3L	-	12901246	12902047	12901246	12902027	12902111	12902737	1.4E-05	-0.617
3	FBgn0036765	chr3L	+	17879949	17880064	17879976	17880064	17879095	17879248	0.01362	0.348
4	FBgn0013342	chr3L	-	1636817	1637272	1636817	1637269	1637558	1637612	5.1E-09	0.653
5	FBgn0267849	chr3L	+	21446160	21446375	21446197	21446375	21445833	21446023	0.04348	0.091
6	FBgn0267376	chr3R	-	10866808	10866976	10866808	10866960	10867971	10868290	0	0.669
7	FBgn0267376	chr3R	-	10866808	10866976	10866808	10866960	10868438	10868642	0	0.845
8	FBgn0262166	chr2R	-	16195716	16196000	16195716	16195970	16196066	16196333	1.5E-08	-0.456
9	FBgn0082582	chr3R	+	30548420	30548749	30548451	30548749	30546405	30546587	0	-0.481
10	FBgn0028703	chr2L	-	6678911	6679262	6678911	6679082	6679985	6680114	0.00373	0.213
11	FBgn0038716	chr3R	+	19603778	19604256	19603833	19604256	19603374	19603412	8.8E-09	0.69
12	FBgn0051973	chr2L	-	34719	35212	34719	34912	38534	38731	0.00392	-0.146
13	FBgn0038652	chr3R	-	18768871	18769116	18768871	18769113	18793457	18793833	0.02604	-0.216
14	FBgn0003386	chr2L	+	3726678	3729145	3726696	3729145	3724883	3725196	0.00661	0.234
15	FBgn0032202	chr2L	+	10264548	10264633	10264551	10264633	10264124	10264264	0.00391	0.112
16	FBgn0032172	chr2L	-	9960449	9961617	9960449	9961614	9961728	9962059	4.6E-06	-0.352
17	FBgn0038897	chr3R	+	21742720	21743987	21742741	21743987	21742427	21742666	0.0049	0.631
18	FBgn0034858	chr2R	+	23386941	23387612	23387006	23387612	23386520	23386622	2.4E-08	-0.525
19	FBgn0032940	chr2L	-	21308287	21309091	21308287	21308995	21310000	21310050	2.5E-05	0.501
20	FBgn0040964	chr2L	+	8997722	8998544	8997761	8998544	8997604	8997668	9.2E-05	0.515
21	FBgn0015222	chr3R	-	30387463	30387602	30387463	30387550	30387774	30387860	7.9E-07	-0.179
22	FBgn0015222	chr3R	-	30387463	30387642	30387463	30387550	30387774	30387860	1.6E-07	-0.19
23	FBgn0015222	chr3R	-	30387463	30387642	30387463	30387602	30387774	30387860	0.023	0.112
24	FBgn0028496	chr2R	-	18496746	18496905	18496746	18496868	18497841	18498026	0	0.504
25	FBgn0039406	chr3R	-	26058979	26059054	26058979	26059051	26059234	26059267	0.00046	0.643
26	FBgn0034075	chr2R	+	16112195	16112530	16112201	16112530	16111982	16112141	0	0.3
27	FBgn0034002	chr2R	+	15221898	15222425	15222043	15222425	15221727	15221823	0.00508	0.359
28	FBgn0034903	chr2R	-	23755494	23757166	23755494	23756551	23757414	23757494	0.00025	-0.216
29	FBgn0031857	chr2L	-	6768845	6769127	6768845	6768968	6769397	6769736	0	-0.36
30	FBgn0085399	chr2R	-	22037391	22037719	22037391	22037716	22039355	22039431	6E-14	-0.313
31	FBgn0041150	chr2L	-	4925828	4926350	4925828	4926346	4928243	4928393	2.4E-08	0.345
32	FBgn0261283	chr3L	+	19794563	19794699	19794673	19794699	19793934	19794159	1E-05	0.667
33	FBgn0011603	chr2L	-	4475394	4475508	4475394	4475410	4476841	4477022	2.2E-05	0.627
34	FBgn0011603	chr2L	-	4475394	4475508	4475394	4475496	4476841	4477022	9.1E-12	1
35	FBgn0031636	chr2L	+	4841135	4841465	4841162	4841465	4840883	4840983	0	1
36	FBgn0035688	chr3L	-	6169568	6170031	6169568	6169942	6170116	6171169	0.00917	-0.611
37	FBgn0052549	chrX	-	18409049	18409178	18409049	18409079	18409883	18410144	0.00923	-0.611
38	FBgn0052549	chrX	-	18409049	18409209	18409049	18409079	18409883	18410144	0.00069	-0.719
39	FBgn0001105	chrX	+	15860744	15861189	15861139	15861189	15860114	15860322	9.1E-05	-0.477
40	FBgn0001105	chrX	+	15861057	15861153	15861151	15861153	15860114	15860322	0.01982	-0.566
41	FBgn0001105	chrX	+	15861057	15861189	15861139	15861189	15860114	15860322	0.00936	-0.554
42	FBgn0030364	chrX	+	12019586	12019758	12019627	12019758	12018639	12019325	0	0.307
43	FBgn0267376	chr3R	-	10866808	10866994	10866808	10866960	10867971	10868290	0	0.912
44	FBgn0267376	chr3R	-	10866808	10866994	10866808	10866960	10868438	10868642	0	0.854
45	FBgn0267252	chr2L	+	9256892	9257116	9256895	9257116	9256562	9256696	0.02655	0.337
46	FBgn0266487	chr2R	+	12618214	12618311	12618235	12618311	12617986	12618140	2.1E-05	-0.324
47	FBgn0026374	chrX	+	15790233	15790386	15790246	15790386	15788896	15789112	0.00453	0.339
48	FBgn0036331	chr3L	+	13008622	13008873	13008654	13008873	13008284	13008567	8.4E-09	0.421
49	FBgn0263102	chr2R	+	10561685	10561954	10561695	10561954	10557887	10558533	4.4E-07	0.171
50	FBgn0262149	chr3R	+	7128702	7129067	7128940	7129067	7128463	7128647	0.00014	0.703
51	FBgn0082582	chr3R	+	30548420	30548749	30548451	30548749	30546404	30546598	1E-05	-0.134
52	FBgn0082582	chr3R	+	30548278	30548749	30548420	30548749	30546405	30546587	0	-0.963
53	FBgn0082582	chr3R	+	30548278	30548749	30548451	30548749	30546405	30546587	0	-0.791
54	FBgn0082582	chr3R	+	30548283	30548749	30548420	30548749	30546405	30546587	0	-0.993
55	FBgn0082582	chr3R	+	30548283	30548749	30548451	30548749	30546405	30546587	0	-0.808
56	FBgn0082582	chr3R	+	30548303	30548749	30548420	30548749	30546405	30546587	0	-0.968
57	FBgn0082582	chr3R	+	30548303	30548749	30548451	30548749	30546405	30546587	0	-0.795
58	FBgn0082582	chr3R	+	30548399	30548749	30548420	30548749	30546405	30546587	0	-0.998
59	FBgn0082582	chr3R	+	30548399	30548749	30548451	30548749	30546405	30546587	0	-0.744
60	FBgn0263981	chr2R	-	16291123	16291222	16291123	16291186	16291301	16291474	4.7E-13	-0.849
61	FBgn0027356	chr2R	+	12609717	12609876	12609729	12609876	12609228	12609636	0.02155	-0.772
62	FBgn0263395	chr2R	+	19178941	19179109	19179059	19179109	19178737	19178872	1E-05	-0.809
63	FBgn0263395	chr2R	+	19179376	19179858	19179498	19179858	19178737	19178872	2.2E-05	0.297
64	FBgn0000056	chr2L	+	14616057	14616437	14616215	14616437	14615551	14615643	0	0.458
65	FBgn0000056	chr2L	+	14616128	14616437	14616215	14616437	14615551	14615643	8.6E-09	0.359
66	FBgn0000056	chr2L	+	14616215	14616437	14616282	14616437	14615551	14615643	0.023	-0.286
67	FBgn0013334	chr3R	+	15935783	15935912	15935839	15935912	15935118	15935584	1.3E-05	-0.289
68	FBgn0031220	chr2L	-	120199	120360	120199	120265	120420	120742	2.8E-06	-0.747
69	FBgn0032726	chr2L	-	18953403	18953790	18953403	18953778	18954271	18954431	0.01498	-0.334
70	FBgn0032946	chr2L	+	21382041	21382330	21382099	21382330	21380132	21380361	0.00011	0.312
71	FBgn0032946	chr2L	+	21382099	21382330	21382104	21382330	21380132	21380361	0.01425	-0.351
72	FBgn0039667	chr3R	+	29495587	29495801	29495595	29495801	29494781	29494885	0.01555	-0.624
73	FBgn0039667	chr3R	+	29495587	29495801	29495645	29495801	29494781	29494885	0.04589	-0.389
74	FBgn0036768	chr3L	+	17889239	17889462	17889330	17889462	17887704	17887790	2.6E-05	0.894
75	FBgn0036768	chr3L	+	17889313	17889462	17889330	17889462	17887704	17887790	0	0.879
76	FBgn0031213	chr2L	+	73331	73585	73389	73585	72386	72977	0.01854	0.667
77	FBgn0053978	chr4	-	158499	158699	158499	158617	159221	159712	0.04193	0.667
78	FBgn0030976	chrX	+	18906084	18906134	18906088	18906134	18900730	18900960	0.00124	-0.708
79	FBgn0031228	chr2L	+	155493	155784	155513	155784	155334	155429	0.02936	0.547
80	FBgn0028546	chr2L	-	13642019	13642226	13642019	13642192	13642295	13642396	0.01314	0.563
81	FBgn0033054	chr2R	-	6038507	6038721	6038507	6038625	6040081	6040334	0.00032	0.667
82	FBgn0011739	chr3R	-	30800037	30800313	30800037	30800177	30806287	30806619	0.00016	-0.523
83	FBgn0011739	chr3R	-	30800037	30800427	30800037	30800177	30806287	30806619	1.8E-06	-0.606
84	FBgn0011739	chr3R	-	30800037	30800448	30800037	30800177	30806287	30806619	9.5E-05	-0.508
85	FBgn0052816	chrX	-	333564	333936	333564	333897	334666	334801	0.00055	0.365
86	FBgn0278608	chrX	+	16336266	16336743	16336347	16336743	16334300	16334413	1.7E-05	0.551
87	FBgn0285913	chr3R	+	14290596	14290820	14290705	14290820	14289273	14289449	0.02243	-0.428
88	FBgn0285913	chr3R	+	14290620	14290820	14290705	14290820	14289273	14289449	0.04589	-0.417
89	FBgn0015221	chr3R	+	30389415	30389442	30389436	30389442	30389198	30389328	5.2E-05	-0.226
90	FBgn0040532	chr3R	+	8822405	8822666	8822441	8822666	8822007	8822125	0.01977	-0.174
91	FBgn0028527	chr2L	+	13670835	13671247	13671018	13671247	13670437	13670597	0.00653	-0.301
92	FBgn0027279	chrX	+	22504108	22504333	22504207	22504333	22503368	22503740	7E-07	-0.741
93	FBgn0086690	chr3L	-	15073306	15073330	15073306	15073324	15074324	15074492	4.6E-05	0.43
94	FBgn0015222	chr3R	-	30387463	30387587	30387463	30387550	30387774	30387860	0.00987	-0.123
95	FBgn0015222	chr3R	-	30387463	30387713	30387463	30387550	30387774	30387860	2.7E-07	-0.179
96	FBgn0015222	chr3R	-	30387463	30387716	30387463	30387550	30387774	30387860	4.4E-07	-0.178
97	FBgn0015222	chr3R	-	30387463	30387713	30387463	30387602	30387774	30387860	0.00375	0.142
98	FBgn0015222	chr3R	-	30387463	30387716	30387463	30387602	30387774	30387860	0.00197	0.146
99	FBgn0032479	chr2L	-	13187032	13187417	13187032	13187349	13187572	13189618	0.00071	0.667
100	FBgn0053558	chr2R	-	6934348	6934709	6934348	6934363	6934805	6935108	0.01592	0.122
101	FBgn0083124	chr2R	+	9097061	9097312	9097241	9097312	9096925	9096991	3.5E-08	0.329
102	FBgn0031118	chrX	-	20509365	20509754	20509365	20509498	20510518	20510694	3.5E-07	0.952
103	FBgn0034515	chr2R	-	20539511	20539676	20539511	20539664	20539744	20539813	0	0.242
104	FBgn0259110	chrX	-	15972431	15972733	15972431	15972582	15972812	15973067	0.01977	-0.571
105	FBgn0028496	chr2R	-	18496746	18496929	18496746	18496868	18497841	18498026	1.5E-09	0.539
106	FBgn0028496	chr2R	-	18496746	18496937	18496746	18496868	18497841	18498026	2.6E-10	0.548
107	FBgn0028496	chr2R	-	18496746	18497156	18496746	18496868	18497841	18498026	2.3E-08	0.497
108	FBgn0050295	chr2R	+	20876869	20877312	20877254	20877312	20876474	20876621	0.0134	0.223
109	FBgn0016726	chr2R	+	21294007	21294159	21294050	21294159	21293849	21293874	1.7E-07	-0.563
110	FBgn0034605	chr2R	-	21217015	21217285	21217015	21217174	21217727	21217779	1.9E-06	0.495
111	FBgn0045842	chr2L	-	15257266	15257703	15257266	15257586	15257798	15258105	0.04986	0.425
112	FBgn0034075	chr2R	+	16114560	16114866	16114771	16114866	16114034	16114472	2.4E-12	-0.195
113	FBgn0034051	chr2R	-	15937678	15938125	15937678	15937879	15938446	15938978	0.02243	-0.124
114	FBgn0034903	chr2R	-	23756363	23757166	23756363	23756542	23757414	23757494	1.1E-12	-0.416
115	FBgn0034903	chr2R	-	23756363	23757166	23756363	23756545	23757414	23757494	4.8E-11	-0.44
116	FBgn0038744	chr3R	+	19892516	19894015	19892532	19894015	19890688	19890828	4.5E-06	-0.777
117	FBgn0036493	chr3L	+	15306504	15306798	15306512	15306798	15305576	15305679	0	1
118	FBgn0005614	chr2R	+	9754929	9755312	9755120	9755312	9753696	9753977	0.0096	0.542
119	FBgn0037146	chr3L	+	21976277	21976459	21976297	21976459	21972001	21972242	0.00426	0.562
120	FBgn0024811	chr4	+	211574	211764	211627	211764	210581	210680	0	-0.818
121	FBgn0024811	chr4	+	211591	211764	211627	211764	210581	210680	6E-14	-0.526
122	FBgn0024811	chr4	+	211594	211764	211627	211764	210581	210680	2E-09	-0.367
123	FBgn0051999	chr4	-	217404	217846	217404	217532	218247	218380	0.00291	-0.762
124	FBgn0015521	chr2L	+	2856742	2857029	2856768	2857029	2856630	2856672	2E-12	-0.806
125	FBgn0039817	chr3R	-	31099105	31099214	31099105	31099182	31099273	31099321	0	0.988
126	FBgn0022361	chr4	+	560634	560820	560702	560820	560470	560583	0.02447	-0.693
127	FBgn0034479	chr2R	+	19982283	19982703	19982297	19982703	19981069	19981275	0.00107	0.344
128	FBgn0262109	chr3L	+	227517	227894	227542	227894	225725	227459	6.9E-11	0.303
129	FBgn0025741	chr4	-	1014317	1014620	1014317	1014526	1014777	1014908	0.00069	0.685
130	FBgn0031857	chr2L	-	6770590	6770718	6770590	6770709	6770885	6770968	0	-1
131	FBgn0031857	chr2L	-	6770590	6770718	6770590	6770709	6772551	6772755	0	-0.997
132	FBgn0058006	chr2L	-	22720281	22720407	22720281	22720388	22755856	22756349	0.00084	-0.75
133	FBgn0039993	chr2R	+	1595743	1595931	1595765	1595931	1590939	1591017	0.00029	-0.311
134	FBgn0085399	chr2R	-	22037391	22037719	22037391	22037716	22039477	22039559	0	-1
135	FBgn0085399	chr2R	-	22037391	22037719	22037391	22037716	22039481	22039559	4.3E-05	-0.819
136	FBgn0263397	chr2R	+	14277280	14279642	14279242	14279642	14276129	14276526	0.00029	0.373

A5SS											
	**FlyBase ID**	**chr**	**strand**	**longExonStart_0base**	**longExonEnd**	**shortES**	**shortEE**	**flankingES**	**flankingEE**	**FDR**	**ΔPSI**
1	FBgn0020762	chr2L	+	4333912	4334439	4333912	4334104	4336449	4336957	0	-0.254
2	FBgn0036663	chr3L	-	16800071	16800189	16800092	16800189	16799483	16799954	0.00244	-0.667
3	FBgn0030893	chrX	+	18093510	18094097	18093510	18093622	18095084	18096453	0.01564	0.469
4	FBgn0030693	chrX	-	15795809	15796028	15795892	15796028	15794496	15795101	0.0003	-0.169
5	FBgn0030693	chrX	-	15795809	15796028	15796001	15796028	15794496	15795101	0.00056	-0.367
6	FBgn0030669	chrX	-	15700904	15701259	15700988	15701259	15700171	15700807	7.9E-06	-0.106
7	FBgn0000173	chrX	+	13996656	13996797	13996656	13996755	13998094	13999805	0.0275	0.278
8	FBgn0035375	chr3L	+	2782254	2782555	2782254	2782496	2782737	2783472	5.7E-10	-1
9	FBgn0267252	chr2L	+	9256562	9256814	9256562	9256696	9256892	9257206	0.02772	-0.091
10	FBgn0267252	chr2L	+	9256562	9256814	9256562	9256696	9272465	9272607	0.00473	-0.11
11	FBgn0028371	chr2R	-	22729100	22729272	22729194	22729272	22727563	22727781	0.01201	0.824
12	FBgn0034408	chr2R	+	18947316	18947820	18947316	18947586	18954977	18956126	0	-0.568
13	FBgn0259984	chr2L	+	13550138	13550420	13550138	13550293	13551141	13551666	0.00117	-0.315
14	FBgn0030850	chrX	-	17624263	17625658	17625529	17625658	17623220	17624205	0	0.725
15	FBgn0000581	chr2R	+	11452768	11452885	11452768	11452880	11453392	11454469	0.0008	0.523
16	FBgn0039790	chr3R	-	30599918	30600323	30600123	30600323	30595389	30595626	0.00846	0.191
17	FBgn0262562	chr3R	-	17794182	17794490	17794292	17794490	17783934	17784095	0.00604	-0.602
18	FBgn0020279	chr2R	+	8075859	8076108	8075859	8076102	8076292	8076448	0.00668	-0.149
19	FBgn0051716	chr2L	+	10397178	10397647	10397178	10397474	10398034	10398219	1.7E-05	0.501
20	FBgn0259923	chrX	-	16694830	16695144	16694950	16695144	16694042	16694352	0.01011	-0.457
21	FBgn0031449	chr2L	+	2730103	2730469	2730103	2730372	2736129	2736430	0.00049	-0.479
22	FBgn0085427	chr2L	+	8931081	8932323	8931081	8931147	8932900	8933382	0.00377	-0.142
23	FBgn0044047	chrX	-	2332275	2332408	2332343	2332408	2331925	2332139	3.2E-09	0.951
24	FBgn0031589	chr2L	-	4197872	4198071	4197964	4198071	4196474	4196814	9.2E-08	0.655
25	FBgn0050296	chr2R	+	21017783	21017952	21017783	21017912	21019158	21019582	0.01814	0.15
26	FBgn0027596	chr2R	-	14907024	14907971	14907487	14907971	14905513	14905953	1.6E-05	-0.433
27	FBgn0037117	chr3L	+	21680049	21680232	21680049	21680152	21680299	21682433	1.3E-05	-0.589
28	FBgn0037117	chr3L	+	21680049	21680232	21680049	21680223	21680299	21682433	8.9E-11	-0.39

29	FBgn0264953	chr2L	+	8182626	8182878	8182626	8182875	8183188	8183563	0.00023	0.89
30	FBgn0031673	chr2L	-	5042960	5043158	5042983	5043158	5042490	5042887	0.02338	0.249
31	FBgn0031673	chr2L	-	5042960	5043158	5042983	5043158	5042490	5042890	0.00306	0.26
32	FBgn0031673	chr2L	-	5042960	5043158	5043077	5043158	5042490	5042890	2.5E-07	0.382
33	FBgn0063649	chr3R	+	16158160	16158306	16158160	16158295	16158857	16159204	0.00491	0.52
34	FBgn0063649	chr3R	+	16158160	16158313	16158160	16158306	16158857	16159204	8.3E-05	-0.724
35	FBgn0265607	chr2L	+	17131023	17131624	17131023	17131225	17133482	17133816	0.00544	-0.271
36	FBgn0028572	chr2L	+	5066011	5066167	5066011	5066071	5066869	5067249	9.3E-06	-0.597
37	FBgn0033224	chr2R	-	7805100	7805566	7805421	7805566	7803511	7804938	0.00735	-0.198
38	FBgn0026401	chr2R	-	4728778	4729017	4728813	4729017	4728432	4728551	0.00489	-0.229
39	FBgn0260388	chr3L	-	16496052	16496236	16496138	16496236	16495299	16495536	0	0.764
40	FBgn0051038	chr3R	+	29877058	29877178	29877058	29877115	29877926	29879810	2.5E-07	0.266
41	FBgn0039774	chr3R	+	30460466	30460890	30460466	30460789	30462814	30462969	8.3E-05	0.141
42	FBgn0033313	chr2R	+	8615902	8616156	8615902	8616003	8616326	8616516	0.00382	-0.254
43	FBgn0004893	chr2L	+	3771699	3771867	3771699	3771784	3772460	3772694	0.0001	0.979
44	FBgn0004893	chr2L	+	3771699	3771873	3771699	3771784	3772460	3772694	0.00244	0.9
45	FBgn0034897	chr2R	+	23713899	23714118	23713899	23714074	23714665	23714899	0.00161	0.127
46	FBgn0039924	chr4	-	1196707	1196848	1196757	1196848	1195841	1196091	3.9E-13	-0.957
47	FBgn0260722	chr3R	-	9551570	9551748	9551700	9551748	9548103	9549810	0.01448	0.465
48	FBgn0260794	chr3R	-	4806174	4806463	4806361	4806463	4801212	4801991	3.9E-13	0.609
49	FBgn0260794	chr3R	-	4806256	4806463	4806361	4806463	4801212	4801991	9.4E-12	0.563
50	FBgn0041150	chr2L	-	4928193	4928393	4928227	4928393	4925828	4926346	0.01316	-0.505
51	FBgn0041150	chr2L	-	4928197	4928393	4928227	4928393	4925828	4926346	0.00028	-0.635
52	FBgn0041150	chr2L	-	4928193	4928393	4928243	4928393	4925828	4926350	0	-0.856
53	FBgn0260938	chrX	-	15887012	15887311	15887117	15887311	15884442	15885943	0.00139	0.208
54	FBgn0260938	chrX	-	15887037	15887311	15887117	15887311	15884442	15885943	0.00111	0.209
55	FBgn0035719	chr3L	+	6943393	6943824	6943393	6943753	6944431	6944769	1.5E-05	-0.793
56	FBgn0052085	chr3L	-	11679951	11680344	11680043	11680344	11674267	11675665	0.00306	-0.195
57	FBgn0036663	chr3L	-	16800037	16800189	16800092	16800189	16799483	16799954	0.00119	-0.807
58	FBgn0036663	chr3L	-	16800090	16800189	16800092	16800189	16799483	16799954	0.01061	-0.418
59	FBgn0036663	chr3L	-	16807411	16807731	16807633	16807731	16799483	16799954	6.9E-05	0.579
60	FBgn0027621	chrX	+	19505766	19505848	19505766	19505846	19506482	19509350	0.00735	-0.543
61	FBgn0030685	chrX	-	15760255	15760649	15760466	15760649	15757855	15758317	0.00031	0.245
62	FBgn0030685	chrX	-	15760424	15760649	15760466	15760649	15757855	15758317	0.00029	0.309
63	FBgn0030521	chrX	-	13781839	13782170	13781985	13782170	13781472	13781733	0.00177	-0.667
64	FBgn0036921	chr3L	+	19928742	19929125	19928742	19928810	19930814	19931014	6.9E-06	1
65	FBgn0028582	chr3L	+	7532428	7532613	7532428	7532458	7532671	7532828	5E-10	0.411
66	FBgn0052654	chrX	+	12596561	12596969	12596561	12596737	12597345	12600441	0.03582	0.521
67	FBgn0030364	chrX	+	12018639	12019325	12018639	12018940	12019627	12019758	0.00163	-0.203
68	FBgn0052264	chr3L	-	3765505	3765731	3765604	3765731	3758406	3759258	0.01448	0.375
69	FBgn0035266	chr3L	+	1739162	1739544	1739162	1739281	1743211	1743834	0.02569	-0.426
70	FBgn0284408	chrX	-	2545109	2545410	2545360	2545410	2544464	2544762	0.01083	-0.552
71	FBgn0284408	chrX	-	2545109	2545410	2545380	2545410	2544464	2544762	0.00333	0.593
72	FBgn0267861	chr2R	-	1526781	1527095	1527004	1527095	1521331	1521506	0.00064	-0.666
73	FBgn0267252	chr2L	+	9256562	9256818	9256562	9256696	9256892	9257206	0.00021	-0.132
74	FBgn0267252	chr2L	+	9256756	9257116	9256756	9256814	9272465	9272607	0	0.473
75	FBgn0267252	chr2L	+	9256756	9257122	9256756	9256814	9272465	9272607	0	0.469
76	FBgn0026374	chrX	+	15788896	15789112	15788896	15788957	15790233	15790386	0.03654	-0.572
77	FBgn0036715	chr3L	-	17379360	17379530	17379362	17379530	17378285	17378592	0.01836	0.301
78	FBgn0015218	chr3L	-	9401784	9402137	9402055	9402137	9401039	9401367	9.4E-06	-0.834
79	FBgn0264002	chr3L	+	2282996	2283442	2282996	2283036	2297474	2297723	0.00788	0.514
80	FBgn0264002	chr3L	+	2282996	2283456	2282996	2283036	2297474	2297723	0.00382	0.515
81	FBgn0264490	chr3R	+	21949113	21949402	21949113	21949398	21949639	21949872	0.00306	-0.565
82	FBgn0263593	chr2R	-	8145751	8145981	8145903	8145981	8142642	8143074	0	-0.995
83	FBgn0263593	chr2R	-	8145754	8145981	8145903	8145981	8142642	8143074	0	-0.768
84	FBgn0028497	chr2R	-	23394916	23395421	23395328	23395421	23393363	23393480	0.00012	0.152
85	FBgn0031453	chr2L	+	2752799	2753118	2752799	2752837	2753371	2753660	0.00525	0.514
86	FBgn0262975	chr3R	-	23226276	23226711	23226453	23226711	23222796	23223120	0.00011	0.637
87	FBgn0262169	chr2R	+	10052540	10052919	10052540	10052657	10057048	10057343	0.00112	-0.762
88	FBgn0262081	chr3R	-	11646499	11646973	11646787	11646973	11642275	11643205	0.02586	0.503
89	FBgn0262081	chr3R	-	11646503	11646973	11646787	11646973	11642275	11643205	0.0244	0.501
90	FBgn0262081	chr3R	-	11646538	11646973	11646787	11646973	11642275	11643205	0.01749	0.524
91	FBgn0262081	chr3R	-	11646592	11646973	11646787	11646973	11642275	11643205	0.04333	0.437
92	FBgn0039970	chr2R	+	5608634	5608831	5608634	5608782	5609012	5609994	0.02421	-0.354
93	FBgn0004910	chr3L	-	3925462	3925602	3925558	3925602	3924223	3924474	0.01579	0.178
94	FBgn0082582	chr3R	+	30546404	30546598	30546404	30546448	30548420	30548749	0	1
95	FBgn0082582	chr3R	+	30546405	30546587	30546405	30546448	30548420	30548749	0	1
96	FBgn0082582	chr3R	+	30546405	30546587	30546405	30546546	30548420	30548749	0	0.652
97	FBgn0082582	chr3R	+	30546404	30546587	30546404	30546448	30548451	30548749	0	0.985
98	FBgn0082582	chr3R	+	30546404	30546598	30546404	30546448	30548451	30548749	0	0.949
99	FBgn0082582	chr3R	+	30546404	30546587	30546404	30546546	30548451	30548749	0	0.837
100	FBgn0082582	chr3R	+	30546404	30546598	30546404	30546546	30548451	30548749	0	0.239
101	FBgn0082582	chr3R	+	30546404	30546587	30546404	30546550	30548451	30548749	0	0.944
102	FBgn0082582	chr3R	+	30546404	30546598	30546404	30546550	30548451	30548749	0	1
103	FBgn0082582	chr3R	+	30546404	30546684	30546404	30546587	30548451	30548749	0	-0.953
104	FBgn0082582	chr3R	+	30546404	30546684	30546404	30546598	30548451	30548749	0	-1
105	FBgn0032192	chr2L	-	10209247	10209744	10209362	10209744	10208644	10208802	0.00234	-0.647
106	FBgn0001139	chr3R	+	26042819	26042903	26042819	26042899	26042994	26043379	0.00216	-0.754
107	FBgn0039359	chr3R	-	25684576	25685051	25684742	25685051	25684006	25684516	0.04729	0.35
108	FBgn0005649	chr3R	-	24046908	24047012	24046941	24047012	24045437	24046491	6.4E-05	0.816
109	FBgn0005649	chr3R	-	24046937	24047012	24046941	24047012	24045437	24046491	0.04871	0.287
110	FBgn0005649	chr3R	-	24046941	24047368	24047116	24047368	24045437	24046491	0.00274	-0.417
111	FBgn0030850	chrX	-	17625255	17625657	17625529	17625657	17623220	17624205	0	0.263
112	FBgn0030850	chrX	-	17625370	17625657	17625529	17625657	17623220	17624205	0	0.335
113	FBgn0030850	chrX	-	17625493	17625657	17625529	17625657	17623220	17624205	0	0.362
114	FBgn0041605	chr3R	+	4281826	4282302	4281826	4281915	4282673	4282834	0.03958	0.182
115	FBgn0041605	chr3R	+	4281826	4282302	4281826	4282003	4282673	4282834	1.6E-06	0.227
116	FBgn0041605	chr3R	+	4281826	4282309	4281826	4282003	4282673	4282834	5.6E-06	0.241
117	FBgn0041605	chr3R	+	4281826	4282314	4281826	4282003	4282673	4282834	0.00204	0.191
118	FBgn0034718	chr2R	-	22301306	22301423	22301363	22301423	22298213	22301009	0.01102	0.845
119	FBgn0260003	chr3R	+	19463532	19463931	19463532	19463639	19464499	19464846	0.04729	0.267
120	FBgn0002781	chr3R	-	21356317	21356699	21356546	21356699	21355757	21356251	0.00228	-0.715
121	FBgn0052428	chr3L	-	20471203	20471428	20471229	20471428	20470953	20471148	0.0021	-0.667
122	FBgn0030884	chrX	+	17911461	17911768	17911461	17911648	17918327	17918588	0.04617	0.594
123	FBgn0013305	chr2R	-	12879447	12879656	12879573	12879656	12879196	12879222	0.02722	0.615
124	FBgn0037955	chr3R	-	11821975	11822241	11822191	11822241	11821279	11821819	0.00058	0.468
125	FBgn0035533	chr3L	-	4363747	4363974	4363769	4363974	4363026	4363127	0.00937	-0.756
126	FBgn0003462	chr3L	-	11113266	11113740	11113531	11113740	11112280	11112848	0.01963	-0.834
127	FBgn0031896	chr2L	-	7203934	7204264	7203938	7204264	7197023	7197296	1.8E-11	-0.911
128	FBgn0014010	chr2L	-	2364627	2364954	2364884	2364954	2361159	2361585	0.02021	-0.417
129	FBgn0000412	chr3R	-	9239969	9240068	9239975	9240068	9239489	9239908	0.00936	0.667
130	FBgn0052057	chr3L	-	10185978	10186108	10186022	10186108	10182242	10182515	0.00924	-0.361
131	FBgn0036369	chr3L	-	13469135	13469212	13469201	13469212	13468952	13469069	0.00038	-0.621
132	FBgn0015568	chr3R	-	7548692	7549124	7548887	7549124	7547769	7548236	0.00073	-0.693
133	FBgn0032946	chr2L	+	21380132	21380600	21380132	21380315	21382018	21382330	0.01294	0.425
134	FBgn0032946	chr2L	+	21380470	21380728	21380470	21380478	21382018	21382330	0	0.214
135	FBgn0032946	chr2L	+	21380470	21380728	21380470	21380600	21382018	21382330	0.01749	0.488
136	FBgn0032946	chr2L	+	21380470	21380826	21380470	21380600	21382018	21382330	1.2E-07	-0.367
137	FBgn0032946	chr2L	+	21380470	21380826	21380470	21380728	21382018	21382330	1.8E-07	-0.538
138	FBgn0061515	chr3L	-	14031153	14031367	14031307	14031367	14030806	14031002	0.02558	0.63
139	FBgn0061515	chr3L	-	14031155	14031367	14031307	14031367	14030806	14031002	0.00473	0.733
140	FBgn0061515	chr3L	-	14031157	14031367	14031307	14031367	14030806	14031002	0.03958	0.683
141	FBgn0061515	chr3L	-	14031159	14031367	14031307	14031367	14030806	14031002	0.00689	0.686
142	FBgn0061515	chr3L	-	14031161	14031367	14031307	14031367	14030806	14031002	0.02686	0.666
143	FBgn0061515	chr3L	-	14031225	14031367	14031307	14031367	14030806	14031002	0.0133	0.665
144	FBgn0040208	chr3R	-	5231905	5232209	5232056	5232209	5230992	5231313	0.02799	0.619
145	FBgn0030593	chrX	-	14905427	14905549	14905442	14905549	14903963	14904966	0.01261	-0.762
146	FBgn0033368	chr2R	-	9059663	9059907	9059723	9059907	9059254	9059548	0.00333	0.665
147	FBgn0040778	chr2R	-	8067125	8067293	8067184	8067293	8066273	8066758	0	-0.99
148	FBgn0040778	chr2R	-	8067166	8067293	8067184	8067293	8066273	8066758	1.4E-13	-0.917
149	FBgn0040778	chr2R	-	8067342	8067655	8067407	8067655	8067125	8067293	3.5E-09	-0.607
150	FBgn0051100	chr3R	-	9001642	9001886	9001798	9001886	9000286	9000544	1.5E-10	-1
151	FBgn0032587	chr2L	-	16532458	16532877	16532584	16532877	16527389	16528439	0.00782	0.592
152	FBgn0034180	chr2R	-	17013284	17013481	17013287	17013481	17011951	17012708	0.01256	0.242
153	FBgn0034180	chr2R	-	17015420	17015894	17015693	17015894	17014948	17015202	6.4E-06	0.78
154	FBgn0034180	chr2R	-	17015637	17015894	17015693	17015894	17014948	17015202	8.5E-08	0.928
155	FBgn0051716	chr2L	+	10397042	10397474	10397042	10397194	10398034	10398219	3.3E-06	-0.722
156	FBgn0259923	chrX	-	16694613	16694836	16694814	16694836	16694042	16694371	0.00162	-0.462
157	FBgn0259923	chrX	-	16694814	16695144	16694840	16695144	16694042	16694371	6.8E-05	0.76
158	FBgn0053978	chr4	-	175361	175639	175520	175639	174784	174968	0.02814	-0.667
159	FBgn0034071	chr2R	+	16048983	16049411	16048983	16049405	16049486	16049619	0.00247	0.446
160	FBgn0031449	chr2L	+	2730103	2730469	2730103	2730372	2735890	2736136	0.00194	-0.441
161	FBgn0010909	chr3L	-	2586165	2586540	2586265	2586540	2585604	2585738	0.00174	-0.528
162	FBgn0010909	chr3L	-	2586329	2586540	2586413	2586540	2585604	2585738	0.01741	0.813
163	FBgn0030830	chrX	+	17221441	17221776	17221441	17221487	17222125	17222209	0.0102	0.335
164	FBgn0052432	chr3L	-	20885036	20885188	20885102	20885188	20881629	20881810	0.00023	-0.419
165	FBgn0050296	chr2R	+	21023434	21023754	21023434	21023586	21023975	21024125	2.8E-13	0.265
166	FBgn0003710	chr3L	+	4188498	4188977	4188498	4188757	4189675	4191886	0.02134	0.464
167	FBgn0259221	chr2R	-	13429633	13429895	13429728	13429895	13425512	13426038	0.00021	0.667
168	FBgn0052944	chr3R	-	4419106	4419578	4419359	4419578	4415682	4415886	1.3E-07	0.602
169	FBgn0052944	chr3R	-	4419138	4419578	4419359	4419578	4415682	4415886	0.00082	0.611
170	FBgn0015754	chr2R	+	16181248	16181805	16181248	16181665	16181980	16182090	9.7E-11	-0.579
171	FBgn0000579	chr2L	-	1726953	1727402	1727317	1727402	1725059	1726655	0.00296	0.667
172	FBgn0000579	chr2L	-	1726964	1727402	1727317	1727402	1725059	1726655	0.00687	0.667
173	FBgn0031322	chr2L	+	1191402	1191538	1191402	1191534	1191648	1191909	0.03958	-0.251
174	FBgn0032210	chr2L	-	10299431	10299847	10299656	10299847	10299043	10299220	6.1E-05	-0.833
175	FBgn0004169	chrX	-	13600541	13600949	13600873	13600949	13600069	13600117	8.7E-05	-0.712
176	FBgn0038652	chr3R	-	18792793	18793061	18793048	18793061	18792050	18792120	0.00146	-0.9
177	FBgn0031673	chr2L	-	5042964	5043158	5043077	5043158	5042490	5042890	0.03744	0.348
178	FBgn0036179	chr3L	+	11601430	11601515	11601430	11601508	11602647	11603254	0.00023	0.35
179	FBgn0036179	chr3L	+	11601430	11601520	11601430	11601508	11602647	11603254	1.2E-10	1
180	FBgn0063649	chr3R	+	16158160	16158313	16158160	16158306	16158920	16159204	0.00159	-0.69
181	FBgn0039883	chr3R	+	31825843	31826305	31825843	31826211	31827722	31828103	3.3E-07	-0.915
182	FBgn0015221	chr3R	+	30389198	30389332	30389198	30389328	30389415	30389442	4.9E-09	-0.172
183	FBgn0015221	chr3R	+	30388829	30389326	30388829	30388902	30389696	30390584	0.00951	0.277
184	FBgn0032172	chr2L	-	9963377	9963755	9963451	9963755	9962734	9963012	0.01644	-0.55
185	FBgn0000024	chr3R	-	13258302	13258425	13258304	13258425	13245809	13246654	0	0.551
186	FBgn0004242	chr2L	-	2799488	2799979	2799908	2799979	2798485	2798836	0.00384	0.463
187	FBgn0038460	chr3R	-	16986118	16986245	16986126	16986245	16984570	16984617	0.01594	-0.237
188	FBgn0011693	chr3L	-	16354133	16354268	16354170	16354268	16353679	16354081	0.00667	-0.658
189	FBgn0020620	chr2R	-	13990208	13990707	13990444	13990707	13989684	13989986	0.00525	-0.291
190	FBgn0260388	chr3L	-	16495841	16496236	16496138	16496236	16495299	16495536	0.00033	0.491
191	FBgn0051038	chr3R	+	29877058	29877247	29877058	29877115	29877926	29879810	1.5E-06	0.738
192	FBgn0004657	chrX	+	8061644	8062091	8061644	8061933	8063839	8064459	0	0.952
193	FBgn0032414	chr2L	+	12114359	12114650	12114359	12114410	12124561	12125185	0.00315	0.277
194	FBgn0031275	chr2L	+	750608	750984	750608	750766	752730	753730	0.02257	-0.619
195	FBgn0015222	chr3R	-	30386717	30386828	30386738	30386828	30386318	30386596	0.0051	-0.548
196	FBgn0019890	chr2L	-	13904025	13904208	13904104	13904208	13903780	13903931	0.00343	0.223
197	FBgn0051635	chr2L	-	6647707	6648145	6647753	6648145	6645971	6646947	2.7E-10	0.908
198	FBgn0051635	chr2L	-	6647707	6648145	6647793	6648145	6645971	6646947	3.1E-07	0.871
199	FBgn0051635	chr2L	-	6647746	6648145	6647753	6648145	6645971	6646947	0.00735	0.455
200	FBgn0010348	chr3L	-	22871401	22871559	22871529	22871559	22869841	22870150	0.03582	0.667
201	FBgn0053080	chrX	-	5713339	5713527	5713343	5713527	5711634	5711803	0.00445	0.474
202	FBgn0032796	chr2L	-	19508320	19508764	19508351	19508764	19507489	19508265	3.9E-06	0.718

203	FBgn0023213	chr4	-	930281	930715	930647	930715	930079	930131	0.0049	-0.495
204	FBgn0003380	chrX	-	17990304	17990614	17990327	17990614	17976350	17976791	0.00228	0.558
205	FBgn0003380	chrX	-	17990304	17990614	17990532	17990614	17976350	17976791	0.00288	0.619
206	FBgn0003380	chrX	-	17990304	17990614	17990580	17990614	17976350	17976791	0.02008	0.411
207	FBgn0003380	chrX	-	17990304	17990614	17990588	17990614	17976350	17976791	0.00114	0.341
208	FBgn0028496	chr2R	-	18497704	18498026	18497885	18498026	18496746	18496905	0.00616	-0.483
209	FBgn0028496	chr2R	-	18497755	18498026	18497773	18498026	18496746	18496905	4.5E-05	0.664
210	FBgn0028496	chr2R	-	18497773	18498026	18497885	18498026	18496746	18496905	0.00011	-0.69
211	FBgn0034691	chr2R	+	22080442	22080692	22080442	22080538	22081074	22082637	0.04712	0.34
212	FBgn0016059	chr2R	-	17679059	17679255	17679143	17679255	17678271	17678782	0.00491	0.28
213	FBgn0016059	chr2R	-	17683048	17683287	17683075	17683287	17678271	17678782	0.00417	-0.853
214	FBgn0052772	chrX	+	4909101	4909319	4909101	4909208	4909451	4909797	0.04446	-0.442
215	FBgn0017566	chrX	-	8251445	8251687	8251551	8251687	8251200	8251388	0.00494	-0.822
216	FBgn0030089	chrX	+	9109549	9109758	9109549	9109694	9110821	9111005	0.02868	0.243
217	FBgn0030089	chrX	+	9109549	9109884	9109549	9109694	9110821	9111005	0.01518	0.213
218	FBgn0034645	chr2R	+	21658123	21658275	21658123	21658180	21658345	21658480	0.04333	0.294
219	FBgn0260943	chr3L	-	17237823	17238212	17237885	17238212	17235351	17236062	0.01047	0.651
220	FBgn0260943	chr3L	-	17237831	17238212	17237885	17238212	17235351	17236062	9.4E-05	0.288
221	FBgn0260943	chr3L	-	17237879	17238212	17237885	17238212	17235351	17236062	0.00937	0.437
222	FBgn0260943	chr3L	-	17237885	17238212	17238071	17238212	17235351	17236062	0.01406	-0.315
223	FBgn0260943	chr3L	-	17237885	17238212	17238075	17238212	17235351	17236062	0.03962	-0.469
224	FBgn0260943	chr3L	-	17237885	17238212	17238094	17238212	17235351	17236062	0.01254	-0.427
225	FBgn0016131	chr2R	+	16579469	16579735	16579469	16579533	16580841	16581015	0.01391	0.263
226	FBgn0016131	chr2R	+	16579563	16579926	16579563	16579735	16580841	16581015	0.00016	-0.4
227	FBgn0016131	chr2R	+	16579563	16579926	16579563	16579755	16580841	16581015	0.01963	-0.328
228	FBgn0033313	chr2R	+	8615902	8616266	8615902	8616003	8616326	8616516	0.00888	-0.244
229	FBgn0002773	chr3R	-	30174064	30174551	30174126	30174551	30173571	30173814	0.04333	0.462
230	FBgn0039714	chr3R	-	29867743	29867817	29867752	29867817	29865056	29865399	0	0.79
231	FBgn0053182	chr2R	-	13189560	13189980	13189696	13189980	13187393	13187908	0.02007	0.35
232	FBgn0053182	chr2R	-	13198158	13198337	13198212	13198337	13187393	13187908	0.04712	-0.478
233	FBgn0038744	chr3R	+	19890688	19891254	19890688	19890828	19892516	19894015	0.01963	0.574
234	FBgn0038744	chr3R	+	19890688	19891254	19890688	19890952	19892516	19894015	0.00065	-0.697
235	FBgn0022382	chr2R	-	10025828	10026042	10025849	10026042	10024050	10024249	0.04846	-0.255
236	FBgn0004893	chr2L	+	3771699	3771871	3771699	3771784	3772460	3772694	1.2E-05	0.979
237	FBgn0050344	chr2R	+	9147549	9147876	9147549	9147872	9147938	9148095	0	-0.174
238	FBgn0028697	chr3L	+	27267254	27267523	27267254	27267322	27267591	27267918	0.02814	0.583
239	FBgn0265356	chr2R	+	19002417	19002833	19002417	19002780	19005788	19006571	3.2E-09	0.163
240	FBgn0016126	chr4	+	450995	451456	450995	451338	452617	452744	0.00296	0.667
241	FBgn0026199	chr4	-	695696	696055	695856	696055	695213	695337	0.00107	-0.518
242	FBgn0039890	chr4	-	193048	193489	193365	193489	189997	190061	0.00025	0.717
243	FBgn0003141	chr2L	-	20074389	20074460	20074391	20074460	20074190	20074336	5E-06	-0.72
244	FBgn0011754	chrX	+	11695823	11696311	11695823	11696061	11696402	11696540	0.0121	-0.59
245	FBgn0011695	chr2R	+	24026375	24026498	24026375	24026461	24027003	24027518	2.3E-12	-0.657
246	FBgn0011695	chr2R	+	24026375	24026871	24026375	24026461	24027003	24027518	0.0041	-0.543
247	FBgn0003969	chrX	-	16005319	16005812	16005745	16005812	16003714	16004086	0.00418	0.49
248	FBgn0010263	chr2L	+	2956910	2957051	2956910	2956992	2960689	2960839	1.3E-07	0.694
249	FBgn0053516	chr2L	-	2109665	2109878	2109818	2109878	2066741	2067160	4.5E-06	0.97
250	FBgn0035331	chr3L	+	2322692	2323109	2322692	2322748	2323299	2323495	0.00296	0.471
251	FBgn0033970	chr2R	+	14833142	14833475	14833142	14833354	14833534	14833951	1.3E-06	0.605
252	FBgn0034420	chr2R	+	19176587	19176953	19176587	19176743	19177302	19177573	1.1E-05	-0.472
253	FBgn0039900	chr4	-	305742	306161	306059	306161	305015	305178	0.01152	-0.247
254	FBgn0039900	chr4	-	305813	306161	306059	306161	305015	305178	0.02569	-0.239
255	FBgn0259700	chrX	-	16095896	16096231	16096161	16096231	16095457	16095690	0.00362	0.447
256	FBgn0000370	chr2L	+	21310865	21311032	21310865	21311017	21311192	21311531	9.4E-05	0.257
257	FBgn0034897	chr2R	+	23713899	23714185	23713899	23714074	23714665	23714899	6.4E-10	0.284
258	FBgn0037998	chr3R	-	12216230	12216686	12216637	12216686	12215657	12215953	0	0.917
259	FBgn0058191	chr2R	+	1265154	1265531	1265154	1265293	1266191	1266349	4.3E-06	-0.457
260	FBgn0058191	chr2R	+	1265342	1266052	1265342	1265531	1266191	1266349	6.4E-10	0.627
261	FBgn0039936	chr4	-	865368	865642	865374	865642	861792	864411	1.7E-11	-0.849
262	FBgn0038465	chr3R	+	17006066	17006274	17006066	17006271	17006333	17006501	2.4E-06	0.831
263	FBgn0085399	chr2R	-	22039095	22039559	22039403	22039559	22037391	22037716	0	-0.627
264	FBgn0085399	chr2R	-	22039097	22039559	22039403	22039559	22037391	22037716	0	-0.633
265	FBgn0085399	chr2R	-	22039236	22039559	22039403	22039559	22037391	22037716	0	-0.627
266	FBgn0085399	chr2R	-	22039330	22039559	22039403	22039559	22037391	22037716	0	-0.495
267	FBgn0085399	chr2R	-	22039336	22039559	22039403	22039559	22037391	22037716	0	-0.54
268	FBgn0085478	chrX	-	9356083	9356227	9356114	9356227	9354461	9355178	0.00055	-0.851
269	FBgn0039805	chr3R	+	30867520	30867803	30867520	30867682	30867906	30868154	0.02086	0.667
270	FBgn0086711	chr2L	-	14983776	14984084	14983875	14984084	14977214	14977646	4.3E-05	-0.759
271	FBgn0261268	chr2L	-	15271617	15272024	15271900	15272024	15270904	15271143	0.01374	0.354
272	FBgn0259994	chrX	-	5648322	5648487	5648440	5648487	5647731	5648172	0.00255	0.319
273	FBgn0260934	chr2R	+	19462501	19462960	19462501	19462651	19464309	19465694	0.00687	-0.484
274	FBgn0260938	chrX	-	15887041	15887311	15887117	15887311	15884442	15885943	1.2E-07	0.932
275	FBgn0260938	chrX	-	15887117	15887311	15887211	15887311	15884442	15885943	6.1E-08	-0.733
276	FBgn0016694	chr3L	-	7832702	7833073	7832863	7833073	7830386	7831827	0.02021	-0.576
277	FBgn0261794	chr2R	+	23909677	23909833	23909677	23909809	23911682	23911868	0.00016	-0.326

MXE

	FlyBase ID	chr	strand	1stExonStart_0base	1stExonEnd	lExonStart_0b	2ndExonEnc	upstreamES	upstreamEE	wnstrean	downstreamEE	FDR	ΔPSI
1	FBgn0052600	chrX	+	14324737	14324858	14355462	14355542	14322225	14322915	1.4E+07	14389682	0.013163709	0.228
2	FBgn0264695	chr2L	+	16775210	16775381	16776262	16776433	16774449	16774857	1.7E+07	16777439	0.003151712	-0.209
3	FBgn0263346	chr3R	+	4670796	4670850	4680778	4680837	4659578	4660399	4697190	4697542	0	-0.386
4	FBgn0039970	chr2R	+	5609012	5609489	5610063	5610256	5608634	5608797	5611191	5611586	0.045272846	0.121
5	FBgn0013305	chr2R	-	12879051	12879146	12879196	12879222	12878384	12878710	1.3E+07	12879656	0	0.689
6	FBgn0013305	chr2R	-	12879051	12879146	12879196	12879222	12878384	12878710	1.3E+07	12879798	0	0.504
7	FBgn0264443	chr2L	-	19982566	19982612	19999336	19999373	19971405	19971990	2E+07	20017282	0.001162542	0.257
8	FBgn0033159	chr2R	-	7338249	7338537	7338640	7338928	7332260	7332380	7347848	7348010	0.001008034	0.516
9	FBgn0033159	chr2R	-	7338249	7338537	7341327	7341618	7332260	7332380	7347848	7348010	0.003501158	0.529
10	FBgn0033159	chr2R	-	7338249	7338537	7343331	7343622	7332260	7332380	7347848	7348010	9.78111E-06	0.794
11	FBgn0033159	chr2R	-	7338249	7338537	7344663	7344954	7332260	7332380	7347848	7348010	0.012030345	0.531
12	FBgn0033159	chr2R	-	7338249	7338537	7345875	7346166	7332260	7332380	7347848	7348010	0.047412135	0.318
13	FBgn0033159	chr2R	-	7338249	7338537	7346268	7346556	7332260	7332380	7347848	7348010	0.001008034	0.635
14	FBgn0033159	chr2R	-	7338249	7338537	7346641	7346929	7332260	7332380	7347848	7348010	0.003408964	0.69
15	FBgn0033159	chr2R	-	7338249	7338537	7347006	7347294	7332260	7332380	7347848	7348010	3.37682E-06	0.738
16	FBgn0033159	chr2R	-	7343331	7343622	7345060	7345351	7332260	7332380	7347848	7348010	0.039334569	-0.555
17	FBgn0033159	chr2R	-	7344663	7344954	7345060	7345351	7332260	7332380	7347848	7348010	0.045171494	-0.432
18	FBgn0033159	chr2R	-	7345060	7345351	7347006	7347294	7332260	7332380	7347848	7348010	0.002918982	0.73
19	FBgn0033159	chr2R	-	7349392	7349513	7350580	7350704	7348130	7348975	7361717	7361867	4.82874E-06	0.563
20	FBgn0033159	chr2R	-	7349392	7349513	7351698	7351822	7348130	7348975	7361717	7361867	9.06692E-05	0.507
21	FBgn0033159	chr2R	-	7349392	7349513	7353244	7353365	7348130	7348975	7361717	7361867	6.98458E-06	0.757
22	FBgn0033159	chr2R	-	7349392	7349513	7353565	7353692	7348130	7348975	7361717	7361867	1.79087E-06	1
23	FBgn0033159	chr2R	-	7349392	7349513	7354056	7354177	7348130	7348975	7361717	7361867	1.74087E-06	0.796
24	FBgn0033159	chr2R	-	7349392	7349513	7354276	7354400	7348130	7348975	7361717	7361867	0.00023689	0.565
25	FBgn0033159	chr2R	-	7349392	7349513	7354881	7355005	7348130	7348975	7361717	7361867	2.80312E-05	0.709
26	FBgn0033159	chr2R	-	7349392	7349513	7355290	7355414	7348130	7348975	7361717	7361867	0.003180701	0.39
27	FBgn0033159	chr2R	-	7349392	7349513	7355685	7355809	7348130	7348975	7361717	7361867	2.28115E-06	0.834
28	FBgn0033159	chr2R	-	7349392	7349513	7356316	7356440	7348130	7348975	7361717	7361867	3.75609E-09	0.851
29	FBgn0033159	chr2R	-	7349392	7349513	7356526	7356650	7348130	7348975	7361717	7361867	3.75609E-09	0.851
30	FBgn0033159	chr2R	-	7349392	7349513	7356744	7356868	7348130	7348975	7361717	7361867	1.38194E-07	0.821
31	FBgn0033159	chr2R	-	7349392	7349513	7356972	7357096	7348130	7348975	7361717	7361867	0.000412786	0.732
32	FBgn0033159	chr2R	-	7349392	7349513	7357182	7357306	7348130	7348975	7361717	7361867	0.000620813	0.491
33	FBgn0033159	chr2R	-	7349392	7349513	7357590	7357714	7348130	7348975	7361717	7361867	0.001860412	0.369
34	FBgn0033159	chr2R	-	7349392	7349513	7357788	7357915	7348130	7348975	7361717	7361867	1.78104E-06	0.657
35	FBgn0033159	chr2R	-	7349392	7349513	7358002	7358129	7348130	7348975	7361717	7361867	7.54226E-07	0.741
36	FBgn0033159	chr2R	-	7349392	7349513	7358215	7358339	7348130	7348975	7361717	7361867	0.000211166	0.544
37	FBgn0033159	chr2R	-	7349392	7349513	7358806	7358930	7348130	7348975	7361717	7361867	0.000106953	0.591
38	FBgn0033159	chr2R	-	7349392	7349513	7359874	7360007	7348130	7348975	7361717	7361867	0.000101513	0.592
39	FBgn0033159	chr2R	-	7349392	7349513	7360081	7360205	7348130	7348975	7361717	7361867	0.003538209	0.357
40	FBgn0033159	chr2R	-	7349392	7349513	7360525	7360649	7348130	7348975	7361717	7361867	0.001925387	0.613
41	FBgn0033159	chr2R	-	7351915	7352039	7356316	7356440	7348130	7348975	7361717	7361867	0.026120483	0.684
42	FBgn0033159	chr2R	-	7353815	7353936	7356316	7356440	7348130	7348975	7361717	7361867	0.003101762	0.646
43	FBgn0033159	chr2R	-	7356316	7356440	7357182	7357306	7348130	7348975	7361717	7361867	0.033599082	-0.408
44	FBgn0033159	chr2R	-	7356316	7356440	7359441	7359565	7348130	7348975	7361717	7361867	0.028480825	-0.66
45	FBgn0033159	chr2R	-	7356526	7356650	7359666	7359790	7348130	7348975	7361717	7361867	0.029055369	-0.689
46	FBgn0034903	chr2R	-	23759725	23759781	23760621	23760674	23758475	23758602	2.4E+07	23760995	1.81415E-09	0.25
47	FBgn0265356	chr2R	+	19001040	19001789	19004604	19004883	18997127	18998423	1.9E+07	19006571	0.027513247	-0.111
48	FBgn0261545	chr2R	+	12734920	12735119	12746608	12746661	12722304	12723006	1.3E+07	12747747	2.06234E-05	0.258
49	FBgn0261988	chr3R	+	31410353	31410401	31433458	31433888	31405244	31405956	3.1E+07	31453406	2.31016E-07	0.196
50	FBgn0041150	chr2L	-	4911722	4911867	4912671	4912778	4911580	4911641	4925828	4926278	3.15985E-07	0.124
51	FBgn0261788	chr3L	-	7656374	7661506	7662470	7662890	7655388	7656203	7663258	7663324	3.89541E-09	0.213
52	FBgn0001105	chrX	+	15860744	15861189	15861223	15861266	15860114	15860322	1.6E+07	15861486	0.001600536	-0.491
53	FBgn0001105	chrX	+	15861057	15861189	15861223	15861266	15860114	15860322	1.6E+07	15861486	0.001053138	-0.496
54	FBgn0285963	chr3R	+	26926395	26926680	26935958	26936291	26924633	26925261	2.7E+07	26938631	1.38512E-10	0.55
55	FBgn0267252	chr2L	+	9256756	9256814	9256892	9257116	9256562	9256696	9272465	9272607	0.029944523	-0.315
56	FBgn0264308	chr2L	-	13320033	13320480	13330402	13330735	13309639	13310264	1.3E+07	13331121	8.26196E-10	-0.75
57	FBgn0264308	chr2L	-	13320235	13320480	13330402	13330735	13309639	13310264	1.3E+07	13331121	3.47742E-08	-0.726
58	FBgn0263257	chrX	-	15401102	15401268	15402870	15402955	15400678	15400814	1.5E+07	15403628	0.025644275	0.678
59	FBgn0263257	chrX	-	15401102	15401268	15402896	15402955	15400678	15400814	1.5E+07	15403628	1.37353E-05	0.837
60	FBgn0263006	chr2R	-	23933018	23933343	23933446	23933819	23932666	23932814	2.4E+07	23934084	0.032658153	0.333
61	FBgn0263006	chr2R	-	23933093	23933343	23933446	23933819	23932666	23932814	2.4E+07	23934084	0.006104204	0.667
62	FBgn0053144	chr2R	-	10766480	10766550	10783473	10783512	10759862	10759915	1.1E+07	10794484	0.002256063	0.694
63	FBgn0027844	chr2L	+	13807326	13807425	13808025	13808222	13803625	13803962	1.4E+07	13808967	1.8563E-05	0.369
64	FBgn0027844	chr2L	+	13807346	13807425	13808025	13808222	13803625	13803962	1.4E+07	13808967	3.91108E-07	0.658
65	FBgn0028703	chr2L	-	6678542	6678756	6678911	6679082	6677834	6678224	6679985	6680114	1.95283E-05	-0.285
66	FBgn0039465	chr3R	-	26962539	26962820	26963157	26963520	26961063	26962451	2.7E+07	26964285	0.000336444	-0.157
67	FBgn0051145	chr3R	-	23658756	23659160	23666790	23667081	23653738	23653768	2.4E+07	23668467	0.005486016	-0.617
68	FBgn0051145	chr3R	-	23658756	23659160	23666890	23667081	23653738	23653768	2.4E+07	23668467	4.62306E-05	-0.72
69	FBgn0039790	chr3R	-	30595720	30595750	30601007	30601308	30595389	30595626	3.1E+07	30601489	0.000695901	0.647
70	FBgn0041605	chr3R	+	4281826	4282003	4282087	4282309	4280384	4280469	4282673	4282834	0.003124994	-0.166
71	FBgn0041605	chr3R	+	4281826	4282003	4282156	4282309	4280384	4280469	4282673	4282834	0.007949001	-0.169
72	FBgn0041605	chr3R	+	4281826	4282003	4282177	4282309	4280384	4280469	4282673	4282834	0.030094088	-0.162
73	FBgn0041605	chr3R	+	4281839	4282003	4282087	4282309	4280384	4280469	4282673	4282834	0.004547626	-0.168
74	FBgn0041605	chr3R	+	4281839	4282003	4282156	4282309	4280384	4280469	4282673	4282834	0.0114224	-0.171
75	FBgn0041605	chr3R	+	4281839	4282003	4282177	4282309	4280384	4280469	4282673	4282834	0.039496401	-0.164
76	FBgn0041605	chr3R	+	4281839	4282072	4282156	4282511	4280384	4280469	4282673	4282834	0.006424353	-0.484
77	FBgn0041605	chr3R	+	4281839	4282072	4282156	4282551	4280384	4280469	4282673	4282834	0.004327009	-0.617
78	FBgn0041605	chr3R	+	4296299	4296502	4296891	4297083	4295567	4295700	4297487	4304604	0.031319277	0.5
79	FBgn0033494	chr2R	-	10148636	10149094	10149347	10149440	10148081	10148152	1E+07	10151052	0.001746741	0.227
80	FBgn0039897	chr4	+	240649	240833	242487	242637	240313	240398	243169	243585	1.78104E-06	0.819
81	FBgn0039897	chr4	+	240695	240833	242460	242637	240313	240398	243169	243585	0.003089914	0.529
82	FBgn0039897	chr4	+	240695	240833	242487	242637	240313	240398	243169	243585	1.2896E-07	0.864
83	FBgn0038198	chr3R	+	14089920	14090031	14090501	14090569	14089516	14089599	1.4E+07	14091401	9.83307E-05	-0.613
84	FBgn0013305	chr2R	-	12879051	12879134	12879196	12879222	12878384	12878710	1.3E+07	12879798	0	0.54
85	FBgn0263395	chr2R	+	19178941	19179109	19179376	19179433	19178737	19178872	1.9E+07	19179858	5.21969E-05	-0.606
86	FBgn0263395	chr2R	+	19179040	19179109	19179376	19179433	19178737	19178872	1.9E+07	19179858	0.01537992	-0.406
87	FBgn0263395	chr2R	+	19179059	19179109	19179376	19179433	19178737	19178872	1.9E+07	19179858	0.008782174	-0.456
88	FBgn0051522	chr3R	-	4448033	4448289	4452645	4452826	4447700	4447954	4453191	4453316	0.003124994	0.627
89	FBgn0051522	chr3R	-	4448033	4448289	4452645	4453120	4447700	4447954	4453191	4453316	9.24904E-05	0.723
90	FBgn0051522	chr3R	-	4448033	4448289	4452932	4453120	4447700	4447954	4453191	4453316	0.001859175	0.583
91	FBgn0032946	chr2L	+	21380470	21380478	21380679	21380728	21380132	21380361	2.1E+07	21382330	0.038927644	-0.548
92	FBgn0032946	chr2L	+	21380470	21380600	21380679	21380826	21380132	21380361	2.1E+07	21382330	1.0615E-05	0.468
93	FBgn0032946	chr2L	+	21380470	21380606	21380679	21380826	21380132	21380361	2.1E+07	21382330	3.91108E-07	0.761
94	FBgn0032946	chr2L	+	21380470	21380606	21380858	21381199	21380132	21380361	2.1E+07	21382330	0.01915756	0.314

95	FBgn0032946	chr2L	+	21380470	21380606	21380963	21381199	21380132	21380361	2.1E+07	21382330	0.0228717	0.314
96	FBgn0032946	chr2L	+	21380470	21380709	21380858	21381199	21380132	21380361	2.1E+07	21382330	0.000140741	0.363
97	FBgn0032946	chr2L	+	21380470	21380709	21380963	21381199	21380132	21380361	2.1E+07	21382330	0.000142012	0.361
98	FBgn0032946	chr2L	+	21380470	21380709	21381006	21381199	21380132	21380361	2.1E+07	21382330	0.003101762	0.231
99	FBgn0032946	chr2L	+	21380470	21380709	21381006	21381278	21380132	21380361	2.1E+07	21382330	0.000278261	0.345
100	FBgn0032946	chr2L	+	21380470	21380709	21381080	21381199	21380132	21380361	2.1E+07	21382330	0.007029652	0.373
101	FBgn0032946	chr2L	+	21380470	21380709	21381080	21381278	21380132	21380361	2.1E+07	21382330	9.22781E-05	0.777
102	FBgn0032946	chr2L	+	21380470	21380728	21380858	21381199	21380132	21380361	2.1E+07	21382330	3.52381E-06	0.607
103	FBgn0032946	chr2L	+	21380470	21380728	21380918	21381199	21380132	21380361	2.1E+07	21382330	3.47742E-08	0.568
104	FBgn0032946	chr2L	+	21380470	21380728	21380918	21381278	21380132	21380361	2.1E+07	21382330	0.002780794	0.512
105	FBgn0032946	chr2L	+	21380470	21380728	21380963	21381199	21380132	21380361	2.1E+07	21382330	4.3944E-06	0.602
106	FBgn0032946	chr2L	+	21380470	21380728	21381006	21381199	21380132	21380361	2.1E+07	21382330	1.79087E-06	0.587
107	FBgn0032946	chr2L	+	21380470	21380728	21381080	21381199	21380132	21380361	2.1E+07	21382330	1.37962E-05	0.611
108	FBgn0032946	chr2L	+	21380470	21380728	21381372	21381668	21380132	21380361	2.1E+07	21382330	0	0.653
109	FBgn0032946	chr2L	+	21380470	21380728	21381372	21381788	21380132	21380361	2.1E+07	21382330	1.05128E-07	0.447
110	FBgn0032946	chr2L	+	21380470	21380728	21381372	21381832	21380132	21380361	2.1E+07	21382330	9.44119E-09	0.559
111	FBgn0032946	chr2L	+	21380470	21380728	21381644	21381668	21380132	21380361	2.1E+07	21382330	3.51682E-13	0.6
112	FBgn0032946	chr2L	+	21380470	21380728	21381644	21381788	21380132	21380361	2.1E+07	21382330	1.082E-07	0.447
113	FBgn0032946	chr2L	+	21380470	21380728	21381644	21381832	21380132	21380361	2.1E+07	21382330	1.18529E-08	0.559
114	FBgn0032946	chr2L	+	21380647	21380728	21380918	21381199	21380132	21380478	2.1E+07	21382330	1.08614E-06	0.633
115	FBgn0032946	chr2L	+	21380647	21380728	21380918	21381278	21380132	21380478	2.1E+07	21382330	0.006229358	0.494
116	FBgn0032946	chr2L	+	21380647	21380826	21380918	21381199	21380132	21380478	2.1E+07	21382330	2.04852E-06	-0.313
117	FBgn0032946	chr2L	+	21380679	21380728	21380858	21381199	21380132	21380361	2.1E+07	21382330	0.001677702	0.564
118	FBgn0032946	chr2L	+	21380679	21380728	21380918	21381199	21380132	21380361	2.1E+07	21382330	0.000167748	0.421
119	FBgn0032946	chr2L	+	21380679	21380728	21380918	21381278	21380132	21380361	2.1E+07	21382330	0.002304995	0.522
120	FBgn0032946	chr2L	+	21380679	21380728	21380963	21381199	21380132	21380361	2.1E+07	21382330	0.002042078	0.558
121	FBgn0032946	chr2L	+	21380679	21380728	21381006	21381199	21380132	21380361	2.1E+07	21382330	0.004188421	0.469
122	FBgn0032946	chr2L	+	21380679	21380728	21381080	21381199	21380132	21380361	2.1E+07	21382330	0.004257294	0.562
123	FBgn0032946	chr2L	+	21380679	21380728	21381372	21381668	21380132	21380361	2.1E+07	21382330	3.80567E-07	0.489
124	FBgn0032946	chr2L	+	21380679	21380728	21381372	21381788	21380132	21380361	2.1E+07	21382330	0.002187438	0.255
125	FBgn0032946	chr2L	+	21380679	21380728	21381372	21381832	21380132	21380361	2.1E+07	21382330	2.75107E-05	0.399
126	FBgn0032946	chr2L	+	21380679	21380728	21381644	21381668	21380132	21380361	2.1E+07	21382330	2.52037E-06	0.383
127	FBgn0032946	chr2L	+	21380679	21380728	21381644	21381788	21380132	21380361	2.1E+07	21382330	0.002352197	0.253
128	FBgn0032946	chr2L	+	21380679	21380728	21381644	21381832	21380132	21380361	2.1E+07	21382330	3.32273E-05	0.398
129	FBgn0032946	chr2L	+	21380679	21380728	21380918	21381199	21380132	21380478	2.1E+07	21382330	0.000375154	0.594
130	FBgn0032946	chr2L	+	21380679	21380728	21380918	21381278	21380132	21380478	2.1E+07	21382330	4.97214E-05	0.613
131	FBgn0032946	chr2L	+	21380679	21380826	21380858	21381199	21380132	21380361	2.1E+07	21382330	0.012868854	-0.202
132	FBgn0032946	chr2L	+	21380679	21380826	21380918	21381199	21380132	21380361	2.1E+07	21382330	0.004547626	-0.219
133	FBgn0032946	chr2L	+	21380679	21380826	21380918	21381278	21380132	21380361	2.1E+07	21382330	0.001677702	-0.423
134	FBgn0032946	chr2L	+	21380679	21380826	21380963	21381199	21380132	21380361	2.1E+07	21382330	0.010436783	-0.207
135	FBgn0032946	chr2L	+	21380679	21380826	21381006	21381199	21380132	21380361	2.1E+07	21382330	0.003101762	-0.205
136	FBgn0032946	chr2L	+	21380679	21380826	21381006	21381278	21380132	21380361	2.1E+07	21382330	0.000909371	-0.479
137	FBgn0032946	chr2L	+	21380679	21380826	21381080	21381199	21380132	21380361	2.1E+07	21382330	0.028480825	-0.144
138	FBgn0032946	chr2L	+	21380679	21380826	21381372	21381788	21380132	21380361	2.1E+07	21382330	0.001099891	-0.18
139	FBgn0032946	chr2L	+	21380679	21380826	21381372	21381832	21380132	21380361	2.1E+07	21382330	0.007667272	-0.221
140	FBgn0032946	chr2L	+	21380679	21380826	21381644	21381788	21380132	21380361	2.1E+07	21382330	0.0019943	-0.18
141	FBgn0032946	chr2L	+	21380679	21380826	21381644	21381832	21380132	21380361	2.1E+07	21382330	0.006810712	-0.223
142	FBgn0025683	chr2L	-	127380	127797	128554	128743	126109	126227	128799	129245	0.039797723	-0.445
143	FBgn0265959	chr3L	+	20258711	20259085	20260209	20260310	20255897	20256638	2E+07	20260989	0.048295451	-0.238
144	FBgn0261625	chr2R	-	12673898	12673928	12674205	12674702	12673099	12673339	1.3E+07	12675418	0.002514736	-0.155
145	FBgn0031213	chr2L	+	73389	73620	74479	74572	72386	72977	74902	75018	0.010986767	-0.309
146	FBgn0014184	chr2R	-	12169026	12169254	12169722	12170044	12167993	12168203	1.2E+07	12174264	1.68005E-08	-1
147	FBgn0014184	chr2R	-	12169722	12170044	12173275	12173458	12167993	12168203	1.2E+07	12174264	0.000173298	0.833
148	FBgn0259923	chrX	-	16694613	16694809	16694830	16694836	16694042	16694371	1.7E+07	16695144	0.000784659	0.768
149	FBgn0031449	chr2L	+	2735720	2736136	2736173	2736220	2730103	2730469	2736310	2736430	9.20131E-06	0.746
150	FBgn0031449	chr2L	+	2735720	2736136	2736173	2736229	2730103	2730469	2736310	2736430	1.80739E-06	0.749
151	FBgn0031449	chr2L	+	2735890	2736136	2736173	2736220	2730103	2730469	2736310	2736430	4.82874E-06	0.724
152	FBgn0031449	chr2L	+	2735890	2736136	2736173	2736229	2730103	2730469	2736310	2736430	3.58886E-07	0.733
153	FBgn0030830	chrX	+	17222137	17222140	17222149	17222220	17221441	17221772	1.7E+07	17222651	0.002902998	0.667
154	FBgn0033054	chr2R	-	6038507	6038617	6039696	6039790	6038070	6038446	6040081	6040334	0.000356764	-0.362
155	FBgn0033054	chr2R	-	6038507	6038625	6039696	6039790	6038070	6038446	6040081	6040334	0.0019986	-0.293
156	FBgn0033054	chr2R	-	6038507	6038721	6039696	6039790	6038070	6038446	6040081	6040334	0.001450995	-0.354
157	FBgn0033807	chr2R	-	13213532	13213558	13213750	13213931	13213022	13213436	1.3E+07	13214496	0.010233232	0.136
158	FBgn0032901	chr2L	-	20882029	20882464	20885288	20885584	20877552	20877677	2.1E+07	20890395	0.000173298	0.833
159	FBgn0038652	chr3R	-	18792793	18792879	18793048	18793159	18792050	18792120	1.9E+07	18793833	0.011073583	0.738
160	FBgn0001128	chr2L	+	5944651	5944862	5945748	5945885	5944267	5944445	5946043	5946250	0.046375401	-0.389
161	FBgn0001128	chr2L	+	5944655	5944862	5945748	5945885	5944267	5944445	5946043	5946250	0.00241443	-0.486
162	FBgn0015221	chr3R	+	30389415	30389436	30389436	30389442	30389198	30389328	3E+07	30390584	1.60588E-05	-0.186
163	FBgn0024804	chr3L	+	27292886	27293013	27323293	27323574	27279480	27279539	2.7E+07	27332108	0.022183832	-0.36
164	FBgn0036821	chr3L	-	18888123	18888313	18889200	18889516	18887552	18887746	1.9E+07	18890483	0.001277286	0.768
165	FBgn0011224	chr3R	-	32010576	32010675	32011039	32011102	31979522	31979619	3.2E+07	32015520	1.91718E-09	-0.954
166	FBgn0032684	chr2L	+	18509985	18510058	18510949	18511075	18509559	18509706	1.9E+07	18511878	0.045171494	-0.465
167	FBgn0032684	chr2L	+	18509985	18510058	18510890	18511075	18509559	18509778	1.9E+07	18511878	0.029646032	-0.725
168	FBgn0027492	chr3R	-	27580653	27580754	27581042	27581205	27580156	27580325	2.8E+07	27582241	0.004547626	-0.696
169	FBgn0033159	chr2R	-	7321366	7321646	7322382	7322397	7319553	7320764	7322559	7322718	7.37711E-10	-0.86
170	FBgn0033159	chr2R	-	7338249	7338537	7344278	7344558	7332260	7332380	7347848	7348010	0.007055756	0.383
171	FBgn0036556	chr3L	-	15999169	15999238	16002660	16003005	15995609	15995845	1.6E+07	16004074	0.016232278	0.465
172	FBgn0010482	chr2R	-	6729999	6730114	6730300	6731266	6728590	6729073	6731439	6731490	0.000984545	0.34
173	FBgn0259110	chrX	-	15972431	15972582	15972639	15972733	15972204	15972329	1.6E+07	15973067	0.000398274	-0.636
174	FBgn0030183	chrX	-	10210551	10210566	10210717	10211100	10207977	10208294	1E+07	10212374	0.032658153	-0.738
175	FBgn0030183	chrX	-	10210551	10210566	10210717	10211172	10207977	10208294	1E+07	10212374	0.032658153	-0.738
176	FBgn0038063	chr3R	-	12561942	12562049	12562818	12563119	12560400	12561804	1.3E+07	12595725	0.000618901	-0.641
177	FBgn0263077	chr2R	+	7637283	7637365	7638052	7638180	7636894	7637193	7638597	7638905	0.014945652	-0.359
178	FBgn0263077	chr2R	+	7637283	7637365	7638052	7638313	7636894	7637193	7638597	7638905	0.045272846	-0.335
179	FBgn0263077	chr2R	+	7637283	7637365	7638052	7638329	7636894	7637193	7638597	7638905	0.046547921	-0.329
180	FBgn0263077	chr2R	+	7637283	7637365	7638122	7638313	7636894	7637193	7638597	7638905	0.000436343	-0.448
181	FBgn0263077	chr2R	+	7637283	7637365	7638122	7638329	7636894	7637193	7638597	7638905	0.000846997	-0.446
182	FBgn0034051	chr2R	-	15937678	15937879	15938114	15938125	15935892	15936580	1.6E+07	15938978	0.000278797	0.182
183	FBgn0034051	chr2R	-	15937678	15938072	15938114	15938125	15935892	15936580	1.6E+07	15938978	1.76759E-07	0.288
184	FBgn0005614	chr2R	+	9753896	9753977	9755282	9755759	9753696	9753839	9761411	9761554	0.012246086	-0.492
185	FBgn0016126	chr4	+	445912	446171	446820	446987	445503	445648	447100	447235	0.014632483	0.41
186	FBgn0051999	chr4	-	219237	219617	219641	219796	218445	218890	219890	220028	0.013807656	-0.371
187	FBgn0011642	chr4	-	1060492	1060634	1064244	1064381	1059418	1059836	1064822	1065001	2.97847E-09	-0.575
188	FBgn0011642	chr4	-	1060492	1060634	1064244	1064506	1059418	1059836	1064822	1065001	0.0091058	-0.449
189	FBgn0002921	chr3R	+	20959236	20959380	20960725	20960829	20948779	20948829	2.1E+07	20963856	0.038252453	0.258
190	FBgn0002921	chr3R	+	20959236	20959380	20960725	20960841	20948779	20948829	2.1E+07	20963856	0.024082432	0.403
191	FBgn0002921	chr3R	+	20959236	20959380	20960725	20961113	20948779	20948829	2.1E+07	20963856	0.008834122	0.453
192	FBgn0027515	chr2L	+	7821549	7821648	7821717	7821892	7821150	7821308	7822641	7823100	0.000925513	0.371
193	FBgn0020496	chr3R	+	13014368	13014589	13018596	13018876	13011648	13011748	1.3E+07	13019814	1.72914E-08	-0.785
194	FBgn0020496	chr3R	+	13014472	13014589	13018596	13018876	13011648	13011748	1.3E+07	13019814	2.53756E-07	-0.759
195	FBgn0020496	chr3R	+	13014489	13014589	13018596	13018876	13011648	13011748	1.3E+07	13019814	2.81523E-07	-0.763
196	FBgn0264607	chr4	-	1040740	1041115	1042902	1043118	1040012	1040078	1043467	1043524	0.025461096	0.168
197	FBgn0264607	chr4	-	1050681	1050862	1051187	1051587	1050454	1050609	1053497	1053703	0.033686848	-0.37
198	FBgn0011361	chr3L	+	1330846	1330964	1331025	1331170	1330130	1330290	1331279	1331543	0.004828276	0.13
199	FBgn0011361	chr3L	+	1330846	1330964	1331029	1331170	1330130	1330290	1331279	1331543	0.005319204	0.129
200	FBgn0011361	chr3L	+	1330865	1330964	1331025	1331170	1330130	1330290	1331279	1331543	0.007062964	0.13
201	FBgn0011361	chr3L	+	1330865	1330964	1331029	1331170	1330130	1330290	1331279	1331543	0.007667272	0.129
202	FBgn0265991	chr2R	-	15808179	15808259	15814310	15814544	15803360	15803487	1.6E+07	15814917	8.26196E-10	-1
203	FBgn0261985	chr3L	+	348122	348217	351083	351286	347458	347779	351366	351778	0.0091058	0.526
204	FBgn0260938	chrX	-	15886045	15886352	15886505	15886796	15884442	15885943	1.6E+07	15887311	0.013004431	-0.583
205	FBgn0260938	chrX	-	15886192	15886264	15886505	15886796	15884442	15885943	1.6E+07	15887311	0.046629988	-0.629
206	FBgn0260938	chrX	-	15886192	15886352	15886505	15886796	15884442	15885943	1.6E+07	15887311	0.015554595	-0.462
207	FBgn0261788	chr3L	-	7656374	7661506	7662704	7662890	7655388	7656203	7663258	7663324	1.8094E-10	0.23

RI											
	FlyBase ID	chr	strand	riExonStart_0base	riExonEnd	upstreamES	upstreamEE	downstreamES	downstreamEE	FDR	ΔPSI
1	FBgn0267849	chr3L	+	21445833	21446375	21445833	21446023	21446197	21446375	5.1E-12	0.179
2	FBgn0267487	chr3L	-	1346276	1349542	1346276	1346450	1349198	1349542	1.4E-06	0.822
3	FBgn0267376	chr3R	-	10866808	10868290	10866808	10866960	10867971	10868290	0	0.892
4	FBgn0267376	chr3R	-	10866808	10868642	10866808	10866960	10868438	10868642	0	0.797
5	FBgn0267252	chr2L	+	9256562	9257206	9256562	9256814	9256892	9257206	0	0.463
6	FBgn0267252	chr2L	+	9256562	9272607	9256562	9256814	9272465	9272607	0	0.428
7	FBgn0266139	chr2L	+	10208999	10209786	10208999	10209397	10209484	10209786	0.01794	-0.358
8	FBgn0266417	chr3L	-	16355633	16356448	16355633	16356036	16356304	16356448	0.0132	-0.436
9	FBgn0265413	chrX	-	9072819	9078499	9072819	9073305	9078336	9078499	0.00022	0.681
10	FBgn0265191	chr2R	+	21205360	21205814	21205360	21205541	21205745	21205814	0.03035	0.228
11	FBgn0264695	chr2L	+	16774449	16775381	16774449	16774857	16775210	16775381	0.00458	0.126
12	FBgn0264695	chr2L	+	16775210	16777439	16775210	16775381	16776849	16777439	0.00644	0.135
13	FBgn0264386	chrX	-	6108029	6111823	6108029	6109946	6111673	6111823	0	-0.282
14	FBgn0264386	chrX	-	6115416	6118283	6115416	6115572	6118117	6118283	0.02054	-0.106
15	FBgn0264386	chrX	-	6118117	6123598	6118117	6118283	6120234	6123598	0.03223	-0.334
16	FBgn0264386	chrX	-	6139605	6146219	6139605	6139717	6145264	6146219	0	0.754
17	FBgn0264002	chr3L	+	2282996	2297723	2282996	2283036	2297474	2297723	0.00575	0.502
18	FBgn0263980	chr2R	+	15464470	15465491	15464470	15464686	15465330	15465491	0.00692	-0.281
19	FBgn0263773	chr2L	-	20079269	20085070	20079269	20079472	20084577	20085070	0.00867	0.336
20	FBgn0263603	chr3L	-	16110482	16112098	16110482	16111914	16111997	16112098	0.0191	0.503
21	FBgn0263593	chr2R	-	8140242	8140613	8140242	8140371	8140513	8140613	0.00079	-0.137
22	FBgn0263346	chr3R	+	4710615	4711945	4710615	4710720	4710897	4711945	6.9E-09	-0.144
23	FBgn0263197	chr2R	-	17531210	17531653	17531210	17531446	17531517	17531653	9E-05	-0.544
24	FBgn0262975	chr3R	-	23191840	23193280	23191840	23192281	23193025	23193280	0.00334	0.525
25	FBgn0262975	chr3R	-	23222796	23226711	23222796	23223120	23226453	23226711	0.0048	0.567
26	FBgn0262614	chr3R	-	8844915	8846289	8844915	8845150	8846160	8846289	2.6E-05	0.552
27	FBgn0262614	chr3R	-	8865404	8867435	8865404	8866025	8867271	8867435	0.01124	0.589
28	FBgn0262574	chr2R	+	11310830	11311165	11310830	11310916	11310979	11311165	0.00117	0.579
29	FBgn0262570	chr2R	-	17482790	17483433	17482790	17482839	17482905	17483433	0.00093	-0.289
30	FBgn0261954	chrX	+	2010859	2018004	2010859	2010960	2014341	2018004	0.03273	-0.454
31	FBgn0266696	chr3L	+	2439235	2439830	2439235	2439415	2439498	2439830	0.00017	0.349
32	FBgn0261625	chr2R	-	12673099	12675418	12673099	12673339	12674990	12675418	9E-05	-0.422
33	FBgn0261625	chr2R	-	12673898	12675418	12673898	12673928	12674990	12675418	0	-0.495
34	FBgn0261625	chr2R	-	12673099	12676231	12673099	12673339	12676124	12676231	0.03241	0.293
35	FBgn0261554	chr2R	+	21295651	21300371	21295651	21296213	21299836	21300371	0.01495	-0.322
36	FBgn0261283	chr3L	+	19793934	19794325	19793934	19794159	19794223	19794325	0.01917	-0.279
37	FBgn0261283	chr3L	+	19793934	19794699	19793934	19794159	19794673	19794699	0.00224	0.468
38	FBgn0260990	chr3R	+	29752138	29752731	29752138	29752267	29752328	29752731	0	-0.229
39	FBgn0260964	chr2R	-	13527887	13529875	13527887	13528386	13529510	13529875	0.00677	-0.201
40	FBgn0260945	chr3L	-	12788540	12797856	12788540	12788628	12797682	12797856	0.00189	0.103
41	FBgn0260938	chrX	-	15884442	15887311	15884442	15885943	15887117	15887311	0.04007	0.157
42	FBgn0260660	chr3L	+	7019625	7034538	7019625	7019898	7034502	7034538	0.01036	0.535
43	FBgn0260462	chr3R	-	5239491	5244254	5239491	5239619	5243837	5244254	0.00636	-0.154
44	FBgn0259984	chr2L	+	13550138	13551666	13550138	13550293	13551141	13551666	0.00315	-0.385
45	FBgn0259926	chrX	+	5683239	5684456	5683239	5684102	5684173	5684456	1.7E-05	0.767
46	FBgn0259179	chr2R	+	22825092	22827333	22825092	22825250	22827261	22827333	0.00936	-0.517
47	FBgn0250867	chr2L	-	20965519	20967746	20965519	20966080	20966303	20967746	0	0.464
48	FBgn0250839	chr3R	-	4956998	4957442	4956998	4957238	4957289	4957442	0.00077	0.395
49	FBgn0265001	chr2L	-	9770398	9770996	9770398	9770605	9770883	9770996	0.00017	0.722
50	FBgn0265194	chr2R	-	15273325	15274691	15273325	15273388	15274538	15274691	0.0332	0.3
51	FBgn0085427	chr2L	+	8930483	8931147	8930483	8930550	8931081	8931147	0.01042	-0.308
52	FBgn0085427	chr2L	+	8931081	8933382	8931081	8931147	8932900	8933382	0.00856	-0.138
53	FBgn0085421	chr2R	-	6764374	6765697	6764374	6764736	6765535	6765697	0	0.861
54	FBgn0085399	chr2R	-	22037391	22039431	22037391	22037719	22039355	22039431	4E-13	0.837
55	FBgn0085399	chr2R	-	22037391	22039559	22037391	22037716	22039403	22039559	0	-0.531

56	FBgn0085390	chr2R	-	7850926	7852346	7850926	7851052	7852265	7852346	0.01445	-0.324
57	FBgn0085390	chr2R	-	7852265	7853591	7852265	7852346	7853405	7853591	0.00182	-0.608
58	FBgn0085342	chr2L	+	16879516	16880660	16879516	16879920	16880588	16880660	7E-08	0.664
59	FBgn0085305	chr3R	+	15925628	15926302	15925628	15925648	15925708	15926302	0.00979	-0.527
60	FBgn0082831	chr3R	+	12414351	12416582	12414351	12414756	12414870	12416582	7.7E-08	-0.311
61	FBgn0082582	chr3R	+	30532606	30533507	30532606	30532982	30533118	30533507	1.2E-06	0.2
62	FBgn0082582	chr3R	+	30546405	30548749	30546405	30546587	30548420	30548749	0	-0.679
63	FBgn0082582	chr3R	+	30546404	30548749	30546404	30546587	30548451	30548749	0	-0.622
64	FBgn0053978	chr4	-	158643	159712	158643	158662	159221	159712	0.01826	0.557
65	FBgn0053087	chr2R	+	8174164	8186982	8174164	8174831	8186904	8186982	0.00308	-0.7
66	FBgn0259734	chrX	+	9042643	9050935	9042643	9042830	9049249	9050935	0.00094	-0.392
67	FBgn0052447	chr3L	-	21690611	21706365	21690611	21693721	21704547	21706365	0.03225	0.748
68	FBgn0052333	chr3L	+	1289998	1290473	1289998	1290028	1290285	1290473	0.00176	-0.285
69	FBgn0052105	chr3L	+	12329231	12335085	12329231	12330253	12334209	12335085	0.02494	0.333
70	FBgn0051975	chr2L	-	139740	140992	139740	139954	140113	140992	0.01043	0.546
71	FBgn0051973	chr2L	-	34719	38731	34719	34912	38534	38731	0.00481	-0.17
72	FBgn0051781	chr2L	+	16869461	16878300	16869461	16870997	16877499	16878300	0.01003	0.373
73	FBgn0051678	chr2L	-	20663997	20675570	20663997	20664209	20674994	20675570	0.0332	0.134
74	FBgn0051619	chr2L	+	21684105	21684911	21684105	21684339	21684545	21684911	0.02384	0.342
75	FBgn0051716	chr2L	+	10397178	10398219	10397178	10397474	10398034	10398219	9.9E-05	0.429
76	FBgn0053182	chr2R	-	13176552	13180110	13176552	13179013	13179587	13180110	0.00495	-0.488
77	FBgn0051100	chr3R	-	9000286	9001886	9000286	9000544	9001768	9001886	0.00011	0.35
78	FBgn0050390	chr2R	-	21293320	21293646	21293320	21293414	21293496	21293646	0.00429	-0.541
79	FBgn0050295	chr2R	+	20875604	20876621	20875604	20875663	20876474	20876621	2.4E-08	0.612
80	FBgn0050158	chr2R	+	6891759	6892026	6891759	6891923	6891979	6892026	0.01487	-0.171
81	FBgn0050156	chr2R	-	6981445	6981909	6981445	6981685	6981742	6981909	0.03368	-0.298
82	FBgn0050050	chr2R	+	12409100	12409735	12409100	12409424	12409474	12409735	8.8E-07	-0.253
83	FBgn0265920	chr3L	-	4494651	4495215	4494651	4495059	4495120	4495215	0.0001	0.782
84	FBgn0044047	chrX	-	2331925	2332408	2331925	2332139	2332343	2332408	2.8E-10	0.963
85	FBgn0032796	chr2L	-	19507489	19508764	19507489	19508265	19508325	19508764	4E-13	0.82
86	FBgn0032796	chr2L	-	19507489	19508764	19507489	19508265	19508351	19508764	1.7E-05	0.411
87	FBgn0260388	chr3L	-	16495299	16496236	16495299	16495536	16496052	16496236	3.8E-10	-0.669
88	FBgn0260388	chr3L	-	16495299	16496236	16495299	16495536	16496138	16496236	5.2E-10	0.651
89	FBgn0003867	chr3R	-	21779551	21781905	21779551	21779783	21781717	21781905	0.01954	0.339
90	FBgn0265959	chr3L	+	20248079	20256638	20248079	20248325	20255897	20256638	0.03046	0.262
91	FBgn0265959	chr3L	+	20248627	20256638	20248627	20249060	20255897	20256638	7E-07	0.677
92	FBgn0265959	chr3L	+	20263176	20263733	20263176	20263584	20263633	20263733	9E-05	0.108
93	FBgn0031952	chr2L	-	7801667	7804918	7801667	7803576	7804570	7804918	0	-0.469
94	FBgn0031935	chr2L	-	7718977	7719487	7718977	7719109	7719251	7719487	7.9E-05	0.755
95	FBgn0031816	chr2L	-	6463261	6465669	6463261	6464139	6465528	6465669	0.00769	-0.218
96	FBgn0031816	chr2L	-	6463261	6465669	6463261	6464139	6465550	6465669	0.00149	-0.32
97	FBgn0004242	chr2L	-	2789346	2792371	2789346	2789946	2792194	2792371	1.1E-12	-0.497
98	FBgn0004242	chr2L	-	2793647	2795544	2793647	2793908	2795488	2795544	6.9E-05	-0.9
99	FBgn0004242	chr2L	-	2798485	2799979	2798485	2798836	2799908	2799979	0.0373	0.404
100	FBgn0031589	chr2L	-	4196474	4197380	4196474	4196814	4197146	4197380	0.00054	0.445
101	FBgn0031589	chr2L	-	4196474	4198071	4196474	4196814	4197964	4198071	0.00075	0.38
102	FBgn0031636	chr2L	+	4838461	4839510	4838461	4838530	4838991	4839510	0.02141	0.469
103	FBgn0031636	chr2L	+	4840883	4841465	4840883	4840983	4841135	4841465	0	-0.276
104	FBgn0031673	chr2L	-	5042490	5043158	5042490	5042887	5042983	5043158	3.5E-05	0.401
105	FBgn0031673	chr2L	-	5042490	5043158	5042490	5042890	5043077	5043158	0.01242	0.37
106	FBgn0039719	chr3R	+	29905463	29906099	29905463	29905515	29905971	29906099	0.00052	-0.186
107	FBgn0033979	chr2R	+	14877785	14879533	14877785	14878886	14878940	14879533	0.00375	0.743
108	FBgn0035240	chr3L	+	1602953	1603399	1602953	1603249	1603306	1603399	0.02075	0.631
109	FBgn0284246	chr2R	-	6074470	6076114	6074470	6075000	6075063	6076114	0.00406	-0.315
110	FBgn0031016	chrX	+	19353180	19363021	19353180	19353583	19362966	19363021	0.02044	0.446
111	FBgn0030749	chrX	-	16418667	16422242	16418667	16419049	16422166	16422242	0.00388	0.448
112	FBgn0030669	chrX	-	15700171	15701259	15700171	15700807	15700904	15701259	0.00209	0.553
113	FBgn0039774	chr3R	+	30459786	30462969	30459786	30459842	30462814	30462969	0.01245	-0.465
114	FBgn0031196	chrX	-	22542968	22544322	22542968	22544135	22544201	22544322	1.7E-08	1
115	FBgn0030091	chrX	+	9142492	9144947	9142492	9142786	9142861	9144947	5.6E-05	0.75
116	FBgn0028487	chr3R	-	13696436	13697227	13696436	13696768	13696826	13697227	8.8E-07	-0.545
117	FBgn0263995	chr3R	+	18010227	18015779	18010227	18010574	18013149	18015779	0.04032	-0.333
118	FBgn0033500	chr2R	+	10249219	10254025	10249219	10249439	10252957	10254025	1E-05	-0.165
119	FBgn0040001	chr3L	-	25752858	25754790	25752858	25753634	25753694	25754790	0.0103	-0.805
120	FBgn0040001	chr3L	-	25753694	25755202	25753694	25754790	25754852	25755202	1.1E-06	-0.904
121	FBgn0040001	chr3L	-	25755783	25757738	25755783	25756396	25756460	25757738	0.03677	-0.46
122	FBgn0028931	chr2L	+	13790958	13791595	13790958	13791164	13791217	13791595	0.00317	0.412
123	FBgn0038419	chr3R	-	16345824	16347528	16345824	16345998	16347055	16347528	2.2E-05	-0.765
124	FBgn0028546	chr2L	-	13642019	13642396	13642019	13642192	13642295	13642396	0.03396	0.128
125	FBgn0028490	chr2L	-	11524567	11526012	11524567	11525667	11525916	11526012	4E-07	-0.536
126	FBgn0028490	chr2L	-	11524567	11528205	11524567	11525667	11528120	11528205	6.5E-07	0.532
127	FBgn0041789	chr2L	-	19419868	19423552	19419868	19420191	19423414	19423552	0.02662	0.312
128	FBgn0028703	chr2L	-	6677834	6679262	6677834	6678756	6678911	6679262	0.03306	0.213
129	FBgn0028703	chr2L	-	6678911	6680114	6678911	6679082	6679985	6680114	0.03812	0.152
130	FBgn0028703	chr2L	-	6681463	6682084	6681463	6681772	6681942	6682084	0	0.862
131	FBgn0032600	chr2L	+	16743404	16744067	16743404	16743570	16743759	16744067	0	0.313
132	FBgn0261836	chr2L	+	5204450	5204903	5204450	5204592	5204835	5204903	0.00018	0.1
133	FBgn0037565	chr3R	-	8299287	8300743	8299287	8299948	8300723	8300743	0.03391	0.529
134	FBgn0027356	chr2R	+	12608055	12609876	12608055	12608178	12609168	12609876	5E-07	0.164
135	FBgn0027339	chr3L	-	22799250	22800784	22799250	22799448	22800024	22800784	0.02612	0.195
136	FBgn0003174	chr2R	+	7282571	7283727	7282571	7282772	7283563	7283727	0.00157	-0.637
137	FBgn0030364	chrX	+	12018639	12019758	12018639	12019325	12019627	12019758	0	0.685
138	FBgn0016034	chr3L	+	22724192	22724929	22724192	22724685	22724739	22724929	0.02287	-0.435
139	FBgn0052442	chr3L	-	21528769	21529085	21528769	21528896	21528957	21529085	0.0373	-0.286
140	FBgn0052442	chr3L	-	21528769	21529085	21528769	21528908	21528957	21529085	3.2E-09	-0.643
141	FBgn0052442	chr3L	-	21528769	21529085	21528769	21528908	21528965	21529085	0.01106	-0.234
142	FBgn0052442	chr3L	-	21528957	21529333	21528957	21529085	21529145	21529333	0.00078	-0.181
143	FBgn0010415	chr2R	-	21480090	21481176	21480090	21480378	21481013	21481176	0.02079	-0.404
144	FBgn0027596	chr2R	-	14905513	14907971	14905513	14905953	14907487	14907971	0.00078	-0.51
145	FBgn0032938	chr2L	+	21263647	21273771	21263647	21263786	21273323	21273771	0.00013	0.104
146	FBgn0032938	chr2L	+	21273323	21274139	21273323	21273771	21273827	21274139	6.5E-07	0.124
147	FBgn0032938	chr2L	+	21275723	21278429	21275723	21275806	21277196	21278429	0.0042	-0.359
148	FBgn0004907	chr2R	+	10099676	10104409	10099676	10099738	10104255	10104409	0.03625	0.523
149	FBgn0004907	chr2R	+	10101035	10104409	10101035	10101097	10104255	10104409	0.03848	0.299
150	FBgn0036044	chr3L	-	9788274	9788972	9788274	9788744	9788939	9788972	0.00232	-0.5
151	FBgn0036044	chr3L	-	9787932	9790673	9787932	9788744	9790455	9790673	0.00071	-0.584
152	FBgn0260794	chr3R	-	4801212	4806463	4801212	4801991	4806361	4806463	3.1E-12	0.609
153	FBgn0004595	chr3R	+	11371312	11378189	11371312	11377217	11378034	11378189	0	0.897
154	FBgn0052062	chr3L	+	10516002	10518306	10516002	10516162	10517961	10518306	0.01272	0.597
155	FBgn0031925	chr2L	+	7605599	7606334	7605599	7605844	7605947	7606334	0.04388	0.395
156	FBgn0004838	chr2L	+	6920703	6924400	6920703	6920861	6923783	6924400	0.04602	-0.619
157	FBgn0039271	chr3R	+	25069926	25070914	25069926	25070050	25070202	25070914	0	0.855
158	FBgn0011224	chr3R	-	31935665	31937392	31935665	31935775	31937269	31937392	0.02073	0.393
159	FBgn0034093	chr2R	-	16225106	16226252	16225106	16225325	16226058	16226252	1.9E-05	-0.564
160	FBgn0250824	chr2R	-	21317792	21320017	21317792	21319803	21319864	21320017	7.2E-06	0.761
161	FBgn0013305	chr2R	-	12878384	12879146	12878384	12878710	12879051	12879146	4.2E-09	0.583
162	FBgn0013305	chr2R	-	12879051	12879656	12879051	12879146	12879447	12879656	0.04136	-0.353
163	FBgn0003371	chrX	+	2641589	2645418	2641589	2642093	2645375	2645418	3.8E-08	0.783
164	FBgn0003371	chrX	+	2649735	2670103	2649735	2649739	2669912	2670103	0.02447	0.583
165	FBgn0028496	chr2R	-	18496746	18498026	18496746	18496905	18497755	18498026	0.0034	-0.354
166	FBgn0028496	chr2R	-	18496746	18498026	18496746	18496868	18497841	18498026	1E-08	0.438
167	FBgn0028496	chr2R	-	18496746	18498026	18496746	18496905	18497841	18498026	0.04361	-0.226
168	FBgn0042135	chr2R	-	7725867	7726430	7725867	7725995	7726294	7726430	0.01346	0.321
169	FBgn0261556	chr3L	-	20168475	20192299	20168475	20169063	20190657	20192299	8E-05	-0.778
170	FBgn0037581	chr3R	+	8347578	8348624	8347578	8347635	8347777	8348624	7E-05	0.772
171	FBgn0030869	chrX	-	17823190	17825481	17823190	17823318	17823377	17825481	0.04625	-0.269
172	FBgn0037698	chr3R	-	9467353	9468404	9467353	9467440	9467979	9468404	0.00053	-0.337
173	FBgn0029820	chrX	+	5880533	5881321	5880533	5880669	5880833	5881321	0.01327	0.466
174	FBgn0003435	chr2R	-	19546011	19564512	19546011	19546059	19564461	19564512	0.01102	-0.45
175	FBgn0265376	chr3R	-	4596282	4598871	4596282	4597174	4598468	4598871	3.3E-07	0.444
176	FBgn0043070	chr2R	+	21502096	21507722	21502096	21502479	21507294	21507722	0	-0.239
177	FBgn0043070	chr2R	+	21502096	21507722	21502096	21502479	21507321	21507722	5.9E-05	-0.256
178	FBgn0043070	chr2R	+	21502096	21510201	21502096	21502479	21510105	21510201	5E-13	-0.237
179	FBgn0043070	chr2R	+	21502096	21510201	21502096	21502479	21510117	21510201	4.5E-07	-0.158
180	FBgn0043070	chr2R	+	21502096	21511060	21502096	21502479	21510866	21511060	6.1E-07	-0.097
181	FBgn0010622	chr2R	-	23061419	23062593	23061419	23061594	23061682	23062593	4.8E-05	-0.347
182	FBgn0010225	chr3R	+	4236087	4236804	4236087	4236281	4236552	4236804	0.04357	0.437
183	FBgn0034002	chr2R	+	15221727	15222425	15221727	15221823	15222043	15222425	0.03035	0.289
184	FBgn0027795	chrX	-	1455749	1457054	1455749	1455949	1456022	1457054	0.02604	-0.363
185	FBgn0010482	chr2R	-	6734728	6738127	6734728	6734911	6737960	6738127	0.00463	0.317
186	FBgn0031857	chr2L	-	6768845	6769736	6768845	6768968	6769397	6769736	4.9E-09	-0.239
187	FBgn0031857	chr2L	-	6768845	6769736	6768845	6769127	6769397	6769736	1.3E-07	0.22
188	FBgn0031857	chr2L	-	6770590	6770968	6770590	6770709	6770885	6770968	0	-0.51
189	FBgn0031857	chr2L	-	6770590	6772614	6770590	6770709	6772551	6772614	0	-0.979
190	FBgn0031450	chr2L	+	2740388	2740784	2740388	2740473	2740519	2740784	0.00386	-0.105
191	FBgn0016976	chrX	-	22994483	23004648	22994483	22995914	23004549	23004648	0.02196	0.386
192	FBgn0000581	chr2R	+	11452768	11454469	11452768	11452885	11453392	11454469	0.03935	-0.446
193	FBgn0038716	chr3R	+	19603374	19604256	19603374	19603412	19603833	19604256	6.9E-13	0.698
194	FBgn0033373	chr2R	-	9090810	9091653	9090810	9091051	9091406	9091653	0.04077	0.143
195	FBgn0000173	chrX	+	13996344	13996728	13996344	13996523	13996615	13996728	0.00809	0.527
196	FBgn0032172	chr2L	-	9960449	9962059	9960449	9961614	9961728	9962059	0.00208	-0.246
197	FBgn0033388	chr2R	-	9163328	9164025	9163328	9163474	9163702	9164025	0.02224	0.457
198	FBgn0028572	chr2L	+	5066011	5067249	5066011	5066071	5066869	5067249	3.5E-05	-0.462
199	FBgn0004179	chr3L	+	22271557	22273298	22271557	22271762	22272368	22273298	0.02928	-0.131
200	FBgn0037974	chr3R	-	11980703	11981550	11980703	11980854	11981011	11981550	0.0025	0.125
201	FBgn0031879	chr2L	-	7008399	7009909	7008399	7008649	7009872	7009909	0.00239	0.639
202	FBgn0031702	chr2L	-	5331721	5336170	5331721	5332850	5335458	5336170	0.00119	-0.423
203	FBgn0038740	chr3R	-	19874344	19875224	19874344	19874510	19875168	19875224	3.4E-05	-0.594
204	FBgn0038917	chr3R	+	21888838	21907182	21888838	21889267	21905927	21907182	0.00207	-0.538
205	FBgn0034691	chr2R	+	22080442	22082637	22080442	22080538	22081074	22082637	0.02986	0.344
206	FBgn0030850	chrX	-	17623220	17625658	17623220	17624205	17625529	17625658	0	0.794
207	FBgn0030850	chrX	-	17624263	17627240	17624263	17625658	17626939	17627240	1.1E-13	0.123
208	FBgn0265180	chr2R	+	21200482	21212478	21200482	21200658	21211242	21212478	1.5E-05	-0.12
209	FBgn0261261	chr3R	+	5685854	5687369	5685854	5686270	5686788	5687369	0.01219	0.317
210	FBgn0025469	chr2R	-	8082014	8083075	8082014	8082293	8082858	8083075	0.00652	-0.5
211	FBgn0032836	chr2L	+	19964135	19964365	19964135	19964281	19964338	19964365	0	0.261
212	FBgn0032836	chr2L	+	19964338	19964581	19964338	19964365	19964425	19964581	0	0.251
213	FBgn0032836	chr2L	+	19964425	19964775	19964425	19964581	19964643	19964775	0	0.205
214	FBgn0033339	chr2R	+	8893612	8894141	8893612	8893737	8893925	8894141	0	-0.321
215	FBgn0001991	chr2L	+	16173318	16173823	16173318	16173544	16173717	16173823	0	0.199
216	FBgn0032079	chr2L	-	8953480	8955807	8953480	8955586	8955681	8955807	0.00982	0.537
217	FBgn0004698	chr2R	+	15312032	15316614	15312032	15312817	15314617	15316614	0.03589	0.166
218	FBgn0039970	chr2R	+	5609012	5610256	5609012	5609489	5610063	5610256	3E-06	0.212
219	FBgn0031628	chr2L	+	4795842	4796219	4795842	4795975	4796092	4796219	0	0.786
220	FBgn0283657	chrX	+	3772639	3783559	3772639	3773034	3781654	3783559	0.02075	-0.674
221	FBgn0058178	chr3L	-	25977966	26008177	25977966	25978150	26008060	26008177	0.01369	0.29
222	FBgn0058178	chr3L	-	26010106	26013721	26010106	26010354	26013542	26013721	0.03677	0.331
223	FBgn0001122	chr2R	+	10438470	10439631	10438470	10438492	10439374	10439631	1.2E-05	0.806
224	FBgn0001122	chr2R	+	10438605	10439631	10438605	10438737	10439374	10439631	7.7E-09	-0.775
225	FBgn0050296	chr2R	+	21017783	21019582	21017783	21017952	21019158	21019582	0.02375	-0.137
226	FBgn0266633	chr2R	+	12868934	12869783	12868934	12869007	12869223	12869783	0.00595	-0.206
227	FBgn0027079	chr2R	+	13200888	13202027	13200888	13200906	13200997	13202027	0.02494	0.18
228	FBgn0036039	chr3L	+	9728503	9729650	9728503	9728614	9728712	9729650	0	0.923
229	FBgn0028956	chr2R	-	17450318	17451032	17450318	17450469	17450528	17451032	1.3E-06	1

230	FBgn0010905	chr3L	-	2513695	2514330	2513695	2514197	2514309	2514330	0.00423	-0.164
231	FBgn0010905	chr3L	-	2534710	2536585	2534710	2536218	2536515	2536585	0.00083	-0.276
232	FBgn0010909	chr3L	-	2585604	2586254	2585604	2585732	2586036	2586254	0.04466	-0.317
233	FBgn0020278	chr3R	-	22622123	22638947	22622123	22622906	22636800	22638947	0.00106	0.423
234	FBgn0030699	chrX	+	15844636	15845468	15844636	15844672	15845326	15845468	0.02109	-0.288
235	FBgn0263077	chr2R	+	7628796	7630328	7628796	7629112	7630176	7630328	0	0.514
236	FBgn0011817	chr3L	+	7979045	7979680	7979045	7979173	7979509	7979680	0.0006	-0.218
237	FBgn0011817	chr3L	+	8045890	8047161	8045890	8045946	8046569	8047161	0	0.417
238	FBgn0033307	chr2R	+	8590496	8591084	8590496	8590590	8591022	8591084	4.8E-05	0.971
239	FBgn0031717	chr2L	+	5549016	5549218	5549016	5549076	5549135	5549218	5.7E-06	-0.496
240	FBgn0034025	chr2R	+	15511709	15512765	15511709	15511731	15511813	15512765	0.02873	0.437
241	FBgn0039465	chr3R	-	26963157	26964285	26963157	26963520	26964117	26964285	0	0.566
242	FBgn0015222	chr3R	-	30387463	30387860	30387463	30387550	30387774	30387860	4.6E-09	-0.212
243	FBgn0013307	chr2R	+	8005628	8006232	8005628	8005926	8005983	8006232	0.00016	0.16
244	FBgn0013342	chr3L	-	1636817	1637612	1636817	1637269	1637558	1637612	0.02472	0.22
245	FBgn0033639	chr2R	-	11650814	11651616	11650814	11651224	11651323	11651616	5E-05	0.168
246	FBgn0034858	chr2R	+	23386520	23386827	23386520	23386622	23386789	23386827	0.01067	0.354
247	FBgn0034858	chr2R	+	23386520	23387612	23386520	23386622	23387006	23387612	0.00012	-0.312
248	FBgn0033717	chr2R	-	12341487	12341930	12341487	12341782	12341844	12341930	0.01061	-0.586
249	FBgn0015037	chr2R	+	9240603	9240842	9240603	9240685	9240741	9240842	0	-0.97
250	FBgn0037655	chr3R	-	9012044	9013261	9012044	9012305	9012836	9013261	0	0.417
251	FBgn0037655	chr3R	-	9013315	9014909	9013315	9013492	9014867	9014909	0.00636	-0.306
252	FBgn0034046	chr2R	+	15848509	15852541	15848509	15849679	15852387	15852541	0	0.273
253	FBgn0053547	chr3R	-	17886148	17887543	17886148	17886541	17887420	17887543	0.01861	-0.175
254	FBgn0053547	chr3R	-	17887420	17889780	17887420	17887543	17889594	17889780	5.8E-05	-0.309
255	FBgn0033159	chr2R	-	7349392	7361867	7349392	7349513	7361717	7361867	0.0003	0.889
256	FBgn0033159	chr2R	-	7353815	7361867	7353815	7353936	7361717	7361867	2.1E-07	1
257	FBgn0033159	chr2R	-	7357182	7361867	7357182	7357306	7361717	7361867	0.03368	0.467
258	FBgn0051683	chr2L	-	20448350	20449313	20448350	20448873	20448941	20449313	0.00566	-0.202
259	FBgn0040212	chr3R	-	25488523	25490466	25488523	25489103	25490201	25490466	2.7E-08	0.498
260	FBgn0262509	chr3L	+	23037637	23038277	23037637	23037753	23038011	23038277	0.0467	-0.21
261	FBgn0015521	chr2L	+	2856110	2856305	2856110	2856175	2856222	2856305	0.01869	-0.621
262	FBgn0015521	chr2L	+	2856376	2856672	2856376	2856568	2856630	2856672	0	0.845
263	FBgn0032726	chr2L	-	18953403	18954431	18953403	18953790	18954271	18954431	6.5E-05	0.408
264	FBgn0050054	chr2R	+	12624983	12625352	12624983	12625091	12625269	12625352	0.03098	0.524
265	FBgn0034631	chr2R	-	21498902	21501732	21498902	21501547	21501624	21501732	1.3E-09	-0.462
266	FBgn0015925	chr2R	+	16264651	16265626	16264651	16265122	16265166	16265626	0.04912	-0.218
267	FBgn0013973	chr3R	+	31158237	31171632	31158237	31158395	31171008	31171632	0.01331	0.158
268	FBgn0032363	chr2L	-	11516508	11516801	11516508	11516657	11516722	11516801	0.00302	0.147
269	FBgn0038140	chr3R	+	13338329	13340647	13338329	13338567	13339958	13340647	0.04478	-0.494
270	FBgn0020272	chrX	+	21389147	21390983	21389147	21389877	21389936	21390983	0.00423	0.279
271	FBgn0031730	chr2L	+	5711902	5712424	5711902	5712149	5712237	5712424	0.04759	0.469
272	FBgn0036834	chr3L	+	18950628	18953886	18950628	18950714	18953597	18953886	1.5E-05	0.953
273	FBgn0003943	chr3L	-	3899258	3902050	3899258	3901770	3901852	3902050	9E-08	-0.854
274	FBgn0020762	chr2L	+	4333912	4336957	4333912	4334104	4336449	4336957	4E-13	-0.29
275	FBgn0020762	chr2L	+	4333912	4336957	4333912	4334439	4336449	4336957	0.00124	0.17
276	FBgn0264469	chr3L	+	21528545	21529115	21528545	21528729	21528785	21529115	0.00294	0.607
277	FBgn0053108	chr3R	+	23996031	23997208	23996031	23996279	23997111	23997208	7.1E-08	0.737
278	FBgn0053108	chr3R	+	23997111	23997544	23997111	23997208	23997266	23997544	3.2E-07	0.856
279	FBgn0038476	chr3R	+	17081945	17084781	17081945	17082027	17082663	17084781	2.8E-05	0.64
280	FBgn0023096	chr2L	-	17967314	17969172	17967314	17968644	17968716	17969172	2E-10	0.952
281	FBgn0026076	chrX	-	15851584	15853175	15851584	15852739	15853041	15853175	4.8E-05	0.403
282	FBgn0023515	chrX	-	1864169	1865835	1864169	1865622	1865688	1865835	0.02258	0.605
283	FBgn0020370	chr2R	-	13156767	13157436	13156767	13156830	13156886	13157436	1.4E-11	0.639
284	FBgn0023550	chrX	-	2203132	2203866	2203132	2203221	2203288	2203866	0.03368	-0.303
285	FBgn0050197	chr2R	+	14514029	14514215	14514029	14514084	14514152	14514215	1.7E-06	0.089
286	FBgn0033724	chr2R	+	12383044	12384520	12383044	12383204	12383603	12384520	5E-06	0.748
287	FBgn0046687	chrX	-	5673829	5674709	5673829	5674173	5674221	5674709	1.2E-05	0.745
288	FBgn0039406	chr3R	-	26058979	26059267	26058979	26059051	26059234	26059267	0.00028	0.569
289	FBgn0036837	chr3L	-	18990025	18993993	18990025	18992725	18993725	18993993	0.02974	0.106
290	FBgn0039955	chr3R	+	3846728	3847059	3846728	3846911	3846980	3847059	0.03953	-0.565
291	FBgn0032940	chr2L	-	21308287	21310050	21308287	21308995	21310000	21310050	1.8E-06	0.4
292	FBgn0051460	chr3R	+	8562672	8563445	8562672	8562886	8562942	8563445	0.03677	0.288
293	FBgn0085285	chr3L	-	18989466	18989815	18989466	18989533	18989602	18989815	0.04408	-0.202
294	FBgn0250816	chr3L	+	23649123	23689640	23649123	23649372	23689484	23689640	0.00138	0.59
295	FBgn0033170	chr2R	+	7443042	7443827	7443042	7443382	7443442	7443827	4E-06	0.782
296	FBgn0263780	chr2R	-	2118976	2246006	2118976	2119244	2245971	2246006	0.00156	0.368
297	FBgn0033395	chr2R	+	9237511	9238037	9237511	9237756	9237836	9238037	1.6E-05	-0.548
298	FBgn0027109	chr3R	+	16609219	16611054	16609219	16609239	16610774	16611054	0.03368	-0.399
299	FBgn0039714	chr3R	-	29865056	29867817	29865056	29865399	29867743	29867817	2.7E-06	-0.63
300	FBgn0086904	chr2R	-	12759692	12760792	12759692	12760521	12760726	12760792	0.00078	0.32
301	FBgn0086904	chr2R	-	12759692	12760836	12759692	12760521	12760800	12760836	0.03273	0.507
302	FBgn0086904	chr2R	-	12759692	12760999	12759692	12760521	12760804	12760999	0.00447	0.186
303	FBgn0036493	chr3L	+	15305460	15306798	15305460	15305679	15306512	15306798	3.6E-06	0.851
304	FBgn0033609	chr2R	-	11287644	11289772	11287644	11288560	11289392	11289772	5.4E-05	0.743
305	FBgn0034634	chr2R	-	21518456	21519106	21518456	21518870	21518925	21519106	0.03225	0.312
306	FBgn0033725	chr2R	+	12388517	12389086	12388517	12388533	12388922	12389086	1.3E-10	-0.231
307	FBgn0031629	chr2L	-	4799745	4801923	4799745	4799878	4801443	4801923	0.00039	-0.597
308	FBgn0259236	chr3L	-	15631897	15634490	15631897	15632804	15634181	15634490	2.9E-07	0.713
309	FBgn0037084	chr2R	+	11219933	11221629	11219933	11220621	11220927	11221629	0	0.693
310	FBgn0034158	chr2R	+	16784769	16788260	16784769	16784908	16788154	16788260	0.03629	0.567
311	FBgn0042173	chr2R	+	7829591	7830429	7829591	7829981	7830043	7830429	0.00508	0.688
312	FBgn0033853	chr2R	-	13538197	13539349	13538197	13538855	13538971	13539349	0.00206	-0.085
313	FBgn0033853	chr2R	-	13538971	13542563	13538971	13539349	13542160	13542563	6.9E-06	-0.11
314	FBgn0033853	chr2R	-	13542160	13545449	13542160	13542563	13545307	13545449	0.00015	-0.258
315	FBgn0033853	chr2R	-	13538971	13547012	13538971	13539349	13546514	13547012	0.00045	-0.235
316	FBgn0033853	chr2R	-	13538971	13547577	13538971	13539349	13547449	13547577	0.0315	-0.479
317	FBgn0264815	chr2L	-	11818599	11832787	11818599	11818849	11832724	11832787	0.00428	-0.223
318	FBgn0264815	chr2L	-	11818599	11833373	11818599	11818849	11833259	11833373	4.6E-08	-0.329
319	FBgn0031734	chr2L	-	5736820	5740954	5736820	5736938	5740660	5740954	5.4E-06	0.179
320	FBgn0033942	chr2R	-	14512558	14515211	14512558	14513066	14515068	14515211	0	-0.5
321	FBgn0033968	chr2R	+	14808418	14808643	14808418	14808478	14808565	14808643	0.02326	0.108
322	FBgn0011642	chr4	-	1059418	1064381	1059418	1059836	1064244	1064381	0.00015	0.303
323	FBgn0034010	chr2R	-	15325502	15326386	15325502	15326290	15326350	15326386	0.03009	-0.312
324	FBgn0036481	chr3L	-	15122623	15124324	15122623	15123061	15124124	15124324	0.00181	0.677
325	FBgn0036481	chr3L	-	15124124	15125321	15124124	15124324	15125083	15125321	6E-09	0.595
326	FBgn0003392	chrX	+	15896365	15896763	15896365	15896472	15896706	15896763	0.03424	0.448
327	FBgn0041605	chr3R	+	4281837	4282834	4281837	4282003	4282673	4282834	1.9E-06	0.23
328	FBgn0013308	chr2R	+	8007787	8008295	8007787	8007945	8007997	8008295	0.04832	-0.485
329	FBgn0034965	chr2R	-	24053197	24053461	24053197	24053319	24053373	24053461	8.6E-08	0.915
330	FBgn0053460	chr2R	+	15694374	15694628	15694374	15694417	15694476	15694628	2.7E-10	-1
331	FBgn0034408	chr2R	+	18947316	18956126	18947316	18947586	18954977	18956126	0.00013	-0.359
332	FBgn0034420	chr2R	+	19173703	19175248	19173703	19174121	19174881	19175248	0.01439	-0.481
333	FBgn0034435	chr2R	+	19343012	19343430	19343012	19343191	19343253	19343430	1.5E-07	-1
334	FBgn0034517	chr2R	-	20548367	20549125	20548367	20548960	20549026	20549125	0.00148	0.685
335	FBgn0034075	chr2R	+	16114034	16114866	16114034	16114472	16114771	16114866	0.00023	-0.122
336	FBgn0034565	chr2R	-	20977594	20978304	20977594	20978126	20978198	20978304	4.9E-12	-0.928
337	FBgn0034605	chr2R	-	21217015	21217434	21217015	21217174	21217236	21217434	2.4E-11	-0.436
338	FBgn0034636	chr2R	-	21570761	21571808	21570761	21570824	21571416	21571808	0.00514	0.321
339	FBgn0034693	chr2R	-	22091488	22092273	22091488	22092062	22092141	22092273	1.2E-10	0.892
340	FBgn0034731	chr2R	+	22471027	22471655	22471027	22471184	22471277	22471655	0.00119	-0.152
341	FBgn0030997	chrX	-	19199826	19201780	19199826	19199885	19201463	19201780	9.9E-05	-0.778
342	FBgn0038221	chr3R	+	14302654	14303394	14302654	14302772	14302831	14303394	0.02927	0.399
343	FBgn0031690	chr2L	-	5212714	5213332	5212714	5212796	5212862	5213332	4.8E-05	-0.573
344	FBgn0031690	chr2L	-	5213836	5214730	5213836	5214023	5214316	5214730	0.04632	0.53
345	FBgn0034897	chr2R	+	23713899	23714899	23713899	23714074	23714665	23714899	1.2E-10	0.285
346	FBgn0034903	chr2R	-	23753713	23757166	23753713	23754337	23756363	23757166	0	-0.398
347	FBgn0054008	chr3R	-	20580682	20581374	20580682	20581044	20581112	20581374	0.00102	-0.562
348	FBgn0035040	chr2R	-	24610800	24613971	24610800	24611282	24613913	24613971	0.04042	-0.528
349	FBgn0266917	chr3R	-	17001863	17002355	17001863	17002028	17002150	17002355	1.1E-13	0.722
350	FBgn0030693	chrX	-	15794266	15795101	15794266	15794433	15794496	15795101	0.00042	-0.139
351	FBgn0030693	chrX	-	15794496	15796028	15794496	15795101	15795809	15796028	0.0026	0.237
352	FBgn0036058	chr3L	-	9896453	9896746	9896453	9896635	9896707	9896746	0.00598	-0.464
353	FBgn0036091	chr3L	+	10639739	10640136	10639739	10639856	10639919	10640136	0.04641	0.489
354	FBgn0040859	chrX	+	12091263	12091742	12091263	12091518	12091569	12091742	0.00566	-0.717
355	FBgn0031264	chr2L	-	560567	563819	560567	563189	563258	563819	5.9E-05	0.504
356	FBgn0032819	chr2L	+	19759001	19759504	19759001	19759152	19759203	19759504	0.01866	-0.21
357	FBgn0036290	chr3L	-	12500630	12501042	12500630	12500800	12500874	12501042	0.00656	0.5
358	FBgn0036661	chr3L	+	16780099	16780790	16780099	16780420	16780504	16780790	0.00108	0.193
359	FBgn0041150	chr2L	-	4925828	4928393	4925828	4926346	4928193	4928393	0.03629	-0.279
360	FBgn0041150	chr2L	-	4925828	4928393	4925828	4926346	4928227	4928393	0.01272	-0.348
361	FBgn0041150	chr2L	-	4925828	4928393	4925828	4926346	4928243	4928393	0.01807	-0.418
362	FBgn0041150	chr2L	-	4925828	4928393	4925828	4926350	4928243	4928393	3E-12	-0.54
363	FBgn0036566	chr3L	+	16095105	16096151	16095105	16096003	16096056	16096151	2.3E-08	-0.51
364	FBgn0036663	chr3L	-	16799483	16800189	16799483	16799954	16800092	16800189	0.01756	-0.683
365	FBgn0039290	chr3R	+	25209116	25210640	25209116	25209185	25210298	25210640	2.4E-05	0.599
366	FBgn0265356	chr2R	+	19004604	19006571	19004604	19004883	19005788	19006571	0.00162	-0.107
367	FBgn0004893	chr2L	+	3771699	3772694	3771699	3771784	3772460	3772694	0.00091	0.724
368	FBgn0037027	chr3L	+	20810980	20812197	20810980	20811450	20811529	20812197	0.02642	-0.35
369	FBgn0037057	chr3L	+	21202919	21206626	21202919	21203322	21205676	21206626	0.0373	-0.173
370	FBgn0015778	chr3R	+	13647003	13650497	13647003	13647200	13647382	13650497	0.02284	0.161
371	FBgn0037143	chr3L	+	21950455	21950801	21950455	21950684	21950736	21950801	8.6E-05	0.858
372	FBgn0264953	chr2L	+	8182626	8183563	8182626	8182878	8183188	8183563	0.00138	-0.456
373	FBgn0010548	chr2R	-	7473721	7474659	7473721	7473843	7474571	7474659	0.00098	-0.21
374	FBgn0010548	chr2R	-	7475341	7475559	7475341	7475377	7475439	7475559	0.04479	0.271
375	FBgn0037215	chr3R	+	4197205	4197562	4197205	4197301	4197414	4197562	0.01922	0.197
376	FBgn0040743	chr2R	+	16792641	16793179	16792641	16792781	16792872	16793179	2.2E-11	0.955
377	FBgn0038488	chr3R	-	17146720	17147737	17146720	17147351	17147414	17147737	0.00546	0.112
378	FBgn0085478	chrX	-	9337697	9338225	9337697	9337892	9338052	9338225	2.2E-13	0.195
379	FBgn0037612	chr3R	+	8712932	8714104	8712932	8713770	8713831	8714104	1.9E-05	0.49
380	FBgn0037975	chr3R	-	11983097	11984024	11983097	11983283	11983485	11984024	0.00924	0.134
381	FBgn0051038	chr3R	+	29877058	29879810	29877058	29877115	29877926	29879810	4.7E-11	0.403
382	FBgn0014906	chr2L	+	3163644	3166651	3163644	3164074	3166527	3166651	0.03393	0.464
383	FBgn0038311	chr3R	-	15266683	15268538	15266683	15268342	15268470	15268538	0.03878	0.659
384	FBgn0262562	chr3R	-	17783934	17794490	17783934	17784095	17794182	17794490	0.04906	0.8
385	FBgn0034990	chr2R	-	24205810	24206802	24205810	24205969	24206229	24206802	0.0332	0.567
386	FBgn0038598	chr3R	-	18287081	18288777	18287081	18287544	18287601	18288777	0.01922	0.42
387	FBgn0040717	chr2L	+	2008455	2008966	2008455	2008541	2008672	2008966	3.2E-13	0.744
388	FBgn0283471	chrX	-	18114443	18114646	18114443	18114532	18114605	18114646	0.0034	-0.801
389	FBgn0260440	chr3R	+	30476683	30478849	30476683	30478174	30478253	30478849	0.04272	-0.471
390	FBgn0265044	chr2R	-	6216685	6216894	6216685	6216737	6216824	6216894	1.5E-08	-1
391	FBgn0038873	chr3R	+	21242617	21243348	21242617	21242926	21242984	21243348	4.3E-05	-0.566
392	FBgn0039790	chr3R	-	30595389	30600323	30595389	30595626	30599918	30600323	0	-0.824
393	FBgn0063649	chr3R	+	16158160	16159204	16158160	16158306	16158857	16159204	0.00908	-0.247
394	FBgn0050456	chr2R	+	17100240	17103687	17100240	17100388	17102511	17103687	0.00637	-0.299
395	FBgn0034484	chr2R	+	20233939	20234927	20233939	20234374	20234559	20234927	0	-0.384
396	FBgn0033368	chr2R	-	9050842	9059548	9050842	9051151	9059254	9059548	0.00119	0.375
397	FBgn0040964	chr2L	+	8997604	8998544	8997604	8997668	8997761	8998544	1.1E-05	0.567
398	FBgn0038092	chr3R	+	12916055	12918769	12916055	12916143	12918443	12918769	0.00087	-0.358
399	FBgn0263199	chr3L	-	8985418	8986091	8985418	8985667	8985976	8986091	0.00039	0.639
400	FBgn0043841	chr2L	-	12421640	12423439	12421640	12421829	12423322	12423439	0.01245	0.203
401	FBgn0285926	chrX	-	10798618	10799924	10798618	10798993	10799811	10799924	0.00047	-0.629
402	FBgn0284252	chr2R	-	24514535	24514997	24514535	24514870	24514991	24514997	0.00044	0.446
403	FBgn0267376	chr3R	-	10866808	10868290	10866808	10866994	10867971	10868290	3.2E-06	-0.75

404	FBgn0267252	chr2L	+	9256562	9257206	9256562	9256814	9256895	9257206	0	0.484
405	FBgn0285948	chr2L	-	4457739	4458317	4457739	4458057	4458120	4458317	0.04829	0.336
406	FBgn0263930	chr3L	+	8887096	8888134	8887096	8887286	8887967	8888134	0.04528	-0.5
407	FBgn0264855	chr2L	-	413395	414777	413395	413654	414464	414777	0	0.933
408	FBgn0263593	chr2R	-	8142642	8145981	8142642	8143074	8145903	8145981	0	-0.989
409	FBgn0262975	chr3R	-	23222796	23226711	23222796	23223120	23226276	23226711	0.00569	-0.733
410	FBgn0261984	chr3R	-	19857240	19860761	19857240	19857684	19860490	19860761	8E-05	-0.8
411	FBgn0261552	chr3R	+	9419806	9432839	9419806	9420047	9432510	9432839	0.01075	-0.667
412	FBgn0261268	chr2L	-	15270904	15272024	15270904	15271143	15271900	15272024	0.00385	0.513
413	FBgn0261239	chr2L	+	21255896	21256362	21255896	21256046	21256260	21256362	0.01507	-0.332
414	FBgn0250910	chr3R	-	12519011	12519441	12519011	12519072	12519221	12519441	8.8E-07	-0.671
415	FBgn0086711	chr2L	-	14977214	14984084	14977214	14977646	14983875	14984084	0.01814	-0.512
416	FBgn0263397	chr2R	+	14276129	14279642	14276129	14276526	14279242	14279642	0.00017	0.341
417	FBgn0267347	chr2L	+	16680053	16680650	16680053	16680276	16680485	16680650	0.04823	-0.54
418	FBgn0085408	chr2R	+	14308141	14315533	14308141	14308293	14315103	14315533	0.02683	0.5
419	FBgn0085399	chr2R	-	22037391	22039559	22037391	22037716	22039477	22039559	0	-0.854
420	FBgn0085399	chr2R	-	22037391	22039559	22037391	22037716	22039481	22039559	0.03347	-0.677
421	FBgn0083124	chr2R	+	9096925	9097183	9096925	9096991	9097061	9097183	0	-0.645
422	FBgn0082582	chr3R	+	30546405	30548749	30546405	30546587	30548278	30548749	0	-0.246
423	FBgn0082582	chr3R	+	30546405	30548749	30546405	30546587	30548303	30548749	1E-11	-0.206
424	FBgn0064225	chr2L	-	22536114	22536884	22536114	22536403	22536536	22536884	9.6E-07	-0.646
425	FBgn0052944	chr3R	-	4415682	4419578	4415682	4415886	4419359	4419578	0.00015	0.449
426	FBgn0052549	chrX	-	18409049	18410144	18409049	18409079	18409883	18410144	0.02811	-0.508
427	FBgn0261383	chrX	-	5744355	5747254	5744355	5744699	5747083	5747254	0.02645	-0.484
428	FBgn0052264	chr3L	-	3758406	3765731	3758406	3759258	3765604	3765731	0.00026	0.515
429	FBgn0052112	chr3L	+	12742904	12743277	12742904	12743172	12743237	12743277	0.02284	-0.399
430	FBgn0051678	chr2L	-	20664290	20668096	20664290	20664449	20667772	20668096	0.03493	-0.146
431	FBgn0051450	chr3R	-	8561916	8562593	8561916	8562315	8562454	8562593	0.01271	0.44
432	FBgn0053182	chr2R	-	13187393	13190086	13187393	13187908	13189696	13190086	0.00382	0.487
433	FBgn0051100	chr3R	-	9000286	9001886	9000286	9000544	9001642	9001886	0.00037	0.26
434	FBgn0278608	chrX	+	16334300	16336743	16334300	16334413	16336347	16336743	0.00162	0.79
435	FBgn0050295	chr2R	+	20876474	20877312	20876474	20876621	20876869	20877312	0.00514	-0.714
436	FBgn0260388	chr3L	-	16493145	16495268	16493145	16493940	16495051	16495268	0.04188	0.213
437	FBgn0000055	chr2L	+	14615551	14615876	14615551	14615643	14615778	14615876	1.8E-05	-0.266
438	FBgn0000055	chr2L	+	14615551	14616437	14615551	14615643	14616057	14616437	0.01692	-0.261
439	FBgn0000055	chr2L	+	14615551	14616437	14615551	14615643	14616215	14616437	3.2E-10	0.343
440	FBgn0031866	chr2L	+	6903680	6904073	6903680	6903869	6903932	6904073	1.4E-08	0.715
441	FBgn0035388	chr3L	+	3038342	3038905	3038342	3038567	3038703	3038905	0.02025	-0.614
442	FBgn0031636	chr2L	+	4840883	4841465	4840883	4840983	4841162	4841465	0	0.386
443	FBgn0004167	chr3L	+	3336859	3337828	3336859	3337001	3337682	3337828	0.00138	0.527
444	FBgn0038460	chr3R	-	16984570	16986245	16984570	16984617	16986118	16986245	0.01678	0.394
445	FBgn0262149	chr3R	+	7128463	7129067	7128463	7128647	7128940	7129067	0.0042	0.399
446	FBgn0051674	chr2L	+	20831261	20832287	20831261	20831399	20831466	20832287	0.03471	-0.304
447	FBgn0266410	chr3R	+	9354421	9355083	9354421	9354854	9354935	9355083	0.02537	-0.428
448	FBgn0058191	chr2R	+	1265170	1266349	1265170	1265531	1266191	1266349	1.1E-13	0.605
449	FBgn0030306	chrX	+	11485249	11486566	11485249	11485496	11485560	11486566	0.0373	0.667
450	FBgn0021800	chr2L	+	3806968	3807461	3806968	3807056	3807115	3807461	2.7E-12	0.861
451	FBgn0000239	chr2L	-	21177570	21179683	21177570	21177959	21179329	21179683	0.00024	0.464
452	FBgn0028671	chr3R	+	29131192	29131825	29131192	29131271	29131665	29131825	6.9E-05	-0.9
453	FBgn0005614	chr2R	+	9756037	9760014	9756037	9756183	9759786	9760014	0.03393	-0.615
454	FBgn0041789	chr2L	-	19419868	19423552	19419868	19420080	19423278	19423552	0.00307	-0.646
455	FBgn0034282	chr2R	-	17868431	17871495	17868431	17868808	17871337	17871495	0.00126	-0.7
456	FBgn0033889	chr2R	-	13979183	13980165	13979183	13979390	13980027	13980165	2.8E-10	0.753
457	FBgn0027504	chr2R	-	12149589	12151057	12149589	12150139	12150950	12151057	0.00209	-0.47
458	FBgn0010263	chr2L	+	2961738	2962286	2961738	2961996	2962256	2962286	0.00078	-0.517
459	FBgn0030706	chrX	+	15914605	15916689	15914605	15916071	15916475	15916689	0.00129	0.767
460	FBgn0026562	chr3R	-	26870192	26871995	26870192	26870396	26871925	26871995	0	-0.791
461	FBgn0026255	chr2L	+	21208939	21210056	21208939	21209371	21209666	21210056	0.0008	0.543
462	FBgn0003174	chr2R	+	7282571	7286028	7282571	7282626	7283563	7286028	6.9E-05	-0.9
463	FBgn0020620	chr2R	-	13989684	13990400	13989684	13989986	13990208	13990400	2.3E-09	0.762
464	FBgn0020620	chr2R	-	13989684	13990400	13989684	13989986	13990257	13990400	0.04557	0.546
465	FBgn0051326	chr3R	-	14219628	14220205	14219628	14219842	14219984	14220205	0.03677	-0.426
466	FBgn0051708	chr2L	-	9066079	9071164	9066079	9066760	9070899	9071164	0.00186	-0.567
467	FBgn0005626	chr3L	-	6717903	6719525	6717903	6718057	6719004	6719525	0.00138	-0.569
468	FBgn0030521	chrX	-	13781472	13782170	13781472	13781733	13781839	13782170	0.0191	0.224
469	FBgn0036666	chr3L	+	16843102	16843730	16843102	16843378	16843492	16843730	0	-0.615
470	FBgn0085468	chr2R	+	13159016	13159649	13159016	13159103	13159511	13159649	0.00302	-0.641
471	FBgn0005649	chr3R	-	24045437	24047012	24045437	24046491	24046941	24047012	0.01445	0.268
472	FBgn0013305	chr2R	-	12879051	12879373	12879051	12879146	12879286	12879373	0.00511	-0.469
473	FBgn0013305	chr2R	-	12879196	12879656	12879196	12879222	12879573	12879656	0.00285	0.68
474	FBgn0003371	chrX	+	2633951	2634871	2633951	2634178	2634604	2634871	2.4E-07	-0.9
475	FBgn0028496	chr2R	-	18496746	18498026	18496746	18496868	18497885	18498026	0.01052	0.304
476	FBgn0028496	chr2R	-	18496746	18498026	18496746	18496905	18497885	18498026	0.00318	-0.617
477	FBgn0003415	chr3L	-	21024951	21026204	21024951	21025548	21026039	21026204	0.00851	-0.575
478	FBgn0033994	chr2R	+	15148883	15149709	15148883	15149096	15149163	15149709	1.1E-06	0.643
479	FBgn0000119	chr2R	-	13481775	13483229	13481775	13482214	13483154	13483229	2.1E-11	0.86
480	FBgn0002938	chr2L	+	7377735	7378688	7377735	7377865	7378402	7378688	0.01937	-0.365
481	FBgn0024320	chr2L	-	10218778	10219260	10218778	10218889	10219026	10219260	1.9E-06	0.845
482	FBgn0000370	chr2L	+	21310865	21312043	21310865	21311017	21311192	21312043	3.3E-09	0.481
483	FBgn0031857	chr2L	-	6770590	6772755	6770590	6770718	6772551	6772755	0	0.975
484	FBgn0028396	chr3R	+	20871035	20871700	20871035	20871103	20871185	20871700	7.1E-08	-0.447
485	FBgn0040778	chr2R	-	8066273	8067293	8066273	8066758	8067184	8067293	0	-0.927
486	FBgn0040778	chr2R	-	8067125	8067655	8067125	8067293	8067407	8067655	3.6E-12	-0.62
487	FBgn0015221	chr3R	+	30389198	30389442	30389198	30389328	30389436	30389442	2.8E-07	-0.29
488	FBgn0032172	chr2L	-	9962734	9963755	9962734	9963012	9963377	9963755	6.2E-06	0.405
489	FBgn0026374	chrX	+	15788896	15790388	15788896	15788957	15790233	15790388	0.0001	-0.884
490	FBgn0026374	chrX	+	15788896	15790388	15788896	15789112	15790233	15790388	0.00044	-0.7
491	FBgn0031220	chr2L	-	120167	120631	120167	120457	120510	120631	0.02203	0.209
492	FBgn0013767	chr3R	+	14314853	14315716	14314853	14315150	14315335	14315716	1.8E-09	-1
493	FBgn0000052	chr2L	-	6045770	6046050	6045770	6045852	6045920	6046050	0.00873	0.654
494	FBgn0032836	chr2L	+	19964839	19965702	19964839	19964998	19965062	19965702	0	0.407
495	FBgn0010350	chr3L	+	8129851	8130556	8129851	8130340	8130396	8130556	0.00017	0.421
496	FBgn0032197	chr2L	-	10229704	10231692	10229704	10229955	10231394	10231692	0.00169	-0.606
497	FBgn0003380	chrX	-	17976350	17990614	17976350	17976791	17990304	17990614	0.00208	-0.247
498	FBgn0037525	chr3R	-	7885853	7886733	7885853	7886278	7886442	7886733	0.00879	-0.258
499	FBgn0010905	chr3L	-	2534710	2536585	2534710	2536218	2536285	2536585	0.0003	-0.846
500	FBgn0016726	chr2R	+	21293849	21294159	21293849	21293874	21294050	21294159	3.3E-08	-0.606
501	FBgn0263077	chr2R	+	7636894	7638372	7636894	7637193	7638122	7638372	0.0055	-0.243
502	FBgn0030087	chrX	-	9094410	9095333	9094410	9095008	9095075	9095333	0.00505	-0.826
503	FBgn0029663	chrX	-	3480858	3482322	3480858	3481344	3481953	3482322	0.01061	-0.395
504	FBgn0030853	chrX	+	17632589	17633509	17632589	17632911	17633262	17633509	0.03559	0.195
505	FBgn0013307	chr2R	+	8005628	8006232	8005628	8005926	8005987	8006232	3.9E-07	0.862
506	FBgn0259246	chr2R	+	9503764	9505468	9503764	9503845	9505117	9505468	8.3E-06	0.669
507	FBgn0033639	chr2R	-	11644326	11644764	11644326	11644408	11644484	11644764	0.0191	0.571
508	FBgn0032900	chr2L	+	20869853	20870760	20869853	20870191	20870512	20870760	0.00068	-0.511
509	FBgn0036891	chr3L	+	19607245	19608368	19607245	19607825	19607976	19608368	0.0026	-0.422
510	FBgn0023509	chrX	+	1908238	1920318	1908238	1908584	1916976	1920318	0.01766	-0.339
511	FBgn0000308	chr2L	-	5979411	5980481	5979411	5979704	5980263	5980481	2.7E-06	-0.945
512	FBgn0040532	chr3R	+	8822007	8822666	8822007	8822125	8822405	8822666	0.01217	-0.191
513	FBgn0037024	chr3L	+	20798644	20800081	20798644	20799080	20799142	20800081	0.04823	-0.347
514	FBgn0262509	chr3L	+	23029836	23037753	23029836	23029931	23037615	23037753	7.2E-08	-0.834
515	FBgn0015521	chr2L	+	2856246	2856568	2856246	2856305	2856357	2856568	0.01346	0.551
516	FBgn0032726	chr2L	-	18953403	18954431	18953403	18953778	18954271	18954431	0.01725	-0.367
517	FBgn0026718	chr2L	-	8450793	8463994	8450793	8451004	8463641	8463994	0.03368	0.667
518	FBgn0026059	chr3R	-	16070543	16072054	16070543	16071502	16071573	16072054	0.00429	-0.665
519	FBgn0026059	chr3R	-	16097135	16098761	16097135	16097620	16098369	16098761	0.00387	-0.5
520	FBgn0035144	chr3L	-	585016	589274	585016	585506	588962	589274	0.00327	-0.359
521	FBgn0261794	chr2R	+	23909677	23911868	23909677	23909809	23911682	23911868	1.5E-06	-0.395
522	FBgn0029761	chrX	+	5378629	5383379	5378629	5378725	5383075	5383379	0.02075	-0.619
523	FBgn0020762	chr2L	+	4333912	4336957	4333912	4334439	4336500	4336957	3.9E-09	0.47
524	FBgn0031564	chr2L	+	3703658	3704253	3703658	3703812	3703882	3704253	0.0001	-0.51
525	FBgn0024555	chr3R	+	13683920	13687975	13683920	13684150	13687574	13687975	0.03368	-0.738
526	FBgn0024811	chr4	+	210581	211794	210581	210680	211627	211794	5.8E-05	-0.264
527	FBgn0025574	chr3R	-	23889056	23890052	23889056	23889325	23889693	23890052	0.00916	0.563
528	FBgn0037676	chr3R	-	9326687	9328511	9326687	9327312	9328405	9328511	0.01801	0.505
529	FBgn0032859	chr2L	+	20422144	20422411	20422144	20422236	20422298	20422411	0.00631	-0.793
530	FBgn0033031	chr2R	+	5668382	5668998	5668382	5668508	5668792	5668998	4E-13	-0.592
531	FBgn0033205	chr2R	+	7665827	7666409	7665827	7666016	7666114	7666409	0.00259	-0.552
532	FBgn0085385	chr3L	+	9944476	9946023	9944476	9945502	9945811	9946023	0.00036	0.667
533	FBgn0033320	chr2R	-	8694779	8695797	8694779	8695251	8695705	8695797	0.03153	0.415
534	FBgn0039714	chr3R	-	29865056	29867214	29865056	29865399	29867161	29867214	0.0033	-0.524
535	FBgn0039714	chr3R	-	29865056	29867817	29865056	29865399	29867752	29867817	0.00021	0.418
536	FBgn0033437	chr2R	-	9609095	9610290	9609095	9609442	9610045	9610290	9E-05	0.78
537	FBgn0036493	chr3L	+	15305576	15306798	15305576	15305679	15306504	15306798	5E-10	-0.944
538	FBgn0016059	chr2R	-	17675910	17677919	17675910	17676593	17677515	17677919	1.7E-10	0.703
539	FBgn0016059	chr2R	-	17678271	17679255	17678271	17678782	17679143	17679255	0.00198	0.6
540	FBgn0014396	chr2L	-	3501617	3502557	3501617	3502126	3502315	3502557	0.00073	-0.602
541	FBgn0022382	chr2R	-	10024050	10026042	10024050	10024249	10025849	10026042	0.00575	-0.293
542	FBgn0034165	chr2R	+	16873771	16874417	16873771	16873885	16874219	16874417	0.00116	-0.535
543	FBgn0033853	chr2R	-	13542160	13545449	13542160	13542547	13545307	13545449	1.8E-06	-0.388
544	FBgn0002921	chr3R	+	20966397	20973347	20966397	20966613	20973197	20973347	0	0.222
545	FBgn0033872	chr2R	+	13830550	13833503	13830550	13830722	13830777	13833503	0.00026	-0.675
546	FBgn0034051	chr2R	-	15939036	15940073	15939036	15939278	15939909	15940073	1.3E-09	-1
547	FBgn0038243	chr3R	-	14568080	14568847	14568080	14568599	14568659	14568847	0.01676	-0.447
548	FBgn0023508	chrX	+	1926445	1926918	1926445	1926632	1926705	1926918	0.00039	0.675
549	FBgn0033079	chr2R	-	6643002	6644383	6643002	6643541	6644040	6644383	0.02423	-0.232
550	FBgn0039229	chr3R	+	24818410	24818700	24818410	24818438	24818569	24818700	0.04557	-0.335
551	FBgn0034530	chr2R	-	20630683	20633506	20630683	20630836	20632789	20633506	3.6E-06	-0.537
552	FBgn0034538	chr2R	-	20653744	20655096	20653744	20653867	20654969	20655096	0.04937	0.254
553	FBgn0034542	chr2R	-	20664785	20667306	20664785	20665092	20666918	20667306	0.00058	-0.226
554	FBgn0038034	chr3R	-	12397333	12398929	12397333	12397853	12398673	12398929	0.01448	0.767
555	FBgn0036545	chr3L	+	15955387	15956465	15955387	15955549	15955610	15956465	0.00771	0.686
556	FBgn0034808	chr2R	-	22996376	22998884	22996376	22996512	22998432	22998884	0.00783	0.433
557	FBgn0034808	chr2R	-	22996376	22998884	22996376	22996512	22998829	22998884	1.3E-07	0.795
558	FBgn0036331	chr3L	+	13008284	13008873	13008284	13008567	13008654	13008873	9E-05	0.89
559	FBgn0034725	chr2R	+	22404176	22407325	22404176	22404460	22407017	22407325	4.8E-07	-0.744
560	FBgn0264607	chr4	-	1050454	1053703	1050454	1050862	1053497	1053703	0.00138	-0.451
561	FBgn0035719	chr3L	+	6942855	6944769	6942855	6943101	6944332	6944769	0.01067	0.297
562	FBgn0035719	chr3L	+	6942855	6944769	6942855	6943101	6944440	6944769	0.00809	0.376
563	FBgn0036369	chr3L	-	13468952	13469212	13468952	13469069	13469201	13469212	0.00326	-0.422
564	FBgn0263131	chr3L	-	16539145	16543370	16539145	16539340	16543176	16543370	0.00211	0.667
565	FBgn0259749	chr2L	-	6471327	6472072	6471327	6471743	6471960	6472072	0.02075	-0.392
566	FBgn0036663	chr3L	-	16799483	16800189	16799483	16799954	16800096	16800189	0.01338	-0.535
567	FBgn0036663	chr3L	-	16799483	16807731	16799483	16799954	16807633	16807731	0.0006	0.525
568	FBgn0011695	chr2R	+	24026375	24027518	24026375	24026461	24027003	24027518	5E-06	-0.141
569	FBgn0265356	chr2R	+	19005788	19007746	19005788	19006571	19007548	19007746	0.02305	-0.459
570	FBgn0037070	chr3L	-	21303909	21304467	21303909	21304091	21304147	21304467	0.03042	0.624
571	FBgn0037386	chr3R	+	5836856	5837341	5836856	5837042	5837112	5837341	0.0176	-0.454
572	FBgn0037646	chr3R	+	8980859	8981915	8980859	8981109	8981629	8981915	4.3E-05	0.766
573	FBgn0050345	chr2R	+	9149686	9150087	9149686	9149837	9149890	9150087	0.01075	0.456
574	FBgn0037911	chr3R	+	11557775	11558684	11557775	11558364	11558433	11558684	0.01917	0.456
575	FBgn0263395	chr2R	+	19178737	19179858	19178737	19178872	19179410	19179858	3E-06	0.454
576	FBgn0032587	chr2L	-	16527389	16532877	16527389	16528439	16532707	16532877	0.00094	0.647
577	FBgn0030300	chrX	+	11442538	11446012	11442538	11442840	11443566	11446012	0.0373	-0.584

578	FBgn0036454	chr3L	+	14839217	14864930	14839217	14839307	14864804	14864930	0.0038	-0.647
579	FBgn0039098	chr3R	+	23677357	23678164	23677357	23677637	23677800	23678164	0.00345	0.667
580	FBgn0029837	chrX	-	6071804	6072997	6071804	6071875	6072946	6072997	4.3E-05	0.833
581	FBgn0039713	chr3R	+	29862784	29863583	29862784	29863196	29863380	29863583	0.00021	-0.659
582	FBgn0039776	chr3R	+	30484119	30484752	30484119	30484602	30484689	30484752	6.7E-11	0.561
583	FBgn0039902	chr4	-	310929	313642	310929	313298	313360	313642	0.03095	0.576
584	FBgn0039927	chr4	+	1130382	1131396	1130382	1130586	1131171	1131396	0.00259	-0.552
585	FBgn0039936	chr4	-	861792	865642	861792	864411	865368	865642	5.4E-05	0.415
586	FBgn0040529	chr3R	-	8339943	8340771	8339943	8340089	8340512	8340771	0.01124	-0.48
587	FBgn0037146	chr3L	+	21977192	21977560	21977192	21977381	21977461	21977560	3.4E-05	-0.346
588	FBgn0042693	chr3R	+	18171528	18175529	18171528	18171703	18175334	18175529	0.00136	-0.767

SE											
	FlyBase ID	chr	strand	exonStart_0base	exonEnd	upstreamES	upstreamEE	downstreamES	downstreamEE	FDR	ΔPSI
1	FBgn0030749	chrX	-	16419203	16419770	16418667	16419049	16422166	16422242	0.00305	0.242
2	FBgn0052600	chrX	+	14324737	14324858	14322225	14322915	14355462	14355542	0.00423	0.312
3	FBgn0264006	chr3L	-	13876377	13876458	13873071	13873266	13877623	13877629	0.00213	-0.51
4	FBgn0263346	chr3R	+	4680228	4680579	4659578	4660399	4680778	4680837	0.02413	-0.278
5	FBgn0263346	chr3R	+	4680778	4680837	4670796	4670850	4697190	4697542	0	0.465
6	FBgn0035162	chr3L	-	830621	830728	830033	830341	830907	831242	0.04786	-0.278
7	FBgn0263197	chr2R	-	17521831	17521900	17520645	17520928	17523499	17523571	0.04898	0.163
8	FBgn0263197	chr2R	-	17521831	17521906	17520645	17520928	17523499	17523571	0.02492	0.226
9	FBgn0261984	chr3R	-	19860646	19860761	19857240	19857684	19860885	19861291	0.01555	0.102
10	FBgn0082582	chr3R	+	30576597	30576818	30575431	30575948	30577178	30577558	0.00128	0.154
11	FBgn0037698	chr3R	-	9478544	9478697	9475533	9475840	9486600	9487203	0.00239	-0.216
12	FBgn0028703	chr2L	-	6678911	6679082	6677834	6678224	6679985	6680114	0.00011	-0.236
13	FBgn0033494	chr2R	-	10149347	10149440	10148632	10149094	10150855	10151052	8.3E-05	0.441
14	FBgn0034718	chr2R	-	22308207	22308256	22298213	22301009	22311695	22311937	0.00012	0.145
15	FBgn0028490	chr2L	-	11525916	11526012	11524567	11525667	11528120	11528205	0	0.783
16	FBgn0027932	chr2L	+	8428319	8429462	8427614	8428067	8430280	8430544	0.04134	0.152
17	FBgn0261836	chr2L	+	5202764	5202803	5199342	5202302	5203115	5203742	0.00128	0.21
18	FBgn0032456	chr2L	+	12728020	12728241	12726745	12727116	12737958	12738176	0.00052	-0.275
19	FBgn0032456	chr2L	+	12736035	12736256	12726745	12727116	12737958	12738176	0.01122	-0.595
20	FBgn0050296	chr2R	+	21023661	21023754	21023433	21023586	21023975	21024125	6.9E-14	0.268
21	FBgn0267002	chr2R	-	16764137	16764374	16763197	16763340	16772253	16772500	0	0.333
22	FBgn0267002	chr2R	-	16764137	16764374	16763197	16763340	16772257	16772671	0	0.545
23	FBgn0033807	chr2R	-	13213750	13213931	13213022	13213436	13214418	13214496	0	0.778
24	FBgn0003386	chr2L	+	3725655	3725901	3724883	3725196	3726696	3729145	1.2E-06	0.384
25	FBgn0266696	chr3L	+	2442435	2442960	2439498	2439830	2443389	2443838	2E-05	-0.247
26	FBgn0266696	chr3L	+	2442438	2442960	2439498	2439830	2443389	2443838	2E-07	-0.268
27	FBgn0004838	chr2L	+	6922730	6922826	6920703	6920861	6923783	6924400	1.2E-05	-0.123
28	FBgn0033484	chr2R	+	10091756	10091996	10090542	10090799	10093143	10094143	0.025	0.145
29	FBgn0033501	chr2R	+	10260012	10260429	10259515	10259585	10260862	10261276	0.00232	-0.682
30	FBgn0034093	chr2R	-	16226567	16226804	16225106	16225325	16230077	16230402	0.00239	-0.479
31	FBgn0010482	chr2R	-	6737493	6737529	6734728	6734911	6737960	6738127	0.01588	0.13
32	FBgn0034605	chr2R	-	21217236	21217434	21217015	21217174	21217727	21217779	0.00029	0.331
33	FBgn0263077	chr2R	+	7630176	7630328	7628796	7629112	7634215	7634314	0	-0.494
34	FBgn0031717	chr2L	+	5549016	5549076	5548167	5548709	5549443	5549614	0	-0.438
35	FBgn0031717	chr2L	+	5549016	5549081	5548167	5548709	5549443	5549614	0	-0.376
36	FBgn0031717	chr2L	+	5549135	5549218	5549016	5549076	5549443	5549614	0.04972	0.18
37	FBgn0024189	chr2R	+	8833792	8833981	8832703	8832870	8834951	8835101	0.00583	0.204
38	FBgn0027835	chr2R	+	18414828	18414933	18413325	18413429	18415198	18415286	0.02413	-0.654
39	FBgn0261988	chr3R	+	31410353	31410401	31405244	31405956	31433458	31433888	0	0.661
40	FBgn0016930	chr2L	-	14229458	14229536	14222016	14222081	14233647	14234108	0	-0.371
41	FBgn0016930	chr2L	-	14229531	14229536	14222016	14222081	14233647	14234108	0	-0.404
42	FBgn0260794	chr3R	-	4810671	4810787	4801212	4801991	4811982	4812096	0.0335	0.412
43	FBgn0261262	chr3R	+	18968451	18968550	18958361	18959924	18974990	18975170	0.00477	0.535
44	FBgn0283712	chrX	-	12589255	12589321	12587969	12589167	12589840	12590484	0.02804	-0.241
45	FBgn0036272	chr3L	-	12275075	12275187	12274002	12274111	12275998	12276050	0.00242	-0.49
46	FBgn0005536	chr3L	+	16077503	16077809	16077191	16077282	16078819	16079589	0.02245	-0.291
47	FBgn0005536	chr3L	+	16077647	16077809	16077191	16077282	16078819	16079589	0.02245	-0.291
48	FBgn0005536	chr3L	+	16077703	16077809	16077191	16077282	16078819	16079589	0.02064	-0.279
49	FBgn0030749	chrX	-	16420049	16420085	16418667	16419049	16422166	16422242	0.00578	0.198
50	FBgn0001105	chrX	+	15861139	15861266	15860114	15860322	15861376	15861486	0.00049	0.86
51	FBgn0001105	chrX	+	15861139	15861304	15860114	15860322	15861376	15861486	0.03058	0.705
52	FBgn0001105	chrX	+	15861151	15861266	15860114	15860322	15861376	15861486	0.00438	0.716
53	FBgn0030412	chrX	+	12643872	12644767	12639153	12639296	12645903	12645938	0.00041	0.353
54	FBgn0030412	chrX	+	12647413	12647759	12639153	12639296	12651109	12651226	8E-08	-1
55	FBgn0035715	chr3L	-	6754946	6755123	6754744	6754807	6755419	6756158	0.02948	0.254
56	FBgn0267252	chr2L	+	9256756	9256814	9256562	9256696	9256892	9257206	0	-0.328
57	FBgn0266579	chr3R	-	27640406	27640617	27639885	27640296	27643814	27643973	0.02413	-0.446
58	FBgn0263593	chr2R	-	8145754	8146076	8142642	8143074	8156054	8156654	2.3E-11	-0.268
59	FBgn0263593	chr2R	-	8145903	8146076	8142642	8143074	8156054	8156654	0	0.327
60	FBgn0036211	chr3L	-	11817388	11817724	11816982	11817316	11818087	11818187	0.00057	-0.098
61	FBgn0263346	chr3R	+	4680708	4680837	4670796	4670850	4697190	4697542	0	0.748
62	FBgn0035162	chr3L	-	830621	830744	830033	830341	830907	831242	0.01583	-0.277
63	FBgn0262579	chr3L	+	8096970	8097251	8096521	8096876	8098345	8098725	0.00673	-0.452
64	FBgn0262579	chr3L	+	8096970	8097312	8096521	8096876	8098345	8098725	0.00673	-0.452
65	FBgn0261984	chr3R	-	19860490	19860761	19857240	19857684	19860885	19861291	8.9E-06	0.246
66	FBgn0082582	chr3R	+	30576543	30576818	30575431	30575948	30577178	30577558	0.00158	0.14
67	FBgn0082582	chr3R	+	30576556	30576818	30575431	30575948	30577178	30577558	0.00153	0.14
68	FBgn0082582	chr3R	+	30576583	30576818	30575431	30575948	30577178	30577558	0.00128	0.142
69	FBgn0082582	chr3R	+	30576624	30576818	30575431	30575948	30577178	30577558	0.00132	0.141
70	FBgn0025574	chr3R	-	23888826	23889325	23865562	23865655	23889693	23890052	0.00143	-0.718
71	FBgn0002566	chr2L	+	22938871	22939296	22927437	22927765	22939353	22939540	0.00124	0.45
72	FBgn0039790	chr3R	-	30599918	30600173	30595389	30595626	30600263	30600323	2.9E-08	0.6
73	FBgn0042693	chr3R	+	18172813	18172949	18171528	18171703	18173125	18174190	0.02919	-0.348
74	FBgn0033494	chr2R	-	10148636	10149094	10148081	10148152	10150855	10151052	0.0253	-0.115
75	FBgn0039897	chr4	+	232394	232848	231934	231977	236394	236475	0.04815	0.417
76	FBgn0039897	chr4	+	234863	234948	231934	231977	236394	236475	0.00673	0.447
77	FBgn0039897	chr4	+	242487	242637	240644	240833	243169	243585	0.02228	-0.326
78	FBgn0037955	chr3R	-	11821975	11822117	11821279	11821819	11822191	11822241	0.00107	0.421
79	FBgn0051363	chr3R	-	11603851	11604211	11593632	11593766	11604585	11604754	1.8E-06	1
80	FBgn0036975	chr3L	+	20354817	20355129	20353958	20354405	20355304	20356280	0.01811	0.32
81	FBgn0032946	chr2L	+	21380647	21380728	21380132	21380478	21382018	21382330	5.9E-05	0.135
82	FBgn0039667	chr3R	+	29495008	29495125	29494781	29494885	29495742	29495801	0.0279	0.667
83	FBgn0051678	chr2L	-	20664290	20664312	20663997	20664209	20674994	20675570	0.03776	0.201
84	FBgn0051678	chr2L	-	20664290	20664660	20663997	20664209	20674994	20675570	2.8E-07	0.279
85	FBgn0265959	chr3L	+	20260209	20260487	20255897	20256638	20260861	20260989	0.0086	0.169
86	FBgn0265959	chr3L	+	20260421	20260487	20255897	20256638	20260861	20260989	0.01304	0.144
87	FBgn0261625	chr2R	-	12673898	12673925	12673099	12673339	12674990	12675418	4.2E-05	-0.397
88	FBgn0261625	chr2R	-	12674205	12674702	12673099	12673339	12674990	12675418	5.6E-06	-0.462
89	FBgn0038720	chr3R	-	19620614	19620832	19619084	19619380	19623315	19623476	0.0473	-0.134
90	FBgn0038720	chr3R	-	19620614	19620853	19619084	19619380	19623315	19623476	0.03905	-0.136
91	FBgn0038720	chr3R	-	19620614	19620946	19619084	19619380	19623315	19623476	0.03367	-0.137
92	FBgn0038720	chr3R	-	19620614	19620984	19619084	19619380	19623315	19623476	0.0473	-0.134
93	FBgn0031449	chr2L	+	2736173	2736229	2730103	2730469	2736310	2736430	0.02763	-0.528
94	FBgn0032456	chr2L	+	12728070	12728241	12726745	12727116	12737958	12738176	0.0004	-0.402
95	FBgn0032456	chr2L	+	12729902	12730151	12726745	12727116	12737958	12738176	0.00399	-0.196
96	FBgn0032456	chr2L	+	12736031	12736256	12726745	12727116	12737958	12738176	0.0069	-0.653
97	FBgn0051374	chr3R	-	11448500	11448628	11447118	11448359	11451066	11451150	1.2E-05	0.367
98	FBgn0051374	chr3R	-	11448500	11448752	11447118	11448359	11451066	11451150	0.00209	0.288
99	FBgn0051374	chr3R	-	11448500	11448755	11447118	11448359	11451066	11451150	0.00128	0.3
100	FBgn0034364	chr2R	-	18617878	18617907	18617199	18617758	18617977	18618018	0.03367	0.616
101	FBgn0015754	chr2R	+	16181565	16181805	16179332	16179372	16181980	16182090	0.00523	-0.599
102	FBgn0033807	chr2R	-	13213532	13213931	13213022	13213436	13214418	13214496	0	0.858
103	FBgn0031673	chr2L	-	5042960	5043016	5042490	5042890	5043077	5043158	1.2E-05	0.444
104	FBgn0036510	chr3L	+	15564015	15564057	15563426	15563928	15564261	15565559	0.0069	-0.537
105	FBgn0266696	chr3L	+	2442490	2442960	2439498	2439830	2443389	2443838	0.00242	-0.217
106	FBgn0266696	chr3L	+	2442680	2442960	2439498	2439830	2443389	2443838	0.00769	-0.211
107	FBgn0264449	chrX	-	884214	884278	880918	881788	884394	884464	0.00025	-0.659
108	FBgn0004242	chr2L	-	2798486	2798836	2793647	2793908	2799908	2799979	0	-0.352
109	FBgn0033484	chr2R	+	10091552	10091996	10090542	10090799	10093143	10094143	0.00673	0.152
110	FBgn0085342	chr2L	+	16880592	16880660	16879516	16879920	16881034	16881801	0.02936	-0.43
111	FBgn0029580	chrX	+	1467696	1467964	1467492	1467617	1470014	1470097	0.03367	-0.31
112	FBgn0034542	chr2R	-	20665363	20665463	20664785	20665092	20666918	20667306	6.9E-14	-0.617
113	FBgn0011224	chr3R	-	32011039	32011102	31979522	31979619	32014359	32015520	3.7E-06	-0.394
114	FBgn0083124	chr2R	+	9097061	9097183	9096925	9096991	9097241	9097312	9.3E-06	0.321
115	FBgn0058045	chr3L	+	24034877	24035018	24030754	24030938	24035431	24035859	0.00156	0.511
116	FBgn0034605	chr2R	-	21217231	21217434	21217015	21217174	21217727	21217779	0	0.941
117	FBgn0034605	chr2R	-	21217236	21217285	21217015	21217174	21217727	21217779	0.00169	0.362
118	FBgn0034645	chr2R	+	21658216	21658275	21658077	21658109	21658345	21658480	9.8E-08	0.261
119	FBgn0034645	chr2R	+	21658219	21658275	21658077	21658109	21658345	21658480	6E-08	0.264
120	FBgn0034693	chr2R	-	22094523	22094787	22092141	22094043	22099121	22099534	0.02228	0.268
121	FBgn0053170	chr3L	+	22867115	22867318	22865877	22866034	22867399	22867535	0.00144	0.706
122	FBgn0053170	chr3L	+	22867191	22867318	22865877	22866034	22867399	22867535	0.00013	0.739
123	FBgn0003091	chr2R	+	16925618	16925745	16924654	16924790	16926345	16926807	0.00275	0.905
124	FBgn0003091	chr2R	+	16925660	16925745	16924654	16924790	16926345	16926807	0.00153	0.938
125	FBgn0003091	chr2R	+	16925704	16925745	16924654	16924790	16926345	16926807	0.00103	0.937
126	FBgn0263077	chr2R	+	7629833	7630328	7628800	7629112	7634215	7634314	0	-0.495
127	FBgn0263077	chr2R	+	7629870	7630328	7628800	7629112	7634215	7634314	0	-0.5
128	FBgn0263077	chr2R	+	7630056	7630328	7628800	7629112	7634215	7634314	0	-0.496
129	FBgn0263077	chr2R	+	7630101	7630328	7628800	7629112	7634215	7634314	0	-0.499
130	FBgn0263077	chr2R	+	7630147	7630328	7628800	7629112	7634215	7634314	0	-0.499
131	FBgn0263077	chr2R	+	7630185	7630328	7628800	7629112	7634215	7634314	0	-0.499
132	FBgn0263077	chr2R	+	7637283	7637533	7636894	7637193	7638052	7638372	0.00379	-0.341
133	FBgn0034075	chr2R	+	16114560	16114633	16114034	16114472	16114771	16114866	3.9E-06	-0.176
134	FBgn0034075	chr2R	+	16114939	16115053	16114771	16114866	16115353	16115594	0.00153	0.125
135	FBgn0034075	chr2R	+	16114939	16115252	16114771	16114866	16115353	16115594	0.00159	0.119
136	FBgn0034051	chr2R	-	15938114	15938125	15935892	15936580	15938446	15938978	0.00017	0.195
137	FBgn0053182	chr2R	-	13189560	13189980	13187393	13187908	13198212	13198337	0.01382	-0.545
138	FBgn0053182	chr2R	-	13189696	13189980	13187393	13187908	13198212	13198337	1.3E-10	-1
139	FBgn0265356	chr2R	+	19002696	19002780	18997127	18998423	19005788	19006571	2.4E-07	0.575
140	FBgn0265356	chr2R	+	19002696	19002780	19001091	19001789	19005788	19006571	0.0004	0.122
141	FBgn0265356	chr2R	+	19002696	19002833	18997127	18998423	19005788	19006571	0	0.871
142	FBgn0265356	chr2R	+	19002696	19002833	19001091	19001789	19005788	19006571	1.8E-05	0.133
143	FBgn0024811	chr4	+	211627	211792	210581	210680	211853	211982	3E-06	0.302
144	FBgn0024811	chr4	+	211627	211794	210581	210680	212627	212729	4.8E-05	0.275
145	FBgn0016126	chr4	+	446630	446987	445503	445648	447100	447235	2.2E-10	-1
146	FBgn0016126	chr4	+	446820	446987	445503	445648	447100	447235	7.1E-07	-0.834
147	FBgn0039890	chr4	-	190002	190061	188577	189248	193343	193489	0.01055	-0.707
148	FBgn0039890	chr4	-	190024	190061	188577	189248	193343	193489	0.01871	-0.683
149	FBgn0011642	chr4	-	1060492	1060634	1059418	1059836	1064244	1064381	1.7E-12	0.687
150	FBgn0027835	chr2R	+	18414863	18414933	18413325	18413429	18415198	18415286	0.03018	-0.647
151	FBgn0052000	chr4	+	133794	133924	133185	133231	134871	134964	0.00381	0.424
152	FBgn0002921	chr3R	+	20968391	20968486	20966211	20966613	20973197	20973347	0.00057	0.1
153	FBgn0053547	chr3R	-	17893904	17894902	17886148	17886541	17899568	17899728	0.0335	0.34
154	FBgn0024807	chrX	-	21628919	21629105	21628360	21628754	21630066	21630148	0.00578	-0.8
155	FBgn0024807	chrX	-	21628919	21629123	21628360	21628754	21630066	21630148	0.00578	-0.8
156	FBgn0024807	chrX	-	21628919	21629286	21628360	21628754	21630066	21630148	0.00578	-0.8
157	FBgn0010263	chr2L	+	2960724	2960991	2959146	2959520	2961214	2961672	0.02413	-0.667
158	FBgn0053516	chr2L	-	2095751	2096130	2066741	2067160	2109818	2109878	1.4E-05	0.984

159	FBgn0053516	chr2L	-	2095751	2096138	2066741	2067160	2109818	2109878	8.4E-07	0.97
160	FBgn0053516	chr2L	-	2095751	2096226	2066741	2067160	2109818	2109878	0	0.952
161	FBgn0025726	chr4	-	882960	883251	878850	878992	883566	883641	0.04653	0.415
162	FBgn0016930	chr2L	-	14229453	14229536	14222016	14222081	14233647	14234108	1.2E-07	-0.198
163	FBgn0265194	chr2R	-	15272116	15272608	15271389	15271539	15273325	15273388	0.00143	0.391
164	FBgn0265194	chr2R	-	15272117	15272608	15271389	15271539	15273325	15273388	0.025	0.311
165	FBgn0086711	chr2L	-	14994108	14994303	14993898	14994035	14997515	14997559	0.01188	0.573
166	FBgn0086711	chr2L	-	14994108	14994388	14993898	14994035	14997515	14997559	0.00769	0.573
167	FBgn0086711	chr2L	-	14996728	14996970	14992306	14992506	14997515	14997559	0.00311	0.467
168	FBgn0086778	chrX	-	19346731	19346771	19344904	19344989	19347637	19348447	0.01912	0.495
169	FBgn0259174	chr3L	+	17533744	17533849	17533505	17533648	17534350	17534562	0.02413	0.175
170	FBgn0041150	chr2L	-	4925828	4926263	4912671	4912778	4931913	4932384	0	0.226
171	FBgn0260938	chrX	-	15886192	15886352	15884442	15885943	15887117	15887311	0	0.947

2) List of DASs in *Drosophila* heads of *DmAnkrd11* $^{\Delta/\Delta}$ *vs WT*

A3SS

	FlyBase ID	chr	strand	longExonStart_0base	longExonEnd	shortES	shortEE	flankingES	flankingEE	FDR	ΔPSI
1	FBgn0000256	chr2L	-	3886983	3887799	3886983	3887556	3888648	3888824	8.3191E-07	0.332
2	FBgn0016081	chr3L	-	9631833	9632173	9631833	9632170	9632232	9632599	0	0.326
3	FBgn0267376	chr3R	-	10866808	10866976	10866808	10866960	10867971	10868290	0	0.665
4	FBgn0267376	chr3R	-	10866808	10866976	10866808	10866960	10868438	10868642	0	0.829
5	FBgn0266186	chr2R	-	9841846	9842225	9841846	9842167	9842300	9842502	2.8041E-06	-0.133
6	FBgn0263456	chr3L	+	9142572	9143323	9143297	9143323	9141786	9141843	1.0522E-05	0.168
7	FBgn0262975	chr3R	-	23220416	23220896	23220416	23220643	23221761	23221961	0.0014127	0.203
8	FBgn0264489	chr3L	+	9831920	9832679	9832198	9832679	9830633	9830785	0	-0.847
9	FBgn0264489	chr3L	+	9832195	9832679	9832198	9832679	9830633	9830785	0	-0.789
10	FBgn0264489	chr3L	+	9831920	9832679	9832195	9832679	9830999	9831440	0.00E+00	0.597
11	FBgn0264489	chr3L	+	9831920	9832679	9832201	9832679	9830999	9831440	3.70E-09	0.686
12	FBgn0264489	chr3L	+	9832195	9832679	9832198	9832679	9830999	9831440	0.00E+00	-0.718
13	FBgn0264489	chr3L	+	9832195	9832679	9832477	9832679	9830999	9831440	4.28E-02	-0.084
14	FBgn0039897	chr4	+	243169	243585	243265	243585	240644	240833	1.82E-02	0.199
15	FBgn0032938	chr2L	+	21277196	21278429	21277229	21278429	21275723	21275806	3.35E-02	0.311
16	FBgn0259740	chr3R	+	10851540	10851788	10851548	10851788	10851284	10851488	1.99E-08	0.252
17	FBgn0039941	chrX	+	23073103	23073247	23073106	23073247	23071682	23071770	2.70E-03	-0.721
18	FBgn0053558	chr2R	-	6933123	6933927	6933123	6933441	6934065	6934180	8.08E-04	0.19
19	FBgn0260745	chr3R	-	11992741	11992874	11992741	11992852	11993131	11993315	7.09E-03	-0.252
20	FBgn0030087	chrX	-	9095394	9095975	9095394	9095825	9096765	9096825	4.02E-02	0.197
21	FBgn0030087	chrX	-	9097354	9097513	9097354	9097459	9097755	9098007	4.36E-02	-0.164
22	FBgn0034075	chr2R	+	16112195	16112530	16112201	16112530	16111982	16112141	2.79E-10	0.18
23	FBgn0033983	chr2R	-	14932854	14933325	14932854	14933003	14934800	14934861	4.28E-02	0.164
24	FBgn0011640	chr3L	+	7339867	7340770	7339961	7340770	7339463	7339617	1.63E-08	-0.232
25	FBgn0265991	chr2R	-	15765122	15765447	15765122	15765252	15773040	15773253	1.19E-09	0.178
26	FBgn0265991	chr2R	-	15765122	15766713	15765122	15765252	15773040	15773253	1.103E-09	0.159
27	FBgn0265991	chr2R	-	15765122	15767253	15765122	15765252	15773040	15773253	5.0915E-08	0.147
28	FBgn0265991	chr2R	-	15765122	15769551	15765122	15765252	15773040	15773253	3.7132E-08	0.148
29	FBgn0265991	chr2R	-	15765122	15767253	15765122	15766713	15773040	15773253	0.0013941	-0.344
30	FBgn0265991	chr2R	-	15765122	15769551	15765122	15766713	15773040	15773253	0.00164491	-0.337
31	FBgn0265991	chr2R	-	15765122	15765447	15765122	15765252	15773815	15773966	3.0824E-07	0.322
32	FBgn0265991	chr2R	-	15765122	15766713	15765122	15765252	15773815	15773966	8.1032E-10	0.334
33	FBgn0265991	chr2R	-	15765122	15767253	15765122	15765252	15773815	15773966	1.1282E-09	0.335
34	FBgn0265991	chr2R	-	15765122	15769551	15765122	15765252	15773815	15773966	9.7625E-09	0.323
35	FBgn0261565	chr3L	+	16919466	16919702	16919470	16919702	16916254	16916446	0.00723055	0.436
36	FBgn0011603	chr2L	-	4475394	4475504	4475394	4475410	4476841	4477022	0.01686622	0.69
37	FBgn0036059	chr3L	+	9899725	9900084	9899955	9900084	9899240	9899355	0	0.482
38	FBgn0036398	chr3L	+	14003169	14003415	14003196	14003415	14002126	14002301	1.8552E-09	-1
39	FBgn0285963	chr3R	+	26916502	26916562	26916506	26916562	26915760	26915908	0	0.8
40	FBgn0285963	chr3R	+	26916502	26916562	26916512	26916562	26915760	26915908	0	0.868
41	FBgn0285925	chr3R	+	16635545	16635700	16635548	16635700	16632607	16632768	0.00074427	-0.106
42	FBgn0267376	chr3R	-	10866808	10866976	10866808	10866960	10867720	10868081	2.5136E-08	0.357
43	FBgn0267376	chr3R	-	10866808	10866994	10866808	10866960	10867971	10868290	0	0.887
44	FBgn0267376	chr3R	-	10866808	10866994	10866808	10866960	10868438	10868642	0	0.713
45	FBgn0036239	chr3L	-	12001922	12002318	12001922	12001931	12002585	12002915	0.00055248	0.215
46	FBgn0036239	chr3L	-	12001922	12002318	12001922	12001996	12002585	12002915	0.04363596	0.117
47	FBgn0265487	chr2R	+	17276390	17276778	17276745	17276778	17275409	17276063	0.01226282	0.244
48	FBgn0036451	chr3L	+	14791269	14791550	14791375	14791550	14789934	14790072	7.196E-10	1
49	FBgn0264492	chr3L	+	23101017	23101095	23101040	23101095	23100244	23100357	8.652E-07	1
50	FBgn0264386	chrX	-	6117025	6117216	6117025	6117202	6120234	6120349	0.03264162	-0.348
51	FBgn0264386	chrX	-	6191348	6191543	6191348	6191519	6192346	6192483	0.0060798	-0.435
52	FBgn0036331	chr3L	+	13008622	13008873	13008654	13008873	13008284	13008567	1.6958E-12	-0.565
53	FBgn0023000	chr3L	-	345374	346338	345374	345885	346480	346636	0.04017494	0.38
54	FBgn0034725	chr2R	+	22406420	22406814	22406720	22406814	22404176	22404460	0.04686516	0.667
55	FBgn0264324	chr3R	+	28845605	28846000	28845906	28846000	28845299	28845409	3.1335E-08	0.487
56	FBgn0264324	chr3R	+	28845686	28846000	28845906	28846000	28845299	28845409	3.7053E-08	0.483
57	FBgn0264324	chr3R	+	28845848	28846000	28845906	28846000	28845299	28845409	8.1309E-08	0.433
58	FBgn0264324	chr3R	+	28845866	28846000	28845906	28846000	28845299	28845409	6.693E-07	0.467
59	FBgn0263102	chr2R	+	10561685	10561954	10561695	10561954	10557887	10558533	0	-0.565
60	FBgn0262149	chr3R	+	7128702	7129067	7128940	7129067	7128463	7128647	3.235E-05	0.703
61	FBgn0261928	chr3R	+	10239205	10239497	10239476	10239497	10226487	10226619	0.00017194	0.84
62	FBgn0051619	chr2L	+	21708986	21709485	21709383	21709485	21704458	21704603	1.6775E-11	0.822
63	FBgn0264489	chr3L	+	9832195	9832679	9832531	9832679	9830999	9831440	4.3008E-12	0.54
64	FBgn0264489	chr3L	+	9832198	9832679	9832531	9832679	9830999	9831440	0	0.786
65	FBgn0264489	chr3L	+	9832201	9832679	9832531	9832679	9830999	9831440	0	0.912
66	FBgn0264489	chr3L	+	9832354	9832679	9832477	9832679	9830999	9831440	0.0013941	0.205
67	FBgn0264489	chr3L	+	9832477	9832679	9832531	9832679	9830999	9831440	0	0.933
68	FBgn0039282	chr3R	+	25132320	25132501	25132405	25132501	25131612	25131809	0.00028435	-0.291
69	FBgn0051217	chr3R	-	16654462	16654897	16654462	16654832	16655439	16655662	2.86E-09	1
70	FBgn0038420	chr3R	-	16352088	16352359	16352088	16352341	16356799	16357015	1.36E-06	0.834
71	FBgn0085408	chr2R	+	14315103	14315533	14315104	14315533	14308141	14308293	2.96E-06	0.703
72	FBgn0085408	chr2R	+	14315103	14315533	14315236	14315533	14308141	14308293	8.05E-07	0.359
73	FBgn0000056	chr2L	+	14616051	14616437	14616296	14616437	14615551	14615643	4.36E-02	0.244
74	FBgn0000056	chr2L	+	14616155	14616437	14616296	14616437	14615551	14615643	4.02E-02	0.246
75	FBgn0051522	chr3R	-	4445331	4445529	4445331	4445411	4446424	4446532	8.55E-03	0.487
76	FBgn0013334	chr3R	+	15935730	15935912	15935839	15935912	15935118	15935584	1.96E-03	-0.167
77	FBgn0013334	chr3R	+	15935730	15935912	15935861	15935912	15935118	15935584	2.44E-04	-0.303
78	FBgn0012344	chr3R	+	9649776	9650110	9649800	9650110	9648982	9649723	0.00E+00	-0.838
79	FBgn0024963	chr3R	+	19762374	19762797	19762386	19762797	19760899	19761034	1.20E-02	0.413
80	FBgn0004652	chr3R	-	18463266	18463357	18463266	18463281	18506493	18506562	1.20E-02	-0.501
81	FBgn0004652	chr3R	-	18506328	18506360	18506328	18506357	18506493	18506562	2.77E-02	0.667
82	FBgn0039667	chr3R	+	29495587	29495801	29495645	29495801	29494781	29494885	1.41E-02	-0.402
83	FBgn0029746	chrX	+	5089341	5090145	5090015	5090145	5085758	5086201	3.55E-03	-0.289
84	FBgn0036768	chr3L	+	17889156	17889462	17889330	17889462	17887704	17887790	1.74E-04	0.875
85	FBgn0036768	chr3L	+	17889235	17889462	17889330	17889462	17887704	17887790	3.2537E-05	0.893
86	FBgn0036768	chr3L	+	17889313	17889462	17889330	17889462	17887704	17887790	1.3913E-12	0.864
87	FBgn0024321	chr3R	-	14367192	14367499	14367192	14367496	14369792	14369817	0.00164491	0.706
88	FBgn0037299	chr3R	+	5227353	5227526	5227372	5227526	5226157	5226978	0.01874792	0.605
89	FBgn0010772	chr3R	-	5667097	5667412	5667097	5667341	5667692	5667950	0.0137775	-0.454
90	FBgn0263131	chr3L	-	16492170	16492515	16492170	16492462	16492661	16493000	0	0.791
91	FBgn0265726	chrX	+	13711505	13711893	13711515	13711893	13709828	13710395	0.0366772	0.296
92	FBgn0038652	chr3R	-	18768871	18769116	18768871	18769113	18786389	18786830	6.2652E-14	-0.674
93	FBgn0045038	chr2L	-	7870632	7870782	7870632	7870758	7871170	7871432	0.03304839	0.447
94	FBgn0045038	chr2L	-	7870632	7870810	7870632	7870758	7871170	7871432	0.01383702	0.478
95	FBgn0032172	chr2L	-	9960449	9961617	9960449	9961614	9961677	9962059	0.04689545	0.224
96	FBgn0051092	chr3R	-	25718397	25718614	25718397	25718556	25719951	25720117	0	0.669
97	FBgn0051092	chr3R	-	25718397	25718623	25718397	25718614	25719951	25720117	0	-0.627
98	FBgn0051092	chr3R	-	25718397	25718614	25718397	25718556	25719965	25720117	0	0.842
99	FBgn0051092	chr3R	-	25718397	25718623	25718397	25718614	25719965	25720117	0	-0.479
100	FBgn0040532	chr3R	+	8822405	8822666	8822509	8822666	8822007	8822125	0.03347859	0.441
101	FBgn0035501	chr3L	+	4150500	4150722	4150528	4150722	4148695	4149025	0.01686622	0.209
102	FBgn0037094	chr3L	+	21523894	21524067	21523923	21524067	21523185	21523605	8.2547E-05	0.646
103	FBgn0023407	chr2L	-	13546910	13547269	13546910	13547132	13549131	13549328	7.468E-06	1
104	FBgn0023407	chr2L	-	13546910	13547377	13546910	13547132	13549131	13549328	1.1125E-06	0.758
105	FBgn0261261	chr3R	+	5687144	5687369	5687174	5687369	5685854	5686270	0.04363596	-0.573
106	FBgn0085383	chr3R	+	28147499	28148251	28147677	28148251	28146879	28146972	0.00360643	0.384

107	FBgn0038975	chr3R	+	22469231	22469722	22469361	22469722	22468511	22468615	0.04415185	0.534
108	FBgn0010217	chr4	+	1032124	1032237	1032188	1032237	1031863	1031953	0.02691668	-0.306
109	FBgn0010217	chr4	+	1032154	1032237	1032188	1032237	1031863	1031953	0.00180842	-0.627
110	FBgn0004179	chr3L	+	22271137	22271284	22271166	22271284	22270913	22270976	0.02241549	-0.667
111	FBgn0037993	chr3R	-	12156108	12156621	12156108	12156605	12158404	12158595	0.00110207	-0.504
112	FBgn0013576	chr3R	-	5334404	5335060	5334404	5334701	5335713	5336635	0.01431423	-0.612
113	FBgn0013576	chr3R	-	5334404	5334701	5334404	5334698	5349467	5350010	0.02935971	-0.307
114	FBgn0267347	chr2L	+	16680449	16680650	16680485	16680650	16680053	16680276	0.03506431	-0.367
115	FBgn0034075	chr2R	+	16112203	16112530	16112213	16112530	16111982	16112141	0.00164491	0.323
116	FBgn0034075	chr2R	+	16112203	16112530	16112240	16112530	16111982	16112141	7.3963E-08	0.553
117	FBgn0034075	chr2R	+	16114752	16114866	16114771	16114866	16113180	16113258	0.00208099	0.577
118	FBgn0033983	chr2R	-	14932854	14933220	14932854	14933003	14934800	14934861	0.01990835	0.202
119	FBgn0033983	chr2R	-	14932854	14933230	14932854	14933003	14934800	14934861	0.02241549	0.178
120	FBgn0260941	chr3L	-	12264620	12264905	12264620	12264883	12266654	12266841	0.01145706	-0.667
121	FBgn0033246	chr2R	-	7985601	7985781	7985601	7985764	7987065	7987237	3.0032E-05	-0.737
122	FBgn0035049	chr2R	-	24674114	24674531	24674114	24674182	24674972	24675141	0.01689014	-0.667
123	FBgn0024811	chr4	+	211574	211764	211627	211764	210581	210680	3.9338E-11	-0.731
124	FBgn0024811	chr4	+	211591	211764	211627	211764	210581	210680	4.9984E-05	-0.415
125	FBgn0052000	chr4	+	134871	134964	134876	134964	133794	133924	2.2603E-07	-0.356
126	FBgn0052000	chr4	+	136638	136777	136690	136777	136271	136371	0.01145706	0.706
127	FBgn0053547	chr3R	-	17886148	17886544	17886148	17886541	17887420	17887543	0.00768942	0.141
128	FBgn0022361	chr4	+	560473	560820	560717	560820	560371	560411	0.00193685	0.257
129	FBgn0022361	chr4	+	560490	560820	560717	560820	560371	560411	0.00046929	0.321
130	FBgn0038294	chr3R	-	15172599	15173062	15172599	15172830	15173980	15174343	0	0.29
131	FBgn0038294	chr3R	-	15172843	15173063	15172843	15173062	15174136	15174343	0	0.413
132	FBgn0052230	chr3L	+	23341172	23341552	23341457	23341552	23339533	23339680	0.01686622	0.461
133	FBgn0036165	chr3L	+	11490172	11490251	11490204	11490251	11487549	11488601	0.00853847	0.665
134	FBgn0262109	chr3L	+	227517	227759	227553	227759	225725	227459	0	-0.351
135	FBgn0262109	chr3L	+	227542	227759	227553	227759	225725	227459	0	-0.508
136	FBgn0037070	chr3L	-	21303712	21304106	21303712	21304090	21304289	21304467	0.00269667	0.697
137	FBgn0031857	chr2L	-	6770590	6770718	6770590	6770709	6772551	6772755	0	-0.66
138	FBgn0058006	chr2L	-	22720281	22720407	22720281	22720388	22755856	22756349	0.03304839	-0.515
139	FBgn0034137	chr2R	+	16566604	16566992	16566614	16566992	16566022	16566277	0.03681069	-0.336
140	FBgn0259243	chr3L	+	20829079	20829540	20829387	20829540	20828224	20828340	0.03681069	0.667
141	FBgn0259938	chr3R	+	10396236	10396820	10396704	10396820	10388257	10389437	0.04559251	-0.286
142	FBgn0259938	chr3R	+	10396701	10396820	10396704	10396820	10388257	10389437	0.0091826	-0.551
143	FBgn0039525	chr3R	-	27560273	27560620	27560273	27560527	27560762	27560875	0.02909063	0.667
144	FBgn0261243	chr3L	+	1518302	1518790	1518373	1518790	1517850	1517956	0.02761643	0.459

A5SS

	FlyBase ID	chr	strand	longExonStart_0base	longExonEnd	shortES	shortEE	flankingES	flankingEE	FDR	ΔPSI
1	FBgn0026418	chr3L	+	14,037,018	14037412	14037018	14037111	14037477	14037750	6.524E-09	0.73
2	FBgn0036433	chr3L	+	14622795	14623033	14622795	14622935	14626840	14626894	6.4717E-05	-0.152
3	FBgn0035400	chr3L	-	3133648	3134180	3133948	3134180	3132308	3133244	9.64E-07	0.53
4	FBgn0035400	chr3L	-	3,133,648	3134180	3133948	3134180	3132632	3133338	1.91E-09	0.508
5	FBgn0035375	chr3L	+	2,782,254	2782555	2782254	2782496	2782737	2783472	2.05E-08	-1
6	FBgn0030274	chrX	+	11,149,924	11150204	11149924	11150114	11150635	11152127	7.94E-03	-0.429
7	FBgn0267390	chr3L	+	15,566,012	15566799	15566012	15566153	15568205	15568970	2.20E-08	0.244
8	FBgn0264695	chr2L	+	16,767,776	16767954	16767776	16767941	16768583	16768884	2.09E-02	0.688
9	FBgn0264561	chr3L	-	21504541	21504939	21504553	21504939	21503990	21504359	6.91E-05	-0.209
10	FBgn0263346	chr3R	+	4659578	4659893	4659578	4.66E+06	4.66E+06	4660399	1.13E-02	-0.176
11	FBgn0263289	chr3R	+	26536349	26536531	26536349	2.65E+07	2.65E+07	26537784	5.36E-07	0.457
12	FBgn0263289	chr3R	+	26536349	26536531	26536349	2.65E+07	2.65E+07	26537784	0.00322024	0.413
13	FBgn0263289	chr3R	+	26536349	26536601	26536349	2.65E+07	2.65E+07	26537784	0	-0.469
14	FBgn0264357	chr3R	+	21199180	21199396	21199180	2.12E+07	2.12E+07	21201408	3.5698E-12	0.595
15	FBgn0264357	chr3R	+	21199180	21199763	21199180	2.12E+07	2.12E+07	21201408	1.335E-05	0.438
16	FBgn0038947	chr3R	+	22356684	22357047	22356684	2.24E+07	2.24E+07	22357671	0.00028258	-0.15
17	FBgn0039467	chr3R	+	26980517	26980888	26980517	2.70E+07	2.70E+07	26981751	0.02601945	-0.432
18	FBgn0051716	chr2L	+	10397178	10397647	10397178	1.04E+07	1.04E+07	10398219	0.04935921	0.43
19	FBgn0003984	chr3L	-	5816646	5816700	5816650	5.82E+06	5.81E+06	5813474	0.00133886	-0.655
20	FBgn0034364	chr2R	-	18617878	18618018	18617977	1.86E+07	1.86E+07	18617758	0.02374871	-0.454
21	FBgn0032775	chr2L	-	19398334	19398493	19398346	1.94E+07	1.94E+07	19393656	0.0416753	-0.452
22	FBgn0027596	chr2R	-	14,907,024	14907971	14907487	1.49E+07	1.49E+07	14905953	0.00081892	-0.395
23	FBgn0264953	chr2L	+	8182626	8182878	8182626	8.18E+06	8.18E+06	8183563	0.02372429	0.638
24	FBgn0003175	chr2R	-	22561898	22562065	22561918	2.26E+07	2.26E+07	22561142	0.02272179	0.307
25	FBgn0000723	chr3R	-	9371014	9372577	9372376	9.37E+06	9.37E+06	9369710	2.5623E-12	-0.35
26	FBgn0031769	chr2L	+	6050838	6051067	6050838	6.05E+06	6051616	6052022	1.5216E-09	-0.933
27	FBgn0030478	chrX	+	13370994	13371261	13370994	1.34E+07	13373862	13374103	0.00143852	-0.225
28	FBgn0031885	chr2L	+	7056300	7057156	7056300	7.06E+06	7057347	7057674	0.01182415	-0.407
29	FBgn0038846	chr3R	+	21015948	21016041	21015948	2.10E+07	21016219	21016427	1.2372E-07	-0.924
30	FBgn0260388	chr3L	-	16,496,052	16496236	16496138	1.65E+07	16495299	16495536	1.5959E-13	0.475
31	FBgn0033501	chr2R	+	10259515	10259627	10259515	1.03E+07	10260862	10261276	0.00515932	0.736
32	FBgn0260745	chr3R	-	11993131	11993315	11993248	1.20E+07	11992741	11992852	4.2634E-12	0.166
33	FBgn0260745	chr3R	-	11993131	11993315	11993248	1.20E+07	11992741	11992874	0	0.202
34	FBgn0033609	chr2R	-	11289067	11289772	11289669	1.13E+07	11287644	11288560	0.01441122	0.348
35	FBgn0033609	chr2R	-	11289392	11289772	11289669	1.13E+07	11287644	11288560	0.00621385	0.294
36	FBgn0038294	chr3R	-	15173980	15174343	15174136	1.52E+07	15172843	15173063	0	-0.347
37	FBgn0031914	chr2L	+	7440793	7441756	7440793	7.44E+06	7443583	7443832	2.08E-11	-0.782
38	FBgn0031914	chr2L	+	7440793	7441756	7440793	7.44E+06	7443583	7443832	1.2878E-08	-0.708
39	FBgn0261854	chr2R	-	14962816	14962969	14962886	1.50E+07	14961567	14961841	0.02839318	-0.498
40	FBgn0031515	chr2L	-	3294048	3294706	3294082	3.29E+06	3293322	3293949	0.0087097	0.237
41	FBgn0086711	chr2L	-	14996713	14997559	14997270	1.50E+07	14993898	14994035	0.03597968	-0.228
42	FBgn0086711	chr2L	-	14996713	14997559	14997515	1.50E+07	14993898	14994035	1.5736E-10	-0.618
43	FBgn0086711	chr2L	-	14997270	14997559	14997515	1.50E+07	14993898	14994035	1.6489E-09	-0.443
44	FBgn0260722	chr3R	-	9551570	9551748	9551700	9.55E+06	9548103	9549810	0.04832992	0.432
45	FBgn0041150	chr2L	-	4,928,193	4928393	4928227	4.93E+06	4925828	4926346	2.055E-08	-0.62
46	FBgn0041150	chr2L	-	4928197	4928393	4928227	4.93E+06	4925828	4926346	4.3377E-09	-0.642
47	FBgn0041150	chr2L	-	4928227	4928393	4928243	4.93E+06	4925828	4926346	0.00103149	0.389
48	FBgn0261553	chr3L	+	11215778	11215987	11215778	1.12E+07	11216585	11217282	0.00202414	-0.607
49	FBgn0261553	chr3L	+	11215778	11215987	11215778	1.12E+07	11216585	11217282	0.00021146	-0.562
50	FBgn0036134	chr3L	-	11120111	11120411	11120407	1.11E+07	11117304	11118065	8.2021E-05	-0.9
51	FBgn0036433	chr3L	+	14622795	14622946	14622795	1.46E+07	14626840	14626894	0	-0.75
52	FBgn0036663	chr3L	-	16800037	16800189	16800092	16800189	16799483	16799954	0.00145268	-0.807
53	FBgn0040089	chrX	-	19712809	19713045	19712842	19713045	19711944	19712267	0.00460062	-0.763
54	FBgn0027621	chrX	+	19505766	19505848	19505766	19505846	19506482	19509350	0.00926354	-0.667
55	FBgn0035400	chr3L	-	3133956	3134058	3133976	3134058	3132632	3133338	0.00958783	-0.514
56	FBgn0035431	chr3L	-	3302855	3303112	3302892	3303112	3300722	3300883	0.00550664	0.657
57	FBgn0284408	chrX	-	2472754	2473099	2472947	2473099	2470459	2471194	0.02372429	-0.667
58	FBgn0283510	chr2R	+	9963078	9963133	9963078	9963124	9963197	9963853	0.01911723	0.351
59	FBgn0267390	chr3L	+	15566012	15566188	15566012	15566153	15568205	15568970	9.9009E-08	0.271
60	FBgn0023001	chr3L	+	7138225	7138511	7138225	7138350	7139172	7139327	0.01184434	0.346
61	FBgn0266599	chr3R	+	15242909	15243180	15242909	15242970	15244359	15246614	0.02289611	0.137
62	FBgn0266599	chr3R	+	15242909	15243184	15242909	15242970	15244359	15246614	0.03013249	0.179
63	FBgn0266599	chr3R	+	15242909	15243294	15242909	15242970	15244359	15246614	0.02033254	0.182
64	FBgn0266178	chr3R	-	21359362	21359768	21359584	21359768	21358004	21359069	0.0032895	0.355
65	FBgn0036715	chr3L	-	17379360	17379530	17379362	17379530	17378285	17378592	0.02327636	0.301
66	FBgn0015218	chr3L	-	9,401,568	9401805	9401784	9401805	9401039	9401367	3.3629E-05	0.808
67	FBgn0015218	chr3L	-	9401570	9401805	9401784	9401805	9401039	9401367	0.00106915	0.752

68	FBgn0015218	chr3L	-	9,401,606	9401805	9401784	9401805	9401039	9401367	8.2625E-12	0.927
69	FBgn0263996	chr2L	-	20876586	20877083	20876911	20877083	20874273	20874432	0.03627719	0.156
70	FBgn0265002	chr2L	-	10180106	10180256	10180216	10180256	10178040	10178477	0.04128301	0.325
71	FBgn0263456	chr3L	+	9137859	9138163	9137859	9137889	9138581	9138697	0.00220275	0.17
72	FBgn0013272	chr2R	+	22317661	22317810	22317661	22317768	22317881	22317954	0.02535718	-0.213
73	FBgn0263346	chr3R	+	4659578	4659885	4659578	4659724	4660238	4660399	0.00133886	-0.649
74	FBgn0263346	chr3R	+	4,659,578	4659885	4659578	4659825	4660238	4660399	0.00288994	-0.619
75	FBgn0263346	chr3R	+	4659578	4660032	4659578	4659825	4660238	4660399	0.00306776	-0.227
76	FBgn0263346	chr3R	+	4659578	4659885	4659578	4659827	4660238	4660399	1.40E-02	-0.728
77	FBgn0262593	chr3L	+	2,895,119	2895518	2895119	2895182	2897243	2897429	1.58E-04	-0.595
78	FBgn0262593	chr3L	+	2,895,119	2895518	2895119	2895284	2897243	2897429	9.55E-12	-0.915
79	FBgn0039970	chr2R	+	5,608,634	5608835	5608634	5608831	5609012	5609994	4.52E-03	0.749
80	FBgn0260795	chr3L	+	11,123,347	11123680	11123347	11123676	11128691	11128744	1.10E-03	0.4
81	FBgn0028671	chr3R	+	29,131,266	29131557	29131266	29131447	29131685	29131825	3.60E-02	0.21
82	FBgn0266918	chr3L	-	3070650	3070839	3070712	3070839	3058065	3058124	1.40E-02	-0.667
83	FBgn0264489	chr3L	+	9813846	9813975	9813846	9.81E+06	9.82E+06	9820559	2.02E-03	-0.268
84	FBgn0264489	chr3L	+	9813846	9813975	9813846	9.81E+06	9.82E+06	9820559	5.41E-06	-0.65
85	FBgn0000206	chr3R	+	25961993	25962043	25961993	2.60E+07	2.60E+07	25963615	0	-0.236
86	FBgn0000206	chr3R	+	25961993	25962058	25961993	2.60E+07	2.60E+07	25963615	4.4031E-08	-0.844
87	FBgn0000206	chr3R	+	25961993	25962221	25961993	2.60E+07	2.60E+07	25963615	0	-0.875
88	FBgn0005649	chr3R	-	24046706	24047012	24046941	2.40E+07	2.40E+07	24046491	2.2005E-05	1
89	FBgn0005649	chr3R	-	24046937	24047012	24046941	2.40E+07	2.40E+07	24046491	1.283E-05	0.389
90	FBgn0005649	chr3R	-	24046941	24047368	24047116	2.40E+07	2.40E+07	24046491	5.5916E-06	-0.456
91	FBgn0038947	chr3R	+	22356684	22356852	22356684	2.24E+07	2.24E+07	22357671	9.0859E-07	-0.54
92	FBgn0038947	chr3R	+	22356684	22356852	22356684	2.24E+07	2.24E+07	22357671	0.00012052	-0.454
93	FBgn0038947	chr3R	+	22356684	22356852	22356684	2.24E+07	2.24E+07	22357671	0.00358474	-0.151
94	FBgn0038947	chr3R	+	22356684	22357075	22356684	2.24E+07	2.24E+07	22357671	0.00299884	-0.143
95	FBgn0038947	chr3R	+	22,356,684	22357075	22356684	2.24E+07	2.24E+07	22357671	0.00010987	1
96	FBgn0260003	chr3R	+	19569209	19569672	19569209	1.96E+07	1.96E+07	19586514	0.014001	0.581
97	FBgn0013305	chr2R	-	12879447	12879656	12879573	1.29E+07	1.29E+07	12879222	0.00201738	0.615
98	FBgn0039467	chr3R	+	26980517	26981002	26980517	2.70E+07	2.70E+07	26981751	0.02461071	-0.433
99	FBgn0003462	chr3L	-	11113266	11113740	11113270	1.11E+07	11112280	11112848	0.01111174	-0.724
100	FBgn0025879	chr3R	-	10205508	10205667	10205592	1.02E+07	10205249	10205452	0.03498415	0.587
101	FBgn0051522	chr3R	-	4448012	4448283	4448033	4.45E+06	4447700	4447954	1.2878E-08	1
102	FBgn0264908	chr3L	-	15898598	15898920	15898756	1.59E+07	15893619	15893961	0.00343794	-0.641
103	FBgn0264908	chr3L	-	15,898,756	15898920	15898831	1.59E+07	15893619	15893961	0.0004775	0.47
104	FBgn0264908	chr3L	-	15898756	15898920	15898834	1.59E+07	15893619	15893961	0.00046005	0.336
105	FBgn0264908	chr3L	-	15898756	15899029	15898935	1.59E+07	15893619	15893961	0.00026695	0.42
106	FBgn0052057	chr3L	-	10185978	10186108	10186022	1.02E+07	10182242	10182530	0.00051127	0.667
107	FBgn0039637	chr3R	+	29123369	29123489	29123369	2.91E+07	29123800	29124104	0	0.511
108	FBgn0039637	chr3R	+	29123415	29123499	29123415	2.91E+07	29123800	29124104	0.00052944	0.199
109	FBgn0024321	chr3R	-	14369389	14369817	14369792	1.44E+07	14367192	14367496	0.00025784	0.592
110	FBgn0085443	chrX	-	10585287	10585422	10585309	1.06E+07	10584909	10585073	0.00052944	0.785
111	FBgn0024944	chr3R	-	20713832	20713912	20713852	2.07E+07	20713318	20713750	0.00105495	-0.847
112	FBgn0003255	chr2L	-	14000967	14001248	14001134	1.40E+07	13992936	13993830	0.01095633	0.641
113	FBgn0034180	chr2R	-	17015420	17015894	17015693	1.70E+07	17014948	17015202	7.9374E-07	0.846
114	FBgn0014184	chr2R	-	12173920	12174264	12173942	1.22E+07	12167993	12168203	0.00676364	0.593
115	FBgn0033558	chr2R	-	10834016	10834236	10834204	1.08E+07	10833807	10833962	5.9248E-07	-0.889
116	FBgn0003984	chr3L	-	5816612	5816700	5816650	5.82E+06	5813337	5813474	0.00022352	-0.378
117	FBgn0261714	chr3R	-	12165852	12166186	12166010	1.22E+07	12163621	12165673	0.00717051	0.702
118	FBgn0261714	chr3R	-	12,165,852	12166186	12166158	1.22E+07	12163621	12165673	6.817E-07	0.856
119	FBgn0261714	chr3R	-	12165852	12166186	12166162	1.22E+07	12163621	12165673	1.5531E-06	0.521
120	FBgn0010909	chr3L	-	2586165	2586540	2586265	2.59E+06	2585604	2585738	0.03074376	-0.602
121	FBgn0038256	chr3R	-	14731383	14731509	14731404	1.47E+07	14730510	14730629	0.02592231	-0.208
122	FBgn0038256	chr3R	-	14731404	14731509	14731408	1.47E+07	14730510	14730629	3.6626E-12	0.443
123	FBgn0031589	chr2L	-	4197951	4198071	4197964	4.20E+06	4196474	4196814	0.04935921	0.475
124	FBgn0052432	chr3L	-	20884989	20885188	20885036	2.09E+07	20881629	20881810	5.4148E-06	-0.195
125	FBgn0052432	chr3L	-	20885031	20885188	20885102	20885188	20881629	20881810	0.00159659	0.555
126	FBgn0051140	chr3R	+	23963179	23963384	23963179	23963233	23965799	23966702	0.01467677	-0.41
127	FBgn0051140	chr3R	+	23963179	23963545	23963179	23963384	23965799	23966702	4.102E-05	0.68
128	FBgn0052944	chr3R	-	4419106	4419578	4419359	4419578	4415682	4415886	7.612E-10	0.594
129	FBgn0052944	chr3R	-	4419138	4419578	4419359	4419578	4415682	4415886	0.01189656	0.596
130	FBgn0040370	chrX	-	294683	294962	294822	294962	290252	290596	0.04198454	0.219
131	FBgn0037213	chr3R	+	4174631	4174796	4174631	4174765	4174855	4176191	0.00110723	-0.591
132	FBgn0000579	chr2L	-	1726752	1727717	1727317	1727717	1725059	1726655	0.00351144	0.667
133	FBgn0000579	chr2L	-	1726953	1727402	1727317	1727402	1725059	1726655	0.00351144	0.667
134	FBgn0000579	chr2L	-	1726964	1727402	1727317	1727402	1725059	1726655	0.0087097	0.667
135	FBgn0003175	chr2R	-	22561898	22562065	22561933	22562065	22560745	22561142	0.00265924	0.574
136	FBgn0261822	chr2L	+	8088303	8088516	8088303	8088370	8089184	8089736	0.0008593	-0.696
137	FBgn0261822	chr2L	+	8088303	8088715	8088303	8088516	8089184	8089736	0.00058372	0.338
138	FBgn0250823	chr3R	+	16280888	16281024	16280888	16281011	16282088	16282408	0.014001	0.681
139	FBgn0000024	chr3R	-	13,258,302	13258425	13258304	13258425	13245809	13246654	0.00598325	0.231
140	FBgn0051092	chr3R	-	25719965	25720117	25720004	25720117	25718397	25718623	0.0032201	0.201
141	FBgn0051092	chr3R	-	25,740,151	25740465	25740245	25740465	25739054	25739622	2.4971E-06	0.672
142	FBgn0051092	chr3R	-	25740244	25740465	25740245	25740465	25739054	25739622	1.5959E-13	0.512
143	FBgn0002719	chr3R	-	12720543	12720784	12720638	12720784	12719402	12720027	0.00740208	-0.747
144	FBgn0002719	chr3R	-	12720543	12720784	12720735	12720784	12719402	12720027	8.7123E-06	-0.655
145	FBgn0002719	chr3R	-	12720700	12720784	12720735	12720784	12719402	12720027	0.04051592	-0.667
146	FBgn0262869	chr3R	-	20449234	20449687	20449363	20449687	20437680	20438135	0.00048368	-0.815
147	FBgn0020621	chr2R	-	9,274,854	9275030	9274883	9275030	9271984	9272174	0.03137242	-0.772
148	FBgn0259978	chr2R	+	5702111	5702299	5702111	5702187	5702866	5702947	0.00923101	-0.491
149	FBgn0038460	chr3R	-	16986118	16986245	16986126	16986245	16984570	16984617	1.36E-03	-0.248
150	FBgn0032123	chr2L	+	9,521,209	9521694	9521209	9521395	9523162	9523343	4.83E-02	0.242
151	FBgn0032123	chr2L	+	9,521,209	9521694	9521209	9521476	9523162	9523343	6.68E-04	0.667
152	FBgn0036821	chr3L	-	18,884,583	18884845	18884716	18884845	18882318	18882530	1.74E-04	-0.838
153	FBgn0039941	chrX	+	23,071,682	23071770	23071682	23071766	23073106	23073247	3.07E-02	0.681
154	FBgn0035501	chr3L	+	4,148,695	4149028	4148695	4149020	4150221	4150391	2.09E-02	0.667
155	FBgn0029155	chr3R	+	27156140	27156518	27156140	27156305	27157143	27157603	1.80E-07	-0.23
156	FBgn0260388	chr3L	-	16494910	16495268	16495228	1.65E+07	1.65E+07	16493940	2.88E-06	-0.723
157	FBgn0260388	chr3L	-	16495051	16495268	16495228	1.65E+07	1.65E+07	16493940	2.53E-07	-0.746
158	FBgn0087007	chr3L	-	14536038	14536276	14536066	1.45E+07	1.45E+07	14533722	0.03875956	-0.504
159	FBgn0087007	chr3L	-	14536054	14536276	14536066	1.45E+07	1.45E+07	14533722	0.00021154	-0.753
160	FBgn0051038	chr3R	+	29877058	29877455	29877058	2.99E+07	2.99E+07	29879810	0.02397626	0.369
161	FBgn0027779	chr2L	-	16725891	16726113	16725942	1.67E+07	1.67E+07	16724993	0.01751297	0.308
162	FBgn0051635	chr2L	-	6647707	6648076	6647793	6.65E+06	6.65E+06	6646947	4.3667E-07	0.871
163	FBgn0051635	chr2L	-	6647784	6648076	6647793	6.65E+06	6.65E+06	6646947	1.4674E-07	1
164	FBgn0053080	chrX	-	5713339	5713527	5713343	5.71E+06	5.71E+06	5711803	0.00050603	0.582
165	FBgn0029837	chrX	-	6072946	6072997	6072988	6.07E+06	6.07E+06	6071875	0.00023507	-0.813
166	FBgn0025592	chr3L	-	205531	205809	205664	2.06E+05	2.05E+05	205472	0.00800644	0.667
167	FBgn0051183	chr3R	-	15875494	15875939	15875565	1.59E+07	1.59E+07	15870232	0.0087483	-0.596
168	FBgn0051183	chr3R	-	15,875,494	15875939	15875572	1.59E+07	1.59E+07	15870232	0	0.785
169	FBgn0051183	chr3R	-	15875494	15875939	15875577	1.59E+07	1.59E+07	15870232	0	-0.835
170	FBgn0051183	chr3R	-	15875565	15875939	15875572	1.59E+07	1.59E+07	15870232	6.8871E-10	1
171	FBgn0051183	chr3R	-	15875572	15875939	15875577	1.59E+07	1.59E+07	15870232	0	-1
172	FBgn0034530	chr2R	-	20632789	20633506	20633350	2.06E+07	20630683	20630836	0.00911232	-0.318
173	FBgn0003380	chrX	-	17990304	17990614	17990327	1.80E+07	17976350	17976791	0.04125829	0.432
174	FBgn0003380	chrX	-	17990304	17990614	17990333	1.80E+07	17976350	17976791	0.03050529	0.355
175	FBgn0003380	chrX	-	17990304	17990614	17990532	1.80E+07	17976350	17976791	0.00343327	0.619
176	FBgn0028496	chr2R	-	18,497,773	18498026	18497848	1.85E+07	18496746	18496868	1.8004E-07	-0.895
177	FBgn0028496	chr2R	-	18497848	18498026	18497885	1.85E+07	18496746	18496868	0.0007192	0.673
178	FBgn0265318	chr3R	-	8563396	8563798	8563408	8.56E+06	8562934	8563329	0.00130418	0.684

179	FBgn0034691	chr2R	+	22080684	22080763	22080684	2.21E+07	22081074	22082637	0.00058096	-0.798
180	FBgn0030699	chrX	+	15844636	15845005	15844636	1.58E+07	15845326	15845468	0.03498415	-0.42
181	FBgn0052675	chrX	-	10763715	10764093	10763874	1.08E+07	10761914	10762434	8.2564E-09	-0.875
182	FBgn0003748	chr2R	+	21080607	21080737	21080607	2.11E+07	21084492	21084698	0.0022189	-0.829
183	FBgn0003748	chr2R	+	21080607	21080885	21080607	2.11E+07	21084492	21084698	0.00110013	0.778
184	FBgn0034644	chr2R	+	21653638	21654067	21653638	2.17E+07	21654774	21654907	0.02422322	0.774
185	FBgn0260943	chr3L	-	17237831	17238212	17237885	1.72E+07	17235351	17236062	3.5896E-06	0.3
186	FBgn0260943	chr3L	-	17237885	17238212	17237949	1.72E+07	17235351	17236062	2.9635E-10	-1
187	FBgn0260943	chr3L	-	17237885	17238212	17238071	1.72E+07	17235351	17236062	0.00109799	-0.371
188	FBgn0260943	chr3L	-	17237885	17238212	17238094	1.72E+07	17235351	17236062	0.01647437	-0.444
189	FBgn0260943	chr3L	-	17237949	17238212	17238075	1.72E+07	17235351	17236062	0.01334168	0.675
190	FBgn0038063	chr3R	-	12595394	12595725	12595631	1.26E+07	12560400	12561804	0.014001	0.344
191	FBgn0038063	chr3R	-	12,595,560	12595725	12595631	1.26E+07	12560400	12561804	0.00314812	0.207
192	FBgn0033799	chr2R	-	13167625	13168032	13167788	1.32E+07	13166423	13166635	0.02839318	0.667
193	FBgn0038744	chr3R	+	19890688	19891254	19890688	1.99E+07	19892516	19894015	0.03373264	-0.834
194	FBgn0031717	chr2L	+	5547289	5547756	5547289	5.55E+06	5547807	5547912	9.784E-08	0.506
195	FBgn0033437	chr2R	-	9609938	9610290	9610045	9.61E+06	9609095	9609442	0.00245423	0.68
196	FBgn0028697	chr3L	+	27267254	27267523	27267254	2.73E+07	27267591	27267918	0.01931295	0.667
197	FBgn0035785	chr3L	+	7457700	7457959	7457700	7.46E+06	7458106	7458608	1.5959E-13	1
198	FBgn0035049	chr2R	-	24674786	24675139	24674824	24675139	24671293	24672306	0.01472137	0.705
199	FBgn0016126	chr4	+	445912	446171	445912	445965	447100	447235	0.03578298	-0.601
200	FBgn0261674	chr3L	+	12790191	12790382	12790191	12790324	12791434	12791633	0.02981691	0.218
201	FBgn0261674	chr3L	+	12790191	12790402	12790191	12790324	12791434	12791633	0.04607957	0.52
202	FBgn0261674	chr3L	+	12790191	12790513	12790191	12790324	12791434	12791633	0.02571623	0.542
203	FBgn0261674	chr3L	+	12790191	12790612	12790191	12790324	12791434	12791633	0.02571623	0.542
204	FBgn0039890	chr4	-	193343	193489	193393	193489	189997	190061	0.00618616	-0.538
205	FBgn0261617	chrX	-	9685471	9685667	9685549	9685667	9682551	9684151	0.00976876	-0.515
206	FBgn0005666	chr4	+	734341	734812	734341	734537	735514	735823	0.02305809	-0.299
207	FBgn0022361	chr4	+	560348	560574	560348	560411	560717	560820	0.0007265	0.297
208	FBgn0025835	chrX	-	147953	148006	147961	148006	146434	147785	0.01622025	-0.375
209	FBgn0003969	chrX	-	16005319	16005812	16005745	16005812	16003714	16004086	0.03025712	0.419
210	FBgn0010263	chr2L	+	2956910	2957051	2956910	2956992	2960689	2960839	0.00859831	0.585
211	FBgn0037747	chr3R	+	9767833	9768242	9767833	9768199	9768922	9770027	0.01816021	-0.667
212	FBgn0039900	chr4	-	305,742	306161	306059	306161	305015	305178	0.02744776	-0.226
213	FBgn0039900	chr4	-	305813	306161	306059	306161	305015	305178	0.0463602	-0.218
214	FBgn0036144	chr3L	-	11,197,290	11197338	11197304	11197338	11196566	11196875	0.03496477	0.436
215	FBgn0036144	chr3L	-	11197304	11197536	11197408	11197536	11196566	11196875	2.3935E-07	-1
216	FBgn0031914	chr2L	+	7440793	7441021	7440793	7440958	7443583	7443832	0	-0.801
217	FBgn0031914	chr2L	+	7440793	7441021	7440793	7440962	7443583	7443832	0	-0.695
218	FBgn0011640	chr3L	+	7339463	7339905	7339463	7339617	7339961	7340770	0	-0.623
219	FBgn0011640	chr3L	+	7339867	7339908	7339867	7339905	7339961	7340770	0.00246376	0.352
220	FBgn0051361	chr3R	+	12,095,629	12095922	12095629	12095716	12096048	12097016	0.01911723	0.627
221	FBgn0250785	chr2L	-	20793877	20794146	20793967	20794146	20792113	20792266	0.0001296	0.602
222	FBgn0250785	chr2L	-	20793877	20794146	20794059	20794146	20792113	20792266	7.01E-03	0.335
223	FBgn0003514	chrX	-	6,224,692	6225116	6224696	6225116	6223584	6224116	3.52E-02	0.497
224	FBgn0035976	chr3L	+	9,340,355	9340593	9340355	9340501	9342164	9343129	3.12E-02	0.501
225	FBgn0034897	chr2R	+	23,721,785	23722069	23721785	23721883	23722209	23722875	2.41E-06	-1
226	FBgn0013718	chr3L	+	14,221,439	14221503	14221439	14221500	14225430	14226403	3.85E-02	0.285
227	FBgn0261854	chr2R	-	14,958,217	14958456	14958227	14958456	14953086	14953655	3.88E-02	-0.561
228	FBgn0034985	chr2R	+	24162488	24162847	24162488	24162705	24166412	24166498	3.31E-03	-0.479
229	FBgn0016976	chrX	-	23004428	23004648	23004487	2.30E+07	2.30E+07	22995914	2.69E-03	0.371
230	FBgn0040005	chr2R	+	4672277	4673041	4672277	4.67E+06	4.67E+06	4673223	2.20E-03	-0.778
231	FBgn0038465	chr3R	+	17006104	17006274	17006104	1.70E+07	1.70E+07	17006501	6.5545E-09	1
232	FBgn0265194	chr2R	-	15308841	15308997	15308892	1.53E+07	1.53E+07	15307154	0.02610508	0.77
233	FBgn0250910	chr3R	-	12547286	12547704	12547290	1.25E+07	1.25E+07	12522659	0	-0.552
234	FBgn0030701	chrX	+	15870182	15870520	15870182	1.59E+07	1.59E+07	15871523	0.00468538	-0.215
235	FBgn0035253	chr3L	-	1660625	1660683	1660650	1.66E+06	1.66E+06	1659990	0.02973171	-0.767
236	FBgn0259163	chr3L	-	9736946	9737274	9737128	9.74E+06	9.73E+06	9735289	0.00017835	-0.141
237	FBgn0016694	chr3L	-	7832553	7833073	7832863	7.83E+06	7.83E+06	7831810	0.00060258	0.478
238	FBgn0016694	chr3L	-	7832702	7833073	7832863	7.83E+06	7.83E+06	7831810	1.8004E-07	0.811
239	FBgn0016694	chr3L	-	7832855	7833073	7832863	7.83E+06	7.83E+06	7831810	0.00046005	0.61
240	FBgn0016694	chr3L	-	7832861	7833073	7832863	7.83E+06	7.83E+06	7831810	0.00636334	0.543
241	FBgn0016694	chr3L	-	7,832,553	7833073	7832702	7.83E+06	7.83E+06	7831827	0.01202624	0.389
242	FBgn0039830	chr3R	-	31219305	31219559	31219471	3.12E+07	3.12E+07	31219048	0.00201359	0.667

MXE

	FlyBase ID	chr	strand	1stExonStart_0base	1stExonEnd	ExonStart_0	2ndExonEnc	upstreamES	upstreamEE	ownstreamE	downstreamEE	FDR	∆PSI
1	FBgn0035756	chr3L	-	7284604	7285217	7294790	7294919	7282153	7282403	7311928	7312230	0.00014	0.262
2	FBgn0035914	chr3L	+	8609733	8610356	8613657	8613819	8604656	8604914	8614601	8614809	0.01464	-0.135
3	FBgn0263346	chr3R	+	4670796	4670850	4680778	4680837	4659578	4660399	4697190	4697542	0	-0.386
4	FBgn0051973	chr2L	-	30393	33270	33844	34288	28981	29068	34557	34604	0.00096	-0.181
5	FBgn0033159	chr2R	-	7336116	7336410	7336530	7336818	7332260	7332380	7347848	7348010	0.00051	-0.833
6	FBgn0033159	chr2R	-	7336116	7336410	7337088	7337376	7332260	7332380	7347848	7348010	0.00048	-0.889
7	FBgn0033159	chr2R	-	7336116	7336410	7337871	7338159	7332260	7332380	7347848	7348010	0.00412	-0.414
8	FBgn0033159	chr2R	-	7336116	7336410	7338249	7338537	7332260	7332380	7347848	7348010	0.00037	-0.736
9	FBgn0033159	chr2R	-	7336116	7336410	7338640	7338928	7332260	7332380	7347848	7348010	0.00145	-0.489
10	FBgn0033159	chr2R	-	7336116	7336410	7340154	7340442	7332260	7332380	7347848	7348010	0.02017	-0.517
11	FBgn0033159	chr2R	-	7336116	7336410	7341327	7341618	7332260	7332380	7347848	7348010	4.8E-06	-0.752
12	FBgn0033159	chr2R	-	7336116	7336410	7342103	7342391	7332260	7332380	7347848	7348010	7.6E-08	-0.782
13	FBgn0033159	chr2R	-	7336116	7336410	7342582	7342881	7332260	7332380	7347848	7348010	0.00049	-0.556
14	FBgn0033159	chr2R	-	7336116	7336410	7343717	7344008	7332260	7332380	7347848	7348010	0.00202	-0.402
15	FBgn0033159	chr2R	-	7336116	7336410	7344663	7344954	7332260	7332380	7347848	7348010	0.00037	-0.735
16	FBgn0033159	chr2R	-	7336116	7336410	7345060	7345351	7332260	7332380	7347848	7348010	0.00727	-0.371
17	FBgn0033159	chr2R	-	7336116	7336410	7345484	7345775	7332260	7332380	7347848	7348010	0.0007	-0.415
18	FBgn0033159	chr2R	-	7336116	7336410	7347391	7347697	7332260	7332380	7347848	7348010	9.4E-05	-0.681
19	FBgn0033159	chr2R	-	7350373	7350497	7356106	7356230	7348130	7348975	7361717	7361867	0.02376	-0.292
20	FBgn0033159	chr2R	-	7350373	7350497	7357788	7357915	7348130	7348975	7361717	7361867	0.02585	-0.469
21	FBgn0033159	chr2R	-	7353244	7353365	7354685	7354809	7348130	7348975	7361717	7361867	0.01588	0.415
22	FBgn0033159	chr2R	-	7354685	7354809	7358603	7358727	7348130	7348975	7361717	7361867	0.01344	-0.5
23	FBgn0033159	chr2R	-	7363331	7363490	7365017	7365179	7361717	7361867	7368387	7368732	0.00075	-0.514
24	FBgn0033159	chr2R	-	7363331	7363490	7365311	7365482	7361717	7361867	7368387	7368732	0.00142	-0.488
25	FBgn0033159	chr2R	-	7363880	7364048	7365017	7365179	7361717	7361867	7368387	7368732	0.02921	-0.585
26	FBgn0023023	chr3R	+	5576174	5576413	5577035	5577259	5575778	5575925	5577592	5577828	0.01413	-0.157
27	FBgn0002921	chr3R	+	20967107	20967202	20968391	20968486	20966397	20966613	20973172	20973347	0.01344	-0.119
28	FBgn0041182	chr2L	-	7696593	7696807	7697400	7697581	7696027	7696206	7698724	7698992	0.00069	-0.241
29	FBgn0261788	chr3L	-	7656374	7661506	7662470	7662890	7655388	7656203	7663258	7663324	0.00293	0.153
30	FBgn0035914	chr3L	+	8607058	8607422	8608829	8609134	8604656	8604858	8609733	8610356	0.01413	-0.306
31	FBgn0001105	chrX	+	15860744	15861189	15861235	15861311	15860114	15860322	15861376	15861486	0.00128	-0.734
32	FBgn0001105	chrX	+	15860744	15861189	15861246	15861311	15860114	15860322	15861376	15861486	0.00822	-0.709
33	FBgn0001105	chrX	+	15861057	15861189	15861246	15861311	15860114	15860250	15861376	15861486	0.02723	-0.563
34	FBgn0001105	chrX	+	15861057	15861189	15861235	15861311	15860114	15860322	15861376	15861486	0.00116	-0.737
35	FBgn0001105	chrX	+	15861057	15861189	15861246	15861311	15860114	15860322	15861376	15861486	0.0077	-0.711
36	FBgn0285955	chr3R	-	14439407	14439482	14445472	14445689	14431973	14433205	14451953	14452006	0.00727	-0.764
37	FBgn0266599	chr3R	+	15242909	15242970	15243159	15243180	15242686	15242799	15244359	15246614	0.00012	-0.599
38	FBgn0266599	chr3R	+	15242909	15242970	15243159	15243294	15242686	15242799	15244359	15246614	0.04003	-0.596
39	FBgn0265487	chr2R	+	17276390	17276778	17332574	17332641	17275409	17276063	17351093	17351264	1.5E-05	0.667
40	FBgn0265487	chr2R	+	17276390	17276778	17332575	17332641	17275409	17276063	17351093	17351264	0.00051	0.621
41	FBgn0263346	chr3R	+	4670795	4670850	4680778	4680837	4660238	4660399	4697190	4697542	0	-0.48

42	FBgn0263006	chr2R	-	23933018	23933343	23933446	23933819	23932666	23932814	23933908	23934084	0.04828	0.3
43	FBgn0263006	chr2R	-	23933093	23933343	23933446	23933819	23932666	23932814	23933908	23934084	0.01236	0.667
44	FBgn0053144	chr2R	-	10766431	10766550	10782673	10782969	10759862	10759915	10798339	10798709	0.00033	0.491
45	FBgn0053144	chr2R	-	10766480	10766550	10782673	10782969	10759862	10759915	10798339	10798709	0.00325	0.581
46	FBgn0051619	chr2L	+	21708986	21709062	21709383	21709485	21704458	21704603	21710563	21710693	1.7E-05	0.705
47	FBgn0041605	chr3R	+	4296299	4296502	4296891	4297083	4295567	4295700	4297487	4304604	0.02766	0.521
48	FBgn0032946	chr2L	+	21380470	21380803	21380918	21381278	21380132	21380361	21382018	21382330	0.00255	0.326
49	FBgn0032946	chr2L	+	21380470	21380803	21381006	21381278	21380132	21380361	21382018	21382330	0.01016	0.448
50	FBgn0032946	chr2L	+	21380470	21380803	21381080	21381278	21380132	21380361	21382018	21382330	0.0015	0.54
51	FBgn0032946	chr2L	+	21380679	21380728	21381080	21381199	21380132	21380361	21382018	21382330	0.00755	0.392
52	FBgn0032946	chr2L	+	21380679	21380728	21380918	21381199	21380132	21380478	21382018	21382330	0.02723	0.297
53	FBgn0032946	chr2L	+	21380679	21380728	21380918	21381278	21380132	21380478	21382018	21382330	0.02238	0.489
54	FBgn0032946	chr2L	+	21380679	21380803	21380918	21381278	21380132	21380478	21382018	21382330	0.03265	0.604
55	FBgn0013995	chr3R	+	21010155	21010203	21010606	21010615	21008721	21008736	21011266	21011415	0.00573	-0.667
56	FBgn0031213	chr2L	+	73389	73620	74465	74572	72386	72977	74902	75018	0.01937	-0.319
57	FBgn0031213	chr2L	+	73389	73620	74479	74572	72386	72977	74902	75018	0.02045	-0.309
58	FBgn0014184	chr2R	-	12168863	12169254	12169722	12170044	12167993	12168203	12170923	12171285	0.00291	0.381
59	FBgn0016696	chr3L	+	20772681	20772970	20773092	20773132	20771667	20771864	20773793	20776053	0.00032	-0.391
60	FBgn0016696	chr3L	+	20772681	20772980	20773092	20773132	20771667	20771864	20773793	20776053	0.00584	-0.513
61	FBgn0069969	chr2R	-	928893	929309	949391	949802	927355	927570	949870	949946	4.5E-07	0.722
62	FBgn0069969	chr2R	-	928893	929309	949391	949817	927355	927570	949870	949946	0.02682	0.324
63	FBgn0004449	chr3L	-	22327103	22327139	22327139	22327256	22307546	22308191	22340461	22340641	4.7E-10	-1
64	FBgn0033807	chr2R	-	13213532	13213558	13213750	13213931	13213022	13213436	13214418	13214496	0.01937	0.134
65	FBgn0032901	chr2L	-	20882029	20882464	20885288	20885584	20877552	20877677	20890182	20890395	0.00051	0.833
66	FBgn0032901	chr2L	-	20882029	20882464	20885288	20885731	20877552	20877677	20890182	20890395	6E-07	1
67	FBgn0051973	chr2L	-	30393	30586	33844	34283	28981	29068	34557	34604	0.01337	-0.176
68	FBgn0051973	chr2L	-	30393	30586	33844	34288	28981	29068	34557	34604	0.00573	-0.162
69	FBgn0051973	chr2L	-	30393	30673	33844	34283	28981	29068	34557	34604	0.01344	-0.175
70	FBgn0051973	chr2L	-	30393	30673	33844	34288	28981	29068	34557	34604	0.00776	-0.162
71	FBgn0051973	chr2L	-	30393	33270	33844	34283	28981	29068	34557	34604	0.00468	-0.177
72	FBgn0036821	chr3L	-	18888123	18888313	18889200	18889406	18887552	18887746	18890189	18890483	0.01213	0.611
73	FBgn0036821	chr3L	-	18888123	18888313	18889200	18889516	18887552	18887746	18890189	18890483	0.02837	0.559
74	FBgn0263995	chr3R	+	18011096	18011255	18012029	18012150	18010227	18010574	18013147	18015779	0.00304	-0.197
75	FBgn0263995	chr3R	+	18011096	18011255	18012104	18012150	18010227	18010574	18013147	18015779	0.00069	-0.223
76	FBgn0263995	chr3R	+	18011096	18011713	18012029	18012150	18010227	18010574	18013147	18014732	0.00165	-0.205
77	FBgn0263995	chr3R	+	18011096	18011713	18012104	18012150	18010227	18010574	18013147	18014732	0.00046	-0.23
78	FBgn0037443	chr3R	-	6397188	6397291	6405373	6405548	6393731	6393911	6405741	6405895	0.00116	-0.464
79	FBgn0033159	chr2R	-	7321366	7321646	7322382	7322397	7319553	7320764	7322559	7322718	0.00854	-0.489
80	FBgn0033159	chr2R	-	7363880	7364337	7365017	7365179	7361717	7361867	7368387	7368732	0.01442	-0.709
81	FBgn0033159	chr2R	-	7363880	7364337	7365311	7365482	7361717	7361867	7368387	7368732	0.04205	-0.633
82	FBgn0000541	chr3L	-	236858	237052	237365	237524	236138	236427	239054	239185	0.02917	-0.466
83	FBgn0010482	chr2R	-	6729999	6730114	6730300	6731266	6728590	6729073	6731439	6731490	0.01016	0.276
84	FBgn0013576	chr3R	-	5336380	5336635	5349829	5350153	5334404	5335060	5350239	5350443	0.00293	0.747
85	FBgn0038063	chr3R	-	12561942	12562049	12562818	12563119	12560400	12561804	12595560	12595725	0.02376	-0.41
86	FBgn0263077	chr2R	+	7637283	7637365	7638122	7638313	7636894	7637193	7638597	7638905	0.04055	-0.378
87	FBgn0023023	chr3R	+	5576099	5576413	5577035	5577259	5575778	5575925	5577592	5577828	0.00917	-0.14
88	FBgn0035049	chr2R	-	24674114	24674182	24674475	24674531	24672550	24672851	24674972	24675141	0.01856	-0.667
89	FBgn0002921	chr3R	+	20959236	20959380	20960725	20960829	20948779	20948829	20963724	20963856	0.00046	0.309
90	FBgn0002921	chr3R	+	20959236	20959380	20960725	20960841	20948779	20948829	20963724	20963856	0.00051	0.469
91	FBgn0002921	chr3R	+	20959236	20959380	20960725	20961113	20948779	20948829	20963724	20963856	2E-05	0.676
92	FBgn0002921	chr3R	+	20966714	20967202	20968391	20968486	20966397	20966613	20973172	20973347	0.00263	-0.152
93	FBgn0027515	chr2L	+	7821362	7821386	7821717	7821892	7821150	7821308	7822641	7823100	0.0254	0.656
94	FBgn0027515	chr2L	+	7821362	7821648	7821717	7821892	7821150	7821308	7822641	7823100	0.02646	0.393
95	FBgn0027515	chr2L	+	7821549	7821648	7821717	7821892	7821150	7821308	7822641	7823100	0.03579	0.341
96	FBgn0038294	chr3R	-	15171935	15172106	15172599	15173062	15170624	15171374	15174136	15174343	0.01337	-0.099
97	FBgn0010263	chr2L	+	2959319	2959520	2960724	2960991	2956910	2957264	2961214	2961672	0.00293	0.667
98	FBgn0020496	chr3R	+	13014368	13014589	13016616	13016793	13011648	13011748	13019341	13019814	0	-0.746
99	FBgn0020496	chr3R	+	13014368	13014589	13016616	13017019	13011648	13011748	13019341	13019814	0.00075	-0.403
100	FBgn0020496	chr3R	+	13014472	13014589	13016616	13016793	13011648	13011748	13019341	13019814	0	-0.727
101	FBgn0020496	chr3R	+	13014472	13014589	13016616	13017019	13011648	13011748	13019341	13019814	0.0015	-0.345
102	FBgn0020496	chr3R	+	13014489	13014589	13016616	13016793	13011648	13011748	13019341	13019814	6.4E-13	-0.753
103	FBgn0020496	chr3R	+	13014489	13014589	13016616	13017019	13011648	13011748	13019341	13019814	0.00066	-0.355
104	FBgn0025726	chr4	-	878840	878992	882960	883251	876869	877194	883566	883641	0.02723	0.445
105	FBgn0025726	chr4	-	878851	878992	882960	883251	876869	877194	883566	883641	0.00351	0.61
106	FBgn0053653	chr4	+	1242191	1242259	1243028	1243108	1241729	1241897	1243476	1243652	0.0391	0.513
107	FBgn0250871	chrX	-	11912650	11912824	11916015	11916456	11912230	11912471	11917068	11917260	0.00573	-0.52
108	FBgn0033402	chr2R	-	9304603	9304728	9304855	9304905	9303677	9304335	9304973	9305160	0.02723	0.538
109	FBgn0261788	chr3L	-	7656374	7661506	7662704	7662890	7655388	7656203	7663258	7663324	6.1E-06	0.188

RI											
	FlyBase ID	chr	strand	riExonStart_0base	riExonEnd	upstreamES	upstreamEE	downstreamES	downstreamEE	FDR	ΔPSI
1	FBgn0285963	chr3R	+	26915760	26916562	26915760	26915908	26916502	26916562	0	-0.987
2	FBgn0284408	chrX	-	2492987	2499678	2492987	2493215	2499210	2499678	0.00135114	-0.39
3	FBgn0267689	chr2L	+	7754173	7754549	7754173	7754394	7754460	7754549	0.03383909	0.241
4	FBgn0267376	chr3R	-	10866808	10868290	10866808	10866960	10867971	10868290	0	0.86
5	FBgn0267376	chr3R	-	10866808	10868642	10866808	10866960	10868438	10868642	0	0.67
6	FBgn0266653	chr3L	+	5135541	5135749	5135541	5135610	5135668	5135749	1.9872E-09	0.838
7	FBgn0266408	chr3R	-	19909180	19910318	19909180	19910165	19910241	19910318	0.00366781	-0.579
8	FBgn0266258	chr3R	-	29755485	29756264	29755485	29755701	29755766	29756264	0.04657765	-0.535
9	FBgn0266219	chr2L	+	6872713	6874198	6872713	6873544	6873937	6874198	0.02180759	0.492
10	FBgn0266101	chr3L	+	9461049	9464706	9461049	9461559	9461887	9464706	1.2801E-07	-0.267
11	FBgn0266019	chrX	+	11818336	11824478	11818336	11818515	11823282	11824478	0.03788857	-0.344
12	FBgn0265413	chrX	-	9072819	9078499	9072819	9073305	9078336	9078499	0.00033057	0.681
13	FBgn0264816	chr3R	+	18403222	18403622	18403222	18403318	18403519	18403622	0.00208704	0.566
14	FBgn0266136	chr3L	-	19789963	19793569	19789963	19790254	19793427	19793569	4.9519E-06	0.656
15	FBgn0263584	chr2L	+	21951	24237	21951	22941	22997	24237	0.02926062	0.448
16	FBgn0263456	chr3L	+	9141786	9143323	9141786	9141843	9143297	9143323	1.7986E-13	0.265
17	FBgn0263048	chr3R	+	22383411	22386548	22383411	22383504	22383561	22386548	0.0223423	-0.594
18	FBgn0262975	chr3R	-	23220416	23221961	23220416	23220643	23221761	23221961	0.03113141	0.184
19	FBgn0262854	chr3L	+	21258019	21258758	21258019	21258058	21258115	21258758	0.01730923	-0.365
20	FBgn0262593	chr3L	+	2895119	2897429	2895119	2895284	2897243	2897429	0	-0.705
21	FBgn0262593	chr3L	+	2896742	2897429	2896742	2896787	2897243	2897429	1.0599E-10	-0.603
22	FBgn0266084	chr3L	+	8796999	8797821	8796999	8797226	8797689	8797821	5.2728E-08	0.452
23	FBgn0266084	chr3L	+	8796999	8798719	8796999	8797226	8798120	8798719	0	0.333
24	FBgn0266084	chr3L	+	8796999	8798719	8796999	8797821	8798120	8798719	0	0.48
25	FBgn0261976	chr2L	-	4386763	4387356	4386763	4387013	4387319	4387356	0.04225734	0.209
26	FBgn0261954	chrX	+	2010859	2018004	2010859	2010960	2014341	2018004	0.00236562	-0.502
27	FBgn0261931	chrX	+	9608945	9612206	9608945	9609691	9609759	9612206	0.00018	0.563
28	FBgn0261873	chrX	+	8216144	8217074	8216144	8216415	8216902	8217074	0.00056424	-0.778
29	FBgn0261859	chr3R	-	15102087	15112189	15102087	15102204	15111592	15112189	1.8963E-08	0.155
30	FBgn0261793	chrX	+	8424096	8427887	8424096	8424312	8424375	8427887	0.0119581	0.667
31	FBgn0261625	chr2R	-	12673898	12675418	12673898	12673928	12674990	12675418	0.00023339	-0.297
32	FBgn0261562	chr3L	+	1803432	1804355	1803432	1803733	1803794	1804355	0.03029625	-0.431
33	FBgn0261113	chr3R	+	18924069	18925796	18924069	18924145	18925077	18925796	5.7112E-05	-0.348
34	FBgn0260745	chr3R	-	11992741	11993315	11992741	11992852	11993248	11993315	0.03476432	0.161
35	FBgn0260745	chr3R	-	11992741	11993315	11992741	11992874	11993248	11993315	0.00177105	0.206
36	FBgn0260642	chr3R	-	6896252	6908738	6896252	6898538	6908512	6908738	0.00085882	0.667

37	FBgn0259878	chr2R	-	15224321	15232559	15224321	15224446	15232310	15232559	0.01393994	0.146
38	FBgn0259203	chr3L	+	7330298	7331336	7330298	7331231	7331296	7331336	7.5218E-05	-0.685
39	FBgn0259179	chr2R	+	22824623	22827333	22824623	22824744	22827261	22827333	0.0047729	-0.594
40	FBgn0259179	chr2R	+	22825092	22827333	22825092	22825250	22827261	22827333	0.00011748	-0.795
41	FBgn0259146	chr3R	+	25970079	25970936	25970079	25970291	25970840	25970936	0.01764742	-0.313
42	FBgn0250910	chr3R	-	12547286	12548229	12547286	12547704	12547910	12548229	0.00011487	0.577
43	FBgn0086711	chr2L	-	14993898	14997559	14993898	14994035	14997515	14997559	2.6249E-07	-0.488
44	FBgn0260479	chr2L	-	7736337	7738809	7736337	7736989	7738618	7738809	1.0088E-09	0.416
45	FBgn0087007	chr3L	-	14412827	14413715	14412827	14413510	14413574	14413715	4.9519E-06	0.148
46	FBgn0082831	chr3R	+	12414351	12416582	12414351	12414756	12414870	12416582	0	-0.393
47	FBgn0063368	chr2L	+	23311747	23312464	23311747	23311800	23311860	23312464	0.04093351	-0.233
48	FBgn0265991	chr2R	-	15765122	15773253	15765122	15765252	15773040	15773253	4.7356E-07	0.148
49	FBgn0265991	chr2R	-	15765122	15773253	15765122	15766713	15773040	15773253	0.00056719	-0.317
50	FBgn0265991	chr2R	-	15765122	15773966	15765122	15765252	15773815	15773966	2.7546E-09	0.324
51	FBgn0260722	chr3R	-	9548103	9551748	9548103	9549810	9551700	9551748	0.00057698	0.459
52	FBgn0053093	chr3R	-	22530693	22531013	22530693	22530807	22530870	22531013	0.00120377	0.287
53	FBgn0053061	chr3L	+	16329794	16330522	16329794	16330144	16330249	16330522	0.0010871	-0.702
54	FBgn0259740	chr3R	+	10851284	10851788	10851284	10851488	10851540	10851788	0	-0.494
55	FBgn0259740	chr3R	+	10851284	10851788	10851284	10851488	10851548	10851788	0.02335448	-0.175
56	FBgn0052373	chr3L	+	7737112	7757179	7737112	7737199	7756982	7757179	0.03220746	0.488
57	FBgn0052264	chr3L	-	3769241	3794950	3769241	3769406	3794516	3794950	0	-0.909
58	FBgn0051975	chr2L	-	139441	139954	139441	139687	139740	139954	2.0166E-09	0.69
59	FBgn0051975	chr2L	-	139740	140992	139740	139954	140113	140992	1.3034E-06	0.637
60	FBgn0051324	chr3R	+	26106867	26124657	26106867	26107342	26124434	26124657	0	-0.55
61	FBgn0051300	chr3R	+	25283619	25284835	25283619	25284245	25284306	25284835	0.00051109	-0.47
62	FBgn0051189	chr3R	-	21045200	21045842	21045200	21045684	21045741	21045842	0.00287437	0.123
63	FBgn0051431	chr3R	+	21870680	21872140	21870680	21871007	21871971	21872140	0.01358978	0.484
64	FBgn0051029	chr3R	-	30130457	30132395	30130457	30131717	30131779	30132395	0.04723588	0.602
65	FBgn0050286	chr2R	-	21535715	21536527	21535715	21536423	21536481	21536527	1.7816E-06	0.695
66	FBgn0083123	chr2R	+	14623686	14623978	14623686	14623731	14623919	14623978	0.00014498	0.399
67	FBgn0050021	chr2R	-	11228508	11229033	11228508	11228676	11229003	11229033	2.4858E-06	-0.199
68	FBgn0050021	chr2R	-	11229003	11229542	11229003	11229033	11229410	11229542	5.0225E-05	-0.262
69	FBgn0052194	chr3L	-	18085304	18087058	18085304	18086961	18087036	18087058	0.01960141	0.509
70	FBgn0260388	chr3L	-	16492170	16493000	16492170	16492499	16492661	16493000	5.4246E-05	0.107
71	FBgn0260388	chr3L	-	16495299	16496236	16495299	16495536	16496052	16496236	4.4882E-12	-0.725
72	FBgn0040056	chr3L	+	23748139	23748716	23748139	23748373	23748423	23748716	0.02859502	0.114
73	FBgn0265959	chr3L	+	20248627	20256638	20248627	20249060	20255897	20256638	0.04432347	0.384
74	FBgn0031837	chr2L	-	6617424	6618115	6617424	6617706	6617759	6618115	0.01208457	-0.777
75	FBgn0031816	chr2L	-	6463261	6465669	6463261	6464139	6465528	6465669	0.00047505	-0.236
76	FBgn0032603	chr2L	+	16791567	16791869	16791567	16791707	16791763	16791869	2.9217E-12	0.401
77	FBgn0031650	chr2L	+	4943347	4943988	4943347	4943748	4943802	4943988	1.8649E-05	0.459
78	FBgn0030268	chrX	-	11134879	11136778	11134879	11136095	11136618	11136778	0.04374296	0.401
79	FBgn0031914	chr2L	+	7440793	7443832	7440793	7440958	7443583	7443832	0	-0.708
80	FBgn0031914	chr2L	+	7440793	7443832	7440793	7440962	7443583	7443832	1.6592E-10	-0.587
81	FBgn0031142	chrX	-	21024399	21025888	21024399	21024698	21024788	21025888	0.02458599	0.417
82	FBgn0013718	chr3L	+	14219741	14226403	14219741	14219936	14225430	14226403	0.01112225	0.17
83	FBgn0039858	chr3R	-	31601415	31610978	31601415	31603073	31610880	31610978	5.7727E-05	-0.339
84	FBgn0030795	chrX	+	16806633	16807957	16806633	16807300	16807362	16807957	0.03070352	0.335
85	FBgn0030912	chrX	-	18315016	18315901	18315016	18315336	18315581	18315901	0.02974855	0.667
86	FBgn0030437	chrX	-	12953532	12954583	12953532	12953833	12954364	12954583	0.02595806	0.222
87	FBgn0027375	chr3L	-	14629740	14631818	14629740	14630444	14630519	14631818	0.00071143	-0.68
88	FBgn0004381	chr3L	+	11795613	11796621	11795613	11795698	11795857	11796621	0.0032516	-0.229
89	FBgn0263995	chr3R	+	18010227	18015779	18010227	18010574	18013149	18015779	0.02731674	-0.326
90	FBgn0266801	chr3R	-	8382723	8390010	8382723	8388800	8389976	8390010	1.8963E-08	0.194
91	FBgn0040370	chrX	-	290252	294962	290252	290596	294822	294962	0.01222065	0.229
92	FBgn0039602	chr3R	+	28820220	28820718	28820220	28820505	28820571	28820718	0.0002442	-0.732
93	FBgn0010235	chr3L	-	12748055	12749839	12748055	12748682	12749709	12749839	0.00328171	-0.357
94	FBgn0031392	chr2L	+	2152232	2152944	2152232	2152465	2152662	2152944	0.00167106	-0.608
95	FBgn0028387	chr2L	+	7417372	7417813	7417372	7417478	7417546	7417813	0	-0.918
96	FBgn0264489	chr3L	+	9830999	9832679	9830999	9831440	9832195	9832679	0.00012126	0.398
97	FBgn0264489	chr3L	+	9830633	9832679	9830633	9830785	9832198	9832679	0	-0.894
98	FBgn0264489	chr3L	+	9830999	9832679	9830999	9831440	9832198	9832679	0	-0.447
99	FBgn0031170	chrX	-	21298345	21300435	21298345	21298934	21300150	21300435	0.01341856	0.328
100	FBgn0028699	chr3L	+	4918758	4919399	4918758	4918861	4919100	4919399	3.1447E-05	0.204
101	FBgn0027505	chr2L	-	12058303	12059027	12058303	12058763	12058829	12059027	0.02926062	0.618
102	FBgn0032821	chr2L	-	19776256	19778946	19776256	19777014	19777081	19778946	0.01013485	-0.617
103	FBgn0039431	chr3R	-	26532112	26535217	26532112	26532422	26535158	26535217	0.02561314	-0.447
104	FBgn0261788	chr3L	-	7655388	7662298	7655388	7656203	7661980	7662298	0.01810673	0.249
105	FBgn0261260	chrX	-	9471998	9500129	9471998	9473266	9499885	9500129	0.00077894	0.682
106	FBgn0062413	chrX	+	6663663	6665078	6663663	6663761	6664999	6665078	0.01517264	-0.65
107	FBgn0003742	chr2R	-	14603733	14604123	14603733	14603925	14603983	14604123	0.04225734	0.34
108	FBgn0003174	chr2R	+	7298816	7299842	7298816	7299485	7299684	7299842	0.04240325	0.291
109	FBgn0005677	chr2L	-	16475274	16484807	16475274	16475432	16483807	16484807	0.03881839	-0.417
110	FBgn0033603	chr2R	-	11268078	11268341	11268078	11268135	11268284	11268341	0.02506771	0.619
111	FBgn0260794	chr3R	-	4793217	4800678	4793217	4795064	4799691	4800678	0.04723588	0.177
112	FBgn0039271	chr3R	+	25069926	25070914	25069926	25070050	25070202	25070914	0.00854279	0.219
113	FBgn0027779	chr2L	-	16724858	16726113	16724858	16724993	16725891	16726113	0.01086485	-0.238
114	FBgn0031515	chr2L	-	3292173	3292576	3292173	3292298	3292459	3292576	0.02983651	0.149
115	FBgn0031515	chr2L	-	3292173	3292576	3292173	3292301	3292459	3292576	8.7347E-05	0.305
116	FBgn0031515	chr2L	-	3293322	3294706	3293322	3293949	3294082	3294706	0.00193906	0.271
117	FBgn0025388	chrX	-	4631051	4631962	4631051	4631730	4631813	4631962	0.03659845	-0.748
118	FBgn0042135	chr2R	-	7725688	7725995	7725688	7725771	7725867	7725995	0.02513823	-0.178
119	FBgn0042135	chr2R	-	7725867	7726430	7725867	7725995	7726294	7726430	0.02255749	0.293
120	FBgn0033195	chr2R	-	7589594	7590109	7589594	7589708	7589933	7590109	5.7765E-11	-0.962
121	FBgn0000139	chr3R	+	24651179	24652241	24651179	24651251	24651860	24652241	0.01016046	0.433
122	FBgn0033408	chr2R	-	9395752	9396206	9395752	9395864	9395928	9396206	3.0131E-08	-0.926
123	FBgn0000256	chr2L	-	3886983	3888824	3886983	3887556	3888648	3888824	1.5558E-06	0.325
124	FBgn0000256	chr2L	-	3886983	3890031	3886983	3887556	3889430	3890031	1.2202E-06	0.35
125	FBgn0000256	chr2L	-	3884035	3893604	3884035	3884518	3893261	3893604	4.9912E-13	1
126	FBgn0000256	chr2L	-	3889430	3893604	3889430	3889784	3893261	3893604	1.6511E-10	0.36
127	FBgn0038846	chr3R	+	21015948	21016427	21015948	21016008	21016219	21016427	1.1122E-06	-0.538
128	FBgn0038846	chr3R	+	21015948	21016427	21015948	21016107	21016219	21016427	0	-0.912
129	FBgn0010225	chr3R	+	4235216	4236148	4235216	4235324	4236087	4236148	6.0018E-09	0.673
130	FBgn0000228	chr2L	-	5276935	5278548	5276935	5277180	5277957	5278548	0.00142542	-0.339
131	FBgn0037993	chr3R	-	12156108	12158595	12156108	12156621	12158533	12158595	0.00640968	-0.425
132	FBgn0036732	chr3L	+	17487117	17488332	17487117	17487189	17488064	17488332	0.008698	0.433
133	FBgn0000723	chr3R	-	9369335	9372577	9369335	9369710	9372376	9372577	2.0166E-06	-0.391
134	FBgn0038397	chr3R	-	16065675	16066437	16065675	16066117	16066211	16066437	0.01774596	-0.567
135	FBgn0031857	chr2L	-	6770590	6772614	6770590	6770709	6772551	6772614	0	-0.931
136	FBgn0039780	chr3R	-	30499035	30500326	30499035	30499190	30500043	30500326	0.00016541	0.82
137	FBgn0003401	chr2R	-	23841460	23843078	23841460	23842252	23842327	23843078	0.00081354	0.638
138	FBgn0016081	chr3L	-	9631833	9632599	9631833	9632173	9632232	9632599	0.0192262	-0.192
139	FBgn0031769	chr2L	+	6050799	6052022	6050799	6050921	6051616	6052022	1.9573E-09	-0.934
140	FBgn0031688	chr2L	+	5207657	5207965	5207657	5207796	5207857	5207965	0.00244101	-0.657
141	FBgn0031879	chr2L	-	7008399	7009909	7008399	7008646	7009872	7009909	0.0108807	0.516
142	FBgn0038098	chr3R	-	12973576	12974450	12973576	12973844	12974288	12974450	0.03858467	-0.465
143	FBgn0030319	chrX	-	11603242	11604194	11603242	11603894	11604074	11604194	1.2585E-10	-0.762
144	FBgn0026418	chr3L	+	14037018	14037750	14037018	14037111	14037477	14037750	1.4801E-07	0.647
145	FBgn0026418	chr3L	+	14037018	14037750	14037018	14037136	14037477	14037750	1.1886E-07	0.635
146	FBgn0035815	chr3L	-	7738106	7738482	7738106	7738244	7738334	7738482	0.00053012	-0.365
147	FBgn0038858	chr3R	+	21127922	21128217	21127922	21128054	21128120	21128217	0.00250652	0.098

148	FBgn0023083	chr3R	+	18573090	18589453	18573090	18573525	18586278	18589453	0.02691996	-0.56
149	FBgn0003353	chr2R	+	24047615	24050297	24047615	24048003	24049422	24050297	0.00694069	0.519
150	FBgn0001991	chr2L	+	16170258	16173248	16170258	16171048	16171971	16173248	1.272E-05	0.157
151	FBgn0001991	chr2L	+	16170258	16173248	16170258	16171144	16171971	16173248	2.1007E-10	0.365
152	FBgn0003149	chr3L	-	8738639	8738907	8738639	8738747	8738816	8738907	0.00759364	-0.533
153	FBgn0002945	chr3L	-	19003740	19008254	19003740	19005991	19007430	19008254	0.00023909	-0.603
154	FBgn0037448	chr3R	+	6480861	6481251	6480861	6480927	6480996	6481251	7.5218E-05	-0.442
155	FBgn0052677	chrX	+	10627736	10631808	10627736	10627889	10631388	10631808	0.01352736	0.374
156	FBgn0033504	chr2R	+	10271883	10293761	10271883	10272080	10293621	10293761	0.04290899	0.411
157	FBgn0033504	chr2R	+	10296434	10304930	10296434	10300268	10304623	10304930	0.02754638	-0.321
158	FBgn0010348	chr3L	-	22870214	22872052	22870214	22870294	22871923	22872052	0.01085428	-0.222
159	FBgn0040309	chrX	+	13329823	13331994	13329823	13330030	13331121	13331994	0.03116786	-0.248
160	FBgn0011640	chr3L	+	7339463	7340770	7339463	7339617	7339961	7340770	1.9228E-08	-0.307
161	FBgn0034417	chr2R	-	19124536	19127219	19124536	19124940	19127191	19127219	0.00529358	-0.695
162	FBgn0001208	chr3L	+	7760983	7762235	7760983	7761277	7761562	7762235	0.00651874	0.233
163	FBgn0016694	chr3L	-	7818068	7827287	7818068	7818223	7825583	7827287	0.04252921	0.22
164	FBgn0015949	chr2R	-	19668353	19669832	19668353	19668478	19669117	19669832	0.03999246	-0.7
165	FBgn0038199	chr3R	-	14092503	14093183	14092503	14093037	14093136	14093183	0.01224428	-0.288
166	FBgn0033639	chr2R	+	11650814	11651616	11650814	11651224	11651323	11651616	0	-0.457
167	FBgn0036685	chr3L	-	17015140	17016051	17015140	17015258	17015672	17016051	0.04768233	-0.25
168	FBgn0013759	chr3R	-	21807598	21808689	21807598	21807734	21808616	21808689	0	-0.858
169	FBgn0013995	chr3R	+	20989365	20994423	20989365	20989599	20992240	20994423	0.03087323	0.168
170	FBgn0015037	chr2R	+	9240603	9240842	9240603	9240685	9240741	9240842	0	-0.894
171	FBgn0038256	chr3R	-	14730510	14731509	14730510	14730629	14731404	14731509	5.6227E-11	-0.422
172	FBgn0033159	chr2R	-	7332260	7345775	7332260	7332380	7345484	7345775	0.01456095	0.365
173	FBgn0033159	chr2R	-	7345060	7348010	7345060	7345351	7347848	7348010	0.00202018	-0.433
174	FBgn0033159	chr2R	-	7353815	7361867	7353815	7353936	7361717	7361867	0.01778398	0.818
175	FBgn0033159	chr2R	-	7361717	7365179	7361717	7361867	7365017	7365179	0.00680699	0.6
176	FBgn0033159	chr2R	-	7361717	7365482	7361717	7361867	7365311	7365482	0.03113423	0.456
177	FBgn0033159	chr2R	-	7364455	7368732	7364455	7364617	7368387	7368732	3.5026E-13	0.597
178	FBgn0015793	chr3L	+	8297814	8298506	8297814	8298071	8298119	8298506	0.03139654	0.102
179	FBgn0263656	chr2R	+	7020132	7020396	7020132	7020235	7020295	7020396	0.00221077	-0.504
180	FBgn0020647	chr3R	-	24030493	24031827	24030493	24031031	24031711	24031827	5.9503E-11	0.447
181	FBgn0003943	chr3L	-	3899258	3902050	3899258	3901770	3901852	3902050	0.0408275	-0.47
182	FBgn0032705	chr2L	+	18709543	18711297	18709543	18710385	18710440	18711297	0.02506771	-0.514
183	FBgn0003984	chr3L	-	5813337	5816700	5813337	5813474	5816650	5816700	0.00292217	-0.24
184	FBgn0023023	chr3R	+	5576174	5577828	5576174	5576413	5577592	5577828	0.00847521	0.202
185	FBgn0023096	chr2L	-	17970164	17971284	17970164	17970281	17970356	17971284	0.04340497	0.29
186	FBgn0262508	chr3L	+	8581762	8586956	8581762	8581855	8585970	8586956	8.9072E-08	-0.873
187	FBgn0020370	chr2R	-	13156767	13157436	13156767	13156830	13156886	13157436	0.0428803	0.446
188	FBgn0050197	chr2R	+	14514029	14514215	14514029	14514084	14514152	14514215	0.00372071	0.078
189	FBgn0035046	chr2R	+	24664422	24664679	24664422	24664456	24664515	24664679	0.02510545	0.293
190	FBgn0036768	chr3L	+	17887704	17889462	17887704	17887790	17889330	17889462	0	0.859
191	FBgn0024963	chr3R	+	19766489	19775231	19766489	19766647	19774599	19775231	0.01888643	0.336
192	FBgn0039406	chr3R	-	26058979	26059267	26058979	26059051	26059234	26059267	0.00583791	0.444
193	FBgn0040251	chr3R	-	11153233	11153849	11153233	11153493	11153558	11153849	0.02191241	0.257
194	FBgn0025681	chr2L	+	2997491	2998726	2997491	2997530	2997790	2998726	0.02202766	0.156
195	FBgn0032878	chr2L	-	20724208	20725047	20724208	20724911	20724971	20725047	0.0002298	-0.869
196	FBgn0035539	chr3L	+	4384441	4391221	4384441	4384627	4390449	4391221	0.03895168	0.165
197	FBgn0085285	chr3L	-	18988735	18989533	18988735	18989217	18989466	18989533	1.1176E-06	0.368
198	FBgn0033170	chr2R	+	7443042	7443827	7443042	7443382	7443442	7443827	3.0474E-05	0.782
199	FBgn0263780	chr2R	-	1866864	1926681	1866864	1867050	1926294	1926681	0.01558228	0.215
200	FBgn0265977	chr2R	-	22318994	22320895	22318994	22320622	22320781	22320895	7.4335E-07	0.322
201	FBgn0086904	chr2R	-	12759692	12760836	12759692	12760521	12760800	12760836	0.01661385	0.259
202	FBgn0086904	chr2R	-	12759692	12760999	12759692	12760521	12760804	12760999	0.00635152	0.201
203	FBgn0033518	chr2R	+	10418717	10419324	10418717	10418887	10418949	10419324	2.3432E-05	0.128
204	FBgn0033518	chr2R	+	10419387	10419761	10419387	10419463	10419526	10419761	1.2326E-06	0.135
205	FBgn0033609	chr2R	-	11287644	11289772	11287644	11288560	11289067	11289772	0.01214723	-0.509
206	FBgn0033609	chr2R	-	11287644	11289772	11287644	11288560	11289669	11289772	0.04723588	0.292
207	FBgn0050274	chr2R	+	22825481	22827333	22825481	22826987	22827261	22827333	4.2781E-12	-1
208	FBgn0034087	chr2R	+	16187888	16189664	16187888	16188154	16188577	16189664	0.00459723	0.667
209	FBgn0040606	chr3R	-	26424697	26425102	26424697	26425019	26425076	26425102	0.00012968	0.345
210	FBgn0035515	chr3L	+	4240172	4240523	4240172	4240274	4240338	4240523	0.0310328	0.553
211	FBgn0038435	chr3R	+	16472600	16475477	16472600	16472755	16473358	16475477	0.00827963	-0.447
212	FBgn0036538	chr3L	-	15837091	15837482	15837091	15837128	15837256	15837482	0.0248231	0.416
213	FBgn0053654	chr3R	-	9582514	9582872	9582514	9582670	9582725	9582872	0.01323514	0.51
214	FBgn0033853	chr2R	-	13538971	13542563	13538971	13539349	13542160	13542563	0.00139816	-0.101
215	FBgn0033853	chr2R	-	13538971	13547012	13538971	13539349	13546514	13547012	6.2598E-10	-0.27
216	FBgn0033853	chr2R	-	13538971	13547577	13538971	13539349	13547449	13547577	0.01810673	-0.411
217	FBgn0033942	chr2R	-	14512558	14515211	14512558	14513066	14515068	14515211	2.6737E-06	0.363
218	FBgn0031937	chr2L	-	7724172	7724517	7724172	7724301	7724368	7724517	0.02939064	0.211
219	FBgn0033983	chr2R	-	14932854	14934861	14932854	14933003	14934800	14934861	0.02311627	0.182
220	FBgn0034175	chr2R	-	16998609	17000126	16998609	16999944	16999997	17000126	0.0050168	0.667
221	FBgn0034382	chr2R	-	18742516	18743485	18742516	18743238	18743300	18743485	2.9947E-11	-1
222	FBgn0034493	chr2R	+	20265776	20267521	20265776	20266919	20266968	20267521	0.01832954	-0.617
223	FBgn0034530	chr2R	-	20630683	20633506	20630683	20631114	20632789	20633506	0.00815588	0.401
224	FBgn0035771	chr3L	+	7361743	7363770	7361743	7362453	7362521	7363770	0.0016195	-0.094
225	FBgn0051523	chr3R	-	4468463	4473188	4468463	4468599	4472840	4473188	0.0316905	-0.267
226	FBgn0034657	chr2R	+	21720733	21721327	21720733	21720874	21720947	21721327	0.00080055	0.765
227	FBgn0038221	chr3R	+	14302427	14302772	14302427	14302590	14302654	14302772	5.7112E-05	-0.665
228	FBgn0261617	chrX	-	9682551	9685667	9682551	9684151	9685549	9685667	0.01224428	-0.367
229	FBgn0031690	chr2L	-	5212714	5213332	5212714	5212796	5212862	5213332	0.00490149	-0.369
230	FBgn0035936	chr3L	+	8912498	8913052	8912498	8912596	8912968	8913052	0.0009243	-0.256
231	FBgn0034903	chr2R	-	23753713	23757166	23753713	23754337	23756363	23757166	5.8987E-12	-0.281
232	FBgn0034935	chr2R	-	23947758	23948397	23947758	23948195	23948351	23948397	1.0599E-10	-0.867
233	FBgn0051183	chr3R	-	15869172	15875939	15869172	15870232	15875577	15875939	7.0986E-12	-0.554
234	FBgn0051097	chr3R	-	25296599	25297520	25296599	25296969	25297023	25297520	1.7593E-06	-0.719
235	FBgn0039637	chr3R	+	29123415	29124104	29123415	29123499	29123800	29124104	0.01392793	-0.627
236	FBgn0266183	chrX	+	19712933	19713821	19712933	19713296	19713352	19713821	0.01180454	0.686
237	FBgn0035755	chr3L	-	7253806	7263095	7253806	7253957	7262948	7263095	0.01934428	0.351
238	FBgn0035755	chr3L	-	7262948	7263920	7262948	7263095	7263631	7263920	3.0727E-06	-0.51
239	FBgn0035766	chr3L	-	7335441	7338988	7335441	7335875	7335937	7338988	0.00713022	-0.185
240	FBgn0035769	chr3L	-	7354041	7356459	7354041	7356005	7356066	7356459	0	-0.512
241	FBgn0259822	chr2L	-	11159879	11162525	11159879	11160336	11162261	11162525	3.5635E-06	0.199
242	FBgn0035888	chr3L	+	8395999	8399046	8395999	8396185	8398986	8399046	4.229E-09	0.37
243	FBgn0035888	chr3L	+	8398085	8399046	8398085	8398364	8398986	8399046	0.0270003	0.31
244	FBgn0035888	chr3L	+	8399658	8400689	8399658	8399943	8400081	8400689	0.02335448	0.279
245	FBgn0036624	chr3L	+	16386326	16386956	16386326	16386494	16386625	16386956	0.01085428	0.373
246	FBgn0035959	chr3L	-	9069648	9071521	9069648	9070644	9070704	9071521	7.9321E-07	-0.751
247	FBgn0035976	chr3L	+	9346590	9347249	9346590	9346745	9346807	9347249	0.00091935	-0.245
248	FBgn0036031	chr3L	-	9701690	9703470	9701690	9702630	9702681	9703470	0.00011879	-0.79
249	FBgn0266186	chr2R	-	9841846	9842502	9841846	9842225	9842300	9842502	0.04448534	0.205
250	FBgn0036117	chr3L	-	11059436	11060721	11059436	11060349	11060419	11060721	0.0003321	0.218
251	FBgn0036290	chr3L	-	12500211	12500800	12500211	12500554	12500630	12500800	0.00579835	-0.433
252	FBgn0036305	chr3L	+	12750230	12750739	12750230	12750344	12750603	12750739	0.00619392	-0.291
253	FBgn0036305	chr3L	+	12750507	12752922	12750507	12750739	12751306	12752922	0	0.971
254	FBgn0036661	chr3L	+	16780099	16780790	16780099	16780420	16780504	16780790	0	0.325
255	FBgn0036360	chr3L	-	13430278	13435229	13430278	13430963	13434741	13435229	2.3897E-08	-0.63
256	FBgn0036433	chr3L	+	14622795	14626894	14622795	14622935	14626840	14626894	5.4423E-06	-0.167
257	FBgn0041150	chr2L	-	4925828	4928393	4925828	4926346	4928227	4928393	7.9783E-05	-0.421
258	FBgn0031885	chr2L	+	7056300	7057674	7056300	7056536	7057347	7057674	0.02926062	-0.127

259	FBgn0052698	chrX	+	9788465	9812156	9788465	9788629	9811787	9812156	0.01124835	-0.589
260	FBgn0036569	chr3L	+	16100859	16102310	16100859	16101064	16101116	16102310	0.02145823	0.35
261	FBgn0267390	chr3L	+	15566012	15568970	15566012	15566153	15568205	15568970	4.9912E-13	0.344
262	FBgn0262607	chrX	-	18129584	18130178	18129584	18130077	18130130	18130178	0.0050168	0.667
263	FBgn0037027	chr3L	+	20810980	20812197	20810980	20811450	20811529	20812197	0.00292217	-0.425
264	FBgn0052451	chr3L	+	22773535	22778824	22773535	22773716	22777110	22778824	0.00190777	0.283
265	FBgn0261553	chr3L	+	11215778	11217282	11215778	11215951	11216585	11217282	6.7272E-05	-0.47
266	FBgn0264953	chr2L	+	8180195	8181320	8180195	8180860	8181058	8181320	1.0909E-08	0.499
267	FBgn0037368	chr3R	+	5733512	5734432	5733512	5733616	5733685	5734432	0.00815588	-0.518
268	FBgn0037483	chr3R	+	7202782	7203362	7202782	7202940	7202997	7203362	8.7347E-05	-0.807
269	FBgn0037488	chr3R	+	7241759	7242674	7241759	7241903	7242627	7242674	0.00154587	-0.55
270	FBgn0037520	chr3R	-	7802830	7804048	7802830	7803220	7803280	7804048	0.00016136	-0.826
271	FBgn0037569	chr3R	-	8307472	8308430	8307472	8307840	8307895	8308430	0.00403758	-0.302
272	FBgn0035772	chr3L	-	7364583	7366516	7364583	7364964	7366441	7366516	0.0471161	-0.173
273	FBgn0035772	chr3L	-	7366441	7367984	7366441	7366516	7367558	7367984	0	-0.448
274	FBgn0039709	chr3R	+	29841656	29845760	29841656	29841790	29845598	29845760	0.00178649	0.275
275	FBgn0039897	chr4	+	240644	243585	240644	240833	243265	243585	0.0443459	0.184
276	FBgn0038545	chr3R	+	17795074	17795639	17795074	17795195	17795360	17795639	0.00372071	0.255
277	FBgn0024187	chr3L	-	7927487	7927820	7927487	7927579	7927759	7927820	0.02510545	-0.297
278	FBgn0038894	chr3R	+	21665838	21666676	21665838	21666136	21666252	21666676	0.00422966	0.814
279	FBgn0038909	chr3R	-	21866766	21867744	21866766	21867264	21867404	21867744	0.00197094	0.667
280	FBgn0039593	chr3R	+	28670837	28672021	28670837	28671286	28671348	28672021	0	0.35
281	FBgn0038294	chr3R	-	15172599	15174343	15172599	15172830	15173980	15174343	0	0.29
282	FBgn0038294	chr3R	-	15172843	15174343	15172843	15173063	15174136	15174343	0	-0.338
283	FBgn0260440	chr3R	+	30476683	30478849	30476683	30478174	30478253	30478849	0.00467132	0.406
284	FBgn0039349	chr3R	-	25626343	25626924	25626343	25626645	25626742	25626924	0.04621769	-0.422
285	FBgn0039358	chr3R	+	25682355	25683780	25682355	25682431	25683472	25683780	3.6977E-07	-0.208
286	FBgn0039360	chr3R	-	25686314	25687149	25686314	25686866	25686945	25687149	6.6549E-11	-0.806
287	FBgn0266377	chr2R	+	23669106	23672088	23669106	23669367	23671975	23672088	0.00197695	0.466
288	FBgn0039616	chr3R	+	28923043	28923417	28923043	28923211	28923265	28923417	0.0026969	0.667
289	FBgn0004395	chr3R	-	23154844	23156011	23154844	23154970	23155808	23156011	0.00010116	0.479
290	FBgn0039790	chr3R	-	30595389	30601489	30595389	30595621	30601200	30601489	0.00892783	-0.326
291	FBgn0265380	chr3R	+	26260901	26261515	26260901	26261113	26261177	26261515	4.285E-05	-0.278
292	FBgn0040337	chrX	+	1026790	1028290	1026790	1027546	1027634	1028290	0.00131148	-0.218
293	FBgn0050456	chr2R	+	17100240	17103687	17100240	17100388	17102511	17103687	0.01456095	-0.291
294	FBgn0041210	chrX	-	13276434	13285632	13276434	13276547	13284891	13285632	9.5483E-12	0.889
295	FBgn0051051	chr3R	-	28519547	28520284	28519547	28520006	28520050	28520284	0.00202018	-0.619
296	FBgn0042178	chr3L	+	16598195	16599661	16598195	16599318	16599443	16599661	2.0111E-06	1
297	FBgn0263199	chr3L	-	8985418	8986091	8985418	8985667	8985976	8986091	2.7982E-07	0.708
298	FBgn0265045	chr2R	-	15951157	15952088	15951157	15951466	15951953	15952088	2.3701E-06	0.822
299	FBgn0264574	chr3L	+	959659	967537	959659	959970	967308	967537	0.00177105	0.667
300	FBgn0264855	chr2L	-	413395	414777	413395	413654	414464	414777	5.9283E-07	0.833
301	FBgn0263231	chr3R	-	8655986	8658387	8655986	8657843	8657933	8658387	0.02453322	0.484
302	FBgn0263111	chrX	-	11939920	11943112	11939920	11940042	11943075	11943112	0.00132082	-0.316
303	FBgn0264324	chr3R	+	28845299	28846000	28845299	28845409	28845906	28846000	0.00190777	0.176
304	FBgn0262636	chr4	-	386906	388322	386906	386984	387923	388322	0.0002159	-0.834
305	FBgn0266084	chr3L	+	8796999	8797821	8796999	8797226	8797336	8797821	0.0003321	0.658
306	FBgn0261243	chr3L	+	1517850	1518796	1517850	1517956	1518302	1518796	0.00982944	-0.203
307	FBgn0260943	chr3L	-	17235351	17238212	17235351	17236062	17237885	17238212	1.7986E-13	0.654
308	FBgn0260943	chr3L	-	17235351	17238212	17235351	17236062	17237949	17238212	0.00224168	-0.667
309	FBgn0260941	chr3L	-	12264620	12266841	12264620	12264883	12266654	12266841	7.4272E-08	-0.569
310	FBgn0260660	chr3L	+	7035213	7035881	7035213	7035315	7035744	7035881	0.02859502	-0.734
311	FBgn0260463	chr3R	-	9691164	9692504	9691164	9691475	9692009	9692504	5.5547E-10	-0.537
312	FBgn0243486	chr2L	+	18062361	18063932	18062361	18062562	18063781	18063932	0.0049205	0.6
313	FBgn0085427	chr2L	+	8898785	8916053	8898785	8898850	8915554	8916053	0.025049	-0.282
314	FBgn0085408	chr2R	+	14308141	14315533	14308141	14308293	14315104	14315533	0	0.902
315	FBgn0053988	chr2R	-	24725338	24736657	24725338	24727159	24736438	24736657	0.04217989	-0.482
316	FBgn0053556	chr3L	+	7117317	7117772	7117317	7117536	7117598	7117772	9.4298E-06	0.514
317	FBgn0053113	chr2L	-	4994786	4995482	4994786	4994994	4995154	4995482	0.00976921	0.272
318	FBgn0052944	chr3R	-	4415682	4419578	4415682	4415886	4419359	4419578	0.00056719	0.43
319	FBgn0052112	chr3L	+	12742904	12743277	12742904	12743172	12743237	12743277	0	-0.892
320	FBgn0052056	chr3L	-	10223867	10225240	10223867	10224224	10224859	10225240	0.00065174	0.641
321	FBgn0051935	chr2L	-	1595891	1609658	1595891	1596421	1609184	1609658	0.00039555	0.513
322	FBgn0051217	chr3R	-	16654462	16655662	16654462	16654897	16655439	16655662	0.0037855	-0.606
323	FBgn0051189	chr3R	-	21045200	21045842	21045200	21045591	21045741	21045842	0	0.885
324	FBgn0050344	chr2R	+	9146862	9147301	9146862	9147046	9147131	9147301	4.7931E-05	-0.399
325	FBgn0032123	chr2L	+	9521209	9533751	9521209	9521694	9523162	9533751	1.1886E-07	-0.56
326	FBgn0031942	chr2L	-	7752169	7753153	7752169	7752814	7752989	7753153	4.4316E-08	-0.298
327	FBgn0262149	chr3R	+	7128463	7129067	7128463	7128647	7128940	7129067	0.00557972	0.399
328	FBgn0002772	chr3R	-	27657562	27658555	27657562	27657953	27658066	27658555	0.03895168	0.285
329	FBgn0031759	chr2L	-	5995028	5999501	5995028	5996531	5999224	5999501	0.00388325	-0.413
330	FBgn0034271	chr2R	+	17739900	17740357	17739900	17740044	17740205	17740357	1.7986E-13	-1
331	FBgn0031771	chr2L	-	6062078	6062720	6062078	6062435	6062583	6062720	0.01865078	-0.383
332	FBgn0035989	chr3L	-	9415407	9417814	9415407	9415466	9417628	9417814	0.02335448	-0.429
333	FBgn0261436	chr3R	-	4228479	4228988	4228479	4228634	4228745	4228988	0.0002767	0.267
334	FBgn0035756	chr3L	-	7311928	7312896	7311928	7312230	7312713	7312896	0.00174499	0.417
335	FBgn0031689	chr2L	+	5210953	5211821	5210953	5211061	5211601	5211821	0.00361326	-0.359
336	FBgn0029746	chrX	+	5085758	5090145	5085758	5086201	5090015	5090145	0.03087323	-0.344
337	FBgn0005672	chr2L	-	19571571	19572610	19571571	19571767	19572142	19572610	0.00947294	-0.543
338	FBgn0036134	chr3L	-	11117304	11120411	11117304	11118065	11120111	11120411	0.04851568	0.55
339	FBgn0005614	chr2R	+	9763788	9764321	9763788	9763953	9764104	9764321	2.5628E-11	-0.709
340	FBgn0264489	chr3L	+	9830999	9832679	9830999	9831440	9832531	9832679	1.7986E-13	0.646
341	FBgn0039431	chr3R	-	26532112	26535217	26532112	26532422	26535102	26535217	0.00185169	-0.65
342	FBgn0000721	chr2L	-	3655269	3656953	3655269	3655585	3656593	3656953	1.1182E-05	-0.834
343	FBgn0030706	chrX	+	15914605	15916689	15914605	15916071	15916475	15916689	0.00179705	0.767
344	FBgn0036843	chr3L	+	19063164	19064595	19063164	19063716	19064269	19064595	0.00292217	0.627
345	FBgn0026255	chr2L	+	21210110	21211081	21210110	21210541	21210802	21211081	0.01224428	0.491
346	FBgn0004648	chrX	+	499957	502220	499957	500608	501988	502220	3.7674E-05	-0.395
347	FBgn0024321	chr3R	-	14367192	14369817	14367192	14367496	14369792	14369817	0.00028549	0.575
348	FBgn0030521	chrX	-	13781472	13782170	13781472	13781733	13781839	13782170	0.0248231	0.224
349	FBgn0013576	chr3R	-	5334404	5350010	5334404	5334701	5349467	5350010	0.0032516	0.582
350	FBgn0005649	chr3R	-	24045437	24047012	24045437	24046491	24046941	24047012	7.5218E-05	0.303
351	FBgn0259243	chr3L	+	20828224	20829540	20828224	20828340	20829079	20829540	0.03894716	-0.429
352	FBgn0013305	chr2R	-	12879196	12879656	12879196	12879222	12879573	12879656	0.00150441	0.668
353	FBgn0003371	chrX	+	2672913	2673618	2672913	2673103	2673176	2673618	6.6716E-06	-0.722
354	FBgn0003575	chrX	+	635889	638720	635889	636279	638462	638720	0.03139654	-0.488
355	FBgn0037698	chr3R	-	9486600	9493677	9486600	9487203	9493473	9493677	0.00287437	-0.47
356	FBgn0030812	chrX	-	17072453	17072724	17072453	17072539	17072597	17072724	0.00158529	0.731
357	FBgn0000119	chr2R	-	13481775	13483229	13481775	13482214	13483154	13483229	1.401E-10	0.86
358	FBgn0052174	chr3L	-	17428935	17429600	17428935	17429303	17429372	17429600	0.00244101	0.618
359	FBgn0037993	chr3R	-	12156108	12158595	12156108	12156605	12158404	12158595	0.00082369	-0.391
360	FBgn0031857	chr2L	-	6770590	6772755	6770590	6770718	6772551	6772755	9.388E-06	0.194
361	FBgn0026199	chr4	-	695213	696055	695213	695526	695696	696055	0.00328171	-0.294
362	FBgn0016976	chrX	-	22994483	23004648	22994483	22995914	23004428	23004648	0.0016195	-0.343
363	FBgn0004436	chr3R	-	4950466	4952682	4950466	4950648	4952452	4952682	0	-0.727
364	FBgn0033494	chr2R	-	10150855	10152422	10150855	10151052	10151960	10152422	0.04351221	-0.387
365	FBgn0025835	chrX	-	146434	148426	146434	147785	147961	148426	0.01981292	-0.255
366	FBgn0033358	chr2R	-	8963614	8964002	8963614	8963720	8963784	8964002	0.04897253	0.622
367	FBgn0011206	chr3L	-	9123615	9125313	9123615	9123800	9125166	9125313	0.00120377	0.325
368	FBgn0036398	chr3L	+	14002126	14003415	14002126	14002301	14003196	14003415	0.0010871	-0.75
369	FBgn0031702	chr2L	-	5331262	5331660	5331262	5331446	5331511	5331660	0.01661385	0.465

370	FBgn0002719	chr3R	-	12719402	12720784	12719402	12720027	12720638	12720784	0.0240862	-0.438
371	FBgn0002719	chr3R	-	12719402	12720784	12719402	12720027	12720735	12720784	0.00337326	-0.445
372	FBgn0032836	chr2L	+	19964839	19965702	19964839	19964998	19965062	19965702	0.01469144	0.382
373	FBgn0001987	chr2L	-	15760401	15762406	15760401	15760574	15762342	15762406	0.02445162	-0.564
374	FBgn0051522	chr3R	-	4447700	4453316	4447700	4447954	4452932	4453316	0.00086029	-0.387
375	FBgn0034371	chr2R	-	18642404	18658222	18642404	18642762	18658071	18658222	0.04252921	-0.404
376	FBgn0039941	chrX	+	23073106	23075070	23073106	23073247	23074624	23075070	0.00894045	-0.778
377	FBgn0003941	chr2L	+	4227698	4227956	4227698	4227749	4227827	4227956	0.00035195	-0.675
378	FBgn0051092	chr3R	-	25718397	25720117	25718397	25718614	25719951	25720117	0	-0.843
379	FBgn0051092	chr3R	-	25718397	25720117	25718397	25718614	25719965	25720117	0	-0.697
380	FBgn0051092	chr3R	-	25718397	25720117	25718397	25718623	25720004	25720117	0.00486462	0.383
381	FBgn0000541	chr3L	-	245927	246912	245927	246323	246438	246912	0.00236562	-0.569
382	FBgn0039543	chr3R	-	27703378	27704329	27703378	27703856	27704021	27704329	0.00412507	-0.65
383	FBgn0259246	chr2R	+	9503764	9505468	9503764	9503845	9505117	9505468	0.04216921	0.383
384	FBgn0051999	chr4	-	215329	217494	215329	215560	217404	217494	0.00084186	-0.325
385	FBgn0053547	chr3R	-	17886148	17894902	17886148	17886544	17887420	17894902	4.7413E-10	-0.295
386	FBgn0040532	chr3R	+	8822007	8822666	8822007	8822125	8822509	8822666	0.01525773	0.144
387	FBgn0033092	chr2R	+	6691697	6692063	6691697	6691825	6691888	6692063	0.00077526	0.615
388	FBgn0026718	chr2L	-	8450793	8463994	8450793	8451004	8463641	8463994	0.04319289	0.667
389	FBgn0261714	chr3R	-	12163621	12166186	12163621	12165673	12166010	12166186	0.01348399	0.575
390	FBgn0261714	chr3R	-	12163621	12166186	12163621	12165673	12166158	12166186	0.00040939	0.475
391	FBgn0261714	chr3R	-	12163621	12166186	12163621	12165673	12166162	12166186	0.04210353	0.155
392	FBgn0016754	chr3R	-	23921517	23922973	23921517	23921780	23922629	23922973	0.02859502	-0.734
393	FBgn0019650	chr4	+	992065	996749	992065	992246	996491	996749	0.02202766	0.493
394	FBgn0023213	chr4	-	930079	930715	930079	930131	930647	930715	3.1605E-05	0.889
395	FBgn0024733	chr3L	-	23095274	23095471	23095274	23095305	23095363	23095471	0.02255749	-0.319
396	FBgn0034143	chr2R	-	16597903	16604238	16597903	16598263	16603917	16604238	0.04956185	0.515
397	FBgn0025574	chr3R	-	23889056	23890052	23889056	23889325	23889693	23890052	0.01222065	0.563
398	FBgn0033320	chr2R	-	8694779	8695797	8694779	8695251	8695705	8695797	0.00038919	0.498
399	FBgn0033437	chr2R	-	9609095	9610290	9609095	9609442	9610045	9610290	0.02633806	0.655
400	FBgn0033544	chr2R	+	10732698	10736055	10732698	10732796	10735823	10736055	0.00020149	-0.335
401	FBgn0014396	chr2L	-	3501617	3502557	3501617	3502126	3502315	3502557	6.7032E-05	-0.772
402	FBgn0000299	chr2L	+	5030136	5030790	5030136	5030237	5030567	5030790	0.00010116	-0.9
403	FBgn0033853	chr2R	-	13542160	13545449	13542160	13542547	13545307	13545449	8.0235E-11	-0.516
404	FBgn0002921	chr3R	+	20957733	20959380	20957733	20958167	20959348	20959380	0.03246023	0.166
405	FBgn0031752	chr2L	+	5952251	5952627	5952251	5952384	5952469	5952627	3.4464E-08	-1
406	FBgn0023508	chrX	+	1924098	1924789	1924098	1924285	1924531	1924789	0.00242612	-0.863
407	FBgn0034509	chr2R	+	20503583	20504202	20503583	20503720	20503800	20504202	0.04556249	0.52
408	FBgn0034530	chr2R	-	20630683	20633506	20630683	20630836	20633350	20633506	0.04227326	-0.295
409	FBgn0034075	chr2R	+	16111982	16112530	16111982	16112141	16112203	16112530	2.7916E-05	-0.38
410	FBgn0038034	chr3R	-	12397333	12398929	12397333	12397853	12398673	12398929	0.01923234	0.767
411	FBgn0036545	chr3L	+	15955387	15956465	15955387	15955549	15955610	15956465	0.04921928	0.555
412	FBgn0052057	chr3L	-	10182242	10186108	10182242	10182530	10185978	10186108	6.2635E-09	-0.867
413	FBgn0051183	chr3R	-	15869172	15875939	15869172	15870232	15875572	15875939	1.1948E-09	0.558
414	FBgn0250785	chr2L	-	20792113	20794146	20792113	20792266	20793877	20794146	6.5163E-05	-0.561
415	FBgn0035481	chr3L	-	3961571	3963977	3961571	3962669	3963510	3963977	0.01923234	0.767
416	FBgn0035501	chr3L	+	4148695	4150722	4148695	4149025	4150528	4150722	1.2202E-06	0.446
417	FBgn0020304	chr2L	-	836545	842943	836545	837378	842848	842943	0.00894045	-0.748
418	FBgn0264607	chr4	-	1050454	1053703	1050454	1050862	1053497	1053703	0.0410707	-0.339
419	FBgn0035802	chr3L	+	7636747	7637901	7636747	7637480	7637600	7637901	6.7136E-06	0.858
420	FBgn0035811	chr3L	+	7719349	7720416	7719349	7719457	7720021	7720416	9.9851E-06	0.718
421	FBgn0036059	chr3L	+	9899240	9900084	9899240	9899355	9899955	9900084	1.1122E-06	0.458
422	FBgn0087011	chr2R	+	1761923	1762213	1761923	1762059	1762118	1762213	0.00179448	0.614
423	FBgn0032233	chr2L	+	10376765	10377388	10376765	10376884	10377237	10377388	0.0016195	-0.511
424	FBgn0036318	chr3L	+	12850914	12851722	12850914	12851153	12851386	12851722	0.01517757	0.201
425	FBgn0035400	chr3L	-	3132632	3134180	3132632	3133338	3133952	3134180	0.04252921	0.282
426	FBgn0261565	chr3L	+	16916254	16919702	16916254	16916424	16919455	16919702	0.00033531	0.304
427	FBgn0010741	chr3L	-	11073465	11074388	11073465	11074011	11074064	11074388	0.02438296	0.432
428	FBgn0003041	chr3L	-	7907121	7911954	7907121	7907338	7911781	7911954	0.00321349	0.667
429	FBgn0037646	chr3R	+	8980859	8981915	8980859	8981109	8981629	8981915	6.2048E-05	0.766
430	FBgn0033669	chr2R	-	11968283	11968850	11968283	11968636	11968739	11968850	0.01046827	-0.573
431	FBgn0038720	chr3R	-	19619084	19623476	19619084	19619380	19623336	19623476	0.04380305	0.493
432	FBgn0038721	chr3R	-	19636039	19636739	19636039	19636215	19636273	19636739	0.00236562	0.667
433	FBgn0002781	chr3R	-	21366449	21375912	21366449	21366711	21366973	21375912	0.00907554	0.215
434	FBgn0023416	chr2L	+	21638229	21639503	21638229	21638368	21639279	21639503	1.2683E-07	1
435	FBgn0038294	chr3R	-	15172843	15174343	15172843	15173063	15174137	15174343	0.00244101	0.521
436	FBgn0039481	chr3R	-	27089703	27091130	27089703	27090606	27090671	27091130	0.00458429	-0.454
437	FBgn0259221	chr2R	-	13422601	13424736	13422601	13422725	13424485	13424736	0.00610174	-0.623
438	FBgn0037218	chr3R	+	4222257	4225008	4222257	4223190	4224670	4225008	0.02573501	-0.778
439	FBgn0041337	chr2L	-	2564885	2565567	2564885	2565248	2565309	2565567	0.02202766	-0.598
440	FBgn0042693	chr3R	+	18176003	18176699	18176003	18176234	18176309	18176699	0.00982944	0.449

SE											
	FlyBase ID	chr	strand	exonStart_0base	exonEnd	upstreamES	upstreamEE	downstreamES	downstreamEE	FDR	ΔPSI
1	FBgn0020251	chr3L	+	6505082	6505119	6495679	6496481	6523029	6524096	0.02226376	0.222
2	FBgn0052082	chr3L	+	11163386	11163488	11160780	11160932	11163554	11163984	0.00075916	-0.324
3	FBgn0022709	chr3L	-	12201778	12201862	12201373	12201496	12203718	12204082	0.01605325	-0.118
4	FBgn0030349	chrX	-	11883100	11883621	11881329	11881902	11886955	11887162	0.04960835	-0.136
5	FBgn0030349	chrX	-	11883187	11883621	11881329	11881902	11886955	11887162	0.00403134	-0.185
6	FBgn0030349	chrX	-	11884797	11885056	11881329	11881902	11886955	11887052	0.0332422	-0.393
7	FBgn0285963	chr3R	+	26916502	26916562	26915760	26915908	26924633	26925261	0.00221692	0.211
8	FBgn0052112	chr3L	+	12742594	12742650	12741804	12741949	12742904	12743172	0.00315713	0.3
9	FBgn0263346	chr3R	+	4680228	4680579	4670796	4670850	4697190	4697542	0.01434871	0.657
10	FBgn0263346	chr3R	+	4680778	4680837	4670796	4670850	4697190	4697542	0	0.462
11	FBgn0262579	chr3L	+	8096970	8097172	8096521	8096872	8098345	8098725	0.00279113	0.229
12	FBgn0266084	chr3L	+	8786621	8787344	8786299	8786437	8787518	8787720	1.87E-02	0.179
13	FBgn0261984	chr3R	-	19860646	19860761	19857240	19857684	19860885	19861291	6.26E-03	0.111
14	FBgn0261928	chr3R	+	10228704	10229234	10226487	10226619	10239476	10239497	5.74E-03	0.15
15	FBgn0050021	chr2R	-	11229003	11229033	11228508	11228676	11229410	11229542	9.72E-03	0.242
16	FBgn0264489	chr3L	+	9830999	9831440	9830633	9830785	9832195	9832679	0.00E+00	-0.212
17	FBgn0264489	chr3L	+	9830999	9831440	9830633	9830785	9832198	9832679	0.00E+00	-0.474
18	FBgn0264489	chr3L	+	9830999	9831440	9830633	9830785	9832477	9832679	3.22E-03	-0.256
19	FBgn0263199	chr3L	-	8985283	8985308	8985052	8985231	8985418	8985816	2.55E-02	0.553
20	FBgn0035772	chr3L	-	7366441	7366516	7364583	7364961	7367558	7367984	4.70E-02	0.167
21	FBgn0035772	chr3L	-	7366441	7366516	7364583	7364964	7367558	7367984	4.94E-02	0.144
22	FBgn0034718	chr2R	-	22308207	22308256	22298213	22301009	22311695	22311937	6.26E-03	0.136
23	FBgn0038840	chr3R	+	20907211	20907361	20907037	20907153	20907932	20908263	3.10E-02	-0.379
24	FBgn0013995	chr3R	+	20991574	20991680	20989365	20989599	20992240	20994423	1.87E-02	0.253
25	FBgn0052451	chr3L	+	22774737	22775137	22773535	22773716	22777110	22778824	0.01280971	0.117
26	FBgn0033807	chr2R	-	13213750	13213931	13213022	13213436	13214418	13214496	0	0.599
27	FBgn0032901	chr2L	-	20875637	20875724	20874273	20874432	20876586	20876613	0.00294683	-0.212
28	FBgn0051973	chr2L	-	30393	33270	28981	29068	33844	34288	0.02298829	0.166
29	FBgn0000723	chr3R	-	9372376	9372580	9369335	9369710	9372755	9372933	0.0005594	0.152
30	FBgn0262508	chr3L	+	8581762	8581855	8579473	8579639	8585970	8586956	0.00337325	0.776
31	FBgn0027580	chr2R	-	9853682	9853730	9852547	9853205	9854510	9856020	0	0.265
32	FBgn0053558	chr2R	-	6933123	6933927	6930248	6930370	6934065	6934180	0.03145649	0.148
33	FBgn0032586	chr2L	+	16497010	16497151	16491539	16492051	16505495	16505948	0.00757131	0.122
34	FBgn0033504	chr2R	+	10294149	10294182	10293860	10293933	10294611	10294765	0.00140402	0.295

35	FBgn0263077	chr2R	+	7630176	7630328	7628796	7629112	7634215	7634314	0.01677618	-0.136
36	FBgn0034051	chr2R	-	15937678	15937879	15936090	15936583	15938446	15938978	2.8988E-05	0.348
37	FBgn0034051	chr2R	-	15938114	15938267	15937678	15937879	15938446	15938978	2.6341E-05	-0.173
38	FBgn0013467	chr2R	+	15163885	15164026	15155436	15156215	15180512	15180710	5.2712E-06	-0.203
39	FBgn0013467	chr2R	+	15164581	15164639	15163885	15164026	15180512	15180710	0.0001087	0.465
40	FBgn0002921	chr3R	+	20967107	20967202	20966397	20966613	20968391	20968486	0.00115762	-0.16
41	FBgn0011640	chr3L	+	7339867	7339908	7339463	7339617	7339961	7340770	1.7665E-08	-0.669
42	FBgn0016930	chr2L	-	14229458	14229536	14222016	14222081	14233647	14234108	0.00140402	0.189
43	FBgn0016930	chr2L	-	14229531	14229536	14222016	14222081	14233647	14234108	0.01459388	0.097
44	FBgn0259481	chr3L	-	11541777	11542834	11527181	11527342	11561893	11562233	3.5249E-07	0.219
45	FBgn0261788	chr3L	-	7661980	7662298	7655388	7656203	7663258	7663324	0.03340301	-0.254
46	FBgn0052082	chr3L	+	11163415	11163488	11160780	11160932	11163554	11163984	0.02516284	-0.314
47	FBgn0022709	chr3L	-	12201778	12201919	12201373	12201496	12203718	12204082	0.00076463	-0.1
48	FBgn0004401	chr3L	-	17643978	17644079	17643362	17643726	17644818	17644866	0.01266576	-0.743
49	FBgn0030349	chrX	-	11883201	11883621	11881329	11881902	11886955	11887162	0.00200745	-0.224
50	FBgn0052112	chr3L	+	12742224	12742650	12741860	12741949	12742904	12743172	0.00155497	0.314
51	FBgn0052112	chr3L	+	12742224	12742650	12741860	12741949	12743512	12743625	2.7457E-05	0.662
52	FBgn0052112	chr3L	+	12742572	12742650	12741860	12741949	12742904	12743172	0.01726544	0.292
53	FBgn0052112	chr3L	+	12742572	12742650	12741860	12741949	12743512	12743625	0.01870742	0.447
54	FBgn0266599	chr3R	+	15243040	15243180	15242889	15242970	15244359	15246614	0.00034783	0.32
55	FBgn0266599	chr3R	+	15243050	15243180	15242889	15242970	15244359	15246614	0.00041971	0.31
56	FBgn0266599	chr3R	+	15243050	15243294	15242889	15242970	15244359	15246614	0.00337325	0.623
57	FBgn0266599	chr3R	+	15243069	15243180	15242889	15242970	15244359	15246614	0.00025004	0.353
58	FBgn0266599	chr3R	+	15243069	15243294	15242889	15242970	15244359	15246614	0.02749916	0.627
59	FBgn0266599	chr3R	+	15243159	15243180	15242889	15242970	15244359	15246614	0.00180478	0.598
60	FBgn0266599	chr3R	+	15243159	15243294	15242889	15242970	15244359	15246614	0.01266576	0.654
61	FBgn0263456	chr3L	+	9142572	9142605	9141786	9141843	9143297	9143323	0.00150343	0.165
62	FBgn0036211	chr3L	-	11817370	11817652	11816982	11817316	11818087	11818187	0.00160298	0.135
63	FBgn0036211	chr3L	-	11817388	11817652	11816982	11817316	11818087	11818187	1.0074E-07	0.206
64	FBgn0036211	chr3L	-	11817388	11817694	11816982	11817316	11818087	11818187	1.032E-05	0.177
65	FBgn0036211	chr3L	-	11817388	11817724	11816982	11817316	11818087	11818187	1.2071E-06	0.171
66	FBgn0036211	chr3L	-	11817399	11817652	11816982	11817316	11818087	11818187	0.00182737	0.134
67	FBgn0263346	chr3R	+	4680708	4680837	4670796	4670850	4697190	4697542	0	0.739
68	FBgn0262579	chr3L	+	8097023	8097172	8096521	8096872	8098345	8098725	0.00319262	0.229
69	FBgn0266084	chr3L	+	8786621	8786979	8786299	8786437	8787518	8787720	0.00685961	0.212
70	FBgn0261984	chr3R	-	19860490	19860761	19857240	19857684	19860885	19861291	3.6451E-09	0.273
71	FBgn0261928	chr3R	+	10228711	10228912	10226487	10226619	10239476	10239497	0.00129919	0.179
72	FBgn0261928	chr3R	+	10228711	10228912	10226487	10226619	10239617	10239958	0.00463426	0.678
73	FBgn0261928	chr3R	+	10228745	10228912	10226487	10226619	10239476	10239497	0.00128092	0.178
74	FBgn0261928	chr3R	+	10228745	10228912	10226487	10226619	10239617	10239958	0.00476102	0.565
75	FBgn0261928	chr3R	+	10228745	10229234	10226487	10226619	10239476	10239497	0.00019475	0.548
76	FBgn0261928	chr3R	+	10229069	10229234	10226487	10226619	10239476	10239497	0.00025336	0.542
77	FBgn0004910	chr3L	-	3933246	3933389	3930957	3930996	3935485	3935617	2.3785E-05	-0.417
78	FBgn0004910	chr3L	-	3933246	3933492	3930957	3930996	3935485	3935617	1.4905E-05	-0.432
79	FBgn0004910	chr3L	-	3933246	3933511	3930957	3930996	3935485	3935617	1.7814E-05	-0.428
80	FBgn0033446	chr2R	-	9818996	9819008	9812481	9812664	9819839	9820053	0.03337783	0.123
81	FBgn0264489	chr3L	+	9830975	9831440	9830633	9830785	9832195	9832679	3.1575E-13	-0.385
82	FBgn0264489	chr3L	+	9830975	9831440	9830633	9830785	9832198	9832679	3.9585E-10	-0.603
83	FBgn0264489	chr3L	+	9830975	9831440	9830633	9830785	9832201	9832679	2.66E-05	-0.444
84	FBgn0264489	chr3L	+	9831293	9831440	9830633	9830785	9832195	9832679	1.0408E-11	-0.371
85	FBgn0264489	chr3L	+	9831293	9831440	9830633	9830785	9832198	9832679	0	-0.629
86	FBgn0264489	chr3L	+	9831293	9831440	9830633	9830785	9832201	9832679	0.0014013	-0.272
87	FBgn0264489	chr3L	+	9831431	9831440	9830633	9830785	9832195	9832679	1.7103E-13	-0.4
88	FBgn0264489	chr3L	+	9831431	9831440	9830633	9830785	9832198	9832679	0	-0.891
89	FBgn0264489	chr3L	+	9831431	9831440	9830633	9830785	9832201	9832679	0.00290414	-0.461
90	FBgn0264489	chr3L	+	9831434	9831440	9830633	9830785	9832195	9832679	0	-0.409
91	FBgn0264489	chr3L	+	9831434	9831440	9830633	9830785	9832198	9832679	0	-0.907
92	FBgn0264489	chr3L	+	9831434	9831440	9830633	9830785	9832201	9832679	0.0032006	-0.457
93	FBgn0025574	chr3R	-	23888826	23889325	23865562	23865655	23889693	23890052	0.00019475	-0.818
94	FBgn0005561	chr4	+	1109077	1109537	1108479	1108572	1110780	1110974	0.01240444	0.416
95	FBgn0039790	chr3R	-	30595720	30596017	30595389	30595621	30600263	30600323	0.00476102	0.542
96	FBgn0036240	chr3L	+	12004395	12004545	12003154	12003294	12005253	12005858	0.01262759	-0.403
97	FBgn0035772	chr3L	-	7366441	7366562	7364583	7364964	7367558	7367984	0.00077931	0.214
98	FBgn0035772	chr3L	-	7366441	7366581	7364583	7364964	7367558	7367984	0.00060036	0.225
99	FBgn0035772	chr3L	-	7366441	7366662	7364583	7364964	7367558	7367984	0.00050518	0.23
100	FBgn0035772	chr3L	-	7366441	7366673	7364583	7364964	7367558	7367984	0.00050518	0.23
101	FBgn0039897	chr4	+	234863	234948	231934	231977	236394	236475	0.00778091	0.667
102	FBgn0038198	chr3R	+	14090501	14090879	14089516	14089599	14091297	14091401	0.00041971	0.166
103	FBgn0038198	chr3R	+	14090651	14090879	14089516	14089599	14091297	14091401	0.00041971	0.166
104	FBgn0033368	chr2R	-	9056572	9056627	9050842	9051151	9059254	9059548	0.00246237	-0.293
105	FBgn0259214	chr4	-	337535	337547	330270	330332	342921	343060	0.00107036	0.289
106	FBgn0031995	chr2L	-	8194501	8194528	8193124	8193229	8195020	8195569	0.00614084	0.186
107	FBgn0002121	chr2L	-	18025	18168	17052	17212	18330	18570	7.8265E-05	0.637
108	FBgn0052451	chr3L	+	22774761	22775137	22773535	22773716	22777110	22778824	0.00444266	0.126
109	FBgn0052451	chr3L	+	22774817	22775137	22773535	22773716	22777110	22778824	0.00445712	0.126
110	FBgn0052451	chr3L	+	22774974	22775137	22773535	22773716	22777110	22778824	0.00472967	0.125
111	FBgn0033807	chr2R	-	13213532	13213931	13213022	13213436	13214418	13214496	0	0.473
112	FBgn0051973	chr2L	-	30393	30586	28981	29068	33844	34288	0.04160173	0.158
113	FBgn0011224	chr3R	-	32011039	32011102	31979522	31979619	32014359	32015520	0.00132264	-0.326
114	FBgn0264270	chrX	-	7091673	7092170	7090133	7090504	7097862	7098053	0.00476102	0.562
115	FBgn0019650	chr4	+	992051	992246	989724	990502	1005806	1006009	0.00061589	0.667
116	FBgn0085385	chr3L	+	9960861	9960879	9958789	9958849	9961636	9961786	0.0005594	-0.755
117	FBgn0263077	chr2R	+	7629833	7630328	7628800	7629112	7634215	7634314	0.02549463	-0.183
118	FBgn0263077	chr2R	+	7629870	7630328	7628800	7629112	7634215	7634314	0.02317172	-0.185
119	FBgn0263077	chr2R	+	7630056	7630328	7628800	7629112	7634215	7634314	0.02319524	-0.184
120	FBgn0263077	chr2R	+	7630101	7630328	7628800	7629112	7634215	7634314	0.0238181	-0.184
121	FBgn0263077	chr2R	+	7630147	7630328	7628800	7629112	7634215	7634314	0.02554212	-0.183
122	FBgn0263077	chr2R	+	7630185	7630328	7628800	7629112	7634215	7634314	0.02298829	-0.189
123	FBgn0034075	chr2R	+	16113645	16113883	16113180	16113258	16114771	16114866	0.00489824	0.617
124	FBgn0020767	chr2R	-	14768729	14769053	14768003	14768370	14769277	14769423	0.00465139	0.264
125	FBgn0001624	chrX	+	11373816	11373856	11369664	11369799	11374256	11374510	0.02226376	0.294
126	FBgn0053182	chr2R	-	13189696	13189980	13187393	13187908	13198212	13198337	0.0400996	-0.834
127	FBgn0005666	chr4	+	734716	734812	734477	734537	735514	735823	0.02226376	-0.31
128	FBgn0022361	chr4	+	560473	560574	560371	560411	560717	560820	1.737E-08	0.763
129	FBgn0024807	chrX	-	21628919	21629105	21628360	21628754	21630066	21630148	0.00114839	-0.627
130	FBgn0024807	chrX	-	21628919	21629123	21628360	21628754	21630066	21630148	0.0001905	-0.691
131	FBgn0024807	chrX	-	21628919	21629128	21628360	21628754	21630066	21630148	0.00045556	-0.643
132	FBgn0024807	chrX	-	21628919	21629273	21628360	21628754	21630066	21630148	0.0003712	-0.667
133	FBgn0024807	chrX	-	21628919	21629286	21628360	21628754	21630066	21630148	0.0001905	-0.691
134	FBgn0010263	chr2L	+	2960724	2960991	2959146	2959520	2961214	2961672	0.02673343	-0.667
135	FBgn0025726	chr4	-	878840	878992	876869	877194	882960	883081	8.8444E-06	-0.599
136	FBgn0025726	chr4	-	878851	878992	876869	877194	882960	883081	8.5317E-06	-0.671
137	FBgn0261361	chr3L	-	19748479	19748539	19747563	19748414	19749115	19749744	0.04867842	0.567
138	FBgn0011640	chr3L	+	7339867	7339905	7339463	7339617	7339961	7340770	7.6895E-11	-0.806
139	FBgn0016930	chr2L	-	14229453	14229536	14222016	14222081	14233647	14234108	0.03145649	0.241
140	FBgn0035976	chr3L	+	9340355	9340593	9338809	9338866	9342164	9343129	0.01434871	0.446
141	FBgn0083975	chr3R	+	20206317	20206712	20204808	20204978	20211080	20211505	0.00483494	0.63
142	FBgn0051323	chr3R	+	26171195	26171455	26170846	26171038	26183799	26183964	8.7401E-08	-0.426
143	FBgn0051323	chr3R	+	26171300	26171455	26170846	26171038	26183799	26183964	2.2423E-07	-0.4
144	FBgn0051323	chr3R	+	26171331	26171455	26170846	26171038	26183799	26183964	2.834E-07	-0.442
145	FBgn0259174	chr3L	+	17542593	17543085	17542199	17542384	17543504	17543612	0.02454233	-0.449

3) List of DASs in *Drosophila* adults of *Dm65k* $^{Δ/Δ}$ *vs* *WT*

	FlyBase ID	chr	strand	longExonStart_0base	longExonEnd	shortES	shortEE	flankingES	flankingEE	FDR	ΔPSI
						A3SS					
1	FBgn0036958	chr3L	-	20238737	20239613	20238737	20239547	20239862	20240532	0.043430015	0.192
2	FBgn0267376	chr3R	-	10866808	10866976	10866808	10866960	10867971	10868290	0	0.518
3	FBgn0267376	chr3R	-	10866808	10866976	10866808	10866960	10868438	10868642	0	0.85
4	FBgn0265067	chr3R	+	21094948	21095744	21094953	21095744	21094761	21094888	0.039914432	-0.409
5	FBgn0263980	chr2R	+	15465330	15465491	15465357	15465491	15464470	15464686	4.34563E-05	0.302
6	FBgn0262737	chr3L	+	21921056	21921327	21921092	21921327	21920902	21920987	0.001222421	-0.18
7	FBgn0262480	chr2R	+	18864142	18864288	18864165	18864288	18863895	18864094	0.002989215	0.29
8	FBgn0262166	chr2R	-	16195716	16196000	16195716	16195970	16196066	16196333	0.010313448	-0.211
9	FBgn0082582	chr3R	+	30548420	30548749	30548451	30548749	30546405	30546587	0	-0.488
10	FBgn0000454	chr3R	+	13782694	13782841	13782707	13782841	13782287	13782451	0.031182476	-0.21
11	FBgn0264908	chr3L	-	15852138	15852276	15852138	15852249	15858100	15858151	0.000986099	-0.668
12	FBgn0264908	chr3L	-	15852138	15852276	15852138	15852249	15859811	15860026	2.51806E-07	-0.699
13	FBgn0032889	chr2L	+	20825470	20826087	20825667	20826087	20824433	20824493	0.040658144	-0.122
14	FBgn0024321	chr3R	-	14367192	14367499	14367192	14367496	14372546	14372951	6.24215E-07	0.799
15	FBgn0030976	chrX	+	18908249	18908374	18908276	18908374	18907730	18907859	6.32899E-14	0.241
16	FBgn0004360	chr2R	+	9501370	9501588	9501375	9501588	9494165	9495253	0.031829624	0.22
17	FBgn0053098	chr3R	+	12387530	12387637	12387543	12387637	12387187	12387286	1.46679E-07	0.274
18	FBgn0051973	chr2L	-	34719	35212	34719	34912	38534	38731	7.3061E-08	-0.182
19	FBgn0037182	chr3L	-	22716970	22717080	22716970	22717066	22717193	22717276	0.033175715	0.162
20	FBgn0037182	chr3L	-	22716970	22717363	22716970	22717349	22717490	22717577	1.97244E-07	0.793
21	FBgn0032172	chr2L	-	9960449	9961617	9960449	9961614	9961728	9962059	9.17229E-12	-0.374
22	FBgn0032172	chr2L	-	9960449	9961617	9960449	9961614	9961794	9962059	0.025383667	-0.515
23	FBgn0034858	chr2R	+	23386941	23387612	23387006	23387612	23386520	23386622	5.88596E-12	-0.404
24	FBgn0051814	chr2L	-	13713972	13714311	13713972	13714166	13717020	13717320	0.006756223	-0.35
25	FBgn0037614	chr3R	+	8716895	8717341	8716900	8717341	8716242	8716568	0.046593214	-0.236
26	FBgn0015222	chr3R	-	30387463	30387602	30387463	30387550	30387774	30387860	0.006251383	-0.132
27	FBgn0015222	chr3R	-	30387463	30387642	30387463	30387550	30387774	30387860	0.013843187	-0.13
28	FBgn0015222	chr3R	-	30387463	30387642	30387463	30387602	30387774	30387860	2.22014E-12	0.156
29	FBgn0266692	chr3L	+	25844171	25844502	25844287	25844502	25844007	25844121	0.007829906	-0.402
30	FBgn0028496	chr2R	-	18496746	18496905	18496746	18496868	18497841	18498026	5.4674E-11	0.475
31	FBgn0010226	chr2R	-	17094315	17094458	17094315	17094449	17094516	17094696	0.000198584	-0.153
32	FBgn0022343	chr2R	+	24938955	24939217	24938973	24939217	24938759	24938842	0.001596542	0.153
33	FBgn0034645	chr2R	+	21658345	21658480	21658350	21658480	21658077	21658109	0.035354916	0.109
34	FBgn0053170	chr3L	+	22867115	22867535	22867399	22867535	22865877	22866034	0.018342023	0.22
35	FBgn0011260	chr2R	+	16516919	16517341	16517018	16517341	16516149	16516270	0.002948958	0.359
36	FBgn0034075	chr2R	+	16112195	16112530	16112201	16112530	16111982	16112141	0	0.296
37	FBgn0020236	chr2R	-	15996166	15996365	15996166	15996314	15996894	15997218	0.004946291	-0.133
38	FBgn0020236	chr2R	-	15996166	15996365	15996166	15996317	15996894	15997218	0.00069638	-0.194
39	FBgn0034051	chr2R	-	15935892	15936583	15935892	15936580	15938114	15938267	1.08745E-05	-0.238
40	FBgn0034903	chr2R	-	23755494	23757166	23755494	23756551	23757414	23757494	0.000308129	-0.244
41	FBgn0040260	chr2L	+	16799660	16800520	16799680	16800520	16799024	16799133	1.45474E-11	0.236
42	FBgn0033373	chr2R	-	9090810	9091051	9090810	9091039	9091406	9091653	9.88481E-06	-0.286
43	FBgn0027515	chr2L	+	7822641	7823100	7822675	7823100	7821534	7822056	0.018595097	-0.18
44	FBgn0030657	chrX	-	15576985	15577765	15576985	15577749	15577810	15577998	8.06632E-05	0.424
45	FBgn0038294	chr3R	-	15176363	15176427	15176363	15176412	15178279	15178489	0.006288371	-0.154
46	FBgn0265991	chr2R	-	15774427	15775507	15774427	15774757	15777177	15777286	6.53995E-13	0.234
47	FBgn0085399	chr2R	-	22037391	22037719	22037391	22037716	22039355	22039431	0.029562288	-0.157
48	FBgn0265194	chr2R	-	15306877	15307298	15306877	15307154	15308841	15308997	0.009646038	-0.332
49	FBgn0250819	chr4	+	1186328	1186634	1186472	1186634	1185767	1185834	0.032954846	0.274
50	FBgn0261552	chr3R	+	9440680	9441665	9441415	9441665	9439345	9439705	0.004047179	0.246
51	FBgn0031636	chr2L	+	4841135	4841465	4841162	4841465	4840883	4840983	0	1
52	FBgn0030839	chrX	+	17425899	17426333	17426095	17426333	17423590	17423856	0.007759931	-0.287
53	FBgn0001105	chrX	+	15860744	15861153	15861151	15861153	15860114	15860250	0.001755524	-0.414
54	FBgn0001105	chrX	+	15860988	15861311	15861151	15861311	15860114	15860250	0.000818539	-0.441
55	FBgn0030364	chrX	+	12019586	12019758	12019627	12019758	12018639	12019325	1.78362E-13	0.234
56	FBgn0267376	chr3R	-	10866808	10866994	10866808	10866960	10867971	10868290	0	0.829
57	FBgn0267376	chr3R	-	10866808	10866994	10866808	10866960	10868438	10868642	0	0.88
58	FBgn0264695	chr2L	+	16783768	16783909	16783797	16783909	16782774	16783661	0.001564051	-0.408
59	FBgn0036331	chr3L	+	13008622	13008873	13008654	13008873	13008284	13008567	0	0.479
60	FBgn0263593	chr2R	-	8138434	8138802	8138434	8138617	8138937	8139041	6.16624E-13	-0.816
61	FBgn0034968	chr2R	+	24061132	24061413	24061154	24061413	24060812	24060993	0.025893439	-0.311
62	FBgn0263102	chr2R	+	10561685	10561954	10561695	10561954	10557887	10558533	0	0.298
63	FBgn0262794	chr3R	+	17644552	17644892	17644777	17644892	17630716	17630956	3.61714E-05	0.522
64	FBgn0082582	chr3R	+	30548420	30548749	30548451	30548749	30546404	30546598	0.009878538	-0.115
65	FBgn0082582	chr3R	+	30548278	30548749	30548420	30548749	30546405	30546587	6.32899E-14	-0.971
66	FBgn0082582	chr3R	+	30548278	30548749	30548451	30548749	30546405	30546587	0	-0.811
67	FBgn0082582	chr3R	+	30548283	30548749	30548420	30548749	30546405	30546587	0	-0.993
68	FBgn0082582	chr3R	+	30548283	30548749	30548451	30548749	30546405	30546587	0	-0.823
69	FBgn0082582	chr3R	+	30548303	30548749	30548420	30548749	30546405	30546587	0	-0.972
70	FBgn0082582	chr3R	+	30548303	30548749	30548451	30548749	30546405	30546587	0	-0.811
71	FBgn0082582	chr3R	+	30548309	30548749	30548420	30548749	30546405	30546587	0	-0.993
72	FBgn0082582	chr3R	+	30548309	30548749	30548451	30548749	30546405	30546587	0	-0.82
73	FBgn0261808	chr3R	+	11205236	11205674	11205583	11205674	11204564	11204831	0.009287274	-0.59
74	FBgn0053092	chr3R	+	21878171	21878308	21878251	21878308	21877935	21878071	1.68845E-09	0.445
75	FBgn0085408	chr2R	+	14315103	14315533	14315104	14315533	14308141	14308293	3.66424E-05	-0.43
76	FBgn0038181	chr3R	+	13774800	13775169	13775091	13775169	13774452	13774737	0.00039818	-0.733
77	FBgn0000056	chr2L	+	14616051	14616437	14616215	14616437	14615551	14615643	0.038996724	0.321
78	FBgn0000056	chr2L	+	14616057	14616437	14616215	14616437	14615551	14615643	0	0.724
79	FBgn0000056	chr2L	+	14616057	14616437	14616282	14616437	14615551	14615643	0.029327431	-0.226
80	FBgn0000056	chr2L	+	14616128	14616437	14616215	14616437	14615551	14615643	2.30822E-13	0.553
81	FBgn0000056	chr2L	+	14616215	14616437	14616282	14616437	14615551	14615643	1.00848E-07	-0.542
82	FBgn0013334	chr3R	+	15935937	15936340	15935981	15936340	15935118	15935584	0.019167029	-0.313
83	FBgn0029152	chr3L	-	21531643	21531683	21531643	21531664	21531757	21532031	1.51626E-07	0.725
84	FBgn0039667	chr3R	+	29495587	29495801	29495645	29495801	29494781	29494885	0.038997902	0.355
85	FBgn0039667	chr3R	+	29495567	29495801	29495645	29495801	29495008	29495125	0.003614813	0.463
86	FBgn0051678	chr2L	-	20664290	20664788	20664290	20664660	20667865	20668096	0.033950755	0.667
87	FBgn0033669	chr2R	-	11968283	11968631	11968283	11968591	11968739	11968850	0.028795437	0.384
88	FBgn0033669	chr2R	-	11968283	11968636	11968283	11968591	11968739	11968850	0.001222421	0.54
89	FBgn0037288	chr3R	-	4954335	4954724	4954335	4954451	4955100	4955252	1.90952E-10	-0.67
90	FBgn0036768	chr3L	+	17889109	17889462	17889330	17889462	17887704	17887790	2.06009E-11	1
91	FBgn0036768	chr3L	+	17889235	17889462	17889330	17889462	17887704	17887790	1.5908E-12	1
92	FBgn0036768	chr3L	+	17889313	17889462	17889330	17889462	17887704	17887790	0	0.873
93	FBgn0031089	chrX	+	20149902	20150125	20150067	20150125	20148123	20148261	4.78701E-05	0.401
94	FBgn0031089	chrX	+	20150031	20150125	20150067	20150125	20148123	20148261	0.001351589	0.307
95	FBgn0031089	chrX	+	20150043	20150125	20150067	20150125	20148123	20148261	0.003206225	0.236
96	FBgn0034253	chr2R	-	17637219	17637593	17637219	17637519	17639020	17639138	0.002989215	0.384
97	FBgn0031228	chr2L	+	155493	155784	155513	155784	155334	155429	0.029213458	-0.424
98	FBgn0000667	chrX	-	2035403	2035746	2035403	2035440	2036406	2036466	0.033019928	0.17
99	FBgn0003371	chrX	+	2634604	2634871	2634835	2634871	2633951	2634025	0.002948958	-0.504
100	FBgn0052944	chr3R	-	4418565	4419036	4418565	4418652	4419106	4419578	0.012064336	-0.472
101	FBgn0033054	chr2R	-	6038070	6038443	6038070	6038407	6040081	6040334	0.047968271	-0.645
102	FBgn0053098	chr3R	+	12387462	12387637	12387530	12387637	12387187	12387286	5.4482E-05	0.196
103	FBgn0053098	chr3R	+	12387462	12387637	12387543	12387637	12387187	12387286	1.67236E-11	0.231
104	FBgn0053098	chr3R	+	12387462	12387637	12387547	12387637	12387187	12387286	2.75763E-06	0.498
105	FBgn0053098	chr3R	+	12387475	12387637	12387530	12387637	12387187	12387286	0.000232366	0.197
106	FBgn0053098	chr3R	+	12387475	12387637	12387543	12387637	12387187	12387286	1.74297E-11	0.238
107	FBgn0053098	chr3R	+	12387475	12387637	12387547	12387637	12387187	12387286	4.78262E-06	0.473
108	FBgn0053098	chr3R	+	12387480	12387637	12387530	12387637	12387187	12387286	0.001667755	0.24
109	FBgn0053098	chr3R	+	12387480	12387637	12387543	12387637	12387187	12387286	0	0.386
110	FBgn0053098	chr3R	+	12387480	12387637	12387547	12387637	12387187	12387286	0.000218145	0.292
111	FBgn0053098	chr3R	+	12387495	12387637	12387530	12387637	12387187	12387286	0.001198027	0.189
112	FBgn0053098	chr3R	+	12387495	12387637	12387543	12387637	12387187	12387286	6.02622E-10	0.235
113	FBgn0053098	chr3R	+	12387495	12387637	12387547	12387637	12387187	12387286	8.8602E-06	0.46
114	FBgn0053098	chr3R	+	12387530	12387637	12387547	12387637	12387187	12387286	0.027438476	0.137
115	FBgn0031728	chr2L	+	5668768	5668871	5668817	5668871	5662848	5662981	0.003563146	-0.17
116	FBgn0031728	chr2L	+	5668802	5668871	5668817	5668871	5662848	5662981	1.18683E-06	-0.28
117	FBgn0002579	chrX	-	628750	628898	628750	628878	629381	629483	0.035418932	0.135
118	FBgn0033807	chr2R	-	13213532	13213949	13213532	13213558	13214418	13214496	0	0.713
119	FBgn0264894	chr2L	+	13274476	13274850	13274669	13274850	13274309	13274376	0.021833641	-0.32
120	FBgn0264894	chr2L	+	13274528	13274850	13274669	13274850	13274309	13274376	0.035045218	-0.314
121	FBgn0052017	chr4	-	1144965	1145103	1144965	1145100	1146469	1146529	0.003206225	0.667
122	FBgn0004575	chr3R	+	10196465	10196781	10196617	10196781	10193859	10194013	0.000842137	0.78
123	FBgn0250823	chr3R	+	16282080	16282408	16282193	16282408	16274953	16275169	0.029539823	0.364
124	FBgn0031815	chr2L	+	6456326	6456682	6456364	6456682	6456051	6456208	0.001914997	0.597
125	FBgn0028527	chr2L	+	13670835	13671247	13671018	13671247	13670437	13670597	0.000681926	0.692
126	FBgn0015222	chr3R	-	30387149	30387631	30387149	30387550	30387774	30387860	0.020491863	-0.126
127	FBgn0015222	chr3R	-	30387149	30387631	30387149	30387602	30387774	30387860	1.22624E-13	0.232

128	FBgn0015222	chr3R	-	30387463	30387716	30387463	30387550	30387774	30387860	0.026767112	-0.122
129	FBgn0015222	chr3R	-	30387463	30387716	30387463	30387602	30387774	30387860	0	0.202
130	FBgn0083124	chr2R	+	9097061	9097312	9097241	9097312	9096925	9096991	5.11875E-06	0.179
131	FBgn0034538	chr2R	-	20653744	20654199	20653744	20653867	20654969	20655096	0.009982852	0.395
132	FBgn0034538	chr2R	-	20653744	20654225	20653744	20653867	20654969	20655096	0.007759931	0.395
133	FBgn0034538	chr2R	-	20653744	20654238	20653744	20653867	20654969	20655096	0.006847418	0.404
134	FBgn0034515	chr2R	-	20539511	20539676	20539511	20539664	20539744	20539813	0	0.358
135	FBgn0034645	chr2R	+	21658216	21658480	21658350	21658480	21658077	21658109	0.00051078	0.211
136	FBgn0034645	chr2R	+	21658219	21658480	21658350	21658480	21658077	21658109	0.001332025	0.203
137	FBgn0010548	chr2R	-	7473721	7473993	7473721	7473843	7474571	7474659	0.013012627	-0.261
138	FBgn0011260	chr2R	+	16516880	16517341	16517018	16517341	16516149	16516270	0.002727055	0.355
139	FBgn0011260	chr2R	+	16516951	16517341	16517018	16517341	16516149	16516270	0.00312424	0.376
140	FBgn0033313	chr2R	+	8616069	8616516	8616326	8616516	8615902	8616003	0.040185777	-0.178
141	FBgn0034075	chr2R	+	16115115	16115594	16115353	16115594	16114771	16114866	0.001107099	0.123
142	FBgn0022160	chr2R	-	15860376	15860787	15860376	15860722	15861881	15862020	1.41167E-05	-0.162
143	FBgn0022160	chr2R	-	15860376	15860870	15860376	15860722	15861881	15862020	2.16288E-06	-0.189
144	FBgn0022160	chr2R	-	15860599	15860892	15860599	15860722	15861881	15862020	2.16288E-06	-0.189
145	FBgn0022160	chr2R	-	15860599	15861026	15860599	15860722	15861881	15862020	3.04652E-06	-0.187
146	FBgn0034903	chr2R	-	23756363	23757166	23756363	23756542	23757414	23757494	0.010501358	-0.329
147	FBgn0034903	chr2R	-	23756363	23757166	23756363	23756545	23757414	23757494	0.003791097	-0.325
148	FBgn0053013	chr2R	+	12364215	12364300	12364245	12364300	12363984	12364159	0.002000774	0.678
149	FBgn0022382	chr2R	-	10024050	10024297	10024050	10024249	10025828	10026042	0.000731147	-0.584
150	FBgn0036493	chr3L	+	15306504	15306798	15306512	15306798	15305576	15305679	0	0.737
151	FBgn0033373	chr2R	-	9090810	9091230	9090810	9091051	9091406	9091653	0.046980008	0.133
152	FBgn0265356	chr2R	+	19007548	19007746	19007557	19007746	19005788	19006571	2.22979E-05	0.404
153	FBgn0033246	chr2R	-	7985601	7985781	7985601	7985778	7987065	7987237	0.001491801	0.221
154	FBgn0015521	chr2L	+	2856742	2857029	2856768	2857029	2856630	2856672	0	-0.916
155	FBgn0039817	chr3R	-	31099105	31099214	31099105	31099182	31099273	31099321	0	1
156	FBgn0039757	chr3R	-	30208092	30208395	30208092	30208392	30208489	30208942	0.000345494	-0.458
157	FBgn0005666	chr4	+	728334	728814	728672	728814	728103	728245	0.000436152	0.236
158	FBgn0038294	chr3R	-	15175624	15176116	15175624	15175863	15176183	15176293	0.000308129	-0.136
159	FBgn0038294	chr3R	-	15176053	15176432	15176053	15176412	15178279	15178489	2.7058E-05	-0.185
160	FBgn0038294	chr3R	-	15176053	15176432	15176053	15176427	15178279	15178489	6.32899E-14	-0.246
161	FBgn0020493	chr3R	+	17055035	17055174	17055051	17055174	17054006	17054389	0.047968271	-0.321
162	FBgn0034420	chr2R	+	19174338	19174748	19174576	19174748	19173703	19174121	2.1143E-05	0.186
163	FBgn0031857	chr2L	-	6770590	6770718	6770590	6770709	6770885	6770968	0	-1
164	FBgn0031857	chr2L	-	6770590	6770718	6770590	6770709	6772551	6772755	0	-0.992
165	FBgn0031857	chr2L	-	6770590	6770995	6770590	6770709	6772551	6772755	0	-0.927
166	FBgn0003256	chr2R	+	1072267	1072439	1072270	1072439	1071974	1072045	0.034859335	0.26
167	FBgn0039805	chr3R	+	30867894	30868154	30867941	30868154	30867520	30867683	0.01474869	-0.613
168	FBgn0261556	chr3L	-	20156679	20157222	20156679	20156889	20158479	20158668	0.001332025	0.471
169	FBgn0034137	chr2R	+	16566614	16566992	16566795	16566992	16566022	16566262	8.8602E-06	-0.718
170	FBgn0034137	chr2R	+	16566768	16566992	16566795	16566992	16566022	16566262	0.004598513	-0.406
171	FBgn0250819	chr4	+	1186177	1186634	1186472	1186634	1185767	1185834	0.024841556	0.28
172	FBgn0250819	chr4	+	1186177	1186634	1186499	1186634	1185767	1185834	0.002997535	0.382
173	FBgn0250819	chr4	+	1186328	1186634	1186499	1186634	1185767	1185834	0.001790967	0.379
174	FBgn0259209	chr2R	+	24075166	24075337	24075182	24075337	24075011	24075069	0.020491863	-0.307
175	FBgn0259243	chr3L	+	20829079	20829319	20829291	20829319	20828224	20828340	0.001102792	0.611
176	FBgn0259244	chr3R	+	16570940	16571363	16570943	16571363	16569484	16569682	0.005787906	-0.451
177	FBgn0039525	chr3R	-	27560099	27560564	27560099	27560497	27560762	27560875	0.000213124	0.742
178	FBgn0262351	chr3L	+	18925443	18925585	18925458	18925585	18925090	18925387	0.018342023	0.33
179	FBgn0262351	chr3L	+	18925453	18925585	18925458	18925585	18925090	18925387	0.005453799	0.554
180	FBgn0261439	chr2R	-	19395484	19395813	19395484	19395777	19395910	19395971	0.003206225	0.157
181	FBgn0261552	chr3R	+	9439114	9439229	9439124	9439229	9432601	9432839	0.039914432	0.283
182	FBgn0261552	chr3R	+	9439124	9439229	9439141	9439229	9432601	9432839	0.003186667	-0.683

A5SS											
	FlyBase ID	chr	strand	longExonStart_0base	longExonEnd	shortES	shortEE	flankingES	flankingEE	FDR	ΔPSI
1	FBgn0020762	chr2L	+	4333912	4334439	4333912	4334104	4336449	4336957	1.59506E-08	-0.206
2	FBgn0030893	chrX	+	18094589	18094815	18094589	18094754	18095084	18096453	0.001911989	0.378
3	FBgn0000173	chrX	+	13996656	13996797	13996656	13996755	13998094	13999805	0.031397649	0.319
4	FBgn0035335	chr3L	-	2373596	2373678	2373627	2373678	2373128	2373444	0.010466606	-0.514
5	FBgn0042177	chr3L	+	16604005	16604724	16604005	16604721	16604780	16605187	0.003531997	0.44
6	FBgn0265434	chr2R	-	25011311	25011463	25011375	25011463	25010379	25010616	0.000277859	-0.182
7	FBgn0005636	chr2R	+	24284686	24284762	24284686	24284755	24284827	24285074	1.64572E-11	-0.605
8	FBgn0262160	chr2L	-	13026528	13027744	13027065	13027744	13022670	13025497	0.005179512	0.201
9	FBgn0034408	chr2R	+	18947316	18947820	18947316	18947586	18954977	18956126	1.23987E-13	-0.579
10	FBgn0030850	chrX	-	17624263	17625658	17625529	17625658	17623220	17624205	0	0.798
11	FBgn0039790	chr3R	-	30599918	30600323	30600123	30600323	30595389	30595626	5.68272E-13	0.269
12	FBgn0039790	chr3R	-	30599918	30600323	30600263	30600323	30595389	30595626	0	0.78
13	FBgn0003944	chr3R	-	16732697	16734426	16732724	16734426	16725255	16725306	0.012883436	0.173
14	FBgn0020445	chr2L	-	3353838	3354829	3354590	3354829	3352475	3353714	0.000496862	-0.57
15	FBgn0027932	chr2L	+	8427614	8429462	8427614	8428067	8430280	8430977	0.047088279	0.137
16	FBgn0051961	chr2L	-	4187566	4187793	4187710	4187793	4186249	4187499	0.000792003	-0.228
17	FBgn0032889	chr2L	+	20824433	20824724	20824433	20824630	20825271	20825512	0.034025231	-0.41
18	FBgn0025631	chrX	-	2048238	2048395	2048315	2048395	2047557	2047647	0.037893648	-0.344
19	FBgn0050296	chr2R	+	21017783	21017952	21017783	21017912	21019158	21019582	0.000226747	0.218
20	FBgn0036624	chr3L	+	16386326	16386570	16386326	16386494	16386631	16386956	0.005355434	-0.285
21	FBgn0003371	chrX	+	2633951	2634178	2633951	2634025	2634835	2634871	0.001643813	-0.326
22	FBgn0037117	chr3L	+	21680049	21680232	21680049	21680152	21680299	21682433	0.011230643	-0.432
23	FBgn0037117	chr3L	+	21680049	21680232	21680049	21680223	21680299	21682433	0	-0.521
24	FBgn0024733	chr3L	-	23095425	23095471	23095436	23095471	23095274	23095305	0.009480804	0.183
25	FBgn0016070	chr3L	-	8992043	8993041	8992452	8993041	8990316	8991537	1.21762E-06	0.305
26	FBgn0025595	chr2L	-	6712337	6712675	6712346	6712675	6711285	6711742	0.010473981	0.241
27	FBgn0027608	chr3R	-	5776924	5777125	5776951	5777125	5762704	5762755	0.011625642	-0.154
28	FBgn0026401	chr2R	-	4728806	4729017	4728813	4729017	4728432	4728551	0.043579338	-0.331
29	FBgn0260388	chr3L	-	16496052	16496236	16496138	16496236	16495299	16495536	0	0.626
30	FBgn0051038	chr3R	+	29877058	29877178	29877058	29877115	29877926	29879810	3.70189E-11	0.28
31	FBgn0000541	chr3L	-	246427	246912	246885	246912	245927	246323	0.032301062	0.451
32	FBgn0028496	chr2R	-	18497755	18498026	18497841	18498026	18496746	18496868	1.63675E-06	0.677
33	FBgn0262871	chr3R	+	17379943	17380135	17379943	17380051	17384605	17384946	0.013089668	-0.293
34	FBgn0033313	chr2R	+	8615902	8616156	8615902	8616003	8616326	8616516	2.29862E-06	-0.289
35	FBgn0000536	chrX	+	16278356	16278615	16278356	16278547	16279908	16279991	0.000136934	0.406
36	FBgn0033725	chr2R	+	12387381	12387517	12387381	12387427	12387912	12387939	2.20361E-06	0.259
37	FBgn0023531	chrX	-	1692296	1692768	1692438	1692768	1691206	1691357	0.019816935	0.244
38	FBgn0016930	chr2L	-	14229458	14229536	14229531	14229536	14222016	14222081	0.003074377	0.143
39	FBgn0034897	chr2R	+	23713899	23714118	23713899	23714074	23714665	23714899	0.039739047	0.099
40	FBgn0063670	chr3L	-	27083723	27083906	27083736	27083906	27083337	27083593	0.002932822	-0.178
41	FBgn0030674	chrX	+	15713900	15714393	15713900	15714381	15714716	15714956	0.001891049	0.296
42	FBgn0250819	chr4	+	1185767	1185966	1185767	1185834	1186472	1186634	6.43707E-07	0.239
43	FBgn0250819	chr4	+	1185767	1185966	1185767	1185834	1187521	1187614	0.019085225	0.355
44	FBgn0250819	chr4	+	1187521	1187614	1187521	1187572	1187734	1187880	0.015836887	0.115
45	FBgn0259170	chrX	-	10325632	10325958	10325813	10325958	10323565	10324356	0.003830424	0.535
46	FBgn0259170	chrX	-	10325675	10325958	10325813	10325958	10323565	10324356	0.000494928	0.527
47	FBgn0260722	chr3R	-	9551570	9551748	9551700	9551748	9548103	9549810	0.002715133	0.362
48	FBgn0260794	chr3R	-	4806174	4806463	4806361	4806463	4801212	4801991	1.47963E-05	0.746
49	FBgn0260794	chr3R	-	4806256	4806463	4806361	4806463	4801212	4801991	2.6807E-05	0.682
50	FBgn0003502	chr2L	-	8300505	8301079	8300817	8301079	8298070	8298340	0.000208432	0.33
51	FBgn0011603	chr2L	-	4476851	4477022	4476934	4477022	4474331	4475318	0.000358582	0.555
52	FBgn0035889	chr3L	-	8404879	8405277	8405213	8405277	8404102	8404510	0.01664816	0.169
53	FBgn0011206	chr3L	-	9130329	9130608	9130554	9130608	9113045	9113487	0.010431664	0.35
54	FBgn0035987	chr3L	+	9412154	9412411	9412154	9412335	9413202	9413317	0.036058376	0.667
55	FBgn0052085	chr3L	-	11679951	11680344	11680043	11680344	11674267	11675665	0.007627262	-0.252
56	FBgn0052111	chr3L	+	12642046	12642878	12642046	12642361	12644801	12645040	0.000455987	0.833
57	FBgn0036663	chr3L	-	16807411	16807731	16807560	16807731	16799483	16799954	0.000121088	0.873
58	FBgn0052219	chr3L	-	19951002	19951135	19951008	19951135	19950765	19950936	0	0.668
59	FBgn0002590	chrX	-	17147717	17147942	17147727	17147942	17146797	17147200	0.005017557	-0.494
60	FBgn0002590	chrX	-	17147727	17147942	17147791	17147942	17146797	17147200	0.006670197	0.469
61	FBgn0030693	chrX	-	15795839	15796028	15796026	15796028	15794496	15795101	1.3142E-10	-1
62	FBgn0030364	chrX	+	12018639	12019325	12018639	12018940	12019627	12019758	1.68062E-11	-0.327
63	FBgn0010905	chr3L	-	2536285	2536585	2536515	2536585	2534710	2536218	2.79879E-08	0.792
64	FBgn0004636	chr3L	-	1861777	1862239	1861929	1862239	1859114	1861676	0.00319034	0.484
65	FBgn0004636	chr3L	-	1861785	1862239	1861929	1862239	1859114	1861676	0.003983773	0.507
66	FBgn0286213	chr3L	+	13023482	13023870	13023482	13023525	13024369	13024762	0.001266511	0.5
67	FBgn0285952	chr2R	+	24057852	24058351	24057852	24058052	24058483	24058691	0.002593753	-0.463
68	FBgn0284252	chr2R	-	24514991	24515320	24515017	24515320	24514535	24514870	0.032865007	-0.336
69	FBgn0284252	chr2R	-	24515017	24515320	24515286	24515320	24514535	24514870	0.031371844	0.583
70	FBgn0026374	chrX	+	15788896	15789112	15788896	15788957	15790233	15790388	0.001655554	-0.8
71	FBgn0015218	chr3L	-	9402055	9402225	9402170	9402225	9401039	9401367	0.000600934	0.44
72	FBgn0031574	chr2L	+	3863129	3863370	3863129	3863347	3863554	3864195	0.000871907	0.463
73	FBgn0031574	chr2L	+	3863129	3863462	3863129	3863370	3863554	3864195	0.015008663	-0.471

74	FBgn0265045	chr2R	-	15959534	15959876	15959788	15959876	15957993	15959317	0.000586665	0.229
75	FBgn0263593	chr2R	-	8145641	8145981	8145903	8145981	8142642	8143074	4.28227E-06	-0.996
76	FBgn0263593	chr2R	-	8145751	8145981	8145903	8145981	8142642	8143074	0	-0.974
77	FBgn0263593	chr2R	-	8145754	8145981	8145903	8145981	8142642	8143074	0	-0.707
78	FBgn0263391	chr2R	-	19424008	19424114	19424039	19424114	19423593	19423797	6.04036E-10	-1
79	FBgn0031359	chr2L	-	1718683	1718903	1718739	1718903	1718420	1718491	0.028214873	-0.155
80	FBgn0013272	chr2R	+	22317661	22317810	22317661	22317768	22317881	22317954	0.039304126	-0.263
81	FBgn0031453	chr2L	+	2752799	2753118	2752799	2752837	2753371	2753660	1.26074E-07	0.778
82	FBgn0263006	chr2R	-	23935502	23935884	23935824	23935884	23932666	23932814	6.36801E-10	0.2
83	FBgn0262169	chr2R	+	10052540	10052919	10052540	10052657	10057048	10057343	0.010981643	0.69
84	FBgn0039969	chr2R	-	5604476	5604617	5604516	5604617	5604243	5604411	0.000225704	0.467
85	FBgn0025693	chr2R	-	5782698	5782855	5782711	5782855	5778200	5778713	0.003712453	0.702
86	FBgn0025693	chr2R	-	5782698	5782855	5782714	5782855	5778200	5778713	0.001832999	0.637
87	FBgn0082582	chr3R	+	30546405	30546587	30546405	30546546	30548420	30548749	0	0.607
88	FBgn0082582	chr3R	+	30546404	30546587	30546404	30546546	30548451	30548749	0	0.826
89	FBgn0082582	chr3R	+	30546404	30546598	30546404	30546546	30548451	30548749	2.49386E-08	0.186
90	FBgn0264489	chr3L	+	9813846	9813975	9813846	9813922	9820073	9820559	3.66929E-05	-0.669
91	FBgn0027844	chr2L	+	13807326	13807537	13807326	13807425	13808808	13808967	0.014865263	-0.348
92	FBgn0032192	chr2L	-	10209247	10209744	10209457	10209744	10208644	10208802	0.045694258	0.177
93	FBgn0000108	chrX	+	531169	531193	531169	531189	531626	531876	0.042410124	-0.643
94	FBgn0264357	chr3R	+	21156361	21156560	21156361	21156476	21157437	21157657	6.53912E-11	-1
95	FBgn0030850	chrX	-	17624263	17625658	17625370	17625658	17623220	17624205	0	-0.967
96	FBgn0030850	chrX	-	17625370	17625658	17625529	17625658	17623220	17624205	0	0.444
97	FBgn0030850	chrX	-	17625493	17625658	17625529	17625658	17623220	17624205	0	0.499
98	FBgn0003209	chr2L	-	8735490	8735797	8735505	8735797	8734466	8735200	0.007825589	-0.667
99	FBgn0039790	chr3R	-	30599887	30600323	30600123	30600323	30595389	30595626	0.008758856	0.137
100	FBgn0039790	chr3R	-	30599887	30600323	30600263	30600323	30595389	30595626	6.54476E-10	0.633
101	FBgn0039790	chr3R	-	30599908	30600323	30600123	30600323	30595389	30595626	0.014817356	0.134
102	FBgn0039790	chr3R	-	30599908	30600323	30600263	30600323	30595389	30595626	1.37516E-08	0.618
103	FBgn0038721	chr3R	-	19638584	19638750	19638678	19638750	19638266	19638427	0.005264471	0.304
104	FBgn0038721	chr3R	-	19638651	19638750	19638678	19638750	19638266	19638427	0.016130018	0.346
105	FBgn0041605	chr3R	+	4281826	4282309	4281826	4282003	4282673	4282834	0.002632701	0.199
106	FBgn0039897	chr4	+	231425	231668	231425	231600	236394	236475	1.17564E-08	-0.661
107	FBgn0000047	chr3R	+	15439968	15440136	15439968	15440004	15440599	15441540	0.014876877	-0.538
108	FBgn0027579	chr3R	+	27929406	27929896	27929406	27929791	27929980	27930145	0.010669291	-0.308
109	FBgn0038181	chr3R	+	13774311	13774737	13774311	13774698	13775091	13775169	0.000159338	0.664
110	FBgn0038181	chr3R	+	13774800	13775169	13774800	13774837	13775381	13775459	0.030913857	0.566
111	FBgn0031836	chr2L	+	6611405	6611738	6611405	6611675	6612484	6612640	0.023799547	-0.449
112	FBgn0031836	chr2L	+	6611611	6611775	6611611	6611675	6612484	6612640	0.004311278	-0.489
113	FBgn0013305	chr2R	-	12879051	12879253	12879196	12879253	12878384	12878710	0	-0.256
114	FBgn0013305	chr2R	-	12879447	12879656	12879573	12879656	12879196	12879222	0.008182141	0.492
115	FBgn0267661	chr3R	+	4395548	4395749	4395548	4395728	4395889	4396303	0.000363592	0.79
116	FBgn0003462	chr3L	-	11113266	11113740	11113270	11113740	11112280	11112848	0.004054101	0.56
117	FBgn0000056	chr2L	+	14615551	14615998	14615551	14615876	14616302	14616437	1.97168E-08	0.659
118	FBgn0264495	chr3R	+	6406854	6407191	6406854	6407077	6407477	6407774	7.91906E-06	-0.746
119	FBgn0010435	chr2R	-	24982748	24983204	24983050	24983204	24979532	24979661	0.000432921	0.567
120	FBgn0010435	chr2R	-	24982925	24983204	24983050	24983204	24979532	24979661	0.033158255	0.585
121	FBgn0265935	chr2R	-	6867247	6867524	6867481	6867524	6866686	6866941	0.003049946	0.5
122	FBgn0031869	chr2L	-	6936915	6937314	6937178	6937314	6934989	6936697	0.007377475	0.583
123	FBgn0003093	chr3R	+	29039829	29040229	29039829	29040091	29041095	29041425	0.001899313	-0.558
124	FBgn0035001	chr2R	-	24402216	24402680	24402560	24402680	24401045	24401485	1.28697E-09	0.917
125	FBgn0035001	chr2R	-	24402216	24402680	24402560	24402680	24401662	24401771	8.20447E-08	0.484
126	FBgn0035001	chr2R	-	24402323	24402680	24402560	24402680	24401662	24401771	3.37795E-07	0.485
127	FBgn0031187	chrX	+	22455802	22456086	22455802	22456021	22457426	22457551	0.045065777	-0.209
128	FBgn0025702	chr3L	+	22282903	22283149	22282903	22283138	22283202	22284335	0	0.373
129	FBgn0025702	chr3L	+	22282903	22283149	22282903	22283144	22283202	22284335	0	0.378
130	FBgn0061515	chr3L	-	14031307	14031706	14031446	14031706	14030806	14031002	0.023799547	0.584
131	FBgn0040778	chr2R	-	8067125	8067293	8067184	8067293	8066273	8066758	0	-0.993
132	FBgn0040778	chr2R	-	8067342	8067655	8067407	8067655	8067125	8067293	0.036271592	-0.327
133	FBgn0029897	chrX	-	6743949	6744238	6744108	6744238	6743057	6743504	0.047721074	-0.413
134	FBgn0014184	chr2R	-	12174147	12174264	12174196	12174264	12167993	12168203	0.011230643	-0.597
135	FBgn0032378	chr2L	-	11813308	11813780	11813633	11813780	11811593	11812682	7.46277E-05	0.778
136	FBgn0025936	chr4	+	610683	611016	610683	610928	611284	611655	0.000592158	-0.738
137	FBgn0037292	chr3R	-	4987772	4988178	4988121	4988178	4986499	4986886	0.001295998	-0.607
138	FBgn0051313	chr3R	-	14567153	14567280	14567164	14567280	14566579	14567024	0	-0.777
139	FBgn0032801	chr2L	-	19528770	19528996	19528865	19528996	19528087	19528369	0.00020503	0.667
140	FBgn0031145	chrX	+	21035830	21036155	21035830	21036090	21036398	21036470	0.008112395	-0.607
141	FBgn0267002	chr2R	-	16762184	16762443	16762187	16762443	16761869	16762033	0.016382063	-0.424
142	FBgn0003371	chrX	+	2633951	2634430	2633951	2634025	2634835	2634871	0.000513738	-0.345
143	FBgn0052944	chr3R	-	4419106	4419578	4419359	4419578	4415682	4415886	2.37377E-10	0.794
144	FBgn0052944	chr3R	-	4419138	4419578	4419359	4419578	4415682	4415886	3.55788E-13	0.841
145	FBgn0015754	chr2R	+	16181248	16181805	16181248	16181665	16181980	16182090	0.001396553	-0.487
146	FBgn0033564	chr2R	+	10879973	10880085	10879973	10880033	10880327	10880840	0.006169362	0.31
147	FBgn0033807	chr2R	-	13213532	13213931	13213750	13213931	13213022	13213436	0.000283056	-0.13
148	FBgn0051146	chr3R	+	7658246	7658668	7658246	7658510	7662251	7663203	0.013998442	0.403
149	FBgn0015570	chr3R	+	7541809	7542013	7541809	7541992	7542719	7542896	0.000245303	-0.612
150	FBgn0029687	chrX	+	3948974	3949176	3948974	3949172	3949480	3949713	0.001899313	-0.718
151	FBgn0004169	chrX	-	13594830	13595078	13595003	13595078	13593576	13594050	8.39294E-13	-0.28
152	FBgn0004169	chrX	-	13594831	13595078	13595003	13595078	13593576	13594050	1.61642E-12	-0.283
153	FBgn0004169	chrX	-	13601872	13602011	13601980	13602011	13600069	13600117	0.000144706	-0.648
154	FBgn0004169	chrX	-	13602205	13602506	13602413	13602506	13600069	13600117	4.25253E-06	0.767
155	FBgn0053208	chr3R	-	10042571	10042821	10042626	10042821	10041971	10041998	0.018424649	-0.411
156	FBgn0039944	chrX	-	23096553	23096734	23096612	23096734	23088714	23090413	0.049958118	0.496
157	FBgn0039944	chrX	-	23096553	23096735	23096706	23096735	23088714	23090413	2.82859E-08	0.933
158	FBgn0013770	chr2R	+	13965604	13965785	13965604	13965685	13966754	13966969	0.011625642	0.456
159	FBgn0085432	chr4	+	69325	69731	69325	69394	72320	72594	4.67068E-05	-0.72
160	FBgn0085432	chr4	+	107071	107223	107071	107169	107402	114270	0.002409313	-0.526
161	FBgn0032172	chr2L	-	9963377	9963755	9963451	9963755	9962734	9963012	0.028962166	-0.221
162	FBgn0085421	chr2R	-	6776349	6776910	6776582	6776910	6773639	6773786	4.75225E-09	-0.427
163	FBgn0000024	chr3R	-	13258302	13258425	13258304	13258425	13245809	13246654	0	0.489
164	FBgn0029769	chrX	-	5495831	5496052	5495991	5496052	5494058	5495772	0.026163997	-0.512
165	FBgn0039350	chr3R	+	25647245	25647371	25647245	25647306	25647498	25647653	3.38769E-06	0.863
166	FBgn0260388	chr3L	-	16495841	16496236	16496138	16496236	16495299	16495536	0.007825589	0.194
167	FBgn0051038	chr3R	+	29877058	29877192	29877058	29877115	29877926	29879810	3.63427E-11	0.844
168	FBgn0051038	chr3R	+	29877058	29877247	29877058	29877115	29877926	29879810	2.16239E-10	0.787
169	FBgn0027779	chr2L	-	16725891	16726113	16725942	16726113	16724858	16724993	3.06214E-05	0.257
170	FBgn0028412	chr2L	-	11586351	11586628	11586378	11586628	11585432	11586270	1.73039E-11	-0.677
171	FBgn0028956	chr2R	-	17452141	17452598	17452494	17452598	17450528	17451032	3.48816E-11	1
172	FBgn0263120	chr2R	+	8665812	8666071	8665812	8665870	8674646	8674871	0.001771837	-0.342
173	FBgn0019890	chr2L	-	13904025	13904208	13904104	13904208	13903780	13903931	0.042410124	0.211
174	FBgn0042135	chr2R	-	7735153	7735622	7735315	7735622	7729641	7729708	0.002593753	-0.381
175	FBgn0052803	chrX	-	1901667	1901911	1901712	1901911	1901436	1901614	0.048853114	-0.452
176	FBgn0264494	chr2L	+	1742772	1743242	1742772	1743110	1743344	1743540	0.000436895	0.364
177	FBgn0036974	chr3L	+	20348354	20348764	20348354	20348429	20348941	20349206	0.029376868	0.375
178	FBgn0028496	chr2R	-	18497704	18498026	18497841	18498026	18496746	18496868	1.66437E-06	0.688
179	FBgn0000117	chrX	-	1899356	1899524	1899398	1899524	1898979	1899039	0.000479125	-0.728
180	FBgn0004656	chrX	-	8060902	8060974	8060916	8060974	8059866	8060158	0.01815936	0.288
181	FBgn0030089	chrX	+	9109549	9109884	9109549	9109694	9110821	9111005	9.71852E-05	0.258
182	FBgn0030089	chrX	+	9109549	9109897	9109549	9109694	9110821	9111005	1.09136E-05	0.28
183	FBgn0030089	chrX	+	9109549	9109944	9109549	9109694	9110821	9111005	1.42913E-05	0.278
184	FBgn0030089	chrX	+	9109549	9109946	9109549	9109694	9110821	9111005	8.62121E-05	0.286
185	FBgn0034645	chr2R	+	21658123	21658275	21658123	21658180	21658345	21658480	0	0.338
186	FBgn0034645	chr2R	+	21658123	21658275	21658123	21658261	21658345	21658480	0.012992561	0.135
187	FBgn0034645	chr2R	+	21658123	21658275	21658123	21658180	21658350	21658480	0.011031631	0.3
188	FBgn0260943	chr3L	-	17237831	17238212	17237879	17238212	17235351	17236062	0.003046147	0.307
189	FBgn0260943	chr3L	-	17237831	17238212	17237885	17238212	17235351	17236062	1.13989E-10	0.346
190	FBgn0260943	chr3L	-	17237831	17238212	17238094	17238212	17235351	17236062	9.31598E-09	0.515
191	FBgn0260943	chr3L	-	17237879	17238212	17237885	17238212	17235351	17236062	5.95198E-10	0.455
192	FBgn0260943	chr3L	-	17237885	17238212	17238039	17238212	17235351	17236062	9.27868E-09	-0.822
193	FBgn0260943	chr3L	-	17237885	17238212	17238071	17238212	17235351	17236062	9.76856E-08	-0.441
194	FBgn0260943	chr3L	-	17237885	17238212	17238094	17238212	17235351	17236062	0.000979247	-0.212
195	FBgn0053170	chr3L	+	22867115	22867340	22867115	22867318	22867399	22867535	1.66437E-06	0.444
196	FBgn0010548	chr2R	-	7476306	7476641	7476463	7476641	7475439	7475559	0.007808144	0.345
197	FBgn0016131	chr2R	+	16579469	16579755	16579469	16579533	16580841	16581015	0.031397649	-0.165
198	FBgn0004370	chrX	+	11622014	11622255	11622014	11622061	11623752	11624216	0.00308122	0.68
199	FBgn0002773	chr3R	-	30174126	30174551	30174140	30174551	30173571	30173814	0.022309975	-0.312
200	FBgn0002643	chr2R	+	14009198	14009609	14009198	14009238	14010162	14011181	0.040721599	-0.489
201	FBgn0039714	chr3R	-	29867743	29867817	29867752	29867817	29865056	29865399	0.028214873	0.398
202	FBgn0028370	chr2L	+	15552722	15552814	15552722	15552809	15554509	15555030	0.000114449	-0.722
203	FBgn0033799	chr2R	-	13167625	13168032	13167885	13168032	13166423	13166630	0.009264366	0.397
204	FBgn0033799	chr2R	-	13167663	13168032	13167885	13168032	13166423	13166630	0.007808144	0.406
205	FBgn0031717	chr2L	+	5547289	5547756	5547289	5547728	5547807	5547912	1.03327E-07	0.769

206	FBgn0050344	chr2R	+	9147549	9147876	9147549	9147872	9147938	9148095	0	-0.154
207	FBgn0042213	chr3R	+	29725026	29725173	29725026	29725118	29725237	29725690	0.005294044	0.423
208	FBgn0033292	chr2R	-	8440883	8441121	8440916	8441121	8440754	8440824	0.008631791	-0.535
209	FBgn0033246	chr2R	-	7986948	7987237	7987065	7987237	7985601	7985781	4.86746E-06	-0.318
210	FBgn0039890	chr4	-	193338	193489	193393	193489	189997	190061	0.023580204	0.411
211	FBgn0039890	chr4	-	193365	193489	193393	193489	189997	190061	0.008182141	0.601
212	FBgn0039890	chr4	-	193393	193829	193644	193829	189997	190061	0.003983773	-0.8
213	FBgn0003141	chr2L	-	20074389	20074460	20074391	20074460	20074190	20074336	1.96601E-06	-0.744
214	FBgn0035917	chr3L	+	8634582	8635076	8634582	8634783	8638156	8638195	0.010431664	-0.48
215	FBgn0023531	chrX	-	1692313	1692768	1692438	1692768	1691206	1691357	0.015655887	0.283
216	FBgn0022361	chr4	+	558015	558379	558015	558279	560094	560300	0.000262899	-0.667
217	FBgn0022361	chr4	+	558222	558649	558222	558279	560094	560300	0.000149445	-0.667
218	FBgn0010329	chrX	+	7995696	7995797	7995696	7995733	7999270	7999655	0.011751731	0.58
219	FBgn0011695	chr2R	+	24026375	24026498	24026375	24026461	24027003	24027518	6.53912E-11	-0.7
220	FBgn0011695	chr2R	+	24026375	24026871	24026375	24026461	24027003	24027518	0.002296419	-0.508
221	FBgn0033970	chr2R	+	14833142	14833478	14833142	14833475	14833534	14833951	0.004858582	-0.136
222	FBgn0020496	chr3R	+	13011648	13012172	13011648	13012019	13019341	13019814	0.030254384	0.437
223	FBgn0034420	chr2R	+	19174305	19174783	19174305	19174365	19174881	19175086	2.56964E-05	-0.182
224	FBgn0034420	chr2R	+	19176587	19176953	19176587	19176743	19177302	19177573	0.001295998	-0.306
225	FBgn0011361	chr3L	+	1330829	1331170	1330829	1330964	1331279	1331543	7.66424E-07	-0.174
226	FBgn0034073	chr2R	-	16101834	16102050	16101837	16102050	16099693	16101781	0.001232143	-0.26
227	FBgn0043841	chr2L	-	12422960	12423439	12423260	12423439	12421640	12421829	0.000979247	0.611
228	FBgn0043841	chr2L	-	12422960	12423439	12423278	12423439	12421640	12421829	1.45258E-06	0.812
229	FBgn0016977	chr2L	+	160008	160128	160008	160023	162591	163001	0.000455987	-0.249
230	FBgn0034897	chr2R	+	23713899	23714185	23713899	23714074	23714665	23714899	1.21762E-06	0.253
231	FBgn0058191	chr2R	+	1265342	1266052	1265342	1265531	1266191	1266349	2.67705E-10	0.471
232	FBgn0004889	chr3R	-	10134153	10134482	10134436	10134482	10133214	10133587	0.010669291	-0.628
233	FBgn0004889	chr3R	-	10134302	10134482	10134436	10134482	10133214	10133587	0.003042096	-0.654
234	FBgn0004889	chr3R	-	10134302	10134482	10134441	10134482	10133214	10133587	0.009347876	-0.607
235	FBgn0085399	chr2R	-	22039330	22039559	22039403	22039559	22037391	22037716	1.52375E-11	-0.526
236	FBgn0085399	chr2R	-	22039336	22039559	22039403	22039559	22037391	22037716	1.24695E-11	-0.549
237	FBgn0085399	chr2R	-	22039403	22039559	22039481	22039559	22037391	22037716	6.5465E-13	0.879
238	FBgn0085413	chr3R	+	7822034	7822332	7822034	7822317	7836701	7836782	0.028214873	0.615
239	FBgn0034137	chr2R	+	16566022	16566277	16566022	16566262	16566795	16566992	0.032036793	-0.262
240	FBgn0250819	chr4	+	1185767	1186221	1185767	1185834	1186472	1186634	0.003399071	0.316
241	FBgn0259170	chrX	-	10325632	10325958	10325730	10325958	10323565	10324318	0.011625642	0.546
242	FBgn0259176	chr2L	-	12545642	12546611	12546580	12546611	12540515	12540900	0.002314877	0.436
243	FBgn0259176	chr2L	-	12546409	12546611	12546580	12546611	12540515	12540900	0.022309975	0.344
244	FBgn0030701	chrX	+	15870182	15870586	15870182	15870553	15871360	15871523	0.003983773	0.667
245	FBgn0037222	chr3R	-	4242145	4242273	4242174	4242273	4241977	4242087	0.012992561	0.485
246	FBgn0260008	chr3L	+	25120893	25121213	25120893	25120985	25125543	25126171	0.004216986	-0.338
247	FBgn0260008	chr3L	+	25120893	25121352	25120893	25120985	25125543	25126171	3.76724E-09	-0.325
248	FBgn0261239	chr2L	+	21237249	21237567	21237249	21237363	21238280	21238565	0.000665343	-0.822
249	FBgn0016694	chr3L	-	7832553	7833073	7832855	7833073	7830386	7831810	0.04309608	0.429
250	FBgn0016694	chr3L	-	7832553	7833073	7832863	7833073	7830386	7831827	0.004054101	0.34
251	FBgn0039830	chr3R	-	31219109	31219559	31219145	31219559	31218942	31219048	4.12257E-05	-0.194
252	FBgn0039830	chr3R	-	31219145	31219559	31219268	31219559	31218942	31219048	0.049958118	0.509
253	FBgn0039830	chr3R	-	31219145	31219559	31219305	31219559	31218942	31219048	7.76762E-09	0.615
254	FBgn0039830	chr3R	-	31219145	31219559	31219307	31219559	31218942	31219048	2.25186E-09	0.755
255	FBgn0039830	chr3R	-	31219145	31219559	31219388	31219559	31218942	31219048	0.002409313	0.128
256	FBgn0039830	chr3R	-	31219145	31219559	31219410	31219559	31218942	31219048	0	0.907
257	FBgn0039830	chr3R	-	31219173	31219559	31219466	31219559	31218942	31219048	1.09136E-05	0.235
258	FBgn0039830	chr3R	-	31219268	31219559	31219307	31219559	31218942	31219048	0.000979247	0.536
259	FBgn0039830	chr3R	-	31219268	31219559	31219410	31219559	31218942	31219048	3.14003E-10	0.865
260	FBgn0039830	chr3R	-	31219305	31219559	31219410	31219559	31218942	31219048	8.36115E-07	0.537
261	FBgn0039830	chr3R	-	31219410	31219559	31219471	31219559	31218942	31219048	2.71063E-08	-0.778

MXE

	FlyBase ID	chr	strand	1stExonStart_0base	1stExonEnd	2ndExonStart_0base	2ndExonEnd	upstreamES	upstreamEE	downstreamES	downstreamEE	FDR	ΔPSI
1	FBgn0263980	chr2R	+	15458898	15459267	15460872	15461226	15456659	15456815	15463297	15463846	0.00662642	-0.174
2	FBgn0263346	chr3R	+	4670796	4670850	4680778	4680837	4659578	4660399	4697190	4697542	0	-0.478
3	FBgn0263006	chr2R	-	23932573	23932591	23932666	23932814	23932405	23932488	23935579	23935680	0	-0.224
4	FBgn0003944	chr3R	-	16710561	16710612	16725255	16725306	16656622	16659073	16732697	16734426	0.001424537	0.208
5	FBgn0013305	chr2R	-	12879051	12879146	12879196	12879222	12878384	12878710	12879447	12879656	0	0.463
6	FBgn0013305	chr2R	-	12879051	12879146	12879196	12879222	12878384	12878710	12879688	12879798	0	0.328
7	FBgn0051522	chr3R	-	4445331	4445415	4446165	4446246	4444505	4444562	4446424	4446532	0.01607175	-0.111
8	FBgn0264908	chr3L	-	15879319	15879396	15893619	15893961	15869829	15870807	15898834	15898920	0.041683449	0.146
9	FBgn0003261	chr3R	-	6005205	6005297	6005949	6006102	6004016	6005126	6008036	6008573	0.017424251	-0.199
10	FBgn0032456	chr2L	+	12730842	12731066	12737397	12737618	12726745	12727116	12737958	12738176	0.029942522	-0.197
11	FBgn0052982	chr2L	+	9126760	9127466	9135117	9135384	9125736	9125940	9148754	9148799	0.000791618	0.296
12	FBgn0260388	chr3L	-	16493145	16493940	16495299	16495536	16492661	16493000	16496052	16496236	1.65143E-10	0.159
13	FBgn0033159	chr2R	-	7350957	7351081	7357399	7357523	7348130	7348975	7361717	7361867	0.043746998	-0.7
14	FBgn0033159	chr2R	-	7350957	7351081	7357788	7357915	7348130	7348975	7361717	7361867	6.25902E-11	-1
15	FBgn0033159	chr2R	-	7350957	7351081	7358002	7358129	7348130	7348975	7361717	7361867	0.00147731	-0.779
16	FBgn0033159	chr2R	-	7350957	7351081	7359015	7359139	7348130	7348975	7361717	7361867	2.05852E-11	-1
17	FBgn0033159	chr2R	-	7350957	7351081	7359221	7359345	7348130	7348975	7361717	7361867	0.016974461	-0.833
18	FBgn0033159	chr2R	-	7350957	7351081	7360290	7360414	7348130	7348975	7361717	7361867	1.55908E-11	-1
19	FBgn0033159	chr2R	-	7351460	7351584	7358002	7358129	7348130	7348975	7361717	7361867	0.020417638	-0.398
20	FBgn0033159	chr2R	-	7351460	7351584	7359015	7359139	7348130	7348975	7361717	7361867	0.00415885	-0.482
21	FBgn0033159	chr2R	-	7351460	7351584	7360290	7360414	7348130	7348975	7361717	7361867	3.59007E-05	-0.537
22	FBgn0033159	chr2R	-	7351915	7352039	7357399	7357523	7348130	7348975	7361717	7361867	0.002658859	-0.544
23	FBgn0033159	chr2R	-	7351915	7352039	7357590	7357714	7348130	7348975	7361717	7361867	0.012114348	-0.667
24	FBgn0033159	chr2R	-	7351915	7352039	7357788	7357915	7348130	7348975	7361717	7361867	1.19503E-05	-0.832
25	FBgn0033159	chr2R	-	7351915	7352039	7358002	7358129	7348130	7348975	7361717	7361867	0.00011059	-0.683
26	FBgn0033159	chr2R	-	7351915	7352039	7359015	7359139	7348130	7348975	7361717	7361867	1.03639E-07	-0.867
27	FBgn0033159	chr2R	-	7351915	7352039	7359221	7359345	7348130	7348975	7361717	7361867	0.002274679	-0.617
28	FBgn0033159	chr2R	-	7351915	7352039	7360290	7360414	7348130	7348975	7361717	7361867	2.80619E-11	-0.917
29	FBgn0033159	chr2R	-	7358002	7358129	7359441	7359565	7348130	7348975	7361717	7361867	0.002658859	0.466
30	FBgn0033159	chr2R	-	7358215	7358339	7359221	7359345	7348130	7348975	7361717	7361867	0.037694454	-0.667
31	FBgn0033159	chr2R	-	7359015	7359139	7359441	7359565	7348130	7348975	7361717	7361867	0.005133128	0.478
32	FBgn0033159	chr2R	-	7359441	7359565	7360290	7360414	7348130	7348975	7361717	7361867	0.000196006	-0.528
33	FBgn0010548	chr2R	-	7473287	7473346	7473721	7473843	7470701	7470882	7474571	7474659	0.032430213	0.107
34	FBgn0034903	chr2R	-	23759725	23759781	23760621	23760674	23758475	23758602	23760852	23760995	1.69947E-05	0.233
35	FBgn0035348	chr3L	-	2501069	2501141	2502594	2502723	2499690	2500462	2503142	2503606	0.004662197	-0.124
36	FBgn0005666	chr4	+	726661	726683	726931	726962	725668	726029	727460	727623	0.042622251	-0.162
37	FBgn0034420	chr2R	+	19174338	19174365	19174576	19174783	19173703	19174121	19174881	19175086	2.77866E-09	0.21
38	FBgn0034420	chr2R	+	19174576	19174783	19174881	19175248	19173703	19174121	19176247	19176347	9.00119E-09	-0.197
39	FBgn0016930	chr2L	-	14222016	14222081	14229458	14229536	14197656	14197867	14233647	14234108	0.003659156	-0.316
40	FBgn0041182	chr2L	-	7696593	7696807	7697976	7698130	7696027	7696206	7698724	7698992	0.000963366	0.2
41	FBgn0250819	chr4	+	1186472	1186634	1187521	1187614	1185767	1185834	1187734	1187880	0.000481008	-0.199
42	FBgn0250819	chr4	+	1186472	1186634	1187521	1187572	1185767	1185966	1187734	1187880	0.000482661	0.173
43	FBgn0261788	chr3L	-	7656374	7661506	7662470	7662890	7655388	7656203	7663258	7663324	0.011067567	0.158
44	FBgn0035438	chr3L	+	3326522	3326712	3327351	3327553	3326264	3326367	3327859	3328183	0.013738487	-0.132
45	FBgn0035438	chr3L	+	3326522	3326712	3327363	3327553	3326264	3326367	3327859	3328183	0.01386552	-0.132
46	FBgn0035438	chr3L	+	3326586	3326712	3327351	3327553	3326264	3326367	3327859	3328183	0.04400431	-0.123
47	FBgn0035438	chr3L	+	3326586	3326712	3327363	3327553	3326264	3326367	3327859	3328183	0.044282523	-0.123
48	FBgn0285926	chrX	-	10805265	10805456	10805507	10805631	10799750	10799924	10806468	10806856	4.94346E-05	-0.255
49	FBgn0285926	chrX	-	10805265	10805469	10805507	10805631	10799750	10799924	10806468	10806856	4.94346E-05	-0.238
50	FBgn0265313	chr2R	+	9138673	9138805	9139093	9139272	9138485	9138570	9139979	9140298	0.044282523	-0.286
51	FBgn0265313	chr2R	+	9138673	9138805	9139093	9139544	9138485	9138570	9139979	9140298	0.036949784	-0.47
52	FBgn0263980	chr2R	+	15458826	15459267	15460872	15461226	15456659	15456815	15463297	15463846	0.018193889	-0.162
53	FBgn0263980	chr2R	+	15458826	15459267	15460889	15461226	15456659	15456815	15463297	15463846	1.59826E-06	-0.278
54	FBgn0263980	chr2R	+	15458826	15459267	15461025	15461226	15456659	15456815	15463297	15463846	2.79921E-06	-0.273
55	FBgn0263980	chr2R	+	15458868	15459267	15460872	15461226	15456659	15456815	15463297	15463846	0.01607175	-0.163
56	FBgn0263980	chr2R	+	15458868	15459267	15460889	15461226	15456659	15456815	15463297	15463846	2.82045E-07	-0.276
57	FBgn0263980	chr2R	+	15458868	15459267	15461025	15461226	15456659	15456815	15463297	15463846	8.10955E-07	-0.27
58	FBgn0263980	chr2R	+	15458898	15459267	15460889	15461226	15456659	15456815	15463297	15463846	8.88286E-07	-0.255
59	FBgn0263980	chr2R	+	15458898	15459267	15461025	15461226	15456659	15456815	15463297	15463846	1.55436E-06	-0.248
60	FBgn0263980	chr2R	+	15458901	15459267	15460872	15461226	15456659	15456815	15463297	15463846	0.003196333	-0.18
61	FBgn0263980	chr2R	+	15458901	15459267	15460889	15461226	15456659	15456815	15463297	15463846	1.78255E-07	-0.288
62	FBgn0263980	chr2R	+	15458901	15459267	15461025	15461226	15456659	15456815	15463297	15463846	3.19017E-07	-0.282
63	FBgn0034860	chr2R	-	23412965	23413133	23414496	23414976	23412319	23412794	23419226	23419295	0.003025899	-0.333
64	FBgn0263346	chr3R	+	4660235	4660399	4680682	4680837	4659578	4659676	4697190	4697542	1.83508E-10	-0.386
65	FBgn0263346	chr3R	+	4660238	4660399	4680682	4680837	4659578	4659676	4697190	4697542	1.2519E-05	-0.384
66	FBgn0263346	chr3R	+	4670796	4670845	4680778	4680837	4659578	4660399	4697190	4697542	1.29914E-08	-0.339
67	FBgn0263346	chr3R	+	4670808	4670850	4680778	4680837	4660238	4660399	4697190	4697542	0	-0.553
68	FBgn0263346	chr3R	+	4670808	4670850	4680778	4681199	4660238	4660399	4697190	4697542	9.98482E-07	-0.744
69	FBgn0264489	chr3L	+	9829656	9829896	9830459	9830489	9824820	9827895	9830633	9830785	0.002198722	0.573
70	FBgn0027844	chr2L	+	13807326	13807425	13808025	13808222	13803625	13803962	13808808	13808967	0.002198722	0.51
71	FBgn0027844	chr2L	+	13807346	13807425	13808025	13808222	13803625	13803962	13808808	13808967	7.84736E-05	0.685

72	FBgn0051145	chr3R	-	23658756	23659160	23666890	23667081	23653738	23653768	23668218	23668467	0.006649516	0.333
73	FBgn0041605	chr3R	+	4281826	4281930	4282156	4282482	4280384	4280469	4282673	4282834	0.038131851	-0.211
74	FBgn0041605	chr3R	+	4281826	4282003	4282156	4282309	4280384	4280469	4282673	4282834	0.020417638	-0.201
75	FBgn0041605	chr3R	+	4281826	4282003	4282156	4282482	4280384	4280469	4282673	4282834	0.000791644	-0.236
76	FBgn0039897	chr4	+	243815	244007	244417	244755	243633	243748	245179	245903	0.000678315	0.145
77	FBgn0039897	chr4	+	243815	244142	244417	244755	243633	243748	245179	245903	1.83493E-05	0.154
78	FBgn0039897	chr4	+	243815	244142	244439	244834	243633	243748	245179	245903	0.011067567	0.118
79	FBgn0039897	chr4	+	243815	244142	244554	244834	243633	243748	245179	245903	0.010440114	0.119
80	FBgn0038181	chr3R	+	13774919	13775169	13775403	13775463	13774798	13774837	13775529	13776176	0.015074053	0.441
81	FBgn0031836	chr2L	+	6611611	6611675	6611709	6611738	6611010	6611268	6612484	6612640	0.011790728	0.538
82	FBgn0013305	chr2R	-	12879051	12879134	12879196	12879222	12878384	12878710	12879688	12879798	0	0.269
83	FBgn0013305	chr2R	-	12879051	12879134	12879196	12879253	12878384	12878710	12879688	12879798	0	0.499
84	FBgn0013305	chr2R	-	12879051	12879146	12879196	12879253	12878384	12878710	12879688	12879798	2.80619E-11	0.435
85	FBgn0051522	chr3R	-	4445331	4445411	4446165	4446246	4444505	4444562	4446424	4446532	0.006048075	-0.147
86	FBgn0051522	chr3R	-	4446637	4446822	4447700	4447893	4446424	4446532	4462626	4462883	0.01400372	-0.667
87	FBgn0037592	chr3R	+	8518485	8518609	8518946	8519238	8517867	8518391	8519605	8520549	0.001181963	-0.748
88	FBgn0037592	chr3R	+	8518485	8518609	8518946	8519354	8517867	8518391	8519605	8520549	0.000380193	-0.733
89	FBgn0037592	chr3R	+	8518485	8518609	8518949	8519238	8517867	8518391	8519605	8520549	0.000196006	-0.804
90	FBgn0037592	chr3R	+	8518485	8518609	8518949	8519354	8517867	8518391	8519605	8520549	1.92289E-05	-0.821
91	FBgn0037592	chr3R	+	8518485	8518717	8518946	8519238	8517867	8518391	8519605	8520549	0.009417454	-0.733
92	FBgn0037592	chr3R	+	8518485	8518717	8518946	8519354	8517867	8518391	8519605	8520549	0.001809343	-0.667
93	FBgn0037592	chr3R	+	8518485	8518717	8518949	8519238	8517867	8518391	8519605	8520549	0.003444084	-0.79
94	FBgn0037592	chr3R	+	8518485	8518717	8518949	8519354	8517867	8518391	8519605	8520549	0.000318132	-0.75
95	FBgn0037592	chr3R	+	8518485	8518724	8518949	8519354	8517867	8518391	8519605	8520549	0.044282523	-0.246
96	FBgn0261625	chr2R	-	12673610	12673928	12674205	12674702	12673099	12673339	12674990	12675418	0.029942522	-0.115
97	FBgn0261625	chr2R	-	12673610	12673928	12674231	12674702	12673099	12673339	12674990	12675418	0.033998423	-0.593
98	FBgn0261625	chr2R	-	12673898	12673925	12674205	12674702	12673099	12673339	12674990	12675418	0.008000677	-0.177
99	FBgn0261625	chr2R	-	12673898	12673928	12674205	12674702	12673099	12673339	12674990	12675418	0	-0.373
100	FBgn0003261	chr3R	-	6005205	6005297	6005949	6006086	6004016	6005126	6008036	6008573	0.032430213	-0.152
101	FBgn0267428	chr2R	+	438893	439012	459975	460243	432219	432460	495143	495329	0.028359681	0.227
102	FBgn0014184	chr2R	-	12168863	12169254	12169722	12170044	12167993	12168203	12170923	12171285	0.033998423	0.191
103	FBgn0039928	chr4	-	1116139	1116456	1116659	1116792	1115955	1116079	1117850	1117905	0.015793165	0.702
104	FBgn0283471	chrX	-	18113729	18113916	18114443	18114532	18113446	18113569	18114605	18114646	1.83508E-10	0.438
105	FBgn0263772	chrX	+	4076544	4076790	4077874	4079131	4075977	4076426	4082793	4084345	0.006048075	-0.435
106	FBgn0036318	chr3L	+	12851329	12851722	12852426	12852543	12850914	12851153	12852615	12852716	0.048364738	0.169
107	FBgn0031728	chr2L	+	5668748	5668871	5668871	5668881	5662848	5662981	5668942	5670838	1.7642E-05	0.204
108	FBgn0033807	chr2R	-	13213532	13213558	13213750	13213931	13213022	13213436	13214418	13214496	0	0.148
109	FBgn0033807	chr2R	-	13213532	13213558	13213750	13213949	13213022	13213436	13214418	13214496	0	0.215
110	FBgn0004169	chrX	-	13600541	13600949	13601980	13602337	13600069	13600117	13602417	13602578	0.005084333	0.443
111	FBgn0004169	chrX	-	13600579	13600949	13601980	13602163	13600069	13600117	13602417	13602578	0.001004977	0.528
112	FBgn0004169	chrX	-	13600579	13600949	13601980	13602337	13600069	13600117	13602417	13602578	0.000396403	0.583
113	FBgn0004169	chrX	-	13600630	13600949	13601980	13602163	13600069	13600117	13602417	13602578	0.00120337	0.657
114	FBgn0004169	chrX	-	13600630	13600949	13601980	13602337	13600069	13600117	13602417	13602578	1.93435E-05	0.657
115	FBgn0004169	chrX	-	13600873	13600949	13601980	13602163	13600069	13600117	13602417	13602578	0.014127043	0.259
116	FBgn0004169	chrX	-	13600873	13600949	13601980	13602337	13600069	13600117	13602417	13602578	0.005033556	0.325
117	FBgn0261822	chr2L	+	8109296	8109349	8109815	8110216	8107747	8107880	8110480	8116531	0.000196006	0.691
118	FBgn0085432	chr4	+	104327	104401	107075	107223	99405	99475	107402	114270	0.025381108	0.597
119	FBgn0024555	chr3R	+	13684424	13684632	13687425	13687498	13683920	13684150	13687569	13687975	0.017233342	-0.199
120	FBgn0032857	chr2L	-	20399205	20399555	20413049	20413471	20386056	20387592	20414333	20414800	0.036619849	-0.15
121	FBgn0263120	chr2R	+	8666451	8666575	8670621	8670820	8666170	8666311	8674646	8674871	0.031355867	0.559
122	FBgn0263120	chr2R	+	8666451	8666584	8670621	8670820	8666170	8666311	8676471	8676607	0.000824843	-0.667
123	FBgn0263120	chr2R	+	8666466	8666575	8670621	8670820	8666170	8666311	8674646	8674871	0.006565548	0.602
124	FBgn0263120	chr2R	+	8666466	8666575	8672170	8672278	8666170	8666311	8674646	8674871	0.026094393	0.49
125	FBgn0263120	chr2R	+	8674646	8674871	8676471	8676884	8666170	8666311	8678429	8678868	0.005033556	0.187
126	FBgn0263120	chr2R	+	8676952	8677270	8677326	8677785	8676471	8676607	8678429	8678868	0.000149049	-0.889
127	FBgn0263120	chr2R	+	8676952	8677270	8677965	8678186	8676471	8676607	8678429	8678868	0.001676458	-0.278
128	FBgn0263120	chr2R	+	8677064	8677270	8677326	8677785	8676471	8676607	8678429	8678868	0.006825933	-0.833
129	FBgn0263120	chr2R	+	8677064	8677270	8677965	8678186	8676471	8676607	8678429	8678868	0.000481348	-0.365
130	FBgn0011224	chr3R	-	31932071	31932614	31933453	31933644	31873833	31874099	31959034	31959127	0.0441448	-0.389
131	FBgn0037443	chr3R	-	6392875	6392959	6405377	6405548	6391304	6391988	6405741	6405895	0.036103725	0.593
132	FBgn0030087	chrX	-	9099016	9099461	9100512	9100594	9098083	9098413	9101095	9101419	0.009437003	0.158
133	FBgn0030087	chrX	-	9099313	9099461	9100512	9100594	9098083	9098413	9101095	9101419	0.030104691	0.132
134	FBgn0058263	chr2R	-	2515642	2515908	2520578	2520914	2494741	2495023	2521440	2522511	6.62212E-06	0.648
135	FBgn0263077	chr2R	+	7637283	7637408	7638052	7638180	7636894	7637193	7638597	7638905	0.032430213	-0.297
136	FBgn0263077	chr2R	+	7637283	7637408	7638113	7638180	7636894	7637193	7638597	7638905	0.00120337	-0.494
137	FBgn0263077	chr2R	+	7637283	7637408	7638119	7638309	7636894	7637193	7638597	7638905	0.020515494	-0.339
138	FBgn0263077	chr2R	+	7637283	7637408	7638119	7638329	7636894	7637193	7638597	7638905	0.031382768	-0.328
139	FBgn0263077	chr2R	+	7637283	7637408	7638119	7638362	7636894	7637193	7638597	7638905	0.020515494	-0.339
140	FBgn0263077	chr2R	+	7637283	7637408	7638122	7638180	7636894	7637193	7638597	7638905	0.00120337	-0.502
141	FBgn0263077	chr2R	+	7637283	7637408	7638122	7638309	7636894	7637193	7638597	7638905	0.015535497	-0.423
142	FBgn0263077	chr2R	+	7637283	7637408	7638122	7638329	7636894	7637193	7638597	7638905	0.043746998	-0.399
143	FBgn0263077	chr2R	+	7637283	7637408	7638122	7638362	7636894	7637193	7638597	7638905	0.015535497	-0.423
144	FBgn0263077	chr2R	+	7637283	7637541	7638113	7638180	7636894	7637193	7638597	7638905	0.003859598	-0.469
145	FBgn0263077	chr2R	+	7637283	7637541	7638122	7638180	7636894	7637193	7638597	7638905	0.004700886	-0.469
146	FBgn0034075	chr2R	+	16114920	16115053	16115088	16115252	16114771	16114866	16115353	16115594	0	-1
147	FBgn0034075	chr2R	+	16114920	16115053	16115115	16115252	16114771	16114866	16115353	16115594	0	-1
148	FBgn0010352	chr3L	+	16957604	16957851	16958234	16958487	16957438	16957527	16959098	16959216	0.005447044	0.574
149	FBgn0010352	chr3L	+	16957768	16957851	16958234	16958487	16957438	16957527	16959098	16959216	2.30255E-05	0.586
150	FBgn0010352	chr3L	+	16957768	16957851	16958234	16958525	16957438	16957527	16959098	16959216	0.012697082	0.334
151	FBgn0010352	chr3L	+	16957768	16957851	16958923	16959037	16957438	16957527	16959098	16959216	2.30255E-05	0.692
152	FBgn0039261	chr3R	-	25036788	25036866	25037849	25038104	25036622	25036717	25038210	25038318	0.001424537	-0.707
153	FBgn0035348	chr3L	-	2501065	2501141	2502594	2502723	2499690	2500462	2503142	2503606	0.009417454	-0.184
154	FBgn0035348	chr3L	-	2501065	2501141	2502594	2503022	2499690	2500462	2503142	2503606	0.013738487	-0.185
155	FBgn0035348	chr3L	-	2501069	2501141	2502594	2503022	2499690	2500462	2503142	2503606	0.006767329	-0.126
156	FBgn0265356	chr2R	+	19002454	19002780	19004337	19004402	19001091	19001789	19004604	19004889	9.14159E-06	-0.594
157	FBgn0027515	chr2L	+	7821362	7821648	7821685	7822056	7821150	7821308	7822641	7823100	0.00051738	0.187
158	FBgn0027515	chr2L	+	7821362	7821648	7821693	7822056	7821150	7821308	7822641	7823100	0.00045748	0.188
159	FBgn0027515	chr2L	+	7821362	7821648	7821717	7822056	7821150	7821308	7822641	7823100	0.001752015	0.165
160	FBgn0027515	chr2L	+	7821549	7821648	7821685	7822056	7821150	7821308	7822641	7823100	0.000233829	0.211
161	FBgn0027515	chr2L	+	7821549	7821648	7821693	7822056	7821150	7821308	7822641	7823100	0.00019815	0.212
162	FBgn0027515	chr2L	+	7821549	7821648	7821717	7822056	7821150	7821308	7822641	7823100	0.000695166	0.187
163	FBgn0022361	chr4	+	560369	560820	564360	564667	560097	560300	565406	565515	0.010538697	-0.667
164	FBgn0034420	chr2R	+	19174338	19174365	19174576	19174748	19173703	19174121	19176187	19176347	0.000721576	0.223
165	FBgn0011361	chr3L	+	1330829	1330964	1331025	1331170	1330130	1330290	1331279	1331543	1.29596E-12	0.184
166	FBgn0011361	chr3L	+	1330829	1330964	1331029	1331170	1330130	1330290	1331279	1331543	1.43996E-12	0.183
167	FBgn0011361	chr3L	+	1330846	1330964	1331025	1331170	1330130	1330290	1331279	1331543	8.63976E-13	0.189
168	FBgn0011361	chr3L	+	1330846	1330964	1331029	1331170	1330130	1330290	1331279	1331543	8.63976E-13	0.187
169	FBgn0011361	chr3L	+	1330856	1330964	1331025	1331170	1330130	1330290	1331279	1331543	8.02263E-13	0.191
170	FBgn0011361	chr3L	+	1330856	1330964	1331029	1331170	1330130	1330290	1331279	1331543	8.06377E-13	0.19
171	FBgn0011361	chr3L	+	1330862	1330964	1331025	1331170	1330130	1330290	1331279	1331543	0.000325629	0.146
172	FBgn0011361	chr3L	+	1330862	1330964	1331029	1331170	1330130	1330290	1331279	1331543	0.000321019	0.145
173	FBgn0011361	chr3L	+	1330865	1330964	1331025	1331170	1330130	1330290	1331279	1331543	8.63976E-13	0.192
174	FBgn0011361	chr3L	+	1330865	1330964	1331029	1331170	1330130	1330290	1331279	1331543	9.09448E-13	0.191
175	FBgn0016930	chr2L	-	14221791	14222081	14229458	14229536	14217026	14217069	14233647	14234108	0.007288544	-0.283
176	FBgn0016930	chr2L	-	14222030	14222081	14229458	14229536	14217026	14217069	14233647	14234108	0.010034398	-0.296
177	FBgn0016930	chr2L	-	14222030	14222081	14229458	14229536	14217026	14217069	14233893	14234108	0.040664849	0.389
178	FBgn0016930	chr2L	-	14229458	14229536	14233647	14233777	14222016	14222081	14233893	14234108	3.92717E-06	0.259
179	FBgn0034897	chr2R	+	23714547	23714899	23722209	23722601	23713899	23714118	23729048	23729234	0.008150473	0.309
180	FBgn0034897	chr2R	+	23714547	23714899	23722209	23722875	23713899	23714118	23729048	23729234	0.008477315	0.077
181	FBgn0034897	chr2R	+	23714706	23714899	23722209	23722601	23713899	23714118	23729048	23729234	0.005032763	0.316
182	FBgn0034897	chr2R	+	23714706	23714899	23722209	23722875	23713899	23714118	23729048	23729234	0.001424537	0.081
183	FBgn0034897	chr2R	+	23722209	23722875	23729048	23729238	23714665	23714899	23730908	23731091	1.68414E-08	-0.108
184	FBgn0265991	chr2R	-	15766861	15767253	15773040	15773253	15765122	15766713	15773815	15773966	0	-0.376
185	FBgn0020908	chr2R	-	2954910	2955077	2963055	2963171	2937670	2937823	2967094	2967191	5.89165E-11	0.252
186	FBgn0020908	chr2R	-	2955015	2955077	2963055	2963171	2937670	2937823	2967094	2967191	0.034407797	0.152
187	FBgn0041182	chr2L	-	7696373	7696807	7697976	7698130	7696027	7696206	7698724	7698992	0.000958481	0.245
188	FBgn0041182	chr2L	-	7696726	7696807	7697976	7698130	7696027	7696206	7698724	7698992	0.008202756	0.227
189	FBgn0024248	chr2L	-	10246184	10246357	10246403	10246533	10245654	10246039	10246899	10247078	0.012305191	-0.344
190	FBgn0250753	chr3R	-	5599862	5600167	5600507	5600647	5599115	5599364	5601014	5601136	0.015172882	-0.463
191	FBgn0250819	chr4	+	1186472	1186634	1187520	1187614	1185767	1185834	1187734	1187880	0.000662023	-0.199
192	FBgn0250819	chr4	+	1186472	1186634	1187522	1187614	1185767	1185834	1187734	1187880	0.000721576	-0.201
193	FBgn0250819	chr4	+	1186472	1186634	1187520	1187572	1185767	1185966	1187734	1187880	5.51673E-05	0.186
194	FBgn0259243	chr3L	+	20829249	20829319	20829387	20829690	20828224	20828340	20832845	20833205	0.043746998	-0.477
195	FBgn0259243	chr3L	+	20829291	20829319	20829387	20829690	20828224	20828340	20832845	20833205	0.001752015	-0.6
196	FBgn0260008	chr3L	+	25121062	25121352	25122889	25122986	25120893	25120985	25125543	25126171	0.01386552	-0.584
197	FBgn0260008	chr3L	+	25121617	25121745	25122889	25122986	25120893	25120985	25125543	25126171	0.008760908	-0.326
198	FBgn0260008	chr3L	+	25121617	25121968	25122889	25122986	25120893	25120985	25125543	25126171	0.012885395	-0.263
199	FBgn0261552	chr3R	+	9439266	9439705	9440680	9441140	9432601	9432839	9441415	9441665	0.00058407	-0.358
200	FBgn0261552	chr3R	+	9439294	9439705	9440680	9441140	9432601	9432839	9441415	9441665	0.000365168	-0.368
201	FBgn0261552	chr3R	+	9439320	9439705	9440680	9441140	9432601	9432839	9441415	9441665	0.000740775	-0.354
202	FBgn0261552	chr3R	+	9439345	9439705	9440680	9441140	9436834	9437118	9441415	9441665	0.007818369	-0.317

	RI										
	FlyBase ID	chr	strand	riExonStart_0base	riExonEnd	upstreamES	upstreamEE	downstreamES	downstreamEE	FDR	ΔPSI
1	FBgn0286034	chr3R	-	13459670	13463788	13459670	13462250	13462451	13463788	0.002718016	-0.589
2	FBgn0284408	chrX	-	2500562	2500902	2500562	2500676	2500770	2500902	0.000207173	-0.665
3	FBgn0267849	chr3L	+	21445833	21446375	21445833	21446023	21446197	21446375	0	0.207
4	FBgn0267376	chr3R	-	10866808	10868290	10866808	10866960	10867971	10868290	0	0.823
5	FBgn0267376	chr3R	-	10866808	10868642	10866808	10866960	10868438	10868642	0	0.86
6	FBgn0267252	chr2L	+	9256562	9257206	9256562	9256814	9256892	9257206	0	0.578
7	FBgn0267252	chr2L	+	9256562	9272607	9256562	9256814	9272465	9272607	0.025481813	0.274
8	FBgn0265919	chrX	+	18912351	18912752	18912351	18912500	18912584	18912752	0.000766425	0.632
9	FBgn0265540	chr2R	-	17023651	17026444	17023651	17025287	17025352	17026444	0.000609388	0.427
10	FBgn0266137	chr3R	-	29630303	29640310	29630303	29632860	29640228	29640310	0.019267383	-0.497
11	FBgn0265508	chr2R	+	15227405	15230246	15227405	15229627	15229690	15230246	1.09425E-05	0.279
12	FBgn0265487	chr2R	+	17354178	17365386	17354178	17354412	17361979	17365386	0.028088476	0.409
13	FBgn0265045	chr2R	-	15957993	15959876	15957993	15959317	15959788	15959876	0.000287759	0.224
14	FBgn0265042	chr3R	+	23181844	23183060	23181844	23182304	23182945	23183060	0.005186288	0.315
15	FBgn0264945	chr2L	-	2226752	2227604	2226752	2227305	2227363	2227604	0.026188347	-0.386
16	FBgn0264707	chr3L	+	281120	282658	281120	281734	282086	282658	0.000173394	0.391
17	FBgn0264548	chr2L	-	22751303	22751795	22751303	22751519	22751679	22751795	0.002872251	0.471
18	FBgn0264500	chrX	+	10210180	10210636	10210180	10210489	10210564	10210636	0.006199974	-0.487
19	FBgn0264442	chr2L	+	11210677	11211963	11210677	11210725	11211767	11211963	0.016340851	-0.251
20	FBgn0264386	chrX	-	6108029	6111823	6108029	6109946	6111673	6111823	0.004906249	-0.181
21	FBgn0264386	chrX	-	6139605	6146219	6139605	6139717	6145264	6146219	0	0.574
22	FBgn0263980	chr2R	+	15460872	15463846	15460872	15461226	15463297	15463846	0.000185217	-0.231
23	FBgn0263980	chr2R	+	15464470	15465491	15464470	15464686	15465357	15465491	0.001877382	0.299
24	FBgn0263980	chr2R	+	15465330	15465745	15465330	15465491	15465649	15465745	0.037677151	0.147
25	FBgn0263773	chr2L	-	20079269	20085070	20079269	20079472	20084577	20085070	0.033180625	0.199
26	FBgn0263593	chr2R	-	8140242	8140613	8140242	8140371	8140513	8140613	6.23211E-07	-0.152
27	FBgn0263456	chr3L	+	9143526	9144294	9143526	9143866	9143929	9144294	0.047307663	0.174
28	FBgn0263197	chr2R	-	17531210	17531653	17531210	17531446	17531517	17531653	1.02444E-06	-0.57
29	FBgn0263336	chr2R	-	16817518	16818694	16817518	16818531	16818589	16818694	0.001715925	-0.477
30	FBgn0262983	chr3R	-	14236801	14237616	14236801	14236952	14237572	14237616	0.000187359	-0.639
31	FBgn0262897	chr3R	-	6903441	6904682	6903441	6903907	6904065	6904682	0.006137985	0.505
32	FBgn0262794	chr3R	+	17630716	17644892	17630716	17630956	17644777	17644892	5.06893E-05	0.503
33	FBgn0262614	chr3R	-	8844915	8846289	8844915	8845150	8846160	8846289	2.08301E-07	0.79
34	FBgn0262570	chr2R	-	17482790	17483433	17482790	17482839	17482905	17483433	0.037892879	-0.241
35	FBgn0262160	chr2L	-	13022670	13027744	13022670	13025497	13027065	13027744	0.019267383	0.21
36	FBgn0262142	chr2R	+	22666358	22666764	22666358	22666543	22666612	22666764	0.000160276	0.534
37	FBgn0262081	chr3R	-	11642275	11646976	11642275	11643205	11646715	11646976	0.020246968	0.32
38	FBgn0261860	chr3R	-	26783950	26784208	26783950	26784083	26784159	26784208	0.0193085	0.342
39	FBgn0261803	chrX	+	5305878	5306288	5305878	5305995	5306076	5306288	0.017814342	0.218
40	FBgn0261800	chr2L	+	7817732	7820771	7817732	7820400	7820461	7820771	0.000452425	0.097
41	FBgn0261625	chr2R	-	12673099	12674523	12673099	12673339	12674205	12674523	0.000253728	0.18
42	FBgn0261625	chr2R	-	12673099	12675418	12673099	12673339	12674990	12675418	0.022386246	-0.208
43	FBgn0261625	chr2R	-	12673898	12675418	12673898	12673928	12674990	12675418	2.37103E-11	-0.366
44	FBgn0261554	chr2R	+	21301941	21304019	21301941	21302485	21303884	21304019	0.019267383	0.55
45	FBgn0261552	chr3R	+	9434265	9445187	9434265	9434422	9440680	9445187	0.000872875	-0.462
46	FBgn0261552	chr3R	+	9439345	9441665	9439345	9439705	9441415	9441665	0.015570795	0.241
47	FBgn0261341	chr3L	-	19692982	19693571	19692982	19693094	19693499	19693571	3.19943E-08	-0.245
48	FBgn0261341	chr3L	-	19693499	19695042	19693499	19693571	19694972	19695042	1.62484E-06	-0.227
49	FBgn0261243	chr3L	+	1517599	1518052	1517599	1517755	1517986	1518052	0.023178296	-0.244
50	FBgn0260990	chr3R	+	29752138	29752731	29752138	29752267	29752328	29752731	0	-0.231
51	FBgn0260970	chrX	+	7940494	7941195	7940494	7940723	7940782	7941195	0.011037427	0.161
52	FBgn0260945	chr3L	-	12788540	12797856	12788540	12788628	12797682	12797856	1.97736E-06	0.129
53	FBgn0260658	chrX	-	3905736	3907097	3905736	3905897	3906204	3907097	0.019673278	0.427
54	FBgn0260386	chr3R	+	8252052	8258524	8252052	8252221	8258390	8258524	0.01064972	0.611
55	FBgn0260049	chr3L	+	13413005	13413438	13413005	13413073	13413316	13413438	0.002655016	0.565
56	FBgn0259937	chr2R	+	24185714	24186343	24185714	24185767	24186221	24186343	0.032222322	0.213
57	FBgn0259878	chr2R	-	15224321	15232559	15224321	15224446	15232310	15232559	0.009973936	0.191
58	FBgn0259878	chr2R	-	15232310	15233810	15232310	15232559	15233689	15233810	0.007931667	0.246
59	FBgn0259682	chr2R	-	18757628	18758126	18757628	18757977	18758030	18758126	8.42755E-05	0.378
60	FBgn0259682	chr2R	-	18758030	18758284	18758030	18758126	18758224	18758284	0.0002386	0.339
61	FBgn0259231	chrX	-	18707493	18709458	18707493	18709081	18709160	18709458	0.00509435	0.31
62	FBgn0259219	chr2R	+	13262484	13262794	13262484	13262507	13262642	13262794	0.000924658	0.278
63	FBgn0259219	chr2R	+	13262484	13262794	13262484	13262582	13262642	13262794	8.19073E-05	0.307
64	FBgn0261439	chr2R	-	19395686	19395971	19395686	19395777	19395910	19395971	0.00363928	0.132
65	FBgn0259170	chrX	-	10323565	10325958	10323565	10324356	10325813	10325958	0.01094439	0.373
66	FBgn0250867	chr2L	-	20965519	20967746	20965519	20966080	20966303	20967746	1.1752E-07	0.394
67	FBgn0250839	chr3R	-	4956998	4957442	4956998	4957238	4957289	4957442	0.001417737	0.663
68	FBgn0250819	chr4	+	1185767	1186634	1185767	1185834	1186472	1186634	0.002718016	0.289
69	FBgn0243516	chr2R	-	22109586	22110360	22109586	22110023	22110187	22110360	2.89391E-05	0.336
70	FBgn0243516	chr2R	-	22112209	22116342	22112209	22112588	22116164	22116342	0.008063044	0.272
71	FBgn0086906	chr3L	-	2046857	2062271	2046857	2051996	2062046	2062271	0.046448319	0.185
72	FBgn0086906	chr3L	-	2052654	2062271	2052654	2061531	2062046	2062271	0.016139434	0.33
73	FBgn0086855	chr2L	-	276738	277212	276738	276897	276958	277212	0.047830295	-0.506
74	FBgn0086447	chr2L	-	19133022	19133821	19133022	19133351	19133773	19133821	0.001696184	0.623
75	FBgn0265194	chr2R	-	15306877	15308997	15306877	15307154	15308841	15308997	0.023094899	-0.317
76	FBgn0085481	chr3L	-	15686351	15686954	15686351	15686583	15686657	15686954	0.008010922	0.541
77	FBgn0085481	chr3L	-	15686657	15687168	15686657	15686954	15687071	15687168	0	0.86
78	FBgn0085446	chrX	+	6577943	6585785	6577943	6584022	6585692	6585785	0.040952484	0.175
79	FBgn0085421	chr2R	-	6764374	6765697	6764374	6764736	6765535	6765697	0	0.885
80	FBgn0085421	chr2R	-	6769307	6769758	6769307	6769359	6769431	6769758	1.07671E-06	-0.769
81	FBgn0085399	chr2R	-	22037391	22039431	22037391	22037719	22039355	22039431	0.033818212	0.467
82	FBgn0085399	chr2R	-	22037391	22039559	22037391	22037716	22039403	22039559	1.60451E-11	-0.547
83	FBgn0085395	chr2L	-	9370308	9374787	9370308	9373656	9374682	9374787	0.009191144	0.285
84	FBgn0085390	chr2R	-	7850926	7852346	7850926	7851052	7852265	7852346	4.32264E-06	-0.722
85	FBgn0085370	chr2L	-	18532024	18534459	18532024	18534145	18534219	18534459	0.000539916	-0.18
86	FBgn0084001	chr2L	+	22037998	22039183	22037998	22038961	22039036	22039183	0.000816992	0.688
87	FBgn0083984	chr3R	-	21877869	21878419	21877869	21878077	21878232	21878419	0.03845348	0.13
88	FBgn0264494	chr2L	+	1741659	1742260	1741659	1742076	1742142	1742260	0.02253091	-0.116
89	FBgn0082831	chr3R	+	12414351	12416582	12414351	12414756	12414870	12416582	6.95349E-06	-0.229
90	FBgn0082582	chr3R	+	30532606	30533507	30532606	30532644	30533118	30533507	6.60874E-05	0.189
91	FBgn0082582	chr3R	+	30532606	30533507	30532606	30532982	30533118	30533507	0	0.301
92	FBgn0082582	chr3R	+	30546405	30548749	30546405	30546587	30548420	30548749	0	-0.693
93	FBgn0082582	chr3R	+	30546404	30548749	30546404	30546587	30548451	30548749	0	-0.641
94	FBgn0082582	chr3R	+	30572924	30574526	30572924	30573072	30574240	30574526	0	0.248
95	FBgn0082582	chr3R	+	30574240	30576818	30574240	30574526	30576597	30576818	0	0.309
96	FBgn0082582	chr3R	+	30575431	30576818	30575431	30575948	30576597	30576818	4.52712E-12	0.184
97	FBgn0082582	chr3R	+	30572924	30577558	30572924	30573072	30577178	30577558	0	0.267
98	FBgn0082582	chr3R	+	30575431	30577558	30575431	30575948	30577178	30577558	1.40811E-11	0.146
99	FBgn0082582	chr3R	+	30576597	30577558	30576597	30576818	30577178	30577558	0	0.153
100	FBgn0082582	chr3R	+	30572924	30578905	30572924	30573072	30578116	30578905	0	0.252
101	FBgn0064126	chr3R	+	8247708	8248054	8247708	8247894	8247953	8248054	0.04019629	0.419
102	FBgn0265991	chr2R	-	15774427	15777286	15774427	15774757	15777177	15777286	1.22041E-11	0.224
103	FBgn0085419	chr2R	+	18545322	18546181	18545322	18545724	18545890	18546181	0.003343226	0.365
104	FBgn0053513	chrX	-	1483624	1489553	1483624	1486591	1489451	1489553	0.045752569	0.265
105	FBgn0053318	chr2L	+	20923954	20924534	20923954	20924111	20924168	20924534	1.27447E-12	-0.396
106	FBgn0053276	chr3L	-	7640124	7640337	7640124	7640206	7640269	7640337	6.941E-08	0.261
107	FBgn0260722	chr3R	-	9548103	9551748	9548103	9549810	9551700	9551748	0.001979638	0.408
108	FBgn0053092	chr3R	+	21877935	21878308	21877935	21878071	21878171	21878308	0.003020534	-0.369
109	FBgn0053054	chr3L	-	21197759	21202882	21197759	21198103	21202747	21202882	0.003401277	-0.169
110	FBgn0267648	chr3R	-	21356810	21359768	21356810	21357364	21358004	21359768	0.000421636	-0.4
111	FBgn0262166	chr2R	-	16195716	16196333	16195716	16195970	16196066	16196333	4.0971E-08	-0.648
112	FBgn0052284	chr3L	+	3186663	3186899	3186663	3186697	3186759	3186899	0.009886213	-0.178
113	FBgn0052111	chr3L	+	12642046	12645040	12642046	12642878	12644801	12645040	0.005635889	-0.733
114	FBgn0262483	chr3R	+	15381495	15381881	15381495	15381594	15381653	15381881	0.000416654	0.224
115	FBgn0051973	chr2L	-	34719	38731	34719	34912	38534	38731	3.95915E-05	-0.181
116	FBgn0085410	chr2L	+	5851475	5852898	5851475	5851826	5852883	5852898	0.000762104	0.606
117	FBgn0051619	chr2L	+	21684105	21684911	21684105	21684339	21684545	21684911	0.004778518	0.319
118	FBgn0264827	chr3R	-	9810019	9813929	9810019	9810906	9813214	9813929	0.001696184	0.687
119	FBgn0053182	chr2R	-	13176552	13180110	13176552	13179013	13179587	13180110	0.018020785	-0.541
120	FBgn0051100	chr3R	-	9000286	9001886	9000286	9000544	9001768	9001886	7.36577E-09	0.433
121	FBgn0265864	chr2R	+	20562244	20562695	20562244	20562424	20562500	20562695	5.44078E-05	0.564
122	FBgn0050486	chr2R	-	12848033	12848792	12848033	12848346	12848397	12848792	0.000246418	-0.362
123	FBgn0267613	chr3L	+	21194616	21196136	21194616	21195646	21195786	21196136	0.009388636	0.48
124	FBgn0050287	chr2R	-	21537667	21538015	21537667	21537716	21537778	21538015	0.034432431	-0.602
125	FBgn0050082	chr2R	-	15685003	15685701	15685003	15685401	15685519	15685701	0.016757336	-0.485
126	FBgn0083123	chr2R	+	14623919	14624220	14623919	14623978	14624156	14624220	0.001477085	0.533

127	FBgn0083123	chr2R	+	14624156	14624498	14624156	14624220	14624402	14624498	8.27324E-08	0.72
128	FBgn0050021	chr2R	-	11229003	11229542	11229003	11229033	11229410	11229542	0.045047779	0.233
129	FBgn0046253	chr2R	-	23346003	23347641	23346003	23346470	23347296	23347641	0.020135462	0.287
130	FBgn0001222	chr2R	-	18133870	18134634	18133870	18134108	18134406	18134634	2.06584E-05	0.388
131	FBgn0032783	chr2L	-	19437665	19440907	19437665	19437845	19440663	19440907	0.014757725	0.255
132	FBgn0264357	chr3R	+	21180787	21185308	21180787	21181181	21185012	21185308	0.000454743	-0.733
133	FBgn0264357	chr3R	+	21180796	21185308	21180796	21180854	21185012	21185308	0.01823193	-0.423
134	FBgn0260388	chr3L	-	16495299	16496236	16495299	16495536	16496052	16496236	3.40324E-10	-0.796
135	FBgn0260388	chr3L	-	16495299	16496236	16495299	16495536	16496138	16496236	2.21909E-05	0.294
136	FBgn0265959	chr3L	+	20248627	20256638	20248627	20249060	20255897	20256638	0	0.852
137	FBgn0031952	chr2L	-	7802414	7804915	7802414	7803043	7804570	7804915	0	-0.318
138	FBgn0031952	chr2L	-	7801667	7804918	7801667	7803576	7804570	7804918	0	-0.511
139	FBgn0031636	chr2L	+	4840883	4841465	4840883	4840983	4841135	4841465	0.04123389	-0.116
140	FBgn0039187	chr3R	+	24349889	24352351	24349889	24350235	24350726	24352351	0.03759635	0.251
141	FBgn0039736	chr3R	-	30006033	30006519	30006033	30006266	30006336	30006519	0.025481813	-0.405
142	FBgn0033979	chr2R	+	14877785	14879533	14877785	14878886	14878940	14879533	2.56076E-08	0.696
143	FBgn0031395	chr2L	-	2189913	2190784	2189913	2190646	2190706	2190784	0.000566533	-0.28
144	FBgn0083120	chr3L	+	20454394	20454731	20454394	20454457	20454644	20454731	0.044625321	0.589
145	FBgn0031728	chr2L	+	5662848	5668881	5662848	5662981	5668817	5668881	0.002575005	-0.171
146	FBgn0027348	chr2L	+	14013003	14016034	14013003	14013138	14015786	14016034	0.004296068	0.611
147	FBgn0027561	chr2R	-	9125550	9127162	9125550	9126066	9127026	9127162	0.036245317	0.201
148	FBgn0034286	chr2R	-	17977616	17978896	17977616	17977790	17978787	17978896	0.000199704	0.722
149	FBgn0030839	chrX	+	17423590	17426333	17423590	17423856	17426095	17426333	0.008311164	-0.287
150	FBgn0036143	chr3L	+	11174545	11174756	11174545	11174611	11174685	11174756	0.020246968	-0.277
151	FBgn0051674	chr2L	+	20831261	20831878	20831261	20831395	20831466	20831878	0.002178205	-0.286
152	FBgn0030600	chrX	-	15058064	15059737	15058064	15059184	15059350	15059737	0.003540462	0.315
153	FBgn0027580	chr2R	-	9852547	9853730	9852547	9853205	9853682	9853730	0.005186288	0.314
154	FBgn0005536	chr3L	+	16075473	16076648	16075473	16075764	16076229	16076648	0.000129556	0.235
155	FBgn0030670	chrX	-	15702812	15703318	15702812	15703087	15703183	15703318	0.01836984	-0.469
156	FBgn0260776	chr3L	+	15717818	15718242	15717818	15718047	15718097	15718242	0.000420965	0.177
157	FBgn0260776	chr3L	+	15718097	15718435	15718097	15718242	15718303	15718435	7.2563E-05	0.156
158	FBgn0260776	chr3L	+	15718303	15718650	15718303	15718435	15718494	15718650	7.94317E-05	0.154
159	FBgn0260776	chr3L	+	15718494	15718934	15718494	15718650	15718702	15718934	6.01481E-05	0.159
160	FBgn0265498	chr2R	+	13854658	13855894	13854658	13854924	13854982	13855894	0.036882748	-0.426
161	FBgn0034091	chr2R	-	16205900	16206227	16205900	16206079	16206171	16206227	0.011826912	-0.105
162	FBgn0034091	chr2R	-	16205900	16210397	16205900	16206079	16210189	16210397	3.58194E-09	-0.224
163	FBgn0264822	chr4	+	959674	962692	959674	960038	962400	962692	0.045752569	0.372
164	FBgn0038167	chr3R	+	13676688	13678212	13676688	13676932	13677015	13678212	0.000950299	0.482
165	FBgn0034645	chr2R	+	21658123	21658480	21658123	21658275	21658345	21658480	0.001417737	-0.164
166	FBgn0034645	chr2R	+	21658077	21658480	21658077	21658109	21658350	21658480	0.001534957	0.208
167	FBgn0264894	chr2L	+	13274989	13275838	13274989	13275061	13275587	13275838	0.028088476	0.177
168	FBgn0262539	chr3R	-	22769774	22770307	22769774	22770235	22770292	22770307	0.001703153	0.356
169	FBgn0033500	chr2R	+	10249219	10254025	10249219	10249439	10252957	10254025	0	-0.764
170	FBgn0033183	chr2R	-	7493805	7495461	7493805	7494797	7494865	7495461	0.00010134	0.204
171	FBgn0031514	chr2L	-	3277828	3279470	3277828	3277981	3279253	3279470	0.009099537	0.506
172	FBgn0028490	chr2L	-	11524567	11526012	11524567	11525667	11525916	11526012	0.014757725	-0.396
173	FBgn0028490	chr2L	-	11524567	11528205	11524567	11525667	11528120	11528205	3.95915E-05	0.445
174	FBgn0028425	chr2L	-	12053981	12054498	12053981	12054084	12054302	12054498	0.026421859	-0.119
175	FBgn0033777	chr2R	-	12850098	12850948	12850098	12850228	12850774	12850948	0.01991938	0.228
176	FBgn0028424	chr2R	+	16289951	16291333	16289951	16290751	16290927	16291333	0.003136901	0.093
177	FBgn0028412	chr2L	-	11585432	11586628	11585432	11586270	11586351	11586628	0	0.198
178	FBgn0035612	chr3L	-	5543618	5545101	5543618	5543947	5544971	5545101	0.038574817	0.207
179	FBgn0002962	chr3R	+	19157237	19157972	19157237	19157539	19157596	19157972	0.0122901	-0.312
180	FBgn0028703	chr2L	-	6681463	6682084	6681463	6681772	6681942	6682084	0	0.913
181	FBgn0032600	chr2L	+	16743404	16744067	16743404	16743570	16743759	16744067	0	0.372
182	FBgn0264489	chr3L	+	9813846	9820559	9813846	9813922	9820073	9820559	0.030361967	-0.504
183	FBgn0004360	chr2R	+	9494165	9501588	9494165	9495253	9501375	9501588	0.001203107	0.741
184	FBgn0027790	chr3L	+	1821806	1822903	1821806	1822098	1822393	1822903	0.045047779	0.218
185	FBgn0033194	chr2R	+	7566808	7567364	7566808	7566999	7567145	7567364	0.001120181	0.429
186	FBgn0032821	chr2L	-	19777081	19779330	19777081	19778946	19779017	19779330	1.48884E-06	0.91
187	FBgn0024556	chr2R	+	13507556	13509332	13507556	13507658	13507714	13509332	0	0.24
188	FBgn0261788	chr3L	-	7655388	7662298	7655388	7656203	7661980	7662298	0.001581401	0.511
189	FBgn0261788	chr3L	-	7655388	7663324	7655388	7656203	7663258	7663324	0.021494151	0.58
190	FBgn0052971	chr2L	+	14277210	14278002	14277210	14277400	14277461	14278002	2.70931E-12	0.711
191	FBgn0011566	chrX	-	2608170	2608876	2608170	2608412	2608470	2608876	0.026421859	-0.169
192	FBgn0026083	chrX	+	4120097	4120424	4120097	4120138	4120295	4120424	0.025600967	0.756
193	FBgn0030364	chrX	+	12018639	12019758	12018639	12019325	12019627	12019758	0	0.675
194	FBgn0001624	chrX	+	11369664	11374510	11369664	11369799	11374256	11374510	0.004440572	0.189
195	FBgn0029763	chrX	+	5421532	5423017	5421532	5421667	5422579	5423017	0.045396015	0.222
196	FBgn0037222	chr3R	-	4241199	4241919	4241199	4241698	4241754	4241919	1.03382E-10	-0.657
197	FBgn0261059	chr3L	-	21683776	21684042	21683776	21683964	21684018	21684042	0.015110926	0.632
198	FBgn0035793	chr3L	+	7515640	7516050	7515640	7515777	7515870	7516050	0.042911522	0.18
199	FBgn0052442	chr3L	-	21528957	21529333	21528957	21529085	21529145	21529333	9.89119E-06	-0.16
200	FBgn0001149	chr3R	-	12367816	12369268	12367816	12368593	12369202	12369268	0.002035335	-0.265
201	FBgn0062928	chr3L	-	19486744	19487249	19486744	19487106	19487161	19487249	0.006163918	0.186
202	FBgn0062928	chr3L	-	19487674	19488439	19487674	19487767	19487969	19488439	0.00012925	0.29
203	FBgn0027596	chr2R	-	14910223	14911372	14910223	14910716	14911103	14911372	0	-0.176
204	FBgn0004907	chr2R	+	10099676	10104409	10099676	10099738	10104255	10104409	0.002857466	0.549
205	FBgn0004907	chr2R	+	10101035	10104409	10101035	10101097	10104255	10104409	0.023557078	0.355
206	FBgn0003748	chr2R	+	21074017	21077139	21074017	21074112	21076652	21077139	0.001482765	0.36
207	FBgn0010501	chr2R	+	23552300	23553547	23552300	23552863	23552923	23553547	0.028654003	0.122
208	FBgn0032780	chr2L	+	19426297	19430270	19426297	19426698	19429401	19430270	0.038868668	0.511
209	FBgn0260794	chr3R	-	4801212	4806463	4801212	4801991	4806361	4806463	3.58813E-06	0.758
210	FBgn0039710	chr3R	-	29854694	29855648	29854694	29855043	29855129	29855648	0.002359993	-0.449
211	FBgn0264703	chr3L	-	15100053	15102040	15100053	15100640	15100986	15102040	0.010125609	0.442
212	FBgn0000454	chr3R	+	13782287	13782841	13782287	13782451	13782707	13782841	4.41243E-06	-0.342
213	FBgn0004838	chr2L	+	6920703	6924400	6920703	6920861	6923783	6924400	0.000264515	-0.539
214	FBgn0039271	chr3R	+	25069926	25070914	25069926	25070050	25070202	25070914	0	0.722
215	FBgn0013576	chr3R	-	5274965	5287320	5274965	5275087	5286901	5287320	0.01536109	-0.135
216	FBgn0032801	chr2L	-	19528087	19530305	19528087	19528369	19530089	19530305	0.047647437	0.29
217	FBgn0027779	chr2L	-	16724858	16726113	16724858	16724993	16725891	16726113	0.002096439	-0.163
218	FBgn0005666	chr4	+	725668	726683	725668	726029	726661	726683	0.00113095	0.158
219	FBgn0005666	chr4	+	726661	727623	726661	726683	727460	727623	0.012581706	0.186
220	FBgn0005666	chr4	+	728103	728814	728103	728245	728672	728814	1.85637E-06	0.294
221	FBgn0005666	chr4	+	728103	731000	728103	728245	730776	731000	0.019939618	0.212
222	FBgn0011224	chr3R	-	31873833	31933799	31873833	31874099	31933453	31933799	1.09425E-05	0.671
223	FBgn0011224	chr3R	-	31921632	31959127	31921632	31921674	31959034	31959127	0.033858672	0.306
224	FBgn0037998	chr3R	-	12213791	12217546	12213791	12215953	12216880	12217546	0.000109753	-0.242
225	FBgn0051635	chr2L	-	6645971	6649114	6645971	6646947	6649029	6649114	0.000478804	-0.38
226	FBgn0261046	chr3R	-	17480210	17480751	17480210	17480366	17480442	17480751	5.84909E-05	-0.33
227	FBgn0034093	chr2R	-	16226567	16230402	16226567	16226804	16230077	16230402	8.11505E-05	0.429
228	FBgn0023095	chr3L	+	13251153	13279642	13251153	13251211	13275561	13279642	0.002955031	-0.659
229	FBgn0011745	chr3R	+	12691742	12692707	12691742	12691869	12692642	12692707	0.002245084	0.108
230	FBgn0003890	chr3R	-	27969382	27973150	27969382	27969550	27972920	27973150	0.006482975	0.324
231	FBgn0259243	chr3L	+	20825973	20833205	20825973	20826122	20832845	20833205	0.041701658	0.387
232	FBgn0013305	chr2R	-	12878384	12879146	12878384	12878710	12879051	12879146	0	0.756
233	FBgn0003371	chrX	+	2633951	2634871	2633951	2634025	2634835	2634871	0.002644433	-0.264
234	FBgn0000546	chr2R	-	6156157	6157129	6156157	6156414	6156929	6157129	2.10054E-05	-0.733
235	FBgn0038181	chr3R	+	13771596	13772410	13771596	13771649	13772171	13772410	0.00065005	-0.098
236	FBgn0038181	chr3R	+	13771915	13772410	13771915	13772021	13772171	13772410	1.08372E-06	-0.134
237	FBgn0038181	chr3R	+	13774311	13774737	13774311	13774386	13774452	13774737	0	0.165
238	FBgn0032024	chr2L	+	8342694	8343841	8342694	8342989	8343382	8343841	1.87438E-05	-0.694
239	FBgn0052230	chr3L	+	23339325	23339476	23339325	23339369	23339429	23339476	1.12844E-06	0.161
240	FBgn0028496	chr2R	-	18496746	18498026	18496746	18496868	18497841	18498026	5.80569E-10	0.54
241	FBgn0024248	chr2L	-	10246184	10247078	10246184	10246533	10246899	10247078	0.015570795	0.352
242	FBgn0039944	chrX	-	23088714	23096735	23088714	23090413	23096706	23096735	0.007538398	0.611
243	FBgn0264371	chr2L	-	3588484	3590301	3588484	3589571	3589684	3590301	3.15815E-05	-0.776
244	FBgn0032169	chr2L	+	9954595	9956176	9954595	9955513	9955573	9956176	0.005111504	0.231
245	FBgn0264561	chr3L	-	21503990	21504939	21503990	21504359	21504541	21504939	0.03304818	-0.133
246	FBgn0000928	chrX	+	2720467	2723125	2720467	2720849	2720908	2723125	0.001321399	0.334
247	FBgn0265376	chr3R	-	4596282	4598871	4596282	4597174	4598468	4598871	0	0.694
248	FBgn0002626	chr3R	-	30045686	30046140	30045686	30045786	30045861	30046140	6.77352E-05	0.485
249	FBgn0002626	chr3R	-	30045686	30046068	30045686	30045786	30045986	30046068	0.000111882	-0.457
250	FBgn0032156	chr2L	-	9904826	9905971	9904826	9905052	9905874	9905971	0.031954989	0.211
251	FBgn0034368	chr2R	-	18631132	18631902	18631132	18631635	18631749	18631902	0.042085248	-0.242
252	FBgn0043070	chr2R	+	21502096	21507722	21502096	21502479	21507294	21507722	0.000225083	-0.135
253	FBgn0043070	chr2R	+	21502096	21507722	21502096	21502479	21507321	21507722	0.030458822	-0.152
254	FBgn0043070	chr2R	+	21512092	21512725	21512092	21512177	21512392	21512725	1.52308E-08	-0.239
255	FBgn0010622	chr2R	-	23061419	23062593	23061419	23061594	23061682	23062593	0.000565122	-0.308
256	FBgn0004244	chr3L	-	9168799	9171090	9168799	9168867	9170992	9171090	2.00467E-05	0.4
257	FBgn0038840	chr3R	+	20906751	20907153	20906751	20906974	20907037	20907153	0.022036147	0.141
258	FBgn0285917	chr2R	-	18289534	18306135	18289534	18289850	18306030	18306135	0.000556761	-0.501

259	FBgn0010225	chr3R	+	4236787	4237210	4236787	4236939	4236999	4237210	0.040421269	0.311
260	FBgn0035432	chr3L	+	3309792	3310706	3309792	3309927	3310519	3310706	0.002173949	-0.598
261	FBgn0037993	chr3R	-	12156108	12158595	12156108	12156605	12158533	12158595	0.018099719	0.558
262	FBgn0020503	chr2L	+	17401112	17401581	17401112	17401290	17401518	17401581	0.025091284	0.14
263	FBgn0053200	chr2R	+	22408524	22409198	22408524	22408723	22408782	22409198	0.002879519	-0.58
264	FBgn0038397	chr3R	-	16063827	16065575	16063827	16064676	16065374	16065575	0.044840327	-0.186
265	FBgn0010482	chr2R	-	6722751	6723459	6722751	6722908	6723294	6723459	0.014757725	0.417
266	FBgn0031857	chr2L	-	6765970	6767013	6765970	6766526	6766585	6767013	0.002738771	0.1
267	FBgn0031857	chr2L	-	6768845	6769736	6768845	6769127	6769397	6769736	7.8757E-05	0.33
268	FBgn0031857	chr2L	-	6769397	6770081	6769397	6769736	6769952	6770081	0	0.194
269	FBgn0031857	chr2L	-	6770590	6770968	6770590	6770709	6770885	6770968	1.3079E-05	-0.251
270	FBgn0031857	chr2L	-	6770590	6772614	6770590	6770709	6772551	6772614	0	-0.936
271	FBgn0005632	chr3R	+	31777417	31778758	31777417	31777564	31778171	31778758	0.000290289	0.454
272	FBgn0033494	chr2R	-	10150855	10152422	10150855	10151052	10152377	10152422	0.04060897	0.342
273	FBgn0250732	chr3R	-	7146944	7147378	7146944	7147092	7147330	7147378	0.000351764	0.161
274	FBgn0033373	chr2R	-	9090810	9091653	9090810	9091051	9091406	9091653	4.30297E-12	0.244
275	FBgn0032833	chr2L	+	19958831	19959691	19958831	19958909	19959266	19959691	4.59735E-05	-0.228
276	FBgn0011763	chr2R	+	13223590	13225624	13223590	13223660	13225466	13225624	1.62435E-07	-0.548
277	FBgn0003429	chr3R	+	24691777	24692316	24691777	24691933	24692203	24692316	2.96804E-05	0.263
278	FBgn0015221	chr3R	+	30389198	30389442	30389198	30389328	30389415	30389442	0	-0.362
279	FBgn0032172	chr2L	-	9960449	9962059	9960449	9961614	9961728	9962059	0.016410849	-0.207
280	FBgn0037344	chr3R	-	5580723	5584835	5580723	5584678	5584779	5584835	0.042444606	-0.643
281	FBgn0000182	chr2L	+	16044422	16044857	16044422	16044472	16044787	16044857	0.002064474	0.273
282	FBgn0028740	chr2R	-	17770404	17772256	17770404	17771767	17771881	17772256	0.003866775	-0.492
283	FBgn0010226	chr2R	-	17094315	17094696	17094315	17094449	17094516	17094696	0.000164048	-0.154
284	FBgn0020912	chr3R	+	30915939	30923558	30915939	30916721	30923402	30923558	0.037684181	-0.484
285	FBgn0053098	chr3R	+	12387187	12387637	12387187	12387286	12387530	12387637	0.018411034	0.168
286	FBgn0053098	chr3R	+	12387187	12387637	12387187	12387286	12387543	12387637	2.71611E-11	0.247
287	FBgn0026206	chrX	+	9163254	9166459	9163254	9163290	9165681	9166459	0.033689232	0.241
288	FBgn0030850	chrX	-	17623220	17625658	17623220	17624205	17625529	17625658	0	0.82
289	FBgn0030850	chrX	-	17624263	17627240	17624263	17625658	17626939	17627240	1.10102E-07	0.103
290	FBgn0003091	chr2R	+	16924924	16926807	16924924	16925037	16926345	16926807	0.023775085	0.313
291	FBgn0003091	chr2R	+	16925126	16926807	16925126	16925745	16926345	16926807	0.000239933	0.73
292	FBgn0003187	chr2L	-	17489692	17497208	17489692	17489902	17496734	17497208	0.000139895	0.536
293	FBgn0027101	chr4	-	1229579	1230420	1229579	1230118	1230305	1230420	0.009949064	0.176
294	FBgn0010222	chr3R	-	9042851	9049247	9042851	9043639	9049156	9049247	0.032222322	0.191
295	FBgn0028582	chr3L	+	7535542	7536435	7535542	7535644	7535883	7536435	0.002021531	0.201
296	FBgn0028582	chr3L	+	7535542	7538279	7535542	7535644	7536975	7538279	0.01670201	0.133
297	FBgn0266225	chr2L	-	21869955	21870652	21869955	21870418	21870487	21870652	0.013580354	0.589
298	FBgn0032836	chr2L	+	19964135	19964365	19964135	19964281	19964338	19964365	0	0.235
299	FBgn0032836	chr2L	+	19964338	19964581	19964338	19964365	19964425	19964581	0	0.21
300	FBgn0032836	chr2L	+	19964425	19964775	19964425	19964581	19964643	19964775	0	0.171
301	FBgn0051010	chr3R	-	30704761	30706121	30704761	30705989	30706054	30706121	2.37967E-10	-0.546
302	FBgn0266324	chr2L	+	7220566	7221093	7220566	7220647	7220704	7221093	0.031176698	-0.354
303	FBgn0003261	chr3R	-	6004016	6006102	6004016	6005126	6005949	6006102	0.018282641	0.198
304	FBgn0003261	chr3R	-	6005949	6008573	6005949	6006102	6008036	6008573	0.03845348	0.23
305	FBgn0033749	chr2R	-	12512681	12513508	12512681	12512783	12513359	12513508	0.009111546	-0.461
306	FBgn0035348	chr3L	-	2499690	2502815	2499690	2500462	2502390	2502815	0.001011265	0.21
307	FBgn0035348	chr3L	-	2499690	2503606	2499690	2500462	2503142	2503606	0.005089406	0.167
308	FBgn0033339	chr2R	+	8893612	8894141	8893612	8893737	8893925	8894141	3.92286E-07	-0.169
309	FBgn0264693	chr3L	-	4131955	4133822	4131955	4132203	4133548	4133822	5.60234E-05	-0.455
310	FBgn0266596	chr3L	+	9695042	9695650	9695042	9695272	9695325	9695650	0.019145245	-0.212
311	FBgn0003380	chrX	-	17953222	17962251	17953222	17953346	17961632	17962251	0.0483869	-0.223
312	FBgn0051522	chr3R	-	4444505	4446246	4444505	4444562	4446165	4446246	0.034682825	0.133
313	FBgn0001991	chr2L	+	16173318	16173823	16173318	16173544	16173717	16173823	0	0.217
314	FBgn0003149	chr3L	-	8738639	8738907	8738639	8738747	8738816	8738907	8.19073E-05	0.097
315	FBgn0033313	chr2R	+	8615902	8616516	8615902	8616003	8616326	8616516	0.006244564	-0.164
316	FBgn0032079	chr2L	-	8953480	8955807	8953480	8955586	8955681	8955807	0.024114223	0.436
317	FBgn0039970	chr2R	+	5609012	5610256	5609012	5609489	5610063	5610256	4.64531E-10	0.232
318	FBgn0004401	chr3L	-	17643978	17645036	17643978	17644079	17644890	17645036	0.000458015	0.36
319	FBgn0027111	chr3L	+	267298	268459	267298	267391	268219	268459	0.001191929	0.489
320	FBgn0039240	chr3R	-	24874992	24876654	24874992	24875999	24876246	24876654	0.006137985	0.19
321	FBgn0031628	chr2L	+	4795842	4796219	4795842	4795975	4796092	4796219	0	0.822
322	FBgn0263220	chrX	-	10254002	10255590	10254002	10254307	10255492	10255590	0.014079636	0.286
323	FBgn0037941	chr3R	+	11721733	11727686	11721733	11722457	11727014	11727686	0.012507774	0.545
324	FBgn0058178	chr3L	-	26008060	26010354	26008060	26008177	26010106	26010354	0.012486029	0.305
325	FBgn0058178	chr3L	-	26010106	26013721	26010106	26010354	26013542	26013721	0.044347782	0.311
326	FBgn0266633	chr2R	+	12868934	12869783	12868934	12869007	12869223	12869783	0.014362914	-0.188
327	FBgn0024330	chr3R	-	9767176	9768502	9767176	9767878	9768363	9768502	0.00098868	0.201
328	FBgn0033504	chr2R	+	10296434	10304930	10296434	10300268	10304623	10304930	0.044625321	0.219
329	FBgn0263120	chr2R	+	8670722	8676607	8670722	8670820	8674646	8676607	0.001430203	0.356
330	FBgn0263120	chr2R	+	8666170	8676607	8666170	8666311	8676471	8676607	0.000189531	0.233
331	FBgn0036039	chr3L	+	9728503	9729650	9728503	9728614	9728712	9729650	0	0.882
332	FBgn0037130	chr3L	-	21760957	21770534	21760957	21761265	21770445	21770534	0.000219205	0.294
333	FBgn0028956	chr2R	-	17450528	17451296	17450528	17451032	17451088	17451296	0.002173949	0.492
334	FBgn0010611	chr2R	+	16338301	16341242	16338301	16338421	16339446	16341242	0.013095026	0.41
335	FBgn0034837	chr2R	-	23246609	23247893	23246609	23247725	23247777	23247893	0.042109466	0.17
336	FBgn0010348	chr3L	-	22869841	22871559	22869841	22870150	22871319	22871559	0.003989232	0.267
337	FBgn0031228	chr2L	+	155334	155784	155334	155429	155545	155784	0.048193106	0.097
338	FBgn0035104	chr3L	+	179922	181088	179922	179991	180739	181088	0.005916374	0.581
339	FBgn0263077	chr2R	+	7628796	7630328	7628796	7629112	7630176	7630328	0.016662855	0.232
340	FBgn0032914	chr2L	+	21091226	21094637	21091226	21091368	21094408	21094637	0.013456947	0.623
341	FBgn0011817	chr3L	+	8045890	8047161	8045890	8045946	8046569	8047161	0	0.429
342	FBgn0033307	chr2R	+	8590496	8591084	8590496	8590590	8591022	8591084	3.65347E-13	0.959
343	FBgn0033307	chr2R	+	8591022	8591948	8591022	8591084	8591564	8591948	0.019008787	0.195
344	FBgn0031717	chr2L	+	5548167	5549076	5548167	5548709	5549016	5549076	0.000804325	0.267
345	FBgn0031717	chr2L	+	5549016	5549218	5549016	5549076	5549135	5549218	0.013667136	-0.174
346	FBgn0037592	chr3R	+	8519040	8520549	8519040	8519169	8519605	8520549	0.045982482	0.193
347	FBgn0266748	chr3R	+	16992466	16992976	16992466	16992503	16992870	16992976	0.016344237	-0.444
348	FBgn0039465	chr3R	-	26963157	26964285	26963157	26963520	26964117	26964285	0	0.541
349	FBgn0015222	chr3R	-	30387463	30387860	30387463	30387550	30387774	30387860	0.000478804	-0.149
350	FBgn0038097	chr3R	-	12966552	12967378	12966552	12966748	12967159	12967378	0.000402931	0.7
351	FBgn0259246	chr2R	+	9507862	9510004	9507862	9508253	9509887	9510004	0.047337674	-0.45
352	FBgn0033639	chr2R	-	11650814	11651616	11650814	11651224	11651323	11651616	5.41254E-13	0.225
353	FBgn0034858	chr2R	+	23386520	23387612	23386520	23386622	23387006	23387612	2.87276E-08	-0.264
354	FBgn0028370	chr2L	+	15552722	15555030	15552722	15552809	15554509	15555030	0.006453529	-0.611
355	FBgn0013733	chr2R	-	13907588	13910946	13907588	13908351	13910772	13910946	0.005834912	-0.667
356	FBgn0015037	chr2R	+	9240603	9240842	9240603	9240685	9240741	9240842	0	-0.962
357	FBgn0037655	chr3R	-	9012044	9013261	9012044	9012305	9012836	9013261	0	0.395
358	FBgn0034046	chr2R	+	15848509	15852541	15848509	15849679	15852387	15852541	0	0.352
359	FBgn0011286	chr2R	+	8869843	8870682	8869843	8870447	8870582	8870682	7.81412E-05	0.279
360	FBgn0011286	chr2R	+	8869843	8871023	8869843	8870447	8870923	8871023	0.023178296	0.209
361	FBgn0011286	chr2R	+	8870582	8872424	8870582	8870682	8872219	8872424	0.046448319	0.144
362	FBgn0011286	chr2R	+	8883621	8884325	8883621	8883922	8884190	8884325	0.035136373	0.271
363	FBgn0053170	chr3L	+	22865877	22867535	22865877	22865960	22867399	22867535	0.042942761	0.153
364	FBgn0016917	chr3R	-	20541679	20552311	20541679	20542092	20552080	20552311	0.014493842	0.451
365	FBgn0263006	chr2R	-	23932666	23935884	23932666	23932814	23935824	23935884	0.000491103	0.112
366	FBgn0026401	chr2R	-	4728432	4729017	4728432	4728551	4728813	4729017	0.043992296	-0.399
367	FBgn0015034	chr2R	+	8448961	8449745	8448961	8449136	8449190	8449745	0.001785327	0.253
368	FBgn0015278	chr3L	-	11786051	11787110	11786051	11786639	11787057	11787110	0.015656179	0.206
369	FBgn0033159	chr2R	-	7332260	7335218	7332260	7332380	7334933	7335218	0.000237802	0.486
370	FBgn0033159	chr2R	-	7332260	7342881	7332260	7332380	7342582	7342881	0.015951376	0.611
371	FBgn0033159	chr2R	-	7334933	7348010	7334933	7335218	7347848	7348010	0.001199533	0.486
372	FBgn0051683	chr2L	-	20448350	20449313	20448350	20448873	20448941	20449313	0.003989232	-0.169
373	FBgn0003721	chr3R	+	15281370	15284762	15281370	15281857	15284612	15284762	0.002380243	-0.374
374	FBgn0003721	chr3R	+	15287323	15289439	15287323	15287821	15288252	15289439	0.026190317	-0.19
375	FBgn0262509	chr3L	+	23026307	23029931	23026307	23026667	23029836	23029931	0.022763166	-0.498
376	FBgn0015521	chr2L	+	2856110	2856305	2856110	2856175	2856222	2856305	0.000102537	0.642
377	FBgn0015521	chr2L	+	2856376	2856672	2856376	2856568	2856630	2856672	0	0.879
378	FBgn0032726	chr2L	-	18953403	18954431	18953403	18953790	18954271	18954431	0.038477371	0.305
379	FBgn0015600	chr2L	-	3099452	3108142	3099452	3099932	3107915	3108142	0	0.974
380	FBgn0010052	chr2R	+	16119792	16120653	16119792	16119914	16120398	16120653	0.001519782	-0.206
381	FBgn0020306	chr2R	+	21340683	21341847	21340683	21341076	21341419	21341847	0.030251607	-0.175
382	FBgn0034631	chr2R	-	21498902	21501732	21498902	21501547	21501624	21501732	0.003868751	-0.224
383	FBgn0023531	chrX	-	1681807	1683260	1681807	1682270	1683096	1683260	0.030411711	0.409
384	FBgn0023531	chrX	-	1691206	1692768	1691206	1691357	1692438	1692768	0.016757336	0.255
385	FBgn0014931	chr3R	+	7914791	7916550	7914791	7915369	7915912	7916550	0	0.855
386	FBgn0263656	chr2R	+	7020132	7020396	7020132	7020235	7020295	7020396	0.002874765	0.346
387	FBgn0031730	chr2L	+	5711902	5712424	5711902	5712149	5712237	5712424	4.84269E-05	0.483
388	FBgn0020762	chr2L	+	4333912	4336957	4333912	4334104	4336449	4336957	3.86143E-09	-0.24
389	FBgn0020762	chr2L	+	4333912	4336957	4333912	4334439	4336449	4336957	0.005186288	0.135
390	FBgn0266692	chr3L	+	25844007	25844502	25844007	25844121	25844287	25844502	0.046464167	-0.334

391	FBgn0032010	chr2L	-	8245739	8246766	8245739	8246217	8246673	8246766	0.028088476	-0.349
392	FBgn0032634	chr2L	-	17409855	17410177	17409855	17410030	17410087	17410177	0.012321092	0.118
393	FBgn0053108	chr3R	+	23996031	23997208	23996031	23996279	23997111	23997208	0	0.938
394	FBgn0053108	chr3R	+	23997111	23997544	23997111	23997208	23997266	23997544	0.002064474	0.658
395	FBgn0032705	chr2L	+	18709543	18711297	18709543	18710385	18710440	18711297	2.08565E-05	-0.503
396	FBgn0035266	chr3L	+	1739244	1743834	1739244	1739350	1743207	1743834	0.010982399	0.428
397	FBgn0000464	chr2L	+	19719702	19726034	19719702	19719999	19725894	19726034	0.008021823	0.296
398	FBgn0262508	chr3L	+	8579473	8581855	8579473	8579639	8581762	8581855	0.001486193	0.533
399	FBgn0030160	chrX	+	9884441	9885044	9884441	9884868	9884937	9885044	3.44888E-06	0.168
400	FBgn0050197	chr2R	+	14514029	14514215	14514029	14514084	14514152	14514215	4.38565E-06	0.093
401	FBgn0037265	chr3R	+	4655158	4656655	4655158	4655270	4655517	4656655	0.000161654	-0.37
402	FBgn0037265	chr3R	+	4656902	4657985	4656902	4657209	4657580	4657985	0.00169206	0.205
403	FBgn0033724	chr2R	+	12383044	12384520	12383044	12383204	12383603	12384520	0	0.875
404	FBgn0036768	chr3L	+	17887704	17889462	17887704	17887790	17889330	17889462	0	0.828
405	FBgn0038629	chr3R	-	18594099	18595783	18594099	18594884	18595201	18595783	0.012460196	0.317
406	FBgn0024921	chr3L	+	6193745	6194150	6193745	6193847	6194044	6194150	1.29166E-07	0.753
407	FBgn0008646	chr3R	-	13873299	13874916	13873299	13874253	13874596	13874916	0.028088476	0.244
408	FBgn0035539	chr3L	+	4384441	4391221	4384441	4384627	4390449	4391221	6.22442E-05	-0.183
409	FBgn0032968	chr2L	+	21916299	21920324	21916299	21917429	21919528	21920324	0.01267036	0.434
410	FBgn0032940	chr2L	-	21308287	21310050	21308287	21308995	21310000	21310050	0.027770161	0.335
411	FBgn0033042	chr2R	+	5795412	5796448	5795412	5796326	5796384	5796448	0.01065901	-0.536
412	FBgn0033136	chr2R	+	7056054	7056576	7056054	7056252	7056435	7056576	0.002698152	0.384
413	FBgn0033312	chr2R	-	8611378	8614155	8611378	8612059	8613033	8614155	1.20349E-12	0.282
414	FBgn0033166	chr2R	-	7406205	7407819	7406205	7407060	7407739	7407819	0.002502577	-0.329
415	FBgn0037182	chr3L	-	22716970	22717577	22716970	22717349	22717490	22717577	1.81114E-05	0.725
416	FBgn0263780	chr2R	-	2053344	2119244	2053344	2053618	2118976	2119244	0.019361047	0.228
417	FBgn0039925	chr4	+	1165549	1169239	1165549	1166367	1166665	1169239	0.001715925	-0.61
418	FBgn0032889	chr2L	+	20824433	20826087	20824433	20824493	20825667	20826087	0.02886014	-0.123
419	FBgn0032889	chr2L	+	20824433	20826087	20824433	20824724	20825667	20826087	0.008837095	-0.082
420	FBgn0033374	chr2R	-	9093459	9094658	9093459	9094427	9094491	9094658	1.16478E-06	0.335
421	FBgn0033395	chr2R	+	9237511	9238037	9237511	9237756	9237836	9238037	0	-0.713
422	FBgn0039714	chr3R	-	29865056	29867817	29865056	29865399	29867743	29867817	3.48115E-07	-0.398
423	FBgn0039714	chr3R	-	29865056	29868895	29865056	29865399	29868532	29868895	0.000184623	-0.099
424	FBgn0036493	chr3L	+	15305460	15306798	15305460	15305679	15306512	15306798	0	0.854
425	FBgn0032602	chr2L	-	16758183	16758680	16758183	16758508	16758566	16758680	0.002075144	0.645
426	FBgn0033609	chr2R	-	11287644	11289772	11287644	11288560	11289392	11289772	0	0.582
427	FBgn0033708	chr2R	-	12298746	12299876	12298746	12299075	12299137	12299876	0.03845348	-0.333
428	FBgn0033725	chr2R	+	12387381	12388450	12387381	12387427	12388299	12388450	0.002450226	0.234
429	FBgn0033725	chr2R	+	12388517	12389086	12388517	12388533	12388922	12389086	0	-0.214
430	FBgn0034510	chr2R	-	20512696	20513169	20512696	20513004	20513059	20513169	0.000420965	0.304
431	FBgn0259236	chr3L	-	15631897	15634490	15631897	15632804	15634181	15634490	0.000363464	0.551
432	FBgn0037084	chr2R	+	11219933	11221629	11219933	11220621	11220927	11221629	0	0.686
433	FBgn0033853	chr2R	-	13538971	13547012	13538971	13539349	13546514	13547012	2.11473E-05	-0.228
434	FBgn0036909	chr3L	+	19806094	19806452	19806094	19806237	19806294	19806452	0.037684181	-0.387
435	FBgn0037633	chr3R	-	8805191	8805802	8805191	8805701	8805782	8805802	0.021497713	0.286
436	FBgn0033942	chr2R	-	14512558	14515211	14512558	14513066	14515068	14515211	0	-0.412
437	FBgn0035094	chr2R	-	25184305	25184846	25184305	25184653	25184703	25184846	0.01094439	0.403
438	FBgn0033960	chr2R	-	14753336	14755590	14753336	14753890	14753986	14755590	0.03555691	0.45
439	FBgn0033968	chr2R	+	14808418	14808643	14808418	14808478	14808565	14808643	0.012569235	0.139
440	FBgn0034582	chr2R	-	21068697	21069286	21068697	21069139	21069211	21069286	0.017739289	0.468
441	FBgn0033996	chr2R	+	15203960	15204466	15203960	15204175	15204234	15204466	7.02702E-05	0.264
442	FBgn0040068	chrX	+	19264684	19269651	19264684	19265306	19269256	19269651	0.006453529	0.521
443	FBgn0034031	chr2R	+	15557785	15558170	15557785	15557934	15558007	15558170	0.001150943	-0.421
444	FBgn0034031	chr2R	+	15559859	15560203	15559859	15559998	15560097	15560203	6.7443E-11	-0.738
445	FBgn0034031	chr2R	+	15557243	15562436	15557243	15557310	15561814	15562436	0.001104181	-0.409
446	FBgn0034031	chr2R	+	15557528	15562436	15557528	15557607	15561814	15562436	0.020156735	-0.395
447	FBgn0034031	chr2R	+	15558007	15562436	15558007	15558170	15561814	15562436	1.01351E-06	-0.465
448	FBgn0034031	chr2R	+	15559236	15562436	15559236	15559722	15561814	15562436	1.66233E-07	-0.447
449	FBgn0034031	chr2R	+	15560097	15562436	15560097	15560203	15561814	15562436	1.84986E-13	-0.727
450	FBgn0034031	chr2R	+	15560599	15562436	15560599	15561113	15561814	15562436	0.025404419	-0.401
451	FBgn0034031	chr2R	+	15561362	15562436	15561362	15561417	15561814	15562436	0.000753673	-0.397
452	FBgn0034051	chr2R	-	15935892	15938978	15935892	15936580	15938114	15938978	2.24954E-10	-0.359
453	FBgn0034140	chr2R	-	16574578	16576586	16574578	16575338	16576272	16576586	1.00355E-05	-0.5
454	FBgn0039908	chr4	-	474452	478083	474452	474768	477291	478083	0.025091284	-0.294
455	FBgn0034281	chr2R	+	17860759	17861591	17860759	17861207	17861328	17861591	0.014757725	0.453
456	FBgn0036974	chr3L	+	20348354	20349206	20348354	20348385	20348941	20349206	0.0140084	0.246
457	FBgn0003089	chr3L	-	19305254	19305894	19305254	19305554	19305739	19305894	8.29739E-06	-0.733
458	FBgn0053460	chr2R	+	15694374	15694628	15694374	15694417	15694476	15694628	0.004685819	-0.577
459	FBgn0034408	chr2R	+	18947316	18956126	18947316	18947586	18954977	18956126	0.001417737	-0.336
460	FBgn0034605	chr2R	-	21217015	21217434	21217015	21217174	21217236	21217434	1.17465E-09	-0.402
461	FBgn0034636	chr2R	-	21561793	21562651	21561793	21562281	21562543	21562651	4.0632E-10	-0.229
462	FBgn0000045	chr3L	+	21985209	21986275	21985209	21985248	21985324	21986275	0.034307175	0.106
463	FBgn0034657	chr2R	+	21720733	21721327	21720733	21720874	21720947	21721327	0.024330891	0.57
464	FBgn0034670	chr2R	+	21951591	21952562	21951591	21951662	21951843	21952562	0.034136572	-0.534
465	FBgn0034693	chr2R	-	22091488	22092273	22091488	22092062	22092141	22092273	1.40313E-07	0.855
466	FBgn0034715	chr2R	-	22222659	22223269	22222659	22222980	22223039	22223269	0.004530648	-0.482
467	FBgn0034756	chr2R	-	22652572	22653163	22652572	22652844	22652914	22653163	0.041144634	-0.206
468	FBgn0038221	chr3R	+	14302654	14303394	14302654	14302772	14302831	14303394	2.37677E-06	-0.789
469	FBgn0034860	chr2R	-	23413532	23414765	23413532	23413793	23414496	23414765	1.7071E-10	0.243
470	FBgn0000337	chr2R	-	7783458	7785067	7783458	7784502	7784915	7785067	0.023656875	-0.376
471	FBgn0034897	chr2R	+	23713899	23714899	23713899	23714074	23714665	23714899	1.01351E-06	0.238
472	FBgn0037327	chr3R	-	5462073	5463120	5462073	5462809	5463051	5463120	0.032222322	-0.346
473	FBgn0034903	chr2R	-	23753713	23757166	23753713	23754337	23756363	23757166	5.61025E-10	-0.3
474	FBgn0034903	chr2R	-	23755494	23757494	23755494	23757166	23757414	23757494	0.002874765	0.346
475	FBgn0031359	chr2L	-	1718420	1718903	1718420	1718491	1718739	1718903	0.047408542	-0.148
476	FBgn0034999	chr2R	+	24294096	24295849	24294096	24294221	24295572	24295849	0.003786715	0.198
477	FBgn0266917	chr3R	-	17001863	17002355	17001863	17002028	17002150	17002355	0	0.583
478	FBgn0035298	chr3L	+	1964458	1965273	1964458	1964609	1964836	1965273	0	0.898
479	FBgn0264372	chr2L	-	4963975	4965425	4963975	4964618	4964670	4965425	0	-0.762
480	FBgn0030674	chrX	+	15713640	15714381	15713640	15713815	15713900	15714381	5.39348E-07	0.791
481	FBgn0283712	chrX	-	12587969	12589321	12587969	12589167	12589255	12589321	0.010483762	0.319
482	FBgn0264607	chr4	-	1040012	1045248	1040012	1040078	1045155	1045248	0.030932136	0.228
483	FBgn0040388	chrX	-	2469336	2470043	2469336	2469411	2469797	2470043	5.35141E-07	-0.933
484	FBgn0043534	chr2R	+	20504250	20504790	20504250	20504328	20504391	20504790	0.021972192	-0.174
485	FBgn0035766	chr3L	-	7335441	7338988	7335441	7335875	7335937	7338988	0.001166101	0.221
486	FBgn0030505	chrX	+	13655850	13662686	13655850	13656034	13660739	13662686	5.00053E-06	0.209
487	FBgn0032625	chr2L	-	17047770	17048281	17047770	17048068	17048128	17048281	6.62061E-05	0.667
488	FBgn0053558	chr2R	-	6926669	6928148	6926669	6926821	6927389	6928148	0.000205627	0.322
489	FBgn0036152	chr3L	-	11291037	11291368	11291037	11291109	11291335	11291368	0.002597643	0.465
490	FBgn0032819	chr2L	+	19759001	19759504	19759001	19759152	19759203	19759504	0.000118257	-0.211
491	FBgn0036661	chr3L	+	16780099	16780790	16780099	16780420	16780504	16780790	0	0.338
492	FBgn0036368	chr3L	+	13462357	13463083	13462357	13462545	13462864	13463083	0.028039954	-0.267
493	FBgn0041150	chr2L	-	4925828	4928393	4925828	4926350	4928243	4928393	0.020843386	-0.296
494	FBgn0036566	chr3L	+	16090455	16091858	16090455	16090521	16091515	16091858	0.040154949	-0.17
495	FBgn0036829	chr3L	-	18909023	18910686	18909023	18909884	18909980	18910686	1.04623E-05	-0.187
496	FBgn0036958	chr3L	-	20238737	20240532	20238737	20239547	20239862	20240532	0.009813939	0.238
497	FBgn0036958	chr3L	-	20238737	20240532	20238737	20239610	20239862	20240532	0.019294011	0.378
498	FBgn0036995	chr3L	-	20459428	20464862	20459428	20459621	20464745	20464862	0.001906486	-0.502
499	FBgn0265356	chr2R	+	18987343	18989688	18987343	18987691	18989383	18989688	6.01481E-05	-0.223
500	FBgn0265356	chr2R	+	18997127	19001789	18997127	18998423	19001040	19001789	4.05978E-05	0.212
501	FBgn0037027	chr3L	+	20812256	20812670	20812256	20812464	20812523	20812670	0.004237864	0.29
502	FBgn0037057	chr3L	+	21202919	21206626	21202919	21203322	21205676	21206626	4.56954E-05	-0.23
503	FBgn0037057	chr3L	+	21205273	21206626	21205273	21205508	21205676	21206626	0.008209256	-0.213
504	FBgn0260934	chr2R	+	19464309	19470812	19464309	19465694	19468789	19470812	0.000268044	-0.319
505	FBgn0015778	chr3R	+	13647003	13650497	13647003	13647200	13648917	13650497	0.00105922	0.234
506	FBgn0037117	chr3L	+	21680049	21682433	21680049	21680232	21680299	21682433	1.13579E-08	0.88
507	FBgn0010548	chr2R	-	7466878	7467633	7466878	7467316	7467473	7467633	0.001791196	0.26
508	FBgn0010548	chr2R	-	7473721	7474659	7473721	7473843	7474571	7474659	0.00013346	-0.255
509	FBgn0011260	chr2R	+	16516149	16517341	16516149	16516270	16517018	16517341	0.005038052	0.317
510	FBgn0037203	chr3L	+	22885174	22892495	22885174	22885437	22891364	22892495	0.048934795	0.389
511	FBgn0034279	chr2R	-	17769285	17769989	17769285	17769491	17769550	17769989	3.15815E-05	0.477
512	FBgn0085478	chrX	-	9337697	9338225	9337697	9337892	9338052	9338225	0	0.248
513	FBgn0037520	chr3R	-	7803280	7804432	7803280	7804048	7804158	7804432	0.024114223	0.192
514	FBgn0034931	chr2R	+	23925581	23926211	23925581	23925739	23925808	23926211	0.001711313	-0.208
515	FBgn0037614	chr3R	+	8716242	8717341	8716242	8716568	8716895	8717341	2.79326E-06	0.298
516	FBgn0037762	chr3R	+	9814160	9815158	9814160	9814382	9814447	9815158	9.501E-06	0.39
517	FBgn0264908	chr3L	-	15893619	15898920	15893619	15893961	15898831	15898920	0.000491103	-0.53
518	FBgn0264908	chr3L	-	15893619	15898920	15893619	15893961	15898834	15898920	0.000375298	-0.433
519	FBgn0004169	chrX	-	13596378	13598176	13596378	13596748	13598173	13598176	0.005208276	0.111
520	FBgn0037913	chr3R	-	11564439	11565111	11564439	11564711	11564783	11565111	0.003300321	0.266
521	FBgn0037921	chr3R	-	11624354	11625644	11624354	11624828	11625474	11625644	1.01351E-06	0.446
522	FBgn0039709	chr3R	+	29841656	29845760	29841656	29841790	29845598	29845760	0.020843386	-0.321

523	FBgn0051038	chr3R	+	29877058	29879810	29877058	29877115	29877926	29879810	2.40797E-12	0.343
524	FBgn0038224	chr3R	+	14327067	14327735	14327067	14327183	14327252	14327735	0.019005603	0.092
525	FBgn0034876	chr2R	-	23547489	23548151	23547489	23547875	23547983	23548151	0	0.788
526	FBgn0266099	chr3L	-	16391476	16398040	16391476	16391617	16396942	16398040	0.009907925	-0.537
527	FBgn0002643	chr2R	+	14009198	14011181	14009198	14009238	14010162	14011181	0.039226587	-0.489
528	FBgn0263457	chrX	+	23091688	23092384	23091688	23091820	23091933	23092384	0.015570795	-0.336
529	FBgn0038510	chr3R	-	17438967	17439425	17438967	17439233	17439295	17439425	0.002586412	-0.805
530	FBgn0039782	chr3R	+	30506240	30506575	30506240	30506374	30506424	30506575	0.046448319	-0.161
531	FBgn0038547	chr3R	-	17800723	17801293	17800723	17801050	17801125	17801293	5.49073E-05	0.644
532	FBgn0038721	chr3R	-	19638266	19638750	19638266	19638427	19638678	19638750	0.006759945	0.327
533	FBgn0038721	chr3R	-	19639025	19641086	19639025	19639413	19640983	19641086	7.30366E-05	0.589
534	FBgn0022764	chr2R	-	12576567	12577653	12576567	12576993	12577453	12577653	0.013772759	0.25
535	FBgn0038294	chr3R	-	15175624	15176293	15175624	15175863	15176183	15176293	0.006702009	-0.16
536	FBgn0038294	chr3R	-	15176363	15178489	15176363	15176412	15178279	15178489	3.03361E-05	-0.17
537	FBgn0038294	chr3R	-	15176363	15178489	15176363	15176427	15178279	15178489	6.23763E-13	-0.244
538	FBgn0283471	chrX	-	18111937	18113381	18111937	18112038	18113257	18113381	0.007493432	0.126
539	FBgn0002121	chr2L	-	20830	21376	20830	20973	21065	21376	1.71689E-07	0.672
540	FBgn0039186	chr3R	-	24348146	24348791	24348146	24348560	24348661	24348791	0.030705491	0.119
541	FBgn0039235	chr3R	-	24856954	24857559	24856954	24857322	24857371	24857559	0.005606342	-0.269
542	FBgn0033372	chr2R	-	9085363	9085820	9085363	9085664	9085720	9085820	0.000225421	0.246
543	FBgn0027335	chrX	-	18635641	18637573	18635641	18636319	18637330	18637573	0.044347782	-0.249
544	FBgn0039536	chr3R	+	27666426	27667244	27666426	27666539	27667099	27667244	0.00051619	0.509
545	FBgn0053774	chr2R	-	9399752	9400068	9399752	9399868	9399947	9400068	0.045915578	0.152
546	FBgn0039659	chr3R	-	29293742	29296993	29293742	29295085	29295160	29296993	0.023862618	0.628
547	FBgn0039790	chr3R	-	30595389	30600323	30595389	30595626	30599918	30600323	5.26413E-12	-0.772
548	FBgn0039790	chr3R	-	30595389	30600323	30595389	30595626	30600123	30600323	0.00370743	0.164
549	FBgn0039790	chr3R	-	30595389	30600323	30595389	30595626	30600263	30600323	9.62741E-06	0.558
550	FBgn0039790	chr3R	-	30601200	30601489	30601200	30601308	30601416	30601489	0.032222322	-0.134
551	FBgn0039792	chr3R	-	30627240	30629028	30627240	30628666	30628723	30629028	0.003746502	-0.511
552	FBgn0010015	chr3R	+	31039373	31040310	31039373	31039548	31039984	31040310	0.030251607	-0.219
553	FBgn0010015	chr3R	+	31043388	31043795	31043388	31043470	31043717	31043795	0.007931667	0.183
554	FBgn0039817	chr3R	-	31099105	31099321	31099105	31099214	31099273	31099321	4.52712E-12	-0.966
555	FBgn0039830	chr3R	-	31217739	31218904	31217739	31217944	31218792	31218904	0.009886213	-0.31
556	FBgn0016070	chr3L	-	8990316	8993041	8990316	8991537	8992452	8993041	3.60379E-07	0.247
557	FBgn0039890	chr4	-	191308	193489	191308	191386	193343	193489	0.000149053	0.336
558	FBgn0267986	chr2L	+	23239252	23240411	23239252	23239831	23240169	23240411	0.049392003	-0.267
559	FBgn0034484	chr2R	+	20233939	20234927	20233939	20234374	20234559	20234927	0	-0.526
560	FBgn0033368	chr2R	-	9047913	9050132	9047913	9048026	9049879	9050132	1.3614E-11	-0.856
561	FBgn0033368	chr2R	-	9059254	9060608	9059254	9059548	9060588	9060608	4.13259E-05	-0.716
562	FBgn0028875	chr2L	+	14077122	14080734	14077122	14077198	14080539	14080734	0.037566579	-0.147
563	FBgn0043533	chr2R	+	19713394	19713976	19713394	19713478	19713532	19713976	0.002457446	0.48
564	FBgn0285949	chr2R	+	9587446	9587989	9587446	9587708	9587776	9587989	0.004090765	0.511
565	FBgn0285926	chrX	-	10805265	10806856	10805265	10805631	10806468	10806856	4.05978E-05	0.733
566	FBgn0267376	chr3R	-	10866808	10868642	10866808	10866994	10868438	10868642	0.003388406	-0.547
567	FBgn0267336	chr3R	+	9943676	9982370	9943676	9943894	9982254	9982370	0.001651488	-0.622
568	FBgn0267252	chr2L	+	9256562	9257206	9256562	9256814	9256895	9257206	0	0.532
569	FBgn0266869	chr2L	+	13241806	13244724	13241806	13242014	13244345	13244724	1.20349E-12	-1
570	FBgn0285948	chr2L	-	4457739	4458317	4457739	4458057	4458140	4458317	0.04611035	0.228
571	FBgn0265045	chr2R	-	15985861	15987262	15985861	15986181	15987132	15987262	0.006618989	0.587
572	FBgn0263593	chr2R	-	8142642	8145981	8142642	8143074	8145903	8145981	0	-0.98
573	FBgn0262570	chr2R	-	17482905	17483637	17482905	17483433	17483483	17483637	0.045396015	0.824
574	FBgn0261243	chr3L	+	1517850	1518796	1517850	1517956	1518373	1518796	1.81114E-05	-0.335
575	FBgn0261239	chr2L	+	21253554	21255828	21253554	21255313	21255391	21255828	0.000193234	-0.567
576	FBgn0260943	chr3L	-	17235351	17238212	17235351	17236062	17237885	17238212	0	0.882
577	FBgn0260474	chr2R	+	9704870	9706147	9704870	9705017	9705136	9706147	0.023896342	-0.667
578	FBgn0259994	chrX	-	5642933	5643332	5642933	5643111	5643176	5643332	0.000121684	0.197
579	FBgn0250839	chr3R	-	4960667	4961351	4960667	4960831	4960893	4961351	0.044840327	-0.402
580	FBgn0083141	chr2L	+	7582004	7583037	7582004	7582846	7582902	7583037	0.009068423	0.667
581	FBgn0083124	chr2R	+	9096925	9097312	9096925	9096991	9097061	9097312	0.000232009	-0.464
582	FBgn0082582	chr3R	+	30546405	30548749	30546405	30546587	30548278	30548749	1.65836E-07	-0.188
583	FBgn0082582	chr3R	+	30546405	30548749	30546405	30546587	30548303	30548749	0.000452425	-0.163
584	FBgn0064225	chr2L	-	22536114	22536884	22536114	22536403	22536536	22536884	0.015210541	-0.388
585	FBgn0265991	chr2R	-	15774427	15776062	15774427	15774757	15775844	15776062	2.48088E-10	-0.173
586	FBgn0053013	chr2R	+	12363984	12364300	12363984	12364159	12364245	12364300	7.60947E-06	0.871
587	FBgn0052944	chr3R	-	4415682	4419578	4415682	4415886	4419359	4419578	2.83903E-09	0.728
588	FBgn0052719	chrX	-	7508650	7511658	7508650	7508793	7511361	7511658	0.032932553	-0.537
589	FBgn0052264	chr3L	-	3758406	3765731	3758406	3759258	3765604	3765731	0.001769324	0.316
590	FBgn0052207	chr3L	-	19477906	19478720	19477906	19478066	19478299	19478720	0.034690796	0.395
591	FBgn0052085	chr3L	-	11674267	11680344	11674267	11675665	11680043	11680344	0.00061375	-0.3
592	FBgn0051973	chr2L	-	25401	26964	25401	26688	26761	26964	0.01094439	-0.227
593	FBgn0051709	chr2L	-	9579092	9579638	9579092	9579457	9579510	9579638	0.024036289	-0.315
594	FBgn0286785	chr2R	+	15255932	15256595	15255932	15256062	15256209	15256595	1.03166E-09	-0.744
595	FBgn0050440	chr2R	-	5491999	5495861	5491999	5492149	5493890	5495861	2.16901E-06	-0.745
596	FBgn0050344	chr2R	+	9146862	9147246	9146862	9147046	9147131	9147246	0.042444606	0.406
597	FBgn0050147	chr2R	-	20614505	20618217	20614505	20614933	20618085	20618217	3.02049E-05	0.362
598	FBgn0034753	chr2R	-	22644940	22646045	22644940	22645534	22645676	22646045	0.035357297	0.58
599	FBgn0264357	chr3R	+	21208477	21210705	21208477	21208633	21210279	21210705	0.01716546	0.323
600	FBgn0000055	chr2L	+	14615551	14616437	14615551	14615643	14616057	14616437	2.64673E-08	-0.289
601	FBgn0000055	chr2L	+	14615551	14616437	14615551	14615643	14616215	14616437	0	0.454
602	FBgn0027866	chr3R	-	4332289	4332709	4332289	4332477	4332607	4332709	0.000906328	0.292
603	FBgn0038820	chr3R	-	20676441	20677351	20676441	20677205	20677268	20677351	0.033915691	0.55
604	FBgn0031815	chr2L	+	6456051	6456682	6456051	6456208	6456326	6456682	1.01351E-06	-0.771
605	FBgn0034611	chr2R	+	21282297	21283212	21282297	21282391	21282990	21283212	0.001888304	0.445
606	FBgn0039213	chr3R	+	24625670	24632091	24625670	24625812	24631762	24632091	6.99581E-07	-0.685
607	FBgn0069923	chr3R	+	3750362	3752353	3750362	3750543	3752166	3752353	0.01267036	-0.62
608	FBgn0013334	chr3R	+	15935118	15935912	15935118	15935584	15935839	15935912	0	-0.174
609	FBgn0061198	chr2R	-	24028380	24028789	24028380	24028533	24028613	24028789	1.48416E-08	-0.637
610	FBgn0051674	chr2L	+	20831261	20831878	20831261	20831395	20831469	20831878	0.005124775	-0.178
611	FBgn0034743	chr2R	+	22606508	22607048	22606508	22606640	22606897	22607048	0.000117523	-0.687
612	FBgn0030725	chrX	+	16083541	16083850	16083541	16083646	16083701	16083850	0.021988905	-0.529
613	FBgn0058191	chr2R	+	1265170	1266349	1265170	1265531	1266191	1266349	1.01568E-08	0.334
614	FBgn0261238	chr3R	-	7094595	7095843	7094595	7095515	7095575	7095843	0.005129721	-0.52
615	FBgn0039209	chr3R	-	24560889	24572495	24560889	24562942	24572092	24572495	0.000888091	-0.667
616	FBgn0028622	chr2R	-	20581560	20582132	20581560	20581651	20581847	20582132	0.040377951	0.324
617	FBgn0028527	chr2L	+	13670437	13671247	13670437	13670597	13670835	13671247	0.029308878	-0.261
618	FBgn0086358	chr2R	+	19299169	19302878	19299169	19300799	19302649	19302878	0.019545863	-0.497
619	FBgn0028380	chr3L	+	19294766	19296296	19294766	19295098	19296147	19296296	0.001629675	0.439
620	FBgn0264489	chr3L	+	9830999	9832679	9830999	9831440	9832531	9832679	0.00587157	-0.168
621	FBgn0285913	chr3R	+	14289273	14290820	14289273	14289449	14290705	14290820	8.29361E-06	-0.392
622	FBgn0000721	chr2L	-	3655269	3656953	3655269	3655585	3656544	3656953	2.04009E-06	0.778
623	FBgn0000721	chr2L	-	3655269	3656953	3655269	3655585	3656828	3656953	0.000568941	0.503
624	FBgn0026562	chr3R	-	26870192	26871995	26870192	26870396	26871925	26871995	0.01716546	-0.333
625	FBgn0033371	chr2R	+	9082394	9082985	9082394	9082618	9082674	9082985	0.007737851	-0.329
626	FBgn0015283	chr3L	+	21537065	21537380	21537065	21537138	21537210	21537380	0.002387245	0.323
627	FBgn0027596	chr2R	-	14905513	14907971	14905513	14905953	14907705	14907971	0.000845564	-0.59
628	FBgn0004687	chrX	-	5619135	5620575	5619135	5620286	5620397	5620575	0.007077427	0.162
629	FBgn0039923	chr4	-	787080	787614	787080	787209	787380	787614	0.018020785	0.416
630	FBgn0032801	chr2L	-	19528087	19528996	19528087	19528369	19528865	19528996	6.02747E-06	0.708
631	FBgn0051635	chr2L	-	6645971	6648145	6645971	6646947	6647746	6648145	0.000184541	-0.574
632	FBgn0036666	chr3L	+	16843102	16843730	16843102	16843378	16843492	16843730	0	-0.603
633	FBgn0085468	chr2R	+	13159016	13159649	13159016	13159103	13159511	13159649	7.98985E-09	-0.643
634	FBgn0003890	chr3R	-	27974107	27988384	27974107	27974375	27988098	27988384	9.36786E-14	-0.933
635	FBgn0259243	chr3L	+	20828224	20829540	20828224	20828340	20829291	20829540	0.026421859	0.428
636	FBgn0013305	chr2R	-	12879196	12879656	12879196	12879222	12879286	12879656	0.005038052	-0.424
637	FBgn0003415	chr3L	-	21024951	21027434	21024951	21025548	21027185	21027434	0.002380243	0.648
638	FBgn0042213	chr3R	+	29725026	29725690	29725026	29725118	29725237	29725690	2.72162E-05	0.697
639	FBgn0010225	chr3R	+	4236787	4237817	4236787	4236939	4237603	4237817	9.53789E-05	-0.38
640	FBgn0010473	chr2L	+	4304599	4307461	4304599	4304782	4305348	4307461	0.024305782	0.538
641	FBgn0031857	chr2L	-	6770590	6772755	6770590	6770718	6772551	6772755	0	0.971
642	FBgn0053218	chrX	+	2330158	2330826	2330158	2330355	2330508	2330826	0.040952484	-0.418
643	FBgn0025702	chr3L	+	22282903	22284335	22282903	22283138	22283202	22284335	0	0.205
644	FBgn0025702	chr3L	+	22282903	22284335	22282903	22283144	22283202	22284335	0	0.216
645	FBgn0040778	chr2R	-	8066273	8067293	8066273	8066758	8067184	8067293	0	-0.961
646	FBgn0031089	chrX	+	20148123	20150125	20148123	20148261	20150067	20150125	7.81412E-05	0.395
647	FBgn0019929	chrX	+	9865232	9865607	9865232	9865397	9865461	9865607	0.026025933	-0.208
648	FBgn0022160	chr2R	-	15860600	15862020	15860600	15860722	15861881	15862020	9.75974E-05	-0.162
649	FBgn0022160	chr2R	-	15860600	15862755	15860600	15861026	15862682	15862755	0.001011265	-0.282
650	FBgn0001105	chrX	+	15860114	15861153	15860114	15860250	15861151	15861153	0.03502555	-0.305
651	FBgn0029723	chrX	+	4707915	4709010	4707915	4708168	4708812	4709010	0.016625411	0.392
652	FBgn0015221	chr3R	+	30389198	30389442	30389198	30389328	30389436	30389442	0.000821606	-0.248
653	FBgn0003607	chr2L	-	8209625	8210473	8209625	8210103	8210178	8210473	0.047337674	-0.359
654	FBgn0032172	chr2L	-	9962734	9963755	9962734	9963012	9963377	9963755	0.001365774	0.527

655	FBgn0036703	chr3L	+	17243813	17244309	17243813	17244047	17244126	17244309	4.25676E-08	0.841
656	FBgn0037895	chr3R	-	11416217	11416663	11416217	11416420	11416486	11416663	0.01823193	0.3
657	FBgn0033443	chr2R	+	9793325	9795894	9793325	9793567	9795408	9795894	2.05073E-05	-0.335
658	FBgn0011206	chr3L	-	9113045	9130608	9113045	9113487	9130554	9130608	0.00572537	0.347
659	FBgn0053098	chr3R	+	12387187	12387637	12387187	12387286	12387547	12387637	1.28244E-05	0.439
660	FBgn0003091	chr2R	+	16931198	16936530	16931198	16931278	16936372	16936530	0.000367324	0.667
661	FBgn0023441	chr2R	-	15657317	15658848	15657317	15658401	15658469	15658848	0.021092422	-0.387
662	FBgn0026374	chrX	+	15788896	15790388	15788896	15788957	15790233	15790388	2.89391E-05	-0.889
663	FBgn0001215	chr3R	+	28600717	28602947	28600717	28601195	28602745	28602947	0.000169761	-0.563
664	FBgn0032836	chr2L	+	19964839	19965702	19964839	19964998	19965062	19965702	0	0.48
665	FBgn0003714	chrX	-	2442312	2443976	2442312	2442732	2443830	2443976	0.012642683	-0.561
666	FBgn0003141	chr2L	-	20074190	20074460	20074190	20074336	20074391	20074460	4.50555E-05	-0.575
667	FBgn0029897	chrX	-	6743057	6744238	6743057	6743504	6744108	6744238	0.038087706	-0.417
668	FBgn0284245	chr2R	+	11891134	11891529	11891134	11891244	11891329	11891529	0.035422849	-0.337
669	FBgn0028408	chr2R	+	9297265	9302821	9297265	9297542	9298266	9302821	0.004418604	0.578
670	FBgn0036993	chr3L	+	20445947	20446819	20445947	20446401	20446749	20446819	0.000134161	-0.479
671	FBgn0028956	chr2R	-	17450528	17452598	17450528	17451032	17452494	17452598	6.32768E-07	0.889
672	FBgn0020521	chr2R	+	24589376	24592174	24589376	24589425	24591954	24592174	0.044393722	0.663
673	FBgn0039261	chr3R	-	25036622	25038318	25036622	25036717	25037849	25038318	7.39391E-05	0.545
674	FBgn0036335	chr3L	-	13017401	13017867	13017401	13017604	13017675	13017867	0.000291538	-0.591
675	FBgn0015803	chr2L	+	20356561	20360433	20356561	20356801	20359988	20360433	0.005495171	-0.583
676	FBgn0016694	chr3L	-	7830386	7833073	7830386	7831827	7832863	7833073	0.014925626	0.284
677	FBgn0031717	chr2L	+	5547289	5548100	5547289	5547728	5547807	5548100	9.08334E-06	0.826
678	FBgn0035889	chr3L	-	8404102	8405277	8404102	8404510	8404879	8405277	1.96417E-06	-0.345
679	FBgn0013307	chr2R	+	8005628	8006232	8005628	8005926	8005987	8006232	0.041584346	0.463
680	FBgn0033717	chr2R	-	12341487	12344617	12341487	12341782	12344467	12344617	0.020246968	0.463
681	FBgn0039348	chr3R	-	25611052	25611937	25611052	25611449	25611541	25611937	0.004389243	0.46
682	FBgn0051999	chr4	-	218445	220028	218445	218890	219641	220028	0.024036289	0.289
683	FBgn0036891	chr3L	+	19607245	19608368	19607245	19607825	19607976	19608368	9.68389E-13	-0.688
684	FBgn0014127	chr2L	-	20060411	20060977	20060411	20060764	20060828	20060977	0.001575106	-0.654
685	FBgn0037547	chr3R	+	8088643	8089093	8088643	8088769	8088853	8089093	0.049392003	0.599
686	FBgn0052220	chr3L	-	19988400	19988816	19988400	19988605	19988663	19988816	0.001537651	0.706
687	FBgn0053170	chr3L	+	22867115	22867535	22867115	22867340	22867399	22867535	0.031787482	-0.13
688	FBgn0015569	chr3R	-	7488301	7491603	7488301	7488652	7491340	7491603	0.011797962	-0.667
689	FBgn0032378	chr2L	-	11811593	11813780	11811593	11812682	11813633	11813780	0.000112472	0.551
690	FBgn0264495	chr3R	+	6406854	6437934	6406854	6407077	6407477	6437934	0.000113517	-0.468
691	FBgn0264495	chr3R	+	6406854	6437934	6406854	6407191	6407477	6437934	0.001619176	0.277
692	FBgn0262508	chr3L	+	8593466	8595794	8593466	8595441	8595534	8595794	0.01267036	-0.643
693	FBgn0050197	chr2R	+	14514152	14514456	14514152	14514215	14514302	14514456	0.000833669	-0.53
694	FBgn0025683	chr2L	-	128799	130791	128799	129239	130507	130791	0.030186374	-0.5
695	FBgn0033110	chr2R	-	6875174	6875819	6875174	6875440	6875688	6875819	0.030458822	-0.667
696	FBgn0033246	chr2R	-	7985601	7987237	7985601	7985781	7987065	7987237	4.97554E-06	-0.328
697	FBgn0039714	chr3R	-	29865056	29867214	29865056	29865399	29867161	29867214	0.000139514	-0.414
698	FBgn0016059	chr2R	-	17675910	17677919	17675910	17676593	17677515	17677919	0.000943681	0.455
699	FBgn0022382	chr2R	-	10024050	10026042	10024050	10024297	10025828	10026042	0.010422745	0.363
700	FBgn0034073	chr2R	-	16099693	16102050	16099693	16101781	16101837	16102050	0	-0.919
701	FBgn0034180	chr2R	-	17014948	17015894	17014948	17015233	17015658	17015894	0.026421859	-0.311
702	FBgn0038243	chr3R	-	14568080	14568847	14568080	14568568	14568663	14568847	0.016340851	-0.667
703	FBgn0026619	chr2R	-	12753218	12753816	12753218	12753353	12753421	12753816	1.11042E-06	0.711
704	FBgn0083228	chrX	+	18199773	18200744	18199773	18199822	18200522	18200744	3.3368E-05	0.399
705	FBgn0052803	chrX	-	1901436	1901911	1901436	1901614	1901667	1901911	0.001788264	0.406
706	FBgn0034408	chr2R	+	18878390	18880128	18878390	18878640	18880004	18880128	0.049697021	-0.392
707	FBgn0023508	chrX	+	1926693	1927524	1926693	1926922	1926978	1927524	0.047507072	-0.595
708	FBgn0034420	chr2R	+	19172711	19173643	19172711	19172921	19173230	19173643	0.002532298	0.291
709	FBgn0034479	chr2R	+	19981069	19982703	19981069	19981275	19982283	19982703	0.00509435	0.441
710	FBgn0034538	chr2R	-	20653744	20655096	20653744	20653867	20654969	20655096	0.018273576	0.356
711	FBgn0034075	chr2R	+	16111982	16112530	16111982	16112141	16112246	16112530	4.97554E-06	0.375
712	FBgn0034075	chr2R	+	16114771	16115252	16114771	16114866	16115115	16115252	1.3438E-12	-0.575
713	FBgn0260461	chr3L	+	19545682	19546716	19545682	19545964	19546422	19546716	0.012060783	0.55
714	FBgn0034709	chr2R	-	22191661	22202671	22191661	22191855	22202300	22202671	0.006696464	-0.382
715	FBgn0034709	chr2R	-	22191661	22202671	22191661	22191855	22202476	22202671	0.023775085	-0.363
716	FBgn0034808	chr2R	-	22996376	22998884	22996376	22996512	22998432	22998884	3.45068E-06	0.653
717	FBgn0002773	chr3R	-	30173571	30174551	30173571	30173814	30174140	30174551	0.034690796	-0.195
718	FBgn0034943	chr2R	+	23963306	23963717	23963306	23963493	23963558	23963717	0.022518444	0.395
719	FBgn0035001	chr2R	-	24401662	24402384	24401662	24401808	24402216	24402384	0.047408542	-0.318
720	FBgn0039006	chr3R	+	22698318	22699344	22698318	22699084	22699146	22699344	0.021144519	-0.706
721	FBgn0035092	chr2R	+	25037701	25040377	25037701	25037983	25039050	25040377	0.040820425	0.274
722	FBgn0023528	chrX	-	2268937	2270761	2268937	2269704	2270407	2270761	0.032222322	0.355
723	FBgn0035400	chr3L	-	3132632	3139956	3132632	3133338	3139807	3139956	0.01065901	-0.298
724	FBgn0036496	chr3L	-	15472196	15473886	15472196	15473642	15473700	15473886	0.01065901	0.343
725	FBgn0259749	chr2L	-	6471327	6472072	6471327	6471743	6471960	6472072	8.37796E-09	-0.481
726	FBgn0261565	chr3L	+	16891225	16896347	16891225	16891314	16896232	16896347	0.028088476	0.188
727	FBgn0011695	chr2R	+	24026375	24027518	24026375	24026461	24027003	24027518	1.00355E-05	-0.164
728	FBgn0037433	chr3R	+	6344028	6345087	6344028	6344461	6344530	6345087	0.014806093	0.38
729	FBgn0037051	chr3L	-	21133249	21135509	21133249	21134905	21135217	21135509	1.90374E-06	-0.633
730	FBgn0037070	chr3L	-	21303909	21304467	21303909	21304091	21304147	21304467	0.022972748	0.434
731	FBgn0264908	chr3L	-	15867554	15868057	15867554	15867619	15867892	15868057	0.045024825	0.541
732	FBgn0050345	chr2R	+	9149686	9150087	9149686	9149837	9149890	9150087	0.001948959	0.498
733	FBgn0262351	chr3L	+	18925090	18925585	18925090	18925387	18925458	18925585	0.001696184	0.531
734	FBgn0038224	chr3R	+	14326946	14327735	14326946	14327188	14327252	14327735	0.008215323	0.207
735	FBgn0039897	chr4	+	242487	243585	242487	242679	243175	243585	0.025221713	0.189
736	FBgn0033669	chr2R	-	11968283	11968850	11968283	11968591	11968739	11968850	1.90374E-06	0.719
737	FBgn0033669	chr2R	-	11968283	11968850	11968283	11968631	11968739	11968850	0.022690607	0.374
738	FBgn0000259	chrX	+	11795104	11797301	11795104	11795186	11796865	11797301	0.049119846	0.279
739	FBgn0039881	chr3R	+	31805467	31806739	31805467	31805647	31805766	31806739	1.00355E-05	-0.652
740	FBgn0039323	chr3R	-	25315367	25316116	25315367	25315858	25315919	25316116	0.002771496	0.398
741	FBgn0085387	chrX	-	20792604	20805983	20792604	20792738	20805841	20805983	4.8264E-06	0.786
742	FBgn0039667	chr3R	+	29498063	29498503	29498063	29498231	29498322	29498503	0.043000465	-0.62
743	FBgn0039776	chr3R	+	30474033	30479427	30474033	30474228	30479094	30479427	0.0140084	-0.6
744	FBgn0039776	chr3R	+	30484119	30484752	30484119	30484602	30484689	30484752	0	0.859
745	FBgn0039830	chr3R	-	31216769	31217944	31216769	31217177	31217775	31217944	0.000517709	-0.653
746	FBgn0039830	chr3R	-	31218942	31219559	31218942	31219048	31219145	31219559	3.26451E-05	-0.188
747	FBgn0039461	chr3R	+	26876629	26879836	26876629	26876755	26879464	26879836	0.002801912	-0.663
748	FBgn0039936	chr4	-	861792	865642	861792	864411	865374	865642	0.027591346	-0.278
749	FBgn0004656	chrX	-	8059866	8060974	8059866	8060158	8060916	8060974	0.002497282	0.513
750	FBgn0004656	chrX	-	8059866	8061436	8059866	8060158	8061299	8061436	0.002075144	-0.648
751	FBgn0267975	chr3R	-	19226014	19226558	19226014	19226070	19226198	19226558	0.021736326	0.477
752	FBgn0015777	chr2L	-	6791030	6791436	6791030	6791323	6791392	6791436	0.019005603	-0.517
753	FBgn0043841	chr2L	-	12421640	12423439	12421640	12421829	12422960	12423439	0.019941266	-0.472
754	FBgn0043841	chr2L	-	12421640	12423439	12421640	12421829	12423278	12423439	0.026777209	0.367

SE											
	**FlyBase ID**	**chr**	**strand**	**exonStart_0base**	**exonEnd**	**upstreamES**	**upstreamEE**	**downstreamES**	**downstreamEE**	**FDR**	**∆PSI**
1	FBgn0036684	chr3L	-	17001515	17001747	16999738	17000012	17002221	17002360	0.032476431	-0.223
2	FBgn0040068	chrX	+	19264684	19265306	19263880	19263955	19269483	19269651	7.47122E-05	-0.536
3	FBgn0035438	chr3L	+	3327342	3327553	3326264	3326712	3327859	3328183	0.011188284	0.148
4	FBgn0052112	chr3L	+	12742594	12742650	12741804	12741949	12743512	12743625	1.21856E-12	-0.692
5	FBgn0265998	chr3R	+	28905638	28907337	28900823	28901022	28919491	28919567	0.040128071	0.134
6	FBgn0266137	chr3R	-	29638931	29639105	29630303	29632860	29640228	29640310	2.41155E-07	-0.679
7	FBgn0263980	chr2R	+	15457747	15457924	15456659	15456815	15464470	15464686	7.51698E-05	0.486
8	FBgn0263980	chr2R	+	15458898	15459267	15456659	15456815	15464470	15464686	0.013144758	0.291
9	FBgn0263980	chr2R	+	15460872	15461226	15456659	15456815	15464470	15464686	1.65698E-07	0.291
10	FBgn0263980	chr2R	+	15460872	15461226	15458898	15459267	15463297	15463846	3.61583E-05	0.224
11	FBgn0263980	chr2R	+	15465330	15465491	15464470	15464686	15465649	15465745	0.003228288	0.139
12	FBgn0263873	chr2L	+	19845376	19845543	19826473	19828878	19857669	19857840	0.014101908	0.5
13	FBgn0263346	chr3R	+	4680778	4680837	4659578	4660399	4697190	4697542	6.79053E-10	0.381
14	FBgn0263346	chr3R	+	4680778	4680837	4670796	4670850	4697190	4697542	0	0.658
15	FBgn0035162	chr3L	-	830621	830728	830033	830341	830907	831242	0.000261271	-0.22
16	FBgn0262737	chr3L	+	21875296	21875325	21844787	21845235	21907345	21907920	8.44004E-06	-0.362
17	FBgn0011817	chr3L	+	8048069	8048443	8047506	8047600	8049868	8050333	5.04014E-07	0.229
18	FBgn0261984	chr3R	-	19860646	19860761	19857240	19857684	19860885	19861291	4.00697E-12	0.177
19	FBgn0261808	chr3R	+	11204564	11204831	11203031	11203785	11206905	11207138	2.12206E-07	0.226
20	FBgn0039790	chr3R	-	30601200	30601308	30595389	30595626	30601416	30601489	0.009485827	0.265
21	FBgn0004573	chr3R	-	30977446	30977520	30968235	30971947	31016175	31016875	0.009485827	0.329
22	FBgn0034718	chr2R	-	22308207	22308256	22298213	22301009	22311695	22311937	2.41539E-10	0.154
23	FBgn0039897	chr4	+	231934	231977	231425	231600	236394	236475	4.35482E-06	-0.196
24	FBgn0000448	chr2R	-	10222476	10224024	10209879	10210107	10232023	10234607	0.00878452	-0.405
25	FBgn0013812	chr3R	-	21036726	21036756	21035652	21036594	21037016	21037289	0.004505997	-0.605
26	FBgn0051522	chr3R	-	4445331	4445415	4444505	4444562	4446165	4446246	0.005185443	0.156
27	FBgn0264908	chr3L	-	15852694	15852697	15852138	15852249	15859811	15860026	0.009223779	-0.289

28	FBgn0264908	chr3L	-	15867982	15868057	15867554	15867619	15868558	15868647	0.00329197	-0.195
29	FBgn0037705	chr3R	-	9549777	9549810	9546797	9547370	9551765	9551806	0.031991128	-0.223
30	FBgn0028490	chr2L	-	11525916	11526012	11524567	11525667	11528120	11528205	0	0.695
31	FBgn0259223	chrX	+	18424218	18424542	18423493	18423761	18426270	18426376	2.56533E-08	-0.226
32	FBgn0032889	chr2L	+	20825271	20825512	20824433	20824724	20825667	20826087	0.000599821	-0.253
33	FBgn0013995	chr3R	+	21008721	21008736	21008148	21008240	21011266	21011415	0.025605375	-0.131
34	FBgn0030976	chrX	+	18906074	18906134	18900730	18900960	18907498	18907668	5.13586E-05	0.178
35	FBgn0005632	chr3R	+	31777637	31777751	31777417	31777564	31778171	31778758	0.000798147	0.307
36	FBgn0085427	chr2L	+	8928059	8928743	8927551	8927707	8928989	8929663	0.038956342	-0.121
37	FBgn0263772	chrX	+	4087053	4087383	4086169	4086250	4088013	4088348	0.014014354	-0.396
38	FBgn0052982	chr2L	+	9135117	9135384	9126769	9127466	9148754	9148799	0.011188284	-0.165
39	FBgn0036318	chr3L	+	12852426	12852521	12852231	12852277	12852615	12852716	0.007022555	-0.173
40	FBgn0267002	chr2R	-	16764137	16764374	16763197	16763340	16772253	16772500	0	0.408
41	FBgn0267002	chr2R	-	16764137	16764374	16763197	16763340	16772257	16772671	0	0.651
42	FBgn0028487	chr3R	-	13696436	13696768	13696202	13696346	13696826	13697227	0.032800231	0.161
43	FBgn0028487	chr3R	-	13696438	13696768	13696202	13696346	13696826	13697227	0.01181087	0.169
44	FBgn0028487	chr3R	-	13696441	13696768	13696202	13696346	13696826	13697227	0.035780814	0.168
45	FBgn0028487	chr3R	-	13696443	13696768	13696202	13696346	13696826	13697227	0.00219646	0.191
46	FBgn0033807	chr2R	-	13213750	13213931	13213022	13213436	13214418	13214496	0	0.618
47	FBgn0052017	chr4	-	1144965	1145100	1143626	1143868	1146469	1146529	0.000167201	-0.667
48	FBgn0053208	chr3R	-	10004409	10004442	10003373	10003650	10004655	10008636	0.002542978	0.184
49	FBgn0000114	chr2L	+	12288904	12289344	12287222	12287808	12298567	12298749	6.82439E-07	0.284
50	FBgn0004838	chr2L	+	6922730	6922826	6920703	6920861	6923783	6924400	0	-0.206
51	FBgn0004242	chr2L	-	2789346	2789946	2787053	2787168	2792194	2792371	0.000548166	0.114
52	FBgn0034858	chr2R	+	23386789	23386827	23386520	23386622	23387006	23387612	0	-0.665
53	FBgn0032196	chr2L	-	10225873	10225965	10225503	10225811	10226664	10226808	0.003694517	0.228
54	FBgn0029580	chrX	+	1469804	1469953	1467492	1467617	1470014	1470097	7.80462E-06	-0.131
55	FBgn0027580	chr2R	-	9853682	9853730	9852547	9853205	9854510	9856020	0.000147017	-0.091
56	FBgn0027570	chr3R	-	4722607	4722640	4720980	4721390	4722773	4723150	0.000109897	0.19
57	FBgn0027779	chr2L	-	16725891	16726113	16724858	16724993	16727009	16727488	7.78081E-08	0.137
58	FBgn0033748	chr2R	-	12509083	12509470	12508892	12508994	12509570	12509719	0.002417784	0.272
59	FBgn0263120	chr2R	+	8674646	8674871	8666170	8666311	8676471	8676607	9.86054E-05	0.188
60	FBgn0053558	chr2R	-	6928508	6928836	6926018	6926298	6929822	6930098	0.009485827	0.215
61	FBgn0034093	chr2R	-	16226567	16226804	16225106	16225325	16230077	16230402	0.002273466	-0.447
62	FBgn0003023	chrX	-	8487256	8487382	8485748	8486993	8487576	8487713	0.001393389	0.349
63	FBgn0033504	chr2R	+	10296434	10300268	10295868	10296047	10304596	10304930	0.009485827	-0.497
64	FBgn0033504	chr2R	+	10300489	10300588	10295868	10296047	10304596	10304930	8.29421E-06	-0.415
65	FBgn0051523	chr3R	-	4472840	4473188	4468463	4468599	4475251	4475426	4.32853E-06	-0.432
66	FBgn0051523	chr3R	-	4472840	4473188	4468463	4468599	4478632	4479106	0.000113185	-0.311
67	FBgn0058263	chr2R	-	2515642	2515908	2494741	2495023	2553782	2553877	0.000109649	-0.611
68	FBgn0263077	chr2R	+	7630176	7630328	7628796	7629112	7634215	7634314	2.89768E-08	-0.35
69	FBgn0034051	chr2R	-	15937678	15937879	15935892	15936580	15938114	15938267	4.53328E-05	-0.258
70	FBgn0033725	chr2R	+	12387912	12387939	12387381	12387517	12388299	12388450	0	0.333
71	FBgn0031717	chr2L	+	5549016	5549076	5548167	5548709	5549443	5549614	0	-0.341
72	FBgn0031717	chr2L	+	5549016	5549081	5548167	5548709	5549443	5549614	6.5738E-12	-0.264
73	FBgn0031717	chr2L	+	5549135	5549218	5549016	5549076	5549443	5549614	0.010034518	0.165
74	FBgn0035348	chr3L	-	2501069	2501141	2499690	2500462	2502390	2502815	0.012060415	0.184
75	FBgn0035348	chr3L	-	2501069	2501141	2499690	2500462	2502594	2502723	0.004451012	0.146
76	FBgn0265356	chr2R	+	19004604	19004883	19001091	19001789	19005788	19006571	1.01382E-08	0.17
77	FBgn0265356	chr2R	+	19004604	19004889	19001091	19001789	19005788	19006571	4.16412E-05	0.12
78	FBgn0043070	chr2R	+	21507321	21507722	21502096	21502479	21510105	21510201	0.035085152	0.217
79	FBgn0005666	chr4	+	726661	726683	725668	726029	727460	727623	0.015224686	-0.14
80	FBgn0053547	chr3R	-	17893904	17894902	17887420	17887543	17896460	17896528	0.004938927	0.452
81	FBgn0039536	chr3R	+	27666784	27666802	27666426	27666539	27667099	27667244	0.025605375	0.281
82	FBgn0261988	chr3R	+	31410353	31410401	31405244	31405956	31433458	31433888	0	0.644
83	FBgn0034420	chr2R	+	19174576	19174783	19173703	19174121	19174881	19175086	2.60817E-08	-0.142
84	FBgn0034420	chr2R	+	19174576	19174783	19174338	19174365	19174881	19175086	9.25286E-05	-0.171
85	FBgn0264607	chr4	-	1043467	1043521	1040012	1040078	1045155	1045248	0.001989796	0.207
86	FBgn0011361	chr3L	+	1331041	1331170	1330862	1330964	1331279	1331543	4.26492E-07	-0.174
87	FBgn0031835	chr2L	-	6573698	6573805	6571953	6572542	6574015	6574320	0.034832428	0.27
88	FBgn0265376	chr3R	-	4598468	4598871	4596282	4597174	4599684	4599749	1.32467E-10	-0.72
89	FBgn0016930	chr2L	-	14229458	14229536	14222016	14222081	14233647	14234108	1.56367E-08	-0.26
90	FBgn0016930	chr2L	-	14229531	14229536	14222016	14222081	14233647	14234108	0	-0.38
91	FBgn0034897	chr2R	+	23722209	23722875	23714665	23714899	23729048	23729234	7.3663E-05	-0.089
92	FBgn0265991	chr2R	-	15773040	15773253	15765122	15767253	15773815	15773966	2.56533E-08	-0.172
93	FBgn0041182	chr2L	-	7697976	7698130	7696593	7696807	7698724	7698992	0.000317224	0.211
94	FBgn0085410	chr2L	+	5852883	5852898	5851475	5851826	5852992	5853258	0.030783629	-0.254
95	FBgn0086906	chr3L	-	2041565	2041586	2041392	2041497	2041709	2042015	1.92645E-06	0.176
96	FBgn0086906	chr3L	-	2045195	2046164	2043448	2045116	2046281	2046533	1.97527E-06	-0.18
97	FBgn0250819	chr4	+	1186328	1186634	1185767	1185834	1187734	1187880	0.004500945	-0.478
98	FBgn0032783	chr2L	-	19438008	19438133	19437665	19437845	19440663	19440907	0.01027526	0.2
99	FBgn0020762	chr2L	+	4334414	4334439	4333912	4334104	4336449	4336957	0.000317224	0.188
100	FBgn0030412	chrX	+	12647698	12647759	12643872	12644767	12648536	12649182	0.011188284	0.527
101	FBgn0052112	chr3L	+	12742572	12742650	12741860	12741949	12743512	12743625	0	-0.832
102	FBgn0267376	chr3R	-	10867971	10868337	10866808	10866960	10868438	10868642	0.001994078	0.356
103	FBgn0266178	chr3R	-	21355316	21355557	21354444	21355089	21355757	21356704	0.00026995	0.598
104	FBgn0266137	chr3R	-	29638947	29639105	29630303	29632860	29640228	29640310	2.47816E-05	-0.613
105	FBgn0036451	chr3L	+	14791269	14791550	14789934	14790072	14792027	14792460	0.027473838	-0.444
106	FBgn0264493	chr3R	-	13971267	13971667	13970951	13971102	13981633	13983363	1.59791E-08	-0.289
107	FBgn0264386	chrX	-	6133694	6133721	6132191	6132313	6135701	6135740	0.000147458	0.319
108	FBgn0263980	chr2R	+	15420650	15420784	15419350	15419551	15421526	15421643	0.030783629	-0.627
109	FBgn0263980	chr2R	+	15465244	15465506	15464470	15464686	15465649	15465745	0.000592824	0.297
110	FBgn0263980	chr2R	+	15465255	15465506	15464470	15464686	15465649	15465745	0.000738414	0.294
111	FBgn0263346	chr3R	+	4680708	4680837	4670796	4670850	4697190	4697542	0	0.797
112	FBgn0263346	chr3R	+	4680778	4681199	4659578	4660399	4697190	4697542	0.001787944	0.644
113	FBgn0263346	chr3R	+	4680778	4681199	4670796	4670850	4697190	4697542	0	0.796
114	FBgn0035162	chr3L	-	830621	830744	830033	830341	830907	831242	0.022497471	-0.167
115	FBgn0035162	chr3L	-	830621	830813	830033	830341	830907	831242	0.034300478	-0.156
116	FBgn0261984	chr3R	-	19860490	19860761	19857240	19857684	19860885	19861291	0	0.366
117	FBgn0261984	chr3R	-	19860646	19860788	19857240	19857684	19860885	19861291	0	0.311
118	FBgn0082582	chr3R	+	30547082	30547126	30546405	30546587	30548420	30548749	0	-0.98
119	FBgn0082582	chr3R	+	30547082	30547126	30546405	30546587	30548451	30548749	0	-0.99
120	FBgn0082582	chr3R	+	30547082	30547167	30546405	30546587	30548420	30548749	0	-0.989
121	FBgn0261588	chr2R	+	8394565	8394641	8393556	8393737	8395030	8395114	0.011024844	-0.327
122	FBgn0039790	chr3R	-	30595720	30596014	30595389	30595626	30599918	30600323	0	-0.814
123	FBgn0039790	chr3R	-	30601007	30601308	30595389	30595626	30601416	30601489	0.001539235	0.214
124	FBgn0039790	chr3R	-	30601154	30601308	30595389	30595626	30601416	30601489	0.000307394	0.235
125	FBgn0033494	chr2R	-	10152377	10152422	10150855	10151037	10152903	10153091	0.004500945	-0.284
126	FBgn0027356	chr2R	+	12609702	12609876	12609228	12609636	12611008	12612068	0.000796216	-0.58
127	FBgn0027356	chr2R	+	12609741	12609876	12609228	12609636	12611008	12612068	0.007704451	-0.422
128	FBgn0039897	chr4	+	243815	244247	243633	243748	244417	244834	0.037447673	0.094
129	FBgn0038181	chr3R	+	13774800	13774837	13774452	13774737	13775091	13775169	1.67865E-09	-0.116
130	FBgn0037705	chr3R	-	9549587	9549664	9546797	9547370	9549777	9549810	0.002801131	0.828
131	FBgn0037186	chr3L	-	22734580	22734736	22733151	22733588	22734908	22735983	1.13066E-07	0.413
132	FBgn0037186	chr3L	-	22734588	22734736	22733151	22733588	22734908	22735983	1.04195E-07	0.416
133	FBgn0037664	chr3R	+	9249283	9249556	9248824	9248973	9249919	9250199	2.24948E-06	0.159
134	FBgn0037664	chr3R	+	9249283	9249733	9248824	9248973	9249919	9250199	1.81542E-05	0.153
135	FBgn0037944	chr3R	-	11749241	11749302	11748789	11748959	11749697	11750029	9.57562E-09	-0.511
136	FBgn0259223	chrX	+	18424127	18424542	18423493	18423761	18426270	18426376	8.53635E-08	-0.174
137	FBgn0032889	chr2L	+	20825283	20825512	20824433	20824724	20825667	20826087	0.000471714	-0.245
138	FBgn0039667	chr3R	+	29495008	29495125	29494781	29494885	29495645	29495801	3.43532E-05	0.601
139	FBgn0025702	chr3L	+	22277483	22277841	22277363	22277427	22278822	22279044	0.020772279	0.333
140	FBgn0265959	chr3L	+	20248847	20249060	20248079	20248150	20255897	20256638	0.01481326	-0.155
141	FBgn0261625	chr2R	-	12674205	12674702	12673099	12673339	12674990	12675418	8.76884E-07	-0.3
142	FBgn0038720	chr3R	-	19620614	19620745	19619084	19619380	19623315	19623476	0.011188284	-0.126
143	FBgn0038720	chr3R	-	19620614	19620984	19619084	19619380	19623315	19623476	0.034023608	-0.122
144	FBgn0026597	chr3R	+	30023904	30024089	30022841	30023840	30024426	30024622	4.29174E-05	0.199
145	FBgn0027335	chrX	-	18636837	18636873	18635641	18636319	18637330	18637573	0.002326036	-0.553
146	FBgn0030976	chrX	+	18906084	18906134	18900730	18900960	18907498	18907668	0.00417364	0.183
147	FBgn0030976	chrX	+	18906088	18906134	18900730	18900960	18907498	18907668	0.004077185	0.182
148	FBgn0052982	chr2L	+	9135121	9135384	9126880	9127466	9148754	9148799	1.14404E-07	-0.452
149	FBgn0036318	chr3L	+	12852426	12852543	12852231	12852277	12852615	12852716	0.001044783	-0.225
150	FBgn0035346	chr3L	+	2490797	2490847	2490219	2490488	2490979	2492613	0.00198697	0.171
151	FBgn0027561	chr2R	-	9124743	9125103	9123303	9124139	9125550	9126066	0.000387288	-0.472
152	FBgn0027561	chr2R	-	9124743	9125204	9123303	9124139	9125550	9126066	0.000599323	-0.463
153	FBgn0028487	chr3R	-	13696448	13696768	13696202	13696346	13696826	13697227	0.013637805	0.171
154	FBgn0033807	chr2R	-	13213532	13213931	13213022	13213436	13214418	13214496	0	0.776
155	FBgn0033807	chr2R	-	13213532	13213949	13213022	13213436	13214418	13214496	8.53635E-08	0.943
156	FBgn0033807	chr2R	-	13213750	13213949	13213022	13213436	13214418	13214496	0	0.719
157	FBgn0264894	chr2L	+	13274989	13275061	13274476	13274850	13275626	13275838	8.9317E-05	-0.493
158	FBgn0052017	chr4	-	1144965	1145103	1143626	1143868	1146469	1146529	0.001539235	-0.628
159	FBgn0004368	chrX	+	4953724	4953807	4953492	4953621	4954116	4954624	0.001311104	0.25

160	FBgn0004169	chrX	-	13595003	13595502	13593473	13594050	13596375	13596748	0.003311941	-0.229
161	FBgn0004169	chrX	-	13595319	13595502	13593473	13594050	13596375	13596748	0.000650785	-0.469
162	FBgn0085432	chr4	+	97698	97975	94422	94615	99405	99475	0.005185443	0.259
163	FBgn0004838	chr2L	+	6922731	6922826	6920703	6920861	6923783	6924400	3.96825E-05	0.558
164	FBgn0004242	chr2L	-	2798479	2798836	2793647	2793908	2799908	2799979	0	-0.368
165	FBgn0011259	chr2L	+	8664374	8664430	8664157	8664220	8666035	8672041	3.74735E-06	0.679
166	FBgn0029580	chrX	+	1467696	1467964	1467492	1467617	1470014	1470097	2.65149E-12	-0.324
167	FBgn0263120	chr2R	+	8666767	8666794	8665812	8666071	8674646	8674871	0.002358729	0.368
168	FBgn0263120	chr2R	+	8674743	8674871	8666170	8666311	8676471	8676607	0.000362726	0.197
169	FBgn0263120	chr2R	+	8674820	8674871	8666170	8666311	8676471	8676607	0.001311104	0.191
170	FBgn0011224	chr3R	-	31933453	31933644	31873833	31874099	31959034	31959127	0.002672225	-0.241
171	FBgn0028516	chr2L	-	15245557	15245654	15241410	15241521	15246173	15246352	0.022219139	0.143
172	FBgn0028516	chr2L	-	15245557	15245688	15241410	15241521	15246173	15246352	0.005185443	0.153
173	FBgn0053558	chr2R	-	6929822	6930092	6925701	6926298	6930248	6930370	8.44004E-06	-0.754
174	FBgn0032375	chr2L	+	11790277	11790310	11790175	11790214	11790369	11790426	0.000353208	0.627
175	FBgn0034093	chr2R	-	16226449	16226804	16225106	16225325	16230077	16230402	1.51388E-05	-0.523
176	FBgn0032156	chr2L	-	9905137	9905291	9904826	9905083	9905677	9905818	0.003726011	0.364
177	FBgn0016726	chr2R	+	21293849	21293878	21293767	21293789	21294053	21294161	0.02973785	0.396
178	FBgn0034645	chr2R	+	21658216	21658275	21658077	21658109	21658345	21658480	1.04125E-08	0.176
179	FBgn0034645	chr2R	+	21658216	21658275	21658077	21658109	21658350	21658480	0.000137953	0.203
180	FBgn0034645	chr2R	+	21658219	21658275	21658077	21658109	21658345	21658480	2.39415E-08	0.172
181	FBgn0034645	chr2R	+	21658219	21658275	21658077	21658109	21658350	21658480	0.000571157	0.192
182	FBgn0013576	chr3R	-	5335713	5336072	5334404	5334698	5349467	5350010	0.001144227	0.427
183	FBgn0053170	chr3L	+	22867115	22867340	22865877	22866034	22867399	22867535	0.000339531	0.701
184	FBgn0053170	chr3L	+	22867191	22867340	22865877	22866034	22867399	22867535	0.022371091	0.561
185	FBgn0263077	chr2R	+	7629870	7630328	7628800	7629112	7634215	7634314	8.53635E-08	-0.425
186	FBgn0263077	chr2R	+	7630056	7630328	7628800	7629112	7634215	7634314	5.54933E-08	-0.421
187	FBgn0263077	chr2R	+	7630147	7630328	7628800	7629112	7634215	7634314	3.43211E-08	-0.428
188	FBgn0263077	chr2R	+	7630185	7630328	7628800	7629112	7634215	7634314	1.20061E-07	-0.434
189	FBgn0010548	chr2R	-	7473915	7473993	7473721	7473843	7474571	7474659	0.030485001	-0.211
190	FBgn0034075	chr2R	+	16114939	16115053	16114771	16114866	16115353	16115594	0.010091058	0.121
191	FBgn0034075	chr2R	+	16114939	16115252	16114771	16114866	16115353	16115594	0.009383092	0.116
192	FBgn0015777	chr2L	-	6797754	6797939	6791700	6791841	6798227	6798516	0.00022891	-0.34
193	FBgn0260635	chr3L	-	16046484	16046696	16038409	16040065	16050457	16051034	0.005185443	0.157
194	FBgn0265356	chr2R	+	19004613	19004883	19001091	19001789	19005788	19006571	3.14499E-05	0.117
195	FBgn0042696	chr4	-	275662	275719	273760	273916	276290	276369	0.037860405	0.354
196	FBgn0027515	chr2L	+	7821832	7822056	7821534	7821648	7822641	7823100	3.34543E-06	-0.295
197	FBgn0005666	chr4	+	728334	728413	728103	728245	728672	728814	0.030783629	0.179
198	FBgn0005666	chr4	+	728334	728584	728103	728245	728672	728814	0.000449495	0.262
199	FBgn0038294	chr3R	-	15171934	15172106	15170624	15171374	15174136	15174343	0.006856924	-0.422
200	FBgn0034420	chr2R	+	19174305	19174783	19173703	19174121	19174881	19175086	2.23056E-10	-0.19
201	FBgn0034420	chr2R	+	19174349	19174783	19173703	19174121	19174881	19175086	2.41539E-10	-0.189
202	FBgn0025726	chr4	-	885453	885605	884045	884120	885759	885891	0.034300478	0.593
203	FBgn0025726	chr4	-	885453	885687	884045	884120	885759	885891	0.00402337	0.63
204	FBgn0031835	chr2L	-	6573698	6573917	6571953	6572542	6574015	6574320	0.010948107	0.217
205	FBgn0283451	chrX	+	1622859	1622988	1613865	1613945	1624137	1624707	0.015469499	0.311
206	FBgn0011640	chr3L	+	7339728	7339905	7339463	7339617	7339961	7340770	4.32853E-06	-0.7
207	FBgn0011640	chr3L	+	7339833	7339905	7339463	7339617	7339961	7340770	1.76493E-06	-0.698
208	FBgn0016930	chr2L	-	14216962	14217069	14197656	14197867	14222016	14222081	0.000767947	-0.186
209	FBgn0034897	chr2R	+	23722209	23722601	23714665	23714899	23729048	23729234	0	-0.183
210	FBgn0265991	chr2R	-	15766861	15767253	15765122	15766713	15773815	15773966	0	0.325
211	FBgn0020908	chr2R	-	2937670	2937822	2932785	2933284	2954996	2955077	0.005117343	-0.067
212	FBgn0020908	chr2R	-	2937670	2937825	2932785	2933284	2954996	2955077	0.005107463	-0.067
213	FBgn0020908	chr2R	-	2937670	2937832	2932785	2933284	2954996	2955077	0.005107463	-0.067
214	FBgn0020908	chr2R	-	2937724	2937823	2932826	2933284	2954996	2955077	0	-0.097
215	FBgn0020908	chr2R	-	2937724	2937823	2932826	2933284	2963055	2963222	0	-0.356
216	FBgn0020908	chr2R	-	2954996	2955047	2932785	2933284	2963055	2963222	0	-0.344
217	FBgn0020908	chr2R	-	2954996	2955055	2932785	2933284	2963055	2963222	0	-0.307
218	FBgn0020908	chr2R	-	2954996	2955084	2932785	2933284	2963055	2963222	6.15434E-14	-0.211
219	FBgn0020908	chr2R	-	2954996	2955086	2932785	2933284	2963055	2963222	0	-0.212
220	FBgn0020908	chr2R	-	2954996	2955102	2932785	2933284	2963055	2963222	0	-0.214
221	FBgn0020908	chr2R	-	2954996	2955119	2932785	2933284	2963055	2963222	0	-0.234
222	FBgn0020908	chr2R	-	2955026	2955077	2937670	2937823	2963055	2963222	9.37933E-08	-0.082
223	FBgn0027341	chr3R	+	4133979	4134172	4133706	4133919	4135259	4135381	0.005117343	-0.214
224	FBgn0086778	chrX	-	19334414	19334524	19331251	19331592	19340502	19340562	0.026770206	0.192
225	FBgn0250819	chr4	+	1185885	1185966	1185767	1185834	1186472	1186634	0.009485827	0.308
226	FBgn0250819	chr4	+	1185885	1185966	1185767	1185834	1187521	1187572	0.001324876	0.273
227	FBgn0250819	chr4	+	1185892	1185966	1185767	1185834	1186472	1186634	0.00878452	0.314
228	FBgn0250819	chr4	+	1186177	1186634	1185767	1185834	1187734	1187880	0.010948107	-0.389
229	FBgn0250819	chr4	+	1186499	1186634	1185767	1185834	1187734	1187880	0.003850655	-0.32
230	FBgn0250819	chr4	+	1186503	1186634	1185767	1185834	1187734	1187880	0.012256024	-0.384
231	FBgn0260008	chr3L	+	25121617	25121745	25120877	25120985	25125543	25126171	2.67666E-11	-0.497
232	FBgn0260008	chr3L	+	25121617	25121968	25120877	25120985	25125543	25126171	2.38933E-13	-0.423
233	FBgn0041150	chr2L	-	4925828	4926263	4912671	4912778	4931913	4932384	6.16895E-10	0.38
234	FBgn0261552	chr3R	+	9440680	9441140	9439345	9439705	9441415	9441665	8.12664E-06	0.364

4) List of DASs in *Drosophila* adults of *DmAnkrd11*$^{\Delta/\Delta}$ *vs WT*

A3SS

	FlyBase ID	chr	strand	longExonStart_0base	longExonEnd	shortES	shortEE	flankingES	flankingEE	FDR	∆PSI
1	FBgn0016081	chr3L	-	9631833	9632173	9631833	9632170	9632232	9632599	0	0.344
2	FBgn0267376	chr3R	-	10866808	10866976	10866808	10866960	10867971	10868290	0	0.518
3	FBgn0267376	chr3R	-	10866808	10866976	10866808	10866960	10868438	10868642	0	0.848
4	FBgn0266579	chr3R	-	27641841	27642938	27641841	27642057	27643814	27643973	0.003544904	-0.502
5	FBgn0266186	chr2R	-	9841846	9842225	9841846	9842167	9842300	9842502	0.046295511	-0.101
6	FBgn0264489	chr3L	+	9831920	9832679	9832198	9832679	9830633	9830785	0	-0.165
7	FBgn0264489	chr3L	+	9832195	9832679	9832198	9832679	9830633	9830785	0	-0.748
8	FBgn0264489	chr3L	+	9831920	9832679	9832195	9832679	9830999	9831440	0	0.733
9	FBgn0264489	chr3L	+	9831920	9832679	9832198	9832679	9830999	9831440	0.035986585	0.202
10	FBgn0264489	chr3L	+	9832195	9832679	9832198	9832679	9830999	9831440	0	-0.669
11	FBgn0039790	chr3R	-	30593704	30593819	30593704	30593816	30594112	30594169	7.85178E-07	-0.603
12	FBgn0264908	chr3L	-	15852138	15852276	15852138	15852249	15859811	15860026	0.005875567	-0.415
13	FBgn0030976	chrX	+	18908249	18908374	18908276	18908374	18907730	18907859	0.010764626	0.142
14	FBgn0038167	chr3R	+	13679140	13679430	13679150	13679430	13678749	13679066	0.049562982	0.295
15	FBgn0040694	chr3L	-	3620741	3620812	3620741	3620809	3620871	3621032	0	-0.915
16	FBgn0051092	chr3R	-	25718397	25718623	25718397	25718556	25719951	25720117	3.90525E-06	-0.216
17	FBgn0030089	chrX	+	9110617	9111005	9110821	9111005	9109549	9109987	0.000178757	0.323
18	FBgn0034075	chr2R	+	16112195	16112530	16112201	16112530	16111982	16112141	0	0.225
19	FBgn0033809	chr2R	-	13217588	13218303	13217588	13218199	13219087	13219247	0.027035586	0.31
20	FBgn0011640	chr3L	+	7339867	7340770	7339961	7340770	7339463	7339617	0	-0.445
21	FBgn0016694	chr3L	-	7830386	7831827	7830386	7831810	7832553	7833073	0.000451565	-0.432
22	FBgn0052181	chr3L	+	17759932	17760238	17759951	17760238	17759696	17759882	0.024176416	-0.668
23	FBgn0285963	chr3R	+	26916125	26916562	26916502	26916562	26915760	26915908	0	-0.985
24	FBgn0285963	chr3R	+	26916462	26916562	26916502	26916562	26915760	26915908	0	-0.992
25	FBgn0285963	chr3R	+	26916463	26916562	26916502	26916562	26915760	26915908	0	-0.994
26	FBgn0285963	chr3R	+	26916502	26916562	26916506	26916562	26915760	26915908	0	0.798
27	FBgn0285963	chr3R	+	26916502	26916562	26916512	26916562	26915760	26915908	0	0.845
28	FBgn0267376	chr3R	-	10866808	10866994	10866808	10866960	10868438	10868642	0	0.818
29	FBgn0036331	chr3L	+	13008622	13008873	13008654	13008873	13008284	13008567	0	-0.521
30	FBgn0263102	chr2R	+	10561685	10561954	10561695	10561954	10557887	10558533	0	-0.565
31	FBgn0262737	chr3L	+	21916079	21916572	21916140	21916572	21915535	21915655	0.004722959	0.628
32	FBgn0262579	chr3L	+	8099594	8100035	8099733	8100035	8099145	8099536	0	0.774
33	FBgn0262579	chr3L	+	8099601	8100035	8099733	8100035	8099145	8099536	0	0.951
34	FBgn0262483	chr3R	+	15387032	15387370	15387185	15387370	15386716	15386917	0.009445682	-0.686
35	FBgn0264489	chr3L	+	9832192	9832679	9832198	9832679	9830633	9830785	0	-0.871
36	FBgn0264489	chr3L	+	9831920	9832679	9832531	9832679	9830999	9831440	0	0.969
37	FBgn0264489	chr3L	+	9832195	9832679	9832531	9832679	9830999	9831440	0	0.762
38	FBgn0264489	chr3L	+	9832198	9832679	9832531	9832679	9830999	9831440	0	0.912
39	FBgn0264489	chr3L	+	9832201	9832679	9832531	9832679	9830999	9831440	0	0.966
40	FBgn0264357	chr3R	+	21210788	21211079	21210791	21211079	21210366	21210705	0.048623382	-0.126
41	FBgn0039137	chr3R	-	23990714	23991202	23990714	23991027	23991773	23992036	0.00911879	-0.667
42	FBgn0053092	chr3R	+	21878150	21878305	21878251	21878305	21877935	21878071	0	0.982
43	FBgn0053092	chr3R	+	21878154	21878305	21878251	21878305	21877935	21878071	0	0.982
44	FBgn0053092	chr3R	+	21878171	21878305	21878251	21878305	21877935	21878071	0	0.927
45	FBgn0039481	chr3R	-	27090671	27090910	27090671	27090801	27090979	27091130	8.82954E-09	-0.892
46	FBgn0039301	chr3R	+	25247846	25248106	25247958	25248106	25247657	25247777	0.009177868	-0.372
47	FBgn0261932	chr3R	+	17407052	17407199	17407101	17407199	17406677	17406769	0.002587972	0.515
48	FBgn0085408	chr2R	+	14315103	14315533	14315104	14315533	14308141	14308293	1.52094E-07	0.57
49	FBgn0085408	chr2R	+	14315103	14315533	14315236	14315533	14308141	14308293	3.35152E-06	0.235
50	FBgn0013334	chr3R	+	15935937	15936340	15935981	15936340	15935118	15935584	0.034723142	-0.313
51	FBgn0012344	chr3R	+	9649776	9650110	9649800	9650110	9648982	9649723	1.77958E-12	-0.819
52	FBgn0036768	chr3L	+	17889313	17889462	17889330	17889462	17887704	17887790	1.87907E-10	0.815
53	FBgn0010399	chr3R	-	5516211	5516478	5516211	5516408	5523178	5523328	0.03490353	0.23
54	FBgn0031589	chr2L	-	4196474	4196814	4196474	4196779	4197095	4197191	0.002552366	0.624
55	FBgn0262484	chr3L	-	3484457	3484502	3484457	3484496	3484569	3484653	3.68603E-08	0.854
56	FBgn0259146	chr3R	+	25970614	25970936	25970840	25970936	25970079	25970291	0.013633468	-0.47
57	FBgn0051092	chr3R	-	25718397	25718614	25718397	25718556	25719951	25720117	0	0.51
58	FBgn0051092	chr3R	-	25718397	25718788	25718397	25718556	25719951	25720117	0.032634464	-0.22
59	FBgn0051092	chr3R	-	25718397	25718623	25718397	25718614	25719951	25720117	0	-0.684
60	FBgn0051092	chr3R	-	25718397	25718788	25718397	25718614	25719951	25720117	5.49159E-07	-0.996
61	FBgn0051092	chr3R	-	25718397	25718614	25718397	25718556	25719965	25720117	0	0.588
62	FBgn0051092	chr3R	-	25718397	25718623	25718397	25718614	25719965	25720117	0	-0.613
63	FBgn0262719	chr3L	-	8506107	8506295	8506107	8506199	8507089	8507146	0.024141875	0.492
64	FBgn0262719	chr3L	-	8506107	8506306	8506107	8506199	8507089	8507146	0.046075885	0.545
65	FBgn0031118	chrX	-	20509365	20509682	20509365	20509498	20510518	20510694	0.012841395	0.378
66	FBgn0030114	chrX	+	9255633	9255953	9255636	9255953	9254112	9254349	0.000660145	0.66
67	FBgn0036493	chr3L	+	15306504	15306798	15306512	15306798	15305576	15305679	8.36597E-10	-0.263
68	FBgn0002921	chr3R	+	20959238	20959380	20959348	20959380	20957733	20958167	0.049562982	0.158
69	FBgn0038294	chr3R	-	15172599	15173062	15172599	15172830	15173980	15174343	0	0.298
70	FBgn0038294	chr3R	-	15172601	15173063	15172601	15172830	15173980	15174343	0	0.298
71	FBgn0038294	chr3R	-	15172601	15173063	15172601	15172830	15174136	15174343	0	0.201
72	FBgn0038294	chr3R	-	15172601	15173063	15172601	15173062	15174136	15174343	0	0.449
73	FBgn0001228	chr3L	+	9373389	9373491	9373406	9373491	9373161	9373321	0	0.614
74	FBgn0031857	chr2L	-	6770590	6770718	6770590	6770709	6772551	6772755	0	-0.588
75	FBgn0031857	chr2L	-	6770590	6770995	6770590	6770709	6772551	6772755	0	-0.869
76	FBgn0259243	chr3L	+	20829079	20829319	20829291	20829319	20828224	20828340	0.011877786	0.389
77	FBgn0259243	chr3L	+	20829092	20829319	20829291	20829319	20828224	20828340	0.004722959	0.453
78	FBgn0259243	chr3L	+	20829249	20829319	20829291	20829319	20828224	20828340	0.011131424	0.376
79	FBgn0259938	chr3R	+	10396679	10396820	10396701	10396820	10388257	10389437	0.036070916	-0.536
80	FBgn0262351	chr3L	+	18925443	18925585	18925458	18925585	18925090	18925387	0.000900646	0.385
81	FBgn0262351	chr3L	+	18925453	18925585	18925458	18925585	18925090	18925387	0.001149563	0.521
82	FBgn0260997	chr3L	-	26355872	26356260	26355872	26356242	26371226	26371356	0.014571144	-0.517
83	FBgn0261574	chr3L	+	20006285	20006653	20006518	20006653	20005300	20005489	0.025613025	0.667
84	FBgn0016694	chr3L	-	7830386	7831827	7830386	7831810	7832861	7833073	0.001956511	-0.456

A5SS

	FlyBase ID	chr	strand	longExonStart_0base	longExonEnd	shortES	shortEE	flankingES	flankingEE	FDR	∆PSI
1	FBgn0026418	chr3L	+	14037018	14037136	14037018	14037111	14037477	14037750	0.013813522	0.286
2	FBgn0026418	chr3L	+	14037018	14037412	14037018	14037111	14037477	14037750	1.83526E-12	0.598
3	FBgn0026418	chr3L	+	14037018	14037412	14037018	14037136	14037477	14037750	5.033E-12	0.595
4	FBgn0036433	chr3L	+	14622795	14623033	14622795	14622935	14626840	14626894	1.00245E-10	-0.188
5	FBgn0266136	chr3L	-	19793427	19793569	19793453	19793569	19789963	19790254	0.002716638	-0.416
6	FBgn0028371	chr2R	-	22729131	22729272	22729141	22729272	22727563	22727781	0.004425867	-0.605
7	FBgn0263289	chr3R	+	26536349	26536601	26536349	26536397	26537370	26537784	0.001417323	-0.308
8	FBgn0263289	chr3R	+	26536349	26536601	26536349	26536531	26537370	26537784	1.12275E-12	-0.486
9	FBgn0262623	chr3L	-	13299981	13300152	13299985	13300152	13299840	13299929	0	0.324
10	FBgn0262532	chr3L	+	17869615	17870019	17869615	17870007	17870078	17870573	7.70377E-10	0.322
11	FBgn0264489	chr3L	+	9813846	9813961	9813846	9813922	9820073	9820559	4.07431E-09	-0.738
12	FBgn0032666	chr2L	+	18122527	18122920	18122527	18122588	18123348	18123908	0.001445096	-0.216
13	FBgn0038947	chr3R	+	22356684	22357047	22356684	22356798	22357530	22357671	0.004348937	-0.158
14	FBgn0031939	chr2L	-	7732478	7732670	7732516	7732670	7730197	7730849	0.002315495	-0.532
15	FBgn0037429	chr3R	+	6335921	6336198	6335921	6336171	6336279	6336461	0.011973286	-0.394
16	FBgn0265959	chr3L	+	20259985	20260487	20259985	20260310	20260861	20260989	1.97688E-05	0.256
17	FBgn0003175	chr2R	-	22561898	22562065	22561918	22562065	22560745	22561142	7.56298E-05	0.488
18	FBgn0260388	chr3L	-	16496052	16496236	16496138	16496236	16495299	16495536	0	0.537
19	FBgn0260745	chr3R	-	11993131	11993315	11993248	11993315	11992741	11992852	0	0.16
20	FBgn0260745	chr3R	-	11993131	11993315	11993248	11993315	11992741	11992874	0	0.184
21	FBgn0034049	chr2R	-	15931523	15931935	15931736	15931935	15926744	15927624	0.038991601	0.112
22	FBgn0033609	chr2R	-	11289392	11289772	11289669	11289772	11287644	11288560	0.019243705	0.238
23	FBgn0036685	chr3L	-	17015672	17016051	17015742	17016051	17015140	17015266	1.36054E-08	0.142

24	FBgn0038294	chr3R	-	15173980	15174343	15174136	15174343	15172843	15173063	0	-0.222
25	FBgn0003429	chr3R	+	24664896	24665424	24664896	24665085	24669540	24669879	0.000979063	0.711
26	FBgn0051224	chr3R	-	18658525	18659309	18658759	18659309	18656164	18657185	0.021157597	-0.254
27	FBgn0031914	chr2L	+	7440793	7441756	7440793	7440851	7443583	7443832	0.001065988	-0.454
28	FBgn0031914	chr2L	+	7440793	7441756	7440793	7440958	7443583	7443832	0	-0.791
29	FBgn0031914	chr2L	+	7440793	7441756	7440793	7440962	7443583	7443832	0	-0.713
30	FBgn0031515	chr2L	-	3294082	3294706	3294230	3294706	3293322	3293949	0.037377569	-0.345
31	FBgn0086711	chr2L	-	14996713	14997559	14997515	14997559	14993898	14994035	8.00294E-07	-0.578
32	FBgn0086711	chr2L	-	14997270	14997559	14997515	14997559	14993898	14994035	8.59833E-07	-0.394
33	FBgn0261553	chr3L	+	11215778	11215987	11215778	11215951	11216585	11217282	3.2227E-12	-0.646
34	FBgn0023479	chr3L	+	9075671	9075866	9075671	9075832	9076019	9076265	7.8931E-05	-0.39
35	FBgn0026418	chr3L	+	14037018	14037421	14037018	14037111	14037477	14037750	5.70729E-12	0.601
36	FBgn0026418	chr3L	+	14037018	14037421	14037018	14037136	14037477	14037750	4.9673E-11	0.59
37	FBgn0036433	chr3L	+	14622795	14622946	14622795	14622935	14626840	14626894	0	-0.751
38	FBgn0036728	chr3L	+	17474893	17475343	17474893	17475170	17475409	17475594	0.001766016	0.534
39	FBgn0052219	chr3L	-	19951002	19951135	19951008	19951135	19950765	19950936	0	0.668
40	FBgn0284252	chr2R	-	24514991	24515320	24515147	24515320	24514535	24514870	0.000730026	-0.675
41	FBgn0267390	chr3L	+	15566012	15566799	15566012	15566079	15568205	15568970	0.003401109	-0.556
42	FBgn0023001	chr3L	+	7138225	7138511	7138225	7138350	7139172	7139327	0.021036071	0.385
43	FBgn0265363	chr2R	-	11844745	11845099	11844818	11845099	11844377	11844672	3.30809E-12	0.801
44	FBgn0015218	chr3L	-	9401574	9401718	9401656	9401718	9401039	9401367	0.034273651	0.166
45	FBgn0015218	chr3L	-	9401580	9401718	9401656	9401718	9401039	9401367	0.037946069	0.168
46	FBgn0015218	chr3L	-	9401606	9401718	9401656	9401718	9401039	9401367	0.000385506	0.201
47	FBgn0015218	chr3L	-	9402055	9402225	9402170	9402225	9401039	9401367	0.001172215	0.501
48	FBgn0031574	chr2L	+	3863129	3863462	3863129	3863370	3863554	3864195	0.037946069	0.29
49	FBgn0264695	chr2L	+	16766565	16766776	16766565	16766756	16768583	16768884	0.031254434	0.166
50	FBgn0264695	chr2L	+	16766733	16767130	16766733	16766776	16768583	16768884	0.010344568	-0.481
51	FBgn0266702	chr3R	-	5835990	5836178	5836008	5836178	5835477	5835938	0.02366587	0.297
52	FBgn0265150	chr2L	+	262202	262593	262202	262586	262644	263003	0.041645381	-0.333
53	FBgn0035397	chr3L	+	3072546	3072760	3072546	3072677	3073080	3073478	0.007573847	-0.322
54	FBgn0035397	chr3L	+	3072546	3072841	3072546	3072677	3073080	3073478	0.003116537	-0.353
55	FBgn0262975	chr3R	-	23226276	23226711	23226453	23226711	23222796	23223120	0.008878582	0.439
56	FBgn0262593	chr3L	+	2895119	2895518	2895119	2895284	2897243	2897429	0	-0.837
57	FBgn0082582	chr3R	+	30573136	30573543	30573136	30573249	30578116	30578905	0.009000602	-0.275
58	FBgn0082582	chr3R	+	30573136	30573625	30573136	30573249	30578116	30578905	0.000265084	-0.371
59	FBgn0082582	chr3R	+	30573136	30573633	30573136	30573249	30578116	30578905	0.000550126	-0.342
60	FBgn0264489	chr3L	+	9813846	9813973	9813846	9813922	9820279	9820559	0.004348937	-0.189
61	FBgn0264489	chr3L	+	9813846	9813973	9813846	9813927	9820279	9820559	0.00012736	-0.613
62	FBgn0264489	chr3L	+	9813846	9813975	9813846	9813927	9820279	9820559	0.011289468	-0.576
63	FBgn0000206	chr3R	+	25961993	25962043	25961993	25962032	25963441	25963615	0.006687015	-0.198
64	FBgn0000206	chr3R	+	25961993	25962221	25961993	25962032	25963441	25963615	0	-0.871
65	FBgn0038947	chr3R	+	22356684	22356852	22356684	22356744	22357530	22357671	2.18228E-07	-0.397
66	FBgn0038947	chr3R	+	22356684	22356852	22356684	22356767	22357530	22357671	2.47075E-05	-0.477
67	FBgn0038947	chr3R	+	22356684	22357075	22356684	22356798	22357530	22357671	0.001795988	-0.162
68	FBgn0264776	chr3R	-	15508614	15508886	15508663	15508886	15507939	15508562	5.59496E-05	-0.13
69	FBgn0038140	chr3R	+	13342331	13342534	13342331	13342530	13343585	13343809	0.003339469	-0.685
70	FBgn0000056	chr2L	+	14615551	14615998	14615551	14615876	14616302	14616437	0.000584366	-0.252
71	FBgn0264495	chr3R	+	6406854	6407191	6406854	6407077	6407477	6407774	0.019079806	-0.583
72	FBgn0010435	chr2R	-	24982748	24983204	24983050	24983204	24979532	24979661	0.003621114	0.523
73	FBgn0010435	chr2R	-	24982925	24983204	24983050	24983204	24979532	24979661	0.000191832	0.791
74	FBgn0031220	chr2L	-	119291	119540	119430	119540	119133	119235	0.004048353	0.508
75	FBgn0037315	chr3R	-	5362031	5362209	5362102	5362209	5359978	5361760	0.021019831	0.485
76	FBgn0039637	chr3R	+	29123369	29123489	29123369	29123479	29123800	29124104	0	0.603
77	FBgn0039637	chr3R	+	29123415	29123499	29123415	29123479	29123800	29124104	0.011289468	0.275
78	FBgn0031187	chrX	+	22456445	22456559	22456445	22456508	22457426	22457551	0.026742132	0.355
79	FBgn0025702	chr3L	+	22282903	22283149	22282903	22283138	22283202	22284335	0	0.373
80	FBgn0025702	chr3L	+	22282903	22283149	22282903	22283144	22283202	22284335	0	0.37
81	FBgn0051286	chr3R	+	6641917	6642358	6641917	6642246	6642413	6643346	0	-0.748
82	FBgn0033919	chr2R	-	14267338	14267497	14267353	14267497	14264128	14264215	0.016921113	-0.415
83	FBgn0034071	chr2R	+	16048983	16049411	16048983	16049405	16049486	16049619	0.036600601	0.441
84	FBgn0283471	chrX	-	18113729	18113916	18113754	18113916	18113446	18113575	0.001004362	-0.558
85	FBgn0053113	chr2L	-	4999939	5000370	5000201	5000370	4999224	4999385	0.013796306	0.69
86	FBgn0031145	chrX	+	21035830	21036155	21035830	21036090	21036398	21036470	0.012487794	-0.607
87	FBgn0038256	chr3R	-	14731262	14731509	14731404	14731509	14730510	14730629	0	-0.359
88	FBgn0038256	chr3R	-	14731383	14731509	14731404	14731509	14730510	14730629	2.19697E-07	-0.246
89	FBgn0038256	chr3R	-	14731404	14731509	14731408	14731509	14730510	14730629	1.34123E-10	0.428
90	FBgn0031942	chr2L	-	7752976	7753153	7752989	7753153	7752169	7752814	0	-0.935
91	FBgn0052944	chr3R	-	4419106	4419578	4419359	4419578	4415682	4415886	2.63624E-10	0.794
92	FBgn0033054	chr2R	-	6039840	6040334	6040225	6040334	6038070	6038446	0.002317144	-0.337
93	FBgn0033807	chr2R	-	13213532	13213931	13213750	13213931	13213022	13213436	0.000425628	-0.125
94	FBgn0023083	chr3R	+	18585879	18586194	18585879	18585939	18586278	18589459	0.000374829	-0.561
95	FBgn0023083	chr3R	+	18585879	18586202	18585879	18585939	18586278	18589459	0.00038639	-0.558
96	FBgn0003175	chr2R	-	22561898	22562065	22561933	22562065	22560745	22561142	0.000289413	0.582
97	FBgn0004169	chrX	-	13602144	13602506	13602413	13602506	13600069	13600117	0.017247125	0.367
98	FBgn0053208	chr3R	-	10042571	10042821	10042626	10042821	10041971	10041998	0.029930996	-0.218
99	FBgn0037533	chr3R	-	7977736	7977962	7977937	7977962	7977561	7977675	0.037946069	-0.333
100	FBgn0085432	chr4	+	107071	107223	107071	107169	107402	114270	0.010761964	-0.561
101	FBgn0285913	chr3R	+	14298258	14298692	14298258	14298669	14298789	14298987	0	-0.852
102	FBgn0037138	chr3L	-	21839902	21840127	21840098	21840127	21839665	21839831	0.015297216	-0.568
103	FBgn0037138	chr3L	-	21840006	21840127	21840098	21840127	21839665	21839831	0.045512969	-0.575
104	FBgn0004242	chr2R	-	2799488	2799979	2799839	2799979	2798485	2798836	0.000495734	0.719
105	FBgn0051092	chr3R	-	25740244	25740465	25740245	25740465	25739054	25739622	0	0.575
106	FBgn0000308	chr2L	-	5980025	5980481	5980370	5980481	5979411	5979704	0.009000602	-0.75
107	FBgn0038098	chr3R	-	12974136	12974450	12974284	12974450	12973576	12973844	0.016506649	0.583
108	FBgn0029155	chr3R	+	27156140	27156518	27156140	27156305	27157143	27157603	6.88539E-08	-0.148
109	FBgn0028670	chr3R	-	18396712	18396928	18396785	18396928	18393271	18393409	0.023283557	-0.41
110	FBgn0000559	chr2L	-	21681619	21681804	21681649	21681804	21681343	21681558	1.53925E-11	1
111	FBgn0028956	chr2R	-	17452141	17452598	17452494	17452598	17450528	17451032	3.71033E-11	1
112	FBgn0039909	chr4	-	570400	570533	570404	570533	570233	570343	0.034658762	0.308
113	FBgn0263995	chr3R	+	17944105	17944232	17944105	17944213	17947193	17947598	0.02102918	-0.317
114	FBgn0052803	chrX	-	1901667	1901911	1901712	1901911	1901436	1901614	0.000281583	-0.529
115	FBgn0011672	chr3R	-	21060315	21060653	21060584	21060653	21058260	21058468	0.013796306	0.675
116	FBgn0260745	chr3R	-	11992944	11993315	11993248	11993315	11992741	11992852	5.81116E-05	0.108
117	FBgn0051183	chr3R	-	15875565	15875939	15875572	15875939	15869172	15870232	3.87716E-07	0.476
118	FBgn0051183	chr3R	-	15875572	15875939	15875577	15875939	15869172	15870232	0	-0.686
119	FBgn0028496	chr2R	-	18497704	18498026	18497885	18498026	18496746	18496905	0.013796306	0.508
120	FBgn0000117	chrX	-	1899356	1899524	1899398	1899524	1898979	1899039	0.000730026	-0.673
121	FBgn0036044	chr3L	-	9790405	9790671	9790455	9790671	9789215	9789271	0.001065988	0.689
122	FBgn0016726	chr2R	+	21293767	21293878	21293767	21293789	21294053	21294161	0.026178183	0.447
123	FBgn0260943	chr3L	-	17237831	17238212	17237885	17238212	17235351	17236062	0.020069579	0.255
124	FBgn0260943	chr3L	-	17237831	17238212	17238094	17238212	17235351	17236062	3.03652E-05	0.486
125	FBgn0010415	chr2R	-	21481014	21481176	21481093	21481176	21480090	21480356	0.019079806	0.333
126	FBgn0013576	chr3R	-	5281015	5281102	5281062	5281102	5274965	5275087	0.021019831	-0.599
127	FBgn0263077	chr2R	+	7638052	7638372	7638052	7638180	7638597	7638905	6.84111E-07	-0.229
128	FBgn0019936	chr3R	+	20822417	20822859	20822417	20822573	20823131	20823356	6.06417E-06	-0.494
129	FBgn0016131	chr2R	+	16579469	16579755	16579469	16579533	16580841	16581015	0.045651361	-0.165
130	FBgn0034031	chr2R	+	15557243	15557611	15557243	15557310	15561814	15562436	6.24703E-05	-0.338
131	FBgn0004885	chr3R	+	24720056	24720220	24720056	24720216	24724942	24725362	0.02043507	-0.339
132	FBgn0031717	chr2L	+	5547289	5547756	5547289	5547728	5547807	5547912	0	0.833
133	FBgn0050015	chr2R	-	10853398	10854150	10853969	10854150	10848593	10849260	0.011289468	-0.349
134	FBgn0031170	chrX	-	21306094	21306665	21306648	21306665	21305529	21305743	0.000100148	0.702
135	FBgn0031170	chrX	-	21306568	21306665	21306648	21306665	21305529	21305743	1.76726E-06	0.809
136	FBgn0052121	chr3L	-	13421947	13422336	13422136	13422336	13419980	13420125	0.04256792	-0.49
137	FBgn0035049	chr2R	-	24684950	24685090	24684958	24685090	24682213	24682441	0.001165068	0.611
138	FBgn0002521	chr4	-	1174734	1174928	1174758	1174928	1173083	1173203	0.019880312	0.604
139	FBgn0038294	chr3R	-	15173980	15174343	15174136	15174343	15172599	15173062	2.28541E-06	0.273
140	FBgn0038294	chr3R	-	15173980	15174343	15174137	15174343	15172843	15173063	2.78832E-06	0.7

141	FBgn0010263	chr2L	+	2960724	2961150	2960724	2960991	2961214	2961672	0.043019204	0.417
142	FBgn0033403	chr2R	-	9351408	9351655	9351440	9351655	9350671	9351173	0.001361107	-0.586
143	FBgn0038421	chr3R	-	16358861	16359318	16359130	16359318	16358682	16358788	9.74148E-11	-1
144	FBgn0020496	chr3R	+	13011648	13012172	13011648	13012019	13019341	13019814	0.045512969	0.38
145	FBgn0000578	chr2R	+	19141631	19141900	19141631	19141824	19142206	19142354	0.034396769	0.584
146	FBgn0039900	chr4	-	305636	305818	305742	305818	305015	305178	0.007512055	0.667
147	FBgn0051992	chr4	-	659644	659983	659789	659983	657901	658508	0.031649888	0.273
148	FBgn0051992	chr4	-	659667	659983	659789	659983	657901	658508	0.014254653	0.351
149	FBgn0051992	chr4	-	659789	659983	659920	659983	657901	658508	0.048407812	-0.271
150	FBgn0040608	chr3R	+	26727313	26727649	26727313	26727379	26729766	26730117	0.00118746	0.337
151	FBgn0031914	chr2L	+	7440793	7441021	7440793	7440958	7443583	7443832	0	-0.537
152	FBgn0031914	chr2L	+	7440793	7441021	7440793	7440962	7443583	7443832	0	-0.389
153	FBgn0011640	chr3L	+	7339463	7339905	7339463	7339617	7339961	7340770	0	-0.851
154	FBgn0011640	chr3L	+	7339728	7339908	7339728	7339905	7339961	7340770	0.018116411	0.413
155	FBgn0261649	chr3R	+	17821893	17821958	17821893	17821938	17836150	17836236	0.006837225	-0.368
156	FBgn0013718	chr3L	+	14221439	14221503	14221439	14221500	14225430	14226403	0.000331619	0.317
157	FBgn0058191	chr2R	+	1265154	1265531	1265154	1265293	1266191	1266349	0.037377569	0.273
158	FBgn0036053	chr3L	-	9863776	9864099	9864006	9864099	9863320	9863616	0.005959505	0.436
159	FBgn0085390	chr2R	-	7845027	7845199	7845029	7845199	7844885	7844972	0.005511092	0.667
160	FBgn0086768	chr3R	-	5553826	5554316	5554128	5554316	5553270	5553675	1.48371E-05	0.544
161	FBgn0250819	chr4	+	1185767	1185966	1185767	1185834	1186177	1186219	0.010132091	-0.257
162	FBgn0250910	chr3R	-	12547286	12547628	12547290	12547628	12521797	12522659	1.34123E-10	-0.883
163	FBgn0259680	chrX	+	12432223	12432560	12432223	12432384	12433138	12433339	0.007382093	-0.762
164	FBgn0261283	chr3L	+	19793934	19794298	19793934	19794153	19794901	19796392	0.008591353	0.618
165	FBgn0261283	chr3L	+	19793934	19794396	19793934	19794153	19794901	19796392	0.016921113	0.608
166	FBgn0261534	chr2L	-	14350062	14350398	14350230	14350398	14349708	14349782	3.03448E-08	0.976
167	FBgn0016694	chr3L	-	7832553	7833073	7832863	7833073	7830386	7831810	1.24916E-05	0.522
168	FBgn0016694	chr3L	-	7832855	7833073	7832921	7833073	7830386	7831810	0.037377569	-0.503
169	FBgn0016694	chr3L	-	7832861	7833073	7832863	7833073	7830386	7831810	9.25567E-06	0.735
170	FBgn0016694	chr3L	-	7832553	7833073	7832863	7833073	7830386	7831827	0.000361195	0.435

MXE

	FlyBase ID	chr	strand	1stExonStart_0base	1stExonEnd	ExonStart_0b	2ndExonEnd	upstreamES	upstreamEE	downstreamES	downstreamEE	FDR	ΔPSI
1	FBgn0263456	chr3L	+	9141786	9141843	9143297	9143323	9140850	9141508	9143526	9144294	0	0.288
2	FBgn0263346	chr3R	+	4670796	4670850	4680778	4680837	4659578	4660399	4697190	4697542	0	-0.421
3	FBgn0016920	chr2L	+	5361997	5362209	5362582	5362812	5360791	5360878	5363655	5363784	0.02223	0.444
4	FBgn0051973	chr2L	-	30393	33270	33844	34288	28981	29068	34557	34604	1.8E-05	-0.176
5	FBgn0039972	chr2L	+	22325761	22325846	22354811	22354969	22311926	22312038	22363429	22364294	0.03786	0.365
6	FBgn0033159	chr2R	-	7349392	7349513	7357182	7357306	7348130	7348975	7361717	7361867	0.00139	-0.501
7	FBgn0033159	chr2R	-	7349392	7349513	7357788	7357915	7348130	7348975	7361717	7361867	2.9E-07	-0.778
8	FBgn0033159	chr2R	-	7349392	7349513	7358002	7358129	7348130	7348975	7361717	7361867	0.00035	-0.723
9	FBgn0033159	chr2R	-	7349392	7349513	7360290	7360414	7348130	7348975	7361717	7361867	0.00012	-0.759
10	FBgn0033159	chr2R	-	7350580	7350704	7357182	7357306	7348130	7348975	7361717	7361867	0.00996	-0.333
11	FBgn0033159	chr2R	-	7351698	7351822	7357182	7357306	7348130	7348975	7361717	7361867	0.01058	-0.333
12	FBgn0033159	chr2R	-	7351915	7352039	7357182	7357306	7348130	7348975	7361717	7361867	0.0048	-0.333
13	FBgn0033159	chr2R	-	7353815	7353936	7357182	7357306	7348130	7348975	7361717	7361867	0.00139	-0.501
14	FBgn0033159	chr2R	-	7353815	7353936	7357788	7357915	7348130	7348975	7361717	7361867	8.9E-07	-0.805
15	FBgn0033159	chr2R	-	7353815	7353936	7358002	7358129	7348130	7348975	7361717	7361867	0.00268	-0.723
16	FBgn0033159	chr2R	-	7353815	7353936	7360290	7360414	7348130	7348975	7361717	7361867	0.00199	-0.704
17	FBgn0033159	chr2R	-	7354276	7354400	7357788	7357915	7348130	7348975	7361717	7361867	0.00799	-0.646
18	FBgn0033159	chr2R	-	7354276	7354400	7358002	7358129	7348130	7348975	7361717	7361867	0.00173	-0.624
19	FBgn0033159	chr2R	-	7354276	7354400	7360290	7360414	7348130	7348975	7361717	7361867	0.00046	-0.625
20	FBgn0033159	chr2R	-	7354881	7355005	7357788	7357915	7348130	7348975	7361717	7361867	0.00457	-0.667
21	FBgn0033159	chr2R	-	7355086	7355210	7358002	7358129	7348130	7348975	7361717	7361867	0.01694	-0.667
22	FBgn0033159	chr2R	-	7355290	7355414	7358002	7358129	7348130	7348975	7361717	7361867	0.03033	-0.51
23	FBgn0033159	chr2R	-	7355290	7355414	7360290	7360414	7348130	7348975	7361717	7361867	0.03833	-0.437
24	FBgn0033159	chr2R	-	7356526	7356650	7357788	7357915	7348130	7348975	7361717	7361867	0.00799	-0.667
25	FBgn0033159	chr2R	-	7357590	7357714	7358002	7358129	7348130	7348975	7361717	7361867	0.03634	-0.453
26	FBgn0033159	chr2R	-	7357590	7357714	7360290	7360414	7348130	7348975	7361717	7361867	0.00996	-0.508
27	FBgn0033159	chr2R	-	7357788	7357915	7358603	7358727	7348130	7348975	7361717	7361867	0.01077	0.555
28	FBgn0033159	chr2R	-	7357788	7357915	7360525	7360649	7348130	7348975	7361717	7361867	0.01094	0.63
29	FBgn0033159	chr2R	-	7358002	7358129	7360525	7360649	7348130	7348975	7361717	7361867	0.00996	0.583
30	FBgn0033159	chr2R	-	7360290	7360414	7360525	7360649	7348130	7348975	7361717	7361867	0.00139	0.676
31	FBgn0038294	chr3R	-	15171935	15172106	15172599	15172830	15170624	15171374	15174136	15174343	8.9E-07	-0.099
32	FBgn0261788	chr3L	-	7656374	7661506	7662470	7662890	7655388	7656203	7663258	7663324	0.0005	0.187
33	FBgn0035438	chr3L	+	3326522	3326712	3327351	3327553	3326264	3326367	3327859	3328183	0.02823	-0.131
34	FBgn0035438	chr3L	+	3326522	3326712	3327363	3327553	3326264	3326367	3327859	3328183	0.02823	-0.13
35	FBgn0035438	chr3L	+	3326586	3326712	3327351	3327553	3326264	3326367	3327859	3328183	0.04586	-0.125
36	FBgn0035438	chr3L	+	3326586	3326712	3327363	3327553	3326264	3326367	3327859	3328183	0.04586	-0.125
37	FBgn0263980	chr2R	+	15425581	15425666	15447794	15448050	15424258	15424405	15452856	15453008	9.3E-08	-0.527
38	FBgn0263980	chr2R	+	15425581	15425666	15447831	15448050	15424258	15424405	15452856	15453008	6.4E-09	-0.589
39	FBgn0263980	chr2R	+	15425581	15425666	15447838	15448050	15424258	15424405	15452856	15453008	9E-08	-0.536
40	FBgn0263392	chr3L	-	2811680	2811879	2816829	2817087	2796291	2796678	2820058	2821103	0.02032	-0.667
41	FBgn0263346	chr3R	+	4660235	4660399	4680682	4680837	4659578	4659676	4697190	4697542	2.9E-11	-0.369
42	FBgn0263346	chr3R	+	4660238	4660399	4680682	4680837	4659578	4659676	4697190	4697542	6.2E-05	-0.321
43	FBgn0263346	chr3R	+	4670796	4670845	4680778	4680837	4659578	4660399	4697190	4697542	2E-05	-0.284
44	FBgn0263346	chr3R	+	4670808	4670850	4680778	4680837	4660238	4660399	4697190	4697542	0	-0.58
45	FBgn0263346	chr3R	+	4670808	4670850	4680778	4681199	4660238	4660399	4697190	4697542	0	-0.963
46	FBgn0264489	chr3L	+	9828078	9828209	9829432	9829896	9824820	9827895	9830633	9830785	0.01822	-0.33
47	FBgn0259916	chr3L	-	8158198	8158283	8158588	8158679	8154752	8155579	8158756	8158900	0.04422	0.38
48	FBgn0262617	chr3R	+	10299034	10299058	10299413	10299494	10298448	10298591	10299633	10301527	0.01236	-0.425
49	FBgn0032946	chr2L	+	21380679	21380728	21380918	21381199	21380132	21380361	21382018	21382330	0.03833	-0.505
50	FBgn0265959	chr3L	+	20258763	20258906	20260206	20260310	20255897	20256638	20260861	20260989	0.01114	0.582
51	FBgn0265959	chr3L	+	20258763	20258906	20260209	20260310	20255897	20256638	20260861	20260989	1.1E-05	0.627
52	FBgn0261625	chr2R	-	12673610	12673925	12674231	12674702	12673099	12673339	12674990	12675418	0.00832	-0.533
53	FBgn0261625	chr2R	-	12673610	12673928	12674231	12674702	12673099	12673339	12674990	12675418	0.04586	-0.595
54	FBgn0261625	chr2R	-	12673898	12673928	12674205	12674702	12673099	12673339	12674990	12675418	0.00183	-0.226
55	FBgn0283471	chrX	-	18113729	18113916	18114443	18114532	18113446	18113569	18114605	18114646	0.01149	-0.353
56	FBgn0263772	chrX	+	4076544	4076790	4077874	4079131	4075977	4076426	4082793	4084345	0.01068	-0.459
57	FBgn0033807	chr2R	-	13213532	13213558	13213750	13213931	13213022	13213436	13214418	13214496	0	0.139
58	FBgn0033807	chr2R	-	13213532	13213558	13213750	13213949	13213022	13213436	13214418	13214496	0	0.201
59	FBgn0033807	chr2R	-	13213532	13213558	13213751	13213931	13213022	13213436	13214418	13214496	0	0.296
60	FBgn0051973	chr2L	-	30393	30673	33844	34169	28981	29068	34557	34604	5.1E-06	-0.213
61	FBgn0051973	chr2L	-	30393	30673	33844	34283	28981	29068	34557	34604	4.6E-06	-0.213
62	FBgn0051973	chr2L	-	30393	30673	33844	34288	28981	29068	34557	34604	4.8E-05	-0.165
63	FBgn0051973	chr2L	-	30393	30673	33844	34288	28981	29068	38534	38731	2.7E-06	-0.214
64	FBgn0051973	chr2L	-	30393	30673	34557	34904	28981	29068	38534	38731	0.00104	-0.185
65	FBgn0051973	chr2L	-	30393	33270	33844	34169	28981	29068	34557	34604	2.2E-06	-0.205
66	FBgn0051973	chr2L	-	30393	33270	33844	34283	28981	29068	34557	34604	2E-06	-0.206
67	FBgn0004169	chrX	-	13600579	13600949	13601872	13602011	13600069	13600117	13602417	13602578	0.03655	-0.525
68	FBgn0261822	chr2L	+	8109296	8109349	8109815	8110216	8107747	8107880	8110480	8116531	0.03279	0.406
69	FBgn0025595	chr2L	-	6715168	6715323	6715783	6715955	6714554	6714767	6716025	6716186	0.01068	0.333
70	FBgn0025595	chr2L	-	6715168	6715323	6715783	6715969	6714554	6714767	6716025	6716186	0.01312	0.313
71	FBgn0039972	chr2L	+	22325467	22325846	22354811	22354969	22311926	22312038	22363429	22364294	0.03601	0.442
72	FBgn0011207	chr2L	+	9706980	9707354	9707672	9708059	9706639	9706746	9708144	9708883	0.01149	0.75
73	FBgn0263120	chr2R	+	8677064	8677270	8677965	8678186	8676471	8676607	8678429	8678868	0.03634	-0.306
74	FBgn0016059	chr2R	-	17679143	17679253	17683075	17683287	17678271	17678782	17683392	17683499	0.04586	-0.566
75	FBgn0029996	chrX	-	8090863	8091069	8092434	8092506	8088008	8090147	8093774	8094064	2.1E-09	0.824
76	FBgn0263077	chr2R	+	7637283	7637408	7638113	7638180	7636894	7637193	7638597	7638905	2.1E-05	-0.478
77	FBgn0263077	chr2R	+	7637283	7637408	7638122	7638180	7636894	7637193	7638597	7638905	0.0005	-0.436
78	FBgn0263077	chr2R	+	7637283	7637408	7638122	7638252	7636894	7637193	7638597	7638905	0.00155	-0.317
79	FBgn0263077	chr2R	+	7637283	7637541	7638113	7638180	7636894	7637193	7638597	7638905	4.9E-06	-0.486
80	FBgn0263077	chr2R	+	7637283	7637541	7638122	7638180	7636894	7637193	7638597	7638905	0.00027	-0.438
81	FBgn0263077	chr2R	+	7637283	7637541	7638122	7638252	7636894	7637193	7638597	7638905	0.00219	-0.296
82	FBgn0265356	chr2R	+	19002454	19002780	19004613	19004889	19001091	19001789	19005788	19006571	8.4E-06	-0.523

83	FBgn0053087	chr2R	+	8186279	8186434	8186904	8186982	8174164	8174831	8206626	8206740	0.03634	-0.387
84	FBgn0039890	chr4	-	190002	190061	191301	191386	188577	189248	193544	193829	0.02915	0.214
85	FBgn0038294	chr3R	-	15171935	15172106	15172599	15172796	15170624	15171374	15174136	15174343	2.5E-12	-0.144
86	FBgn0038294	chr3R	-	15171935	15172106	15172599	15173062	15170624	15171374	15174136	15174343	0	-0.15
87	FBgn0020496	chr3R	+	13014489	13014589	13018596	13018876	13011648	13011748	13019341	13019814	0.03091	-0.491
88	FBgn0016930	chr2L	-	14229458	14229536	14233647	14233777	14222016	14222081	14233893	14234108	2E-06	-0.259
89	FBgn0010488	chr2R	+	12916150	12916331	12917742	12917904	12915993	12916064	12917994	12920859	0.00289	0.218
90	FBgn0086711	chr2L	-	14994108	14994285	14996728	14996889	14993898	14994035	14997515	14997559	0.01151	0.597
91	FBgn0004598	chrX	+	16373657	16373834	16374540	16374760	16373085	16373187	16374925	16375859	0.00859	-0.381
92	FBgn0261552	chr3R	+	9436933	9437118	9439266	9439705	9432601	9432839	9440680	9441665	0.0017	0.185
93	FBgn0261552	chr3R	+	9436933	9437118	9439294	9439705	9432601	9432839	9440680	9441665	0.00139	0.185
94	FBgn0261552	chr3R	+	9436933	9437118	9439320	9439705	9432601	9432839	9440680	9441665	0.0017	0.185
95	FBgn0261552	chr3R	+	9436933	9437118	9439420	9439705	9432601	9432839	9440680	9441665	0.00139	0.185
96	FBgn0261788	chr3L	-	7656374	7661506	7662704	7662890	7655388	7656203	7663258	7663324	1E-07	0.223

RI											
	FlyBase ID	chr	strand	riExonStart_0base	riExonEnd	upstreamES	upstreamEE	downstreamES	downstreamEE	FDR	ΔPSI
1	FBgn0285963	chr3R	+	26915760	26916562	26915760	26915908	26916502	26916562	0	-0.945
2	FBgn0267376	chr3R	-	10866808	10868290	10866808	10866960	10867971	10868290	0	0.823
3	FBgn0267376	chr3R	-	10866808	10868642	10866808	10866960	10868438	10868642	0	0.796
4	FBgn0267337	chr3R	-	7307856	7309629	7307856	7308475	7309029	7309629	0.030835973	0.493
5	FBgn0266681	chr3L	-	10893645	10894743	10893645	10894625	10894699	10894743	0.027281563	0.311
6	FBgn0266579	chr3R	-	27641841	27643973	27641841	27642057	27643814	27643973	0.034877196	-0.29
7	FBgn0266579	chr3R	-	27643814	27644422	27643814	27643973	27644404	27644422	0.008406155	0.223
8	FBgn0266579	chr3R	-	27646335	27646698	27646335	27646525	27646596	27646698	1.5429E-12	0.673
9	FBgn0266178	chr3R	-	21358004	21359768	21358004	21359069	21359362	21359768	0.013229792	0.299
10	FBgn0265363	chr2R	-	11844377	11845099	11844377	11844672	11844818	11845099	4.82461E-10	0.514
11	FBgn0264915	chr3R	+	20911639	20912345	20911639	20911709	20911770	20912345	1.14776E-10	-0.371
12	FBgn0264789	chr3R	+	5250912	5252132	5250912	5251181	5251932	5252132	0.028354859	-0.3
13	FBgn0264087	chr2L	-	7655999	7656742	7655999	7656463	7656678	7656742	0.006737196	-0.436
14	FBgn0264087	chr2L	-	7657184	7659567	7657184	7657235	7659191	7659567	0.00855413	-0.377
15	FBgn0266136	chr3L	-	19789963	19793569	19789963	19790254	19793427	19793569	0.001412785	0.391
16	FBgn0263456	chr3L	+	9141786	9143323	9141786	9141843	9143297	9143323	0.009500484	0.19
17	FBgn0263219	chr3L	+	8262953	8265984	8262953	8263119	8265893	8265984	2.22867E-09	0.266
18	FBgn0263219	chr3L	+	8265893	8266434	8265893	8265984	8266346	8266434	2.9962E-06	0.255
19	FBgn0263197	chr2R	-	17531210	17531653	17531210	17531446	17531517	17531653	0.038501832	-0.416
20	FBgn0263111	chrX	-	11956921	11960080	11956921	11956924	11960074	11960080	0.024489066	0.704
21	FBgn0263336	chr2R	-	16817518	16818694	16817518	16818531	16818589	16818694	0.021720918	-0.372
22	FBgn0262985	chr3L	+	6185329	6185853	6185329	6185630	6185703	6185853	0.000257649	-0.578
23	FBgn0262975	chr3R	-	23222796	23226711	23222796	23223120	23226453	23226711	0.037896435	0.408
24	FBgn0262614	chr3R	-	8865404	8867435	8865404	8866025	8867271	8867435	0.000715341	-0.592
25	FBgn0262593	chr3L	+	2895119	2897429	2895119	2895284	2897243	2897429	1.81291E-13	-0.668
26	FBgn0262593	chr3L	+	2896742	2897429	2896742	2896787	2897243	2897429	0	-0.88
27	FBgn0266084	chr3L	+	8796999	8797821	8796999	8797226	8797689	8797821	0.042713119	0.088
28	FBgn0266084	chr3L	+	8796999	8798719	8796999	8797821	8798120	8798719	8.74464E-08	0.118
29	FBgn0263776	chr3L	-	11039997	11050440	11039997	11040068	11050291	11050440	4.50541E-06	0.35
30	FBgn0261561	chr3R	+	5816991	5818067	5816991	5817279	5817515	5818067	0.013493456	-0.42
31	FBgn0259916	chr3L	-	8154752	8158679	8154752	8155579	8158588	8158679	0.00264426	-0.601
32	FBgn0259736	chr3R	+	22120060	22120971	22120060	22120462	22120846	22120971	0.000440718	0.846
33	FBgn0259146	chr3R	+	25970079	25970936	25970079	25970291	25970840	25970936	0.000775174	-0.483
34	FBgn0250910	chr3R	-	12547286	12548229	12547286	12547704	12547910	12548229	0.001158448	0.702
35	FBgn0250906	chr2L	-	2748319	2749313	2748319	2748792	2749211	2749313	0.006131416	-0.734
36	FBgn0086711	chr2L	-	14993898	14997559	14993898	14994035	14997515	14997559	3.71067E-06	-0.524
37	FBgn0086681	chr2L	-	18675182	18675957	18675182	18675508	18675565	18675957	3.53739E-13	0.399
38	FBgn0086442	chr2L	+	19035499	19035865	19035499	19035697	19035760	19035865	7.90688E-05	0.51
39	FBgn0085421	chr2R	-	6769307	6769758	6769307	6769359	6769431	6769758	8.14219E-05	-0.726
40	FBgn0085390	chr2R	-	7850926	7852346	7850926	7851052	7852265	7852346	0.000879419	-0.608
41	FBgn0083984	chr3R	-	21877869	21878419	21877869	21878077	21878232	21878419	7.0978E-05	0.206
42	FBgn0260479	chr2L	-	7736337	7738809	7736337	7736989	7738618	7738809	2.86772E-06	0.351
43	FBgn0082831	chr3R	+	12414351	12416582	12414351	12414756	12414870	12416582	1.81291E-13	-0.277
44	FBgn0053978	chr4	-	158643	159712	158643	158662	159221	159712	0.003086733	0.529
45	FBgn0262969	chr3L	+	18441006	18441871	18441006	18441178	18441600	18441871	0.007226821	0.491
46	FBgn0053203	chr3R	-	28872151	28872657	28872151	28872292	28872519	28872657	0.000550597	-0.422
47	FBgn0053093	chr3R	-	22530693	22531013	22530693	22530807	22530870	22531013	4.38069E-08	0.284
48	FBgn0053054	chr3L	-	21197759	21202882	21197759	21198103	21202747	21202882	0.007949375	-0.167
49	FBgn0259740	chr3R	+	10851284	10851788	10851284	10851488	10851540	10851788	0.003268869	-0.307
50	FBgn0052448	chr3L	-	21828070	21828480	21828070	21828326	21828451	21828480	0.001879337	0.648
51	FBgn0052320	chr3L	+	1398585	1399416	1398585	1399055	1399105	1399416	0.005682238	0.521
52	FBgn0052284	chr3L	+	3186663	3186899	3186663	3186697	3186759	3186899	0.015111786	-0.162
53	FBgn0052264	chr3L	-	3723638	3743772	3723638	3723872	3741995	3743772	0.009844823	-0.114
54	FBgn0052264	chr3L	-	3741995	3746584	3741995	3743772	3745827	3746584	0.000976747	-0.415
55	FBgn0052264	chr3L	-	3723638	3759156	3723638	3723872	3758406	3759156	2.45732E-05	-0.165
56	FBgn0052264	chr3L	-	3769241	3781412	3769241	3769406	3780422	3781412	0.039069908	-0.098
57	FBgn0052264	chr3L	-	3769241	3803454	3769241	3769406	3803313	3803454	0.001412785	-0.257
58	FBgn0052264	chr3L	-	3803313	3806459	3803313	3803454	3805820	3806459	0.015111786	-0.352
59	FBgn0052205	chr3L	-	19423378	19424183	19423378	19423567	19423773	19424183	0.019569851	0.736
60	FBgn0264839	chr3R	+	20827094	20828212	20827094	20827338	20827925	20828212	0.000495192	-0.657
61	FBgn0264827	chr3R	-	9810019	9813929	9810019	9810906	9813214	9813929	6.9389E-05	0.712
62	FBgn0051324	chr3R	+	26106867	26124657	26106867	26107342	26124434	26124657	2.03425E-05	-0.546
63	FBgn0051224	chr3R	-	18656164	18659309	18656164	18657185	18658759	18659309	3.86257E-05	-0.361
64	FBgn0051224	chr3R	-	18656164	18659309	18656164	18657185	18659253	18659309	2.45732E-05	-0.254
65	FBgn0051189	chr3R	-	21045200	21045842	21045200	21045684	21045741	21045842	0.0009447	0.134
66	FBgn0050286	chr2R	-	21535715	21536527	21535715	21536423	21536481	21536527	0.000242398	0.685
67	FBgn0050286	chr2R	-	21536481	21536795	21536481	21536527	21536582	21536795	6.71731E-11	0.844
68	FBgn0052685	chrX	-	10074975	10075627	10074975	10075073	10075143	10075627	0.003161995	-0.24
69	FBgn0050082	chr2R	-	15685519	15685806	15685519	15685701	15685760	15685806	0.031826231	-0.53
70	FBgn0050021	chr2R	-	11229003	11229542	11229003	11229033	11229410	11229542	4.10431E-08	-0.388
71	FBgn0260388	chr3L	-	16495299	16496236	16495299	16495536	16496052	16496236	1.4188E-12	-0.885
72	FBgn0003867	chr3R	-	21779551	21782536	21779551	21779783	21781987	21782536	0.001090989	0.415
73	FBgn0265959	chr3L	+	20248627	20256638	20248627	20249060	20255897	20256638	0	0.852
74	FBgn0265959	chr3L	+	20259985	20260989	20259985	20260310	20260861	20260989	8.98972E-09	0.439
75	FBgn0032603	chr2L	+	16791567	16791869	16791567	16791707	16791763	16791869	0	0.34
76	FBgn0031914	chr2L	+	7440793	7443832	7440793	7440851	7443583	7443832	0.020070511	-0.231
77	FBgn0031914	chr2L	+	7440793	7443832	7440793	7440958	7443583	7443832	0	-0.682
78	FBgn0031914	chr2L	+	7440793	7443832	7440793	7440962	7443583	7443832	0	-0.547
79	FBgn0032717	chr2L	-	18838508	18839455	18838508	18838792	18838882	18839455	0.003161995	-0.536
80	FBgn0031238	chr2L	-	253955	254750	253955	254397	254561	254750	0.005274691	0.432
81	FBgn0013718	chr3L	+	14231176	14232500	14231176	14231325	14231926	14232500	0.000242398	-0.261
82	FBgn0030882	chrX	+	17891587	17892904	17891587	17891934	17892451	17892904	0.012489324	-0.607
83	FBgn0030854	chrX	-	17634573	17636044	17634573	17634974	17635341	17636044	0.011140469	0.575
84	FBgn0266410	chr3R	+	9341866	9342894	9341866	9342491	9342575	9342894	0.003161995	-0.6
85	FBgn0040694	chr3L	-	3620741	3621032	3620741	3620809	3620871	3621032	0	-0.645
86	FBgn0037060	chr3L	+	21213128	21214097	21213128	21213491	21213843	21214097	0.001381868	-0.306
87	FBgn0030089	chrX	+	9109549	9111005	9109549	9109987	9110821	9111005	0.018082601	0.218
88	FBgn0039602	chr3R	+	28820220	28820718	28820220	28820505	28820571	28820718	0.004028069	-0.565
89	FBgn0029843	chrX	+	6091632	6096653	6091632	6092215	6096051	6096653	0.021786604	0.5
90	FBgn0031005	chrX	+	19250753	19252045	19250753	19251274	19251450	19252045	0.001426015	0.484
91	FBgn0039831	chr3R	+	31224916	31226076	31224916	31225461	31225812	31226076	0.031417592	-0.43
92	FBgn0005674	chr3R	-	23953445	23955499	23953445	23955305	23955451	23955499	0.00752844	-0.667
93	FBgn0039009	chr3R	-	22704253	22705017	22704253	22704487	22704542	22705017	0.013493456	-0.366
94	FBgn0039009	chr3R	-	22704542	22705311	22704542	22705017	22705069	22705311	0.0374798	-0.264
95	FBgn0039507	chr3R	-	27279177	27282661	27279177	27280042	27282616	27282661	0.012172466	0.323
96	FBgn0039308	chr3R	+	25270208	25271583	25270208	25271287	25271345	25271583	1.04743E-07	-0.388

97	FBgn0263117	chr3R	-	22314659	22315172	22314659	22314766	22314994	22315172	7.36002E-06	-0.709
98	FBgn0264489	chr3L	+	9813846	9820559	9813846	9813922	9820073	9820559	0.000472957	-0.606
99	FBgn0264489	chr3L	+	9824545	9827895	9824545	9824640	9824802	9827895	0.03092574	0.364
100	FBgn0264489	chr3L	+	9830999	9832679	9830999	9831440	9831920	9832679	0	-0.401
101	FBgn0264489	chr3L	+	9830633	9832679	9830633	9830785	9832195	9832679	1.51076E-10	0.296
102	FBgn0264489	chr3L	+	9830999	9832679	9830999	9831440	9832195	9832679	0	0.443
103	FBgn0264489	chr3L	+	9830633	9832679	9830633	9830785	9832198	9832679	0	-0.245
104	FBgn0285913	chr3R	+	14298460	14298987	14298460	14298669	14298789	14298987	2.71937E-12	-0.299
105	FBgn0028699	chr3L	+	4918758	4919399	4918758	4918861	4919100	4919399	0.002855814	0.245
106	FBgn0033194	chr2R	+	7566808	7567364	7566808	7566999	7567145	7567364	0.039069908	0.335
107	FBgn0261788	chr3L	-	7655388	7662890	7655388	7656203	7662470	7662890	0.024034912	0.155
108	FBgn0261788	chr3L	-	7655388	7663324	7655388	7656203	7663258	7663324	1.20986E-05	0.736
109	FBgn0027083	chr3R	-	14252630	14258895	14252630	14252828	14258630	14258895	4.64105E-12	0.33
110	FBgn0039356	chr3R	+	25678682	25679158	25678682	25678899	25678972	25679158	0.000157532	-0.319
111	FBgn0026083	chrX	+	4119713	4120424	4119713	4120100	4120295	4120424	0.000245498	0.671
112	FBgn0026083	chrX	+	4120097	4120424	4120097	4120138	4120295	4120424	0.041831701	0.756
113	FBgn0023177	chrX	-	20422058	20423796	20422058	20422976	20423645	20423796	0.009226871	-0.453
114	FBgn0001624	chrX	+	11381855	11383112	11381855	11381980	11382935	11383112	0.019526451	-0.237
115	FBgn0037222	chr3R	-	4241199	4241919	4241199	4241698	4241754	4241919	0.006627728	0.265
116	FBgn0036801	chr3L	+	18625958	18627031	18625958	18626076	18626864	18627031	0.011534051	-0.247
117	FBgn0013812	chr3R	-	21032221	21033477	21032221	21033045	21033124	21033477	0.000550597	0.331
118	FBgn0032780	chr2L	+	19426297	19430270	19426297	19426698	19429401	19430270	0.016731791	0.659
119	FBgn0260794	chr3R	-	4801212	4806463	4801212	4801991	4806256	4806463	0.002729833	-0.608
120	FBgn0052062	chr3L	+	10580308	10582118	10580308	10580329	10581841	10582118	1.0053E-06	-0.241
121	FBgn0032666	chr2L	+	18122527	18123908	18122527	18122588	18123348	18123908	0.011548008	-0.286
122	FBgn0004838	chr2L	+	6920703	6924400	6920703	6920861	6923783	6924400	0.039458478	-0.467
123	FBgn0265935	chr2R	-	6865107	6865884	6865107	6865374	6865866	6865884	6.59592E-08	-0.594
124	FBgn0023095	chr3L	+	13251153	13279642	13251153	13251211	13275561	13279642	1.74084E-09	-0.889
125	FBgn0035868	chr3L	-	8302363	8304059	8302363	8303490	8303821	8304059	0.00013022	-0.222
126	FBgn0013305	chr2R	-	12879196	12879656	12879196	12879222	12879447	12879656	9.63213E-05	-0.307
127	FBgn0003371	chrX	+	2649735	2670103	2649735	2649739	2669912	2670103	0.013172819	-0.63
128	FBgn0032024	chr2L	+	8342694	8343841	8342694	8342989	8343382	8343841	2.30547E-05	-0.775
129	FBgn0263446	chr2R	+	12873247	12875087	12873247	12874720	12874794	12875087	0.00023291	-0.866
130	FBgn0266702	chr3R	-	5835477	5836178	5835477	5835911	5835990	5836178	0.039584498	0.442
131	FBgn0266702	chr3R	-	5835477	5836178	5835477	5835938	5835990	5836178	0.028354859	0.43
132	FBgn0263448	chr3R	-	15230835	15231503	15230835	15231118	15231173	15231503	6.5924E-13	-0.829
133	FBgn0038874	chr3R	+	21259706	21265134	21259706	21259887	21264019	21265134	0.00264426	-0.42
134	FBgn0038846	chr3R	+	21015948	21016427	21015948	21016008	21016219	21016427	1.20621E-06	-0.571
135	FBgn0038846	chr3R	+	21015948	21016427	21015948	21016107	21016219	21016427	0.002364925	-0.656
136	FBgn0285917	chr2R	-	18289534	18306135	18289534	18289850	18306030	18306135	0.00110852	-0.432
137	FBgn0035432	chr3L	+	3303707	3310706	3303707	3303852	3310519	3310706	2.45732E-05	-0.49
138	FBgn0000228	chr2L	-	5276935	5278548	5276935	5277180	5277957	5278548	1.04882E-06	-0.428
139	FBgn0037993	chr3R	-	12156108	12158595	12156108	12156621	12158533	12158595	0.01203036	0.667
140	FBgn0053200	chr2R	+	22408524	22409198	22408524	22408723	22408782	22409198	0.000852987	-0.736
141	FBgn0031857	chr2L	-	6770590	6772614	6770590	6770709	6772551	6772614	0	-0.869
142	FBgn0051477	chr3R	+	10121772	10121946	10121772	10121805	10121871	10121946	4.92567E-08	-0.568
143	FBgn0023097	chr3R	+	20610386	20612540	20610386	20610510	20610575	20612540	0.020070511	-0.526
144	FBgn0003429	chr3R	+	24662490	24664795	24662490	24662563	24664363	24664795	0.037403342	0.525
145	FBgn0003429	chr3R	+	24664896	24669879	24664896	24665085	24669540	24669879	0.000245498	0.725
146	FBgn0003429	chr3R	+	24696090	24703307	24696090	24696126	24703117	24703307	0.000898801	0.454
147	FBgn0025726	chr4	-	892893	893565	892893	892949	893504	893565	0.007878152	-0.502
148	FBgn0011206	chr3L	-	9112161	9112787	9112161	9112368	9112633	9112787	0.004527691	0.625
149	FBgn0020912	chr3R	+	30915939	30923558	30915939	30916721	30923402	30923558	0.00076412	-0.654
150	FBgn0038917	chr3R	+	21888838	21907182	21888838	21889267	21905927	21907182	0.012091532	-0.645
151	FBgn0003091	chr2R	+	16924924	16926807	16924924	16924977	16926345	16926807	4.59078E-06	0.627
152	FBgn0026418	chr3L	+	14037018	14037750	14037018	14037111	14037477	14037750	1.43558E-10	0.539
153	FBgn0026418	chr3L	+	14037018	14037750	14037018	14037136	14037477	14037750	3.44619E-09	0.548
154	FBgn0004865	chr3L	+	21240346	21263636	21240346	21240803	21262995	21263636	1.10776E-05	0.366
155	FBgn0023083	chr3R	+	18573090	18589453	18573090	18573525	18586278	18589453	0.017481374	-0.404
156	FBgn0267706	chr3R	+	19191980	19193028	19191980	19192051	19192126	19193028	5.91689E-05	-0.671
157	FBgn0001987	chr2L	-	15760401	15762408	15760401	15760574	15762306	15762408	0.000319186	0.664
158	FBgn0053483	chr3R	-	30900933	30901322	30900933	30901101	30901166	30901322	0.004138406	0.612
159	FBgn0004598	chrX	+	16374540	16375859	16374540	16374760	16374925	16375859	0.000143713	-0.65
160	FBgn0262828	chrX	+	7450666	7451651	7450666	7450904	7450985	7451651	0.047789772	-0.434
161	FBgn0027079	chr2R	+	13198849	13199184	13198849	13198919	13198960	13199184	0.002072892	0.304
162	FBgn0001228	chr3L	+	9372721	9373095	9372721	9372839	9372909	9373095	0.028354859	0.202
163	FBgn0037130	chr3L	-	21760957	21770534	21760957	21761265	21770445	21770534	9.8134E-05	0.3
164	FBgn0010348	chr3L	-	22869841	22871559	22869841	22870150	22871319	22871559	0.012986941	-0.317
165	FBgn0019985	chr4	+	943733	944601	943733	944253	944305	944601	0.004101778	-0.283
166	FBgn0035861	chr3L	-	8239136	8240236	8239136	8239417	8239538	8240236	3.54074E-09	0.603
167	FBgn0035861	chr3L	-	8239538	8240601	8239538	8240236	8240299	8240601	0	0.842
168	FBgn0004910	chr3L	-	3930957	3935617	3930957	3930996	3935485	3935617	4.44163E-11	-0.897
169	FBgn0011640	chr3L	+	7339463	7340770	7339463	7339617	7339961	7340770	0	-0.574
170	FBgn0035104	chr3L	+	179922	181088	179922	179991	180739	181088	0.000319376	0.695
171	FBgn0033112	chr2R	-	6879284	6879805	6879284	6879490	6879663	6879805	3.96999E-10	0.812
172	FBgn0011817	chr3L	+	7979045	7980982	7979045	7979173	7980399	7980982	0.007978875	0.181
173	FBgn0016694	chr3L	-	7830386	7833073	7830386	7831810	7832553	7833073	6.13989E-05	-0.331
174	FBgn0051092	chr3R	-	25718397	25720117	25718397	25718623	25719965	25720117	0.036450667	0.212
175	FBgn0031516	chr2L	-	3301122	3303130	3301122	3301753	3303045	3303130	0.003604445	-0.314
176	FBgn0052391	chr3L	-	7068853	7069199	7068853	7068998	7069050	7069199	5.39057E-05	0.366
177	FBgn0033639	chr2R	-	11650814	11651616	11650814	11651207	11651323	11651616	0.006609057	-0.193
178	FBgn0033639	chr2R	-	11650814	11651616	11650814	11651224	11651323	11651616	0	-0.473
179	FBgn0036685	chr3L	-	17015140	17016051	17015140	17015266	17015672	17016051	0.001508832	-0.482
180	FBgn0013995	chr3R	+	20985978	20994423	20985978	20986083	20992240	20994423	0.017642511	0.331
181	FBgn0015037	chr2R	+	9240603	9240842	9240603	9240685	9240741	9240842	0	-0.885
182	FBgn0038256	chr3R	-	14730510	14731509	14730510	14730629	14731404	14731509	0	-0.425
183	FBgn0014127	chr2L	-	20060411	20060977	20060411	20060724	20060828	20060977	0.000150075	0.392
184	FBgn0033159	chr2R	-	7348130	7357915	7348130	7348975	7357788	7357915	0.001997029	0.69
185	FBgn0033159	chr2R	-	7348130	7358129	7348130	7348975	7358002	7358129	0.025238735	0.59
186	FBgn0033159	chr2R	-	7348130	7360414	7348130	7348975	7360290	7360414	0.034313732	0.59
187	FBgn0033159	chr2R	-	7358002	7361867	7358002	7358129	7361717	7361867	0.001809938	0.63
188	FBgn0033159	chr2R	-	7361717	7364348	7361717	7361867	7364177	7364348	3.84781E-12	0.822
189	FBgn0003721	chr3R	+	15281549	15284762	15281549	15281853	15284612	15284762	0.034877196	-0.167
190	FBgn0266395	chr3L	-	22225519	22225878	22225519	22225715	22225772	22225878	0.000462492	-0.809
191	FBgn0027527	chr3R	+	6234707	6236880	6234707	6234954	6235582	6236880	0.005246368	-0.391
192	FBgn0044419	chr3L	+	5781996	5782849	5781996	5782308	5782624	5782849	0.025250314	0.369
193	FBgn0020647	chr3R	-	24030493	24031827	24030493	24031031	24031711	24031827	6.13989E-05	0.336
194	FBgn0032705	chr2L	+	18709543	18711297	18709543	18710385	18710440	18711297	1.1759E-06	-0.533
195	FBgn0024273	chr3R	-	28830501	28831313	28830501	28830677	28831218	28831313	0.010269235	-0.381
196	FBgn0050197	chr2R	+	14514029	14514215	14514029	14514084	14514152	14514215	0.00101362	0.084
197	FBgn0037265	chr3R	+	4655158	4656655	4655158	4655270	4655517	4656655	0.023947618	-0.306
198	FBgn0036768	chr3L	+	17887704	17889462	17887704	17887790	17889330	17889462	1.20986E-05	0.717
199	FBgn0046687	chrX	-	5673829	5674709	5673829	5674173	5674221	5674709	0.010291814	0.421
200	FBgn0025628	chrX	-	2071359	2073483	2071359	2071580	2073376	2073483	0.00475771	0.247
201	FBgn0033042	chr2R	+	5795412	5796448	5795412	5796326	5796384	5796448	0.01670614	-0.685
202	FBgn0033054	chr2R	-	6038070	6040334	6038070	6038446	6040225	6040334	0.009500484	-0.312
203	FBgn0085285	chr3L	-	18988735	18989533	18988735	18989217	18989466	18989533	0.00956675	0.154
204	FBgn0039850	chr3R	+	31463541	31464134	31463541	31463751	31463813	31464134	0.016157373	-0.14
205	FBgn0010352	chr3L	+	16955327	16959216	16955327	16955542	16959098	16959216	0.028354859	-0.1
206	FBgn0010352	chr3L	+	16957438	16959216	16957438	16957505	16959098	16959216	6.54398E-06	-0.383
207	FBgn0053181	chrX	+	8491344	8492030	8491344	8491436	8491947	8492030	0.007839516	-0.29
208	FBgn0039925	chr4	+	1165549	1169239	1165549	1166367	1166665	1169239	0.047724003	-0.506
209	FBgn0265977	chr2R	-	22318994	22320895	22318994	22320622	22320781	22320895	2.17352E-07	0.337
210	FBgn0033518	chr2R	+	10419387	10419761	10419387	10419463	10419526	10419761	0.017651706	0.1
211	FBgn0015904	chr3L	+	12585741	12589634	12585741	12586293	12589518	12589634	2.04042E-09	0.334
212	FBgn0040606	chr3R	-	26424697	26425102	26424697	26425019	26425076	26425102	1.12803E-12	0.373
213	FBgn0033853	chr2R	-	13538971	13547012	13538971	13539349	13546514	13547012	1.97574E-09	-0.273

214	FBgn0037633	chr3R	-	8805191	8805802	8805191	8805701	8805782	8805802	0.013696668	0.267
215	FBgn0033960	chr2R	-	14753336	14755590	14753336	14753880	14753986	14755590	0.004138406	0.63
216	FBgn0034031	chr2R	+	15559859	15560203	15559859	15559998	15560097	15560203	0.001879337	-0.578
217	FBgn0034031	chr2R	+	15555612	15562436	15555612	15555649	15561814	15562436	0.010936611	-0.542
218	FBgn0034031	chr2R	+	15555923	15562436	15555923	15556014	15561814	15562436	2.10106E-09	-0.631
219	FBgn0034031	chr2R	+	15557243	15562436	15557243	15557310	15561814	15562436	0.010728691	-0.42
220	FBgn0034031	chr2R	+	15557528	15562436	15557528	15557607	15561814	15562436	0.004051948	-0.422
221	FBgn0034031	chr2R	+	15559236	15562436	15559236	15559722	15561814	15562436	0.004329641	-0.372
222	FBgn0034031	chr2R	+	15561362	15562436	15561362	15561417	15561814	15562436	9.33411E-09	-0.527
223	FBgn0034051	chr2R	-	15935892	15938978	15935892	15936580	15938114	15938978	0.00017805	-0.239
224	FBgn0034363	chr2R	+	18615987	18616361	18615987	18616137	18616196	18616361	1.10169E-11	0.216
225	FBgn0053460	chr2R	+	15694374	15694628	15694374	15694417	15694476	15694628	0.003604445	-0.515
226	FBgn0034715	chr2R	-	22222659	22223269	22222659	22222980	22223039	22223269	0.005143405	-0.499
227	FBgn0260008	chr3L	+	25120893	25121813	25120893	25120985	25121617	25121813	0.001616786	-0.387
228	FBgn0034903	chr2R	-	23753713	23757166	23753713	23754337	23756363	23757166	0.000935878	-0.22
229	FBgn0051183	chr3R	-	15869172	15875939	15869172	15870232	15875577	15875939	0	-0.656
230	FBgn0039637	chr3R	+	29123415	29124104	29123415	29123499	29123800	29124104	0.004028069	-0.375
231	FBgn0264078	chrX	+	14839375	14841019	14839375	14839535	14840872	14841019	0.006478747	-0.628
232	FBgn0264078	chrX	+	14839967	14841019	14839967	14840356	14840872	14841019	0.017461606	-0.645
233	FBgn0030674	chrX	+	15713640	15714381	15713640	15713815	15713900	15714381	4.20128E-07	0.704
234	FBgn0035476	chr3L	-	3939478	3939887	3939478	3939651	3939719	3939887	0	-0.444
235	FBgn0035476	chr3L	-	3939719	3940125	3939719	3939887	3940006	3940125	0.000405095	-0.269
236	FBgn0040271	chr3R	-	16314364	16322169	16314364	16315246	16321962	16322169	9.61019E-07	0.702
237	FBgn0035755	chr3L	-	7262948	7263920	7262948	7263095	7263631	7263920	1.25666E-06	-0.466
238	FBgn0035766	chr3L	-	7335441	7338988	7335441	7335875	7335937	7338988	0	-0.218
239	FBgn0035769	chr3L	-	7354041	7356459	7354041	7356005	7356066	7356459	0	-0.607
240	FBgn0035769	chr3L	-	7356066	7356713	7356066	7356459	7356580	7356713	0.005144863	-0.371
241	FBgn0259822	chr2L	-	11159879	11161290	11159879	11160336	11161067	11161290	0.000400651	0.297
242	FBgn0259822	chr2L	-	11159879	11161834	11159879	11160336	11161554	11161834	0.015054273	0.349
243	FBgn0035934	chr3L	-	8896851	8897375	8896851	8896962	8897060	8897375	0.01203036	0.501
244	FBgn0035976	chr3L	+	9346590	9347249	9346590	9346745	9346807	9347249	0.004053997	-0.223
245	FBgn0035976	chr3L	+	9346807	9348336	9346807	9347249	9347639	9348336	0.015054273	-0.269
246	FBgn0036007	chr3L	-	9497115	9497366	9497115	9497211	9497293	9497366	0.007839516	0.378
247	FBgn0036110	chr3L	+	10894172	10894693	10894172	10894202	10894266	10894693	3.09542E-07	-0.399
248	FBgn0085292	chr3L	-	265594	265908	265594	265641	265697	265908	0.010546868	0.315
249	FBgn0036290	chr3L	-	12500630	12501042	12500630	12500800	12500874	12501042	0.003684052	-0.619
250	FBgn0036305	chr3L	+	12750230	12750739	12750230	12750344	12750603	12750739	0.001322039	-0.463
251	FBgn0036305	chr3L	+	12750507	12752922	12750507	12750739	12751306	12752922	2.0792E-10	0.901
252	FBgn0036661	chr3L	+	16780099	16780790	16780099	16780420	16780504	16780790	0	0.292
253	FBgn0036350	chr3L	+	13379271	13380380	13379271	13379615	13379993	13380380	3.7562E-07	-0.645
254	FBgn0036360	chr3L	-	13430278	13435229	13430278	13430963	13434741	13435229	0	-0.784
255	FBgn0036433	chr3L	+	14622795	14626894	14622795	14622935	14626840	14626894	1.74577E-11	-0.206
256	FBgn0036566	chr3L	+	16090455	16091858	16090455	16090521	16091515	16091858	5.53445E-05	-0.225
257	FBgn0267390	chr3L	+	15566012	15568970	15566012	15566153	15568205	15568970	0.030212912	0.191
258	FBgn0033499	chr2R	+	10247813	10248244	10247813	10247972	10248074	10248244	0.013172819	-0.482
259	FBgn0260965	chr3L	+	12814253	12818525	12814253	12814880	12816198	12818525	0.000804113	-0.213
260	FBgn0031835	chr2L	-	6571953	6573805	6571953	6572542	6573698	6573805	7.97784E-05	0.503
261	FBgn0261553	chr3L	+	11215778	11217282	11215778	11215951	11216585	11217282	2.28097E-11	-0.584
262	FBgn0264953	chr2L	+	8182626	8183563	8182626	8182878	8183188	8183563	0.044279556	-0.417
263	FBgn0010548	chr2R	-	7473721	7474659	7473721	7473843	7474571	7474659	9.61019E-07	-0.253
264	FBgn0037533	chr3R	-	7978017	7978428	7978017	7978080	7978345	7978428	0.032268277	0.6
265	FBgn0035772	chr3L	-	7366441	7367984	7366441	7366516	7367558	7367984	0	-0.306
266	FBgn0039597	chr3R	-	28755483	28756979	28755483	28755748	28755919	28756979	0.000723819	-0.405
267	FBgn0037364	chr3R	-	5682621	5685434	5682621	5682987	5685397	5685434	0.035209382	-0.595
268	FBgn0038420	chr3R	-	16352088	16357015	16352088	16352359	16356799	16357015	0	-0.952
269	FBgn0038721	chr3R	-	19639025	19641086	19639025	19639413	19640983	19641086	0.000760353	0.522
270	FBgn0038947	chr3R	+	22356684	22357671	22356684	22356798	22357530	22357671	5.26117E-05	-0.269
271	FBgn0039593	chr3R	+	28670837	28672021	28670837	28671236	28671286	28672021	2.32939E-08	0.336
272	FBgn0016031	chr3L	-	5347627	5353787	5347627	5347683	5353678	5353787	0.000550162	0.667
273	FBgn0038294	chr3R	-	15172599	15174343	15172599	15172830	15173980	15174343	0	0.295
274	FBgn0038294	chr3R	-	15172599	15174343	15172599	15172830	15174136	15174343	0.00798947	0.122
275	FBgn0038294	chr3R	-	15172843	15174343	15172843	15173063	15174136	15174343	6.5924E-13	-0.213
276	FBgn0039301	chr3R	+	25247657	25248106	25247657	25247777	25247958	25248106	0.004122981	-0.395
277	FBgn0039360	chr3R	-	25686314	25687149	25686314	25686866	25686945	25687149	5.261E-12	-0.523
278	FBgn0039427	chr3R	+	26452533	26453022	26452533	26452657	26452853	26453022	0.005484806	-0.502
279	FBgn0040608	chr3R	+	26727313	26730117	26727313	26727379	26729766	26730117	0.001236489	0.309
280	FBgn0042138	chr3L	+	11628216	11628501	11628216	11628250	11628342	11628501	0.000921836	-0.427
281	FBgn0285963	chr3R	+	26915760	26916562	26915760	26915908	26916512	26916562	0.011534051	0.216
282	FBgn0285949	chr2R	+	9587446	9587989	9587446	9587708	9587776	9587989	0.001677345	-0.353
283	FBgn0285925	chr3R	+	16625968	16629598	16625968	16626216	16629284	16629598	0.001959492	0.588
284	FBgn0266599	chr3R	+	15242686	15242943	15242686	15242799	15242909	15242943	0.009565725	-0.204
285	FBgn0266449	chr2L	+	6072212	6072983	6072212	6072315	6072413	6072983	0.007745674	-0.509
286	FBgn0264605	chr3L	-	16680811	16681087	16680811	16680947	16681008	16681087	0.00304822	-0.474
287	FBgn0263219	chr3L	+	8279893	8280409	8279893	8279966	8280274	8280409	0.015673521	-0.426
288	FBgn0262623	chr3L	-	13299840	13300152	13299840	13299929	13299989	13300152	0.006478747	0.529
289	FBgn0260745	chr3R	-	11992741	11993315	11992741	11992884	11993248	11993315	0.012866138	0.491
290	FBgn0260745	chr3R	-	11992741	11993315	11992741	11993096	11993248	11993315	3.09542E-07	0.995
291	FBgn0266489	chr2R	-	13590599	13593339	13590599	13591878	13593125	13593339	0.00100177	0.533
292	FBgn0259139	chr3R	-	11818572	11819339	11818572	11818956	11819210	11819339	0.001728752	-0.636
293	FBgn0250868	chr2R	+	17572828	17573330	17572828	17573033	17573095	17573330	0.033684256	-0.582
294	FBgn0250819	chr4	+	1185767	1186221	1185767	1185834	1186177	1186221	0.047831239	-0.201
295	FBgn0085443	chrX	-	10555202	10557362	10555202	10555397	10557217	10557362	0.033684256	0.222
296	FBgn0053092	chr3R	+	21877935	21878305	21877935	21878071	21878251	21878305	0	0.947
297	FBgn0052944	chr3R	-	4415682	4419578	4415682	4415886	4419359	4419578	4.42199E-09	0.728
298	FBgn0052264	chr3L	-	3741995	3746584	3741995	3743772	3746304	3746584	5.69709E-06	-0.446
299	FBgn0052181	chr3L	+	17760720	17761139	17760720	17760975	17761050	17761139	0.022872149	-0.242
300	FBgn0051973	chr2L	-	25401	26964	25401	26688	26761	26964	0.022999231	-0.304
301	FBgn0051365	chr3R	-	23123961	23125195	23123961	23125001	23125064	23125195	0.022872149	0.61
302	FBgn0051286	chr3R	+	6642083	6643346	6642083	6642246	6642413	6643346	0	-0.793
303	FBgn0051221	chr3R	+	19415637	19440831	19415637	19416388	19440705	19440831	0.019062302	0.533
304	FBgn0051189	chr3R	-	21045200	21045842	21045200	21045591	21045741	21045842	0	0.852
305	FBgn0262592	chr3L	-	10625205	10625599	10625205	10625322	10625394	10625599	0.003488987	-0.626
306	FBgn0051388	chr3R	+	11239510	11240114	11239510	11239824	11239895	11240114	0.001158523	-0.781
307	FBgn0050021	chr2R	-	11229939	11232516	11229939	11230048	11232256	11232516	0.004620537	0.55
308	FBgn0264357	chr3R	+	21210366	21211079	21210366	21210705	21210791	21211079	0	-0.801
309	FBgn0031942	chr2L	-	7752169	7753153	7752169	7752814	7752976	7753153	0.003538331	0.184
310	FBgn0031942	chr2L	-	7752169	7753153	7752169	7752814	7752989	7753153	3.07493E-10	-0.267
311	FBgn0031589	chr2L	-	4196474	4197191	4196474	4196779	4197095	4197191	0.005209836	0.608
312	FBgn0031815	chr2L	+	6456051	6456682	6456051	6456208	6456326	6456682	0.027761702	-0.587
313	FBgn0014380	chr3R	-	9497879	9502581	9497879	9498164	9502421	9502581	0.008230211	0.733
314	FBgn0028579	chr2R	+	6741898	6742769	6741898	6742149	6742389	6742769	0.035366283	-0.424
315	FBgn0028380	chr3L	+	19294766	19296296	19294766	19295098	19296147	19296296	0.000145321	0.451
316	FBgn0264489	chr3L	+	9830999	9832679	9830999	9831440	9832531	9832679	0	0.642
317	FBgn0031170	chrX	-	21305529	21306665	21305529	21305743	21306648	21306665	0.004620537	0.544
318	FBgn0030629	chrX	-	15340372	15341162	15340372	15340991	15341138	15341162	0.004541962	-0.401
319	FBgn0000721	chr2L	-	3655269	3656953	3655269	3655585	3656544	3656953	3.27517E-06	0.778
320	FBgn0259243	chr3L	+	20828224	20829540	20828224	20828340	20829291	20829540	0.019901526	0.295
321	FBgn0266702	chr3R	-	5835477	5836178	5835477	5835938	5836008	5836178	3.68533E-07	0.622
322	FBgn0037698	chr3R	-	9486600	9493677	9486600	9487203	9493473	9493677	0.001848323	-0.788
323	FBgn0000289	chr2R	+	14174461	14176542	14174461	14174572	14176393	14176542	0.015289572	0.361
324	FBgn0039234	chr3R	+	24852737	24853814	24852737	24853539	24853611	24853814	0.034313732	-0.667
325	FBgn0031857	chr2L	-	6770590	6772755	6770590	6770718	6772551	6772755	0.006771235	0.147
326	FBgn0025702	chr3L	+	22282903	22284335	22282903	22283138	22283202	22284335	0	0.205
327	FBgn0025702	chr3L	+	22282903	22284335	22282903	22283144	22283202	22284335	5.17975E-13	0.213
328	FBgn0025936	chr4	+	619636	620107	619636	619783	619859	620107	6.4328E-09	0.452
329	FBgn0037376	chr3R	-	5788765	5789384	5788765	5789166	5789328	5789384	0.000290646	-0.667
330	FBgn0033448	chr2R	-	9828103	9833572	9828103	9829859	9833406	9833572	0.041908099	-0.289

331	FBgn0261261	chr3R	+	5685854	5687369	5685854	5686270	5687156	5687369	0.028869934	0.447
332	FBgn0026250	chr3R	+	18274866	18283005	18274866	18275048	18281693	18283005	4.82494E-05	-0.646
333	FBgn0001987	chr2L	-	15760401	15762406	15760401	15760574	15762395	15762406	0.003137433	0.599
334	FBgn0033919	chr2R	-	14264128	14267497	14264128	14264215	14267353	14267497	1.39721E-08	-0.767
335	FBgn0036760	chr3L	-	17841539	17843544	17841539	17842557	17843323	17843544	0.013916309	-0.491
336	FBgn0028408	chr2R	+	9297265	9302821	9297265	9297542	9298266	9302821	0.002346124	0.495
337	FBgn0010408	chr3L	-	9511055	9511531	9511055	9511301	9511399	9511531	0.045459684	-0.474
338	FBgn0053208	chr3R	-	10008695	10009372	10008695	10008854	10009318	10009372	0.043562291	-0.401
339	FBgn0053208	chr3R	-	10041971	10042821	10041971	10041998	10042571	10042821	6.55828E-05	0.66
340	FBgn0023172	chr2R	+	17035654	17036901	17035654	17036407	17036563	17036901	0.012866138	0.37
341	FBgn0028746	chrX	-	3172453	3173140	3172453	3172916	3173044	3173140	0.003281614	0.593
342	FBgn0016694	chr3L	-	7830386	7833073	7830386	7831810	7832861	7833073	0.031533249	-0.374
343	FBgn0016694	chr3L	-	7830386	7833073	7830386	7831827	7832863	7833073	0.039069908	0.302
344	FBgn0031717	chr2L	+	5547289	5548100	5547289	5547728	5547807	5548100	0	0.918
345	FBgn0051092	chr3R	-	25718397	25720117	25718397	25718614	25719951	25720117	0	-0.743
346	FBgn0051092	chr3R	-	25718397	25720117	25718397	25718614	25719965	25720117	0	-0.601
347	FBgn0039348	chr3R	-	25611052	25611937	25611052	25611449	25611541	25611937	0.00730473	0.46
348	FBgn0039348	chr3R	-	25611052	25611937	25611052	25611449	25611580	25611937	0.007878152	-0.378
349	FBgn0000308	chr2L	-	5979411	5980101	5979411	5979704	5980025	5980101	0.038097344	0.608
350	FBgn0000308	chr2L	-	5979411	5981018	5979411	5979704	5980558	5981018	0.013493456	0.619
351	FBgn0032726	chr2L	-	18953403	18954431	18953403	18953778	18954271	18954431	0.013514088	0.463
352	FBgn0016119	chr3R	-	23355633	23356011	23355633	23355785	23355841	23356011	0.032012273	0.403
353	FBgn0028670	chr3R	-	18393271	18396928	18393271	18393409	18396785	18396928	0.004101778	-0.313
354	FBgn0020647	chr3R	-	23999006	24000117	23999006	23999562	23999639	24000117	0.006737196	-0.328
355	FBgn0023529	chrX	-	2274025	2274778	2274025	2274339	2274668	2274778	0.049652783	-0.63
356	FBgn0052473	chr3R	+	13304763	13308904	13304763	13305070	13308511	13308904	0.026185101	-0.564
357	FBgn0037676	chr3R	-	9326687	9328511	9326687	9327312	9328435	9328511	0.012091532	0.433
358	FBgn0032889	chr2L	+	20825667	20826970	20825667	20826087	20826622	20826970	0.040682682	-0.328
359	FBgn0033403	chr2R	-	9350671	9351655	9350671	9351173	9351440	9351655	0.000279888	-0.677
360	FBgn0036493	chr3L	+	15305576	15306798	15305576	15305679	15306504	15306798	0	0.837
361	FBgn0000299	chr2L	+	5030136	5030586	5030136	5030237	5030567	5030586	0.013172819	-0.506
362	FBgn0033853	chr2R	-	13542160	13545449	13542160	13542563	13545215	13545449	0.004681627	-0.329
363	FBgn0004175	chr3R	-	7363306	7363773	7363306	7363631	7363690	7363773	0.001479181	-0.616
364	FBgn0052803	chrX	-	1901436	1901911	1901436	1901614	1901667	1901911	0.004028069	0.335
365	FBgn0051183	chr3R	-	15869172	15875939	15869172	15870232	15875565	15875939	1.42296E-11	-0.628
366	FBgn0039637	chr3R	+	29123415	29125737	29123415	29123479	29123800	29125737	0.030822017	0.224
367	FBgn0035811	chr3L	+	7719349	7720416	7719349	7719457	7720021	7720416	4.50297E-09	0.523
368	FBgn0036305	chr3L	+	12750230	12750739	12750230	12750344	12750605	12750739	0.017832818	0.695
369	FBgn0032639	chr2L	-	17457755	17458044	17457755	17457806	17457960	17458044	0.000707727	0.762
370	FBgn0036821	chr3L	-	18882318	18884845	18882318	18882717	18884801	18884845	0.015875147	0.386
371	FBgn0265356	chr2R	+	19001091	19004883	19001091	19001789	19004613	19004883	5.15774E-06	-0.446
372	FBgn0037778	chr3R	+	10046397	10047260	10046397	10046718	10046775	10047260	0.03985897	0.384
373	FBgn0050345	chr2R	+	9149686	9150087	9149686	9149837	9149890	9150087	0.034877196	0.385
374	FBgn0037963	chr3R	-	11947380	11949129	11947380	11947637	11948836	11949129	8.28932E-07	-0.952
375	FBgn0037963	chr3R	-	11947380	11949129	11947380	11947692	11948836	11949129	0.001624126	-0.272
376	FBgn0262351	chr3L	+	18925090	18925585	18925090	18925387	18925443	18925585	6.37582E-05	0.679
377	FBgn0262351	chr3L	+	18925090	18925585	18925090	18925387	18925458	18925585	1.82778E-06	0.618
378	FBgn0261932	chr3R	+	17406677	17407199	17406677	17406769	17407101	17407199	8.2024E-06	0.655
379	FBgn0039897	chr4	+	231934	232848	231934	231977	232394	232848	0.00018135	0.609
380	FBgn0011225	chr3R	-	24254633	24255224	24254633	24254678	24254938	24255224	0.049652783	-0.262
381	FBgn0038721	chr3R	-	19638266	19638592	19638266	19638427	19638584	19638592	0.032295742	-0.456
382	FBgn0038294	chr3R	-	15172843	15174343	15172843	15173063	15174137	15174343	0	0.805
383	FBgn0039323	chr3R	-	25315367	25316116	25315367	25315858	25315919	25316116	0.003462053	0.46
384	FBgn0039481	chr3R	-	27089703	27091130	27089703	27090606	27090671	27091130	0.005274691	-0.428
385	FBgn0267047	chrX	-	19938478	19938994	19938478	19938510	19938612	19938994	0.004122981	-0.289
386	FBgn0039830	chr3R	-	31216769	31217944	31216769	31217177	31217775	31217944	0.005412706	-0.646
387	FBgn0004656	chrX	-	8059866	8061436	8059866	8060158	8061299	8061436	0.001209728	-0.533
388	FBgn0041096	chr3L	-	12055193	12056474	12055193	12055708	12056186	12056474	0.012489324	0.6
389	FBgn0041337	chr2L	-	2564885	2565567	2564885	2565248	2565309	2565567	0.001077612	-0.529
390	FBgn0041775	chr3L	+	12517427	12518224	12517427	12517957	12518038	12518224	4.10205E-06	0.586

SE											
	FlyBase ID	chr	strand	exonStart_0base	exonEnd	upstreamES	upstreamEE	downstreamES	downstreamEE	FDR	ΔPSI
1	FBgn0052082	chr3L	+	11163386	11163488	11160780	11160932	11163554	11163984	0.009521835	-0.206
2	FBgn0052111	chr3L	+	12642046	12642878	12633328	12633945	12644801	12645040	9.70096E-06	-0.541
3	FBgn0035438	chr3L	+	3327342	3327553	3326264	3326367	3327859	3328183	0.038477975	0.126
4	FBgn0035438	chr3L	+	3327342	3327553	3326264	3326712	3327859	3328183	0.021582272	0.147
5	FBgn0286203	chr2R	+	5439569	5439754	5423749	5424239	5446534	5446765	8.06909E-07	0.756
6	FBgn0285963	chr3R	+	26916502	26916562	26915760	26915908	26924633	26925261	3.32704E-06	0.198
7	FBgn0265998	chr3R	+	28893201	28895062	28890004	28890100	28900823	28901022	4.22E-05	0.219
8	FBgn0266137	chr3R	-	29638931	29639105	29630303	29632860	29640228	29640310	3.18654E-05	-0.511
9	FBgn0263346	chr3R	+	4680778	4680837	4659578	4660399	4697190	4697542	0	0.407
10	FBgn0263346	chr3R	+	4680778	4680837	4670796	4670850	4697190	4697542	0	0.662
11	FBgn0263111	chrX	-	11960074	11960080	11956921	11956924	11962019	11962103	0.00460853	-0.389
12	FBgn0262579	chr3L	+	8096970	8097172	8096521	8096872	8098345	8098725	0.00471794	0.213
13	FBgn0261984	chr3R	-	19860646	19860761	19857240	19857684	19860885	19861291	9.19547E-13	0.18
14	FBgn0264489	chr3L	+	9830999	9831440	9830633	9830785	9832195	9832679	0	-0.29
15	FBgn0264489	chr3L	+	9830999	9831440	9830633	9830785	9832198	9832679	0	-0.612
16	FBgn0034718	chr2R	-	22308207	22308256	22298213	22301009	22311695	22311937	0.036471651	0.111
17	FBgn0046874	chr3R	-	8793977	8794091	8793103	8793367	8796704	8796936	0.03585254	0.127
18	FBgn0013995	chr3R	+	20986146	20986382	20985978	20986083	20992240	20994423	0.01580225	0.289
19	FBgn0033807	chr2R	-	13213750	13213931	13213022	13213436	13214418	13214496	0	0.519
20	FBgn0016920	chr2L	+	5362582	5362812	5361997	5362209	5363655	5363784	0.000344487	-0.651
21	FBgn0051973	chr2L	-	30393	33270	28981	29068	33844	34288	1.33644E-06	0.199
22	FBgn0000723	chr3R	-	9372376	9372577	9369335	9369710	9372755	9372933	0.02084161	0.172
23	FBgn0000723	chr3R	-	9372376	9372580	9369335	9369710	9372755	9372933	0.005198409	0.158
24	FBgn0004838	chr2L	+	6922730	6922826	6920703	6920861	6923783	6924400	0.006826298	-0.093
25	FBgn0027580	chr2R	-	9853682	9853730	9852547	9853205	9854510	9856020	0.004298616	0.114
26	FBgn0030230	chrX	-	10754292	10754904	10753212	10753676	10754970	10755014	0.043691623	0.667
27	FBgn0263077	chr2R	+	7630176	7630328	7628796	7629112	7634215	7634314	0.002749882	-0.166
28	FBgn0034051	chr2R	-	15937678	15937879	15935892	15936580	15938114	15938267	0.011716656	-0.178
29	FBgn0034051	chr2R	-	15937678	15937879	15935892	15936580	15938446	15938978	0.028813563	0.145
30	FBgn0034051	chr2R	-	15937678	15937879	15936090	15936583	15938446	15938978	0.000931606	0.212
31	FBgn0032860	chr2L	-	20423350	20423480	20423105	20423273	20424274	20424427	0.022487223	0.592
32	FBgn0003429	chr3R	+	24699375	24699426	24695340	24695417	24702087	24702162	0.030621037	0.204
33	FBgn0003429	chr3R	+	24702087	24702162	24696090	24696126	24703117	24703307	0.007883506	0.291
34	FBgn0011640	chr3L	+	7339867	7339908	7339463	7339617	7339961	7340770	0	-0.8
35	FBgn0016930	chr2L	-	14229458	14229536	14222016	14222081	14233647	14234108	1.53042E-05	0.233
36	FBgn0016930	chr2L	-	14229531	14229536	14222016	14222081	14233647	14234108	0.002667638	0.105
37	FBgn0013718	chr3L	+	14231596	14231635	14231176	14231325	14231926	14232500	4.15577E-05	-0.163
38	FBgn0058178	chr3L	-	26008060	26008177	25977966	25978150	26014020	26014112	0.021582272	-0.431
39	FBgn0260794	chr3R	-	4810671	4810787	4801212	4801991	4811982	4812096	0.048699972	-0.286
40	FBgn0261552	chr3R	+	9439345	9439705	9436834	9437118	9440680	9441665	0.000163053	-0.27
41	FBgn0031473	chr2L	-	2854723	2855100	2853250	2853605	2855185	2855309	0.03488533	-0.328
42	FBgn0011206	chr3L	-	9123615	9123910	9113045	9113487	9125166	9125313	0.007094376	-0.159
43	FBgn0052082	chr3L	+	11163415	11163488	11160780	11160932	11163554	11163984	0.030726733	-0.394
44	FBgn0030890	chrX	-	18085193	18085587	18082218	18082379	18088365	18088532	0.044384468	0.327
45	FBgn0035539	chr3L	+	4384808	4384887	4384441	4384627	4390449	4391221	4.56675E-06	-0.208
46	FBgn0035438	chr3L	+	3327351	3327553	3326264	3326367	3327859	3328183	0.038893056	0.12
47	FBgn0035438	chr3L	+	3327363	3327553	3326264	3326367	3327859	3328183	0.038893056	0.12
48	FBgn0266137	chr3R	-	29638947	29639105	29630303	29632860	29640228	29640310	0.000171016	-0.579
49	FBgn0264006	chr3L	-	13873533	13874008	13873071	13873266	13877623	13877629	0.004474555	-0.667
50	FBgn0263980	chr2R	+	15447831	15448050	15425227	15426627	15452856	15453008	7.94255E-06	0.23
51	FBgn0263346	chr3R	+	4670796	4670845	4659578	4660399	4697190	4697542	5.11488E-07	0.223
52	FBgn0263346	chr3R	+	4680708	4680837	4670796	4670850	4697190	4697542	0	0.808

53	FBgn0263346	chr3R	+	4680778	4681199	4659578	4660399	4697190	4697542	0.001408831	0.687
54	FBgn0263346	chr3R	+	4680778	4681199	4670796	4670850	4697190	4697542	0	0.833
55	FBgn0262636	chr4	-	392299	392430	390424	391544	393262	393410	0.021728655	0.403
56	FBgn0262579	chr3L	+	8097023	8097172	8096521	8096872	8098345	8098725	0.001896975	0.22
57	FBgn0261984	chr3R	-	19860490	19860761	19857240	19857684	19860885	19861291	0	0.373
58	FBgn0004910	chr3L	-	3933246	3933389	3930957	3930996	3935485	3935617	5.23378E-13	-0.373
59	FBgn0004910	chr3L	-	3933246	3933492	3930957	3930996	3935485	3935617	2.86612E-13	-0.378
60	FBgn0004910	chr3L	-	3933246	3933511	3930957	3930996	3935485	3935617	0	-0.387
61	FBgn0004910	chr3L	-	3933246	3933553	3930957	3930996	3935485	3935617	2.00628E-13	-0.387
62	FBgn0031634	chr2L	-	4834566	4834777	4834106	4834256	4835246	4835300	0.007584292	-0.507
63	FBgn0264489	chr3L	+	9830975	9831440	9830633	9830785	9832195	9832679	1.1146E-13	-0.397
64	FBgn0264489	chr3L	+	9830975	9831440	9830633	9830785	9832198	9832679	3.67304E-10	-0.67
65	FBgn0264489	chr3L	+	9830975	9831440	9830633	9830785	9832201	9832679	0.021728655	-0.415
66	FBgn0264489	chr3L	+	9831023	9831440	9830633	9830785	9832195	9832679	8.5869E-12	-0.383
67	FBgn0264489	chr3L	+	9831023	9831440	9830633	9830785	9832198	9832679	7.03059E-10	-0.666
68	FBgn0264489	chr3L	+	9831125	9831440	9830633	9830785	9832195	9832679	3.64779E-13	-0.391
69	FBgn0264489	chr3L	+	9831125	9831440	9830633	9830785	9832198	9832679	6.46989E-10	-0.667
70	FBgn0264489	chr3L	+	9831125	9831440	9830633	9830785	9832201	9832679	0.038351825	-0.379
71	FBgn0264489	chr3L	+	9831293	9831440	9830633	9830785	9832195	9832679	2.00628E-13	-0.409
72	FBgn0264489	chr3L	+	9831293	9831440	9830633	9830785	9832198	9832679	0	-0.701
73	FBgn0264489	chr3L	+	9831293	9831440	9830633	9830785	9832201	9832679	0.003985982	-0.249
74	FBgn0264489	chr3L	+	9831434	9831440	9830633	9830785	9832195	9832679	0	-0.45
75	FBgn0264489	chr3L	+	9831434	9831440	9830633	9830785	9832198	9832679	0	-0.907
76	FBgn0264489	chr3L	+	9831434	9831440	9830633	9830785	9832201	9832679	0.0008781	-0.478
77	FBgn0039360	chr3R	-	25686945	25687063	25686314	25686866	25687250	25687370	1.56968E-06	0.265
78	FBgn0034718	chr2R	-	22301363	22301716	22298213	22301009	22311695	22311937	0.008034553	0.223
79	FBgn0039897	chr4	+	231068	231410	230729	230895	231934	231977	0.000269093	-0.254
80	FBgn0011666	chr3R	+	25563874	25564127	25519260	25519729	25574883	25574922	0.007083408	-0.332
81	FBgn0015949	chr2R	-	19668789	19668959	19668353	19668478	19669117	19669832	5.46311E-06	0.587
82	FBgn0036975	chr3L	+	20354817	20355129	20353958	20354405	20355304	20356280	0.033501554	0.337
83	FBgn0265959	chr3L	+	20248847	20249060	20248079	20248150	20255897	20256638	0.028170627	-0.155
84	FBgn0051286	chr3R	+	6641906	6642246	6641621	6641842	6642413	6643346	1.36221E-06	-0.186
85	FBgn0051286	chr3R	+	6641917	6642358	6641621	6641842	6642413	6643346	1.7121E-05	-0.5
86	FBgn0031449	chr2L	+	2736068	2736229	2730103	2730469	2736310	2736430	0.016360732	0.652
87	FBgn0033807	chr2R	-	13213532	13213931	13213022	13213436	13214418	13214496	0	0.559
88	FBgn0033807	chr2R	-	13213750	13213949	13213022	13213436	13214418	13214496	0	0.572
89	FBgn0033807	chr2R	-	13213751	13213931	13213022	13213436	13214418	13214496	0	0.594
90	FBgn0032901	chr2L	-	20918291	20918301	20916904	20917120	20918638	20918931	0.037175313	0.667
91	FBgn0023083	chr3R	+	18577574	18577584	18573191	18573525	18586278	18589453	5.48076E-06	-0.592
92	FBgn0051973	chr2L	-	30393	30673	28981	29068	33844	34288	1.3579E-05	0.184
93	FBgn0263120	chr2R	+	8666767	8666794	8665812	8666071	8674646	8674871	0.004065879	0.339
94	FBgn0002772	chr3R	-	27658066	27658124	27657077	27657955	27658468	27658555	0.035024108	-0.073
95	FBgn0261638	chr3R	+	6775848	6775952	6767049	6767276	6779244	6779284	0.008138096	0.513
96	FBgn0016726	chr2R	+	21293849	21293878	21293767	21293789	21294053	21294161	0.014195643	0.431
97	FBgn0016726	chr2R	+	21293853	21293878	21293767	21293789	21294053	21294161	0.0136826	0.458
98	FBgn0051523	chr3R	-	4475260	4475338	4472840	4473188	4479061	4479207	0.005225033	0.588
99	FBgn0053170	chr3L	+	22865328	22865696	22864841	22864925	22865770	22865824	0.043559124	0.111
100	FBgn0053170	chr3L	+	22865425	22865696	22864841	22864925	22865770	22865824	0.043559124	0.111
101	FBgn0053170	chr3L	+	22865500	22865696	22864841	22864925	22865770	22865824	0.03100307	0.113
102	FBgn0053170	chr3L	+	22865527	22865696	22864841	22864925	22865770	22865824	0.046099374	0.111
103	FBgn0263077	chr2R	+	7630056	7630328	7628800	7629112	7634215	7634314	0.00167808	-0.231
104	FBgn0263077	chr2R	+	7630147	7630328	7628800	7629112	7634215	7634314	0.001502622	-0.231
105	FBgn0263077	chr2R	+	7630185	7630328	7628800	7629112	7634215	7634314	0.001439831	-0.242
106	FBgn0034031	chr2R	+	15556206	15556478	15555923	15556014	15561814	15562436	3.1585E-06	-0.506
107	FBgn0034031	chr2R	+	15556282	15556478	15555923	15556014	15561814	15562436	1.68836E-06	-0.518
108	FBgn0033809	chr2R	-	13218264	13218303	13217588	13218199	13219087	13219247	0.006183102	0.43
109	FBgn0039348	chr3R	-	25623477	25623876	25623171	25623405	25624588	25624662	0.019731474	0.149
110	FBgn0264607	chr4	-	1050681	1050898	1050454	1050609	1051258	1051378	0.00058143	-0.809
111	FBgn0264607	chr4	-	1050681	1050913	1050454	1050609	1051258	1051378	3.28445E-05	-0.857
112	FBgn0264607	chr4	-	1050681	1050942	1050454	1050609	1051258	1051378	0.00058143	-0.809
113	FBgn0040608	chr3R	+	26727819	26727918	26727313	26727379	26729766	26730117	0.003343747	0.202
114	FBgn0011640	chr3L	+	7339728	7339905	7339463	7339617	7339961	7340770	1.94476E-06	-0.867
115	FBgn0011640	chr3L	+	7339833	7339905	7339463	7339617	7339961	7340770	5.84138E-07	-0.888
116	FBgn0011640	chr3L	+	7339867	7339905	7339463	7339617	7339961	7340770	0	-0.881
117	FBgn0016930	chr2L	-	14229453	14229536	14222016	14222081	14233647	14234108	0.012507177	0.235
118	FBgn0031515	chr2L	-	3294082	3294118	3293322	3293949	3294230	3294706	0.001073938	-0.565
119	FBgn0031515	chr2L	-	3294082	3294166	3293322	3293949	3294230	3294706	0.042564673	-0.494
120	FBgn0265991	chr2R	-	15773040	15773150	15765122	15765447	15773815	15773966	0.000451776	0.177
121	FBgn0046692	chr2R	+	4561530	4561677	4546690	4547054	4562844	4563081	0.008532542	0.59
122	FBgn0051323	chr3R	+	26171195	26171455	26170846	26171038	26183799	26183964	7.04018E-08	-0.516
123	FBgn0051323	chr3R	+	26171300	26171455	26170846	26171038	26183799	26183964	1.51421E-07	-0.501
124	FBgn0051323	chr3R	+	26171331	26171455	26170846	26171038	26183799	26183964	2.30501E-09	-0.591
125	FBgn0086711	chr2L	-	14994108	14994285	14993898	14994035	14997515	14997559	0.000269093	-0.739
126	FBgn0261552	chr3R	+	9436933	9437118	9432601	9432839	9440680	9441665	0.003362921	0.304
127	FBgn0261552	chr3R	+	9439294	9439705	9436834	9437118	9440680	9441665	2.97412E-05	-0.283
128	FBgn0261552	chr3R	+	9439348	9439705	9436834	9437118	9440680	9441665	0.000125087	-0.283

5) List of DEGs in *Drosophila* heads of *Dm65k*$^{Δ/Δ}$ *vs WT*

	FlyBase ID	Gene Symbol	*WT* (nomarlized rds)	*Dm65k*$^{Δ/Δ}$ (nomarlized rds)	log2FC (*Dm65k*$^{Δ/Δ}$ *vs WT*)	padj
1	FBgn0000055	Adh	103.884	432.469	2.052051563	2.15E-15
2	FBgn0000079	Amy-p	448.456	941.409	1.070137652	4.18E-19
3	FBgn0000276	CecA1	4.63486	32.64	2.837827515	2.37E-02
4	FBgn0000277	CecA2	1.00151	78.7991	6.359759707	6.52E-09
5	FBgn0000473	Cyp6a2	6389.63	1225.45	-2.383389513	3.84E-45
6	FBgn0000639	Fbp1	2462.92	21706.9	3.139746756	1.15E-22
7	FBgn0000640	Fbp2	986.397	3314.97	1.74881758	2.00E-04
8	FBgn0001224	Hsp23	1045.98	2471.99	1.241115796	1.86E-11
9	FBgn0002531	Lcp1	3.00947	56.4963	4.178774893	1.44E-07
10	FBgn0002564	Lsp1gamma	542.94	1166.62	1.103790766	2.75E-09
11	FBgn0002565	Lsp2	343.511	2277.26	2.730338147	5.85E-52
12	FBgn0002570	Mal-A1	124.852	255.764	1.035297175	1.12E-04
13	FBgn0003082	phr	1536.14	33.158	-5.542528478	4.02E-152
14	FBgn0003089	pip	108.842	272.251	1.326065687	1.21E-06
15	FBgn0003250	Rh4	12006.9	5808.76	-1.047462074	1.26E-52
16	FBgn0003935	snRNA:U5:34A	0.660971	21.2986	5.047121494	2.48E-03
17	FBgn0003950	unc	27.8622	7.39888	-1.906701849	1.30E-02
18	FBgn0004781	Ccp84Ac	15.3731	46.9812	1.617879937	2.54E-02
19	FBgn0004783	Ccp84Aa	324.309	103.052	-1.654454489	1.25E-10
20	FBgn0005390	fs(1)M3	32.9169	10.9005	-1.610044636	3.23E-02
21	FBgn0005638	slbo	13.2013	36.3702	1.457765002	4.04E-02
22	FBgn0005778	PpD5	20.4187	3.82127	-2.44525853	1.75E-02
23	FBgn0010197	Gyc32E	90.3912	44.3173	-1.030173002	5.57E-03
24	FBgn0010388	Dro	23.7487	145.008	2.617480307	1.09E-10
25	FBgn0010423	TpnC47D	2395.98	1039.04	-1.204771946	1.40E-12
26	FBgn0012026	tRNA:Tyr-GTA-1-6	11.8342	0	-5.917080851	4.80E-03
27	FBgn0012042	AttA	8.36562	942.015	6.829170894	1.09E-77
28	FBgn0013275	Hsp70Aa	258.367	56.6791	-2.186926581	2.17E-15
29	FBgn0013276	Hsp70Ab	677.034	110.344	-2.616971255	1.71E-51
30	FBgn0013277	Hsp70Ba	171.599	31.9096	-2.427474168	4.04E-14
31	FBgn0013278	Hsp70Bb	320.943	144.202	-1.153148815	2.08E-05
32	FBgn0013308	Odc2	62.115	6.39411	-3.284389897	1.29E-07
33	FBgn0013772	Cyp6a8	320.716	968.739	1.592987373	1.41E-17
34	FBgn0013813	Dhc98D	359.042	68.3105	-2.402685885	9.77E-27
35	FBgn0014469	Cyp4e2	911.609	2066.79	1.179650274	4.04E-25
36	FBgn0014865	Mtk	16.3525	96.3223	2.565138088	2.43E-08
37	FBgn0015037	Cyp4p1	935.003	2726.16	1.54300096	1.72E-44
38	FBgn0015376	cutlet	71.3908	215.39	1.593096983	1.52E-10
39	FBgn0016054	phr6-4	5.04265	29.4737	2.542582161	1.84E-03
40	FBgn0016684	NaPi-T	91.1456	364.48	1.999160081	1.97E-18
41	FBgn0025456	CREG	903.945	2483.19	1.457626175	3.45E-28
42	FBgn0025620	CG13360	79.4541	237.084	1.58035125	4.57E-11
43	FBgn0026563	CG1979	146.408	59.7392	-1.295738763	6.31E-06
44	FBgn0027348	bgm	1027.03	2422.91	1.23738163	4.47E-37
45	FBgn0027500	spd-2	16.1515	42.6274	1.396388697	1.75E-02
46	FBgn0027513	ana2	54.3221	24.1302	-1.175176455	2.48E-02
47	FBgn0027584	CG4757	392.401	921.284	1.231386313	5.86E-20
48	FBgn0027929	NimB1	169.082	342.008	1.018149974	2.24E-08
49	FBgn0028396	TotA	408.328	5236.74	3.680967552	1.44E-18
50	FBgn0028583	lcs	46.8032	122.813	1.389238907	4.32E-03
51	FBgn0028855	CG15282	58.9779	126.725	1.101263981	1.12E-03
52	FBgn0028867	CR15280	16.8869	55.8112	1.727770959	1.69E-03
53	FBgn0028939	NimC2	181.503	394.076	1.117423019	7.70E-06
54	FBgn0029667	Gas8	0	9.84989	5.81016558	8.30E-03
55	FBgn0029798	CG4078	216.778	532.141	1.296603667	1.22E-17
56	FBgn0029813	AgmNAT	12.3214	40.9984	1.743212952	1.65E-02
57	FBgn0029924	CG4586	28.6125	79.5608	1.475469026	2.65E-04
58	FBgn0029990	CG2233	3950.77	11202.1	1.503516696	3.09E-44
59	FBgn0030073	CG10962	150.64	366.489	1.284534497	1.01E-10
60	FBgn0030105	CG15369	131.608	475.509	1.85499362	2.71E-20
61	FBgn0030826	CG12995	84.4252	32.7926	-1.364456767	1.35E-03
62	FBgn0030850	stas	3224.5	7689.64	1.254105257	6.69E-55
63	FBgn0030895	CG7135	94.1068	212.604	1.172634656	8.11E-04
64	FBgn0030993	Mec2	124.174	256.952	1.046164601	2.05E-06
65	FBgn0031012	CG8051	668.438	1372.88	1.038352917	1.35E-09
66	FBgn0031305	Iris	57.879	267.502	2.204321521	1.92E-21
67	FBgn0031471	CG3117	27.1602	84.3561	1.643514298	3.09E-03

68	FBgn0031484	CG3165	383.805	814.957	1.084654888	9.36E-07
69	FBgn0031533	CG2772	15.5529	46.5705	1.578564738	5.78E-03
70	FBgn0031560	CG16713	694.924	1942.23	1.483283534	1.09E-12
71	FBgn0031689	Cyp28d1	3379.36	11073.8	1.712102764	1.39E-48
72	FBgn0031690	CG7742	34.4548	84.9351	1.301356875	2.39E-03
73	FBgn0031701	TotM	3.5811	431.707	6.944292852	5.53E-42
74	FBgn0031707	CG14020	38.5904	7.5207	-2.368497057	5.36E-04
75	FBgn0031801	CG9498	260.714	569.089	1.124539117	6.52E-09
76	FBgn0031835	CG11319	2666.38	6205.26	1.21902199	4.19E-51
77	FBgn0031853	TTLL3B	99.2971	10.9765	-3.162136783	9.02E-12
78	FBgn0032143	CG4017	53.5274	25.2568	-1.092143844	3.68E-02
79	FBgn0032211	CG13138	65.9454	17.3706	-1.917762605	1.25E-04
80	FBgn0032253	LManI	386.075	776.078	1.006819852	6.37E-09
81	FBgn0032282	CG7299	130.853	306.78	1.232416694	3.36E-09
82	FBgn0032490	CG16813	7.84823	0	-5.329014837	1.89E-02
83	FBgn0032493	Mabi	359.126	158.437	-1.18425981	2.91E-06
84	FBgn0032535	Ance-2	38.3693	450.083	3.550431812	1.26E-52
85	FBgn0032549	CG4650	7.86833	57.6839	2.866212821	4.51E-07
86	FBgn0032634	Rpb11	68.813	140.076	1.023493997	2.24E-03
87	FBgn0032638	SPH93	73.4048	158.452	1.11346231	2.86E-03
88	FBgn0032639	CG18563	37.2595	109.568	1.559590315	2.69E-05
89	FBgn0032665	CG15152	2.56954	21.877	3.096297286	4.77E-03
90	FBgn0032693	Cyp310a1	7.47554	28.9562	1.93788302	1.75E-02
91	FBgn0032726	CG10621	413.175	1149.92	1.476732982	1.37E-11
92	FBgn0032848	nesd	22.7944	52.3404	1.194846502	2.65E-02
93	FBgn0032908	CG9270	71.9403	25.135	-1.520307114	4.80E-04
94	FBgn0032945	CG8665	802.475	3047.06	1.92505797	1.35E-21
95	FBgn0033065	Cyp6w1	4134.71	11640.2	1.493299323	1.62E-44
96	FBgn0033135	Tsp42En	157.037	75.1612	-1.062402876	4.49E-04
97	FBgn0033137	Tsp42Ep	26.5182	267.807	3.337840988	9.02E-27
98	FBgn0033149	CG11060	24.7273	3.82127	-2.710860779	1.28E-02
99	FBgn0033154	CG1850	19.2334	57.2424	1.568823062	1.73E-03
100	FBgn0033170	sPLA2	336.291	850.859	1.340424287	9.88E-08
101	FBgn0033204	CG2065	204.061	496.913	1.283743089	1.86E-11
102	FBgn0033216	CG1946	26.8487	55.6592	1.05171099	4.70E-02
103	FBgn0033234	MFS12	169.83	439.961	1.370426358	6.47E-08
104	FBgn0033274	CG14757	387.247	1041.75	1.426342258	1.04E-32
105	FBgn0033275	CG14756	235.479	15.422	-3.93298315	5.59E-30
106	FBgn0033287	CG8701	8.70815	0	-5.472272423	1.34E-02
107	FBgn0033289	CG2121	1169.5	334.534	-1.808807667	2.91E-43
108	FBgn0033302	Cyp6a14	212.197	23.5669	-3.180000563	1.58E-21
109	FBgn0033366	Ance-4	171.572	371.498	1.112277933	3.07E-06
110	FBgn0033374	CG13741	9.72777	54.9741	2.510427961	4.68E-05
111	FBgn0033395	Cyp4p2	2474.26	38.6083	-6.014143881	3.17E-212
112	FBgn0033439	CG1773	54.948	155.605	1.506166062	5.44E-06
113	FBgn0033464	CG1441	438.95	933.584	1.089315147	9.43E-18
114	FBgn0033501	CG12911	53.6219	11.2201	-2.262814615	2.55E-05
115	FBgn0033518	Prx2540-2	2357.29	11060.7	2.230094783	2.20E-237
116	FBgn0033706	Vha36-2	19.6331	3.19705	-2.626992388	1.65E-02
117	FBgn0033736	CG13154	422.195	189.586	-1.158411717	6.22E-08
118	FBgn0033748	vis	74.5039	4.64329	-3.969535335	6.54E-10
119	FBgn0033756	CG17760	27.7928	78.1755	1.487517593	3.91E-04
120	FBgn0033769	CG8768	536.251	264.792	-1.019363424	5.09E-11
121	FBgn0033821	CG10799	207.357	492.087	1.248866497	9.94E-15
122	FBgn0033830	CG10814	2.78349	31.6509	3.479411657	1.26E-04
123	FBgn0033836	CG18278	69.3015	209.94	1.599928641	6.28E-07
124	FBgn0033926	Arc1	1819.4	5075.03	1.479420256	4.74E-61
125	FBgn0033927	CR10102	333.125	888.338	1.412735655	7.80E-18
126	FBgn0033979	Cyp6a19	17.9065	60.3634	1.754747132	4.04E-04
127	FBgn0034059	CG8320	437.869	946.068	1.113003366	1.96E-18
128	FBgn0034129	Parp16	3.60121	45.4439	3.683151914	1.25E-06
129	FBgn0034143	CG8303	4390.75	2166.01	-1.020055107	4.45E-12
130	FBgn0034219	mthl4	32.954	66.6357	1.012548109	2.91E-02
131	FBgn0034221	CG10764	22.3274	55.842	1.327456478	2.37E-02
132	FBgn0034229	CG4847	9718.16	24904.4	1.357659987	6.65E-27
133	FBgn0034279	CG18635	50.4466	2.19228	-4.560880748	1.96E-06
134	FBgn0034290	CG5773	146.576	343.15	1.229184585	2.14E-08
135	FBgn0034294	Muc55B	22.6035	54.8523	1.271259902	1.65E-02
136	FBgn0034301	CG5756	3.13408	18.3754	2.544032989	2.75E-02
137	FBgn0034335	GstE1	2496.71	912.075	-1.454357318	5.64E-24
138	FBgn0034407	DptB	3.54896	22.4704	2.67715174	3.10E-02
139	FBgn0034454	CG15120	68.0819	194.822	1.515595984	8.41E-09

140	FBgn0034470	Obp56d	2151.96	4921.56	1.19396041	7.09E-32
141	FBgn0034471	Obp56e	12.7272	53.2993	2.068250681	1.57E-04
142	FBgn0034479	CG8654	144.394	344.109	1.251498699	1.41E-09
143	FBgn0034480	CG16898	170.84	77.8408	-1.139484631	1.14E-03
144	FBgn0034499	Cpr56F	359.483	120.453	-1.582423057	6.08E-12
145	FBgn0034506	CG13870	19.1429	2.69467	-2.844403567	2.05E-02
146	FBgn0034510	CG13426	1.89852	19.1365	3.333161219	1.64E-02
147	FBgn0034511	GNBP-like3	1147.36	2344.66	1.031943851	1.64E-09
148	FBgn0034539	CG11159	593.713	297.433	-1.000685011	3.57E-06
149	FBgn0034563	CG15649	1.2476	15.4829	3.612863383	8.20E-03
150	FBgn0034565	CG15650	3.75285	19.5779	2.379182359	4.92E-02
151	FBgn0034568	CG3216	27.6493	75.5267	1.447239274	2.31E-03
152	FBgn0034629	Acox57D-d	354.155	90.629	-1.972316481	4.79E-21
153	FBgn0034631	TAF1C-like	1202.42	358.754	-1.743833576	1.36E-43
154	FBgn0034662	CG13492	3.31786	79.4239	4.525876027	3.46E-11
155	FBgn0034664	CG4377	110.089	28.0883	-1.96890431	5.75E-08
156	FBgn0034687	CG11475	183.157	59.3128	-1.623698158	2.56E-10
157	FBgn0034761	CG4250	507.994	1219.84	1.263019326	5.02E-15
158	FBgn0034819	CG9877	26.6859	3.13614	-3.073572543	1.52E-03
159	FBgn0034828	CG13545	6.81968	70.9286	3.39818492	6.24E-09
160	FBgn0034829	CG9899	0.660971	20.248	4.979716384	1.74E-03
161	FBgn0034957	CG3121	4.80661	20.0502	2.060093675	4.87E-02
162	FBgn0034965	ppk29	65.6367	23.3082	-1.506124426	1.30E-02
163	FBgn0034973	CG13564	31.1629	104.726	1.745480696	1.48E-05
164	FBgn0035210	msd5	19.4022	48.4883	1.326097363	3.73E-02
165	FBgn0035325	CG13806	10.3446	44.3933	2.109621668	1.23E-02
166	FBgn0035328	yellow-g2	29.4535	111.44	1.926600174	5.53733E-06
167	FBgn0035434	Drsl5	33.0254	203.332	2.626982288	9.31634E-15
168	FBgn0035496	CG14990	89.7915	205.859	1.198957182	1.47702E-05
169	FBgn0035555	CG13720	380.482	156.443	-1.285599983	1.3397E-06
170	FBgn0035582	CG13705	93.1968	191.245	1.036843142	7.56279E-05
171	FBgn0035623	mthl2	106.74	224.662	1.071475515	0.003564122
172	FBgn0035693	CG8219	14.887	2.63375	-2.486234415	0.046081726
173	FBgn0035791	CG8539	339.573	1594.75	2.231149539	1.11174E-62
174	FBgn0036015	CG3088	1891.09	935.746	-1.014581557	4.53236E-20
175	FBgn0036024	CG18180	48.7853	279.301	2.521215696	6.59362E-21
176	FBgn0286980	lyd	22.695	53.4211	1.23886605	0.01938911
177	FBgn0036186	CG6071	47.3245	17.6752	-1.429091333	0.010921343
178	FBgn0036324	CG12520	52.4044	117.332	1.159576927	0.000881801
179	FBgn0036332	Cul6	61.6057	128.172	1.052355055	0.006671336
180	FBgn0036410	CG8100	36.712	164.527	2.164088006	1.31026E-11
181	FBgn0036436	CG4914	10.5473	66.2244	2.63576539	4.34289E-06
182	FBgn0036493	CG7255	497.482	1030.58	1.049289546	8.02443E-15
183	FBgn0036563	CG13075	6.90095	102.504	3.880955114	1.92939E-11
184	FBgn0036575	CG5157	14.715	0	-6.23647401	0.001566357
185	FBgn0036588	CG13068	108.992	228.559	1.068226833	2.12751E-05
186	FBgn0036590	CG13065	1544.87	3236.21	1.066950916	0.003638528
187	FBgn0036600	CG13043	4405.11	15055.2	1.773210826	3.27755E-86
188	FBgn0036601	CG13063	150.524	503.809	1.745395251	5.00066E-25
189	FBgn0036602	CG13042	11.3378	36.3551	1.662709237	0.047105702
190	FBgn0036607	CG13059	259.647	122.066	-1.08664231	2.35887E-06
191	FBgn0036612	CG4998	88.5397	26.3834	-1.757780295	1.75222E-05
192	FBgn0036627	Gagr	299.669	671.883	1.162777545	5.86563E-16
193	FBgn0036640	nxf2	93.9049	18.2994	-2.370577271	7.48411E-09
194	FBgn0036659	CG9701	1431.36	3280.8	1.196698645	3.03417E-12
195	FBgn0036698	CG7724	398.448	845.299	1.085919704	3.24998E-18
196	FBgn0036731	CG6333	2.29131	15.5438	2.781756386	0.030910691
197	FBgn0036752	Adgf-A	78.9498	250.085	1.666243333	1.7097E-13
198	FBgn0036757	Ir75a	5.99191	84.1124	3.806348266	9.01066E-11
199	FBgn0036787	CG4306	79.04	220.307	1.477827446	9.61145E-10
200	FBgn0036806	Cyp12c1	331.191	820.441	1.309406564	1.08182E-14
201	FBgn0036910	Cyp305a1	518.07	1053.58	1.02465716	1.66667E-14
202	FBgn0036953	CG17145	60.8342	372.883	2.615999713	2.65531E-20
203	FBgn0036995	atk	147.059	21.3746	-2.786743727	2.1687E-15
204	FBgn0037123	CG14569	517.094	159.061	-1.704459645	9.4075E-15
205	FBgn0037126	CG14567	299.003	931.969	1.643100373	4.2224E-18
206	FBgn0037181	CG11370	71.648	267.989	1.905626118	8.84031E-17
207	FBgn0037225	TwdlG	196.022	438.772	1.159579288	3.49956E-11
208	FBgn0037229	CG9766	122.542	296.61	1.275914383	1.51822E-10
209	FBgn0037288	CG14661	703.63	1435.45	1.028216054	5.73814E-26
210	FBgn0037304	CG1113	95.0872	24.9673	-1.916958541	1.55282E-05
211	FBgn0037396	CG11459	125.966	532.674	2.079774329	4.16543E-31

212	FBgn0037420	CG15597	139.979	404.806	1.535196314	2.19509E-11
213	FBgn0037488	CG14607	123.136	53.8626	-1.190968045	0.00020934
214	FBgn0037627	CG13318	139.649	474.275	1.768503841	6.03651E-15
215	FBgn0037727	Nepl12	225.551	66.3312	-1.763701701	1.21581E-12
216	FBgn0037742	Rpt3R	73.3277	28.3929	-1.370993246	0.002587563
217	FBgn0037750	Whamy	136.302	31.5291	-2.116038704	2.92352E-11
218	FBgn0037751	topi	113.826	24.2062	-2.226758975	8.28011E-10
219	FBgn0037761	CG8534	35.4373	1.00477	-5.065741274	0.00014361
220	FBgn0037762	eloF	15.8492	1.00477	-3.918572358	0.017001235
221	FBgn0037930	CG14715	309.194	72.01	-2.110208517	1.06587E-19
222	FBgn0037992	CG4702	32.7814	120.711	1.886916774	0.000404654
223	FBgn0037999	CG4860	146.013	66.9855	-1.116291056	0.002144806
224	FBgn0038001	CG17404	207.048	77.4904	-1.417030405	1.30931E-09
225	FBgn0038017	CG4115	4460.14	2211.21	-1.012769008	6.65972E-12
226	FBgn0038056	CG5961	295.809	606.358	1.035381458	1.82114E-12
227	FBgn0038074	Gnmt	43.0866	101.026	1.233670332	0.001259925
228	FBgn0038079	NijC	1202.51	2743.6	1.189589811	1.10539E-13
229	FBgn0038095	Cyp304a1	10.684	91.0096	3.078876261	8.16774E-11
230	FBgn0038147	CCHa2	884.998	3146.58	1.829570041	2.10634E-87
231	FBgn0038159	CG14369	17.6121	3.13614	-2.474722663	0.038686468
232	FBgn0038214	CG9616	17.3922	73.3036	2.084982169	1.48689E-05
233	FBgn0038299	Spn88Eb	549.323	1211.73	1.142607185	1.03576E-14
234	FBgn0038645	CG7714	562.214	221.632	-1.34558066	2.63645E-14
235	FBgn0038820	CG4000	1125.18	3188.25	1.502689701	0.000919967
236	FBgn0038838	TotB	3.85934	25.135	2.709405042	0.005460039
237	FBgn0038846	CG5697	2097.29	6464.56	1.624120027	1.03589E-07
238	FBgn0039027	CG7031	32.069	80.5656	1.328532568	0.001687462
239	FBgn0039073	CG4408	52.263	326.343	2.650207985	6.41259E-25
240	FBgn0039272	CG11836	12.8236	34.7261	1.439637953	0.040237353
241	FBgn0039684	Obp99d	996.842	192.493	-2.369297136	1.0111E-68
242	FBgn0039685	Obp99b	1669.26	6067.98	1.862071202	3.24861E-10
243	FBgn0039703	CG7829	154.674	1.6899	-6.596733997	1.75022E-12
244	FBgn0039752	CG15530	31.4684	64.8849	1.043080914	0.020433278
245	FBgn0039801	Npc2h	642.386	1298.82	1.016161685	2.04572E-17
246	FBgn0039817	CG15553	151.183	39.0498	-1.958943904	2.91057E-11
247	FBgn0039818	CG11318	0.969364	11.7225	3.610598987	0.033097921
248	FBgn0040099	lectin-28C	144.633	293.687	1.024594864	6.50985E-07
249	FBgn0040102	lectin-24Db	196.851	429.077	1.121983901	0.000692289
250	FBgn0040104	lectin-24A	5.46758	24.5107	2.171350655	0.011874723
251	FBgn0040391	CG2854	28.1795	64.2456	1.184433227	0.021623648
252	FBgn0040503	CG7763	46.2046	196.634	2.089040319	3.71292E-11
253	FBgn0040799	CG13051	11.9045	34.9698	1.557286704	0.029536306
254	FBgn0040849	Ir41a	7.04567	29.885	2.083322705	0.015140802
255	FBgn0041180	Tep4	2288.97	8951.45	1.967625595	8.41759E-63
256	FBgn0041579	AttC	31.817	167.981	2.403021309	3.36202E-09
257	FBgn0041581	AttB	35.2623	194.897	2.464383709	4.31605E-09
258	FBgn0041709	yellow-g	245.795	627.9	1.350876811	5.40048E-12
259	FBgn0041710	yellow-f	149.698	340.197	1.180493724	1.58829E-08
260	FBgn0042178	Apl	38.7733	236.81	2.616222807	1.33895E-21
261	FBgn0043533	Obp56f	25.372	77.7491	1.615076346	0.000107865
262	FBgn0043535	Obp57a	91.6966	386.08	2.070438384	1.31026E-11
263	FBgn0043575	PGRP-SC2	21.0374	112.11	2.406003282	5.73691E-10
264	FBgn0043578	PGRP-SB1	272.174	692.997	1.34882029	1.96472E-18
265	FBgn0043854	slam	35.9114	12.6055	-1.500565993	0.02760011
266	FBgn0044047	Ilp6	500.93	1012.05	1.014306355	1.00883E-08
267	FBgn0044809	TotZ	0	12.986	6.208663978	0.001737962
268	FBgn0044810	TotX	31.836	111.226	1.804622415	0.000222644
269	FBgn0044812	TotC	11.6624	911.656	6.292771947	1.60911E-11
270	FBgn0046253	CG3502	34.9479	71.2941	1.025545827	0.028025169
271	FBgn0046880	Obp56b	0.681076	25.0131	5.279735584	0.000428692
272	FBgn0050033	CG30033	80.7159	31.8487	-1.338242107	0.001737962
273	FBgn0050042	Cpr49Ab	141.854	58.5216	-1.28543025	0.000684259
274	FBgn0050050	CG30050	154.728	73.654	-1.075693969	0.00052104
275	FBgn0050082	CG30082	50.8897	11.9813	-2.070866084	0.000562411
276	FBgn0050090	CG30090	11.2656	155.681	3.80057027	1.68187E-22
277	FBgn0050098	CG30098	46.5159	13.4275	-1.776142373	0.006362739
278	FBgn0050280	CG30280	314.134	132.8	-1.246065236	2.1585E-06
279	FBgn0050287	CG30287	5.77597	37.6186	2.710882148	0.000192724
280	FBgn0050288	CG30288	1.89852	15.8634	3.058998914	0.027446406
281	FBgn0050289	CG30289	6.67298	88.406	3.727487491	2.78122E-13
282	FBgn0050334	CG30334	52.6464	7.33796	-2.831481252	6.50051E-06
283	FBgn0050395	CG30395	158.801	53.1015	-1.582645803	1.7297E-07

284	FBgn0050403	CG30403	36.1233	81.3267	1.175273924	0.003836342
285	FBgn0050488	antr	14.6699	1.1266	-3.766674648	0.020973569
286	FBgn0051029	CG31029	67.6268	21.1159	-1.692393288	0.000530097
287	FBgn0051076	CG31076	11.353	40.9984	1.846755221	0.004763265
288	FBgn0051220	CG31220	41.1962	4.70421	-3.112860289	2.24465E-05
289	FBgn0051274	CG31274	19.5086	47.3467	1.284194705	0.039871149
290	FBgn0051373	CG31373	18.0532	45.8395	1.353758174	0.024700885
291	FBgn0051453	pch2	60.5464	209.346	1.797573246	9.75806E-07
292	FBgn0051634	Oatp26F	11.3751	66.0115	2.531136007	4.41386E-07
293	FBgn0051683	CG31683	319.577	1513.7	2.243738256	7.13697E-55
294	FBgn0051704	CG31704	13.6291	61.6879	2.162202044	0.000133881
295	FBgn0051781	lncRNA:CR31781	7241.62	3172.66	-1.190139081	2.85818E-41
296	FBgn0051860	ZnT33D	90.8484	22.3794	-2.022169678	2.53668E-06
297	FBgn0051909	CG31909	14.3917	1.56807	-3.180137046	0.025161654
298	FBgn0052207	hpRNA:CR32207	78.0125	1.06568	-6.199143195	2.14669E-07
299	FBgn0052266	CG32266	469.583	164.846	-1.512381855	1.1464E-13
300	FBgn0052302	CG32302	4.52837	32.5338	2.857290984	0.000494957
301	FBgn0052303	tRNA:Cys-GCA-3-1	3.71264	23.2014	2.627477731	0.02323761
302	FBgn0052364	tut	116.571	56.5573	-1.042838142	0.00172907
303	FBgn0052475	mthl8	1381.38	0	-12.78518567	1.0459E-16
304	FBgn0052553	lncRNA:CR32553	162.234	38.4406	-2.071360503	3.6789E-12
305	FBgn0052581	CG32581	1425.46	118.808	-3.583442908	1.3433E-150
306	FBgn0052814	CG32814	33.584	81.5101	1.276996332	0.024977361
307	FBgn0053054	CG33054	95.4728	231.816	1.278505652	2.94332E-07
308	FBgn0053108	CG33108	74.5469	29.0322	-1.356001911	0.001349081
309	FBgn0053225	CG33225	6.78754	30.7679	2.186743178	0.009514026
310	FBgn0053281	CG33281	548.489	2225.8	2.020870976	1.13001E-67
311	FBgn0053286	CG33286	23.7817	83.1227	1.798819477	0.006238322
312	FBgn0053307	CG33307	3.59116	30.7221	3.119260045	0.000339126
313	FBgn0053348	CheB42a	5.65137	21.2528	1.902059388	0.041638938
314	FBgn0053458	CG33458	27.2265	94.0848	1.796937055	5.62274E-05
315	FBgn0053460	CG33460	14.6137	48.5951	1.734415387	0.002481533
316	FBgn0053462	CG33462	2.60975	23.6278	3.197063382	0.00320418
317	FBgn0053470	CG33470	228.23	684.777	1.588569941	1.09477E-12
318	FBgn0053474	CG33474	8.47507	35.3503	2.050765214	0.007743734
319	FBgn0053483	CG33483	36.067	1.56807	-4.504146741	9.78179E-05
320	FBgn0053508	ppk13	31.6823	1.1266	-4.889643411	0.000254656
321	FBgn0053758	CG33758	0.681076	17.8121	4.786360772	0.004287241
322	FBgn0053926	CG33926	26.3192	75.5267	1.516830861	0.000735848
323	FBgn0054038	CG34038	176.363	67.8691	-1.384311512	2.20803E-05
324	FBgn0058160	CG40160	722.556	1876.17	1.377332771	9.83316E-35
325	FBgn0062928	hpRNA:CR33940	181.102	15.4829	-3.554064504	1.3689E-23
326	FBgn0066292	CheB42c	69.6297	192.689	1.469108774	0.003398765
327	FBgn0069969	CG40498	3459.62	1709.84	-1.016831437	4.08252E-41
328	FBgn0083972	CG34136	973.757	2256.99	1.212409988	2.13619E-14
329	FBgn0085235	CG34206	23.3137	174.803	2.907415771	9.61668E-17
330	FBgn0085244	CG34215	113.299	44.5151	-1.349239033	0.000228493
331	FBgn0085310	CG34281	113.116	52.3253	-1.115878554	0.012185146
332	FBgn0085421	Epac	9368.58	28773.9	1.618899161	4.6794E-103
333	FBgn0085486	CG34457	5.75587	25.2719	2.14064159	0.011843057
334	FBgn0085491	CG34462	4.61277	30.0979	2.732674821	0.001998657
335	FBgn0085732	CR40190	188.424	0	-9.911333501	5.53562E-10
336	FBgn0250832	Dup99B	122.432	32.7316	-1.897379062	1.21697E-07
337	FBgn0250907	Cht10	159.566	337.381	1.079987713	5.90004E-06
338	FBgn0259209	Mlp60A	4844.89	10084	1.057677295	7.36712E-13
339	FBgn0259710	CG42364	28.0139	0.563299	-5.716748145	0.001837947
340	FBgn0259711	CG42365	96.9643	196.603	1.014629764	0.000541396
341	FBgn0259713	CG42367	13.9346	40.1914	1.526877247	0.013926904
342	FBgn0259716	Nepl6	225.329	526.844	1.224390324	2.14879E-15
343	FBgn0259739	CG42393	0.618773	9.59116	3.924525829	0.046363307
344	FBgn0260474	CG30002	65.0492	212.452	1.705341738	2.02977E-12
345	FBgn0260482	CG32599	134.589	62.3121	-1.111842479	0.000140096
346	FBgn0260720	asRNA:CR42547	23.0878	5.26751	-2.122531572	0.017553602
347	FBgn0260874	Ir76a	16.2209	99.3975	2.628753531	5.76452E-08
348	FBgn0260954	CG42586	22.4439	4.07999	-2.425723805	0.031056154
349	FBgn0260995	dpr21	267.089	618.248	1.21252818	2.75181E-16
350	FBgn0261292	CheB53a	16.6417	136.819	3.029108385	1.18764E-12
351	FBgn0261402	Ir75b	7.02045	55.2178	2.949658878	0.000158065
352	FBgn0261429	lncRNA:CR42646	20.7895	2.51193	-2.995642568	0.014206029
353	FBgn0261505	CR42653	43.9233	0	-7.812546748	8.91399E-06
354	FBgn0261534	l(2)34Fc	4.26712	27.4792	2.664599782	0.010807777
355	FBgn0261566	CG42680	0	17.0051	6.598363022	0.000465559

356	FBgn0261584	CG42694	25.161	54.4717	1.107065698	0.042232463
357	FBgn0261803	CG42749	780.116	364.981	-1.097320432	6.42927E-18
358	FBgn0261925	CG42792	5.43544	156.929	4.858965941	2.43188E-20
359	FBgn0261989	CG42807	59.85	214.446	1.845965531	1.48329E-12
360	FBgn0262107	asRNA:CR42860	5.43544	24.0843	2.154957847	0.018893773
361	FBgn0262362	CG43060	17.7064	111.744	2.648012559	3.6678E-06
362	FBgn0262481	CG43071	9.9859	28.6516	1.528535226	0.046041164
363	FBgn0262531	CG43085	15.4655	81.6922	2.410207146	1.46253E-07
364	FBgn0262532	CR43086	18.3093	0	-6.545263461	0.000571055
365	FBgn0262607	CG43133	19.1872	126.816	2.732169889	6.36447E-06
366	FBgn0262721	CG43165	26.3987	2.19228	-3.621310432	0.000792629
367	FBgn0263321	CG43402	29.5318	100.204	1.772329241	0.00018
368	FBgn0263345	asRNA:CR43426	30.1033	7.84035	-1.927029309	0.013505775
369	FBgn0263376	lncRNA:CR43428	9.01654	42.9929	2.258701452	0.000587399
370	FBgn0264343	CG43799	7.4434	43.7389	2.535323384	0.000550599
371	FBgn0264386	Ca-alpha1T	22617.3	49885.6	1.141206351	4.77536E-29
372	FBgn0264390	lncRNA:CR43838	13.9426	42.3228	1.60097865	0.009762325
373	FBgn0264567	lncRNA:CR43939	12.233	0	-5.963109886	0.003949075
374	FBgn0264745	CR44003	58.9006	9.59116	-2.622975386	1.15152E-06
375	FBgn0264748	CG44006	35.3798	78.0688	1.136664832	0.01531523
376	FBgn0264789	asRNA:CR44019	59.3967	26.2157	-1.174600198	0.023726163
377	FBgn0264834	lncRNA:CR44042	127.196	61.5202	-1.042218284	0.002083304
378	FBgn0265046	CG44163	38.3875	8.6473	-2.165672905	0.001109941
379	FBgn0265180	CG44245	3080.19	6263.87	1.02407358	5.15259E-16
380	FBgn0265185	CG44250	37.0737	87.9343	1.249285949	0.008581037
381	FBgn0265533	CR44383	33.4564	1.00477	-4.983310719	0.000204522
382	FBgn0265668	CG44475	0	21.7702	6.954143141	5.8468E-05
383	FBgn0265813	lncRNA:CR44602	26.5894	69.4064	1.382168742	0.001858259
384	FBgn0265819	lncRNA:CR44608	89.0159	39.8109	-1.160786691	0.002001278
385	FBgn0265856	lncRNA:CR44645	39.3468	85.3759	1.120989033	0.029999515
386	FBgn0265938	lncRNA:CR44725	0.681076	11.6007	4.15972066	0.032374112
387	FBgn0266042	asRNA:CR44807	42.2689	13.2297	-1.673386207	0.004043713
388	FBgn0266633	asRNA:CR45140	3.11397	33.2799	3.396190757	8.77667E-05
389	FBgn0266684	lncRNA:CR45174	12.4458	68.006	2.434197529	1.05107E-05
390	FBgn0266869	asRNA:CR45330	89.2895	16.244	-2.445642072	9.09961E-08
391	FBgn0266916	Cenp-C	46.8647	98.9259	1.081882749	0.003319588
392	FBgn0266935	lncRNA:CR45386	3.6233	33.7213	3.243261002	0.000490766
393	FBgn0267026	CR45470	18.2812	76.8203	2.078091187	1.59907E-05
394	FBgn0267430	Pzl	45.369	118.23	1.386782235	8.66664E-05
395	FBgn0267667	lncRNA:CR46005	42.7631	11.5398	-1.881612818	0.002273454
396	FBgn0267798	lncRNA:CR46123	91.9236	0	-8.87836027	7.73946E-08
397	FBgn0267934	lncRNA:CR46214	23.2254	0	-6.890765559	0.000187555
398	FBgn0283462	IMPPP	97.7449	265.323	1.441341491	0.002780013
399	FBgn0283480	Alp2	1.2898	12.2249	3.256656085	0.03019546
400	FBgn0286723	hll	32.3153	96.2011	1.577936147	0.000860855

6) List of DEGs in *Drosophila* head of *DmAnkrd11* $^{\Delta/\Delta}$ *vs* *WT*

	FlyBase ID	Gene Symbol	*WT* (nomarlized rds)	*DmAnkrd11* $^{\Delta/\Delta}$ (nomarlized rds)	log2FC (*DmAnkrd11* $^{\Delta/\Delta}$ *vs* *WT*)	padj
1	FBgn0000052	Pfas	4655.27	2137.01	-1.122221038	9.70475E-27
2	FBgn0000055	Adh	108.92	30.3627	-1.848297064	3.04514E-05
3	FBgn0000405	CycB	55.6551	12.2646	-2.17640466	7.2886E-05
4	FBgn0000473	Cyp6a2	6696.16	1169.06	-2.517926989	1.674E-100
5	FBgn0001187	Hex-C	4572.55	2246.17	-1.024946185	3.19299E-34
6	FBgn0001208	Hn	5211.32	2330.53	-1.161214829	8.54392E-70
7	FBgn0001217	Hsc70-2	11.6445	41.2671	1.819508539	0.025764404
8	FBgn0001229	Hsp67Bc	230.706	661.27	1.517270456	1.14449E-19
9	FBgn0001230	Hsp68	2610.25	993.769	-1.393213092	3.80842E-20
10	FBgn0001253	ImpE1	238.716	98.108	-1.2809013	0.001646922
11	FBgn0001256	ImpL1	106.438	24.3821	-2.127561033	2.56363E-08
12	FBgn0002565	Lsp2	360.083	163.991	-1.133643112	1.53867E-05
13	FBgn0003137	Ppn	4520.07	1858.15	-1.281366563	7.40501E-31
14	FBgn0003227	rec	223.051	84.8823	-1.398401936	3.40396E-09
15	FBgn0003483	spn-E	84.1952	35.1125	-1.261376708	0.001527581
16	FBgn0004782	Ccp84Ab	3176.71	7769.9	1.290650839	3.8525E-91
17	FBgn0004783	Ccp84Aa	339.84	743.483	1.131540095	2.49431E-08
18	FBgn0004895	FoxL1	0.658196	11.6581	4.129947045	0.040546426
19	FBgn0005391	Yp2	12338.7	5903.79	-1.063401273	0.000989122
20	FBgn0005638	slbo	13.8434	43.7802	1.653952448	0.016895076
21	FBgn0010019	Cyp4g1	11.7078	865.296	6.209048884	5.97079E-16
22	FBgn0010038	GstD2	107.644	244.851	1.18818914	7.50341E-07
23	FBgn0010041	GstD5	31.8821	8.13033	-1.990678693	0.030941071
24	FBgn0010388	Dro	24.8933	61.8873	1.314273935	0.02397799
25	FBgn0011559	Acp36DE	0.302424	41.6841	6.857328481	0.036638073
26	FBgn0011723	byn	34.7936	77.9138	1.160956008	0.016315727
27	FBgn0011768	Fdh	2770	1134.88	-1.286065095	6.75489E-33
28	FBgn0013277	Hsp70Ba	179.884	63.7069	-1.495433276	5.82642E-07
29	FBgn0013672	mt:ATPase6	16179.2	33111.3	1.033208701	0.009979842
30	FBgn0013680	mt:ND2	784.033	2012.41	1.360363037	0.004513999
31	FBgn0013812	Dhc93AB	283.374	1665.03	2.551073702	2.62226E-50
32	FBgn0013813	Dhc98D	376.284	149.37	-1.336872116	9.92869E-13
33	FBgn0014019	Rh5	7055.3	3368.12	-1.066360044	2.19326E-48
34	FBgn0014033	Sr-CI	230.279	98.5161	-1.224336249	1.41128E-07
35	FBgn0015035	Cyp4e3	1190.22	264.39	-2.172806302	1.98388E-31
36	FBgn0015037	Cyp4p1	980.048	2136.47	1.123347645	2.77939E-24
37	FBgn0015376	cutlet	74.8294	319.852	2.096962764	2.92382E-21
38	FBgn0015570	alpha-Est2	1257.45	599.624	-1.066506357	1.19831E-15
39	FBgn0015584	Acp53Ea	0.658196	13.6606	4.356397277	0.040732062
40	FBgn0015714	Cyp6a17	905.8	14.276	-5.999608911	5.98764E-09
41	FBgn0016675	Lectin-galC1	4.91112	40.2257	3.026723931	0.001080986
42	FBgn0017482	T3dh	364.945	116.137	-1.647597995	5.00536E-18
43	FBgn0019828	dj	0.302424	13.1231	5.184601698	0.024044222
44	FBgn0020509	Acp62F	0.347101	16.1915	5.488256308	0.008988481
45	FBgn0020513	Paics	15174.6	7014.66	-1.112948243	1.10753E-22
46	FBgn0020642	Lcp65Ac	929.707	423.859	-1.136033036	1.5427E-09
47	FBgn0021765	scu	3591.48	1710.81	-1.069155907	2.30713E-34
48	FBgn0022355	Tsf1	14344.6	6657.52	-1.107318943	1.63284E-29
49	FBgn0024920	Ts	138.494	59.7978	-1.208389346	0.000233953
50	FBgn0025454	Cyp6g1	149.987	318.735	1.086089956	2.38051E-07
51	FBgn0026314	Ugt35B1	3389.91	1263.84	-1.424695721	1.45048E-23
52	FBgn0026315	Ugt35A1	48.3359	122.751	1.343633116	0.000164507
53	FBgn0026602	Ady43A	905.011	2302.25	1.346961166	8.68552E-36
54	FBgn0027584	CG4757	411.174	181.768	-1.176057162	6.30386E-11
55	FBgn0028396	TotA	428.039	154.474	-1.468685475	1.77519E-12
56	FBgn0028542	NimB4	296.083	107.088	-1.464330881	1.15926E-10
57	FBgn0028936	NimB5	430.204	210.059	-1.032551099	1.13431E-08
58	FBgn0028939	NimC2	190.184	86.7198	-1.13534196	1.72064E-05
59	FBgn0028990	Spn27A	6370.07	2849.05	-1.160965088	2.26976E-55
60	FBgn0029147	NtR	1502.2	639.176	-1.232220321	3.52694E-28
61	FBgn0029167	Hml	858.992	337.248	-1.348366814	4.6208E-24
62	FBgn0029769	frma	501.372	235.706	-1.084458368	1.26281E-08
63	FBgn0029831	CG5966	2985.31	1078.08	-1.468578671	9.03035E-27
64	FBgn0030839	CG5613	469.854	126.036	-1.896840662	1.11078E-26
65	FBgn0031249	CG11911	0.355772	10.9557	4.935964479	0.032207173
66	FBgn0031305	Iris	60.6643	20.3949	-1.577111379	0.001500151

67	FBgn0031389	CG4259	203.188	87.6987	-1.20868187	8.53156E-06
68	FBgn0031484	CG3165	402.295	875.834	1.11976283	6.37045E-06
69	FBgn0031522	CG3285	0.355772	13.3305	5.218859162	0.015195225
70	FBgn0031549	Spindly	47.2473	16.1826	-1.558664845	0.011448484
71	FBgn0031559	CG3513	362.972	141.663	-1.354048311	9.17615E-08
72	FBgn0031688	Cyp28d2	36.011	110.859	1.626967168	6.92755E-05
73	FBgn0031690	CG7742	36.1213	93.2557	1.366172424	0.004949176
74	FBgn0031746	CG9029	2.02794	40.0273	4.315147445	2.11488E-05
75	FBgn0031775	CG9150	31.4506	149.49	2.251620936	2.51878E-10
76	FBgn0031835	CG11319	2794.17	7009.41	1.327460194	2.9874E-61
77	FBgn0031853	TTLL3B	104.049	42.3241	-1.301428699	0.00043454
78	FBgn0031925	Cyp4d21	63.5471	130.335	1.031262214	0.00597046
79	FBgn0031937	CG13795	735.616	363.978	-1.012840852	3.29666E-13
80	FBgn0031957	TwdlE	101.75	26.7569	-1.923855171	7.57629E-05
81	FBgn0031976	CG7367	51.4022	9.95887	-2.339786917	0.000525511
82	FBgn0032161	CG4594	78.7169	37.9557	-1.055270395	0.016962003
83	FBgn0032535	Ance-2	40.2216	16.0265	-1.334951524	0.043572112
84	FBgn0033027	TpnC4	6.21017	51.3486	3.036003578	0.012370498
85	FBgn0033093	CG3270	2452.08	1202.29	-1.027043349	4.92019E-25
86	FBgn0033174	CG11125	47.8892	19.086	-1.323042467	0.029121192
87	FBgn0033204	CG2065	213.922	561.824	1.393744354	5.54131E-12
88	FBgn0033234	MFS12	178.039	87.3687	-1.021615912	0.006261944
89	FBgn0033275	CG14756	246.8	69.2282	-1.831762351	2.501E-14
90	FBgn0033312	CG8642	118.091	42.9998	-1.448788099	3.48263E-05
91	FBgn0033628	CG13203	51.3765	114.023	1.147910719	0.001675423
92	FBgn0033782	sug	628.374	172.485	-1.862520982	1.57593E-23
93	FBgn0033788	CG13323	0	11.7183	5.994848356	0.013291032
94	FBgn0033868	S-Lap7	7.73995	49.8144	2.684791223	0.021280671
95	FBgn0033879	Echs1	2862.78	1385.68	-1.045731901	1.46677E-35
96	FBgn0033942	Cpr51A	566.704	199.92	-1.508789625	8.92663E-15
97	FBgn0033949	Had2	407.631	165.691	-1.299660739	1.60113E-09
98	FBgn0033978	Cyp6a23	502.073	1994.28	1.992257374	1.06183E-53
99	FBgn0034126	jtb	246.38	68.4477	-1.839306582	9.92819E-13
100	FBgn0034127	CG7848	67.1816	21.617	-1.633021558	0.001955031
101	FBgn0034131	CG15712	5.03516	25.613	2.348446291	0.01414227
102	FBgn0034132	S-Lap8	0.649525	37.3067	5.813671766	0.001411723
103	FBgn0034151	CG15617	1.76152	235.681	7.07846653	0.008484767
104	FBgn0034219	mthl4	34.5455	77.2024	1.158540983	0.011303238
105	FBgn0034276	Sardh	200.051	459.219	1.201735175	9.02945E-09
106	FBgn0034295	CG10911	0	15.3241	6.380419521	0.001547502
107	FBgn0034328	IM23	2010.43	947.168	-1.086973784	4.85215E-14
108	FBgn0034356	Pepck2	1406.99	635.684	-1.146834511	1.18003E-24
109	FBgn0034629	Acox57D-d	371.179	157.984	-1.227677912	2.82622E-10
110	FBgn0034796	CG3700	251.455	615.998	1.292012419	3.29785E-19
111	FBgn0035186	CG13912	291.178	143.744	-1.016300606	4.64996E-06
112	FBgn0035328	yellow-g2	30.8521	2.99034	-3.369059088	0.000504493
113	FBgn0035358	CG14949	16.903	62.001	1.87424933	0.032769271
114	FBgn0035545	CG12607	28.2624	70.3632	1.322660514	0.018747974
115	FBgn0035546	CG11345	243.164	510.402	1.068476008	5.56404E-12
116	FBgn0035550	CG11349	66.9855	19.3112	-1.783310058	0.002567134
117	FBgn0035551	CG7465	8.87029	45.999	2.365522387	0.000908481
118	FBgn0035575	CG7509	261.168	101.402	-1.362510385	0.000611629
119	FBgn0035581	Dnah3	20.5768	86.8067	2.071469453	7.25558E-06
120	FBgn0035582	CG13705	97.6749	35.0344	-1.47583531	7.05952E-05
121	FBgn0035623	mthl2	111.821	42.2371	-1.401024476	0.000740398
122	FBgn0035727	CG10063	3.97651	37.106	3.217171675	7.87672E-05
123	FBgn0035777	CG8563	42.5436	8.58969	-2.322529504	0.000466971
124	FBgn0035781	CG8560	14.8054	89.7703	2.594262032	4.51301E-05
125	FBgn0035858	CG13674	14.607	48.53	1.729975524	0.005521261
126	FBgn0035861	CG7213	3.83413	62.6142	4.068819578	6.31202E-07
127	FBgn0035915	S-Lap1	50.4229	143.199	1.500779594	0.000576304
128	FBgn0036024	CG18180	51.1291	18.5664	-1.477215715	0.041255861
129	FBgn0036031	CG6761	33.7964	83.6871	1.298832189	0.036335754
130	FBgn0036093	CG14154	8.28278	30.4408	1.872083069	0.023016364
131	FBgn0036121	CG6310	68.9214	27.5976	-1.317776837	0.004080514
132	FBgn0036143	CG14142	111.498	335.758	1.586900835	4.56044E-14
133	FBgn0036157	CG7560	610.217	299.014	-1.02536595	5.17272E-09
134	FBgn0036225	CG5883	250.363	77.2716	-1.691560389	5.95674E-14
135	FBgn0036563	CG13075	7.2428	36.4906	2.32721877	0.021353184
136	FBgn0036587	CG4950	1139.66	525.118	-1.115713296	9.47869E-20
137	FBgn0036597	CG4962	4300.15	9175.61	1.093312627	0.00027395
138	FBgn0036612	CG4998	92.7975	37.3313	-1.310652895	0.000858569

139	FBgn0036619	Cpr72Ec	2183.31	7217.2	1.724717891	4.27493E-66
140	FBgn0036717	CG13731	20.185	53.8952	1.413484538	0.014286948
141	FBgn0036757	Ir75a	6.28086	58.801	3.220019193	2.42957E-07
142	FBgn0036962	CG17122	13.6824	37.0369	1.434403082	0.046832712
143	FBgn0037127	CG14566	368.061	149.229	-1.296034301	1.28674E-08
144	FBgn0037128	CG14572	3137.89	1066.73	-1.556074822	3.79818E-06
145	FBgn0037151	CG7130	359.921	143.269	-1.334453416	1.04698E-06
146	FBgn0037181	CG11370	75.0762	19.086	-1.965863486	1.74422E-05
147	FBgn0037416	Osi9	77.5673	234.484	1.591390728	3.79818E-06
148	FBgn0037488	CG14607	129.07	48.8511	-1.411231243	6.79535E-05
149	FBgn0037547	CG7910	4.74108	35.7882	2.889153703	0.007419693
150	FBgn0037552	CG7800	257.234	69.4623	-1.890322954	4.18658E-16
151	FBgn0037684	Srr	367.927	135.604	-1.435639309	1.34617E-12
152	FBgn0037726	CG9492	120.954	471.492	1.961197132	0.017234672
153	FBgn0037761	CG8534	37.1343	8.50273	-2.096160502	0.013242239
154	FBgn0037762	eloF	16.5989	2.45292	-2.751402094	0.039614203
155	FBgn0037763	CG16904	0.302424	11.5711	5.013354004	0.022619268
156	FBgn0037807	CG6293	149.75	300.229	1.004606948	1.69534E-06
157	FBgn0037811	CG12592	29.6781	5.13107	-2.489008838	0.012345417
158	FBgn0037826	CG14689	0.96062	13.4086	3.772612726	0.039801169
159	FBgn0037879	scpr-C	10.204	47.8967	2.227809715	0.001600758
160	FBgn0037895	CG6723	4175.15	1883.56	-1.148300088	3.78204E-05
161	FBgn0037940	CG14720	10.6218	0	-5.861697648	0.007204213
162	FBgn0037973	CG18547	542.358	230.854	-1.232803004	1.06964E-16
163	FBgn0037974	CG12224	691.196	164.261	-2.079033921	8.07835E-31
164	FBgn0037975	CG3397	412.554	43.3989	-3.254573472	4.51453E-47
165	FBgn0037992	CG4702	34.3388	112.819	1.71678703	0.000266037
166	FBgn0038001	CG17404	217.003	85.1945	-1.35453557	1.02457E-07
167	FBgn0038011	CG4066	0.996626	12.4988	3.643328288	0.043772039
168	FBgn0038017	CG4115	4674.18	2309.93	-1.016108844	1.00885E-32
169	FBgn0038056	CG5961	310	651.918	1.07109042	2.4311E-14
170	FBgn0038083	Ugt37A3	49.3342	298.044	2.598929435	6.74455E-18
171	FBgn0038252	BigH1	1.31639	14.5704	3.455149809	0.032428706
172	FBgn0038290	CG6912	5722.9	2296.13	-1.318074159	1.04083E-82
173	FBgn0038301	CG6654	1658.43	818.641	-1.019814375	6.13766E-25
174	FBgn0038337	CG6125	1092.15	2655.12	1.282355468	2.11961E-28
175	FBgn0038358	Ttc26	178.224	68.2493	-1.391983281	8.73567E-06
176	FBgn0038397	CG10185	115.661	336.349	1.534740597	4.9547E-08
177	FBgn0038621	CG10864	55.0069	127.924	1.218197303	0.001451427
178	FBgn0038645	CG7714	589.133	221.399	-1.416293752	2.04665E-12
179	FBgn0038658	CG14292	0.649525	88.7535	7.067038796	3.02277E-06
180	FBgn0038742	Arc42	612.152	299.665	-1.027832002	2.07533E-08
181	FBgn0038789	Ir92a	1.36974	15.7143	3.544296891	0.027336863
182	FBgn0038865	cDIP	189.903	388.162	1.029704074	1.48363E-08
183	FBgn0038866	CG5810	12.3843	80.1236	2.679178634	3.16287E-06
184	FBgn0038943	CG5391	10.5788	35.719	1.769210419	0.038287898
185	FBgn0039009	CG13842	7.03676	42.4111	2.565339344	0.003159967
186	FBgn0039052	CG6733	2.99855	19.086	2.671730821	0.033367231
187	FBgn0039311	CG10513	251.982	823.837	1.708167569	2.14692E-22
188	FBgn0039312	CG10514	45.9783	264.057	2.523254186	2.24767E-17
189	FBgn0039415	CG6142	697.459	293.287	-1.25228188	1.86194E-17
190	FBgn0039429	CG14238	8.24678	30.8132	1.898303803	0.022114034
191	FBgn0039452	CG14245	13.4867	91.5877	2.76964055	0.005231177
192	FBgn0039510	CG3339	55.6275	208.329	1.898615211	5.85258E-06
193	FBgn0039537	CG5590	3540.46	1104.35	-1.681875315	1.03569E-61
194	FBgn0039568	CG4815	3.22029	18.9299	2.536000625	0.043291243
195	FBgn0039755	CG15531	1.75285	28.9668	4.094767518	0.000845479
196	FBgn0039972	Marf1	27.5335	77.9562	1.507424481	0.001726417
197	FBgn0287184	FASN3	72.315	14.8736	-2.280361222	1.96218E-05
198	FBgn0040099	lectin-28C	151.558	63.3947	-1.252107145	2.30403E-05
199	FBgn0040102	lectin-24Db	206.285	87.7166	-1.237776379	1.37082E-06
200	FBgn0040251	Ugt302K1	24.3846	145.305	2.565850181	3.33845E-12
201	FBgn0040255	Ugt35E2	52.2044	302.524	2.535441107	2.99215E-19
202	FBgn0040256	Ugt35C1	804.298	388.465	-1.049418268	2.23818E-16
203	FBgn0040257	Ugt302E1	3.71876	87.1435	4.567317381	7.81554E-12
204	FBgn0040699	CG15024	75.3163	160.473	1.090238275	0.000125611
205	FBgn0040827	CG13315	5954.61	2746.96	-1.115589856	8.26105E-47
206	FBgn0041194	Prat2	2565.2	1140.16	-1.168280099	1.77781E-33
207	FBgn0041247	Gr28a	18.4681	3.52775	-2.405280593	0.033027288
208	FBgn0041579	AttC	33.3332	77.9227	1.220897058	0.027040319
209	FBgn0041581	AttB	36.9749	144.265	1.957597075	1.24518E-05
210	FBgn0042178	Apl	40.6197	184.36	2.187598856	7.02992E-12

211	FBgn0043783	CG32444	2805.65	949.295	-1.563026898	1.97129E-52
212	FBgn0045770	S-Lap3	0.347101	29.4016	6.350817041	0.002924487
213	FBgn0046253	CG3502	36.6252	134.472	1.8695698	0.00020564
214	FBgn0050076	CG30076	16.1278	1.53419	-3.428309203	0.019733854
215	FBgn0050098	CG30098	48.7621	10.7304	-2.173912589	0.000342723
216	FBgn0050101	Vajk4	1685.65	691.519	-1.284637563	2.65029E-32
217	FBgn0050395	CG30395	166.429	444.691	1.415749154	5.41841E-11
218	FBgn0050489	Cyp12d1-p	350.347	125.099	-1.480283958	1.12965E-11
219	FBgn0051220	CG31220	43.1644	0.996779	-5.443505544	2.39113E-05
220	FBgn0051268	CG31268	1.70817	32.7198	4.28768579	0.000139391
221	FBgn0051288	CG31288	57.1486	121.382	1.080965289	0.004513999
222	FBgn0051300	CG31300	35.3618	98.5852	1.487347691	0.003270026
223	FBgn0051347	CG31347	48.5927	19.4673	-1.308167731	0.029326065
224	FBgn0051380	CG31380	13.2649	0.537413	-4.725814306	0.031448306
225	FBgn0051453	pch2	63.4327	130.455	1.047618317	0.044785019
226	FBgn0051740	CG31740	0.302424	26.3935	6.194312674	0.000531691
227	FBgn0051781	lncRNA:CR31781	7589.55	3430.43	-1.146172833	1.62581E-51
228	FBgn0052069	CG32069	120.262	58.888	-1.03184105	0.023791237
229	FBgn0052195	CG32195	232.972	492.411	1.080234171	0.000153017
230	FBgn0052207	hpRNA:CR32207	81.7569	10.3491	-2.977869153	8.73057E-10
231	FBgn0052656	Muc11A	3.46102	40.5112	3.572706217	0.006527455
232	FBgn0053192	MtnD	117.798	36.8206	-1.691912903	0.000342172
233	FBgn0053348	CheB42a	5.92509	27.2163	2.19122931	0.014593226
234	FBgn0053349	ppk25	9.67986	44.777	2.209371151	0.000929045
235	FBgn0053465	CG33465	81.8322	22.3885	-1.871298471	2.36068E-05
236	FBgn0053503	Cyp12d1-d	59.8347	16.9363	-1.805924622	0.003917559
237	FBgn0053926	CG33926	27.5778	4.36843	-2.64158226	0.003120389
238	FBgn0058160	CG40160	757.321	1875.52	1.308696504	9.87405E-31
239	FBgn0058470	CG40470	310.667	73.9445	-2.081714479	2.05024E-11
240	FBgn0062928	hpRNA:CR33940	189.784	70.2339	-1.437796975	2.05936E-08
241	FBgn0083945	CG34109	1.26304	20.2656	3.963186832	0.048399685
242	FBgn0085209	CG34180	25.1781	61.9564	1.294164933	0.025175305
243	FBgn0085228	CG34199	166.594	38.6313	-2.101519043	2.14299E-11
244	FBgn0085244	CG34215	118.736	52.4391	-1.181055527	0.001679933
245	FBgn0085310	CG34281	118.549	45.0803	-1.392264142	0.000463685
246	FBgn0085466	CG34437	74.2809	11.3459	-2.706021228	6.43252E-07
247	FBgn0085486	CG34457	6.03178	27.97	2.221874646	0.019178934
248	FBgn0086265	psd	0.649525	13.7297	4.369878236	0.02178552
249	FBgn0086356	tum	40.0245	16.0956	-1.304881968	0.048410554
250	FBgn0086691	UK114	2605.22	1101.19	-1.241498126	8.73112E-28
251	FBgn0243514	eater	126.79	53.2284	-1.261816582	0.001175799
252	FBgn0250816	AGO3	48.4143	14.111	-1.787594303	0.002999821
253	FBgn0259112	asRNA:CR42254	57.9268	25.526	-1.176241066	0.031449212
254	FBgn0259739	CG42393	0.649525	51.139	6.264350486	1.82508E-06
255	FBgn0259795	loopin-1	0.347101	52.1982	7.181906088	0.014544059
256	FBgn0259896	NimC1	513.59	205.82	-1.315386805	5.0368E-10
257	FBgn0260011	NimC4	1534.1	432.488	-1.826866251	1.17907E-61
258	FBgn0260463	Unc-115b	31.377	107.478	1.77212226	4.65768E-06
259	FBgn0260482	CG32599	141.054	51.2081	-1.457414175	1.35617E-06
260	FBgn0260874	Ir76a	17.0023	107.175	2.668636447	1.68233E-08
261	FBgn0260954	CG42586	23.5239	4.60258	-2.370836905	0.030263487
262	FBgn0260955	CG42587	8.15875	0	-5.483983682	0.031350919
263	FBgn0261402	Ir75b	7.36652	35.8662	2.268688665	0.036874673
264	FBgn0261628	CG42711	142.708	67.616	-1.075617735	0.000254263
265	FBgn0261831	CG42763	46.7302	104.791	1.164248074	0.005988109
266	FBgn0262146	MtnE	78.1514	35.806	-1.128254937	0.038512584
267	FBgn0262535	CG43089	0	35.4336	7.583049512	1.40282E-06
268	FBgn0262574	CG43114	39.0225	8.44252	-2.235367813	0.026047908
269	FBgn0262679	CG43149	14.7244	68.2582	2.201062752	0.033920583
270	FBgn0262945	lncRNA:CR43264	18.2824	54.7448	1.579302276	0.004513999
271	FBgn0262983	CG43291	133.169	52.9943	-1.338908436	0.000118364
272	FBgn0262987	CG43295	1.36107	13.1834	3.296647977	0.039614203
273	FBgn0263040	CG43335	17.8273	3.14643	-2.547446894	0.046146299
274	FBgn0263041	CG43336	12.3473	0.537413	-4.626331911	0.037690786
275	FBgn0263083	CG43351	71.2491	34.1938	-1.059774462	0.022551552
276	FBgn0263608	l(3)72Dr	113.975	49.7007	-1.20631107	0.005039766
277	FBgn0263802	lncRNA:CR43605	7.77596	29.8075	1.951458308	0.043158069
278	FBgn0263974	qin	85.8584	40.5558	-1.083013759	0.00781562
279	FBgn0264567	lncRNA:CR43939	12.8098	0	-6.128589998	0.004009029
280	FBgn0264748	CG44006	37.0887	115.653	1.631590425	0.000396301
281	FBgn0264897	CG44088	5.16887	28.057	2.421419746	0.013413772
282	FBgn0265318	asRNA:CR44291	0.355772	12.4207	5.111089062	0.01436356

283	FBgn0265541	CR44391	186.423	68.0842	-1.455121197	9.35738E-09
284	FBgn0265718	lncRNA:CR44525	23.0508	108.787	2.231295176	5.31574E-08
285	FBgn0266084	Fhos	2863.75	6697.3	1.226034729	1.26228E-47
286	FBgn0266148	lncRNA:CR44854	15.4005	46.9177	1.610094847	0.010892108
287	FBgn0266227	lncRNA:CR44922	2542.84	865.149	-1.554156757	3.34763E-72
288	FBgn0266432	CG45062	0.996626	24.2171	4.602084677	0.001171367
289	FBgn0266570	NO66	1673.59	805.897	-1.053172497	1.48785E-28
290	FBgn0266590	lncRNA:CR45115	465.823	989.295	1.085517915	7.01769E-20
291	FBgn0266633	asRNA:CR45140	3.26497	19.8665	2.585389315	0.033115704
292	FBgn0266733	asRNA:CR45206	27.7219	5.75545	-2.30438657	0.017531848
293	FBgn0266963	lncRNA:CR45414	8.24678	34.2629	2.050874551	0.008464851
294	FBgn0267026	CR45470	19.164	102.356	2.421869841	2.20357E-08
295	FBgn0267160	asRNA:CR45600	766.495	314.784	-1.287240674	8.9867E-15
296	FBgn0267240	lncRNA:CR45680	2.67746	81.2074	4.925395094	2.48094E-11
297	FBgn0267270	lncRNA:CR45706	8.01636	28.3692	1.823098143	0.042022724
298	FBgn0267327	Acp33A	0.302424	11.9703	5.052177955	0.027610416
299	FBgn0267430	Pzl	47.533	13.6516	-1.809841897	0.004338971
300	FBgn0267798	lncRNA:CR46123	96.3655	5.13999	-4.27030016	1.01558E-11
301	FBgn0267934	lncRNA:CR46214	24.3302	0	-7.056452581	0.000148944
302	FBgn0283461	Drs	995.073	445.467	-1.16092511	2.12037E-07
303	FBgn0284435	tyn	621.994	1438.02	1.209438926	1.94737E-22
304	FBgn0286030	lncRNA:CR46350	0	70.9274	8.585811837	8.54758E-10
305	FBgn0286031	lncRNA:CR46351	8.00605	0	-5.460527233	0.029306614
306	FBgn0286198	LKRSDH	2112.54	1046.53	-1.012287638	5.92619E-19

7) List of DEGs in *Drosophila* adults of *Dm65k*$^{\Delta/\Delta}$ *vs WT*

	FlyBase ID	Gene Symbol	*WT* (nomarlized rds)	*Dm65K*$^{\Delta/\Delta}$ (nomarlized rds)	log2FC (*Dm65k*$^{\Delta/\Delta}$ *vs WT*)	padj
1	FBgn0000055	Adh	92.8433	319.989	1.768501788	2.38E-21
2	FBgn0000071	Ama	185.686	469.881	1.342062908	3.43E-19
3	FBgn0000166	bcd	50.1836	24.248	-1.064195655	1.19E-02
4	FBgn0000277	CecA2	5.37573	36.9544	2.816005697	1.84E-04
5	FBgn0000351	cort	26.0433	9.43494	-1.47519634	4.58E-02
6	FBgn0000473	Cyp6a2	2228.55	893.332	-1.315806096	1.91E-36
7	FBgn0000564	Eh	107.531	49.7338	-1.103689028	1.47E-04
8	FBgn0000639	Fbp1	15565.5	78420.2	2.332995177	1.70E-134
9	FBgn0000927	fs(1)Ya	174.298	60.4182	-1.536242951	2.48E-11
10	FBgn0001077	ftz	22.0932	7.20397	-1.624599593	1.82E-02
11	FBgn0001120	gnu	101.14	23.6503	-2.113326066	1.92E-09
12	FBgn0001224	Hsp23	2041.26	4085.22	1.000659	5.13E-34
13	FBgn0001225	Hsp26	1978.53	956.676	-1.048937057	2.72E-04
14	FBgn0001257	ImpL2	13750.3	6717.71	-1.032996286	5.95E-34
15	FBgn0002531	Lcp1	12.9556	141.419	3.435618127	6.36E-27
16	FBgn0002535	Lcp4	2.51821	19.6114	2.885777883	8.92E-04
17	FBgn0002565	Lsp2	1858	6256.7	1.750377495	1.83E-99
18	FBgn0002869	MtnB	142.976	35.836	-2.000725533	4.21E-12
19	FBgn0002926	ndl	136.785	21.682	-2.673255719	2.97E-16
20	FBgn0002962	nos	120.246	55.2888	-1.136058341	9.41E-05
21	FBgn0003015	osk	453.238	136.09	-1.749156038	4.65E-21
22	FBgn0003023	otu	307.428	102.633	-1.593669288	1.89E-13
23	FBgn0003082	phr	1275.36	65.2906	-4.274031524	8.35E-192
24	FBgn0003089	pip	138.6	370.772	1.425158542	1.26E-15
25	FBgn0003114	plu	18.0582	1.48514	-3.644999756	7.84E-04
26	FBgn0003257	r-l	772.223	360.539	-1.098090598	8.49E-23
27	FBgn0003358	Jon99Ci	323.562	40.8539	-2.987150208	1.20E-03
28	FBgn0003450	snk	18.2154	5.31062	-1.772068332	2.82E-02
29	FBgn0003525	stg	471.999	111.854	-2.086967985	2.78E-29
30	FBgn0003655	swa	180.123	64.096	-1.503240424	4.63E-11
31	FBgn0003935	snRNA:U5:34A	0.762966	15.0736	4.101004499	1.84E-03
32	FBgn0003980	Vm26Ab	18.4314	0.668296	-4.719835461	1.08E-03
33	FBgn0004034	y	80.0123	587.386	2.872339472	1.11E-07
34	FBgn0004047	Yp3	4004.97	1829.2	-1.130060711	4.55E-32
35	FBgn0004400	rhi	57.9327	25.2478	-1.225697663	8.39E-03
36	FBgn0004649	yl	3417.99	763.924	-2.164683013	1.81E-87
37	FBgn0004779	Ccp84Ae	21.057	6.38799	-1.725166645	2.13E-02
38	FBgn0004783	Ccp84Aa	200.02	41.3458	-2.271285258	4.78E-05
39	FBgn0005390	fs(1)M3	526.51	135.165	-1.972231157	8.23E-34
40	FBgn0010041	GstD5	426.367	150.316	-1.495189179	9.29E-14
41	FBgn0010052	Jhe	636.308	1392.43	1.132001229	1.22E-25
42	FBgn0010097	gammaTub37C	54.6079	27.2597	-1.012044025	1.23E-02
43	FBgn0010241	Mdr50	358.032	133.658	-1.429155138	4.35E-11
44	FBgn0010317	CycJ	85.8543	42.562	-1.019525064	3.02E-03
45	FBgn0010388	Dro	31.0797	91.415	1.547662618	1.38E-05
46	FBgn0010423	TpnC47D	2473.72	1234.53	-1.001551382	9.60E-37
47	FBgn0010431	mtrm	95.6032	44.7539	-1.097750481	4.61E-04
48	FBgn0011670	Mst57Dc	596.549	289.839	-1.043460297	1.12E-02
49	FBgn0011761	dhd	835.439	168.541	-2.313074926	7.55E-15
50	FBgn0011822	Pgcl	110.118	224.935	1.042178642	1.40E-07
51	FBgn0012042	AttA	14.2401	542.594	5.248725147	9.43E-12
52	FBgn0013275	Hsp70Aa	255.13	23.5819	-3.439312419	2.33E-07
53	FBgn0013276	Hsp70Ab	760.038	43.3802	-4.130544073	1.53E-12
54	FBgn0013277	Hsp70Ba	199.912	15.1132	-3.739324087	1.07E-09
55	FBgn0013278	Hsp70Bb	348.225	51.733	-2.753093999	6.47E-06
56	FBgn0013279	Hsp70Bc	176.421	49.4433	-1.832093083	2.13E-03
57	FBgn0013308	Odc2	91.4613	5.09019	-4.118824008	2.21E-15
58	FBgn0014903	CG14630	5.68032	20.7995	1.907037978	1.15E-02
59	FBgn0015035	Cyp4e3	885.328	2318.15	1.389090849	7.86E-03
60	FBgn0015586	Acp76A	1499.04	416.504	-1.849293906	3.33E-13
61	FBgn0016053	pgc	130.564	44.3016	-1.570475108	1.27E-07
62	FBgn0016675	Lectin-galC1	1409.54	192.316	-2.867794796	1.98E-108
63	FBgn0020248	stet	209.97	72.6291	-1.543832079	5.45E-12

64	FBgn0020506	Amyrel	7.58288	43.4041	2.453915344	7.873E-05
65	FBgn0024177	zpg	331.817	104.23	-1.683584973	1.176E-18
66	FBgn0024897	b6	1946.68	775.912	-1.324774124	4.942E-33
67	FBgn0024984	CG3457	29.9939	6.90774	-2.082622554	0.0014796
68	FBgn0025456	CREG	1136.13	2728.49	1.262753049	2.935E-55
69	FBgn0025686	Amnionless	148.969	37.3582	-2.00590306	3.897E-15
70	FBgn0025709	CNT2	705.522	345.195	-1.035760064	4.415E-14
71	FBgn0025837	CG17636	56.5005	152.822	1.411203776	3.123E-07
72	FBgn0026755	Ugt37B1	41.3475	90.0944	1.11735082	0.0026812
73	FBgn0027562	CG10345	5638.46	2805.98	-1.00631685	1.208E-21
74	FBgn0027584	CG4757	375.473	838.255	1.158513223	5.763E-22
75	FBgn0027620	Acf	440.183	911.332	1.048521129	1.862E-25
76	FBgn0027929	NimB1	147.028	296.856	1.007763684	1.563E-08
77	FBgn0028396	TotA	426.435	2405.83	2.492875761	6.42E-148
78	FBgn0028514	CG4793	5.21224	26.2969	2.25587241	0.0018401
79	FBgn0028532	CG7968	128.898	284.961	1.141616522	3.631E-12
80	FBgn0028537	CG31775	150.639	73.5637	-1.034405726	1.553E-05
81	FBgn0028853	CG15263	159.37	533.401	1.735672849	7.367E-30
82	FBgn0028855	CG15282	7.05247	48.8333	2.791184248	5.169E-08
83	FBgn0028872	CG18095	271.518	749.932	1.470039126	1.179E-39
84	FBgn0028987	Spn28F	2118.07	939.273	-1.174011168	7.389E-06
85	FBgn0029167	Hml	2077.15	753.615	-1.460587341	6.549E-47
86	FBgn0029518	CG13376	1.2466	9.91638	3.021193483	0.0238821
87	FBgn0029701	CG12691	53.4366	108.068	1.006606213	0.0002213
88	FBgn0029733	CG6927	69.7237	21.2795	-1.728574407	4.414E-06
89	FBgn0029765	CG16756	414.533	957.305	1.203702983	7.553E-22
90	FBgn0029831	CG5966	4660.46	2327.18	-1.002003194	0.0063721
91	FBgn0029924	CG4586	27.6512	220.79	3.022641016	6.1E-35
92	FBgn0029990	CG2233	6337.85	18764	1.565727812	3.442E-12
93	FBgn0030098	CG12057	360.824	1322.53	1.876575889	2.889E-66
94	FBgn0030105	CG15369	162.634	446.313	1.455016066	6.422E-24
95	FBgn0030331	CG15221	42.8619	148.88	1.827653062	2.048E-10
96	FBgn0030509	CG11162	34.5367	70.2191	1.006724186	0.0062824
97	FBgn0030551	CG11674	206.009	55.1085	-1.90928195	4.695E-18
98	FBgn0030562	CG9400	585.112	287.404	-1.026941764	1.876E-16
99	FBgn0030569	CG9411	130.534	57.373	-1.186009323	3.233E-06
100	FBgn0030586	CG12539	52.6352	14.4476	-1.872306872	0.0001002
101	FBgn0030776	CG4653	18.8464	387.418	4.337988117	4.421E-74
102	FBgn0030804	CG13002	21.5561	6.50128	-1.705463072	0.0212528
103	FBgn0030889	CG15373	2.03781	16.8197	2.995223492	0.0040965
104	FBgn0030992	CG33253	93.8173	44.9342	-1.068070354	0.0003389
105	FBgn0030994	HP1D3csd	41.7373	18.4578	-1.186833018	0.0148296
106	FBgn0031033	CG14219	16.1706	101.914	2.637094304	0.0034275
107	FBgn0031034	CG14205	23.5901	710.528	4.899482952	2.361E-11
108	FBgn0031276	CG12506	40.8581	87.1604	1.087954778	0.0007693
109	FBgn0031305	Iris	313.994	778.957	1.301346211	2.513E-15
110	FBgn0031373	CG15358	111.032	51.2821	-1.132918518	0.0008423
111	FBgn0031547	Sr-CIV	27.2805	103.875	1.947577585	1.441E-09
112	FBgn0031560	CG16713	833.128	1840.21	1.140825072	1.067E-37
113	FBgn0031562	CG3604	204.794	85.2348	-1.264039196	3.703E-10
114	FBgn0031617	CG15635	924.482	421.829	-1.131422114	2.796E-25
115	FBgn0031619	CG3355	28.533	57.6079	1.007868206	0.0179877
116	FBgn0031630	CG15629	1034.46	512.136	-1.014047126	1.278E-24
117	FBgn0031678	Nepl3	25.7231	105.283	2.053259628	3.407E-11
118	FBgn0031689	Cyp28d1	1520.33	5273.28	1.793008641	3.52E-115
119	FBgn0031690	CG7742	1007.82	2253.28	1.160182232	5.57E-51
120	FBgn0031701	TotM	10.1863	87.7105	3.13154153	2.188E-11
121	FBgn0031726	Cyp6a16	38.8766	12.7011	-1.603349403	0.0014902
122	FBgn0031756	CG13992	38.5682	3.78669	-3.423169301	1.175E-06
123	FBgn0031805	Nepl4	341.682	170.643	-1.0096501	2.078E-09
124	FBgn0031829	IFT52	37.1525	9.13436	-2.019419771	0.0003572
125	FBgn0031837	DIP-iota	28.5493	7.91236	-1.821909425	0.0049388
126	FBgn0031961	CG7102	56.7181	18.4534	-1.630344695	0.0001232
127	FBgn0031976	CG7367	16.2653	42.2571	1.378372092	0.0035538
128	FBgn0032008	CG14277	180.58	367.124	1.02274863	6.121E-10
129	FBgn0032067	LManIV	41.2811	12.7725	-1.733271413	0.0036065
130	FBgn0032104	aust	28.7531	60.65	1.07711717	0.0050407
131	FBgn0032211	CG13138	60.5554	26.2952	-1.204448857	0.0019163
132	FBgn0032280	CG17105	4.86708	25.8077	2.451632737	0.0005257

133	FBgn0032362	spz4	18.6923	5.31062	-1.82644122	0.0231721
134	FBgn0032393	Nfs1	1205.49	2867.08	1.250228366	1.199E-56
135	FBgn0032493	Mabi	51.7882	23.6221	-1.115930797	0.0128301
136	FBgn0032505	CG16826	3119.52	1206.34	-1.370919355	0.0010048
137	FBgn0032535	Ance-2	63.5758	389.212	2.599223115	4.821E-43
138	FBgn0032613	Nepl9	284.559	121.686	-1.234561341	1.297E-09
139	FBgn0032638	SPH93	736.356	211.9	-1.79449268	1.961E-38
140	FBgn0032639	CG18563	141.902	358.951	1.349526413	5.29E-16
141	FBgn0032780	CG13085	42.7325	95.7458	1.165053457	5.301E-05
142	FBgn0032824	CG13962	137.326	10.6626	-3.646236693	1.732E-21
143	FBgn0032868	CG17472	365.589	163.806	-1.166724414	1.615E-08
144	FBgn0032913	CG9259	411.004	1139.39	1.46921126	0.0010954
145	FBgn0032945	CG8665	916.482	2179.34	1.248403066	5.016E-23
146	FBgn0033065	Cyp6w1	1563.98	4879.76	1.640845739	1.56E-96
147	FBgn0033131	CR12842	7.8845	22.4358	1.484581223	0.0347916
148	FBgn0033135	Tsp42En	237.321	75.8766	-1.635324897	2.124E-16
149	FBgn0033137	Tsp42Ep	185.183	808.23	2.124790423	3.76E-67
150	FBgn0033167	CG1701	67.3841	19.1217	-1.850505228	9.793E-05
151	FBgn0033170	sPLA2	436.228	1067.65	1.291604934	2.656E-31
152	FBgn0033204	CG2065	224.935	543.456	1.277860577	1.837E-17
153	FBgn0033274	CG14757	1156.31	3505.22	1.599240435	1.061E-98
154	FBgn0033275	CG14756	99.018	4.23412	-4.532471691	3.273E-18
155	FBgn0033289	CG2121	850.865	144.909	-2.554796483	7.059E-66
156	FBgn0033301	CG12780	185.967	41.3626	-2.195589057	9.484E-16
157	FBgn0033302	Cyp6a14	404.437	105.58	-1.942949671	7.646E-35
158	FBgn0033366	Ance-4	1171.53	2403.49	1.034907229	2.041E-22
159	FBgn0033395	Cyp4p2	2097.3	166.035	-3.665203465	1.93E-253
160	FBgn0033408	CG8800	18.2496	2.4144	-2.89209123	0.0016928
161	FBgn0033451	Marc	272.471	549.753	1.00810239	9.739E-14
162	FBgn0033508	Obp46a	10.1264	1.81929	-2.51334309	0.041635
163	FBgn0033518	Prx2540-2	2849.34	10505	1.882002063	2.72E-130
164	FBgn0033725	Cpr49Ac	1139.7	2775.84	1.284462954	8.212E-08
165	FBgn0033736	CG13154	995.988	343.167	-1.532447193	2.862E-29
166	FBgn0033749	achi	191.413	384.924	1.010625789	1.468E-10
167	FBgn0033769	CG8768	867.495	313.07	-1.47087065	1.058E-42
168	FBgn0033773	Mos	45.3576	21.2817	-1.093360242	0.0333887
169	FBgn0033789	CG13324	31.0673	152.789	2.288129093	3.27E-19
170	FBgn0033792	CG13325	95.2599	409.845	2.09717837	3.103E-07
171	FBgn0033808	CG4627	125.839	257.73	1.037183076	1.81E-08
172	FBgn0033820	CG4716	1382.15	340.403	-2.028796768	4.037E-50
173	FBgn0033821	CG10799	335.336	941.856	1.483659773	4.309E-27
174	FBgn0033826	CG4734	700.478	125.307	-2.485443815	7.653E-13
175	FBgn0033830	CG10814	18.8585	109.742	2.502551816	2.551E-13
176	FBgn0033836	CG18278	95.6733	198.122	1.048251422	7.666E-07
177	FBgn0033867	Cpr50Ca	67.3566	31.5688	-1.08107866	0.0027669
178	FBgn0033926	Arc1	2760.48	21302	2.947868304	3.887E-37
179	FBgn0033927	CR10102	486.899	3852.01	2.98359867	2.214E-29
180	FBgn0033936	Achl	2494.29	605.092	-2.045996692	2.663E-62
181	FBgn0033979	Cyp6a19	74.4428	30.9467	-1.262292441	0.0031482
182	FBgn0033982	Cyp317a1	493.571	217.868	-1.182517214	7.307E-17
183	FBgn0033997	Ir51a	15.9199	2.74942	-2.494828525	0.0104425
184	FBgn0034053	Cyp4aa1	460.758	204.534	-1.168689765	1.97E-14
185	FBgn0034143	CG8303	2820.28	1173.37	-1.264380089	1.731E-36
186	FBgn0034145	CG5065	7595.03	3601.08	-1.075888944	2.799E-51
187	FBgn0034160	CG5550	10.1454	26.625	1.367052714	0.0374082
188	FBgn0034166	CG6472	420.793	186.982	-1.168116031	1.065E-13
189	FBgn0034219	mthl4	108.287	231.786	1.095967614	2.255E-08
190	FBgn0034291	CG5770	7.49842	56.2272	2.884285958	2.58E-08
191	FBgn0034327	CG14505	106.626	257.289	1.275505249	2.994E-11
192	FBgn0034330	CG18107	152.846	339.953	1.150476068	1.81E-08
193	FBgn0034335	GstE1	2325.09	582.334	-2.000388758	2.588E-59
194	FBgn0034440	CG10073	113.569	22.2802	-2.366661355	5.129E-10
195	FBgn0034471	Obp56e	1.5324	11.8459	3.155300069	0.0128816
196	FBgn0034480	CG16898	714.861	205.06	-1.803637659	3.481E-07
197	FBgn0034507	CG11192	222.324	48.5013	-2.196853467	2.023E-21
198	FBgn0034538	CG16799	298.65	663.964	1.153233147	3.077E-21
199	FBgn0034539	CG11159	130.515	60.7153	-1.109452983	1.94E-05
200	FBgn0034568	CG3216	9.72149	25.2134	1.361308382	0.0349498
201	FBgn0034624	CG17974	17.7598	49.3552	1.454829052	0.0007295

202	FBgn0034629	Acox57D-d	470.665	185.196	-1.348472968	7.208E-20
203	FBgn0034660	Loxl2	2410.56	963.763	-1.32024507	2.3E-46
204	FBgn0034663	CG4363	841.732	1918.2	1.188390957	2.739E-51
205	FBgn0034715	Oatp58Db	130.857	799.835	2.606935337	9.122E-90
206	FBgn0034741	CG4269	352.508	757.762	1.102067033	3.676E-16
207	FBgn0034942	CG13566	18.4446	51.6254	1.507073103	0.0015078
208	FBgn0034965	ppk29	61.2525	25.2579	-1.267946316	0.0011981
209	FBgn0035006	CG4563	50.7079	126.022	1.310495405	9.958E-06
210	FBgn0035176	CG13905	227.913	813.832	1.839757054	3.92E-06
211	FBgn0035209	msd1	31.1019	79.1373	1.330089744	0.0004251
212	FBgn0035258	CG13931	86.9476	40.3651	-1.108202512	0.0006852
213	FBgn0035260	CG7991	267.392	130.511	-1.036860363	9.604E-08
214	FBgn0035315	CG8960	9.03703	0	-5.512934304	0.001239
215	FBgn0035325	CG13806	38.7946	95.7092	1.295262969	3.113E-05
216	FBgn0035328	yellow-g2	35.359	138.46	1.938650329	3.519E-10
217	FBgn0035343	CG16762	372.472	985.11	1.406858481	1.469E-25
218	FBgn0035429	CG12017	33.2398	68.209	1.020103187	0.0119244
219	FBgn0035434	Drsl5	90.4222	305.768	1.74427818	4.409E-20
220	FBgn0035439	CG14961	3053.42	977.084	-1.643203576	7.612E-08
221	FBgn0035545	CG12607	63.2457	16.9691	-1.918472886	4.633E-06
222	FBgn0035555	CG13720	78.5662	37.468	-1.068881983	0.0020007
223	FBgn0035619	Alp10	216.999	91.4768	-1.248986947	0.024862
224	FBgn0035620	Alp9	230.567	81.8092	-1.506836408	2.29E-07
225	FBgn0035641	CG5568	23.6627	6.64808	-1.835020362	0.0111278
226	FBgn0035669	CG6592	486.074	209.466	-1.20924925	3.28E-14
227	FBgn0035741	BBS1	21.5061	53.2226	1.314820245	0.0011792
228	FBgn0035791	CG8539	364.802	1246.41	1.779460185	1.015E-62
229	FBgn0035859	CG13678	53.2988	20.8308	-1.365616556	0.0022239
230	FBgn0035931	CG13312	16.1831	2.74898	-2.548595716	0.0141535
231	FBgn0036264	CG11529	34.7288	11.9195	-1.55263943	0.0049004
232	FBgn0036290	CG10638	965.272	3789.22	1.974835895	3.25E-111
233	FBgn0036321	CG14120	1511.03	187.265	-3.019543539	1.75E-111
234	FBgn0036410	CG8100	86.2026	416.864	2.273881933	1.499E-36
235	FBgn0036440	CG17177	120.396	12.1813	-3.320390463	1.979E-17
236	FBgn0036474	Or71a	43.624	15.4113	-1.511198096	0.0012215
237	FBgn0036528	CG7579	61.1772	26.6254	-1.2023573	0.0035122
238	FBgn0036592	CG13049	21.7948	7.72546	-1.483664349	0.0343095
239	FBgn0036600	CG13043	1926.66	5867.04	1.607016746	8.69E-128
240	FBgn0036601	CG13063	112.477	437.898	1.981036953	2.23E-30
241	FBgn0036607	CG13059	56.4077	15.0762	-1.908596383	1.648E-05
242	FBgn0036612	CG4998	89.2239	34.9046	-1.367389869	2.428E-05
243	FBgn0036640	nxf2	194.439	95.6529	-1.035840666	1.521E-05
244	FBgn0036690	Ilp8	119.525	56.6402	-1.080398966	8.064E-05
245	FBgn0036715	Cad74A	1854.59	809.251	-1.197428483	3.069E-32
246	FBgn0036767	CG16775	50.9873	115.874	1.181242311	1.773E-05
247	FBgn0036780	CG7330	160.082	75.8382	-1.074533719	1.174E-06
248	FBgn0036782	CG7320	7.88475	22.7329	1.542825994	0.0460851
249	FBgn0036796	CG18231	202.182	75.0871	-1.431847554	5.328E-11
250	FBgn0036806	Cyp12c1	715.755	1522.55	1.087894365	4.581E-32
251	FBgn0036831	CG6839	34.737	1.00244	-5.049414193	5.589E-06
252	FBgn0036948	CG7298	12.7734	33.6896	1.371948553	0.0180396
253	FBgn0036950	CG6996	17.4931	3.6765	-2.195196258	0.0204088
254	FBgn0036953	CG17145	5.9252	59.1557	3.285108124	3.648E-11
255	FBgn0036956	CG13813	419.844	5.57245	-6.203384433	1.718E-52
256	FBgn0036979	CG13247	49.8326	21.6873	-1.197767268	0.0082931
257	FBgn0036996	mag	428.572	171.536	-1.323180236	0.0006223
258	FBgn0036997	CG5955	384.401	151.962	-1.344549479	6.035E-17
259	FBgn0037042	CG12984	25.8271	55.0771	1.107044634	0.0114887
260	FBgn0037045	CG10584	301.351	1157.94	1.943514169	6.406E-70
261	FBgn0037069	Cpr78Cc	30.3675	9.35828	-1.696420143	0.0050393
262	FBgn0037123	CG14569	404.854	80.8248	-2.310211201	7.399E-32
263	FBgn0037124	CG14568	378.103	121.832	-1.621010365	1.228E-14
264	FBgn0037126	CG14567	205.389	1282.35	2.637908897	2.959E-15
265	FBgn0037127	CG14566	660.131	320.685	-1.03771005	1.532E-10
266	FBgn0037128	CG14572	1676.96	837.607	-1.001288675	0.0023057
267	FBgn0037181	CG11370	57.2487	152.201	1.403933523	3.702E-09
268	FBgn0037222	CG14642	132.772	353.777	1.399755986	1.508E-15
269	FBgn0037225	TwdlG	49.2695	109.447	1.131659861	0.0001527
270	FBgn0037304	CG1113	57.9972	14.6301	-1.994174671	4.795E-05

271	FBgn0037396	CG11459	174.964	567.974	1.69177743	7.658E-25
272	FBgn0037485	CG14606	174.931	352.354	1.017084822	3.426E-10
273	FBgn0037562	Nazo	23.9428	59.7614	1.329848602	0.0030061
274	FBgn0037627	CG13318	32.3609	294.942	3.16922497	5.932E-50
275	FBgn0037630	Ir85a	8.98676	22.2079	1.312634498	0.0487068
276	FBgn0037634	hng2	92.0397	206.057	1.157378221	5.766E-08
277	FBgn0037727	Nepl12	264.762	115.477	-1.194353725	4.031E-07
278	FBgn0037764	CG9459	48.0946	163.394	1.759183463	0.0065897
279	FBgn0037765	CG9458	79.0762	646.792	3.026391369	1.276E-08
280	FBgn0037772	Spn85F	32.1126	14.5935	-1.149819198	0.0433996
281	FBgn0037786	Alp13	33.0608	67.3246	1.005557294	0.0091917
282	FBgn0037827	CG4073	282.332	22.4388	-3.637762248	6.452E-42
283	FBgn0037837	CG14693	51.5814	120.88	1.239548504	3.477E-06
284	FBgn0037844	CG4570	82.4314	40.5175	-1.033390535	0.0013008
285	FBgn0037846	CG6574	719.9	1449.05	1.011039369	3.694E-29
286	FBgn0037930	CG14715	420.948	108.937	-1.942978172	1.999E-33
287	FBgn0037975	CG3397	740.987	353.572	-1.064832727	1.739E-14
288	FBgn0037999	CG4860	283.762	92.552	-1.614275615	6.023E-17
289	FBgn0038056	CG5961	234.493	542.876	1.204920716	3.904E-21
290	FBgn0038067	CG11598	197.839	11.2908	-4.127864136	4.857E-31
291	FBgn0038079	NijC	645.107	1613.45	1.321641362	2.382E-57
292	FBgn0038095	Cyp304a1	88.9692	249.323	1.479547896	5.905E-13
293	FBgn0038159	CG14369	31.2684	1.67248	-4.153092739	3.185E-06
294	FBgn0038214	CG9616	73.404	170.415	1.215896486	2.28E-07
295	FBgn0038299	Spn88Eb	652.917	1430.32	1.132076896	3.185E-40
296	FBgn0038339	CG6118	76.4817	35.731	-1.101551525	0.0032501
297	FBgn0038449	CG17562	225.8	725.596	1.682551921	1.767E-07
298	FBgn0038451	CG14893	611.835	2326.02	1.928992044	1.045E-76
299	FBgn0038455	CG14907	256.59	525.303	1.035690592	6.804E-13
300	FBgn0038505	CG17283	7.17801	0	-5.19045472	0.004297
301	FBgn0038506	CG5860	9.41664	1.15099	-3.122879548	0.0338177
302	FBgn0038727	CG7432	20.1809	4.30818	-2.235696939	0.0040607
303	FBgn0038730	CG6300	36.0945	173.248	2.275133375	8.501E-16
304	FBgn0038838	TotB	7.90653	31.747	1.97380603	0.0017732
305	FBgn0038956	CAH8	17.6627	53.7282	1.604577433	0.0003292
306	FBgn0039030	CG6660	146.62	357.121	1.280406075	0.0048616
307	FBgn0039073	CG4408	291.472	1100.16	1.9189622	1.598E-43
308	FBgn0039091	CG10182	0.609182	16.1153	4.721021732	0.0010343
309	FBgn0039092	CG16723	6.05397	30.2274	2.301769736	0.0007193
310	FBgn0039161	CG13606	14395.5	7020.67	-1.035868361	2.977E-22
311	FBgn0039203	CG13618	112.362	51.8015	-1.114002171	0.0003166
312	FBgn0039298	to	100.091	244.789	1.286906457	6.501E-11
313	FBgn0039411	dysf	215.167	446.705	1.060524068	1.18E-09
314	FBgn0039441	TwdlN	8.95529	0.81641	-3.612719571	0.0253662
315	FBgn0039443	TwdlS	167.126	43.5998	-1.956199138	6.993E-12
316	FBgn0039675	ppk21	3.64251	16.2268	2.214343276	0.0124181
317	FBgn0039678	Obp99a	597.957	250.603	-1.254044756	3.737E-25
318	FBgn0039684	Obp99d	453.929	166.168	-1.445891371	3.445E-20
319	FBgn0039685	Obp99b	5651.15	13154	1.219031881	8.773E-74
320	FBgn0039703	CG7829	26.6521	1.00375	-4.66749774	3.643E-05
321	FBgn0039759	CG9733	441.017	189.81	-1.218852494	5.074E-16
322	FBgn0039761	CG18404	291.068	644.003	1.138740102	5.79E-12
323	FBgn0039769	CG15534	14.7009	141.605	3.242827832	0.0001374
324	FBgn0039778	Jon99Fi	46.9897	1.82016	-4.717951793	7.139E-08
325	FBgn0039782	CG15539	183.593	86.2377	-1.094035613	2.639E-07
326	FBgn0039795	Spn100A	7.20327	27.1456	1.925980867	0.0022948
327	FBgn0039798	CG11313	3.08036	23.1371	2.917930734	0.0001438
328	FBgn0039801	Npc2h	676.568	1480.89	1.128228845	4.097E-28
329	FBgn0039811	CG15550	6.99573	22.3617	1.629622037	0.0315532
330	FBgn0287184	FASN3	488.247	3332.78	2.770389003	0.0052681
331	FBgn0040279	Osi14	10.2081	1.33833	-2.875828283	0.0349216
332	FBgn0040371	CG12470	1289.8	3052.62	1.241923096	8.44E-65
333	FBgn0040503	CG7763	74.1509	361.167	2.267056935	2.305E-38
334	FBgn0040602	CG14545	130.002	61.0512	-1.104127083	5.949E-05
335	FBgn0040705	ND-B8	89.8292	376.428	2.059401289	6.927E-27
336	FBgn0040717	Nplp4	149.231	43.5267	-1.766521723	3.694E-08
337	FBgn0040801	CG13053	11.1124	1.33703	-3.005155588	0.0198635
338	FBgn0041180	Tep4	1870.59	4576.89	1.289968184	7.499E-52
339	FBgn0041183	Tep1	6.03194	19.3112	1.668120257	0.023037

340	FBgn0041245	Gr39b	6.54059	0.408205	-4.096892374	0.0305925
341	FBgn0041709	yellow-g	233.524	504.609	1.11404191	3.916E-17
342	FBgn0041710	yellow-f	182.874	482.909	1.391100277	1.6E-20
343	FBgn0043532	Obp56i	36.9735	81.5452	1.136151231	0.0023353
344	FBgn0043533	Obp56f	172.998	40.7358	-2.101169709	4.678E-17
345	FBgn0043535	Obp57a	126.597	345.671	1.457782301	1.78E-17
346	FBgn0043578	PGRP-SB1	279.334	749.041	1.426745692	9.297E-36
347	FBgn0043854	slam	85.9041	32.8697	-1.385019223	2.169E-05
348	FBgn0044812	TotC	15.6024	197.247	3.644028259	3.116E-08
349	FBgn0045483	Gr59a	16.9122	1.22461	-3.934658387	0.0013292
350	FBgn0046253	CG3502	390.477	148.626	-1.384621027	2.571E-15
351	FBgn0046878	Obp83cd	352.157	732.877	1.055652866	0.0037012
352	FBgn0046999	CG6429	4132.65	1639.42	-1.332995461	3.645E-32
353	FBgn0047000	CG33946	97.6542	41.963	-1.232271292	4.627E-05
354	FBgn0047199	CG31517	67.6265	16.523	-2.059006573	2.724E-07
355	FBgn0047334	BG642312	819.524	321.593	-1.350866127	0.0112463
356	FBgn0050026	CG30026	391.937	809.323	1.046934054	7.041E-15
357	FBgn0050076	CG30076	32.8728	4.30905	-2.944164956	1.618E-05
358	FBgn0050083	CG30083	161.396	47.8243	-1.769752667	4.552E-12
359	FBgn0050090	CG30090	91.9305	373.988	2.038762198	9.807E-29
360	FBgn0050091	CG30091	10.2084	40.4787	2.012891972	0.0001324
361	FBgn0050098	CG30098	47.1633	7.84004	-2.549165633	3.066E-06
362	FBgn0050196	CG30196	17.4584	42.261	1.25945413	0.0123959
363	FBgn0050241	tRNA:Leu-AAG-1-1	6.3618	0.408205	-4.040414975	0.0371842
364	FBgn0050280	CG30280	517.772	201.427	-1.367239758	1.477E-18
365	FBgn0050287	CG30287	9.61797	49.8807	2.391944506	1.144E-06
366	FBgn0050289	CG30289	11.5017	98.6103	3.069861393	1.253E-16
367	FBgn0050340	CG30340	18.2211	40.3363	1.121519883	0.0345359
368	FBgn0050375	CG30375	6.21099	23.656	1.945123181	0.0038881
369	FBgn0050458	CG30458	13.9167	1.15099	-3.677619502	0.0037125
370	FBgn0050471	CG30471	39.4643	5.79245	-2.773688191	4.304E-06
371	FBgn0051029	CG31029	4653.12	1794.01	-1.374311895	1.124E-84
372	FBgn0051081	TwdlR	18.994	3.30574	-2.545098754	0.0042464
373	FBgn0051105	ppk22	2154.95	1032.11	-1.061830522	0.0145758
374	FBgn0051148	Gba1a	114.993	395.509	1.765270314	1.665E-17
375	FBgn0051262	CG31262	54.2214	12.9232	-2.07940307	4.176E-06
376	FBgn0051281	Tpl94D	791.393	37.6923	-4.400427731	3.58E-113
377	FBgn0051337	CG31337	8.49666	37.1399	2.073526435	0.0002811
378	FBgn0051356	tRNA:Thr-TGT-1-1	1.5324	14.4436	3.438943392	0.0048123
379	FBgn0051446	CG31446	108.777	235.799	1.111094861	2.604E-08
380	FBgn0051606	CG31606	64.2041	141.223	1.14349563	4.701E-05
381	FBgn0051658	Nnf1b	25.5282	10.6217	-1.267053309	0.0426236
382	FBgn0051681	CG31681	7.91921	25.6948	1.71217888	0.0099242
383	FBgn0051683	CG31683	322.111	1064.94	1.723840767	4.811E-37
384	FBgn0051700	CR31700	8.83298	27.2902	1.640205648	0.0102316
385	FBgn0051704	CG31704	95.2668	345.218	1.835905146	1.061E-16
386	FBgn0051872	CG31872	3904.2	1737.83	-1.16839624	4.963E-08
387	FBgn0051958	CR31958	8.25204	37.0227	2.193806453	5.806E-05
388	FBgn0052040	CG32040	49.6373	113.106	1.17561338	5.911E-05
389	FBgn0052185	edin	0.254322	6.90818	4.384858393	0.0196662
390	FBgn0052207	hpRNA:CR32207	198.111	89.979	-1.143131598	2.134E-07
391	FBgn0052219	CG32219	102.792	47.938	-1.113690083	0.0004003
392	FBgn0052279	Drsl2	17.3829	57.9346	1.722363489	0.0001427
393	FBgn0052283	Drsl3	10.7232	394.71	5.218380723	2.5E-17
394	FBgn0052302	CG32302	14.777	80.6308	2.436435583	9.279E-11
395	FBgn0052303	tRNA:Cys-GCA-3-1	5.46656	20.0946	1.826985676	0.0452565
396	FBgn0052475	mthl8	633.021	0	-11.64930207	6.78E-21
397	FBgn0052551	CG32551	21.6348	43.6725	1.017828494	0.0225505
398	FBgn0052553	lncRNA:CR32553	19.782	4.64189	-2.094591993	0.007317
399	FBgn0052581	CG32581	781.629	98.9631	-2.984119836	3.419E-98
400	FBgn0052625	CG32625	46.3842	17.7529	-1.383542261	0.0021449
401	FBgn0052834	CG32834	859.249	243.644	-1.825138106	5.342E-46
402	FBgn0053012	CG33012	66.9042	151.743	1.180585865	6.538E-07
403	FBgn0053013	CR33013	58.1504	179.216	1.63092901	2.423E-12
404	FBgn0053108	CG33108	62.2384	22.3199	-1.472906623	9.667E-05
405	FBgn0053200	VepD	25.4061	52.7359	1.068746875	0.0085755
406	FBgn0053281	CG33281	1965.58	4830.37	1.29549474	1.115E-70
407	FBgn0053282	CG33282	130.825	298.722	1.18716869	7.975E-13
408	FBgn0053294	CR33294	54.7932	25.5864	-1.110266417	0.0054308

409	FBgn0053300	Muc30E	6.41207	37.8784	2.598873284	9.751E-06
410	FBgn0053301	CG33301	714.219	233.335	-1.612003659	6.119E-05
411	FBgn0053307	CG33307	45.272	217.579	2.237155557	1.389E-19
412	FBgn0053318	CR33318	65.9056	171.376	1.365872533	4.622E-09
413	FBgn0053325	CG33325	44.1201	9.57958	-2.233739994	2.719E-05
414	FBgn0053337	CG33337	0	10.9519	6.003933867	0.0005333
415	FBgn0053458	CG33458	49.8228	110.701	1.151438074	3.282E-05
416	FBgn0053460	CG33460	33.6925	95.8124	1.504755612	1.23E-06
417	FBgn0053462	CG33462	22.1182	81.7034	1.883734968	4.436E-08
418	FBgn0053470	CG33470	312.004	904.758	1.538117025	3.612E-23
419	FBgn0053474	CG33474	15.6623	45.0065	1.521657794	0.0017974
420	FBgn0053509	CG33509	50.3426	192.815	1.914155733	5.293E-10
421	FBgn0053511	CG33511	460.276	49.7417	-3.191077505	7.97E-54
422	FBgn0053757	CG33757	0	14.5613	6.420616649	3.706E-05
423	FBgn0053773	CG33773	43.5587	20.3141	-1.092217651	0.0178163
424	FBgn0053993	CG33993	298.42	146.96	-1.019181914	2.819E-08
425	FBgn0054005	CG34005	32.807	13.8499	-1.241549395	0.0274465
426	FBgn0054038	CG34038	130.545	50.8029	-1.356468387	1.358E-06
427	FBgn0058160	CG40160	528.318	1846.05	1.802770583	3.63E-14
428	FBgn0062928	hpRNA:CR33940	255.645	36.9177	-2.787161541	3.982E-36
429	FBgn0065076	snoRNA:185	0	7.17001	5.402452222	0.0036192
430	FBgn0083015	snoRNA:Psi18S-9:	39.7304	19.3504	-1.035217864	0.0328879
431	FBgn0083951	CG34115	35.1714	15.7807	-1.176300595	0.0275574
432	FBgn0083978	CG17672	44.3251	21.5753	-1.029858625	0.0410282
433	FBgn0085235	CG34206	64.1067	372.781	2.548031111	5.547E-42
434	FBgn0085244	CG34215	178.753	65.2845	-1.465996669	2.86E-08
435	FBgn0085273	CG34244	20.2594	7.31812	-1.451937812	0.0486761
436	FBgn0085312	CG34283	25.4747	0.668296	-5.177652403	7.294E-05
437	FBgn0085415	CG34386	104.073	42.7085	-1.278294629	9.504E-06
438	FBgn0085421	Epac	1980.42	6250.91	1.658763998	6.765E-71
439	FBgn0085425	CG34396	819.116	376.126	-1.119049879	7.442E-21
440	FBgn0085452	CG34423	29.1583	81.345	1.483545621	4.771E-05
441	FBgn0085570	CR41243	5.26898	16.4869	1.653359635	0.0426236
442	FBgn0085732	CR40190	248.219	0	-10.29639708	3.139E-16
443	FBgn0085753	28SrRNA-Psi:CR4	13.5684	2.56208	-2.396944646	0.0401144
444	FBgn0087040	alphaTub67C	392.429	126.332	-1.645118093	1.422E-22
445	FBgn0250817	lncRNA:CR40465	53.5527	0.668296	-6.244834956	1.8E-07
446	FBgn0259140	CG42255	820.276	231.457	-1.832727409	1.397E-38
447	FBgn0259716	Nepl6	428.634	1005.96	1.228958188	3.41E-31
448	FBgn0259951	Sfp24Ba	213.386	18.3062	-3.554490712	3.71E-25
449	FBgn0259953	Sfp24Bd	82.5622	34.2053	-1.285804409	0.0013636
450	FBgn0259955	CG42465	37.3877	16.3022	-1.21787616	0.0201914
451	FBgn0259962	Sfp33A1	53.1226	22.4279	-1.256810502	0.0026313
452	FBgn0259971	CG42481	291.751	132.054	-1.146613348	5.727E-07
453	FBgn0259972	Sfp77F	73.8866	37.1677	-1.01732588	0.0155265
454	FBgn0260000	CG17570	76.5127	3.4909	-4.430650233	1.312E-14
455	FBgn0260393	CG17147	3.00185	18.6791	2.612409362	0.0026965
456	FBgn0260475	CG30059	10.1234	24.8436	1.272988791	0.0458941
457	FBgn0260718	CR42545	34.7289	15.6365	-1.124885784	0.0414427
458	FBgn0260780	wisp	389.51	113.187	-1.793940637	4.06E-23
459	FBgn0260874	Ir76a	30.0312	63.6128	1.080551414	0.0029524
460	FBgn0260953	CG42585	27.5695	61.5762	1.170735415	0.0014796
461	FBgn0260954	CG42586	329.714	63.2452	-2.371611983	1.081E-28
462	FBgn0260995	dpr21	66.9103	146.288	1.125247282	3.76E-06
463	FBgn0261016	clos	591.623	260.871	-1.18937452	9.875E-16
464	FBgn0261057	Sfp36F	16.5161	4.08644	-1.994589449	0.0295442
465	FBgn0261505	CR42653	54.0329	0.334148	-7.141240149	1.452E-07
466	FBgn0261506	CG42654	15.4236	2.41527	-2.65354651	0.0083578
467	FBgn0261632	lncRNA:CR42715	33.3877	2.08025	-3.977882361	8.903E-07
468	FBgn0261675	Npc1b	111.667	240.602	1.09822672	4.094E-07
469	FBgn0261803	CG42749	371.971	138.244	-1.421315194	1.404E-15
470	FBgn0261925	CG42792	37.6229	159.161	2.043671365	3.904E-12
471	FBgn0261987	Pxt	289.144	76.1191	-1.944595536	1.477E-18
472	FBgn0261990	CG42808	4939.34	2374.84	-1.056099437	7.176E-20
473	FBgn0262004	CG42822	22.4102	8.28265	-1.441150778	0.0493317
474	FBgn0262006	CG42824	19.3895	3.49133	-2.462948621	0.0028248
475	FBgn0262025	lncRNA:CR42836	33.1427	0	-7.400908034	1.162E-07
476	FBgn0262099	CG42852	62.1635	30.3755	-1.03036978	0.0113172
477	FBgn0262107	asRNA:CR42860	12.6448	29.0812	1.198387297	0.0381885

478	FBgn0262355	lncRNA:CR43053	10.585	33.4612	1.648940343	0.0025702
479	FBgn0262362	CG43060	97.9057	281.724	1.524357037	1.038E-16
480	FBgn0262363	CG43061	99.9313	42.5938	-1.248037361	0.005279
481	FBgn0262531	CG43085	24.2469	69.7107	1.498277553	5.827E-05
482	FBgn0262532	CR43086	17.8888	2.00532	-3.091894695	0.0020238
483	FBgn0262543	lncRNA:CR43097	32.8602	95.1076	1.530797057	2.343E-07
484	FBgn0262570	CG43110	118.903	286.229	1.26898065	1.912E-14
485	FBgn0262572	CG43112	14.8394	2.56208	-2.571895934	0.0116189
486	FBgn0262604	lncRNA:CR43130	47.1719	14.3735	-1.709318305	0.0004868
487	FBgn0262607	CG43133	195.133	458.926	1.230382308	1.79E-19
488	FBgn0262616	CR43142	2.44294	49.4293	4.338658383	1.077E-10
489	FBgn0262721	CG43165	31.2054	4.49378	-2.776841254	6.312E-05
490	FBgn0262883	lncRNA:CR43238	1.9155	16.9691	3.352106542	0.0021523
491	FBgn0262899	CG43254	81.0149	35.7323	-1.190174496	0.0020689
492	FBgn0263019	lncRNA:CR43314	37.2686	14.0725	-1.425107847	0.0074184
493	FBgn0263024	CG43319	36.7844	8.68998	-2.110805886	0.000611
494	FBgn0263082	CG43350	41.9878	7.80125	-2.433111373	2.261E-05
495	FBgn0263106	DnaJ-1	7445.96	3721.94	-1.000566914	5.844E-21
496	FBgn0263290	lncRNA:CR43399	32.7189	0	-7.373133992	1.284E-07
497	FBgn0263323	lncRNA:CR43404	31.6671	14.2995	-1.130549946	0.0418837
498	FBgn0263333	lncRNA:CR43414	86.6961	21.2028	-2.037758886	2.218E-09
499	FBgn0263337	lncRNA:CR43418	33.1302	10.474	-1.644887299	0.0047088
500	FBgn0263340	lncRNA:CR43421	2.04104	11.3998	2.576047757	0.039632
501	FBgn0263341	lncRNA:CR43422	0	23.0186	7.071381625	1.722E-06
502	FBgn0263345	asRNA:CR43426	15.4648	2.00576	-2.871891866	0.0069554
503	FBgn0263381	asRNA:CR43433	40.2713	7.13035	-2.489544684	1.34E-05
504	FBgn0263389	CG43441	32.6347	0.334583	-6.400444128	6.62E-06
505	FBgn0263506	lncRNA:CR43495	0.3831	8.95007	4.747384483	0.0060832
506	FBgn0263597	Acp98AB	23.8079	5.9053	-1.979561244	0.0053584
507	FBgn0263608	l(3)72Dr	211.411	69.0076	-1.619386202	2.542E-12
508	FBgn0263612	lncRNA:CR43621	0.609182	20.8722	5.097471844	0.0001557
509	FBgn0263621	CG43630	21.5593	5.31062	-2.018507281	0.0055148
510	FBgn0263659	lncRNA:CR43650	600.651	201.6	-1.577301237	4.963E-06
511	FBgn0263748	CG43673	144.609	49.6184	-1.545377895	1.155E-09
512	FBgn0263985	CR43734	17.7127	5.72013	-1.639720743	0.0420423
513	FBgn0263999	CG43742	12.7736	1.41195	-3.170557274	0.0083395
514	FBgn0264343	CG43799	12.9116	62.8387	2.272475654	2.398E-07
515	FBgn0264369	lncRNA:CR43821	21.509	56.8202	1.397783847	0.000349
516	FBgn0264386	Ca-alpha1T	4171.52	8407.91	1.01140012	1.184E-23
517	FBgn0264431	lncRNA:CR43849	88.705	41.9688	-1.075258849	0.0008638
518	FBgn0264470	lncRNA:CR43878	20.7993	54.0773	1.372222343	0.0012871
519	FBgn0264507	asRNA:CR43906	6.57206	20.0161	1.6171455	0.0334153
520	FBgn0264548	lncRNA:CR43927	3.20591	13.666	2.051070632	0.0294011
521	FBgn0264566	CG43938	29.7611	0.334583	-6.274070087	1.074E-05
522	FBgn0264568	lncRNA:CR43940	30.7565	7.94763	-1.96929798	0.001228
523	FBgn0264570	lncRNA:CR43942	25.3112	96.1053	1.888224618	7.764E-08
524	FBgn0264599	asRNA:CR43948	51.7728	0	-8.034038335	1.912E-09
525	FBgn0264726	lncRNA:CR43994	62.7304	20.2361	-1.662166327	0.0001518
526	FBgn0264745	CR44003	32.4051	8.32013	-1.936367479	0.0008722
527	FBgn0264787	lncRNA:CR44017	24.7246	5.93926	-2.083345783	0.004804
528	FBgn0264789	asRNA:CR44019	46.7487	19.3469	-1.290090217	0.0047362
529	FBgn0264827	asRNA:CR44035	37.4231	6.57316	-2.473896042	8.472E-05
530	FBgn0264828	lncRNA:CR44036	6.62233	24.3618	1.902479617	0.0041238
531	FBgn0264833	lncRNA:CR44041	13.2412	37.0655	1.47973262	0.027552
532	FBgn0264834	lncRNA:CR44042	151.503	63.7218	-1.249139867	3.23E-07
533	FBgn0264879	lncRNA:CR44070	20.9283	0.408205	-5.761647598	0.0001066
534	FBgn0264886	lncRNA:CR44077	15.0311	0	-6.249497412	8.622E-05
535	FBgn0264889	lncRNA:CR44080	0.863504	50.132	5.777267169	6.414E-09
536	FBgn0264914	lncRNA:CR44105	21.327	1.67205	-3.603378045	0.0001866
537	FBgn0264988	CG44139	5.9599	0.334583	-3.935386358	0.0492103
538	FBgn0264992	lncRNA:CR44143	4.60008	29.4934	2.631395282	0.0001338
539	FBgn0265039	lncRNA:CR44156	17.1006	36.4729	1.09035914	0.037923
540	FBgn0265047	FDY	36.5687	80.0701	1.138566687	0.0008281
541	FBgn0265076	lncRNA:CR44187	7.22505	46.98	2.650972212	3.07E-07
542	FBgn0265099	lncRNA:CR44204	15.3166	3.56626	-2.122872016	0.0335893
543	FBgn0265143	lncRNA:CR44212	0	8.91041	5.693102139	0.0010728
544	FBgn0265158	CG44227	5.55478	31.4868	2.550669718	0.0001032
545	FBgn0265266	CG13639	2598.77	1065.65	-1.285236845	2.929E-24
546	FBgn0265367	lncRNA:CR44308	36.3017	0.668731	-5.67839914	4.468E-06

547	FBgn0265444	lncRNA:CR44344	73.3662	1.41065	-5.692569653	1.395E-11
548	FBgn0265450	lncRNA:CR44350	22.109	0.668296	-4.966600023	0.0001992
549	FBgn0265455	CG44355	22.4224	6.83499	-1.706798626	0.0387056
550	FBgn0265467	lncRNA:CR44360	8.4088	28.2604	1.743206332	0.0194645
551	FBgn0265509	lncRNA:CR44371	0.942013	14.7099	4.00181964	0.0012484
552	FBgn0265533	CR44383	29.2932	0.742353	-5.358538711	2.207E-05
553	FBgn0265537	lncRNA:CR44387	0	7.2031	5.394371408	0.0031045
554	FBgn0265546	lncRNA:CR44396	14.6103	61.2725	2.051293451	6.901E-07
555	FBgn0265577	lncRNA:IBIN	23.9393	55.2579	1.18914304	0.0039923
556	FBgn0265582	lncRNA:CR44409	0.3831	11.1057	5.058654267	0.0024622
557	FBgn0265665	lncRNA:CR44472	18.1117	5.31149	-1.752372749	0.0333054
558	FBgn0265677	lncRNA:CR44484	15.3518	2.34034	-2.643604536	0.0097056
559	FBgn0265687	lncRNA:CR44494	14.3784	0	-6.168592742	0.0001276
560	FBgn0265690	lncRNA:CR44497	6.94895	20.3468	1.56347006	0.0372696
561	FBgn0265698	lncRNA:CR44505	1.21836	17.0048	3.78242377	0.0011221
562	FBgn0265723	lncRNA:CR44530	9.05583	1.41108	-2.678209168	0.0444206
563	FBgn0265727	lncRNA:CR44534	24.3356	7.65314	-1.641431754	0.0258688
564	FBgn0265728	lncRNA:CR44535	65.8808	13.5153	-2.327087239	1.388E-06
565	FBgn0265746	lncRNA:CR44553	6.05721	17.9729	1.592604633	0.0440747
566	FBgn0265747	lncRNA:CR44554	20.0772	5.60598	-1.885030467	0.0151112
567	FBgn0265764	lncRNA:CR44571	13.8726	0.334148	-5.17200894	0.0011055
568	FBgn0265795	lncRNA:CR44584	6.84195	21.5056	1.609615474	0.0268746
569	FBgn0265802	lncRNA:CR44591	2.00957	15.26	2.855542485	0.0053835
570	FBgn0265817	lncRNA:CR44606	36.7444	86.4502	1.221204028	0.0001092
571	FBgn0265820	lncRNA:CR44609	1.60146	49.8358	5.016415464	5.243E-10
572	FBgn0265822	lncRNA:CR44611	25.5472	89.6475	1.804702744	1.615E-08
573	FBgn0265826	lncRNA:CR44615	67.7014	22.4741	-1.579874434	0.0001147
574	FBgn0265851	lncRNA:CR44640	1.80552	22.5394	3.622599062	5.821E-05
575	FBgn0265854	lncRNA:CR44643	3.07713	88.9832	4.745674616	4.19E-18
576	FBgn0265856	lncRNA:CR44645	40.2931	90.3599	1.179465747	0.0001257
577	FBgn0265858	lncRNA:CR44647	0.609182	8.50616	3.80568692	0.0260689
578	FBgn0265859	lncRNA:CR44648	0.254322	7.3203	4.464382893	0.0234877
579	FBgn0265871	lncRNA:CR44660	13.9386	1.7448	-2.969485288	0.0121307
580	FBgn0265876	lncRNA:CR44665	35.7964	2.56208	-3.837109939	5.153E-07
581	FBgn0265885	lncRNA:CR44674	27.105	2.71019	-3.393657035	8.9E-05
582	FBgn0265943	lncRNA:CR44730	6.99896	0	-5.15988193	0.0052021
583	FBgn0265957	lncRNA:CR44744	22.14	48.2461	1.109169538	0.0125463
584	FBgn0265986	lncRNA:CR44765	0.254322	5.56984	4.071656799	0.0452661
585	FBgn0265992	lncRNA:CR44767	0.942013	7.65227	3.057758678	0.0425705
586	FBgn0265993	lncRNA:CR44768	215.617	105.846	-1.025086001	1.73E-06
587	FBgn0266001	lncRNA:CR44775	23.0072	3.89954	-2.548967682	0.0010542
588	FBgn0266036	lncRNA:CR44801	10.695	31.1593	1.561187017	0.0055729
589	FBgn0266099	CG44836	61.1396	27.7398	-1.148571824	0.0033875
590	FBgn0266168	lncRNA:CR44874	83.1036	17.3487	-2.226792048	2.79E-08
591	FBgn0266208	lncRNA:CR44903	40.2586	16.0826	-1.320927921	0.0114831
592	FBgn0266230	lncRNA:CR44925	2.3959	12.9215	2.523882956	0.0175601
593	FBgn0266246	lncRNA:CR44941	1.2466	11.8886	3.27676232	0.0090726
594	FBgn0266258	lncRNA:CR44953	21.3208	58.3728	1.442062418	0.0006518
595	FBgn0266306	lncRNA:CR44971	38.0093	0	-7.601628769	4.102E-08
596	FBgn0266380	lncRNA:CR45022	6.69761	0	-5.085368205	0.0065201
597	FBgn0266432	CG45062	91.566	45.9397	-1.005658372	0.0020483
598	FBgn0266455	CG45080	154.629	30.2257	-2.357445899	0.0491375
599	FBgn0266595	lncRNA:CR45120	5.77762	17.6409	1.603135911	0.0416432
600	FBgn0266633	asRNA:CR45140	8.84854	27.0306	1.588483313	0.0202893
601	FBgn0266683	lncRNA:CR45173	1.88079	11.5126	2.499663038	0.027816
602	FBgn0266684	lncRNA:CR45174	17.0503	45.2396	1.408235916	0.0019757
603	FBgn0266698	lncRNA:CR45188	36.1667	5.19778	-2.841657069	4.973E-06
604	FBgn0266771	lncRNA:CR45236	2.69403	17.9381	2.629908323	0.0035571
605	FBgn0266817	lncRNA:CR45279	6.76667	29.8188	2.106177923	0.0005241
606	FBgn0266821	lncRNA:CR45283	0.609182	10.8434	4.139730982	0.007146
607	FBgn0266822	lncRNA:CR45284	0.558913	14.1501	4.562479808	0.0018506
608	FBgn0266869	asRNA:CR45330	31.1366	11.6616	-1.395296014	0.0289065
609	FBgn0266873	lncRNA:CR45334	27.1737	9.32301	-1.525438662	0.0151563
610	FBgn0266903	lncRNA:CR45363	19.1728	0	-6.603141147	8.967E-06
611	FBgn0266935	lncRNA:CR45386	0.3831	29.4092	6.463297594	5.763E-06
612	FBgn0266951	lncRNA:CR45402	29.6547	5.27096	-2.524810108	0.0006249
613	FBgn0266966	lncRNA:CR45417	4.70683	16.3405	1.768455521	0.0313222
614	FBgn0266983	lncRNA:CR45434	15.8069	0.408205	-5.359060817	0.000547
615	FBgn0266990	lncRNA:CR45441	42.868	20.9092	-1.022602861	0.0325405

616	FBgn0267026	CR45470	6.236	35.0275	2.476618383	4.65E-05
617	FBgn0267080	lncRNA:CR45524	14.5665	0.669166	-4.363914501	0.0025957
618	FBgn0267137	lncRNA:CR45577	42.1235	6.27384	-2.783038686	3.532E-06
619	FBgn0267290	lncRNA:CR45726	15.7943	2.48933	-2.672412391	0.0086163
620	FBgn0267465	asRNA:CR45815	10.0481	1.82016	-2.487414327	0.0486049
621	FBgn0267489	PRY	17.0815	37.7646	1.130947327	0.0272726
622	FBgn0267541	lncRNA:CR45881	16.218	36.2891	1.158094562	0.0314765
623	FBgn0267542	asRNA:CR45882	1.32188	9.84711	2.791322297	0.0478844
624	FBgn0267557	lncRNA:CR45897	0.558913	14.5957	4.595631256	0.0012541
625	FBgn0267607	lncRNA:CR45945	18.0138	1.48514	-3.662711918	0.0009292
626	FBgn0267624	lncRNA:CR45962	50.8435	19.76	-1.346186085	0.004359
627	FBgn0267639	lncRNA:CR45977	7.99772	21.7208	1.469853112	0.0498095
628	FBgn0267643	lncRNA:CR45981	141.554	25.7715	-2.466798859	6.17E-18
629	FBgn0267671	lncRNA:CR46007	21.12	62.0541	1.565551317	7.806E-05
630	FBgn0267702	lncRNA:CR46035	102.195	24.7691	-2.055300291	3.969E-11
631	FBgn0267715	asRNA:CR46048	3.33145	23.1742	2.718553438	0.0005003
632	FBgn0267742	lncRNA:CR46073	2.3424	12.8121	2.411015867	0.0245648
633	FBgn0267757	lncRNA:CR46088	13.9699	1.48558	-3.280841199	0.0044748
634	FBgn0267777	lncRNA:CR46108	70.1735	145.5	1.059544567	8.635E-06
635	FBgn0267786	lncRNA:CR46117	3.74602	14.8545	2.009905579	0.0295658
636	FBgn0267787	lncRNA:CR46118	2.05984	11.402	2.445969942	0.0288743
637	FBgn0267789	lncRNA:CR46120	26.1215	9.06248	-1.516698998	0.0214846
638	FBgn0267798	lncRNA:CR46123	119.89	1.5592	-6.384228044	2.995E-15
639	FBgn0267817	lncRNA:CR46142	8.96797	1.22461	-2.989514035	0.0477431
640	FBgn0267934	lncRNA:CR46214	8.33055	0	-5.38802454	0.0022239
641	FBgn0267935	lncRNA:CR46215	17.0695	0	-6.419074628	2.803E-05
642	FBgn0267938	lncRNA:CR46218	5.22194	22.576	2.155672246	0.0033885
643	FBgn0267959	lncRNA:CR46239	10.9431	30.2997	1.478182863	0.0113474
644	FBgn0283428	lncRNA:CR46271	2.34564	21.6128	3.295340188	0.0001579
645	FBgn0284238	lncRNA:CR46322	8.93649	0.669166	-3.66614625	0.024855
646	FBgn0286871	lncRNA:CR46393	0.508644	8.54274	3.866307288	0.0207197

8) List of DEGs in *Drosophila* adults of *DmAnkrd11* $^{\Delta/\Delta}$ *vs WT*

	FlyBase ID	Gene Symbol	*WT* (nomarlized rds)	*DmAnkrd11* $^{\Delta/\Delta}$ (nomarlized rds)	log2FC (*DmAnkrd11* $^{\Delta/\Delta}$ *vs WT*)	padj
1	FBgn0000055	Adh	92.202	18.3421	-2.314251864	6.71E-10
2	FBgn0000355	Cp15	3.33743	32.4778	3.14881208	1.29E-04
3	FBgn0000473	Cyp6a2	2212.82	531.173	-2.058780559	1.02E-113
4	FBgn0000551	Edg78E	6.864	0	-5.086322063	2.12E-02
5	FBgn0001229	Hsp67Bc	70.3485	177.799	1.328856884	1.18E-08
6	FBgn0001230	Hsp68	3087.92	1002.5	-1.622963741	5.90E-08
7	FBgn0002531	Lcp1	12.8673	38.6528	1.589461044	6.15E-03
8	FBgn0002939	ninaD	1477.22	4002.81	1.437365531	3.53E-56
9	FBgn0003227	rec	172.459	82.6289	-1.049008616	3.66E-05
10	FBgn0003312	sad	38.0008	85.3884	1.169833177	4.04E-04
11	FBgn0004034	y	79.5012	312.396	1.970057368	2.18E-04
12	FBgn0004782	Ccp84Ab	2442.47	7669.61	1.651793999	3.55E-74
13	FBgn0004783	Ccp84Aa	198.633	735.861	1.885052672	1.97E-06
14	FBgn0010038	GstD2	122.189	245.401	1.004961502	1.17E-05
15	FBgn0010040	GstD4	12.4379	31.7641	1.338159828	3.58E-02
16	FBgn0010041	GstD5	423.465	99.4561	-2.092410072	8.04E-29
17	FBgn0010359	gammaTry	32.3624	9.49826	-1.769761565	2.82E-02
18	FBgn0011802	Gem3	500.688	226.578	-1.13857555	1.22E-14
19	FBgn0011822	Pgcl	109.346	229.913	1.087332666	2.49E-07
20	FBgn0012042	AttA	14.1326	1.20203	-3.388433983	1.58E-02
21	FBgn0013275	Hsp70Aa	253.451	41.6903	-2.604002316	2.51E-04
22	FBgn0013276	Hsp70Ab	755.061	143.405	-2.396538143	2.21E-03
23	FBgn0013277	Hsp70Ba	198.592	19.3383	-3.366802183	4.81E-08
24	FBgn0013278	Hsp70Bb	345.968	94.8168	-1.868401555	5.31E-03
25	FBgn0013279	Hsp70Bc	175.256	61.0722	-1.529702318	1.26E-05
26	FBgn0013772	Cyp6a8	238.71	1015	2.087363342	1.11E-40
27	FBgn0013812	Dhc93AB	533.515	1280.3	1.262124925	1.49E-37
28	FBgn0014865	Mtk	30.0523	10.3292	-1.498744801	4.32E-02
29	FBgn0015035	Cyp4e3	879.192	234.276	-1.906852428	3.01E-07
30	FBgn0015714	Cyp6a17	627.562	18.4598	-5.130334335	2.50E-100
31	FBgn0016675	Lectin-galC1	1399.45	678.919	-1.046745268	6.44E-15
32	FBgn0019928	Ser8	26.1895	70.1328	1.384925046	7.61E-03
33	FBgn0020508	Ag5r2	471.326	221.731	-1.091717949	4.97E-16
34	FBgn0020637	Lcp65Ag2	5.4335	20.5365	1.887085532	4.56E-02
35	FBgn0025837	CG17636	56.1187	115.76	1.024899536	3.75E-03
36	FBgn0026314	Ugt35B1	2041.15	734.49	-1.473502018	1.40E-09
37	FBgn0026315	Ugt35A1	86.7256	309.259	1.833635183	4.70E-26
38	FBgn0027584	CG4757	372.876	134.712	-1.469818642	6.83E-20
39	FBgn0028415	Met75Cb	196.895	22.9118	-3.093625509	1.58E-05
40	FBgn0028949	CG15254	52.8405	20.3607	-1.340096278	1.70E-02
41	FBgn0029161	slmo	58.8977	28.9125	-1.025727503	2.49E-02
42	FBgn0029831	CG5966	4626.73	1827.35	-1.339944966	6.08E-08
43	FBgn0030837	CG8661	65.2511	144.151	1.13767667	4.63E-06
44	FBgn0031248	CG11912	694.2	293.543	-1.249580936	1.22E-14
45	FBgn0031373	CG15358	110.294	46.7893	-1.232422706	7.53E-04
46	FBgn0031522	CG3285	366.106	882.986	1.264156712	2.04E-26
47	FBgn0031547	Sr-CIV	27.0971	62.1147	1.212849466	6.25E-03
48	FBgn0031550	IFT57	12.2786	37.6449	1.585663578	8.28E-03
49	FBgn0031688	Cyp28d2	46.0889	93.0778	1.001689097	4.02E-03
50	FBgn0031753	CG13999	70.7637	151.678	1.089864429	1.15E-04
51	FBgn0032265	CG18301	138.109	36.6307	-1.918067744	3.38E-08
52	FBgn0033135	Tsp42En	235.646	113.649	-1.052247829	2.59E-07
53	FBgn0033154	CG1850	144.624	17.205	-3.077716845	3.83E-23
54	FBgn0033204	CG2065	223.395	529.945	1.252341624	3.67E-16
55	FBgn0033275	CG14756	98.3116	33.906	-1.537542861	1.85E-06
56	FBgn0033520	Prx2540-1	2339.9	1154.42	-1.018369632	1.19E-20
57	FBgn0033580	CG13231	144.505	63.628	-1.190063579	2.83E-05
58	FBgn0033789	CG13324	30.8475	67.2995	1.112514222	5.07E-03
59	FBgn0033820	CG4716	1372.58	548.846	-1.320760748	7.60E-27
60	FBgn0033826	CG4734	695.747	247.088	-1.494666706	1.06E-02
61	FBgn0033830	CG10814	18.7365	49.1959	1.360840503	1.20E-02
62	FBgn0033942	Cpr51A	387.091	104.326	-1.890594735	9.37E-28
63	FBgn0033978	Cyp6a23	1050.39	6205.37	2.56243177	3.14E-08
64	FBgn0034289	CG10910	85.3717	308.02	1.844901302	9.29E-20
65	FBgn0034291	CG5770	7.44048	74.6651	3.305114822	1.57E-12

66	FBgn0034317	CG14499	28.4669	3.0757	-3.188975376	5.96E-04
67	FBgn0034328	IM23	1465.95	320.056	-2.188307805	1.98E-52
68	FBgn0034329	IM1	1369.97	541.854	-1.33540832	1.38E-21
69	FBgn0034715	Oatp58Db	129.921	395.271	1.601579495	1.15E-22
70	FBgn0034796	CG3700	332.261	723.813	1.117692302	6.32E-22
71	FBgn0035312	CG15820	53.0724	25.5334	-1.060015044	2.43E-02
72	FBgn0035328	yellow-g2	35.1223	0.695237	-5.638474284	2.34E-05
73	FBgn0035666	Jon65Aii	2.32558	74.9878	4.91753472	5.89E-05
74	FBgn0035750	CG14826	0.505925	10.0814	4.105815342	2.28E-02
75	FBgn0035780	CG18417	165.552	22.5993	-2.855570083	2.38E-04
76	FBgn0035791	CG8539	362.263	1087.72	1.591567355	1.95E-44
77	FBgn0035792	CG7548	6.72177	25.0531	1.907025007	8.16E-03
78	FBgn0035806	PGRP-SD	463.074	205.565	-1.175044017	3.65E-14
79	FBgn0035845	CG13675	28.864	304.359	3.399772375	5.41E-55
80	FBgn0035929	CG13311	386.775	101.629	-1.926700661	3.43E-26
81	FBgn0035934	TrpA1	87.4208	223.176	1.339116779	6.08E-10
82	FBgn0036023	CG18179	97.4357	9.40692	-3.37048645	1.36E-02
83	FBgn0036024	CG18180	1170.25	136.421	-3.099668188	1.44E-02
84	FBgn0036153	CG7573	146.375	70.4282	-1.050177398	4.46E-05
85	FBgn0036178	CG14137	2.68061	15.5641	2.630519461	1.51E-02
86	FBgn0036181	Muc68Ca	26.4004	53.5036	1.025043015	2.87E-02
87	FBgn0036225	CG5883	43.6726	11.0979	-1.962390958	2.86E-04
88	FBgn0036320	CG10943	85.401	34.0411	-1.335341405	3.54E-04
89	FBgn0036411	Sox21a	10.1867	35.6471	1.78246186	2.56E-03
90	FBgn0036440	CG17177	119.586	270.385	1.160646037	3.66E-06
91	FBgn0036590	CG13065	2357.97	1082.9	-1.121778253	8.09E-34
92	FBgn0036603	CG13062	110.427	224.122	1.009200778	1.64E-06
93	FBgn0036679	CG13022	55.9104	17.971	-1.601642369	8.10E-04
94	FBgn0036767	CG16775	50.6424	217.734	2.098414792	2.33E-21
95	FBgn0036950	CG6996	17.3718	1.69098	-3.299861177	3.66E-03
96	FBgn0036956	CG13813	416.929	176.356	-1.238840428	7.29E-13
97	FBgn0036969	Spn77Bb	1381.03	428.837	-1.687839064	3.67E-03
98	FBgn0037004	CG17637	145.526	72.6851	-1.011601155	6.04E-03
99	FBgn0037126	CG14567	203.962	98.9	-1.051568205	4.00E-04
100	FBgn0037128	CG14572	1664.65	716.506	-1.216465006	4.59E-05
101	FBgn0037181	CG11370	56.8439	20.2836	-1.490204478	6.02E-04
102	FBgn0037197	CG13239	134.505	60.9775	-1.154548413	3.14E-05
103	FBgn0037552	CG7800	332.571	140.86	-1.235938355	6.22E-14
104	FBgn0037627	CG13318	32.1315	129.447	1.998349072	1.21E-12
105	FBgn0037724	Fst	817.234	384.993	-1.078175337	4.41E-13
106	FBgn0037811	CG12592	269.719	27.1657	-3.326380444	3.72E-41
107	FBgn0037837	CG14693	51.2186	112.999	1.156479873	5.62E-05
108	FBgn0037844	CG4570	81.8599	35.6471	-1.196738753	6.06E-04
109	FBgn0037888	scpr-B	531.945	1229.81	1.209141533	4.82E-17
110	FBgn0037974	CG12224	3580.62	385.973	-3.213314934	1.49E-171
111	FBgn0037975	CG3397	735.662	38.7113	-4.235453218	2.61E-106
112	FBgn0037992	CG4702	106.492	250.137	1.243801729	8.37E-08
113	FBgn0037996	CG4830	11.983	35.3759	1.577035577	7.42E-03
114	FBgn0038001	CG17404	61.7648	29.5427	-1.081028576	1.17E-02
115	FBgn0038034	Cyp9f3	360.707	1001.66	1.472942337	6.11E-35
116	FBgn0038056	CG5961	232.847	649.883	1.475637439	7.02E-33
117	FBgn0038083	Ugt37A3	107.29	292.914	1.452219101	5.90E-14
118	FBgn0038095	Cyp304a1	88.3336	31.166	-1.510351489	1.34E-05
119	FBgn0038337	CG6125	572.729	1928.36	1.746912939	1.00E-66
120	FBgn0038339	CG6118	75.9296	34.3422	-1.142028176	2.49E-03
121	FBgn0038358	Ttc26	345.09	81.2545	-2.074848298	2.15E-24
122	FBgn0038414	CG6901	41.4552	105.322	1.323333093	5.41E-05
123	FBgn0038415	CG17929	7.05998	39.5155	2.45321951	9.47E-05
124	FBgn0038439	Cad89D	25.9366	54.4517	1.057915953	2.49E-02
125	FBgn0038462	CG17556	131.664	282.522	1.106393071	5.41E-09
126	FBgn0038613	Vha100-4	254.769	518.25	1.020777749	1.63E-10
127	FBgn0038719	CG16727	331.056	882.536	1.419575224	9.38E-33
128	FBgn0038730	CG6300	35.8551	119.828	1.752108862	7.44E-08
129	FBgn0038790	MtnC	213.312	66.7306	-1.690905558	1.78E-08
130	FBgn0038851	dmrt93B	28.2242	6.42485	-2.168163545	1.07E-02
131	FBgn0038866	CG5810	4.14735	34.4019	2.978034352	6.14E-05
132	FBgn0038873	CG5892	15.8883	36.4842	1.201634261	4.22E-02
133	FBgn0039307	CR13656	606.545	230.122	-1.394019991	5.53E-17
134	FBgn0039310	CG11878	757.639	2090.41	1.464204759	1.68E-33
135	FBgn0039311	CG10513	1273.76	3659.19	1.520113466	4.56E-39
136	FBgn0039315	CG13658	83.3777	326.634	1.969150586	3.73E-24

137	FBgn0039316	CG11893	2263.39	801.151	-1.498445311	1.34E-04
138	FBgn0039356	CG5039	25.3818	53.4531	1.062760392	2.53E-02
139	FBgn0039408	CG14551	3.78819	16.519	2.098138703	4.11E-02
140	FBgn0039443	TwdlS	165.973	61.5269	-1.444240833	2.45E-07
141	FBgn0039473	CG17191	0.603766	83.4272	7.101846665	2.15E-09
142	FBgn0039537	CG5590	5479.36	2635.53	-1.0557814	3.82E-07
143	FBgn0039629	CG11842	643.395	1478.27	1.199001631	1.12E-22
144	FBgn0040104	lectin-24A	12.5358	2.26552	-2.522938187	4.52E-02
145	FBgn0040251	Ugt302K1	13.6602	95.2346	2.819282355	1.17E-13
146	FBgn0040253	Ugt35E1	6.19873	21.5418	1.846463806	2.96E-02
147	FBgn0040255	Ugt35E2	33.5089	142.331	2.084700265	6.04E-14
148	FBgn0040256	Ugt35C1	1532.64	578.373	-1.405131429	4.51E-05
149	FBgn0040257	Ugt302E1	45.5171	131.141	1.539054796	1.47E-08
150	FBgn0040371	CG12470	1280.83	2583.47	1.010998581	9.47E-38
151	FBgn0040717	Nplp4	148.218	55.9832	-1.394269118	5.07E-05
152	FBgn0040734	CG15065	100.826	41.4331	-1.268466741	1.17E-04
153	FBgn0040764	CG13230	0.758888	10.8766	3.645562335	2.24E-02
154	FBgn0041224	Gr97a	0.984266	11.18	3.581238464	1.87E-02
155	FBgn0041621	Or82a	7.75707	23.9367	1.664986669	3.66E-02
156	FBgn0042207	CG18530	23.2114	51.853	1.190805853	2.36E-02
157	FBgn0043578	PGRP-SB1	277.371	566.762	1.033019154	8.04E-16
158	FBgn0043791	phu	323.209	712.091	1.135729431	5.04E-18
159	FBgn0047199	CG31517	67.1558	32.3043	-1.038249973	2.76E-02
160	FBgn0047205	lncRNA:CR3238[illegible]	54.1966	23.6068	-1.19718161	1.60E-02
161	FBgn0047334	BG642312	814.115	378.234	-1.107291569	1.43E-02
162	FBgn0050008	CG30008	880.303	362.474	-1.280031863	3.19E-02
163	FBgn0050031	CG30031	71.7596	17.4761	-2.05827899	4.67E-06
164	FBgn0050076	CG30076	32.6396	8.08994	-2.040126121	1.58E-03
165	FBgn0051041	CG31041	27.7438	92.3662	1.741706051	1.99E-06
166	FBgn0051080	TwdlH	9.58931	0.789457	-3.696159489	4.96E-02
167	FBgn0051091	CG31091	18.4857	41.4243	1.144064755	4.88E-02
168	FBgn0051268	CG31268	0	10.1751	5.894974298	1.89E-03
169	FBgn0051300	CG31300	73.316	563.755	2.944937075	1.13E-66
170	FBgn0051337	CG31337	8.44147	30.5811	1.822388016	2.42E-02
171	FBgn0051354	Hsp70Bbb	147.79	45.9502	-1.688456521	1.02E-07
172	FBgn0051446	CG31446	108.003	357.023	1.718539955	1.38E-21
173	FBgn0051704	CG31704	94.6149	41.1938	-1.223119099	2.94E-03
174	FBgn0052073	CG32073	13.3967	76.4813	2.533259646	4.04E-10
175	FBgn0052107	CG32107	92.8189	34.209	-1.430222666	8.27E-05
176	FBgn0052283	Drsl3	10.6501	122.184	3.529811914	1.56E-05
177	FBgn0052557	Mco4	60.1853	138.375	1.209515451	1.45E-05
178	FBgn0053013	CR33013	57.7304	126.589	1.137992906	3.20E-05
179	FBgn0053192	MtnD	404.413	164.142	-1.309572262	4.58E-13
180	FBgn0053462	CG33462	21.9657	2.98436	-2.813639129	1.84E-03
181	FBgn0053926	CG33926	122.642	3.83581	-5.089946181	1.06E-19
182	FBgn0058160	CG40160	524.579	1836.89	1.805822643	1.48E-13
183	FBgn0058298	CG40298	29.3485	74.8921	1.346053902	2.27E-04
184	FBgn0065047	snoRNA:U3:54A[illegible]	31.0152	9.89587	-1.64362277	1.02E-02
185	FBgn0085228	CG34199	423.92	147.981	-1.529117476	2.60E-19
186	FBgn0085320	CG34291	15.5498	41.0797	1.454499315	3.57E-02
187	FBgn0085411	CG34382	149.517	349.096	1.227255162	3.83E-12
188	FBgn0085465	CG34436	2.98038	16.0386	2.41951631	1.85E-02
189	FBgn0085466	CG34437	136.063	48.4473	-1.499703324	1.20E-08
190	FBgn0085481	CG34452	22.4951	45.2425	1.020894437	4.64E-02
191	FBgn0085736	CG40472	147.928	48.8985	-1.587763793	1.79E-08
192	FBgn0086265	psd	10.3824	29.3015	1.484927932	2.35E-02
193	FBgn0250832	Dup99B	319.08	150.814	-1.092883652	1.62E-05
194	FBgn0250836	Acbp3	741.034	355.947	-1.057227166	1.78E-15
195	FBgn0259748	CG42397	229.321	491.417	1.095287139	6.12E-12
196	FBgn0259917	CG42446	162.251	42.2865	-1.936879236	2.17E-02
197	FBgn0259998	CG17571	527.071	213.028	-1.308654737	3.43E-17
198	FBgn0260393	CG17147	2.98038	14.2799	2.260104004	4.96E-02
199	FBgn0260396	CG42521	29.9883	9.31845	-1.672584417	1.75E-02
200	FBgn0260463	Unc-115b	17.0594	80.0143	2.183799701	5.20E-08
201	FBgn0260874	Ir76a	29.8185	64.0156	1.11336301	1.13E-02
202	FBgn0260954	CG42586	327.392	156.018	-1.055287012	3.78E-06
203	FBgn0261632	lncRNA:CR4271[illegible]	33.156	4.44833	-2.938148702	3.91E-05
204	FBgn0261853	CG42782	204.222	55.7864	-1.874797648	7.20E-03
205	FBgn0262363	CG43061	99.2987	23.2443	-2.105951995	4.20E-06
206	FBgn0262366	CG43064	170.537	78.2877	-1.130937749	2.11E-06
207	FBgn0262587	CG43124	17.8271	2.49541	-2.721442234	1.51E-02

208	FBgn0262616	CR43142	2.42554	21.4027	3.127511368	1.06E-03
209	FBgn0262794	CG43175	18.1541	54.6457	1.563789488	1.11E-03
210	FBgn0262838	CG43202	62.5969	19.3114	-1.686260687	2.16E-04
211	FBgn0262839	CG43203	79.9998	17.7348	-2.167498465	3.14E-08
212	FBgn0262854	CG43218	168.291	1231.85	2.87178352	4.46E-09
213	FBgn0262901	lncRNA:CR4325[illegible]	13.2775	32.2003	1.283117982	4.86E-02
214	FBgn0262983	CG43291	114.778	34.2693	-1.726894325	1.21E-07
215	FBgn0262987	CG43295	4.70431	52.8469	3.463418423	1.21E-09
216	FBgn0262996	lncRNA:CR4330[illegible]	53.4359	24.6974	-1.111440125	2.86E-02
217	FBgn0263219	Dscam4	510.351	1538.31	1.590381179	1.82E-61
218	FBgn0263345	asRNA:CR43426	15.3586	39.8329	1.408645659	2.27E-02
219	FBgn0263405	lncRNA:CR4345[illegible]	23.7257	6.25138	-1.896242185	2.22E-02
220	FBgn0264431	lncRNA:CR4384[illegible]	88.0886	30.9621	-1.50099216	7.11E-04
221	FBgn0264480	lncRNA:CR4388[illegible]	22.3779	1.87942	-3.637421433	8.55E-04
222	FBgn0264512	lncRNA:CR4391[illegible]	11.8513	0	-5.911796611	7.10E-04
223	FBgn0264570	lncRNA:CR4394[illegible]	25.1417	72.6995	1.497067125	2.87E-04
224	FBgn0264699	CG43968	40.1985	109.381	1.450451444	1.99E-06
225	FBgn0264705	lncRNA:CR4397[illegible]	10.242	32.8863	1.721112685	7.68E-03
226	FBgn0264721	lncRNA:CR4398[illegible]	28.4767	5.3677	-2.370749949	3.04E-03
227	FBgn0264843	lncRNA:CR4405[illegible]	16.5536	4.04785	-2.047730557	4.28E-02
228	FBgn0264886	lncRNA:CR4407[illegible]	14.9354	0.695237	-4.392879844	7.59E-03
229	FBgn0265098	lncRNA:CR4420[illegible]	13.9896	1.68811	-2.995867831	1.69E-02
230	FBgn0265160	lncRNA:CR4422[illegible]	0.856728	11.7667	3.710202639	1.25E-02
231	FBgn0265367	lncRNA:CR4430[illegible]	36.0532	10.1722	-1.859560581	6.02E-03
232	FBgn0265378	lncRNA:CR4431[illegible]	5.63966	22.1515	1.958660486	1.40E-02
233	FBgn0265444	lncRNA:CR4434[illegible]	72.8572	29.6314	-1.28895705	8.02E-04
234	FBgn0265450	lncRNA:CR4435[illegible]	21.9469	45.4992	1.054397234	4.87E-02
235	FBgn0265537	lncRNA:CR4438[illegible]	0	8.96211	5.712889084	3.01E-03
236	FBgn0265541	CR44391	189.402	87.5001	-1.111574508	1.09E-04
237	FBgn0265546	lncRNA:CR4439[illegible]	14.5127	49.7407	1.758633068	2.52E-04
238	FBgn0265577	lncRNA:IBIN	23.7728	7.53612	-1.64020015	4.18E-02
239	FBgn0265740	lncRNA:CR4454[illegible]	0	19.9802	6.879732951	7.45E-06
240	FBgn0265746	lncRNA:CR4455[illegible]	6.0118	19.8796	1.74828742	5.00E-02
241	FBgn0265842	lncRNA:CR4463[illegible]	5.36747	25.2358	2.225703286	5.14E-03
242	FBgn0265885	lncRNA:CR4467[illegible]	26.9165	1.20203	-4.275951153	1.01E-04
243	FBgn0265922	lncRNA:CR4471[illegible]	7.55716	30.0821	1.95336469	5.83E-03
244	FBgn0265980	lncRNA:CR4475[illegible]	14.6064	0.601016	-4.387321834	7.11E-03
245	FBgn0266094	lncRNA:CR4483[illegible]	33.0641	82.0939	1.316147919	9.05E-04
246	FBgn0266163	CG44869	14.8572	2.98148	-2.250911714	4.62E-02
247	FBgn0266227	lncRNA:CR4492[illegible]	566.847	189.754	-1.58335876	4.11E-27
248	FBgn0266260	lncRNA:CR4495[illegible]	0.935346	20.345	4.46661037	3.27E-04
249	FBgn0266395	lncRNA:CR4503[illegible]	28.9104	7.52115	-1.949415496	7.10E-03
250	FBgn0266702	asRNA:CR45192	42.1223	17.0223	-1.303134488	1.42E-02
251	FBgn0266733	asRNA:CR45206	43.9469	1.99149	-4.383110329	6.05E-08
252	FBgn0266780	lncRNA:CR4524[illegible]	19.5636	4.76956	-1.995109894	3.28E-02
253	FBgn0266929	lncRNA:CR4538[illegible]	35.62	11.1685	-1.67213906	7.37E-03
254	FBgn0267004	lncRNA:CR4544[illegible]	0.3805	6.61675	4.315299128	5.00E-02
255	FBgn0267026	CR45470	6.1945	36.5928	2.537860106	2.45E-05
256	FBgn0267106	CG45546	62.4633	28.0461	-1.147370067	2.77E-02
257	FBgn0267137	lncRNA:CR4557[illegible]	41.8148	5.54751	-2.891558952	3.78E-06
258	FBgn0267139	lncRNA:CR4557[illegible]	5.18265	22.3163	2.102937925	8.49E-03
259	FBgn0267160	asRNA:CR45600	323.802	110.813	-1.530650599	1.17E-12
260	FBgn0267204	lncRNA:CR4564[illegible]	2.55096	42.2965	3.971279914	1.65E-08
261	FBgn0267205	lncRNA:CR4564[illegible]	2.45312	116.015	5.423956083	2.08E-20
262	FBgn0267240	lncRNA:CR4568[illegible]	0	8.2279	5.599141925	6.47E-03
263	FBgn0267473	lncRNA:CR4582[illegible]	20.6709	2.08571	-3.268881749	2.04E-03
264	FBgn0267492	Mst77Y-7	181.419	88.2457	-1.041711927	1.35E-06
265	FBgn0267621	lncRNA:CR4595[illegible]	22.4315	3.95938	-2.515106923	3.04E-03
266	FBgn0267715	asRNA:CR46048	3.30985	18.7461	2.4400199	1.38E-02
267	FBgn0267730	lncRNA:CR4606[illegible]	14.0661	37.3822	1.402026748	1.97E-02
268	FBgn0267755	lncRNA:CR4608[illegible]	45.9939	10.1022	-2.180618818	8.49E-05
269	FBgn0267798	lncRNA:CR4612[illegible]	119.048	1.59388	-6.067067319	5.57E-16
270	FBgn0267800	lncRNA:CR4612[illegible]	28.4747	9.17991	-1.652675771	1.87E-02
271	FBgn0267993	lncRNA:CR4626[illegible]	51.6753	23.7833	-1.103644702	3.43E-02
272	FBgn0283428	lncRNA:CR4627[illegible]	2.32981	12.948	2.553826729	3.44E-02
273	FBgn0283461	Drs	2616.91	1160.03	-1.174730025	1.62E-35
274	FBgn0283480	Alp2	351.769	2383.98	2.760400027	1.95E-06
275	FBgn0284435	tyn	237.25	554.267	1.230508221	2.84E-19
276	FBgn0286030	lncRNA:CR4635[illegible]	0	100.317	9.203055142	3.98E-12
277	FBgn0286075	DNAlig3	434.558	906.824	1.059270092	4.06E-23

Table S3. CUT&Tag peaks of Hs65K and HDAC3 in human 293T cells

1) List of Hs65K CUT&Tag peaks in 293T cells of *HsANKRD11-KD vs ctrl*

2) List of HDAC3 CUT&Tag peaks in 293T cells of *HsANKRD11-KD vs ctrl*

1) List of Hs65K CUT&Tag peaks in 293T cells of *HsANKRD11-KD vs ctrl*

chrom	peak start	peak end	log2FC(*HsANKRD11-KD vs ctrl*)	p-value	change
chr16	57299971	57300371	-1.097105139	1.37E-18	decrease
chr16	22297053	22297453	-1.33544605	1.04E-16	decrease
chr15	89334855	89335255	-1.237767654	4.87E-16	decrease
chr11	76235303	76235703	-1.07135402	6.87E-16	decrease
chr19	4723847	4724247	-1.340886932	1.03E-15	decrease
chr16	3150495	3150895	-1.157318734	3.57E-15	decrease
chr7	139340420	139340820	-1.067037983	4.51E-15	decrease
chr1	161530844	161531244	-0.999097041	1.71E-14	decrease
chr19	39412122	39412522	-1.198341742	2.10E-14	decrease
chr1	161612498	161612898	-1.004499709	2.47E-14	decrease
chr16	71426430	71426830	-1.014250654	3.23E-14	decrease
chr17	38869312	38869712	-1.055681697	4.22E-14	decrease
chr21	17454648	17455048	-1.13465887	4.49E-14	decrease
chr14	20343215	20343615	-1.127544298	6.48E-14	decrease
chr6	26322731	26323131	-1.006964679	2.49E-13	decrease
chr12	96035623	96036023	-1.204156726	2.85E-13	decrease
chr5	140718773	140719173	-0.945481105	5.34E-13	decrease
chr19	1021195	1021595	-1.203649664	6.22E-13	decrease
chr3	45688882	45689282	-1.078466956	9.44E-13	decrease
chr14	20613519	20613919	-0.982432211	1.14E-12	decrease
chr13	44918110	44918510	-1.038386371	1.52E-12	decrease
chr6	27684416	27684816	-1.153196691	1.68E-12	decrease
chr1	153671022	153671422	-1.0451334	1.90E-12	decrease
chr19	33176899	33177299	-1.141502901	3.19E-12	decrease
chr16	87383814	87384214	-1.20751555	3.58E-12	decrease
chr17	8138847	8139247	-0.985106695	3.67E-12	decrease
chr6	27478771	27479171	-0.893890677	4.41E-12	decrease
chr13	94549502	94549902	-1.04210058	5.28E-12	decrease
chr2	27050724	27051124	-0.918724481	6.36E-12	decrease
chr6	52995231	52995631	-1.086720458	7.47E-12	decrease
chr19	45478618	45479018	-1.042367728	8.06E-12	decrease
chr16	22190833	22191233	-1.323923112	9.16E-12	decrease
chr1	161421992	161422392	-1.008774001	1.29E-11	decrease
chr7	148987189	148987589	-0.923626893	1.63E-11	decrease
chr12	98503319	98503719	-0.973055373	4.51E-11	decrease
chr16	3152658	3153058	-0.919793188	4.66E-11	decrease
chr11	59566097	59566497	-0.983224021	5.48E-11	decrease
chr17	82494563	82494963	-0.946531983	5.64E-11	decrease
chr2	156400840	156401240	-0.926963245	7.70E-11	decrease
chr15	45198596	45198996	-0.941767624	8.62E-11	decrease
chr10	67764107	67764507	-0.953425529	9.00E-11	decrease
chr2	130337209	130337609	-1.025739701	1.31E-10	decrease
chr16	3191445	3191845	-0.871701155	1.32E-10	decrease
chr1	204506524	204506924	-0.973057713	1.51E-10	decrease
chr9	35657821	35658221	-1.094292264	1.51E-10	decrease
chr6	27334766	27335166	-0.924292703	1.95E-10	decrease
chr6	27545540	27545940	-0.865975991	2.31E-10	decrease
chr11	68460259	68460659	-1.082225387	2.73E-10	decrease
chr17	49192332	49192732	-0.928360905	2.75E-10	decrease
chr19	42268626	42269026	-1.377792906	3.02E-10	decrease
chr1	167714737	167715137	-0.898162876	5.00E-10	decrease
chr6	27333212	27333612	-0.972819566	5.30E-10	decrease
chr17	8173250	8173650	-1.116395429	6.19E-10	decrease
chr7	148983370	148983770	-0.998156926	6.31E-10	decrease
chr16	22195718	22196118	-0.937885221	7.71E-10	decrease
chr17	38867570	38867970	-1.058746601	8.00E-10	decrease
chr6	27177850	27178250	-0.942449065	1.02E-09	decrease
chr6	27132632	27133032	-0.959820685	1.05E-09	decrease
chr7	149691027	149691427	-0.890205591	1.30E-09	decrease
chr1	161539998	161540398	-0.993790521	1.34E-09	decrease
chr11	59560251	59560651	-0.894713971	1.49E-09	decrease
chr14	102316807	102317207	-1.159108105	1.87E-09	decrease
chr1	161399636	161400036	-0.917900091	2.15E-09	decrease
chr14	20609434	20609834	-0.927666437	2.18E-09	decrease
chr6	27902971	27903371	-0.874117574	2.28E-09	decrease
chr6	26568689	26569089	-0.83791148	2.47E-09	decrease
chr15	40593923	40594323	-1.203296687	2.72E-09	decrease
chr15	65869154	65869554	-0.909773646	2.89E-09	decrease
chr6	26331213	26331613	-0.831507953	2.93E-09	decrease
chr6	26532993	26533393	-0.88362152	2.98E-09	decrease
chr15	67839992	67840392	-0.999977985	3.67E-09	decrease
chr8	95269745	95270145	-0.917581224	4.07E-09	decrease
chr6	28474418	28474818	-1.044481631	4.58E-09	decrease

chr7	128783258	128783658	-1.076309094	5.33E-09	decrease
chr14	22929434	22929834	-0.902155206	5.72E-09	decrease
chr8	66113169	66113569	-0.751063262	6.37E-09	decrease
chr12	8243177	8243577	-1.528003438	6.66E-09	decrease
chr1	228139844	228140244	-1.301792473	6.73E-09	decrease
chr16	3189333	3189733	-0.862885037	8.27E-09	decrease
chr19	5904276	5904676	-1.271237434	8.40E-09	decrease
chr1	146051875	146052275	-1.285956768	1.13E-08	decrease
chr5	140710957	140711357	-0.798010912	1.17E-08	decrease
chr6	26537292	26537692	-0.812361956	1.22E-08	decrease
chr1	93847209	93847609	-0.881400275	1.26E-08	decrease
chr5	171387445	171387845	-1.43917263	1.41E-08	decrease
chr16	3157240	3157640	-0.854922987	1.66E-08	decrease
chr11	122559732	122560132	-1.014005815	1.67E-08	decrease
chr8	123157226	123157626	-0.884428108	1.72E-08	decrease
chr6	27495646	27496046	-0.866932668	1.87E-08	decrease
chr11	59557332	59557732	-0.792298847	1.93E-08	decrease
chr5	151771424	151771824	-1.514259907	2.23E-08	decrease
chr19	50147959	50148359	-1.459249865	2.30E-08	decrease
chr6	144216333	144216733	-1.034288161	2.30E-08	decrease
chr6	27502912	27503312	-0.809854258	2.66E-08	decrease
chr6	27214209	27214609	-0.826580951	2.84E-08	decrease
chr6	26305227	26305627	-0.83586652	2.90E-08	decrease
chr6	27795803	27796203	-0.880628066	2.90E-08	decrease
chr17	8186713	8187113	-0.816563323	2.91E-08	decrease
chr17	8156410	8156810	-1.068536566	2.96E-08	decrease
chr6	26571736	26572136	-0.837028055	2.98E-08	decrease
chr6	57793334	57793734	-1.472987564	3.12E-08	decrease
chr10	5853576	5853976	-0.917565012	3.52E-08	decrease
chr11	62601750	62602150	-1.47773687	3.64E-08	decrease
chr11	66347987	66348387	-1.191959238	3.91E-08	decrease
chr19	1455682	1456082	-1.504137856	4.17E-08	decrease
chr16	636725	637125	-0.943372821	5.73E-08	decrease
chr22	19479332	19479732	-1.401822665	6.25E-08	decrease
chr6	26327517	26327917	-0.725824266	6.35E-08	decrease
chr17	4967368	4967768	-1.245944759	6.95E-08	decrease
chr14	49862677	49863077	-0.824924917	7.77E-08	decrease
chr6	27230341	27230741	-0.821285652	8.40E-08	decrease
chr14	20683142	20683542	-0.836689426	8.44E-08	decrease
chr19	44890614	44891014	-1.251151027	8.51E-08	decrease
chr19	41363826	41364226	-1.709519921	9.42E-08	decrease
chr1	228457835	228458235	-1.090256307	9.53E-08	decrease
chr14	104752578	104752978	-1.45622393	1.02E-07	decrease
chr11	695243	695643	-1.292659057	1.06E-07	decrease
chr19	893148	893548	-1.158516792	1.10E-07	decrease
chr8	102239189	102239589	-1.417933973	1.12E-07	decrease
chr16	30065310	30065710	-1.588028181	1.12E-07	decrease
chr14	20625204	20625604	-0.836501916	1.24E-07	decrease
chr12	53501194	53501594	-1.25469286	1.29E-07	decrease
chr14	20663097	20663497	-0.780300941	1.37E-07	decrease
chr6	27687535	27687935	-0.749987383	1.39E-07	decrease
chr21	33641962	33642362	-1.120680251	1.41E-07	decrease
chr6	27505616	27506016	-0.81555268	1.44E-07	decrease
chr11	62753838	62754238	-1.360664235	1.46E-07	decrease
chr7	149028557	149028957	-1.374530039	1.47E-07	decrease
chr1	146036975	146037375	-0.810907561	1.57E-07	decrease
chr1	235504601	235505001	-1.193912737	1.68E-07	decrease
chr6	27519200	27519600	-0.744703308	1.88E-07	decrease
chr19	8525608	8526008	-1.215215364	1.91E-07	decrease
chr16	3158685	3159085	-0.970623215	1.99E-07	decrease
chr11	64778659	64779059	-1.342898719	1.99E-07	decrease
chr19	1383298	1383698	-0.835211255	2.32E-07	decrease
chr6	27553189	27553589	-0.776763345	2.36E-07	decrease
chr6	27672249	27672649	-0.778669729	2.60E-07	decrease
chr6	57800002	57800402	-1.034396536	2.93E-07	decrease
chr19	38850168	38850568	-1.070643677	2.94E-07	decrease
chr9	37465511	37465911	-1.383898343	3.06E-07	decrease
chr6	26312819	26313219	-0.684968665	3.12E-07	decrease
chr9	125200293	125200693	-1.558044058	3.22E-07	decrease
chr19	1062966	1063366	-1.711365585	3.32E-07	decrease
chr19	18636476	18636876	-1.347362584	3.58E-07	decrease
chr22	38705433	38705833	-1.47661079	3.74E-07	decrease
chr7	148941389	148941789	-0.898567683	3.80E-07	decrease
chr13	67230190	67230590	-1.605683521	4.04E-07	decrease
chr16	30658421	30658821	-0.988826106	4.53E-07	decrease
chr11	59615853	59616253	-1.374945991	4.57E-07	decrease
chr19	19385363	19385763	-1.748547309	4.77E-07	decrease

chr1	161390048	161390448	-1.192381649	4.87E-07	decrease
chr17	8222374	8222774	-0.79315928	5.23E-07	decrease
chr1	36224318	36224718	-1.288029432	5.50E-07	decrease
chr19	38930476	38930876	-1.596062068	5.75E-07	decrease
chr1	30824243	30824643	-1.62566591	5.89E-07	decrease
chr17	19507943	19508343	-1.126433858	5.95E-07	decrease
chr2	70248638	70249038	-0.837090112	6.47E-07	decrease
chr6	26575515	26575915	-0.779824226	6.76E-07	decrease
chr17	8226485	8226885	-0.76958003	7.35E-07	decrease
chr16	3182694	3183094	-0.882879215	8.07E-07	decrease
chr19	45615918	45616318	-1.494760596	8.92E-07	decrease
chr1	110338937	110339337	-1.098086595	9.30E-07	decrease
chr19	43668507	43668907	-1.327622188	1.09E-06	decrease
chr1	43649841	43650241	-1.415561832	1.17E-06	decrease
chr6	27157956	27158356	-0.833698314	1.19E-06	decrease
chr7	26200850	26201250	-1.241875202	1.34E-06	decrease
chr15	78860046	78860446	-0.87935418	1.41E-06	decrease
chr12	53626037	53626437	-1.294201894	1.42E-06	decrease
chr11	59550548	59550948	-0.663349247	1.42E-06	decrease
chr13	41060779	41061179	-0.928828205	1.42E-06	decrease
chr19	49676718	49677118	-1.074675334	1.45E-06	decrease
chr16	30526925	30527325	-1.564635603	1.47E-06	decrease
chr19	680654	681054	-1.366513086	1.55E-06	decrease
chr6	27295269	27295669	-0.763826643	1.69E-06	decrease
chr1	16727216	16727616	-0.967904381	1.71E-06	decrease
chr14	35826127	35826527	-1.478773725	1.86E-06	decrease
chr16	30922752	30923152	-0.997574764	1.93E-06	decrease
chr1	46303276	46303676	-1.524940235	1.98E-06	decrease
chr19	48468906	48469306	-1.282626448	2.04E-06	decrease
chr2	69914583	69914983	-1.332879659	2.04E-06	decrease
chr2	197453233	197453633	-1.203019017	2.05E-06	decrease
chr1	161524046	161524446	-1.076820041	2.07E-06	decrease
chr14	73950137	73950537	-1.385994583	2.08E-06	decrease
chr1	145287528	145287928	-1.005896883	2.10E-06	decrease
chr6	26285561	26285961	-0.767073579	2.34E-06	decrease
chr19	18932308	18932708	-1.422606282	2.36E-06	decrease
chr19	8592236	8592636	-1.513748417	2.38E-06	decrease
chr3	49094331	49094731	-1.225785196	2.53E-06	decrease
chr17	75046672	75047072	-1.028777272	2.58E-06	decrease
chr19	17266963	17267363	-1.345067228	2.58E-06	decrease
chr6	170553915	170554315	-1.426543206	2.61E-06	decrease
chr1	228486951	228487351	-1.183405397	2.88E-06	decrease
chr12	122526758	122527158	-1.227064518	2.89E-06	decrease
chr13	72727590	72727990	-1.278247855	2.94E-06	decrease
chr11	57667400	57667800	-1.230347974	2.98E-06	decrease
chr6	27592549	27592949	-0.704536888	2.99E-06	decrease
chr16	89557315	89557715	-1.210389674	3.03E-06	decrease
chr16	30370649	30371049	-1.13009309	3.03E-06	decrease
chr14	44961768	44962168	-1.492770238	3.42E-06	decrease
chr17	4731412	4731812	-1.36992435	3.45E-06	decrease
chr19	45692201	45692601	-1.230232224	3.49E-06	decrease
chr17	39154231	39154631	-0.726427409	3.65E-06	decrease
chr19	55599543	55599943	-1.313152209	3.78E-06	decrease
chr10	132335375	132335775	-1.231547651	4.19E-06	decrease
chr17	7329431	7329831	-1.320392792	4.32E-06	decrease
chr19	47113117	47113517	-1.262012849	4.36E-06	decrease
chr6	26521041	26521441	-0.924782645	4.38E-06	decrease
chr11	64241181	64241581	-1.48910411	4.45E-06	decrease
chr19	40778039	40778439	-1.562047256	4.46E-06	decrease
chr17	38861461	38861861	-0.965280754	4.47E-06	decrease
chr15	45200355	45200755	-0.766619689	4.51E-06	decrease
chr1	23980226	23980626	-1.646194754	4.92E-06	decrease
chr16	30748079	30748479	-1.128768215	5.15E-06	decrease
chr15	75451843	75452243	-1.048353984	5.21E-06	decrease
chr17	45061230	45061630	-1.329477916	5.23E-06	decrease
chr1	65420167	65420567	-1.585547335	5.36E-06	decrease
chr12	6851931	6852331	-0.933311368	5.40E-06	decrease
chr7	107891051	107891451	-1.606911783	6.11E-06	decrease
chr17	17972426	17972826	-1.452795877	6.20E-06	decrease
chr4	39527360	39527760	-1.609198337	6.36E-06	decrease
chr6	27791394	27791794	-0.767716218	6.51E-06	decrease
chr19	41397366	41397766	-1.529853958	6.57E-06	decrease
chr2	42810371	42810771	-1.381091992	6.87E-06	decrease
chr14	20454854	20455254	-0.873168627	6.94E-06	decrease
chr7	100586194	100586594	-1.427215251	7.33E-06	decrease
chr1	148522720	148523120	-1.290288618	7.53E-06	decrease
chr19	1082410	1082810	-1.102887644	7.59E-06	decrease

chr14	21269436	21269836	-1.260350823	7.62E-06	decrease
chr17	31549883	31550283	-1.548555601	7.80E-06	decrease
chr6	7389488	7389888	-1.390579916	7.85E-06	decrease
chr19	6424952	6425352	-1.157360685	7.89E-06	decrease
chr6	27251521	27251921	-1.213312491	8.04E-06	decrease
chr16	87560701	87561101	-1.593762405	8.10E-06	decrease
chr1	145281269	145281669	-1.047863217	8.29E-06	decrease
chr12	50111892	50112292	-1.267112232	8.53E-06	decrease
chr5	140725894	140726294	-0.69928826	8.85E-06	decrease
chr6	27547601	27548001	-0.692612957	8.90E-06	decrease
chr7	127651684	127652084	-1.709108914	8.97E-06	decrease
chr17	7614943	7615343	-1.345323917	8.98E-06	decrease
chr6	27605619	27606019	-0.688490382	9.14E-06	decrease
chr1	16678025	16678425	-1.061664816	9.16E-06	decrease
chr16	87388192	87388592	-1.21225639	9.49E-06	decrease
chr9	127224334	127224734	-1.796454525	9.58E-06	decrease
chr1	225777681	225778081	-1.203084896	1.04E-05	decrease
chr3	160449749	160450149	-1.170263728	1.06E-05	decrease
chr19	11155754	11156154	-1.350264622	1.09E-05	decrease
chr14	49586344	49586744	-0.743190131	1.10E-05	decrease
chr12	56190042	56190442	-0.770319112	1.11E-05	decrease
chr17	8119685	8120085	-0.709355184	1.12E-05	decrease
chr14	20461530	20461930	-1.128775435	1.14E-05	decrease
chr1	186375338	186375738	-1.147093892	1.15E-05	decrease
chr17	59106772	59107172	-1.426691881	1.15E-05	decrease
chr22	36388047	36388447	-1.360281275	1.15E-05	decrease
chr17	75979303	75979703	-1.238340566	1.18E-05	decrease
chr2	73233316	73233716	-1.180435798	1.18E-05	decrease
chr1	148264836	148265236	-0.952547342	1.21E-05	decrease
chr14	99481101	99481501	-1.513934814	1.25E-05	decrease
chr1	248873652	248874052	-0.960023394	1.28E-05	decrease
chr4	102828102	102828502	-1.185658225	1.29E-05	decrease
chr1	151281927	151282327	-0.928578285	1.30E-05	decrease
chr1	244864541	244864941	-1.33266939	1.30E-05	decrease
chr11	65615295	65615695	-1.099646619	1.33E-05	decrease
chr19	18152645	18153045	-1.260355096	1.33E-05	decrease
chr6	159761990	159762390	-1.105464721	1.35E-05	decrease
chr15	65862659	65863059	-1.407140473	1.37E-05	decrease
chr2	86195091	86195491	-1.584334276	1.40E-05	decrease
chr1	228646781	228647181	-0.704812521	1.43E-05	decrease
chr7	151028356	151028756	-1.021410982	1.46E-05	decrease
chr12	54280190	54280590	-1.045866656	1.51E-05	decrease
chr19	14529366	14529766	-1.400552582	1.54E-05	decrease
chr19	10717556	10717956	-1.258909811	1.56E-05	decrease
chr6	27617822	27618222	-0.825031246	1.57E-05	decrease
chr19	42242952	42243352	-1.147492734	1.58E-05	decrease
chr1	145859013	145859413	-1.221485237	1.61E-05	decrease
chr12	53379630	53380030	-1.082858414	1.65E-05	decrease
chr12	56300050	56300450	-1.203006881	1.72E-05	decrease
chr17	81860619	81861019	-1.331061541	1.72E-05	decrease
chr8	103414882	103415282	-1.657144212	1.76E-05	decrease
chr15	67254472	67254872	-1.18491101	1.79E-05	decrease
chr2	200889197	200889597	-1.1887227	1.86E-05	decrease
chr19	49877199	49877599	-0.888571951	1.87E-05	decrease
chr6	27235444	27235844	-0.763715058	1.88E-05	decrease
chr5	138543412	138543812	-1.261186732	1.88E-05	decrease
chr19	5680613	5681013	-1.249684006	1.89E-05	decrease
chr22	40856584	40856984	-1.31182793	1.94E-05	decrease
chr10	74826133	74826533	-1.187009872	1.94E-05	decrease
chr20	17968844	17969244	-1.320473277	1.98E-05	decrease
chr17	28951777	28952177	-1.137183888	2.02E-05	decrease
chr17	75034433	75034833	-0.663567967	2.07E-05	decrease
chr17	74872938	74873338	-1.349277979	2.09E-05	decrease
chr21	42892876	42893276	-1.607912697	2.10E-05	decrease
chr17	8176122	8176522	-0.827483696	2.10E-05	decrease
chr9	129080813	129081213	-1.517093825	2.12E-05	decrease
chr1	244652645	244653045	-1.440250167	2.12E-05	decrease
chr17	68019769	68020169	-0.90049299	2.17E-05	decrease
chr9	92325526	92325926	-1.570718114	2.19E-05	decrease
chr1	156338466	156338866	-0.93259965	2.19E-05	decrease
chr12	48682024	48682424	-1.207950254	2.28E-05	decrease
chr4	82900546	82900946	-1.52932249	2.30E-05	decrease
chr10	1048951	1049351	-1.49918657	2.35E-05	decrease
chr1	52366234	52366634	-1.476774729	2.36E-05	decrease
chr17	58517812	58518212	-1.020661497	2.39E-05	decrease
chr17	67717607	67718007	-1.203475449	2.40E-05	decrease
chr11	108222915	108223315	-1.248890418	2.59E-05	decrease

chr19	41263811	41264211	-1.204819593	2.61E-05	decrease
chr1	32013356	32013756	-1.658854672	2.71E-05	decrease
chr5	181169721	181170121	-0.774399242	2.78E-05	decrease
chr1	236795036	236795436	-1.227926802	2.85E-05	decrease
chr1	30718054	30718454	-1.352947645	2.86E-05	decrease
chr16	5097896	5098296	-1.26117412	2.87E-05	decrease
chr16	72093569	72093969	-1.383077482	2.93E-05	decrease
chr9	97633272	97633672	-1.410618887	2.95E-05	decrease
chr16	14285735	14286135	-0.902427325	2.99E-05	decrease
chr12	55973662	55974062	-1.080678508	2.99E-05	decrease
chr1	23959390	23959790	-1.671308517	3.00E-05	decrease
chr5	74767145	74767545	-1.471352525	3.03E-05	decrease
chr19	4791305	4791705	-1.170122769	3.07E-05	decrease
chr1	121016676	121017076	-1.013605129	3.08E-05	decrease
chr12	6554229	6554629	-1.002276153	3.27E-05	decrease
chr11	47767299	47767699	-1.209092367	3.37E-05	decrease
chr12	6533965	6534365	-1.036145575	3.43E-05	decrease
chr19	15418977	15419377	-1.196405468	3.60E-05	decrease
chrX	48958159	48958559	-1.34394794	3.62E-05	decrease
chr17	7560832	7561232	-1.192120157	3.76E-05	decrease
chr19	7871093	7871493	-1.525972358	3.76E-05	decrease
chr1	153658604	153659004	-1.222575368	3.78E-05	decrease
chr1	32072796	32073196	-1.267158007	3.84E-05	decrease
chr10	22229233	22229633	-0.974158444	3.90E-05	decrease
chrX	49269153	49269553	-1.143330515	4.07E-05	decrease
chr9	83980247	83980647	-1.232334033	4.20E-05	decrease
chr7	149635543	149635943	-0.775502556	4.23E-05	decrease
chr16	3124359	3124759	-1.054658044	4.24E-05	decrease
chr19	4471737	4472137	-1.024319884	4.24E-05	decrease
chr6	27091681	27092081	-0.857886669	4.35E-05	decrease
chr1	161621378	161621778	-0.870637549	4.35E-05	decrease
chr1	92785085	92785485	-1.240203683	4.37E-05	decrease
chr1	150629221	150629621	-0.851713156	4.43E-05	decrease
chr10	16817412	16817812	-1.468752796	4.51E-05	decrease
chrX	119852843	119853243	-1.274942076	4.57E-05	decrease
chr17	2401464	2401864	-1.291480386	4.64E-05	decrease
chr2	189441100	189441500	-1.400423679	4.64E-05	decrease
chr13	30870365	30870765	-1.065076235	4.75E-05	decrease
chrX	135022150	135022550	-1.559265695	4.77E-05	decrease
chr1	153633922	153634322	-1.05485304	4.78E-05	decrease
chr6	24666777	24667177	-1.609166896	4.80E-05	decrease
chr2	241315385	241315785	-1.298712432	4.86E-05	decrease
chr6	26026743	26027143	-1.316812931	4.86E-05	decrease
chr10	119080817	119081217	-1.45403594	4.90E-05	decrease
chr5	179698352	179698752	-1.074981755	4.99E-05	decrease
chr3	14948223	14948623	-1.61178459	5.01E-05	decrease
chr20	33993229	33993629	-1.214195638	5.03E-05	decrease
chr1	167936195	167936595	-1.227456823	5.07E-05	decrease
chr7	35800679	35801079	-1.518899522	5.10E-05	decrease
chr22	42615332	42615732	-1.001976535	5.20E-05	decrease
chr3	49929731	49930131	-1.457499602	5.24E-05	decrease
chr5	172006660	172007060	-1.337186	5.26E-05	decrease
chrX	103686724	103687124	-1.120338791	5.31E-05	decrease
chr16	589414	589814	-1.38445919	5.37E-05	decrease
chr19	12938187	12938587	-1.104388512	5.38E-05	decrease
chr2	175168121	175168521	-1.197004133	5.39E-05	decrease
chr1	32351116	32351516	-1.413787688	5.47E-05	decrease
chr7	44606471	44606871	-1.675404343	5.53E-05	decrease
chr19	1812425	1812825	-1.061655834	5.59E-05	decrease
chr1	161201951	161202351	-1.109717532	5.60E-05	decrease
chr9	35604150	35604550	-1.074859405	5.77E-05	decrease
chr14	105490823	105491223	-1.285830054	5.77E-05	decrease
chr12	56645967	56646367	-1.203257816	5.84E-05	decrease
chr19	7605500	7605900	-1.287146398	5.89E-05	decrease
chr21	33542604	33543004	-0.875188089	5.91E-05	decrease
chr1	231528261	231528661	-1.123318931	5.93E-05	decrease
chr14	24114609	24115009	-1.127018186	5.96E-05	decrease
chr7	156893269	156893669	-1.335260159	5.97E-05	decrease
chr1	224356793	224357193	-1.544717234	6.08E-05	decrease
chr1	67430480	67430880	-0.921030709	6.11E-05	decrease
chr21	39183473	39183873	-1.216302583	6.15E-05	decrease
chr3	130111562	130111962	-1.329098529	6.24E-05	decrease
chr6	27280249	27280649	-0.720369697	6.29E-05	decrease
chr15	66497758	66498158	-0.862186635	6.29E-05	decrease
chr2	44168108	44168508	-1.139117945	6.30E-05	decrease
chrX	149939796	149940196	-1.136560935	6.32E-05	decrease
chr19	45584408	45584808	-1.062676897	6.35E-05	decrease

chr9	34646795	34647195	-1.244413214	6.43E-05	decrease
chr4	3074260	3074660	-1.383275	6.45E-05	decrease
chr1	225924056	225924456	-1.415946872	6.49E-05	decrease
chr19	17469879	17470279	-1.217795522	6.55E-05	decrease
chr19	55385354	55385754	-1.133781928	6.60E-05	decrease
chr19	58475815	58476215	-1.04543771	6.60E-05	decrease
chr10	97498313	97498713	-1.366238178	6.63E-05	decrease
chr8	123274538	123274938	-1.332837147	6.65E-05	decrease
chr3	125915893	125916293	-1.026836027	6.81E-05	decrease
chr16	3172048	3172448	-0.650374408	6.84E-05	decrease
chr16	3174165	3174565	-0.844568586	6.86E-05	decrease
chr19	16659878	16660278	-1.112702294	6.87E-05	decrease
chr19	17865598	17865998	-1.071447099	6.87E-05	decrease
chr1	161177436	161177836	-1.191612003	7.03E-05	decrease
chr6	24775008	24775408	-1.328161617	7.04E-05	decrease
chr11	64759663	64760063	-1.273816545	7.07E-05	decrease
chr1	150067356	150067756	-1.182950862	7.18E-05	decrease
chr14	20657382	20657782	-0.674556992	7.18E-05	decrease
chr1	154936867	154937267	-0.951587243	7.35E-05	decrease
chrX	18674818	18675218	-0.897413879	7.46E-05	decrease
chr19	48400233	48400633	-1.152800708	7.62E-05	decrease
chr11	62841088	62841488	-0.87100278	7.72E-05	decrease
chr14	74493096	74493496	-1.591795125	8.02E-05	decrease
chr6	43013889	43014289	-1.247844428	8.11E-05	decrease
chr2	676621	677021	-1.184055809	8.27E-05	decrease
chr2	32063408	32063808	-1.612570558	8.50E-05	decrease
chr7	151227184	151227584	-1.231386426	8.52E-05	decrease
chr17	75904977	75905377	-1.341978707	8.65E-05	decrease
chr1	211675542	211675942	-1.133701132	8.72E-05	decrease
chr7	101244735	101245135	-1.126834883	8.77E-05	decrease
chr14	32201599	32201999	-0.715872487	8.85E-05	decrease
chr6	88963438	88963838	-1.530429028	9.18E-05	decrease
chr17	63773726	63774126	-1.140365932	9.37E-05	decrease
chr1	222464975	222465375	-0.910305563	9.46E-05	decrease
chr16	29811176	29811576	-0.859327299	9.57E-05	decrease
chr6	27602347	27602747	-0.569339814	9.65E-05	decrease
chrX	149938362	149938762	-1.291680835	9.66E-05	decrease
chr14	105300985	105301385	-1.352108062	9.66E-05	decrease
chr1	234724095	234724495	-1.089695676	9.77E-05	decrease
chrX	153794060	153794460	-1.295548495	1.00E-04	decrease
chr14	58239743	58240143	-0.870871264	1.01E-04	decrease
chr12	103965684	103966084	-1.197091975	1.01E-04	decrease
chr15	65296234	65296634	-1.073081739	1.03E-04	decrease
chr1	143735846	143736246	-0.849427203	1.04E-04	decrease
chr1	25430289	25430689	-1.555944113	1.05E-04	decrease
chr4	56435639	56436039	-0.987026078	1.06E-04	decrease
chr2	74482982	74483382	-1.202334539	1.07E-04	decrease
chr12	122500839	122501239	-0.967308546	1.08E-04	decrease
chr6	37819083	37819483	-1.15855075	1.08E-04	decrease
chr6	28212731	28213131	-0.801122316	1.09E-04	decrease
chr1	150363719	150364119	-1.099506782	1.10E-04	decrease
chr17	18183400	18183800	-1.357034484	1.10E-04	decrease
chr3	44338235	44338635	-1.337333399	1.10E-04	decrease
chr17	43907491	43907891	-1.560091453	1.10E-04	decrease
chr15	100602247	100602647	-1.169933114	1.11E-04	decrease
chr5	34929231	34929631	-1.615665097	1.11E-04	decrease
chrX	120560904	120561304	-1.400133422	1.12E-04	decrease
chr3	136196504	136196904	-1.277060773	1.12E-04	decrease
chr17	75855365	75855765	-1.254023251	1.13E-04	decrease
chr7	21427374	21427774	-1.575538224	1.13E-04	decrease
chr7	139359520	139359920	-1.06340627	1.13E-04	decrease
chr12	56117891	56118291	-1.106975469	1.13E-04	decrease
chr3	156554922	156555322	-1.454101221	1.14E-04	decrease
chr4	3955565	3955965	-1.104313535	1.14E-04	decrease
chr1	185157044	185157444	-1.001059324	1.16E-04	decrease
chr13	73058728	73059128	-1.398541119	1.19E-04	decrease
chr14	35122156	35122556	-1.304981567	1.20E-04	decrease
chr1	204516072	204516472	-0.934344696	1.21E-04	decrease
chr1	108746458	108746858	-1.292304542	1.23E-04	decrease
chr19	6737204	6737604	-1.254180622	1.27E-04	decrease
chr1	161225530	161225930	-1.061827491	1.27E-04	decrease
chr19	48637496	48637896	-1.189455368	1.28E-04	decrease
chr16	1983609	1984009	-1.471722049	1.29E-04	decrease
chr11	78188438	78188838	-1.338014925	1.29E-04	decrease
chr17	38845661	38846061	-1.465836132	1.29E-04	decrease
chr20	35454750	35455150	-1.08894084	1.32E-04	decrease
chr11	63681338	63681738	-1.622490816	1.33E-04	decrease

chr19	12736977	12737377	-0.837823952	1.33E-04	decrease
chr1	32817351	32817751	-0.962439044	1.33E-04	decrease
chr19	49866927	49867327	-1.084991163	1.34E-04	decrease
chr17	7252074	7252474	-1.110697629	1.34E-04	decrease
chr11	3422724	3423124	-1.012847138	1.35E-04	decrease
chr1	205749323	205749723	-1.132693797	1.36E-04	decrease
chr17	2399661	2400061	-1.278694042	1.38E-04	decrease
chr5	177426172	177426572	-1.06403405	1.39E-04	decrease
chr1	11908316	11908716	-1.048718182	1.40E-04	decrease
chr17	81346241	81346641	-1.618137324	1.40E-04	decrease
chr6	26457694	26458094	-1.056684168	1.40E-04	decrease
chr19	2236325	2236725	-1.277590646	1.41E-04	decrease
chr19	12681166	12681566	-0.981099353	1.42E-04	decrease
chr11	66616250	66616650	-1.115276255	1.44E-04	decrease
chr16	3175393	3175793	-0.694323953	1.45E-04	decrease
chr7	10940117	10940517	-1.296960751	1.47E-04	decrease
chr17	4710065	4710465	-1.469156842	1.49E-04	decrease
chrX	54808528	54808928	-1.284638313	1.50E-04	decrease
chr7	135557778	135558178	-1.239740656	1.50E-04	decrease
chr1	202888767	202889167	-1.247419426	1.51E-04	decrease
chr19	49443429	49443829	-1.234407605	1.54E-04	decrease
chr17	75240485	75240885	-1.248479911	1.55E-04	decrease
chr1	23368505	23368905	-1.345240919	1.56E-04	decrease
chr3	169772580	169772980	-0.841162263	1.57E-04	decrease
chr16	28823965	28824365	-1.267218981	1.57E-04	decrease
chr1	85276446	85276846	-1.152281415	1.59E-04	decrease
chr1	235161015	235161415	-1.161516094	1.59E-04	decrease
chr12	121839350	121839750	-1.157748532	1.60E-04	decrease
chr1	28643138	28643538	-1.389835236	1.65E-04	decrease
chr20	35616333	35616733	-1.067461403	1.66E-04	decrease
chr7	2354214	2354614	-1.19537321	1.71E-04	decrease
chr18	36129458	36129858	-1.063481418	1.72E-04	decrease
chr19	43192106	43192506	-0.969538357	1.73E-04	decrease
chr11	35526459	35526859	-0.690548904	1.73E-04	decrease
chr8	38231210	38231610	-1.5791245	1.73E-04	decrease
chr7	72828059	72828459	-1.180488132	1.74E-04	decrease
chr5	140647589	140647989	-0.97569742	1.75E-04	decrease
chr12	52069614	52070014	-1.186777989	1.78E-04	decrease
chr1	144418933	144419333	-0.80443269	1.79E-04	decrease
chr16	31459431	31459831	-1.513168228	1.81E-04	decrease
chr22	41589793	41590193	-1.150582785	1.81E-04	decrease
chr20	35699296	35699696	-1.298298249	1.82E-04	decrease
chr19	45779364	45779764	-1.181048192	1.83E-04	decrease
chr1	26233970	26234370	-1.524540259	1.84E-04	decrease
chr1	225845710	225846110	-1.078106864	1.84E-04	decrease
chr11	67996850	67997250	-1.128613814	1.86E-04	decrease
chr3	9792705	9793105	-1.017315354	1.86E-04	decrease
chr7	151057838	151058238	-0.969670509	1.87E-04	decrease
chr19	50329305	50329705	-0.982353739	1.89E-04	decrease
chr2	112764418	112764818	-1.496186231	1.89E-04	decrease
chr11	64185764	64186164	-1.126255741	1.91E-04	decrease
chr6	27583823	27584223	-1.00273748	1.92E-04	decrease
chr12	123633529	123633929	-1.145314138	1.92E-04	decrease
chr17	76737384	76737784	-1.099224247	1.94E-04	decrease
chr2	241355262	241355662	-1.041007711	1.96E-04	decrease
chr17	28711391	28711791	-1.330311289	1.98E-04	decrease
chrX	47218029	47218429	-0.990088434	2.02E-04	decrease
chr6	109382543	109382943	-1.061520776	2.06E-04	decrease
chrX	47650597	47650997	-1.208932583	2.08E-04	decrease
chr22	39311216	39311616	-0.898928016	2.08E-04	decrease
chr8	25458443	25458843	-1.131596135	2.10E-04	decrease
chr11	65040832	65041232	-1.357704741	2.11E-04	decrease
chr1	149927725	149928125	-1.345992545	2.14E-04	decrease
chrX	41333952	41334352	-1.266035334	2.15E-04	decrease
chr3	52287726	52288126	-1.164142882	2.16E-04	decrease
chr17	75012598	75012998	-1.040797466	2.17E-04	decrease
chr6	27693667	27694067	-0.873385958	2.20E-04	decrease
chr14	20652981	20653381	-0.613071849	2.23E-04	decrease
chr11	73779461	73779861	-1.257269115	2.26E-04	decrease
chr6	152983309	152983709	-0.952240583	2.26E-04	decrease
chr14	22919167	22919567	-0.955796138	2.27E-04	decrease
chr2	33599161	33599561	-1.189112356	2.30E-04	decrease
chr1	153967448	153967848	-0.97268594	2.30E-04	decrease
chr6	27146850	27147250	-0.667591426	2.35E-04	decrease
chr9	37120166	37120566	-1.371162695	2.36E-04	decrease
chr19	49453154	49453554	-1.007206057	2.36E-04	decrease
chr1	149636489	149636889	-1.119763237	2.37E-04	decrease

chr6	24720858	24721258	-0.975358099	2.42E-04	decrease
chr7	44582133	44582533	-1.25885417	2.42E-04	decrease
chr6	136250184	136250584	-1.028361013	2.44E-04	decrease
chr6	36874671	36875071	-1.42285365	2.44E-04	decrease
chr2	47335068	47335468	-1.051903255	2.44E-04	decrease
chr9	6006965	6007365	-1.464786922	2.44E-04	decrease
chr19	36054202	36054602	-1.017308943	2.46E-04	decrease
chr20	18467093	18467493	-1.343609273	2.47E-04	decrease
chr1	193059461	193059861	-1.151849311	2.49E-04	decrease
chr20	43514165	43514565	-0.943718586	2.49E-04	decrease
chr15	65517388	65517788	-1.191829443	2.49E-04	decrease
chr1	37690295	37690695	-1.257238185	2.50E-04	decrease
chr9	127396644	127397044	-1.075194894	2.51E-04	decrease
chr6	27752746	27753146	-0.720524441	2.51E-04	decrease
chr2	86105684	86106084	-1.141223077	2.54E-04	decrease
chr1	229558924	229559324	-1.141259433	2.57E-04	decrease
chr16	2882682	2883082	-1.071825176	2.57E-04	decrease
chr10	130110617	130111017	-1.091957896	2.58E-04	decrease
chr5	178153776	178154176	-1.135829014	2.58E-04	decrease
chr17	42388481	42388881	-1.128870063	2.59E-04	decrease
chr10	102132612	102133012	-1.119996701	2.61E-04	decrease
chr2	200811492	200811892	-1.209181922	2.62E-04	decrease
chr4	77818001	77818401	-1.519145565	2.67E-04	decrease
chr1	93180115	93180515	-0.963313624	2.67E-04	decrease
chr21	42974038	42974438	-1.10900063	2.69E-04	decrease
chr3	42600288	42600688	-1.180714896	2.71E-04	decrease
chr1	156725874	156726274	-1.23993682	2.73E-04	decrease
chr15	66356567	66356967	-1.301018614	2.74E-04	decrease
chr6	27472993	27473393	-0.680059973	2.75E-04	decrease
chrX	154547244	154547644	-1.219171699	2.78E-04	decrease
chr10	68720988	68721388	-1.274488628	2.79E-04	decrease
chr4	169620110	169620510	-1.29737395	2.81E-04	decrease
chr4	9152843	9153243	-1.173764549	2.84E-04	decrease
chr9	97039189	97039589	-1.387502955	2.85E-04	decrease
chr1	169893921	169894321	-1.028277653	2.92E-04	decrease
chr1	62436414	62436814	-0.890113801	2.97E-04	decrease
chr17	75393778	75394178	-1.132461944	2.98E-04	decrease
chr12	98644507	98644907	-1.253414589	2.98E-04	decrease
chr7	65750605	65751005	-1.320475693	3.00E-04	decrease
chr17	4940214	4940614	-1.130209831	3.03E-04	decrease
chr19	18203998	18204398	-1.185913911	3.06E-04	decrease
chr6	27237308	27237708	-0.635778869	3.08E-04	decrease
chr12	123232868	123233268	-1.112389261	3.13E-04	decrease
chr6	27290553	27290953	-0.680220521	3.14E-04	decrease
chr15	75450486	75450886	-0.95162724	3.19E-04	decrease
chr3	52706009	52706409	-1.2263368	3.21E-04	decrease
chr12	49967233	49967633	-1.351054828	3.23E-04	decrease
chr6	15245540	15245940	-1.291568445	3.27E-04	decrease
chr16	29863199	29863599	-1.038549786	3.28E-04	decrease
chr3	48556893	48557293	-1.057357868	3.30E-04	decrease
chr3	179562683	179563083	-1.333406852	3.30E-04	decrease
chr6	151451996	151452396	-1.235080574	3.32E-04	decrease
chr17	8295356	8295756	-0.905504909	3.34E-04	decrease
chr13	94595970	94596370	-1.036613637	3.34E-04	decrease
chr19	39391036	39391436	-1.01116252	3.35E-04	decrease
chr19	47515140	47515540	-1.232246056	3.36E-04	decrease
chr4	75724383	75724783	-1.089412124	3.36E-04	decrease
chr1	173868103	173868503	-0.838993944	3.39E-04	decrease
chr7	135510053	135510453	-0.969463439	3.40E-04	decrease
chr5	57173772	57174172	-1.143046451	3.43E-04	decrease
chr1	117059950	117060350	-1.054550985	3.45E-04	decrease
chr2	135741578	135741978	-1.257640614	3.47E-04	decrease
chr12	6470695	6471095	-1.226833577	3.47E-04	decrease
chr17	7579136	7579536	-0.852896942	3.48E-04	decrease
chr6	53061713	53062113	-1.239880233	3.51E-04	decrease
chr13	75549417	75549817	-0.936657442	3.52E-04	decrease
chr1	66924926	66925326	-0.921269618	3.53E-04	decrease
chr16	30698124	30698524	-1.041645439	3.54E-04	decrease
chr19	40570628	40571028	-1.151912469	3.56E-04	decrease
chr1	228647665	228648065	-0.926291712	3.57E-04	decrease
chr22	41468730	41469130	-1.072120473	3.58E-04	decrease
chr5	69189149	69189549	-1.511222405	3.59E-04	decrease
chr15	72117440	72117840	-1.210407722	3.60E-04	decrease
chr6	27651177	27651577	-0.682876366	3.60E-04	decrease
chr1	234373216	234373616	-1.02157163	3.61E-04	decrease
chr12	49088301	49088701	-1.298093664	3.64E-04	decrease
chr2	130342363	130342763	-0.834866514	3.67E-04	decrease

chr6	26103657	26104057	-1.028352916	3.70E-04	decrease
chr12	84911219	84911619	-1.33304226	3.71E-04	decrease
chr5	176447993	176448393	-1.430406289	3.71E-04	decrease
chr17	7572390	7572790	-0.971074149	3.74E-04	decrease
chr14	67816378	67816778	-1.341924735	3.76E-04	decrease
chr14	20413085	20413485	-0.949608278	3.78E-04	decrease
chr10	73246965	73247365	-1.148328866	3.79E-04	decrease
chr14	67600028	67600428	-1.237323619	3.80E-04	decrease
chr17	58007231	58007631	-1.130204639	3.84E-04	decrease
chr19	7943629	7944029	-1.05371551	3.86E-04	decrease
chr1	149886606	149887006	-0.843352322	3.91E-04	decrease
chr11	66638224	66638624	-1.16418633	3.92E-04	decrease
chr16	29877432	29877832	-0.988988825	3.94E-04	decrease
chr16	1610670	1611070	-1.327670218	3.94E-04	decrease
chr16	87950952	87951352	-1.041642089	3.95E-04	decrease
chr12	21501473	21501873	-1.281811551	3.95E-04	decrease
chr12	122974702	122975102	-1.128114348	3.96E-04	decrease
chr15	75453671	75454071	-0.987613338	3.96E-04	decrease
chr11	66312027	66312427	-1.051592438	3.97E-04	decrease
chr18	32018472	32018872	-0.963996935	3.97E-04	decrease
chr17	16438646	16439046	-1.019380587	3.97E-04	decrease
chr15	69462628	69463028	-1.130407927	3.98E-04	decrease
chr3	101724570	101724970	-1.061561065	4.00E-04	decrease
chr5	75336668	75337068	-1.101630874	4.03E-04	decrease
chrX	48801947	48802347	-0.947627013	4.05E-04	decrease
chr1	28369541	28369941	-1.135718037	4.05E-04	decrease
chr2	32165503	32165903	-1.352251587	4.11E-04	decrease
chr14	39432410	39432810	-1.214437034	4.12E-04	decrease
chr10	97407255	97407655	-1.168582382	4.13E-04	decrease
chr20	3173645	3174045	-1.416562742	4.15E-04	decrease
chr1	47314043	47314443	-0.9663958	4.15E-04	decrease
chr1	161166162	161166562	-0.739006699	4.16E-04	decrease
chr2	44361555	44361955	-1.390528727	4.18E-04	decrease
chr17	62065687	62066087	-1.449034881	4.18E-04	decrease
chr12	71663787	71664187	-1.054673207	4.23E-04	decrease
chr12	56636512	56636912	-0.971246132	4.23E-04	decrease
chr17	40100357	40100757	-1.281427238	4.24E-04	decrease
chr19	9819067	9819467	-1.190225863	4.25E-04	decrease
chr2	38603098	38603498	-1.480397587	4.30E-04	decrease
chr6	85643073	85643473	-1.276849397	4.32E-04	decrease
chr1	113389936	113390336	-1.16806706	4.33E-04	decrease
chr1	155084861	155085261	-0.9710856	4.33E-04	decrease
chr6	26157698	26158098	-0.75186371	4.34E-04	decrease
chr1	149887960	149888360	-0.962915913	4.35E-04	decrease
chr2	218568408	218568808	-1.075741553	4.37E-04	decrease
chr14	58297915	58298315	-1.001878362	4.37E-04	decrease
chr11	66344844	66345244	-1.176501863	4.38E-04	decrease
chr3	17742811	17743211	-1.430545889	4.41E-04	decrease
chr6	27188443	27188843	-0.899788754	4.42E-04	decrease
chr11	83156680	83157080	-1.098043299	4.42E-04	decrease
chr1	226002627	226003027	-1.141910822	4.45E-04	decrease
chr19	42075829	42076229	-1.288085084	4.48E-04	decrease
chr5	151252988	151253388	-1.420843705	4.49E-04	decrease
chr17	8125466	8125866	-0.594222274	4.49E-04	decrease
chr1	179954343	179954743	-1.115904497	4.51E-04	decrease
chr13	30673909	30674309	-0.987098741	4.53E-04	decrease
chr19	40348504	40348904	-1.461728976	4.57E-04	decrease
chr2	99154844	99155244	-1.42734363	4.59E-04	decrease
chr7	152675750	152676150	-1.333163057	4.60E-04	decrease
chr19	58326997	58327397	-1.052649067	4.61E-04	decrease
chr1	150236036	150236436	-0.948223088	4.63E-04	decrease
chr5	141923761	141924161	-1.034653216	4.64E-04	decrease
chr11	123062165	123062565	-1.004823472	4.65E-04	decrease
chr2	26785674	26786074	-1.417114384	4.66E-04	decrease
chr5	138464971	138465371	-1.203661668	4.67E-04	decrease
chr17	39980385	39980785	-1.170995548	4.70E-04	decrease
chr3	50569085	50569485	-1.145425609	4.71E-04	decrease
chr6	34249187	34249587	-1.439239162	4.73E-04	decrease
chr16	30407214	30407614	-1.257885881	4.75E-04	decrease
chr6	27772286	27772686	-0.867409745	4.75E-04	decrease
chr2	85538744	85539144	-1.181793228	4.75E-04	decrease
chr4	13484351	13484751	-1.270444948	4.76E-04	decrease
chr17	32349931	32350331	-1.032415491	4.76E-04	decrease
chr6	110179708	110180108	-1.15508017	4.85E-04	decrease
chr19	47447305	47447705	-1.126909843	4.85E-04	decrease
chr4	112637114	112637514	-1.224950873	4.89E-04	decrease
chr18	12377351	12377751	-1.298776749	4.90E-04	decrease

chr4	88284675	88285075	-1.239175311	4.91E-04	decrease
chr5	178120772	178121172	-1.323132892	4.91E-04	decrease
chr5	132293662	132294062	-1.191016095	4.92E-04	decrease
chr10	68988698	68989098	-1.127439237	4.94E-04	decrease
chr7	149646879	149647279	-0.818233006	4.95E-04	decrease
chr4	82891209	82891609	-1.296715396	4.96E-04	decrease
chr1	16613555	16613955	-0.887921501	5.01E-04	decrease
chr21	37267506	37267906	-1.03393762	5.02E-04	decrease
chr16	28950554	28950954	-1.245217051	5.02E-04	decrease
chr6	80106340	80106740	-1.405391039	5.04E-04	decrease
chr3	49007312	49007712	-0.944752284	5.11E-04	decrease
chr12	68209448	68209848	-1.266439595	5.12E-04	decrease
chr12	67268807	67269207	-1.033050168	5.15E-04	decrease
chr9	79571537	79571937	-0.884059276	5.16E-04	decrease
chr3	57755796	57756196	-1.260708638	5.19E-04	decrease
chr17	8288381	8288781	-1.073249566	5.19E-04	decrease
chr19	49533798	49534198	-1.313843731	5.22E-04	decrease
chr1	148038501	148038901	-1.042537815	5.23E-04	decrease
chr19	2273458	2273858	-1.205775012	5.26E-04	decrease
chr7	73522307	73522707	-1.035166713	5.30E-04	decrease
chr17	8630673	8631073	-1.262395594	5.30E-04	decrease
chr11	73876661	73877061	-1.139730376	5.32E-04	decrease
chrX	129905792	129906192	-1.37428554	5.34E-04	decrease
chr19	4327399	4327799	-1.366936515	5.34E-04	decrease
chr17	5419732	5420132	-0.995676523	5.40E-04	decrease
chr16	90077227	90077627	-1.189464935	5.46E-04	decrease
chr4	87391008	87391408	-1.352836911	5.46E-04	decrease
chr16	57735265	57735665	-1.205779181	5.47E-04	decrease
chr1	151539948	151540348	-1.082483728	5.49E-04	decrease
chr2	224585674	224586074	-1.295568056	5.53E-04	decrease
chr12	2812639	2813039	-1.116972973	5.54E-04	decrease
chr5	138337816	138338216	-1.130107242	5.56E-04	decrease
chr6	26123747	26124147	-0.76367632	5.63E-04	decrease
chr6	26553645	26554045	-0.546145817	5.64E-04	decrease
chr17	75205315	75205715	-0.867215308	5.73E-04	decrease
chr1	42767066	42767466	-1.240803349	5.73E-04	decrease
chr19	10194889	10195289	-1.286277796	5.84E-04	decrease
chr2	162343955	162344355	-1.319668158	5.86E-04	decrease
chr19	49128159	49128559	-1.259410224	5.89E-04	decrease
chr6	27575880	27576280	-0.713652793	5.94E-04	decrease
chr7	100538827	100539227	-1.047703758	5.94E-04	decrease
chr9	129110838	129111238	-1.411659077	5.95E-04	decrease
chr1	202341811	202342211	-1.06041131	5.96E-04	decrease
chr11	73787843	73788243	-1.178786582	5.97E-04	decrease
chr9	131373464	131373864	-1.158647502	5.98E-04	decrease
chr6	125780285	125780685	-0.866999154	5.98E-04	decrease
chr3	48635434	48635834	-1.074883751	5.99E-04	decrease
chr9	128159788	128160188	-1.077035514	6.01E-04	decrease
chr1	172532475	172532875	-0.977544329	6.02E-04	decrease
chr7	148966872	148967272	-0.838603445	6.03E-04	decrease
chr6	43170776	43171176	-1.017928026	6.07E-04	decrease
chr16	81006896	81007296	-0.979428124	6.10E-04	decrease
chr6	27838163	27838563	-0.840586931	6.17E-04	decrease
chr14	63641609	63642009	-1.11761761	6.19E-04	decrease
chr1	212857630	212858030	-1.081847327	6.21E-04	decrease
chr17	63741620	63742020	-1.031820199	6.24E-04	decrease
chr3	129121849	129122249	-1.392730898	6.27E-04	decrease
chr6	10694332	10694732	-1.303892905	6.29E-04	decrease
chr5	140664546	140664946	-0.914182029	6.31E-04	decrease
chr15	65792268	65792668	-1.064838865	6.35E-04	decrease
chr10	84139324	84139724	-1.432588122	6.36E-04	decrease
chr4	125314820	125315220	-1.320115111	6.37E-04	decrease
chr17	78140336	78140736	-0.961605516	6.39E-04	decrease
chr1	151790461	151790861	-1.158419058	6.47E-04	decrease
chr19	45469600	45470000	-0.979930868	6.49E-04	decrease
chr4	56387096	56387496	-1.149794497	6.53E-04	decrease
chr17	7307084	7307484	-0.866964185	6.54E-04	decrease
chr10	132537427	132537827	-1.333757381	6.55E-04	decrease
chr3	159988401	159988801	-1.446101354	6.56E-04	decrease
chr2	96266182	96266582	-1.162443794	6.58E-04	decrease
chr9	128419346	128419746	-1.386235874	6.61E-04	decrease
chr2	148020392	148020792	-1.217984377	6.64E-04	decrease
chr6	28335572	28335972	-0.966222426	6.68E-04	decrease
chr2	86563013	86563413	-1.341481093	6.70E-04	decrease
chr16	46689277	46689677	-0.900576049	6.71E-04	decrease
chr17	7844683	7845083	-1.079260491	6.73E-04	decrease
chr1	121184521	121184921	-1.089716489	6.73E-04	decrease

chr3	75435210	75435610	-1.081023001	6.77E-04	decrease
chrX	154378755	154379155	-1.01411575	6.80E-04	decrease
chr1	94417916	94418316	-1.186501586	6.81E-04	decrease
chr1	183471679	183472079	-0.889290236	6.86E-04	decrease
chr15	90717268	90717668	-1.132753635	6.89E-04	decrease
chr16	30378189	30378589	-0.889490818	6.95E-04	decrease
chr1	226272349	226272749	-1.018654646	6.95E-04	decrease
chr1	173824500	173824900	-0.903564162	6.97E-04	decrease
chr1	202926980	202927380	-1.106669511	6.97E-04	decrease
chr4	165112819	165113219	-1.235884975	7.00E-04	decrease
chr2	69829243	69829643	-1.243639227	7.03E-04	decrease
chr11	68030496	68030896	-1.152972205	7.10E-04	decrease
chr11	86244439	86244839	-1.173485644	7.12E-04	decrease
chr14	24141538	24141938	-1.010632419	7.13E-04	decrease
chr9	127771262	127771662	-1.151251812	7.15E-04	decrease
chr11	72080551	72080951	-1.165363349	7.15E-04	decrease
chrX	78103788	78104188	-0.989489586	7.17E-04	decrease
chr5	119268149	119268549	-1.298699396	7.19E-04	decrease
chr1	36149349	36149749	-1.163180174	7.26E-04	decrease
chr1	32205535	32205935	-1.055316056	7.27E-04	decrease
chr1	32394396	32394796	-0.801882857	7.28E-04	decrease
chr19	38899502	38899902	-0.901491824	7.29E-04	decrease
chr17	63827288	63827688	-0.867117205	7.29E-04	decrease
chr3	139389643	139390043	-1.187072012	7.30E-04	decrease
chr2	24047274	24047674	-1.123194255	7.35E-04	decrease
chr19	36489401	36489801	-1.110643503	7.35E-04	decrease
chr20	4023971	4024371	-1.410002009	7.36E-04	decrease
chr6	166956592	166956992	-1.304837834	7.36E-04	decrease
chr1	74197953	74198353	-1.251913319	7.37E-04	decrease
chr3	14124880	14125280	-1.212729385	7.38E-04	decrease
chr22	40951298	40951698	-1.055195259	7.39E-04	decrease
chr11	64304398	64304798	-1.112420558	7.44E-04	decrease
chr4	82374007	82374407	-0.9522993	7.47E-04	decrease
chr2	65130070	65130470	-1.170340047	7.49E-04	decrease
chr10	102502516	102502916	-0.894059862	7.51E-04	decrease
chr6	26383005	26383405	-1.044720131	7.53E-04	decrease
chr5	109410011	109410411	-1.054413924	7.54E-04	decrease
chr17	8127001	8127401	-0.72221955	7.57E-04	decrease
chr12	121399820	121400220	-1.167749549	7.57E-04	decrease
chr2	86442509	86442909	-1.245507044	7.57E-04	decrease
chr6	42890650	42891050	-1.022132114	7.58E-04	decrease
chrX	53684073	53684473	-1.114206874	7.58E-04	decrease
chr1	32650677	32651077	-0.888964226	7.60E-04	decrease
chr7	139230900	139231300	-1.128124487	7.62E-04	decrease
chr1	183635771	183636171	-0.960964154	7.62E-04	decrease
chr22	39523034	39523434	-0.995747767	7.64E-04	decrease
chr2	55519315	55519715	-1.103977659	7.69E-04	decrease
chr4	3075247	3075647	-1.332093513	7.70E-04	decrease
chr5	163437442	163437842	-1.155431408	7.71E-04	decrease
chr22	41444290	41444690	-1.115644015	7.76E-04	decrease
chr3	9396814	9397214	-0.941025208	7.76E-04	decrease
chr5	146182687	146183087	-1.120854742	7.82E-04	decrease
chr10	123154145	123154545	-1.296990658	7.85E-04	decrease
chr1	32464938	32465338	-1.258760037	7.90E-04	decrease
chr1	154220135	154220535	-1.103177326	7.91E-04	decrease
chr1	45583634	45584034	-0.934487802	7.91E-04	decrease
chr1	44730817	44731217	-0.828799913	7.93E-04	decrease
chr22	41381476	41381876	-1.139654823	7.94E-04	decrease
chr19	49640332	49640732	-0.816532627	8.02E-04	decrease
chr1	44721333	44721733	-1.019910435	8.05E-04	decrease
chr17	8383215	8383615	-1.12479468	8.07E-04	decrease
chr19	56314761	56315161	-1.186583258	8.07E-04	decrease
chr19	14136660	14137060	-1.176628647	8.14E-04	decrease
chr1	161726188	161726588	-0.953828722	8.20E-04	decrease
chr22	31661932	31662332	-1.290436181	8.22E-04	decrease
chr9	133459911	133460311	-1.369013461	8.25E-04	decrease
chr2	239401341	239401741	-1.27101901	8.25E-04	decrease
chr1	248905883	248906283	-1.319246071	8.28E-04	decrease
chr16	30875073	30875473	-0.970743592	8.30E-04	decrease
chr21	36156341	36156741	-1.173717412	8.31E-04	decrease
chr16	21952969	21953369	-0.864960544	8.32E-04	decrease
chr19	49929716	49930116	-0.876865926	8.35E-04	decrease
chr7	139133537	139133937	-1.228010582	8.38E-04	decrease
chr3	143971404	143971804	-1.317181689	8.38E-04	decrease
chr11	65570040	65570440	-1.05469274	8.38E-04	decrease
chr1	150926000	150926400	-1.168363217	8.42E-04	decrease
chr19	45770329	45770729	-0.875632031	8.46E-04	decrease

chr17	41579444	41580092	-1.353511314	8.47E-04	decrease
chr5	109409301	109409701	-1.346846543	8.48E-04	decrease
chr14	81221025	81221425	-1.3969638	8.49E-04	decrease
chr3	123585152	123585552	-1.122399874	8.52E-04	decrease
chr6	26421240	26421640	-0.876879804	8.54E-04	decrease
chr7	152463741	152464141	-1.340854242	8.54E-04	decrease
chr5	90558432	90558832	-1.159373316	8.57E-04	decrease
chr1	241847835	241848235	-0.897341038	8.60E-04	decrease
chr12	113185426	113185826	-1.256783923	8.61E-04	decrease
chr6	111483758	111484158	-1.306160963	8.66E-04	decrease
chr19	50658166	50658566	-0.96708455	8.69E-04	decrease
chrX	48521441	48521841	-1.298006316	8.75E-04	decrease
chr1	71081004	71081404	-1.053884951	8.75E-04	decrease
chr19	5720271	5720671	-1.016281747	8.76E-04	decrease
chr16	53703721	53704121	-1.248692195	8.79E-04	decrease
chr10	110871597	110871997	-1.255331357	8.85E-04	decrease
chr19	48321309	48321709	-0.990397441	8.86E-04	decrease
chr1	210373843	210374243	-0.91299245	8.87E-04	decrease
chr5	160119498	160119898	-1.125674407	8.99E-04	decrease
chr7	134646550	134646950	-1.013735521	9.00E-04	decrease
chr1	161209765	161210165	-1.137478101	9.01E-04	decrease
chrX	101407738	101408138	-1.151504502	9.02E-04	decrease
chr2	219552298	219552698	-1.133027244	9.02E-04	decrease
chr19	48619211	48619611	-1.144438594	9.05E-04	decrease
chr19	17579846	17580246	-1.048704766	9.07E-04	decrease
chr7	76379889	76380289	-1.204521199	9.09E-04	decrease
chr17	48048026	48048426	-1.131692687	9.09E-04	decrease
chr1	70221320	70221720	-1.132685872	9.11E-04	decrease
chr17	7835231	7835631	-1.010309687	9.15E-04	decrease
chr2	218270088	218270488	-1.04935493	9.15E-04	decrease
chr1	161566037	161566437	-0.951243336	9.17E-04	decrease
chr5	140401202	140401602	-0.776506885	9.21E-04	decrease
chr22	40636859	40637259	-1.255870515	9.26E-04	decrease
chr19	50511082	50511482	-1.19885301	9.29E-04	decrease
chr10	99620537	99620937	-1.245245389	9.31E-04	decrease
chr22	27919475	27919875	-1.152919834	9.45E-04	decrease
chr5	108748701	108749101	-1.028062366	9.61E-04	decrease
chr3	51499272	51499672	-1.217971804	9.63E-04	decrease
chr1	101025786	101026186	-1.158323372	9.64E-04	decrease
chr17	28041326	28041726	-1.168214601	9.64E-04	decrease
chr18	21600547	21600947	-1.071266456	9.77E-04	decrease
chr19	49766403	49766803	-0.811053419	9.77E-04	decrease
chr22	30423745	30424145	-1.033963633	9.81E-04	decrease
chr17	8210302	8210702	-0.662263496	9.85E-04	decrease
chr11	2089370	2089770	-1.289254672	9.93E-04	decrease
chr14	22956911	22957311	-1.111484996	9.96E-04	decrease
chr9	129036170	129036570	-1.172311046	9.96E-04	decrease
chr10	102713981	102714381	-0.956041749	9.98E-04	decrease
chr19	11381338	11381738	-0.904527111	1.01E-03	decrease
chr19	4968564	4968964	-1.020282403	1.01E-03	decrease
chr17	7931837	7932237	-0.885450257	1.02E-03	decrease
chr1	228407114	228407514	-1.070211462	1.02E-03	decrease
chr11	73598185	73598585	-1.089052986	1.03E-03	decrease
chr3	112562031	112562431	-0.923221584	1.03E-03	decrease
chr1	93846055	93846455	-0.937431413	1.03E-03	decrease
chr15	65304894	65305294	-0.77868273	1.03E-03	decrease
chrX	107206231	107206631	-1.314890797	1.03E-03	decrease
chr7	100429153	100429553	-0.935855423	1.03E-03	decrease
chr9	79663911	79664311	-1.118495591	1.04E-03	decrease
chr20	17682479	17682879	-1.37229914	1.04E-03	decrease
chr18	32158118	32158518	-1.281555142	1.04E-03	decrease
chr11	94973456	94973856	-1.369181228	1.04E-03	decrease
chr13	42271446	42271846	-1.256047494	1.04E-03	decrease
chr6	144095353	144095753	-1.097752825	1.05E-03	decrease
chr1	51878480	51878880	-1.195260625	1.05E-03	decrease
chr2	19901668	19902068	-1.271321054	1.05E-03	decrease
chr10	102169512	102169912	-1.134475338	1.06E-03	decrease
chr22	21014173	21014573	-1.060101202	1.06E-03	decrease
chr1	156054740	156055140	-1.039690943	1.06E-03	decrease
chr19	48170291	48170691	-1.167226276	1.06E-03	decrease
chr13	38349501	38349901	-1.084268921	1.06E-03	decrease
chr12	56116229	56116629	-0.975245263	1.07E-03	decrease
chr19	46600651	46601051	-1.227482797	1.08E-03	decrease
chrX	46573306	46573706	-1.318684792	1.08E-03	decrease
chr3	38024624	38025024	-0.912912957	1.09E-03	decrease
chr19	17209706	17210106	-1.184456336	1.09E-03	decrease
chr1	16896520	16896920	-1.169898856	1.09E-03	decrease

chr1	7961388	7961788	-1.244461393	1.09E-03	decrease
chr19	13952107	13952507	-1.167138584	1.10E-03	decrease
chr19	6828993	6829393	-1.050696749	1.11E-03	decrease
chr19	11374565	11374965	-0.852938434	1.11E-03	decrease
chr1	246566279	246566679	-1.062124865	1.12E-03	decrease
chr4	102869049	102869449	-1.147349586	1.12E-03	decrease
chr1	23168926	23169326	-1.348675995	1.12E-03	decrease
chr9	36190542	36190942	-1.260423876	1.12E-03	decrease
chr1	154974483	154974883	-0.846000558	1.12E-03	decrease
chr1	25247512	25247912	-1.29708643	1.13E-03	decrease
chr5	177496442	177496842	-0.959769467	1.14E-03	decrease
chr12	56725233	56725633	-1.141987126	1.15E-03	decrease
chr2	216412329	216412729	-1.014250045	1.15E-03	decrease
chr3	129440090	129440490	-1.041996068	1.15E-03	decrease
chr11	83286015	83286415	-1.124740243	1.15E-03	decrease
chr5	88674838	88675238	-1.311548202	1.15E-03	decrease
chr2	85595472	85595872	-1.102059728	1.15E-03	decrease
chr4	37686619	37687019	-1.325147343	1.15E-03	decrease
chr5	43121111	43121511	-1.157216066	1.15E-03	decrease
chr12	76084719	76085119	-1.188638477	1.16E-03	decrease
chr19	10835901	10836301	-0.776040084	1.17E-03	decrease
chr5	64768611	64769011	-1.293240454	1.17E-03	decrease
chr1	161117726	161118126	-1.051072096	1.17E-03	decrease
chr16	75433348	75433748	-1.141130375	1.18E-03	decrease
chr10	70146768	70147168	-1.019698326	1.18E-03	decrease
chr18	63422347	63422747	-1.265549615	1.18E-03	decrease
chr10	73744303	73744703	-1.088648993	1.18E-03	decrease
chr15	84980413	84980813	-1.180987529	1.19E-03	decrease
chr1	1919233	1919633	-1.214731851	1.19E-03	decrease
chr13	52011928	52012328	-1.286971941	1.20E-03	decrease
chr2	9630903	9631303	-1.215257887	1.20E-03	decrease
chr9	37079557	37079957	-1.147083232	1.21E-03	decrease
chr11	75814838	75815238	-1.061942524	1.21E-03	decrease
chr9	77177041	77177441	-0.965227995	1.21E-03	decrease
chr1	244833794	244834194	-1.04464052	1.22E-03	decrease
chr14	73785304	73785704	-1.305705437	1.22E-03	decrease
chr7	99472576	99472976	-1.064827611	1.23E-03	decrease
chr11	43358850	43359250	-1.208160725	1.23E-03	decrease
chr20	21125780	21126180	-1.185449518	1.25E-03	decrease
chr11	57712161	57712561	-1.056636178	1.25E-03	decrease
chr14	73886624	73887024	-1.046026186	1.25E-03	decrease
chr1	156193762	156194162	-0.977618173	1.25E-03	decrease
chr1	40979426	40979826	-1.033077671	1.25E-03	decrease
chr5	140700195	140700595	-1.002964101	1.25E-03	decrease
chr20	35664299	35664699	-1.298481251	1.26E-03	decrease
chr12	53006295	53006695	-1.094112254	1.26E-03	decrease
chr5	36875058	36875458	-1.368785876	1.26E-03	decrease
chr6	169702009	169702409	-1.091053758	1.26E-03	decrease
chr16	1960829	1961229	-0.906453349	1.26E-03	decrease
chr17	63961927	63962327	-1.236866473	1.27E-03	decrease
chr16	30634208	30634608	-0.917873633	1.27E-03	decrease
chr7	32495309	32495709	-1.376001541	1.28E-03	decrease
chr1	244970844	244971244	-0.954675729	1.28E-03	decrease
chr6	42879606	42880006	-1.040904845	1.29E-03	decrease
chr4	169612464	169612864	-1.19084554	1.29E-03	decrease
chr7	44848298	44848698	-1.241139594	1.29E-03	decrease
chr19	17226130	17226530	-1.049877491	1.29E-03	decrease
chr12	103956874	103957274	-1.113475979	1.31E-03	decrease
chr3	167734800	167735200	-0.921266641	1.31E-03	decrease
chr7	149662674	149663074	-0.915500403	1.31E-03	decrease
chr1	54764504	54764904	-0.900463872	1.32E-03	decrease
chr10	73997610	73998010	-1.141402643	1.32E-03	decrease
chrX	3814869	3815269	-1.291381694	1.32E-03	decrease
chr19	46850649	46851049	-0.994144564	1.34E-03	decrease
chr17	76264898	76265298	-1.1282983	1.34E-03	decrease
chr3	149657821	149658221	-1.304523116	1.35E-03	decrease
chr19	12766141	12766541	-1.125998073	1.35E-03	decrease
chr7	148963257	148963657	-0.582542867	1.36E-03	decrease
chr11	60906545	60906945	-1.241467582	1.37E-03	decrease
chr15	78540373	78540773	-1.263571205	1.37E-03	decrease
chr19	50025474	50025874	-0.833554227	1.38E-03	decrease
chr16	84014045	84014445	-0.859184289	1.39E-03	decrease
chr10	102432456	102432856	-0.996980342	1.39E-03	decrease
chr6	27297853	27298253	-0.555799658	1.40E-03	decrease
chr10	100997809	100998209	-1.15011074	1.40E-03	decrease
chr5	132556848	132557248	-1.096804437	1.40E-03	decrease
chr1	22025220	22025620	-1.019876212	1.41E-03	decrease

chr15	42548571	42548971	-1.29613601	1.42E-03	decrease
chr2	24076298	24076698	-1.050842333	1.42E-03	decrease
chr19	6476279	6476679	-0.844291479	1.43E-03	decrease
chr19	48492955	48493355	-0.822726765	1.43E-03	decrease
chr1	16352494	16352894	-1.359581149	1.43E-03	decrease
chr2	27663673	27664073	-1.143016194	1.43E-03	decrease
chr19	41859821	41860221	-0.954919549	1.44E-03	decrease
chr21	36060119	36060519	-1.150669296	1.44E-03	decrease
chr4	140756608	140757008	-1.175266677	1.44E-03	decrease
chr2	178413545	178413945	-1.20008726	1.45E-03	decrease
chr8	38176401	38176801	-1.172379765	1.45E-03	decrease
chr10	118046878	118047278	-1.357051143	1.45E-03	decrease
chr1	161132816	161133216	-1.088990618	1.45E-03	decrease
chr20	5113084	5113484	-1.03164675	1.45E-03	decrease
chr19	35557845	35558245	-1.211371186	1.46E-03	decrease
chr2	134918144	134918891	-0.976648897	1.48E-03	decrease
chr16	67806657	67807057	-1.026466936	1.48E-03	decrease
chr11	62545453	62545853	-1.102786672	1.49E-03	decrease
chr17	21126782	21127182	-1.163638457	1.49E-03	decrease
chr10	102152059	102152459	-0.848896045	1.50E-03	decrease
chr16	29790296	29790696	-1.092506939	1.50E-03	decrease
chr12	120201164	120201564	-0.925482169	1.51E-03	decrease
chr5	146446897	146447297	-1.14616894	1.51E-03	decrease
chrX	123648580	123648980	-1.038104991	1.51E-03	decrease
chr14	95534669	95535069	-1.086403947	1.52E-03	decrease
chr1	225999335	225999735	-0.817354906	1.52E-03	decrease
chr12	66302460	66302860	-1.2712579	1.53E-03	decrease
chr6	52284709	52285109	-1.02787317	1.53E-03	decrease
chr1	44799862	44800262	-0.93183648	1.53E-03	decrease
chr4	139453385	139453785	-1.027540955	1.53E-03	decrease
chr17	59155257	59155657	-0.93748893	1.54E-03	decrease
chr9	122264550	122264950	-1.328110754	1.54E-03	decrease
chr7	134316701	134317101	-1.167774779	1.54E-03	decrease
chr3	190424554	190424954	-1.345084092	1.54E-03	decrease
chr16	89701685	89702085	-1.307595313	1.55E-03	decrease
chr6	107958307	107958707	-1.31381618	1.55E-03	decrease
chr19	5693623	5694023	-1.03685856	1.56E-03	decrease
chr7	56033931	56034331	-1.174785219	1.56E-03	decrease
chr9	78235672	78236072	-1.280024581	1.57E-03	decrease
chr6	27893283	27893683	-0.798357879	1.57E-03	decrease
chr6	85678953	85679353	-1.235696675	1.58E-03	decrease
chrX	14873051	14873451	-1.012986501	1.58E-03	decrease
chr16	27203510	27203910	-1.039437711	1.59E-03	decrease
chr1	61081842	61082242	-1.270451606	1.59E-03	decrease
chr12	56104107	56104507	-0.947939646	1.59E-03	decrease
chr11	59668304	59668704	-1.207846589	1.59E-03	decrease
chr5	139341583	139341983	-0.961288474	1.60E-03	decrease
chr8	73293806	73294206	-0.907733077	1.61E-03	decrease
chr1_KI270713v1_random	21657	22057	-0.788699789	1.61E-03	decrease
chr7	151875291	151875691	-0.999339017	1.62E-03	decrease
chr5	178204198	178204598	-0.985959671	1.62E-03	decrease
chr4	77820144	77820544	-1.323931579	1.62E-03	decrease
chr1	155324449	155324849	-0.921076726	1.62E-03	decrease
chr4	15001714	15002114	-1.302865502	1.62E-03	decrease
chr1	156740780	156741180	-0.971115243	1.62E-03	decrease
chr19	11435159	11435559	-0.836922181	1.63E-03	decrease
chr15	72231623	72232023	-1.081705348	1.63E-03	decrease
chr17	21551103	21551503	-1.253416694	1.63E-03	decrease
chr1	148388466	148388866	-0.950199539	1.64E-03	decrease
chr12	48957052	48957452	-1.052379853	1.64E-03	decrease
chr1	229507995	229508395	-0.927080523	1.65E-03	decrease
chr17	8435512	8435912	-1.219908662	1.65E-03	decrease
chr3	33440440	33440840	-0.950993108	1.65E-03	decrease
chr13	27251324	27251724	-1.204466683	1.65E-03	decrease
chr17	1456458	1456858	-1.269312918	1.66E-03	decrease
chr17	28571415	28571815	-1.048649942	1.67E-03	decrease
chr12	120581102	120581502	-1.115215184	1.67E-03	decrease
chr14	20989867	20990267	-1.207986728	1.69E-03	decrease
chr19	19063575	19063975	-1.101713438	1.69E-03	decrease
chr2	206159428	206159828	-1.148668159	1.70E-03	decrease
chr4	87220599	87220999	-1.329453857	1.70E-03	decrease
chr1	28088401	28088801	-0.96102654	1.70E-03	decrease
chr18	34977438	34977838	-1.096832094	1.71E-03	decrease
chr3	100401130	100401530	-1.051816585	1.72E-03	decrease
chr15	66851489	66851889	-1.033841566	1.72E-03	decrease
chr4	56977644	56978044	-1.036312924	1.72E-03	decrease
chr7	128476522	128476922	-1.21087474	1.73E-03	decrease

chr3	120742236	120742636	-1.207062502	1.73E-03	decrease
chr1	223992535	223992935	-1.032758112	1.73E-03	decrease
chr5	93045079	93045479	-1.202798292	1.74E-03	decrease
chr1	80403300	80403700	-1.199735185	1.75E-03	decrease
chr11	71448844	71449244	-1.338470347	1.75E-03	decrease
chr15	89893907	89894307	-1.141753703	1.75E-03	decrease
chr3	33798547	33798947	-1.013562345	1.76E-03	decrease
chr15	26082162	26082562	-0.692355175	1.76E-03	decrease
chr22	42070558	42070958	-0.947323814	1.76E-03	decrease
chr1	212284855	212285255	-1.020415067	1.77E-03	decrease
chr1	45750379	45750779	-1.092322356	1.77E-03	decrease
chr2	24793155	24793555	-1.135782115	1.78E-03	decrease
chr16	2251657	2252057	-1.066202676	1.79E-03	decrease
chr15	84716126	84716526	-0.841699601	1.80E-03	decrease
chr1	63593247	63593647	-1.107145819	1.80E-03	decrease
chr7	67302481	67302881	-1.171319369	1.81E-03	decrease
chr1	23344405	23344805	-1.208492016	1.81E-03	decrease
chr7	33129251	33129651	-1.141612518	1.81E-03	decrease
chr19	58519637	58520037	-0.98697396	1.82E-03	decrease
chr11	117143782	117144182	-1.187516528	1.83E-03	decrease
chr3	129399342	129399742	-1.126932898	1.84E-03	decrease
chrX	154397907	154398307	-0.988794308	1.85E-03	decrease
chr2	74472450	74472850	-1.105163629	1.85E-03	decrease
chr5	65624191	65624591	-0.956681817	1.86E-03	decrease
chr1	40708901	40709301	-1.211508626	1.86E-03	decrease
chr19	39970812	39971212	-0.877873426	1.86E-03	decrease
chr11	18526608	18527008	-0.987901688	1.86E-03	decrease
chr6	20401329	20401729	-1.097038892	1.87E-03	decrease
chr1	147172164	147172564	-1.266156962	1.87E-03	decrease
chr8	11802175	11802575	-1.277304444	1.88E-03	decrease
chr10	70170532	70170932	-1.138442351	1.88E-03	decrease
chr14	75660842	75661242	-1.011059092	1.89E-03	decrease
chr13	27968891	27969291	-1.307932643	1.89E-03	decrease
chr10	101818466	101818866	-0.78559575	1.89E-03	decrease
chrX	103176215	103176615	-1.214396384	1.89E-03	decrease
chr1	145996572	145996972	-0.903470036	1.90E-03	decrease
chr12	122064080	122064480	-0.893871438	1.90E-03	decrease
chr17	7313229	7313629	-0.872897454	1.90E-03	decrease
chr21	42879157	42879557	-1.138956086	1.91E-03	decrease
chr3	46976508	46976908	-0.961224565	1.91E-03	decrease
chr6	27726536	27726936	-0.94295274	1.92E-03	decrease
chr3	122383850	122384250	-1.023578089	1.92E-03	decrease
chr8	47960412	47960812	-0.936530791	1.92E-03	decrease
chr2	214809710	214810110	-1.256239642	1.94E-03	decrease
chr5	177006494	177006894	-1.070318476	1.94E-03	decrease
chr17	39687758	39688158	-1.173212385	1.94E-03	decrease
chr19	40444434	40444834	-1.109709659	1.95E-03	decrease
chr1	207321246	207321646	-0.996624001	1.95E-03	decrease
chr11	62831844	62832244	-0.77199888	1.95E-03	decrease
chr1	248825992	248826392	-0.997065711	1.95E-03	decrease
chr19	3275474	3275874	-1.130578212	1.96E-03	decrease
chr19	40443016	40443416	-0.990174095	1.96E-03	decrease
chr10	119596754	119597154	-0.97306309	1.98E-03	decrease
chrX	74179485	74179885	-0.996278371	1.98E-03	decrease
chr19	17841884	17842284	-1.068801233	1.98E-03	decrease
chr18	12991110	12991510	-1.164227136	1.98E-03	decrease
chr3	42582026	42582426	-1.299431337	1.98E-03	decrease
chr4	101347732	101348132	-1.09863593	1.98E-03	decrease
chr19	40623790	40624190	-1.118590009	1.99E-03	decrease
chr2	222656048	222656448	-0.991909838	1.99E-03	decrease
chr1	212035330	212035730	-0.902776713	2.00E-03	decrease
chr2	26345740	26346140	-1.141719889	2.00E-03	decrease
chr5	149551496	149551896	-1.059000859	2.01E-03	decrease
chr17	75154465	75154865	-0.952422728	2.03E-03	decrease
chr13	72781878	72782278	-0.996084999	2.04E-03	decrease
chr7	16645819	16646219	-1.169726752	2.05E-03	decrease
chr1	43946509	43946909	-1.09186314	2.05E-03	decrease
chr19	1507870	1508270	-1.174799697	2.05E-03	decrease
chr1	179365623	179366023	-0.985933541	2.05E-03	decrease
chr5	141879013	141879413	-1.133268003	2.05E-03	decrease
chr17	28725241	28725641	-0.845116784	2.06E-03	decrease
chr16	89240930	89241330	-1.083888055	2.06E-03	decrease
chr1	26692979	26693379	-1.290686121	2.07E-03	decrease
chr15	48810849	48811249	-1.007790837	2.08E-03	decrease
chr1	20661339	20661739	-1.012875516	2.09E-03	decrease
chr17	75289129	75289529	-0.826961163	2.09E-03	decrease
chr18	22933109	22933509	-1.205530295	2.09E-03	decrease

chr12	121686902	121687302	-1.025766918	2.10E-03	decrease
chr6	5003680	5004080	-1.254979806	2.11E-03	decrease
chr16	89565060	89565460	-0.780289627	2.12E-03	decrease
chr16	75148385	75148785	-1.260196017	2.12E-03	decrease
chr14	21456126	21456526	-0.883932524	2.13E-03	decrease
chr11	72112400	72112800	-1.006502444	2.13E-03	decrease
chr7	151459891	151460291	-1.057234847	2.13E-03	decrease
chr17	5439346	5439746	-1.179643488	2.13E-03	decrease
chr6	35474396	35474796	-0.83990458	2.13E-03	decrease
chr16	31142124	31142524	-1.017015281	2.13E-03	decrease
chrX	149539478	149539878	-1.095332497	2.14E-03	decrease
chr7	151085919	151086319	-0.9795041	2.16E-03	decrease
chr1	1615363	1615763	-1.270161954	2.16E-03	decrease
chr7	77696150	77696550	-1.00123188	2.18E-03	decrease
chr21	37073751	37074151	-1.287236436	2.18E-03	decrease
chr2	202375764	202376164	-1.097919664	2.18E-03	decrease
chr18	31206815	31207215	-1.099960548	2.19E-03	decrease
chr4	112231727	112232127	-1.203536148	2.19E-03	decrease
chr19	10251777	10252177	-0.999575871	2.19E-03	decrease
chr9	125746186	125746586	-1.076048493	2.19E-03	decrease
chr15	64153386	64153786	-1.149376438	2.20E-03	decrease
chr14	55191561	55191961	-1.006324654	2.21E-03	decrease
chr5	180809936	180810336	-0.865151416	2.22E-03	decrease
chr8	38030881	38031281	-1.283378308	2.23E-03	decrease
chr7	151213643	151214043	-1.213753819	2.24E-03	decrease
chr2	42361210	42361610	-0.932365573	2.24E-03	decrease
chr19	57280117	57280517	-0.954207429	2.24E-03	decrease
chr8	17246381	17246781	-1.285467247	2.24E-03	decrease
chr6	43575727	43576127	-1.152781135	2.24E-03	decrease
chr14	88978733	88979133	-0.66804801	2.24E-03	decrease
chr11	111879086	111879486	-1.136225218	2.25E-03	decrease
chr13	45341323	45341723	-1.000083118	2.25E-03	decrease
chr6	135054731	135055131	-0.871529458	2.26E-03	decrease
chr6	159789988	159790388	-0.826125508	2.27E-03	decrease
chr1	32291711	32292111	-1.009032559	2.28E-03	decrease
chr1	27773051	27773451	-1.066717789	2.28E-03	decrease
chr6	108848159	108848559	-1.160905734	2.28E-03	decrease
chr5	173057064	173057464	-0.90865789	2.28E-03	decrease
chr3	9363010	9363410	-0.997667261	2.28E-03	decrease
chr11	130002683	130003083	-1.068831503	2.28E-03	decrease
chr17	75261711	75262111	-1.04366551	2.29E-03	decrease
chr1	200738789	200739189	-1.236244585	2.29E-03	decrease
chr12	45990318	45990718	-1.044661118	2.29E-03	decrease
chr2	192194837	192195237	-1.238407516	2.29E-03	decrease
chr16	73478195	73478595	-0.789407994	2.29E-03	decrease
chr11	93741485	93741885	-1.088380034	2.30E-03	decrease
chr14	39174767	39175167	-0.830125271	2.31E-03	decrease
chr11	119101781	119102181	-0.868311466	2.31E-03	decrease
chr11	62591684	62592084	-0.868352035	2.32E-03	decrease
chr10	14959312	14959712	-1.021802557	2.32E-03	decrease
chr12	31073915	31074315	-0.985014004	2.32E-03	decrease
chr12	82358467	82358867	-1.179500929	2.32E-03	decrease
chr1	109548502	109548902	-1.145600678	2.33E-03	decrease
chr11	46700512	46700912	-0.889397294	2.33E-03	decrease
chr3	14651430	14651830	-1.051455097	2.33E-03	decrease
chr3	88059058	88059458	-1.238299497	2.34E-03	decrease
chr19	17292533	17292933	-1.120600344	2.35E-03	decrease
chr4	113762012	113762412	-1.127234689	2.35E-03	decrease
chr16	30927947	30928347	-0.916926487	2.36E-03	decrease
chr5	138469431	138469831	0.986082456	2.37E-03	increase
chr17	75456393	75456793	-0.946824675	2.37E-03	decrease
chrX	54044419	54044819	-1.092890097	2.38E-03	decrease
chr1	228109098	228109498	-0.837694199	2.38E-03	decrease
chr17	58679470	58679870	-0.983477117	2.39E-03	decrease
chr6	27631163	27631563	-0.527524503	2.40E-03	decrease
chr11	65487727	65488127	-0.975330068	2.41E-03	decrease
chr12	26938077	26938477	-0.99726777	2.41E-03	decrease
chr8	51899175	51899575	-1.124663566	2.42E-03	decrease
chr19	7829639	7830039	-0.957487944	2.42E-03	decrease
chr11	62573879	62574279	-0.924306259	2.43E-03	decrease
chrX	134915017	134915417	-1.161593631	2.43E-03	decrease
chr2	20051711	20052111	-0.942876871	2.44E-03	decrease
chr12	57088262	57088662	-1.058391223	2.44E-03	decrease
chr18	23586535	23586935	-1.190790209	2.44E-03	decrease
chr9	38620393	38620793	-1.226597116	2.44E-03	decrease
chr17	64497171	64497571	-0.798182642	2.45E-03	decrease
chr1	109493999	109494399	-1.147189025	2.46E-03	decrease

chr22	31080844	31081244	-1.192920829	2.46E-03	decrease
chr10	28532709	28533109	-1.158212184	2.47E-03	decrease
chr9	19102685	19103085	-1.234436753	2.47E-03	decrease
chr8	95133372	95133772	-1.25639255	2.48E-03	decrease
chr20	49046192	49046592	-0.974586489	2.48E-03	decrease
chr13	21176588	21176988	-1.280879149	2.48E-03	decrease
chr2	240586291	240586691	-1.189026239	2.48E-03	decrease
chr6	11093599	11093999	-1.034143233	2.49E-03	decrease
chr6	167351176	167351576	-1.235525477	2.49E-03	decrease
chr1	153957897	153958297	-1.029149741	2.50E-03	decrease
chr1	77979053	77979453	-0.795987592	2.50E-03	decrease
chr19	8050433	8050833	-1.117826774	2.50E-03	decrease
chr17	68511993	68512393	-1.171059731	2.50E-03	decrease
chr14	22589319	22589719	-0.998807955	2.50E-03	decrease
chr13	77329116	77329516	-0.841518989	2.51E-03	decrease
chr6	26473859	26474259	-0.902885219	2.51E-03	decrease
chr3	49540008	49540408	-1.218924824	2.52E-03	decrease
chr14	21437248	21437648	-1.053253506	2.52E-03	decrease
chr9	87725963	87726363	-1.235589172	2.53E-03	decrease
chrX	77895266	77895666	-1.049229395	2.53E-03	decrease
chr19	6372435	6372835	-1.0605044	2.53E-03	decrease
chr11	18395882	18396282	-1.015297827	2.53E-03	decrease
chr2	74458127	74458527	-0.893251251	2.54E-03	decrease
chr10	102057992	102058392	-1.233634275	2.54E-03	decrease
chr2	121530711	121531111	-0.964526528	2.54E-03	decrease
chr15	70763512	70763912	-1.169096949	2.55E-03	decrease
chr4	73258841	73259241	-1.176952269	2.55E-03	decrease
chr1	27914565	27914965	-1.068279372	2.55E-03	decrease
chr22	40044265	40044665	-1.039968405	2.56E-03	decrease
chr11	207459	207859	-1.028941549	2.57E-03	decrease
chr14	64986810	64987210	-1.031279459	2.57E-03	decrease
chr7	100091144	100091544	-0.942303513	2.58E-03	decrease
chr19	7506388	7506788	-1.095823078	2.58E-03	decrease
chr2	231452580	231452980	-0.785825608	2.58E-03	decrease
chr10	25016439	25016839	-1.1598183	2.59E-03	decrease
chr19	50608170	50608570	-1.25129015	2.60E-03	decrease
chr11	10792369	10792769	-1.004491762	2.61E-03	decrease
chr22	37844212	37844612	-1.129236113	2.61E-03	decrease
chr21	29024564	29024964	-0.891120525	2.64E-03	decrease
chr6	145964208	145964608	-1.049967796	2.65E-03	decrease
chr17	28227441	28227841	-1.107083917	2.66E-03	decrease
chr14	45134285	45134685	-0.976348172	2.66E-03	decrease
chr6	84227612	84228012	-1.189307575	2.67E-03	decrease
chr6	87155271	87155671	-1.048974146	2.67E-03	decrease
chr19	48993406	48993806	-0.90807964	2.68E-03	decrease
chr22	39502130	39502530	-1.048522295	2.68E-03	decrease
chr4	70839813	70840213	-1.223209523	2.69E-03	decrease
chr20	36773781	36774181	-1.018201532	2.71E-03	decrease
chr6	73520891	73521291	-1.130722761	2.72E-03	decrease
chr8	38105439	38105839	-1.18227401	2.72E-03	decrease
chr6	42929394	42929794	-0.959539644	2.72E-03	decrease
chr15	90265320	90265720	-1.023892723	2.73E-03	decrease
chr4	106316156	106316556	-0.966371918	2.74E-03	decrease
chr22	19432415	19432815	-1.117534167	2.76E-03	decrease
chr12	56590337	56590737	-1.177607894	2.76E-03	decrease
chr9	38392619	38393019	-1.138813845	2.77E-03	decrease
chr6	26021012	26021412	-0.977517582	2.77E-03	decrease
chr8	144901446	144901846	-0.96892127	2.77E-03	decrease
chr2	27212135	27212535	-1.070481143	2.78E-03	decrease
chr3	184017912	184018312	-1.183841917	2.79E-03	decrease
chr10	26860847	26861247	-1.133983543	2.79E-03	decrease
chr17	7204915	7205315	-1.119392297	2.80E-03	decrease
chr19	53394995	53395395	-1.277844065	2.81E-03	decrease
chr6	125789681	125790081	-1.110793304	2.81E-03	decrease
chr12	120317406	120317806	-1.006111925	2.82E-03	decrease
chr11	126062983	126063383	-1.067091601	2.84E-03	decrease
chr2	66071008	66071408	-1.160167218	2.85E-03	decrease
chr17	82367821	82368221	-0.907938367	2.85E-03	decrease
chr2	127810948	127811348	-0.98685813	2.86E-03	decrease
chr10	110210811	110211211	-1.17922463	2.86E-03	decrease
chr6	26224023	26224423	-0.895598703	2.86E-03	decrease
chr1	155745448	155745848	-1.11756916	2.88E-03	decrease
chr6	36027459	36027859	-1.102758842	2.88E-03	decrease
chr6	89638483	89638883	-0.94262664	2.89E-03	decrease
chr8	22245006	22245406	-1.226241869	2.89E-03	decrease
chr19	58400946	58401346	-1.057464457	2.90E-03	decrease
chrX	108091554	108091954	-0.93848246	2.91E-03	decrease

chr17	63842935	63843335	-0.923172028	2.91E-03	decrease
chr19	17215259	17215659	-1.081395502	2.92E-03	decrease
chr12	6872001	6872401	-0.891695979	2.92E-03	decrease
chr14	104794154	104794554	-1.018656982	2.92E-03	decrease
chr15	72272686	72273086	-1.10413068	2.93E-03	decrease
chr20	2102249	2102649	-0.985628399	2.93E-03	decrease
chr3	45593412	45593812	-1.162269683	2.95E-03	decrease
chrX	48897751	48898151	-0.905715405	2.96E-03	decrease
chr4	90838742	90839142	-1.265724712	2.96E-03	decrease
chr10	75613950	75614350	-1.247654627	2.99E-03	decrease
chr1	217630999	217631399	-1.154232756	2.99E-03	decrease
chr2	74440522	74440922	-0.956862703	2.99E-03	decrease
chr1	156728706	156729106	-1.046610536	3.00E-03	decrease
chr9	35072611	35073011	-1.099635126	3.01E-03	decrease
chr22	17628710	17629110	-0.927558801	3.01E-03	decrease
chr15	70100528	70100928	-1.078548626	3.01E-03	decrease
chr11	90240162	90240562	-1.16642967	3.02E-03	decrease
chr2	32627822	32628222	-1.1633303	3.02E-03	decrease
chr4	40057359	40057759	-1.205309911	3.03E-03	decrease
chr13	77918823	77919223	-1.151604462	3.03E-03	decrease
chrX	108438173	108438573	-1.17254859	3.05E-03	decrease
chr19	43463528	43463928	-0.815376702	3.05E-03	decrease
chr19	4007175	4007575	-1.165435518	3.05E-03	decrease
chr9	100099004	100099404	-0.808233036	3.05E-03	decrease
chr4	127782281	127782681	-1.158826168	3.05E-03	decrease
chr2	43995948	43996348	-0.876778657	3.06E-03	decrease
chr3	53347363	53347763	-1.047010931	3.06E-03	decrease
chr1	100132702	100133102	-0.96834914	3.08E-03	decrease
chr8	66667114	66667514	-0.821390036	3.10E-03	decrease
chr19	17606259	17606659	-1.214434794	3.10E-03	decrease
chr4	781992	782392	-0.872683701	3.11E-03	decrease
chr15	84600467	84600867	-0.993653757	3.12E-03	decrease
chr12	103930043	103930443	-1.000871552	3.13E-03	decrease
chr7	156951432	156951832	-1.002675474	3.13E-03	decrease
chr4	84966761	84967161	-1.239196216	3.14E-03	decrease
chr19	10552660	10553060	-1.023884014	3.15E-03	decrease
chr14	50828979	50829379	-1.180817523	3.15E-03	decrease
chr7	149125928	149126328	-0.906025682	3.16E-03	decrease
chr6	57813494	57813894	-0.898344343	3.16E-03	decrease
chr17	51153430	51153830	-1.160488449	3.16E-03	decrease
chr3	20040980	20041380	-1.260181096	3.18E-03	decrease
chr2	178451178	178451578	-1.041609935	3.18E-03	decrease
chr2	27582845	27583245	-1.072040993	3.19E-03	decrease
chr19	48713904	48714304	-1.015788853	3.20E-03	decrease
chr3	50235806	50236206	-1.014963596	3.21E-03	decrease
chr5	80488125	80488525	-1.218400814	3.22E-03	decrease
chr19	2151634	2152034	-1.065124751	3.22E-03	decrease
chr1	22451929	22452329	-1.256204927	3.23E-03	decrease
chr1	156749567	156749967	-1.052387272	3.23E-03	decrease
chr16	5071805	5072205	-1.234514942	3.24E-03	decrease
chr1	214551222	214551622	-1.209004632	3.25E-03	decrease
chr11	9460608	9461008	-0.838693427	3.25E-03	decrease
chr19	3573823	3574223	-1.006376837	3.26E-03	decrease
chr3	119498483	119498883	-1.147309443	3.28E-03	decrease
chr12	114684126	114684526	-1.043562873	3.28E-03	decrease
chr10	104122034	104122434	-1.228315973	3.29E-03	decrease
chr19	50050789	50051189	-0.865504674	3.30E-03	decrease
chr11	64810524	64810924	-1.046799428	3.30E-03	decrease
chr11	72812926	72813326	-1.062806069	3.30E-03	decrease
chr11	64317670	64318070	-1.099805111	3.31E-03	decrease
chr12	112125349	112125749	-1.074970165	3.31E-03	decrease
chr1	16534919	16535319	-1.116068683	3.31E-03	decrease
chr19	19320452	19320852	-0.92125942	3.32E-03	decrease
chr20	34516025	34516425	-1.023378656	3.32E-03	decrease
chr11	8911196	8911596	-1.110231313	3.32E-03	decrease
chr19	35628631	35629031	-0.983010405	3.33E-03	decrease
chr13	27125692	27126092	-1.025811638	3.33E-03	decrease
chr1	121020713	121021113	-0.720554484	3.34E-03	decrease
chr15	41774065	41774465	-0.984365045	3.34E-03	decrease
chr14	49688242	49688642	-1.118528194	3.34E-03	decrease
chr21	32612252	32612652	-0.890192511	3.35E-03	decrease
chr9	4792368	4792768	-1.029386308	3.36E-03	decrease
chr6	97282905	97283305	-1.180181849	3.37E-03	decrease
chr19	57633070	57633470	-1.253907911	3.37E-03	decrease
chr15	22838211	22838611	-1.021923388	3.39E-03	decrease
chr17	47649462	47649862	-0.991720496	3.41E-03	decrease
chr11	95231229	95231629	-1.051350781	3.43E-03	decrease

chr2	112541976	112542376	-0.981505994	3.43E-03	decrease
chr16	30762121	30762521	-0.836856239	3.43E-03	decrease
chr17	32444226	32444626	-1.033050695	3.43E-03	decrease
chr17	28726628	28727028	-0.825672836	3.44E-03	decrease
chr15	55408017	55408417	-1.184519446	3.44E-03	decrease
chr15	39782520	39782920	-1.247160792	3.44E-03	decrease
chr5	72320255	72320655	-1.040421168	3.45E-03	decrease
chr3	158110272	158110672	-1.014373098	3.45E-03	decrease
chr1	235128697	235129097	-0.781276609	3.46E-03	decrease
chr1	174159585	174159985	-1.109421948	3.47E-03	decrease
chr8	143952466	143952866	-1.144993343	3.48E-03	decrease
chr11	18322188	18322588	-1.118526588	3.49E-03	decrease
chr1	146376973	146377373	-0.791297877	3.50E-03	decrease
chr1	112700600	112701000	-0.937543653	3.51E-03	decrease
chr1	148334600	148335000	-0.827099564	3.51E-03	decrease
chr11	65121995	65122395	-0.887709327	3.52E-03	decrease
chr15	78299232	78299632	-1.087895538	3.52E-03	decrease
chr19	45902791	45903191	-0.969648858	3.52E-03	decrease
chr1	209827789	209828189	-1.020572948	3.53E-03	decrease
chr1	45339909	45340309	-1.006402175	3.53E-03	decrease
chr3	51941861	51942261	-1.034383166	3.53E-03	decrease
chr1	28736382	28736782	-1.073205928	3.53E-03	decrease
chr9	71911231	71911631	-0.982975009	3.53E-03	decrease
chr16	1309043	1309443	-1.118326309	3.53E-03	decrease
chr16	88770784	88771184	-0.953808727	3.54E-03	decrease
chr9	128340143	128340543	-0.721088237	3.54E-03	decrease
chr5	171418775	171419175	-1.034867177	3.56E-03	decrease
chr3	66220617	66221017	-1.042209094	3.56E-03	decrease
chr15	74938473	74938873	-1.117862372	3.57E-03	decrease
chr5	131635084	131635484	-0.960176588	3.57E-03	decrease
chr16	30775095	30775495	-0.839049604	3.59E-03	decrease
chr19	46638569	46638969	-1.005574533	3.60E-03	decrease
chr9	6681333	6681733	-0.904275145	3.61E-03	decrease
chr1	153922993	153923393	-0.928710812	3.61E-03	decrease
chr11	74170894	74171294	-0.855318997	3.62E-03	decrease
chr16	57185976	57186376	-0.877540426	3.62E-03	decrease
chr12	6944009	6944409	-0.746224748	3.64E-03	decrease
chr6	107459424	107459824	-1.096160645	3.64E-03	decrease
chr11	34105353	34105753	-0.843992272	3.65E-03	decrease
chr6	166627340	166627740	-1.05636213	3.65E-03	decrease
chr19	45178321	45178721	-0.867471913	3.68E-03	decrease
chr16	1708540	1708940	-1.042742406	3.68E-03	decrease
chr13	30465821	30466221	-1.075416193	3.68E-03	decrease
chr1	218284911	218285311	-1.231237751	3.68E-03	decrease
chr2	202032805	202033205	-1.154036652	3.69E-03	decrease
chr9	35815193	35815593	-0.924008949	3.69E-03	decrease
chr9	108933749	108934149	-1.191094286	3.71E-03	decrease
chr19	57302541	57302941	-1.202787849	3.71E-03	decrease
chr14	21071547	21071947	-1.157011083	3.72E-03	decrease
chr3	113746727	113747127	-1.022755536	3.73E-03	decrease
chr6	43629531	43629931	-0.871569921	3.73E-03	decrease
chr1	184754881	184755281	-0.837287869	3.74E-03	decrease
chr20	35742247	35742647	-0.764643167	3.74E-03	decrease
chr5	52787799	52788199	-1.239775197	3.74E-03	decrease
chr6	36547159	36547559	-1.120882028	3.74E-03	decrease
chr22	41832644	41833044	-0.941830284	3.75E-03	decrease
chr19	46347001	46347401	-1.112114607	3.75E-03	decrease
chr14	103518507	103518907	-0.840054003	3.75E-03	decrease
chr10	114174214	114174614	-1.123186685	3.76E-03	decrease
chr1	75786015	75786415	-0.874038917	3.77E-03	decrease
chr9	133346875	133347275	-1.001172293	3.77E-03	decrease
chr11	103109130	103109530	-1.095937533	3.79E-03	decrease
chr1	113072744	113073144	-0.812833165	3.79E-03	decrease
chr3	41962040	41962440	-0.914753988	3.79E-03	decrease
chr5	168579606	168580006	-1.129936151	3.79E-03	decrease
chr2	189784251	189784651	-1.048424022	3.81E-03	decrease
chr1	42958929	42959329	-1.025523439	3.82E-03	decrease
chr11	5225403	5225803	-1.233459173	3.82E-03	decrease
chr10	102854328	102854728	-0.998355333	3.83E-03	decrease
chr1	70205457	70205857	-0.978303762	3.83E-03	decrease
chr8	55772808	55773208	-1.10689099	3.83E-03	decrease
chr3	141875575	141875975	-1.108309764	3.83E-03	decrease
chr1	150515633	150516033	-0.867660243	3.84E-03	decrease
chr19	57320328	57320728	-1.025242994	3.84E-03	decrease
chr10	73252419	73252819	-1.002569223	3.88E-03	decrease
chr1	151189802	151190202	-0.917328441	3.88E-03	decrease
chr1	11645772	11646172	-1.213461422	3.90E-03	decrease

chr20	43457490	43457890	-1.10824762	3.90E-03	decrease
chr14	21511076	21511476	-0.961211581	3.90E-03	decrease
chr12	110501598	110501998	-1.122690919	3.91E-03	decrease
chr15	95557179	95557579	-1.203772852	3.94E-03	decrease
chr7	140175192	140175592	-1.089388053	3.96E-03	decrease
chr16	3443711	3444111	-1.15089969	3.96E-03	decrease
chr6	99425228	99425628	-0.983576713	3.98E-03	decrease
chr4	173334421	173334821	-0.818950414	3.99E-03	decrease
chr6	26204276	26204676	-0.747301633	3.99E-03	decrease
chr9	128128084	128128484	-1.051614838	4.00E-03	decrease
chr12	124862941	124863341	-1.083265777	4.00E-03	decrease
chr14	61695413	61695813	-1.186041136	4.01E-03	decrease
chr19	2427588	2427988	-0.95225515	4.01E-03	decrease
chr13	19633331	19633731	-1.07403115	4.02E-03	decrease
chr17	63621932	63622332	-0.81839512	4.03E-03	decrease
chr16	69339630	69340030	-1.082782941	4.03E-03	decrease
chr16	3264293	3264693	-0.956450797	4.03E-03	decrease
chr19	47257054	47257454	-0.843192277	4.05E-03	decrease
chr2	208265808	208266208	-1.042766389	4.08E-03	decrease
chr22	36776232	36776632	-1.010875206	4.09E-03	decrease
chr12	56360104	56360504	-0.949603401	4.09E-03	decrease
chr2	176104972	176105372	-1.05652086	4.09E-03	decrease
chr1	31065413	31065813	-1.065311288	4.10E-03	decrease
chrX	132023470	132023870	-1.141053548	4.10E-03	decrease
chr22	44685084	44685484	-1.050127018	4.11E-03	decrease
chr9	127786386	127786786	-1.116101698	4.11E-03	decrease
chr6	93419690	93420090	-1.215391751	4.11E-03	decrease
chr1	161314259	161314659	-0.92465983	4.12E-03	decrease
chr1	10398558	10398958	-1.02106324	4.12E-03	decrease
chr3	100492110	100492510	-1.095967701	4.14E-03	decrease
chr1	114780468	114780868	-1.014011938	4.14E-03	decrease
chr14	90383199	90383599	-1.001585755	4.14E-03	decrease
chr12	95473832	95474232	-0.900886918	4.14E-03	decrease
chr2	202870810	202871210	-1.2250252	4.15E-03	decrease
chr1	235328432	235328832	-0.745046767	4.15E-03	decrease
chr10	89643913	89644313	-1.042059128	4.16E-03	decrease
chr1	21176776	21177176	-1.030093553	4.17E-03	decrease
chr1	11979722	11980122	-1.15510373	4.17E-03	decrease
chr5	32585539	32585939	-1.193571217	4.17E-03	decrease
chr1	156501169	156501569	-1.053181828	4.17E-03	decrease
chr14	70809718	70810118	-1.026602658	4.19E-03	decrease
chr12	113221039	113221439	-0.98581047	4.20E-03	decrease
chr11	83193559	83193959	-1.07810345	4.21E-03	decrease
chr15	90954736	90955136	-0.871112897	4.21E-03	decrease
chr13	52652479	52652879	-0.925950399	4.21E-03	decrease
chr11	119121217	119121617	-0.896391914	4.22E-03	decrease
chr17	3913802	3914202	-1.062098184	4.22E-03	decrease
chr3	58433512	58433912	-1.13176256	4.23E-03	decrease
chr11	47426561	47426961	-0.907455045	4.23E-03	decrease
chr16	23510502	23510902	-1.211873975	4.23E-03	decrease
chr19	19663690	19664090	-1.161670712	4.24E-03	decrease
chr19	41958552	41958952	-0.951235292	4.24E-03	decrease
chr16	53503299	53503699	-1.064366162	4.25E-03	decrease
chr9	128067643	128068043	-0.82427819	4.25E-03	decrease
chr12	49741323	49741723	-0.778373653	4.25E-03	decrease
chr7	151969455	151969855	-1.079100004	4.25E-03	decrease
chr3	159764145	159764545	-1.138438718	4.27E-03	decrease
chr15	65533193	65533593	-1.136665419	4.27E-03	decrease
chr10	12195665	12196065	-0.967990108	4.28E-03	decrease
chr10	14953919	14954319	-1.007161262	4.28E-03	decrease
chr11	118401250	118401650	-1.058746768	4.29E-03	decrease
chr14	24146868	24147268	-0.950001597	4.31E-03	decrease
chr13	64723702	64724102	-1.077520946	4.31E-03	decrease
chr11	59669158	59669558	-0.913450372	4.32E-03	decrease
chr1	213015418	213015818	-0.854987294	4.35E-03	decrease
chrX	49043484	49043884	-1.036492944	4.35E-03	decrease
chr20	14337363	14337763	-1.091407996	4.36E-03	decrease
chr11	44565136	44565536	-1.071207308	4.36E-03	decrease
chr19	11243414	11243814	-1.130585868	4.38E-03	decrease
chr9	4662949	4663349	-1.181085998	4.40E-03	decrease
chr7	76359114	76359514	-0.999749034	4.41E-03	decrease
chr7	77798395	77798795	-0.885751322	4.42E-03	decrease
chr10	97445711	97446111	-0.981387694	4.42E-03	decrease
chr11	64924138	64924538	-0.947392302	4.43E-03	decrease
chr3	9933680	9934080	-1.010957799	4.44E-03	decrease
chr20	45910863	45911263	-1.039115998	4.45E-03	decrease
chr3	141231329	141231729	-1.206200459	4.46E-03	decrease

chr19	15125265	15125665	-1.027611277	4.46E-03	decrease
chr17	7214730	7215130	-0.738837677	4.48E-03	decrease
chr1	236063832	236064232	-0.992222669	4.49E-03	decrease
chr7	6104359	6104759	-1.066384199	4.50E-03	decrease
chr12	6764153	6764553	-0.972666587	4.51E-03	decrease
chr6	27288432	27288832	-0.643698665	4.54E-03	decrease
chr15	68229753	68230153	-1.171757933	4.54E-03	decrease
chr5	127517587	127517987	-1.182129327	4.54E-03	decrease
chr2	218710885	218711285	-1.136019037	4.57E-03	decrease
chr3	10115399	10115799	-0.945145943	4.59E-03	decrease
chr16	9091161	9091561	-1.067426073	4.59E-03	decrease
chr19	39031531	39031931	-1.064755866	4.60E-03	decrease
chr5	119070672	119071072	-0.925943712	4.61E-03	decrease
chr16	29816026	29816426	-0.718848847	4.62E-03	decrease
chr5	90474422	90474822	-0.95461071	4.63E-03	decrease
chr5	131797143	131797543	-1.090160626	4.63E-03	decrease
chr21	28992632	28993032	-0.783457274	4.64E-03	decrease
chr2	55050428	55050828	-0.887299014	4.65E-03	decrease
chr19	55156443	55156843	-1.087435391	4.65E-03	decrease
chr19	11597254	11597654	-0.96885659	4.65E-03	decrease
chr16	2459714	2460114	-1.05223211	4.65E-03	decrease
chr19	35748242	35748642	-0.835393008	4.66E-03	decrease
chr19	18919352	18919752	-0.969709411	4.66E-03	decrease
chr2	237966443	237966843	-1.151868977	4.66E-03	decrease
chr17	78187011	78187411	-0.8728174	4.67E-03	decrease
chr3	78994518	78994918	-1.081847964	4.68E-03	decrease
chr14	58244425	58244825	-0.863525444	4.68E-03	decrease
chr1	228165207	228165607	-0.922597153	4.68E-03	decrease
chr16	58734031	58734431	-1.170367497	4.69E-03	decrease
chr14	73058200	73058600	-1.02040589	4.69E-03	decrease
chr17	59892884	59893284	-0.915726206	4.70E-03	decrease
chr7	135662089	135662489	-0.962420914	4.72E-03	decrease
chr12	56041488	56041888	-0.958151853	4.73E-03	decrease
chr6	28080580	28080980	-0.784939118	4.73E-03	decrease
chr1	169106207	169106607	-1.143349589	4.73E-03	decrease
chrX	120629737	120630137	-1.112128912	4.74E-03	decrease
chr11	3797617	3798017	-0.899404097	4.74E-03	decrease
chr12	120302249	120302649	-0.986518993	4.75E-03	decrease
chr1	161021141	161021541	-0.942237817	4.76E-03	decrease
chr14	45253211	45253611	-1.088870095	4.76E-03	decrease
chr1	113812217	113812617	-1.024425677	4.77E-03	decrease
chr19	10569119	10569519	-1.032346207	4.77E-03	decrease
chr5	33440796	33441196	-0.874632737	4.78E-03	decrease
chr13	45418174	45418574	-1.026675335	4.79E-03	decrease
chr10	97334709	97335109	-1.16593737	4.79E-03	decrease
chr3	50616207	50616607	-1.10889692	4.82E-03	decrease
chr15	90087212	90087612	-1.114624789	4.83E-03	decrease
chr7	108569564	108569964	-0.97110622	4.84E-03	decrease
chr12	55817688	55818088	-0.866710039	4.86E-03	decrease
chr14	21308619	21309019	-0.884039811	4.89E-03	decrease
chr5	115626093	115626493	-1.077553396	4.91E-03	decrease
chr19	53980393	53980793	-0.863869686	4.92E-03	decrease
chr7	12686911	12687311	-1.200657244	4.93E-03	decrease
chr11	66438757	66439157	-0.820123474	4.93E-03	decrease
chr2	39121113	39121513	-1.010054413	4.95E-03	decrease
chr2	210002520	210002920	-1.075962121	4.97E-03	decrease
chr1	229625068	229625468	-0.996098727	4.97E-03	decrease
chr19	48502274	48502674	-1.060828099	4.98E-03	decrease
chr6	28267041	28267441	-1.086461135	4.99E-03	decrease
chr13	97433214	97433614	-0.882811231	5.00E-03	decrease
chr9	89310759	89311159	-1.061415311	5.01E-03	decrease
chr19	6393229	6393629	-0.845444878	5.01E-03	decrease
chr18	31943010	31943410	-0.923238737	5.02E-03	decrease
chr1	151909450	151909850	-1.009913388	5.02E-03	decrease
chr16	28863432	28863832	-0.921467703	5.02E-03	decrease
chr8	144522116	144522516	-1.003895406	5.04E-03	decrease
chrX	47233376	47233776	-1.173733831	5.05E-03	decrease
chr2	61536542	61536942	-0.980755297	5.05E-03	decrease
chr19	17337124	17337524	-0.67017026	5.05E-03	decrease
chr10	110567294	110567694	-0.773411992	5.06E-03	decrease
chr7	138001897	138002297	-0.790617035	5.07E-03	decrease
chr1	145979225	145979625	-0.8294911	5.07E-03	decrease
chr6	41072553	41072953	-0.868385172	5.09E-03	decrease
chr11	66677920	66678320	-1.023284962	5.09E-03	decrease
chr12	69359627	69360027	-0.909084133	5.10E-03	decrease
chr11	65382151	65382551	-0.901284038	5.12E-03	decrease
chr1	92831917	92832317	-0.892788594	5.12E-03	decrease

chr22	40534126	40534526	-1.154108089	5.13E-03	decrease
chr19	1044875	1045275	-1.017786114	5.13E-03	decrease
chr2	202911721	202912121	-1.139838263	5.14E-03	decrease
chr7	65252134	65252534	-1.159494743	5.14E-03	decrease
chr11	6234850	6235250	-0.930163467	5.15E-03	decrease
chr9	99221926	99222326	-0.891074439	5.16E-03	decrease
chr22	38308877	38309277	-0.985434843	5.16E-03	decrease
chr3	120094584	120094984	-0.709564326	5.17E-03	decrease
chr6	89829710	89830110	-0.837919872	5.17E-03	decrease
chr18	26090647	26091047	-0.791597286	5.18E-03	decrease
chr12	71685992	71686392	-1.127720199	5.18E-03	decrease
chr19	14419057	14419457	-0.98478948	5.18E-03	decrease
chr14	52730100	52730500	-0.867350399	5.21E-03	decrease
chr19	47348993	47349393	-0.996271405	5.21E-03	decrease
chr6	140897964	140898364	-1.194153284	5.23E-03	decrease
chr2	196926077	196926477	-1.138055685	5.24E-03	decrease
chr19	55624582	55624982	-1.063703089	5.24E-03	decrease
chr9	33473696	33474096	-0.933053553	5.25E-03	decrease
chr7	128862743	128863143	-0.882254545	5.27E-03	decrease
chr18	31931855	31932255	-1.0632122	5.27E-03	decrease
chr19	36214668	36215068	-0.799210598	5.30E-03	decrease
chr9	128554592	128554992	-1.059438365	5.32E-03	decrease
chr18	36829181	36829581	-0.926329301	5.34E-03	decrease
chr8	94822653	94823053	-1.040528924	5.36E-03	decrease
chr12	7018492	7018892	-0.967776601	5.36E-03	decrease
chr17	17237097	17237497	-0.770815162	5.37E-03	decrease
chrX	118345701	118346101	-0.824492782	5.37E-03	decrease
chr6	149717704	149718104	-1.071884561	5.38E-03	decrease
chr17	42998098	42998498	-0.897179395	5.38E-03	decrease
chrX	135004077	135004477	-0.969799591	5.40E-03	decrease
chr19	52189815	52190215	-0.768509355	5.40E-03	decrease
chr19	43504281	43504681	-1.091027754	5.40E-03	decrease
chr2	99180937	99181337	-1.11420062	5.41E-03	decrease
chr19	3285058	3285458	-0.815723859	5.41E-03	decrease
chr9	83956365	83956765	-1.059784548	5.41E-03	decrease
chr1	170531953	170532353	-0.936927184	5.41E-03	decrease
chr12	48852163	48852563	-1.051682272	5.42E-03	decrease
chr7	39949405	39949805	-1.031232359	5.43E-03	decrease
chr6	26594836	26595236	-0.524443939	5.46E-03	decrease
chr4	165327388	165327788	-1.080243922	5.47E-03	decrease
chr6	53064844	53065244	-1.014208461	5.47E-03	decrease
chr10	73096724	73097124	-1.000383113	5.47E-03	decrease
chr10	104121353	104121753	-1.086673589	5.48E-03	decrease
chr8	102655148	102655548	-1.157069763	5.49E-03	decrease
chr7	33062827	33063227	-0.819614298	5.51E-03	decrease
chr14	49879928	49880328	-0.997320588	5.51E-03	decrease
chr5	77491960	77492360	-1.157536002	5.52E-03	decrease
chr20	41028423	41028823	-0.955122664	5.52E-03	decrease
chr11	64803331	64803731	-1.010157754	5.52E-03	decrease
chr7	143901864	143902264	-1.005546933	5.53E-03	decrease
chr7	128879853	128880253	-1.074125615	5.53E-03	decrease
chr7	28854308	28854708	-1.173761945	5.59E-03	decrease
chr2	70257694	70258094	-0.835984027	5.61E-03	decrease
chr16	2970487	2970887	-0.842791844	5.62E-03	decrease
chr9	113410355	113410755	-0.967663582	5.63E-03	decrease
chr11	59536910	59537310	-1.039433381	5.65E-03	decrease
chr1	85581429	85581829	-1.050234094	5.65E-03	decrease
chr17	63834742	63835142	-0.953950126	5.66E-03	decrease
chr9	98255704	98256104	-0.996962822	5.66E-03	decrease
chr11	67317420	67317820	-0.985065477	5.67E-03	decrease
chr7	129948959	129949359	-0.958649236	5.70E-03	decrease
chr4	147683958	147684358	-1.096672726	5.71E-03	decrease
chr11	66958345	66958745	-0.98561288	5.72E-03	decrease
chrX	135520447	135520847	-1.036139696	5.73E-03	decrease
chr7	6448007	6448407	-0.905523262	5.74E-03	decrease
chrX	13653145	13653545	-1.026295177	5.76E-03	decrease
chr10	74176348	74176748	-1.098775314	5.77E-03	decrease
chr1	226083299	226083699	-0.90411908	5.79E-03	decrease
chr5	151758564	151758964	-1.128918095	5.79E-03	decrease
chr5	154937661	154938061	-1.126628334	5.82E-03	decrease
chr6	85643838	85644238	-1.096803997	5.82E-03	decrease
chr1	28247020	28247420	-0.885297	5.85E-03	decrease
chr7	140517825	140518225	-1.114599217	5.85E-03	decrease
chr1	145964318	145964718	-0.827451125	5.85E-03	decrease
chrX	119943358	119943758	-0.922266144	5.86E-03	decrease
chr11	71786804	71787204	-1.002402208	5.87E-03	decrease
chr5	10441674	10442074	-0.95081701	5.87E-03	decrease

chr9	128829735	128830135	-1.000814302	5.88E-03	decrease
chrX	51893298	51893698	-1.067923247	5.88E-03	decrease
chr2	37196222	37196622	-1.131990285	5.89E-03	decrease
chr8	65633987	65634387	-0.969547031	5.89E-03	decrease
chr6	36885679	36886079	-1.061675857	5.89E-03	decrease
chr3	132417478	132417878	-1.018156289	5.89E-03	decrease
chr7	6008973	6009373	-0.937671864	5.91E-03	decrease
chr14	77320947	77321347	-0.784253771	5.91E-03	decrease
chr4	169660177	169660577	-1.048400795	5.91E-03	decrease
chr10	11773966	11774366	-1.124879768	5.92E-03	decrease
chr2	99141209	99141609	-1.069523613	5.92E-03	decrease
chr1	50968107	50968507	-1.051792226	5.92E-03	decrease
chr17	7404022	7404422	-0.878202144	5.95E-03	decrease
chr18	11980927	11981327	-1.045039031	5.96E-03	decrease
chr9	123687203	123687603	-1.161562551	5.97E-03	decrease
chr17	409954	410354	-0.999748185	5.98E-03	decrease
chr11	63974219	63974619	-0.891562155	5.98E-03	decrease
chr1	146387130	146387530	-0.721978028	5.98E-03	decrease
chr5	139560916	139561316	-1.154249584	5.99E-03	decrease
chr2	231464677	231465077	-0.923894309	5.99E-03	decrease
chr5	134758470	134758870	-0.945376304	5.99E-03	decrease
chrX	123859961	123860361	-0.957433456	5.99E-03	decrease
chr11	71928669	71929069	-0.99459781	6.00E-03	decrease
chr1	70410837	70411237	-0.694977658	6.01E-03	decrease
chr19	38596202	38596602	-1.061555284	6.01E-03	decrease
chr3	107522775	107523175	-1.133733854	6.01E-03	decrease
chr1	226109497	226109897	-0.995356741	6.02E-03	decrease
chr11	125592516	125592916	-0.810951524	6.03E-03	decrease
chr6	56542625	56543025	-0.98134659	6.03E-03	decrease
chr15	92904102	92904502	-1.008628143	6.06E-03	decrease
chr9	113339944	113340344	-1.055716258	6.06E-03	decrease
chr1	56531693	56532093	-1.06034496	6.06E-03	decrease
chr6	25992679	25993079	-0.981119702	6.07E-03	decrease
chr10	124418950	124419350	-1.075044662	6.08E-03	decrease
chr12	56127979	56128379	-0.702776162	6.08E-03	decrease
chr6	26659582	26659982	-0.740964463	6.09E-03	decrease
chr1	174999292	174999692	-0.785116866	6.09E-03	decrease
chr1	1692988	1693388	-1.168637782	6.10E-03	decrease
chr12	88141941	88142341	-0.890059433	6.11E-03	decrease
chr11	125625835	125626235	-0.892339591	6.12E-03	decrease
chr19	12890795	12891195	-0.819789699	6.12E-03	decrease
chr19	35740654	35741054	-0.742341374	6.14E-03	decrease
chr16	28846354	28846754	-0.84735231	6.14E-03	decrease
chr17	51260074	51260474	-0.788878116	6.14E-03	decrease
chr1	61076729	61077129	-0.953463067	6.16E-03	decrease
chr12	22624789	22625189	-1.025360037	6.16E-03	decrease
chr1	113930345	113930745	-0.983772123	6.17E-03	decrease
chr6	153002712	153003112	-1.038065752	6.19E-03	decrease
chr3	126703790	126704190	-1.061457696	6.20E-03	decrease
chr1	58699867	58700267	-0.879648878	6.20E-03	decrease
chr19	10106158	10106558	-1.022815212	6.21E-03	decrease
chr9	122931378	122931778	-0.963593196	6.21E-03	decrease
chr21	33324549	33324949	-0.768554297	6.24E-03	decrease
chr19	1444717	1445117	-0.905284419	6.25E-03	decrease
chr11	33736238	33736638	-1.087836169	6.28E-03	decrease
chr12	14770304	14770704	-1.042697558	6.29E-03	decrease
chr12	69585421	69585821	-0.939328774	6.30E-03	decrease
chr14	69398322	69398722	-0.969185125	6.30E-03	decrease
chr14	54565446	54565846	-1.166689202	6.31E-03	decrease
chr16	67660197	67660597	-0.837661099	6.31E-03	decrease
chr20	5119676	5120076	-0.79011765	6.32E-03	decrease
chr4	80072775	80073175	-1.108608246	6.33E-03	decrease
chr19	50724513	50724913	-0.899658349	6.34E-03	decrease
chr1	21783280	21783680	-1.018864319	6.34E-03	decrease
chr1	213051404	213051804	-1.05233617	6.35E-03	decrease
chr13	74324666	74325066	-1.06318201	6.36E-03	decrease
chr1	37692078	37692478	-0.855117124	6.36E-03	decrease
chr19	12440843	12441243	-1.112883273	6.39E-03	decrease
chr12	101408170	101408570	-1.086392666	6.40E-03	decrease
chr19	12696592	12696992	-0.813445204	6.41E-03	decrease
chr15	89243831	89244231	-0.897700087	6.42E-03	decrease
chr19	18192771	18193171	-0.887492334	6.45E-03	decrease
chr2	28894120	28894520	-1.028363704	6.47E-03	decrease
chr1	203305400	203305800	-1.041522489	6.48E-03	decrease
chr11	61776549	61776949	-0.987362125	6.49E-03	decrease
chr3	129161410	129161810	-0.949500569	6.53E-03	decrease
chr19	10653796	10654196	-0.996011631	6.54E-03	decrease

chr2	97664138	97664538	-0.954263244		6.55E-03	decrease
chr22	20858152	20858552	-1.060421276		6.57E-03	decrease
chr22	40651738	40652138	-0.891844362		6.59E-03	decrease
chr9	122940988	122941388	-1.064695831		6.59E-03	decrease
chr16	89948030	89948430	-0.965518059		6.60E-03	decrease
chr1	161749890	161750290	-0.592616495		6.60E-03	decrease
chr12	21657726	21658126	-0.948926671		6.62E-03	decrease
chr4	44678204	44678604	-1.145418862		6.65E-03	decrease
chr1	230642438	230642838	-0.928538577		6.65E-03	decrease
chrUn_KI270467v1	2457	2857	0.379553127		6.67E-03	increase
chr20	347911	348311	-1.056067747		6.68E-03	decrease
chr10	92290719	92291119	-1.03089518		6.69E-03	decrease
chr1	167273036	167273436	-1.15989904		6.69E-03	decrease
chr10	15096908	15097308	-1.009798253		6.69E-03	decrease
chr17	40417736	40418136	-0.892300968		6.70E-03	decrease
chr22	23717044	23717444	-1.050528196		6.71E-03	decrease
chr1	22052339	22052739	-1.112818		6.71E-03	decrease
chr6	132512788	132513188	-1.034610278		6.72E-03	decrease
chr4	98995040	98995440	-1.086891403		6.72E-03	decrease
chr1	40257824	40258224	-0.912697379		6.74E-03	decrease
chr17	41786662	41787062	-0.983002379		6.74E-03	decrease
chr7	130205106	130205506	-0.969516531		6.75E-03	decrease
chr13	75537820	75538220	-0.752154713		6.75E-03	decrease
chr5	87268114	87268843	-1.053653574		6.76E-03	decrease
chr3	87226969	87227369	-1.059136969		6.76E-03	decrease
chr17	38853793	38854193	-0.872498109		6.76E-03	decrease
chr3	47380876	47381276	-0.857950559		6.76E-03	decrease
chrX	119791627	119792027	-0.784338988		6.77E-03	decrease
chr9	35732363	35732763	-0.699234172		6.78E-03	decrease
chr5	81971970	81972370	-1.131711351		6.79E-03	decrease
chr8	70668795	70669195	-1.079728618		6.79E-03	decrease
chr4	82430298	82430698	-1.010586218		6.79E-03	decrease
chr19	51108144	51108544	-0.971482742		6.80E-03	decrease
chr20	23357956	23358356	-0.946791796		6.81E-03	decrease
chr13	38686690	38687090	-1.01750583		6.81E-03	decrease
chr13	44989323	44989723	-1.044413251		6.82E-03	decrease
chr6	63635165	63635565	-1.136113205		6.83E-03	decrease
chr11	94493809	94494209	-0.903274677		6.83E-03	decrease
chr2	213284289	213284689	-1.077392149		6.85E-03	decrease
chr5	73565458	73565858	-0.920337881		6.86E-03	decrease
chr10	68074853	68075253	-0.890333312		6.87E-03	decrease
chr1	9910487	9910887	-0.982239314		6.87E-03	decrease
chr16	71895094	71895494	-1.082248335		6.89E-03	decrease
chr12	51047831	51048231	-0.878679523		6.90E-03	decrease
chr3	23916794	23917194	-0.8397796		6.90E-03	decrease
chr12	56333846	56334246	-0.869318617		6.91E-03	decrease
chr12	55928046	55928446	-1.098226992		6.91E-03	decrease
chr3	169661588	169661988	-1.07060195		6.95E-03	decrease
chr19	42325264	42325664	-0.819351718		6.95E-03	decrease
chr1	160031521	160031921	-1.017523576		6.96E-03	decrease
chr14	60248365	60248765	-1.064157462		6.96E-03	decrease
chr15	68819620	68820020	-1.12446814		6.96E-03	decrease
chr5	75717294	75717694	-0.978110607		6.98E-03	decrease
chr6	136289715	136290115	-0.720415274		6.99E-03	decrease
chr9	76394792	76395192	-1.147491944		7.00E-03	decrease
chr4	83034832	83035232	-0.758916997		7.02E-03	decrease
chr6	27807610	27808010	-0.636732289		7.02E-03	decrease
chr22	20702829	20703229	-0.916731184		7.04E-03	decrease
chr11	14891982	14892382	-1.003690146		7.04E-03	decrease
chr14	24232378	24232778	-0.971624482		7.04E-03	decrease
chr19	38387886	38388286	-0.91624653		7.05E-03	decrease
chrX	48676374	48676774	-1.101023653		7.06E-03	decrease
chr3	31532071	31532471	-0.92519758		7.07E-03	decrease
chr12	118016602	118017002	-0.962476103		7.07E-03	decrease
chr17	44186525	44186925	-0.794312095		7.08E-03	decrease
chr10	69179917	69180317	-0.829023736		7.08E-03	decrease
chr19	58362441	58362841	-0.817496168		7.09E-03	decrease
chr13	78658882	78659282	-0.972608641		7.10E-03	decrease
chr9	18976697	18977097	-1.024481027		7.11E-03	decrease
chrX	155071345	155071745	-0.884252943		7.11E-03	decrease
chr3	150603038	150603438	-0.882346797		7.14E-03	decrease
chr10	118754944	118755344	-0.907873214		7.15E-03	decrease
chr1	23484390	23484790	-1.094908154		7.15E-03	decrease
chr12	53295125	53295827	-0.81393657		7.16E-03	decrease
chr1	10034673	10035073	-1.144713559		7.16E-03	decrease
chr10	97686800	97687200	-1.001773126		7.17E-03	decrease
chr19	9834851	9835251	-0.905106495		7.17E-03	decrease

chr4	102760599	102760999	-1.035400211	7.18E-03	decrease
chr13	49444074	49444474	-0.852715506	7.19E-03	decrease
chr16	87317083	87317483	-0.952746431	7.20E-03	decrease
chr12	8949727	8950127	-1.044675339	7.21E-03	decrease
chr20	63973670	63974070	-1.012968126	7.24E-03	decrease
chr1	204493870	204494270	-0.991861459	7.25E-03	decrease
chr2	186485972	186486372	-0.812886512	7.25E-03	decrease
chr6	57089827	57090227	-0.966488017	7.26E-03	decrease
chr19	50376445	50376845	-0.922713217	7.26E-03	decrease
chr14	64387825	64388225	-0.935063428	7.28E-03	decrease
chr17	75516134	75516534	-0.906190334	7.28E-03	decrease
chr15	66701456	66701856	-0.822452457	7.28E-03	decrease
chr4	152779893	152780293	-0.968154843	7.29E-03	decrease
chr1	155135408	155135808	-0.873321618	7.29E-03	decrease
chr17	28743491	28743891	-0.893748865	7.29E-03	decrease
chr1	162562150	162562550	-0.984868512	7.31E-03	decrease
chr2	177224570	177224970	-0.960644162	7.31E-03	decrease
chr19	12881244	12881644	-0.935029886	7.31E-03	decrease
chr2	73737158	73737558	-1.017139932	7.37E-03	decrease
chr2	135531036	135531436	-0.758666113	7.42E-03	decrease
chr10	73730103	73730503	-1.004053033	7.44E-03	decrease
chr5	163459891	163460291	-0.988909445	7.45E-03	decrease
chr11	43680440	43680840	-0.915244489	7.45E-03	decrease
chr19	40750748	40751148	-0.761565001	7.46E-03	decrease
chr2	151409774	151410174	-0.941156204	7.46E-03	decrease
chr4	18021957	18022357	-0.974258575	7.48E-03	decrease
chr10	63268913	63269313	-1.087998958	7.48E-03	decrease
chr11	110092328	110092728	-1.125964716	7.49E-03	decrease
chr5	43514741	43515141	-1.106824839	7.51E-03	decrease
chr17	41930256	41930656	-0.90075485	7.52E-03	decrease
chr2	38875666	38876066	-0.8648551	7.53E-03	decrease
chr12	121794488	121794888	-0.840211141	7.53E-03	decrease
chr6	27097048	27097448	-0.603839363	7.55E-03	decrease
chr1	54199781	54200181	-0.938754703	7.56E-03	decrease
chr15	58771202	58771602	-1.06428905	7.58E-03	decrease
chr17	45247482	45247882	-0.935542918	7.60E-03	decrease
chr2	197515817	197516217	-0.990415651	7.60E-03	decrease
chr11	110305459	110305859	-1.06191727	7.62E-03	decrease
chrX	104166022	104166422	-0.991974666	7.62E-03	decrease
chr19	506789	507189	-0.806420218	7.63E-03	decrease
chr16	50741711	50742111	-1.049844659	7.63E-03	decrease
chr5	134904301	134904701	-1.06937372	7.64E-03	decrease
chr22	37849244	37849644	-0.84724564	7.64E-03	decrease
chr3	153161195	153161595	-0.846599337	7.65E-03	decrease
chr12	49750239	49750639	-1.093430094	7.66E-03	decrease
chr1	31644678	31645078	-0.814357821	7.66E-03	decrease
chr19	6460257	6460657	-0.887499307	7.68E-03	decrease
chr9	34376841	34377241	-0.941538256	7.69E-03	decrease
chr9	78296960	78297360	-0.987409924	7.71E-03	decrease
chr11	62855882	62856282	-0.775362335	7.71E-03	decrease
chr3	48473003	48473403	-0.981447409	7.72E-03	decrease
chr10	21525591	21525991	-0.97152139	7.72E-03	decrease
chr3	9731251	9731651	-0.886445835	7.75E-03	decrease
chr16	4734386	4734786	-0.99790924	7.78E-03	decrease
chr1	153627254	153627654	-0.783034054	7.78E-03	decrease
chr12	48197914	48198314	-0.968706386	7.81E-03	decrease
chr17	44141461	44141861	-0.950985867	7.81E-03	decrease
chr5	55307515	55307915	-1.02781551	7.81E-03	decrease
chr2	48315535	48315935	-1.070484954	7.81E-03	decrease
chr4	6673650	6674050	-1.057328455	7.82E-03	decrease
chr5	34915245	34915645	-0.863323953	7.83E-03	decrease
chr4	17810551	17810951	-0.84009155	7.84E-03	decrease
chr6	42979205	42979605	-1.022519327	7.84E-03	decrease
chr6	105176317	105176717	-1.129332146	7.89E-03	decrease
chr14	52552390	52552790	-1.019349948	7.90E-03	decrease
chr3	101574633	101575033	-1.045103227	7.92E-03	decrease
chr17	45148574	45148974	-1.002733179	7.96E-03	decrease
chr16	68310860	68311260	-0.879603589	7.96E-03	decrease
chr22	19144579	19144979	-0.931186664	7.98E-03	decrease
chr7	134986465	134986865	-0.934397321	7.99E-03	decrease
chr22	25333839	25334239	-1.107267444	7.99E-03	decrease
chr2	109613927	109614327	-1.028268372	7.99E-03	decrease
chr14	64338150	64338550	-0.975944237	8.00E-03	decrease
chr5	62412400	62412800	-0.843289578	8.00E-03	decrease
chr19	49665462	49665862	-0.863692264	8.02E-03	decrease
chr16	3400886	3401286	-0.966879892	8.04E-03	decrease
chrX	154804815	154805215	-1.079177778	8.06E-03	decrease

chr16	75517262	75517662	-1.01932839	8.08E-03	decrease
chr1	32179264	32179664	-0.950712589	8.08E-03	decrease
chr18	9137831	9138231	-1.131262338	8.09E-03	decrease
chr17	80414825	80415225	-0.931406212	8.10E-03	decrease
chr6	10722874	10723274	-0.986809504	8.10E-03	decrease
chr1	28505575	28505975	-0.914781656	8.11E-03	decrease
chr11	10750823	10751223	-0.945798922	8.12E-03	decrease
chrX	149540630	149541030	-0.818877627	8.12E-03	decrease
chr9	34126404	34126804	-1.061817661	8.12E-03	decrease
chr5	146203297	146203697	-0.905533579	8.13E-03	decrease
chr21	33230290	33230690	-0.96833317	8.13E-03	decrease
chr17	77373929	77374329	-1.020977308	8.17E-03	decrease
chr12	64759281	64759681	-1.075671432	8.17E-03	decrease
chr12	6961855	6962255	-0.712951795	8.17E-03	decrease
chr17	50867210	50867610	-0.855409299	8.17E-03	decrease
chr8	98825992	98826392	-1.096721082	8.18E-03	decrease
chr2	108786328	108786728	-0.961890043	8.20E-03	decrease
chr15	44427109	44427509	-0.911281108	8.20E-03	decrease
chr7	26864466	26864866	-0.900840094	8.21E-03	decrease
chr22	28741998	28742398	-0.876762232	8.21E-03	decrease
chrX	54639593	54639993	-0.947304638	8.22E-03	decrease
chr22	40370315	40370715	-1.048436149	8.22E-03	decrease
chr4	9691500	9691900	-0.978646691	8.22E-03	decrease
chr14	62739919	62740319	-1.115122714	8.22E-03	decrease
chr10	87863011	87863411	-1.003133254	8.23E-03	decrease
chr1	246931895	246932295	-0.963458428	8.23E-03	decrease
chr1	117605524	117605924	-1.005679261	8.24E-03	decrease
chr10	90871611	90872011	-0.975192238	8.24E-03	decrease
chr12	121803031	121803431	-0.951074035	8.24E-03	decrease
chr7	92528363	92528763	-0.929292938	8.25E-03	decrease
chr15	72226706	72227106	-0.904067393	8.25E-03	decrease
chr15	77420290	77420690	-0.943295955	8.26E-03	decrease
chr7	48088436	48088836	-1.090460976	8.29E-03	decrease
chr11	22829486	22829886	-1.083958083	8.30E-03	decrease
chr4	128287917	128288317	-0.934346986	8.30E-03	decrease
chr4	102431232	102431632	-1.1285336	8.31E-03	decrease
chr6	28399176	28399576	-0.810168123	8.32E-03	decrease
chr7	90211436	90211836	-1.119726698	8.33E-03	decrease
chr1	182869208	182869608	-0.910668024	8.34E-03	decrease
chrX	108736509	108736909	-0.973398348	8.35E-03	decrease
chr19	16496118	16496518	-0.839019924	8.35E-03	decrease
chr11	809443	809843	-0.828114249	8.38E-03	decrease
chr18	36067282	36067682	-0.962625963	8.39E-03	decrease
chr13	19863485	19863885	-1.04705151	8.40E-03	decrease
chr4	118591673	118592073	-1.111960188	8.40E-03	decrease
chr1	161045662	161046062	-0.892122444	8.42E-03	decrease
chr19	48501596	48501996	-0.978462698	8.42E-03	decrease
chr16	19555353	19555753	-0.79454601	8.43E-03	decrease
chr4	121823355	121823755	-0.914625026	8.43E-03	decrease
chr6	42984038	42984438	-0.945726093	8.44E-03	decrease
chr2	111884130	111884530	-0.969258405	8.45E-03	decrease
chr9	26892371	26892771	-1.047975815	8.46E-03	decrease
chr2	38777838	38778238	-0.981348671	8.47E-03	decrease
chr2	27489607	27490007	-0.856780083	8.50E-03	decrease
chr12	6336387	6336787	-0.838790067	8.51E-03	decrease
chr20	63290996	63291396	-0.916984186	8.52E-03	decrease
chr19	46859981	46860381	-1.001952008	8.52E-03	decrease
chr6	75284807	75285207	-1.046834694	8.53E-03	decrease
chr18	21740592	21740992	-0.938545628	8.53E-03	decrease
chr9	128947230	128947630	-1.017310958	8.57E-03	decrease
chr1	3857114	3857514	-0.964342218	8.57E-03	decrease
chr9	33076512	33076912	-0.894885636	8.58E-03	decrease
chr1	27959587	27959987	-1.067773542	8.59E-03	decrease
chr6	41787182	41787582	-0.797637894	8.60E-03	decrease
chr14	36835089	36835489	-1.036123899	8.61E-03	decrease
chr1	3900676	3901076	-1.006520066	8.62E-03	decrease
chr10	102403825	102404225	-0.936899935	8.62E-03	decrease
chr1	151347018	151347418	-0.871067401	8.62E-03	decrease
chr10	73625491	73625891	-1.082468976	8.63E-03	decrease
chr15	88467297	88467697	-0.964078923	8.63E-03	decrease
chr5	179858743	179859143	-0.84761281	8.64E-03	decrease
chr3	168095652	168096052	-0.95052009	8.65E-03	decrease
chr6	158999860	159000260	-0.87235797	8.65E-03	decrease
chr1	206612577	206612977	-1.062768086	8.66E-03	decrease
chr19	47427627	47428027	-0.960687775	8.68E-03	decrease
chr19	3505854	3506254	-0.901519468	8.68E-03	decrease
chr19	14006380	14006780	-0.921207612	8.70E-03	decrease

chr21	42009943	42010343	-0.997911317	8.71E-03	decrease
chr16	75623146	75623546	-1.021252878	8.72E-03	decrease
chr2	158456478	158456878	-1.100933049	8.73E-03	decrease
chr16	30650478	30650878	-0.755032978	8.74E-03	decrease
chr19	5790640	5791040	-0.80553516	8.76E-03	decrease
chr1	55214030	55214430	-0.821003032	8.76E-03	decrease
chr19	49850618	49851018	-0.925308399	8.76E-03	decrease
chr2	85132938	85133338	-1.01095691	8.78E-03	decrease
chr8	144787402	144787802	-0.890076029	8.79E-03	decrease
chr20	2658544	2658944	-0.832438458	8.83E-03	decrease
chr19	49813303	49813703	-0.759047603	8.86E-03	decrease
chr13	41696536	41696936	-1.117979452	8.86E-03	decrease
chr8	124474385	124474785	-0.937936342	8.86E-03	decrease
chr2	3518771	3519171	-0.975216586	8.86E-03	decrease
chr1	67700703	67701103	-0.848343109	8.86E-03	decrease
chr19	44093924	44094324	-1.113459734	8.87E-03	decrease
chr17	3636372	3636772	-0.864278637	8.87E-03	decrease
chr19	40603812	40604212	-0.823936117	8.87E-03	decrease
chr2	68467213	68467613	-1.094037692	8.88E-03	decrease
chr19	4342721	4343121	-0.77088676	8.88E-03	decrease
chrX	72181352	72181752	-0.86988696	8.89E-03	decrease
chr19	12610795	12611195	-0.867310098	8.91E-03	decrease
chr2	241635452	241635852	-1.094613292	8.93E-03	decrease
chr16	70778972	70779372	-0.903562604	8.93E-03	decrease
chr12	57112022	57112422	-0.880474602	8.96E-03	decrease
chr10	73966011	73966411	-0.978291307	8.96E-03	decrease
chr19	36838247	36838647	-0.939455778	8.97E-03	decrease
chr1	63523226	63523626	-0.864907591	8.97E-03	decrease
chr22	21641599	21641999	-0.917634566	8.98E-03	decrease
chr2	112254792	112255192	-1.060526182	9.00E-03	decrease
chr1	151059428	151059828	-0.819358248	9.01E-03	decrease
chr16	11742885	11743285	-0.987394983	9.01E-03	decrease
chr7	99504653	99505053	-0.815913791	9.01E-03	decrease
chr19	29844818	29845218	-0.916781356	9.02E-03	decrease
chr7	72829473	72829873	-0.987419685	9.03E-03	decrease
chr2	191678015	191678415	-1.046811365	9.04E-03	decrease
chr6	57317036	57317436	-1.107182443	9.05E-03	decrease
chr13	27620597	27620997	-1.028163642	9.05E-03	decrease
chr13	91347071	91347471	-1.004517183	9.09E-03	decrease
chr19	1605536	1605936	-1.012612318	9.09E-03	decrease
chr1	107056896	107057296	-1.01486316	9.09E-03	decrease
chr13	74132667	74133067	-0.997417146	9.10E-03	decrease
chr16	58630225	58630625	-0.715958816	9.11E-03	decrease
chr3	143001199	143001599	-0.947508903	9.12E-03	decrease
chr9	38069577	38069977	-0.92227129	9.14E-03	decrease
chr14	24442358	24442758	-0.810700529	9.17E-03	decrease
chr1	236604430	236604830	-0.698446343	9.17E-03	decrease
chr1	18902521	18902921	-0.950482747	9.18E-03	decrease
chr19	46661343	46661743	-0.767470871	9.18E-03	decrease
chr6	113971103	113971503	-0.890325741	9.19E-03	decrease
chr4	174255866	174256266	-1.009654568	9.20E-03	decrease
chr17	8201114	8201514	-0.910905328	9.21E-03	decrease
chr19	3500635	3501035	-0.939292565	9.21E-03	decrease
chr1	28328892	28329292	-0.896918123	9.22E-03	decrease
chr6	26171979	26172379	-0.768828827	9.23E-03	decrease
chr1	231337764	231338164	-0.700961463	9.24E-03	decrease
chr17	4703974	4704374	-1.011793678	9.24E-03	decrease
chr4	1167177	1167577	-0.997204141	9.24E-03	decrease
chrX	112840834	112841234	-0.859094033	9.25E-03	decrease
chr3	47824620	47825020	-1.044198945	9.27E-03	decrease
chr11	67508350	67508750	-1.109237232	9.28E-03	decrease
chr16	68023224	68023624	-1.026109258	9.28E-03	decrease
chr4	87006761	87007161	-0.926666319	9.29E-03	decrease
chr5	40835240	40835640	-0.879517329	9.29E-03	decrease
chr13	45464863	45465263	-0.944144725	9.36E-03	decrease
chr2	24971671	24972071	-0.892447977	9.36E-03	decrease
chr15	85296677	85297077	-1.059693562	9.37E-03	decrease
chr17	28719596	28719996	-0.716293348	9.38E-03	decrease
chr10	91409652	91410052	-1.012878608	9.41E-03	decrease
chr2	173965456	173965856	-1.084266447	9.41E-03	decrease
chr3	194573587	194573987	-1.110392198	9.42E-03	decrease
chr11	134253137	134253537	-0.963579278	9.43E-03	decrease
chr20	45363061	45363461	-0.904235275	9.43E-03	decrease
chr5	122076790	122077190	-1.09786924	9.43E-03	decrease
chr19	17873833	17874233	-1.006550927	9.45E-03	decrease
chr12	123436456	123436856	-0.953177236	9.46E-03	decrease
chr2	173354385	173354785	-1.071029141	9.47E-03	decrease

chr20	50509870	50510270	-1.059586242	9.47E-03	decrease
chr3	136752710	136753110	-1.075306922	9.49E-03	decrease
chr7	25862235	25862635	-0.762932611	9.49E-03	decrease
chr7	66996616	66997016	-0.842233816	9.52E-03	decrease
chr22	41941465	41941865	-0.93608766	9.52E-03	decrease
chr17	27294334	27294734	-1.038707198	9.52E-03	decrease
chr1	151327379	151327779	-0.905266785	9.54E-03	decrease
chr2	61538245	61538645	-0.780373336	9.55E-03	decrease
chr1	81803350	81803750	-1.034961496	9.55E-03	decrease
chr5	43313302	43313702	-0.960572144	9.55E-03	decrease
chr14	104827459	104827859	-0.972775741	9.56E-03	decrease
chr1	235867049	235867449	-0.857254885	9.59E-03	decrease
chrX	136496562	136496962	-1.067875069	9.61E-03	decrease
chr17	8200184	8200584	-0.774552104	9.61E-03	decrease
chr15	84747563	84747963	-1.02753785	9.62E-03	decrease
chr16	89910063	89910463	-0.955030481	9.62E-03	decrease
chr1	224114251	224114651	-0.989960272	9.64E-03	decrease
chr14	52791222	52791622	-1.039465591	9.65E-03	decrease
chr11	64269970	64270370	-0.721377698	9.67E-03	decrease
chr7	76318099	76318499	-0.889575686	9.68E-03	decrease
chr19	48363952	48364352	-0.795941364	9.73E-03	decrease
chr2	177597757	177598157	-1.061102282	9.73E-03	decrease
chr14	23286123	23286523	-0.838988178	9.74E-03	decrease
chr6	45378093	45378493	-1.039285543	9.75E-03	decrease
chr3	12664162	12664562	-1.036400494	9.77E-03	decrease
chr12	53441349	53441749	-0.765953673	9.77E-03	decrease
chr2	203238873	203239273	-0.950208674	9.79E-03	decrease
chr6	159693554	159693954	-0.935435239	9.79E-03	decrease
chr16	68084553	68084953	-0.960312151	9.79E-03	decrease
chr1	209784451	209784851	-0.961140579	9.82E-03	decrease
chr19	15556011	15556411	-0.93654119	9.83E-03	decrease
chr4	83455617	83456017	-0.920517681	9.83E-03	decrease
chr19	47130742	47131142	-0.922058587	9.83E-03	decrease
chr22	37933827	37934227	-0.834644958	9.84E-03	decrease
chr20	34955575	34955975	-0.870044627	9.84E-03	decrease
chr7	87345242	87345642	-0.862645814	9.85E-03	decrease
chr4	474044	474444	-0.820941338	9.89E-03	decrease
chr7	36152758	36153158	-0.825888741	9.90E-03	decrease
chr11	65027411	65027811	-0.999863922	9.91E-03	decrease
chr1	114511199	114511599	-1.039233215	9.92E-03	decrease
chr9	6703943	6704343	-1.03986282	9.96E-03	decrease
chr2	86440716	86441116	-0.785479641	9.98E-03	decrease
chr1	84479192	84479592	-0.999639459	9.99E-03	decrease
chr7	135977256	135977656	-0.881446294	1.00E-02	decrease
chrX	47361808	47362208	-0.866499374	1.00E-02	decrease
chr5	113203182	113203582	-0.953520834	1.00E-02	decrease
chr1	16644322	16644722	-0.853468757	1.00E-02	decrease
chr12	6568328	6568728	-0.777669797	1.00E-02	decrease
chr7	42888417	42888817	-0.989002529	1.00E-02	decrease
chr9	128322420	128322820	-0.921870052	1.00E-02	decrease
chr10	104232320	104232720	-0.963531719	1.01E-02	decrease
chr5	157731328	157731728	-0.899816517	1.01E-02	decrease
chr4	55546870	55547270	-1.061898972	1.01E-02	decrease
chr11	65711996	65712396	-0.76624997	1.01E-02	decrease
chr9	72365366	72365766	-1.013666069	1.01E-02	decrease
chr5	95731829	95732229	-1.099602831	1.01E-02	decrease
chr1	205122120	205122520	-0.813715876	1.01E-02	decrease
chr8	91070130	91070530	-0.971540638	1.01E-02	decrease
chr15	40564860	40565260	-0.834037516	1.01E-02	decrease
chr12	108731706	108732106	-0.7496227	1.01E-02	decrease
chr3	48240982	48241382	-0.869386281	1.01E-02	decrease
chr6	79233927	79234327	-0.929152116	1.02E-02	decrease
chr12	57771942	57772342	-0.784846325	1.02E-02	decrease
chr14	75002535	75002935	-0.877687098	1.02E-02	decrease
chr12	116738174	116738574	-0.907976475	1.02E-02	decrease
chr15	75180758	75181158	-0.888819309	1.02E-02	decrease
chr14	24242309	24242709	-0.838201762	1.02E-02	decrease
chr19	45862999	45863399	-0.741200303	1.02E-02	decrease
chr22	36239441	36239841	-0.971554582	1.02E-02	decrease
chr8	98116985	98117385	-0.883549952	1.02E-02	decrease
chr20	25623873	25624273	-1.022386579	1.02E-02	decrease
chr19	18522161	18522561	-0.797786706	1.02E-02	decrease
chr1	211259333	211259733	-1.097759341	1.03E-02	decrease
chr4	39638976	39639376	-1.003221469	1.03E-02	decrease
chr5	178626302	178626702	-0.98494538	1.03E-02	decrease
chr3	160755124	160755524	-0.859175065	1.03E-02	decrease
chr11	82900626	82901026	-0.795164957	1.03E-02	decrease

chr20	32357989	32358389	-0.894453076	1.03E-02	decrease
chr13	51452913	51453313	-1.039681126	1.03E-02	decrease
chr2	131673353	131673753	-1.067663371	1.03E-02	decrease
chr22	31399400	31399800	-0.89573255	1.04E-02	decrease
chr4	127880968	127881368	-0.771428169	1.04E-02	decrease
chr5	90529407	90529807	-0.894936886	1.04E-02	decrease
chr17	28645257	28645657	-0.986472814	1.04E-02	decrease
chr19	32675178	32675578	-0.946695345	1.04E-02	decrease
chr14	73458549	73458949	-0.904441547	1.04E-02	decrease
chr1	15526573	15526973	-0.920595402	1.04E-02	decrease
chr3	49939804	49940204	-0.898426244	1.04E-02	decrease
chr2	195656902	195657302	-1.000357988	1.04E-02	decrease
chr5	66144326	66144726	-0.913396276	1.04E-02	decrease
chr3	112086387	112086787	-0.997726468	1.04E-02	decrease
chr1	228130875	228131275	-0.891390924	1.05E-02	decrease
chr1	53945944	53946344	-1.031080101	1.05E-02	decrease
chr1	119140312	119140712	-0.7702834	1.05E-02	decrease
chr16	30075641	30076041	-0.923965879	1.05E-02	decrease
chr5	163504709	163505109	-1.069215137	1.05E-02	decrease
chr16	31508046	31508446	-1.06762104	1.05E-02	decrease
chr14	34982470	34982870	-0.739358566	1.05E-02	decrease
chr17	29591488	29591888	-0.816492447	1.05E-02	decrease
chr3	155854395	155854795	-0.835801903	1.06E-02	decrease
chr3	50337322	50337722	-0.859893781	1.06E-02	decrease
chr14	54396636	54397297	-0.898174121	1.06E-02	decrease
chr21	46635520	46635920	-0.88860711	1.06E-02	decrease
chr7	100867106	100867506	-1.000632386	1.06E-02	decrease
chr9	34049124	34049524	-0.675082183	1.06E-02	decrease
chr11	65497630	65498030	-1.009053066	1.06E-02	decrease
chr9	109013599	109013999	-1.019672435	1.06E-02	decrease
chr8	92965871	92966271	-0.973224458	1.06E-02	decrease
chr1	92298639	92299039	-0.864104752	1.07E-02	decrease
chr13	72648330	72648730	-1.079177873	1.07E-02	decrease
chr20	37527787	37528187	-0.97022515	1.07E-02	decrease
chr12	120437923	120438323	-0.842342304	1.07E-02	decrease
chr20	2664259	2664659	-1.001166693	1.07E-02	decrease
chr3	152269759	152270159	-0.979375384	1.08E-02	decrease
chr15	82750231	82750631	-0.954248887	1.08E-02	decrease
chr5	178164216	178164616	-1.017052876	1.08E-02	decrease
chr1	6785077	6785477	-1.056818795	1.08E-02	decrease
chr11	108121216	108121616	-0.947663721	1.08E-02	decrease
chr11	15074750	15075150	-0.917466129	1.08E-02	decrease
chr17	73231946	73232711	-0.853229391	1.08E-02	decrease
chr14	56579434	56579834	-0.991086787	1.08E-02	decrease
chr7	149330859	149331259	-0.63456554	1.08E-02	decrease
chr16	23557180	23557580	-0.78126301	1.08E-02	decrease
chr8	144792424	144792824	-0.836583695	1.09E-02	decrease
chr1	35079141	35079541	-0.979258704	1.09E-02	decrease
chr7	156640598	156640998	-1.005277537	1.09E-02	decrease
chr17	31901433	31901833	-0.806534577	1.09E-02	decrease
chr3	27369079	27369479	-1.019539958	1.09E-02	decrease
chr7	25856925	25857325	-0.840019373	1.09E-02	decrease
chr7	99465855	99466255	-0.884607075	1.09E-02	decrease
chr2	231052848	231053248	-0.922745707	1.09E-02	decrease
chrX	48539670	48540070	-0.973010778	1.09E-02	decrease
chr6	170584670	170585070	-0.93666917	1.10E-02	decrease
chr14	21103783	21104183	-0.708872458	1.10E-02	decrease
chr12	68807520	68807920	-0.98827071	1.10E-02	decrease
chr4	139300981	139301381	-0.906756218	1.10E-02	decrease
chr10	97640293	97640693	-0.874493687	1.10E-02	decrease
chr3	113019288	113019688	-1.044470952	1.10E-02	decrease
chr9	7165193	7165593	-0.858305929	1.10E-02	decrease
chr5	171415083	171415483	-0.867293374	1.11E-02	decrease
chr14	74084504	74084904	-0.672717968	1.11E-02	decrease
chr14	36661742	36662142	-0.946504356	1.11E-02	decrease
chr7	32490087	32490487	-0.999172588	1.11E-02	decrease
chr1	186680366	186680766	-1.009868032	1.11E-02	decrease
chr19	39833605	39834005	-0.779514897	1.11E-02	decrease
chr19	12840504	12840904	-0.834115388	1.12E-02	decrease
chr5	69166926	69167326	-0.902592055	1.12E-02	decrease
chr2	216498590	216498990	-0.93666288	1.12E-02	decrease
chr1	50969799	50970199	-0.992594811	1.12E-02	decrease
chr19	45423722	45424122	-0.690661332	1.12E-02	decrease
chr13	79528546	79528946	-0.690823548	1.12E-02	decrease
chr1	147084677	147085077	-0.735290541	1.12E-02	decrease
chr6	133952629	133953029	-0.975773549	1.12E-02	decrease
chr11	134224233	134224633	-0.951624565	1.13E-02	decrease

chr17	49764160	49764560	-0.826367936	1.13E-02	decrease
chrX	120603934	120604334	-0.928411071	1.13E-02	decrease
chr2	74198324	74198724	-0.924442702	1.13E-02	decrease
chr9	124862001	124862401	-0.786454691	1.13E-02	decrease
chr16	683345	683745	-0.860046262	1.13E-02	decrease
chr5	150441647	150442047	-0.944362108	1.13E-02	decrease
chr1	204411582	204411982	-0.731082698	1.13E-02	decrease
chr7	44986619	44987019	-0.853199621	1.13E-02	decrease
chr16	29943891	29944291	-0.747218491	1.13E-02	decrease
chrX	16836324	16836724	-1.046070658	1.13E-02	decrease
chr2	172085368	172085768	-0.878444247	1.13E-02	decrease
chr1	144412523	144412923	-0.780742377	1.14E-02	decrease
chr6	82247438	82247838	-1.076309027	1.14E-02	decrease
chrUn_KI270438v1	109234	109634	0.440025731	1.14E-02	increase
chr3	132722825	132723225	-1.04934975	1.14E-02	decrease
chr16	19067423	19067823	-0.909188061	1.14E-02	decrease
chr1	100266148	100266548	-0.864004348	1.14E-02	decrease
chr2	99336926	99337326	-0.851712346	1.14E-02	decrease
chr8	144413272	144413672	-0.919868902	1.14E-02	decrease
chr7	158856127	158856527	-0.824352649	1.15E-02	decrease
chrX	120469000	120469400	-0.889785439	1.15E-02	decrease
chr12	56316005	56316405	-0.704472509	1.15E-02	decrease
chr17	76029480	76029880	-0.811952863	1.15E-02	decrease
chr7	6348993	6349393	-0.961707715	1.15E-02	decrease
chr10	103772328	103772728	-0.975678702	1.15E-02	decrease
chr2	121074705	121075105	-0.965576923	1.15E-02	decrease
chr16	88857591	88857991	-0.938602205	1.16E-02	decrease
chr19	47047908	47048308	-0.81975935	1.16E-02	decrease
chr15	67521593	67521993	-0.926947988	1.16E-02	decrease
chr16	67160688	67161088	-0.940139775	1.16E-02	decrease
chr17	13024797	13025197	-1.079078253	1.16E-02	decrease
chrX	135032016	135032416	-0.803125434	1.17E-02	decrease
chr1	54053206	54053606	-0.808824391	1.17E-02	decrease
chr1	207752084	207752484	-0.937800216	1.17E-02	decrease
chr7	105532035	105532435	-0.809292064	1.17E-02	decrease
chr10	102918128	102918528	-0.942089083	1.17E-02	decrease
chr1	33036698	33037098	-0.70741004	1.17E-02	decrease
chrX	119887678	119888078	-0.851819815	1.17E-02	decrease
chr22	41544485	41544885	-0.862766572	1.18E-02	decrease
chr13	79481558	79481958	-0.93218064	1.18E-02	decrease
chr19	36139646	36140046	-0.824025923	1.18E-02	decrease
chr6	83192934	83193334	-1.041580582	1.19E-02	decrease
chr17	39235752	39236152	-0.898234158	1.19E-02	decrease
chr1	167220417	167220817	-0.880866877	1.19E-02	decrease
chr19	55259085	55259485	-0.89943443	1.19E-02	decrease
chr6	21953933	21954333	-0.962605673	1.19E-02	decrease
chr2	46616734	46617134	-0.981686697	1.19E-02	decrease
chr20	31607117	31607517	-0.884787507	1.19E-02	decrease
chr11	17208022	17208422	-0.972207848	1.19E-02	decrease
chr19	10333280	10333680	-0.707696215	1.19E-02	decrease
chr17	63848887	63849287	-0.914129369	1.19E-02	decrease
chr12	116506412	116506812	-1.017423476	1.19E-02	decrease
chr1	227730459	227730859	-0.774214233	1.19E-02	decrease
chr13	110706361	110706761	-1.002660701	1.19E-02	decrease
chr12	132274985	132275385	-0.89651307	1.20E-02	decrease
chr2	64751152	64751552	-0.86561968	1.20E-02	decrease
chr16	2968851	2969251	-0.771178799	1.20E-02	decrease
chr14	39267174	39267574	-0.88160781	1.20E-02	decrease
chr3	129183725	129184125	-0.932601648	1.20E-02	decrease
chr10	87504577	87504977	-0.958413908	1.21E-02	decrease
chr7	100081454	100081854	-0.897065693	1.21E-02	decrease
chr2	27429284	27429684	-0.975759198	1.21E-02	decrease
chr1	31715410	31715810	-0.701905488	1.21E-02	decrease
chr4	66565237	66565637	-1.045426773	1.21E-02	decrease
chr9	95875529	95875929	-0.911129677	1.22E-02	decrease
chr17	63600643	63601043	-0.972348503	1.22E-02	decrease
chr3	186570395	186570795	-0.89852274	1.22E-02	decrease
chr1	97921101	97921501	-1.056105441	1.22E-02	decrease
chrX	47144846	47145246	-0.810480017	1.22E-02	decrease
chr6	155314483	155314883	-0.68814061	1.23E-02	decrease
chr15	72473830	72474230	-0.863013447	1.23E-02	decrease
chr9	113275364	113275764	-0.830657141	1.23E-02	decrease
chr20	3846755	3847155	-1.013133063	1.23E-02	decrease
chr16	1611847	1612247	-0.841145623	1.24E-02	decrease
chr19	17327793	17328193	-0.902354334	1.24E-02	decrease
chr3	134485894	134486294	-0.916512083	1.24E-02	decrease
chr7	44036377	44036777	-0.912139755	1.24E-02	decrease

chr11	47248711	47249111	-0.867407166	1.24E-02	decrease
chr12	48716727	48717127	-0.725362836	1.24E-02	decrease
chr20	32109703	32110103	-0.90690223	1.24E-02	decrease
chr21	44428358	44428758	-0.968891919	1.24E-02	decrease
chr3	136861994	136862394	-0.915954153	1.24E-02	decrease
chr14	59484062	59484462	-0.885031349	1.25E-02	decrease
chr1	67686530	67686930	-0.776869359	1.25E-02	decrease
chr18	47150135	47150535	-1.033485314	1.25E-02	decrease
chr19	57240423	57240823	-0.997147426	1.26E-02	decrease
chr6	28141527	28141927	-0.67688408	1.26E-02	decrease
chr1	160343051	160343451	-0.839732181	1.26E-02	decrease
chr17	28335587	28335987	-0.758731541	1.26E-02	decrease
chr5	39074517	39074917	-1.052785904	1.26E-02	decrease
chr6	36028299	36028699	-0.951775677	1.26E-02	decrease
chr8	63168159	63168559	-1.011703901	1.26E-02	decrease
chr1	161192581	161192981	-0.897207474	1.27E-02	decrease
chr16	2223281	2223681	-0.862966902	1.27E-02	decrease
chr1	182588976	182589376	-0.979215967	1.27E-02	decrease
chr19	39397516	39397916	-0.807657029	1.27E-02	decrease
chr19	56140835	56141235	-0.902906932	1.27E-02	decrease
chr2	191013981	191014381	-1.008272611	1.28E-02	decrease
chr16	75565895	75566295	-0.975486415	1.28E-02	decrease
chr9	95509497	95509897	-1.050806192	1.28E-02	decrease
chr10	100185517	100185917	-0.932215495	1.28E-02	decrease
chr5	171365247	171365647	-0.977279094	1.28E-02	decrease
chr16	2027657	2028057	-0.869597774	1.29E-02	decrease
chr19	45671024	45671424	-0.977893879	1.29E-02	decrease
chr11	126211365	126211765	-0.865522285	1.29E-02	decrease
chr22	37658242	37658642	-0.834834767	1.29E-02	decrease
chr11	67606528	67606928	-0.967782701	1.29E-02	decrease
chr21	33915640	33916040	-0.749982893	1.29E-02	decrease
chr11	19712180	19712580	-1.047609038	1.29E-02	decrease
chr12	111685955	111686355	-0.959697318	1.29E-02	decrease
chr6	117602479	117602879	-0.90975682	1.29E-02	decrease
chr3	52154474	52154874	-0.829319664	1.30E-02	decrease
chr1	77773979	77774379	-0.987000049	1.30E-02	decrease
chr4	139556156	139556556	-0.929517965	1.30E-02	decrease
chr19	12712135	12712535	-0.843950699	1.30E-02	decrease
chr20	18288123	18288523	-0.914254573	1.30E-02	decrease
chr3	146160305	146160705	-1.050920944	1.30E-02	decrease
chr9	37485510	37485910	-0.936954581	1.30E-02	decrease
chr12	98645026	98645426	-0.961969392	1.30E-02	decrease
chr1	40374576	40374976	-0.918779896	1.30E-02	decrease
chr5	154858170	154858570	-0.935717686	1.31E-02	decrease
chr15	66386482	66386882	-0.880536913	1.31E-02	decrease
chrX	132022892	132023292	-1.043796914	1.31E-02	decrease
chr10	68331646	68332046	-0.757450078	1.31E-02	decrease
chr19	39497978	39498378	-1.025161366	1.31E-02	decrease
chr4	129093243	129093643	-0.948261901	1.31E-02	decrease
chrX	154516350	154516750	-0.927119056	1.31E-02	decrease
chr16	68843529	68843929	-0.948994251	1.31E-02	decrease
chr3	57597578	57597978	-0.947844756	1.32E-02	decrease
chr13	51583944	51584344	-0.756610724	1.32E-02	decrease
chr22	29766990	29767390	-0.739563802	1.32E-02	decrease
chr11	45917835	45918235	-0.773431897	1.32E-02	decrease
chr20	34876128	34876528	-0.852599284	1.32E-02	decrease
chr20	46689457	46689857	-0.977784839	1.32E-02	decrease
chr12	123365520	123365920	-0.777717028	1.32E-02	decrease
chr1	150163040	150163440	-0.899649762	1.32E-02	decrease
chr9	37592245	37592645	-1.010916302	1.32E-02	decrease
chr6	34223734	34224134	-1.046284636	1.32E-02	decrease
chr2	74507417	74507817	-0.669339649	1.33E-02	decrease
chr11	8964512	8964912	-0.852276058	1.33E-02	decrease
chr8	106657520	106657920	-1.015024814	1.33E-02	decrease
chr17	41688634	41689034	-0.579837018	1.33E-02	decrease
chr20	43657273	43657673	-0.903302063	1.33E-02	decrease
chr12	15881985	15882385	-0.847411889	1.34E-02	decrease
chr19	46704331	46704731	-0.946613228	1.34E-02	decrease
chr19	19776255	19776655	-0.796252323	1.34E-02	decrease
chr19	9768552	9768952	-0.949869204	1.34E-02	decrease
chr2	218671908	218672308	-0.83478729	1.35E-02	decrease
chr5	150449692	150450092	-0.745840818	1.35E-02	decrease
chr1	204363245	204363645	-0.967431233	1.35E-02	decrease
chr19	55461589	55461989	-0.819881988	1.35E-02	decrease
chr2	71276581	71276981	-1.02564	1.35E-02	decrease
chr5	37371196	37371596	-0.809398698	1.35E-02	decrease
chr22	41800315	41800715	-0.898741072	1.36E-02	decrease

chr11	118928249	118928649	-0.849407262	1.36E-02	decrease
chr6	26365142	26365542	-0.756306935	1.36E-02	decrease
chr5	65563009	65563409	-0.876322293	1.36E-02	decrease
chr4	163494562	163494962	-0.877193504	1.36E-02	decrease
chr14	31026416	31026816	-0.79758863	1.36E-02	decrease
chr12	110403622	110404022	-0.945744533	1.36E-02	decrease
chr1	229626374	229626774	-0.881701108	1.36E-02	decrease
chr12	119877074	119877474	-0.930318929	1.37E-02	decrease
chrX	72238501	72238901	-0.991371052	1.37E-02	decrease
chr12	53220414	53220814	-0.92251198	1.37E-02	decrease
chr21	36385018	36385418	-0.897284186	1.37E-02	decrease
chr17	7857249	7857649	-0.67401441	1.37E-02	decrease
chr10	125823225	125823625	-0.829859266	1.37E-02	decrease
chr19	48203806	48204206	-0.791532324	1.37E-02	decrease
chr3	58306987	58307387	-0.916705574	1.37E-02	decrease
chr2	46642779	46643179	-0.930885723	1.37E-02	decrease
chr3	5122636	5123036	-1.019973898	1.38E-02	decrease
chr11	111870871	111871271	-0.9243994	1.38E-02	decrease
chr6	37222355	37222755	-0.845418972	1.38E-02	decrease
chr4	109815404	109815804	-0.977490008	1.38E-02	decrease
chr2	240561148	240561548	-0.96324028	1.39E-02	decrease
chr22	41091437	41091837	-0.88826931	1.39E-02	decrease
chr1	52404321	52404721	-0.73447609	1.39E-02	decrease
chr10	97584128	97584528	-0.935323843	1.39E-02	decrease
chr6	85593977	85594377	-0.893512262	1.39E-02	decrease
chr19	49085099	49085499	-0.761137936	1.39E-02	decrease
chr5	111073097	111073497	-1.042896678	1.40E-02	decrease
chr12	56151710	56152110	-0.886891067	1.40E-02	decrease
chr16	89732613	89733013	-0.809487295	1.40E-02	decrease
chr2	197435014	197435414	-0.872888064	1.40E-02	decrease
chr7	73578690	73579090	-0.928516932	1.40E-02	decrease
chr1	99850001	99850401	-0.839448333	1.40E-02	decrease
chr1	226569758	226570158	-0.948747231	1.40E-02	decrease
chr19	3971231	3971631	-0.681393095	1.41E-02	decrease
chr5	141969009	141969409	-0.968133474	1.41E-02	decrease
chr4	4541928	4542328	-0.963879781	1.41E-02	decrease
chr8	124538808	124539208	-0.979472317	1.41E-02	decrease
chr3	52536366	52536766	-0.745179581	1.41E-02	decrease
chr12	57520260	57520660	-0.706285612	1.41E-02	decrease
chr4	76377675	76378075	-1.03717994	1.41E-02	decrease
chr2	118088080	118088480	-0.989616482	1.41E-02	decrease
chr3	180602076	180602476	-0.969696303	1.41E-02	decrease
chr1	61724933	61725333	-0.698637996	1.41E-02	decrease
chr14	91060734	91061134	-0.689625099	1.42E-02	decrease
chr8	56993980	56994380	-0.988300006	1.42E-02	decrease
chr19	11035727	11036127	-0.909146142	1.42E-02	decrease
chr9	34637817	34638217	-0.865587801	1.43E-02	decrease
chr12	104286654	104287054	-0.782560268	1.43E-02	decrease
chr1	148263312	148263712	-0.772808354	1.43E-02	decrease
chr13	37000379	37000779	-0.897614172	1.43E-02	decrease
chr1	52055393	52055793	-0.959932906	1.43E-02	decrease
chr20	6769449	6769849	-1.016882345	1.43E-02	decrease
chr1	44739546	44739946	-0.758547406	1.43E-02	decrease
chr6	27541633	27542033	-0.547870492	1.43E-02	decrease
chr6	43369710	43370110	-0.944285757	1.43E-02	decrease
chr11	57657404	57657804	-0.862815087	1.44E-02	decrease
chr22	22682479	22682879	1.036217046	1.44E-02	increase
chr14	24195632	24196032	-0.834556013	1.44E-02	decrease
chr7	100612089	100612489	-0.799166679	1.44E-02	decrease
chr7	73005658	73006058	-0.904159143	1.44E-02	decrease
chr10	68827013	68827413	-0.980039489	1.44E-02	decrease
chr17	67585141	67585541	-0.982687777	1.44E-02	decrease
chr1	109426407	109426807	-0.78745546	1.44E-02	decrease
chr5	172983628	172984028	-0.794777184	1.44E-02	decrease
chr16	30814139	30814539	-0.828268603	1.45E-02	decrease
chr13	50082444	50082844	-0.757281744	1.45E-02	decrease
chr10	101060610	101061010	-0.915526469	1.45E-02	decrease
chr2	130727430	130727830	-0.881241063	1.45E-02	decrease
chr21	32727359	32727759	-0.932936689	1.45E-02	decrease
chr1	149899511	149899911	-0.823869619	1.45E-02	decrease
chr1	21439764	21440164	-1.005707467	1.45E-02	decrease
chr12	67647904	67648304	-0.966973989	1.45E-02	decrease
chrX	41085773	41086173	-1.037257985	1.45E-02	decrease
chr18	35240694	35241094	-0.788161574	1.45E-02	decrease
chr9	92765399	92765799	-0.924043209	1.45E-02	decrease
chr10	124791844	124792244	-0.921335854	1.46E-02	decrease
chr3	89107548	89107948	-1.008681897	1.46E-02	decrease

chr5	140564425	140564825	-0.770713896	1.46E-02	decrease
chr14	88824452	88824852	-0.917609434	1.46E-02	decrease
chr16	72008591	72008991	-0.834150896	1.46E-02	decrease
chr19	9785766	9786166	-0.985545134	1.47E-02	decrease
chr7	43824799	43825199	-0.937532614	1.47E-02	decrease
chr2	54115566	54115966	-1.015010541	1.47E-02	decrease
chr5	142324851	142325251	-0.963246491	1.48E-02	decrease
chr13	40921364	40921764	-0.887189401	1.48E-02	decrease
chr5	90409300	90409700	-0.820964178	1.48E-02	decrease
chr15	89690485	89690885	-0.913064724	1.48E-02	decrease
chr18	69839026	69839426	-0.809187377	1.48E-02	decrease
chr14	50311895	50312295	-0.899096839	1.48E-02	decrease
chr1	109090500	109090900	-0.800551246	1.49E-02	decrease
chr22	50562858	50563258	-0.864441295	1.49E-02	decrease
chr14	103333891	103334291	-0.897402897	1.49E-02	decrease
chr6	13614979	13615379	-0.954060158	1.49E-02	decrease
chr12	29380680	29381080	-0.938527173	1.49E-02	decrease
chr6	154734058	154734458	-0.876382996	1.49E-02	decrease
chr4	184649788	184650188	-1.002334051	1.50E-02	decrease
chr2	169573491	169573891	-0.859697888	1.50E-02	decrease
chr1	31296923	31297323	-0.859311525	1.50E-02	decrease
chr2	26244578	26244978	-0.76244171	1.50E-02	decrease
chr2	178072738	178073138	-0.950282393	1.50E-02	decrease
chr2	231781236	231781636	-0.716708303	1.50E-02	decrease
chr6	149749949	149750349	-0.758564974	1.50E-02	decrease
chr1	145885846	145886246	-0.716000476	1.50E-02	decrease
chr2	113889546	113889946	-0.840940186	1.50E-02	decrease
chr15	90351852	90352252	-0.918573102	1.51E-02	decrease
chr19	703631	704031	-0.909304469	1.51E-02	decrease
chr6	28252093	28252493	-0.881261601	1.51E-02	decrease
chr17	56977703	56978103	-1.021069516	1.51E-02	decrease
chr12	93886486	93886886	-0.931183131	1.51E-02	decrease
chr12	76879463	76879863	-1.00362921	1.51E-02	decrease
chr14	21384160	21384560	-0.740943314	1.51E-02	decrease
chr15	89655040	89655440	-0.962250152	1.51E-02	decrease
chr1	222711754	222712154	-0.947616849	1.52E-02	decrease
chr4	151099231	151099631	-0.81260908	1.52E-02	decrease
chr1	234607319	234607719	-0.856253465	1.52E-02	decrease
chr14	66507851	66508251	-0.910368403	1.52E-02	decrease
chr8	96262675	96263075	-0.826321155	1.52E-02	decrease
chr6	28137078	28137478	-0.762312047	1.53E-02	decrease
chr11	47642620	47643020	-1.001207372	1.53E-02	decrease
chr1	148151978	148152378	-0.966238443	1.53E-02	decrease
chr17	7583785	7584185	-0.65434266	1.53E-02	decrease
chr13	87671069	87671469	-0.810208346	1.53E-02	decrease
chr10	42638202	42638602	-0.846270933	1.54E-02	decrease
chr11	119018426	119018826	-0.771385706	1.54E-02	decrease
chr19	44954646	44955046	-0.742314337	1.54E-02	decrease
chr15	92882172	92882572	-0.878278987	1.54E-02	decrease
chr19	2785086	2785486	-0.890951676	1.55E-02	decrease
chr19	49869394	49869794	-0.697030558	1.55E-02	decrease
chr3	131381542	131381942	-0.815827036	1.55E-02	decrease
chr13	65908817	65909217	-0.899866778	1.55E-02	decrease
chrX	54529723	54530123	-0.975646646	1.55E-02	decrease
chr20	31604365	31604765	-0.851588543	1.55E-02	decrease
chr17	57849757	57850546	-0.818535817	1.55E-02	decrease
chrX	54358339	54358739	-0.976500204	1.55E-02	decrease
chr12	50283542	50283942	-1.008921114	1.55E-02	decrease
chr13	48532646	48533046	-0.924384241	1.56E-02	decrease
chr12	49842721	49843121	-0.816605391	1.56E-02	decrease
chr7	129825523	129825923	-0.969225113	1.56E-02	decrease
chr7	149664562	149664962	-0.775590485	1.56E-02	decrease
chr8	56074494	56074894	-0.954402969	1.56E-02	decrease
chr16	67846466	67846866	-0.755508667	1.56E-02	decrease
chr16	2905089	2905489	-0.845407229	1.56E-02	decrease
chr3	185586008	185586408	-0.829567391	1.56E-02	decrease
chr5	88661194	88661594	-0.933913859	1.56E-02	decrease
chrX	119605069	119605469	-0.91840256	1.57E-02	decrease
chr13	28658889	28659289	-0.981649778	1.57E-02	decrease
chr6	122789705	122790105	-0.939024594	1.57E-02	decrease
chr11	61430021	61430421	-0.973009913	1.57E-02	decrease
chr3	51539270	51539670	-0.978700865	1.57E-02	decrease
chr2	88627798	88628198	-0.798039806	1.57E-02	decrease
chr11	119381312	119381712	-1.007155232	1.57E-02	decrease
chr12	120686497	120686897	-0.630321478	1.58E-02	decrease
chr1	150697092	150697492	-0.971127725	1.58E-02	decrease
chr3	152835012	152835412	-1.011209473	1.58E-02	decrease

chr2	70190683	70191083	-0.736773281	1.58E-02	decrease
chr6	79078375	79078775	-0.985872909	1.58E-02	decrease
chr17	77088627	77089027	-0.883499591	1.58E-02	decrease
chr12	54028331	54028731	-0.980198498	1.58E-02	decrease
chr7	91940735	91941135	-0.923498938	1.59E-02	decrease
chr2	120879804	120880204	-1.025968411	1.59E-02	decrease
chrX	53071594	53071994	-0.936923344	1.60E-02	decrease
chr1	27490272	27490672	-0.876788462	1.60E-02	decrease
chr19	30004165	30004565	-0.998848223	1.60E-02	decrease
chr5	173055946	173056346	-0.908566786	1.61E-02	decrease
chr11	65989102	65989502	-0.905971062	1.61E-02	decrease
chr10	131900263	131900663	-0.950924971	1.61E-02	decrease
chr5	150700676	150701076	-0.950145112	1.61E-02	decrease
chr13	23888845	23889245	-1.016190612	1.62E-02	decrease
chr15	64381164	64381564	-0.955230478	1.62E-02	decrease
chr22	24156909	24157309	-0.871148677	1.62E-02	decrease
chr12	56257476	56257876	-0.883941527	1.62E-02	decrease
chrX	53422480	53422880	-0.698124596	1.63E-02	decrease
chr20	35542056	35542456	-0.825702442	1.63E-02	decrease
chr8	129939826	129940226	-0.987590875	1.63E-02	decrease
chr8	113437848	113438248	-1.019500916	1.63E-02	decrease
chr3	50299350	50299750	-0.689347328	1.64E-02	decrease
chr19	17282738	17283138	-0.881312944	1.64E-02	decrease
chr5	179623856	179624256	-0.68797482	1.64E-02	decrease
chr5	140691217	140691617	-0.716901617	1.64E-02	decrease
chr19	57435257	57435657	-0.900434191	1.64E-02	decrease
chr13	30301308	30301708	-0.942450217	1.65E-02	decrease
chr4	26860339	26860739	-0.963731449	1.65E-02	decrease
chr20	5609955	5610355	-0.98643847	1.65E-02	decrease
chr1	222712518	222712918	-0.775432813	1.65E-02	decrease
chr17	61927551	61927951	-0.88527575	1.65E-02	decrease
chr10	13586940	13587340	-1.025457535	1.65E-02	decrease
chr9	124856059	124856459	-0.755204614	1.65E-02	decrease
chr22	30357134	30357534	-0.932847365	1.65E-02	decrease
chr10	100913015	100913415	-0.843671473	1.66E-02	decrease
chr18	32091863	32092263	-0.761349836	1.66E-02	decrease
chr3	194769098	194769498	-0.947228087	1.66E-02	decrease
chr7	105013110	105013510	-0.790418358	1.66E-02	decrease
chr7	73725806	73726206	-0.877706659	1.66E-02	decrease
chr17	8248308	8248708	-0.606894578	1.66E-02	decrease
chr7	101856876	101857276	-0.880614749	1.66E-02	decrease
chr8	85220341	85220741	-0.788670082	1.66E-02	decrease
chr3	49358336	49358736	-0.843470086	1.67E-02	decrease
chr1	58783962	58784362	-0.549665895	1.67E-02	decrease
chr11	75562308	75562708	-0.897310981	1.67E-02	decrease
chr12	95217539	95217939	-0.806778465	1.67E-02	decrease
chr11	64688470	64688870	-0.852025186	1.67E-02	decrease
chr21	25801430	25801830	-0.963091628	1.67E-02	decrease
chr15	59657468	59657868	-0.921199139	1.67E-02	decrease
chr19	53867411	53867811	-0.923303385	1.68E-02	decrease
chr19	48646151	48646551	-0.92296655	1.68E-02	decrease
chr17	16217108	16217508	-0.851398351	1.68E-02	decrease
chr20	407600	408000	-0.957059663	1.68E-02	decrease
chr1	154558871	154559271	-0.862510032	1.68E-02	decrease
chr6	98834227	98834627	-0.934990281	1.68E-02	decrease
chr1	23777974	23778374	-1.020706172	1.69E-02	decrease
chr3	193593067	193593467	-0.97603727	1.69E-02	decrease
chr12	49110586	49110986	-0.860304729	1.69E-02	decrease
chr19	814248	814648	-0.844601346	1.69E-02	decrease
chr6	132768058	132768458	-0.915464676	1.69E-02	decrease
chr6	169168104	169168504	-0.973126135	1.69E-02	decrease
chr16	46883885	46884285	-0.842791276	1.69E-02	decrease
chr17	21043295	21043695	-0.860118433	1.69E-02	decrease
chr1	145156826	145157226	-0.668296359	1.70E-02	decrease
chr3	150546636	150547036	-0.870566732	1.70E-02	decrease
chr3	47282445	47282845	-0.848902795	1.70E-02	decrease
chr5	73498028	73498428	-0.884753947	1.70E-02	decrease
chr2	169479494	169479894	-0.898466609	1.70E-02	decrease
chr22	41205080	41205480	-0.815331634	1.70E-02	decrease
chr6	57046500	57046900	-1.00213363	1.70E-02	decrease
chr1	26865003	26865403	-0.799216417	1.71E-02	decrease
chr5	150904937	150905337	-0.891856499	1.71E-02	decrease
chrX	48737351	48737751	-0.714952671	1.71E-02	decrease
chr16	3021208	3021608	-0.732418962	1.71E-02	decrease
chr6	26199314	26199714	-0.626859614	1.71E-02	decrease
chr1	117121827	117122227	-0.767866895	1.71E-02	decrease
chr19	15452172	15452572	-0.856801038	1.71E-02	decrease

chrX	12976791	12977191	-0.680735604	1.71E-02	decrease
chr15	75625668	75626068	-0.844422554	1.71E-02	decrease
chr7	66493561	66493961	-0.889419175	1.71E-02	decrease
chr20	37289497	37289897	-0.939212144	1.72E-02	decrease
chr5	172958900	172959300	-0.828481138	1.72E-02	decrease
chr17	47895846	47896246	-0.730481254	1.72E-02	decrease
chr4	139295459	139295859	-0.902724691	1.72E-02	decrease
chr10	87095450	87095850	-0.9401337	1.72E-02	decrease
chr17	81707477	81707877	-0.803970614	1.73E-02	decrease
chr13	85266885	85267285	-0.960472566	1.73E-02	decrease
chr20	3888915	3889315	-0.763755553	1.73E-02	decrease
chr17	82592946	82593346	-0.855697505	1.73E-02	decrease
chr15	42575189	42575589	-0.94041974	1.73E-02	decrease
chr2	241149711	241150111	-0.759422929	1.73E-02	decrease
chr1	116374809	116375209	-0.873808073	1.73E-02	decrease
chr6	109250937	109251337	-1.00058461	1.74E-02	decrease
chr6	26402191	26402591	-0.825639315	1.74E-02	decrease
chr12	6904686	6905086	-0.846917761	1.74E-02	decrease
chr13	52850522	52850922	-0.952065782	1.74E-02	decrease
chr1	221741963	221742363	-0.869227022	1.74E-02	decrease
chr10	125719239	125719639	-0.72716086	1.75E-02	decrease
chr7	112939845	112940245	-0.856831135	1.75E-02	decrease
chr2	64144402	64144802	-0.921652658	1.75E-02	decrease
chr6	132814215	132814615	-0.694163985	1.75E-02	decrease
chrX	102900392	102900792	-0.859216421	1.75E-02	decrease
chr14	80941397	80941797	-0.867437574	1.75E-02	decrease
chr19	45717354	45717754	-0.806401923	1.75E-02	decrease
chr12	57752209	57752609	-0.660006679	1.75E-02	decrease
chr16	85461965	85462365	-0.92796583	1.76E-02	decrease
chr16	58129347	58129747	-0.856688618	1.76E-02	decrease
chr4	120066849	120067249	-0.801227461	1.76E-02	decrease
chr17	7686147	7686547	-0.700239659	1.76E-02	decrease
chrX	52995588	52995988	-0.713149574	1.77E-02	decrease
chr13	79435599	79435999	-0.885775544	1.77E-02	decrease
chr15	78607825	78608225	-0.954560408	1.77E-02	decrease
chr6	110815542	110815942	-0.714124842	1.77E-02	decrease
chr7	44999792	45000192	-0.861706201	1.77E-02	decrease
chr19	1490423	1490823	-0.799311559	1.77E-02	decrease
chr1	110963771	110964171	-0.748614381	1.77E-02	decrease
chr10	68901016	68901416	-0.710107649	1.77E-02	decrease
chr5	112976322	112976722	-0.719197753	1.77E-02	decrease
chr19	4247070	4247470	-0.688136026	1.78E-02	decrease
chr1	226062679	226063079	-0.816167174	1.78E-02	decrease
chr19	45994918	45995318	-0.816959351	1.79E-02	decrease
chr1	66606684	66607084	-0.992527889	1.79E-02	decrease
chr1	145891825	145892225	-0.699744773	1.79E-02	decrease
chr12	57846984	57847384	-0.922456879	1.79E-02	decrease
chr1	151070899	151071299	-0.817169364	1.79E-02	decrease
chr18	62714901	62715301	-0.914580294	1.80E-02	decrease
chr9	33001196	33001596	-0.929956619	1.80E-02	decrease
chr16	24538961	24539361	-0.87248074	1.80E-02	decrease
chr12	132083758	132084158	-0.844171166	1.81E-02	decrease
chr12	100573913	100574313	-0.960098294	1.81E-02	decrease
chr19	37370689	37371089	-0.981634812	1.81E-02	decrease
chr16	2842718	2843118	-0.974311546	1.81E-02	decrease
chr14	37172108	37172508	-0.919660591	1.81E-02	decrease
chr5	96808210	96808610	-0.920364428	1.81E-02	decrease
chr4	17614323	17614723	-0.852449339	1.81E-02	decrease
chr10	102420776	102421176	-0.768627029	1.82E-02	decrease
chr18	57678599	57678999	-1.008880251	1.82E-02	decrease
chr9	124771303	124771703	-0.823847988	1.82E-02	decrease
chr22	31490460	31490860	-0.906173081	1.82E-02	decrease
chr2	111897976	111898376	-0.870211851	1.82E-02	decrease
chr16	28936405	28936805	-0.755814248	1.83E-02	decrease
chr1	171781174	171781574	-0.786399271	1.83E-02	decrease
chr11	62665056	62665456	-0.762469184	1.83E-02	decrease
chr19	33184358	33184758	-0.86049636	1.83E-02	decrease
chr10	71851061	71851461	-0.76167855	1.83E-02	decrease
chr2	128091014	128091414	-0.924001334	1.84E-02	decrease
chr15	40807430	40807830	-0.751137805	1.84E-02	decrease
chrX	74343591	74343991	-0.97107584	1.84E-02	decrease
chr10	964717	965117	-0.99888964	1.84E-02	decrease
chr1	16216223	16216623	-0.864542258	1.84E-02	decrease
chr15	96171083	96171483	-1.000870782	1.84E-02	decrease
chr11	119067427	119067827	-0.84684129	1.84E-02	decrease
chr14	31207969	31208369	-0.954623934	1.84E-02	decrease
chr15	65385023	65385423	-0.722122767	1.85E-02	decrease

chr4	75514303	75514703	-0.846307945	1.85E-02	decrease
chr1	227563553	227563953	-0.728679544	1.85E-02	decrease
chr19	50476422	50476822	-0.817382146	1.85E-02	decrease
chr3	47522253	47522653	-0.927247563	1.86E-02	decrease
chr8	94474863	94475263	-0.741732231	1.86E-02	decrease
chr1	231241142	231241542	-0.704831777	1.86E-02	decrease
chr5	32312962	32313362	-1.00686634	1.86E-02	decrease
chr5	115841514	115841914	-0.953274131	1.86E-02	decrease
chr19	45326109	45326509	-0.854740554	1.86E-02	decrease
chr5	141636834	141637234	-0.916352436	1.86E-02	decrease
chr17	74213075	74213475	-0.809971642	1.87E-02	decrease
chr9	21802148	21802548	-1.002741064	1.87E-02	decrease
chr7	64666071	64666471	-0.990740967	1.87E-02	decrease
chr8	140464344	140464744	-0.835902322	1.87E-02	decrease
chr6	78867411	78867811	-0.876373858	1.87E-02	decrease
chr7	65373766	65374166	-0.884033679	1.87E-02	decrease
chr15	72118262	72118662	-0.745666659	1.87E-02	decrease
chr2	73111392	73111792	-0.936858872	1.88E-02	decrease
chr5	132866360	132866760	-0.751787602	1.88E-02	decrease
chr17	3048903	3049303	-0.97051167	1.88E-02	decrease
chr2	17752987	17753387	-1.002174273	1.88E-02	decrease
chr2	150486046	150486446	-0.916449551	1.88E-02	decrease
chr14	23469314	23469714	-0.882654313	1.89E-02	decrease
chr13	78660202	78660602	-0.880024048	1.89E-02	decrease
chr1	1505039	1505439	-0.907604251	1.90E-02	decrease
chr1	244836024	244836424	-0.757019018	1.90E-02	decrease
chr6	142147202	142147602	-0.881075946	1.90E-02	decrease
chr22	38656413	38656813	-0.755580606	1.90E-02	decrease
chr4	77156562	77156962	-0.8318443	1.91E-02	decrease
chr11	106022135	106022535	-0.83253806	1.91E-02	decrease
chr14	20631328	20631728	-0.450845043	1.91E-02	decrease
chr17	78378202	78378602	-0.975630684	1.91E-02	decrease
chr17	49788795	49789195	-0.754558851	1.91E-02	decrease
chr9	124419431	124419831	-0.950240856	1.91E-02	decrease
chr8	10839490	10839890	-0.999585055	1.91E-02	decrease
chr12	51172566	51172966	-0.86035123	1.91E-02	decrease
chr3	130893488	130893888	-0.953342656	1.92E-02	decrease
chr1	55021930	55022330	-0.848954943	1.92E-02	decrease
chr17	60599774	60600174	-0.85984739	1.92E-02	decrease
chr12	117190485	117190885	-0.999415187	1.93E-02	decrease
chr2	27428192	27428592	-0.887032191	1.94E-02	decrease
chr1	171485453	171485853	-0.813650152	1.94E-02	decrease
chr15	74374124	74374524	-0.791436902	1.94E-02	decrease
chr12	8081955	8082355	-0.899797631	1.94E-02	decrease
chrX	101051589	101051989	-0.834420542	1.94E-02	decrease
chr4	141133726	141134126	-0.998675542	1.94E-02	decrease
chr4	146521590	146521990	-0.912867053	1.94E-02	decrease
chr11	76445153	76445553	-0.887664982	1.94E-02	decrease
chr13	36919002	36919402	-0.949263325	1.95E-02	decrease
chrX	53093309	53093709	-0.846600577	1.95E-02	decrease
chr19	4851721	4852121	-0.911401347	1.95E-02	decrease
chrX	75273988	75274388	-0.868351134	1.95E-02	decrease
chr1	182838893	182839293	-0.836548801	1.96E-02	decrease
chr9	125241444	125241844	-0.678262949	1.96E-02	decrease
chr14	49620393	49620793	-0.770986736	1.96E-02	decrease
chr12	123050407	123050807	-0.958693576	1.97E-02	decrease
chr15	78873592	78873992	-0.751088599	1.97E-02	decrease
chr1	25338247	25338647	-0.93677833	1.97E-02	decrease
chr19	18277609	18278009	-0.870324558	1.97E-02	decrease
chr3	133805637	133806037	-0.851611233	1.97E-02	decrease
chr19	49495998	49496398	-0.594874509	1.98E-02	decrease
chr12	89524505	89524905	-0.954891987	1.98E-02	decrease
chr22	41946766	41947166	-0.697060252	1.98E-02	decrease
chr5	96935434	96935834	-0.784125591	1.98E-02	decrease
chr1	109984234	109984634	-0.936532962	1.98E-02	decrease
chr1	169794688	169795088	-0.794204983	1.99E-02	decrease
chr11	96389569	96389969	-0.794464414	1.99E-02	decrease
chr16	86555200	86555600	-0.984801404	1.99E-02	decrease
chr1	52703022	52703422	-0.840540002	1.99E-02	decrease
chr10	96586231	96586631	-0.959337676	1.99E-02	decrease
chr11	106077008	106077408	-0.770038392	1.99E-02	decrease
chr1	209747661	209748061	-0.790078826	1.99E-02	decrease
chr17	2054461	2054861	-0.96569881	1.99E-02	decrease
chr15	70701962	70702362	-0.89272531	2.00E-02	decrease
chr3	33114554	33114954	-0.910409426	2.00E-02	decrease
chr3	114056375	114056775	-0.820458896	2.00E-02	decrease
chr14_GL000225v1_random	51740	52140	0.993024089	2.00E-02	increase

chr2	183078909	183079309	-0.93544657	2.01E-02	decrease
chr8	73089925	73090325	-0.908928986	2.01E-02	decrease
chr1	54887397	54887797	-0.798999131	2.01E-02	decrease
chr6	27020071	27020471	-0.479216555	2.01E-02	decrease
chr8	30082284	30082684	-0.941311567	2.01E-02	decrease
chr8	42338274	42338674	-0.991038647	2.03E-02	decrease
chr13	37047008	37047408	-0.87627438	2.03E-02	decrease
chr2	112054237	112054637	-0.765132063	2.03E-02	decrease
chr19	50333572	50333972	-0.738887181	2.03E-02	decrease
chrX	57121237	57121637	-0.992123879	2.03E-02	decrease
chr16	2501259	2501659	-0.749941719	2.03E-02	decrease
chr1	84620721	84621121	-0.901967276	2.04E-02	decrease
chr1	149812208	149812608	-0.807367494	2.04E-02	decrease
chrX	47561077	47561477	-0.828485042	2.04E-02	decrease
chr21	29302268	29302668	-0.936051496	2.04E-02	decrease
chr16	85615986	85616386	-0.845980489	2.05E-02	decrease
chr2	3379688	3380088	-0.837098073	2.05E-02	decrease
chr6	108559022	108559422	-0.941381081	2.05E-02	decrease
chr1	153647152	153647552	-0.762803667	2.06E-02	decrease
chr2	201071635	201072035	-0.797946937	2.06E-02	decrease
chr12	120495996	120496396	-0.847662071	2.06E-02	decrease
chr5	176388646	176389046	-0.600498363	2.06E-02	decrease
chr4	185925902	185926302	-0.970488619	2.07E-02	decrease
chr4	174283242	174283642	-0.891724213	2.07E-02	decrease
chr19	40702769	40703169	-0.958670421	2.08E-02	decrease
chr4	41935101	41935501	-0.880392527	2.08E-02	decrease
chr4	683788	684188	-0.756478665	2.08E-02	decrease
chr1	175023364	175023764	-0.815541507	2.08E-02	decrease
chr12	53421378	53421778	-0.823715505	2.09E-02	decrease
chr19	41872922	41873322	-0.850465867	2.09E-02	decrease
chr22	38506336	38506736	-0.620791785	2.09E-02	decrease
chr5	164327219	164327619	-0.895871014	2.09E-02	decrease
chr3	47802416	47802816	-0.923021055	2.10E-02	decrease
chr4	139082828	139083228	-0.784044911	2.10E-02	decrease
chr2	112584515	112584915	-0.692876014	2.10E-02	decrease
chr2	238060469	238060869	-0.984498568	2.10E-02	decrease
chr16	29961651	29962051	-0.787434201	2.10E-02	decrease
chr19	17332583	17332983	-0.685281713	2.10E-02	decrease
chr14	60649577	60649977	-0.882524379	2.11E-02	decrease
chr5	78294525	78294925	-0.793693643	2.11E-02	decrease
chr14	23321011	23321411	-0.705309257	2.11E-02	decrease
chr19	45886401	45886801	-0.720839746	2.11E-02	decrease
chr2	180540674	180541074	-0.973662624	2.11E-02	decrease
chr15	90994497	90994897	-0.781133002	2.11E-02	decrease
chr11	65354281	65354681	-0.862535581	2.12E-02	decrease
chr3	50350736	50351136	-0.722988499	2.12E-02	decrease
chr1	63773624	63774024	-0.858470329	2.12E-02	decrease
chr1	225809696	225810096	-0.77397059	2.12E-02	decrease
chr6	2999597	2999997	-0.875341048	2.12E-02	decrease
chr12	118035028	118035428	-0.817974402	2.13E-02	decrease
chr15	69452533	69452933	-0.712451734	2.13E-02	decrease
chr6	110876054	110876454	-0.759039082	2.13E-02	decrease
chr11	74492899	74493299	-0.894529549	2.13E-02	decrease
chr1	113929189	113929589	-0.828882445	2.14E-02	decrease
chr14	65412018	65412418	-0.861450712	2.14E-02	decrease
chr15	82262384	82262784	-0.881612561	2.14E-02	decrease
chr6	28489187	28489587	-0.62836072	2.14E-02	decrease
chr6	109095162	109095562	-0.82277112	2.14E-02	decrease
chr14	58200158	58200558	-0.958977609	2.14E-02	decrease
chr2	85418316	85418716	-0.805908122	2.14E-02	decrease
chr5	138331692	138332092	-0.708363372	2.14E-02	decrease
chr17	35567992	35568392	-0.803559646	2.14E-02	decrease
chr17	39199974	39200374	-0.721037437	2.15E-02	decrease
chr18	35497603	35498003	-0.816000029	2.15E-02	decrease
chr17	48107397	48107797	-0.815499107	2.15E-02	decrease
chr1	6205167	6205567	-0.821878534	2.15E-02	decrease
chr12	118135533	118135933	-0.930312791	2.16E-02	decrease
chr3	53130426	53130826	-0.912528626	2.16E-02	decrease
chr7	13920192	13920592	-0.90857049	2.16E-02	decrease
chr8	33513141	33513541	-0.837056047	2.16E-02	decrease
chr2	197499842	197500242	-0.735536613	2.16E-02	decrease
chr22	30667609	30668009	-0.79495534	2.16E-02	decrease
chr11	62727513	62727913	-0.706401075	2.17E-02	decrease
chr12	949432	949832	-0.812834015	2.17E-02	decrease
chr1	6962739	6963139	-0.856558797	2.17E-02	decrease
chr6	26299578	26299978	-0.543558611	2.17E-02	decrease
chrX	119574152	119574552	-0.853091649	2.17E-02	decrease

chrX	43654546	43654946	-0.86591205	2.18E-02	decrease
chr12	2890525	2890925	-0.755701049	2.18E-02	decrease
chr1	45521985	45522385	-0.774666576	2.18E-02	decrease
chr6	43506578	43506978	-0.804122204	2.19E-02	decrease
chr8	66428724	66429124	-0.801604671	2.20E-02	decrease
chr2	74653942	74654342	-0.797925438	2.20E-02	decrease
chr16	30558380	30558780	-0.734076896	2.20E-02	decrease
chr7	33734055	33734455	-0.927129477	2.20E-02	decrease
chr16	58000980	58001380	-0.799079829	2.20E-02	decrease
chr12	6613986	6614386	-0.810312189	2.21E-02	decrease
chr6	44387515	44387915	-0.793248366	2.21E-02	decrease
chr4	110623094	110623494	-0.883266832	2.21E-02	decrease
chr6	37018134	37018534	-0.863557812	2.22E-02	decrease
chr21	36319864	36320264	-0.785455128	2.22E-02	decrease
chr19	17309264	17309664	-0.672529803	2.22E-02	decrease
chr4	51842819	51843219	-0.860001712	2.22E-02	decrease
chr10	96513859	96514259	-0.888471069	2.22E-02	decrease
chr16	30805473	30805873	-0.821422918	2.22E-02	decrease
chr7	73842261	73842661	-0.937041767	2.22E-02	decrease
chr16	3129365	3129765	-0.804145253	2.22E-02	decrease
chr5	88516296	88516696	-0.838541385	2.23E-02	decrease
chr12	64608930	64609330	-0.875010982	2.23E-02	decrease
chr7	88218917	88219317	-0.959087655	2.23E-02	decrease
chr11	119084801	119085201	-0.690899775	2.23E-02	decrease
chr6	131628001	131628401	-0.756901908	2.23E-02	decrease
chr17	30477328	30477728	-0.85778558	2.23E-02	decrease
chr2	189762511	189762911	-0.830604273	2.24E-02	decrease
chr1	155934364	155934764	-0.77993622	2.24E-02	decrease
chr8	119832519	119832919	-0.823491615	2.25E-02	decrease
chr20	25696647	25697047	-0.854827826	2.25E-02	decrease
chrX	130165692	130166092	-0.902465533	2.25E-02	decrease
chr4	56544696	56545096	-0.932730472	2.25E-02	decrease
chr3	39051608	39052008	-0.944609029	2.26E-02	decrease
chr10	116609415	116609815	-0.954854472	2.26E-02	decrease
chr22	18001444	18001844	-0.915519271	2.26E-02	decrease
chrX	101390799	101391199	-0.73379472	2.26E-02	decrease
chr17	58219121	58219521	-0.857538151	2.26E-02	decrease
chr2	231514213	231514613	-0.815237771	2.26E-02	decrease
chr6	156780202	156780602	-0.926870118	2.26E-02	decrease
chr19	10119825	10120225	-0.652788252	2.27E-02	decrease
chr3	108589465	108589865	-0.829348988	2.27E-02	decrease
chr13	20902051	20902451	-0.923860448	2.27E-02	decrease
chr7	100049691	100050091	-0.879217607	2.28E-02	decrease
chr4	119212710	119213110	-0.727478802	2.28E-02	decrease
chr1	182839682	182840082	-0.807903359	2.28E-02	decrease
chr5	62403813	62404213	-0.702758316	2.28E-02	decrease
chr5	139765622	139766022	-0.814327618	2.28E-02	decrease
chr4	2321657	2322057	0.955924471	2.28E-02	increase
chr22	40346356	40346756	-0.731715434	2.28E-02	decrease
chr20	37095774	37096174	-0.823477209	2.28E-02	decrease
chr11	3807692	3808092	-0.804816408	2.29E-02	decrease
chr10	43455794	43456194	-0.966447293	2.29E-02	decrease
chr17	7887807	7888207	-0.751472203	2.29E-02	decrease
chr12	74537598	74537998	-0.88190286	2.29E-02	decrease
chr11	18394466	18394866	-0.820316588	2.30E-02	decrease
chr17	21286125	21286525	-0.904895355	2.30E-02	decrease
chr11	77994704	77995104	-0.817756445	2.30E-02	decrease
chr17	59565546	59565946	-0.877031853	2.30E-02	decrease
chr10	101042737	101043137	-0.863977564	2.30E-02	decrease
chr17	81513559	81513959	-0.704957172	2.30E-02	decrease
chr1	228867011	228867411	-0.781761352	2.31E-02	decrease
chr4	139015350	139015750	-0.748013989	2.31E-02	decrease
chr19	50804639	50805039	-0.740208339	2.31E-02	decrease
chr6	137219081	137219481	-0.801724923	2.31E-02	decrease
chr5	176537549	176537949	-0.712477414	2.31E-02	decrease
chr8	77000261	77000661	-0.884445974	2.32E-02	decrease
chrX	30652874	30653274	-0.910462192	2.32E-02	decrease
chr11	118566053	118566453	-0.852702441	2.32E-02	decrease
chr15	65377033	65377433	-0.908337489	2.32E-02	decrease
chr11	67805550	67805950	-0.647443976	2.32E-02	decrease
chr12	6662970	6663370	-0.805576392	2.32E-02	decrease
chr1	11736028	11736428	-0.920827825	2.32E-02	decrease
chr15	101277798	101278198	-0.797797756	2.33E-02	decrease
chr20	3767955	3768355	-0.734116647	2.33E-02	decrease
chr20	37178664	37179064	-0.715035673	2.33E-02	decrease
chr14	20333025	20333425	-0.695622035	2.33E-02	decrease
chr17	60079258	60079658	-0.959487338	2.33E-02	decrease

chr9	121201833	121202233	-0.825640843	2.33E-02	decrease
chr13	48037751	48038151	-0.841821699	2.33E-02	decrease
chr6	11232373	11232773	-0.802706264	2.34E-02	decrease
chr2	152717862	152718262	-0.671732163	2.34E-02	decrease
chr1	156026113	156026513	-0.841638128	2.34E-02	decrease
chr19	47273710	47274110	-0.63113456	2.34E-02	decrease
chr2	73071459	73071859	-0.741444374	2.34E-02	decrease
chr5	80407765	80408165	-0.732122891	2.34E-02	decrease
chr1	84506291	84506691	-0.772695262	2.35E-02	decrease
chr19	42387139	42387539	-0.798646015	2.35E-02	decrease
chr1	45686709	45687109	-0.717312735	2.35E-02	decrease
chr7	92833677	92834077	-0.857829631	2.35E-02	decrease
chr20	11369058	11369458	0.967504705	2.35E-02	increase
chr2	69437273	69437673	-0.738464802	2.35E-02	decrease
chr10	97318890	97319290	-0.835697922	2.35E-02	decrease
chr12	51026116	51026516	-0.816703159	2.35E-02	decrease
chr5	177351609	177352009	-0.685402745	2.36E-02	decrease
chr11	75350697	75351097	-0.857047478	2.36E-02	decrease
chr15	90931812	90932212	-0.760165938	2.36E-02	decrease
chr15	84630981	84631381	-0.854745169	2.36E-02	decrease
chr5	112707426	112707826	-0.790806301	2.37E-02	decrease
chr1	193122004	193122404	-0.874499709	2.37E-02	decrease
chr10	101012988	101013388	-0.812880672	2.37E-02	decrease
chr13	102845760	102846160	-0.94093516	2.38E-02	decrease
chr22	41197535	41197935	-0.86467359	2.38E-02	decrease
chr6	135497471	135497871	-0.946063828	2.38E-02	decrease
chr19	13825709	13826109	-0.883014121	2.38E-02	decrease
chr14	88551542	88551942	-0.933131445	2.38E-02	decrease
chr16	27549923	27550323	-0.834961486	2.38E-02	decrease
chr16	30430090	30430490	-0.679684667	2.39E-02	decrease
chr8	26382937	26383337	-0.750035827	2.40E-02	decrease
chr1	201955243	201955643	-0.641580757	2.40E-02	decrease
chr2	68252709	68253109	-0.951452454	2.40E-02	decrease
chr6	122471468	122471868	-0.864027772	2.40E-02	decrease
chr11	85685959	85686359	-0.753356988	2.40E-02	decrease
chr2	177265013	177265413	-0.842738766	2.41E-02	decrease
chr6	41895357	41895757	-0.920583438	2.41E-02	decrease
chr16	70289234	70289634	-0.811846767	2.41E-02	decrease
chr11	113896170	113896570	-0.915967691	2.41E-02	decrease
chrX	70454291	70454691	-0.84177405	2.41E-02	decrease
chr6	27209735	27210135	-0.456736586	2.41E-02	decrease
chr1	90851311	90851711	-0.94206634	2.41E-02	decrease
chr8	140810588	140810988	0.950367336	2.42E-02	increase
chrX	130902920	130903320	-0.674471023	2.42E-02	decrease
chr1	169427041	169427441	-0.932367918	2.43E-02	decrease
chr2	32039621	32040021	-0.890353622	2.43E-02	decrease
chr13	75482258	75482658	-0.80683379	2.43E-02	decrease
chrX	129931704	129932104	-0.859939831	2.43E-02	decrease
chr13	50529119	50529519	-0.925361634	2.43E-02	decrease
chr15	41603887	41604287	-0.937463006	2.44E-02	decrease
chr1	35557414	35557814	-0.695411805	2.44E-02	decrease
chr17	17281026	17281426	-0.810981139	2.44E-02	decrease
chr19	35869845	35870245	-0.788120665	2.44E-02	decrease
chr17	13018171	13018571	-0.918086745	2.45E-02	decrease
chr3	49429444	49429844	-0.793781204	2.45E-02	decrease
chr12	120195156	120195556	-0.70029652	2.45E-02	decrease
chr2	42494806	42495206	-0.893336131	2.45E-02	decrease
chr20	44210987	44211387	-0.911515503	2.46E-02	decrease
chr1	15849713	15850113	-0.850067253	2.46E-02	decrease
chr9	101486211	101486611	-0.894086093	2.46E-02	decrease
chr22	46013559	46013959	-0.828874466	2.47E-02	decrease
chr10	58268833	58269233	-0.738895457	2.47E-02	decrease
chr1	205631091	205631491	-0.885937785	2.47E-02	decrease
chr7	66628374	66628774	-0.893507852	2.48E-02	decrease
chr1	148529153	148529553	-0.564816145	2.48E-02	decrease
chr2	159286875	159287275	-0.888085566	2.48E-02	decrease
chr1	146938351	146938751	-0.939033163	2.48E-02	decrease
chr10	97633521	97633921	-0.704275031	2.48E-02	decrease
chr19	45641206	45641606	-0.68449945	2.50E-02	decrease
chr6	42563851	42564251	-0.785609802	2.51E-02	decrease
chr8	144103971	144104371	-0.742824882	2.51E-02	decrease
chr6	34163642	34164042	-0.956193562	2.51E-02	decrease
chr2	53587053	53587453	-0.931640754	2.52E-02	decrease
chr1	150487140	150487540	-0.742316711	2.52E-02	decrease
chr17	44324875	44325275	-0.829050514	2.52E-02	decrease
chr19	14450434	14450834	-0.791385248	2.52E-02	decrease
chr7	112206713	112207113	-0.877920344	2.52E-02	decrease

chr16	31094631	31095031	-0.80935728	2.53E-02	decrease
chr16	68328460	68328860	-0.816575145	2.54E-02	decrease
chr17	47831210	47831610	-0.85031723	2.54E-02	decrease
chr17	76072494	76072894	-0.843859144	2.55E-02	decrease
chr2	61764126	61764526	-0.653707337	2.55E-02	decrease
chr22	29267926	29268326	-0.645774196	2.55E-02	decrease
chr2	183124578	183124978	-0.776616687	2.56E-02	decrease
chr12	95859004	95859404	-0.926554336	2.56E-02	decrease
chr4	53365602	53366002	-0.87344422	2.57E-02	decrease
chr7	100148584	100148984	-0.74476376	2.57E-02	decrease
chr20	62182620	62183020	-0.908584717	2.57E-02	decrease
chr12	49018385	49018785	-0.649481798	2.57E-02	decrease
chr9	37422391	37422791	-0.710720981	2.57E-02	decrease
chr15	75367906	75368306	-0.593635222	2.57E-02	decrease
chr6	99614446	99614846	-0.929056892	2.57E-02	decrease
chr16	11797369	11797769	-0.844147313	2.57E-02	decrease
chr13	71864607	71865007	-0.816841539	2.57E-02	decrease
chr15	75640235	75640635	-0.656638056	2.57E-02	decrease
chr19	10695576	10695976	-0.652664294	2.58E-02	decrease
chr1	161291415	161291815	-0.880859979	2.58E-02	decrease
chr3	48663795	48664195	-0.585882082	2.58E-02	decrease
chr6	6687098	6687498	-0.847691081	2.58E-02	decrease
chr2	108533876	108534276	-0.782834781	2.59E-02	decrease
chr5	97183313	97183713	-0.770460636	2.59E-02	decrease
chr17	7484443	7484843	-0.717470858	2.59E-02	decrease
chr9	15422639	15423039	-0.832993207	2.59E-02	decrease
chr6	34415772	34416172	-0.837234606	2.60E-02	decrease
chr12	6970433	6970833	-0.723271169	2.60E-02	decrease
chr18	70205684	70206084	-0.940194287	2.60E-02	decrease
chr3	48609430	48609830	-0.772579996	2.60E-02	decrease
chr7	36389934	36390334	-0.83746716	2.60E-02	decrease
chr12	32678977	32679377	-0.788132049	2.60E-02	decrease
chr19	16185275	16185675	-0.874791315	2.60E-02	decrease
chr9	35079737	35080137	-0.720295485	2.61E-02	decrease
chr4	153467346	153467746	-0.936484084	2.61E-02	decrease
chr1	200620849	200621249	-0.898154662	2.61E-02	decrease
chr17	42609156	42609556	-0.833572033	2.61E-02	decrease
chr1	16367279	16367679	-0.832265946	2.61E-02	decrease
chr3	13451822	13452222	-0.829158791	2.61E-02	decrease
chr1	156456854	156457254	-0.642020375	2.62E-02	decrease
chr1	52033215	52033615	-0.844754088	2.62E-02	decrease
chr19	12551468	12551868	-0.724134726	2.62E-02	decrease
chr17	17591691	17592091	-0.68204592	2.62E-02	decrease
chr11	6682964	6683364	-0.911095155	2.62E-02	decrease
chr19	45645741	45646141	-0.776042877	2.63E-02	decrease
chr16	31179790	31180190	-0.671790775	2.63E-02	decrease
chr15	90973174	90973574	-0.865917362	2.63E-02	decrease
chr1	78004557	78004957	-0.736369445	2.63E-02	decrease
chr17	75368222	75368622	-0.810117012	2.63E-02	decrease
chr13	80338910	80339310	-0.840461234	2.64E-02	decrease
chr3	136250294	136250694	-0.694735887	2.64E-02	decrease
chr17	74970499	74970899	-0.803739592	2.64E-02	decrease
chrX	103629525	103629925	-0.78937995	2.64E-02	decrease
chr9	127388268	127388668	-0.778409155	2.64E-02	decrease
chr8	37452360	37452760	-0.91481444	2.64E-02	decrease
chr12	124988883	124989283	-0.727361314	2.65E-02	decrease
chr19	44914987	44915387	-0.765783324	2.65E-02	decrease
chr5	179732348	179732748	-0.770746448	2.65E-02	decrease
chr12	57590907	57591307	-0.821425575	2.65E-02	decrease
chr4	185396592	185396992	-0.948258218	2.66E-02	decrease
chr1	100351578	100351978	-0.612621031	2.66E-02	decrease
chr5	154754656	154755056	-0.834682041	2.66E-02	decrease
chr11	95093110	95093510	-0.90495892	2.66E-02	decrease
chr18	21111858	21112258	-0.78698881	2.67E-02	decrease
chr2	229922319	229922719	-0.821057685	2.67E-02	decrease
chr19	48584070	48584470	-0.944442413	2.68E-02	decrease
chr1	28581748	28582148	-0.689896392	2.68E-02	decrease
chr7	17940586	17940986	-0.901315857	2.68E-02	decrease
chr16	67561910	67562310	-0.800845608	2.68E-02	decrease
chrX	123959528	123959928	-0.801211883	2.68E-02	decrease
chr9	137454140	137454540	-0.874849033	2.69E-02	decrease
chr16	30021341	30021741	-0.671799353	2.69E-02	decrease
chr5	157142597	157142997	-0.907497164	2.69E-02	decrease
chr3	9749850	9750250	-0.610915836	2.69E-02	decrease
chr7	44885568	44885968	-0.841495937	2.69E-02	decrease
chr17	42154896	42155296	-0.674919185	2.70E-02	decrease
chr2	70301459	70301859	-0.799371602	2.70E-02	decrease

chr12	113184687	113185087	-0.870498753	2.70E-02	decrease
chr1	112955697	112956097	-0.746325767	2.71E-02	decrease
chr2	42141429	42141829	-0.927343709	2.71E-02	decrease
chr1	29182003	29182403	-0.826469681	2.71E-02	decrease
chr3	38164964	38165364	-0.659252694	2.71E-02	decrease
chr3	40452908	40453308	-0.80726291	2.71E-02	decrease
chr19	41310028	41310428	-0.755087083	2.71E-02	decrease
chr16	30953728	30954128	-0.635594081	2.71E-02	decrease
chr12	120469844	120470244	-0.726695887	2.71E-02	decrease
chr5	98929241	98929641	-0.734448288	2.72E-02	decrease
chr13	19960101	19960501	-0.917583408	2.72E-02	decrease
chr17	59093400	59093800	-0.818378508	2.72E-02	decrease
chr3	107429602	107430002	-0.922220058	2.73E-02	decrease
chr16	8868684	8869084	-0.867933305	2.73E-02	decrease
chr20	44966446	44966846	-0.848403519	2.73E-02	decrease
chr13	85142445	85142845	-0.908292031	2.73E-02	decrease
chr10	12042883	12043283	-0.739976964	2.73E-02	decrease
chrX	16719411	16719811	-0.834248739	2.73E-02	decrease
chr9	2014774	2015174	-0.858666453	2.73E-02	decrease
chr22	32204843	32205243	-0.812024013	2.73E-02	decrease
chr21	26843035	26843435	-0.71702783	2.73E-02	decrease
chr10	80205609	80206009	-0.876243299	2.73E-02	decrease
chr1	28648687	28649087	-0.614104168	2.74E-02	decrease
chr1	36187993	36188393	-0.800035347	2.74E-02	decrease
chr6	27388583	27388983	-0.637933043	2.74E-02	decrease
chr9	127724860	127725260	-0.856708614	2.74E-02	decrease
chr6	43702939	43703339	-0.805812475	2.74E-02	decrease
chr19	35717156	35717556	-0.769113586	2.75E-02	decrease
chr7	99616615	99617015	-0.766089961	2.75E-02	decrease
chr3	128617814	128618214	-0.736307698	2.76E-02	decrease
chr11	62671960	62672360	-0.752296128	2.76E-02	decrease
chr2	45009714	45010114	-0.85187313	2.76E-02	decrease
chr1	99969555	99969955	-0.865163469	2.76E-02	decrease
chr2	68157681	68158081	-0.773787251	2.78E-02	decrease
chr1	235367313	235367713	-0.571104369	2.78E-02	decrease
chr19	55339386	55339786	-0.582350786	2.79E-02	decrease
chr10	12068547	12068947	-0.816975181	2.80E-02	decrease
chr16	22205820	22206220	-0.838583935	2.80E-02	decrease
chr1	59296879	59297279	-0.814890524	2.80E-02	decrease
chr10	100342431	100342831	-0.805470715	2.81E-02	decrease
chr11	108467277	108467677	-0.781589719	2.81E-02	decrease
chr22	24555319	24555719	-0.692683709	2.81E-02	decrease
chr12	71754505	71754905	-0.86014805	2.81E-02	decrease
chr1	42658027	42658427	-0.838691021	2.81E-02	decrease
chr2	68062998	68063398	-0.901772743	2.81E-02	decrease
chr1	1746321	1746721	-0.938649685	2.82E-02	decrease
chr5	108728078	108728478	-0.857346148	2.82E-02	decrease
chr10	88346455	88346855	-0.90182078	2.82E-02	decrease
chr16	25111601	25112001	-0.785987521	2.82E-02	decrease
chr1	16904846	16905246	-0.579733297	2.83E-02	decrease
chr15	51622012	51622412	-0.885563379	2.83E-02	decrease
chr9	130824998	130825398	-0.913861419	2.83E-02	decrease
chr20	31738808	31739208	-0.72588299	2.83E-02	decrease
chr17	81867264	81867664	-0.648791275	2.84E-02	decrease
chr1	111755556	111755956	-0.811592224	2.84E-02	decrease
chr14	65102558	65102958	-0.877611449	2.84E-02	decrease
chr11	47161242	47161642	-0.781042651	2.85E-02	decrease
chr17	7014418	7014818	-0.787306781	2.85E-02	decrease
chr9	114587449	114587849	-0.714990339	2.85E-02	decrease
chr16	66650828	66651228	-0.864567724	2.85E-02	decrease
chr7	135716557	135716957	-0.819166041	2.85E-02	decrease
chr3	33659094	33659494	-0.799756918	2.85E-02	decrease
chr10	50622207	50622607	-0.897217395	2.86E-02	decrease
chr17	39056847	39057247	-0.843506373	2.86E-02	decrease
chr8	54022405	54022805	-0.887017891	2.86E-02	decrease
chr4	82796676	82797076	-0.837162597	2.88E-02	decrease
chr12	72271681	72272081	-0.896652902	2.88E-02	decrease
chr11	10808928	10809328	-0.676484732	2.88E-02	decrease
chr17	17300989	17301389	-0.752685052	2.88E-02	decrease
chr17	35764469	35764869	-0.927299743	2.88E-02	decrease
chr17	59619244	59619644	-0.716288978	2.89E-02	decrease
chr3	4493047	4493447	-0.811426059	2.89E-02	decrease
chr17	40287620	40288020	-0.675021575	2.89E-02	decrease
chrX	120250555	120250955	-0.801824222	2.89E-02	decrease
chr12	1592673	1593073	-0.791662989	2.90E-02	decrease
chr2	26033847	26034247	-0.708864778	2.90E-02	decrease
chr5	111512327	111512727	-0.779168674	2.90E-02	decrease

chr17	59220666	59221066	-0.838922839	2.90E-02	decrease
chr7	12210934	12211334	-0.834664939	2.90E-02	decrease
chr19	3185274	3185674	-0.758758272	2.91E-02	decrease
chr5	56909812	56910212	-0.824322067	2.91E-02	decrease
chr3	16264648	16265048	-0.708417899	2.92E-02	decrease
chr17	65865613	65866013	0.922635978	2.92E-02	increase
chr15	41660364	41660764	-0.866066528	2.92E-02	decrease
chr1	46340480	46340880	-0.650968706	2.92E-02	decrease
chr11	66289186	66289586	-0.708482958	2.92E-02	decrease
chr2	101252312	101252712	-0.773866993	2.93E-02	decrease
chr12	49336862	49337262	-0.865750095	2.93E-02	decrease
chr1	228174456	228174856	-0.618241155	2.93E-02	decrease
chr6	43174334	43174734	-0.653334822	2.94E-02	decrease
chr19	45485232	45485632	-0.67253552	2.94E-02	decrease
chr4	39182314	39182714	-0.911337469	2.95E-02	decrease
chr1	145927234	145927634	-0.653684129	2.95E-02	decrease
chr9	100411436	100411836	-0.891747755	2.95E-02	decrease
chr15	74540773	74541173	-0.852067324	2.95E-02	decrease
chr4	1712807	1713207	-0.759559406	2.95E-02	decrease
chr15	41493097	41493497	-0.80043193	2.95E-02	decrease
chr14	67819442	67819842	-0.786544413	2.96E-02	decrease
chr2	216114668	216115068	-0.715539052	2.96E-02	decrease
chr16	1343107	1343507	-0.669880773	2.96E-02	decrease
chr14	95157512	95157912	-0.890488059	2.97E-02	decrease
chr14	101948107	101948507	-0.639066435	2.97E-02	decrease
chr17	81869820	81870220	-0.66835928	2.97E-02	decrease
chr3	48739841	48740241	-0.828497798	2.97E-02	decrease
chr12	52213619	52214019	-0.902051242	2.98E-02	decrease
chr4	123508677	123509077	-0.674577375	2.98E-02	decrease
chr2	73926736	73927136	-0.844133645	3.00E-02	decrease
chr8	73972120	73972520	-0.719344311	3.00E-02	decrease
chr4	143513196	143513596	-0.691834286	3.00E-02	decrease
chr1	38012420	38012820	-0.641693252	3.01E-02	decrease
chr6	75243684	75244084	-0.83175884	3.01E-02	decrease
chr1	155050555	155050955	-0.812814712	3.01E-02	decrease
chr17	60525895	60526295	-0.843820559	3.02E-02	decrease
chr1	91500707	91501107	-0.72379427	3.02E-02	decrease
chr11	22193555	22193955	-0.874223283	3.02E-02	decrease
chr18	59139366	59139766	-0.915539268	3.02E-02	decrease
chr11	2400318	2400718	-0.723606781	3.02E-02	decrease
chr1	54715409	54715809	-0.794879707	3.02E-02	decrease
chr14	32076307	32076707	-0.761593016	3.03E-02	decrease
chr6	87472977	87473377	-0.834682185	3.03E-02	decrease
chr17	75130916	75131316	-0.695796415	3.03E-02	decrease
chr2	126657321	126657721	-0.767723025	3.04E-02	decrease
chr4	105473678	105474078	-0.872041688	3.04E-02	decrease
chr3	173395308	173395708	-0.906460392	3.05E-02	decrease
chr6	93342167	93342567	-0.823417292	3.06E-02	decrease
chr1	155562850	155563250	-0.724637187	3.07E-02	decrease
chr1	24019002	24019402	-0.896717226	3.07E-02	decrease
chr1	197201446	197201846	-0.824711238	3.07E-02	decrease
chr13	49792658	49793058	-0.835913512	3.08E-02	decrease
chr14	69766955	69767355	-0.680386666	3.08E-02	decrease
chr11	66466439	66466839	-0.829791757	3.08E-02	decrease
chr5	123423357	123423757	-0.815323728	3.09E-02	decrease
chr12	101698572	101698972	-0.910936562	3.09E-02	decrease
chr7	66995239	66995639	-0.746323279	3.09E-02	decrease
chr1	155308505	155308905	-0.59165091	3.09E-02	decrease
chr19	4556809	4557209	-0.814647408	3.09E-02	decrease
chr9	104020878	104021278	0.904605137	3.10E-02	increase
chr10	100373477	100373877	-0.721504849	3.10E-02	decrease
chr7	100335901	100336301	-0.796427474	3.10E-02	decrease
chr19	38224342	38224742	-0.854749938	3.10E-02	decrease
chrX	154478907	154479307	-0.676015955	3.11E-02	decrease
chr10	78029488	78029888	-0.823435982	3.11E-02	decrease
chr10	72215985	72216385	-0.858775266	3.11E-02	decrease
chr12	53251472	53251872	-0.806978257	3.11E-02	decrease
chr2	64652541	64652941	-0.880103127	3.11E-02	decrease
chr19	9827526	9827926	-0.800006115	3.12E-02	decrease
chr2	28870230	28870630	-0.764257046	3.12E-02	decrease
chr2	55232205	55232605	-0.696539973	3.13E-02	decrease
chr13	80340037	80340437	-0.696636333	3.13E-02	decrease
chr4	67545308	67545708	-0.872864277	3.13E-02	decrease
chr17	44689623	44690023	-0.670157584	3.13E-02	decrease
chr14	90331663	90332063	-0.7107921	3.14E-02	decrease
chr20	18496698	18497098	-0.879151032	3.14E-02	decrease
chr14	61331989	61332389	-0.89208722	3.14E-02	decrease

chr12	27523841	27524241	-0.825057652	3.14E-02	decrease
chr8	98045624	98046024	-0.80622732	3.15E-02	decrease
chr11	6468946	6469346	-0.841749598	3.15E-02	decrease
chr12	49342324	49342724	-0.791836911	3.15E-02	decrease
chr12	70243705	70244105	-0.887294563	3.15E-02	decrease
chr14	49852717	49853117	-0.717805613	3.15E-02	decrease
chr16	54929256	54929656	-0.774784264	3.16E-02	decrease
chr18	31041755	31042155	-0.800928037	3.16E-02	decrease
chr4	121380292	121380692	-0.873645005	3.16E-02	decrease
chrX	48597733	48598133	-0.799541301	3.16E-02	decrease
chr2	55282213	55282613	-0.817885297	3.16E-02	decrease
chr16	67521318	67521718	-0.789029151	3.16E-02	decrease
chr10	97292344	97292744	-0.826736183	3.17E-02	decrease
chr6	159727037	159727437	-0.808679721	3.17E-02	decrease
chr10	98134640	98135040	-0.860088889	3.17E-02	decrease
chr13	62379290	62379690	0.856111337	3.18E-02	increase
chr2	63588474	63588874	-0.71549263	3.18E-02	decrease
chr3	101573796	101574196	-0.713301849	3.18E-02	decrease
chr1	20507815	20508215	-0.852120341	3.18E-02	decrease
chr3	93979952	93980352	-0.793241728	3.19E-02	decrease
chr17	50200243	50200643	-0.857652713	3.20E-02	decrease
chr8	48921566	48921966	-0.845109439	3.20E-02	decrease
chr1	169485802	169486202	-0.748397538	3.20E-02	decrease
chr1	212608372	212608772	-0.651708605	3.21E-02	decrease
chr5	72849016	72849416	-0.814760296	3.21E-02	decrease
chr13	90055657	90056057	-0.890898761	3.21E-02	decrease
chr1	28669486	28669886	-0.846850573	3.21E-02	decrease
chr8	22400641	22401041	-0.878802658	3.21E-02	decrease
chr12	51951207	51951607	-0.854233506	3.21E-02	decrease
chr3	121545738	121546138	-0.791815921	3.22E-02	decrease
chr16	3282724	3283124	-0.679482519	3.22E-02	decrease
chr6	28349840	28350240	-0.801482028	3.22E-02	decrease
chr5	179820200	179820600	-0.67728011	3.22E-02	decrease
chr2	231706978	231707378	-0.635953729	3.22E-02	decrease
chr10	73110740	73111140	-0.800480202	3.22E-02	decrease
chr12	109999070	109999470	-0.786201209	3.23E-02	decrease
chr1	150213630	150214030	-0.845438458	3.23E-02	decrease
chr15	83446995	83447395	-0.891010126	3.23E-02	decrease
chr15	81266093	81266493	-0.860655778	3.23E-02	decrease
chr7	5570071	5570471	-0.768030235	3.24E-02	decrease
chr14	28767931	28768331	-0.88146185	3.24E-02	decrease
chr7	40134522	40134922	-0.875955176	3.24E-02	decrease
chr2	112480982	112481382	-0.821895039	3.24E-02	decrease
chr5	181161181	181161581	-0.526700238	3.24E-02	decrease
chr13	41455848	41456248	-0.667181611	3.25E-02	decrease
chr16	89995250	89995650	-0.914345896	3.25E-02	decrease
chr5	123511832	123512232	-0.830479215	3.25E-02	decrease
chr5	134226313	134226713	-0.850033215	3.25E-02	decrease
chr22	41621067	41621467	-0.692594163	3.26E-02	decrease
chr12	55829616	55830016	-0.723462359	3.26E-02	decrease
chr10	21533664	21534064	-0.803646865	3.26E-02	decrease
chr8	42842984	42843384	-0.834696466	3.27E-02	decrease
chr14	75258979	75259379	-0.763929207	3.27E-02	decrease
chr17	30708262	30708662	-0.834144613	3.27E-02	decrease
chr21	26843771	26844171	-0.814715878	3.28E-02	decrease
chr5	94618342	94618742	-0.763440601	3.28E-02	decrease
chr10	17643830	17644230	-0.788631844	3.28E-02	decrease
chr3	157160209	157160609	-0.841163483	3.29E-02	decrease
chr12	12812940	12813340	-0.873900841	3.30E-02	decrease
chr2	176001934	176002334	-0.871552735	3.30E-02	decrease
chr5	6633005	6633405	-0.813316815	3.31E-02	decrease
chr12	132638785	132639185	-0.774837711	3.31E-02	decrease
chr2	156332764	156333164	-0.836940527	3.31E-02	decrease
chr1	8878760	8879160	-0.84850194	3.31E-02	decrease
chr16	2023510	2023910	-0.737720728	3.31E-02	decrease
chr3	134373384	134373784	-0.883350693	3.32E-02	decrease
chr21	33588751	33589151	-0.72882259	3.32E-02	decrease
chr7	101252115	101252515	-0.788578093	3.32E-02	decrease
chrX	134372960	134373360	-0.764600889	3.32E-02	decrease
chr12	123601810	123602210	-0.727729654	3.32E-02	decrease
chr2	156341543	156341943	-0.719192103	3.32E-02	decrease
chr7	22822485	22822885	-0.855325174	3.33E-02	decrease
chr1	2231674	2232074	-0.783638224	3.33E-02	decrease
chr16	13919820	13920220	-0.752878948	3.33E-02	decrease
chr1	178726411	178726811	-0.88575674	3.33E-02	decrease
chr22	49902316	49902716	0.899068014	3.34E-02	increase
chr1	228059783	228060183	-0.638833491	3.34E-02	decrease

chr1	154272361	154272761	-0.756183318	3.34E-02	decrease
chr1	6199715	6200115	-0.896563125	3.34E-02	decrease
chr12	130878553	130878953	-0.780095998	3.34E-02	decrease
chr14	74881514	74881914	-0.782582398	3.35E-02	decrease
chr16	31034079	31034479	-0.713393768	3.35E-02	decrease
chr6	21593669	21594069	-0.820071051	3.35E-02	decrease
chr2	71068186	71068586	-0.751406759	3.35E-02	decrease
chr17	42964301	42964701	-0.689375655	3.36E-02	decrease
chr8	100950363	100950763	-0.762181652	3.37E-02	decrease
chr15	76311280	76311680	-0.771675957	3.37E-02	decrease
chr22	25447733	25448133	-0.797443779	3.37E-02	decrease
chr3	128680600	128681000	-0.75818985	3.37E-02	decrease
chr13	30306703	30307103	-0.797252459	3.38E-02	decrease
chr3	170222035	170222435	-0.720183641	3.38E-02	decrease
chr5	140547110	140547510	-0.794661679	3.38E-02	decrease
chr1	1724263	1724663	-0.777956934	3.38E-02	decrease
chr19	37906824	37907224	-0.851635188	3.38E-02	decrease
chr19	48110435	48110835	-0.786517413	3.39E-02	decrease
chr2	40451019	40451419	-0.897904293	3.40E-02	decrease
chr1	226408253	226408653	-0.690254363	3.40E-02	decrease
chr15	78566147	78566547	-0.792469347	3.40E-02	decrease
chr3	196941904	196942304	-0.86391602	3.41E-02	decrease
chr19	41439558	41439958	-0.631508671	3.41E-02	decrease
chr5	79612197	79612597	-0.78459811	3.43E-02	decrease
chr2	37231570	37231970	-0.762432231	3.44E-02	decrease
chr6	154509602	154510002	-0.853528502	3.44E-02	decrease
chrX	41084891	41085291	-0.771517816	3.44E-02	decrease
chr6	106629532	106629932	-0.843664338	3.44E-02	decrease
chr9	92293697	92294097	-0.710342408	3.45E-02	decrease
chr4	155426908	155427308	-0.820493007	3.45E-02	decrease
chr10	15860007	15860407	-0.807654142	3.45E-02	decrease
chr1	30782546	30782946	-0.786457957	3.46E-02	decrease
chr19	14518562	14518962	-0.795726417	3.46E-02	decrease
chr22	19291909	19292309	-0.841887262	3.46E-02	decrease
chr2	105037783	105038183	-0.712347617	3.46E-02	decrease
chr5	78359898	78360298	-0.756505198	3.47E-02	decrease
chr10	17201301	17201701	-0.759420115	3.47E-02	decrease
chrX	70133742	70134142	-0.827867931	3.47E-02	decrease
chr6	27312447	27312847	-0.695440666	3.47E-02	decrease
chr9	85743389	85743789	-0.765998032	3.48E-02	decrease
chr7	91264015	91264415	-0.867522641	3.49E-02	decrease
chr7	149707368	149707768	-0.57026844	3.50E-02	decrease
chr17	45161090	45161490	-0.89949918	3.50E-02	decrease
chr4	82421973	82422373	-0.717190388	3.50E-02	decrease
chr14	20305611	20306011	-0.799185038	3.51E-02	decrease
chr5	124739073	124739473	-0.84056885	3.51E-02	decrease
chr2	58046467	58047102	-0.742240326	3.51E-02	decrease
chr5	95554731	95555131	-0.742496894	3.51E-02	decrease
chr12	57078587	57078987	-0.656342245	3.51E-02	decrease
chr2	32010978	32011378	-0.834478743	3.51E-02	decrease
chr11	112226013	112226413	-0.864395602	3.52E-02	decrease
chr19	50414639	50415039	-0.729283876	3.53E-02	decrease
chr4	118278380	118278780	-0.796612591	3.54E-02	decrease
chr7	129224544	129224944	-0.729444814	3.54E-02	decrease
chr7	89798188	89798588	0.895419546	3.55E-02	increase
chr7	99408733	99409133	-0.735714027	3.55E-02	decrease
chr1	154183060	154183460	-0.589181627	3.55E-02	decrease
chr9	22005338	22005738	-0.83006038	3.55E-02	decrease
chr10	68955794	68956194	-0.717842824	3.56E-02	decrease
chr11	20387174	20387574	-0.862048382	3.56E-02	decrease
chrX	71068418	71068818	-0.778201506	3.56E-02	decrease
chr6	34757078	34757478	-0.846929368	3.57E-02	decrease
chr12	49322728	49323128	-0.76279437	3.57E-02	decrease
chrX	54999939	55000339	-0.882798562	3.57E-02	decrease
chr13	31162103	31162503	-0.667270665	3.58E-02	decrease
chr16	2752225	2752625	-0.693699483	3.58E-02	decrease
chr14	39114203	39114603	-0.76685273	3.58E-02	decrease
chr9	137064254	137064654	-0.858430862	3.58E-02	decrease
chr16	89873549	89873949	-0.822627756	3.58E-02	decrease
chr5	125484828	125485228	-0.871781554	3.59E-02	decrease
chr11	108497425	108497825	-0.846069539	3.59E-02	decrease
chr17	42798505	42798905	-0.757331535	3.60E-02	decrease
chr5	52800208	52800608	-0.765870066	3.60E-02	decrease
chr7	88220085	88220485	-0.846394181	3.60E-02	decrease
chr18	24271323	24271723	-0.844747884	3.62E-02	decrease
chr1	33127193	33127593	-0.647540497	3.62E-02	decrease
chr1	156601262	156601662	-0.75389972	3.62E-02	decrease

chr2	240566186	240566586	-0.830443934	3.63E-02	decrease
chr13	79307637	79308037	-0.792628772	3.63E-02	decrease
chr3	160661630	160662030	-0.876679804	3.64E-02	decrease
chr17	74981979	74982379	-0.714366421	3.64E-02	decrease
chr9	35161972	35162372	-0.826346622	3.65E-02	decrease
chr8	101615083	101615483	-0.80593542	3.66E-02	decrease
chr14	64338891	64339291	-0.804713406	3.66E-02	decrease
chr11	63838664	63839064	-0.745546028	3.66E-02	decrease
chr3	152298783	152299183	-0.770242569	3.66E-02	decrease
chr2	36663957	36664357	-0.889069817	3.67E-02	decrease
chr6	84764381	84764781	-0.810428146	3.68E-02	decrease
chr17	29180602	29181002	-0.840690035	3.68E-02	decrease
chr7	23467738	23468138	-0.812004551	3.68E-02	decrease
chr13	27450134	27450534	-0.727044388	3.69E-02	decrease
chrX	109536758	109537158	-0.833914845	3.69E-02	decrease
chr1	202958768	202959168	-0.794600304	3.69E-02	decrease
chr11	47578683	47579083	-0.72008674	3.70E-02	decrease
chr8	98944380	98944780	-0.857131923	3.71E-02	decrease
chr19	40420885	40421285	-0.778396649	3.71E-02	decrease
chr20	59042361	59042761	-0.87464347	3.71E-02	decrease
chr1	96721786	96722186	-0.840543802	3.73E-02	decrease
chr17	71033025	71033425	-0.821691944	3.73E-02	decrease
chr9	127715715	127716115	-0.669592996	3.73E-02	decrease
chr2	151828498	151828898	-0.842366236	3.74E-02	decrease
chr5	103119983	103120383	-0.654488002	3.74E-02	decrease
chr9	93566650	93567050	-0.835113029	3.74E-02	decrease
chr19	19138169	19138569	-0.741889732	3.75E-02	decrease
chr19	17234906	17235306	-0.809242995	3.75E-02	decrease
chr12	121016165	121016565	-0.762960538	3.75E-02	decrease
chr12	48119000	48119400	-0.778802178	3.75E-02	decrease
chr12	49366285	49366685	-0.818173934	3.76E-02	decrease
chrX	20141858	20142258	-0.795565298	3.77E-02	decrease
chr17	68413650	68414050	-0.886502847	3.77E-02	decrease
chr1	185892822	185893222	-0.848804488	3.77E-02	decrease
chr6	165309228	165309628	-0.870897776	3.77E-02	decrease
chr19	35612721	35613121	-0.83125328	3.77E-02	decrease
chr12	82686210	82686610	-0.838771101	3.77E-02	decrease
chr1	203006854	203007254	-0.649782348	3.78E-02	decrease
chr7	135211621	135212021	-0.799118594	3.78E-02	decrease
chr19	43207301	43207701	-0.618691175	3.78E-02	decrease
chrX	130268583	130268983	-0.780701702	3.79E-02	decrease
chr15	50686727	50687127	-0.822032337	3.81E-02	decrease
chr9	4679406	4679806	-0.727372834	3.81E-02	decrease
chr5	95961857	95962257	-0.804528721	3.81E-02	decrease
chr3	48088953	48089353	-0.736430939	3.81E-02	decrease
chr1	228173845	228174245	-0.569671566	3.82E-02	decrease
chr7	35695122	35695522	-0.799758735	3.82E-02	decrease
chr1	28552606	28553006	-0.73572587	3.83E-02	decrease
chr6	133242788	133243188	-0.755976474	3.83E-02	decrease
chr1	145919028	145919428	-0.69747891	3.84E-02	decrease
chr11	126115723	126116123	-0.744693221	3.85E-02	decrease
chr20	48921343	48921743	-0.873464859	3.86E-02	decrease
chr9	112379559	112379959	-0.784476059	3.86E-02	decrease
chr3	121749608	121750008	-0.740318015	3.86E-02	decrease
chr6	143843020	143843420	-0.704242298	3.86E-02	decrease
chr1	203861397	203861797	-0.676567945	3.87E-02	decrease
chr3	38335014	38335414	0.875244299	3.87E-02	increase
chr6	52200030	52200430	-0.817332464	3.88E-02	decrease
chr1	151146709	151147109	-0.736266066	3.88E-02	decrease
chr1	30718745	30719145	-0.714129315	3.88E-02	decrease
chr20	45812301	45812701	-0.709702007	3.88E-02	decrease
chr1	100249695	100250095	-0.719351871	3.88E-02	decrease
chr21	29147519	29147919	-0.691442165	3.89E-02	decrease
chr17	28812460	28812860	-0.807957369	3.89E-02	decrease
chrX	70434020	70434420	-0.813370491	3.89E-02	decrease
chr3	184135006	184135406	-0.77417857	3.89E-02	decrease
chr2	202265641	202266041	-0.724208734	3.91E-02	decrease
chr5	36606540	36606940	-0.843773624	3.91E-02	decrease
chr1	28510392	28510792	-0.686445989	3.91E-02	decrease
chr19	53559917	53560317	0.828874441	3.91E-02	increase
chr4	142405580	142405980	-0.871233873	3.91E-02	decrease
chr22	19179253	19179653	-0.685318705	3.91E-02	decrease
chr5	132829690	132830090	-0.843989325	3.92E-02	decrease
chr1	244047889	244048289	-0.754632528	3.92E-02	decrease
chr7	66115204	66115604	-0.824312562	3.92E-02	decrease
chr14	58806335	58806735	-0.844360111	3.92E-02	decrease
chr11	8688484	8688884	-0.720030024	3.92E-02	decrease

chr11	62851289	62851689	-0.642156177	3.93E-02	decrease
chr2	9423495	9423895	-0.794607545	3.93E-02	decrease
chr12	49188860	49189260	-0.73567804	3.93E-02	decrease
chr1	173862111	173862511	-0.551688848	3.94E-02	decrease
chr14	22614108	22614508	-0.855587381	3.94E-02	decrease
chr4	56465768	56466168	-0.747421336	3.94E-02	decrease
chr9	93868397	93868797	0.879858819	3.95E-02	increase
chr11	67239586	67239986	-0.665281102	3.96E-02	decrease
chr18	12883861	12884261	-0.879955738	3.96E-02	decrease
chr5	141524769	141525169	-0.788825263	3.96E-02	decrease
chr17	44123714	44124114	-0.727215371	3.96E-02	decrease
chr10	14837912	14838312	-0.669696631	3.97E-02	decrease
chr12	66130503	66130903	-0.864667114	3.97E-02	decrease
chr3	120907813	120908213	-0.773441906	3.97E-02	decrease
chr19	3761801	3762201	-0.798057025	3.97E-02	decrease
chr6	127516550	127516950	-0.831030291	3.98E-02	decrease
chr14	49598772	49599172	-0.687499684	3.98E-02	decrease
chr10	101588004	101588404	-0.757396754	3.98E-02	decrease
chr15	78507323	78507723	-0.63629825	3.99E-02	decrease
chr2	206085899	206086299	-0.760384446	4.00E-02	decrease
chr7	149625403	149625803	-0.677636958	4.00E-02	decrease
chr1	26306264	26306664	-0.843824502	4.01E-02	decrease
chr15	101295319	101295719	-0.770731756	4.01E-02	decrease
chr9	6015576	6015976	-0.786663679	4.01E-02	decrease
chr7	107743755	107744155	-0.782455264	4.01E-02	decrease
chr12	121812364	121812764	-0.744579299	4.02E-02	decrease
chr17	2030125	2030525	-0.757184651	4.02E-02	decrease
chr19	17488821	17489221	-0.716597033	4.02E-02	decrease
chr11	102347941	102348341	-0.865204762	4.03E-02	decrease
chr21	16194217	16194617	-0.832933584	4.03E-02	decrease
chr10	103368611	103369011	-0.735879532	4.03E-02	decrease
chr11	126268840	126269240	-0.655245807	4.03E-02	decrease
chr11	9314554	9314954	-0.725311661	4.03E-02	decrease
chr20	23350191	23350591	-0.769376202	4.04E-02	decrease
chr15	70892378	70892778	-0.685744575	4.04E-02	decrease
chr2	70293619	70294019	-0.670054633	4.05E-02	decrease
chr19	49118671	49119071	-0.650004077	4.05E-02	decrease
chrX	15675069	15675469	-0.610954228	4.06E-02	decrease
chr3	56556978	56557378	-0.713293601	4.06E-02	decrease
chr9	131125197	131125597	-0.678510943	4.06E-02	decrease
chr20	35619387	35619787	-0.717891883	4.06E-02	decrease
chr19	9621116	9621516	-0.866266786	4.06E-02	decrease
chr9	68779759	68780159	-0.799455226	4.07E-02	decrease
chr15	71279235	71279635	-0.836996853	4.07E-02	decrease
chrX	123732736	123733136	-0.711450946	4.10E-02	decrease
chr11	65134597	65134997	-0.586867196	4.11E-02	decrease
chr19	57389654	57390054	-0.719406896	4.11E-02	decrease
chr11	9575099	9575499	-0.768009323	4.12E-02	decrease
chr17	29293987	29294387	-0.715134118	4.12E-02	decrease
chr1	223429669	223430069	-0.863743127	4.13E-02	decrease
chr8	17495326	17495726	-0.619239017	4.13E-02	decrease
chr17	64145731	64146131	-0.706523147	4.13E-02	decrease
chr17	76353870	76354270	-0.663821158	4.13E-02	decrease
chr1	161097730	161098130	-0.777478147	4.13E-02	decrease
chr17	64975242	64975642	-0.719569487	4.13E-02	decrease
chr22	21665934	21666334	-0.677439037	4.13E-02	decrease
chr6_GL000256v2_alt	99948	100348	-0.783391302	4.13E-02	decrease
chr5	75511715	75512115	-0.806405811	4.13E-02	decrease
chrX	49002179	49002579	-0.735842222	4.13E-02	decrease
chr5	112737734	112738134	-0.825863903	4.14E-02	decrease
chr3	50088717	50089117	-0.715214186	4.14E-02	decrease
chr5	271102	271502	-0.694918743	4.15E-02	decrease
chr8	78666036	78666436	-0.800098717	4.15E-02	decrease
chr12	56079045	56079445	-0.698103529	4.15E-02	decrease
chr1	226939743	226940143	-0.561030768	4.16E-02	decrease
chr1	146369928	146370328	-0.496090936	4.16E-02	decrease
chr17	7650452	7650852	-0.770692817	4.16E-02	decrease
chr19	49684435	49684835	-0.715469918	4.16E-02	decrease
chr19	13774124	13774524	-0.729889842	4.18E-02	decrease
chr20	18567324	18567724	-0.830251184	4.18E-02	decrease
chr1	161159194	161159594	-0.655324421	4.18E-02	decrease
chr17	3668298	3668698	-0.70026388	4.18E-02	decrease
chr10	110918874	110919274	-0.601064786	4.18E-02	decrease
chr14	20469294	20469694	-0.509086299	4.18E-02	decrease
chr7	133252965	133253365	-0.853701682	4.19E-02	decrease
chr19	55166754	55167154	-0.653334409	4.19E-02	decrease
chr1	51517890	51518290	-0.821502201	4.20E-02	decrease

chr13	48001012	48001412	-0.801396741	4.20E-02	decrease
chr11	34438664	34439064	-0.694515596	4.20E-02	decrease
chr7	100119512	100119912	-0.6374509	4.21E-02	decrease
chrX	154762648	154763048	-0.727943648	4.22E-02	decrease
chr15	74136017	74136417	-0.818171217	4.22E-02	decrease
chr17	28750553	28750953	-0.737287946	4.22E-02	decrease
chr12	64451743	64452143	-0.773121009	4.22E-02	decrease
chr1	109740175	109740575	-0.774004748	4.22E-02	decrease
chr17	29929500	29929900	-0.793537765	4.22E-02	decrease
chr21	14383054	14383454	-0.630297996	4.23E-02	decrease
chr6	43427467	43427867	-0.678355041	4.23E-02	decrease
chr5	138032761	138033161	-0.814291709	4.23E-02	decrease
chr8	42152619	42153019	-0.802825558	4.24E-02	decrease
chr1	214603008	214603408	-0.722977089	4.24E-02	decrease
chr15	64163336	64163736	-0.818171634	4.24E-02	decrease
chr17	59707498	59707898	-0.707168665	4.24E-02	decrease
chr7	77122275	77122675	-0.74819102	4.25E-02	decrease
chr10	70559150	70559550	-0.815354006	4.25E-02	decrease
chr8	6851115	6851515	0.853095544	4.25E-02	increase
chr7	5526380	5526780	-0.63095755	4.25E-02	decrease
chr14	31420258	31420658	-0.864673354	4.25E-02	decrease
chr21	43728495	43728895	-0.688413401	4.25E-02	decrease
chr1	219839680	219840080	-0.813421448	4.25E-02	decrease
chr11	120991964	120992364	0.837189616	4.26E-02	increase
chr2	38751277	38751677	-0.631633054	4.26E-02	decrease
chr17	28318981	28319381	-0.688712663	4.26E-02	decrease
chr6	37257679	37258079	-0.672177137	4.27E-02	decrease
chr12	110449953	110450353	-0.789030208	4.27E-02	decrease
chr18	59358694	59359094	-0.850099682	4.27E-02	decrease
chr5	79507825	79508225	-0.851482836	4.28E-02	decrease
chr1	224357740	224358140	-0.677901482	4.28E-02	decrease
chr15	40694714	40695114	-0.734094964	4.28E-02	decrease
chr22	31496397	31496797	-0.748087288	4.28E-02	decrease
chr20	10434367	10434767	-0.773726544	4.28E-02	decrease
chr12	100266751	100267151	-0.67244851	4.28E-02	decrease
chr14	51989892	51990292	-0.847772902	4.28E-02	decrease
chr1	114346278	114346678	-0.794358014	4.29E-02	decrease
chr17	49677858	49678258	-0.681591843	4.29E-02	decrease
chr16	56932153	56932553	-0.675450825	4.30E-02	decrease
chr10	43408894	43409294	-0.786961197	4.30E-02	decrease
chr10	102644958	102645358	-0.827480084	4.30E-02	decrease
chr20	59032486	59032886	-0.851463346	4.30E-02	decrease
chr8	70402121	70402521	-0.714704823	4.30E-02	decrease
chr12	76630621	76631021	-0.861873032	4.31E-02	decrease
chr19	40465679	40466079	-0.653713005	4.31E-02	decrease
chr2	58241074	58241474	-0.818119945	4.31E-02	decrease
chr5	138213477	138213877	-0.73056691	4.31E-02	decrease
chr6	154548004	154548404	-0.83367092	4.31E-02	decrease
chr1	214471793	214472193	-0.843162728	4.31E-02	decrease
chr1	161503296	161503696	-0.691603118	4.32E-02	decrease
chr10	91909086	91909486	-0.814813116	4.33E-02	decrease
chr12	49130897	49131297	-0.679617434	4.33E-02	decrease
chr3	51384762	51385162	-0.710491605	4.33E-02	decrease
chr2	62705216	62705616	-0.783394427	4.33E-02	decrease
chr12	57229538	57229938	-0.732013361	4.33E-02	decrease
chr7	100852372	100852772	-0.793001504	4.34E-02	decrease
chr20	24992440	24992840	-0.80021407	4.34E-02	decrease
chr2	73214150	73214550	-0.752429526	4.34E-02	decrease
chrX	119880774	119881174	-0.705709538	4.35E-02	decrease
chr14	77616669	77617069	-0.735679122	4.36E-02	decrease
chr9	33263378	33263778	-0.780048777	4.36E-02	decrease
chr8	53880181	53880581	-0.757813521	4.36E-02	decrease
chr7	102396005	102396405	-0.750852648	4.36E-02	decrease
chr14	102524657	102525057	-0.64990909	4.36E-02	decrease
chr4	98928728	98929128	-0.719986155	4.37E-02	decrease
chr3	53881822	53882222	-0.844633186	4.37E-02	decrease
chr19	45523513	45523913	-0.782705987	4.38E-02	decrease
chr11	66480322	66480722	-0.730049352	4.38E-02	decrease
chr12	43758861	43759261	-0.772699906	4.38E-02	decrease
chr3	171460470	171460870	-0.772927269	4.39E-02	decrease
chr1	46423076	46423476	0.84707816	4.41E-02	increase
chr7	92590077	92590477	-0.668711558	4.41E-02	decrease
chr1	114669849	114670249	-0.636439857	4.41E-02	decrease
chr9	35646576	35646976	-0.721070425	4.42E-02	decrease
chr17	7915439	7915839	-0.743508024	4.42E-02	decrease
chr19	3626802	3627202	-0.693138267	4.43E-02	decrease
chr16	67183431	67183831	-0.662451115	4.43E-02	decrease

chr4	15681436	15681836	-0.699411659	4.43E-02	decrease
chr16	67226778	67227178	-0.644391987	4.43E-02	decrease
chr1	110407473	110407873	-0.681366648	4.44E-02	decrease
chr14	51095233	51095633	-0.673469648	4.44E-02	decrease
chr1	26471636	26472036	-0.785424785	4.44E-02	decrease
chrX	68828879	68829279	-0.789047315	4.45E-02	decrease
chr1	181022820	181023220	-0.749353662	4.45E-02	decrease
chr15	90387727	90388127	-0.729216391	4.45E-02	decrease
chr19	3035685	3036085	-0.710948719	4.45E-02	decrease
chr15	82197868	82198268	-0.817727884	4.45E-02	decrease
chr1	24150272	24150672	-0.853265613	4.45E-02	decrease
chr21	43975261	43975661	-0.737907668	4.46E-02	decrease
chr6	143450644	143451044	-0.714950181	4.47E-02	decrease
chrX	119693143	119693543	-0.752652099	4.47E-02	decrease
chr5	107669267	107669667	-0.793364524	4.47E-02	decrease
chr3	14178472	14178872	-0.702499631	4.48E-02	decrease
chr3	49856469	49856869	-0.698668309	4.48E-02	decrease
chr19	49817909	49818309	-0.63925462	4.48E-02	decrease
chr16	77190781	77191181	-0.737421	4.49E-02	decrease
chr1	46132608	46133008	-0.567569745	4.50E-02	decrease
chr9	6758677	6759077	-0.745375719	4.51E-02	decrease
chr9	113401094	113401494	-0.655562488	4.51E-02	decrease
chr1	211258382	211258782	-0.785078295	4.51E-02	decrease
chr9	97697317	97697717	-0.794222808	4.51E-02	decrease
chr10	67849461	67849861	-0.776503226	4.51E-02	decrease
chr2	200963378	200963778	-0.675716434	4.52E-02	decrease
chr7	90245160	90245560	-0.817989733	4.52E-02	decrease
chr19	43754826	43755226	-0.703202595	4.53E-02	decrease
chr4	176195262	176195662	-0.741953759	4.53E-02	decrease
chr22	23751141	23751541	-0.785813826	4.53E-02	decrease
chr2	27356648	27357048	-0.542476881	4.53E-02	decrease
chr4	99950462	99950862	-0.743499444	4.54E-02	decrease
chr2	152175447	152175847	-0.729842925	4.54E-02	decrease
chr6	28443163	28443563	-0.633467302	4.54E-02	decrease
chr14	96501932	96502332	-0.742428873	4.54E-02	decrease
chr20	20052359	20052759	-0.710730495	4.55E-02	decrease
chr8	144509770	144510170	-0.634380438	4.55E-02	decrease
chr1	21324356	21324756	-0.839120771	4.55E-02	decrease
chr7	100101304	100101704	-0.642134414	4.55E-02	decrease
chr3	184298678	184299078	-0.788526833	4.56E-02	decrease
chr6	125956901	125957301	-0.697589353	4.56E-02	decrease
chr22	46335305	46335705	-0.758580641	4.57E-02	decrease
chrX	153507203	153507603	-0.746124613	4.57E-02	decrease
chr16	21820211	21820611	-0.710578742	4.58E-02	decrease
chr4	5710434	5710834	-0.796430984	4.58E-02	decrease
chr12	109573215	109573615	-0.651715049	4.58E-02	decrease
chr1	200669989	200670389	-0.739309131	4.58E-02	decrease
chr17	68759472	68759872	-0.851912871	4.59E-02	decrease
chr1	53838217	53838617	-0.6857936	4.59E-02	decrease
chr15	40894268	40894668	-0.772654732	4.60E-02	decrease
chr1	228213350	228213750	-0.677871352	4.60E-02	decrease
chr1	64745945	64746345	-0.748858647	4.60E-02	decrease
chr5	126594943	126595343	-0.724066436	4.60E-02	decrease
chr9	33375235	33375635	-0.77357755	4.62E-02	decrease
chr6	46652654	46653054	-0.771817042	4.62E-02	decrease
chr3	105869006	105869406	-0.769931672	4.62E-02	decrease
chr2	219160358	219160758	-0.674928212	4.63E-02	decrease
chr4	31996721	31997121	-0.770256127	4.63E-02	decrease
chr17	81101617	81102017	-0.67036972	4.64E-02	decrease
chr2	97995923	97996323	-0.828207649	4.64E-02	decrease
chr10	87658830	87659230	-0.785875899	4.64E-02	decrease
chr1	155172683	155173083	-0.660671922	4.64E-02	decrease
chr8	37479092	37479492	-0.746145342	4.64E-02	decrease
chr6	2989575	2989975	-0.746079265	4.65E-02	decrease
chr4	73069548	73069948	-0.61697745	4.65E-02	decrease
chr13	91398239	91398639	-0.801312085	4.66E-02	decrease
chr6	87702461	87702861	-0.737642149	4.66E-02	decrease
chr2	85066795	85067195	-0.761126623	4.66E-02	decrease
chr15	69160103	69160503	-0.804622575	4.68E-02	decrease
chr11	118997842	118998242	-0.63244968	4.68E-02	decrease
chr2	219509799	219510199	-0.719990961	4.68E-02	decrease
chr15	84654326	84654726	-0.761445227	4.68E-02	decrease
chr17	76540654	76541054	-0.619973704	4.69E-02	decrease
chr4	65670415	65670815	-0.8328351	4.69E-02	decrease
chr3	47513342	47513742	-0.594527919	4.69E-02	decrease
chr3	156674129	156674529	-0.751893179	4.69E-02	decrease
chr19	1107001	1107401	-0.725357529	4.69E-02	decrease

chr17	2336395	2336795	-0.633350007		4.69E-02	decrease
chr7	7640525	7640925	-0.743032658		4.70E-02	decrease
chr17	48892454	48892854	-0.650975874		4.70E-02	decrease
chr11	74988928	74989328	-0.759672289		4.70E-02	decrease
chr2	46698539	46698939	-0.807189115		4.70E-02	decrease
chr16	68237414	68237814	-0.661838235		4.70E-02	decrease
chr1	44631462	44631862	-0.747088447		4.71E-02	decrease
chr3	43622246	43622646	-0.772351253		4.72E-02	decrease
chr2	71227213	71227613	-0.652811286		4.73E-02	decrease
chr3	185937738	185938138	-0.772225956		4.73E-02	decrease
chr22	35343278	35343678	-0.739342757		4.73E-02	decrease
chr7	149090340	149090740	-0.681415779		4.74E-02	decrease
chr14	24036472	24036872	-0.803682072		4.74E-02	decrease
chr11	83071868	83072268	-0.695370964		4.74E-02	decrease
chr11	66067939	66068339	-0.768076781		4.75E-02	decrease
chr8	123042029	123042429	-0.655280031		4.75E-02	decrease
chr1	110035155	110035555	-0.746722373		4.75E-02	decrease
chr19	43652086	43652486	-0.811044999		4.75E-02	decrease
chr4	99563548	99563948	-0.68445987		4.76E-02	decrease
chr22	38681751	38682151	-0.660912885		4.76E-02	decrease
chr10	60778327	60778727	-0.790521088		4.76E-02	decrease
chr22	37193670	37194070	-0.719983716		4.76E-02	decrease
chr8	11201496	11201896	-0.84363199		4.77E-02	decrease
chr7	139108950	139109350	-0.726712999		4.77E-02	decrease
chr1	46603992	46604392	-0.660158528		4.77E-02	decrease
chr5	98773186	98773586	-0.762544061		4.78E-02	decrease
chr10	101830533	101830933	-0.578401103		4.78E-02	decrease
chr9	128504715	128505115	-0.736402986		4.78E-02	decrease
chr17	76709238	76709638	-0.597942425		4.79E-02	decrease
chr22	24729068	24729468	-0.828463405		4.80E-02	decrease
chr11	73375950	73376350	-0.727625787		4.80E-02	decrease
chr2	71130089	71130489	-0.681769226		4.80E-02	decrease
chr1	99970270	99970670	-0.744675498		4.81E-02	decrease
chr20	5126335	5126735	-0.771103188		4.81E-02	decrease
chr11	77821162	77821562	-0.741449088		4.82E-02	decrease
chr16	18801448	18801848	-0.570196609		4.82E-02	decrease
chr9	105694958	105695358	-0.808095429		4.82E-02	decrease
chr13	41194241	41194641	-0.799904033		4.82E-02	decrease
chr1	75724615	75725015	-0.76717258		4.83E-02	decrease
chr11	65013617	65014017	-0.747048005		4.83E-02	decrease
chr9	104093804	104094204	-0.667545865		4.83E-02	decrease
chr11	72822215	72822615	-0.784137613		4.83E-02	decrease
chr20	49915482	49915882	-0.771433387		4.84E-02	decrease
chr5	60699611	60700011	-0.794477235		4.84E-02	decrease
chr2	121649362	121649762	-0.731900476		4.84E-02	decrease
chr7	102404458	102404858	-0.767638969		4.85E-02	decrease
chr12	49069693	49070093	-0.59391831		4.85E-02	decrease
chr7	76392882	76393282	-0.813266479		4.85E-02	decrease
chr7	100673314	100673714	-0.590855269		4.85E-02	decrease
chr10	93496079	93496479	-0.761827581		4.85E-02	decrease
chr10	23096083	23096483	-0.838064259		4.85E-02	decrease
chr18	34630769	34631169	-0.796441577		4.85E-02	decrease
chr14	21476918	21477318	-0.672542176		4.86E-02	decrease
chr1	9942964	9943364	-0.74280286		4.86E-02	decrease
chr14	92794282	92794682	-0.707676852		4.87E-02	decrease
chr5	143404972	143405372	-0.785609032		4.87E-02	decrease
chr1	27012897	27013297	-0.686261074		4.87E-02	decrease
chr3	48439790	48440190	-0.703377773		4.88E-02	decrease
chr2	45610907	45611307	-0.811637467		4.88E-02	decrease
chr11	65110492	65110892	-0.639268038		4.89E-02	decrease
chr15	42154948	42155348	0.782033225		4.92E-02	increase
chr5	111092361	111092761	-0.781543249		4.93E-02	decrease
chr9	32551053	32551453	-0.758152205		4.93E-02	decrease
chr14	103384961	103385361	-0.705872653		4.93E-02	decrease
chr16	31074199	31074599	-0.590029422		4.93E-02	decrease
chr7	23181753	23182153	-0.734302908		4.93E-02	decrease
chr11	108008553	108008953	-0.809517644		4.93E-02	decrease
chr4	85474982	85475382	-0.757685601		4.93E-02	decrease
chr2	27370009	27370409	-0.541616409		4.94E-02	decrease
chr4	6986750	6987150	-0.811816125		4.94E-02	decrease
chr17	81395026	81395426	-0.603507641		4.95E-02	decrease
chr14	92121635	92122035	-0.689139018		4.95E-02	decrease
chr16	20806492	20806892	-0.765529987		4.95E-02	decrease
chr19	49363451	49363851	-0.495520129		4.96E-02	decrease
chr18	36828504	36828904	-0.728244194		4.96E-02	decrease
chr1	224434762	224435162	-0.602347818		4.96E-02	decrease
chr10	1056688	1057088	-0.769395605		4.97E-02	decrease

chr17	43024692	43025092	-0.723684918	4.97E-02	decrease
chr2	119366842	119367242	-0.657774963	4.97E-02	decrease
chr17	79087412	79087812	-0.706332343	4.98E-02	decrease
chr13	67048473	67048873	-0.736508251	4.98E-02	decrease
chr7	122887175	122887575	-0.814495078	4.99E-02	decrease
chr1	161765074	161765474	-0.634093167	4.99E-02	decrease
chr1	145824051	145824451	-0.750472501	5.00E-02	decrease
chr19	57477177	57477577	-0.834075519	5.00E-02	decrease
chr8	108443460	108443860	-0.706452354	5.00E-02	decrease
chr17	58631544	58631944	-0.664474027	5.00E-02	same
chr1	223846131	223846844	-0.647642752	5.00E-02	same
chr13	79393552	79393952	-0.595861106	5.01E-02	same
chr5	84382256	84382656	-0.774006328	5.01E-02	same
chr22	29603190	29603590	-0.700312046	5.03E-02	same
chr1	155989168	155989568	-0.749903633	5.03E-02	same
chr12	122225915	122226315	-0.641897738	5.04E-02	same
chr7	5822863	5823263	-0.681384682	5.04E-02	same
chr14	69610683	69611083	-0.807953214	5.04E-02	same
chr14	31457089	31457489	-0.734345291	5.04E-02	same
chr4	48340888	48341288	-0.792671394	5.04E-02	same
chr8	109334206	109334606	-0.621766485	5.04E-02	same
chr1	50976956	50977356	-0.790899829	5.04E-02	same
chr11	6619101	6619501	-0.731419908	5.05E-02	same
chr3	130747192	130747592	-0.758055825	5.05E-02	same
chr18	54269534	54269934	-0.833175088	5.06E-02	same
chr10	74150820	74151220	-0.621418119	5.07E-02	same
chr2	175807131	175807531	-0.771030111	5.07E-02	same
chr7	27739912	27740312	-0.734707686	5.07E-02	same
chr2	43595859	43596259	-0.613703476	5.07E-02	same
chr5	132963572	132963972	-0.718416444	5.08E-02	same
chr4	76147987	76148387	-0.624632408	5.10E-02	same
chr1	236162956	236163356	0.778315723	5.11E-02	same
chr13	28718977	28719377	-0.802978084	5.11E-02	same
chr8	140510760	140511160	-0.782664194	5.11E-02	same
chr1	168178926	168179326	-0.698452429	5.11E-02	same
chr1	114757687	114758087	-0.633704146	5.12E-02	same
chr16	53357	53757	-0.674291439	5.12E-02	same
chr16	74985135	74985535	-0.722032729	5.12E-02	same
chr4	3293848	3294248	-0.767359131	5.12E-02	same
chr7	2372961	2373361	-0.772550322	5.13E-02	same
chr6	3118338	3118738	-0.723211807	5.13E-02	same
chr1	151048290	151048690	-0.545735303	5.14E-02	same
chr5	133026211	133026611	-0.735219233	5.14E-02	same
chr19	12933701	12934101	-0.656048045	5.14E-02	same
chr19	48965460	48965860	-0.632961996	5.15E-02	same
chr1	32413749	32414149	-0.714181891	5.15E-02	same
chr22	20495855	20496255	-0.786729833	5.15E-02	same
chr4	75630262	75630662	-0.678692921	5.17E-02	same
chr20	62926509	62926909	-0.755109007	5.17E-02	same
chr17	43368128	43368528	-0.707725039	5.17E-02	same
chr3	47475937	47476337	-0.793369853	5.18E-02	same
chr11	65261838	65262238	-0.756186356	5.19E-02	same
chr4	121071222	121071622	-0.770945928	5.19E-02	same
chr17	30906096	30906496	-0.651839424	5.19E-02	same
chr1	35641374	35641774	-0.654333331	5.20E-02	same
chr12	53299701	53300101	-0.659306061	5.20E-02	same
chr16	58392530	58392930	-0.768310181	5.20E-02	same
chr19	48624256	48624656	-0.713301792	5.20E-02	same
chr6	116680873	116681273	-0.664101367	5.21E-02	same
chr14	57390194	57390594	-0.787984299	5.21E-02	same
chr4	85827818	85828218	-0.788053342	5.21E-02	same
chr9	127245143	127245543	-0.700834835	5.21E-02	same
chr3	171809839	171810239	-0.705637954	5.21E-02	same
chr19	40690756	40691156	-0.701818665	5.22E-02	same
chr14	102984713	102985113	-0.817015698	5.22E-02	same
chr20	31723617	31724017	-0.770816742	5.23E-02	same
chr11	45771505	45771905	-0.78009308	5.23E-02	same
chr17	44014899	44015299	-0.660868409	5.23E-02	same
chr19	17075162	17075562	-0.691259442	5.24E-02	same
chr11	67482963	67483363	-0.704889147	5.24E-02	same
chr14	101761055	101761455	-0.786813851	5.24E-02	same
chr19	48900137	48900537	-0.701906742	5.24E-02	same
chr17	47664188	47664588	-0.776337536	5.24E-02	same
chr1	2199399	2199799	-0.744910703	5.25E-02	same
chr10	103918537	103918937	-0.79361402	5.25E-02	same
chr1	32773090	32773490	-0.550146343	5.26E-02	same
chr1	108691894	108692294	-0.649928458	5.27E-02	same

chr8	8227978	8228378	-0.760010981	5.27E-02	same
chr6	26055981	26056381	-0.577396637	5.27E-02	same
chr11	64743653	64744053	-0.683708815	5.27E-02	same
chr19	55407886	55408286	-0.654437319	5.27E-02	same
chr2	202634579	202634979	-0.765043648	5.28E-02	same
chr3	128650745	128651145	-0.792975003	5.28E-02	same
chr21	17513628	17514028	-0.76900222	5.28E-02	same
chr6	43635658	43636058	-0.665330937	5.28E-02	same
chr4	152536253	152536653	-0.764153455	5.29E-02	same
chr19	11552025	11552425	-0.665141081	5.29E-02	same
chr12	120291561	120291961	-0.485073904	5.30E-02	same
chr6	36123699	36124099	-0.721510527	5.30E-02	same
chr2	9555401	9555801	-0.700580446	5.30E-02	same
chr1	225772154	225772554	-0.665990216	5.31E-02	same
chr5	170633159	170633559	-0.720447488	5.31E-02	same
chr3	72903879	72904279	-0.812542127	5.32E-02	same
chrX	15737905	15738305	-0.631080051	5.33E-02	same
chr8	23528306	23528706	-0.811735377	5.33E-02	same
chr11	61581487	61581887	-0.824118483	5.34E-02	same
chrX	152941609	152942009	-0.760097287	5.34E-02	same
chr2	66434618	66435018	-0.760460376	5.35E-02	same
chr12	92144046	92144446	-0.699487369	5.35E-02	same
chr17	19648577	19648977	-0.635520555	5.35E-02	same
chr17	43388557	43388957	-0.618901617	5.35E-02	same
chr1	35031663	35032063	-0.690591164	5.35E-02	same
chr19	3557618	3558018	-0.710516851	5.35E-02	same
chr1	67439297	67439697	-0.709275457	5.36E-02	same
chr1	178093288	178093688	-0.792665593	5.36E-02	same
chr19	4803989	4804389	-0.662937535	5.37E-02	same
chr9	89318862	89319262	-0.775313754	5.38E-02	same
chr8	65644408	65644808	-0.614911803	5.39E-02	same
chr2	43773943	43774343	-0.771304137	5.39E-02	same
chr19	3762618	3763018	-0.577655363	5.39E-02	same
chr16	2136140	2136540	-0.759990832	5.39E-02	same
chr13	45236132	45236532	-0.765084446	5.40E-02	same
chr4	158810328	158810728	-0.697418499	5.40E-02	same
chr3	141402909	141403309	-0.81531219	5.41E-02	same
chrX	129982490	129982890	-0.61437582	5.41E-02	same
chr12	106986801	106987201	-0.70944316	5.42E-02	same
chr9	26947103	26947503	-0.743370883	5.44E-02	same
chr19	58380311	58380711	-0.726406061	5.44E-02	same
chr11	71972545	71972945	-0.775341943	5.44E-02	same
chr12	50763647	50764047	-0.75110799	5.45E-02	same
chr5	55535095	55535495	-0.769480423	5.45E-02	same
chr1	215569747	215570147	-0.781306926	5.46E-02	same
chr17	8163745	8164145	-0.601173216	5.46E-02	same
chr3	49340160	49340560	-0.653699518	5.47E-02	same
chr1	205210810	205211210	-0.754146754	5.48E-02	same
chr5	148826113	148826513	-0.704931263	5.49E-02	same
chr19	27793583	27793983	-0.773867656	5.49E-02	same
chr16	30355364	30355764	-0.566314459	5.49E-02	same
chr15	97960231	97960631	-0.69102576	5.49E-02	same
chr10	46030384	46030784	-0.731199154	5.49E-02	same
chr17	45161522	45161922	-0.71564233	5.49E-02	same
chr11	90223069	90223469	-0.759247746	5.50E-02	same
chr5	149960622	149961022	-0.733596067	5.50E-02	same
chr2	197692959	197693359	-0.815405364	5.50E-02	same
chr10	68527489	68527889	-0.733456016	5.51E-02	same
chr1	28553183	28553583	-0.719700304	5.51E-02	same
chr19	6677956	6678356	-0.79427782	5.52E-02	same
chr19	46633995	46634395	-0.724610085	5.53E-02	same
chr4	141220772	141221172	-0.780635665	5.54E-02	same
chr19	1443786	1444186	-0.660508799	5.55E-02	same
chr1	32108093	32108493	-0.638614564	5.55E-02	same
chr17	42562098	42562498	-0.681440033	5.55E-02	same
chr7	10973502	10973902	-0.655852071	5.57E-02	same
chr17	18261174	18261574	-0.700375633	5.58E-02	same
chr5	176361391	176361791	-0.649961841	5.58E-02	same
chr11	66002114	66002514	-0.664595329	5.58E-02	same
chr1	93345492	93345892	-0.701213841	5.58E-02	same
chr3	132231674	132232074	-0.426502385	5.58E-02	same
chr6_GL000251v2_alt	133006	133406	-0.816320653	5.58E-02	same
chr17	21099148	21099548	-0.677960097	5.59E-02	same
chr17	67992878	67993278	-0.696257726	5.59E-02	same
chrX	23907321	23907721	-0.801711991	5.61E-02	same
chr14	77580989	77581389	-0.785572323	5.62E-02	same
chrX	123784973	123785373	-0.642718264	5.62E-02	same

chr1	155688699	155689099	-0.702904546	5.63E-02	same
chr12	133080107	133080507	-0.550324837	5.63E-02	same
chr2	174697104	174697504	-0.782663188	5.64E-02	same
chr6	693099	693499	-0.785432812	5.64E-02	same
chr11	66510575	66510975	-0.739018744	5.65E-02	same
chr11	62792001	62792401	-0.667719532	5.65E-02	same
chr8	38386100	38386500	-0.647643405	5.66E-02	same
chr1	150876658	150877058	-0.551884638	5.66E-02	same
chr1	222643986	222644386	-0.606311835	5.66E-02	same
chr11	59171624	59172024	-0.792524137	5.67E-02	same
chr2	231464188	231464588	-0.71703669	5.68E-02	same
chr1	224183638	224184038	-0.655752781	5.69E-02	same
chr6	26607493	26607893	-0.660195094	5.70E-02	same
chr21	33025506	33026160	-0.638732189	5.70E-02	same
chr1	229341332	229341732	-0.685158907	5.70E-02	same
chr1	179293675	179294075	-0.719631583	5.70E-02	same
chr11	62880604	62881004	-0.603842841	5.71E-02	same
chr8	48514165	48514565	-0.751813776	5.71E-02	same
chr4	174156940	174157340	-0.80288345	5.71E-02	same
chrX	131830622	131831022	-0.655922658	5.71E-02	same
chr12	62259949	62260349	-0.646636521	5.73E-02	same
chr12	100200425	100200825	-0.673023719	5.75E-02	same
chr10	110007851	110008251	-0.754292462	5.76E-02	same
chr11	72814629	72815029	-0.684510725	5.76E-02	same
chrX	48696138	48696538	-0.751322432	5.76E-02	same
chr13	26253857	26254257	-0.77969801	5.77E-02	same
chr14	22852929	22853329	-0.72854667	5.77E-02	same
chr2	37376891	37377291	-0.77700256	5.77E-02	same
chr5	139403777	139404177	-0.649077282	5.77E-02	same
chr10	14185676	14186076	-0.764560929	5.77E-02	same
chr6	111259180	111259580	-0.701419173	5.78E-02	same
chr2	222709274	222709674	-0.707580886	5.78E-02	same
chr9	6757537	6757937	-0.684582568	5.79E-02	same
chr7	93622132	93622532	-0.794482946	5.79E-02	same
chr14	49853735	49854135	-0.688703354	5.80E-02	same
chr8	75324310	75324710	-0.804053641	5.80E-02	same
chr2	174395587	174395987	-0.79907665	5.80E-02	same
chr1	76074709	76075109	-0.714623026	5.81E-02	same
chr9	124658231	124658631	-0.710805485	5.82E-02	same
chr20	462657	463057	-0.716717694	5.82E-02	same
chr7	24980365	24980765	-0.754795159	5.82E-02	same
chr7	92452414	92452814	0.80141196	5.82E-02	same
chr19	34677259	34677659	-0.717685798	5.83E-02	same
chrX	47836453	47836853	-0.688190995	5.83E-02	same
chr18	9474908	9475308	-0.706582312	5.83E-02	same
chr22	39519815	39520215	-0.596130799	5.83E-02	same
chr12	44876495	44876895	-0.753405507	5.83E-02	same
chr1	108693059	108693459	-0.717698207	5.83E-02	same
chr1	45802861	45803261	-0.706976492	5.84E-02	same
chr16	30572051	30572451	-0.614115867	5.84E-02	same
chr12	121888613	121889013	-0.644866948	5.84E-02	same
chr2	96857593	96857993	-0.734758712	5.85E-02	same
chr10	112446905	112447305	-0.702334712	5.85E-02	same
chr17	50372869	50373269	-0.705600629	5.85E-02	same
chr20	3204356	3204756	-0.691905568	5.85E-02	same
chrX	127878137	127878537	-0.761451964	5.85E-02	same
chr11	65888180	65888580	-0.615756953	5.86E-02	same
chr7	108525878	108526278	-0.568286727	5.86E-02	same
chr14	51240098	51240498	-0.68078637	5.86E-02	same
chr19	49937807	49938207	-0.658550431	5.86E-02	same
chr20	34303014	34303414	-0.728630064	5.86E-02	same
chr2	189661317	189661717	-0.691335245	5.87E-02	same
chr8	18083344	18083744	-0.787221299	5.87E-02	same
chr4	55395519	55395919	-0.712848464	5.87E-02	same
chr17	18855880	18856280	-0.694977496	5.87E-02	same
chr1	32062394	32062794	-0.670736144	5.87E-02	same
chr10	31318509	31318909	-0.803644536	5.87E-02	same
chr11	112025177	112025577	-0.648147956	5.88E-02	same
chr10	80078728	80079128	-0.737307117	5.88E-02	same
chr11	17276336	17276736	-0.708912538	5.88E-02	same
chr1	149558281	149558681	-0.656339694	5.88E-02	same
chr4	47836839	47837239	-0.799885311	5.88E-02	same
chrUn_KI270438v1	103881	104281	0.376170392	5.88E-02	same
chr19	8002100	8002500	-0.712996001	5.89E-02	same
chr8	54381885	54382285	-0.70137665	5.90E-02	same
chr1	37514757	37515157	-0.793948448	5.91E-02	same
chr7	92134464	92134864	-0.567831308	5.92E-02	same

chr19	5689400	5689800	-0.571170999	5.94E-02	same
chr11	66592891	66593291	-0.675095642	5.94E-02	same
chr2	177552680	177553080	-0.690303048	5.95E-02	same
chr10	68231168	68231568	-0.688040151	5.96E-02	same
chr15	78075866	78076266	-0.637210045	5.96E-02	same
chr2	85637429	85637829	-0.730102152	5.96E-02	same
chr1	228392117	228392517	-0.620179955	5.97E-02	same
chr17	75667109	75667509	-0.679520139	5.97E-02	same
chr1	50960379	50960779	-0.752006489	5.98E-02	same
chr7	129434010	129434410	-0.723586466	5.98E-02	same
chr2	69307226	69307626	-0.75833933	5.99E-02	same
chr12	14803425	14803825	-0.692431447	5.99E-02	same
chr22	21001809	21002209	-0.54821725	6.00E-02	same
chr12	8026723	8027123	-0.685326613	6.00E-02	same
chr22	38569933	38570333	-0.761343583	6.00E-02	same
chrX	73850783	73851183	-0.723740312	6.00E-02	same
chr18	39800038	39800438	-0.786788545	6.01E-02	same
chr7	12519233	12519633	-0.795311127	6.01E-02	same
chr7	99558587	99558987	-0.632098719	6.03E-02	same
chr6	33579933	33580333	-0.722308389	6.03E-02	same
chr17	31858130	31858530	-0.724970942	6.04E-02	same
chr2	174486553	174486953	-0.767818937	6.04E-02	same
chr11	93784079	93784479	-0.677293351	6.04E-02	same
chr9	19380294	19380694	-0.680571969	6.04E-02	same
chrX	110413027	110413427	-0.693891484	6.05E-02	same
chrX	38803923	38804323	-0.70985934	6.05E-02	same
chr13	77027330	77027730	-0.664761501	6.06E-02	same
chr11	435070	435470	-0.682804188	6.07E-02	same
chr20	2470747	2471147	-0.631842894	6.07E-02	same
chr14	57268627	57269027	-0.731291136	6.08E-02	same
chr18	3247454	3247854	-0.689449463	6.09E-02	same
chr16	53054920	53055320	-0.782596772	6.09E-02	same
chr6	83717611	83718011	0.780901063	6.09E-02	same
chr10	87818016	87818416	-0.637559791	6.10E-02	same
chr6	82363707	82364107	-0.767524516	6.10E-02	same
chr1	63327505	63327905	-0.695013177	6.10E-02	same
chr6	138773516	138773916	-0.672594453	6.10E-02	same
chr2	96739877	96740277	-0.722836557	6.11E-02	same
chr12	45727755	45728155	-0.678689377	6.12E-02	same
chr3	184186334	184186734	-0.725124287	6.12E-02	same
chrX	18984719	18985119	-0.775344649	6.12E-02	same
chr10	16436752	16437152	-0.689962147	6.12E-02	same
chr19	5828234	5828634	-0.728933277	6.13E-02	same
chr7	30504680	30505080	-0.60439666	6.14E-02	same
chr5	180493899	180494299	-0.691374544	6.14E-02	same
chr10	63629538	63629938	-0.580262588	6.14E-02	same
chr16	66717136	66717536	-0.696226839	6.15E-02	same
chrUn_GL000195v1	137571	137971	-0.70776226	6.15E-02	same
chr5	179559445	179559845	-0.560915955	6.15E-02	same
chr12	6753374	6753774	-0.620339115	6.16E-02	same
chr11	61361891	61362291	-0.560194849	6.18E-02	same
chr1	220918043	220918443	-0.793742849	6.19E-02	same
chr16	8797380	8797780	-0.675159965	6.19E-02	same
chr10	7787664	7788064	-0.751087708	6.20E-02	same
chr15	74800070	74800470	-0.610823326	6.20E-02	same
chr1	206202950	206203350	-0.661396014	6.21E-02	same
chr15	34969586	34969986	-0.673202701	6.21E-02	same
chr1	20696582	20696982	-0.73915282	6.22E-02	same
chr14	102139354	102139754	-0.644710169	6.22E-02	same
chr10	101066354	101066754	-0.7132455	6.23E-02	same
chr1	100895768	100896168	-0.620237777	6.24E-02	same
chr4	173168204	173168604	-0.76001498	6.25E-02	same
chr14	36320688	36321088	-0.756829535	6.25E-02	same
chr21	46225256	46225656	-0.752384719	6.25E-02	same
chr7	100827599	100827999	-0.680444509	6.26E-02	same
chr17	60677489	60677889	-0.514790267	6.26E-02	same
chr8	67063937	67064337	-0.746114155	6.27E-02	same
chr13	49936120	49936520	-0.716744632	6.28E-02	same
chrX	47556178	47556578	-0.708786666	6.28E-02	same
chr10	101693896	101694296	-0.733603816	6.28E-02	same
chr19	11639643	11640043	-0.753997463	6.29E-02	same
chr16	11836347	11836747	-0.725219982	6.29E-02	same
chr15	41230828	41231228	-0.681015027	6.30E-02	same
chr20	63846273	63846673	-0.723079269	6.31E-02	same
chr2	60881492	60881892	-0.682722321	6.31E-02	same
chr7	149065409	149065809	-0.612393395	6.32E-02	same
chr16	81076837	81077237	-0.581645847	6.33E-02	same

chr16	3500845	3501245	-0.622640763	6.33E-02	same
chr15	91022192	91022592	-0.682015717	6.33E-02	same
chr7	90403450	90403850	-0.711577254	6.34E-02	same
chr8	123241331	123241731	-0.784293441	6.35E-02	same
chr11	86302189	86302589	-0.765328559	6.36E-02	same
chrX	47482368	47482768	-0.737052727	6.36E-02	same
chr20	62900354	62900754	-0.606791698	6.37E-02	same
chr6	4018467	4018867	-0.623336946	6.38E-02	same
chr17	49361017	49361417	-0.697401634	6.40E-02	same
chr7	140479477	140479877	-0.640104486	6.40E-02	same
chr11	47642173	47642573	-0.762804015	6.40E-02	same
chr17	45127546	45127946	-0.700653446	6.41E-02	same
chr2	61176914	61177314	-0.718315399	6.41E-02	same
chr8	127735260	127735660	-0.736256183	6.41E-02	same
chr11	6604087	6604487	-0.690872168	6.42E-02	same
chr12	132561085	132561485	-0.604624908	6.43E-02	same
chrX	119871767	119872167	-0.641720652	6.43E-02	same
chr9	34179451	34179851	-0.754881549	6.43E-02	same
chr19	7535327	7535727	-0.637986673	6.43E-02	same
chr12	27780785	27781185	-0.765763673	6.43E-02	same
chr14	69880180	69880580	-0.759949289	6.44E-02	same
chr18	9102650	9103050	-0.744742663	6.45E-02	same
chr2	72150185	72150585	-0.769649577	6.46E-02	same
chr22	36529105	36529505	-0.527347353	6.46E-02	same
chr19	41884083	41884483	-0.586029697	6.46E-02	same
chr11	16738308	16738708	-0.663571124	6.47E-02	same
chr3	142724630	142725030	-0.746436865	6.47E-02	same
chr3	196287439	196287839	-0.746550356	6.48E-02	same
chr19	10403624	10404024	-0.590984277	6.48E-02	same
chr7	134432464	134432864	-0.650161567	6.50E-02	same
chr16	70381372	70381772	-0.587676645	6.50E-02	same
chr9	109705462	109705862	-0.693069917	6.50E-02	same
chr12	56449080	56449480	-0.587179915	6.52E-02	same
chr6	90586904	90587304	-0.688398278	6.53E-02	same
chr2	172098508	172098908	-0.626554164	6.54E-02	same
chr1	63367431	63367831	-0.646779908	6.54E-02	same
chr11	6612323	6612723	-0.630316378	6.55E-02	same
chr13	30617501	30617901	-0.6102559	6.56E-02	same
chr4	1746034	1746434	-0.637128524	6.56E-02	same
chr14	20643300	20643700	-0.711477716	6.56E-02	same
chr3	184249940	184250340	-0.628982632	6.57E-02	same
chr7	13990888	13991288	-0.706276348	6.58E-02	same
chr1	46247057	46247457	-0.591413553	6.58E-02	same
chr14	30621914	30622314	-0.571632766	6.58E-02	same
chr6	35919667	35920067	-0.705259024	6.59E-02	same
chr16	66552274	66552674	-0.677398195	6.59E-02	same
chr6	18836006	18836406	-0.734265115	6.59E-02	same
chr21	25734897	25735297	-0.543154499	6.60E-02	same
chr7	112450657	112451057	-0.556362917	6.60E-02	same
chr8	18084296	18084696	-0.735467792	6.60E-02	same
chrX	123961297	123961697	-0.772318115	6.61E-02	same
chr2	178478631	178479031	-0.712601908	6.61E-02	same
chr19	42220220	42220620	-0.648562756	6.61E-02	same
chr15	74906781	74907181	-0.659013033	6.61E-02	same
chr11	31509619	31510019	-0.687869861	6.62E-02	same
chr10	122162910	122163310	-0.748112111	6.63E-02	same
chr11	118572398	118572798	-0.686054821	6.63E-02	same
chr9	35116393	35116793	-0.564557655	6.64E-02	same
chr2	108719235	108719635	-0.67788572	6.66E-02	same
chr6	28489886	28490286	-0.524206324	6.66E-02	same
chr11	10857813	10858213	-0.590548265	6.66E-02	same
chr2	61426330	61426730	-0.7136853	6.66E-02	same
chr7	38177799	38178199	-0.773634606	6.67E-02	same
chrX	56729178	56729578	-0.740281625	6.68E-02	same
chr2	159712360	159712760	-0.764839783	6.68E-02	same
chr10	988016	988416	-0.661613172	6.68E-02	same
chr18	34222655	34223055	-0.702154538	6.69E-02	same
chr7	149742243	149742643	-0.645367056	6.70E-02	same
chr14	75176569	75176969	-0.598069194	6.70E-02	same
chr1	81808801	81809201	-0.750790468	6.73E-02	same
chr22	50756859	50757259	-0.672398818	6.73E-02	same
chr16	78099420	78099820	-0.705923288	6.73E-02	same
chr17	39244594	39244994	-0.67250464	6.75E-02	same
chr19	38647105	38647505	-0.67741121	6.75E-02	same
chr7	128404575	128404975	-0.6887003	6.75E-02	same
chr1	244451330	244451730	-0.688973613	6.75E-02	same
chr14	55411829	55412229	-0.715447634	6.75E-02	same

chr2	84926700	84927100	-0.725150776	6.76E-02	same
chr1	223089865	223090265	-0.699978706	6.76E-02	same
chr19	45506900	45507300	-0.705996863	6.76E-02	same
chr2	217069476	217069876	0.780777024	6.76E-02	same
chr4	87220116	87220516	-0.773354177	6.76E-02	same
chr12	130871205	130871605	-0.630450463	6.77E-02	same
chr11	46593674	46594074	-0.734235147	6.79E-02	same
chr4	138241909	138242309	-0.774164094	6.80E-02	same
chr11	64246919	64247319	-0.632656531	6.81E-02	same
chr19	51002688	51003088	-0.714049045	6.81E-02	same
chr10	99659138	99659538	-0.684899753	6.81E-02	same
chrX	49190946	49191346	-0.567549485	6.81E-02	same
chr12	49568152	49568552	-0.623682337	6.82E-02	same
chr6	28124409	28124809	-0.591990743	6.82E-02	same
chr5	81301520	81301920	-0.649790608	6.82E-02	same
chr2	55419438	55419838	-0.728465922	6.83E-02	same
chr13	33818249	33818649	-0.702591469	6.83E-02	same
chrX	103376174	103376574	-0.701399681	6.85E-02	same
chr8	38996428	38996828	-0.689087731	6.85E-02	same
chr3	48898348	48898748	-0.702997549	6.86E-02	same
chr1	108876616	108877016	-0.691184693	6.86E-02	same
chr6	26250041	26250441	-0.5556592	6.87E-02	same
chr1	43358749	43359149	-0.647550343	6.88E-02	same
chr11	70398107	70398507	-0.687343004	6.88E-02	same
chrX	115560814	115561606	-0.600891732	6.88E-02	same
chr19	44644416	44644816	-0.686721887	6.88E-02	same
chr8	94436869	94437269	-0.710404198	6.89E-02	same
chr10	67884201	67884601	-0.605220062	6.89E-02	same
chr7	90346551	90346951	-0.707715497	6.90E-02	same
chr20	49610664	49611064	-0.750546149	6.90E-02	same
chr2	64831515	64831915	-0.762691748	6.90E-02	same
chr10	100987155	100987555	-0.66146234	6.90E-02	same
chr12	132622443	132622843	-0.673731852	6.91E-02	same
chr7	7182021	7182421	-0.752359308	6.91E-02	same
chr7	55366282	55366682	-0.762572236	6.91E-02	same
chrX	106802258	106802658	-0.662944712	6.91E-02	same
chr11	85647601	85648001	-0.68550613	6.91E-02	same
chr10	92592828	92593228	-0.619133612	6.91E-02	same
chr5	159712634	159713034	-0.759169167	6.93E-02	same
chr3	48087813	48088213	-0.691000874	6.93E-02	same
chr7	157557110	157557510	0.688951774	6.93E-02	same
chr19	42396907	42397307	-0.602946194	6.94E-02	same
chrX	111681144	111681544	-0.677270316	6.94E-02	same
chr20	45934008	45934408	-0.644631208	6.95E-02	same
chr10	12129134	12129534	-0.731177062	6.95E-02	same
chr9	126804708	126805436	-0.640424297	6.97E-02	same
chr7	30284185	30284585	-0.703387728	6.97E-02	same
chr1	67832355	67832755	-0.711741846	6.97E-02	same
chr11	95789977	95790377	-0.595978087	6.97E-02	same
chr8	109611090	109611490	-0.670817127	6.98E-02	same
chr2	130391341	130391741	-0.681424664	6.98E-02	same
chr10	52365670	52366070	-0.760084686	6.99E-02	same
chr12	119667764	119668164	-0.636801311	7.02E-02	same
chr2	171433669	171434069	-0.676782274	7.02E-02	same
chr4	88591970	88592370	-0.749227501	7.03E-02	same
chr1	183149433	183149833	-0.773951308	7.04E-02	same
chr7	99439235	99439635	-0.691679709	7.04E-02	same
chr14	35292220	35292620	-0.657753848	7.04E-02	same
chr11	62974989	62975389	-0.730192607	7.05E-02	same
chr6	26341497	26341897	-0.429430881	7.07E-02	same
chr5	150436207	150436607	-0.693326841	7.08E-02	same
chr16	69310715	69311115	-0.737476249	7.08E-02	same
chr4	78056901	78057301	-0.648407523	7.09E-02	same
chr9	89180087	89180487	-0.757449898	7.10E-02	same
chr1	29156706	29157106	-0.637189346	7.10E-02	same
chr17	43170218	43170618	-0.480265238	7.10E-02	same
chr12	51238874	51239274	-0.669436669	7.10E-02	same
chr17	78840829	78841229	-0.587426308	7.10E-02	same
chr11	117138759	117139159	-0.727269975	7.13E-02	same
chr5	138110263	138110663	0.770412894	7.13E-02	same
chr1	109099989	109100389	-0.589704175	7.13E-02	same
chr1	40862459	40862859	-0.740804869	7.13E-02	same
chr12	6605887	6606287	-0.659885601	7.14E-02	same
chr11	1745813	1746213	0.739157005	7.15E-02	same
chr8	37302355	37302755	-0.680322158	7.15E-02	same
chr12	69475680	69476080	-0.728434099	7.16E-02	same
chr1	227953702	227954102	-0.605240031	7.16E-02	same

chr5	36066608	36067008	-0.729814233	7.17E-02	same
chr7	124929742	124930142	-0.742623281	7.18E-02	same
chr2	61854381	61854781	-0.557351093	7.20E-02	same
chr17	6651491	6651891	-0.695933709	7.20E-02	same
chr9	98034580	98034980	-0.725471808	7.21E-02	same
chr15	79923747	79924147	-0.609295949	7.21E-02	same
chr14	74882828	74883228	-0.680146309	7.21E-02	same
chr1	11273180	11273580	-0.694354654	7.21E-02	same
chr16	3023907	3024307	-0.495309265	7.22E-02	same
chr6	28355751	28356151	-0.622130256	7.22E-02	same
chr18	23516075	23516475	-0.667127198	7.23E-02	same
chr14	59188443	59188843	-0.605335325	7.24E-02	same
chr1	151166336	151166736	-0.64510055	7.25E-02	same
chr3	12841442	12841842	-0.645437028	7.26E-02	same
chr20	31951745	31952145	-0.718314129	7.26E-02	same
chr5	173917870	173918270	-0.621916343	7.28E-02	same
chr19	49590089	49590489	-0.529411734	7.29E-02	same
chr14	100376299	100376699	-0.630593264	7.29E-02	same
chr5	179678193	179678593	-0.589784062	7.30E-02	same
chr14	55027088	55027488	-0.642918297	7.32E-02	same
chr5	43483676	43484076	-0.641681415	7.32E-02	same
chr19	3434859	3435259	-0.612907346	7.32E-02	same
chr1	145465326	145465726	-0.652931646	7.32E-02	same
chr6	79537109	79537509	-0.597061593	7.33E-02	same
chr20	47356837	47357237	-0.734547277	7.34E-02	same
chr6	96897401	96897801	-0.765903144	7.34E-02	same
chr19	49301332	49301732	-0.669342043	7.34E-02	same
chr17	49414899	49415299	-0.562189599	7.35E-02	same
chr17	21511695	21512095	-0.739248059	7.35E-02	same
chr5	133968454	133968854	-0.660560052	7.36E-02	same
chr2	171687469	171687869	-0.698344819	7.37E-02	same
chr12	13000363	13000763	-0.695099195	7.39E-02	same
chr19	55654022	55654422	-0.507574226	7.39E-02	same
chr18	74147988	74148388	-0.748975993	7.41E-02	same
chrX	46836819	46837219	-0.654386119	7.41E-02	same
chr17	18087638	18088038	-0.662962337	7.41E-02	same
chr3	52056988	52057388	-0.637569623	7.41E-02	same
chr1	33349961	33350361	-0.728123292	7.44E-02	same
chr11	116772751	116773151	-0.696412643	7.44E-02	same
chr5	159209854	159210254	-0.712808493	7.45E-02	same
chr19	19034066	19034466	-0.718066204	7.45E-02	same
chr8	89984396	89984796	-0.679220017	7.46E-02	same
chr14	67359852	67360252	-0.595131652	7.46E-02	same
chr1	93079157	93079557	-0.636145335	7.46E-02	same
chr4	127632988	127633388	-0.750396987	7.47E-02	same
chr19	50368786	50369186	-0.719205648	7.47E-02	same
chr1	155609836	155610236	-0.621530777	7.48E-02	same
chr1	151006628	151007028	-0.614995739	7.48E-02	same
chr1	84494173	84494573	-0.745534994	7.50E-02	same
chr1	233241529	233241929	-0.703884108	7.50E-02	same
chr17	7281136	7281536	-0.59769431	7.50E-02	same
chr2	232605910	232606310	-0.725619637	7.51E-02	same
chr6	158168282	158168682	-0.66592485	7.52E-02	same
chr15	80153221	80153621	-0.708705909	7.52E-02	same
chr17	9645079	9645479	-0.714843757	7.53E-02	same
chr16	68022340	68022740	-0.696008667	7.53E-02	same
chr21	26833300	26833700	-0.711955204	7.53E-02	same
chr16	28835096	28835496	-0.622625373	7.53E-02	same
chr21	34129221	34129621	-0.665158883	7.54E-02	same
chr5	138178677	138179077	-0.568974196	7.55E-02	same
chr7	84150351	84150751	-0.733172037	7.56E-02	same
chr16	57984783	57985183	-0.627200276	7.56E-02	same
chr5	92641293	92641693	-0.747360516	7.56E-02	same
chr22	36511122	36511522	-0.661526434	7.57E-02	same
chr10	72354588	72354988	-0.694759637	7.57E-02	same
chrX	23743030	23743430	-0.695028459	7.58E-02	same
chr11	61792570	61792970	-0.573720698	7.58E-02	same
chr13	75960619	75961019	-0.732020594	7.59E-02	same
chr7	139776219	139776619	-0.726768555	7.59E-02	same
chr2	64644607	64645007	-0.676564985	7.59E-02	same
chr5	93607598	93607998	-0.678716001	7.60E-02	same
chr19	11505036	11505436	-0.663527259	7.60E-02	same
chrX	153970983	153971383	-0.525626414	7.61E-02	same
chr17	43483784	43484184	-0.640939957	7.62E-02	same
chr1	107965611	107966011	-0.668106591	7.62E-02	same
chr10	78407039	78407439	-0.721317064	7.62E-02	same
chrX	48834565	48834965	-0.750295233	7.63E-02	same

chr19	45406192	45406592	-0.523104206	7.63E-02	same
chr16	56608545	56608945	-0.715041176	7.63E-02	same
chr13	110712926	110713326	-0.665916807	7.64E-02	same
chr2	61017419	61017819	-0.584537928	7.64E-02	same
chr17	6640820	6641220	-0.598564917	7.65E-02	same
chr9	133418020	133418420	-0.640075911	7.65E-02	same
chr1	44674115	44674515	-0.467827829	7.66E-02	same
chrX	103464499	103464899	-0.63197228	7.66E-02	same
chr2	25877899	25878299	-0.751518797	7.66E-02	same
chr8	80367047	80367447	-0.742270022	7.66E-02	same
chr16	89320529	89320929	-0.662659687	7.67E-02	same
chr1	45491023	45491423	-0.560368714	7.68E-02	same
chr1	11012281	11012681	-0.661918924	7.70E-02	same
chr17	49976037	49976437	-0.666374402	7.70E-02	same
chr1	226723922	226724322	-0.596525681	7.70E-02	same
chr2	210170413	210170813	-0.709363812	7.70E-02	same
chr12	56221769	56222169	-0.495675868	7.71E-02	same
chr14	49892526	49892926	-0.701975322	7.71E-02	same
chr6	73513992	73514392	-0.528034971	7.71E-02	same
chr4	90958397	90958797	-0.730485793	7.72E-02	same
chr3	50292505	50292905	-0.590825501	7.72E-02	same
chr5	150758554	150758954	-0.629872941	7.72E-02	same
chr16	53435224	53435624	-0.742131721	7.72E-02	same
chr3	150410975	150411375	-0.720691461	7.73E-02	same
chr2	176097339	176097739	-0.690025237	7.73E-02	same
chr17	42101606	42102006	-0.666904654	7.73E-02	same
chr12	57583364	57583764	-0.671479888	7.74E-02	same
chr17	28599145	28599545	-0.542234674	7.75E-02	same
chr8	38769735	38770135	-0.724314416	7.75E-02	same
chr10	74886359	74886759	0.739380039	7.76E-02	same
chr4	77862824	77863224	-0.635412614	7.76E-02	same
chr1	36397358	36397758	-0.615201819	7.78E-02	same
chr1	247007807	247008207	-0.719291123	7.78E-02	same
chr17	39927673	39928073	-0.697283932	7.79E-02	same
chr1	145845707	145846107	-0.56617407	7.79E-02	same
chr4	122732547	122732947	-0.619425914	7.80E-02	same
chr13	30659931	30660331	-0.670316047	7.83E-02	same
chr11	11620706	11621106	-0.726407795	7.83E-02	same
chr10	100286243	100286643	-0.627237814	7.83E-02	same
chr1	203487459	203487859	-0.598269342	7.84E-02	same
chr17	29140820	29141220	-0.724342161	7.84E-02	same
chr10	73413575	73413975	-0.671122141	7.84E-02	same
chr11	92838665	92839065	-0.717580981	7.84E-02	same
chr22	40818919	40819319	-0.65835836	7.85E-02	same
chr14	24215643	24216043	-0.591413691	7.86E-02	same
chr4	155759031	155759431	-0.733968619	7.88E-02	same
chr6	169247925	169248325	0.73452476	7.88E-02	same
chrX	21839489	21839889	-0.734506113	7.89E-02	same
chr16	2003961	2004361	-0.595011729	7.90E-02	same
chr3	32570812	32571212	-0.688227374	7.91E-02	same
chr9	133351908	133352308	-0.530864518	7.92E-02	same
chr1	244214364	244214764	-0.656854012	7.92E-02	same
chr1	32215679	32216079	-0.621645366	7.93E-02	same
chr4	122922469	122922869	-0.70614865	7.93E-02	same
chr5	73448672	73449072	-0.633630239	7.94E-02	same
chr12	6341787	6342187	-0.631770381	7.95E-02	same
chr11	47269122	47269522	-0.622191406	7.96E-02	same
chr12	118029069	118029469	-0.518491473	7.96E-02	same
chr17	81230807	81231414	-0.604322299	7.97E-02	same
chr1	156572281	156572681	-0.611817638	7.97E-02	same
chr6	116668417	116668817	-0.600270587	7.97E-02	same
chr5	88015013	88015413	-0.699680629	7.97E-02	same
chr2	161160326	161160726	-0.708796038	7.97E-02	same
chr19	36850537	36850937	-0.623837944	7.99E-02	same
chr12	75390998	75391398	-0.672629453	8.00E-02	same
chr10	5685357	5685757	-0.674313017	8.00E-02	same
chr17	28552601	28553001	-0.680981204	8.00E-02	same
chr4	163153086	163153486	-0.745185726	8.01E-02	same
chr4	109206906	109207306	-0.738015299	8.01E-02	same
chr13	52194294	52194694	-0.655452488	8.02E-02	same
chr1	201170810	201171210	-0.566418585	8.03E-02	same
chr3	155703349	155703749	-0.690539537	8.04E-02	same
chr14	79278933	79279333	-0.741103316	8.04E-02	same
chr7	73556714	73557114	-0.679826709	8.05E-02	same
chr2	158967540	158967940	-0.686801465	8.05E-02	same
chr12	26299025	26299425	0.733183102	8.06E-02	same
chr1	161766347	161766747	-0.538782563	8.06E-02	same

chr22	26483471	26483871	-0.664869256	8.07E-02	same
chr11	34916519	34916919	-0.689129805	8.07E-02	same
chr11	73647317	73647717	-0.697608001	8.08E-02	same
chr12	54379634	54380034	-0.638382581	8.08E-02	same
chr11	70270239	70270639	-0.658106913	8.08E-02	same
chr1	162024005	162024405	-0.670702345	8.08E-02	same
chrX	101097880	101098280	-0.624252698	8.09E-02	same
chr9	15552981	15553381	-0.676896631	8.09E-02	same
chr3	42497067	42497467	-0.646676192	8.09E-02	same
chr6	118894238	118894638	-0.696411301	8.10E-02	same
chrX	24054523	24054923	-0.627927072	8.10E-02	same
chr2	219498446	219498846	-0.556190813	8.11E-02	same
chr10	95693666	95694066	-0.636960666	8.11E-02	same
chr19	7920988	7921388	-0.567674213	8.12E-02	same
chr2	197705275	197705675	-0.703554372	8.13E-02	same
chr1	220045987	220046387	-0.629237216	8.13E-02	same
chr6	153070827	153071227	0.691112673	8.13E-02	same
chr15	51971473	51971873	-0.706825005	8.15E-02	same
chr1	45550678	45551078	-0.564362476	8.17E-02	same
chr17	44345164	44345564	-0.582743532	8.18E-02	same
chr16	3690558	3690958	-0.674714458	8.19E-02	same
chr6	36593924	36594324	-0.587981517	8.21E-02	same
chr22	20885548	20885948	-0.620597364	8.21E-02	same
chr10	103396506	103396906	-0.584857177	8.21E-02	same
chr16	4624322	4624722	-0.618763743	8.21E-02	same
chr5	137754373	137754773	-0.600806798	8.22E-02	same
chr12	110469163	110469563	-0.659668506	8.22E-02	same
chr17	16535658	16536058	-0.653532117	8.23E-02	same
chr2	10122513	10122913	-0.632741062	8.23E-02	same
chr1	85061911	85062311	-0.670991538	8.24E-02	same
chr4	95547653	95548053	-0.670624174	8.24E-02	same
chr11	68271256	68271656	-0.654222891	8.25E-02	same
chr9	5947944	5948344	-0.707878768	8.26E-02	same
chr7	5563417	5563817	-0.655958684	8.26E-02	same
chr20	43590611	43591011	-0.60846493	8.27E-02	same
chr14	67695022	67695422	-0.665634338	8.28E-02	same
chr1	59393271	59393671	-0.713152503	8.28E-02	same
chr13	45178738	45179138	-0.726167065	8.28E-02	same
chr1	30929371	30929771	-0.529370733	8.29E-02	same
chr16	18926461	18926861	-0.57197661	8.29E-02	same
chr7	107168567	107168967	-0.5651668	8.29E-02	same
chr16	18790189	18790589	-0.539400598	8.30E-02	same
chr5	172943424	172943824	-0.65227839	8.30E-02	same
chr1	209978726	209979126	-0.732187362	8.31E-02	same
chr13	25301170	25301570	-0.706527723	8.32E-02	same
chr9	70746327	70746727	-0.724891399	8.33E-02	same
chr3	50274368	50274768	-0.614908894	8.33E-02	same
chr1	203306471	203306871	-0.621664562	8.34E-02	same
chr17	7687921	7688321	-0.507486183	8.34E-02	same
chr3	121835011	121835411	-0.634287837	8.35E-02	same
chr15	44195313	44195713	-0.671997301	8.35E-02	same
chr18	9913535	9913935	-0.662117433	8.35E-02	same
chr1	11805813	11806213	-0.532228292	8.35E-02	same
chr19	44113057	44113457	-0.64480802	8.35E-02	same
chr1	8525748	8526148	-0.716348742	8.35E-02	same
chr19	11528850	11529250	-0.588182773	8.35E-02	same
chr7	56064045	56064445	-0.675338743	8.36E-02	same
chr12	106774174	106774574	-0.625315569	8.36E-02	same
chr17	4796082	4796482	-0.652410164	8.37E-02	same
chr5	163505532	163505932	-0.669083225	8.37E-02	same
chr16	77409264	77409664	-0.724439904	8.37E-02	same
chr22	29306373	29306773	-0.573205408	8.38E-02	same
chr1	205631947	205632347	-0.654983975	8.38E-02	same
chr1	85259312	85259712	-0.587467534	8.38E-02	same
chr1	156767246	156767646	-0.513265052	8.40E-02	same
chr15	78872821	78873221	-0.579552289	8.40E-02	same
chr2	27890174	27890574	-0.719424979	8.40E-02	same
chr11	43880376	43880776	-0.711939118	8.41E-02	same
chr5	59317447	59317847	-0.681722065	8.43E-02	same
chr5	173887783	173888183	-0.576357303	8.43E-02	same
chr10	43420538	43420938	-0.724852041	8.43E-02	same
chr1	43171945	43172345	-0.737826162	8.44E-02	same
chr7	74365221	74365621	-0.640929094	8.45E-02	same
chr12	85317883	85318283	-0.737687539	8.45E-02	same
chr1	143720483	143720883	-0.573067531	8.46E-02	same
chr17	77140349	77140749	-0.615445071	8.46E-02	same
chr11	78079339	78079739	-0.671355044	8.47E-02	same

chr2	38666088	38666488	-0.618131887	8.47E-02	same
chr7	100082550	100082950	-0.55053656	8.48E-02	same
chr2	149587650	149588050	-0.652031855	8.49E-02	same
chr4	25914102	25914502	-0.714330321	8.50E-02	same
chr16	11851335	11851735	-0.634587627	8.51E-02	same
chr8	94553364	94553764	-0.603933651	8.51E-02	same
chr10	110265755	110266155	-0.727951356	8.52E-02	same
chr17	48101438	48101838	-0.655244967	8.52E-02	same
chr2	43219980	43220380	-0.628887022	8.52E-02	same
chr1	31944384	31944784	-0.629323762	8.53E-02	same
chr1	112715883	112716283	-0.577801107	8.53E-02	same
chr19	48332827	48333227	-0.564027818	8.53E-02	same
chr14	20971289	20971689	-0.53979337	8.53E-02	same
chr4	70688099	70688499	-0.616214846	8.54E-02	same
chr19	1279572	1279972	-0.55085313	8.54E-02	same
chr16	82029177	82029577	0.716718474	8.54E-02	same
chr16	173397	173797	-0.735620875	8.55E-02	same
chr10	70816350	70816750	-0.710637743	8.55E-02	same
chr8	11703200	11703600	-0.688214699	8.56E-02	same
chr15	89040788	89041188	-0.626364192	8.56E-02	same
chr20	36605284	36605684	-0.653043327	8.56E-02	same
chr11	9385203	9385603	-0.674020378	8.57E-02	same
chr16	31025920	31026320	-0.639340535	8.58E-02	same
chr20	34676414	34676814	-0.696168355	8.58E-02	same
chr19	38336546	38336946	-0.666435667	8.59E-02	same
chr2	231714671	231715071	-0.546919313	8.59E-02	same
chr5	134145914	134146314	-0.697030465	8.60E-02	same
chr9	37486143	37486543	-0.6031462	8.61E-02	same
chr20	3795772	3796172	-0.627910315	8.61E-02	same
chr1	21345544	21345944	-0.698849064	8.61E-02	same
chr3	113514909	113515309	-0.623390343	8.62E-02	same
chr1	145607967	145608367	-0.70835314	8.62E-02	same
chr7	26376886	26377286	-0.670773082	8.63E-02	same
chr15	41283903	41284303	-0.702394867	8.63E-02	same
chr1	40097354	40097754	-0.70898294	8.63E-02	same
chr19	39793085	39793485	-0.682772607	8.64E-02	same
chr6	99568601	99569001	-0.652982083	8.65E-02	same
chr9	35096203	35096603	-0.642617014	8.65E-02	same
chr8	76672594	76672994	-0.678537451	8.68E-02	same
chr14	24270957	24271357	-0.611536645	8.68E-02	same
chr11	236517	236917	-0.677744378	8.68E-02	same
chr14	102305094	102305494	-0.521584074	8.68E-02	same
chr4	159104605	159105005	-0.698808575	8.69E-02	same
chr16	69726388	69726788	-0.623675194	8.70E-02	same
chr13	44141760	44142160	-0.696016942	8.70E-02	same
chr7	123534708	123535108	-0.682791407	8.71E-02	same
chr18	51197947	51198347	-0.630474721	8.71E-02	same
chr5	5422302	5422702	-0.619243664	8.73E-02	same
chr12	5616919	5617319	0.71395183	8.73E-02	same
chr17	28426133	28426533	-0.679643377	8.73E-02	same
chr1	154324670	154325070	-0.543538181	8.73E-02	same
chr1	156367347	156367747	-0.659058558	8.73E-02	same
chr21	14027182	14027582	-0.672086492	8.74E-02	same
chr14	76152168	76152568	-0.702443504	8.74E-02	same
chr7	43729246	43729646	-0.668446687	8.77E-02	same
chr20	45972019	45972419	-0.698060544	8.78E-02	same
chr9	32572829	32573229	-0.678197487	8.78E-02	same
chr15	71252288	71252688	0.582550258	8.79E-02	same
chr8	73008735	73009135	-0.6594497	8.79E-02	same
chr10	60140475	60140875	-0.681020231	8.82E-02	same
chr5	36151793	36152193	-0.597741576	8.83E-02	same
chr1	27725561	27725961	-0.554961012	8.83E-02	same
chrX	48579347	48579747	-0.647675545	8.83E-02	same
chr11	125111550	125111950	-0.640361925	8.83E-02	same
chr18	23502967	23503367	-0.665925522	8.83E-02	same
chr1	203039965	203040365	-0.641343304	8.83E-02	same
chr19	51594267	51594667	-0.674730622	8.84E-02	same
chr19	48513902	48514302	-0.631960636	8.85E-02	same
chr1	77219103	77219503	-0.641136668	8.86E-02	same
chr1	113759512	113759912	-0.647612014	8.88E-02	same
chr2	61888594	61888994	-0.622520755	8.89E-02	same
chrX	48021341	48021741	-0.635145473	8.89E-02	same
chr17	68035575	68035975	-0.488199626	8.90E-02	same
chr16	3106680	3107080	-0.641074783	8.91E-02	same
chr5	83473229	83473629	-0.660488302	8.91E-02	same
chr10	97418789	97419189	-0.610977607	8.92E-02	same
chr16	81096356	81096756	-0.622900393	8.93E-02	same

chr14	41605364	41605764	-0.660448532	8.93E-02	same
chr4	151015129	151015529	-0.615263235	8.94E-02	same
chr2	74421893	74422293	-0.631813223	8.94E-02	same
chr2	160473109	160473509	-0.703575241	8.97E-02	same
chr1	36384642	36385042	-0.643693488	8.97E-02	same
chr7	44573912	44574312	-0.606614538	8.98E-02	same
chr22	37953489	37953889	-0.578188982	8.98E-02	same
chr1	193186246	193186646	-0.684851594	8.99E-02	same
chr6	43516833	43517233	-0.486313625	9.00E-02	same
chr2	68319576	68319976	-0.699344412	9.00E-02	same
chr7	154985799	154986199	-0.663422405	9.01E-02	same
chr5	173328389	173328789	-0.544122875	9.01E-02	same
chr15	65611117	65611517	-0.519312368	9.02E-02	same
chr14	60734699	60735099	-0.701563902	9.02E-02	same
chr11	58578380	58578780	-0.598032209	9.03E-02	same
chr14	63543495	63543895	-0.667290947	9.04E-02	same
chr2	55693237	55693637	-0.654177424	9.04E-02	same
chr6	157379748	157380148	-0.684960937	9.05E-02	same
chr17	47941404	47941804	-0.646450935	9.06E-02	same
chr16	21934996	21935396	-0.663630181	9.06E-02	same
chr14	50532653	50533053	-0.679384359	9.06E-02	same
chr5	93620636	93621036	-0.695429349	9.07E-02	same
chrX	107450814	107451214	-0.662538989	9.07E-02	same
chr1	62783700	62784100	-0.499956449	9.08E-02	same
chr7	42932467	42932867	-0.71939054	9.09E-02	same
chr8	101623828	101624228	-0.686586845	9.09E-02	same
chr12	132615012	132615412	-0.635912583	9.09E-02	same
chr9	132944214	132944614	-0.649189553	9.10E-02	same
chr12	54033019	54033419	-0.64547672	9.11E-02	same
chr5	180293276	180293676	-0.542500502	9.12E-02	same
chr3	141804822	141805222	-0.681007176	9.12E-02	same
chr12	68686857	68687257	-0.5326397	9.13E-02	same
chr19	40426004	40426404	-0.647039676	9.14E-02	same
chr14	74251628	74252028	-0.622144342	9.14E-02	same
chr20	2508967	2509367	-0.60148779	9.14E-02	same
chr6	57961172	57961572	-0.657078081	9.15E-02	same
chr17	2484721	2485121	-0.591618283	9.15E-02	same
chr6	53547739	53548139	-0.68318974	9.15E-02	same
chr6	87408166	87408566	-0.621122945	9.16E-02	same
chr11	3856091	3856491	-0.64722704	9.16E-02	same
chr15	42273134	42273534	-0.553321259	9.17E-02	same
chr2	88691176	88691576	-0.693283903	9.17E-02	same
chr4	92305633	92306033	-0.621223044	9.19E-02	same
chr9	86127181	86127581	-0.620307892	9.20E-02	same
chr20	18507486	18507886	-0.512713828	9.21E-02	same
chr2	201780865	201781265	-0.528389821	9.22E-02	same
chr16	89874282	89874682	-0.569787307	9.22E-02	same
chr12	93467477	93467877	-0.632408482	9.23E-02	same
chr1	155193591	155193991	-0.48277202	9.24E-02	same
chr6	78808625	78809025	0.710624578	9.24E-02	same
chr2	85561501	85561901	-0.620911193	9.24E-02	same
chr19	48255651	48256051	-0.660362707	9.25E-02	same
chr3	142447654	142448054	-0.676838845	9.26E-02	same
chr7	155297007	155297407	-0.611875885	9.27E-02	same
chr8	69833782	69834182	-0.678528364	9.27E-02	same
chr22	50675468	50675868	-0.609605024	9.27E-02	same
chr15	48963263	48963663	-0.707340124	9.28E-02	same
chr1	226353129	226353529	-0.510886876	9.28E-02	same
chr5	141617915	141618315	-0.642059175	9.30E-02	same
chr3	57555824	57556224	-0.609394829	9.31E-02	same
chr7	87151990	87152390	-0.684597395	9.32E-02	same
chr4	158671826	158672510	-0.594958645	9.33E-02	same
chr8	48864002	48864402	0.708950099	9.33E-02	same
chr17	42823934	42824334	-0.625223793	9.33E-02	same
chr12	78102089	78102489	-0.713282815	9.34E-02	same
chr1	17054027	17054427	-0.584754651	9.34E-02	same
chr5	51378679	51379079	-0.712235328	9.35E-02	same
chr3	123201560	123201960	-0.683740332	9.36E-02	same
chr2	175594085	175594485	-0.704176474	9.36E-02	same
chr21	17512277	17512677	-0.633770831	9.36E-02	same
chr8	58056917	58057317	-0.672532285	9.37E-02	same
chr9	78030533	78030933	-0.691186762	9.40E-02	same
chr1	241855140	241855540	-0.589321541	9.40E-02	same
chr7	101217885	101218285	-0.618861407	9.41E-02	same
chr1	161738886	161739286	-0.585997831	9.42E-02	same
chr19	48795250	48795650	-0.577322454	9.42E-02	same
chr6	27162161	27162561	-0.443431936	9.42E-02	same

chr17	78214075	78214475	-0.623027018	9.42E-02	same
chr3	179347387	179347787	-0.670312602	9.42E-02	same
chr1	226186685	226187085	-0.518099238	9.43E-02	same
chrX	40580459	40580859	-0.673037741	9.43E-02	same
chr1	51296900	51297300	-0.65599576	9.44E-02	same
chr19	47229026	47229426	-0.589534127	9.44E-02	same
chr1	150561155	150561555	-0.437126977	9.44E-02	same
chr14	52950108	52950508	-0.706295886	9.44E-02	same
chr6	144286153	144286553	-0.552785939	9.44E-02	same
chr5	23507213	23507613	-0.609003606	9.44E-02	same
chr19	11138785	11139185	-0.521531645	9.45E-02	same
chr21	36069795	36070195	-0.681045712	9.46E-02	same
chr11_KI270830v1_alt	16944	17344	-0.708714763	9.46E-02	same
chr16	22206795	22207195	-0.559431608	9.47E-02	same
chr20	37693959	37694359	-0.679294993	9.47E-02	same
chr15	40405489	40405889	-0.616656381	9.47E-02	same
chr5	154038649	154039049	-0.64846249	9.48E-02	same
chr6	116253794	116254194	-0.604233081	9.50E-02	same
chr1	95072570	95072970	-0.592026012	9.50E-02	same
chr6	26863727	26864127	-0.69064937	9.51E-02	same
chr4	152486135	152486535	-0.706330246	9.51E-02	same
chr13	32315406	32315806	-0.625139114	9.52E-02	same
chr3	98522600	98523000	-0.679507498	9.55E-02	same
chrX	55717732	55718132	-0.707488656	9.56E-02	same
chr1	93080150	93080550	-0.637841921	9.57E-02	same
chr12	6493408	6493808	-0.626185213	9.58E-02	same
chr19	13151842	13152242	-0.56726644	9.61E-02	same
chr6	26066037	26066437	-0.663850297	9.61E-02	same
chr2	218659190	218659590	-0.548127773	9.62E-02	same
chr1	36464359	36464759	-0.587983384	9.63E-02	same
chr3	64686086	64686486	-0.646557306	9.63E-02	same
chr1	182634311	182634711	-0.652069829	9.63E-02	same
chr20	31572616	31573016	-0.597545094	9.64E-02	same
chr1	77682479	77682879	-0.513778459	9.64E-02	same
chr9	86354096	86354496	-0.608080325	9.65E-02	same
chr21	39349322	39349722	-0.620653844	9.65E-02	same
chr5	180860834	180861234	-0.573183282	9.66E-02	same
chr11	61638280	61638680	-0.644719323	9.67E-02	same
chr13	52455053	52455453	-0.649733519	9.68E-02	same
chr19	17830454	17830854	-0.616233642	9.68E-02	same
chr9	33290410	33290810	-0.528271941	9.69E-02	same
chr6	85660156	85660556	-0.708745974	9.70E-02	same
chr1	77779619	77780019	-0.57517623	9.71E-02	same
chr16	67783141	67783541	-0.624209448	9.75E-02	same
chr7	44082495	44082895	-0.659548471	9.76E-02	same
chr4	75673150	75673550	-0.589099197	9.77E-02	same
chr16	4271319	4271719	-0.615955877	9.78E-02	same
chr9	104968255	104968655	-0.660344907	9.78E-02	same
chr13	73223120	73223520	-0.669255531	9.79E-02	same
chr5	138493474	138493874	-0.61439941	9.80E-02	same
chr5	134738420	134738820	-0.543670444	9.80E-02	same
chr17	5486488	5486888	-0.520126882	9.81E-02	same
chr3	89114367	89114767	-0.654327399	9.81E-02	same
chr5	59768365	59768765	-0.699110505	9.87E-02	same
chr7	99551844	99552244	-0.704915884	9.87E-02	same
chr15	45023036	45023436	-0.62530559	9.90E-02	same
chr16	8621636	8622036	-0.675368064	9.91E-02	same
chr12	79935079	79935479	-0.559611924	9.92E-02	same
chr3	49133321	49133721	-0.605335875	9.93E-02	same
chrX	119784751	119785151	-0.616800489	9.94E-02	same
chr1	228664023	228664423	-0.664301826	9.95E-02	same
chr6	116571040	116571440	-0.552672722	9.95E-02	same
chr5	55355927	55356327	-0.690705725	9.95E-02	same
chr9	33024829	33025229	-0.627347563	9.96E-02	same
chr17	80544896	80545296	-0.612635759	9.99E-02	same
chr19	52048961	52049361	-0.679019381	9.99E-02	same
chr1	88684336	88684736	-0.549165224	9.99E-02	same
chr12	119602500	119602900	-0.687824689	9.99E-02	same
chr8	42270701	42271101	-0.661655709	1.00E-01	same
chr1	154983156	154983556	-0.533911304	1.00E-01	same
chr16	30535596	30535996	-0.612861664	1.00E-01	same
chr7	80919359	80919759	-0.697970485	1.00E-01	same
chr12	123711970	123712370	-0.604344399	1.00E-01	same
chr11	62805479	62805879	-0.601327756	1.01E-01	same
chr20	5950212	5950612	-0.621362315	1.01E-01	same
chr15	44536603	44537003	-0.595998209	1.01E-01	same
chr10	5888865	5889265	-0.652851515	1.01E-01	same

chr14	75063747	75064147	-0.634910719	1.01E-01	same
chr10	42782709	42783109	-0.593450131	1.01E-01	same
chr22	37538723	37539123	-0.577622797	1.01E-01	same
chr20	35284776	35285176	-0.56397863	1.01E-01	same
chr9	34329332	34329732	-0.558440714	1.01E-01	same
chr1	25905142	25905542	-0.691898253	1.01E-01	same
chr11	62761413	62761813	-0.623320636	1.01E-01	same
chr19	19120697	19121097	-0.650484709	1.01E-01	same
chrUn_GL000216v2	355	755	0.513199361	1.01E-01	same
chr10	22554560	22554960	-0.653489337	1.01E-01	same
chr1	40040158	40040558	-0.575520506	1.01E-01	same
chr2	188293134	188293534	-0.661329497	1.01E-01	same
chr2	54723334	54723734	-0.58921915	1.01E-01	same
chr4	65998899	65999299	-0.68630125	1.01E-01	same
chr10	122980287	122980687	-0.657900565	1.01E-01	same
chr8	97775389	97775789	-0.690559711	1.01E-01	same
chr14	101463488	101463888	-0.695566115	1.01E-01	same
chr2	74148285	74148685	-0.624201325	1.01E-01	same
chr17	7220740	7221140	-0.494162113	1.01E-01	same
chr22	36481520	36481920	-0.543562847	1.02E-01	same
chr15	98548202	98548602	-0.624164511	1.02E-01	same
chr4	39698039	39698439	-0.690394769	1.02E-01	same
chr22	19448042	19448442	-0.520311133	1.02E-01	same
chrX	135051678	135052078	-0.574181794	1.02E-01	same
chr17	59964409	59964809	-0.652567809	1.02E-01	same
chr21	37547179	37547579	-0.665974548	1.02E-01	same
chr20	44017961	44018361	-0.616420661	1.02E-01	same
chr17	58447460	58447860	-0.664129096	1.02E-01	same
chr8	26291076	26291476	-0.686358395	1.02E-01	same
chr16	68382137	68382537	-0.662331558	1.02E-01	same
chr7	70092471	70092871	-0.664326926	1.02E-01	same
chr1	87718407	87718807	-0.641916073	1.02E-01	same
chr19	51367508	51367908	-0.499592925	1.02E-01	same
chr7	141541382	141541782	0.6728242	1.02E-01	same
chr7	65269296	65269696	-0.636956134	1.03E-01	same
chr15	101334331	101334731	-0.680094277	1.03E-01	same
chr13	39038040	39038440	-0.609474738	1.03E-01	same
chr11	66267701	66268101	-0.609292992	1.03E-01	same
chr1	149712593	149712993	-0.521402063	1.03E-01	same
chr2	96788098	96788498	-0.665779907	1.03E-01	same
chr22	26672673	26673073	-0.598625428	1.03E-01	same
chr12	22543558	22543958	-0.64175405	1.03E-01	same
chr11	61161597	61161997	-0.652278657	1.03E-01	same
chr11	18634398	18634798	-0.547466026	1.03E-01	same
chr13	62465511	62465911	-0.621870433	1.03E-01	same
chr12	48105986	48106386	-0.628652554	1.03E-01	same
chr7	143380233	143380633	-0.544795342	1.03E-01	same
chr2	39124021	39124421	-0.591042299	1.03E-01	same
chr19	57363090	57363490	-0.631519436	1.03E-01	same
chr2	237085622	237086022	-0.595207323	1.03E-01	same
chr10	95655783	95656183	-0.626784351	1.04E-01	same
chr5	150001559	150001959	-0.601412763	1.04E-01	same
chr19	9323985	9324385	-0.650834587	1.04E-01	same
chr5	10249268	10249668	-0.62184879	1.04E-01	same
chr11	67353334	67353734	-0.610904825	1.04E-01	same
chr2	238287661	238288061	-0.623442733	1.04E-01	same
chr12	121352237	121352637	-0.49624516	1.04E-01	same
chr19	8389999	8390399	-0.549066509	1.04E-01	same
chr16	66880299	66880699	-0.576226368	1.04E-01	same
chr16	66662643	66663043	-0.645572256	1.04E-01	same
chr11	65333603	65334003	-0.580319834	1.04E-01	same
chrX	18424918	18425318	-0.641133379	1.04E-01	same
chr2	232550273	232550673	-0.636842658	1.05E-01	same
chr14	67619449	67619849	-0.596764428	1.05E-01	same
chr19	48445904	48446304	-0.60911859	1.05E-01	same
chr9	136842311	136842711	-0.472013068	1.05E-01	same
chr19	18021992	18022392	-0.599032633	1.05E-01	same
chr11	93661299	93661699	-0.683003084	1.05E-01	same
chr10	27100201	27100601	-0.65069744	1.05E-01	same
chr1	146472416	146472816	-0.658988023	1.05E-01	same
chr6	125986425	125986825	-0.510065937	1.05E-01	same
chrX	47658866	47659266	-0.535957399	1.05E-01	same
chr11	65169968	65170368	-0.57528494	1.05E-01	same
chr17	42017400	42017800	-0.507137057	1.05E-01	same
chr1	161153783	161154183	-0.551446908	1.05E-01	same
chr4	112706482	112706882	-0.647438472	1.06E-01	same
chr11	126303981	126304381	-0.619059889	1.06E-01	same

chr11	129695619	129696019	0.667721239	1.06E-01	same
chr5	154682462	154682862	-0.612045132	1.06E-01	same
chr3	52771004	52771404	-0.597201769	1.06E-01	same
chr11	4608031	4608431	-0.6303887	1.06E-01	same
chr15	34988390	34988790	-0.659431383	1.06E-01	same
chr10	27155258	27155658	-0.5935505	1.06E-01	same
chr1	224456925	224457325	-0.553708809	1.06E-01	same
chr1	53220314	53220714	-0.556157443	1.06E-01	same
chr9	99820475	99820875	-0.659818523	1.06E-01	same
chr11	16613739	16614139	-0.639295103	1.07E-01	same
chr19	4867728	4868128	-0.585447376	1.07E-01	same
chr12	132908526	132908926	-0.619817629	1.07E-01	same
chr6	99515625	99516025	-0.615691349	1.07E-01	same
chr17	39450982	39451382	-0.590955524	1.07E-01	same
chr4	3480625	3481025	-0.580411687	1.07E-01	same
chr7	74079231	74079631	-0.566139451	1.07E-01	same
chr20	13638591	13638991	-0.613938151	1.07E-01	same
chr12	68610391	68610791	-0.688070713	1.07E-01	same
chr1	37595719	37596119	-0.56330664	1.07E-01	same
chr3	111978856	111979256	-0.581885287	1.07E-01	same
chr7	33722387	33722787	-0.605606784	1.07E-01	same
chr1	68496879	68497279	-0.503775325	1.07E-01	same
chr1	177170788	177171188	-0.64828439	1.07E-01	same
chr2	74390797	74391197	-0.668314087	1.08E-01	same
chr7	144505572	144505972	0.657563319	1.08E-01	same
chrX	77785982	77786382	-0.635108386	1.08E-01	same
chr7	6590604	6591004	-0.6513745	1.08E-01	same
chr17	19377955	19378355	-0.576358568	1.08E-01	same
chr1	191813698	191814098	-0.685358358	1.08E-01	same
chr14	30690402	30690802	-0.653779993	1.08E-01	same
chr17	45147998	45148398	-0.557145283	1.08E-01	same
chr1	12478230	12478630	-0.63746122	1.09E-01	same
chr3	184336491	184336891	-0.539937127	1.09E-01	same
chr4	154416614	154417014	-0.684496611	1.09E-01	same
chr1	179882253	179882653	-0.404424476	1.09E-01	same
chr12	113966161	113966561	-0.577373664	1.09E-01	same
chr19	6516328	6516728	-0.511921576	1.09E-01	same
chr17	75784626	75785026	-0.535209236	1.09E-01	same
chr12	53054178	53054578	-0.499967995	1.09E-01	same
chr7	103297405	103297805	-0.654592672	1.09E-01	same
chr22	37686281	37686681	-0.519669908	1.09E-01	same
chr7	87039855	87040255	-0.645252858	1.09E-01	same
chr2	157838890	157839290	-0.632510765	1.09E-01	same
chr4	176692290	176692690	-0.67678867	1.09E-01	same
chr18	62522582	62522982	-0.644730815	1.10E-01	same
chr19	45492884	45493284	-0.508151921	1.10E-01	same
chr14	37034934	37035334	-0.677537993	1.10E-01	same
chr1	183235155	183235555	-0.492367412	1.10E-01	same
chr1	228071787	228072187	-0.468949257	1.10E-01	same
chr6	27667794	27668194	-0.317874661	1.10E-01	same
chrX	71532545	71532945	-0.628477606	1.10E-01	same
chr11	43311856	43312256	-0.654857987	1.10E-01	same
chrX	136880607	136881007	-0.550726551	1.10E-01	same
chr22	39399502	39399902	-0.61445652	1.10E-01	same
chr7	8303990	8304390	-0.674897063	1.10E-01	same
chr1	167926773	167927173	-0.612953544	1.10E-01	same
chr7	128739152	128739552	-0.570233672	1.10E-01	same
chr2	196799304	196799704	-0.643685426	1.10E-01	same
chr15	74889585	74889985	-0.571787733	1.10E-01	same
chr9	98280563	98280963	-0.641972817	1.10E-01	same
chr5	150000287	150000687	-0.571619669	1.10E-01	same
chr17	7455494	7455894	-0.600142414	1.10E-01	same
chr2	231961650	231962050	-0.63292009	1.11E-01	same
chr17	62965601	62966001	-0.615691867	1.11E-01	same
chr20	41693158	41693558	-0.623534635	1.11E-01	same
chr16	71564840	71565240	-0.581314187	1.11E-01	same
chr13	77697255	77697655	-0.638205751	1.11E-01	same
chr17	75171975	75172375	-0.623544612	1.11E-01	same
chr1	147541569	147541969	-0.59245388	1.11E-01	same
chr12	41288129	41288529	-0.645182956	1.11E-01	same
chr1	32716765	32717165	-0.517548871	1.11E-01	same
chr9	129006599	129006999	-0.512825374	1.11E-01	same
chrX	47193254	47193654	-0.594498467	1.11E-01	same
chr19	57819371	57819771	-0.56968193	1.11E-01	same
chr5	138575405	138575805	-0.64741243	1.11E-01	same
chr11	69675401	69675801	-0.640215254	1.12E-01	same
chrX	14029492	14029892	-0.656040862	1.12E-01	same

chr1	211630217	211630617	-0.614697135	1.12E-01	same
chr17	35578385	35578785	-0.583061992	1.12E-01	same
chr4	74157708	74158108	-0.640703314	1.12E-01	same
chr3	81761044	81761444	-0.586164674	1.12E-01	same
chr9	33523367	33523767	-0.619959896	1.12E-01	same
chr9	121055444	121055844	-0.673558908	1.12E-01	same
chr17	4143165	4143565	-0.519260142	1.12E-01	same
chrX	103918742	103919142	-0.587160442	1.12E-01	same
chr12	76764199	76764599	-0.562380491	1.12E-01	same
chr14	24213144	24213544	-0.462824325	1.12E-01	same
chr11	72434313	72434713	-0.546863728	1.12E-01	same
chr15	75903805	75904205	-0.507896948	1.12E-01	same
chr2	70994966	70995366	-0.653210298	1.13E-01	same
chr6	42050465	42050865	-0.60710712	1.13E-01	same
chr1	109483405	109483805	-0.56652944	1.13E-01	same
chr6	145815004	145815404	-0.56722414	1.13E-01	same
chr3	50978900	50979300	-0.667980189	1.13E-01	same
chr1	20633906	20634306	-0.661714639	1.13E-01	same
chr3	120596213	120596613	-0.605288213	1.13E-01	same
chr10	75210061	75210461	-0.599296032	1.13E-01	same
chr17	42577915	42578315	-0.583437325	1.13E-01	same
chr12	96400838	96401238	-0.64308053	1.13E-01	same
chr14	55581079	55581479	-0.630684363	1.13E-01	same
chr11	10933875	10934275	-0.574918508	1.13E-01	same
chr11	19240672	19241072	-0.628054087	1.13E-01	same
chr12	123383658	123384058	-0.513533001	1.13E-01	same
chr7	14389634	14390034	-0.661029152	1.13E-01	same
chr11	31812594	31812994	-0.599745884	1.13E-01	same
chr5	140303066	140303466	-0.564472367	1.13E-01	same
chr11	114400325	114400725	-0.587880604	1.14E-01	same
chr16	4693198	4693598	-0.556023423	1.14E-01	same
chr14	24367525	24367925	-0.558208576	1.14E-01	same
chr5	126778319	126778719	-0.580685719	1.14E-01	same
chr4	153249238	153249638	-0.62367085	1.14E-01	same
chr22	21938232	21938632	-0.556830427	1.14E-01	same
chr10	55899868	55900268	-0.67407225	1.14E-01	same
chr6	112087277	112087677	-0.50318877	1.14E-01	same
chr1	109089424	109089824	-0.587230867	1.14E-01	same
chr19	41706747	41707147	-0.571081048	1.14E-01	same
chr14	73644806	73645206	-0.566058906	1.14E-01	same
chr2	37084296	37084696	-0.539856751	1.15E-01	same
chr3	75785251	75785651	-0.674933122	1.15E-01	same
chr14	50321158	50321558	-0.637178027	1.15E-01	same
chr19	52570161	52570561	-0.642250447	1.15E-01	same
chr2	219206431	219206831	-0.593305039	1.15E-01	same
chr1	156213021	156213421	-0.457568477	1.15E-01	same
chr9	70420440	70420840	-0.608562546	1.15E-01	same
chr19	50461065	50461465	-0.607265168	1.15E-01	same
chrX	119984588	119984988	-0.598736942	1.15E-01	same
chr17	71063424	71063824	-0.661011482	1.15E-01	same
chr2	75646694	75647094	-0.609747997	1.16E-01	same
chr1	119647448	119647848	-0.590639519	1.16E-01	same
chr5	59275764	59276164	-0.672246052	1.16E-01	same
chr10	5666830	5667230	-0.669009684	1.16E-01	same
chr6	152301816	152302216	-0.610643036	1.16E-01	same
chr5	140639426	140639826	-0.672248901	1.16E-01	same
chr11	76211664	76212064	-0.606019284	1.16E-01	same
chr17	81793875	81794275	-0.601637049	1.16E-01	same
chr1	39799602	39800002	-0.575437472	1.16E-01	same
chr11	121226651	121227051	-0.656492422	1.16E-01	same
chr15	101772998	101773398	-0.621061958	1.16E-01	same
chr4	89056859	89057259	-0.61893993	1.16E-01	same
chr17	62703590	62703990	-0.652171954	1.16E-01	same
chr7	76166539	76166939	-0.625490717	1.16E-01	same
chr12	53451774	53452174	-0.595801289	1.16E-01	same
chr6	26188808	26189208	-0.426390207	1.17E-01	same
chr1	204889720	204890120	-0.605466162	1.17E-01	same
chr10	14878101	14878501	-0.531467184	1.17E-01	same
chr6	138498697	138499097	-0.64831028	1.17E-01	same
chr1	169367861	169368261	-0.543978909	1.17E-01	same
chr19	3545079	3545479	-0.587170693	1.17E-01	same
chr3	180912253	180912653	-0.623731671	1.17E-01	same
chr15	82986012	82986412	-0.549145838	1.17E-01	same
chr17	55750966	55751366	-0.605323965	1.17E-01	same
chr1	8439266	8439666	-0.653822116	1.18E-01	same
chr17	50178218	50178618	-0.557515065	1.18E-01	same
chr8	124425868	124426268	-0.635453421	1.18E-01	same

chr17	8376411	8376811	-0.501128031	1.18E-01	same
chr14_GL000225v1_random	74333	74733	0.665114478	1.18E-01	same
chr5	116572956	116573356	-0.657849899	1.18E-01	same
chr17	67726984	67727384	-0.607269095	1.18E-01	same
chr7	149196631	149197031	-0.520452348	1.19E-01	same
chr14	60659118	60659518	-0.637100066	1.19E-01	same
chr20	6960006	6960406	-0.619682118	1.19E-01	same
chr17	80024518	80024918	-0.650306722	1.19E-01	same
chr4	84499465	84499865	-0.582378255	1.19E-01	same
chr5	88268551	88268951	-0.59946276	1.19E-01	same
chr10	13302056	13302456	-0.604337997	1.20E-01	same
chr11	76721804	76722204	-0.606651549	1.20E-01	same
chr1	103525540	103525940	-0.621400023	1.20E-01	same
chr9	120868971	120869371	-0.608095951	1.20E-01	same
chrX	74292513	74292913	-0.545515496	1.20E-01	same
chr3	13648394	13648794	-0.65124464	1.20E-01	same
chr14	23301871	23302271	-0.601641129	1.20E-01	same
chr9	137200868	137201268	-0.554517564	1.20E-01	same
chr8	144373366	144373766	-0.505976545	1.20E-01	same
chr16	267343	267743	-0.647930702	1.20E-01	same
chr16	3235106	3235506	-0.516532788	1.20E-01	same
chr4	177441878	177442278	-0.625354817	1.20E-01	same
chr4	119730348	119730748	-0.646295252	1.21E-01	same
chrX	53281296	53281696	-0.607287387	1.21E-01	same
chr14	72894314	72894714	-0.574469638	1.21E-01	same
chr22	35299835	35300235	-0.497743342	1.21E-01	same
chr17	15699407	15699807	-0.647201191	1.21E-01	same
chr4	94207520	94207920	-0.641112949	1.21E-01	same
chr9	98056251	98056651	-0.545595998	1.21E-01	same
chr7	28410673	28411073	-0.561757145	1.21E-01	same
chr10	68471688	68472088	-0.572387211	1.21E-01	same
chr6	13615685	13616085	-0.650140502	1.21E-01	same
chr1	151613042	151613442	-0.590479599	1.21E-01	same
chr11	63872009	63872409	-0.593496268	1.21E-01	same
chr19	45091737	45092137	-0.565981627	1.22E-01	same
chr11	128999109	128999509	-0.631847641	1.22E-01	same
chr11	8682333	8682733	-0.548784844	1.22E-01	same
chr1	59814300	59814700	-0.529505111	1.22E-01	same
chr9	70258581	70258981	-0.594400623	1.22E-01	same
chr17	47188965	47189365	-0.46882474	1.22E-01	same
chrX	49163923	49164323	-0.57804723	1.22E-01	same
chr18	48952554	48952954	-0.636748707	1.22E-01	same
chr8	80029934	80030334	-0.530254815	1.23E-01	same
chr19	49500319	49500719	-0.482892765	1.23E-01	same
chr9	34665566	34665966	-0.555286494	1.23E-01	same
chr12	15688455	15688855	-0.632655795	1.23E-01	same
chr3	64023119	64023519	-0.642805814	1.23E-01	same
chr17	7836405	7836805	-0.491935105	1.23E-01	same
chr4	134200785	134201185	-0.632394053	1.23E-01	same
chrX	150982809	150983209	-0.625737604	1.23E-01	same
chr1	204529107	204529507	-0.615338539	1.23E-01	same
chr16	14325456	14325856	-0.643629038	1.23E-01	same
chr11	9613521	9613921	-0.486651307	1.23E-01	same
chr11	77047205	77047605	-0.635176421	1.24E-01	same
chr1	150293774	150294174	-0.562280817	1.24E-01	same
chr1	165630994	165631394	-0.529809853	1.24E-01	same
chr12	62602513	62602913	-0.630395327	1.24E-01	same
chr1	144461326	144461726	-0.631403737	1.24E-01	same
chr12	132157846	132158246	-0.609430356	1.24E-01	same
chr1	113904880	113905280	-0.419545042	1.24E-01	same
chr11	33039235	33039635	-0.559887978	1.24E-01	same
chr19	21329043	21329443	-0.620236429	1.25E-01	same
chr2	16113146	16113546	-0.615609669	1.25E-01	same
chr12	75329998	75330398	-0.648663073	1.25E-01	same
chr8	92627874	92628274	-0.6415054	1.25E-01	same
chr14	54509791	54510191	-0.532144727	1.25E-01	same
chr2	85612021	85612421	-0.648504582	1.25E-01	same
chr17	48996373	48996773	-0.502498017	1.25E-01	same
chr1	234326654	234327054	-0.571388934	1.25E-01	same
chr10	71377202	71377602	-0.602848216	1.25E-01	same
chr8	100559396	100559796	-0.619459962	1.25E-01	same
chr11	12800206	12800606	-0.640961139	1.26E-01	same
chr2	46916058	46916458	-0.615105827	1.26E-01	same
chr12	54017999	54018399	-0.536738792	1.26E-01	same
chr1	42682067	42682467	-0.497196193	1.26E-01	same
chr5	177516990	177517390	-0.463252821	1.26E-01	same
chr18	35344056	35344456	-0.554677301	1.26E-01	same

chr12	30753443	30753843	-0.648990703	1.26E-01	same
chr11	108593519	108593919	-0.615901081	1.26E-01	same
chr15	73994617	73995017	-0.571102816	1.26E-01	same
chr22	37561816	37562216	-0.565040483	1.26E-01	same
chr1	117929222	117929622	-0.487235947	1.27E-01	same
chr22	37661069	37661469	-0.499359918	1.27E-01	same
chr1	247078616	247079016	-0.55591534	1.27E-01	same
chr15	68054790	68055190	-0.607679029	1.27E-01	same
chr10	47553158	47553558	-0.571104531	1.27E-01	same
chr5	37379085	37379485	-0.624837294	1.27E-01	same
chr1	16514042	16514442	-0.572209806	1.27E-01	same
chr1	212950253	212950653	-0.543422225	1.27E-01	same
chr11	57325125	57325525	-0.53726646	1.27E-01	same
chr9	130690163	130690563	-0.509195759	1.27E-01	same
chr2	183038524	183038924	-0.610270523	1.27E-01	same
chr6	10838148	10838548	-0.586628787	1.27E-01	same
chr1	62419833	62420233	0.595248361	1.27E-01	same
chr12	57693769	57694169	-0.504066935	1.27E-01	same
chr13	45120237	45120637	-0.595014365	1.27E-01	same
chr5	169980502	169980902	-0.623917218	1.27E-01	same
chr7	2555455	2555855	-0.602132052	1.28E-01	same
chr15	43330337	43330737	-0.558262872	1.28E-01	same
chr19	37594568	37594968	-0.572753241	1.28E-01	same
chrX	116806260	116806660	0.631111708	1.28E-01	same
chrX	120485883	120486283	-0.534549065	1.28E-01	same
chr3	134213082	134213482	-0.637878626	1.28E-01	same
chr14	54366998	54367398	-0.579492569	1.28E-01	same
chrX	102769004	102769404	-0.525763017	1.28E-01	same
chr17	81200872	81201272	-0.562613352	1.28E-01	same
chr20	45857291	45857691	-0.530912707	1.28E-01	same
chr1	41360763	41361163	-0.567200611	1.28E-01	same
chr11	64449215	64449615	-0.59953433	1.28E-01	same
chr22	31970161	31970561	-0.598879025	1.28E-01	same
chr2	175454886	175455286	-0.610554722	1.29E-01	same
chr11	121024817	121025217	-0.633797445	1.29E-01	same
chr17	16380644	16381044	-0.576320834	1.29E-01	same
chr19	2061608	2062008	-0.54763162	1.29E-01	same
chr19	7098914	7099314	-0.588143418	1.29E-01	same
chr2	85354332	85354732	-0.493713642	1.29E-01	same
chr17	76121193	76121593	-0.623800606	1.29E-01	same
chr17	29389924	29390324	-0.552423998	1.29E-01	same
chr10	126216014	126216414	-0.60193484	1.29E-01	same
chr1	112674304	112674704	-0.536034365	1.29E-01	same
chr11	74948964	74949364	-0.555304215	1.29E-01	same
chr1	75614589	75614989	-0.522965386	1.30E-01	same
chr10	89701313	89701713	-0.516678685	1.30E-01	same
chr12	110468306	110468706	-0.575203143	1.30E-01	same
chr18	48221346	48221746	-0.64528801	1.30E-01	same
chr12	120087105	120087505	-0.485119327	1.30E-01	same
chr1	109532442	109532842	-0.594806494	1.30E-01	same
chr20	324665	325065	-0.545085032	1.30E-01	same
chr8	81731774	81732174	-0.611064439	1.30E-01	same
chr19	2950520	2950920	-0.541464673	1.30E-01	same
chr5	23527249	23527649	-0.64349486	1.30E-01	same
chrUn_KI270538v1	88551	88951	0.627413621	1.30E-01	same
chr15	41972628	41973028	-0.611341508	1.30E-01	same
chr7	103344488	103344888	-0.594191475	1.31E-01	same
chr4	155376494	155376894	-0.542436121	1.31E-01	same
chr12	56266807	56267207	-0.52430625	1.31E-01	same
chr19	51703833	51704233	-0.572173891	1.31E-01	same
chr17	60886955	60887355	-0.643785069	1.32E-01	same
chr12	107761471	107761871	-0.620229734	1.32E-01	same
chr19	19192426	19192826	-0.438341998	1.32E-01	same
chr2	196639575	196639975	-0.527080795	1.32E-01	same
chr6	112254884	112255284	-0.597687257	1.33E-01	same
chr3	197298642	197299042	-0.623279609	1.33E-01	same
chr13	93227019	93227419	-0.574605592	1.33E-01	same
chr3	49554055	49554455	-0.565724858	1.33E-01	same
chr6	7590076	7590476	-0.57347786	1.33E-01	same
chr15	99251002	99251402	-0.550190382	1.33E-01	same
chr13	68023810	68024210	-0.557507549	1.33E-01	same
chr16	53384681	53385081	-0.628138532	1.33E-01	same
chr2	15591793	15592193	-0.581815988	1.33E-01	same
chr17	64965375	64965775	-0.5950327	1.33E-01	same
chr15	83011606	83012006	-0.561817591	1.33E-01	same
chr1	45570215	45570615	-0.580017528	1.33E-01	same
chr9	97982374	97982774	-0.59076424	1.33E-01	same

chr15	101276665	101277065	-0.597575801	1.34E-01	same
chr12	109097231	109097631	-0.55901926	1.34E-01	same
chr5	158054523	158054923	-0.620968532	1.34E-01	same
chr10	78033593	78033993	-0.551876576	1.34E-01	same
chr17	7717307	7717707	-0.533393488	1.34E-01	same
chr6	157630571	157630971	0.591564523	1.34E-01	same
chr6	37711757	37712157	-0.620770896	1.34E-01	same
chr18	35673870	35674270	-0.607423968	1.35E-01	same
chr19	1207300	1207700	-0.522218253	1.35E-01	same
chr12	6778970	6779370	-0.464605147	1.35E-01	same
chr1	45508368	45508768	-0.540867067	1.35E-01	same
chr22	41597146	41597546	-0.540243735	1.35E-01	same
chr3	79019408	79019808	-0.551472332	1.35E-01	same
chr17	41667109	41667509	-0.559033387	1.35E-01	same
chr6	7339057	7339457	-0.615779612	1.35E-01	same
chr3	9843736	9844136	-0.50082563	1.35E-01	same
chr9	70418497	70418897	-0.54610966	1.35E-01	same
chr12	27710483	27710883	-0.590872713	1.35E-01	same
chrX	49155681	49156081	-0.575013412	1.35E-01	same
chr14_GL000225v1_random	21415	21815	0.615316928	1.35E-01	same
chr12	109154747	109155147	-0.580311037	1.35E-01	same
chrX	101347837	101348237	-0.575778905	1.36E-01	same
chr8	63241521	63241921	-0.616936267	1.36E-01	same
chr19	11482125	11482525	-0.564567236	1.36E-01	same
chr6	41936070	41936470	-0.581990309	1.36E-01	same
chr15	73636299	73636699	-0.549184636	1.36E-01	same
chr7	80783683	80784083	-0.58761637	1.36E-01	same
chr9	127981069	127981469	-0.591873489	1.36E-01	same
chr8	102807361	102807761	-0.548658004	1.36E-01	same
chr10	52314289	52314689	-0.550217512	1.36E-01	same
chr6	110981957	110982357	-0.536357961	1.37E-01	same
chr10	75402105	75402505	-0.516105181	1.37E-01	same
chr4	174012371	174012771	-0.559193991	1.37E-01	same
chr4	80307490	80307890	-0.609326198	1.37E-01	same
chr14	77457456	77457856	-0.478647308	1.37E-01	same
chr14	96888839	96889239	-0.618990964	1.37E-01	same
chr5	177607492	177607892	-0.487762384	1.37E-01	same
chr3	169242510	169242910	-0.586492749	1.37E-01	same
chr2	25877418	25877818	-0.624469195	1.37E-01	same
chr22	31749855	31750255	-0.522168605	1.37E-01	same
chr5	139439459	139439859	-0.572217997	1.38E-01	same
chr2	85546162	85546562	-0.471405754	1.38E-01	same
chr15	74872997	74873397	-0.478906189	1.38E-01	same
chr20	35954060	35954460	-0.527889693	1.38E-01	same
chr19	38618756	38619156	-0.484502684	1.38E-01	same
chr16	75464499	75464899	-0.55717303	1.38E-01	same
chr9	111630862	111631262	-0.523307688	1.38E-01	same
chr20	35883839	35884239	-0.535671737	1.38E-01	same
chr5	154445328	154445728	-0.536258957	1.38E-01	same
chr1	238629371	238629771	-0.62509266	1.38E-01	same
chr15	50423869	50424269	-0.566285226	1.38E-01	same
chr1	155140080	155140480	-0.527013831	1.39E-01	same
chr16	23149484	23149884	-0.606202443	1.39E-01	same
chrX	135344235	135344635	-0.521305362	1.39E-01	same
chr21	14216189	14216589	-0.588018297	1.39E-01	same
chr17	781977	782377	-0.551596831	1.39E-01	same
chr5	31531920	31532320	-0.552781177	1.39E-01	same
chr20	26010202	26010602	-0.557025669	1.39E-01	same
chr3	42804226	42804626	-0.483428138	1.39E-01	same
chr15	78437718	78438118	-0.568382791	1.39E-01	same
chr5	72955633	72956033	-0.612478944	1.39E-01	same
chr6	16761922	16762322	-0.569058903	1.39E-01	same
chr10	22316540	22316940	-0.538447578	1.40E-01	same
chrX	152830658	152831058	-0.497563503	1.40E-01	same
chr8	93700065	93700465	-0.599430639	1.40E-01	same
chr11	65859496	65859896	-0.402028352	1.40E-01	same
chr15	63157259	63157659	-0.585333224	1.40E-01	same
chr9	137297587	137297987	-0.624888942	1.40E-01	same
chr4	42657167	42657567	-0.548839749	1.40E-01	same
chr4	173879122	173879522	-0.611115422	1.40E-01	same
chr5	72308042	72308442	-0.529608998	1.40E-01	same
chr14	23953744	23954144	-0.556925915	1.40E-01	same
chr13	54573004	54573404	-0.607909511	1.40E-01	same
chr2	74221745	74222145	-0.550147718	1.40E-01	same
chr6	27375102	27375502	-0.353954744	1.41E-01	same
chr9	111482598	111482998	-0.553237804	1.41E-01	same
chr1	148402441	148402841	-0.596764816	1.41E-01	same

chr22	37608416	37608816	-0.543927088	1.41E-01	same
chr11	73761126	73761526	-0.58880144	1.41E-01	same
chr1	197146362	197146762	-0.561602062	1.41E-01	same
chr15	90934867	90935267	-0.414686677	1.41E-01	same
chr1	111507895	111508295	-0.53723467	1.41E-01	same
chr1	145425579	145425979	-0.481951771	1.41E-01	same
chr6	56955375	56955775	-0.575894963	1.41E-01	same
chr11	118435468	118435868	-0.478772875	1.42E-01	same
chr16	85011656	85012056	-0.560110324	1.42E-01	same
chr3	169966289	169966689	-0.549978151	1.42E-01	same
chr15	77910503	77910903	-0.478611956	1.42E-01	same
chr5	178212588	178212988	-0.462660285	1.42E-01	same
chr4	88823369	88823769	-0.561315739	1.42E-01	same
chr19	45039222	45039622	-0.563418112	1.42E-01	same
chr10	99520047	99520447	-0.502714857	1.42E-01	same
chr1	111140087	111140487	-0.511890464	1.43E-01	same
chr12	25692575	25692975	-0.595677719	1.43E-01	same
chr2	118014364	118014764	-0.587945962	1.43E-01	same
chr19	42302302	42302702	-0.48021148	1.43E-01	same
chr13	20773614	20774014	-0.521512251	1.43E-01	same
chr2	203014610	203015010	-0.483127442	1.43E-01	same
chr14	89955094	89955494	-0.560157137	1.43E-01	same
chr15	72784010	72784410	-0.547885901	1.44E-01	same
chr1	43596884	43597284	-0.53987407	1.44E-01	same
chr10	69123600	69124000	-0.424361852	1.44E-01	same
chr2	169584358	169584758	-0.59905352	1.44E-01	same
chr4	99088765	99089165	-0.62493195	1.44E-01	same
chr12	107093536	107093936	-0.511767035	1.44E-01	same
chr7	23105472	23105872	-0.547935044	1.44E-01	same
chr17	75601249	75601649	-0.510221709	1.44E-01	same
chr1	165698133	165698533	-0.538731136	1.44E-01	same
chr1	157445871	157446271	-0.609160036	1.44E-01	same
chr1	44819738	44820138	-0.460562208	1.45E-01	same
chr12	7960553	7960953	-0.602463295	1.45E-01	same
chr13	65926762	65927162	-0.563121951	1.45E-01	same
chr4	155675722	155676122	-0.571283846	1.45E-01	same
chr8	54101948	54102348	-0.591446826	1.45E-01	same
chr8	67062129	67062529	-0.490664599	1.45E-01	same
chr4	111806433	111806833	-0.617510191	1.46E-01	same
chr7	6577307	6577707	-0.541868625	1.46E-01	same
chr19	11587801	11588201	-0.602248637	1.46E-01	same
chr8	123072598	123072998	-0.536508564	1.46E-01	same
chr7	97117585	97117985	-0.599009802	1.46E-01	same
chrX	54440327	54440727	-0.572418445	1.46E-01	same
chr17	75827645	75828045	-0.442218684	1.46E-01	same
chr17	58658955	58659355	-0.491763604	1.46E-01	same
chr7	149005890	149006290	-0.475218279	1.46E-01	same
chr3	20064992	20065392	-0.595209514	1.46E-01	same
chr16	2603405	2603805	-0.494765738	1.46E-01	same
chr3	128879526	128879926	-0.530471781	1.47E-01	same
chr12	103929459	103929859	-0.557327913	1.47E-01	same
chr15	34101489	34101889	-0.608704403	1.47E-01	same
chr19	1051158	1051558	-0.57082546	1.47E-01	same
chr3	9422976	9423376	-0.605448249	1.47E-01	same
chr17	61863075	61863475	-0.499879555	1.47E-01	same
chrX	110229823	110230223	-0.615243794	1.48E-01	same
chr1	109213910	109214310	-0.556894785	1.48E-01	same
chr10	99732068	99732468	-0.507397228	1.48E-01	same
chr16	2009791	2010191	-0.542849994	1.48E-01	same
chr19	55476208	55476608	-0.604878596	1.48E-01	same
chr17	81918286	81918686	-0.536516276	1.48E-01	same
chr1	228842064	228842464	-0.515056267	1.48E-01	same
chr15	64841318	64841718	-0.573340579	1.48E-01	same
chr6	125946034	125946434	-0.595340409	1.48E-01	same
chr19	36528385	36528785	-0.567415469	1.48E-01	same
chr19	40810798	40811198	-0.560979721	1.48E-01	same
chr19	53879400	53879800	-0.519436157	1.48E-01	same
chr1	32520631	32521031	-0.537410464	1.49E-01	same
chr11	67630434	67630834	-0.528616265	1.49E-01	same
chr10	93554858	93555258	-0.591935203	1.49E-01	same
chr7	102748655	102749055	-0.495913201	1.49E-01	same
chr11	30322961	30323361	-0.539505804	1.49E-01	same
chr19	55447648	55448048	-0.59053603	1.49E-01	same
chr1	181158989	181159389	0.609042492	1.49E-01	same
chr19	45405199	45405599	-0.487227457	1.49E-01	same
chr2	71331771	71332171	-0.567580818	1.49E-01	same
chr5	96868793	96869193	-0.593155078	1.50E-01	same

chr17	81552784	81553184	-0.464845448	1.50E-01	same
chr5	102671335	102671735	-0.609415325	1.50E-01	same
chr7	149496354	149496754	-0.486452969	1.50E-01	same
chr19	45730520	45730920	-0.565130361	1.50E-01	same
chr2	60756540	60756940	-0.578343088	1.50E-01	same
chr2	32946403	32946803	-0.599175007	1.50E-01	same
chr16	30957209	30957609	-0.443498181	1.50E-01	same
chr19	49476304	49476704	-0.434924082	1.50E-01	same
chr1	150619844	150620244	-0.509233276	1.50E-01	same
chr9	97238404	97238804	-0.551585801	1.51E-01	same
chr5	87412602	87413002	-0.520210274	1.51E-01	same
chr9	132355173	132355573	-0.564171398	1.51E-01	same
chr22	38317824	38318224	-0.439459875	1.51E-01	same
chr16	3134619	3135019	-0.460120674	1.51E-01	same
chr13	93228165	93228565	-0.525231021	1.51E-01	same
chr4	184537221	184537621	-0.597794399	1.52E-01	same
chr22	30591891	30592291	-0.422133339	1.52E-01	same
chr3	183884604	183885004	-0.544437024	1.52E-01	same
chr9	127424131	127424531	-0.513606612	1.52E-01	same
chrX	41442261	41442661	-0.57474986	1.52E-01	same
chr19	35702323	35702723	-0.472822937	1.52E-01	same
chrX	78092337	78092737	-0.588654125	1.52E-01	same
chr10	79445791	79446191	-0.545450121	1.52E-01	same
chr10	100535569	100535969	-0.527807245	1.52E-01	same
chr11	96243304	96243704	-0.538860066	1.52E-01	same
chr21	37338839	37339239	-0.565335255	1.52E-01	same
chr6	154852871	154853271	-0.561396183	1.53E-01	same
chr14	74302712	74303112	-0.477385515	1.53E-01	same
chr12	111834657	111835057	-0.540797392	1.53E-01	same
chr21	45555862	45556262	-0.529411931	1.53E-01	same
chr7	11026272	11026672	-0.595693479	1.53E-01	same
chr1	147225184	147225584	-0.552378342	1.53E-01	same
chr16	56682014	56682414	-0.542940894	1.54E-01	same
chr21	32569589	32569989	-0.498751151	1.54E-01	same
chr22	50473277	50473677	-0.50413059	1.54E-01	same
chr3	9986591	9986991	-0.475862968	1.54E-01	same
chr4	163166913	163167313	-0.55930906	1.54E-01	same
chrX	77910174	77910574	-0.599490009	1.54E-01	same
chr11	65096130	65096530	-0.524969826	1.54E-01	same
chr6	123091346	123091746	-0.594466727	1.54E-01	same
chr6	97215205	97215605	-0.59294597	1.55E-01	same
chr6	27078778	27079178	-0.499866013	1.55E-01	same
chr9	2843779	2844179	-0.509990423	1.55E-01	same
chr3	10249292	10249692	-0.567327979	1.55E-01	same
chr17	44557114	44557514	-0.559490065	1.55E-01	same
chr15	64825681	64826081	-0.547829633	1.55E-01	same
chr13	33285470	33285870	-0.581608804	1.55E-01	same
chr2	11343089	11343489	-0.576186782	1.56E-01	same
chr5	41904014	41904414	-0.479629137	1.56E-01	same
chr17	10618241	10618641	-0.606307602	1.56E-01	same
chr20	25196131	25196531	-0.543777564	1.56E-01	same
chr20	38110151	38110551	-0.545417023	1.56E-01	same
chr2	10518755	10519155	0.581638291	1.56E-01	same
chr16	21300920	21301320	-0.535328894	1.56E-01	same
chr19	35644390	35644790	-0.423213743	1.56E-01	same
chr14	60164805	60165205	-0.556029359	1.56E-01	same
chr22	50507300	50507700	-0.524770715	1.57E-01	same
chr13	69828553	69828953	-0.523709263	1.57E-01	same
chr15	41332343	41332743	-0.488864192	1.57E-01	same
chr6	4135235	4135635	-0.547170097	1.57E-01	same
chr10	97401088	97401488	-0.495530431	1.57E-01	same
chr1	86774559	86774959	-0.572751208	1.57E-01	same
chr3	194003542	194003942	-0.546961835	1.57E-01	same
chr6	149545884	149546284	-0.499377704	1.57E-01	same
chrX	106611884	106612284	-0.498832851	1.57E-01	same
chr1	153986713	153987113	-0.48158409	1.57E-01	same
chr17	75625869	75626269	-0.480208818	1.57E-01	same
chr21	32306008	32306408	-0.506712987	1.58E-01	same
chr9	101533649	101534049	-0.501140983	1.58E-01	same
chr6	27721194	27721594	-0.365871363	1.58E-01	same
chr8	73878692	73879092	-0.52997841	1.58E-01	same
chr7	26187384	26187784	-0.424939645	1.58E-01	same
chr7	159166214	159166614	0.598862973	1.58E-01	same
chr14	77760837	77761237	-0.543135552	1.58E-01	same
chr1	47388717	47389117	-0.541835155	1.58E-01	same
chr16	30092304	30092704	-0.433834194	1.58E-01	same
chr8	140791217	140791617	-0.556210051	1.58E-01	same

chr9	136849313	136849713	-0.515151687	1.58E-01	same
chr7	123527790	123528190	-0.584368448	1.58E-01	same
chr5	134371026	134371426	-0.574377081	1.59E-01	same
chr19	18224064	18224464	-0.431468342	1.59E-01	same
chr12	54325057	54325457	-0.502917168	1.59E-01	same
chr18	46173746	46174146	-0.578873857	1.59E-01	same
chr7	66844600	66845000	-0.523576642	1.59E-01	same
chrX	16771963	16772363	-0.572898107	1.59E-01	same
chr19	51926792	51927192	-0.533493716	1.59E-01	same
chr16	2128308	2128708	-0.536503287	1.60E-01	same
chr4	118685272	118685672	-0.525562097	1.60E-01	same
chr9	94259079	94259479	-0.555152668	1.60E-01	same
chr11	76784134	76784534	-0.542720879	1.60E-01	same
chr1	145721277	145721677	-0.538967172	1.60E-01	same
chr14	59350055	59350455	-0.598868455	1.60E-01	same
chrX	47366105	47366505	-0.520572279	1.60E-01	same
chr20	33674836	33675236	-0.491046998	1.60E-01	same
chr13	92297570	92297970	0.581923229	1.61E-01	same
chr15	41543913	41544313	-0.535390451	1.61E-01	same
chr4	102501838	102502238	-0.546291037	1.61E-01	same
chr19	11089690	11090090	-0.483955894	1.61E-01	same
chr11	5684710	5685110	-0.58958922	1.61E-01	same
chr1	224224131	224224531	-0.54800873	1.61E-01	same
chr1	1511713	1512113	-0.570137436	1.61E-01	same
chrX	69616077	69616477	-0.58731542	1.61E-01	same
chr14	50559983	50560383	-0.53986638	1.61E-01	same
chr17	4909208	4909608	-0.532122453	1.61E-01	same
chr7	106285312	106285712	-0.566658916	1.61E-01	same
chr5	181163753	181164153	-0.294887296	1.61E-01	same
chr7	102363531	102363931	-0.538998719	1.62E-01	same
chr2	55617394	55617794	-0.474995036	1.62E-01	same
chr1	16709620	16710020	-0.510678757	1.62E-01	same
chr2	133293727	133294127	-0.597255812	1.62E-01	same
chr12	27022292	27022692	-0.504638362	1.62E-01	same
chr21	44010171	44010571	-0.546902031	1.63E-01	same
chr2	53767509	53767909	-0.547775825	1.63E-01	same
chr11	118689948	118690348	-0.445836796	1.63E-01	same
chr7	158695794	158696194	-0.540003871	1.63E-01	same
chrX	115957969	115958369	-0.553544895	1.63E-01	same
chr13	82275525	82275925	-0.566115383	1.63E-01	same
chr1	187073187	187073587	-0.568719684	1.63E-01	same
chr17	48879053	48879453	-0.551438363	1.63E-01	same
chr8	37694940	37695340	-0.552260807	1.63E-01	same
chr21	28885077	28885477	-0.509500404	1.63E-01	same
chr10	42475624	42476024	-0.58036178	1.64E-01	same
chr10	47894598	47894998	0.59075637	1.64E-01	same
chr17	50087606	50088006	-0.540329622	1.64E-01	same
chr2	63841108	63841508	-0.540110323	1.65E-01	same
chr15	74835364	74835764	-0.46637772	1.65E-01	same
chr1	86704408	86704808	-0.527583064	1.65E-01	same
chr12	55996853	55997253	-0.494296702	1.65E-01	same
chr2	191677452	191677852	-0.58572211	1.65E-01	same
chr12	51424721	51425121	-0.562681306	1.65E-01	same
chr1	149390208	149390608	-0.575355689	1.65E-01	same
chr8	66774674	66775074	-0.542503629	1.65E-01	same
chr20	47663874	47664274	-0.561809904	1.65E-01	same
chr1	9538974	9539374	-0.549391547	1.65E-01	same
chr9	35103192	35103592	-0.470664303	1.65E-01	same
chr2	202238376	202238776	-0.543785309	1.66E-01	same
chr17	7242038	7242438	-0.453258121	1.66E-01	same
chr15	89473026	89473426	-0.563074887	1.66E-01	same
chr12	53675857	53676257	-0.440130405	1.66E-01	same
chr2	75340605	75341005	-0.563818331	1.66E-01	same
chr14	73851755	73852155	-0.521993424	1.66E-01	same
chrX	72276942	72277342	-0.520230841	1.67E-01	same
chr11	119057300	119057700	-0.504701276	1.67E-01	same
chr5	61162105	61162505	-0.549634478	1.67E-01	same
chr17	40140005	40140405	-0.458624872	1.67E-01	same
chr15	59372611	59373011	-0.586681045	1.67E-01	same
chr3	52685939	52686339	-0.525488593	1.67E-01	same
chr6	116616056	116616456	-0.53203638	1.67E-01	same
chr9	124940720	124941120	-0.536819004	1.68E-01	same
chr21	31343654	31344054	-0.549329766	1.68E-01	same
chr21	42496377	42496777	-0.490954668	1.68E-01	same
chr1	212477290	212477690	-0.527462096	1.68E-01	same
chr6	134052260	134052660	-0.563455467	1.68E-01	same
chr5	64939849	64940249	-0.583603067	1.68E-01	same

chr9	128881695	128882095	-0.501588715	1.68E-01	same
chr10	31032058	31032458	-0.556601333	1.68E-01	same
chr14	23555971	23556371	-0.471683609	1.68E-01	same
chr9	26956240	26956640	-0.489118907	1.68E-01	same
chr11	60915191	60915591	-0.474088578	1.68E-01	same
chr8	93916337	93916737	-0.528948946	1.68E-01	same
chr18	59900574	59900974	-0.583483695	1.68E-01	same
chr19	6587028	6587428	-0.53562481	1.68E-01	same
chr19	11766654	11767054	-0.552058921	1.69E-01	same
chr22	19218356	19218756	-0.544381031	1.69E-01	same
chr16	29595375	29595775	-0.495561886	1.69E-01	same
chr15	45279159	45279559	-0.520393092	1.69E-01	same
chr2	135985343	135985743	-0.463826956	1.69E-01	same
chr4	119627866	119628266	-0.557661691	1.69E-01	same
chr1	36386103	36386503	-0.474514414	1.70E-01	same
chr1	83999076	83999476	-0.546741174	1.70E-01	same
chr3	49171160	49171560	-0.488999007	1.70E-01	same
chr6	108294821	108295221	-0.468198934	1.70E-01	same
chr10	113679555	113679955	-0.479259468	1.70E-01	same
chr13	40770991	40771391	-0.522390385	1.71E-01	same
chr7	29146516	29146916	-0.558573698	1.71E-01	same
chr6	33711488	33711888	-0.521515461	1.71E-01	same
chr22	24027235	24027635	-0.531343513	1.71E-01	same
chr20	63894669	63895069	-0.549311067	1.71E-01	same
chr1	56645793	56646193	-0.484107686	1.71E-01	same
chr5	150544603	150545003	-0.52873309	1.71E-01	same
chr19	12785280	12785680	-0.466315864	1.72E-01	same
chr12	53159458	53159858	-0.537937428	1.72E-01	same
chr19	45558433	45558833	-0.548792714	1.72E-01	same
chr20	17939132	17939532	-0.481450015	1.73E-01	same
chr15	77633565	77633965	-0.489466516	1.73E-01	same
chr21	33931208	33931608	-0.554134343	1.73E-01	same
chr12	1690572	1690972	-0.549744739	1.73E-01	same
chr12	6689240	6689640	-0.50595573	1.73E-01	same
chr5	124629080	124629480	-0.487541117	1.73E-01	same
chr15	91049785	91050185	-0.534221971	1.74E-01	same
chr22	47004082	47004482	0.521602052	1.74E-01	same
chr11	10540950	10541350	-0.49448656	1.74E-01	same
chr17	21214162	21214562	-0.501384032	1.74E-01	same
chr22	29908088	29908488	-0.526054437	1.74E-01	same
chr2	26033158	26033558	-0.500257433	1.74E-01	same
chr1	84298939	84299339	-0.536220192	1.75E-01	same
chr1	70354454	70354854	-0.405040507	1.75E-01	same
chr19	36114908	36115308	-0.39144189	1.75E-01	same
chr2	108448632	108449032	-0.51340218	1.75E-01	same
chr13	92763789	92764189	-0.558184002	1.75E-01	same
chr7	103074517	103074917	-0.566666287	1.75E-01	same
chr16	57244655	57245055	-0.529389337	1.75E-01	same
chr17	4881846	4882246	-0.50974048	1.75E-01	same
chr1	156689148	156689548	-0.491999161	1.76E-01	same
chr19	48881537	48881937	-0.539722687	1.76E-01	same
chr6	52420247	52420647	-0.484706354	1.76E-01	same
chr2	241702630	241703030	-0.512555825	1.76E-01	same
chr1	235104551	235104951	-0.434800487	1.76E-01	same
chr6	96571765	96572165	-0.575588524	1.76E-01	same
chr14	60091805	60092205	-0.449105999	1.76E-01	same
chr16	70687193	70687593	-0.485565023	1.76E-01	same
chr21	37563962	37564362	-0.510304774	1.76E-01	same
chr10	72173800	72174200	-0.519358428	1.77E-01	same
chr7	29370577	29370977	0.553183673	1.77E-01	same
chr19	18007494	18007894	-0.554860181	1.77E-01	same
chr1	168161345	168161745	0.556180381	1.77E-01	same
chr19	29605833	29606233	-0.532326601	1.77E-01	same
chr1	111449442	111449842	-0.486606217	1.78E-01	same
chr16	71461953	71462353	-0.53603884	1.78E-01	same
chr11	33802434	33802834	-0.53249901	1.78E-01	same
chr6	158644729	158645129	-0.435330536	1.78E-01	same
chr1	232538138	232538538	-0.503939248	1.78E-01	same
chr21	31559550	31559950	-0.45746038	1.78E-01	same
chr5	893608	894008	-0.521057845	1.78E-01	same
chr3	32177375	32177775	-0.536028121	1.79E-01	same
chr1	212791520	212791920	-0.495232153	1.79E-01	same
chr20	34112359	34112759	-0.52677053	1.79E-01	same
chrX	47190582	47190982	-0.434568229	1.79E-01	same
chr4	127965235	127965635	-0.446193898	1.79E-01	same
chr2	38742397	38742797	-0.50519705	1.79E-01	same
chr16	30941754	30942154	-0.457036164	1.79E-01	same

chr11	27506562	27506962	-0.48044224	1.79E-01	same
chr2	42133079	42133479	-0.523085164	1.80E-01	same
chr1	115147940	115148340	-0.571682083	1.80E-01	same
chr21	33404133	33404533	-0.512768758	1.80E-01	same
chr3	40477137	40477537	-0.537948978	1.80E-01	same
chr4	108650260	108650660	-0.496052159	1.80E-01	same
chr20	63658272	63658672	-0.454067971	1.80E-01	same
chr12	2004156	2004556	-0.512469082	1.80E-01	same
chr15	78909581	78909981	-0.510951911	1.80E-01	same
chr16	67935461	67935861	-0.455799561	1.80E-01	same
chrX	106726378	106726778	-0.540987635	1.80E-01	same
chr14	52706786	52707186	-0.481798938	1.80E-01	same
chr5	3483880	3484280	-0.53720185	1.81E-01	same
chr1	118185143	118185543	-0.535414169	1.81E-01	same
chr3	179147525	179147925	-0.545625912	1.81E-01	same
chr18	13726687	13727087	-0.550288981	1.81E-01	same
chr17	49707929	49708329	-0.460391835	1.81E-01	same
chr16	31442848	31443248	-0.501060216	1.81E-01	same
chr11	47259365	47259765	-0.492030784	1.82E-01	same
chr19	9309838	9310238	-0.516704631	1.82E-01	same
chr10	3173076	3173476	-0.507917039	1.82E-01	same
chr13	67136076	67136476	-0.403355564	1.83E-01	same
chr4	140373262	140373662	-0.499262141	1.83E-01	same
chr5	95646756	95647156	-0.500434571	1.83E-01	same
chr7	97024257	97024657	-0.532254741	1.83E-01	same
chr5	76818488	76818888	-0.548964675	1.83E-01	same
chr19	10942089	10942489	-0.558074187	1.84E-01	same
chr9	125261401	125261801	-0.50069921	1.84E-01	same
chr16	57388723	57389123	0.498293832	1.84E-01	same
chr12	12611800	12612200	-0.493725918	1.84E-01	same
chr12	120446158	120446558	-0.413002997	1.84E-01	same
chr22	50775118	50775682	-0.559085702	1.84E-01	same
chr2	128321509	128321909	-0.545634006	1.84E-01	same
chr8	106270919	106271319	-0.537671572	1.84E-01	same
chr2	174485886	174486286	-0.562904227	1.84E-01	same
chr9	128098046	128098775	-0.398613748	1.84E-01	same
chr1	155857051	155857451	-0.403428013	1.85E-01	same
chr12	120528882	120529282	-0.463645784	1.85E-01	same
chr1	152630090	152630490	-0.554446938	1.85E-01	same
chr4	133151135	133151535	-0.470054865	1.85E-01	same
chr11	95068393	95068793	-0.462579417	1.85E-01	same
chr12	6723959	6724359	-0.453390581	1.85E-01	same
chr9	93453020	93453420	-0.495717091	1.85E-01	same
chr15	92890910	92891310	-0.564767169	1.85E-01	same
chrX	48476113	48476513	-0.514194298	1.85E-01	same
chr4	13483283	13483683	-0.48404938	1.86E-01	same
chr6	27809912	27810312	-0.367558074	1.86E-01	same
chr6	136989625	136990025	-0.537052047	1.86E-01	same
chr1	151198086	151198486	-0.471275587	1.86E-01	same
chr17	35587228	35587628	-0.509865513	1.86E-01	same
chr1_KI270764v1_alt	45209	45609	-0.546345726	1.86E-01	same
chr12	109477381	109477781	-0.49670048	1.86E-01	same
chr6	144423119	144423519	-0.53946608	1.86E-01	same
chr9	36257777	36258177	-0.518207924	1.86E-01	same
chrX	140764560	140764960	-0.546013913	1.86E-01	same
chr3	138935812	138936212	-0.523630724	1.86E-01	same
chr8	122673603	122674003	-0.556537615	1.86E-01	same
chr17	63550140	63550540	-0.418703168	1.87E-01	same
chr3	98593703	98594103	-0.489951184	1.87E-01	same
chr16	75445504	75445904	-0.535658054	1.87E-01	same
chr7	100156937	100157337	-0.505707554	1.87E-01	same
chr10	100267597	100267997	-0.520997347	1.87E-01	same
chr8	109539200	109539600	-0.54179596	1.87E-01	same
chr2	20351043	20351443	-0.512273195	1.88E-01	same
chr3	142595964	142596364	-0.51172145	1.88E-01	same
chr2	206230373	206230773	-0.523978528	1.88E-01	same
chr12	13098539	13099313	-0.467700919	1.88E-01	same
chr14	73137063	73137463	-0.516529637	1.88E-01	same
chr7	73587629	73588029	-0.516653938	1.88E-01	same
chr3	99083218	99083618	-0.522455251	1.88E-01	same
chr10	122267923	122268323	-0.5425492	1.88E-01	same
chr2	27409748	27410148	-0.510876994	1.88E-01	same
chr7	101814997	101815397	-0.551677052	1.88E-01	same
chr13	80132804	80133204	-0.482350139	1.88E-01	same
chr1	222618986	222619386	-0.484293323	1.88E-01	same
chr3	132659390	132659790	-0.484778714	1.88E-01	same
chr10	100743682	100744082	-0.509823516	1.88E-01	same

chr12	9447944	9448344	-0.561204573	1.89E-01	same
chr17	75267496	75267896	-0.448415637	1.89E-01	same
chr7	142019788	142020188	-0.56166256	1.89E-01	same
chr15	64093903	64094303	-0.486371685	1.89E-01	same
chr11	59810401	59810801	-0.471037627	1.89E-01	same
chr6	87589969	87590369	-0.478222839	1.89E-01	same
chrX	136909535	136909935	-0.400594486	1.89E-01	same
chr4	72593770	72594170	0.561502167	1.89E-01	same
chr6	26234671	26235071	-0.44214661	1.89E-01	same
chr19	11345847	11346247	-0.506334454	1.89E-01	same
chr10	102394115	102394515	-0.455630029	1.90E-01	same
chr6	43020921	43021321	-0.496116448	1.90E-01	same
chr3	128051864	128052264	-0.486260185	1.90E-01	same
chr13	60396467	60396867	-0.453940585	1.90E-01	same
chr9	112717753	112718153	-0.456249117	1.91E-01	same
chr6	121334296	121334696	-0.454920628	1.91E-01	same
chrX	107628215	107628615	-0.402029713	1.91E-01	same
chr11	50412399	50412799	0.523388985	1.91E-01	same
chrX	77494210	77494610	-0.518095713	1.91E-01	same
chr9	131178177	131178577	-0.489770653	1.91E-01	same
chr12	68746123	68746523	-0.407371671	1.92E-01	same
chr3	128123600	128124000	-0.463400621	1.92E-01	same
chr19	21082018	21082418	-0.483882507	1.92E-01	same
chr2	3558385	3558785	-0.458737964	1.92E-01	same
chr6	10403786	10404186	-0.537079948	1.92E-01	same
chr1	996967	997367	-0.477141469	1.93E-01	same
chr7	105522260	105522660	-0.533946382	1.93E-01	same
chr10	90930661	90931061	-0.519711554	1.93E-01	same
chr3	48918390	48918790	-0.472368539	1.93E-01	same
chr22	46420123	46420523	0.502735299	1.93E-01	same
chr9	121074975	121075375	-0.4851059	1.93E-01	same
chr1	938728	939128	-0.470764021	1.93E-01	same
chr19	18590206	18590606	-0.445389807	1.93E-01	same
chrX	49101227	49101627	-0.463549388	1.93E-01	same
chr3	175270412	175270812	-0.535327838	1.93E-01	same
chr6	106325378	106325778	-0.449619475	1.93E-01	same
chr9	133030533	133030933	-0.473941081	1.93E-01	same
chr4	139666048	139666448	-0.514557463	1.93E-01	same
chr7	27606322	27606722	-0.506126278	1.94E-01	same
chr14	23006800	23007200	-0.40811087	1.94E-01	same
chr7	100705517	100705917	-0.424382651	1.94E-01	same
chr7	30478235	30478635	-0.526324376	1.94E-01	same
chr2	172555541	172555941	-0.525250481	1.94E-01	same
chr4	110411460	110411860	-0.48138998	1.95E-01	same
chr3	172750569	172750969	-0.471174632	1.95E-01	same
chr10	15850573	15850973	-0.519232229	1.95E-01	same
chr7	93232020	93232420	-0.457648988	1.95E-01	same
chr8	63039216	63039616	-0.5280434	1.95E-01	same
chr10	52707428	52707828	-0.53809567	1.95E-01	same
chr6	168147124	168147524	-0.507003805	1.95E-01	same
chr19	18416572	18416972	-0.52711077	1.95E-01	same
chr9	101398268	101398668	-0.507853448	1.95E-01	same
chr3	149086254	149086654	-0.450479182	1.95E-01	same
chr11	66258031	66258431	-0.463349427	1.95E-01	same
chr19	1479223	1479623	-0.458745255	1.96E-01	same
chr7	44559970	44560370	-0.490568342	1.96E-01	same
chr2	127526399	127526799	-0.468666007	1.96E-01	same
chr11	78416528	78416928	-0.516786661	1.96E-01	same
chrX	155026564	155026964	-0.488793804	1.96E-01	same
chr20	50460544	50460944	-0.50931269	1.96E-01	same
chr1	28437899	28438299	-0.50854869	1.96E-01	same
chr19	43596052	43596452	-0.395240012	1.96E-01	same
chr9	104747847	104748247	-0.521906663	1.96E-01	same
chr5	131312361	131312761	-0.50216281	1.96E-01	same
chr6	26522477	26522877	-0.369949517	1.97E-01	same
chr17	44502946	44503346	-0.50255057	1.97E-01	same
chr2	71277660	71278060	-0.486479343	1.97E-01	same
chr8	104223634	104224034	-0.537867464	1.97E-01	same
chr14	101810060	101810460	-0.484696123	1.97E-01	same
chr2	218979855	218980255	-0.507538808	1.97E-01	same
chr12	48157395	48157795	-0.418034969	1.97E-01	same
chr15	92831709	92832109	-0.434789764	1.97E-01	same
chr19	24033416	24033816	-0.477927829	1.97E-01	same
chr10	75508800	75509200	-0.525308699	1.98E-01	same
chr17	62423769	62424169	-0.473170429	1.98E-01	same
chr6	38974931	38975331	-0.53559758	1.98E-01	same
chr15	89088051	89088451	-0.36041071	1.98E-01	same

chr22	21713025	21713425	-0.459712212	1.98E-01	same
chr14_GL000225v1_random	61830	62230	0.536144436	1.98E-01	same
chr12	92146063	92146463	-0.487769317	1.98E-01	same
chr5	1570551	1570951	-0.516165173	1.99E-01	same
chr10	23438541	23438941	-0.540093599	1.99E-01	same
chr1	10033135	10033535	-0.453530507	1.99E-01	same
chr8	37849579	37849979	-0.531731814	1.99E-01	same
chr10	24724177	24724577	-0.514176443	2.00E-01	same
chr2	84925904	84926304	-0.490578782	2.00E-01	same
chr10	133209160	133209560	-0.511158527	2.00E-01	same
chr16	4957739	4958139	-0.461019299	2.00E-01	same
chr3	99817717	99818117	-0.495789296	2.00E-01	same
chr4	153204697	153205097	-0.514498168	2.00E-01	same
chr6	12044605	12045005	-0.547755794	2.00E-01	same
chr12	128824058	128824458	-0.513876391	2.00E-01	same
chr15	26051622	26052022	-0.542170816	2.00E-01	same
chr12	990518	990918	-0.532539458	2.01E-01	same
chr12	30851368	30851768	-0.542724772	2.01E-01	same
chr12	131895888	131896288	-0.448322842	2.01E-01	same
chr7	116524557	116524957	-0.521384812	2.01E-01	same
chr16	29805173	29805573	-0.395261342	2.01E-01	same
chr11	65961291	65961691	-0.471265953	2.01E-01	same
chr8	88327760	88328160	-0.522915175	2.01E-01	same
chr3	8589857	8590257	-0.502215976	2.01E-01	same
chr5	112861221	112861621	-0.478934383	2.02E-01	same
chr19	34733704	34734104	-0.487066564	2.02E-01	same
chr10	128126571	128126971	-0.537818402	2.02E-01	same
chr18	30151632	30152032	-0.539097444	2.02E-01	same
chr7	151341202	151341602	-0.442275062	2.02E-01	same
chr4	173369571	173369971	-0.46574754	2.03E-01	same
chr8	28890010	28890410	-0.445592704	2.03E-01	same
chr17	17860070	17860470	-0.490833677	2.03E-01	same
chr1	192809423	192809823	-0.50300421	2.03E-01	same
chr12	93442339	93442739	-0.496008242	2.03E-01	same
chr12	38904957	38905357	-0.496857513	2.03E-01	same
chr1	155167486	155167886	-0.477872697	2.03E-01	same
chr8	86508492	86508892	-0.442269944	2.04E-01	same
chr4	95044407	95044807	-0.528777588	2.04E-01	same
chr4	3746279	3746679	-0.540144011	2.04E-01	same
chr14	75295929	75296329	-0.510809019	2.04E-01	same
chr12	122356250	122356650	-0.500192116	2.04E-01	same
chr19	10864576	10864976	-0.494060774	2.05E-01	same
chr7	116218036	116218436	-0.489096162	2.05E-01	same
chr19	11694954	11695354	-0.464515705	2.05E-01	same
chr11	2433932	2434332	0.481408822	2.05E-01	same
chr17	47970834	47971234	-0.442131157	2.05E-01	same
chr16	29926314	29926714	-0.444903942	2.05E-01	same
chr15	73376473	73376873	-0.529256085	2.05E-01	same
chr2	171922290	171922690	-0.477698515	2.05E-01	same
chr4	48830919	48831319	-0.503680182	2.06E-01	same
chr22	39319932	39320332	-0.441868829	2.06E-01	same
chr20	25310497	25310897	-0.539086837	2.06E-01	same
chr16	30113197	30113597	-0.429699634	2.06E-01	same
chr15	78565066	78565466	-0.445808881	2.06E-01	same
chr13	35762602	35763002	-0.526868053	2.06E-01	same
chr17	48830928	48831328	-0.450348288	2.07E-01	same
chr10	46106659	46107059	-0.5168473	2.07E-01	same
chr19	47035874	47036274	-0.463473026	2.07E-01	same
chr7	55951628	55952028	-0.474747191	2.07E-01	same
chr3	20185909	20186309	-0.488801997	2.07E-01	same
chr3	169812691	169813091	-0.495180895	2.07E-01	same
chr7	154575107	154575507	0.53800328	2.08E-01	same
chr11	62706309	62706709	-0.440850112	2.08E-01	same
chr1	35193212	35193612	-0.40778548	2.08E-01	same
chr2	207711034	207711434	-0.448822661	2.08E-01	same
chr14	81533599	81533999	-0.500233424	2.08E-01	same
chr22	29553491	29553891	-0.417182343	2.09E-01	same
chr1	160262100	160262500	-0.392227892	2.09E-01	same
chr12	56752017	56752417	-0.495922713	2.09E-01	same
chr9	131383037	131383437	-0.474881074	2.09E-01	same
chr5	32174322	32174722	-0.465607193	2.09E-01	same
chr12	122266343	122266743	-0.395952116	2.09E-01	same
chr17_KI270729v1_random	2833	3233	0.36552929	2.10E-01	same
chr17	75774736	75775136	-0.406156355	2.10E-01	same
chr7	141014794	141015194	-0.401245539	2.10E-01	same
chr16	67841916	67842316	-0.417476301	2.11E-01	same
chr5	131170984	131171384	-0.444914489	2.11E-01	same

chr8	100150276	100150676	-0.478811836	2.11E-01	same
chr8	53842888	53843288	-0.458229292	2.11E-01	same
chr3	15860097	15860497	-0.527681226	2.12E-01	same
chr1	230978851	230979251	-0.409302939	2.12E-01	same
chr15	45401973	45402373	-0.481546936	2.12E-01	same
chr6	13709800	13710200	-0.518764998	2.12E-01	same
chr16	71882884	71883284	-0.523747226	2.13E-01	same
chr9	95567656	95568056	0.515646848	2.13E-01	same
chr7	151736415	151736815	-0.47724583	2.13E-01	same
chr18	79777116	79777516	-0.528967878	2.13E-01	same
chr4	86935703	86936103	-0.465646001	2.13E-01	same
chrX	46903613	46904013	-0.508231602	2.13E-01	same
chr1	86914080	86914480	-0.410663642	2.13E-01	same
chr7	140696636	140697036	-0.381800639	2.13E-01	same
chr17	9576824	9577224	-0.50934061	2.13E-01	same
chr10	102449469	102449869	-0.480160739	2.14E-01	same
chr13	68257198	68257598	-0.399999021	2.14E-01	same
chr11	14520120	14520520	-0.386366723	2.14E-01	same
chr14	77097815	77098215	-0.466080048	2.14E-01	same
chr10	97736886	97737286	-0.456869705	2.15E-01	same
chr2	118185958	118186358	-0.510502877	2.15E-01	same
chr5	144170436	144170836	-0.483085236	2.15E-01	same
chr10	72260517	72260917	-0.451940266	2.15E-01	same
chr4	116274665	116275065	0.527250617	2.16E-01	same
chr3	58306183	58306583	-0.448593386	2.16E-01	same
chr2	158973480	158973880	-0.47661875	2.16E-01	same
chr1	19210559	19210959	-0.494010866	2.16E-01	same
chr15	66862776	66863176	-0.465906915	2.16E-01	same
chr10	101442114	101442514	-0.473173277	2.16E-01	same
chr1	86915090	86915490	-0.469007926	2.17E-01	same
chr2	238098288	238098688	-0.418389357	2.17E-01	same
chr17	82613312	82613712	-0.488119656	2.17E-01	same
chrX	155216104	155216504	-0.471622483	2.17E-01	same
chr6	1667053	1667453	-0.461268573	2.17E-01	same
chr1	39026075	39026475	-0.449507158	2.17E-01	same
chrX	48003729	48004129	-0.508052735	2.18E-01	same
chr12	128333444	128333844	0.523748036	2.18E-01	same
chr15	98554481	98554881	-0.486889789	2.18E-01	same
chr1	244439294	244439694	-0.427412409	2.18E-01	same
chr1	92080317	92080717	-0.433705722	2.19E-01	same
chr1	228500994	228501394	-0.368318395	2.19E-01	same
chr19	48872802	48873202	-0.434327937	2.19E-01	same
chr12	53321300	53321700	-0.40790731	2.19E-01	same
chr5	58582612	58583325	-0.455755352	2.19E-01	same
chr22	37643159	37643559	-0.403920303	2.19E-01	same
chr1	44793569	44793969	-0.395362879	2.20E-01	same
chr1	147670019	147670419	-0.432519237	2.20E-01	same
chr7	64903153	64903553	-0.503112969	2.20E-01	same
chr5	160399720	160400120	-0.489713868	2.20E-01	same
chr1_KI270713v1_random	28583	28983	-0.350732197	2.20E-01	same
chr4	124712977	124713377	-0.499390946	2.20E-01	same
chr22	37199145	37199545	-0.431674717	2.21E-01	same
chr1	151175852	151176252	-0.470044661	2.21E-01	same
chr2	63840590	63840990	-0.459327477	2.21E-01	same
chr5	147946603	147947003	-0.503744399	2.21E-01	same
chr6	1872650	1873050	-0.493410096	2.21E-01	same
chr16	46831513	46831913	-0.495206612	2.21E-01	same
chr5	60945478	60945878	-0.458401286	2.22E-01	same
chr19	19430572	19430972	-0.47471939	2.22E-01	same
chr1	44986401	44986801	-0.454539235	2.22E-01	same
chr4	2843122	2843522	-0.510580809	2.22E-01	same
chr5	134586985	134587385	-0.449598426	2.22E-01	same
chr11	85628108	85628508	-0.406230431	2.22E-01	same
chr16	11916169	11916569	-0.465241824	2.23E-01	same
chr10	118341985	118342385	-0.46313416	2.23E-01	same
chr2	65662445	65662845	-0.483452846	2.23E-01	same
chr3	48188394	48188794	-0.418774438	2.23E-01	same
chr4	169010435	169010835	-0.493006162	2.23E-01	same
chr8	124372700	124373100	-0.467456736	2.23E-01	same
chr1	220090019	220090419	-0.456752571	2.24E-01	same
chr19	58182987	58183387	-0.516142695	2.24E-01	same
chr16	77722439	77722839	-0.501768293	2.24E-01	same
chr2	234156399	234156799	-0.493940422	2.24E-01	same
chr14_GL000225v1_random	19506	19906	0.512192943	2.24E-01	same
chr1	179081637	179082037	-0.423782263	2.25E-01	same
chr14	67240752	67241152	-0.500749657	2.25E-01	same
chr8	81732588	81732988	-0.493529069	2.25E-01	same

chr2	47403082	47403482	-0.422194667	2.25E-01	same
chr8	41529031	41529431	-0.480055232	2.25E-01	same
chr3	112990859	112991259	-0.449729307	2.26E-01	same
chr9	4297492	4297892	-0.498559429	2.26E-01	same
chr4	80335774	80336174	-0.489149969	2.26E-01	same
chr2	11744289	11744689	-0.496835258	2.26E-01	same
chr3	49104479	49104879	-0.41515344	2.26E-01	same
chr15	49155131	49155531	-0.474063577	2.26E-01	same
chr22	25110734	25111134	-0.433508463	2.27E-01	same
chr6	89819343	89819743	-0.447452497	2.27E-01	same
chr3	38137177	38137577	-0.413289273	2.27E-01	same
chr19	39341690	39342090	-0.441082407	2.27E-01	same
chr14	94889710	94890110	0.482548372	2.27E-01	same
chrX	153226346	153226746	-0.490361974	2.27E-01	same
chr12	48001241	48001966	-0.434873705	2.27E-01	same
chrX	119468487	119468887	-0.455201533	2.27E-01	same
chr17	57988629	57989029	-0.434334751	2.27E-01	same
chr3	195907941	195908341	-0.476634534	2.27E-01	same
chr3	72875383	72875783	-0.488922295	2.28E-01	same
chr6	70566737	70567137	-0.461780392	2.28E-01	same
chr15	82809084	82809484	-0.450170057	2.28E-01	same
chr19	10701426	10701826	-0.406906395	2.28E-01	same
chr3	113745236	113745636	-0.423223887	2.28E-01	same
chr17	34961153	34961553	-0.506900864	2.29E-01	same
chr7	93591139	93591539	-0.490154804	2.29E-01	same
chr16	29995749	29996149	-0.398977335	2.29E-01	same
chr4	2962134	2962534	-0.380692426	2.30E-01	same
chr4	174225467	174225867	0.490991805	2.30E-01	same
chr19	40404059	40404459	-0.450143107	2.30E-01	same
chr17	16653496	16653896	-0.478528403	2.30E-01	same
chr14	81170346	81170746	-0.424527502	2.30E-01	same
chr7	112150914	112151314	-0.475471153	2.31E-01	same
chr22	46036365	46036765	-0.444061293	2.31E-01	same
chr14	60981062	60981462	-0.446010426	2.31E-01	same
chr5	138212809	138213209	-0.477479594	2.31E-01	same
chrX	100636470	100636870	-0.455453516	2.31E-01	same
chr7	97872177	97872577	-0.405543199	2.31E-01	same
chr3	51637503	51637903	-0.490872656	2.31E-01	same
chr12	110613751	110614151	-0.447801512	2.31E-01	same
chr19	3700555	3700955	-0.451990736	2.31E-01	same
chr13	52617082	52617482	-0.435806996	2.31E-01	same
chr9	129103167	129103567	-0.420700456	2.31E-01	same
chr21	36567267	36567667	-0.461711051	2.32E-01	same
chr7	23531901	23532301	-0.419773749	2.32E-01	same
chr20	56392243	56392643	-0.450094987	2.32E-01	same
chr15	59105086	59105486	-0.467960175	2.33E-01	same
chr12	21437850	21438250	-0.468797561	2.33E-01	same
chr1	230268674	230269074	-0.416006385	2.33E-01	same
chr13	62944983	62945383	0.467176733	2.33E-01	same
chr3	47788847	47789247	-0.463386105	2.33E-01	same
chr10	68600110	68600510	-0.478793361	2.33E-01	same
chr11	78574754	78575154	-0.429632119	2.34E-01	same
chr6	165619222	165619622	-0.467156406	2.34E-01	same
chr1	99836204	99836604	0.507440685	2.34E-01	same
chr5	133051502	133051902	-0.467469649	2.35E-01	same
chr19	51339361	51339761	-0.469110629	2.35E-01	same
chr2	3377364	3377764	-0.463460788	2.35E-01	same
chr11	124800471	124800871	-0.473500847	2.35E-01	same
chr6	27272652	27273052	-0.438373874	2.35E-01	same
chr15	72376091	72376491	-0.445552913	2.35E-01	same
chr1	222546806	222547206	-0.462248277	2.35E-01	same
chr1	204417352	204417752	-0.402213451	2.36E-01	same
chr1	224329940	224330340	-0.32778078	2.36E-01	same
chr19	38831403	38831803	-0.390021256	2.36E-01	same
chr22	43945973	43946373	-0.432606134	2.36E-01	same
chr5	118988585	118988985	-0.472153827	2.37E-01	same
chr3	127074903	127075303	-0.439481979	2.37E-01	same
chr5	124748558	124748958	-0.469138702	2.37E-01	same
chr1	94236276	94236676	-0.463576868	2.37E-01	same
chr8	37736180	37736580	-0.492712503	2.38E-01	same
chr7	148976136	148976536	-0.417772083	2.38E-01	same
chr1	171742077	171742477	-0.458245713	2.38E-01	same
chr1	92960445	92960845	-0.444984479	2.38E-01	same
chr7	146345470	146345870	-0.488410986	2.39E-01	same
chr15	45587133	45587533	-0.474482135	2.39E-01	same
chr16	1827153	1827553	-0.431287741	2.39E-01	same
chr5	119299599	119299999	-0.48797781	2.39E-01	same

chr1	204398397	204398797	-0.451714969	2.39E-01	same
chr17	39461541	39461941	-0.430470055	2.39E-01	same
chr22_KI270736v1_random	157219	157619	0.49898066	2.39E-01	same
chr22	36409871	36410271	-0.426368963	2.40E-01	same
chr1	75375948	75376348	-0.4499618	2.40E-01	same
chr19	44051634	44052034	-0.465956762	2.40E-01	same
chr21	41783336	41783736	-0.466674604	2.40E-01	same
chr3	132228957	132229357	-0.283248559	2.40E-01	same
chr1	180502558	180502958	-0.417556802	2.41E-01	same
chrX	71283365	71283765	-0.438790556	2.41E-01	same
chr17	74514072	74514472	-0.495680873	2.41E-01	same
chr12	27969181	27969581	-0.482210314	2.41E-01	same
chrX	100820128	100820528	-0.410031421	2.41E-01	same
chr10	47340443	47340843	-0.480981773	2.41E-01	same
chr2	70871594	70871994	-0.458395327	2.42E-01	same
chrX	76172890	76173290	-0.43587259	2.42E-01	same
chr3	94179023	94179423	-0.453077492	2.42E-01	same
chr8	73489982	73490382	-0.493090772	2.42E-01	same
chr7	105581208	105581608	-0.418996471	2.43E-01	same
chr12	67068066	67068466	-0.469073795	2.43E-01	same
chr2	128063493	128063893	-0.485931009	2.44E-01	same
chr1	241306194	241306594	-0.395903912	2.44E-01	same
chr12	7188648	7189048	-0.465530529	2.44E-01	same
chr3	25790050	25790450	-0.387949073	2.44E-01	same
chr6	10521197	10521597	-0.446465494	2.44E-01	same
chr10	99430425	99430825	-0.441285066	2.45E-01	same
chr18	35583113	35583513	-0.447102245	2.45E-01	same
chr3	49469712	49470112	-0.430465598	2.45E-01	same
chr2	27628616	27629016	-0.423900282	2.46E-01	same
chr2	100291573	100291973	0.489466445	2.46E-01	same
chr2	64453392	64453792	-0.488345576	2.46E-01	same
chr13	81956783	81957183	0.435889756	2.46E-01	same
chr3	131026293	131026693	-0.450553926	2.46E-01	same
chr12	53727521	53727921	-0.427249706	2.46E-01	same
chr17	28661675	28662075	-0.427480201	2.47E-01	same
chr20	31613027	31613427	-0.452180904	2.47E-01	same
chr17	75861282	75861682	-0.416215919	2.47E-01	same
chr7	72413593	72413993	0.494472071	2.47E-01	same
chr4	167234165	167234565	-0.470654064	2.47E-01	same
chr2	219597919	219598319	-0.42700649	2.47E-01	same
chr12	112012894	112013294	-0.409139346	2.47E-01	same
chr11	59680447	59680847	-0.457277654	2.48E-01	same
chr9	128684590	128684990	-0.402174786	2.48E-01	same
chr17	64506520	64506920	-0.352068784	2.49E-01	same
chr3	172710867	172711267	-0.433079349	2.49E-01	same
chr10	120128112	120128512	-0.47457087	2.49E-01	same
chr6	118800633	118801033	-0.488919834	2.50E-01	same
chr3	15098773	15099173	-0.430248777	2.50E-01	same
chr7	155205317	155205717	-0.392977941	2.50E-01	same
chr11	96307057	96307457	-0.425239395	2.50E-01	same
chr9	17268586	17268986	-0.479924056	2.50E-01	same
chr11	7665727	7666127	-0.487917363	2.50E-01	same
chr9	95312984	95313384	-0.44371669	2.50E-01	same
chr2	105398672	105399072	-0.470939267	2.50E-01	same
chr7	127585443	127585843	-0.457662114	2.51E-01	same
chr2	77567719	77568119	-0.476694588	2.51E-01	same
chr1	176058682	176059082	-0.479732897	2.51E-01	same
chr12	116256940	116257340	-0.486653693	2.51E-01	same
chr12	122031492	122031892	-0.488376907	2.51E-01	same
chr3	142578446	142578846	-0.44028476	2.51E-01	same
chr8	61714742	61715142	-0.448599049	2.52E-01	same
chrX	85003618	85004018	-0.440419933	2.52E-01	same
chr11	64111674	64112074	-0.441990014	2.52E-01	same
chr16	66961057	66961457	-0.40551967	2.53E-01	same
chr13	27426017	27426417	-0.470532282	2.53E-01	same
chr15	98000926	98001326	-0.477074794	2.53E-01	same
chr7	45111656	45112056	-0.404529974	2.53E-01	same
chr19	49659728	49660128	-0.384921647	2.54E-01	same
chr18	30192164	30192564	0.459000419	2.54E-01	same
chr19	43028079	43028479	-0.422923912	2.54E-01	same
chrX	49184402	49184802	-0.402426641	2.54E-01	same
chr19	35643541	35643941	-0.402610787	2.54E-01	same
chr3	25428261	25428661	-0.45803123	2.55E-01	same
chr13	65472406	65472806	-0.435319702	2.55E-01	same
chr3	49719424	49719824	-0.340926086	2.55E-01	same
chr15	64093212	64093612	-0.469796844	2.55E-01	same
chr17	44708735	44709135	-0.4361139	2.55E-01	same

chr17	82154267	82154667	-0.398030315	2.55E-01	same
chr12	4538317	4538717	-0.429484806	2.55E-01	same
chr6	47034901	47035301	-0.480277013	2.55E-01	same
chr9	2242028	2242428	-0.456504703	2.56E-01	same
chr1	40691663	40692458	-0.371725537	2.56E-01	same
chr1	19596589	19596989	-0.465965603	2.56E-01	same
chr17	41737823	41738223	-0.404118235	2.56E-01	same
chr6	57870184	57870584	-0.397379271	2.56E-01	same
chr11	107017321	107017721	-0.479360384	2.56E-01	same
chr19	40090622	40091022	-0.448781646	2.56E-01	same
chr20	32665238	32665638	-0.435400373	2.57E-01	same
chrX	111649175	111649575	0.46028776	2.57E-01	same
chr12	108561948	108562348	-0.41285749	2.57E-01	same
chr2	199955251	199955651	-0.426958241	2.57E-01	same
chr18	812544	812944	-0.473583835	2.58E-01	same
chr19	14689757	14690157	-0.445548654	2.58E-01	same
chr13	69638906	69639306	-0.469454737	2.58E-01	same
chr9	130693643	130694043	-0.405489319	2.58E-01	same
chr2	222299028	222299428	-0.468311088	2.58E-01	same
chr16	67481003	67481403	-0.367417144	2.58E-01	same
chr16	56451332	56451732	-0.355534055	2.58E-01	same
chr7	87627803	87628203	-0.440423356	2.59E-01	same
chr21	38989817	38990217	-0.443610252	2.59E-01	same
chr1	190474908	190475308	-0.449764219	2.59E-01	same
chr4	89753493	89753893	-0.472772534	2.59E-01	same
chr19_KI270866v1_alt	41818	42218	-0.47141914	2.60E-01	same
chr6	142845865	142846265	-0.440113826	2.60E-01	same
chr1	163321821	163322221	-0.417155111	2.60E-01	same
chr8	48284026	48284426	-0.444132318	2.60E-01	same
chr19	47241955	47242355	-0.429913671	2.60E-01	same
chr3	50227521	50227921	-0.423073372	2.60E-01	same
chr6	164913084	164913484	0.479289997	2.60E-01	same
chr2	26012085	26012485	-0.44398518	2.61E-01	same
chr12	78863954	78864354	-0.455639736	2.61E-01	same
chr5	168486766	168487166	-0.478691668	2.61E-01	same
chr6	96924612	96925012	-0.44420102	2.61E-01	same
chr15	43122832	43123232	-0.451826146	2.62E-01	same
chrX	135973600	135974000	-0.414692719	2.62E-01	same
chr16	2868127	2868527	-0.416403849	2.62E-01	same
chr5	164188581	164188981	-0.42509465	2.62E-01	same
chr4	147617337	147617737	-0.431499147	2.62E-01	same
chr13	63427318	63427718	-0.411828851	2.62E-01	same
chr19	17932602	17933002	-0.402551388	2.62E-01	same
chr13	79325152	79325552	0.462593293	2.62E-01	same
chr8	22561686	22562086	-0.407969468	2.63E-01	same
chr18	23453014	23453414	-0.35979848	2.63E-01	same
chr22	19761950	19762350	-0.413244452	2.63E-01	same
chr19	6412173	6412573	-0.384115087	2.63E-01	same
chr6	149060485	149060885	-0.446066952	2.63E-01	same
chr9	93649873	93650273	0.46671861	2.63E-01	same
chr19	38976410	38976810	-0.418817447	2.64E-01	same
chr19	42296073	42296473	-0.39433433	2.64E-01	same
chr11	115504576	115504976	-0.444587179	2.64E-01	same
chr1	248890642	248891042	-0.465210831	2.64E-01	same
chr12	45215529	45215929	-0.467776475	2.65E-01	same
chr7	149623658	149624058	-0.383750852	2.65E-01	same
chr3	139344104	139344504	-0.396020836	2.65E-01	same
chr18	50281480	50281880	-0.442752871	2.65E-01	same
chr1	51617338	51617738	-0.410655442	2.65E-01	same
chr1	45303816	45304216	-0.386199687	2.65E-01	same
chr5	181160231	181160631	-0.308458237	2.66E-01	same
chr16	2022868	2023268	-0.38255216	2.67E-01	same
chr3	160565750	160566150	-0.456164979	2.67E-01	same
chr6	10721003	10721403	-0.463495867	2.67E-01	same
chrX	153628412	153628812	-0.454631242	2.67E-01	same
chr5	124662424	124662824	-0.435092313	2.67E-01	same
chr4	102345240	102345640	-0.4154665	2.67E-01	same
chrX	123846662	123847062	-0.405121205	2.67E-01	same
chr10	94362652	94363052	-0.380888982	2.68E-01	same
chr16	20900469	20900869	-0.394315976	2.68E-01	same
chr5	6378023	6378423	-0.447843288	2.68E-01	same
chr22	46296376	46296776	-0.375933704	2.68E-01	same
chr1	89259693	89260093	-0.459585123	2.68E-01	same
chr15	70280095	70280617	0.463109181	2.68E-01	same
chr11	92331142	92331542	-0.469859139	2.68E-01	same
chr2	27322925	27323325	-0.336676185	2.69E-01	same
chr1	9724788	9725188	-0.450130638	2.69E-01	same

chr8	78445018	78445418	-0.440822757	2.69E-01	same
chr11	45804733	45805133	-0.418374382	2.69E-01	same
chr7	6602104	6602504	-0.430178457	2.70E-01	same
chr6	141814963	141815363	-0.454322588	2.70E-01	same
chr1	45500064	45500464	-0.377468968	2.71E-01	same
chr17	47067234	47067634	-0.461685234	2.71E-01	same
chr1	95234097	95234497	-0.467097734	2.71E-01	same
chr1	25232601	25233001	-0.423711306	2.72E-01	same
chr6	48827059	48827459	-0.466206858	2.72E-01	same
chr12	76551608	76552008	-0.467338926	2.73E-01	same
chr14	58427559	58427959	-0.346347879	2.73E-01	same
chr16	56730147	56730547	-0.417914023	2.73E-01	same
chr4	185425894	185426294	-0.380322082	2.73E-01	same
chr22	44335017	44335417	-0.430948085	2.73E-01	same
chr6	138988374	138988774	-0.46079848	2.73E-01	same
chr1	109239531	109239931	-0.387902461	2.73E-01	same
chr12	50025227	50025627	-0.400411713	2.74E-01	same
chr5	135834163	135834563	-0.435189386	2.74E-01	same
chr12	93677631	93678031	-0.432392068	2.74E-01	same
chr2	96305511	96305911	-0.378151495	2.74E-01	same
chr3	28349012	28349412	-0.351366803	2.74E-01	same
chr12	53352871	53353271	-0.433013474	2.75E-01	same
chr12	122437666	122438066	-0.458662556	2.75E-01	same
chr5	143087119	143087519	-0.408011451	2.75E-01	same
chr10	133443278	133443678	-0.451328527	2.75E-01	same
chr11	112074084	112074484	-0.410065938	2.75E-01	same
chr15	84570871	84571271	-0.363151993	2.75E-01	same
chr12	106956664	106957064	-0.429752278	2.75E-01	same
chr1	114716469	114716869	-0.390544092	2.76E-01	same
chr9	128921943	128922343	-0.403750042	2.76E-01	same
chr12	105109351	105109751	-0.445202557	2.76E-01	same
chr12	6924331	6924731	-0.386883238	2.77E-01	same
chr22	41446953	41447353	-0.374359639	2.77E-01	same
chr16	57475921	57476321	-0.38447554	2.78E-01	same
chr3	197960143	197960543	-0.384412917	2.78E-01	same
chr7	100344567	100344967	-0.397707014	2.78E-01	same
chr1	27830866	27831266	-0.409146832	2.78E-01	same
chr17	62987117	62987517	-0.424785118	2.78E-01	same
chr1	218460419	218460819	-0.447882853	2.79E-01	same
chr5	75050139	75050539	-0.439703939	2.79E-01	same
chr17	56743572	56743972	-0.455957219	2.79E-01	same
chr3	88149892	88150292	-0.428729383	2.79E-01	same
chr11	64331650	64332050	-0.429677989	2.79E-01	same
chr5	180816715	180817115	-0.413593938	2.80E-01	same
chr6	64999400	64999800	-0.459605626	2.80E-01	same
chr1	112619715	112620115	-0.376485919	2.80E-01	same
chr4	705305	705705	-0.36757535	2.81E-01	same
chr8	97777805	97778205	-0.434100397	2.81E-01	same
chr16	1518461	1518861	-0.431871765	2.81E-01	same
chr9	128656699	128657099	-0.428819299	2.82E-01	same
chr6	122569169	122569569	-0.425808776	2.82E-01	same
chr2	61439479	61439879	-0.434869543	2.82E-01	same
chr1	212463292	212463692	-0.421255643	2.82E-01	same
chr6	52882172	52882572	-0.42236031	2.82E-01	same
chr10	92517215	92517615	-0.440354611	2.83E-01	same
chr10	79335825	79336225	-0.437647652	2.83E-01	same
chr15	80059120	80059520	-0.408636447	2.83E-01	same
chr19	55173882	55174282	-0.423942994	2.84E-01	same
chr11	120400869	120401269	-0.444772922	2.84E-01	same
chr16	82169845	82170245	-0.444963738	2.86E-01	same
chr15	90301092	90301492	-0.409308573	2.86E-01	same
chrX	48574018	48574418	-0.359642193	2.86E-01	same
chr3	45712434	45712834	-0.426206219	2.87E-01	same
chr2	55268898	55269298	-0.418185295	2.87E-01	same
chr15	66504966	66505366	-0.314694264	2.87E-01	same
chr1	156591958	156592358	-0.392937308	2.88E-01	same
chr19	37217884	37218284	-0.3786555	2.88E-01	same
chr2	95165573	95165973	-0.407373144	2.88E-01	same
chr11	74398469	74398869	-0.420413674	2.88E-01	same
chr19	45506416	45506816	-0.39508478	2.88E-01	same
chr13	32538572	32538972	-0.381494808	2.88E-01	same
chr14	39169773	39170173	-0.336527568	2.88E-01	same
chr5	134648735	134649135	-0.434145937	2.88E-01	same
chr2	200748018	200748418	-0.440704609	2.88E-01	same
chr8	53881037	53881437	-0.433949921	2.88E-01	same
chr18	29309408	29309808	-0.428536734	2.88E-01	same
chr21	29073700	29074100	-0.359035032	2.88E-01	same

chr6	28225150	28225762	-0.362071429	2.89E-01	same
chr16	71484345	71484745	-0.415361199	2.89E-01	same
chr9	125143619	125144019	-0.409350904	2.89E-01	same
chr6	106011750	106012150	0.452001216	2.89E-01	same
chr8	125431786	125432186	-0.418522802	2.89E-01	same
chr12	72520081	72520481	-0.432357529	2.90E-01	same
chr6	45377036	45377436	-0.426649939	2.90E-01	same
chr9	124868775	124869175	-0.391847098	2.90E-01	same
chr9	127567418	127567818	-0.395663	2.90E-01	same
chr13	79441335	79441735	-0.343103463	2.90E-01	same
chr1	37859654	37860054	-0.359313696	2.90E-01	same
chr17	44006904	44007613	-0.30918709	2.90E-01	same
chr16	70428401	70428801	-0.386738583	2.91E-01	same
chr19	58386830	58387230	-0.366404057	2.91E-01	same
chr4	26319837	26320237	-0.359883636	2.91E-01	same
chr7	149310151	149310551	-0.306574798	2.91E-01	same
chr11	113773555	113773955	-0.369288415	2.91E-01	same
chr11	46826793	46827193	-0.401100603	2.92E-01	same
chr1	58546608	58547008	-0.362272071	2.92E-01	same
chr2	24084115	24084515	-0.393940571	2.92E-01	same
chr1	219399339	219399739	-0.446779129	2.92E-01	same
chr3	73143936	73144336	-0.440167055	2.93E-01	same
chr1	36306941	36307341	-0.375801835	2.93E-01	same
chr1	195948009	195948409	0.446069927	2.93E-01	same
chr5	134632477	134632877	-0.375082217	2.93E-01	same
chr18	33577740	33578140	-0.36947613	2.93E-01	same
chr7	2232886	2233286	-0.422130583	2.93E-01	same
chr1	151254653	151255053	-0.326198173	2.93E-01	same
chr1	119712984	119713384	-0.36667666	2.94E-01	same
chr1	234475206	234475606	-0.387571256	2.94E-01	same
chr6	17586901	17587301	0.441598601	2.94E-01	same
chr4	119067480	119067880	-0.440072643	2.95E-01	same
chr7	120987874	120988274	-0.416826814	2.95E-01	same
chr1	204807045	204807445	-0.410100954	2.95E-01	same
chrX	49235351	49235751	-0.402317119	2.96E-01	same
chr8	93740945	93741345	-0.365514036	2.96E-01	same
chr1	147928352	147928752	-0.407251343	2.96E-01	same
chr6	8064011	8064411	-0.392900346	2.96E-01	same
chr15	74546083	74546483	-0.367567594	2.96E-01	same
chr11	121298250	121298650	-0.415647592	2.96E-01	same
chr20	34824717	34825117	-0.421445582	2.96E-01	same
chr8	142904014	142904414	0.441989497	2.97E-01	same
chr17	46922906	46923306	-0.356142316	2.97E-01	same
chr17	58692523	58692923	-0.373387902	2.97E-01	same
chr5	82278315	82278715	-0.380545254	2.98E-01	same
chr19	39996889	39997289	-0.363925346	2.98E-01	same
chr19	4267132	4267532	-0.399722845	2.98E-01	same
chr3	42590490	42590890	-0.332867479	2.98E-01	same
chr22	41286095	41286495	-0.351511241	2.98E-01	same
chr12	9647998	9648398	-0.412619735	2.98E-01	same
chr10	120851259	120851659	-0.413732356	2.99E-01	same
chrX	155612592	155612992	0.39296056	2.99E-01	same
chr17	29369928	29370328	-0.416929228	2.99E-01	same
chr12	98593607	98594007	-0.386106552	2.99E-01	same
chr17	7815541	7815941	-0.426038819	3.00E-01	same
chr14	92039757	92040157	-0.394571454	3.00E-01	same
chr13	65387891	65388291	0.370449975	3.00E-01	same
chr1	160194444	160194844	0.390374134	3.00E-01	same
chr10	120978767	120979167	-0.378058847	3.01E-01	same
chr6	149565817	149566217	-0.394347833	3.01E-01	same
chr3	49412073	49412473	-0.308075592	3.01E-01	same
chr2	136118170	136118570	-0.424334629	3.01E-01	same
chrX	102951347	102951747	-0.344462129	3.01E-01	same
chr9	77012343	77012743	-0.424584205	3.02E-01	same
chr17	48620398	48620798	-0.368567978	3.02E-01	same
chr8	30556165	30556565	0.42152549	3.02E-01	same
chr14	102057111	102057511	-0.391043462	3.02E-01	same
chr6	35259166	35259566	-0.343329679	3.02E-01	same
chr3	47781988	47782388	-0.38216941	3.02E-01	same
chr14	96979446	96979846	-0.423885559	3.02E-01	same
chr1	228238610	228239010	0.396870743	3.02E-01	same
chr13	49997008	49997408	-0.391760445	3.03E-01	same
chr6	117675039	117675439	-0.370525556	3.03E-01	same
chr1	220990854	220991254	-0.398236408	3.03E-01	same
chr4	90950489	90951242	-0.388655412	3.04E-01	same
chr1	59321919	59322319	0.419091778	3.04E-01	same
chr17	82671185	82671585	-0.430301432	3.04E-01	same

chr9	137029335	137029735	-0.329180625	3.04E-01	same
chr14	39103203	39103603	-0.360607077	3.04E-01	same
chr7	144488725	144489125	-0.433827144	3.05E-01	same
chr19	32971906	32972306	-0.31280418	3.05E-01	same
chr3	180989421	180989821	-0.400011354	3.05E-01	same
chr13	84527953	84528353	0.39854082	3.06E-01	same
chr5	55711913	55712313	-0.433302614	3.06E-01	same
chr7	155958767	155959167	-0.293007311	3.06E-01	same
chr7	131586920	131587320	0.421321936	3.07E-01	same
chr1	147599056	147599456	-0.333145914	3.07E-01	same
chr10	92574263	92574663	-0.373720462	3.07E-01	same
chr18	35041325	35041725	-0.355653111	3.07E-01	same
chr1	55149863	55150263	-0.414517994	3.08E-01	same
chr19	7197411	7197811	-0.416831789	3.08E-01	same
chr2	19347720	19348120	-0.40075868	3.08E-01	same
chr2	74120238	74120638	-0.430182256	3.08E-01	same
chr13	65373941	65374341	0.387158772	3.08E-01	same
chr17	35089575	35089975	-0.420715625	3.08E-01	same
chr1	162613566	162613966	-0.405137221	3.09E-01	same
chr18	36879399	36879799	0.421653065	3.09E-01	same
chr2	43877875	43878275	-0.379770722	3.09E-01	same
chr14	45084275	45084675	-0.391537971	3.09E-01	same
chr7	155298991	155299391	-0.371281358	3.09E-01	same
chr1	199778340	199778740	-0.404607277	3.09E-01	same
chr12	121295522	121295922	-0.349999628	3.09E-01	same
chr19	40716355	40717049	-0.331595097	3.11E-01	same
chr4	2068132	2068532	-0.411545708	3.11E-01	same
chr5	142769940	142770340	-0.378198023	3.11E-01	same
chr3	50359251	50360005	-0.303183244	3.11E-01	same
chr9	124258625	124259025	-0.409209852	3.11E-01	same
chr14	72962789	72963189	-0.225056637	3.11E-01	same
chr13	67171920	67172320	0.345373483	3.12E-01	same
chr7	112789978	112790378	-0.430894363	3.12E-01	same
chr9	130053622	130054022	-0.403384449	3.12E-01	same
chr16	22374696	22375096	-0.377064313	3.12E-01	same
chr6	69866597	69866997	-0.401229681	3.12E-01	same
chr8	84182406	84182806	-0.426487734	3.13E-01	same
chr2	24539992	24540392	-0.393346681	3.13E-01	same
chr12	55940283	55940683	-0.378921423	3.13E-01	same
chr14	76784706	76785106	-0.340065925	3.13E-01	same
chr1	89524199	89524599	-0.382215371	3.14E-01	same
chr1	34852754	34853154	-0.396741728	3.14E-01	same
chr19	12722651	12723051	-0.352682751	3.15E-01	same
chr2	218909086	218909486	-0.363918351	3.15E-01	same
chr17	75405931	75406331	-0.335346278	3.15E-01	same
chr18	62186629	62187029	-0.376290459	3.16E-01	same
chr17	16215634	16216034	-0.381363695	3.16E-01	same
chr8	144463815	144464427	-0.327435751	3.16E-01	same
chr5	126776570	126776970	-0.36144338	3.16E-01	same
chr4	98143125	98143525	-0.405211281	3.17E-01	same
chr6	110874069	110874469	-0.363309957	3.17E-01	same
chr11	134275873	134276273	-0.398183919	3.17E-01	same
chr5	43039853	43040253	-0.380829601	3.17E-01	same
chr11	34483770	34484170	-0.358333611	3.17E-01	same
chr7	114085495	114085895	-0.375886974	3.17E-01	same
chr9	92877807	92878207	-0.348822935	3.17E-01	same
chr15	71023674	71024074	-0.427513704	3.18E-01	same
chr1	240562745	240563145	-0.329269704	3.18E-01	same
chr6	28104720	28105120	-0.327474224	3.18E-01	same
chr1	152354301	152354701	0.421673657	3.18E-01	same
chr6	26956104	26956504	-0.384435227	3.18E-01	same
chr15	74620936	74621336	-0.335824236	3.18E-01	same
chr17	3683067	3683467	-0.375939953	3.18E-01	same
chr1	211382985	211383385	0.413138543	3.18E-01	same
chrX	40735683	40736083	-0.387314009	3.19E-01	same
chr15	43791913	43792313	-0.345717087	3.19E-01	same
chr6	158040080	158040480	-0.352328855	3.19E-01	same
chr7	112182708	112183108	-0.414977195	3.19E-01	same
chr1	168709567	168709967	-0.395335845	3.19E-01	same
chr5	174799831	174800231	-0.388716178	3.19E-01	same
chr22	20981904	20982304	-0.366994468	3.20E-01	same
chr11	72793681	72794081	-0.363583671	3.20E-01	same
chr3	45995470	45995870	-0.34186371	3.20E-01	same
chr1	227257747	227258147	-0.402062621	3.20E-01	same
chr2	48314191	48314591	-0.377860432	3.20E-01	same
chr3	48717427	48717827	-0.347438334	3.20E-01	same
chr12	122400032	122400432	-0.352485244	3.20E-01	same

chr16	87777715	87778115	-0.37859869	3.20E-01	same
chr1	32606102	32606502	-0.383521112	3.21E-01	same
chr12	56689016	56689416	-0.306195177	3.21E-01	same
chr18	21612127	21612527	-0.332173606	3.21E-01	same
chr16	84141101	84141501	-0.395587646	3.21E-01	same
chr19	43619613	43620013	-0.372489406	3.21E-01	same
chr16	54286381	54286781	-0.38183252	3.21E-01	same
chr3	10263997	10264397	-0.412728457	3.21E-01	same
chr1	90508438	90508838	0.385918801	3.21E-01	same
chr7	100126877	100127277	-0.3384186	3.21E-01	same
chr18	35290152	35290552	-0.347105881	3.22E-01	same
chr8	11467398	11467798	-0.413882623	3.22E-01	same
chr13	88390273	88390673	-0.409747957	3.22E-01	same
chr6	6008324	6008724	-0.406063765	3.22E-01	same
chr20	45881111	45881511	-0.367143903	3.22E-01	same
chr12	108856434	108856834	-0.393775199	3.23E-01	same
chr10	19816198	19816598	-0.410649398	3.23E-01	same
chr13	66834998	66835398	-0.317353671	3.23E-01	same
chr19	45091070	45091470	-0.407397059	3.23E-01	same
chr11	65873353	65873753	-0.338850876	3.23E-01	same
chr19	37317670	37318070	-0.41964734	3.24E-01	same
chr7	129054814	129055214	-0.333130724	3.24E-01	same
chr1	20787540	20787940	-0.387276988	3.24E-01	same
chr6	37499891	37500291	-0.357055129	3.24E-01	same
chr1	11934248	11934648	-0.403364882	3.25E-01	same
chr17	57084727	57085127	-0.371744149	3.25E-01	same
chr1	212665350	212665750	-0.330952503	3.25E-01	same
chr19	14443891	14444291	-0.392965841	3.26E-01	same
chr1	43349170	43349570	-0.393106908	3.26E-01	same
chr3	136328747	136329147	0.419803111	3.26E-01	same
chr17	56810128	56810528	-0.417828947	3.27E-01	same
chr17	4366730	4367130	-0.391345064	3.27E-01	same
chr16	57417021	57417421	-0.295429956	3.27E-01	same
chr17	28903026	28903426	-0.343561598	3.27E-01	same
chr2	201642872	201643272	-0.368215539	3.28E-01	same
chr3	129249266	129249666	-0.328104657	3.28E-01	same
chr19	16542546	16542946	-0.411862732	3.28E-01	same
chrX	127866727	127867127	-0.361441332	3.28E-01	same
chr17	47492512	47492912	-0.397839588	3.29E-01	same
chr9	97922166	97922566	-0.322986902	3.29E-01	same
chr1	198054259	198054659	-0.394904394	3.29E-01	same
chr2	61694408	61694808	-0.396651049	3.30E-01	same
chr6	80004245	80004645	-0.358596895	3.32E-01	same
chr1	201946511	201946911	-0.317231361	3.32E-01	same
chr7	157228813	157229213	-0.351534724	3.32E-01	same
chrX	154526992	154527392	-0.352506805	3.32E-01	same
chr1	190294763	190295163	-0.403531551	3.32E-01	same
chr19	36127993	36128393	-0.350017185	3.33E-01	same
chr16	53208221	53208621	-0.388108675	3.33E-01	same
chr8	99013015	99013415	-0.340054934	3.33E-01	same
chr6	13574806	13575206	-0.354163424	3.33E-01	same
chr17	63889472	63889872	-0.391774165	3.34E-01	same
chr12	12725790	12726190	-0.387727199	3.34E-01	same
chr15	81324243	81324643	-0.349400122	3.34E-01	same
chr1	164772631	164773162	-0.348861432	3.34E-01	same
chr17	76671406	76671806	-0.29452487	3.35E-01	same
chr22	50498398	50498798	-0.346019283	3.36E-01	same
chr19	58451110	58451510	-0.378303134	3.36E-01	same
chr8	68325371	68325771	-0.410100081	3.36E-01	same
chr1	88992769	88993169	-0.332477391	3.37E-01	same
chr7	105298854	105299254	-0.395371237	3.37E-01	same
chr4	145097975	145098375	-0.335550845	3.37E-01	same
chr19	8269034	8269434	-0.352801465	3.37E-01	same
chr17	82260047	82260447	-0.374732273	3.38E-01	same
chr11	64372993	64373393	-0.342904456	3.38E-01	same
chr19	52028250	52028650	-0.371546909	3.39E-01	same
chr7	151313215	151313615	0.33937285	3.39E-01	same
chr11	31369804	31370204	-0.38625284	3.39E-01	same
chr12	131791868	131792268	-0.36353023	3.39E-01	same
chr6	27824332	27824732	-0.303945988	3.39E-01	same
chr17	43900652	43901052	-0.347507637	3.39E-01	same
chr7	12403847	12404247	-0.365525236	3.39E-01	same
chr7	116211149	116211549	-0.360345646	3.40E-01	same
chr5	57095278	57095678	-0.397846439	3.41E-01	same
chr7	92133584	92133984	-0.334811524	3.41E-01	same
chr4	1940086	1940486	-0.385387179	3.41E-01	same
chr16	21598932	21599332	-0.380337306	3.41E-01	same

chr4	139808544	139808944	-0.398030593	3.41E-01	same
chr8	81685961	81686361	-0.320398322	3.42E-01	same
chr2	193102040	193102440	-0.405759372	3.42E-01	same
chr5	100535257	100535657	-0.400834224	3.42E-01	same
chr13	61766294	61766694	0.32600007	3.42E-01	same
chr11	9384573	9384973	-0.399428131	3.43E-01	same
chr6	2988506	2988906	-0.322848697	3.43E-01	same
chr15	89647198	89647598	-0.31217711	3.44E-01	same
chr9	83965318	83965718	-0.311028742	3.44E-01	same
chr17	9575822	9576222	-0.38827666	3.46E-01	same
chr16	70252224	70252624	-0.338917587	3.46E-01	same
chr5	140043273	140043673	-0.380274405	3.46E-01	same
chr8	37359743	37360143	-0.376973198	3.47E-01	same
chr10	86980176	86980576	-0.402517103	3.47E-01	same
chr17	67246596	67246996	-0.359442146	3.47E-01	same
chr1	69157330	69157730	0.392170662	3.47E-01	same
chr13	21140211	21140611	-0.377076237	3.47E-01	same
chr1	152034381	152034781	-0.331068717	3.47E-01	same
chr15	42495782	42496182	0.379067027	3.48E-01	same
chr2	127700903	127701303	-0.336467266	3.48E-01	same
chr3	158644463	158644863	-0.372060924	3.48E-01	same
chr17	49216798	49217198	-0.393096158	3.49E-01	same
chr12	132685301	132685701	-0.33954603	3.49E-01	same
chr7	134779444	134779844	-0.357412439	3.49E-01	same
chr2	47322890	47323290	-0.378092756	3.49E-01	same
chr11	85664847	85665247	-0.352359965	3.49E-01	same
chr12	54259237	54259637	-0.364630155	3.49E-01	same
chr20	36236415	36236815	-0.365831572	3.49E-01	same
chr1	31549034	31549434	-0.321439179	3.50E-01	same
chr1	120942460	120942860	-0.361384886	3.50E-01	same
chr19	2944969	2945369	-0.36489231	3.50E-01	same
chr11	119368191	119368591	0.397656572	3.51E-01	same
chr8	70244964	70245364	-0.384615168	3.51E-01	same
chr2	169694378	169694778	-0.36257545	3.51E-01	same
chr15	44578289	44578689	-0.380229855	3.51E-01	same
chr9	136190806	136191206	-0.375051919	3.52E-01	same
chr1	44708054	44708454	-0.357668971	3.52E-01	same
chr11	9529465	9529865	-0.367712595	3.52E-01	same
chr6	146914100	146914500	-0.382671988	3.52E-01	same
chr6	125153558	125153958	-0.397072272	3.53E-01	same
chr10	96130146	96130546	-0.364127984	3.53E-01	same
chr17	13602061	13602461	-0.388208734	3.53E-01	same
chr3	9249353	9249753	-0.329135486	3.53E-01	same
chr19	46784676	46785076	-0.311809362	3.54E-01	same
chr18	267778	268178	-0.36327777	3.54E-01	same
chr4	47914310	47914710	-0.368496532	3.54E-01	same
chr1	203273156	203273556	-0.309581616	3.54E-01	same
chr17	44947455	44947855	-0.314058722	3.55E-01	same
chr3	13372037	13372437	-0.369179312	3.56E-01	same
chr1	81920369	81920769	-0.349527034	3.56E-01	same
chr5	154857527	154857927	-0.344158645	3.57E-01	same
chr2	19845398	19845798	0.386960735	3.57E-01	same
chr13	41131990	41132390	-0.337511989	3.57E-01	same
chr11	116276462	116276862	-0.359986654	3.58E-01	same
chr5	92552613	92553013	-0.368200521	3.58E-01	same
chr16	28491711	28492111	-0.319631609	3.59E-01	same
chr1	77448908	77449308	0.373518474	3.59E-01	same
chr2	214310797	214311197	0.373834215	3.59E-01	same
chr11	118659251	118659651	-0.322557813	3.59E-01	same
chr13	86409604	86410004	0.362222234	3.59E-01	same
chr9	137680576	137680976	-0.37818007	3.59E-01	same
chr10	65570433	65570833	-0.391314501	3.60E-01	same
chr17	76398190	76398590	-0.297718683	3.60E-01	same
chr19	43934933	43935333	-0.376255987	3.60E-01	same
chr17	32142031	32142431	-0.361924679	3.60E-01	same
chr16	4767198	4767598	-0.348509699	3.61E-01	same
chr1	196786589	196786989	0.37592279	3.61E-01	same
chr12	110124282	110124682	-0.37467887	3.61E-01	same
chr17	44899544	44899944	-0.310671385	3.61E-01	same
chr19	47483845	47484245	-0.314278734	3.63E-01	same
chr10	26939480	26939880	-0.350165564	3.63E-01	same
chr7	25125247	25125647	-0.29533928	3.63E-01	same
chr1	85708247	85708647	-0.274327509	3.63E-01	same
chr4	158670940	158671340	-0.361280877	3.63E-01	same
chr2	88016743	88017143	-0.286876464	3.64E-01	same
chr17	68247579	68247979	-0.344155117	3.64E-01	same
chr10	97676757	97677157	-0.341923358	3.64E-01	same

chr15	79853650	79854050	-0.360876144	3.64E-01	same
chr9	133336198	133336598	-0.341594005	3.65E-01	same
chr1	40531502	40531902	-0.340786865	3.65E-01	same
chr3	52704814	52705214	-0.322621705	3.65E-01	same
chr12	64222034	64222434	-0.350630535	3.65E-01	same
chr20	32820136	32820536	-0.346541762	3.67E-01	same
chr7	117872272	117872672	-0.358674723	3.67E-01	same
chr13	21298466	21298866	0.369548346	3.67E-01	same
chr11	6481572	6481972	-0.351959227	3.67E-01	same
chr7	157517991	157518391	-0.382165045	3.67E-01	same
chr9	128276030	128276430	-0.307808776	3.68E-01	same
chr3	32398897	32399297	-0.380542262	3.68E-01	same
chr4	97633833	97634233	-0.366913989	3.68E-01	same
chr12	123458266	123458666	-0.279321901	3.68E-01	same
chr16	48365536	48365936	0.370380211	3.69E-01	same
chr12	132710346	132710746	-0.349955321	3.69E-01	same
chr2	218387691	218388091	-0.347181578	3.69E-01	same
chr12	110048220	110048620	-0.334941781	3.70E-01	same
chr10	103917695	103918095	-0.339442333	3.70E-01	same
chr4	84582462	84582862	-0.354339367	3.71E-01	same
chr20	6746928	6747328	-0.332220955	3.72E-01	same
chr7	156155792	156156192	0.37934926	3.73E-01	same
chr1	235511630	235512030	-0.333232474	3.73E-01	same
chr7	5495042	5495442	0.32310635	3.74E-01	same
chr20	35092855	35093255	-0.355014405	3.74E-01	same
chr16	69754613	69755013	-0.343036815	3.75E-01	same
chr1	186307662	186308062	-0.377421657	3.75E-01	same
chr4	39459101	39459501	-0.31151804	3.75E-01	same
chr5	94111240	94111640	-0.347615053	3.76E-01	same
chr9	128341266	128341666	-0.26981783	3.76E-01	same
chr10	102743800	102744200	-0.301952104	3.76E-01	same
chr13	79406302	79406702	-0.282609696	3.76E-01	same
chr16	84504396	84505173	-0.281981702	3.76E-01	same
chr14	32134499	32134899	-0.313974438	3.77E-01	same
chr7	116526218	116526618	-0.349439117	3.77E-01	same
chr12	70242930	70243330	-0.358683728	3.77E-01	same
chr1	64862733	64863133	-0.370305608	3.78E-01	same
chr3	163878066	163878466	-0.369362843	3.78E-01	same
chr15	55741343	55741743	-0.351640763	3.79E-01	same
chr11	13462624	13463024	-0.353022507	3.79E-01	same
chr17	8057531	8057931	-0.321164082	3.79E-01	same
chr14_GL000225v1_random	67403	67803	-0.373452234	3.80E-01	same
chr6	168074274	168074674	-0.35980261	3.80E-01	same
chr17	40647742	40648142	-0.368692544	3.80E-01	same
chr5	84435768	84436168	-0.330487456	3.81E-01	same
chr17	61957201	61957601	-0.356162077	3.81E-01	same
chr21	30993818	30994218	-0.348582222	3.82E-01	same
chr2	70141088	70141488	-0.299429267	3.82E-01	same
chr17	41835285	41835685	-0.323432032	3.82E-01	same
chr3	129279423	129279823	-0.352602854	3.82E-01	same
chr19	4140156	4140556	-0.358699259	3.83E-01	same
chr5	178357940	178358340	0.366393909	3.83E-01	same
chr5	172771857	172772257	-0.319521671	3.83E-01	same
chr1	210074840	210075240	-0.36334649	3.83E-01	same
chr17	78054389	78054789	0.299862552	3.83E-01	same
chr6	44234305	44234705	-0.314505397	3.84E-01	same
chr15	74461284	74461684	-0.29504229	3.84E-01	same
chr12	79045140	79045540	-0.367379808	3.85E-01	same
chr7	140154054	140154454	-0.34075654	3.86E-01	same
chr2	47906583	47906983	-0.361858901	3.86E-01	same
chrX	38561543	38561943	-0.362997855	3.86E-01	same
chr6	99520936	99521336	-0.303918668	3.87E-01	same
chr12	94459868	94460268	-0.343045579	3.87E-01	same
chr3	15738456	15738856	-0.339932303	3.88E-01	same
chrUn_KI270744v1	111130	111888	-0.2906029	3.89E-01	same
chr8	75780617	75781017	-0.362602284	3.89E-01	same
chr9	20658268	20658668	-0.355694773	3.90E-01	same
chr2	74454985	74455385	-0.30375484	3.90E-01	same
chr17_KI270729v1_random	1311	1711	0.337456528	3.91E-01	same
chr8	93754777	93755177	-0.296607818	3.91E-01	same
chr16	89343670	89344070	-0.321859924	3.91E-01	same
chr6	116279623	116280023	-0.302043838	3.92E-01	same
chr10	96089850	96090250	-0.303306905	3.92E-01	same
chr7	158704309	158704709	-0.324481905	3.93E-01	same
chr12	50400682	50401082	-0.299181249	3.93E-01	same
chr5	91380577	91380977	-0.337697256	3.94E-01	same
chr3	8963121	8963521	-0.344437675	3.94E-01	same

chr6	112043267	112043667	-0.347039023	3.94E-01	same
chr3	51974244	51974644	-0.306150291	3.95E-01	same
chr9	123678529	123678929	-0.3434748	3.95E-01	same
chr13	67200306	67200706	-0.30413032	3.95E-01	same
chr12	48553724	48554124	-0.349674256	3.96E-01	same
chr1_KI270711v1_random	8085	8485	0.36031008	3.97E-01	same
chr22	41666896	41667296	-0.327997598	3.97E-01	same
chr3	120622994	120623394	-0.328679571	3.97E-01	same
chr1	46442369	46442769	-0.281347168	3.98E-01	same
chr12	47706108	47706508	-0.334445165	3.98E-01	same
chr2	180034080	180034757	-0.315849188	3.98E-01	same
chr13	102596402	102596802	-0.336987192	3.99E-01	same
chr4	184733619	184734019	-0.34451902	3.99E-01	same
chr2	192682668	192683068	-0.35225219	4.00E-01	same
chr1	24642508	24642908	-0.352188071	4.00E-01	same
chr13	65553438	65553838	-0.295872105	4.01E-01	same
chr21	29213853	29214253	0.325738344	4.01E-01	same
chr16	71808533	71808933	-0.324859216	4.01E-01	same
chr9	90433171	90433571	-0.354120008	4.01E-01	same
chr1	229229535	229229935	-0.295582135	4.01E-01	same
chr4	126427949	126428349	-0.335814581	4.01E-01	same
chr3	187745412	187745812	-0.346599118	4.01E-01	same
chr12	23950006	23950406	-0.356353066	4.02E-01	same
chr9	122912984	122913384	-0.321413615	4.02E-01	same
chr15	44663764	44664164	-0.332924437	4.02E-01	same
chr19	40614282	40614682	-0.283247963	4.03E-01	same
chr12	29726589	29726989	0.348282983	4.03E-01	same
chr14	69191488	69191888	-0.308744354	4.03E-01	same
chr17	42833143	42833543	-0.283590672	4.03E-01	same
chr16	69132536	69132936	-0.356882939	4.03E-01	same
chr4	121870241	121870641	-0.268256649	4.04E-01	same
chr13	62847512	62847912	-0.279883535	4.04E-01	same
chr7	36258405	36258805	-0.334409421	4.04E-01	same
chr13	63044573	63044973	0.314821451	4.05E-01	same
chr22	44993195	44993595	-0.356157253	4.05E-01	same
chr5	173973447	173973847	-0.346941534	4.05E-01	same
chr15	43800556	43800956	-0.323727146	4.05E-01	same
chr6	11427354	11427754	0.351264618	4.06E-01	same
chr2	199910645	199911045	-0.33628798	4.06E-01	same
chr18	36933566	36933966	0.317373702	4.06E-01	same
chr9	94726671	94727071	-0.335720092	4.06E-01	same
chr6	43059551	43059951	-0.312924348	4.07E-01	same
chr18	9136733	9137133	-0.351589065	4.07E-01	same
chr19	43198692	43199092	-0.270537207	4.07E-01	same
chr6	144063686	144064086	-0.334059675	4.08E-01	same
chr22	35399947	35400347	-0.281286751	4.08E-01	same
chr16	3365045	3365445	-0.324767923	4.09E-01	same
chr7	45768920	45769320	-0.280233775	4.09E-01	same
chr14	59507230	59507630	-0.333220451	4.09E-01	same
chr8	145002990	145003390	-0.334233115	4.09E-01	same
chr13	71621374	71621774	-0.31687895	4.10E-01	same
chr17	81040629	81041029	-0.273381195	4.10E-01	same
chr22	37696670	37697070	-0.268118461	4.10E-01	same
chr1	152047929	152048329	-0.297081363	4.10E-01	same
chrX	104156849	104157249	-0.265387597	4.10E-01	same
chr3	77191325	77191725	-0.342945479	4.12E-01	same
chrUn_KI270587v1	907	1307	0.263857396	4.12E-01	same
chr11	69101272	69101672	0.30968039	4.12E-01	same
chr3	126355754	126356154	-0.316156016	4.13E-01	same
chr17	42458784	42459184	-0.340753578	4.14E-01	same
chr8	72479495	72479895	-0.332513014	4.14E-01	same
chr4	4290247	4290647	-0.332468076	4.14E-01	same
chr12	121811623	121812023	-0.291657445	4.14E-01	same
chr9	134135672	134136072	-0.345547515	4.15E-01	same
chr1	155207147	155207547	-0.280876764	4.15E-01	same
chr5	150161379	150161779	-0.278293971	4.15E-01	same
chr7	5309064	5309464	0.330991412	4.15E-01	same
chr10	15169000	15169400	-0.313378231	4.16E-01	same
chr11	67464385	67464785	-0.297438992	4.16E-01	same
chr19	36573048	36573448	-0.312766418	4.16E-01	same
chr4	128265435	128265835	-0.305673517	4.16E-01	same
chr1	32150966	32151366	-0.314440637	4.16E-01	same
chr4	52722126	52722526	-0.314796348	4.16E-01	same
chr16	29612185	29612585	-0.337542425	4.17E-01	same
chr18	36997966	36998366	0.323988757	4.18E-01	same
chr11	47214370	47214770	-0.294614498	4.18E-01	same
chr8	100907743	100908143	-0.318114202	4.18E-01	same

chr3	155744189	155744589	-0.318368874	4.19E-01	same
chr8	116766404	116766804	-0.298223094	4.19E-01	same
chr1	7655404	7655804	-0.330150814	4.19E-01	same
chr6	1802526	1802926	-0.334370497	4.19E-01	same
chr14	75127107	75127507	-0.323351209	4.19E-01	same
chr14	50943742	50944142	-0.308389709	4.19E-01	same
chr9	112486376	112486776	-0.323019239	4.19E-01	same
chr1	80848115	80848515	-0.337829788	4.19E-01	same
chr11	114439431	114439831	-0.313720974	4.20E-01	same
chr18	24076222	24076622	-0.289504131	4.20E-01	same
chr4	115130902	115131302	-0.34309863	4.21E-01	same
chr13	66646948	66647348	0.302903387	4.21E-01	same
chr17	64146805	64147205	-0.302392625	4.21E-01	same
chr13	89167541	89167941	0.3333602	4.22E-01	same
chr5	138536690	138537090	-0.284514294	4.22E-01	same
chr15	40038929	40039329	-0.30586303	4.22E-01	same
chrX	129471898	129472298	-0.313438197	4.23E-01	same
chr2	30146602	30147002	-0.278699382	4.23E-01	same
chr10	102245220	102245620	-0.238493962	4.23E-01	same
chr1	165828658	165829058	-0.314929285	4.23E-01	same
chr5	34838965	34839365	-0.291191343	4.24E-01	same
chr12	49283866	49284266	0.317950693	4.24E-01	same
chr9	85742214	85742614	-0.302841918	4.25E-01	same
chr17	63082757	63083157	-0.328348717	4.26E-01	same
chr13	49247785	49248185	-0.287012894	4.26E-01	same
chr19	57411176	57411576	-0.321840271	4.26E-01	same
chr8	69466666	69467066	-0.330215017	4.26E-01	same
chr9	92670290	92670690	-0.317448224	4.26E-01	same
chr11	19117109	19117509	-0.324709548	4.27E-01	same
chr20	3854943	3855343	-0.324284188	4.27E-01	same
chr18	12430209	12430609	-0.335338367	4.27E-01	same
chr22	32333768	32334168	0.32095961	4.28E-01	same
chr14	71628350	71628750	-0.320130223	4.28E-01	same
chr12	10212286	10212686	-0.288246788	4.28E-01	same
chr6	36422964	36423364	-0.3137274	4.28E-01	same
chr10	74023238	74023638	-0.3319969	4.28E-01	same
chr17	74916796	74917196	-0.318034509	4.28E-01	same
chr17	7435454	7435854	-0.294093808	4.29E-01	same
chr19	14207194	14207594	-0.336006623	4.29E-01	same
chr1	165768587	165768987	-0.290920061	4.29E-01	same
chr12	130134939	130135339	0.318117021	4.29E-01	same
chr1_KI270709v1_random	7287	7687	0.335282822	4.29E-01	same
chr6	99588630	99589030	-0.320914182	4.29E-01	same
chr5	43105408	43105808	-0.318715959	4.30E-01	same
chr1	112637347	112637747	-0.332052639	4.30E-01	same
chr22	41928457	41928857	-0.274291856	4.31E-01	same
chr16	28974311	28974711	-0.313688663	4.31E-01	same
chr6	34887949	34888349	-0.290884229	4.31E-01	same
chr18	40976475	40977139	-0.321418481	4.31E-01	same
chr7	155509651	155510051	-0.295772965	4.32E-01	same
chr18	24139060	24139460	-0.287625472	4.32E-01	same
chr19	48720822	48721222	-0.26227387	4.32E-01	same
chr12	66169441	66169841	-0.285424022	4.32E-01	same
chr2	138088522	138088922	-0.3225587	4.32E-01	same
chr2	42168924	42169324	-0.335277469	4.32E-01	same
chr1	26531386	26531786	-0.326093305	4.33E-01	same
chr19	48836895	48837295	-0.299038893	4.33E-01	same
chr17_GL000205v2_random	56434	56834	0.277934246	4.33E-01	same
chr19	58305248	58305648	-0.284465402	4.33E-01	same
chr17	78782427	78782827	-0.284471306	4.33E-01	same
chr14	104725308	104725708	-0.273187092	4.34E-01	same
chr22	26512332	26512732	-0.302131803	4.34E-01	same
chr21	33360996	33361396	-0.307292199	4.35E-01	same
chr21	32728029	32728429	-0.260560576	4.35E-01	same
chr12	57744945	57745345	-0.249844399	4.35E-01	same
chr17	34981240	34981640	-0.313267392	4.36E-01	same
chr14	91114015	91114415	-0.306030052	4.36E-01	same
chr12	31728917	31729317	-0.296045031	4.36E-01	same
chr1	231627286	231627686	-0.280077354	4.36E-01	same
chrX	101348715	101349115	-0.284559731	4.36E-01	same
chr1	228781357	228781757	-0.250901577	4.36E-01	same
chr2	180007064	180007464	-0.307006041	4.36E-01	same
chr6	111873342	111873742	-0.309440897	4.37E-01	same
chr6	2852451	2852851	-0.291112956	4.38E-01	same
chr19	38506904	38507304	-0.248756103	4.39E-01	same
chr1	196608645	196609045	-0.268837699	4.40E-01	same
chr13	27425519	27425919	0.320546413	4.40E-01	same

chr19	2900744	2901144	-0.31072632	4.41E-01	same
chr2	135876516	135876916	-0.298969119	4.41E-01	same
chr6	79946343	79946743	-0.301497684	4.41E-01	same
chr14	68292128	68292528	-0.323866482	4.41E-01	same
chr1	204446299	204446699	-0.27501514	4.44E-01	same
chr13	35762095	35762495	0.304489326	4.46E-01	same
chrX	107654999	107655399	-0.286937658	4.46E-01	same
chr15	40383096	40383496	0.302433023	4.46E-01	same
chr10	58385115	58385515	-0.266104486	4.47E-01	same
chr2	133435170	133435570	-0.29098697	4.48E-01	same
chr10	42552602	42553002	-0.293058594	4.49E-01	same
chr11	17730770	17731170	-0.264332942	4.50E-01	same
chr10	95907858	95908258	-0.292708313	4.50E-01	same
chr8	144335054	144335454	-0.28775656	4.51E-01	same
chr10	79904648	79905048	-0.30378565	4.51E-01	same
chr1	151195867	151196267	-0.25351191	4.51E-01	same
chr12	46160846	46161246	-0.300689694	4.52E-01	same
chr2	27032626	27033026	-0.274613387	4.52E-01	same
chr5	92802159	92802559	-0.317474555	4.52E-01	same
chr14	71320022	71320422	-0.30116577	4.52E-01	same
chr11	36594509	36594909	-0.288528112	4.53E-01	same
chr3	4867987	4868387	-0.299700333	4.53E-01	same
chr15	40098455	40098855	0.294301794	4.53E-01	same
chr2	170930155	170930555	-0.274038345	4.53E-01	same
chr1	223863982	223864382	-0.278179015	4.53E-01	same
chr2	131682305	131682705	-0.275421153	4.54E-01	same
chr1	235078158	235078558	0.274740706	4.54E-01	same
chr4	91064455	91064855	-0.305975836	4.54E-01	same
chr1	157812008	157812408	0.317259624	4.54E-01	same
chr12	108822983	108823383	-0.300983411	4.55E-01	same
chr1	25239849	25240249	-0.291728776	4.55E-01	same
chr12	132181317	132181717	0.301476359	4.56E-01	same
chr19	796583	796983	-0.266740604	4.56E-01	same
chr1	229011217	229011617	-0.266036866	4.57E-01	same
chr10	126143084	126143484	0.294038289	4.57E-01	same
chr19	13015494	13015894	-0.305594752	4.57E-01	same
chr13	72313742	72314142	0.315637346	4.57E-01	same
chr17	28515727	28516127	-0.242945692	4.57E-01	same
chr6	138972358	138972758	-0.297497287	4.58E-01	same
chr12	128189779	128190179	0.316650066	4.58E-01	same
chr21	43938920	43939320	-0.250827453	4.58E-01	same
chr16	69796368	69796768	0.295217096	4.59E-01	same
chr7	77877853	77878253	-0.310625103	4.59E-01	same
chr1	224851453	224851853	-0.267831307	4.60E-01	same
chr15	79744384	79744784	-0.217000728	4.60E-01	same
chr6	73654056	73654456	0.28925466	4.60E-01	same
chr3	156826178	156826578	-0.281500952	4.61E-01	same
chr9	92115193	92115593	-0.250744368	4.61E-01	same
chrX	115259474	115259874	-0.307069672	4.61E-01	same
chr1	143803992	143804392	-0.23238659	4.61E-01	same
chr8	54135182	54135582	-0.310395722	4.61E-01	same
chr8	31033716	31034116	-0.310872871	4.62E-01	same
chr8	52714495	52714895	-0.274344972	4.63E-01	same
chr3	119463448	119463848	-0.264170749	4.63E-01	same
chr1	35930459	35930859	-0.291582206	4.65E-01	same
chr10	111076211	111076611	-0.300967271	4.65E-01	same
chr17	17922520	17922920	-0.290629756	4.66E-01	same
chr1	93447695	93448095	-0.3111157	4.66E-01	same
chr18	33669860	33670260	-0.278689846	4.66E-01	same
chr1	103377688	103378088	-0.311455114	4.66E-01	same
chr2	95325764	95326164	-0.286377918	4.67E-01	same
chr1	77280895	77281295	0.288499996	4.69E-01	same
chr3	61251156	61251556	-0.287907566	4.70E-01	same
chr15	34225071	34225471	-0.28861559	4.71E-01	same
chr13	87793172	87793572	-0.294022736	4.71E-01	same
chr16	25015316	25015716	-0.281505134	4.72E-01	same
chr10	136839	137239	-0.243002846	4.72E-01	same
chr1	45011360	45011760	-0.258328395	4.72E-01	same
chr20	33401045	33401445	-0.274186272	4.73E-01	same
chr10	50992466	50992866	-0.29430459	4.75E-01	same
chr13	63566063	63566463	-0.257964788	4.75E-01	same
chr11	35821981	35822381	-0.294116045	4.75E-01	same
chr17	6443706	6444106	-0.280463926	4.76E-01	same
chr7	76997382	76997782	0.279645494	4.76E-01	same
chr6	26032771	26033171	-0.232491366	4.76E-01	same
chr13	46796789	46797189	-0.254710299	4.77E-01	same
chr1	151165533	151165933	-0.241376166	4.77E-01	same

chr14	74140419	74140819	-0.270377528	4.77E-01	same
chrX	47802892	47803292	-0.216618988	4.77E-01	same
chr10	27504250	27504650	-0.288363734	4.77E-01	same
chr16	28880079	28880479	-0.25873872	4.78E-01	same
chr5	171122494	171122894	0.297892137	4.78E-01	same
chr16	29302754	29303154	-0.257624535	4.79E-01	same
chr13	66165000	66165400	-0.249056963	4.79E-01	same
chr12	751722	752122	-0.272744927	4.80E-01	same
chrX	154763374	154763774	-0.257531061	4.82E-01	same
chr17	51121254	51121654	-0.286596663	4.82E-01	same
chr11	57530046	57530446	-0.275205596	4.82E-01	same
chr4	152352642	152353042	-0.278380819	4.82E-01	same
chr22	44604607	44605007	0.296557817	4.82E-01	same
chr12	105107662	105108062	-0.27486954	4.83E-01	same
chr2	177618641	177619041	-0.240518249	4.83E-01	same
chr7	149355425	149355825	0.260219443	4.83E-01	same
chr2	208254990	208255390	-0.250840195	4.83E-01	same
chr20	30579781	30580181	-0.294530202	4.84E-01	same
chr6	26957150	26957550	-0.277693483	4.84E-01	same
chr17	48908040	48908440	-0.2717045	4.85E-01	same
chr2	25078626	25079026	-0.291812604	4.85E-01	same
chr1	44238334	44238734	-0.264451457	4.85E-01	same
chr1	10210062	10210462	-0.243542796	4.85E-01	same
chr12	12891138	12891538	-0.258186233	4.87E-01	same
chr7	94004054	94004454	-0.273601512	4.87E-01	same
chr1	159141504	159141904	-0.281473471	4.88E-01	same
chr22	24259351	24259751	-0.294041662	4.88E-01	same
chr1	68232614	68233014	-0.276995207	4.89E-01	same
chr8	85107118	85107518	-0.280813263	4.89E-01	same
chr17	14300709	14301109	-0.238327161	4.90E-01	same
chr9	111400096	111400496	-0.272027499	4.90E-01	same
chr15	43184998	43185398	-0.276262949	4.91E-01	same
chr17	32205449	32205849	-0.268672708	4.92E-01	same
chr1	214514309	214514709	-0.234609168	4.92E-01	same
chr11	75625938	75626338	-0.291111679	4.92E-01	same
chr1	236635628	236636028	-0.282999036	4.92E-01	same
chr8	109539953	109540353	-0.250598593	4.93E-01	same
chr2	3605507	3605907	-0.273750172	4.94E-01	same
chr11	113314486	113314886	0.290039618	4.94E-01	same
chr8	102528430	102528830	-0.264779646	4.94E-01	same
chr8	132892622	132893022	0.284466946	4.94E-01	same
chr1	821158	821558	0.288209927	4.94E-01	same
chr20	36199583	36199983	0.285629588	4.95E-01	same
chr6	27347985	27348385	0.275052848	4.96E-01	same
chr22	27805445	27805845	-0.263880592	4.96E-01	same
chr9	130941162	130941562	-0.258566601	4.97E-01	same
chr1	206923982	206924382	0.249832786	4.97E-01	same
chr13	65180453	65180853	0.245292042	4.97E-01	same
chr1	207053043	207053443	-0.223577937	4.97E-01	same
chr2	175181254	175181654	-0.240876589	4.99E-01	same
chr5	171404450	171404850	-0.282129222	4.99E-01	same
chr12	49879098	49879498	0.279415194	5.00E-01	same
chr18	37091813	37092213	0.279096971	5.00E-01	same
chr17	75238768	75239168	-0.202442211	5.00E-01	same
chr12	121748287	121748687	-0.272184692	5.00E-01	same
chr18	37780676	37781076	0.271490339	5.00E-01	same
chr1	225509236	225509636	-0.246778673	5.00E-01	same
chr6	109691091	109691491	-0.248303828	5.01E-01	same
chr2	5509845	5510245	-0.265919563	5.01E-01	same
chr12	132133892	132134292	-0.242961125	5.02E-01	same
chr1	225305648	225306048	0.281050079	5.02E-01	same
chr17	67366280	67366680	-0.224409288	5.02E-01	same
chr1	212760038	212760438	-0.248890865	5.03E-01	same
chr6	122495236	122495636	0.282185432	5.03E-01	same
chr4	138816918	138817318	-0.258005864	5.04E-01	same
chr1	74733429	74733829	-0.23778094	5.04E-01	same
chr10	779976	780376	-0.280297499	5.04E-01	same
chr5	179535000	179535400	0.279578716	5.04E-01	same
chr14_GL000225v1_random	99127	99527	0.275165924	5.05E-01	same
chr12	114904345	114904745	0.276254437	5.05E-01	same
chr1	229148929	229149329	-0.195663083	5.05E-01	same
chr15	43510830	43511230	-0.243250635	5.06E-01	same
chr17	78992967	78993367	0.279809375	5.06E-01	same
chr19	49149652	49150052	-0.159061121	5.07E-01	same
chr12	132649081	132649481	-0.246002434	5.07E-01	same
chr3	131331494	131331894	-0.26691042	5.07E-01	same
chr1	32448555	32448955	-0.236242079	5.08E-01	same

chr19	45952756	45953156	-0.266456682	5.08E-01	same
chr2	241316049	241316449	-0.246923165	5.09E-01	same
chr1	220786202	220786602	-0.23425147	5.10E-01	same
chr3	10008857	10009257	-0.240498794	5.10E-01	same
chr9	35664817	35665217	-0.233469834	5.11E-01	same
chr19	1259290	1259690	-0.218325978	5.11E-01	same
chr1	145475250	145475650	-0.254586926	5.12E-01	same
chr17	78736657	78737057	-0.221057582	5.13E-01	same
chr11	124644442	124644842	-0.263708757	5.13E-01	same
chr7	145168852	145169252	0.270922497	5.15E-01	same
chr12	106357658	106358058	-0.23461963	5.15E-01	same
chr5	415427	415827	-0.263103274	5.15E-01	same
chr7	151948050	151948450	0.227494056	5.16E-01	same
chr21	29299642	29300042	-0.244419398	5.16E-01	same
chr1	109283010	109283410	-0.219507695	5.17E-01	same
chr19	58278523	58278923	-0.226676362	5.18E-01	same
chr10	68956277	68956677	-0.260290179	5.19E-01	same
chr6	12008629	12009029	-0.27039819	5.19E-01	same
chr1	32612064	32612464	-0.198500483	5.19E-01	same
chr4	149915559	149915959	-0.26810248	5.20E-01	same
chr6	125363531	125363931	-0.254231167	5.22E-01	same
chr14	29736511	29736911	-0.26437368	5.22E-01	same
chr4	41990434	41990834	-0.236866261	5.22E-01	same
chr1	112369666	112370066	-0.254074747	5.22E-01	same
chr13	113449214	113449614	-0.235273086	5.22E-01	same
chrUn_KI270442v1	391109	391509	0.241029984	5.24E-01	same
chr13	86840613	86841013	-0.245633418	5.24E-01	same
chr6	8102087	8102487	-0.242097211	5.24E-01	same
chr1	108963425	108963825	-0.23249302	5.26E-01	same
chr2	69893947	69894347	-0.25979209	5.26E-01	same
chr2	153871332	153871732	-0.268695139	5.26E-01	same
chr1	65254851	65255251	-0.269807445	5.26E-01	same
chr2	14747076	14747476	0.246469522	5.26E-01	same
chrX	81121520	81121920	-0.244858983	5.27E-01	same
chr5	125275193	125275593	-0.224076554	5.27E-01	same
chr7	149146995	149147395	0.222065493	5.27E-01	same
chr2	206765315	206765715	-0.231494601	5.27E-01	same
chr3	138187226	138187626	-0.239064554	5.28E-01	same
chr20	16188171	16188571	0.262428713	5.28E-01	same
chr4	107719642	107720042	-0.263387912	5.29E-01	same
chr6	10404556	10404956	-0.240198872	5.29E-01	same
chr14	44917058	44917458	-0.258943475	5.29E-01	same
chrX	104641100	104641500	0.250010431	5.29E-01	same
chr1	104671804	104672204	-0.243257812	5.29E-01	same
chr20	33138777	33139177	-0.26166862	5.30E-01	same
chr2	55618450	55618850	-0.213741894	5.30E-01	same
chr19	56393158	56393558	-0.263676346	5.31E-01	same
chr1	154627344	154627744	0.251631868	5.31E-01	same
chr22	17638421	17638821	-0.20168254	5.32E-01	same
chr12	121400617	121401017	-0.241919396	5.32E-01	same
chr3	52144426	52144826	-0.232662874	5.33E-01	same
chr3	10873567	10873967	-0.238716683	5.33E-01	same
chr2	20052782	20053182	-0.262375251	5.34E-01	same
chr14	69829004	69829404	-0.260761489	5.35E-01	same
chr1	155983670	155984070	-0.22320296	5.35E-01	same
chr14_GL000225v1_random	80901	81301	-0.259858394	5.38E-01	same
chr20	3408351	3408751	0.238463663	5.38E-01	same
chr7	151854684	151855084	0.257375844	5.38E-01	same
chr21	42670211	42670611	0.233957852	5.39E-01	same
chr17	40013594	40013994	-0.225290259	5.39E-01	same
chr2	40471199	40471599	-0.248390782	5.40E-01	same
chr17	18038949	18039349	-0.218484091	5.40E-01	same
chr2	88469401	88469801	-0.228352819	5.40E-01	same
chr13	65264513	65264913	-0.224452664	5.40E-01	same
chr11	44065998	44066398	-0.222302327	5.41E-01	same
chr2	219245063	219245463	-0.222454977	5.41E-01	same
chr12	115961012	115961412	-0.230838756	5.42E-01	same
chr6	141195831	141196231	-0.252287459	5.42E-01	same
chr3	94062908	94063308	-0.227442206	5.42E-01	same
chr6	32666417	32666817	-0.231877659	5.43E-01	same
chr16	28925314	28925714	-0.228819071	5.45E-01	same
chr1	40161229	40161629	-0.219249282	5.45E-01	same
chr1	108200321	108200721	-0.245791375	5.46E-01	same
chr11	2471626	2472026	0.23034857	5.46E-01	same
chr12	122423434	122423834	-0.218591924	5.47E-01	same
chr9	134146894	134147294	0.240747619	5.47E-01	same
chr16	67109490	67109890	-0.219463252	5.47E-01	same

chr13	91801491	91801891	-0.253496171	5.48E-01	same
chr2	650038	650438	0.256256587	5.48E-01	same
chr2	127508463	127508863	-0.235352058	5.50E-01	same
chr14	100383764	100384164	-0.245357548	5.51E-01	same
chrX	17737803	17738203	-0.250400199	5.53E-01	same
chr15	74696068	74696468	-0.217606161	5.53E-01	same
chr4	7070619	7071019	-0.240681964	5.53E-01	same
chr5	134184224	134184624	-0.237277053	5.55E-01	same
chr4	76306588	76306988	-0.218643999	5.57E-01	same
chr19	19515816	19516216	0.212443599	5.58E-01	same
chr1	168354442	168354842	-0.243005737	5.59E-01	same
chr18	76897138	76897538	0.216902039	5.59E-01	same
chr10	73358450	73358850	-0.217882052	5.63E-01	same
chr11	32583395	32583795	-0.209841973	5.63E-01	same
chr7	2807995	2808575	-0.211598499	5.63E-01	same
chr9	94554574	94554974	-0.236530879	5.64E-01	same
chr3	158672427	158672827	-0.211406041	5.64E-01	same
chr16	31404339	31404739	-0.229837179	5.64E-01	same
chr14	104805065	104805465	-0.202895181	5.65E-01	same
chr7	140924136	140924536	-0.221340617	5.66E-01	same
chr16	23641302	23641702	-0.197436955	5.66E-01	same
chr20	11791789	11792189	-0.228000252	5.66E-01	same
chr1	70902376	70902776	-0.24466347	5.67E-01	same
chr6	166925216	166925616	-0.22355465	5.67E-01	same
chr16	71845577	71845977	-0.225412885	5.68E-01	same
chr22	49998353	49998753	-0.204146764	5.68E-01	same
chr10	17228328	17228728	-0.224407719	5.68E-01	same
chr17	81904459	81904859	-0.189943633	5.68E-01	same
chr9	87042067	87042467	-0.239294265	5.69E-01	same
chr4	3814992	3815392	0.234308093	5.69E-01	same
chr11	74748463	74748863	-0.220504989	5.69E-01	same
chr14	68673593	68673993	-0.218013534	5.69E-01	same
chr2	45891568	45891968	-0.240113225	5.70E-01	same
chr4	177309933	177310333	-0.22729623	5.72E-01	same
chr1	15603191	15603591	-0.227126657	5.73E-01	same
chr7	143263364	143263764	-0.20806756	5.74E-01	same
chrX	47129247	47129647	-0.198682795	5.74E-01	same
chr16	1420394	1421153	-0.171942432	5.74E-01	same
chrX	70290073	70290473	-0.224735204	5.74E-01	same
chr17	42677741	42678141	-0.195244335	5.75E-01	same
chr1	72380449	72380849	-0.227608083	5.75E-01	same
chr3	187139479	187139879	-0.22821254	5.76E-01	same
chr18	23505340	23505740	-0.209815123	5.77E-01	same
chr1	53238430	53238830	-0.197086554	5.77E-01	same
chr5	95589212	95589612	0.238189377	5.77E-01	same
chr2	22703255	22703655	0.237712848	5.77E-01	same
chr5	140557407	140557807	-0.175772305	5.78E-01	same
chr4	143381617	143382017	0.212531418	5.78E-01	same
chr13	62887466	62887866	-0.196906043	5.78E-01	same
chr1	231806723	231807123	-0.21054641	5.80E-01	same
chr16	89374805	89375205	-0.199847428	5.81E-01	same
chr8	51501263	51501663	0.230580274	5.81E-01	same
chr2	127024950	127025350	-0.217215435	5.83E-01	same
chr1	61057631	61058031	-0.182598358	5.83E-01	same
chr17	44919928	44920328	-0.199458107	5.83E-01	same
chr7	139283815	139284215	0.228541017	5.85E-01	same
chr1	73549762	73550162	0.228275754	5.85E-01	same
chr6	158853058	158853458	-0.198271302	5.85E-01	same
chr5	57137997	57138397	-0.219196651	5.85E-01	same
chr3	133693403	133693803	-0.22163836	5.87E-01	same
chr8	79827692	79828092	-0.212921851	5.87E-01	same
chr14	102362211	102362611	-0.21102218	5.87E-01	same
chrUn_KI270411v1	1163	1563	0.209775822	5.87E-01	same
chr3	135439362	135439762	-0.229994809	5.88E-01	same
chr8	51700396	51700796	0.212809351	5.88E-01	same
chr2	47176609	47177009	-0.198102107	5.90E-01	same
chr13	63525965	63526365	-0.212544284	5.90E-01	same
chr1	37989663	37990063	-0.195233059	5.91E-01	same
chr14	94937885	94938285	0.217487127	5.91E-01	same
chr4	176238504	176238904	-0.221027016	5.93E-01	same
chr8	38769060	38769460	-0.222060834	5.93E-01	same
chr12	43104580	43104980	-0.227172772	5.93E-01	same
chr20	1277567	1277967	0.218970104	5.93E-01	same
chr7	156344686	156345086	-0.222153282	5.94E-01	same
chr12	132593725	132594125	-0.177530121	5.94E-01	same
chr22	48349032	48349432	-0.225716242	5.95E-01	same
chr14	49677380	49677780	-0.209536754	5.95E-01	same

chr2	29280354	29280754	0.222271171	5.95E-01	same
chr13	68297106	68297506	-0.218043659	5.96E-01	same
chr13	92456927	92457327	-0.22179148	5.97E-01	same
chr9	37785005	37785405	-0.198766431	5.97E-01	same
chr19	41918645	41919045	-0.204178293	5.98E-01	same
chr16	1264368	1264768	-0.219426267	5.98E-01	same
chr6	159112029	159112429	-0.184821859	5.99E-01	same
chr1	211620015	211620415	-0.191355985	5.99E-01	same
chrX	155246963	155247363	0.221414773	6.00E-01	same
chr12	132731836	132732236	-0.217517644	6.00E-01	same
chr5	149345225	149345625	-0.197849244	6.01E-01	same
chr7	145902358	145902758	-0.20773846	6.01E-01	same
chr1	43707157	43707557	-0.194436217	6.01E-01	same
chr12	43552036	43552436	-0.213081291	6.01E-01	same
chr7	49788533	49788933	0.220835486	6.02E-01	same
chr19	21142003	21142403	-0.21031371	6.02E-01	same
chr8	60516312	60516712	-0.21742901	6.02E-01	same
chr12	119804221	119804621	-0.202610561	6.03E-01	same
chr16	18946363	18946763	-0.211144725	6.04E-01	same
chr11	65834115	65834515	-0.198623707	6.04E-01	same
chr1	148345231	148345631	0.206608761	6.04E-01	same
chr14	48805276	48805676	-0.218276848	6.06E-01	same
chr5	173144700	173145100	-0.18738193	6.07E-01	same
chr6	141288765	141289165	0.213139985	6.07E-01	same
chr18	28684355	28684755	-0.217542591	6.08E-01	same
chr4	773878	774278	0.20998703	6.09E-01	same
chr7	135714945	135715345	-0.158516027	6.09E-01	same
chr3	132376949	132377349	-0.215259314	6.10E-01	same
chr7	133081761	133082161	-0.195798672	6.11E-01	same
chr5	151757954	151758354	-0.212934112	6.11E-01	same
chr13	57582390	57582790	0.209503941	6.11E-01	same
chr1	62532323	62532723	-0.206525918	6.11E-01	same
chr10	121087638	121088038	-0.211836441	6.12E-01	same
chr11	96411298	96411698	-0.213077389	6.13E-01	same
chr3	42819713	42820113	-0.207641656	6.13E-01	same
chr1	211723875	211724275	0.20939531	6.13E-01	same
chr2	238124646	238125046	0.195688668	6.13E-01	same
chr14	76904468	76904868	0.207026721	6.13E-01	same
chr7	30593957	30594357	-0.178484272	6.14E-01	same
chr2	24490196	24490596	-0.192437444	6.15E-01	same
chr3	128077524	128077924	0.212735956	6.15E-01	same
chr3	97764391	97764791	-0.191186127	6.15E-01	same
chr3	136818692	136819092	0.199988673	6.15E-01	same
chr6	148342502	148342902	-0.199598931	6.17E-01	same
chr11	65983474	65983874	-0.19518484	6.17E-01	same
chr1	8026836	8027236	-0.197021706	6.17E-01	same
chr12	130839309	130839709	-0.181236705	6.19E-01	same
chr6	93088580	93088980	-0.20678744	6.20E-01	same
chr4	155666894	155667294	-0.200958864	6.21E-01	same
chr12	112296394	112296794	0.195962161	6.21E-01	same
chr19	33720182	33720582	0.197414533	6.21E-01	same
chr15	48878001	48878401	-0.200663799	6.21E-01	same
chrUn_GL000224v1	3733	4133	0.161042326	6.22E-01	same
chr9	122940578	122940978	-0.189940099	6.22E-01	same
chr13	75009424	75009824	-0.171654725	6.22E-01	same
chr17	60142719	60143119	-0.17612915	6.23E-01	same
chr17	7107549	7107949	0.196973603	6.24E-01	same
chr19	40921237	40921637	0.186568928	6.24E-01	same
chrUn_GL000220v1	142721	143121	0.181172141	6.24E-01	same
chr13	86731525	86731925	-0.194270967	6.25E-01	same
chr1	116878233	116878633	0.20137484	6.26E-01	same
chr19	38683777	38684177	-0.176254905	6.26E-01	same
chr1	198935254	198935654	0.182835505	6.26E-01	same
chr11	113664224	113664624	-0.189585545	6.26E-01	same
chr11	18721283	18721683	-0.180534637	6.27E-01	same
chr6	49462719	49463119	-0.19300413	6.27E-01	same
chr16	81615374	81615774	-0.197803843	6.28E-01	same
chr19	47778252	47778652	-0.171126309	6.28E-01	same
chr1	228089944	228090344	-0.172983736	6.29E-01	same
chr1	62266256	62266656	-0.18236315	6.29E-01	same
chr7	101308241	101308641	-0.196678183	6.34E-01	same
chr19	7069332	7069732	-0.160760383	6.34E-01	same
chr7	56106220	56106620	-0.187001739	6.35E-01	same
chr6	26258140	26258540	-0.184197382	6.35E-01	same
chr13	60163784	60164184	0.159181414	6.35E-01	same
chr15	97295926	97296326	-0.193673774	6.35E-01	same
chr6	71084530	71084930	-0.198269171	6.36E-01	same

chr17	82042815	82043215	-0.166442837	6.36E-01	same
chr1	55277161	55277561	0.180735146	6.37E-01	same
chr11	62124314	62124714	-0.17265939	6.38E-01	same
chr13	79581763	79582163	0.17236957	6.39E-01	same
chr2	100562625	100563025	-0.163629544	6.40E-01	same
chr20	59116436	59116836	0.188739265	6.40E-01	same
chr13	61857713	61858330	0.142572138	6.41E-01	same
chr5	117455117	117455517	-0.188262278	6.41E-01	same
chr7	151043240	151043640	-0.16989131	6.41E-01	same
chr13	93891646	93892046	0.175975789	6.44E-01	same
chr1	149904648	149905048	-0.177180299	6.44E-01	same
chr19	36687352	36687752	-0.18691735	6.44E-01	same
chr1	233635980	233636380	-0.172315677	6.45E-01	same
chr10	1004536	1004936	0.191641907	6.46E-01	same
chr12	113114574	113114974	-0.169831576	6.46E-01	same
chr15	75023489	75023889	-0.162621504	6.46E-01	same
chr17	74960248	74960648	-0.153564153	6.47E-01	same
chr15	36579386	36579786	-0.185163189	6.47E-01	same
chr12	119894338	119894738	0.166922093	6.50E-01	same
chr17	69394520	69394920	-0.188930363	6.52E-01	same
chr15	84749330	84749730	-0.157024181	6.52E-01	same
chr13	66803246	66803646	-0.15412083	6.52E-01	same
chr15	65401197	65401597	-0.164089212	6.52E-01	same
chr12	56594205	56594605	0.176953155	6.53E-01	same
chr3	183099224	183099624	-0.191117269	6.54E-01	same
chr6	36678609	36679009	-0.174904712	6.55E-01	same
chr10	129076590	129076990	-0.184736676	6.55E-01	same
chr13	63700185	63700585	-0.160830427	6.55E-01	same
chr5	8208326	8208726	-0.190998673	6.55E-01	same
chr6	55913644	55914044	-0.190321334	6.56E-01	same
chr6_GL000256v2_alt	28896	29296	-0.165206112	6.56E-01	same
chr8	73136150	73136550	-0.17948909	6.58E-01	same
chr19	45398281	45398681	-0.159998844	6.58E-01	same
chr17	64774293	64774693	-0.127657822	6.59E-01	same
chr19	58409399	58409799	-0.180487233	6.59E-01	same
chr19	52942269	52942669	-0.188242842	6.59E-01	same
chr16	90032056	90032456	-0.178545152	6.60E-01	same
chrX	130810914	130811314	-0.184021558	6.61E-01	same
chr5	76257419	76257819	0.175130327	6.62E-01	same
chr1	76868157	76868557	-0.164370821	6.63E-01	same
chr9	70413971	70414371	-0.159267186	6.65E-01	same
chr7	116723831	116724231	-0.172070141	6.68E-01	same
chr10	119786298	119786698	-0.176986548	6.68E-01	same
chr22	37266921	37267321	-0.176297621	6.68E-01	same
chr3	51982583	51982983	0.148226945	6.69E-01	same
chr6	167651369	167651769	-0.182123031	6.69E-01	same
chr7	87600343	87600743	-0.160857359	6.71E-01	same
chr4	98260645	98261045	-0.175453225	6.71E-01	same
chr6	42547419	42547819	-0.17113519	6.71E-01	same
chr2	117814483	117814883	-0.155379798	6.72E-01	same
chr12	85280389	85280789	-0.162876104	6.73E-01	same
chr4_GL000008v2_random	2522	2922	0.14625092	6.73E-01	same
chr14	21846455	21846855	-0.171486986	6.75E-01	same
chr13	63606001	63606401	0.164229947	6.76E-01	same
chr8	17923062	17923462	-0.163441199	6.77E-01	same
chr22	39388412	39388812	-0.154304874	6.77E-01	same
chr1	39955293	39955693	-0.166787066	6.77E-01	same
chr13	62045858	62046258	-0.140468473	6.78E-01	same
chr1	224503486	224503886	0.148132196	6.78E-01	same
chr2	11483367	11483767	-0.167171353	6.79E-01	same
chr11	122580705	122581105	-0.173502741	6.81E-01	same
chr1	16236279	16236679	-0.162409229	6.81E-01	same
chr18	32724232	32724632	-0.147692288	6.81E-01	same
chr19	46009547	46009947	0.156843324	6.82E-01	same
chr13	71286702	71287102	-0.162346508	6.82E-01	same
chr13	69924486	69924886	0.173366335	6.82E-01	same
chr2	97645601	97646001	-0.151550641	6.85E-01	same
chr13	66617090	66617490	0.160443068	6.86E-01	same
chr20	35771588	35771988	-0.154783839	6.88E-01	same
chr5	131659613	131660013	-0.161052113	6.89E-01	same
chr16	28212294	28212694	-0.159828521	6.89E-01	same
chr15	88586628	88587028	0.154205258	6.89E-01	same
chr16	70346830	70347230	-0.151006075	6.89E-01	same
chr3	184812059	184812459	-0.150967971	6.90E-01	same
chr1	116405742	116406142	0.16411111	6.90E-01	same
chrX	74614243	74614643	-0.155894686	6.91E-01	same
chr1	173715002	173715402	-0.138313126	6.91E-01	same

chr22	30634868	30635268	0.150405795	6.91E-01	same
chr1	70487494	70487894	0.14496545	6.92E-01	same
chr19	57947399	57947799	-0.157271782	6.93E-01	same
chr7	130010199	130010599	-0.157572937	6.96E-01	same
chr13	71364312	71364712	0.153637767	6.97E-01	same
chr1	112923593	112923993	-0.161981246	6.97E-01	same
chr13	75733400	75733800	0.15366103	6.97E-01	same
chr6	165663584	165663984	-0.164900716	6.97E-01	same
chr12	49265346	49265746	-0.157356591	6.98E-01	same
chr12	106075151	106075551	-0.151097657	7.00E-01	same
chr6	34392784	34393184	-0.149974426	7.01E-01	same
chr6	98948013	98948413	0.159432609	7.02E-01	same
chr1	38569128	38569528	0.15706337	7.07E-01	same
chr9_KI270718v1_random	37509	37909	-0.137716471	7.07E-01	same
chr12	50084909	50085309	-0.150474998	7.07E-01	same
chr5	83251773	83252173	0.15810723	7.08E-01	same
chr21	31410255	31410655	0.144568165	7.09E-01	same
chr5	10601447	10601847	-0.153819659	7.09E-01	same
chr12	69484490	69484890	-0.1527543	7.10E-01	same
chrX	122913926	122914326	0.151394139	7.10E-01	same
chr4	55841935	55842335	0.15449106	7.13E-01	same
chr13	67651980	67652380	-0.144798368	7.13E-01	same
chr13	26222235	26222635	0.149847673	7.13E-01	same
chr10	17914516	17914916	-0.129506786	7.14E-01	same
chr17	75524005	75524405	-0.131869076	7.14E-01	same
chr3	14452590	14452990	-0.142510446	7.14E-01	same
chr1	182484292	182484692	-0.150737698	7.15E-01	same
chr5	95711422	95711822	-0.130483717	7.15E-01	same
chr18	3296886	3297286	0.152723959	7.15E-01	same
chrX	103831734	103832134	0.139588971	7.16E-01	same
chr20	3159415	3159815	0.138307545	7.16E-01	same
chr9	129468396	129468796	0.141772506	7.16E-01	same
chr8	120123684	120124084	-0.150063814	7.17E-01	same
chr13	72029226	72029626	0.146556372	7.17E-01	same
chr18	34331206	34331606	-0.141504518	7.18E-01	same
chr10	87735112	87735512	-0.148051016	7.18E-01	same
chr19	52139557	52139957	0.144975925	7.18E-01	same
chr20	9067878	9068278	-0.148951213	7.19E-01	same
chr2	55111842	55112242	0.153176186	7.20E-01	same
chr2	219541615	219542015	-0.145484873	7.20E-01	same
chr2	127370931	127371331	0.143648965	7.20E-01	same
chr20	543734	544134	-0.139532062	7.20E-01	same
chr11	370923	371323	-0.145291476	7.22E-01	same
chr21	32043542	32043942	-0.137528587	7.23E-01	same
chr6	123841301	123841701	-0.12565537	7.24E-01	same
chr2	122274559	122274959	0.135099402	7.25E-01	same
chr17	79785228	79785628	0.134254113	7.25E-01	same
chrX	65595336	65595736	-0.149055756	7.27E-01	same
chr1	151225379	151225779	0.145420832	7.27E-01	same
chr6	96521756	96522156	-0.134053015	7.28E-01	same
chr17	75270219	75270619	-0.109750837	7.28E-01	same
chr21	42840839	42841239	-0.123161317	7.28E-01	same
chr18	28175734	28176134	-0.136368372	7.30E-01	same
chr7	122284434	122284834	-0.144394925	7.32E-01	same
chr17	81969397	81969797	-0.134648956	7.32E-01	same
chr15	80444978	80445378	-0.134821295	7.33E-01	same
chr20	58623567	58623967	-0.134186001	7.33E-01	same
chr3	39152226	39152626	0.119042064	7.33E-01	same
chr2	3755025	3755425	-0.13800062	7.36E-01	same
chr4	174189592	174189992	-0.12346789	7.36E-01	same
chr6	111552338	111552738	-0.118845084	7.37E-01	same
chr1	63285870	63286270	0.131608216	7.37E-01	same
chr19	36772721	36773121	-0.123930952	7.38E-01	same
chr8	117191518	117191918	-0.129677766	7.39E-01	same
chr16	67833503	67833903	-0.12326579	7.39E-01	same
chr6	169973627	169974027	0.140989647	7.40E-01	same
chr9	137153245	137153645	-0.132261225	7.40E-01	same
chr5	4973651	4974051	0.140104109	7.41E-01	same
chr13	77737355	77737755	0.105077165	7.41E-01	same
chr4	48269894	48270294	-0.13211478	7.42E-01	same
chr1	81032739	81033139	-0.123467894	7.42E-01	same
chr6	43522935	43523335	-0.110548632	7.43E-01	same
chr7	74890730	74891130	0.138646294	7.43E-01	same
chr6	169621308	169621708	-0.120661105	7.43E-01	same
chr1	239408139	239408539	-0.12110765	7.44E-01	same
chr7	144111329	144111729	-0.130545685	7.44E-01	same
chr3	107697606	107698006	-0.134525247	7.45E-01	same

chr1	112957036	112957436	-0.112192213	7.47E-01	same
chr15	40507163	40507563	-0.127888856	7.48E-01	same
chr4	75573537	75573937	-0.127931448	7.51E-01	same
chr11	73983017	73983417	-0.105509061	7.51E-01	same
chr6	167826468	167826868	-0.121274178	7.52E-01	same
chr13	62512221	62512621	-0.123452177	7.52E-01	same
chr14_GL000225v1_random	13777	14177	-0.134312902	7.52E-01	same
chr17	43124996	43125396	0.115440257	7.52E-01	same
chr12	132986310	132986710	-0.126773773	7.53E-01	same
chrUn_KI270466v1	697	1097	0.055191263	7.53E-01	same
chr7	26858040	26858440	-0.133180245	7.54E-01	same
chr12	93570558	93570958	-0.126822507	7.55E-01	same
chr16	25049610	25050010	0.11430419	7.56E-01	same
chr17	16285915	16286315	0.115111882	7.56E-01	same
chr8	23083820	23084220	-0.13106224	7.56E-01	same
chr16	4668727	4669127	-0.114798839	7.57E-01	same
chr13	83462037	83462437	0.125393037	7.57E-01	same
chr10	46786796	46787196	-0.131686705	7.57E-01	same
chr6	83511894	83512294	-0.129570983	7.57E-01	same
chr15	38251666	38252066	0.128020219	7.61E-01	same
chrX	83819345	83819745	-0.129188918	7.61E-01	same
chr3	40524473	40524873	-0.116867428	7.62E-01	same
chr14	105448717	105449117	0.117035848	7.62E-01	same
chr1	35715368	35715768	-0.107009865	7.62E-01	same
chr6	125748258	125748658	-0.114663279	7.63E-01	same
chr22	36606696	36607096	-0.126516873	7.64E-01	same
chr2	27771702	27772102	-0.120271298	7.64E-01	same
chr1	65002185	65002585	-0.116413749	7.64E-01	same
chr5	134005016	134005416	-0.113179667	7.65E-01	same
chr9	113221076	113221476	-0.109472192	7.65E-01	same
chr9	122159385	122159785	-0.119686851	7.65E-01	same
chr15	40857773	40858173	-0.118045312	7.66E-01	same
chr13	64256861	64257261	-0.116108967	7.66E-01	same
chr13	80882065	80882465	-0.102881147	7.67E-01	same
chr13	79791327	79791727	0.108121968	7.68E-01	same
chrX	86148501	86148901	-0.120840902	7.68E-01	same
chr2	37196850	37197250	-0.118662639	7.68E-01	same
chr19	23250245	23250645	-0.114224395	7.69E-01	same
chr22	20108153	20108553	-0.097770348	7.70E-01	same
chr9	122827982	122828382	-0.105139978	7.71E-01	same
chr10	92756760	92757160	-0.120082675	7.72E-01	same
chr7	64313991	64314391	-0.120360652	7.72E-01	same
chr10	67629628	67630028	-0.120460095	7.73E-01	same
chr1	113146977	113147377	-0.114802072	7.73E-01	same
chr10	87845613	87846013	0.109714709	7.73E-01	same
chr2	222476883	222477283	-0.105590135	7.75E-01	same
chr16	66750531	66750931	-0.114764723	7.76E-01	same
chr10	119178489	119178889	-0.099894754	7.76E-01	same
chr13	62105787	62106187	-0.099636187	7.79E-01	same
chr8	100927185	100927585	-0.109102757	7.79E-01	same
chr19	4511060	4511460	-0.114326601	7.80E-01	same
chr19	35758154	35758554	-0.093530512	7.81E-01	same
chr4	98929572	98929972	0.10639695	7.82E-01	same
chr1	232037724	232038124	-0.101329201	7.83E-01	same
chr6	41767120	41767520	-0.09495644	7.84E-01	same
chr4	102718908	102719308	-0.110506317	7.84E-01	same
chr22	44617233	44617633	-0.114373636	7.85E-01	same
chr11	65559971	65560371	-0.104284119	7.86E-01	same
chr12	66234173	66234573	0.104104925	7.86E-01	same
chr10	71235810	71236210	0.105193766	7.86E-01	same
chr8	96518742	96519142	-0.103708444	7.88E-01	same
chr18	35410775	35411175	0.102565263	7.88E-01	same
chr3	160870153	160870553	-0.11336174	7.88E-01	same
chr10	117268852	117269252	0.101626829	7.88E-01	same
chr21	33551968	33552368	-0.088478214	7.90E-01	same
chrX	78024666	78025066	-0.112973957	7.90E-01	same
chr4	180566372	180566772	-0.107790847	7.90E-01	same
chr17	81508514	81508914	-0.078206435	7.91E-01	same
chr19	3429340	3429740	-0.110553862	7.93E-01	same
chr10	62804352	62804752	-0.102695109	7.94E-01	same
chr3	50627623	50628023	0.099797596	7.94E-01	same
chr20	3818740	3819140	-0.101954959	7.95E-01	same
chr1	67105656	67106056	-0.104441366	7.95E-01	same
chr2	85666100	85666500	-0.097917915	7.96E-01	same
chr8	120444216	120444616	-0.106920046	7.96E-01	same
chr14	22766647	22767047	0.10100255	7.96E-01	same
chr12	118181968	118182368	-0.096611188	7.97E-01	same

chr19	7643562	7643962	-0.106270652	7.97E-01	same
chr3	150106947	150107347	0.097650253	7.98E-01	same
chr17_KI270729v1_random	4425	4825	0.100239714	7.98E-01	same
chr22	22942434	22942834	-0.106476165	7.99E-01	same
chr14	61762350	61762750	-0.093375567	7.99E-01	same
chr13	66298600	66299000	0.100189593	7.99E-01	same
chr14	35808587	35808987	-0.09534115	7.99E-01	same
chr9	36765771	36766171	-0.098203551	7.99E-01	same
chr19	38544057	38544457	-0.096462666	8.00E-01	same
chr3	61250126	61250526	-0.10270275	8.02E-01	same
chr5	180213332	180213732	0.096111934	8.03E-01	same
chr14	103921173	103921573	-0.098575884	8.04E-01	same
chrX	106966521	106966921	-0.092345204	8.04E-01	same
chr21	32393043	32393443	-0.08502805	8.05E-01	same
chr3	43565820	43566220	-0.101968446	8.05E-01	same
chr1	244395169	244395569	0.076550229	8.08E-01	same
chr1	196723468	196723868	0.09806163	8.10E-01	same
chr16	23596559	23596959	-0.088593853	8.11E-01	same
chr2	224918733	224919133	0.095121875	8.11E-01	same
chr4	2330334	2330734	0.086055417	8.11E-01	same
chr2	217434231	217434631	-0.092169436	8.11E-01	same
chr19	13794875	13795275	-0.088553606	8.12E-01	same
chr5	102296324	102296724	-0.097738013	8.12E-01	same
chr1	202234377	202234777	-0.069147118	8.13E-01	same
chr17	17041733	17042133	-0.092587272	8.14E-01	same
chr12	123671086	123671486	0.085390421	8.15E-01	same
chr20	38590005	38590405	0.089254164	8.15E-01	same
chr10	104268688	104269088	0.084930448	8.15E-01	same
chr21	25607168	25607568	-0.091242765	8.16E-01	same
chr1	111740071	111740471	-0.090309313	8.16E-01	same
chrUn_KI270744v1	110665	111065	0.094349093	8.17E-01	same
chr5	172597188	172597588	-0.09585732	8.18E-01	same
chr1	81127114	81127514	-0.092917088	8.19E-01	same
chr6	19247051	19247451	-0.093185055	8.20E-01	same
chr2	120277286	120277686	0.094716924	8.23E-01	same
chr18	39140652	39141052	-0.092015265	8.23E-01	same
chr6	52664335	52664735	-0.086495821	8.23E-01	same
chr4	77075913	77076313	-0.091890287	8.23E-01	same
chr14	97895904	97896304	-0.087064299	8.24E-01	same
chr7	6374077	6374477	-0.086584862	8.24E-01	same
chr1	189110656	189111056	-0.094845376	8.24E-01	same
chr1	192657669	192658069	0.091204933	8.24E-01	same
chr4	61372768	61373168	-0.088810661	8.25E-01	same
chr19	52922966	52923366	0.088676388	8.26E-01	same
chr9	127608086	127608486	-0.080970327	8.27E-01	same
chr21	33553483	33553883	-0.075717277	8.28E-01	same
chr20	62088014	62088414	-0.083971929	8.28E-01	same
chr14	69772683	69773083	-0.080143066	8.29E-01	same
chr11	102747100	102747500	0.090213151	8.29E-01	same
chr6	28446946	28447346	-0.081801261	8.30E-01	same
chr5	59357698	59358098	-0.082034952	8.30E-01	same
chr19	11813853	11814253	0.087529943	8.30E-01	same
chr5	148384140	148384540	0.089282966	8.30E-01	same
chr10	72273499	72273899	-0.063389579	8.31E-01	same
chr10	117084460	117084860	0.088465473	8.32E-01	same
chr19	48660880	48661280	-0.077842673	8.32E-01	same
chr16	69424495	69424895	-0.077608871	8.32E-01	same
chr17	81524634	81525034	-0.076474292	8.33E-01	same
chr13	95676595	95676995	-0.080432883	8.36E-01	same
chr12	98361094	98361494	0.086589537	8.37E-01	same
chr7	103380960	103381360	0.077847045	8.37E-01	same
chr1	234098329	234098729	0.087050673	8.37E-01	same
chr2	85679061	85679461	-0.07491103	8.38E-01	same
chr12	96316037	96316437	0.084284913	8.39E-01	same
chr4	67603392	67603792	0.086338838	8.39E-01	same
chr2	109187425	109187825	0.085797514	8.39E-01	same
chr1	74280643	74281043	-0.082919142	8.40E-01	same
chr17	2511932	2512332	-0.074216557	8.43E-01	same
chr8	93006367	93006767	0.082611265	8.43E-01	same
chr19	43332978	43333378	-0.070137443	8.46E-01	same
chr15	64356216	64356616	-0.075331208	8.47E-01	same
chr12	46133882	46134282	-0.080191982	8.47E-01	same
chr11	28107637	28108037	0.075630998	8.47E-01	same
chr1	16860119	16860519	-0.073021971	8.48E-01	same
chr17	73437817	73438217	0.079965229	8.49E-01	same
chr12	14257885	14258285	0.072971294	8.49E-01	same
chrX	121231825	121232225	0.078856852	8.50E-01	same

chr1	174238726	174239126	0.077962989	8.50E-01	same
chr22	41245859	41246259	-0.068058558	8.51E-01	same
chr12	110552145	110552545	-0.073684611	8.53E-01	same
chr20	57082983	57083383	0.076337795	8.55E-01	same
chr7	104933005	104933405	-0.074680331	8.56E-01	same
chr17	61517923	61518323	-0.070612279	8.56E-01	same
chr7	132647558	132647958	0.066955534	8.57E-01	same
chr1	161647532	161647932	0.068830858	8.57E-01	same
chr12	10525686	10526086	-0.074997348	8.58E-01	same
chr8	54858826	54859226	-0.071751683	8.59E-01	same
chr21	33801499	33801899	0.066982464	8.60E-01	same
chr10	99779768	99780168	0.074826876	8.61E-01	same
chr4	88105047	88105447	0.074020389	8.61E-01	same
chr6	44845678	44846078	-0.07397617	8.62E-01	same
chr14	68191601	68192001	0.06682791	8.62E-01	same
chr17	81976628	81977028	-0.069774313	8.62E-01	same
chr1	84224732	84225132	-0.070607007	8.63E-01	same
chr14	22983058	22983458	-0.060690083	8.66E-01	same
chr5	174020007	174020407	-0.065877375	8.68E-01	same
chr21	45845308	45845708	0.070127545	8.68E-01	same
chr21	40993767	40994167	-0.070060913	8.69E-01	same
chr4	123396841	123397241	-0.068039723	8.70E-01	same
chr3	196567824	196568224	-0.067570187	8.70E-01	same
chr1	119327580	119327980	-0.065440649	8.71E-01	same
chr13	65871690	65872090	-0.059188602	8.72E-01	same
chr13	87810842	87811242	-0.063850088	8.73E-01	same
chr7	54589761	54590161	-0.064129676	8.74E-01	same
chr10	100186049	100186449	-0.060791889	8.75E-01	same
chr1	101061477	101061877	0.066343169	8.75E-01	same
chr3	32239796	32240196	-0.059035866	8.76E-01	same
chrUn_KI270751v1	150069	150469	0.062293132	8.76E-01	same
chr16	11976164	11976564	-0.061999468	8.77E-01	same
chr1	159924944	159925344	-0.052782131	8.77E-01	same
chr3	141384478	141384878	-0.063314172	8.78E-01	same
chr15	101578313	101578713	0.052574204	8.79E-01	same
chr18	33666857	33667257	-0.058094919	8.79E-01	same
chr6	148578333	148578733	-0.06169514	8.79E-01	same
chr9	134339443	134339843	0.05948061	8.80E-01	same
chr19	35114674	35115074	-0.056222511	8.80E-01	same
chr5	169583671	169584071	-0.056865973	8.81E-01	same
chr3	149812710	149813110	-0.051707923	8.81E-01	same
chr13	62919508	62919908	0.056189506	8.83E-01	same
chr6	125791528	125791928	-0.054097275	8.83E-01	same
chr13	26557169	26557569	-0.058191103	8.84E-01	same
chr1	237309036	237309436	-0.061787058	8.84E-01	same
chr17	27695217	27695617	-0.051611845	8.85E-01	same
chr13	68856275	68856675	-0.059651793	8.86E-01	same
chr8	52713143	52713543	-0.053424348	8.87E-01	same
chr12	83642202	83642602	-0.059811112	8.88E-01	same
chr12	69243238	69243638	0.052355603	8.89E-01	same
chr13	64130402	64130802	0.049721032	8.89E-01	same
chr7	85071048	85071448	-0.055084107	8.90E-01	same
chr7	127447176	127447576	-0.057786311	8.91E-01	same
chr1	41395674	41396074	-0.053886221	8.92E-01	same
chr7	97016640	97017040	-0.050352847	8.92E-01	same
chr6	147202559	147202959	-0.050397597	8.92E-01	same
chr17_KI270857v1_alt	996571	996971	-0.057230862	8.93E-01	same
chr11	65418924	65419324	-0.038673687	8.94E-01	same
chr15	70181121	70181521	-0.055903074	8.95E-01	same
chr16	23452828	23453228	-0.048896405	8.95E-01	same
chr13	65844055	65844455	0.045332727	8.97E-01	same
chr12	102120127	102120527	0.042314608	9.00E-01	same
chr6	27865215	27865615	-0.040103984	9.02E-01	same
chrX	115968475	115968875	-0.051396327	9.02E-01	same
chr7	131897118	131897518	0.048641095	9.02E-01	same
chr9	94479402	94479802	-0.051572503	9.02E-01	same
chr16	73247531	73247931	-0.050845341	9.03E-01	same
chr5	133051941	133052341	-0.046261341	9.04E-01	same
chr2	84459196	84459596	-0.044951574	9.04E-01	same
chr11	66370914	66371314	-0.043265644	9.05E-01	same
chr1	169620969	169621369	-0.04868226	9.06E-01	same
chr13	67156493	67156893	0.042513285	9.07E-01	same
chrUn_KI270591v1	5062	5462	-0.045755116	9.09E-01	same
chr15	64145666	64146066	0.047977641	9.09E-01	same
chr1	145155819	145156219	-0.04782546	9.09E-01	same
chr4	158847582	158847982	-0.047097279	9.11E-01	same
chr9	133238558	133238958	-0.043384087	9.11E-01	same

chr1	170074530	170074930	0.035727212	9.12E-01	same
chr1	35588876	35589276	-0.043034665	9.12E-01	same
chr7	2564984	2565384	-0.046163673	9.13E-01	same
chr6	77368843	77369243	-0.04195301	9.14E-01	same
chr4	114599361	114599761	-0.044434853	9.16E-01	same
chr3	138174535	138174935	-0.041385621	9.16E-01	same
chr12	95003378	95003778	-0.041745874	9.16E-01	same
chr1	93878876	93879276	-0.035661106	9.18E-01	same
chr2	176124962	176125362	-0.0434036	9.18E-01	same
chr7	155858140	155858540	0.037114186	9.19E-01	same
chr21	13986267	13986667	0.042046531	9.20E-01	same
chr13	87124134	87124534	-0.03588162	9.21E-01	same
chr19	47241403	47241803	-0.040578147	9.22E-01	same
chr1	68576158	68576558	-0.040560033	9.22E-01	same
chr13	84126831	84127231	0.038877943	9.23E-01	same
chr7	55204614	55205014	0.039144582	9.23E-01	same
chr1	222825707	222826107	-0.036025566	9.25E-01	same
chrUn_KI270310v1	487	887	-0.037802187	9.26E-01	same
chr7	150003375	150003775	-0.027185076	9.29E-01	same
chr5	172303432	172303832	-0.034545234	9.30E-01	same
chr19	52897409	52897809	-0.036106585	9.30E-01	same
chr4	1580949	1581349	0.036600608	9.31E-01	same
chr17	21031484	21031884	0.03683104	9.31E-01	same
chr13	69235251	69235651	-0.03374705	9.31E-01	same
chr2	23673336	23673736	-0.032795597	9.32E-01	same
chrUn_GL000219v1	177095	177495	0.031679602	9.32E-01	same
chr19	40798441	40798841	-0.02935289	9.34E-01	same
chr1	120176072	120176472	-0.030465031	9.35E-01	same
chr10	6386865	6387265	0.030894749	9.35E-01	same
chr13	110715370	110715770	0.032286453	9.38E-01	same
chr13	64263570	64263970	0.02883299	9.39E-01	same
chr7	157839621	157840021	0.028580563	9.40E-01	same
chr10	132139074	132139474	0.03166601	9.40E-01	same
chr1	179806313	179806713	0.030184358	9.41E-01	same
chr1	171645026	171645426	-0.027650648	9.41E-01	same
chr7	49438356	49438756	-0.02843138	9.42E-01	same
chr10	14449212	14449612	-0.029327004	9.43E-01	same
chr11	62771329	62771729	-0.024182889	9.43E-01	same
chr3	19946883	19947283	0.026097311	9.44E-01	same
chr20	63537764	63538164	0.026855854	9.45E-01	same
chr1	52054910	52055310	0.026580222	9.46E-01	same
chr10	120966134	120966534	0.025526927	9.46E-01	same
chr15	76259437	76259837	0.02800844	9.46E-01	same
chr10	123008864	123009264	0.025384702	9.46E-01	same
chr19	37655597	37655997	-0.025527887	9.48E-01	same
chr9	130288107	130288507	0.027462691	9.48E-01	same
chr6	45501288	45501688	-0.026354178	9.50E-01	same
chr17	76547988	76548388	0.022729044	9.51E-01	same
chr2	200526250	200526650	-0.0238476	9.52E-01	same
chr8	53692965	53693365	-0.02418201	9.52E-01	same
chr1	185257235	185257635	-0.022527352	9.52E-01	same
chr9	130186884	130187284	-0.023838561	9.54E-01	same
chr8	79783565	79783965	0.02132174	9.55E-01	same
chr7	98294920	98295320	0.019319774	9.56E-01	same
chr22	46639321	46639721	0.0220122	9.56E-01	same
chr6	127593498	127593898	-0.02215367	9.57E-01	same
chr19	4036271	4036671	0.018022275	9.58E-01	same
chrX	123798168	123798568	-0.021166118	9.58E-01	same
chr2	70108462	70108862	-0.019688403	9.60E-01	same
chr6	170264606	170265006	-0.020248186	9.61E-01	same
chr6	164925868	164926268	0.020188033	9.61E-01	same
chr7	156053392	156053792	-0.019374719	9.62E-01	same
chr1	161832490	161832890	0.018935871	9.62E-01	same
chr11	20112329	20112729	0.019234825	9.62E-01	same
chr1	36613648	36614048	-0.019332409	9.63E-01	same
chr17	10697221	10697621	0.018997867	9.63E-01	same
chr15	78396371	78396771	0.017438398	9.67E-01	same
chr18	33510305	33510705	0.015726363	9.67E-01	same
chr1	74325070	74325470	0.016857582	9.68E-01	same
chr22_KI270736v1_random	171248	171648	0.017043959	9.68E-01	same
chr14	54773600	54774000	0.015367506	9.68E-01	same
chr1	202236971	202237371	0.015556916	9.69E-01	same
chr17	46977225	46977625	-0.014984281	9.71E-01	same
chr4	89542100	89542500	-0.014518342	9.72E-01	same
chr11	125949846	125950246	0.014668118	9.72E-01	same
chr2	216098703	216099103	0.013516594	9.72E-01	same
chr17	43913084	43913484	-0.011744545	9.73E-01	same

chr11	122655534	122655934	-0.012638043	9.74E-01	same
chr2	27211288	27211688	-0.01213646	9.75E-01	same
chr13	65818298	65818698	0.0119342	9.75E-01	same
chr10	50739869	50740269	0.012448546	9.75E-01	same
chr10	132964933	132965333	0.012888553	9.75E-01	same
chr1	244487099	244487499	0.01262864	9.76E-01	same
chr17	68200788	68201188	-0.010660376	9.76E-01	same
chr16	2105236	2105636	-0.0121457	9.76E-01	same
chr13	61942778	61943178	0.008476787	9.76E-01	same
chr17	40087047	40087447	0.010824599	9.77E-01	same
chr16	1839587	1839987	0.01044343	9.79E-01	same
chr2	1306669	1307069	-0.010276239	9.79E-01	same
chr10	97687607	97688007	-0.00992722	9.80E-01	same
chr1	228818611	228819011	-0.010083085	9.80E-01	same
chr13	61918538	61918938	-0.006715155	9.83E-01	same
chr17	39547766	39548166	-0.008473839	9.83E-01	same
chr8	63124703	63125103	0.007466694	9.85E-01	same
chr1	75943294	75943694	0.007060483	9.86E-01	same
chr7	91800893	91801293	0.007193539	9.86E-01	same
chr11	11002103	11002503	-0.007219935	9.86E-01	same
chr1	67811487	67811887	-0.006893453	9.87E-01	same
chr1	12019461	12019861	-0.006116415	9.87E-01	same
chr1	235586818	235587218	-0.006200001	9.88E-01	same
chrX	130959019	130959419	0.006379322	9.88E-01	same
chr16	22232621	22233021	-0.005129533	9.89E-01	same
chr13	88895862	88896262	-0.005610308	9.89E-01	same
chr6	149202259	149202659	-0.005609311	9.89E-01	same
chr2	100004762	100005162	-0.005121752	9.90E-01	same
chr11	57271056	57271456	-0.005470578	9.90E-01	same
chr14	59576490	59576890	-0.004661588	9.91E-01	same
chr13	63976247	63976647	0.004582918	9.91E-01	same
chr15	79452209	79452609	-0.004227887	9.91E-01	same
chr16	5640488	5640888	-0.004086779	9.92E-01	same
chr10	77471484	77471884	-0.004066558	9.92E-01	same
chr14_GL000225v1_random	32324	32724	0.003960676	9.93E-01	same
chr1	62690317	62690717	-0.003070952	9.93E-01	same
chr14	35046416	35046816	-0.00266155	9.95E-01	same
chr13	88153010	88153410	-0.002589194	9.95E-01	same
chr10	100228782	100229182	-0.000729301	9.98E-01	same
chrUn_KI270751v1	140484	140884	0.000402705	9.99E-01	same
chr22	38606490	38606890	0.000286583	9.99E-01	same
chr16	84616841	84617241	-1.28245093	9.82E-04	decrease
chr7	121023648	121024048	-1.251816184	3.42E-03	decrease
chr18	25351902	25352302	-1.207152455	4.24E-03	decrease
chr18	51029830	51030230	-1.184381263	4.90E-03	decrease
chr2	127886316	127886716	-1.094625079	7.03E-03	decrease
chr8	83934162	83934562	-1.144042342	7.37E-03	decrease
chr6	104969745	104970145	-1.116471688	8.86E-03	decrease
chr20	46938888	46939288	-1.103591709	9.70E-03	decrease
chr12	91381896	91382296	-1.03695315	9.75E-03	decrease
chr12	40733961	40734361	-1.073879526	1.18E-02	decrease
chr5	64769134	64769534	1.044061809	1.25E-02	increase
chr4	185142766	185143166	-1.040878172	1.41E-02	decrease
chr14	60349952	60350352	-1.028019374	1.42E-02	decrease
chr2	70549168	70549568	-1.043353538	1.47E-02	decrease
chr5	160009051	160009451	-1.035661499	1.50E-02	decrease
chr2	78417769	78418169	-1.005787624	1.66E-02	decrease
chr22	39040742	39041142	-1.014021224	1.69E-02	decrease
chr7	143836492	143836892	-0.854601612	1.96E-02	decrease
chr5	17379315	17379715	-0.958792144	2.27E-02	decrease
chr5	40798265	40798665	-0.957712439	2.40E-02	decrease
chr11	9705231	9705631	-0.771775258	2.60E-02	decrease
chr7	112715327	112715727	-0.82457455	2.74E-02	decrease
chr12	66678785	66679185	0.871311188	2.94E-02	increase
chrUn_KI270435v1	91924	92324	-0.818165151	3.05E-02	decrease
chr14	63854092	63854492	-0.911552753	3.12E-02	decrease
chr10	87342437	87342837	-0.801114631	3.27E-02	decrease
chr19	491915	492315	-0.78992982	3.43E-02	decrease
chr17	48805086	48805486	-0.898108397	3.48E-02	decrease
chr1	53392086	53392486	-0.879355388	3.50E-02	decrease
chr1	149103342	149103742	-0.790313483	3.51E-02	decrease
chr2	40361418	40361818	-0.887988243	3.71E-02	decrease
chr8	54134636	54135036	-0.879065705	3.97E-02	decrease
chr5	90208552	90208952	-0.841650205	4.71E-02	decrease
chr5	110738690	110739090	-0.840081901	4.95E-02	decrease
chr9	135365967	135366367	0.830451956	5.03E-02	same
chr1_KI270714v1_random	9724	10124	-0.710437379	5.31E-02	same

chr13	43787749	43788149	-0.826103697	5.34E-02	same
chr7	136582276	136582676	0.652479621	5.52E-02	same
chr4	107826967	107827367	-0.812735707	5.53E-02	same
chrX	81202277	81202677	-0.816642627	5.60E-02	same
chrUn_GL000216v2	147030	147430	-0.645363013	5.72E-02	same
chr5	115117625	115118025	0.784395524	5.78E-02	same
chr6	114805371	114805771	-0.780568593	6.19E-02	same
chr13	24511453	24511853	-0.776993476	6.33E-02	same
chr7	122850699	122851099	0.791430507	6.38E-02	same
chr10	3808847	3809247	-0.788119762	6.51E-02	same
chr12	49264884	49265284	-0.780246591	6.64E-02	same
chr20	38962615	38963015	-0.778625176	6.68E-02	same
chr7	85185586	85185986	-0.782513804	6.71E-02	same
chr14_GL000225v1_random	112456	112856	0.579648697	6.73E-02	same
chr11	43312491	43312891	-0.77599759	6.95E-02	same
chr2	185864690	185865090	-0.753315164	6.97E-02	same
chr9	19465480	19465880	-0.773232388	7.04E-02	same
chr1	827208	827608	-0.652215292	7.36E-02	same
chrX	136762828	136763228	-0.761865529	7.41E-02	same
chrX	45850627	45851027	-0.749938648	7.55E-02	same
chr3	479646	480046	0.722767549	7.94E-02	same
chr5	54431436	54431836	-0.747276713	7.94E-02	same
chrUn_KI270304v1	684	1084	-0.610026735	8.71E-02	same
chr11	30567176	30567576	-0.717665473	8.88E-02	same
chr17	62148656	62149056	0.721966844	9.09E-02	same
chr9	136632692	136633092	0.577131192	9.26E-02	same
chr6_GL000252v2_alt	1197944	1198344	-0.586300116	9.38E-02	same
chr22	22357375	22357775	-0.694906552	9.74E-02	same
chr12	12266970	12267370	-0.702577734	9.83E-02	same
chr9	88076238	88076638	-0.701840469	1.01E-01	same
chr6_GL000256v2_alt	35227	35627	-0.590260324	1.01E-01	same
chr12	20090656	20091056	-0.69065379	1.06E-01	same
chr7	25047325	25047725	-0.679162864	1.06E-01	same
chr14_KI270724v1_random	327	727	0.562363727	1.09E-01	same
chr5	59762338	59762738	0.606570783	1.13E-01	same
chr8	66176640	66177040	-0.675199729	1.14E-01	same
chr15	44287884	44288284	-0.666961303	1.14E-01	same
chr17	65438625	65439025	-0.664521948	1.18E-01	same
chr2	740066	740466	-0.660635833	1.18E-01	same
chr17	64837401	64837801	-0.664587941	1.20E-01	same
chr6	5891790	5892190	-0.659124694	1.22E-01	same
chr2	185424898	185425298	-0.660590594	1.22E-01	same
chr10	50993058	50993458	-0.6586621	1.24E-01	same
chr18	8707387	8707787	-0.608291545	1.25E-01	same
chr11	116691859	116692259	-0.651320136	1.25E-01	same
chr15	63916316	63916716	-0.652663185	1.25E-01	same
chr20	25067471	25067871	0.600060674	1.28E-01	same
chr1	201257933	201258333	0.469683561	1.29E-01	same
chr7	26857563	26857963	-0.645662402	1.31E-01	same
chr5	110739090	110739490	-0.617207661	1.32E-01	same
chr14	56447214	56447614	-0.49404683	1.34E-01	same
chr9	39464306	39464706	-0.531144381	1.37E-01	same
chr3	12003954	12004354	-0.612243738	1.41E-01	same
chr19	14288936	14289336	-0.62134608	1.44E-01	same
chr13	20482258	20482658	-0.623574781	1.44E-01	same
chr2	187934582	187934982	-0.622213944	1.45E-01	same
chr17_GL000205v2_random	140614	141014	-0.589228048	1.53E-01	same
chr6	76876566	76876966	-0.60798971	1.55E-01	same
chr17	50297488	50297888	-0.601420175	1.58E-01	same
chr15	83383758	83384158	0.588937154	1.65E-01	same
chr2	214683973	214684373	-0.561750266	1.69E-01	same
chr22_KI270733v1_random	153554	153954	-0.58167977	1.73E-01	same
chr5	58557654	58558054	0.569807099	1.74E-01	same
chr8	142482297	142482697	-0.552540261	1.74E-01	same
chr11	123653756	123654156	-0.578294973	1.75E-01	same
chr10	72612047	72612447	-0.578317605	1.76E-01	same
chr14_GL000225v1_random	133527	133927	0.546881349	1.78E-01	same
chr7	158667958	158668358	0.532766468	1.79E-01	same
chr1	174848790	174849190	0.550549036	1.79E-01	same
chr16	70065884	70066284	-0.572945538	1.80E-01	same
chr10	60573118	60573518	-0.562872225	1.82E-01	same
chr6	122301939	122302339	0.568824196	1.84E-01	same
chr11	72470858	72471258	-0.564016907	1.87E-01	same
chr17	18681995	18682395	-0.55768053	1.87E-01	same
chr15	48855378	48855778	-0.557152099	1.89E-01	same
chr6_GL000253v2_alt	56068	56468	-0.49833378	1.90E-01	same
chr1	154696412	154696812	0.558573413	1.91E-01	same

chr12	51308209	51308609	-0.551716675	1.96E-01	same
chr1	246785645	246786045	0.410260197	2.01E-01	same
chr12	132383511	132383911	-0.52159565	2.04E-01	same
chr1	65564080	65564480	0.529738723	2.04E-01	same
chr8	138206296	138206696	-0.322635662	2.08E-01	same
chr17	32066651	32067051	-0.536661991	2.08E-01	same
chr7	77142319	77142719	0.476984406	2.08E-01	same
chr13	29491771	29492171	-0.536371586	2.09E-01	same
chr16	58087466	58087866	0.530251602	2.14E-01	same
chr6	32624791	32625191	-0.526809064	2.18E-01	same
chr10	132158393	132158793	-0.473426221	2.19E-01	same
chr4	119454875	119455275	-0.509023898	2.21E-01	same
chr1	148048336	148048736	-0.480565232	2.21E-01	same
chr2	133293259	133293659	-0.467885155	2.22E-01	same
chr17_KI270729v1_random	22497	22897	-0.462366101	2.25E-01	same
chr2	203534123	203534523	-0.507987636	2.31E-01	same
chr14_GL000225v1_random	41368	41768	0.511790694	2.31E-01	same
chr15	77951591	77951991	0.346189473	2.40E-01	same
chr7	65770458	65770858	-0.496839145	2.45E-01	same
chr1_KI270706v1_random	165430	165830	0.491499238	2.46E-01	same
chr6_GL000255v2_alt	73087	73487	-0.315155882	2.47E-01	same
chr1	8712635	8713035	-0.491141579	2.47E-01	same
chr6	166783716	166784116	0.467126549	2.51E-01	same
chr18	22232404	22232804	0.46504565	2.51E-01	same
chrX	125006200	125006600	-0.484272732	2.52E-01	same
chr14_GL000225v1_random	49425	49825	0.480915522	2.60E-01	same
chr16	29106137	29106537	-0.453801977	2.62E-01	same
chr9	137121590	137121990	-0.478631952	2.63E-01	same
chr3	108575407	108575807	0.456153604	2.67E-01	same
chr2	86719903	86720303	-0.400405455	2.69E-01	same
chr21	46133612	46134012	-0.469088645	2.72E-01	same
chr19	57190970	57191370	0.459433187	2.73E-01	same
chr4	7387341	7387741	-0.418336847	2.73E-01	same
chr15	40519861	40520261	-0.467752862	2.73E-01	same
chr17_KI270907v1_alt	94375	94775	-0.378925061	2.83E-01	same
chr4	707689	708089	0.33456392	2.83E-01	same
chr15	38454224	38454624	-0.456221299	2.85E-01	same
chrUn_KI270589v1	42908	43308	0.419525473	2.86E-01	same
chr2	196905469	196905869	-0.441638233	2.88E-01	same
chr19	57291460	57291860	-0.44901913	2.92E-01	same
chr21	37756464	37756864	-0.400629533	2.93E-01	same
chr11_KI270927v1_alt	163394	163794	0.341463883	2.95E-01	same
chr12	132793480	132793880	-0.425053159	2.98E-01	same
chr22	28663273	28663673	0.443278091	3.00E-01	same
chr6	82940107	82940507	-0.435537106	3.02E-01	same
chr16	1025215	1025615	-0.422436287	3.02E-01	same
chr6	28597506	28597906	-0.409447477	3.05E-01	same
chr10	77519815	77520215	0.429137173	3.11E-01	same
chr2_KI270772v1_alt	5552	5952	0.318382271	3.13E-01	same
chr17_GL000205v2_random	87947	88347	-0.423510377	3.18E-01	same
chr12	71564340	71564740	0.336003703	3.25E-01	same
chr4	119454303	119454703	-0.408385205	3.26E-01	same
chr16	86845500	86845900	0.415757071	3.26E-01	same
chr5	33049811	33050211	0.42003122	3.26E-01	same
chr17	74465340	74465740	-0.41778448	3.29E-01	same
chr4	186546663	186547063	-0.358891797	3.30E-01	same
chr9	41359154	41359554	-0.403472705	3.34E-01	same
chr14_GL000194v1_random	114659	115059	-0.353302572	3.35E-01	same
chr14	24007260	24007660	-0.410420232	3.37E-01	same
chr6_GL000256v2_alt	44588	44988	-0.353118629	3.37E-01	same
chr1	184616584	184616984	-0.404512339	3.43E-01	same
chr1	176132725	176133125	0.392224346	3.45E-01	same
chrUn_GL000195v1	30837	31237	-0.39415501	3.47E-01	same
chr7_KI270804v1_alt	105042	105442	-0.302657508	3.51E-01	same
chr14_KI270723v1_random	35116	35516	0.278986076	3.51E-01	same
chr4	72400085	72400485	0.365731376	3.56E-01	same
chr22	17104097	17104497	-0.380435123	3.57E-01	same
chr4	54050262	54050662	-0.391611871	3.57E-01	same
chr12	129302904	129303304	-0.376269886	3.62E-01	same
chr8	7020857	7021257	0.387753171	3.62E-01	same
chr5	753919	754319	-0.233734374	3.66E-01	same
chr6	80053908	80054308	0.383919383	3.69E-01	same
chr1	120558583	120558983	-0.306046688	3.80E-01	same
chr8	38012479	38012879	-0.373535603	3.82E-01	same
chr10	794079	794479	-0.309593772	3.83E-01	same
chr17	15563245	15563645	-0.371289668	3.85E-01	same
chr4	137167635	137168035	0.246870701	3.90E-01	same

chr2	197640195	197640595	-0.365464185	3.91E-01	same
chr10	46396573	46396973	0.365455717	3.92E-01	same
chr8	144946757	144947157	0.298387745	3.95E-01	same
chr14	88935786	88936186	0.356402262	4.04E-01	same
chr12	132732798	132733198	0.354813841	4.06E-01	same
chr12	51401517	51401917	0.354399524	4.07E-01	same
chr21	30741942	30742342	-0.323384654	4.08E-01	same
chr3	876267	876667	0.225562776	4.08E-01	same
chr10	119001815	119002215	0.3484052	4.12E-01	same
chr1	145161103	145161503	-0.236148838	4.12E-01	same
chr14	30363629	30364029	0.331637224	4.13E-01	same
chr21	28875150	28875550	-0.342866573	4.14E-01	same
chr12	131296790	131297190	-0.334946948	4.17E-01	same
chr6	49410048	49410448	-0.336794011	4.22E-01	same
chr3	146702695	146703095	0.342253687	4.23E-01	same
chr9	76906245	76906645	-0.341251033	4.24E-01	same
chr12	3198160	3198560	-0.298961992	4.26E-01	same
chr15	84360479	84360879	-0.281230834	4.31E-01	same
chr14	44916376	44916776	0.296949365	4.31E-01	same
chr6	169247463	169247863	0.336360224	4.31E-01	same
chr9	132377471	132377871	0.283734418	4.36E-01	same
chr1	210891441	210891841	0.331642742	4.37E-01	same
chr9_KI270720v1_random	655	1055	0.317413198	4.37E-01	same
chr6	117244746	117245146	-0.331557353	4.38E-01	same
chr5	121962077	121962477	-0.329656332	4.41E-01	same
chrUn_GL000214v1	129209	129609	0.220154282	4.43E-01	same
chr19	32746402	32746802	-0.286679811	4.44E-01	same
chr7	74932662	74933062	-0.309961674	4.45E-01	same
chr16	53054421	53054821	0.323267382	4.50E-01	same
chr17	18781085	18781485	-0.320597843	4.52E-01	same
chr1	162790346	162790746	-0.307247472	4.54E-01	same
chr7	14903217	14903617	-0.31564315	4.58E-01	same
chr12	129105301	129105701	0.272804255	4.58E-01	same
chr7	156265578	156265978	-0.243279303	4.59E-01	same
chr1	149842971	149843371	0.312871015	4.60E-01	same
chr10	63267530	63267930	-0.3144944	4.62E-01	same
chr7	44039141	44039541	-0.311113383	4.63E-01	same
chr16	88933545	88933945	0.294302118	4.69E-01	same
chrUn_KI270744v1	120436	120836	-0.307514316	4.70E-01	same
chr11	120328598	120328998	-0.303533383	4.78E-01	same
chr2	74330527	74330927	0.301496754	4.81E-01	same
chr1_KI270709v1_random	1589	1989	0.24896615	4.81E-01	same
chr2	130898305	130898705	0.300461494	4.81E-01	same
chr19	350828	351228	-0.186826251	4.82E-01	same
chr11	319150	319550	-0.293951876	4.84E-01	same
chr8	144767834	144768234	-0.29489425	4.86E-01	same
chr17	82958920	82959320	0.292838977	4.92E-01	same
chr8	38013133	38013533	-0.291972243	4.94E-01	same
chr5	1539758	1540158	0.26697461	4.94E-01	same
chr12	105995806	105996206	0.289643092	4.96E-01	same
chr3	163891936	163892336	0.282549079	4.98E-01	same
chr14_GL000225v1_random	46469	46869	0.283072873	5.07E-01	same
chr14_GL000225v1_random	56266	56666	0.282309533	5.07E-01	same
chr7_KI270899v1_alt	12242	12642	0.173501214	5.07E-01	same
chr1	86105460	86105860	-0.28259065	5.09E-01	same
chrUn_KI270442v1	371648	372048	-0.277040199	5.09E-01	same
chr14_GL000225v1_random	127094	127494	-0.275825288	5.17E-01	same
chr4	3227432	3227832	0.217624939	5.18E-01	same
chr2	155600122	155600522	0.271673697	5.21E-01	same
chr1	146400453	146400853	0.257321885	5.26E-01	same
chrUn_KI270329v1	151	551	0.106749366	5.27E-01	same
chr6	168659650	168660050	0.26869605	5.29E-01	same
chr14_KI270723v1_random	11103	11503	-0.248700584	5.30E-01	same
chr1	16872506	16872906	-0.184135483	5.32E-01	same
chr18	49066938	49067338	0.245854921	5.34E-01	same
chr12	130887754	130888154	-0.256776338	5.37E-01	same
chr22_KI270733v1_random	24786	25186	-0.229279309	5.38E-01	same
chr9	137874855	137875255	-0.261905867	5.38E-01	same
chr9	94074736	94075136	-0.254119041	5.41E-01	same
chr8	11456206	11456606	-0.219077207	5.43E-01	same
chr2	117044134	117044534	-0.259430873	5.44E-01	same
chr9	73313211	73313611	0.251744385	5.44E-01	same
chr8	25216025	25216425	0.1871961	5.44E-01	same
chr21	45932287	45932687	0.253133596	5.45E-01	same
chr6	87306581	87306981	-0.224239806	5.47E-01	same
chr4	167394081	167394481	0.211515252	5.54E-01	same
chr4	1283138	1283538	-0.212998407	5.57E-01	same

chr2	71416769	71417169	-0.21828676	5.58E-01	same
chr4	118888837	118889237	-0.242862681	5.60E-01	same
chr12	132688444	132688844	-0.21457833	5.61E-01	same
chr5	4080704	4081104	0.247746409	5.61E-01	same
chr10	3458356	3458756	0.229204561	5.62E-01	same
chr9	108247740	108248140	0.242749594	5.64E-01	same
chr1	120415000	120415400	-0.241824119	5.65E-01	same
chr4	188231403	188231803	0.239245627	5.67E-01	same
chrUn_KI270330v1	1047	1447	0.186408663	5.69E-01	same
chr9	137101960	137102360	-0.241810044	5.72E-01	same
chr1	148951592	148951992	0.240668903	5.72E-01	same
chr1	223948431	223948831	-0.239462794	5.75E-01	same
chrUn_GL000220v1	144832	145232	-0.227122553	5.76E-01	same
chr1_KI270711v1_random	8631	9031	0.237043405	5.77E-01	same
chr1	154714194	154714594	-0.237922214	5.78E-01	same
chr9	4713784	4714184	0.235337875	5.82E-01	same
chr17	81792863	81793263	0.234291537	5.84E-01	same
chr1	16862010	16862410	-0.138582559	5.85E-01	same
chr9	137102769	137103169	-0.17978572	5.91E-01	same
chr14_GL000225v1_random	25961	26361	-0.219141559	6.00E-01	same
chrUn_KI270757v1	15675	16075	-0.205624289	6.03E-01	same
chr5	167096853	167097253	-0.221025032	6.04E-01	same
chr4	102463997	102464397	-0.198746485	6.06E-01	same
chr10	7377999	7378399	-0.199912714	6.14E-01	same
chr2	241637284	241637684	-0.201831214	6.17E-01	same
chr4	141811201	141811601	-0.200933363	6.20E-01	same
chr1	149850435	149850835	-0.210982666	6.21E-01	same
chr7	159208796	159209196	0.186237537	6.22E-01	same
chr1	224378633	224379033	0.207667285	6.24E-01	same
chr1	16545616	16546016	-0.19422791	6.28E-01	same
chr6	139413464	139413864	0.202812255	6.31E-01	same
chr1	228620994	228621394	-0.148810213	6.32E-01	same
chr22_KI270733v1_random	139007	139407	-0.196796472	6.33E-01	same
chr5	179658210	179658610	-0.202756974	6.34E-01	same
chr16	81314325	81314725	-0.200603809	6.37E-01	same
chr1	152305952	152306352	0.199814495	6.38E-01	same
chr10	47328995	47329395	0.1272079	6.41E-01	same
chr2	71618064	71618464	-0.194097891	6.42E-01	same
chr9	77248515	77248915	-0.17868106	6.53E-01	same
chr6	57821409	57821809	-0.166149174	6.54E-01	same
chr5	118542248	118542648	-0.189790306	6.55E-01	same
chr14_GL000225v1_random	16897	17297	0.189464932	6.57E-01	same
chr17	841437	841837	0.148933257	6.63E-01	same
chr8	90684732	90685132	-0.178609455	6.64E-01	same
chr17_KI270729v1_random	27524	27924	0.146288214	6.70E-01	same
chr11_KI270902v1_alt	83661	84061	-0.161328917	6.73E-01	same
chr17_KI270730v1_random	108601	109001	-0.176397301	6.76E-01	same
chr11	26965418	26965818	-0.150279665	6.78E-01	same
chr19	37751512	37751912	0.116805696	6.79E-01	same
chr6	40509629	40510029	-0.173672754	6.84E-01	same
chr14_GL000225v1_random	85528	85928	-0.168946015	6.85E-01	same
chrX	114162293	114162693	-0.172975031	6.86E-01	same
chr6_GL000256v2_alt	73318	73718	-0.144257925	6.88E-01	same
chr3	78496784	78497184	0.167698181	6.88E-01	same
chr10	131486490	131486890	0.145376148	6.90E-01	same
chr6	95758619	95759019	-0.168250273	6.94E-01	same
chr14	90653067	90653467	-0.165988459	6.98E-01	same
chr1	228556318	228556718	-0.162622335	6.98E-01	same
chrUn_KI270538v1	53651	54051	0.124057281	7.01E-01	same
chr1	121097363	121097763	-0.104273553	7.01E-01	same
chr14_GL000225v1_random	70595	70995	-0.159262742	7.02E-01	same
chr3	132536107	132536507	-0.162310108	7.04E-01	same
chr5	4144640	4145040	0.161284665	7.05E-01	same
chr17	82586281	82586681	-0.160343053	7.06E-01	same
chrUn_KI270538v1	80997	81397	0.160062297	7.07E-01	same
chr1	171373609	171374009	0.159039093	7.10E-01	same
chr1	145541302	145541702	0.156545204	7.14E-01	same
chr14	92441228	92441628	0.155951351	7.15E-01	same
chr5	180658325	180658725	-0.144458758	7.19E-01	same
chr5	181045814	181046214	0.137359527	7.20E-01	same
chr3	57448946	57449346	-0.142721644	7.20E-01	same
chrX	115290167	115290567	0.153151445	7.20E-01	same
chr4	8620981	8621381	-0.146300684	7.21E-01	same
chr9	136802778	136803178	0.135512593	7.22E-01	same
chr4	80843571	80843971	0.151360595	7.22E-01	same
chr14_GL000225v1_random	128981	129381	0.14343522	7.22E-01	same
chr2	2580358	2580758	0.151755051	7.23E-01	same

chr22_KI270735v1_random	40306	40706	0.140182911	7.26E-01	same
chr1	95701201	95701601	-0.147695733	7.28E-01	same
chr14_GL000225v1_random	95318	95718	0.146725267	7.31E-01	same
chr13	33925076	33925476	0.135769435	7.31E-01	same
chr4	88539340	88539740	0.145458719	7.34E-01	same
chr10	132821834	132822234	-0.139093105	7.35E-01	same
chrUn_KI270589v1	42050	42450	0.143665165	7.37E-01	same
chr1_KI270709v1_random	3526	3926	0.133157622	7.40E-01	same
chr20	46788884	46789284	0.11923156	7.41E-01	same
chr2	9405928	9406328	0.132796884	7.41E-01	same
chr10	11284174	11284574	0.116676068	7.41E-01	same
chr1	236097048	236097448	0.139072785	7.45E-01	same
chr16	618777	619177	-0.12342914	7.54E-01	same
chr5	344372	344772	0.114830116	7.55E-01	same
chr2	231841763	231842163	0.115390815	7.58E-01	same
chr14_KI270723v1_random	36869	37269	0.119765696	7.62E-01	same
chr10	264939	265339	0.129033523	7.62E-01	same
chr1	245102967	245103367	0.111592465	7.65E-01	same
chr7	157648786	157649186	-0.111543379	7.65E-01	same
chr16	904107	904507	0.05578164	7.66E-01	same
chr6	6261974	6262374	0.110137388	7.66E-01	same
chr2	77593006	77593406	-0.126426705	7.66E-01	same
chr2	239281764	239282164	-0.117624711	7.68E-01	same
chr6	44711005	44711405	-0.120998512	7.69E-01	same
chrUn_KI270744v1	122448	122848	-0.12491444	7.70E-01	same
chr22_KI270736v1_random	180486	180886	-0.122734067	7.72E-01	same
chr7	23035709	23036109	-0.122432392	7.73E-01	same
chr11	134522184	134522584	-0.123381344	7.73E-01	same
chr6_GL000256v2_alt	105645	106045	-0.11548455	7.73E-01	same
chrUn_KI270465v1	721	1121	0.11747844	7.78E-01	same
chrUn_GL000216v2	5667	6067	-0.092046632	7.90E-01	same
chr20	48514662	48515062	-0.070709887	7.91E-01	same
chr6	151498754	151499154	-0.111504871	7.91E-01	same
chrUn_GL000220v1	7316	7716	-0.074329903	8.04E-01	same
chr8	67241988	67242388	-0.104946497	8.06E-01	same
chr7	157657704	157658104	-0.098917934	8.17E-01	same
chr1	201257154	201257554	-0.083266317	8.20E-01	same
chr12	100636448	100636848	-0.074249967	8.21E-01	same
chr1	155687334	155687734	-0.069574915	8.24E-01	same
chr10	45971829	45972229	0.089058804	8.35E-01	same
chr11	102374579	102374979	-0.088586175	8.36E-01	same
chrUn_KI270303v1	959	1359	0.085205663	8.41E-01	same
chr3	168807154	168807554	-0.082624043	8.47E-01	same
chr1	222107608	222108008	0.079209428	8.53E-01	same
chr18	63198716	63199116	0.078134241	8.55E-01	same
chr1	31431712	31432112	0.077963484	8.55E-01	same
chrUn_GL000216v2	163340	163740	-0.063326826	8.57E-01	same
chr16	9313251	9313651	-0.074627979	8.59E-01	same
chr1	42167240	42167640	-0.074315073	8.62E-01	same
chrUn_KI270544v1	378	778	-0.071259792	8.64E-01	same
chr22	15628383	15628783	0.072103145	8.66E-01	same
chr6	100196582	100196982	-0.06848927	8.67E-01	same
chr16	50579702	50580102	-0.066485007	8.68E-01	same
chr9	39809795	39810195	0.069417969	8.71E-01	same
chrUn_KI270337v1	198	598	-0.058098448	8.71E-01	same
chr14_GL000225v1_random	40427	40827	0.069134359	8.72E-01	same
chr7	158493866	158494266	0.068665652	8.72E-01	same
chr2	118142390	118142790	-0.064674794	8.79E-01	same
chr2	232899663	232900063	-0.058998405	8.82E-01	same
chr7	77063773	77064173	-0.054921604	8.83E-01	same
chr7	157469967	157470367	-0.062866438	8.83E-01	same
chr12	89827189	89827589	0.059032973	8.90E-01	same
chr4	1356974	1357374	-0.058907984	8.90E-01	same
chr1	163037604	163038004	-0.05531741	8.91E-01	same
chr7	65081058	65081458	-0.056786949	8.94E-01	same
chr1	176313854	176314254	-0.054380593	8.97E-01	same
chr20	64088528	64088928	-0.050071442	9.02E-01	same
chr20	47535371	47535771	-0.051161948	9.03E-01	same
chr2	18295430	18295830	0.044268852	9.03E-01	same
chr8	27490681	27491081	0.05083466	9.03E-01	same
chr20	16288467	16288867	-0.045495441	9.04E-01	same
chr9	113061407	113061807	-0.048516333	9.07E-01	same
chr1	161606397	161606797	0.04846975	9.10E-01	same
chr7	66292351	66292751	0.035770229	9.10E-01	same
chr17_KI270729v1_random	24016	24416	0.04621275	9.12E-01	same
chr17	70858802	70859202	0.039551325	9.15E-01	same
chr2	3304405	3304805	-0.044585242	9.16E-01	same

chr2	179397467	179397867	0.028958109	9.17E-01	same
chr18	26829194	26829594	0.041376152	9.17E-01	same
chr14_GL000225v1_random	92423	92823	-0.04165823	9.18E-01	same
chrUn_GL000216v2	173833	174233	0.036562841	9.19E-01	same
chr12	132816972	132817372	0.041702847	9.19E-01	same
chr9	34318548	34318948	-0.041310971	9.23E-01	same
chr4	174778479	174778879	-0.040190144	9.25E-01	same
chr7	158493131	158493531	-0.037506712	9.29E-01	same
chr2	7133328	7133728	0.033243174	9.34E-01	same
chr5	1781229	1781629	-0.034053392	9.37E-01	same
chr14_GL000225v1_random	6404	6804	0.031149707	9.39E-01	same
chr14_GL000225v1_random	134815	135215	0.020424267	9.41E-01	same
chr19	2281431	2281831	-0.028925122	9.44E-01	same
chr20	29884214	29884614	-0.027928086	9.46E-01	same
chr5	175397918	175398318	-0.028637526	9.46E-01	same
chr2	238767505	238767905	0.020966185	9.46E-01	same
chr3	162318956	162319356	0.020017997	9.46E-01	same
chr16	89587942	89588342	-0.023601592	9.46E-01	same
chr7	67129001	67129401	-0.027410003	9.48E-01	same
chr22	47170461	47170861	0.018338199	9.49E-01	same
chr12	63151847	63152247	-0.025002719	9.53E-01	same
chrUn_GL000216v2	159178	159578	0.013390579	9.57E-01	same
chr1	240184744	240185144	-0.022725193	9.57E-01	same
chr1	170426656	170427056	-0.02259867	9.58E-01	same
chr11	4175671	4176071	-0.022265039	9.58E-01	same
chr22	17876638	17877038	-0.021237765	9.59E-01	same
chr14_GL000225v1_random	50688	51088	-0.019323273	9.64E-01	same
chr10	131918227	131918627	0.018308561	9.66E-01	same
chr2	187758814	187759214	-0.017429559	9.67E-01	same
chr8	142390076	142390476	-0.016447048	9.69E-01	same
chr14_GL000225v1_random	118866	119266	0.010109414	9.69E-01	same
chr9	111387271	111387671	-0.015496122	9.71E-01	same
chrX	104725045	104725445	-0.014648088	9.73E-01	same
chr1	145459164	145459564	-0.00893044	9.83E-01	same
chr14_GL000225v1_random	33534	33934	0.007540744	9.86E-01	same
chr4	110233848	110234248	-0.006417303	9.88E-01	same
chr2	32110709	32111109	-0.004440886	9.92E-01	same
chr1	47104437	47104837	-0.00421139	9.92E-01	same
chrUn_GL000216v2	167720	168120	0.002930731	9.95E-01	same
chr9	134654267	134654667	0.002065274	9.96E-01	same
chr6	144677496	144677896	-0.000244422	1.00E+00	same
chr6_GL000256v2_alt	174930	175330	0	1.00E+00	same
chr7	148647928	148648328	0	1.00E+00	same

2) List of HDAC3 CUT&Tag peaks in 293T cells of *HsANKRD11-KD vs ctrl*

chrom	peak start	peak end	log2FC(*HsANKRD11-KD vs ctrl*)	p-value	change
chr1	62436463	62436863	-1.229048249	1.68871E-11	decrease
chr5	151771458	151771858	-1.326159354	5.36462E-10	decrease
chr1	113904794	113905194	-1.474092998	7.04792E-10	decrease
chr15	75451883	75452283	-1.284144896	1.36049E-09	decrease
chr19	45692236	45692636	-1.236138857	2.11121E-09	decrease
chr12	56117863	56118263	-1.445153213	2.55361E-09	decrease
chr2	197453230	197453630	-1.264907628	2.89607E-09	decrease
chr19	6425023	6425423	-1.204784692	3.39534E-09	decrease
chr17	59155295	59155695	-1.302019341	3.44768E-09	decrease
chr1	110338942	110339342	-1.202623053	3.80244E-09	decrease
chr5	138469570	138469970	1.618806707	5.90085E-09	increase
chr12	67268958	67269358	-1.30376962	7.0829E-09	decrease
chr19	5904301	5904701	-1.116489237	7.36376E-09	decrease
chr1	244970944	244971344	-1.350221733	9.88706E-09	decrease
chr16	81006974	81007374	-1.42080614	1.01838E-08	decrease
chr12	54280125	54280525	-1.35703505	1.40979E-08	decrease
chr5	96935272	96935672	-1.199758365	1.46857E-08	decrease
chr22	19479281	19479681	-1.309252195	1.74803E-08	decrease
chr2	218270113	218270513	-1.243289426	1.98965E-08	decrease
chr19	49676704	49677104	-1.133153571	2.37517E-08	decrease
chr14	58298048	58298448	-1.347561882	3.6416E-08	decrease
chr14	77320926	77321326	-1.358732147	4.39253E-08	decrease
chr16	30370623	30371023	-1.134745175	4.72777E-08	decrease
chr13	38349491	38349891	-1.261756832	5.80638E-08	decrease
chr3	120094587	120094987	-1.101648346	5.86528E-08	decrease
chr1	154974603	154975003	-1.109349684	6.63262E-08	decrease
chr1	58783958	58784358	-1.084389687	6.64263E-08	decrease
chr1	44730628	44731028	-1.402177516	7.12206E-08	decrease
chr1	235160966	235161366	-1.491404456	7.9035E-08	decrease
chr4	82374053	82374453	-1.274711761	9.04268E-08	decrease
chr1	225777569	225777969	-1.17976417	1.13417E-07	decrease
chr21	39183450	39183850	-1.294559028	1.18154E-07	decrease
chr12	53379471	53379871	-1.180188612	1.24904E-07	decrease
chr11	57667443	57667843	-1.1727876	1.37104E-07	decrease
chr21	33642003	33642403	-1.065997528	1.45462E-07	decrease
chr1	92785057	92785457	-1.138915822	1.80205E-07	decrease
chrX	130165859	130166259	-1.036489266	1.89299E-07	decrease
chr6	139374878	139375278	-1.222558099	2.03513E-07	decrease
chr19	1455694	1456094	-1.42785444	2.38697E-07	decrease
chr11	62840995	62841395	-0.919022809	2.41117E-07	decrease
chr1	231528275	231528675	-0.984423516	2.4497E-07	decrease
chr1	155688700	155689100	-1.512694525	2.50886E-07	decrease
chr17	51260086	51260486	-1.218027614	2.57699E-07	decrease
chr4	15681453	15681853	-1.388613106	2.68748E-07	decrease
chrX	48801886	48802286	-1.142990621	3.49478E-07	decrease
chr5	140401244	140401644	-1.109766568	3.67225E-07	decrease
chr19	49877152	49877552	-1.115949408	4.4635E-07	decrease
chr11	64185715	64186115	-1.170167833	4.49263E-07	decrease
chr22	42615367	42615767	-1.06398816	4.60495E-07	decrease
chr4	139453408	139453808	-1.403729985	4.9571E-07	decrease
chr16	30658232	30658632	-1.003108346	5.32506E-07	decrease
chr19	11381346	11381746	-1.197283046	5.53051E-07	decrease
chr19	42268676	42269076	-0.987527256	6.78612E-07	decrease
chr1	46303301	46303701	-1.280678214	7.09614E-07	decrease
chr16	29877477	29877877	-1.147879569	7.37821E-07	decrease
chrX	48897749	48898149	-1.217568125	7.60743E-07	decrease
chr15	89334916	89335316	-1.12493051	7.74168E-07	decrease
chr17	4967371	4967771	-0.963365475	8.29477E-07	decrease
chr13	75549410	75549810	-1.100115942	8.49641E-07	decrease
chr7	92833788	92834188	-1.493294112	8.78268E-07	decrease
chr1	150363774	150364174	-1.15717183	8.80258E-07	decrease
chr6	152983287	152983687	-1.067039262	8.80424E-07	decrease
chr1	77682440	77682840	-1.162876884	8.95767E-07	decrease
chr1	26692771	26693171	-1.010735598	9.2882E-07	decrease
chr2	86440781	86441181	-1.165837975	9.57855E-07	decrease
chr19	10717573	10717973	-1.230661682	9.91487E-07	decrease
chrUn_KI270467v1	2556	2956	0.716208269	1.04237E-06	increase
chr7	26200869	26201269	-1.148866696	1.10184E-06	decrease
chr3	153161285	153161685	-1.00298724	1.10953E-06	decrease
chr9	79571503	79571903	-1.020371847	1.14823E-06	decrease
chr14	20454813	20455213	-1.023722219	1.16702E-06	decrease
chr5	177006547	177006947	-1.390680948	1.18379E-06	decrease

chr3	31532204	31532604	-1.165467719	1.23185E-06	decrease
chr12	120291418	120291818	-1.03186208	1.28777E-06	decrease
chr6	110179761	110180161	-1.252902527	1.29137E-06	decrease
chr19	17266970	17267370	-1.113678764	1.30745E-06	decrease
chr17	19378026	19378426	-1.169287298	1.37382E-06	decrease
chr15	84980373	84980773	-1.038884119	1.49138E-06	decrease
chr10	69179922	69180322	-1.161858734	1.52758E-06	decrease
chr3	152834804	152835204	-1.522101804	1.55522E-06	decrease
chr4	102827907	102828307	-0.974227893	1.61615E-06	decrease
chr21	37267488	37267888	-1.237987195	1.78759E-06	decrease
chr19	48468933	48469333	-1.161863192	1.8654E-06	decrease
chr16	46689256	46689656	-1.129061149	2.04038E-06	decrease
chr2	27370046	27370446	-1.184429597	2.1182E-06	decrease
chr1	70410879	70411279	-1.05077209	2.20467E-06	decrease
chrX	47218086	47218486	-1.108654049	2.23268E-06	decrease
chr3	49724078	49724478	-1.357789847	2.27668E-06	decrease
chr17	48048103	48048503	-1.283734283	2.53403E-06	decrease
chr10	123154086	123154486	-1.40531616	2.58167E-06	decrease
chr6	137219122	137219522	-1.287593924	2.65499E-06	decrease
chr9	121074863	121075263	-1.348166398	2.71982E-06	decrease
chr9	71911258	71911658	-1.216018879	2.86874E-06	decrease
chr18	32092189	32092589	-1.242454247	2.89123E-06	decrease
chr1	169894006	169894406	-1.141883663	3.03729E-06	decrease
chr8	73293834	73294234	-0.926070314	3.0643E-06	decrease
chr14	74084416	74084816	-1.061749842	3.19812E-06	decrease
chrX	119791616	119792016	-1.151096749	3.20067E-06	decrease
chr1	71080991	71081391	-1.058306989	3.21242E-06	decrease
chrX	111681337	111681737	-1.448587593	3.27995E-06	decrease
chr13	75537797	75538197	-1.044961423	3.28966E-06	decrease
chr21	26843008	26843408	-1.192504562	3.40537E-06	decrease
chr6	110815524	110815924	-1.154105135	3.41759E-06	decrease
chr19	45886380	45886780	-1.323638561	3.45205E-06	decrease
chr3	152298710	152299110	-1.417892999	3.72874E-06	decrease
chr1	40691882	40692282	-1.385253622	3.81804E-06	decrease
chr17	7857285	7857685	-1.077952299	3.9972E-06	decrease
chr9	83980317	83980717	-1.130661883	4.00604E-06	decrease
chr5	150758686	150759086	-1.265381018	4.28814E-06	decrease
chr4	139015327	139015727	-1.1683603	4.29986E-06	decrease
chr3	114056452	114056852	-1.273897736	4.46593E-06	decrease
chr4	106316197	106316597	-0.984009745	4.47577E-06	decrease
chr19	10695640	10696040	-1.16552671	4.61736E-06	decrease
chr14	67600006	67600406	-1.321662292	4.61883E-06	decrease
chr1	32394442	32394842	-0.952919895	4.73394E-06	decrease
chr19	14136579	14136979	-1.249473452	4.90337E-06	decrease
chr16	1827178	1827578	-1.397487703	5.05278E-06	decrease
chr6	24720830	24721230	-0.998761751	5.10515E-06	decrease
chr19	10835843	10836243	-0.872059197	5.21528E-06	decrease
chr16	21952972	21953372	-1.139065263	5.25841E-06	decrease
chr11	95789868	95790268	-1.26223478	5.3653E-06	decrease
chr14	35826227	35826627	-1.255201965	5.48584E-06	decrease
chr5	173917869	173918269	-1.218472759	5.71125E-06	decrease
chr7	107168532	107168932	-1.126424769	5.90481E-06	decrease
chr17	64497255	64497655	-1.161820456	6.15149E-06	decrease
chr5	90474437	90474837	-1.299714902	6.15883E-06	decrease
chr11	64270057	64270457	-1.130771882	6.17061E-06	decrease
chrX	149540640	149541040	-1.13219364	6.28388E-06	decrease
chr8	11802326	11802726	-1.485878257	6.2992E-06	decrease
chr6	136250110	136250510	-1.13815679	6.31116E-06	decrease
chr4	101347788	101348188	-1.25780612	6.44656E-06	decrease
chr7	149028563	149028963	-1.080272681	6.66929E-06	decrease
chr10	102132614	102133014	-1.258732376	6.67444E-06	decrease
chr2	201071588	201071988	-1.218994988	6.8173E-06	decrease
chr5	103119984	103120384	-1.140298452	6.98325E-06	decrease
chr19	7605543	7605943	-1.255662602	7.28008E-06	decrease
chr19	5680579	5680979	-1.139720573	7.56283E-06	decrease
chr1	234724069	234724469	-1.034070369	7.61027E-06	decrease
chr9	2241885	2242285	-1.024252078	7.76674E-06	decrease
chr7	92133556	92133956	-1.355654931	8.21222E-06	decrease
chr19	1490429	1490829	-1.304787164	8.23784E-06	decrease
chr4	9152856	9153256	-1.108272159	8.25319E-06	decrease
chr19	55339361	55339761	-1.036340037	8.27021E-06	decrease
chr7	72828020	72828420	-1.202282049	8.31981E-06	decrease
chr2	73233415	73233815	-0.992028414	8.46893E-06	decrease
chr1	45583570	45583970	-1.125708096	8.5383E-06	decrease
chr6	136289667	136290067	-1.001479776	8.65391E-06	decrease
chr7	10973505	10973905	-1.203796946	8.72986E-06	decrease

chr19	49640322	49640722	-0.950119404	8.87523E-06	decrease
chr8	140464388	140464788	-1.023725831	8.90516E-06	decrease
chr19	49149738	49150138	-1.019269249	9.20442E-06	decrease
chr6	24774995	24775395	-1.178029223	9.2305E-06	decrease
chr16	67183490	67183890	-1.256296623	9.30396E-06	decrease
chr16	87950994	87951394	-1.089587164	9.52391E-06	decrease
chr14	105490791	105491191	-1.102518655	9.65816E-06	decrease
chr19	56140787	56141187	-1.135843239	9.82765E-06	decrease
chr1	28648863	28649263	-0.987583887	9.84097E-06	decrease
chr1	156338464	156338864	-0.931087674	9.85094E-06	decrease
chr1	244652631	244653031	-1.185930787	9.97208E-06	decrease
chr19	43463542	43463942	-1.147276766	1.01748E-05	decrease
chr12	104286710	104287110	-1.269062332	1.02743E-05	decrease
chr17	8173302	8173702	-1.126650614	1.03564E-05	decrease
chr19	38850213	38850613	-0.978568108	1.03961E-05	decrease
chr5	119070658	119071058	-1.104209025	1.06264E-05	decrease
chr9	37465480	37465880	-1.048723148	1.06771E-05	decrease
chr11	6234806	6235206	-1.100432015	1.11156E-05	decrease
chr7	2354065	2354465	-1.019773773	1.11249E-05	decrease
chr19	55117774	55118174	-1.384527326	1.12001E-05	decrease
chr11	130002591	130002991	-1.121539344	1.14163E-05	decrease
chr1	119711745	119712145	-1.475170061	1.15857E-05	decrease
chr22	36529174	36529574	-1.135718787	1.21948E-05	decrease
chr1	47314043	47314443	-1.121780224	1.2465E-05	decrease
chr19	47130762	47131162	-1.276611121	1.25092E-05	decrease
chr22	30591897	30592297	-1.071813456	1.25697E-05	decrease
chr6	41072480	41072880	-0.976260211	1.26714E-05	decrease
chr3	143971493	143971893	-1.233815548	1.31846E-05	decrease
chr1	32650524	32650924	-0.95353703	1.35716E-05	decrease
chr19	9834786	9835186	-1.225973658	1.36023E-05	decrease
chr6	155314479	155314879	-1.025571733	1.41921E-05	decrease
chr12	109999082	109999482	-1.234428697	1.45216E-05	decrease
chr2	32063406	32063806	-1.407288815	1.45352E-05	decrease
chr15	59657443	59657843	-1.438236877	1.52077E-05	decrease
chr7	100586171	100586571	-1.109109823	1.55686E-05	decrease
chr2	70086821	70087221	-1.413709891	1.5643E-05	decrease
chrX	103686709	103687109	-1.011426188	1.57728E-05	decrease
chr5	90409450	90409850	-1.114980141	1.62445E-05	decrease
chr2	38602842	38603242	-1.450803857	1.6313E-05	decrease
chr2	44168092	44168492	-1.048774394	1.65216E-05	decrease
chr5	399684	400084	-1.2436792	1.67364E-05	decrease
chr2	229922340	229922740	-1.28261178	1.67521E-05	decrease
chr9	35604139	35604539	-1.047348564	1.67627E-05	decrease
chr1	43946622	43947022	-1.039086611	1.69927E-05	decrease
chr17	8138900	8139300	-1.2073797	1.70446E-05	decrease
chr1	167714868	167715268	-1.028955741	1.7429E-05	decrease
chr13	110713038	110713438	-1.353454509	1.74474E-05	decrease
chr19	680655	681055	-1.062789813	1.74671E-05	decrease
chr2	676623	677023	-0.97177399	1.77288E-05	decrease
chr10	58268822	58269222	-1.005731643	1.77986E-05	decrease
chr1	228457760	228458160	-0.923554158	1.78355E-05	decrease
chr3	23916754	23917154	-1.124043433	1.7962E-05	decrease
chrX	49269404	49269804	-0.918167721	1.81897E-05	decrease
chr2	96266039	96266439	-1.129714315	1.82911E-05	decrease
chr3	57597368	57597768	-1.154894353	1.86069E-05	decrease
chr4	112231956	112232356	-1.174731789	1.86877E-05	decrease
chr12	55973628	55974028	-0.956185463	1.9785E-05	decrease
chrX	30652847	30653247	-1.35679877	1.99311E-05	decrease
chr2	222656039	222656439	-0.976819011	1.9978E-05	decrease
chr11	59615941	59616341	-1.186066992	2.06083E-05	decrease
chr17	7251993	7252393	-1.092885151	2.06231E-05	decrease
chr20	10434241	10434641	-0.97185261	2.07657E-05	decrease
chr2	189784007	189784407	-1.10415461	2.07813E-05	decrease
chr17	82367818	82368218	-0.93427441	2.15514E-05	decrease
chr16	31459428	31459828	-1.351207659	2.17512E-05	decrease
chr5	103258667	103259067	-1.332672225	2.18108E-05	decrease
chr1	86914074	86914474	-1.023185069	2.19294E-05	decrease
chr2	203238907	203239307	-1.285519389	2.22035E-05	decrease
chr9	37079590	37079990	-1.144775452	2.2229E-05	decrease
chr5	150449687	150450087	-1.083555618	2.28799E-05	decrease
chr17	59619327	59619727	-1.346456485	2.30522E-05	decrease
chr1	165768764	165769164	-1.216762556	2.31008E-05	decrease
chr5	66144356	66144756	-1.244817054	2.31092E-05	decrease
chr13	72727619	72728019	-0.901590488	2.32104E-05	decrease
chr7	44606480	44606880	-1.282993472	2.40298E-05	decrease
chr7	100335938	100336338	-1.399619265	2.40527E-05	decrease

chr15	65304837	65305237	-0.976886201	2.42126E-05	decrease
chr1	184754855	184755255	-0.953152431	2.45158E-05	decrease
chr15	75453601	75454001	-1.018786887	2.47814E-05	decrease
chr18	35497654	35498054	-0.944598738	2.4983E-05	decrease
chr1	67430472	67430872	-0.849925216	2.51345E-05	decrease
chr7	135510211	135510611	-1.012542918	2.51644E-05	decrease
chr1	145281295	145281695	-0.873421651	2.54553E-05	decrease
chr2	20051772	20052172	-1.027025007	2.5643E-05	decrease
chr17	60677422	60677822	-1.090955697	2.59491E-05	decrease
chr19	49813351	49813751	-1.115643585	2.60875E-05	decrease
chr13	49936129	49936529	-1.361796203	2.63819E-05	decrease
chr17	17281206	17281606	-1.398769967	2.6752E-05	decrease
chr1	153633967	153634367	-1.03677522	2.7327E-05	decrease
chr16	56451328	56451728	-1.101802725	2.75429E-05	decrease
chr2	55232301	55232701	-1.040698163	2.76038E-05	decrease
chr17	17237085	17237485	-1.006235806	2.82453E-05	decrease
chr20	2102277	2102677	-1.016821736	2.83385E-05	decrease
chr3	123585145	123585545	-1.074515286	2.83615E-05	decrease
chr1	44819871	44820271	-1.11172518	2.9153E-05	decrease
chr13	31162125	31162525	-1.053944185	2.98979E-05	decrease
chr1	235504571	235504971	-0.827316818	3.00572E-05	decrease
chr14	64387865	64388265	-1.126019038	3.00807E-05	decrease
chr19	50804619	50805019	-0.970038341	3.0924E-05	decrease
chr17	75205348	75205748	-0.923983984	3.10786E-05	decrease
chr16	58630179	58630579	-0.961707828	3.13715E-05	decrease
chr7	5523513	5523913	-1.163776292	3.14595E-05	decrease
chrX	101407777	101408177	-1.056562023	3.20074E-05	decrease
chr2	121074938	121075338	-1.19267966	3.20384E-05	decrease
chr6	42890663	42891063	-1.095812886	3.20636E-05	decrease
chr7	99504578	99504978	-1.165391938	3.25921E-05	decrease
chr14	24114668	24115068	-0.942556673	3.26082E-05	decrease
chr19	1812476	1812876	-0.944570732	3.26147E-05	decrease
chr13	27251235	27251635	-1.120850206	3.28737E-05	decrease
chr7	123557731	123558131	-1.314392938	3.30423E-05	decrease
chr13	60163739	60164139	-1.117839334	3.31163E-05	decrease
chr5	33440610	33441010	-0.983095072	3.34572E-05	decrease
chr9	124771281	124771681	-1.167038278	3.38375E-05	decrease
chr3	9363029	9363429	-1.093824959	3.39531E-05	decrease
chr1	248825926	248826326	-1.202788509	3.41752E-05	decrease
chr11	77994723	77995123	-1.101001462	3.43204E-05	decrease
chr1	93180113	93180513	-1.060068364	3.44663E-05	decrease
chr16	30762062	30762462	-0.934645131	3.44799E-05	decrease
chr1	81801091	81801491	-1.153786674	3.45215E-05	decrease
chr16	57299986	57300386	-0.952914407	3.57128E-05	decrease
chr14	67674432	67674832	-1.458841733	3.5931E-05	decrease
chr14	34982522	34982922	-1.055257503	3.60116E-05	decrease
chr1	154936917	154937317	-0.82982824	3.6271E-05	decrease
chr7	23299165	23299565	-1.349179989	3.6644E-05	decrease
chr19	57849541	57849941	-1.329915876	3.6714E-05	decrease
chr17	63842578	63842978	-1.00940513	3.67718E-05	decrease
chr19	41859829	41860229	-1.015936698	3.74158E-05	decrease
chr19	16659947	16660347	-0.998995303	3.7875E-05	decrease
chr12	56636432	56636832	-1.029179062	3.79832E-05	decrease
chr12	76878938	76879338	-1.364541064	3.80452E-05	decrease
chr17	47895808	47896208	-1.113787167	3.81744E-05	decrease
chr1	55215597	55215997	-1.344411165	3.87495E-05	decrease
chr2	197705286	197705686	-1.392147711	3.90484E-05	decrease
chr19	47047886	47048286	-1.026539222	3.91585E-05	decrease
chr7	100852274	100852674	-1.32005112	3.93125E-05	decrease
chr1	225924109	225924509	-1.219686103	3.9493E-05	decrease
chr12	53501166	53501566	-0.932163979	3.96285E-05	decrease
chr14	24141576	24141976	-0.866960666	3.96479E-05	decrease
chr19	49453210	49453610	-0.998412323	4.07775E-05	decrease
chr7	76359237	76359637	-1.077104651	4.12179E-05	decrease
chr16	30378183	30378583	-0.886470604	4.12544E-05	decrease
chr17	59106799	59107199	-1.173619464	4.1282E-05	decrease
chr5	146446889	146447289	-1.169530945	4.21196E-05	decrease
chr14	44961902	44962302	-0.945457088	4.21876E-05	decrease
chr2	65130016	65130416	-1.232829409	4.23561E-05	decrease
chrX	52995607	52996007	-1.03046525	4.26079E-05	decrease
chr16	3150636	3151036	-1.039003744	4.32143E-05	decrease
chr1	244834923	244835323	-1.109969964	4.37219E-05	decrease
chr10	68900969	68901369	-1.118472193	4.4072E-05	decrease
chr16	30571856	30572256	-1.158535428	4.44689E-05	decrease
chr2	130342548	130342948	-0.870769421	4.46149E-05	decrease
chr2	207625870	207626270	-1.320560611	4.69367E-05	decrease

chr9	99221915	99222315	-0.972554001	4.7013E-05	decrease
chr6	110874422	110874822	-1.043386805	4.71061E-05	decrease
chr1	234479149	234479549	-1.339545222	4.74536E-05	decrease
chr19	36114931	36115331	-1.015378676	4.75E-05	decrease
chr17	2401422	2401822	-0.90496462	4.75893E-05	decrease
chr19	36214612	36215012	-1.026311196	4.77854E-05	decrease
chr6	52284738	52285138	-1.18038504	4.80777E-05	decrease
chr2	86195123	86195523	-1.122088612	4.85868E-05	decrease
chr4	56435618	56436018	-0.961660726	4.88048E-05	decrease
chr17	78140342	78140742	-0.925271161	4.88889E-05	decrease
chr13	50082208	50082608	-1.098047138	4.89461E-05	decrease
chr18	32018463	32018863	-0.938910204	4.89651E-05	decrease
chr6	26285424	26285824	-1.075647305	4.95695E-05	decrease
chr1	173824478	173824878	-0.979739193	4.96352E-05	decrease
chr1	150629185	150629585	-0.850322873	4.96943E-05	decrease
chr4	2962675	2963075	-1.202510075	4.97341E-05	decrease
chr3	101724563	101724963	-0.953959188	4.97721E-05	decrease
chr7	77798377	77798777	-1.014792064	5.1078E-05	decrease
chr5	146203313	146203713	-1.109758639	5.18788E-05	decrease
chr1	75785988	75786388	-0.859024463	5.20527E-05	decrease
chr2	74507489	74507889	-0.925988521	5.2105E-05	decrease
chrX	48574143	48574543	-1.169686645	5.27049E-05	decrease
chr11	94493760	94494160	-1.010746956	5.2719E-05	decrease
chr11	119101847	119102247	-1.001944137	5.37219E-05	decrease
chr1	150067352	150067752	-1.087084206	5.40624E-05	decrease
chr7	91940754	91941154	-1.280157536	5.43104E-05	decrease
chr15	67254512	67254912	-0.974005695	5.48902E-05	decrease
chr8	102239160	102239560	-0.970108001	5.50391E-05	decrease
chr19	44890664	44891064	-0.863612227	5.52492E-05	decrease
chr19	55259019	55259419	-0.964125039	5.64427E-05	decrease
chr2	172101919	172102319	-1.267668809	5.72376E-05	decrease
chr14	39432443	39432843	-1.07927879	5.82959E-05	decrease
chr11	83156651	83157051	-1.011603554	5.8404E-05	decrease
chr14	31026417	31026817	-1.202080411	5.91871E-05	decrease
chr4	170027243	170027643	-1.079113144	6.01507E-05	decrease
chr1	95072670	95073070	-1.04901303	6.03057E-05	decrease
chr9	35815199	35815599	-1.086837087	6.23951E-05	decrease
chr1	183471652	183472052	-0.898507794	6.2448E-05	decrease
chr3	38024597	38024997	-0.897084978	6.25656E-05	decrease
chr5	138337945	138338345	-1.002362529	6.34763E-05	decrease
chr16	89490601	89491001	-1.249954078	6.3621E-05	decrease
chr2	197499865	197500265	-1.010763088	6.36602E-05	decrease
chr19	17469859	17470259	-1.04636108	6.48475E-05	decrease
chr19	6476297	6476697	-0.942727398	6.49781E-05	decrease
chr19	596917	597317	-1.05124129	6.54418E-05	decrease
chr1	11908350	11908750	-0.970120727	6.56E-05	decrease
chr2	32627847	32628247	-1.299014667	6.58751E-05	decrease
chr4	75514340	75514740	-1.196224881	6.72054E-05	decrease
chr5	176388693	176389093	-0.897985588	6.80652E-05	decrease
chr19	4007183	4007583	-1.322418068	6.84745E-05	decrease
chr17	7836591	7836991	-0.948024938	6.85482E-05	decrease
chr22	40951334	40951734	-1.011216226	6.89957E-05	decrease
chr7	100538739	100539139	-1.006303588	7.07713E-05	decrease
chr3	48635353	48635753	-1.043727204	7.13175E-05	decrease
chr16	29811165	29811565	-0.788982929	7.1489E-05	decrease
chr2	175168165	175168565	-0.941321518	7.24491E-05	decrease
chr7	134316700	134317100	-1.137855182	7.26019E-05	decrease
chr22	29553454	29553854	-1.180140396	7.28184E-05	decrease
chr20	35616297	35616697	-0.96511484	7.30439E-05	decrease
chr6	159693499	159693899	-1.035656972	7.31965E-05	decrease
chr13	79481103	79481503	-0.999591496	7.32563E-05	decrease
chr14	64986848	64987248	-1.051660231	7.39908E-05	decrease
chr1	186375283	186375683	-0.998859768	7.48095E-05	decrease
chr3	52536305	52536705	-0.981500903	7.51658E-05	decrease
chr2	156400707	156401107	-1.144879918	7.5575E-05	decrease
chr1	70354448	70354848	-0.971372037	7.61806E-05	decrease
chr20	3888983	3889383	-1.052930394	7.62029E-05	decrease
chr3	49939874	49940274	-1.13986666	7.65844E-05	decrease
chr18	24397869	24398269	-1.266245018	7.77217E-05	decrease
chr19	7506425	7506825	-1.143422995	7.78098E-05	decrease
chr11	117143748	117144148	-1.223091251	7.80124E-05	decrease
chr6	52995191	52995591	-0.98351021	7.8178E-05	decrease
chr2	86442545	86442945	-1.127452525	7.83316E-05	decrease
chr1	156501044	156501444	-1.303569431	7.89021E-05	decrease
chr1	151165590	151165990	-1.060399912	8.0019E-05	decrease
chr11	62727490	62727890	-0.970795762	8.05122E-05	decrease

chr10	73744197	73744597	-1.082028915	8.25608E-05	decrease
chr19	4247041	4247441	-0.950010255	8.25765E-05	decrease
chr9	107282849	107283249	-1.163383457	8.33411E-05	decrease
chr1	85708214	85708614	-0.961616828	8.3464E-05	decrease
chr2	189441086	189441486	-1.009496401	8.34748E-05	decrease
chr15	74724002	74724402	-1.088080385	8.49359E-05	decrease
chr1	40979439	40979839	-1.050019404	8.57502E-05	decrease
chr22	40856583	40856983	-0.916797564	8.58566E-05	decrease
chr14	49862634	49863034	-0.990176331	8.59402E-05	decrease
chr19	55407799	55408199	-1.133407657	8.60048E-05	decrease
chr4	153153376	153153776	-1.341905861	8.74764E-05	decrease
chr19	48400264	48400664	-1.035457542	8.85244E-05	decrease
chr14	45253156	45253556	-1.250556134	8.97202E-05	decrease
chr5	170633151	170633551	-1.225222223	9.02831E-05	decrease
chr5	140664484	140664884	-1.050897913	9.12678E-05	decrease
chr15	75450492	75450892	-0.942022378	9.30301E-05	decrease
chr19	47035878	47036278	-1.201351877	9.31249E-05	decrease
chrX	135344262	135344662	-1.130463077	9.31355E-05	decrease
chr3	47513328	47513728	-1.009565523	9.34279E-05	decrease
chr13	77328907	77329307	-0.838360263	9.3981E-05	decrease
chr19	35644268	35644668	-0.983853305	9.41993E-05	decrease
chr5	177426213	177426613	-0.965016513	9.51897E-05	decrease
chr6	109382573	109382973	-0.938127356	9.59569E-05	decrease
chr6	125956735	125957135	-1.110845904	9.68654E-05	decrease
chr19	49766366	49766766	-0.90309687	9.70097E-05	decrease
chr3	46976501	46976901	-0.968675228	9.74573E-05	decrease
chr11	62753809	62754209	-0.925330483	9.79421E-05	decrease
chr1	193121885	193122285	-1.058563837	9.81006E-05	decrease
chr12	22624843	22625243	-1.140759936	9.82859E-05	decrease
chr19	48965440	48965840	-1.099339368	9.9459E-05	decrease
chr19	52189875	52190275	-0.883121991	9.95813E-05	decrease
chr14	39174860	39175260	-0.95583586	9.98293E-05	decrease
chr2	152717889	152718289	-0.930760993	0.000100431	decrease
chr19	57280087	57280487	-0.949768926	0.000100502	decrease
chr7	108525989	108526389	-0.906285052	0.000100796	decrease
chr12	71685981	71686381	-1.262357828	0.000101698	decrease
chr2	68157543	68157943	-0.998659309	0.000103131	decrease
chr19	17292546	17292946	-1.15984191	0.000103281	decrease
chr1	77979056	77979456	-0.803086102	0.000103505	decrease
chr14	99481074	99481474	-1.053226523	0.000104152	decrease
chr9	120793157	120793557	-1.211619586	0.000104674	decrease
chr6	132814190	132814590	-0.896212988	0.000106103	decrease
chr15	89088084	89088484	-0.970689379	0.000106655	decrease
chr6	144095308	144095708	-1.03780864	0.000107177	decrease
chr7	139340597	139340997	-0.816698661	0.000107541	decrease
chr16	30634174	30634574	-0.986554668	0.000107926	decrease
chr19	4723862	4724262	-1.115941599	0.000108384	decrease
chr12	123232940	123233340	-1.047462086	0.000108456	decrease
chr1	234608136	234608536	-1.126912241	0.000108703	decrease
chr8	123041883	123042283	-1.116889833	0.000108946	decrease
chr8	144104041	144104441	-1.035844984	0.000109446	decrease
chr3	49094323	49094723	-0.838902158	0.000110534	decrease
chr9	98255747	98256147	-1.112157397	0.000111559	decrease
chr12	114684153	114684553	-1.072172427	0.000111797	decrease
chr10	110567325	110567725	-0.894591534	0.000112018	decrease
chr7	12210936	12211336	-1.097921714	0.000115097	decrease
chr12	113221043	113221443	-1.094542707	0.000116025	decrease
chr19	45584457	45584857	-0.991086197	0.000116815	decrease
chr19	41397411	41397811	-0.928965215	0.000117314	decrease
chr1	202348525	202348925	-1.208679208	0.000117371	decrease
chr22	20918030	20918430	-1.242209046	0.000118343	decrease
chr1	231337768	231338168	-0.875079065	0.000118564	decrease
chr19	42242951	42243351	-0.929843535	0.00012065	decrease
chr3	49171184	49171584	-1.093149143	0.000121315	decrease
chr13	72781888	72782288	-0.933360121	0.000121871	decrease
chr19	39970769	39971169	-0.950762365	0.000122706	decrease
chr1	119140296	119140696	-0.902799655	0.000123995	decrease
chr11	3422732	3423132	-0.924995711	0.000124856	decrease
chr6	117602439	117602839	-1.037154673	0.000125064	decrease
chr1	155308581	155308981	-0.936594052	0.000125692	decrease
chr10	17643828	17644228	-0.905441265	0.000125916	decrease
chr16	2968854	2969254	-1.061306195	0.000126332	decrease
chr19	40750705	40751105	-0.993577559	0.000126495	decrease
chr2	111884086	111884486	-1.129637313	0.000127332	decrease
chr1	100132733	100133133	-1.020822731	0.000128536	decrease
chr14	21269393	21269793	-0.873805342	0.000129296	decrease

chr10	130110529	130110929	-0.819002993	0.000130357	decrease
chr2	113756313	113756713	-1.35039821	0.000130734	decrease
chr1	46132654	46133054	-0.918954643	0.000131584	decrease
chr9	37120151	37120551	-1.057343022	0.000131705	decrease
chr16	31201566	31201966	-1.041794147	0.000131787	decrease
chr12	108731656	108732056	-1.026579123	0.000132597	decrease
chr17	7484195	7484595	-1.027361902	0.000132831	decrease
chr4	119212624	119213024	-0.950968752	0.000132943	decrease
chr7	27130904	27131304	-1.25006927	0.000135628	decrease
chr16	27203512	27203912	-1.017675546	0.000136665	decrease
chr2	26244577	26244977	-0.901862919	0.00013669	decrease
chrX	48958198	48958598	-0.968013984	0.000137918	decrease
chr4	165112848	165113248	-1.008957816	0.000137924	decrease
chr1	84620730	84621130	-1.256988253	0.000139853	decrease
chr7	112939700	112940100	-1.070462737	0.000139857	decrease
chr1	145996606	145997006	-1.117920552	0.000139895	decrease
chr1	91021745	91022145	-1.158433948	0.000140728	decrease
chr11	62672068	62672468	-1.087729786	0.000142084	decrease
chr22	41621045	41621445	-1.06776818	0.000144151	decrease
chr10	12042853	12043253	-0.967872407	0.00014421	decrease
chr5	137754366	137754766	-0.963782983	0.00014433	decrease
chr12	53006194	53006594	-1.001569548	0.000145537	decrease
chr19	48321314	48321714	-1.015524223	0.000145897	decrease
chr11	125592504	125592904	-0.907010614	0.000146512	decrease
chr5	172188478	172188878	-1.333412001	0.000147405	decrease
chr11	73598176	73598576	-0.975623326	0.000147906	decrease
chr15	66701454	66701854	-1.0152298	0.000147914	decrease
chr19	55653994	55654394	-0.918715036	0.000151521	decrease
chr5	6712189	6712589	-1.257469576	0.000151681	decrease
chr1	204506634	204507034	-0.899189968	0.000151867	decrease
chr7	155644099	155644499	-1.314191761	0.000153312	decrease
chr6	26421163	26421563	-1.110190359	0.000153375	decrease
chr19	2236307	2236707	-0.977377374	0.000153438	decrease
chr10	101693866	101694266	-1.294338439	0.000153847	decrease
chr5	172006652	172007052	-0.977127349	0.0001544	decrease
chr1	235328375	235328775	-0.859750705	0.000154725	decrease
chr4	151014961	151015361	-1.121632409	0.000154754	decrease
chr12	116738121	116738521	-1.15587606	0.000155605	decrease
chr6	169168156	169168556	-1.222080937	0.000157079	decrease
chr13	45120378	45120778	-1.234719155	0.000157309	decrease
chr15	72473943	72474343	-1.112349786	0.000158117	decrease
chr2	241734059	241734459	-1.332522076	0.0001582	decrease
chr7	100081853	100082253	-1.243801313	0.000158919	decrease
chr17	7686233	7686633	-0.790108821	0.000159746	decrease
chr7	100673316	100673716	-1.003360959	0.000160639	decrease
chr5	75336784	75337184	-0.854799418	0.000161717	decrease
chr11	113895665	113896065	-1.311576586	0.000162551	decrease
chr15	65296216	65296616	-0.903824482	0.000162996	decrease
chr7	90403467	90403867	-1.173655655	0.000165521	decrease
chr17	75855309	75855709	-1.037072361	0.000167893	decrease
chr12	120686455	120686855	-0.915267355	0.000168846	decrease
chr12	103965718	103966118	-0.949170066	0.000169254	decrease
chr8	38176471	38176871	-0.991428793	0.000169499	decrease
chr3	33440439	33440839	-0.99003982	0.000170201	decrease
chr5	141923783	141924183	-0.967973341	0.000171016	decrease
chr3	57755805	57756205	-1.082306436	0.000171375	decrease
chr22	27919672	27920072	-0.991294251	0.000171856	decrease
chr20	44210575	44210975	-1.20178021	0.00017266	decrease
chr2	189762566	189762966	-1.105627869	0.000173549	decrease
chr13	97433104	97433504	-0.847498754	0.000174315	decrease
chr1	248858782	248859182	-1.299942024	0.000174336	decrease
chr12	48716663	48717063	-1.044943584	0.000174583	decrease
chr19	42325287	42325687	-0.982443506	0.000175074	decrease
chr3	9792714	9793114	-0.840389445	0.000175318	decrease
chr20	324577	324977	-1.156367373	0.000175847	decrease
chr11	2400358	2400758	-0.94802881	0.000177779	decrease
chr11	76235448	76235848	-1.052982468	0.000178125	decrease
chr1	174999324	174999724	-0.87688221	0.000179091	decrease
chr14	91060670	91061070	-0.886308292	0.000181135	decrease
chr10	113679661	113680061	-1.152718571	0.000181617	decrease
chrX	47366084	47366484	-1.189287498	0.000182253	decrease
chr7	25856917	25857317	-0.940497468	0.000182685	decrease
chr1	50968206	50968606	-1.180812676	0.00018383	decrease
chr4	128287861	128288261	-1.055686265	0.000184286	decrease
chr19	43766577	43766977	-1.292900003	0.000184679	decrease
chr2	27356667	27357067	-0.9111429	0.000184739	decrease

chr1	206202957	206203357	-1.090152797	0.000186527	decrease
chr17	81395061	81395461	-0.997825539	0.000187284	decrease
chr1	170531974	170532374	-0.864881117	0.000188286	decrease
chr10	97334701	97335101	-1.312897792	0.000188872	decrease
chr2	191677921	191678321	-1.176987746	0.000190004	decrease
chr11	67239703	67240103	-1.069741192	0.000190367	decrease
chr2	11466202	11466602	-0.950716797	0.000190516	decrease
chr6	87408181	87408581	-1.138580636	0.000191676	decrease
chr1	171781247	171781647	-0.971787001	0.000191835	decrease
chr6	159761954	159762354	-0.954755827	0.00019186	decrease
chr7	156951432	156951832	-1.007522471	0.000191867	decrease
chr17	63827241	63827641	-0.88196005	0.000192471	decrease
chr1	38012362	38012762	-1.050363769	0.000193433	decrease
chr1	205122018	205122418	-0.873895863	0.000193456	decrease
chr2	66434565	66434965	-1.141337574	0.000193893	decrease
chr3	158110220	158110620	-0.985313683	0.000194103	decrease
chr14	45134313	45134713	-1.004793813	0.000194911	decrease
chr11	118928382	118928782	-0.996137797	0.00019503	decrease
chr19	12696584	12696984	-0.996878153	0.000195172	decrease
chr17	7307097	7307497	-0.843017841	0.00019568	decrease
chr7	44986534	44986934	-0.951468021	0.000197008	decrease
chr8	80029865	80030265	-0.987240016	0.000198318	decrease
chr17	4940318	4940718	-0.988707798	0.00019832	decrease
chr14	35122037	35122437	-0.977314645	0.000199243	decrease
chr1	28552677	28553077	-0.917836282	0.000200279	decrease
chr13	60396520	60396920	-0.909922736	0.000201417	decrease
chr14	20343071	20343471	-0.825754846	0.000202936	decrease
chr17	63773746	63774146	-0.918316213	0.000204233	decrease
chr6	118894039	118894439	-1.176407082	0.00020437	decrease
chr4	77156631	77157031	-1.065821703	0.000204456	decrease
chr3	49104596	49104996	-1.031722281	0.000204683	decrease
chr1	201955265	201955665	-0.943851551	0.000204838	decrease
chr7	21945815	21946215	-1.191279037	0.000204882	decrease
chr4	17810593	17810993	-0.946469986	0.000204891	decrease
chr15	92809053	92809453	-1.054469507	0.000205521	decrease
chr11	87037653	87038053	-1.194160828	0.000205837	decrease
chr6	77368859	77369259	-1.1834017	0.000206091	decrease
chr1	148522725	148523125	-0.886752564	0.000206257	decrease
chr5	179698412	179698812	-0.874663357	0.000206372	decrease
chrX	48737322	48737722	-0.862051244	0.000206845	decrease
chr10	70146730	70147130	-0.92516727	0.000207572	decrease
chr21	42892776	42893176	-1.060761361	0.000208088	decrease
chr2	58046416	58046816	-1.080502593	0.00021216	decrease
chrX	123959899	123960299	-1.066955772	0.000212756	decrease
chr17	68020141	68020541	-1.271518199	0.000213086	decrease
chr6	170553940	170554340	-0.918502322	0.000213792	decrease
chr8	25458400	25458800	-0.919521793	0.000213954	decrease
chr5	102671349	102671749	-1.120530943	0.000214161	decrease
chrX	41333786	41334186	-1.000621972	0.000214178	decrease
chr19	17337164	17337564	-0.828879279	0.000214946	decrease
chrX	48539515	48539915	-1.125713433	0.00021537	decrease
chr1	212035284	212035684	-0.854767498	0.00021545	decrease
chr21	45555873	45556273	-1.084573556	0.000216485	decrease
chr19	47273800	47274200	-0.898680902	0.000217042	decrease
chr1	236064945	236065345	-1.164417332	0.000217787	decrease
chr1	117059940	117060340	-0.841471918	0.000218083	decrease
chr2	88691457	88691857	-1.062187434	0.000219561	decrease
chrX	106726451	106726851	-1.21612735	0.000219828	decrease
chr2	151409743	151410143	-1.043476877	0.000220747	decrease
chr19	10419918	10420318	-1.096151501	0.000221626	decrease
chr8	70402123	70402523	-1.085547216	0.000223008	decrease
chr21	42879062	42879462	-1.001537025	0.000223306	decrease
chr9	86354140	86354540	-0.970654576	0.000226775	decrease
chr10	17228477	17228877	-1.205203284	0.00022884	decrease
chr4	105473800	105474200	-1.052369744	0.000229932	decrease
chr2	32010724	32011124	-1.19625723	0.000231795	decrease
chr5	157731366	157731766	-1.010434251	0.000231848	decrease
chr5	119268316	119268716	-1.018926784	0.000232083	decrease
chr9	125241417	125241817	-0.974913988	0.000232937	decrease
chr1	2204521	2204921	-1.281048621	0.000232974	decrease
chr19	18192801	18193201	-0.942572778	0.00023432	decrease
chr21	33542617	33543017	-0.758921894	0.00023476	decrease
chr5	23507268	23507668	-1.00075161	0.000235219	decrease
chr1	27490244	27490644	-1.122843985	0.000236253	decrease
chr4	140154180	140154580	-1.198343716	0.000236642	decrease
chr4	158671918	158672318	-0.831832722	0.000237249	decrease

chr6	7389492	7389892	-0.939942984	0.000239738	decrease
chr2	61854211	61854611	-1.07088036	0.000243363	decrease
chr2	42361196	42361596	-0.866304401	0.000243685	decrease
chr1	155017310	155017710	-0.98405978	0.000244466	decrease
chr19	40444426	40444826	-1.108824004	0.000245044	decrease
chr2	48314231	48314631	-1.160538798	0.000245814	decrease
chr11	134224215	134224615	-1.122250825	0.000246158	decrease
chr19	12890799	12891199	-0.855103678	0.0002463	decrease
chr2	200963443	200963843	-0.982042474	0.000248024	decrease
chr12	57459278	57459678	-0.974538567	0.000248748	decrease
chr6	21593573	21593973	-1.079219368	0.000249251	decrease
chr1	63316987	63317387	-1.230599528	0.000249782	decrease
chr11	62831562	62831962	-0.770456833	0.000251053	decrease
chr9	89310751	89311151	-1.055448449	0.000255626	decrease
chr11	74170924	74171324	-0.983126027	0.000255978	decrease
chr7	73005705	73006105	-1.073044983	0.000260758	decrease
chr2	41840763	41841163	-1.095767002	0.000262314	decrease
chr14	103518651	103519051	-0.846624676	0.000266456	decrease
chr2	208265850	208266250	-1.033196689	0.000267408	decrease
chr14	92121639	92122039	-1.051967161	0.000268584	decrease
chr12	46373239	46373639	-1.195404959	0.000268732	decrease
chr19	8592229	8592629	-0.979690764	0.000270879	decrease
chr1	210373869	210374269	-0.832682482	0.000273937	decrease
chr17	7572421	7572821	-0.877231257	0.000274377	decrease
chr1	207751970	207752370	-0.974640356	0.000276894	decrease
chr3	170222045	170222445	-0.963391763	0.000278915	decrease
chr6	159790009	159790409	-0.826066795	0.00027905	decrease
chr19	38930443	38930843	-0.964329966	0.000279444	decrease
chr12	26938164	26938564	-0.985940419	0.000281721	decrease
chr20	3204371	3204771	-1.096364665	0.000282482	decrease
chr3	32684882	32685282	-1.118676554	0.000284856	decrease
chr5	141876936	141877336	-1.217951425	0.000285792	decrease
chr1	181088361	181088761	-1.073268929	0.000289403	decrease
chr12	6568243	6568643	-1.117280687	0.000290265	decrease
chr1	152034750	152035150	-1.172027009	0.000290484	decrease
chr17	75784636	75785036	-0.993307683	0.000291096	decrease
chr7	149838152	149838552	-0.990772304	0.000291515	decrease
chr6	4135242	4135642	-1.242796303	0.000291785	decrease
chr16	28936419	28936819	-0.997593154	0.000294289	decrease
chr6	149750050	149750450	-0.899202824	0.000294315	decrease
chr5	123423379	123423779	-1.047783536	0.000294753	decrease
chr3	128123647	128124047	-1.086029794	0.000295662	decrease
chr17	4731417	4731817	-0.854534772	0.000296131	decrease
chr14	20632963	20633363	-1.105052169	0.00029641	decrease
chr2	231056559	231056959	-1.016850653	0.000296637	decrease
chr7	148983464	148983864	-1.081294015	0.000296694	decrease
chr2	148020387	148020787	-0.977134258	0.000298255	decrease
chr13	71864711	71865111	-0.991514701	0.000299103	decrease
chr2	3558302	3558702	-0.996210201	0.000302462	decrease
chr1	156689279	156689679	-1.06695444	0.000302556	decrease
chr14	63543289	63543689	-1.128119915	0.000303742	decrease
chr7	107891107	107891507	-0.975251014	0.000304087	decrease
chrX	14873124	14873524	-1.044683739	0.000306205	decrease
chr3	50350723	50351123	-0.973055491	0.000306744	decrease
chr8	99013167	99013567	-1.165093905	0.000307525	decrease
chr1	204516080	204516480	-0.9715973	0.00030753	decrease
chr4	151099252	151099652	-0.931980549	0.000309584	decrease
chr8	65644364	65644764	-1.015052384	0.000312053	decrease
chr17	44141626	44142026	-1.135912892	0.000313654	decrease
chr1_KI270713v1_random	21583	21983	-0.886085916	0.000314335	decrease
chr11	82900787	82901187	-0.892744509	0.000316448	decrease
chr1	165828679	165829079	-1.096564794	0.000317526	decrease
chr2	72825645	72826045	-1.197519947	0.000321053	decrease
chr5	131165077	131165477	-1.191948814	0.000322407	decrease
chr4	163166781	163167181	-1.164517764	0.000322494	decrease
chr19	51002541	51002941	-1.143398132	0.000324033	decrease
chrX	149940008	149940408	-0.88717233	0.00032496	decrease
chr2	121736762	121737162	-1.209472906	0.000324985	decrease
chr11	103109276	103109676	-1.027185215	0.00032521	decrease
chr1	108746447	108746847	-1.009861342	0.000325311	decrease
chr19	15419014	15419414	-0.843068001	0.000326305	decrease
chr9	34049153	34049553	-0.803457604	0.000326476	decrease
chr1	202341808	202342208	-0.938576629	0.000328678	decrease
chr9	15422628	15423028	-1.128450583	0.00033597	decrease
chr7	30284167	30284567	-1.00944752	0.000337043	decrease
chr18	35343977	35344377	-0.915322545	0.000337707	decrease

chr11	86244432	86244832	-0.982271977	0.000338875	decrease
chr4	53365743	53366143	-1.027594052	0.0003396	decrease
chr9	113401137	113401537	-0.921877888	0.000342288	decrease
chr5	154858150	154858550	-1.010444819	0.000343598	decrease
chr19	46860067	46860467	-1.117625918	0.00034471	decrease
chr3	108589381	108589781	-1.001914063	0.000345549	decrease
chr1	62783699	62784099	-0.923165137	0.000345873	decrease
chr9	26956271	26956671	-0.979120622	0.000345995	decrease
chr4	87006745	87007145	-1.03549722	0.000346832	decrease
chr1	109090573	109090973	-0.991589186	0.000347229	decrease
chr1	185157040	185157440	-0.755919211	0.000351747	decrease
chr10	101061726	101062126	-1.059446444	0.000352707	decrease
chr2	135530937	135531337	-0.875018237	0.000354908	decrease
chr11	118435865	118436265	-0.945193771	0.000355229	decrease
chr1	235867081	235867481	-0.879670511	0.000355314	decrease
chr6	13615057	13615457	-1.054893057	0.0003557	decrease
chr9	6006998	6007398	-0.996958366	0.000356897	decrease
chr16	2882695	2883095	-0.888842173	0.000357077	decrease
chr2	207711174	207711574	-1.033633783	0.000358384	decrease
chr2	202911588	202911988	-1.057670516	0.000360863	decrease
chr3	48301149	48301549	-1.055853161	0.000361582	decrease
chr4	70688130	70688530	-0.941344409	0.000361868	decrease
chr10	119080951	119081351	-1.059193162	0.000362482	decrease
chr10	99620543	99620943	-0.961385577	0.000364614	decrease
chr4	169660136	169660536	-1.137171282	0.000368432	decrease
chr19	4342704	4343104	-0.777377974	0.00036903	decrease
chr17	17972473	17972873	-0.867570019	0.000370321	decrease
chr4	140151787	140152187	-1.220804742	0.00037195	decrease
chr7	23474408	23474808	-1.026659937	0.000373341	decrease
chr3	156675227	156675627	-1.240208567	0.000374071	decrease
chr10	102502260	102502660	-0.869979714	0.000374274	decrease
chr6	137867242	137867642	-1.151809222	0.00037971	decrease
chr4	143185226	143185626	-1.240167692	0.000379866	decrease
chr1	70205539	70205939	-0.916041176	0.000380436	decrease
chr5	72320168	72320568	-0.973859305	0.000383468	decrease
chr1	2199316	2199716	-1.123477755	0.000383564	decrease
chr9	35079746	35080146	-0.970687003	0.000384916	decrease
chr19	40603901	40604301	-0.996130944	0.000385165	decrease
chrX	100820125	100820525	-1.007834519	0.000391466	decrease
chrX	49191002	49191402	-0.900616734	0.000393823	decrease
chr5	104079844	104080244	-1.124468207	0.000394179	decrease
chr1	155562860	155563260	-0.963691198	0.000394556	decrease
chr11	809396	809796	-0.887228533	0.000395389	decrease
chr17	35578305	35578705	-1.147467638	0.000396539	decrease
chr3	141875580	141875980	-1.062147075	0.000396642	decrease
chr19	11737977	11738377	-1.24092479	0.000396933	decrease
chr3	44338274	44338674	-1.13972703	0.00039717	decrease
chr2	43222012	43222412	-1.150315519	0.000397842	decrease
chr15	69160284	69160684	-1.096646428	0.0003985	decrease
chr1	244969739	244970139	-1.046327651	0.000399494	decrease
chr15	92904114	92904514	-0.919500133	0.000401518	decrease
chr13	87671027	87671427	-0.895041301	0.000405872	decrease
chr14	50231775	50232175	-1.240697929	0.000406528	decrease
chr7	102395957	102396357	-1.081656914	0.000412034	decrease
chr5	69189460	69189860	-1.061878914	0.000412814	decrease
chr17	82022841	82023241	-0.926750856	0.000413571	decrease
chr3	5188735	5189135	-1.054068014	0.000416687	decrease
chr5	140647529	140647929	-0.842542976	0.000416839	decrease
chr6	11232351	11232751	-0.965039672	0.000419443	decrease
chr4	102431179	102431579	-1.167121474	0.000422495	decrease
chr1	156728685	156729085	-1.01079174	0.000422751	decrease
chr9	33166232	33166910	-1.110398301	0.000425973	decrease
chr14	23320989	23321389	-0.928363956	0.000426386	decrease
chr19	41439621	41440021	-0.995955183	0.000426417	decrease
chr3	143001400	143001800	-1.074256745	0.000427894	decrease
chrX	74179437	74179837	-0.928283355	0.000428853	decrease
chr1	202927008	202927408	-0.949041088	0.000428999	decrease
chr19	18590471	18590871	-0.957534845	0.000429192	decrease
chr7	107743655	107744055	-0.998538987	0.000429507	decrease
chr12	121839475	121839875	-0.910434555	0.000430155	decrease
chr19	40777734	40778134	-0.913824613	0.000431379	decrease
chr15	78540236	78540636	-1.052318797	0.000432715	decrease
chr11	64778645	64779045	-0.78288158	0.000432861	decrease
chr7	26864587	26864987	-0.987158406	0.000434073	decrease
chr6	149717483	149717883	-0.9618795	0.0004368	decrease
chr1	233635971	233636371	-1.073168425	0.000438296	decrease

chr10	43436691	43437091	-1.052198374	0.000439365	decrease
chr15	72226772	72227172	-0.974218722	0.000441556	decrease
chr19	33177029	33177429	-0.747991358	0.000441752	decrease
chr1	160343116	160343516	-0.927496897	0.000443628	decrease
chr1	214551558	214551958	-1.152645099	0.000444381	decrease
chr12	8026794	8027194	-1.109887813	0.000446194	decrease
chr9	35732350	35732750	-0.857445702	0.000447231	decrease
chr12	56725261	56725661	-1.041630279	0.000447711	decrease
chr8	101615071	101615471	-1.148404413	0.000449109	decrease
chr4	140373266	140373666	-0.89242426	0.000449286	decrease
chr14	103333835	103334235	-1.05967183	0.000449845	decrease
chr3	159988589	159988989	-1.194922767	0.000450282	decrease
chr14	91510381	91510781	-1.15039686	0.000451367	decrease
chr11	96389565	96389965	-0.999008305	0.000451948	decrease
chr1	27321993	27322393	-1.172540643	0.000452984	decrease
chr12	53626015	53626415	-0.823653862	0.000456158	decrease
chr17	18183354	18183754	-0.909724874	0.00045621	decrease
chr9	83921337	83921737	-1.111936212	0.000456538	decrease
chr15	68277508	68277908	-0.998971689	0.000460728	decrease
chr8	103414871	103415271	-0.919257632	0.00046104	decrease
chr4	169620104	169620504	-0.929980093	0.000461851	decrease
chr20	34515959	34516359	-1.086635412	0.000461974	decrease
chr3	125915871	125916271	-0.838804558	0.000463075	decrease
chr6	110875988	110876388	-1.056232845	0.000463229	decrease
chr7	131106330	131106730	-1.19159747	0.000464761	decrease
chr4	145097919	145098319	-0.970225576	0.000466207	decrease
chr17	8187302	8187702	-0.845218031	0.000467772	decrease
chr11	119121308	119121708	-0.891215058	0.000471367	decrease
chr6	148747812	148748212	-1.175631686	0.000473133	decrease
chr6	26322496	26322896	-1.133899953	0.000475133	decrease
chr10	97292273	97292673	-1.009208598	0.000475901	decrease
chrX	153928249	153928649	-0.894978721	0.000478788	decrease
chr19	47515152	47515552	-0.937783137	0.000483649	decrease
chr1	146376985	146377385	-0.834121086	0.000484503	decrease
chr12	121399843	121400243	-0.999604349	0.000485404	decrease
chr2	86105732	86106132	-0.921859333	0.000487273	decrease
chr13	52011726	52012126	-1.08075734	0.000490675	decrease
chr11	18634405	18634805	-0.942895629	0.000496131	decrease
chr9	131502947	131503347	-1.045407834	0.000496514	decrease
chr11	64759899	64760299	-0.930791487	0.000497269	decrease
chr22	38461350	38461750	-0.880956266	0.0005026	decrease
chr19	58362441	58362841	-0.894511307	0.000502804	decrease
chr13	27125703	27126103	-0.980176891	0.000503297	decrease
chr13	68023814	68024214	-1.059579143	0.000504055	decrease
chrX	16719367	16719767	-1.081740425	0.000506459	decrease
chr18	21600596	21600996	-0.88326791	0.000506666	decrease
chr2	219218146	219218546	-1.150234371	0.000511089	decrease
chr19	4791373	4791773	-1.024080903	0.000512181	decrease
chr3	155854348	155854748	-0.920849886	0.000514344	decrease
chr5	177516959	177517359	-0.886366471	0.000518031	decrease
chr22	40044241	40044641	-0.916480815	0.000518662	decrease
chr4	185425849	185426249	-0.934480305	0.000519748	decrease
chr14	63641832	63642232	-0.745046419	0.000520047	decrease
chr11	33039226	33039626	-1.040468723	0.000523227	decrease
chr20	18507491	18507891	-0.923756203	0.000523691	decrease
chr11	72112330	72112730	-0.917574661	0.000524301	decrease
chr1	203006738	203007138	-1.005750234	0.000527642	decrease
chr2	85132857	85133257	-1.052507081	0.00052852	decrease
chr1	70221321	70221721	-0.897002293	0.000530775	decrease
chr1	75796841	75797241	-0.985027128	0.000531276	decrease
chr13	44918131	44918531	-0.86124684	0.000532153	decrease
chr19	6737211	6737611	-0.835654372	0.000533829	decrease
chr7	16645802	16646202	-0.968577958	0.000534361	decrease
chr1	220046367	220046767	-1.004349726	0.000538396	decrease
chr19	49118581	49118981	-0.966434447	0.000539213	decrease
chr18	35240700	35241100	-0.786684824	0.000541138	decrease
chr11	66289189	66289589	-0.942358243	0.000542593	decrease
chr1	226062581	226062981	-1.00900377	0.000545927	decrease
chr9	112379577	112379977	-1.064858008	0.000546148	decrease
chr4	51842823	51843223	-0.941115393	0.00054651	decrease
chr1	113389927	113390327	-0.904079988	0.000547613	decrease
chr14	58427504	58427904	-1.012960074	0.000548211	decrease
chr20	37527833	37528233	-0.891223171	0.000548856	decrease
chr14	67359883	67360283	-1.050117091	0.000549254	decrease
chr1	23959377	23959777	-1.140449519	0.000550133	decrease
chr10	68471646	68472046	-1.145111175	0.000551001	decrease

chr10	7787999	7788399	-1.079460318	0.000552736	decrease
chr19	45423664	45424064	-0.901605206	0.000555163	decrease
chr17	63621959	63622359	-0.906948696	0.000555664	decrease
chr1	228646843	228647243	-0.868477075	0.000556241	decrease
chr5	64768543	64768943	-0.951716886	0.000558032	decrease
chr19	18932306	18932706	-0.944685524	0.00056246	decrease
chr6	113971107	113971507	-0.875181616	0.000563244	decrease
chr7	112205231	112205631	-1.104759182	0.00056363	decrease
chr9	70413878	70414278	-0.954463172	0.000563638	decrease
chr6	99515470	99515870	-1.087099371	0.000564321	decrease
chr19	48660941	48661341	-1.100408494	0.000568443	decrease
chr19	14207225	14207625	-0.648587573	0.0005692	decrease
chr3	39051656	39052056	-1.149076486	0.000569722	decrease
chr17	68512072	68512472	-1.057304415	0.000569876	decrease
chr11	64241258	64241658	-0.839759596	0.000571393	decrease
chr10	102245230	102245630	-0.958795304	0.000572668	decrease
chr20	38962221	38962621	-1.180977061	0.000574683	decrease
chrX	46836784	46837184	-1.109978208	0.000574796	decrease
chr5	93677221	93677621	-1.069159918	0.000575244	decrease
chr5	112976418	112976818	-0.837841708	0.000579466	decrease
chr4	121870199	121870599	-0.930639654	0.000579902	decrease
chr1	146037282	146037682	-0.906635478	0.000580863	decrease
chr19	36489372	36489772	-0.887462697	0.000582351	decrease
chr12	21501561	21501961	-0.985535834	0.000583128	decrease
chr3	45688862	45689262	-0.889022646	0.000585124	decrease
chr5	65624244	65624644	-0.897172858	0.000586148	decrease
chr6	11093663	11094063	-0.951835851	0.000586335	decrease
chr7	138001916	138002316	-0.797041683	0.000586612	decrease
chr10	93702493	93702893	-1.199452881	0.00058693	decrease
chr12	53321282	53321682	-0.942440454	0.000591736	decrease
chr11	93784009	93784409	-0.999586882	0.000592275	decrease
chr19	36772817	36773217	-1.066483395	0.000598521	decrease
chr4	31996642	31997042	-0.953326632	0.00060117	decrease
chrX	154762502	154762902	-0.935863753	0.000601243	decrease
chr10	78029512	78029912	-1.119492973	0.000603489	decrease
chr2	71331695	71332095	-1.119800308	0.000604201	decrease
chr4	30717773	30718173	-1.095828814	0.000604462	decrease
chr9	97039179	97039579	-0.907166006	0.00060722	decrease
chr5	43483666	43484066	-0.963338837	0.000607437	decrease
chr9	95317328	95317728	-1.139872281	0.000607593	decrease
chr1	205631920	205632320	-1.035386273	0.000613465	decrease
chr6	89829673	89830073	-0.891738986	0.000615243	decrease
chr1	31065490	31065890	-1.040574495	0.000621756	decrease
chr15	72117424	72117824	-0.993929693	0.000623387	decrease
chr5	73448651	73449051	-1.11224316	0.000625436	decrease
chr15	74635276	74635676	-1.050628775	0.000626702	decrease
chr14	24146841	24147241	-0.763267413	0.00062718	decrease
chrX	120485974	120486374	-1.071876885	0.000627821	decrease
chr1	113812217	113812617	-0.970852434	0.000628057	decrease
chr3	149657758	149658158	-1.062259581	0.000630772	decrease
chr10	101818382	101818782	-0.790620726	0.000631974	decrease
chr3	142447844	142448244	-1.128561178	0.000635021	decrease
chr2	158457871	158458271	-1.176955363	0.000636401	decrease
chr7	77122255	77122655	-0.933457902	0.000636832	decrease
chr3	128488252	128488652	-0.959473941	0.000636888	decrease
chr4	61202951	61203351	-1.139145529	0.000637301	decrease
chr10	97736854	97737254	-0.973253425	0.000638555	decrease
chr2	27050738	27051138	-0.71767535	0.000641968	decrease
chr11	83285959	83286359	-0.899148085	0.000642768	decrease
chr14	66507881	66508281	-0.863902524	0.000644554	decrease
chr1	248838658	248839058	-1.040913337	0.00064531	decrease
chr11	14520215	14520615	-0.916193442	0.000646346	decrease
chr16	22190724	22191124	-0.84336252	0.000649417	decrease
chr16	1309080	1309480	-0.988601389	0.00065304	decrease
chr11	65615287	65615687	-0.788307983	0.000654304	decrease
chr13	79516647	79517047	-1.178411072	0.000655635	decrease
chr13	36919146	36919546	-1.082801988	0.000656281	decrease
chrX	47193128	47193528	-1.018321916	0.000657812	decrease
chr12	6943967	6944367	-0.721171058	0.00065879	decrease
chr15	100602338	100602738	-0.832458635	0.000661511	decrease
chr19	45994984	45995384	-0.933578466	0.000661775	decrease
chr1	155934267	155934667	-0.973816643	0.000663025	decrease
chr2	28894318	28894718	-1.051486275	0.000663863	decrease
chr6	37819076	37819476	-0.801625496	0.000670549	decrease
chr6	81752986	81753386	-1.14022744	0.000671856	decrease
chr3	150703690	150704090	-1.181652782	0.000672701	decrease

chr17	75289197	75289597	-0.820363138	0.000673039	decrease
chr16	30092243	30092643	-0.875600227	0.000673979	decrease
chr19	36139764	36140164	-1.018582479	0.000675958	decrease
chr2	54723411	54723811	-1.001536098	0.000677209	decrease
chr12	38905319	38905719	-1.051613358	0.000679686	decrease
chr9	70258522	70258922	-1.047703079	0.000679888	decrease
chr2	47176643	47177043	-1.026325257	0.000681464	decrease
chr12	13098618	13099018	-1.031236901	0.000681689	decrease
chr22	41448389	41448789	-0.92238807	0.000683357	decrease
chr17	62065772	62066172	-0.990783096	0.00068361	decrease
chr19	47447293	47447693	-0.924425454	0.000685239	decrease
chrX	47650648	47651048	-0.870383789	0.000685785	decrease
chrX	132217090	132217490	-1.113265104	0.000688409	decrease
chr11	64317799	64318199	-0.934759181	0.000689807	decrease
chr10	75233977	75234685	-0.994272081	0.000690517	decrease
chr1	179365604	179366004	-0.944989092	0.00069123	decrease
chr5	160119469	160119869	-0.915044495	0.000692718	decrease
chr11	118401331	118401731	-0.895500301	0.000695281	decrease
chr11	65540700	65541100	-1.027067665	0.000697057	decrease
chr12	121794453	121794853	-0.902100424	0.000698569	decrease
chr19	17305508	17305908	-0.996294777	0.000699168	decrease
chr11	103111276	103111676	-1.154166804	0.000699541	decrease
chr1	179882243	179882643	-0.76533849	0.000700032	decrease
chr2	63588572	63588972	-0.83781449	0.000701352	decrease
chr2	46698668	46699068	-1.015330683	0.000701888	decrease
chr12	98644499	98644899	-0.951737634	0.000704827	decrease
chr12	122526803	122527203	-0.785694524	0.000708368	decrease
chr7	138459874	138460274	-0.98196239	0.000709551	decrease
chr3	129183731	129184131	-1.008612091	0.000709736	decrease
chr7	44848035	44848435	-1.052199014	0.000711353	decrease
chr1	181022824	181023224	-1.053265089	0.0007131	decrease
chr1	151048301	151048701	-0.911156414	0.000714249	decrease
chr1	35079130	35079530	-1.066671931	0.000714734	decrease
chr2	174673093	174673493	-1.182760282	0.000716097	decrease
chr4	83034802	83035202	-0.829585157	0.000716276	decrease
chr1	12019333	12019733	-1.064461261	0.000717452	decrease
chr5	96702735	96703135	-1.069272837	0.000720111	decrease
chr1	153967482	153967882	-0.839863917	0.000720742	decrease
chr1	172532552	172532952	-0.794031536	0.000721989	decrease
chrX	149539523	149539923	-1.002205289	0.000724819	decrease
chr11	67508332	67508732	-1.009659541	0.000726692	decrease
chr1	161166247	161166647	-0.768957091	0.00072684	decrease
chr6	89080461	89080861	-1.049658175	0.00072863	decrease
chr2	61538314	61538714	-0.876228195	0.000728841	decrease
chr3	53219967	53220367	-1.007630785	0.000734753	decrease
chr19	39532760	39533160	-1.103216866	0.000735038	decrease
chr3	131026540	131026940	-1.066120557	0.00073564	decrease
chr1	156193760	156194160	-0.864336215	0.000737984	decrease
chr10	102169551	102169951	-1.041090737	0.000741132	decrease
chr14	23469324	23469724	-1.004873621	0.000741555	decrease
chr19	49443498	49443898	-0.960166769	0.000745428	decrease
chr10	97640354	97640754	-1.009412109	0.000748902	decrease
chr15	92831748	92832148	-0.968481645	0.000748962	decrease
chr3	14178511	14178911	-1.029373289	0.000752251	decrease
chr5	157142644	157143044	-1.041502041	0.000753844	decrease
chr7	27150475	27150875	-1.012141132	0.000753849	decrease
chr15	65792308	65792708	-0.981083203	0.000754607	decrease
chr4	173334465	173334865	-0.825122256	0.000757547	decrease
chr22	41468716	41469116	-0.800008745	0.000758311	decrease
chr7	90346570	90346970	-1.025490412	0.000759265	decrease
chr3	40452932	40453332	-0.959847312	0.000759811	decrease
chr19	47391159	47391559	-1.130953639	0.000762844	decrease
chr18	33579009	33579409	-1.086202066	0.00076415	decrease
chr11	62591637	62592037	-0.836721739	0.000768826	decrease
chr7	102748670	102749070	-0.896165733	0.000769827	decrease
chr11	62706071	62706471	-0.999717283	0.000773066	decrease
chr1	184051557	184051957	-1.06440654	0.000773982	decrease
chr11	66958327	66958727	-0.983771963	0.000776923	decrease
chr13	66835134	66835534	-0.853916267	0.000777992	decrease
chr18	9475006	9475406	-1.00876233	0.000778168	decrease
chr14	61695405	61695805	-1.138934416	0.000781614	decrease
chr19	1021221	1021621	-0.947850525	0.000782576	decrease
chr4	107741095	107741495	-1.180356843	0.000783544	decrease
chrX	123962673	123963073	-1.062051501	0.000784046	decrease
chrX	16786122	16786522	-0.996163785	0.000785577	decrease
chr2	186486069	186486469	-0.829091818	0.000786994	decrease

chr1	156213001	156213401	-0.822815888	0.000793284	decrease
chr16	25111473	25111873	-0.929543012	0.000794523	decrease
chr2	183037224	183037624	-1.175120795	0.000795108	decrease
chr13	30870353	30870753	-0.829456683	0.000800199	decrease
chr11	66638220	66638620	-0.81919741	0.000800527	decrease
chr19	45645941	45646341	-0.955395186	0.000802639	decrease
chr16	18801527	18801927	-0.897749849	0.000805382	decrease
chr4	67700965	67701365	-1.147406421	0.000806484	decrease
chr5	124739053	124739453	-1.069766573	0.00080675	decrease
chr2	131492652	131493052	-0.974026162	0.000807028	decrease
chr4	174283455	174283855	-1.039536444	0.000809	decrease
chr1	212608399	212608799	-0.877214755	0.000810284	decrease
chr5	43121108	43121508	-0.930366599	0.000816482	decrease
chr11	10933876	10934276	-0.907118272	0.000816532	decrease
chr12	50111914	50112314	-0.841762606	0.000817692	decrease
chr14	35292098	35292498	-1.008086738	0.000818586	decrease
chr11	126062950	126063350	-1.017587157	0.000820405	decrease
chr6	84227617	84228017	-0.926413715	0.000821013	decrease
chr8	33513137	33513537	-0.944054705	0.000823781	decrease
chr2	175454876	175455276	-1.131484575	0.000824197	decrease
chr1	147541465	147541865	-1.07560771	0.000824563	decrease
chr6	75284788	75285188	-1.042500445	0.000827987	decrease
chr7	25125207	25125607	-0.93988125	0.000834193	decrease
chr19	58401149	58401549	-0.928513217	0.000834433	decrease
chr18	26090654	26091054	-0.799183028	0.000834976	decrease
chrX	54639500	54639900	-0.911141707	0.000835132	decrease
chr5	6633000	6633400	-0.940223658	0.000835415	decrease
chr8	108248633	108249033	-1.010015681	0.000835945	decrease
chr9	128455711	128456111	-1.131008995	0.000838259	decrease
chr17	14300722	14301122	-0.926197028	0.000839568	decrease
chr19	1507782	1508182	-1.037864397	0.000845719	decrease
chr17	42964259	42964659	-1.023903899	0.000847219	decrease
chr5	177351536	177351936	-0.912704441	0.000848138	decrease
chr6	145964255	145964655	-0.885877321	0.000850779	decrease
chr12	75329999	75330399	-0.888264309	0.000853	decrease
chr18	3247433	3247833	-0.943814667	0.00086218	decrease
chr11	57712068	57712468	-0.906281019	0.000863045	decrease
chr10	5853500	5853900	-1.014115397	0.000863803	decrease
chr2	142130856	142131256	-1.103126656	0.000867919	decrease
chr15	84716080	84716480	-0.801359493	0.000868406	decrease
chr5	75050266	75050666	-1.122656042	0.000870009	decrease
chr9	6681270	6681670	-0.830031	0.000870594	decrease
chr20	50958387	50958787	-1.061635886	0.000871051	decrease
chr15	84600548	84600948	-0.883259063	0.000873396	decrease
chr6	148342494	148342894	-1.035724482	0.000874554	decrease
chr19	16111432	16111832	-1.038966601	0.000876135	decrease
chrX	23742962	23743362	-1.013473384	0.000876141	decrease
chr1	99849992	99850392	-0.91786337	0.000879714	decrease
chrX	49043548	49043948	-0.912134453	0.000882668	decrease
chr14	52552429	52552829	-0.935564713	0.000884619	decrease
chr16	30922658	30923058	-0.688500304	0.000885449	decrease
chr1	32817357	32817757	-0.819988374	0.000895194	decrease
chr14	55191536	55191936	-0.969457679	0.000895245	decrease
chr1	112955791	112956191	-0.945849299	0.000896546	decrease
chr19	3573928	3574328	-1.037834017	0.00089817	decrease
chr19	50795293	50795693	-0.86203285	0.000900598	decrease
chr22	36776206	36776606	-0.976839132	0.000901705	decrease
chr7	36389930	36390330	-1.018889805	0.000902528	decrease
chr3	12484151	12484551	-1.081712085	0.000904017	decrease
chr2	112541980	112542380	-0.850652907	0.000904302	decrease
chr12	8243133	8243533	-0.789664975	0.000911535	decrease
chr15	72475129	72475529	-1.000177141	0.000915073	decrease
chr1	27349482	27349882	-1.125239625	0.000915306	decrease
chr19	56121364	56121764	-0.965781579	0.000916304	decrease
chr6	143842906	143843306	-0.902163274	0.000916717	decrease
chr2	85611868	85612268	-1.019832803	0.000918096	decrease
chr1	143720477	143720877	-1.053527444	0.000918333	decrease
chr20	35742158	35742558	-0.829075528	0.000918727	decrease
chr19	55385417	55385817	-0.824285682	0.000919704	decrease
chr11	126211384	126211784	-0.949889295	0.000923142	decrease
chr11	9573073	9573473	-1.055555361	0.000923621	decrease
chr19	56314805	56315205	-0.913099638	0.000932237	decrease
chr13	91347059	91347459	-1.012448743	0.000933021	decrease
chr19	46850634	46851034	-0.881330455	0.000933847	decrease
chr8	47960438	47960838	-0.843804303	0.000936225	decrease
chr19	51108093	51108493	-0.963142001	0.000937079	decrease

chr12	57078601	57079001	-0.897663193	0.000938495	decrease
chr10	50624413	50624813	-0.928246426	0.00093932	decrease
chr2	232550467	232550867	-1.023510617	0.000942623	decrease
chr19	18224103	18224503	-0.831790767	0.000942987	decrease
chr2	24971673	24972073	-0.917101286	0.000943759	decrease
chr1	161399566	161399966	-0.909745048	0.000943818	decrease
chr14	103384992	103385392	-0.983226861	0.000944658	decrease
chr7	35037914	35038314	-1.164982907	0.000945354	decrease
chr16	75433310	75433710	-0.969655434	0.000946538	decrease
chr9	38069556	38069956	-0.883172339	0.000946978	decrease
chr15	40092239	40092949	-1.012466323	0.000948753	decrease
chr2	101252273	101252673	-0.956259935	0.000949149	decrease
chr9	128362003	128362403	-1.013099734	0.000958603	decrease
chrX	118345751	118346151	-0.825646134	0.000960247	decrease
chr10	110918683	110919083	-0.780618881	0.000961339	decrease
chr11	60906592	60906992	-1.037292977	0.000962049	decrease
chr1	121184485	121184885	-0.890804689	0.000962403	decrease
chr13	41061670	41062070	-0.838287683	0.000962833	decrease
chr20	32109367	32109767	-0.97897786	0.000965667	decrease
chr3	169773167	169773567	-0.882343118	0.000967508	decrease
chr7	56033958	56034358	-0.954946906	0.00097058	decrease
chr7	140696652	140697052	-0.91865544	0.000971525	decrease
chr1	25338295	25338695	-1.055318828	0.000977933	decrease
chr11	108222926	108223326	-0.817913605	0.000978208	decrease
chr1	228139844	228140244	-0.672852105	0.000979001	decrease
chr12	53441272	53441672	-0.921509986	0.000980156	decrease
chr17	82359075	82359475	-0.977927435	0.000981715	decrease
chr18	23134698	23135098	-1.094223807	0.000985675	decrease
chr2	32277851	32278251	-1.127088806	0.000989083	decrease
chr14	52706893	52707293	-1.00532606	0.000995126	decrease
chr10	74826142	74826542	-0.730337713	0.001000698	decrease
chr16	30065353	30065753	-0.756607671	0.001003077	decrease
chr1	32773063	32773463	-0.883783079	0.001003844	decrease
chr3	52411199	52411599	-1.080803432	0.001007875	decrease
chrX	101051602	101052002	-0.923208412	0.0010091	decrease
chr7	149690968	149691368	-0.806519123	0.001009249	decrease
chr3	57555823	57556223	-0.918551122	0.001009683	decrease
chr12	53295147	53295547	-0.976960895	0.001018219	decrease
chr9	124941060	124941460	-1.034311214	0.001018378	decrease
chr17	5419808	5420208	-0.851131111	0.001021774	decrease
chrX	38801328	38801728	-1.111653637	0.001025502	decrease
chr12	95217571	95217971	-0.978521496	0.00102552	decrease
chr4	13484338	13484738	-0.841892378	0.001026935	decrease
chr2	197435006	197435406	-0.856863996	0.001027034	decrease
chr4	39527414	39527814	-0.913186518	0.0010283	decrease
chr14	50532638	50533038	-1.089306442	0.001029185	decrease
chr7	44999806	45000206	-1.053088312	0.001031347	decrease
chr1	180631674	180632074	-0.979617209	0.001032539	decrease
chr3	121749577	121749977	-0.912181971	0.001034944	decrease
chr19	7829646	7830046	-0.849749314	0.00103822	decrease
chr19	10416703	10417103	-1.00942738	0.001039293	decrease
chr8	108443502	108443902	-0.924927684	0.001040547	decrease
chr14	22919175	22919575	-0.86916318	0.001042859	decrease
chr4	82430274	82430674	-0.969272765	0.001043152	decrease
chr7	66996562	66996962	-0.884610292	0.001043326	decrease
chr5	180860853	180861253	-1.060100354	0.001043662	decrease
chr16	11496765	11497165	-1.10903276	0.001050955	decrease
chr13	99971582	99971982	-1.090023528	0.001051121	decrease
chr1	155084933	155085333	-0.821843939	0.001051286	decrease
chr6	107958311	107958711	-1.093966826	0.001052655	decrease
chr19	32971925	32972325	-0.944414531	0.001053106	decrease
chr3	160449835	160450235	-0.811605304	0.001053437	decrease
chr1	113072776	113073176	-0.837932654	0.001056488	decrease
chr2	121530958	121531358	-0.784656349	0.001066315	decrease
chr18	21111758	21112158	-0.958626102	0.001066967	decrease
chr6	82363552	82363952	-1.006859836	0.001071925	decrease
chr15	40421763	40422163	-1.077645611	0.001074021	decrease
chr13	48976218	48976618	-1.055840525	0.001074354	decrease
chr3	48663776	48664176	-0.739479141	0.001074651	decrease
chr21	36060283	36060683	-0.862871651	0.001076062	decrease
chr19	19406894	19407294	-1.044017074	0.001076961	decrease
chr19	55599621	55600021	-0.818811744	0.001080147	decrease
chr15	48810844	48811244	-0.961479669	0.001080981	decrease
chr2	24047318	24047718	-0.914386928	0.001081831	decrease
chr15	69074287	69074687	-0.948087269	0.001084789	decrease
chr2	226835837	226836237	-1.124268702	0.001087714	decrease

chr17	7435392	7435792	-0.961634745	0.001090079	decrease
chr5	131635079	131635479	-0.886382009	0.00109193	decrease
chr8	124998424	124998824	-1.140824377	0.001093884	decrease
chr1	149636502	149636902	-0.893553367	0.001094511	decrease
chr7	43729295	43729695	-1.024383563	0.001095626	decrease
chr3	69013412	69013812	-1.095984846	0.001096506	decrease
chr15	70097764	70098164	-0.848207724	0.001096508	decrease
chr11	60913784	60914184	-0.973842412	0.00110226	decrease
chr11	64304366	64304766	-0.905051111	0.001104488	decrease
chr15	97960255	97960655	-0.832010229	0.001105921	decrease
chr21	37073859	37074259	-1.028105382	0.00110604	decrease
chr4	82900534	82900934	-0.919853764	0.001111013	decrease
chrX	53683975	53684375	-0.860308086	0.00111188	decrease
chr3	136196479	136196879	-0.779790301	0.001112431	decrease
chr9	26947111	26947511	-0.918266905	0.001113865	decrease
chr3	100401160	100401560	-0.830864281	0.001114392	decrease
chr3	98522651	98523051	-0.99648463	0.00111628	decrease
chr17	48892526	48892926	-0.824806531	0.001117164	decrease
chr8	73008667	73009067	-0.946490092	0.001118333	decrease
chr13	26221552	26221952	-1.096769083	0.001119283	decrease
chr1	205749328	205749728	-0.79305366	0.001120422	decrease
chr9	101533537	101533937	-0.938913784	0.001120857	decrease
chrX	130902901	130903301	-0.80289294	0.001121584	decrease
chr6	27592524	27592924	-0.739374983	0.001121603	decrease
chr19	4471724	4472124	-0.738726084	0.001121635	decrease
chr7	92832820	92833220	-1.114208928	0.001121917	decrease
chr16	28863445	28863845	-0.878356468	0.001122905	decrease
chr9	37592277	37592677	-1.113678103	0.00112714	decrease
chr2	96208666	96209066	-1.05426339	0.001128563	decrease
chr12	118372819	118373219	-1.003703659	0.001128616	decrease
chr5	134758412	134758812	-0.965338892	0.001129235	decrease
chr14	39267114	39267514	-0.967755854	0.001134799	decrease
chr14	77097887	77098287	-1.016389499	0.001135746	decrease
chr6	151451980	151452380	-0.872555767	0.001137067	decrease
chr3	28348882	28349282	-0.823651437	0.001137745	decrease
chr5	126778313	126778713	-0.982236502	0.001138536	decrease
chr5	138575386	138575786	-0.737779204	0.001138907	decrease
chr1	23344329	23344729	-0.99305851	0.001139276	decrease
chr6	116681010	116681410	-0.904912331	0.001143261	decrease
chr2	180006828	180007228	-1.008057987	0.001144419	decrease
chr1	96721698	96722098	-1.11734763	0.00114477	decrease
chr15	72118263	72118663	-0.870926008	0.001150339	decrease
chr11	10808890	10809290	-0.89519884	0.001154423	decrease
chr5	121961631	121962031	-1.039527154	0.001156644	decrease
chr9	34637756	34638156	-0.994002451	0.001156889	decrease
chr7	10940098	10940498	-0.771027585	0.001157258	decrease
chr19	48637365	48637765	-0.807999718	0.001158842	decrease
chr17	38869443	38869843	-0.910400659	0.001161273	decrease
chr2	70248432	70248832	-0.859472903	0.001166248	decrease
chr1	84944786	84945186	-1.039941033	0.001168555	decrease
chr17	8156393	8156793	-0.736193538	0.001169927	decrease
chr1	198935443	198935843	-0.945589792	0.001169944	decrease
chr16	66650831	66651231	-1.029197359	0.001174032	decrease
chr17	58517821	58518221	-0.765378247	0.001175583	decrease
chr11	10857853	10858253	-0.914124276	0.001177153	decrease
chr4	2008936	2009336	-1.135890949	0.001179581	decrease
chr1	95234127	95234527	-1.13473393	0.001180164	decrease
chr7	105012997	105013397	-0.774652505	0.00118156	decrease
chr12	55817585	55817985	-0.872573782	0.001182059	decrease
chr20	33993264	33993664	-0.844681718	0.001184028	decrease
chr6	121334427	121334827	-0.911531352	0.001184926	decrease
chr15	82985995	82986395	-0.918574622	0.001188112	decrease
chr17	80474854	80475254	-0.996771979	0.001188505	decrease
chr19	3626756	3627156	-0.828449936	0.001197219	decrease
chr17	7583693	7584093	-0.852945997	0.001197654	decrease
chr1	147243056	147243456	-1.119475828	0.001199132	decrease
chr2	109613927	109614327	-1.080001474	0.001199675	decrease
chr13	75482266	75482666	-0.972315598	0.001202149	decrease
chrX	68828818	68829218	-0.941118641	0.001203455	decrease
chr1	64471207	64471607	-1.006973154	0.001205036	decrease
chr8	113437924	113438324	-1.08141325	0.001207415	decrease
chr6	42879566	42879966	-0.865176349	0.001208951	decrease
chr12	6606064	6606464	-1.055065962	0.001214034	decrease
chr19	49085113	49085513	-0.764019605	0.00121597	decrease
chr17	45061222	45061622	-0.746843205	0.001215999	decrease
chr6	26327976	26328376	-1.024949184	0.001217027	decrease

chr5	131796998	131797398	-0.883649228	0.001218055	decrease
chr12	57772084	57772484	-0.8618765	0.001219455	decrease
chr3	159765443	159765843	-1.058847008	0.001220843	decrease
chr1	151347024	151347424	-0.929201975	0.00122212	decrease
chr1	116476271	116476671	-1.011773561	0.001223548	decrease
chr16	89557363	89557763	-0.880754926	0.001225829	decrease
chr19	40614227	40614627	-0.850390447	0.001231947	decrease
chr12	76031485	76031885	-1.14034032	0.001233169	decrease
chr22	29267922	29268322	-0.769563496	0.001236293	decrease
chr1	77759443	77759843	-1.028257919	0.001237086	decrease
chr19	45406248	45406648	-0.83325868	0.001240207	decrease
chr3	41962003	41962403	-0.777341062	0.00124451	decrease
chr16	50152596	50152996	-1.057731613	0.001245264	decrease
chr1	28259303	28259703	-0.968370973	0.001246265	decrease
chr5	177607483	177607883	-0.868090645	0.001250135	decrease
chr3	25790083	25790483	-0.869464173	0.001251155	decrease
chr7	99472636	99473036	-0.862412653	0.001253472	decrease
chr1	241847872	241848272	-0.747413882	0.001254499	decrease
chr17	63433810	63434210	-1.051523897	0.001255214	decrease
chr19	50329331	50329731	-0.854120757	0.001258225	decrease
chr14	81170339	81170739	-0.918786668	0.001259958	decrease
chr7	77696134	77696534	-0.868239958	0.001262211	decrease
chr14	23286157	23286557	-0.849606146	0.001262765	decrease
chr16	2868210	2868610	-1.047595598	0.001264489	decrease
chr1	231241091	231241491	-0.806114767	0.001265077	decrease
chr9	18976709	18977109	-0.947990276	0.001265679	decrease
chr1	82136444	82136844	-1.109972193	0.00126622	decrease
chr11	71928665	71929065	-0.830548526	0.001269923	decrease
chr22	40636762	40637162	-0.957353595	0.001273059	decrease
chr8	48921526	48921926	-0.96991896	0.001273117	decrease
chr1	85276482	85276882	-0.792646941	0.001273619	decrease
chr18	21740581	21740981	-0.937130139	0.001275319	decrease
chr11	108121440	108121840	-1.058465907	0.001277035	decrease
chr19	18636513	18636913	-0.768320757	0.001277293	decrease
chr4	129096032	129096432	-1.092102802	0.001279717	decrease
chr2	101001935	101002335	-0.981141388	0.001280576	decrease
chr11	73787960	73788360	-0.936392994	0.001281999	decrease
chr1	33182292	33182692	-1.087819209	0.001283606	decrease
chr22	18001434	18001834	-1.00674056	0.001283972	decrease
chr9	122931327	122931727	-0.867587867	0.001285021	decrease
chr8	143952478	143952878	-1.047174093	0.001287587	decrease
chr17	48585702	48586102	-1.022456838	0.001287947	decrease
chr2	69914604	69915004	-0.717406986	0.00128885	decrease
chr5	123511814	123512214	-0.992551068	0.001294025	decrease
chr5	134904305	134904705	-1.106335343	0.001294502	decrease
chr11	695307	695707	-0.754625765	0.001299704	decrease
chr18	22933209	22933609	-0.958243002	0.001302049	decrease
chr1	222712469	222712869	-0.840367727	0.001302465	decrease
chr1	117605506	117605906	-0.952297453	0.001303506	decrease
chr16	11915938	11916338	-0.983210711	0.001306097	decrease
chr9	126804881	126805281	-1.013984611	0.001312123	decrease
chr7	44123515	44123915	-1.076800921	0.001320743	decrease
chr21	33025482	33025882	-0.830924346	0.001321004	decrease
chr5	154754801	154755201	-0.928326166	0.001322259	decrease
chr10	119178488	119178888	-0.957178816	0.001324154	decrease
chr17	28951840	28952240	-0.713602475	0.001324178	decrease
chr10	97318895	97319295	-1.035974592	0.001328357	decrease
chr5	115841542	115841942	-1.095773264	0.001329671	decrease
chr9	127424031	127424431	-0.95253876	0.001330032	decrease
chr19	10251755	10252155	-0.82626864	0.001332741	decrease
chr1	76040367	76040767	-0.972410466	0.001333268	decrease
chr4	53377468	53377868	-1.069505784	0.001336142	decrease
chr2	202265627	202266027	-0.938750822	0.001336445	decrease
chr13	39038047	39038447	-0.84005696	0.001341754	decrease
chr9	70418359	70418759	-0.821743741	0.00134192	decrease
chr12	31073865	31074265	-0.848671281	0.001342914	decrease
chr2	231781260	231781660	-0.793731084	0.001349203	decrease
chr19	19203035	19203435	-0.993696738	0.001351254	decrease
chr11	64688499	64688899	-0.926993763	0.001353217	decrease
chr14	24442351	24442751	-0.781779261	0.001354919	decrease
chr16	3264444	3264844	-0.952128373	0.001359021	decrease
chr1	32179254	32179654	-0.889231471	0.001361986	decrease
chr3	150603041	150603441	-0.821944377	0.00136301	decrease
chr7	122144052	122144452	-1.096203418	0.001363347	decrease
chr7	124929660	124930060	-0.915963586	0.001367373	decrease
chr7	44044296	44044696	-1.081983893	0.001367408	decrease

chrX	72181332	72181732	-0.752258036	0.00137034	decrease
chr10	14837886	14838286	-0.944892921	0.001375377	decrease
chr4	2242087	2242487	-0.94221839	0.00137713	decrease
chr19	46346959	46347359	-0.959914897	0.001378996	decrease
chr22	21652029	21652429	-0.899404222	0.001379705	decrease
chr19	11155729	11156129	-0.884963478	0.001385327	decrease
chr18	5629680	5630080	-1.09923808	0.001391499	decrease
chr22	41946739	41947139	-0.771900019	0.00139302	decrease
chr1	31296837	31297237	-0.911761898	0.001395516	decrease
chr3	167734780	167735180	-0.644456572	0.001396415	decrease
chr12	49130690	49131090	-0.839087561	0.001404059	decrease
chr5	171387471	171387871	-0.692122231	0.001407774	decrease
chr17	75779750	75780150	-0.899065549	0.001408182	decrease
chr13	79406268	79406668	-0.828669611	0.001408324	decrease
chr1	197146504	197146904	-0.950771524	0.00142387	decrease
chr1	220089684	220090084	-0.758638137	0.00143058	decrease
chr4	147617267	147617667	-0.916154928	0.001432329	decrease
chr4	82891117	82891517	-0.918761481	0.001442355	decrease
chr14	20469148	20469548	-0.834623568	0.001446866	decrease
chr16	84013995	84014395	-0.748347773	0.001454907	decrease
chr14	20461520	20461920	-0.848680615	0.00145937	decrease
chr21	29073631	29074031	-0.883806998	0.001460304	decrease
chr9	100099014	100099414	-0.771209966	0.001460382	decrease
chr11	113014502	113014902	-1.108689072	0.001462657	decrease
chr6	43575471	43575871	-1.04190879	0.001480076	decrease
chr7	5822760	5823160	-0.931221605	0.001485701	decrease
chr1	65420107	65420507	-0.812225455	0.001490529	decrease
chr1	150163040	150163440	-0.942899712	0.001505432	decrease
chr1	15849811	15850211	-0.963724243	0.00151386	decrease
chr3	38137119	38137519	-0.846176809	0.001516158	decrease
chr13	94549379	94549779	-0.738867132	0.001516315	decrease
chr2	169573447	169573847	-0.941214557	0.001520361	decrease
chr1	200669708	200670108	-0.878301966	0.001522626	decrease
chr1	55213869	55214269	-0.84510763	0.00152283	decrease
chr17	64975232	64975632	-0.956741819	0.001528302	decrease
chr3	127597925	127598325	-1.014857887	0.001530855	decrease
chr15	41332381	41332781	-0.959402883	0.001533596	decrease
chr1	43763556	43763956	-1.110560829	0.001535741	decrease
chr22	42444222	42444622	-1.015413971	0.001538661	decrease
chr7	100827638	100828038	-0.975564295	0.001539681	decrease
chr6	26032064	26032464	-0.910816416	0.001539749	decrease
chr11	9460670	9461070	-0.84690293	0.001543476	decrease
chr12	106955360	106955760	-1.023396558	0.001545172	decrease
chrX	64976200	64976600	-1.095238103	0.001545864	decrease
chr15	89672912	89673312	-1.076112827	0.001550013	decrease
chr7	45768958	45769358	-0.93042165	0.001551927	decrease
chr2	121755464	121755864	-0.936699908	0.001553502	decrease
chr1	236794972	236795372	-0.731897122	0.001557368	decrease
chr2	178451047	178451447	-0.936812733	0.001561991	decrease
chr15	75625782	75626182	-0.924666155	0.001564677	decrease
chr2	64453299	64453699	-1.103605124	0.001567628	decrease
chr2	74483004	74483404	-0.781882423	0.001568018	decrease
chr6	43170754	43171154	-0.840861978	0.001570568	decrease
chrX	37349262	37349662	-1.107802911	0.001576522	decrease
chr19	18418703	18419103	-0.918574181	0.001579122	decrease
chr7	6009053	6009453	-0.935857336	0.001579681	decrease
chr12	119667812	119668212	-0.947471225	0.001582235	decrease
chr11	90223085	90223485	-1.00420346	0.00158406	decrease
chr16	30021348	30021748	-0.869907376	0.00158474	decrease
chr5	138543240	138543640	-0.780284386	0.001596308	decrease
chr11	58578486	58578886	-0.884448355	0.00159877	decrease
chr4	90127232	90127632	-1.051872335	0.00160355	decrease
chr2	231464582	231464982	-0.866921514	0.001603605	decrease
chr5	10441555	10441955	-0.916280165	0.001614235	decrease
chr10	63629463	63629863	-0.784218951	0.00161546	decrease
chr2	134918459	134918859	-0.974645561	0.001615833	decrease
chr9	97633226	97633626	-0.895863924	0.001619612	decrease
chr17	7931904	7932304	-0.823090496	0.001624753	decrease
chr6	26457638	26458038	-0.980551691	0.001625432	decrease
chr5	129094210	129094610	-1.071535077	0.001627723	decrease
chr2	32165529	32165929	-0.894078321	0.001628736	decrease
chr15	101293355	101293755	-1.038466966	0.001632824	decrease
chr18	22169755	22170155	-1.051351687	0.001636416	decrease
chr15	72231601	72232001	-0.920307679	0.001636728	decrease
chr19	8525645	8526045	-0.737298041	0.001640557	decrease
chr14	90331604	90332004	-0.940676612	0.001641317	decrease

chr1	156740913	156741313	-0.819629595	0.001641796	decrease
chr13	41194277	41194677	-1.023784221	0.001646792	decrease
chr16	1960804	1961204	-0.693080778	0.001649906	decrease
chr19	17215297	17215697	-0.950915497	0.001654144	decrease
chr17	39199958	39200358	-0.886925678	0.001656332	decrease
chr1	161612482	161612882	-0.813543517	0.00166075	decrease
chr19	8513564	8513964	-0.925371141	0.001660864	decrease
chr12	48197818	48198218	-0.897395839	0.001661904	decrease
chr19	41263857	41264257	-0.756292507	0.001665416	decrease
chr17	7560764	7561164	-0.876578674	0.001668283	decrease
chr15	74760145	74760545	-0.940790891	0.001672552	decrease
chr5	95961949	95962349	-0.984829581	0.00167476	decrease
chr8	89984047	89984447	-0.877635742	0.001677235	decrease
chr6	132768052	132768452	-1.044594662	0.001682551	decrease
chr11	57741214	57741614	-0.94996508	0.001688205	decrease
chr8	109334249	109334649	-0.829167072	0.001697089	decrease
chr7	92134579	92134979	-0.834084966	0.001702791	decrease
chr15	41230946	41231346	-0.757511586	0.001707225	decrease
chrX	101097902	101098302	-0.921646564	0.001707233	decrease
chr6	84764396	84764796	-0.876761847	0.001709775	decrease
chr11	65663020	65663420	-1.022923063	0.001716753	decrease
chr1	207321209	207321609	-0.850436931	0.001726626	decrease
chr6	122471598	122471998	-0.975116981	0.001727044	decrease
chr1	211675614	211676014	-0.701326277	0.001731274	decrease
chr12	120201183	120201583	-0.795338644	0.001734738	decrease
chr3	38003466	38003866	-0.958875286	0.00173546	decrease
chr15	42273175	42273575	-0.878964106	0.001738206	decrease
chr12	6961874	6962274	-0.725999356	0.001738484	decrease
chr14	22589500	22589900	-0.868207945	0.001741614	decrease
chr4	134200701	134201101	-1.00706391	0.00174989	decrease
chr1	154870093	154870493	-1.032708805	0.001751776	decrease
chr9	34665217	34665617	-0.95588242	0.001760051	decrease
chr7	5425617	5426017	-0.871481281	0.001766537	decrease
chr10	69123668	69124068	-0.800612107	0.001767065	decrease
chr4	102868981	102869381	-0.885113447	0.001768776	decrease
chr4	139295767	139296167	-1.007310453	0.001771024	decrease
chr1	228059773	228060173	-0.864023328	0.001771131	decrease
chr17	44111306	44111706	-1.033917093	0.001774234	decrease
chr14	60091771	60092171	-0.834884108	0.001775443	decrease
chr7	100091070	100091470	-0.813006504	0.00178846	decrease
chr17	59892950	59893350	-0.904969899	0.001790294	decrease
chr5	75511626	75512026	-0.958782648	0.001791332	decrease
chr1	151327360	151327760	-0.878057271	0.001793683	decrease
chr6	53651744	53652144	-1.015495048	0.001797022	decrease
chr5	65927491	65927891	-1.054241789	0.001798835	decrease
chr16	86575925	86576325	-1.077287269	0.001799183	decrease
chr12	52069533	52069933	-0.732602253	0.001799382	decrease
chr2	96638310	96638710	-0.824532181	0.00180346	decrease
chr1	50976949	50977349	-1.003107737	0.001805072	decrease
chr2	26345749	26346149	-0.860243773	0.001808945	decrease
chrX	135004024	135004424	-0.912705141	0.001812134	decrease
chr20	45910847	45911247	-0.952590318	0.001819632	decrease
chr19	50147978	50148378	-0.841631943	0.001830892	decrease
chr15	70101664	70102064	-0.835432113	0.001832243	decrease
chr7	140175262	140175662	-0.964869537	0.0018334	decrease
chr9	6757543	6757943	-0.837324364	0.001834309	decrease
chr8	73174140	73174540	-1.057587474	0.001837915	decrease
chr20	41028438	41028838	-0.958061562	0.001845811	decrease
chr6	109095199	109095599	-0.823020525	0.001847448	decrease
chr1	108692175	108692575	-0.797714558	0.001849955	decrease
chr22	21712996	21713396	-0.954537081	0.001852132	decrease
chr3	52057011	52057411	-0.892821816	0.001852755	decrease
chr9	113340065	113340465	-0.925188854	0.001854558	decrease
chr2	3518820	3519220	-0.910474698	0.001855455	decrease
chr8	98117097	98117497	-0.883155498	0.001856859	decrease
chr15	75180716	75181116	-0.855968835	0.001861241	decrease
chr11	33161291	33161691	-0.98315895	0.001873926	decrease
chr6	34887903	34888303	-0.98723977	0.001874038	decrease
chr1	228647686	228648086	-0.705952249	0.001874641	decrease
chr14	53217344	53217744	-1.034217682	0.001879502	decrease
chr1	183635906	183636306	-0.845993772	0.001882175	decrease
chr7	133081994	133082394	-0.885776048	0.001883486	decrease
chr19	57320301	57320701	-0.880372546	0.001887676	decrease
chr13	78658809	78659209	-0.888090519	0.001887824	decrease
chr20	62926499	62926899	-0.985204937	0.001890395	decrease
chr17	74873212	74873612	-0.87100834	0.001896609	decrease

chr15	77633529	77633929		-0.911432231	0.001902149	decrease
chr1	12478266	12478666		-0.978589421	0.001906843	decrease
chr6	79537125	79537525		-0.824278205	0.001906988	decrease
chr22	19179142	19179542		-0.879150135	0.001909249	decrease
chr19	55461465	55461865		-0.893217421	0.001921545	decrease
chr1	151539981	151540381		-0.815825192	0.001923045	decrease
chr12	45727769	45728169		-0.892231681	0.001923815	decrease
chr2	26785640	26786040		-0.991410703	0.001924102	decrease
chr8	133571571	133571971		-1.006711356	0.001929757	decrease
chr11	62805338	62805738		-0.97730811	0.001930681	decrease
chr2	200966673	200967073		-1.031011825	0.001932485	decrease
chr13	49444043	49444443		-0.79365905	0.001941698	decrease
chr6	149565778	149566178		-0.929582015	0.001942166	decrease
chr16	57186043	57186443		-0.87983289	0.001942491	decrease
chr7	127651693	127652093		-0.779294859	0.001946592	decrease
chr3	45593067	45593467		-0.915478455	0.001953801	decrease
chr1	156767099	156767499		-0.783392108	0.00195631	decrease
chr7	99408729	99409129		-0.850961582	0.001956478	decrease
chr2	127810955	127811355		-0.850324785	0.001958451	decrease
chr19	3700540	3700940		-0.767861677	0.00196528	decrease
chr1	150236050	150236450		-0.732157218	0.001969953	decrease
chrX	74292562	74292962		-0.910750642	0.001973996	decrease
chr1	35193098	35193498		-0.853574748	0.001976222	decrease
chr2	183172252	183172652		-1.079264443	0.001978201	decrease
chr9	75890361	75890761		-0.955856514	0.001978207	decrease
chr3	112561994	112562394		-0.751676402	0.001978225	decrease
chr8	96262670	96263070		-0.849612734	0.001979793	decrease
chr4	109815448	109815848		-0.994669529	0.001980407	decrease
chr7	44036377	44036777		-1.021728732	0.001990114	decrease
chr21	37072788	37073188		-1.057410284	0.001992237	decrease
chrX	132957785	132958185		-1.073102125	0.001994221	decrease
chr5	61162063	61162463		-1.004957528	0.001999667	decrease
chr12	102120097	102120497		-0.861994279	0.001999939	decrease
chr19	49665582	49665982		-0.85323455	0.002000904	decrease
chr13	44989276	44989676		-0.885676185	0.002001707	decrease
chr11	66616252	66616652		-0.789049473	0.002017604	decrease
chr7	66114876	66115276		-0.915638079	0.002022153	decrease
chr5	179550985	179551385		-1.082730305	0.002029905	decrease
chr3	47788858	47789258		-1.014169745	0.002034092	decrease
chr2	24084201	24084601		-0.868067173	0.002036088	decrease
chr17	75426169	75426569		-1.050762878	0.002036257	decrease
chr4	55395523	55395923		-0.958906792	0.002048339	decrease
chr1	154220311	154220711		-0.80048646	0.002059479	decrease
chr18	69838985	69839385		-0.85806133	0.002067645	decrease
chr19	37906795	37907195		-1.008404372	0.00207188	decrease
chr6	135497474	135497874		-1.018019917	0.002078838	decrease
chr19	51367482	51367882		-0.843432297	0.002088067	decrease
chr11	65859456	65859856		-0.70026619	0.00209559	decrease
chr1	155193514	155193914		-0.809461387	0.002097445	decrease
chr11	111870872	111871272		-0.973338465	0.002100332	decrease
chr3	64685970	64686370		-0.951308576	0.002101833	decrease
chr16	23557232	23557632		-0.850644695	0.002108904	decrease
chr18	31041519	31041919		-0.943438636	0.002116642	decrease
chr17	58679398	58679798		-0.739739951	0.002117092	decrease
chr10	13302103	13302503		-0.939797531	0.002127319	decrease
chr5	36151799	36152199		-0.898551299	0.002127405	decrease
chr19	41884110	41884510		-0.844235043	0.002129675	decrease
chr8	22244988	22245388		-0.985655321	0.002131718	decrease
chr2	66070977	66071377		-0.96560873	0.002134466	decrease
chr1	100351429	100351829		-0.770476499	0.002134618	decrease
chr11	71786746	71787146		-0.851458778	0.002137329	decrease
chr1	30824196	30824596		-0.783160023	0.002144702	decrease
chrX	24693758	24694158		-1.032103734	0.002147671	decrease
chr1	40374379	40374779		-0.951898161	0.00215422	decrease
chr6	3063866	3064266		-1.055938706	0.002159581	decrease
chr15	69462540	69462940		-0.836108359	0.002162996	decrease
chr5	65721763	65722163		-1.04889391	0.002167789	decrease
chr17	42154942	42155342		-0.837370669	0.002176481	decrease
chr13	102596471	102596871		-1.066000672	0.002182232	decrease
chr17	49788817	49789217		-0.797026094	0.00218532	decrease
chr2	183038508	183038908		-1.043210725	0.00218545	decrease
chr19	48170290	48170690		-0.891462124	0.00220419	decrease
chr3	63911604	63912004		-1.044795116	0.002204784	decrease
chr11	46700561	46700961		-0.820959612	0.00220885	decrease
chr22	36481674	36482074		-0.873320132	0.002215804	decrease
chr1	109548467	109548867		-0.904619846	0.002218898	decrease

chr11	65989162	65989562	-0.951682089	0.002223321	decrease
chr4	3075252	3075652	-0.953740853	0.002236147	decrease
chr20	63956434	63956834	-1.007819661	0.002237643	decrease
chr7	151028347	151028747	-0.644485926	0.002244042	decrease
chr12	2004182	2004582	-0.912523093	0.002248946	decrease
chr16	57735291	57735691	-0.842708278	0.00224945	decrease
chr6	131627943	131628343	-0.894611719	0.002251531	decrease
chr1	187073044	187073444	-0.93653975	0.002251553	decrease
chr15	77821302	77821702	-1.034280666	0.002253934	decrease
chr16	31458192	31458592	-0.912904253	0.002257184	decrease
chr15	90233316	90233716	-0.943385233	0.002258447	decrease
chr4	76377788	76378188	-1.052239557	0.002258451	decrease
chr17	80415025	80415425	-0.907543365	0.002264342	decrease
chr5	128084478	128084878	-1.042274585	0.00226768	decrease
chr10	104232162	104232562	-0.9104171	0.002268534	decrease
chr15	98852714	98853114	-0.794868026	0.002270422	decrease
chr3	49133278	49133678	-0.676902369	0.002271339	decrease
chr12	54028574	54028974	-0.979982554	0.002272494	decrease
chr3	38164829	38165229	-0.71533102	0.002277895	decrease
chr5	90529400	90529800	-0.857167662	0.002279193	decrease
chr2	73214134	73214534	-0.878526171	0.002282798	decrease
chr21	31559577	31559977	-0.868380221	0.002285933	decrease
chr15	78437967	78438367	-0.883182621	0.002286137	decrease
chr2	95165476	95165876	-0.911417858	0.002286462	decrease
chr15	78873578	78873978	-0.949916747	0.002293779	decrease
chr4	140756644	140757044	-0.855811545	0.002296001	decrease
chr19	45178352	45178752	-0.711220764	0.00230408	decrease
chr1	107057083	107057483	-0.929940258	0.00230584	decrease
chr16	30610266	30610666	-0.790329527	0.002308286	decrease
chr17	68247634	68248034	-0.963462229	0.002310613	decrease
chr12	132908440	132908840	-0.997456065	0.002315325	decrease
chr10	102645000	102645400	-0.974953545	0.002320176	decrease
chr6	159726843	159727243	-0.830557938	0.002328435	decrease
chr20	43590614	43591014	-0.910690924	0.002330845	decrease
chr3	78994546	78994946	-0.856468054	0.00233266	decrease
chr5	36875059	36875459	-0.992902483	0.002334125	decrease
chr12	6724003	6724403	-0.871956344	0.002334245	decrease
chr21	29302211	29302611	-0.980888465	0.002337397	decrease
chr16	29863216	29863616	-0.701920567	0.002339039	decrease
chr7	45111664	45112064	-0.860069912	0.002343574	decrease
chr11	448113	448513	-0.942194313	0.002343636	decrease
chr17	7313263	7313663	-0.749899433	0.002344606	decrease
chr2	9630870	9631270	-0.840854616	0.002344744	decrease
chr3	38455471	38455871	-0.959944999	0.002345556	decrease
chr12	132887619	132888019	-1.014603197	0.002348358	decrease
chr1	112957067	112957467	-0.891384475	0.002348709	decrease
chr22	35299756	35300156	-0.83280358	0.002350964	decrease
chr3	40524603	40525003	-0.96038797	0.002354065	decrease
chr4	13544875	13545275	-1.015829887	0.002367009	decrease
chr5	138178652	138179052	-0.812313995	0.00237796	decrease
chr11	46616892	46617292	-1.054855058	0.002379152	decrease
chr22	38705448	38705848	-0.737941473	0.002380503	decrease
chr6	91052780	91053180	-1.034195596	0.002380632	decrease
chr2	88016651	88017051	-0.824936139	0.002384744	decrease
chr7	23014043	23014443	-1.026918672	0.00238655	decrease
chr13	91358640	91359040	-0.907083281	0.002387153	decrease
chr13	42047739	42048139	-1.001283439	0.002388459	decrease
chr8	23246499	23246899	-1.035663451	0.002389721	decrease
chrX	70133313	70133713	-0.924747296	0.002390642	decrease
chr4	39459095	39459495	-0.927205537	0.002392923	decrease
chr21	14382896	14383296	-0.713800435	0.002405743	decrease
chr3	152269592	152269992	-0.994333696	0.002410973	decrease
chr1	66924886	66925286	-0.689476166	0.002411838	decrease
chr11	65833984	65834384	-1.017615545	0.002413051	decrease
chr17	6640767	6641167	-0.926525261	0.002423332	decrease
chr17	76353886	76354286	-0.825931686	0.002426099	decrease
chr1	151006598	151006998	-0.919074607	0.002434308	decrease
chr7	156640592	156640992	-0.860805231	0.002448293	decrease
chr10	114174145	114174545	-0.865679091	0.002448786	decrease
chr9	34633974	34634374	-0.964730196	0.002452344	decrease
chr9	33024874	33025274	-0.956157884	0.002454374	decrease
chr14	22956855	22957255	-0.941669978	0.002454896	decrease
chr10	73997724	73998124	-0.823055335	0.002456666	decrease
chr9	22009073	22009473	-0.93693284	0.002459283	decrease
chr19	12681234	12681634	-0.68700926	0.002464184	decrease
chr6	150005170	150005570	-0.89204277	0.002466191	decrease

chr5	57173692	57174092	-0.776865124	0.002476706	decrease
chr3	12556916	12557316	-0.927932071	0.002479538	decrease
chr12	53220459	53220859	-0.970959123	0.002480423	decrease
chr9	20622610	20623010	-1.062229078	0.002480564	decrease
chr11	61361899	61362299	-0.796594568	0.002486885	decrease
chr7	100148582	100148982	-0.872462686	0.002488524	decrease
chr1	146051894	146052294	-0.82716554	0.002489694	decrease
chr6	116571074	116571474	-0.736715112	0.002497888	decrease
chr22	20981902	20982302	-0.928418231	0.002498975	decrease
chr7	13990576	13990976	-0.942660478	0.002502476	decrease
chr10	47553184	47553584	-0.734496329	0.002505035	decrease
chr10	96719715	96720115	-1.033864433	0.00250512	decrease
chr11	108497365	108497765	-0.968427548	0.002508146	decrease
chr2	85561485	85561885	-0.950758825	0.002515118	decrease
chr1	151281901	151282301	-0.585891203	0.00252234	decrease
chr8	51899137	51899537	-0.806915835	0.002537294	decrease
chr11	106022142	106022542	-0.843760672	0.0025374	decrease
chr2	218710830	218711230	-0.995593701	0.00253951	decrease
chr17	71033018	71033418	-0.937990022	0.002539683	decrease
chr6	132820673	132821073	-0.899133706	0.00254285	decrease
chr1	229558918	229559318	-0.792510584	0.002547519	decrease
chr5	172187520	172187920	-0.986967439	0.002549184	decrease
chr11	60915273	60915673	-0.846093405	0.002550694	decrease
chr12	131833369	131833769	-0.933419058	0.002561105	decrease
chr13	67136136	67136536	-0.739226516	0.002568271	decrease
chr14	75660954	75661354	-0.800199705	0.002571231	decrease
chr3	186570326	186570726	-0.850767108	0.002572602	decrease
chr1	23368301	23368701	-0.845667684	0.002576931	decrease
chr3	12841414	12841814	-0.853513201	0.002579251	decrease
chr2	227324971	227325371	-1.011094995	0.002580232	decrease
chr17	43170714	43171114	-0.777406591	0.002583303	decrease
chr6	147202378	147202778	-0.884524681	0.002585053	decrease
chr16	30875111	30875511	-0.759411633	0.002587841	decrease
chr11	96307028	96307428	-0.851210114	0.002589014	decrease
chr12	120302218	120302618	-0.93403495	0.002592376	decrease
chr4	42657194	42657594	-0.897737056	0.00259517	decrease
chr17	1469179	1469579	-0.822383673	0.00260245	decrease
chr1	100896060	100896460	-0.845697249	0.002608234	decrease
chr1	35031671	35032071	-0.923357018	0.002608492	decrease
chr6	79233907	79234307	-0.880101277	0.00261099	decrease
chrX	53422425	53422825	-0.753234849	0.002620242	decrease
chr3	107429473	107429873	-0.986449118	0.002623962	decrease
chr11	46243297	46243697	-0.97133044	0.002627484	decrease
chr2	177263557	177263957	-0.976448309	0.002629389	decrease
chr17	8630720	8631120	-0.941511565	0.002638413	decrease
chr19	39445376	39445776	-0.855019284	0.002640336	decrease
chr8	26291096	26291496	-1.032419874	0.002642276	decrease
chr1	113929177	113929577	-0.949208723	0.00264244	decrease
chr15	75204641	75205041	-0.849367433	0.002655494	decrease
chr10	15096853	15097253	-0.91172888	0.002659645	decrease
chr1	108963562	108963962	-1.009273503	0.002669885	decrease
chr2	9843298	9843698	-0.968389609	0.002670135	decrease
chrX	129982464	129982864	-0.806310598	0.002672548	decrease
chr16	87560684	87561084	-0.793988717	0.002676277	decrease
chr3	134795009	134795409	-0.958222355	0.002684276	decrease
chr20	35454742	35455142	-0.744003545	0.002684515	decrease
chr14	39103193	39103593	-0.935924789	0.002692925	decrease
chr2	126657544	126657944	-0.840820177	0.002694842	decrease
chr11	57515716	57516116	-1.028510268	0.002695213	decrease
chr19	49128160	49128560	-0.8730952	0.002696141	decrease
chr10	22554721	22555121	-0.986166563	0.002699797	decrease
chr19	58475778	58476178	-0.764792772	0.002700477	decrease
chr19	7069332	7069732	-0.855433846	0.002702765	decrease
chrX	118823719	118824119	-0.92667885	0.002703346	decrease
chr2	73737289	73737689	-0.814734111	0.002703434	decrease
chr16	77212941	77213341	-0.994980153	0.002709641	decrease
chr2	169479405	169479805	-0.907677104	0.002713358	decrease
chr10	132335357	132335757	-0.70376445	0.002719102	decrease
chr7	44559990	44560390	-0.996372973	0.002728872	decrease
chr5	140691217	140691617	-0.837694388	0.00273112	decrease
chr19	2273441	2273841	-0.894648192	0.002738591	decrease
chrX	154378894	154379294	-0.785733736	0.00273949	decrease
chr20	290384	290784	-0.953671332	0.002747764	decrease
chr6	135054733	135055133	-0.684343174	0.00275008	decrease
chr10	94362709	94363109	-0.879954811	0.00275586	decrease
chr17	50161437	50161837	-0.96650565	0.002762476	decrease

chr12	14257710	14258110	-0.763923017	0.002762793	decrease
chr20	19758597	19758997	-1.006199812	0.002763429	decrease
chr18	34493145	34493545	-0.97566616	0.002765953	decrease
chr14	21476940	21477340	-0.91521484	0.002771696	decrease
chr10	132537419	132537819	-0.927241556	0.002774571	decrease
chr19	39390903	39391303	-0.782190094	0.002774833	decrease
chr2	241149738	241150138	-0.839007351	0.002783946	decrease
chr17	7242442	7242842	-0.861510984	0.00278607	decrease
chr12	93886411	93886811	-0.926617606	0.002786682	decrease
chr15	45023038	45023438	-0.984020589	0.002793203	decrease
chrX	123648517	123648917	-0.934641048	0.002799325	decrease
chr12	106774156	106774556	-0.748341246	0.002802248	decrease
chr19	12736999	12737399	-0.65407839	0.002812454	decrease
chr4	88456712	88457112	-0.978452828	0.002814267	decrease
chr4	70993257	70993657	-0.916887506	0.002817701	decrease
chr9	35657760	35658160	-0.778498432	0.002818141	decrease
chr14	37171996	37172396	-0.938025487	0.002827622	decrease
chr11	65122115	65122515	-0.811105609	0.002831956	decrease
chr7	72829554	72829954	-0.916089822	0.002833655	decrease
chr7	134986455	134986855	-0.808400795	0.002835024	decrease
chr16	28491948	28492348	-0.872214282	0.00283595	decrease
chr16	3282705	3283105	-0.939728347	0.002836772	decrease
chr14	104794247	104794647	-0.871243273	0.002841215	decrease
chr1	149899459	149899859	-0.817186067	0.002841774	decrease
chr14	60248332	60248732	-0.929347043	0.002847577	decrease
chr6	96897672	96898072	-0.883164965	0.002848137	decrease
chr7	157010755	157011155	-0.950042789	0.002849267	decrease
chr13	79307735	79308135	-0.933857249	0.002857174	decrease
chr12	56189871	56190271	-0.966507142	0.002860545	decrease
chr9	131395561	131395961	-0.8512191	0.00286317	decrease
chr2	135875601	135876001	-1.025442723	0.002864264	decrease
chr13	38686772	38687172	-0.86860333	0.002865422	decrease
chr1	45550720	45551120	-0.87937893	0.002867832	decrease
chr8	53842924	53843324	-0.874513925	0.002874006	decrease
chr22	50264500	50264900	-0.946575008	0.00287498	decrease
chr17	28719480	28719880	-0.760530904	0.002876069	decrease
chrX	104112583	104112983	-0.96778295	0.002883789	decrease
chr7	129225383	129225783	-1.01940451	0.002885444	decrease
chr15	99251053	99251453	-0.937386719	0.002887071	decrease
chr4	107036974	107037374	-1.031817345	0.002888104	decrease
chr1	246931910	246932310	-0.905482722	0.00288833	decrease
chr11	119095372	119095772	-0.859320553	0.002888999	decrease
chr13	75903959	75904359	-0.914114569	0.002890313	decrease
chr10	101060691	101061091	-0.881767997	0.002892182	decrease
chr1	18902498	18902898	-0.928411423	0.002892292	decrease
chr1	32992301	32992977	-0.878571807	0.002894104	decrease
chrX	40580468	40580868	-1.027343232	0.002903638	decrease
chr11	72080581	72080981	-0.854262656	0.002907404	decrease
chr6	43629569	43629969	-0.776182684	0.002907882	decrease
chr11	66371928	66372328	-0.923798503	0.002908171	decrease
chr1	236281034	236281434	-0.949292111	0.002909393	decrease
chr9	77177045	77177445	-0.827455908	0.002913285	decrease
chr2	74472218	74472618	-0.969704449	0.002915997	decrease
chr1	151790485	151790885	-0.769692081	0.002921268	decrease
chr10	35642312	35642712	-1.014854919	0.002924511	decrease
chr6	107489036	107489436	-0.989933583	0.00293064	decrease
chr1	169485770	169486170	-0.809542174	0.002934297	decrease
chr4	95549370	95549770	-1.012734727	0.002939911	decrease
chr1	6199560	6199960	-0.980991436	0.002940764	decrease
chr1	193059415	193059815	-0.722060489	0.002942872	decrease
chr11	31369766	31370166	-0.941942497	0.002944396	decrease
chr20	46689424	46689824	-0.966198407	0.002944499	decrease
chr13	30465853	30466253	-0.934840653	0.002945741	decrease
chr19	50376469	50376869	-0.88265726	0.002951149	decrease
chr17	75261573	75261973	-0.768305733	0.002953363	decrease
chr16	18790203	18790603	-0.822601319	0.002953985	decrease
chr12	68807681	68808081	-0.995557227	0.002954431	decrease
chr15	74889804	74890204	-0.837184187	0.002956031	decrease
chr1	44405216	44405616	-1.029685142	0.002961341	decrease
chr7	151085980	151086380	-0.783052146	0.002962378	decrease
chr17	46922822	46923222	-0.753305432	0.002965159	decrease
chr10	45672744	45673144	-0.933085412	0.00296747	decrease
chr10	104121668	104122068	-0.843488208	0.002967956	decrease
chr15	75738568	75738968	-0.905707201	0.002968018	decrease
chr7	101743421	101743821	-1.036612424	0.00297244	decrease
chr15	74614861	74615261	-0.914169559	0.002973298	decrease

chr1	239408097	239408497	-0.8839526	0.002977493	decrease
chr2	149587794	149588194	-0.787273192	0.002978798	decrease
chr1	31644747	31645147	-0.791494475	0.002979002	decrease
chr22	41381439	41381839	-0.805268316	0.002979439	decrease
chr11	123062105	123062505	-0.658402517	0.00298321	decrease
chr6	108848142	108848542	-0.842701984	0.002991583	decrease
chr17	81513608	81514008	-0.774819073	0.002998706	decrease
chr18	10122077	10122477	-1.047747175	0.002999382	decrease
chr1	112700658	112701058	-0.78358938	0.003008772	decrease
chr7	99465926	99466326	-0.852146513	0.00300905	decrease
chr5	98929153	98929553	-0.808135522	0.00301047	decrease
chr1	90062762	90063162	-0.999880009	0.00301248	decrease
chr12	132593895	132594295	-0.831378216	0.00301433	decrease
chr2	192195073	192195473	-0.951322157	0.00302184	decrease
chr9	76394829	76395229	-0.940038067	0.003022419	decrease
chr2	47904442	47904842	-1.003038062	0.003028248	decrease
chr9	5628571	5628971	-0.938973429	0.00302986	decrease
chr2	27663474	27663874	-0.881883968	0.00303023	decrease
chr17	7842268	7842668	-0.857988444	0.003033591	decrease
chr2	231706854	231707254	-0.652733826	0.003036241	decrease
chr4	110411448	110411848	-0.866607367	0.003038562	decrease
chr7	21427429	21427829	-0.81509964	0.003040522	decrease
chr4	169010194	169010594	-0.922197931	0.00304164	decrease
chr1	114669781	114670181	-0.786308369	0.003046739	decrease
chr21	33915641	33916041	-0.73828764	0.003048757	decrease
chr11	66438761	66439161	-0.71511251	0.003057716	decrease
chr15	41972575	41972975	-1.03188162	0.003065103	decrease
chr10	102057909	102058309	-0.965847133	0.003075005	decrease
chr10	133394211	133394611	-0.914425574	0.003076401	decrease
chr9	128067557	128067957	-0.733432092	0.003080804	decrease
chr14	74881498	74881898	-0.957096054	0.003086208	decrease
chr19	2599053	2599453	-1.039054667	0.003088872	decrease
chr19	49817906	49818306	-0.857753221	0.003091477	decrease
chr2	11744154	11744554	-0.819284383	0.003091616	decrease
chr1	45521848	45522248	-0.844617764	0.003092887	decrease
chr19	48492895	48493295	-0.675305935	0.003092991	decrease
chr20	35171887	35172287	-0.995225885	0.003098969	decrease
chr9	127388254	127388654	-0.856058327	0.003101837	decrease
chr19	13774194	13774594	-0.90193436	0.003105922	decrease
chr4	76147976	76148376	-0.778734052	0.003107259	decrease
chr9	33473696	33474096	-0.78763992	0.003107944	decrease
chr3	49429270	49429670	-0.905712348	0.003111986	decrease
chr5	78457522	78457922	-1.011210621	0.003113651	decrease
chr5	148320136	148320536	-1.031226812	0.003114726	decrease
chr6	149941898	149942298	-0.7764305	0.003116593	decrease
chr3	47164143	47164543	-1.015863711	0.003118782	decrease
chr1	67832518	67832918	-0.907606284	0.003120794	decrease
chr19	17539706	17540106	-1.016969427	0.00312124	decrease
chr2	73071341	73071741	-0.817267931	0.003121816	decrease
chr5	179858681	179859081	-0.701200407	0.00312193	decrease
chr5	132293785	132294185	-0.792871962	0.003122319	decrease
chr5	93620810	93621210	-0.988042232	0.003122522	decrease
chr7	130440796	130441196	-1.014508255	0.003122828	decrease
chr15	62060201	62060601	-1.019521257	0.003124063	decrease
chr15	80059182	80059582	-0.933197889	0.003142284	decrease
chr15	90954730	90955130	-0.754298128	0.003148161	decrease
chr14	99604407	99604807	-0.947636464	0.003159685	decrease
chr19	58278636	58279036	-0.787426437	0.003176047	decrease
chrX	154489728	154490128	-0.935900939	0.003179786	decrease
chrX	77895236	77895636	-0.871055507	0.003181047	decrease
chr17	21099048	21099448	-0.784931765	0.003186859	decrease
chr6	53064945	53065345	-0.966865368	0.003188308	decrease
chr6	82247592	82247992	-0.904084219	0.003190725	decrease
chr1	67054195	67054595	-0.896556151	0.003200151	decrease
chr19	11813888	11814288	-0.982696788	0.003205891	decrease
chr2	24076221	24076621	-0.839754726	0.003218038	decrease
chr2	27081226	27081626	-0.909287112	0.003222736	decrease
chr5	112921264	112921664	-0.958777696	0.003223012	decrease
chr1	88684290	88684690	-0.8244212	0.003226831	decrease
chr12	123584110	123584510	-0.941420339	0.003228182	decrease
chr12	85280443	85280843	-0.929588658	0.003231618	decrease
chr19	35977636	35978036	-0.998646408	0.003236032	decrease
chr8	30082686	30083086	-0.989402456	0.003239224	decrease
chr17	76737403	76737803	-0.720849509	0.003248438	decrease
chr19	18919328	18919728	-0.771987503	0.003251865	decrease
chr1	110963759	110964159	-0.775914385	0.003254294	decrease

chr15	40857746	40858146	-1.016569197	0.003257724	decrease
chr7	5427929	5428329	-0.783007564	0.003258317	decrease
chr2	61017405	61017805	-0.796817237	0.003264865	decrease
chr10	27504027	27504427	-0.901379948	0.003267071	decrease
chr16	2905504	2905904	-0.864389789	0.003268289	decrease
chr5	178153802	178154202	-0.764587127	0.003275436	decrease
chr5	83473653	83474053	-0.862290093	0.003278498	decrease
chr17	7012271	7012671	-0.943020809	0.003278676	decrease
chr6	44338408	44338808	-0.996249218	0.003286768	decrease
chr1	78004479	78004879	-0.837605792	0.003289091	decrease
chr3	8501454	8501854	-0.938517998	0.003299503	decrease
chrX	130268610	130269010	-0.901306591	0.003301183	decrease
chr4	1712646	1713046	-0.870389176	0.003306273	decrease
chrX	131830636	131831036	-0.765100354	0.003309313	decrease
chr14	65411878	65412278	-0.841667315	0.003316627	decrease
chr2	55050373	55050773	-0.743226663	0.003320325	decrease
chr3	32570860	32571260	-0.913499662	0.003326761	decrease
chr5	150129802	150130202	-0.935711351	0.003334554	decrease
chr5	81184146	81184546	-0.965647439	0.003337774	decrease
chr10	103368603	103369003	-0.88145005	0.003340939	decrease
chr12	104986089	104986489	-1.026557216	0.00334308	decrease
chr1	50969761	50970161	-0.890271656	0.003352627	decrease
chr13	78603565	78603965	-0.917846498	0.003357097	decrease
chr2	74440518	74440918	-0.810749702	0.003367685	decrease
chr4	165378712	165379112	-1.034191957	0.003367893	decrease
chr6	10694542	10694942	-0.739321541	0.003373288	decrease
chr15	76905149	76905549	-0.936673315	0.003382455	decrease
chr4	474032	474432	-0.722460541	0.003383085	decrease
chr12	49741364	49741764	-0.664999874	0.003395614	decrease
chr2	113889657	113890057	-0.751123932	0.00339597	decrease
chr16	46621199	46621599	-0.957382531	0.003402918	decrease
chr20	31607101	31607501	-0.832092643	0.003409256	decrease
chr6	119349413	119349813	-0.979142192	0.003418753	decrease
chr12	98503468	98503868	-0.825010632	0.003420929	decrease
chr12	6689130	6689530	-0.94141198	0.003421834	decrease
chr1	161389901	161390301	-0.842571678	0.003432581	decrease
chr1	153963559	153963959	-0.829435005	0.003434961	decrease
chr9	35116353	35116753	-0.725837493	0.003440162	decrease
chr12	49568186	49568586	-0.885149811	0.0034402	decrease
chr2	28870104	28870504	-0.869182078	0.003449048	decrease
chr17	75113877	75114277	-0.943468811	0.00345028	decrease
chr14	20613371	20613771	-0.667423122	0.003452636	decrease
chr6	28212262	28212662	-0.965972103	0.003473404	decrease
chr12	4538405	4538805	-0.840465199	0.003490178	decrease
chr9	109012428	109012828	-0.903926265	0.003492467	decrease
chr15	66497748	66498148	-0.745884125	0.003493478	decrease
chr8	66667094	66667494	-0.73513312	0.003495066	decrease
chr15	89575158	89575558	-0.866410569	0.003510027	decrease
chr14	60656389	60656789	-0.973941914	0.003512845	decrease
chr15	65869348	65869748	-0.75776695	0.003513327	decrease
chr1	226939778	226940178	-0.702573349	0.003515708	decrease
chr3	136862049	136862449	-0.819646873	0.003521179	decrease
chr4	1167240	1167640	-0.821138752	0.003525788	decrease
chr1	235104364	235104764	-0.871160076	0.00353673	decrease
chr15	99565666	99566066	-1.010899674	0.003552245	decrease
chr20	17968824	17969224	-0.708350841	0.003555065	decrease
chr10	68721006	68721406	-0.879943708	0.003557715	decrease
chr4	90950575	90950975	-0.960379505	0.003562885	decrease
chr17	31437230	31437630	-1.012977504	0.003563345	decrease
chr9	4984673	4985073	-0.909629604	0.003563565	decrease
chr19	38899547	38899947	-0.709192447	0.003568023	decrease
chr19	45485207	45485607	-0.880528835	0.003568932	decrease
chr4	145935926	145936326	-0.992882958	0.003569472	decrease
chr19	52049017	52049417	-0.975892642	0.003571249	decrease
chr5	95646632	95647032	-0.844969287	0.003572577	decrease
chr3	168249934	168250334	-0.99386095	0.003574377	decrease
chr10	43408892	43409292	-0.898826692	0.003579068	decrease
chr19	14419136	14419536	-0.875928605	0.003586045	decrease
chr4	124712042	124712442	-0.951618486	0.00358691	decrease
chr2	9423444	9423844	-0.842240728	0.003591932	decrease
chr12	22543593	22543993	-0.981587956	0.003592258	decrease
chr1	44986331	44986731	-0.893405923	0.00359384	decrease
chr1	235128664	235129064	-0.681855143	0.003594619	decrease
chr8	27908348	27908748	-1.004473768	0.003597008	decrease
chr17	18856037	18856437	-0.916969001	0.003600888	decrease
chr1	93079043	93079443	-0.763067176	0.003615073	decrease

chr19	50384061	50384461	-0.836647711	0.003619489	decrease
chr2	73385955	73386355	-0.900778715	0.003620357	decrease
chr2	219177826	219178226	-0.909096179	0.003620794	decrease
chr18	32471117	32471517	-0.898676205	0.00363323	decrease
chr17	6651333	6651733	-0.889654212	0.003635273	decrease
chr5	146182524	146182924	-0.714537868	0.003646493	decrease
chr7	99919530	99919930	-1.003914191	0.003653768	decrease
chr17	18260476	18260876	-0.910708361	0.003657952	decrease
chr15	75307950	75308350	-0.879940298	0.00366606	decrease
chr1	16613555	16613955	-0.724395291	0.003670379	decrease
chr3	62318880	62319280	-0.978189434	0.00367225	decrease
chr16	23452910	23453310	-0.905635604	0.003674152	decrease
chr3	94179078	94179478	-0.880447747	0.003675333	decrease
chr19	35717258	35717658	-0.907353239	0.003683351	decrease
chr20	13638693	13639093	-0.894620281	0.003686216	decrease
chr3	33659164	33659564	-0.901445112	0.00368729	decrease
chr7	28410725	28411125	-0.820923697	0.003690435	decrease
chr1	228165213	228165613	-0.70875992	0.003693633	decrease
chr18	35290117	35290517	-0.634295755	0.003694491	decrease
chr1	241785256	241785656	-1.005399483	0.003695942	decrease
chr1	183235226	183235626	-0.758862782	0.003708067	decrease
chr5	62412491	62412891	-0.864497545	0.003717393	decrease
chr11	111879096	111879496	-0.846702936	0.003719693	decrease
chr3	168095794	168096194	-0.830986904	0.003722167	decrease
chr12	14803370	14803770	-0.871857036	0.00372232	decrease
chr6	1666985	1667385	-0.825764361	0.00372896	decrease
chr1	109640085	109640485	-0.896439349	0.00373586	decrease
chr11	13277281	13277681	-0.833726228	0.003737206	decrease
chr2	37925323	37925723	-0.967004467	0.00374891	decrease
chr2	189661191	189661591	-0.86231022	0.00375292	decrease
chr5	82278326	82278726	-0.757279159	0.003759631	decrease
chr9	128829691	128830091	-0.846210268	0.003764772	decrease
chr6	17706595	17706995	-0.8449106	0.003773456	decrease
chr1	112619719	112620119	-0.824340678	0.003790111	decrease
chr2	196926624	196927024	-0.832903347	0.003794406	decrease
chr3	130111670	130112070	-0.742707732	0.003798705	decrease
chr17	31901541	31901941	-0.806964777	0.003801425	decrease
chr19	43596235	43596635	-0.699385516	0.003801714	decrease
chr2	171687415	171687815	-0.94046685	0.003802454	decrease
chr6	117483281	117483681	-0.965197377	0.003807941	decrease
chr2	99180974	99181374	-0.921564721	0.003808658	decrease
chr3	88058934	88059334	-0.915884372	0.003817265	decrease
chr2	128090962	128091362	-0.853061519	0.003819312	decrease
chrX	155071356	155071756	-0.859157826	0.003819495	decrease
chr17	7717316	7717716	-0.672511996	0.003825309	decrease
chr5	127030580	127030980	-0.881259378	0.003826642	decrease
chr8	105319401	105319801	-0.986586997	0.003832151	decrease
chr14	59484152	59484552	-0.81644152	0.003834884	decrease
chr1	44799806	44800206	-0.735447251	0.003838752	decrease
chr13	74132765	74133165	-0.862458667	0.003842865	decrease
chr1	109249175	109249575	-0.950234101	0.003843622	decrease
chr3	25428122	25428522	-0.803595248	0.003859104	decrease
chr1	44238199	44238599	-0.891122433	0.003860443	decrease
chr17	28041340	28041740	-0.8652102	0.003865408	decrease
chr12	25386273	25386673	-0.96537015	0.0038675	decrease
chr7	27098245	27098645	-0.875597762	0.003881334	decrease
chr11	69675137	69675537	-0.90734328	0.003882887	decrease
chr16	2214888	2215288	-0.830312025	0.003900917	decrease
chr21	42107608	42108008	-0.931380307	0.003901666	decrease
chr19	40425865	40426265	-0.905957656	0.003901822	decrease
chr4	133151428	133151828	-0.809352056	0.003905688	decrease
chr6	27230198	27230598	-0.874746882	0.003924022	decrease
chr7	76047702	76048102	-0.910081239	0.003924954	decrease
chr6	158644730	158645130	-0.756840946	0.00392636	decrease
chr4	8410612	8411012	-0.906849625	0.003930197	decrease
chr1	93847441	93847841	-0.780497469	0.003930328	decrease
chr2	238098398	238098798	-0.824007179	0.003938153	decrease
chr1	156054643	156055043	-0.788167882	0.003948016	decrease
chr14	74302685	74303085	-0.761774311	0.003949422	decrease
chr19	46728850	46729250	-0.88828047	0.003949659	decrease
chr4	55546895	55547295	-0.921550782	0.00395055	decrease
chr7	73522305	73522705	-0.738066646	0.003951174	decrease
chr4	26860392	26860792	-0.958566164	0.003952144	decrease
chr17	45161504	45161904	-0.814184919	0.003959328	decrease
chr13	33542597	33542997	-0.932774509	0.00396673	decrease
chr14	73785784	73786184	-0.896454874	0.003978314	decrease

chr20	5119779	5120179	-0.702226502	0.003979921	decrease
chr19	17226155	17226555	-0.796227025	0.003982812	decrease
chr2	71227112	71227512	-0.810321637	0.003983096	decrease
chr17	78840749	78841149	-0.766760797	0.003988253	decrease
chr11	4094437	4094837	-0.882222098	0.003989911	decrease
chr1	211258625	211259025	-0.825185918	0.004000771	decrease
chr9	128504512	128504912	-0.839374049	0.00400573	decrease
chr2	23927179	23927579	-0.99361278	0.004019427	decrease
chr10	12195738	12196138	-0.868037171	0.004022694	decrease
chr14	55051336	55051736	-0.916510907	0.004026742	decrease
chr22	19144659	19145059	-0.848176879	0.004030568	decrease
chr1	197201353	197201753	-0.722024301	0.004036231	decrease
chr8	81721157	81721557	-0.840125244	0.004046257	decrease
chr3	15427146	15427546	-0.948750384	0.004047001	decrease
chr12	57462464	57462864	-0.787741379	0.004048931	decrease
chrX	72238829	72239229	-0.924176878	0.004051014	decrease
chr19	18571324	18571724	-0.953918192	0.004053143	decrease
chr4	153257247	153257647	-0.979971878	0.004053735	decrease
chr9	124868628	124869028	-0.905550195	0.00406093	decrease
chr16	50025092	50025492	-0.942957475	0.004068777	decrease
chr4	13483382	13483782	-0.941957678	0.004078348	decrease
chr19	50025579	50025979	-0.800563049	0.004079002	decrease
chr2	45009615	45010015	-0.833425879	0.004080676	decrease
chr2	27071375	27071775	-1.000368123	0.0040901	decrease
chr10	16436766	16437166	-0.797322912	0.004103745	decrease
chr1	235367303	235367703	-0.698304263	0.004105138	decrease
chr22	38681816	38682216	-0.823335748	0.004106119	decrease
chr1	217630910	217631310	-0.878106882	0.004108657	decrease
chr7	108569445	108569845	-0.786421182	0.004116438	decrease
chr11	95231272	95231672	-0.78755999	0.004117496	decrease
chr1	244864458	244864858	-0.703524106	0.004119314	decrease
chr1	44721433	44721833	-0.720364651	0.004122354	decrease
chr19	41363864	41364264	-0.746994643	0.004127823	decrease
chr19	11528814	11529214	-0.830295198	0.004128138	decrease
chr15	41116383	41116783	-0.999436759	0.004128202	decrease
chr1	30929328	30929728	-0.812364018	0.004134498	decrease
chrX	115226976	115227376	-0.991049433	0.004136434	decrease
chr9	128656743	128657143	-0.962383239	0.004138984	decrease
chr3	48240961	48241361	-0.783907474	0.00414089	decrease
chr17	44345035	44345435	-0.847467824	0.004141676	decrease
chrX	150983019	150983419	-0.903679783	0.004143474	decrease
chr3	132417400	132417800	-0.860525702	0.004152519	decrease
chr12	24562006	24562406	-0.93493506	0.004153234	decrease
chr2	188292736	188293136	-0.81646635	0.00415932	decrease
chr21	28992700	28993100	-0.72580918	0.004160842	decrease
chr9	104093764	104094164	-0.66371821	0.004161379	decrease
chr22	33921293	33921693	-0.790301147	0.004165567	decrease
chr22	29388604	29389004	-0.933667847	0.004165774	decrease
chr2	65432628	65433028	-0.886118661	0.00418378	decrease
chr1	209827729	209828129	-0.805614762	0.004188848	decrease
chr1	229410964	229411364	-0.951605585	0.004193465	decrease
chr3	122383992	122384392	-0.723846623	0.00419449	decrease
chr10	110858162	110858562	-0.982098907	0.00419588	decrease
chr19	52028396	52028796	-0.898376079	0.004199	decrease
chr1	160070306	160070706	-0.925897829	0.004203893	decrease
chr16	19067474	19067874	-0.782049734	0.004213818	decrease
chr9	85940810	85941210	-0.909596242	0.004223944	decrease
chr13	77025897	77026297	-0.900211101	0.004228223	decrease
chr7	75914913	75915313	-0.985078347	0.004230383	decrease
chr1	68881558	68881958	-0.997620133	0.004232919	decrease
chr12	123149109	123149509	-0.937730428	0.004233555	decrease
chr1	51878628	51879028	-0.799414726	0.004233583	decrease
chr10	3783005	3783405	-0.955306126	0.004239942	decrease
chr14	21308606	21309006	-0.825126479	0.004251198	decrease
chr10	132223169	132223569	-0.917367403	0.004252867	decrease
chr16	53054721	53055121	-0.930407485	0.00425334	decrease
chr9	90433469	90433869	-0.94928271	0.004254313	decrease
chr15	74461170	74461570	-0.749351802	0.004256015	decrease
chr2	86623559	86623959	-1.008129658	0.004258346	decrease
chr2	201642651	201643051	-0.949326825	0.004271633	decrease
chr7	140923975	140924375	-0.890254818	0.004273808	decrease
chrX	150898832	150899232	-0.9335852	0.00427913	decrease
chr1	153671071	153671471	-0.710263676	0.004283475	decrease
chr14	73026994	73027394	-0.869218199	0.004284515	decrease
chrX	43654546	43654946	-0.859739239	0.00428636	decrease
chr1	28235766	28236166	-0.836297319	0.004289457	decrease

chrX	41084824	41085224	-0.863740021	0.004300565	decrease
chr17	49192605	49193005	-0.687401187	0.004303296	decrease
chr11	65315238	65315638	-0.891690642	0.004321104	decrease
chr19	17294053	17294453	-0.90027223	0.004324437	decrease
chr14	99602636	99603036	-0.995359045	0.004328918	decrease
chr20	23357951	23358351	-0.757744745	0.004336896	decrease
chr10	68331687	68332087	-0.638218876	0.004337061	decrease
chr12	56116234	56116634	-0.780284344	0.004339082	decrease
chr16	28824046	28824446	-0.796872	0.004340668	decrease
chr2	70190754	70191154	-0.822536202	0.004347869	decrease
chr2	241316092	241316492	-0.838537492	0.00435549	decrease
chr1	39026146	39026546	-0.963150996	0.004360482	decrease
chr16	81076857	81077257	-0.806992644	0.004363253	decrease
chr6	112087195	112087595	-0.795245388	0.004374162	decrease
chr2	64652354	64652754	-0.964182053	0.004378222	decrease
chr2	112055848	112056248	-0.950564962	0.004382001	decrease
chr10	100743723	100744123	-0.939549336	0.004382187	decrease
chr16	2009788	2010188	-0.851624296	0.004384898	decrease
chr2	202237616	202238016	-0.970765045	0.004386063	decrease
chr10	28678810	28679210	-0.970186463	0.004387068	decrease
chr17	30831855	30832255	-0.965822154	0.004398439	decrease
chr22	26672684	26673084	-0.916671705	0.004404827	decrease
chr1	147172049	147172449	-0.871851078	0.004404935	decrease
chr2	108719263	108719663	-0.836371331	0.004405961	decrease
chr19	3500644	3501044	-0.835644039	0.004407652	decrease
chr7	129224511	129224911	-0.85604277	0.004411419	decrease
chr7	143380567	143380967	-0.778410316	0.004416939	decrease
chr5	115262630	115263030	-0.915578091	0.004417581	decrease
chr1	61056933	61057333	-0.901919394	0.004418874	decrease
chr3	160565666	160566066	-0.913673038	0.004434205	decrease
chr9	36190580	36190980	-0.873299923	0.004437576	decrease
chr1	213015398	213015798	-0.714369036	0.004441115	decrease
chr10	92574078	92574478	-0.811051044	0.004448313	decrease
chr1	25997926	25998326	-0.952984377	0.004455761	decrease
chrX	149505298	149505698	-0.966279433	0.004461215	decrease
chrX	71283277	71283677	-0.869319867	0.004462451	decrease
chr5	10353291	10353691	-0.956029258	0.004463266	decrease
chr8	85245532	85245932	-0.936551565	0.004468675	decrease
chr2	74458075	74458475	-0.700991543	0.004476331	decrease
chr19	11374583	11374983	-0.651777807	0.004511205	decrease
chr19	17260804	17261204	-0.903298561	0.00451389	decrease
chr2	200864086	200864486	-0.91155329	0.004515338	decrease
chr6	36423114	36423514	-0.954711365	0.00451805	decrease
chr19	38224326	38224726	-0.888155694	0.004518643	decrease
chr20	62302527	62302927	-0.954153613	0.004523017	decrease
chr17	80544790	80545190	-0.825140429	0.004532993	decrease
chr5	151686844	151687244	-0.859594158	0.004546921	decrease
chr6	114343384	114343784	-0.917891189	0.004552565	decrease
chr9	34646615	34647015	-0.766850735	0.004553478	decrease
chr18	33577682	33578082	-0.797266263	0.004553924	decrease
chr7	27136118	27136518	-0.91391269	0.004557442	decrease
chr8	120266156	120266556	-0.958256268	0.004559044	decrease
chr3	52154433	52154833	-0.781663226	0.004562414	decrease
chr1	28369576	28369976	-0.761430831	0.004577227	decrease
chr9	26892354	26892754	-0.89233802	0.00457826	decrease
chr8	52587085	52587485	-0.965443612	0.004587801	decrease
chr16	67719276	67719676	-0.838461841	0.004592791	decrease
chr12	111405498	111405898	-0.970811142	0.004601962	decrease
chr6	36547189	36547589	-0.882254944	0.004602989	decrease
chr17	8119604	8120004	-0.651898856	0.004611566	decrease
chr8	65633941	65634341	-0.781149027	0.00461186	decrease
chr11	59669190	59669590	-0.899496415	0.00461222	decrease
chr17	38853868	38854268	-0.938976482	0.004627686	decrease
chr1	182838921	182839321	-0.903103271	0.004631631	decrease
chr11	13668316	13668716	-0.914082214	0.004632804	decrease
chr9	79663917	79664317	-0.718263153	0.004634549	decrease
chr3	113151580	113151980	-0.944878155	0.004644014	decrease
chr4	153249230	153249630	-0.859713765	0.004655683	decrease
chr6	56843975	56844375	-0.91462611	0.004656106	decrease
chr19	10653886	10654286	-0.779050807	0.004658523	decrease
chr3	50569062	50569462	-0.710226523	0.004661537	decrease
chr19	45641114	45641514	-0.765343132	0.004664178	decrease
chr3	75435235	75435635	-0.68501978	0.004687582	decrease
chr1	185892661	185893061	-0.910062434	0.004687764	decrease
chr4	147683867	147684267	-0.838942575	0.004688934	decrease
chr17	81860628	81861028	-0.686894741	0.004698516	decrease

chr14	60659133	60659533	-0.945167765	0.004699884	decrease
chr1	33045180	33045580	-0.968498549	0.0047119	decrease
chr2	74653923	74654323	-0.826607057	0.004718764	decrease
chr9	127931138	127931538	-0.865373647	0.004729358	decrease
chr4	87391095	87391495	-0.85849896	0.004730584	decrease
chr20	56392240	56392640	-0.802303135	0.004735726	decrease
chr17	79779059	79779459	-0.911591089	0.004736295	decrease
chr14	54509703	54510103	-0.824384648	0.004740498	decrease
chr3	156554956	156555356	-0.882019204	0.004745118	decrease
chr2	218568340	218568740	-0.678060561	0.004747914	decrease
chr19	18152615	18153015	-0.677620586	0.004756608	decrease
chr16	58733982	58734382	-0.856846099	0.004758624	decrease
chr11	85663443	85663843	-0.96854807	0.004759875	decrease
chr1	179954389	179954789	-0.699913826	0.004766989	decrease
chr16	1782607	1783007	-0.824556264	0.004767362	decrease
chr22	38656416	38656816	-0.794301926	0.004770562	decrease
chr8	86508560	86508960	-0.869657895	0.004775089	decrease
chr18	57437695	57438095	-0.90719157	0.004779466	decrease
chr4	92305542	92305942	-0.794860194	0.004783118	decrease
chr7	94004006	94004406	-0.956171265	0.004784477	decrease
chr12	57088189	57088589	-0.794208048	0.004800651	decrease
chr1	91404158	91404558	-0.958106075	0.004821841	decrease
chr1	155745448	155745848	-0.840919841	0.004839756	decrease
chr8	95269506	95269906	-0.779128507	0.004844792	decrease
chr1	84479314	84479714	-0.903258663	0.004856254	decrease
chr7	47991858	47992258	-0.986876856	0.004856788	decrease
chr19	40570617	40571017	-0.796528189	0.004860338	decrease
chr12	6970420	6970820	-0.825112385	0.00487248	decrease
chr3	66997938	66998338	-0.875534033	0.004874462	decrease
chr1	151059439	151059839	-0.745252942	0.004880464	decrease
chr5	111512347	111512747	-0.765781194	0.004882645	decrease
chr8	89758332	89758732	-0.892825778	0.004899466	decrease
chr5	79612024	79612424	-0.926882291	0.004909793	decrease
chr2	216498576	216498976	-0.820168077	0.00491105	decrease
chr17	62000495	62000895	-0.979945618	0.004914429	decrease
chr5	14145322	14145722	-0.895845619	0.004922747	decrease
chr11	47767299	47767699	-0.71771108	0.004924112	decrease
chr10	119166055	119166455	-0.913677415	0.004936627	decrease
chrX	16771723	16772123	-0.868201594	0.004938389	decrease
chr10	73246942	73247342	-0.740439337	0.004941812	decrease
chr1	144412582	144412982	-0.706446799	0.00495105	decrease
chr7	32495397	32495797	-0.888872564	0.004958032	decrease
chr7	25862191	25862591	-0.69004467	0.004962746	decrease
chr15	79923610	79924010	-0.830200694	0.004963289	decrease
chr3	4466999	4467399	-0.924310929	0.004963571	decrease
chr4	8117284	8117684	-0.850356701	0.004984127	decrease
chr20	20712624	20713024	-0.921813004	0.004986187	decrease
chr11	126268902	126269302	-0.727516721	0.004991621	decrease
chr10	71851013	71851413	-0.816405344	0.004996328	decrease
chr1	92960456	92960856	-0.802085044	0.004997457	decrease
chr8	76581191	76581591	-0.984870357	0.005007132	decrease
chr20	38472481	38472881	-0.940039063	0.005012495	decrease
chr7	123747613	123748013	-0.896206072	0.005012961	decrease
chr9	136950148	136950548	-0.88400335	0.005013253	decrease
chr14	104752576	104752976	-0.650753193	0.00501904	decrease
chr7	129054772	129055172	-0.783941563	0.005019411	decrease
chr8	94822867	94823267	-0.924576539	0.005030546	decrease
chr16	4767130	4767530	-0.913467935	0.005032003	decrease
chr4	122922753	122923153	-0.855317797	0.005032488	decrease
chr5	134371071	134371471	-0.902977711	0.005040953	decrease
chr20	25623777	25624177	-0.753683382	0.005043349	decrease
chr19	12722758	12723158	-0.869567207	0.005043367	decrease
chrX	149938299	149938699	-0.755095658	0.005049489	decrease
chr3	48088951	48089351	-0.873701654	0.005055236	decrease
chr2	159286848	159287248	-0.875388805	0.005068883	decrease
chr4	75724420	75724820	-0.729241645	0.005071851	decrease
chr17	60599893	60600293	-0.841627871	0.005077367	decrease
chr10	110646465	110646865	-0.940727606	0.005100768	decrease
chr10	125823221	125823621	-0.743545931	0.00510646	decrease
chr11	62573811	62574211	-0.711568904	0.00511334	decrease
chr6	85643034	85643434	-0.761888751	0.005128703	decrease
chr3	165206876	165207276	-0.965280501	0.005129683	decrease
chr1	198156481	198156881	-0.919824462	0.005130659	decrease
chr14	100221387	100221787	-0.95367237	0.005142894	decrease
chr19	52734790	52735190	-0.959849293	0.005144896	decrease
chr2	197515929	197516329	-0.865584914	0.005146113	decrease

chr19	36850414	36850814	-0.797808634	0.005164059	decrease
chr16	29816035	29816435	-0.613427502	0.005166195	decrease
chr1	168135872	168136272	-0.947364295	0.00516644	decrease
chr19	48835811	48836211	-0.943501253	0.005172342	decrease
chr4	122732539	122732939	-0.783566658	0.005176094	decrease
chr6	110981933	110982333	-0.815655758	0.005180214	decrease
chr1	154183070	154183470	-0.693232204	0.00518679	decrease
chr2	177212223	177212623	-0.906278839	0.005189532	decrease
chr1	8707118	8707518	-0.959076754	0.005190141	decrease
chr13	49792641	49793041	-0.91079429	0.005194981	decrease
chr12	15881986	15882386	-0.765045212	0.005196186	decrease
chr1	90851307	90851707	-0.896395837	0.005204937	decrease
chr3	50292441	50292841	-0.772560612	0.005207896	decrease
chr1	159925359	159925759	-0.688782363	0.005208356	decrease
chr11	124954007	124954407	-0.855238028	0.005209661	decrease
chr1	153647313	153647713	-0.858926824	0.005213594	decrease
chr3	158644453	158644853	-0.868057094	0.005218516	decrease
chr16	23596347	23596747	-0.903871898	0.005221312	decrease
chr10	26860825	26861225	-0.894298524	0.005228189	decrease
chr2	201780917	201781317	-0.777686631	0.005256003	decrease
chr6	44667924	44668324	-0.966328617	0.005256474	decrease
chr19	58386861	58387261	-0.785889916	0.005260209	decrease
chr19	55476211	55476611	-0.812487606	0.005261997	decrease
chr1	85200266	85200666	-0.936602727	0.005264172	decrease
chr19	49929689	49930089	-0.711400855	0.005273663	decrease
chr16	29973471	29973871	-0.742980878	0.005274136	decrease
chr6	27132642	27133042	-0.669227976	0.00528232	decrease
chr3	182739664	182740064	-0.970844825	0.005285393	decrease
chr5	181169785	181170185	-0.827984629	0.005295933	decrease
chr15	43791935	43792335	-0.886607885	0.005296834	decrease
chr16	31382412	31382812	-0.981397715	0.005299115	decrease
chr2	47906639	47907039	-0.965697899	0.005305068	decrease
chr2	131493805	131494205	-0.964171847	0.005313852	decrease
chr19	8050496	8050896	-0.853964311	0.005321606	decrease
chr13	94595975	94596375	-0.678447907	0.005322761	decrease
chr8	19817092	19817492	-0.859976214	0.005332294	decrease
chr9	136790777	136791177	-0.787141304	0.005335066	decrease
chr4	110196032	110196432	-0.935517636	0.005335942	decrease
chr18	28175861	28176261	-0.845388578	0.005337762	decrease
chr8	144082245	144082645	-0.879712315	0.005337859	decrease
chr19	5686763	5687163	-0.960131819	0.005341911	decrease
chr8	144901608	144902008	-0.716844517	0.005342295	decrease
chr4	6664649	6665049	-0.952242005	0.005342503	decrease
chr5	154191897	154192297	-0.96137106	0.005347776	decrease
chr6	161274041	161274441	-0.768192504	0.005351279	decrease
chr11	85647768	85648168	-0.84680774	0.005353731	decrease
chr19	19320419	19320819	-0.759932171	0.005362617	decrease
chr1	167721505	167721905	-0.91340135	0.005370122	decrease
chr13	41311126	41311526	-0.922968271	0.005380602	decrease
chr22	37608444	37608844	-0.780773911	0.005380872	decrease
chr1	162062513	162062913	-0.955216466	0.005384195	decrease
chr8	124372744	124373144	-0.860033173	0.005394056	decrease
chr1	75724575	75724975	-0.860813921	0.005396824	decrease
chr15	42575170	42575570	-0.923438953	0.005400619	decrease
chr1	53220363	53220763	-0.793358196	0.005405652	decrease
chr2	191013961	191014361	-0.912450867	0.005411435	decrease
chr17	40100316	40100716	-0.82775045	0.005424635	decrease
chr4	77818000	77818400	-0.880457998	0.005427241	decrease
chr4	99563665	99564065	-0.796797822	0.005431873	decrease
chr1	120176249	120176649	-0.876882216	0.005432405	decrease
chr14	75176552	75176952	-0.764937597	0.005432864	decrease
chr3	32781261	32781661	-0.965535483	0.005433624	decrease
chr3	49021699	49022099	-0.727974617	0.005436856	decrease
chr18	46946640	46947040	-0.960463299	0.005436954	decrease
chr17	58658761	58659161	-0.62924172	0.005442249	decrease
chr15	51971556	51971956	-0.910717642	0.005458102	decrease
chr22	37658256	37658656	-0.754933693	0.005475243	decrease
chr12	7018515	7018915	-0.720262383	0.005489713	decrease
chr12	56300127	56300527	-0.66635722	0.005493077	decrease
chr7	151080134	151080534	-0.758805229	0.00549674	decrease
chr3	184185935	184186335	-0.90319957	0.005497277	decrease
chr1	229626210	229626610	-0.808086899	0.005499666	decrease
chr5	51383282	51383682	-0.935222	0.005505825	decrease
chr7	87220403	87220803	-0.938516184	0.005509807	decrease
chr6	144286275	144286675	-0.82618717	0.00551559	decrease
chr6	41921008	41921408	-0.880101063	0.005525805	decrease

chr14	96391380	96391780	-0.955141461	0.005546841	decrease
chr4	127965081	127965481	-0.693352651	0.005559141	decrease
chr7	6104352	6104752	-0.851178119	0.005560831	decrease
chr12	32679014	32679414	-0.831778641	0.005574982	decrease
chr18	51197949	51198349	-0.833021761	0.005587512	decrease
chr11	2089346	2089746	-0.882210062	0.005598513	decrease
chr6	82260162	82260562	-0.952846171	0.005600464	decrease
chr10	72354615	72355015	-0.824208001	0.005604168	decrease
chr19	57389660	57390060	-0.819888443	0.005614371	decrease
chr11	22193147	22193547	-0.933727774	0.005615014	decrease
chr10	131900287	131900687	-0.768925093	0.005620373	decrease
chr16	78099365	78099765	-0.649761563	0.005623042	decrease
chr6	18155723	18156123	-0.936058906	0.00562582	decrease
chr12	68209427	68209827	-0.788555341	0.00563926	decrease
chr5	109410079	109410479	-0.66340094	0.005643443	decrease
chr6	34415918	34416318	-0.892905286	0.005649915	decrease
chr2	64644411	64644811	-0.812546872	0.00565476	decrease
chr6	10886751	10887151	-0.88864533	0.005655301	decrease
chr2	180540650	180541050	-0.961674431	0.005656771	decrease
chr1	77219047	77219447	-0.857398229	0.005657146	decrease
chr12	110468305	110468705	-0.802111902	0.005658524	decrease
chr3	64023084	64023484	-0.954026091	0.005660169	decrease
chr19	19034050	19034450	-0.854877277	0.005661901	decrease
chr7	27160185	27160585	-0.87581777	0.005670276	decrease
chr1	36149392	36149792	-0.743437303	0.005675904	decrease
chr8	73972152	73972552	-0.724973341	0.005686712	decrease
chr2	97996040	97996440	-0.880475775	0.00568745	decrease
chr1	153922975	153923375	-0.745089781	0.005689336	decrease
chr12	112418429	112418829	-0.962432456	0.005691229	decrease
chr1	161153968	161154368	-0.919380548	0.005692314	decrease
chr6	144423117	144423517	-0.974633172	0.005696424	decrease
chr4	112706424	112706824	-0.955526779	0.005701998	decrease
chr19	14689727	14690127	-0.914847622	0.005704763	decrease
chr14	24188431	24188831	-0.802957187	0.005719002	decrease
chr1	50978573	50978973	-0.948668849	0.005726024	decrease
chr5	32313004	32313404	-0.868695857	0.005732872	decrease
chr8	33484671	33485071	-0.914368562	0.005736523	decrease
chr13	74133676	74134076	-0.817878477	0.005739773	decrease
chr5	43514825	43515225	-0.891087042	0.005742866	decrease
chr12	48957083	48957483	-0.760767707	0.00574364	decrease
chr1	35557339	35557739	-0.789992135	0.005747392	decrease
chr11	112074003	112074403	-0.858015244	0.005770815	decrease
chr16	4538558	4538958	-0.919564846	0.005778437	decrease
chr14	21511200	21511600	-0.766881705	0.00578138	decrease
chr3	4979968	4980368	-0.84269361	0.005781985	decrease
chr15	84142930	84143330	-0.864542255	0.005793572	decrease
chr22	17159319	17159719	-0.901305911	0.005794885	decrease
chr14	33950738	33951138	-0.944550082	0.00579795	decrease
chr14	100246547	100246947	-0.934701868	0.005800594	decrease
chr5	5422289	5422689	-0.750790345	0.005805689	decrease
chr20	45363021	45363421	-0.80605758	0.005809102	decrease
chrX	48003937	48004337	-0.89375126	0.005813699	decrease
chr1	167936226	167936626	-0.716163364	0.005823874	decrease
chr19	55166710	55167110	-0.665123411	0.005826395	decrease
chr17	63549955	63550355	-0.730554075	0.005839734	decrease
chr3	175270448	175270848	-0.896032364	0.00584196	decrease
chr4	155758901	155759301	-0.84828992	0.005846846	decrease
chr8	66925600	66926000	-0.894477988	0.005848097	decrease
chr1	148334618	148335018	-0.684890364	0.005849027	decrease
chr2	160269919	160270319	-0.858920045	0.005849252	decrease
chr12	123436452	123436852	-0.825269495	0.00585013	decrease
chrX	154805168	154805568	-0.906548054	0.005854948	decrease
chr6	42563836	42564236	-0.773578072	0.00585517	decrease
chr16	87388326	87388726	-0.756191316	0.005857894	decrease
chr14	24195503	24195903	-0.804393738	0.005870907	decrease
chr3	51499292	51499692	-0.824407548	0.005874183	decrease
chr22	41832685	41833085	-0.762706115	0.005875755	decrease
chr3	141738158	141738558	-0.924264562	0.005891728	decrease
chr2	193170992	193171392	-0.937839215	0.005897203	decrease
chr4	127622820	127623220	-0.911187611	0.005902956	decrease
chr12	107093529	107093929	-0.777333063	0.005907237	decrease
chrX	130060395	130060795	-0.80366195	0.005910519	decrease
chr2	88627708	88628108	-0.689628783	0.005916868	decrease
chr10	42638206	42638606	-0.715069087	0.005917663	decrease
chr15	49169917	49170317	-0.941914682	0.005918083	decrease
chr12	56315972	56316372	-0.660690921	0.005922685	decrease

chr8	96518739	96519139	-0.897379564	0.005924087	decrease
chr12	45990285	45990685	-0.703158063	0.005925414	decrease
chr19	13905859	13906259	-0.948695172	0.005931379	decrease
chr3	64239153	64239553	-0.932530863	0.00593246	decrease
chr13	36345507	36345907	-0.913492651	0.005932982	decrease
chr1	224434790	224435190	-0.748395379	0.005943278	decrease
chr20	49046181	49046581	-0.765469289	0.005948551	decrease
chr1	224615801	224616201	-0.862920943	0.005948877	decrease
chr1	212286419	212286819	-0.908155431	0.005953209	decrease
chr16	71426474	71426874	-0.523405226	0.005958021	decrease
chr22	21938358	21938758	-0.804938687	0.005965714	decrease
chr1	28577172	28577572	-0.85979367	0.005968114	decrease
chr13	80342640	80343040	-0.816592777	0.005978105	decrease
chr5	113294955	113295355	-0.869811501	0.005978797	decrease
chr5	107673228	107673628	-0.895157931	0.00598364	decrease
chr8	26382832	26383232	-0.793683931	0.005988246	decrease
chr8	116766306	116766706	-0.791952661	0.005992981	decrease
chr15	45200365	45200765	-0.875065186	0.005996679	decrease
chrX	154547217	154547617	-0.751933136	0.006000009	decrease
chr13	30617305	30617705	-0.764257257	0.006004484	decrease
chr17	47649438	47649838	-0.650716819	0.0060241	decrease
chr5	80488178	80488578	-0.893950189	0.006025708	decrease
chr16	72093560	72093960	-0.698820835	0.00602818	decrease
chr4	26320249	26320649	-0.694054792	0.006029396	decrease
chr14	64914214	64914614	-0.86576505	0.006031699	decrease
chr5	37248977	37249377	-0.965161294	0.006037341	decrease
chr17	75273190	75273590	-0.845695564	0.006064573	decrease
chrX	74420100	74420500	-0.898773118	0.006065116	decrease
chr19	48332844	48333244	-0.777499846	0.006065547	decrease
chr22	37893631	37894031	-0.925971147	0.006069267	decrease
chr1	182391524	182391924	-0.874270578	0.006079223	decrease
chr2	85595396	85595796	-0.773221632	0.006082169	decrease
chr17	64506777	64507177	-0.679446087	0.006083751	decrease
chr8	47659608	47660008	-0.962552552	0.006096247	decrease
chr5	40835230	40835630	-0.768798889	0.006103063	decrease
chrX	20265277	20265677	-0.948483657	0.006115862	decrease
chr14	59188416	59188816	-0.750277313	0.006121309	decrease
chr19	10119778	10120178	-0.698300949	0.00612291	decrease
chr4	98995363	98995763	-0.855959646	0.006123287	decrease
chr1	35930453	35930853	-0.906037043	0.006124474	decrease
chr11	64810504	64810904	-0.741148771	0.006128778	decrease
chr17	76671536	76671936	-0.679477457	0.006133082	decrease
chr11	106076998	106077398	-0.680033949	0.006152257	decrease
chr4	41990301	41990701	-0.879699416	0.006155103	decrease
chr3	121834940	121835340	-0.840574383	0.006158393	decrease
chr3	10154722	10155122	-0.95032035	0.006159943	decrease
chr6	149648656	149649056	-0.722569994	0.006161703	decrease
chr6	125050926	125051326	-0.935065776	0.006165817	decrease
chr17	49414794	49415194	-0.727858656	0.006169314	decrease
chr11	34438527	34438927	-0.763653627	0.006183019	decrease
chr18	31943303	31943703	-0.79099568	0.006186618	decrease
chr1	32013406	32013806	-0.836193232	0.006193049	decrease
chr7	148987423	148987823	-0.69697135	0.006196678	decrease
chr7	23532010	23532410	-0.783992411	0.006199991	decrease
chr8	69466573	69466973	-0.85881434	0.006206123	decrease
chr8	93700020	93700420	-0.937862621	0.006212919	decrease
chr9	133346892	133347292	-0.800810009	0.006216901	decrease
chr4	6673554	6673954	-0.828237953	0.00623536	decrease
chr17	81515348	81515748	-0.778548125	0.006244861	decrease
chr1	175023351	175023751	-0.715826393	0.006264708	decrease
chr1	54053123	54053523	-0.73303314	0.006265134	decrease
chr13	50911216	50911616	-0.893692554	0.006268374	decrease
chr18	12991143	12991543	-0.783184288	0.006269984	decrease
chr10	73730229	73730629	-0.807072454	0.006270066	decrease
chr20	36156498	36156898	-0.917604705	0.006276447	decrease
chr4	127881043	127881443	-0.684171567	0.006278437	decrease
chr1	148402377	148402777	-0.799104023	0.006280587	decrease
chr7	64313881	64314281	-0.9256086	0.006289578	decrease
chr7	83162728	83163128	-0.863361035	0.006291188	decrease
chr14	54396692	54397092	-0.80243166	0.006292029	decrease
chr3	97764353	97764753	-0.791846909	0.006303556	decrease
chr6	93419604	93420004	-0.918875459	0.006340537	decrease
chr7	112789879	112790279	-0.834351901	0.006350856	decrease
chr5	88674846	88675246	-0.763802255	0.006355554	decrease
chr5	177496465	177496865	-0.642243792	0.006363234	decrease
chr8	69834611	69835011	-0.876492667	0.006364686	decrease

chr1	225772189	225772589	-0.786134586	0.006371586	decrease
chr15	48877989	48878389	-0.918254682	0.006386594	decrease
chr2	199459354	199459754	-0.920109988	0.006389324	decrease
chr13	73058715	73059115	-0.711720838	0.006390017	decrease
chr4	73257331	73257731	-0.944226067	0.006420188	decrease
chr3	10141699	10142099	-0.790351164	0.006422302	decrease
chrX	75274064	75274464	-0.824522092	0.006429761	decrease
chr19	13825691	13826091	-0.873740428	0.006431643	decrease
chr10	87818125	87818525	-0.776716257	0.006438938	decrease
chr2	172084455	172084855	-0.80881341	0.006441314	decrease
chr20	43457460	43457860	-0.813075193	0.006445078	decrease
chr21	32306196	32306596	-0.886534181	0.006446682	decrease
chr17	61511436	61511836	-0.92125736	0.006447059	decrease
chr6	169701893	169702293	-0.786037749	0.006450138	decrease
chr16	67521190	67521590	-0.833135581	0.006454877	decrease
chrX	47561073	47561473	-0.786858963	0.006456847	decrease
chr19	58326981	58327381	-0.681017301	0.00645724	decrease
chr7	26194412	26194812	-0.923172152	0.006462727	decrease
chr1	160031832	160032232	-0.720472392	0.006477185	decrease
chr6	87702313	87702713	-0.807867351	0.006488832	decrease
chr7	155412550	155412950	-0.786497896	0.006492239	decrease
chr2	71130138	71130538	-0.800270072	0.006499591	decrease
chr17	48501235	48501635	-0.920907466	0.006500147	decrease
chr5	172983630	172984030	-0.732957587	0.006528551	decrease
chr3	52705973	52706373	-0.798099522	0.006528656	decrease
chr5	163459905	163460305	-0.801964656	0.006529831	decrease
chr22	23290181	23290581	-0.897522693	0.006532817	decrease
chr17	77772460	77772860	-0.907598194	0.006546511	decrease
chr9	132241130	132241530	-0.862468305	0.006553044	decrease
chr11	121292544	121292944	-0.873555425	0.006556467	decrease
chr7	156893283	156893683	-0.675880818	0.006572185	decrease
chrX	102951378	102951778	-0.774146791	0.006572759	decrease
chr14	60165099	60165499	-0.931058445	0.006574054	decrease
chr7	5562105	5562505	-0.855519497	0.006576243	decrease
chr11	31810975	31811375	-0.851557644	0.006580547	decrease
chr9	72365359	72365759	-0.826581436	0.006585932	decrease
chr1	156456856	156457256	-0.659310851	0.006588282	decrease
chr11	75814877	75815277	-0.735620873	0.006599921	decrease
chr15	44427100	44427500	-0.790452822	0.006603649	decrease
chr12	75390994	75391394	-0.857565554	0.006605393	decrease
chr22	31490259	31490659	-0.848934661	0.006606958	decrease
chr11	90240560	90240960	-0.815943681	0.006611422	decrease
chr2	231961486	231961886	-0.804417724	0.006612094	decrease
chr17	74211739	74212139	-0.730632012	0.006612681	decrease
chr4	67545354	67545754	-0.837726824	0.006614664	decrease
chr6	84052522	84052922	-0.929894491	0.006620236	decrease
chr6	165674280	165674680	-0.911159011	0.00664278	decrease
chr6	87472841	87473241	-0.78162037	0.006647728	decrease
chr14	31457134	31457534	-0.839259316	0.006647862	decrease
chr2	47402951	47403351	-0.835199892	0.006652205	decrease
chr4	86594928	86595328	-0.852287895	0.006653384	decrease
chr1	46340469	46340869	-0.687973743	0.006654288	decrease
chr11	64710546	64710946	-0.930243368	0.00666525	decrease
chr2	126308146	126308546	-0.832624085	0.006667962	decrease
chr10	97446001	97446401	-0.795107856	0.006670699	decrease
chr5	133026286	133026686	-0.864122997	0.006672405	decrease
chr5	42994805	42995205	-0.881936236	0.006680401	decrease
chr15	66851492	66851892	-0.679017937	0.006692789	decrease
chr7	117020009	117020409	-0.886797112	0.006709882	decrease
chr19	40716559	40716959	-0.807852655	0.006719006	decrease
chr17	40317976	40318376	-0.79308967	0.006728821	decrease
chr16	20806424	20806824	-0.849340753	0.006736779	decrease
chr5	163437507	163437907	-0.718549412	0.006738373	decrease
chr20	14337344	14337744	-0.773329055	0.00673855	decrease
chr2	127197793	127198193	-0.880712812	0.00674107	decrease
chr1	160284933	160285333	-0.825094684	0.00674167	decrease
chr15	101276868	101277268	-0.825862465	0.006755596	decrease
chr7	67302403	67302803	-0.796765066	0.006759624	decrease
chr19	47257025	47257425	-0.731876704	0.006762206	decrease
chr1	22451866	22452266	-0.886898671	0.006764442	decrease
chr1	153958280	153958680	-0.884773149	0.006766026	decrease
chr17	49361103	49361503	-0.890240074	0.006767	decrease
chr4	107720009	107720409	-0.840963996	0.006768603	decrease
chr11	72434280	72434680	-0.788671327	0.006786572	decrease
chr10	1056632	1057032	-0.848281707	0.006795506	decrease
chr1	54357242	54357642	-0.911995351	0.006812258	decrease

chr16	67660219	67660619	-0.698143243	0.006814473	decrease
chr7	100177342	100177742	-0.933965686	0.006821548	decrease
chr13	54572360	54572760	-0.901883188	0.006821827	decrease
chr1	146387177	146387577	-0.682706602	0.006822596	decrease
chr2	71068252	71068652	-0.717753386	0.006846426	decrease
chr15	73633384	73633784	-0.833264024	0.006881616	decrease
chr20	25407512	25407912	-0.869570888	0.006884132	decrease
chr6	27617742	27618142	-0.870505206	0.006889952	decrease
chr1	61724950	61725350	-0.673065977	0.006896321	decrease
chr11	129374437	129374837	-0.953093435	0.006898835	decrease
chr12	112013099	112013499	-0.74043413	0.006903729	decrease
chr12	65729018	65729418	-0.940283594	0.006908996	decrease
chr3	98901646	98902046	-0.91133041	0.006909329	decrease
chr2	95207154	95207554	-0.887107484	0.006917415	decrease
chr12	105107668	105108068	-0.923388267	0.006918328	decrease
chr3	184249974	184250374	-0.80032515	0.006919399	decrease
chr1	54764526	54764926	-0.649364462	0.006920521	decrease
chr21	16194137	16194537	-0.902265657	0.006934707	decrease
chr2	42494773	42495173	-0.908094376	0.006934833	decrease
chr19	45770314	45770714	-0.610870336	0.006951868	decrease
chr11	122656413	122656813	-0.926344529	0.006955601	decrease
chr14	64338476	64338876	-0.828332523	0.006956372	decrease
chr11	18526619	18527019	-0.703060891	0.006962087	decrease
chr1	40257630	40258030	-0.843055484	0.006967669	decrease
chr10	70233463	70233863	-0.905400095	0.006978019	decrease
chr1	230642374	230642774	-0.736137967	0.006979216	decrease
chr2	111897969	111898369	-0.821630978	0.006981037	decrease
chr1	155000832	155001232	-0.617985845	0.006984519	decrease
chrX	47190596	47190996	-0.76103056	0.006992754	decrease
chr1	156282878	156283278	-0.808250076	0.006996101	decrease
chr22	36065999	36066399	-0.882404926	0.006997926	decrease
chr5	72955543	72955943	-0.932952472	0.006999946	decrease
chr5	113203090	113203490	-0.807467255	0.007007092	decrease
chr18	9136518	9136918	-0.916637005	0.00701532	decrease
chr12	132760489	132760889	-0.799308892	0.007030412	decrease
chr2	240586284	240586684	-0.725348704	0.007045326	decrease
chr14	73644729	73645129	-0.781420944	0.007051192	decrease
chr21	33324496	33324896	-0.668796334	0.007060324	decrease
chr14	22982783	22983183	-0.844779596	0.007064838	decrease
chr19	35869796	35870196	-0.790869297	0.007081946	decrease
chr12	100573678	100574078	-0.893036191	0.007104697	decrease
chr6	42979227	42979627	-0.882081507	0.007108422	decrease
chr15	81266053	81266453	-0.851418988	0.007111426	decrease
chr1	168178799	168179199	-0.762898448	0.007112901	decrease
chr4	781937	782337	-0.628631135	0.007113093	decrease
chr8	63039196	63039596	-0.828867659	0.007115128	decrease
chr19	3721338	3721738	-0.855772729	0.007132687	decrease
chr18	62714962	62715362	-0.868529243	0.00713467	decrease
chr3	149086223	149086623	-0.766161865	0.00713676	decrease
chr15	74873018	74873418	-0.770459777	0.007148184	decrease
chr3	56556978	56557378	-0.743411436	0.007148202	decrease
chr6	132512833	132513233	-0.799752196	0.007152971	decrease
chr19	43668460	43668860	-0.670087835	0.007155903	decrease
chr10	87504665	87505065	-0.808832547	0.007160281	decrease
chr13	94601526	94601926	-0.821652365	0.007174595	decrease
chr22	50562847	50563247	-0.765232533	0.007175132	decrease
chr1	150973098	150973498	-0.855597782	0.007175869	decrease
chr17	32350034	32350434	-0.790968299	0.007183271	decrease
chr8	38386113	38386513	-0.792572743	0.007187501	decrease
chr21	17512564	17512964	-0.9062601	0.00719067	decrease
chr17	47189053	47189453	-0.741101986	0.007194369	decrease
chrX	73850776	73851176	-0.713185043	0.007209566	decrease
chr10	25016288	25016688	-0.737738614	0.007214714	decrease
chr7	143288119	143288519	-0.774503721	0.007231448	decrease
chr8	127735461	127735861	-0.859695795	0.007232668	decrease
chrX	53071615	53072015	-0.850962021	0.007237225	decrease
chr2	9555425	9555825	-0.81170993	0.007237698	decrease
chr1	28088335	28088735	-0.734142479	0.007240472	decrease
chr8	11703207	11703607	-0.824954858	0.00724436	decrease
chr16	30400526	30400926	-0.788750372	0.007246639	decrease
chr10	87735092	87735492	-0.917199049	0.007254386	decrease
chr22	30356747	30357147	-0.827787655	0.007257972	decrease
chr2	240438098	240438498	-0.872424239	0.007265574	decrease
chr2	61536512	61536912	-0.810579172	0.007282744	decrease
chr14	20305685	20306085	-0.906981528	0.007301907	decrease
chrX	127866776	127867176	-0.830642512	0.00730469	decrease

chr7	4973740	4974140	-0.907106349	0.007319906	decrease
chr1	229341322	229341722	-0.836914585	0.007325306	decrease
chr11	67805560	67805960	-0.679598769	0.007342109	decrease
chr6	113858593	113858993	-0.839724493	0.007349764	decrease
chr10	15860172	15860572	-0.885590706	0.007362546	decrease
chr16	54929267	54929667	-0.731226211	0.007369374	decrease
chr8	54382021	54382421	-0.834132907	0.007370141	decrease
chr2	216114643	216115043	-0.81808808	0.007387401	decrease
chr19	17579837	17580237	-0.75720043	0.007390634	decrease
chr1	236063973	236064373	-0.80571941	0.007402762	decrease
chr6	167827805	167828205	-0.942022217	0.007407587	decrease
chr2	60555186	60555586	-0.862328341	0.007408124	decrease
chr1	234373244	234373644	-0.589633356	0.007410968	decrease
chr7	23105609	23106009	-0.81082869	0.00741441	decrease
chr6	98833739	98834139	-0.742267811	0.007417189	decrease
chr15	99567253	99567653	-0.906696614	0.007422611	decrease
chr3	172750558	172750958	-0.816638407	0.007440296	decrease
chr1	33036687	33037087	-0.64562848	0.007447438	decrease
chr1	26864900	26865300	-0.797951479	0.00746701	decrease
chr12	72640059	72640459	-0.872495128	0.00746918	decrease
chr14	24270958	24271358	-0.809776069	0.00748285	decrease
chr6	156776527	156776927	-0.910835718	0.007484503	decrease
chr13	95676778	95677178	-0.827893331	0.007497354	decrease
chr9	96449671	96450071	-0.93577416	0.00751548	decrease
chr11	47426520	47426920	-0.764682766	0.007527817	decrease
chr20	13995174	13995574	-0.904078402	0.007545157	decrease
chr12	81077599	81077999	-0.858020563	0.007547547	decrease
chr3	59049774	59050174	-0.877315481	0.007555908	decrease
chr10	100347246	100347646	-0.903180639	0.007562113	decrease
chr4	161818058	161818458	-0.8882533	0.007573847	decrease
chr5	65563061	65563461	-0.726531366	0.007598687	decrease
chr12	122500935	122501335	-0.712614323	0.007623707	decrease
chr19	35758021	35758421	-0.74180266	0.007632728	decrease
chr9	27080816	27081216	-0.903539918	0.007642283	decrease
chr15	42495515	42495915	-0.896197524	0.00765322	decrease
chr9	111630909	111631309	-0.854008043	0.00765887	decrease
chr4	54063652	54064052	-0.883643322	0.007661827	decrease
chr6	683895	684295	-0.845211391	0.007669891	decrease
chr1	145927196	145927596	-0.736637551	0.007671578	decrease
chr9	124861999	124862399	-0.714843767	0.007696549	decrease
chr16	68843620	68844020	-0.817946607	0.007700816	decrease
chr1	232950380	232950780	-0.732775154	0.007709712	decrease
chrX	13653073	13653473	-0.784474867	0.007711236	decrease
chr1	155050652	155051052	-0.815200277	0.007713018	decrease
chr7	134458801	134459201	-0.861340035	0.007716513	decrease
chr18	9102547	9102947	-0.891738774	0.007736059	decrease
chr11	46389456	46389856	-0.806637908	0.007736123	decrease
chr14	21456095	21456495	-0.663065773	0.007739834	decrease
chr20	16574830	16575230	-0.819101145	0.007743311	decrease
chr20	10673936	10674336	-0.924189317	0.00774965	decrease
chr2	65056057	65056457	-0.817803581	0.007754085	decrease
chr12	50222324	50222724	-0.879096355	0.007757508	decrease
chr5	77776140	77776540	-0.878498204	0.007763842	decrease
chr4	143513211	143513611	-0.687876119	0.00776454	decrease
chr20	31604607	31605007	-0.749862953	0.007764871	decrease
chr6	53547809	53548209	-0.757800704	0.007767671	decrease
chr3	48717280	48717680	-0.772107627	0.007768682	decrease
chr12	114669287	114669687	-0.899440167	0.007779554	decrease
chr3	25782908	25783308	-0.922841636	0.007785938	decrease
chr1	226186673	226187073	-0.79100397	0.007792561	decrease
chr6	57222273	57222673	-0.859078311	0.007792587	decrease
chr5	150441611	150442011	-0.806288286	0.007807993	decrease
chr4	118685305	118685705	-0.813705297	0.007808512	decrease
chr1	28736472	28736872	-0.756425197	0.007813863	decrease
chr17	44899510	44899910	-0.680212586	0.007821754	decrease
chr4	48340984	48341384	-0.891650693	0.007823994	decrease
chr4	184734054	184734454	-0.666304477	0.007841433	decrease
chr7	55366040	55366440	-0.814253289	0.007848811	decrease
chr1	192808762	192809162	-0.852037541	0.007858835	decrease
chr17	43367872	43368272	-0.878027986	0.007864573	decrease
chr7	33062832	33063232	-0.663059258	0.007871751	decrease
chr4	124713217	124713617	-0.824901152	0.007871915	decrease
chr2	25877761	25878161	-0.855647605	0.007872784	decrease
chr1	52843624	52844024	-0.895777179	0.007882518	decrease
chr16	14285099	14285499	-0.885820414	0.007882578	decrease
chr6	26365134	26365534	-0.825841175	0.007889767	decrease

chr8	18084003	18084403	-0.819137027	0.007889859	decrease
chr4	89307251	89307651	-0.887233103	0.007891353	decrease
chr1	150561182	150561582	-0.667265754	0.007892419	decrease
chr1	56645787	56646187	-0.792259368	0.007893782	decrease
chr2	218671923	218672323	-0.719913801	0.007896854	decrease
chr8	37694942	37695342	-0.825878831	0.007901021	decrease
chr1	226408148	226408548	-0.779774265	0.00791782	decrease
chr19	3684473	3684873	-0.900607594	0.00793068	decrease
chr12	9448038	9448438	-0.908997651	0.007939062	decrease
chr15	77959311	77959711	-0.836144094	0.007939978	decrease
chr7	6348912	6349312	-0.854262473	0.007944441	decrease
chr5	142024839	142025239	-0.879167346	0.007946018	decrease
chr10	117541795	117542195	-0.689335604	0.007948881	decrease
chr12	121859249	121859649	-0.830827824	0.007963313	decrease
chr7	112206645	112207045	-0.807588014	0.007968457	decrease
chr1	220529051	220529451	-0.920904144	0.007970773	decrease
chr3	128650589	128650989	-0.866789594	0.00797382	decrease
chr14	30690397	30690797	-0.891364064	0.007978517	decrease
chr6	53795722	53796122	-0.899159589	0.007980758	decrease
chr1	36224407	36224807	-0.58700532	0.008006953	decrease
chr1	228174219	228174619	-0.688483644	0.008009117	decrease
chr1	223142862	223143262	-0.804448167	0.0080234	decrease
chr6	99393851	99394251	-0.823756623	0.008023591	decrease
chr8	106271119	106271519	-0.871559748	0.008033268	decrease
chrX	18984537	18984937	-0.923223434	0.008041086	decrease
chr6	73461687	73462087	-0.897065524	0.008042016	decrease
chr17	80147173	80147573	-0.820533812	0.008044331	decrease
chr4	107654684	107655084	-0.919331891	0.008044608	decrease
chr2	206230303	206230703	-0.898283962	0.00804827	decrease
chr3	49358326	49358726	-0.745680285	0.008052344	decrease
chr12	108773867	108774267	-0.924866388	0.00805861	decrease
chr14	51095139	51095539	-0.754077147	0.008081658	decrease
chr1	165630936	165631336	-0.724978535	0.008083752	decrease
chr1	60281291	60281691	-0.891440603	0.008088742	decrease
chr1	39559578	39559978	-0.823827454	0.008090463	decrease
chr7	23181637	23182037	-0.79930463	0.008090708	decrease
chr10	125719246	125719646	-0.672757949	0.008094242	decrease
chr1	28544416	28544816	-0.852827884	0.008095075	decrease
chr6	26033336	26033736	-0.795572218	0.008100003	decrease
chr3	42819587	42819987	-0.849612649	0.008111278	decrease
chr19	38683764	38684164	-0.829841563	0.008114046	decrease
chr7	104945017	104945417	-0.87191627	0.008114746	decrease
chr11	8682359	8682759	-0.753485273	0.008133036	decrease
chr7	134432539	134432939	-0.719746257	0.008134031	decrease
chr8	96261293	96261693	-0.800982843	0.008135569	decrease
chrX	103464324	103464724	-0.769224763	0.008151536	decrease
chr5	16894565	16894965	-0.87881336	0.008152164	decrease
chr5	138331742	138332142	-0.659761352	0.008153162	decrease
chr16	1492923	1493323	-0.839670539	0.008158647	decrease
chr7	155298813	155299213	-0.75076813	0.008162962	decrease
chr1	11099323	11099723	-0.905664817	0.008165087	decrease
chr2	186694398	186694798	-0.868008778	0.008170807	decrease
chr1	225809721	225810121	-0.701433139	0.008205621	decrease
chr19	45615935	45616335	-0.680752491	0.008207006	decrease
chr6	10722796	10723196	-0.791932968	0.008213346	decrease
chr19	5720264	5720664	-0.713195003	0.008214216	decrease
chr16	72099255	72099655	-0.859173996	0.008214341	decrease
chr19	51703878	51704278	-0.886360558	0.008215596	decrease
chr3	45388317	45388717	-0.905648144	0.008240347	decrease
chrX	123860070	123860470	-0.792396362	0.008245307	decrease
chr19	45492889	45493289	-0.783832917	0.00824868	decrease
chr1	95116880	95117280	-0.872919784	0.008260184	decrease
chr13	67230993	67231393	-0.652550271	0.008261248	decrease
chr12	100200317	100200717	-0.799316197	0.008261416	decrease
chr12	49366574	49366974	-0.878878449	0.008279774	decrease
chr6	42929299	42929699	-0.711267939	0.008280682	decrease
chr5	173887845	173888245	-0.718257669	0.00828081	decrease
chr17	42388464	42388864	-0.705292389	0.008286198	decrease
chr15	58771036	58771436	-0.830508822	0.008294122	decrease
chr1	21345563	21345963	-0.899683423	0.008299754	decrease
chr15	64093665	64094065	-0.812890622	0.008316129	decrease
chr2	10121892	10122292	-0.868235252	0.008331236	decrease
chr3	133805672	133806072	-0.785585611	0.008337879	decrease
chr4	932328	932728	-0.826986012	0.008347815	decrease
chr1	214602986	214603386	-0.698696884	0.008369241	decrease
chr5	41903981	41904381	-0.70655682	0.008374238	decrease

chr1	11012274	11012674	-0.855030101	0.008376768	decrease
chr8	124997800	124998200	-0.862304378	0.00838754	decrease
chr2	151828456	151828856	-0.913711758	0.008391755	decrease
chr19	1605590	1605990	-0.815122588	0.008391805	decrease
chr4	112637078	112637478	-0.710855223	0.008392764	decrease
chr6	1388284	1388684	-0.833721755	0.008394959	decrease
chr4	98143241	98143641	-0.823094414	0.008395421	decrease
chr10	102917960	102918360	-0.714872352	0.008409542	decrease
chr11	74492983	74493383	-0.817682392	0.00844224	decrease
chr16	4253461	4253861	-0.877693905	0.008460272	decrease
chr16	68310769	68311169	-0.750698313	0.008461303	decrease
chr11	64917202	64917602	-0.769636838	0.008477884	decrease
chr10	97407252	97407652	-0.758469121	0.008478301	decrease
chr3	150408777	150409177	-0.900234326	0.008499301	decrease
chr5	109690171	109690571	-0.846277219	0.008505098	decrease
chr11	64878225	64878625	-0.851409876	0.008524814	decrease
chr5	94618596	94618996	-0.806911101	0.008541438	decrease
chr10	32055625	32056025	-0.920712428	0.008549588	decrease
chr3	10008822	10009222	-0.812930785	0.00854977	decrease
chr18	49487122	49487522	-0.872108855	0.008551793	decrease
chr3	51390786	51391186	-0.797047187	0.008555993	decrease
chr17	75845114	75845514	-0.765390019	0.008567628	decrease
chr15	101295370	101295770	-0.784572577	0.008569207	decrease
chr17	27294318	27294718	-0.829231146	0.00857353	decrease
chr19	39397491	39397891	-0.824567792	0.008582862	decrease
chr19	510737	511137	-0.748172206	0.008583546	decrease
chr2	195656929	195657329	-0.740949631	0.008591749	decrease
chr12	82358435	82358835	-0.784030016	0.008601109	decrease
chr5	178626515	178626915	-0.820053625	0.008627132	decrease
chr1	44674121	44674521	-0.617727945	0.008627698	decrease
chr7	100119475	100119875	-0.717505891	0.008639051	decrease
chr19	6372433	6372833	-0.730157747	0.008640675	decrease
chr1	236604438	236604838	-0.590405014	0.008642095	decrease
chr7	54759177	54759577	-0.897147468	0.008643467	decrease
chr14	89416265	89416665	-0.871392653	0.00864817	decrease
chr3	49469673	49470073	-0.810841906	0.008649413	decrease
chr14	88562873	88563273	-0.854746315	0.00866095	decrease
chr16	89874163	89874563	-0.745049238	0.008667336	decrease
chr1	8862566	8862966	-0.809303418	0.008678248	decrease
chr7	158704618	158705018	-0.794165777	0.008678875	decrease
chr11	35617861	35618261	-0.848129814	0.008679316	decrease
chr3	169242435	169242835	-0.81369235	0.008679436	decrease
chr19	48993376	48993776	-0.69914234	0.008686398	decrease
chr6	26659553	26659953	-0.786166231	0.008696727	decrease
chr4	176195267	176195667	-0.836027568	0.008710932	decrease
chr12	121811832	121812232	-0.763478155	0.008711747	decrease
chr11	62601786	62602186	-0.618155093	0.008739314	decrease
chr2	202375571	202375971	-0.76284247	0.008740652	decrease
chr20	62123389	62123789	-0.909069646	0.008742838	decrease
chr13	74324360	74324760	-0.704000606	0.008744916	decrease
chr12	98593450	98593850	-0.830825684	0.008776628	decrease
chr11	8911135	8911535	-0.780059547	0.008785305	decrease
chr17	82494422	82494822	-0.615201094	0.00878853	decrease
chr1	153986680	153987080	-0.758000438	0.008797661	decrease
chr1	149927655	149928055	-0.770630941	0.008813023	decrease
chr6	140897817	140898217	-0.799907061	0.008825209	decrease
chr10	110007834	110008234	-0.923444344	0.008831678	decrease
chr1	8026736	8027136	-0.842850935	0.008834236	decrease
chr22	20702836	20703236	-0.746771859	0.008848269	decrease
chr1	211382757	211383157	-0.817069744	0.008857834	decrease
chr4	3955572	3955972	-0.624258705	0.008864558	decrease
chr3	13308397	13308797	-0.906062272	0.008868971	decrease
chr5	61408622	61409022	-0.825593069	0.008934298	decrease
chr9	19380179	19380579	-0.823677338	0.008939687	decrease
chr3	48188346	48188746	-0.770022739	0.008941811	decrease
chr19	36838320	36838720	-0.773305688	0.008946248	decrease
chr14	20631816	20632216	-0.890877215	0.008958689	decrease
chr9	128098242	128098642	-0.751126693	0.008972835	decrease
chr16	5097895	5098295	-0.680141311	0.00897387	decrease
chr12	6937714	6938114	-0.757949301	0.008978982	decrease
chrX	104922029	104922429	-0.847536707	0.008982349	decrease
chr16	67226805	67227205	-0.692967894	0.00898331	decrease
chr2	200811475	200811875	-0.766337935	0.008984565	decrease
chr20	24992529	24992929	-0.813611824	0.008988909	decrease
chr3	157174831	157175231	-0.753702017	0.009015949	decrease
chr6	145814898	145815298	-0.760341371	0.009017599	decrease

chrX	48021278	48021678	-0.740106607	0.009020093	decrease
chr1	225999194	225999594	-0.670146512	0.009035713	decrease
chr22	29766987	29767387	-0.696907164	0.009037623	decrease
chr12	52156787	52157187	-0.907434286	0.009037825	decrease
chr3	47824601	47825001	-0.860456937	0.009062336	decrease
chr11	3824879	3825279	-0.845521932	0.009072157	decrease
chr2	85538744	85539144	-0.745466768	0.009082898	decrease
chr4	85827749	85828149	-0.875420765	0.00908498	decrease
chr13	113448930	113449330	-0.840981658	0.009091535	decrease
chr19	12806068	12806468	-0.84684161	0.009101859	decrease
chr19	9309775	9310175	-0.824910433	0.009106134	decrease
chr7	87152076	87152476	-0.819481872	0.009110805	decrease
chr3	141311655	141312055	-0.806527539	0.009137496	decrease
chr1	93833438	93833838	-0.855257206	0.009147369	decrease
chr1	26620829	26621229	-0.64812697	0.009147972	decrease
chr20	62011018	62011418	-0.901609095	0.009160478	decrease
chr14	23953686	23954086	-0.808055397	0.009162882	decrease
chr3	165751663	165752063	-0.912771659	0.00916704	decrease
chr18	50287629	50288029	-0.769689906	0.009176637	decrease
chr19	3185362	3185762	-0.722288566	0.009177086	decrease
chr5	180809976	180810376	-0.702939849	0.009178001	decrease
chr3	181712786	181713186	-0.906867604	0.009178147	decrease
chr4	127623395	127623795	-0.901217678	0.009182536	decrease
chr19	37078173	37078573	-0.82900937	0.009182818	decrease
chr6	109691163	109691563	-0.791613339	0.009199471	decrease
chr3	129399299	129399699	-0.776320938	0.009225691	decrease
chr1	228099640	228100040	-0.624723273	0.009230459	decrease
chr5	111757764	111758164	-0.907841191	0.009233153	decrease
chr17	2511589	2511989	-0.803256526	0.009235928	decrease
chr18	35972154	35972554	-0.744113076	0.009241617	decrease
chr8	125430045	125430445	-0.778710217	0.009249481	decrease
chr2	172085338	172085738	-0.736602936	0.009251975	decrease
chr14	69398397	69398797	-0.755961792	0.009265347	decrease
chr3	33097187	33097587	-0.894434717	0.00926854	decrease
chr11	67996866	67997266	-0.671712714	0.009269862	decrease
chr6	56842857	56843257	-0.881097842	0.009286616	decrease
chr6	36593820	36594220	-0.808752159	0.009313982	decrease
chr11	62787310	62787710	-0.837195976	0.00932379	decrease
chr12	98515250	98515650	-0.866967603	0.009345041	decrease
chr1	33180511	33180911	-0.786493321	0.009354167	decrease
chr1	54489537	54489937	-0.880763921	0.009355194	decrease
chr12	76084715	76085115	-0.768560413	0.009358186	decrease
chr1	97920972	97921372	-0.908808136	0.009360971	decrease
chr1	161530764	161531164	-0.776227381	0.009369401	decrease
chr6	166627337	166627737	-0.736902321	0.009385838	decrease
chr10	119596789	119597189	-0.685245453	0.009397978	decrease
chrX	154478953	154479353	-0.682708816	0.009401827	decrease
chr18	59139437	59139837	-0.786970253	0.009403454	decrease
chr19	35748232	35748632	-0.674486165	0.009410222	decrease
chrX	136909776	136910176	-0.724112033	0.009417281	decrease
chr14	60649675	60650075	-0.79702005	0.009441674	decrease
chr12	112108993	112109393	-0.877510198	0.009451738	decrease
chr12	31728908	31729308	-0.795807126	0.009452675	decrease
chr9	127786392	127786792	-0.807336722	0.009470713	decrease
chr14	37197761	37198161	-0.860869612	0.009470998	decrease
chr6	157379914	157380314	-0.80085028	0.009474125	decrease
chr5	107571841	107572413	-0.889281125	0.009474437	decrease
chr14	24213095	24213495	-0.703808621	0.009491179	decrease
chr16	31179788	31180188	-0.653405196	0.009495511	decrease
chr5	10249736	10250136	-0.79729966	0.009499617	decrease
chr11	59171542	59171942	-0.88135309	0.009501494	decrease
chr19	17209143	17209543	-0.854034993	0.009509652	decrease
chr22	39311154	39311554	-0.566300809	0.009512223	decrease
chr3	115147677	115148077	-0.76427878	0.009513455	decrease
chr9	69672972	69673372	-0.877271081	0.009517127	decrease
chr9	76460019	76460419	-0.911974345	0.009523369	decrease
chr1	228109097	228109497	-0.646591405	0.009528541	decrease
chr8	63168304	63168704	-0.791041827	0.009537078	decrease
chr20	34709615	34710015	-0.848788265	0.009552067	decrease
chr3	146161321	146161721	-0.828189878	0.00955431	decrease
chr4	94207529	94207929	-0.844120507	0.009555527	decrease
chr10	31760230	31760630	-0.883364115	0.009567521	decrease
chr19	42244544	42244944	-0.746967821	0.009569912	decrease
chr6	142147159	142147559	-0.764652554	0.009589247	decrease
chr10	14959276	14959676	-0.680422982	0.009599214	decrease
chr17	4365036	4365436	-0.852829694	0.009599677	decrease

chr3	33798596	33798996	-0.692776497	0.009608778	decrease
chr6	77650264	77650664	-0.798737569	0.009613598	decrease
chr1	225824712	225825112	-0.811698967	0.009614017	decrease
chr1	114757672	114758072	-0.621293691	0.009614668	decrease
chr17	28854860	28855260	-0.768338495	0.00961666	decrease
chr1	116570741	116571141	-0.847749399	0.009619469	decrease
chr11	47269148	47269548	-0.775891818	0.009627019	decrease
chr9	127396757	127397157	-0.629985326	0.009633309	decrease
chr9	34376942	34377342	-0.815183678	0.009639675	decrease
chr10	75402045	75402445	-0.680317752	0.009658076	decrease
chr3	142596206	142596606	-0.688728717	0.009658772	decrease
chr6	10404462	10404862	-0.841685835	0.00965895	decrease
chr16	29961636	29962036	-0.75488652	0.009664875	decrease
chr1	42959060	42959460	-0.617925101	0.009667316	decrease
chrX	135032031	135032431	-0.702942315	0.009672394	decrease
chr3	50121461	50121861	-0.728818744	0.009685044	decrease
chr2	36618303	36618703	-0.910632074	0.00968693	decrease
chr2	70124667	70125067	-0.864810394	0.009694133	decrease
chr3	39406493	39406893	-0.820829805	0.009704647	decrease
chr3	5026750	5027150	-0.841785925	0.009716525	decrease
chr6	43635668	43636068	-0.725844216	0.009734116	decrease
chr9	130287686	130288086	-0.882702142	0.009752172	decrease
chr10	70405334	70405734	-0.816346361	0.009755066	decrease
chr6	26204319	26204719	-0.709360957	0.009761277	decrease
chr2	176269408	176269808	-0.851207115	0.009762357	decrease
chr4	56387078	56387478	-0.733130584	0.009762502	decrease
chr19	50658250	50658650	-0.63625345	0.009787962	decrease
chr1	226724076	226724476	-0.692888264	0.009792249	decrease
chr4	121380738	121381138	-0.840241103	0.009795454	decrease
chr14	32134513	32134913	-0.832511091	0.009796766	decrease
chr5	179678243	179678643	-0.70467531	0.009799548	decrease
chr22	20394102	20394502	-0.835367469	0.009810929	decrease
chr1	75614601	75615001	-0.684026204	0.009811141	decrease
chr16	8797477	8797877	-0.800640076	0.009819208	decrease
chr17	5191677	5192077	-0.833645883	0.009849723	decrease
chr12	6768473	6768873	-0.839958904	0.009850178	decrease
chr10	72625828	72626228	-0.782701103	0.009851632	decrease
chr11	66282585	66282985	-0.844291705	0.009861514	decrease
chr9	122264651	122265051	-0.840006922	0.00986166	decrease
chr2	112584440	112584840	-0.637335701	0.009870049	decrease
chr1	107626449	107626849	-0.87915078	0.009879453	decrease
chr8	102807382	102807782	-0.790136955	0.009880929	decrease
chr16	11851592	11851992	-0.817621506	0.009898216	decrease
chr2	77567797	77568197	-0.833649901	0.009904463	decrease
chr7	149149209	149149609	-0.722766949	0.009904955	decrease
chr16	2459680	2460080	-0.771487685	0.009916353	decrease
chr1	58903822	58904222	-0.791940462	0.009921996	decrease
chr20	5126353	5126753	-0.797276307	0.009929556	decrease
chr19	47483924	47484324	-0.766938606	0.009933401	decrease
chr19	35745315	35745715	-0.700492645	0.009982934	decrease
chr17	8383149	8383549	-0.804004748	0.00999013	decrease
chr14	95534649	95535049	-0.702849359	0.009996311	decrease
chr2	24793139	24793539	-0.711517954	0.010003114	decrease
chr13	26557168	26557568	-0.831240862	0.010012448	decrease
chrX	154516360	154516760	-0.762963421	0.010019146	decrease
chr19	40443080	40443480	-0.720537542	0.010029298	decrease
chr19	40420933	40421333	-0.799813203	0.010040986	decrease
chr7	55255375	55255775	-0.894267921	0.010068019	decrease
chr19	42302326	42302726	-0.651607029	0.010072892	decrease
chrX	64205882	64206282	-0.762834579	0.010074712	decrease
chr13	91349029	91349429	-0.820171021	0.010076854	decrease
chr4	98657594	98657994	-0.805651096	0.010078005	decrease
chr19	48795238	48795638	-0.744916067	0.010107031	decrease
chr1	221741986	221742386	-0.653727913	0.010110051	decrease
chr19	2165380	2165780	-0.85703834	0.010118113	decrease
chr1	43649821	43650221	-0.595280601	0.010118693	decrease
chr8	72479489	72479889	-0.851330646	0.010122803	decrease
chr2	117814407	117814807	-0.745523632	0.010135714	decrease
chr7	135662261	135662661	-0.711416327	0.010148723	decrease
chr5	148383732	148384132	-0.773200097	0.010151251	decrease
chr20	62376967	62377367	-0.855306326	0.010160303	decrease
chr14	59919234	59919634	-0.881015465	0.01017226	decrease
chr10	126249837	126250237	-0.883330649	0.010184771	decrease
chr9	86282358	86282758	-0.834844342	0.010185724	decrease
chr2	70293406	70293806	-0.77406126	0.010186831	decrease
chr8	127736141	127736541	-0.833888128	0.010194695	decrease

chr14	50311839	50312239	-0.709805524	0.010201829	decrease
chr16	31074250	31074650	-0.691089704	0.010202407	decrease
chr16	31442853	31443253	-0.81253725	0.010219668	decrease
chr19	10333292	10333692	-0.633282794	0.010225756	decrease
chr2	26033703	26034103	-0.736194667	0.010253697	decrease
chr1	65002409	65002809	-0.831028662	0.010268104	decrease
chr4	4672995	4673395	-0.886043898	0.010299252	decrease
chr1	117929239	117929639	-0.717038293	0.010304174	decrease
chr19	9819088	9819488	-0.729937194	0.010309372	decrease
chr1	218284876	218285276	-0.84067195	0.010312648	decrease
chr6	26123611	26124011	-0.632930393	0.010322443	decrease
chr11	129895576	129895976	-0.838793548	0.010340517	decrease
chr16	57736642	57737042	-0.810153855	0.010346916	decrease
chr2	222861889	222862289	-0.833081171	0.010349903	decrease
chr12	108561331	108561731	-0.710674871	0.010353136	decrease
chr17	8295406	8295806	-0.779470158	0.01035796	decrease
chr12	48815145	48815545	-0.818249914	0.010358593	decrease
chr1	28328899	28329299	-0.779084556	0.010366028	decrease
chr1	214514221	214514621	-0.747775197	0.010372414	decrease
chr11	108008738	108009138	-0.770091131	0.010372591	decrease
chr12	6533930	6534330	-0.601387447	0.010387296	decrease
chr21	36156305	36156705	-0.778649039	0.010390283	decrease
chr12	51172687	51173087	-0.768816735	0.010393817	decrease
chr19	42075815	42076215	-0.755086348	0.010398217	decrease
chr17	5439258	5439658	-0.860752903	0.010407966	decrease
chr7	97872195	97872595	-0.749457119	0.01041056	decrease
chr5	55995105	55995505	-0.886052341	0.010416059	decrease
chr5	117455173	117455573	-0.765907051	0.010416652	decrease
chr5	55534844	55535244	-0.822318007	0.010452856	decrease
chrX	119871663	119872063	-0.713601402	0.01046275	decrease
chr14	56579420	56579820	-0.738294697	0.010464335	decrease
chr5	112707357	112707757	-0.746306775	0.010475614	decrease
chr7	98252050	98252450	-0.810994128	0.010496398	decrease
chr4	91791510	91791910	-0.889170473	0.010499232	decrease
chr17	42998101	42998501	-0.705421159	0.010517143	decrease
chr16	29805449	29805849	-0.708452289	0.010522981	decrease
chr19	56104976	56105376	-0.872691051	0.010530419	decrease
chr22	17638366	17638766	-0.721718839	0.010554978	decrease
chr1	1693014	1693414	-0.819210573	0.010585054	decrease
chr16	29944005	29944405	-0.626850507	0.010590332	decrease
chr9	129080791	129081191	-0.660044853	0.010596335	decrease
chr5	135834316	135834716	-0.783989372	0.01059883	decrease
chr5	176537530	176537930	-0.645201913	0.010604678	decrease
chr7	105532006	105532406	-0.689999774	0.010605217	decrease
chr4	9691555	9691955	-0.711318101	0.010607727	decrease
chr15	88546335	88546735	-0.805079321	0.01061021	decrease
chr16	678683	679083	-0.651875903	0.010633371	decrease
chr11	4607974	4608374	-0.767847654	0.010659618	decrease
chr6	52882170	52882570	-0.853592275	0.010659748	decrease
chr8	27948266	27948666	-0.872073709	0.010661734	decrease
chr16	88809681	88810081	-0.624274169	0.010669614	decrease
chr20	3045669	3046069	-0.827301265	0.010672898	decrease
chr5	173056937	173057337	-0.691836664	0.010685463	decrease
chr19	12610839	12611239	-0.681741665	0.010689289	decrease
chr21	14216166	14216566	-0.826631704	0.010689828	decrease
chr15	74545950	74546350	-0.783801991	0.010690187	decrease
chr2	232524248	232524648	-0.836281126	0.010702352	decrease
chr10	136708	137108	-0.653044662	0.010705759	decrease
chr2	224585695	224586095	-0.747974141	0.010707576	decrease
chr3	120417395	120417795	-0.887699228	0.010738106	decrease
chr9	129036220	129036620	-0.697609008	0.010752076	decrease
chrX	136880479	136880879	-0.81194884	0.010756583	decrease
chr5	31531988	31532388	-0.727486984	0.010763217	decrease
chr3	50227672	50228072	-0.799525674	0.010766706	decrease
chr3	50359424	50359824	-0.695728776	0.010775306	decrease
chr7	99558558	99558958	-0.751323788	0.010775666	decrease
chr2	43219564	43219964	-0.767054591	0.010784675	decrease
chr19	12766141	12766541	-0.759735774	0.010789274	decrease
chr2	9813988	9814388	-0.878871733	0.010789372	decrease
chr17	41688725	41689125	-0.593179011	0.01078981	decrease
chr11	73982428	73982828	-0.861717939	0.010801369	decrease
chr19	36054286	36054686	-0.598781641	0.01080747	decrease
chr17	21280042	21280442	-0.755370604	0.010817477	decrease
chr19	506804	507204	-0.639264611	0.010817834	decrease
chr19	57681742	57682142	-0.777665567	0.01082303	decrease
chr13	68257365	68257765	-0.671539321	0.010828934	decrease

chr10	79904667	79905067	-0.721931198	0.010838959	decrease
chr7	87219508	87219908	-0.834265965	0.01083972	decrease
chr2	135876443	135876843	-0.771692678	0.010850413	decrease
chr3	50274322	50274722	-0.748535182	0.010885435	decrease
chr10	1048954	1049354	-0.70033403	0.01090703	decrease
chr12	116533300	116533700	-0.843117471	0.010910461	decrease
chr13	68159852	68160252	-0.811355386	0.010911823	decrease
chr13	32315587	32315987	-0.744820554	0.010912535	decrease
chr6	136792849	136793249	-0.814715803	0.010922843	decrease
chr19	1279613	1280013	-0.733783484	0.010936183	decrease
chr9	121370102	121370502	-0.780190835	0.010964486	decrease
chr19	57599719	57600119	-0.839466221	0.01097097	decrease
chr1	234610558	234610958	-0.785224311	0.010977802	decrease
chr4	119730209	119730609	-0.806075953	0.010992697	decrease
chr2	200889199	200889599	-0.659391731	0.010995736	decrease
chr22	46296467	46296867	-0.686092054	0.010996254	decrease
chr2	219498448	219498848	-0.708275266	0.010999577	decrease
chr16	74606920	74607320	-0.813983146	0.011022899	decrease
chr21	31732502	31732902	-0.788443018	0.011023413	decrease
chr9	15510917	15511317	-0.831604954	0.011025431	decrease
chr3	42804201	42804601	-0.654300792	0.011032399	decrease
chr7	44582113	44582513	-0.697340866	0.01103746	decrease
chr14	77457397	77457797	-0.643208845	0.011045635	decrease
chr11	66268018	66268418	-0.654629961	0.011069524	decrease
chr22	37199329	37199729	-0.727977505	0.011077253	decrease
chr12	6852018	6852418	-0.519433366	0.011086463	decrease
chr6	26383090	26383490	-0.864001865	0.011088737	decrease
chr2	25376912	25377312	-0.831883141	0.011093924	decrease
chr20	31723335	31723735	-0.778533395	0.011126802	decrease
chr20	62386099	62386499	-0.808749141	0.01113246	decrease
chr6	28399474	28399874	-0.831750507	0.011133363	decrease
chr14	62739803	62740203	-0.820351234	0.011135613	decrease
chr19	39435862	39436262	-0.678527594	0.011145828	decrease
chr1	66332116	66332516	-0.694388525	0.011147908	decrease
chr3	111674492	111674892	-0.768597988	0.011156633	decrease
chr10	68988656	68989056	-0.734562922	0.011160425	decrease
chr11	85628096	85628496	-0.685489653	0.011171747	decrease
chr17	16380628	16381028	-0.719037039	0.011178215	decrease
chr7	102063944	102064344	-0.86662545	0.011183806	decrease
chr5	95711555	95711955	-0.631260924	0.011201204	decrease
chr1	42682086	42682486	-0.668364155	0.011204886	decrease
chr13	37000203	37000603	-0.698036408	0.011215374	decrease
chr3	50616230	50616630	-0.728083646	0.011234723	decrease
chr9	89180087	89180487	-0.87762727	0.011241502	decrease
chr10	89643910	89644310	-0.783220756	0.011255967	decrease
chr2	11483282	11483682	-0.836393312	0.01125693	decrease
chr14	41605604	41606004	-0.639683966	0.011296355	decrease
chr2	69829322	69829722	-0.760732167	0.011314703	decrease
chr12	132622759	132623159	-0.759856123	0.011321182	decrease
chr19	6460158	6460558	-0.701391018	0.011328192	decrease
chr9	35072569	35072969	-0.734585425	0.01133702	decrease
chr10	78033599	78033999	-0.733132684	0.011355094	decrease
chr19	10864592	10864992	-0.824511407	0.01135888	decrease
chr2	240998222	240998622	-0.833715144	0.011360341	decrease
chr6	139708742	139709142	-0.819249633	0.011364082	decrease
chr9	137022100	137022500	-0.794613048	0.011365241	decrease
chr17	30600096	30600496	-0.769351516	0.011367543	decrease
chr6	43506591	43506991	-0.756605169	0.011382434	decrease
chr19	48363803	48364203	-0.631889356	0.01139784	decrease
chr21	44517298	44517698	-0.765312708	0.011402328	decrease
chr19	6828938	6829338	-0.701392623	0.011405089	decrease
chr10	104268867	104269267	-0.762980021	0.011409087	decrease
chr10	124791864	124792264	-0.837997996	0.011409454	decrease
chr10	95445751	95446151	-0.875491754	0.011430671	decrease
chr2	39121136	39121536	-0.732388433	0.011454311	decrease
chr19	34926872	34927272	-0.871241739	0.011463114	decrease
chr6	111259055	111259455	-0.783304384	0.011495128	decrease
chr1	161201899	161202299	-0.693695732	0.011514418	decrease
chr6	116616007	116616407	-0.72729773	0.011524541	decrease
chr1	25239657	25240057	-0.840276604	0.011533257	decrease
chr2	70257710	70258110	-0.807054055	0.011537435	decrease
chr1	37692061	37692461	-0.693218562	0.011537695	decrease
chr4	165327314	165327714	-0.671480646	0.011543743	decrease
chr3	184335333	184335733	-0.742308916	0.011551733	decrease
chr1	68496933	68497333	-0.651284163	0.011557665	decrease
chr20	37289478	37289878	-0.79875555	0.011590519	decrease

chr17	7440449	7440849	-0.740998182	0.011595308	decrease
chr5	132866317	132866717	-0.661171275	0.01159555	decrease
chr2	8681840	8682240	-0.784346611	0.011602124	decrease
chr8	31033885	31034285	-0.850653113	0.01162218	decrease
chr10	110210925	110211325	-0.804727284	0.011630687	decrease
chr16	89565149	89565549	-0.586385195	0.011631032	decrease
chr2	183078725	183079125	-0.789089034	0.011635588	decrease
chr6	133242636	133243036	-0.766980011	0.011637486	decrease
chr7	131327756	131328156	-0.854520535	0.011643574	decrease
chr14	35046363	35046763	-0.859961208	0.01164817	decrease
chr10	102152063	102152463	-0.621569135	0.011652043	decrease
chr15	101277469	101277869	-0.768310014	0.011653616	decrease
chr14	32076263	32076663	-0.803660593	0.011672968	decrease
chr5	149551457	149551857	-0.68941508	0.011678369	decrease
chr3	179604381	179604781	-0.85296094	0.011690425	decrease
chr17	58007320	58007720	-0.657619648	0.011695277	decrease
chr2	10120066	10120466	-0.828371979	0.011697314	decrease
chr8	142738522	142739135	-0.736789493	0.011704468	decrease
chr3	186806377	186806777	-0.85053339	0.011706553	decrease
chr7	139118925	139119325	-0.797228109	0.011715638	decrease
chr9	32551020	32551420	-0.770261442	0.011720299	decrease
chr11	93741365	93741765	-0.649011222	0.011724134	decrease
chr3	4868211	4868611	-0.784193449	0.01173181	decrease
chr4	15479269	15479669	-0.836106576	0.011750342	decrease
chr10	89414675	89415075	-0.87516445	0.011757389	decrease
chr4	86891817	86892217	-0.876557704	0.011774324	decrease
chr12	132561062	132561462	-0.634401427	0.011775632	decrease
chr1	86704292	86704692	-0.750860488	0.011778871	decrease
chr12	120994751	120995151	-0.696287387	0.011784506	decrease
chr6	26157667	26158067	-0.622807972	0.011790444	decrease
chr19	46009566	46009966	-0.810348654	0.011792195	decrease
chr17	2399651	2400051	-0.709285923	0.011799175	decrease
chr6	83193050	83193450	-0.815495877	0.011800545	decrease
chr1	219839590	219839990	-0.791534322	0.011808167	decrease
chr12	133080087	133080487	-0.562563936	0.011844428	decrease
chr17	42609191	42609591	-0.795209762	0.011850387	decrease
chr9	32572362	32572762	-0.76961128	0.011868843	decrease
chr22	17158565	17158965	-0.840693148	0.011871099	decrease
chr20	32665183	32665583	-0.868644478	0.011940584	decrease
chrX	41085787	41086187	-0.859699467	0.011941714	decrease
chr3	24494342	24494742	-0.840874801	0.011945841	decrease
chr5	150904942	150905342	-0.652021075	0.011963021	decrease
chr7	93621948	93622348	-0.849572604	0.011984632	decrease
chr7	13920130	13920530	-0.810554322	0.011991785	decrease
chr6	166008585	166008985	-0.749053942	0.012019121	decrease
chr19	8308078	8308478	-0.763321815	0.012061603	decrease
chr1	197915874	197916274	-0.771629266	0.012073527	decrease
chr16	3124358	3124758	-0.687061496	0.012085309	decrease
chr6	26199144	26199544	-0.722960838	0.012085999	decrease
chr6	6008313	6008713	-0.810895742	0.012091859	decrease
chr13	80132881	80133281	-0.690728306	0.012098386	decrease
chr7	7640490	7640890	-0.720240243	0.012109132	decrease
chr1	32520567	32520967	-0.750875083	0.012123295	decrease
chr5	179623757	179624157	-0.614687418	0.012126536	decrease
chr3	107599049	107599449	-0.856400595	0.012128864	decrease
chr12	107685639	107686039	-0.752443932	0.012134396	decrease
chr1	203267334	203267734	-0.85640534	0.012141897	decrease
chr11	64246944	64247344	-0.759527076	0.012177596	decrease
chr15	39782501	39782901	-0.856385412	0.012179545	decrease
chr2	176297108	176297508	-0.842492791	0.012181217	decrease
chr2	100562694	100563094	-0.725907311	0.012205763	decrease
chr21	17454526	17454926	-0.707944673	0.012230882	decrease
chr1	64745954	64746354	-0.754842169	0.012231111	decrease
chr6	68634484	68634884	-0.873538314	0.012237315	decrease
chr19	47113120	47113520	-0.616793769	0.012238119	decrease
chr18	21612153	21612553	-0.679138265	0.01226506	decrease
chr1	226109597	226109997	-0.763402655	0.012271595	decrease
chr17	2303259	2303659	-0.796403527	0.012277464	decrease
chr3	107522764	107523164	-0.791442121	0.012280005	decrease
chr6	88963385	88963785	-0.753869523	0.012282624	decrease
chr2	37084249	37084649	-0.710210763	0.012282882	decrease
chr14	72894309	72894709	-0.777380868	0.012291393	decrease
chr11	27233988	27234388	-0.856246709	0.012293132	decrease
chr9	127608113	127608513	-0.788923717	0.012308276	decrease
chr1	21607824	21608224	-0.767507178	0.012313228	decrease
chr17	58631378	58631778	-0.614219625	0.012315694	decrease

chr2	202634598	202634998	-0.793274006	0.012325846	decrease
chr11	72041600	72042000	-0.740880431	0.012326968	decrease
chr13	52652505	52652905	-0.642992639	0.012332089	decrease
chr3	150546645	150547045	-0.688880944	0.012335502	decrease
chr2	135741493	135741893	-0.705005013	0.012335536	decrease
chr3	179147747	179148147	-0.821871919	0.012346611	decrease
chr4	102501862	102502262	-0.787956514	0.012355455	decrease
chr19	12772389	12772789	-0.833761716	0.012357834	decrease
chr6	5003709	5004109	-0.721900244	0.012397955	decrease
chr12	49018427	49018827	-0.677547746	0.012407303	decrease
chr19	11597251	11597651	-0.725505313	0.012417616	decrease
chr1	145721262	145721662	-0.822991251	0.012431219	decrease
chr6	28141631	28142031	-0.705893569	0.012444461	decrease
chr5	73565552	73565952	-0.655665711	0.012455608	decrease
chr2	161160276	161160676	-0.790224821	0.012461708	decrease
chr6	36874581	36874981	-0.797060545	0.012464921	decrease
chr20	2658443	2658843	-0.682106404	0.012467298	decrease
chr12	123618397	123618797	-0.837528242	0.01246917	decrease
chr16	54286383	54286783	-0.780621092	0.012475568	decrease
chr18	70205690	70206090	-0.852880588	0.012484732	decrease
chr11	123741320	123741720	-0.840014345	0.012498942	decrease
chr1	67827819	67828219	-0.802302654	0.012500465	decrease
chr17	75393679	75394079	-0.672237554	0.012501116	decrease
chr1	160262259	160262659	-0.640912404	0.012530855	decrease
chr16	81314557	81314957	-0.840003109	0.0125459	decrease
chr8	75324330	75324730	-0.819354136	0.012549115	decrease
chr19	35740592	35740992	-0.570948232	0.012553667	decrease
chr19	1062931	1063331	-0.738035122	0.012559958	decrease
chr10	55899740	55900140	-0.841271993	0.012567177	decrease
chr15	90265336	90265736	-0.660464255	0.012570273	decrease
chr5	98773148	98773548	-0.708215258	0.012612008	decrease
chr7	123748914	123749314	-0.770678303	0.012624606	decrease
chr11	126079495	126079895	-0.8440122	0.012627474	decrease
chr3	119463559	119463959	-0.722464449	0.012662107	decrease
chr5	88516201	88516601	-0.758218262	0.012667222	decrease
chr2	210170449	210170849	-0.772387914	0.012667335	decrease
chr15	76147902	76148302	-0.817403657	0.012668434	decrease
chr9	100427499	100427899	-0.84217776	0.012675305	decrease
chr2	65350697	65351097	-0.875045601	0.012678751	decrease
chr11	34916360	34916760	-0.755043669	0.012680144	decrease
chr9	95875635	95876035	-0.676898862	0.012712436	decrease
chr6	108558663	108559063	-0.70892457	0.012713066	decrease
chr13	67230270	67230670	-0.573789088	0.012726932	decrease
chr7	128455500	128456120	-0.872443657	0.012734852	decrease
chr11	125887390	125887790	-0.845491906	0.012742621	decrease
chr11	76784109	76784509	-0.791897534	0.012748083	decrease
chr16	30814103	30814503	-0.71786679	0.012751656	decrease
chr2	218287036	218287436	-0.833692657	0.012753299	decrease
chrX	54358361	54358761	-0.725312948	0.01275707	decrease
chr1	178542926	178543326	-0.818591594	0.01276996	decrease
chr5	141879006	141879406	-0.739173688	0.012772824	decrease
chr6	99425348	99425748	-0.668617364	0.012776421	decrease
chr1	10033009	10033409	-0.736287663	0.012779842	decrease
chr11	116772712	116773112	-0.831705649	0.012790571	decrease
chr4	122152596	122152996	-0.841449747	0.012791793	decrease
chr4	141133833	141134233	-0.79560539	0.012794434	decrease
chrX	104166010	104166410	-0.726836558	0.012795797	decrease
chr17	21126780	21127180	-0.723416858	0.012795987	decrease
chr15	78565076	78565476	-0.75311728	0.012802413	decrease
chr11	61581489	61581889	-0.742182323	0.012808318	decrease
chr1	56531478	56531878	-0.696766456	0.012808394	decrease
chr12	123383808	123384208	-0.690775763	0.012810599	decrease
chr5	140043368	140043768	-0.752920848	0.012813891	decrease
chrX	119606185	119606585	-0.781991764	0.012817229	decrease
chr3	101573761	101574161	-0.694561431	0.012821905	decrease
chr12	4908768	4909168	-0.865282298	0.012829133	decrease
chr3	129439967	129440367	-0.66297839	0.012839209	decrease
chr14	105480816	105481216	-0.689852388	0.012839557	decrease
chr17	40287563	40287963	-0.654369959	0.0128465	decrease
chr2	177597747	177598147	-0.791025308	0.012851935	decrease
chr1	205255986	205256386	-0.804222178	0.012857589	decrease
chr7	65750650	65751050	-0.673820901	0.01285991	decrease
chr10	102646099	102646499	-0.779066992	0.012860616	decrease
chr15	90249128	90249855	-0.774906563	0.012869395	decrease
chr10	102714037	102714437	-0.586347396	0.012886072	decrease
chr5	92552552	92552952	-0.837429715	0.012889086	decrease

chr1	168439576	168439976	-0.84494845	0.0128943	decrease
chr2	144448858	144449258	-0.851580568	0.012894405	decrease
chr17	50867084	50867484	-0.664712224	0.01292779	decrease
chr1	52404281	52404681	-0.658451676	0.012930629	decrease
chr7	92590033	92590433	-0.731724	0.012978214	decrease
chr19	39498231	39498631	-0.797145999	0.012996034	decrease
chr19	52269252	52269652	-0.829359603	0.012996273	decrease
chrX	54044467	54044867	-0.688423563	0.012998657	decrease
chr1	114780410	114780810	-0.695507889	0.013004188	decrease
chr6	116253809	116254209	-0.741734312	0.013012234	decrease
chr12	53508923	53509323	-0.83652489	0.013026708	decrease
chr4	109302810	109303210	-0.836823329	0.013033802	decrease
chr12	104457542	104457942	-0.8682205	0.013034923	decrease
chr17	81241535	81241935	-0.716491306	0.013038481	decrease
chr4	146637080	146637480	-0.84223288	0.013042841	decrease
chr21	25639694	25640094	-0.838989586	0.013067131	decrease
chr12	120528965	120529365	-0.789943865	0.01307255	decrease
chr12	94459737	94460137	-0.773006663	0.013075514	decrease
chr2	55617714	55618114	-0.740191449	0.013082288	decrease
chr6	27807768	27808168	-0.712899435	0.01309096	decrease
chr8	140619222	140619622	-0.802169013	0.01309258	decrease
chr1	54887351	54887751	-0.63669812	0.013095763	decrease
chr1	46198491	46198891	-0.856031698	0.013099285	decrease
chr1	42658032	42658432	-0.663108541	0.013099563	decrease
chr19	56404086	56404486	-0.76870238	0.013131848	decrease
chr11	6604169	6604569	-0.76077907	0.013132282	decrease
chr11	17730721	17731121	-0.695436914	0.013139431	decrease
chr8	141001402	141001802	-0.843832415	0.013151614	decrease
chr3	136250312	136250712	-0.626220581	0.013166077	decrease
chr10	21525593	21525993	-0.74042565	0.013173763	decrease
chr16	85011525	85011925	-0.810958797	0.013174476	decrease
chr15	74695893	74696293	-0.740704911	0.013215675	decrease
chrX	38327211	38327611	-0.852496635	0.013225296	decrease
chr4	177309753	177310153	-0.782750723	0.013232072	decrease
chr1	93814186	93814586	-0.844504948	0.013241372	decrease
chr19	12965456	12965856	-0.766128564	0.013249134	decrease
chr2	177254545	177254945	-0.809895365	0.013252942	decrease
chr3	9986583	9986983	-0.705358857	0.013258231	decrease
chr1	163321735	163322135	-0.736613865	0.01327255	decrease
chr12	57132042	57132442	-0.829252301	0.013274169	decrease
chr1	147084629	147085029	-0.625546117	0.013277192	decrease
chr1	226083403	226083803	-0.68874167	0.01330674	decrease
chr11	19240803	19241203	-0.79818792	0.013329612	decrease
chr2	169733391	169733791	-0.829855511	0.013336628	decrease
chr2	130337226	130337626	-0.719024289	0.013339372	decrease
chr9	113275174	113275574	-0.650849863	0.013345012	decrease
chr5	176447984	176448384	-0.615062736	0.013349219	decrease
chr1	65467491	65467891	-0.795822928	0.013368301	decrease
chr5	109688963	109689363	-0.84599598	0.013390057	decrease
chr4	150579062	150579462	-0.819995769	0.01341154	decrease
chr3	112640993	112641393	-0.805528307	0.01341385	decrease
chr13	62105941	62106341	0.765170389	0.013421153	increase
chr15	79311181	79311581	-0.810122689	0.013428061	decrease
chr8	117520640	117521040	-0.798894315	0.013459377	decrease
chr2	235666370	235666770	-0.671537031	0.013460686	decrease
chr10	68074872	68075272	-0.628222961	0.013479294	decrease
chr1	217696113	217696513	-0.857534871	0.013492345	decrease
chr17	80133166	80133566	-0.792469082	0.013523444	decrease
chr19	34501698	34502098	-0.79872061	0.013528599	decrease
chr14	96501949	96502349	-0.721707993	0.013539606	decrease
chr5	164327268	164327668	-0.72353244	0.013543715	decrease
chr12	120228364	120228764	-0.630198938	0.013543891	decrease
chr3	61250999	61251399	-0.767919645	0.013547595	decrease
chr12	71663894	71664294	-0.637164377	0.013553557	decrease
chr17	56960740	56961140	-0.843305546	0.013555451	decrease
chr1	212857684	212858084	-0.642043875	0.013556145	decrease
chr17	69327114	69327514	-0.824483704	0.013602991	decrease
chr14	50829216	50829616	-0.830355178	0.01360387	decrease
chr2	241355287	241355687	-0.62635907	0.013618516	decrease
chr1	234599661	234600061	-0.766903383	0.01364087	decrease
chr13	23579166	23579566	-0.816903121	0.013647601	decrease
chr13	99501181	99501581	-0.727847116	0.013661096	decrease
chr4	52051676	52052076	-0.808645455	0.013672235	decrease
chr19	43192088	43192488	-0.616906873	0.013680158	decrease
chr6	44151805	44152205	-0.777285507	0.013706528	decrease
chr12	15788665	15789065	-0.840026719	0.013718491	decrease

chr19	550054	550454	-0.84297435	0.013721676	decrease
chr6	27752310	27752710	-0.767647267	0.013723514	decrease
chr4	6986685	6987085	-0.831152543	0.013739043	decrease
chr12	69739663	69740063	-0.83971497	0.013741167	decrease
chr6	27553123	27553523	-0.684408998	0.013757987	decrease
chr5	169980486	169980886	-0.828020348	0.013769446	decrease
chr6	5261111	5261511	-0.739053568	0.01377434	decrease
chr12	56360074	56360474	-0.682638287	0.013777352	decrease
chr19	49590100	49590500	-0.629216651	0.013816791	decrease
chr2	108786396	108786796	-0.726110786	0.013817941	decrease
chr13	48037759	48038159	-0.738189399	0.013820697	decrease
chr20	2652028	2652428	-0.843797445	0.013834285	decrease
chr16	13919788	13920188	-0.766411572	0.013843482	decrease
chr2	60530785	60531185	-0.813329296	0.013849316	decrease
chr7	24979103	24979503	-0.853264246	0.013855976	decrease
chr2	175594051	175594451	-0.820234234	0.013859616	decrease
chr15	80060490	80060890	-0.795071425	0.013862729	decrease
chr1	117121861	117122261	-0.673044252	0.01386361	decrease
chr6	149545566	149545966	-0.756118412	0.013863898	decrease
chr1	174799403	174799803	-0.822187485	0.013877171	decrease
chr3	98732152	98732552	-0.826745572	0.01388826	decrease
chr7	56064075	56064475	-0.766378764	0.013888638	decrease
chr15	65832836	65833236	-0.845096423	0.013900768	decrease
chr6	115485386	115485786	-0.853497673	0.013920318	decrease
chr7	5781574	5781974	-0.815406933	0.013944609	decrease
chr6	144216295	144216695	-0.69678511	0.013953431	decrease
chr5	124748611	124749011	-0.773716118	0.01396335	decrease
chr4	139082954	139083354	-0.705218458	0.013980559	decrease
chr6	52420181	52420581	-0.687477028	0.013982316	decrease
chr19	19776299	19776699	-0.617383753	0.013990157	decrease
chr16	75445556	75445956	-0.841240517	0.013991792	decrease
chr5	52787828	52788228	-0.830099092	0.014012279	decrease
chr6	89638491	89638891	-0.675649102	0.014020381	decrease
chr15	78507257	78507657	-0.64339357	0.014020589	decrease
chr16	23641336	23641736	-0.750444172	0.014033743	decrease
chr12	21657757	21658157	-0.704893281	0.014056645	decrease
chr14	101761136	101761536	-0.764760777	0.014063452	decrease
chr1	220786250	220786650	-0.689609749	0.01407039	decrease
chr17	77959825	77960225	-0.819202724	0.014109793	decrease
chr9	92634787	92635187	-0.77890571	0.014120708	decrease
chr14	70809733	70810133	-0.661040308	0.014125308	decrease
chr18	63367401	63367801	-0.711030072	0.014138272	decrease
chr2	152175508	152175908	-0.58538036	0.014144339	decrease
chr6	42050468	42050868	-0.765638297	0.014148036	decrease
chr3	89107439	89107839	-0.798220845	0.014154526	decrease
chr13	63566188	63566588	0.794959455	0.014155611	increase
chr11	18105892	18106292	-0.837258388	0.014163215	decrease
chr12	751469	751869	-0.703024086	0.014175641	decrease
chr1	43974485	43974885	-0.714316805	0.014196306	decrease
chr11	62556357	62556757	-0.75728222	0.01420419	decrease
chr2	54330546	54330946	-0.754003375	0.014211825	decrease
chr3	165195756	165196156	-0.835410336	0.014213946	decrease
chr7	128039525	128039925	-0.832958789	0.01422086	decrease
chr5	168579605	168580005	-0.740258123	0.014235703	decrease
chr11	85685984	85686384	-0.670067287	0.014238424	decrease
chr4	88592048	88592448	-0.804369223	0.014245821	decrease
chr9	104968282	104968682	-0.750181426	0.014248588	decrease
chr1	149888190	149888590	-0.657957892	0.014253072	decrease
chr8	144787464	144787864	-0.647693407	0.01426154	decrease
chr11	134050354	134050754	-0.842432505	0.014272513	decrease
chr20	2524861	2525261	-0.840930469	0.014293739	decrease
chr9	128881668	128882068	-0.742570689	0.014294909	decrease
chr5	140564387	140564787	-0.699699625	0.014322103	decrease
chrX	15674948	15675348	-0.700491885	0.014332918	decrease
chr12	84911909	84912309	-0.766296235	0.014335285	decrease
chr1	166876276	166876676	-0.781719106	0.014362829	decrease
chr6	34462530	34462930	-0.807270399	0.014369536	decrease
chr13	42271475	42271875	-0.730135079	0.014371025	decrease
chr9	128697132	128697532	-0.825647685	0.014377413	decrease
chr19	15432733	15433133	-0.820133901	0.014395875	decrease
chr10	101830419	101830819	-0.660239719	0.014398907	decrease
chr11	6683143	6683543	-0.781662434	0.01440143	decrease
chrX	47233375	47233775	-0.791303537	0.014410206	decrease
chr6	19836863	19837263	-0.811215329	0.01441137	decrease
chr14	81221085	81221485	-0.745302765	0.014420563	decrease
chr9	35749241	35749641	-0.824461629	0.014420632	decrease

chr4	123396756	123397156	-0.792257094	0.014458368	decrease
chr22	19762042	19762442	-0.758968581	0.014469701	decrease
chr17	81040692	81041092	-0.771414448	0.014475553	decrease
chr3	184134286	184134686	-0.795683933	0.014485405	decrease
chr2	53970007	53970407	-0.820580742	0.014489862	decrease
chr18	34516340	34516740	-0.843444588	0.014498878	decrease
chr6	47168178	47168578	-0.838132344	0.014500455	decrease
chr7	139230852	139231252	-0.650110907	0.01450605	decrease
chr6	96837193	96837593	-0.824459626	0.014507682	decrease
chr13	90867134	90867534	-0.848603872	0.014508651	decrease
chr10	3172946	3173346	-0.749093997	0.014519197	decrease
chr19	46697406	46697806	-0.730187078	0.014525004	decrease
chr4	84499360	84499760	-0.741690258	0.014542424	decrease
chr11	118689872	118690272	-0.724975065	0.01456493	decrease
chr1	9910471	9910871	-0.78902845	0.014628811	decrease
chr19	14006432	14006832	-0.686722574	0.014631543	decrease
chr5	59021178	59021578	-0.830223557	0.014640701	decrease
chrX	78103868	78104268	-0.579520929	0.014642475	decrease
chr1	148038514	148038914	-0.634500368	0.014645429	decrease
chr12	95473803	95474203	-0.656300341	0.014666879	decrease
chr15	78872852	78873252	-0.804399911	0.014675488	decrease
chr6	10403840	10404240	-0.791568186	0.014703596	decrease
chr9	124856238	124856638	-0.629883754	0.014712959	decrease
chr6	19805235	19805635	-0.842094252	0.014718895	decrease
chr2	65991373	65991773	-0.811482222	0.014723227	decrease
chr22	37696692	37697092	-0.654268836	0.01472415	decrease
chr1	226309970	226310370	-0.757637647	0.014728608	decrease
chr7	100866978	100867378	-0.716977444	0.014730454	decrease
chr6	11043162	11043562	-0.852689465	0.01473658	decrease
chr6	13395432	13395832	-0.796276647	0.014741836	decrease
chr11	59566040	59566440	-0.728947769	0.014746577	decrease
chr1	155857100	155857500	-0.681098166	0.014754897	decrease
chr6	154510714	154511114	-0.754235205	0.014762497	decrease
chr1	1375279	1375679	-0.763001677	0.014771266	decrease
chr7	86643752	86644152	-0.824609596	0.014787229	decrease
chr1	84506280	84506680	-0.649254518	0.01480983	decrease
chr9	104926765	104927165	-0.822802551	0.014819975	decrease
chr19	45717298	45717698	-0.709806824	0.014829209	decrease
chr1	52927577	52927977	-0.837810441	0.014829909	decrease
chr17	32138741	32139141	-0.850286156	0.014839762	decrease
chr2	10097319	10097719	-0.813321129	0.01487754	decrease
chr5	40755696	40756096	-0.734728151	0.014897366	decrease
chr12	57752279	57752679	-0.592989282	0.014903607	decrease
chr7	4775337	4775737	-0.760806985	0.014911934	decrease
chr18	26226320	26226720	-0.780100118	0.014948413	decrease
chr12	56266767	56267167	-0.772547323	0.014951325	decrease
chr8	110798506	110798906	-0.801070994	0.014960122	decrease
chr3	48685893	48686293	-0.726293113	0.01497938	decrease
chr4	77862804	77863204	-0.663163678	0.014987237	decrease
chr19	46600660	46601060	-0.678998804	0.015005383	decrease
chr17	45052219	45052619	-0.813266856	0.015007843	decrease
chr11	16606071	16606471	-0.750889651	0.015008068	decrease
chr14	49598709	49599109	-0.647821249	0.015012608	decrease
chr11	107928478	107928878	-0.852220428	0.015018136	decrease
chr13	51583866	51584266	-0.612460942	0.01501837	decrease
chrX	48521466	48521866	-0.715136526	0.015042195	decrease
chr17	63741619	63742019	-0.642036719	0.01504807	decrease
chr18	36829114	36829514	-0.651961699	0.015063474	decrease
chr14	55026961	55027361	-0.737894244	0.015088757	decrease
chr17	15999345	15999745	-0.800798122	0.015111064	decrease
chr2	241315350	241315750	-0.626121946	0.015112728	decrease
chr3	47475947	47476347	-0.807785363	0.015117847	decrease
chr3	72887555	72887955	-0.842019974	0.015118373	decrease
chr5	172958780	172959180	-0.596879707	0.015124981	decrease
chr16	11797400	11797800	-0.763903388	0.015139402	decrease
chr13	46052550	46052950	-0.792127686	0.015139596	decrease
chr5	139273806	139274206	-0.74429467	0.01516116	decrease
chr8	104588070	104588470	-0.828032699	0.015161348	decrease
chr22	23751090	23751490	-0.829678725	0.01517768	decrease
chr3	155805870	155806270	-0.770814683	0.015192193	decrease
chr17	59565618	59566018	-0.807480921	0.015208168	decrease
chrX	120244911	120245311	-0.731278491	0.015209753	decrease
chr20	63864999	63865399	-0.740693252	0.015246924	decrease
chr6	12012921	12013321	-0.805056429	0.015255722	decrease
chr14	49688164	49688564	-0.801595665	0.015259097	decrease
chr9	131349990	131350390	-0.788405736	0.015262752	decrease

chr11	15074771	15075171	-0.727520035	0.015273082	decrease
chr10	79336444	79336844	-0.810892378	0.015285618	decrease
chr5	78359900	78360300	-0.601250611	0.015287765	decrease
chr2	86807343	86807743	-0.780869149	0.015289016	decrease
chr2	33599136	33599536	-0.617163917	0.015324942	decrease
chr7	101244667	101245067	-0.596756532	0.015336354	decrease
chr10	119725508	119725908	-0.783708087	0.015359701	decrease
chr1	186680333	186680733	-0.721443846	0.015362137	decrease
chr17	76709266	76709666	-0.586356529	0.015364961	decrease
chr9	121650106	121650506	-0.610098895	0.015378342	decrease
chr12	53079398	53079798	-0.788263657	0.015387785	decrease
chr19	49659678	49660078	-0.664597167	0.015407627	decrease
chr1	58546611	58547011	-0.731679526	0.015409518	decrease
chr7	77877994	77878394	-0.809475127	0.015418079	decrease
chr10	50625147	50625547	-0.743344966	0.015419965	decrease
chr19	7197624	7198024	-0.710059248	0.015426505	decrease
chr22	28741890	28742290	-0.654697475	0.01543297	decrease
chr2	19901614	19902014	-0.680635219	0.015443882	decrease
chr9	92325569	92325969	-0.706669628	0.015446445	decrease
chr22	40651739	40652139	-0.670123949	0.015453526	decrease
chr1	185316034	185316434	-0.734184338	0.015458033	decrease
chr5	150544655	150545055	-0.75556873	0.015491392	decrease
chr7	7968408	7968808	-0.777721591	0.015524597	decrease
chr2	184599505	184599905	-0.791818113	0.015533439	decrease
chrX	74614414	74614814	-0.735256895	0.015547505	decrease
chr16	55509103	55509503	-0.833458608	0.015549034	decrease
chr14	50000599	50000999	-0.831186734	0.015553509	decrease
chr5	173235046	173235446	-0.724838891	0.015575328	decrease
chr1	161749912	161750312	-0.661337132	0.015576614	decrease
chr13	52617226	52617626	-0.70726259	0.015586412	decrease
chr5	140107316	140107716	-0.782489314	0.015595674	decrease
chr11	119018418	119018818	-0.696466837	0.015595921	decrease
chr11	33258464	33258864	-0.813020561	0.01560766	decrease
chr4	88284596	88284996	-0.632606955	0.015630902	decrease
chr12	109477334	109477734	-0.705386288	0.015634938	decrease
chr7	77536611	77537011	-0.829017571	0.015638273	decrease
chr11	36594410	36594810	-0.769142091	0.01563956	decrease
chr10	79381912	79382312	-0.771038708	0.015639809	decrease
chr17	68035482	68035882	-0.700406979	0.015660957	decrease
chr10	13161274	13161674	-0.786340923	0.015677681	decrease
chr10	93496183	93496583	-0.761352789	0.015706401	decrease
chr4	88823315	88823715	-0.738970783	0.015710891	decrease
chr6	124076448	124076848	-0.792356586	0.015732103	decrease
chrX	120468907	120469307	-0.692111092	0.015735626	decrease
chr5	84435843	84436243	-0.748404526	0.015740005	decrease
chr5	60699805	60700205	-0.791167406	0.015755684	decrease
chr12	56645953	56646353	-0.64327859	0.015801574	decrease
chr9	83965452	83965852	-0.770607377	0.015804621	decrease
chr1	223846151	223846551	-0.648911062	0.015813547	decrease
chr10	89701269	89701669	-0.666206617	0.015821904	decrease
chr2	239401294	239401694	-0.684302331	0.015858989	decrease
chr2	3605327	3605727	-0.780795065	0.0158723	decrease
chrX	48891529	48891929	-0.798778038	0.015879141	decrease
chr1	64965441	64965841	-0.754639521	0.015900928	decrease
chr1	156572411	156572811	-0.728290188	0.015909454	decrease
chr19	44954681	44955081	-0.661401963	0.015920338	decrease
chr10	90871615	90872015	-0.688477984	0.015930321	decrease
chr2	99336939	99337339	-0.690530137	0.015931145	decrease
chr15	75367911	75368311	-0.624765924	0.015946838	decrease
chr13	21603520	21603920	-0.847155459	0.015955446	decrease
chr7	139768029	139768429	-0.833667987	0.015957249	decrease
chr11	65110340	65110740	-0.599856955	0.015963713	decrease
chr5	157948926	157949326	-0.828329301	0.015971731	decrease
chr8	37479049	37479449	-0.697463405	0.015982296	decrease
chr3	125595431	125595831	-0.760691783	0.015984181	decrease
chr1	178726227	178726627	-0.750153595	0.015993427	decrease
chr3	31980446	31980846	-0.824725076	0.016011492	decrease
chr4	169757635	169758035	-0.805809411	0.016034874	decrease
chr18	31206854	31207254	-0.738960419	0.016074692	decrease
chr6	116668399	116668799	-0.667985011	0.016078587	decrease
chr1	65264353	65264753	-0.62182516	0.016085715	decrease
chr19	13952055	13952455	-0.703017231	0.016092261	decrease
chr17	42578061	42578461	-0.743834775	0.016103628	decrease
chr15	76336264	76336664	-0.71335789	0.016106384	decrease
chr2	156254688	156255088	-0.833443543	0.016111669	decrease
chr10	74150798	74151198	-0.5491487	0.01612347	decrease

chr19	48713981	48714381	-0.714693756	0.0161331	decrease
chr7	151123928	151124328	-0.683961578	0.016158327	decrease
chr7	100429157	100429557	-0.613264395	0.016167539	decrease
chr10	97687101	97687501	-0.696824596	0.016179948	decrease
chr3	129161396	129161796	-0.705743173	0.016191804	decrease
chr7	135557650	135558050	-0.565883998	0.016194084	decrease
chr1	35769168	35769568	-0.835702593	0.016196917	decrease
chr7	139359594	139359994	-0.566508955	0.016215143	decrease
chr22	38215358	38215758	-0.726353156	0.016231016	decrease
chr7	155297073	155297473	-0.724508558	0.016235093	decrease
chr1	202967373	202967773	-0.805268504	0.016240931	decrease
chr3	13078893	13079293	-0.831176692	0.016271071	decrease
chr11	83193523	83193923	-0.73739954	0.016276712	decrease
chrX	155026696	155027096	-0.752753772	0.016278433	decrease
chr16	8868695	8869095	-0.768769315	0.016294957	decrease
chr11	9385158	9385558	-0.79674625	0.016302864	decrease
chr12	122400163	122400563	-0.789480144	0.01630743	decrease
chr19	3971308	3971708	-0.532612714	0.01631941	decrease
chr14	20413109	20413509	-0.674733192	0.016323986	decrease
chr12	57831491	57831891	-0.840399402	0.016324918	decrease
chr17	14069246	14069646	-0.764799491	0.016330752	decrease
chr1	224356786	224357186	-0.712156465	0.016348596	decrease
chr5	108748629	108749029	-0.765145377	0.016352969	decrease
chr17	73311701	73312101	-0.761014622	0.016393409	decrease
chr19	49500395	49500795	-0.6425934	0.016396203	decrease
chr7	101252042	101252442	-0.724914881	0.016406139	decrease
chr8	98944857	98945257	-0.819388243	0.016409988	decrease
chr6	167351186	167351586	-0.739914014	0.016426646	decrease
chr19	3210669	3211069	-0.724822221	0.016428977	decrease
chr19	55216474	55216874	-0.609725849	0.016459024	decrease
chr16	89157482	89157882	0.821169127	0.016473104	increase
chr19	44071902	44072302	-0.800965749	0.016473546	decrease
chr5	150161311	150161711	-0.668760966	0.01649214	decrease
chr1	88992726	88993126	-0.629518805	0.016523397	decrease
chr20	38731136	38731536	-0.718121223	0.016523664	decrease
chr19	40541319	40541719	-0.805079916	0.016540184	decrease
chr2	111121939	111122339	-0.788011037	0.016551057	decrease
chr3	32502386	32502786	-0.790220181	0.016555607	decrease
chr21	15735474	15735874	-0.807363941	0.016564989	decrease
chr11	66546117	66546517	-0.77565551	0.016583055	decrease
chrX	52921309	52921709	-0.797229712	0.016603085	decrease
chr5	131170801	131171201	-0.648893782	0.016618527	decrease
chr14	77707891	77708291	-0.760984297	0.016627669	decrease
chr17	60391005	60391405	-0.767282869	0.016628794	decrease
chr20	41693181	41693581	-0.758237368	0.016636147	decrease
chr1	28914133	28914533	-0.70479803	0.016653509	decrease
chr13	33818223	33818623	-0.744652013	0.016661616	decrease
chr19	50724513	50724913	-0.647406485	0.016672421	decrease
chr18	11849853	11850253	-0.830871878	0.016684031	decrease
chr2	184407140	184407540	-0.77947418	0.016689996	decrease
chr15	98580555	98580955	-0.820780562	0.016701326	decrease
chr11	45917849	45918249	-0.673187349	0.016701854	decrease
chr20	23350345	23350745	-0.72477049	0.016719221	decrease
chr12	49060713	49061113	-0.725189745	0.016733044	decrease
chr14	34873432	34873832	-0.735357919	0.016735606	decrease
chr17	78815765	78816165	-0.781507677	0.016748128	decrease
chr3	27369230	27369630	-0.784564799	0.016755231	decrease
chr1	50960366	50960766	-0.765249087	0.016770102	decrease
chr1	207053012	207053412	-0.635179731	0.016774752	decrease
chr5	139439363	139439763	-0.75662891	0.01677567	decrease
chr10	99520081	99520481	-0.708470852	0.016804637	decrease
chr17	61467438	61467838	-0.759204501	0.016807701	decrease
chr15	50353821	50354221	-0.826621801	0.016822716	decrease
chr1	100249716	100250116	-0.673183552	0.016824945	decrease
chr9	101398298	101398698	-0.724567835	0.016848419	decrease
chr17	18314971	18315371	-0.729157122	0.016870412	decrease
chr2	96763483	96764211	-0.681573506	0.016876538	decrease
chr7	12403707	12404107	-0.714127141	0.016880128	decrease
chr7	128387715	128388115	-0.746268495	0.016888629	decrease
chr17	51165892	51166292	-0.714843884	0.016898394	decrease
chr14	77760912	77761312	-0.690968833	0.016912499	decrease
chr22	30667641	30668041	-0.695168031	0.016914656	decrease
chr4	144646507	144646907	-0.74273666	0.016933055	decrease
chr14	60161500	60161900	-0.823609723	0.016940743	decrease
chr2	55223754	55224154	-0.826861712	0.016958347	decrease
chr1	99645942	99646342	-0.77820422	0.016993502	decrease

chr7	97016514	97016914	-0.728047622	0.016994866	decrease
chr1	145964346	145964746	-0.762667913	0.016994968	decrease
chr17	67717731	67718131	-0.638495914	0.017014849	decrease
chr5	39074593	39074993	-0.785595382	0.017027017	decrease
chr7	120857834	120858234	-0.785058209	0.017031544	decrease
chr19	10572515	10572915	-0.805474041	0.017043632	decrease
chr1	153627368	153627768	-0.627401267	0.017045241	decrease
chr1	220879000	220879400	-0.734634094	0.017058467	decrease
chr12	92929380	92929780	-0.763342235	0.017070385	decrease
chr2	31233842	31234242	-0.765664942	0.017073025	decrease
chr10	73168346	73168746	-0.831940638	0.017078446	decrease
chr3	115146021	115146421	-0.82398862	0.017086191	decrease
chr11	47161149	47161549	-0.701205671	0.017088009	decrease
chr1	100037857	100038257	-0.743735937	0.017102477	decrease
chr1	37859559	37859959	-0.669894693	0.017116419	decrease
chr22	42582317	42582717	-0.747669927	0.017135136	decrease
chr7	90928739	90929139	-0.795435744	0.01713629	decrease
chr8	85220435	85220835	-0.635591458	0.017145464	decrease
chr15	90931807	90932207	-0.650991929	0.017161695	decrease
chr16	81096382	81096782	-0.718615297	0.017170377	decrease
chr12	120469839	120470239	-0.663396269	0.017185168	decrease
chr9	37800405	37800805	-0.782060336	0.017188962	decrease
chr11	111766339	111766739	-0.802512829	0.017202441	decrease
chr2	27212020	27212420	-0.749813421	0.017205904	decrease
chr4	77076056	77076456	-0.749314005	0.017231058	decrease
chr11	112025102	112025502	-0.599200876	0.01726071	decrease
chr9	131125151	131125551	-0.683486495	0.017289167	decrease
chr2	86563089	86563489	-0.63915597	0.017293351	decrease
chr6	27251737	27252137	-0.75980046	0.017302523	decrease
chr2	38875611	38876011	-0.685317349	0.017306083	decrease
chr11	32583477	32583877	-0.718256318	0.017307583	decrease
chr19	51339193	51339593	-0.710021685	0.017308902	decrease
chr3	4303209	4303609	-0.787304498	0.01730983	decrease
chrX	3814861	3815261	-0.712379474	0.017335239	decrease
chr5	127517584	127517984	-0.758210093	0.01733789	decrease
chr14	102510850	102511250	-0.760161555	0.017345126	decrease
chr5	102153460	102153860	-0.814893144	0.017380825	decrease
chr6	1622897	1623297	-0.761166857	0.017385004	decrease
chr21	32278800	32279200	-0.72145489	0.017386107	decrease
chr16	89739188	89739588	-0.717816214	0.017393834	decrease
chr4	81470570	81470970	-0.777224512	0.01739981	decrease
chr2	135985287	135985687	-0.612331237	0.01741914	decrease
chr2	206085910	206086310	-0.75265901	0.017425595	decrease
chr16	75517279	75517679	-0.745200599	0.017442989	decrease
chr2	215435498	215435898	-0.83235064	0.017450047	decrease
chr10	101012987	101013387	-0.702611871	0.017455925	decrease
chr12	76764192	76764592	-0.75978087	0.017463312	decrease
chr18	46173633	46174033	-0.788947222	0.017479149	decrease
chr7	65252116	65252516	-0.726484179	0.017481114	decrease
chr2	176093169	176093569	-0.791183463	0.017486516	decrease
chr12	6556000	6556400	-0.682390367	0.017489778	decrease
chr12	14365603	14366003	-0.773606176	0.017490093	decrease
chr3	134485826	134486226	-0.648231037	0.0174957	decrease
chr22	21867711	21868111	-0.769653337	0.017520758	decrease
chr1	220747904	220748304	-0.70829703	0.017538025	decrease
chr11	113773633	113774033	-0.638681069	0.017543773	decrease
chr13	46796763	46797163	-0.701588391	0.017576428	decrease
chr6	28335550	28335950	-0.639832124	0.017577832	decrease
chr15	90300986	90301386	-0.793006694	0.017594094	decrease
chr5	163505429	163505829	-0.711789253	0.017595101	decrease
chr7	93231941	93232341	-0.646002315	0.017598475	decrease
chr11	66258100	66258500	-0.684513268	0.017598892	decrease
chrX	71533036	71533436	-0.802095431	0.017621412	decrease
chr11	72812852	72813252	-0.668489703	0.017648802	decrease
chr4	73069585	73069985	-0.580212267	0.017667094	decrease
chr1	204411616	204412016	-0.67290943	0.017672965	decrease
chr17	50151114	50151514	-0.832252678	0.017678924	decrease
chr2	219552378	219552778	-0.701035723	0.017681592	decrease
chr15	78565881	78566281	-0.770981131	0.017691408	decrease
chrX	54999867	55000267	-0.729412229	0.017694819	decrease
chr2	42133040	42133440	-0.740905964	0.017698452	decrease
chr6	133952795	133953195	-0.677604184	0.017718795	decrease
chr4	184649882	184650282	-0.788585572	0.017740551	decrease
chr8	118112955	118113355	-0.762630262	0.017756415	decrease
chr2	175805770	175806170	-0.784149562	0.017761369	decrease
chr11	31509621	31510021	-0.709182411	0.01776779	decrease

chr15	98609698	98610098	-0.801972974	0.017804045	decrease
chr10	69124704	69125104	-0.770335606	0.017815157	decrease
chr11	10304859	10305259	-0.705758226	0.017821637	decrease
chr18	13726481	13726881	-0.807804331	0.017826359	decrease
chr2	213284211	213284611	-0.746968519	0.017828779	decrease
chr16	66961055	66961455	-0.740295026	0.017846159	decrease
chr7	876092	876492	-0.724761946	0.017849246	decrease
chr18	34659511	34659911	-0.801715943	0.017854865	decrease
chr8	55772864	55773264	-0.716363132	0.017875958	decrease
chr17	28335521	28335921	-0.622157027	0.017899817	decrease
chr19	7943697	7944097	-0.5773674	0.017936151	decrease
chr11	119030635	119031035	-0.736610867	0.017936871	decrease
chr8	24956285	24956685	-0.821675305	0.017939035	decrease
chr15	72272601	72273001	-0.705880392	0.017982622	decrease
chr1	47334361	47334761	-0.75774175	0.017993753	decrease
chr19	2900617	2901017	-0.676460017	0.018021598	decrease
chr8	80872620	80873020	-0.757762572	0.018034933	decrease
chr8	73089943	73090343	-0.716783808	0.018063891	decrease
chr13	74135924	74136324	-0.718829733	0.018074868	decrease
chr17	76726414	76726814	-0.731992871	0.018083912	decrease
chr19	45863097	45863497	-0.61647752	0.018092336	decrease
chr3	50235815	50236215	-0.687007109	0.01810065	decrease
chr22	21643206	21643606	-0.719487421	0.01812987	decrease
chr9	125261358	125261758	-0.771002613	0.018147152	decrease
chr2	199955366	199955766	-0.671325826	0.018170211	decrease
chr11	117907238	117907638	-0.772787727	0.018172772	decrease
chr1	44213491	44213891	-0.759517552	0.01817919	decrease
chr6	27893133	27893533	-0.676470779	0.018205471	decrease
chr12	49945372	49945772	-0.799236008	0.018222858	decrease
chr1	26890123	26890523	-0.777970115	0.018231067	decrease
chr5	124902261	124902661	-0.829455722	0.018237487	decrease
chr5	80407892	80408292	-0.649947884	0.018288416	decrease
chr21	46323572	46323972	-0.725854564	0.018302127	decrease
chr2	20351054	20351454	-0.710531797	0.018306322	decrease
chr17	35587223	35587623	-0.758746081	0.018309692	decrease
chr12	133130025	133130425	-0.799211082	0.018312061	decrease
chr10	103564774	103565174	-0.795232615	0.018341464	decrease
chr20	5950245	5950645	-0.74020125	0.018343134	decrease
chr10	88880097	88880497	-0.802428923	0.018348285	decrease
chr5	56950757	56951157	-0.815412893	0.01835847	decrease
chr7	112450288	112450688	-0.607944572	0.018368585	decrease
chr14	39170053	39170453	-0.616238616	0.018371046	decrease
chr17	41786716	41787116	-0.709645431	0.018385861	decrease
chr1	222589825	222590225	-0.7972475	0.018429163	decrease
chr6	27693877	27694277	-0.795415968	0.01843373	decrease
chr5	178120765	178121165	-0.706798566	0.018460199	decrease
chr19	43618970	43619370	-0.794698943	0.018470453	decrease
chr3	160210766	160211166	-0.815496467	0.018486035	decrease
chr1	113759522	113759922	-0.785434214	0.018487105	decrease
chr1	153545058	153545458	-0.729566768	0.018510128	decrease
chr13	20902315	20902715	-0.828342762	0.018517367	decrease
chr15	89690438	89690838	-0.726913873	0.01855613	decrease
chr6	43076986	43077386	-0.769458038	0.018565248	decrease
chr9	129006621	129007021	-0.671016287	0.018567823	decrease
chr7	66628365	66628765	-0.718454538	0.018570797	decrease
chr5	104793719	104794119	-0.789941119	0.018574452	decrease
chr14	49586404	49586804	-0.588615065	0.018586182	decrease
chr15	49155462	49155862	-0.760845802	0.018594393	decrease
chr1	155206994	155207394	-0.706622316	0.018616717	decrease
chr17	21214267	21214667	-0.715297962	0.01865403	decrease
chr6	21953915	21954315	-0.717354328	0.018659355	decrease
chr11	18394334	18394734	-0.713859054	0.018662679	decrease
chr5	150357417	150357817	-0.735704825	0.018683301	decrease
chr1	224330053	224330453	-0.48130711	0.018704046	decrease
chr1	164612018	164612418	-0.775744147	0.018737915	decrease
chr10	46023172	46023572	-0.745581043	0.018749985	decrease
chr2	159615909	159616309	-0.800243598	0.018765991	decrease
chr20	833649	834049	-0.774294689	0.01877109	decrease
chr12	57693730	57694130	-0.634956814	0.018780535	decrease
chr22	17628687	17629087	-0.648647704	0.018787902	decrease
chr12	54359018	54359418	-0.757410201	0.018806109	decrease
chr5	134873843	134874243	-0.815091557	0.018810543	decrease
chr12	71438880	71439280	-0.815335795	0.018842367	decrease
chr3	194136925	194137325	-0.749721768	0.01885549	decrease
chr3	9649100	9649500	-0.797546901	0.018856281	decrease
chr2	264840	265240	-0.813607768	0.018862434	decrease

chr11	72793680	72794080	-0.718268766	0.018925483	decrease
chr6	70566661	70567061	-0.762476861	0.018930371	decrease
chr5	97183306	97183706	-0.635324251	0.01894548	decrease
chr9	71769249	71769649	-0.732274364	0.018948203	decrease
chr18	34630850	34631250	-0.733712137	0.01896909	decrease
chr1	76868181	76868581	-0.725989041	0.018971458	decrease
chr17	48996365	48996765	-0.616945694	0.018973232	decrease
chr19	11639801	11640201	-0.668963267	0.018989753	decrease
chr4	98928843	98929243	-0.63246589	0.019000169	decrease
chr19	58451416	58451816	-0.751309837	0.019028066	decrease
chr16	29926136	29926536	-0.720850084	0.019063089	decrease
chr3	119498328	119498728	-0.705812709	0.019064318	decrease
chr6	36885716	36886116	-0.740100133	0.019069031	decrease
chr19	2944985	2945385	-0.695140034	0.019097052	decrease
chr17	40417854	40418254	-0.64958125	0.019111567	decrease
chr15	34224866	34225266	-0.791962363	0.019112614	decrease
chr5	159207968	159208368	-0.760605271	0.019121525	decrease
chr4	683783	684183	-0.587704675	0.019127481	decrease
chr4	77820121	77820521	-0.737842684	0.01912774	decrease
chr12	124567732	124568132	-0.796251716	0.019154814	decrease
chr1	6624828	6625228	-0.811428697	0.019165843	decrease
chr1	203861301	203861701	-0.657240705	0.019202945	decrease
chr7	143263328	143263728	-0.710595033	0.019209478	decrease
chr3	129121861	129122261	-0.773706174	0.019216492	decrease
chr2	101308737	101309137	-0.78875675	0.019256709	decrease
chrX	107775586	107775986	-0.72566203	0.019271156	decrease
chr3	128680640	128681040	-0.721447123	0.019293311	decrease
chr11	108664821	108665221	-0.729581507	0.019299572	decrease
chr7	92246481	92246881	-0.804109987	0.019303099	decrease
chr3	128617782	128618182	-0.646876325	0.019318253	decrease
chr19	56567175	56567575	-0.801326715	0.019319757	decrease
chr1	156749172	156749572	-0.682297635	0.019326938	decrease
chr4	121823401	121823801	-0.661987551	0.019344706	decrease
chr21	29002539	29002939	-0.68016184	0.019359477	decrease
chr3	9749864	9750264	-0.595153782	0.019369608	decrease
chr5	95554814	95555214	-0.657891769	0.019380904	decrease
chr20	35699343	35699743	-0.729985212	0.019405142	decrease
chrX	152830717	152831117	-0.640941607	0.019406318	decrease
chr17	67366352	67366752	-0.618067565	0.019422683	decrease
chr11	67262832	67263232	-0.746625337	0.019458666	decrease
chr7	92528535	92528935	-0.713951679	0.019481655	decrease
chr2	162343949	162344349	-0.643885181	0.019487734	decrease
chr16	30409249	30409649	-0.685810685	0.019488333	decrease
chr11	67317440	67317840	-0.680075083	0.019499593	decrease
chr1	36386087	36386487	-0.659313766	0.019576268	decrease
chr5	88784872	88785272	-0.781012355	0.019589447	decrease
chr6	158168251	158168651	-0.721946289	0.019597713	decrease
chrX	70454418	70454818	-0.657735672	0.019600807	decrease
chr2	183124474	183124874	-0.640211978	0.019622718	decrease
chr12	96035294	96035694	-0.663189552	0.019622736	decrease
chr11	62380835	62381235	-0.792275779	0.019625581	decrease
chr18	12702738	12703138	-0.750290044	0.01962744	decrease
chrX	40013674	40014074	-0.774486725	0.019637839	decrease
chr5	140175667	140176067	-0.729226067	0.019652563	decrease
chr10	78303447	78303847	-0.803895758	0.019664222	decrease
chr1	154559014	154559414	-0.656117784	0.019665839	decrease
chr16	19086676	19087076	-0.802773436	0.019674398	decrease
chr17	41665725	41666125	-0.763171084	0.019680418	decrease
chr13	79503191	79503591	-0.788373329	0.019682364	decrease
chr21	44956966	44957366	-0.741921212	0.019684861	decrease
chr21	42009976	42010376	-0.732394639	0.01969742	decrease
chr2	190880528	190880928	-0.77572021	0.019715086	decrease
chr11	10792395	10792795	-0.698278641	0.019722497	decrease
chr1	161177488	161177888	-0.639807757	0.019746794	decrease
chr6	118651678	118652078	-0.811291014	0.019753819	decrease
chr6	27548351	27548751	-0.811252128	0.019803369	decrease
chr7	6447808	6448208	-0.617599942	0.019824695	decrease
chr19	12551432	12551832	-0.656040848	0.019848548	decrease
chr3	179322540	179322940	-0.780914873	0.019852203	decrease
chr2	130727403	130727803	-0.643115571	0.019867734	decrease
chr9	33922136	33922536	-0.739226281	0.019882141	decrease
chr9	34178722	34179122	-0.734085974	0.019883526	decrease
chr8	144019940	144020340	-0.786557358	0.019927554	decrease
chr3	72997359	72997759	-0.801234168	0.019946458	decrease
chr3	156825971	156826371	-0.693454509	0.019948763	decrease
chr1	111449344	111449744	-0.620190446	0.019964797	decrease

chr8	37359816	37360216	-0.756603855	0.019974951	decrease
chr14	67240972	67241372	-0.786550249	0.019990743	decrease
chr17	60525854	60526254	-0.695308372	0.019999636	decrease
chr2	68251200	68251600	-0.759936503	0.020001465	decrease
chr11	73876681	73877081	-0.650608221	0.020004228	decrease
chr10	74176488	74176888	-0.67551923	0.020006885	decrease
chr11	108593538	108593938	-0.746785725	0.020014485	decrease
chr6	87695942	87696342	-0.77416334	0.020055262	decrease
chr7	7182086	7182486	-0.740468952	0.020060845	decrease
chr19	45779506	45779906	-0.603709369	0.020064647	decrease
chr17	76567865	76568265	-0.634938275	0.020071992	decrease
chrX	152915533	152915933	-0.723052862	0.020079018	decrease
chr1	150487176	150487576	-0.677093635	0.020090314	decrease
chr2	218396269	218396669	-0.652837316	0.020114917	decrease
chr8	143974933	143975333	-0.718089153	0.020129209	decrease
chr19	10403556	10403956	-0.602089744	0.020167152	decrease
chr12	74537527	74537927	-0.729866361	0.02017568	decrease
chr16	89910201	89910601	-0.668874427	0.020183356	decrease
chr10	102420683	102421083	-0.60454995	0.020199886	decrease
chr19	50511167	50511567	-0.676726859	0.020220349	decrease
chr9	17268533	17268933	-0.76701393	0.020224112	decrease
chr3	42590502	42590902	-0.625651479	0.020235811	decrease
chr22	37661011	37661411	-0.61246951	0.0202384	decrease
chr1	46442453	46442853	-0.666491338	0.02024094	decrease
chr2	118855460	118855860	-0.803059893	0.020270971	decrease
chr5	34839031	34839431	-0.736125527	0.020298797	decrease
chr19	10194949	10195349	-0.653376273	0.020339072	decrease
chr3	28241407	28241807	-0.699841405	0.020352174	decrease
chr7	42912083	42912483	-0.787553472	0.020354824	decrease
chr12	106357706	106358106	-0.704896862	0.020384187	decrease
chr12	49265174	49265574	-0.719366306	0.020394721	decrease
chr19	6516427	6516827	-0.643659278	0.020399925	decrease
chr5	671658	672058	-0.641832972	0.020420387	decrease
chr13	48303369	48303769	-0.800435602	0.020434793	decrease
chr19	48720780	48721180	-0.654921124	0.020443565	decrease
chr9	130693696	130694096	-0.759755711	0.020450126	decrease
chr12	122031541	122031941	-0.70290284	0.020450926	decrease
chr3	66037337	66037737	-0.760296176	0.020453564	decrease
chr5	173328367	173328767	-0.605520592	0.02046169	decrease
chr12	6538378	6538778	-0.58207586	0.020482112	decrease
chr8	66113258	66113658	-0.639175284	0.020505534	decrease
chr14	22829816	22830216	-0.781064449	0.02051446	decrease
chr19	10292176	10292576	-0.646976619	0.02052298	decrease
chr16	4624335	4624735	-0.690378101	0.020531291	decrease
chr8	93991351	93991751	-0.795664431	0.020542463	decrease
chr15	66504817	66505217	-0.651884343	0.02056768	decrease
chr11	18395913	18396313	-0.630488391	0.020596131	decrease
chr19	9323971	9324371	-0.750089891	0.020617781	decrease
chr19	12985281	12985681	-0.796465232	0.020642782	decrease
chr4	56977631	56978031	-0.667219039	0.020653015	decrease
chr1	47432770	47433170	-0.79866829	0.020659549	decrease
chr3	150106996	150107396	-0.732130006	0.020713301	decrease
chr11	63938226	63938626	-0.712885268	0.020739399	decrease
chr1	161159193	161159593	-0.672781202	0.020761367	decrease
chr19	39031506	39031906	-0.739442411	0.020788811	decrease
chr14	31420264	31420664	-0.799176296	0.0208023	decrease
chr7	44072374	44072774	-0.66737411	0.020803385	decrease
chr1	32107967	32108367	-0.656547483	0.020806712	decrease
chrX	16882824	16883224	-0.800178022	0.020818354	decrease
chr18	63422326	63422726	-0.634271768	0.020856965	decrease
chr5	54390308	54390708	-0.765662255	0.020865053	decrease
chrX	101348563	101348963	-0.653893675	0.020865256	decrease
chr1	200316442	200316842	-0.802455501	0.020876287	decrease
chr19	796930	797330	-0.671068657	0.020877956	decrease
chr5	172282905	172283305	-0.760944242	0.02090015	decrease
chr1	156726115	156726515	-0.709256672	0.020915445	decrease
chr12	104458998	104459398	-0.788510475	0.020940085	decrease
chr20	31572589	31572989	-0.669363398	0.02095779	decrease
chr17	7614916	7615316	-0.683593758	0.020988182	decrease
chr6	19804451	19804851	-0.775478896	0.020995642	decrease
chr8	109611103	109611503	-0.711496826	0.021016793	decrease
chr7	27606471	27606871	-0.750812726	0.021020916	decrease
chrX	134990864	134991264	-0.813631305	0.021055822	decrease
chr1	63523228	63523628	-0.641413048	0.021068356	decrease
chr2	172555572	172555972	-0.781983652	0.021079231	decrease
chr1	156728082	156728482	-0.733669561	0.021105324	decrease

chr4	180057471	180057871	-0.771558405	0.021105731	decrease
chr1	154983199	154983599	-0.573168268	0.021124739	decrease
chr5	138283130	138283530	-0.791326331	0.021126973	decrease
chr19	55305340	55305740	-0.721384568	0.021135579	decrease
chr9	136400224	136400624	-0.71866375	0.021156626	decrease
chr16	30748098	30748498	-0.527044955	0.021175758	decrease
chr2	25664759	25665159	-0.762973134	0.021235916	decrease
chr7	151875302	151875702	-0.569626375	0.021283115	decrease
chr11	62545270	62545670	-0.649356825	0.021285086	decrease
chr16	27549932	27550332	-0.713389886	0.02130199	decrease
chr18	36067383	36067783	-0.6361345	0.02130226	decrease
chr19	2950563	2950963	-0.676614435	0.021302611	decrease
chr10	73999158	73999558	-0.69704414	0.021317799	decrease
chr1	109099928	109100328	-0.633803972	0.021317917	decrease
chr14	23301829	23302229	-0.812784419	0.021323369	decrease
chr2	118088522	118088922	-0.709783313	0.021347212	decrease
chrX	53093507	53093907	-0.676464153	0.021348014	decrease
chr17	76264926	76265326	-0.642337469	0.02135008	decrease
chr6	129710134	129710534	-0.705744138	0.021380502	decrease
chr16	22297173	22297573	-0.78109003	0.021387824	decrease
chr13	64834302	64834702	-0.776538465	0.021392418	decrease
chr19	49580166	49580566	-0.732231527	0.021426924	decrease
chr15	90387811	90388211	-0.750732332	0.021464099	decrease
chr9	99104541	99104941	-0.772879673	0.021477256	decrease
chr5	178235066	178235466	-0.66651627	0.021477738	decrease
chr11	73307103	73307503	-0.768628971	0.021512623	decrease
chr13	32538510	32538910	-0.639884184	0.021533924	decrease
chr9	2014820	2015220	-0.71094486	0.021537823	decrease
chr10	68560170	68560570	-0.673335791	0.021538991	decrease
chr2	216081391	216081791	-0.805636848	0.021555603	decrease
chr9	121201831	121202231	-0.674496124	0.02155801	decrease
chr1	167220427	167220827	-0.636983527	0.021563397	decrease
chr1	200620710	200621110	-0.674546126	0.021579717	decrease
chrX	108091605	108092005	-0.558748742	0.021618208	decrease
chr12	122974757	122975157	-0.676144635	0.021621947	decrease
chr13	79393490	79393890	-0.578258467	0.021623397	decrease
chr11	77821050	77821450	-0.697054737	0.021702577	decrease
chr10	29409342	29409742	-0.796440522	0.021715941	decrease
chr19	48255644	48256044	-0.765542421	0.021716189	decrease
chr7	134646533	134646933	-0.546111978	0.021719706	decrease
chr1	27959570	27959970	-0.761590788	0.021722605	decrease
chr12	68686775	68687175	-0.654596911	0.021729642	decrease
chr20	2509108	2509508	-0.66154603	0.021742606	decrease
chr11	78188492	78188892	-0.602140765	0.02174367	decrease
chr5	55438134	55438534	-0.762276688	0.021746706	decrease
chr7	1044146	1044546	-0.737082291	0.021773969	decrease
chr3	184017887	184018287	-0.742730618	0.021774932	decrease
chr1	92298682	92299082	-0.666147242	0.021807804	decrease
chr10	94545491	94545891	-0.741288001	0.021817692	decrease
chr11	44037381	44037781	-0.77899332	0.021829269	decrease
chr7	23468171	23468571	-0.726671489	0.021831689	decrease
chr8	123274553	123274953	-0.759067967	0.021834637	decrease
chr14	23306567	23306967	-0.710281376	0.021838955	decrease
chr19	15125506	15125906	-0.628426947	0.021856431	decrease
chr5	168486453	168486853	-0.74442812	0.021885974	decrease
chr12	53675851	53676251	-0.602704369	0.021894337	decrease
chr22	37953487	37953887	-0.651266391	0.02190078	decrease
chr6	57317166	57317566	-0.726411433	0.021928887	decrease
chr14	30558890	30559290	-0.733134143	0.021932023	decrease
chr17	48590366	48590766	-0.710717813	0.02193488	decrease
chr5	178204158	178204558	-0.600987105	0.021944598	decrease
chr13	75437454	75437854	-0.72350247	0.021956541	decrease
chr2	61471498	61471898	-0.73081797	0.021978324	decrease
chr13	27450050	27450450	-0.655832019	0.021988089	decrease
chr7	100101233	100101633	-0.628840488	0.022001903	decrease
chr2	3379631	3380031	-0.618428261	0.022017802	decrease
chr10	49538859	49539259	-0.744513941	0.022039326	decrease
chr18	12947786	12948186	-0.789418711	0.022050369	decrease
chr16	89722758	89723158	-0.605002277	0.022102882	decrease
chr1	32150988	32151388	-0.758320565	0.022103881	decrease
chr11	47595285	47595685	-0.784227516	0.022108323	decrease
chr4	139178196	139178596	-0.766287548	0.022111377	decrease
chr3	57170168	57170568	-0.767110322	0.022124148	decrease
chr4	118278575	118278975	-0.685184451	0.022129053	decrease
chr12	49069697	49070097	-0.599032868	0.022129281	decrease
chr1	92485889	92486289	-0.776971119	0.022142639	decrease

chr11	64372946	64373346	-0.641227143	0.022146948	decrease
chr1	151070826	151071226	-0.713314972	0.022147397	decrease
chr3	136752646	136753046	-0.739000773	0.022177777	decrease
chr5	177312383	177312783	-0.650527511	0.022188724	decrease
chr19	14032018	14032418	-0.761244301	0.022194485	decrease
chr22	43945903	43946303	-0.701071218	0.022196418	decrease
chr19	45154302	45154702	-0.688549838	0.022197421	decrease
chr10	91883760	91884160	-0.765966898	0.022209255	decrease
chr3	48898617	48899017	-0.733091989	0.022215812	decrease
chr2	149329670	149330070	-0.769291936	0.022233088	decrease
chr1	185257291	185257691	-0.735512177	0.022243114	decrease
chr16	3305301	3305701	-0.797828182	0.022251418	decrease
chr12	53421345	53421745	-0.678539446	0.022316656	decrease
chrX	49922280	49922680	-0.775070263	0.022333478	decrease
chr20	25237731	25238131	0.762593646	0.022373911	increase
chr1	150321406	150321806	-0.723083499	0.022388142	decrease
chrX	127968229	127968629	-0.785968141	0.022390485	decrease
chr16	88663036	88663436	-0.682070304	0.0224636	decrease
chr2	158973538	158973938	-0.690452999	0.022474992	decrease
chr1	215572326	215572726	-0.69624469	0.022520694	decrease
chr2	153871446	153871846	-0.765637164	0.02254058	decrease
chrX	9463178	9463578	-0.779912454	0.022555607	decrease
chr8	77000228	77000628	-0.668349206	0.022582661	decrease
chr5	81751399	81751799	-0.763697741	0.022611944	decrease
chr13	77697221	77697621	-0.680747165	0.022626101	decrease
chr22	41544495	41544895	-0.644613715	0.022662673	decrease
chr7	152463692	152464092	-0.691168192	0.02266487	decrease
chr17	81665808	81666208	-0.637410941	0.022675484	decrease
chr2	121006173	121006573	-0.787865108	0.022698375	decrease
chr5	128082970	128083370	-0.779306797	0.022713456	decrease
chr7	28685914	28686314	-0.764975276	0.022725472	decrease
chr8	80893652	80894052	-0.753549153	0.022733801	decrease
chr5	140302992	140303392	-0.665926853	0.022739947	decrease
chr5	177022440	177022840	-0.703630983	0.022747964	decrease
chr7	65269178	65269578	-0.661160378	0.022750082	decrease
chr16	53384626	53385026	-0.802556855	0.022773782	decrease
chr14	69766936	69767336	-0.665685202	0.022785026	decrease
chr17	58417530	58417930	-0.703494718	0.022794961	decrease
chr7	100407526	100407926	-0.769197206	0.022819748	decrease
chr9	27573627	27574027	-0.742957364	0.02283837	decrease
chr10	73966083	73966483	-0.700891768	0.02284448	decrease
chrX	103919007	103919407	-0.594127904	0.022846774	decrease
chr4	3074260	3074660	-0.640114566	0.02295027	decrease
chr11	114439512	114439912	-0.69222044	0.022981606	decrease
chr15	70100654	70101054	-0.628781873	0.022996531	decrease
chr19	16542488	16542888	-0.735774814	0.023004772	decrease
chr21	42833093	42833493	-0.698833503	0.023037402	decrease
chr4	83012955	83013355	-0.718599669	0.023039282	decrease
chr16	67480948	67481348	-0.624045959	0.023047035	decrease
chrX	134373056	134373456	-0.668267753	0.023074093	decrease
chr13	74040221	74040621	-0.723976446	0.023087437	decrease
chr19	44304618	44305018	-0.777550779	0.023089222	decrease
chr16	30650607	30651007	-0.573882017	0.023090331	decrease
chr5	36150869	36151269	-0.793489808	0.023101355	decrease
chr2	24540005	24540405	-0.775207019	0.023114066	decrease
chr5	78294485	78294885	-0.578149357	0.023115473	decrease
chr13	85694002	85694402	-0.768947056	0.023119365	decrease
chr4	108650277	108650677	-0.669720244	0.023132649	decrease
chr19	49869434	49869834	-0.545509134	0.023138792	decrease
chr19	44113195	44113595	-0.723568691	0.023141028	decrease
chr3	50321625	50322025	-0.640432544	0.023157185	decrease
chr16	30957426	30957826	-0.567245716	0.023160752	decrease
chr11	30322944	30323344	-0.646368448	0.023166919	decrease
chr6	43702902	43703302	-0.675736043	0.023177999	decrease
chr1	83999089	83999489	-0.682683026	0.023193412	decrease
chrX	129931840	129932240	-0.744395771	0.023199711	decrease
chr8	106269897	106270297	-0.780787749	0.02320112	decrease
chr5	126776629	126777029	-0.644319566	0.023208837	decrease
chr19	58407930	58408330	-0.727959276	0.023234163	decrease
chr1	193104582	193104982	-0.710441389	0.023236945	decrease
chr17	39235729	39236129	-0.704717812	0.02325294	decrease
chr22	20496214	20496614	-0.716085453	0.023259712	decrease
chr10	97498337	97498737	-0.637048059	0.023268808	decrease
chr5	154937909	154938309	-0.679736625	0.023269776	decrease
chr4	119067586	119067986	-0.74235216	0.023272116	decrease
chr20	18467077	18467477	-0.548425334	0.02327384	decrease

chr3	120596162	120596562	-0.665865673	0.023288454	decrease
chr16	30698066	30698466	-0.540453043	0.023296344	decrease
chr19	17234960	17235360	-0.71844487	0.023301339	decrease
chr14	65102769	65103169	-0.733751069	0.023316234	decrease
chr11	62975075	62975475	-0.730045216	0.023337064	decrease
chr9	134449529	134449929	-0.781144076	0.023342617	decrease
chr10	101042764	101043164	-0.667231164	0.023351257	decrease
chr5	16936371	16936771	-0.729743358	0.02337395	decrease
chr3	184176393	184176793	-0.71458515	0.023387584	decrease
chr11	3807707	3808107	-0.705604451	0.023404422	decrease
chr10	87095447	87095847	-0.741064184	0.023446963	decrease
chr22	31661888	31662288	-0.682302145	0.023473831	decrease
chr7	134779472	134779872	-0.660504577	0.023495294	decrease
chr2	159712418	159712818	-0.774369608	0.023497623	decrease
chr7	155456478	155456878	-0.698004291	0.023515774	decrease
chr21	34202062	34202462	-0.773351768	0.023519496	decrease
chr5	115295980	115296380	-0.792888298	0.02352387	decrease
chr11	134253173	134253573	-0.629724532	0.023532847	decrease
chr11	9314479	9314879	-0.689129217	0.023549271	decrease
chr1	182839663	182840063	-0.709491823	0.023566217	decrease
chr14	69580035	69580435	-0.779686359	0.023567252	decrease
chr4	143337166	143337566	-0.779690446	0.023589354	decrease
chrX	53225116	53225516	-0.694926107	0.023603688	decrease
chr3	138329242	138329642	-0.74006392	0.023604052	decrease
chr5	108728108	108728508	-0.67646312	0.023627417	decrease
chr2	8626640	8627040	-0.77192356	0.023636091	decrease
chr7	158856132	158856532	-0.608394854	0.023664969	decrease
chr10	97400771	97401171	-0.681973977	0.023693182	decrease
chr1	223992519	223992919	-0.635244241	0.023699017	decrease
chr17	17591611	17592011	-0.609503562	0.02372612	decrease
chr9	75088432	75088832	-0.773441366	0.023737064	decrease
chr10	100373366	100373766	-0.620256099	0.023745043	decrease
chr10	124917940	124918340	-0.736932082	0.023748577	decrease
chr22	26483466	26483866	-0.743096378	0.023764545	decrease
chr10	101588058	101588458	-0.675025005	0.023784821	decrease
chr6	107675176	107675576	-0.775625662	0.023787119	decrease
chr2	9770492	9770892	-0.757434239	0.02378968	decrease
chr9	3526362	3526762	-0.651685916	0.023800896	decrease
chr17	43024706	43025106	-0.725147978	0.023802716	decrease
chr12	56333924	56334324	-0.639419365	0.023816078	decrease
chr13	92500198	92500598	-0.749024258	0.023819643	decrease
chr12	51026191	51026591	-0.649717019	0.023824302	decrease
chr8	27311338	27311738	-0.782543889	0.023829219	decrease
chr13	76019388	76019788	-0.728910343	0.023835775	decrease
chr19	55624636	55625036	-0.677522641	0.023864273	decrease
chr11	75768311	75768711	-0.691863769	0.023879227	decrease
chr6	27684184	27684584	-0.766943007	0.023904346	decrease
chr2	74529582	74529982	-0.617720228	0.023909966	decrease
chr11	71448014	71448414	-0.795939647	0.023917744	decrease
chrX	107655212	107655612	-0.713776699	0.023930752	decrease
chr10	100535583	100535983	-0.701418383	0.023942996	decrease
chr11	95068396	95068796	-0.6684616	0.023983192	decrease
chr17	75456439	75456839	-0.61966917	0.02398413	decrease
chr10	27100278	27100678	-0.761096078	0.023986508	decrease
chr17	74982074	74982474	-0.687860844	0.023999831	decrease
chr19	19170636	19171036	-0.732823274	0.024015149	decrease
chr17	76540687	76541087	-0.568970943	0.024074407	decrease
chr6	27545548	27545948	-0.728605998	0.024074853	decrease
chr2	233854349	233854749	-0.748922334	0.024076002	decrease
chr14	92748173	92748573	-0.768917518	0.024084354	decrease
chr17	49764144	49764544	-0.65723892	0.024105769	decrease
chr6	26224080	26224480	-0.719113033	0.024128194	decrease
chr1	173862184	173862584	-0.673857165	0.024180787	decrease
chr3	48556902	48557302	-0.587260327	0.024189728	decrease
chr17	41812094	41812494	-0.693353436	0.024192159	decrease
chr3	184336706	184337106	-0.627119231	0.02420448	decrease
chr7	100126829	100127229	-0.655035595	0.024207307	decrease
chr6	26021273	26021673	-0.652418105	0.024211179	decrease
chr6	122789655	122790055	-0.717293751	0.024214594	decrease
chr19	45092063	45092463	-0.675135802	0.024224178	decrease
chr17	68019642	68020042	-0.755277193	0.024243756	decrease
chr2	219279019	219279419	-0.770577708	0.024268973	decrease
chr8	73286016	73286416	-0.764473945	0.02429678	decrease
chr6	154548311	154548711	-0.743298739	0.024299563	decrease
chr1	80403457	80403857	-0.687076964	0.024332627	decrease
chr4	84582434	84582834	-0.732912338	0.02435005	decrease

chr11	65027336	65027736	-0.666986405	0.024358263	decrease
chr8	81685956	81686356	-0.662795701	0.024383304	decrease
chr7	56051312	56051712	-0.666719063	0.024384931	decrease
chr1	179293684	179294084	-0.694708692	0.024399813	decrease
chr15	75640254	75640654	-0.546285581	0.02441071	decrease
chr7	129434022	129434422	-0.751910076	0.024414562	decrease
chr17	74213016	74213416	-0.604968521	0.02445269	decrease
chr4	168831868	168832268	-0.694147723	0.024459013	decrease
chr11	117178337	117178737	-0.70992028	0.024472947	decrease
chr9	105448372	105448772	-0.783085139	0.02450857	decrease
chr17	7835252	7835652	-0.634775406	0.02452845	decrease
chr10	117084603	117085003	-0.762952089	0.024533603	decrease
chr1	111507909	111508309	-0.660978085	0.024560801	decrease
chr5	219224	219624	-0.775169381	0.02456525	decrease
chr3	97821640	97822040	-0.771443466	0.024585603	decrease
chr21	43728380	43728780	-0.597577262	0.024603423	decrease
chr1	171485465	171485865	-0.637309592	0.024606109	decrease
chr1	203128107	203128507	-0.708582703	0.02460663	decrease
chr6	63572249	63572649	-0.743091391	0.024634492	decrease
chr11	59560089	59560489	-0.713235294	0.024642555	decrease
chr17	63848885	63849285	-0.661621504	0.024649526	decrease
chr1	89709222	89709622	-0.750025936	0.024655347	decrease
chr6	148760973	148761373	-0.775511826	0.024672535	decrease
chr19	1082399	1082799	-0.749135097	0.024678694	decrease
chrX	72572781	72573181	-0.755981696	0.024688646	decrease
chr9	131178223	131178623	-0.731536126	0.024768022	decrease
chr2	19990000	19990400	-0.743656052	0.024780429	decrease
chr3	52412040	52412440	-0.722024636	0.024787198	decrease
chr18	35386884	35387284	-0.721977083	0.024827136	decrease
chr13	76884459	76884859	-0.696206386	0.024861495	decrease
chr13	78605355	78605755	-0.625120179	0.024862401	decrease
chr10	132389316	132389716	-0.714110735	0.024875725	decrease
chr15	68875495	68875895	-0.741306014	0.024911999	decrease
chr17	59964716	59965116	-0.738163996	0.024926857	decrease
chr1	224114175	224114575	-0.70527695	0.024948862	decrease
chr20	63628148	63628548	-0.680762191	0.024958937	decrease
chr1	56579350	56579750	-0.71259479	0.024973573	decrease
chr2	70301293	70301693	-0.736383879	0.024979615	decrease
chr9	37785100	37785500	-0.719621841	0.025024921	decrease
chr12	57487751	57488151	-0.685880675	0.025042263	decrease
chr9	4679506	4679906	-0.670764257	0.025070766	decrease
chr18	74495898	74496298	-0.769886903	0.025077172	decrease
chr11	113681834	113682234	-0.752661897	0.025090428	decrease
chr13	44574093	44574493	-0.741348229	0.025121944	decrease
chr11	47248700	47249100	-0.658776435	0.02515109	decrease
chr12	103929962	103930362	-0.643720587	0.025179592	decrease
chr20	37095812	37096212	-0.679622546	0.025181038	decrease
chr10	44959302	44959702	-0.736091896	0.025186157	decrease
chr4	121071205	121071605	-0.702478512	0.025197997	decrease
chr2	58241047	58241447	-0.723244384	0.025207885	decrease
chr6	35467996	35468396	-0.607382823	0.025215205	decrease
chr10	100481535	100481935	-0.75855738	0.02522153	decrease
chr11	20388024	20388424	-0.734188804	0.025223833	decrease
chr21	45512752	45513152	-0.65878447	0.025228152	decrease
chr16	24539003	24539403	-0.69644759	0.025228674	decrease
chr19	46661375	46661775	-0.594757569	0.025235894	decrease
chr2	75646676	75647076	-0.694502476	0.025265727	decrease
chr2	85066863	85067263	-0.756876427	0.025267235	decrease
chr12	2795928	2796328	-0.783239067	0.025303831	decrease
chr12	52025737	52026137	-0.683611189	0.025314481	decrease
chr5	132556805	132557205	-0.573531584	0.025341646	decrease
chr7	149126818	149127218	-0.734471086	0.025343661	decrease
chr8	66177228	66177628	-0.779609938	0.025349621	decrease
chr3	49705647	49706047	-0.706298617	0.025362715	decrease
chr4	55853607	55854007	-0.767769649	0.025377274	decrease
chr3	197737075	197737475	-0.773163195	0.025382826	decrease
chr19	14529289	14529689	-0.602690951	0.025385651	decrease
chr11	65961243	65961643	-0.694983151	0.025397315	decrease
chr6	43516765	43517165	-0.608637199	0.025423341	decrease
chr20	34069622	34070022	-0.760371899	0.025426894	decrease
chr5	55307421	55307821	-0.698675747	0.025429906	decrease
chr1	182029364	182029764	-0.710309461	0.025455893	decrease
chr1	75376148	75376548	-0.655632226	0.025457649	decrease
chr1	182615036	182615436	-0.632109325	0.025459063	decrease
chr5	37371171	37371571	-0.631774733	0.025463517	decrease
chr2	219229590	219229990	-0.632366286	0.025493911	decrease

chr7	128476531	128476931	-0.702309874	0.025498773	decrease
chr12	49842669	49843069	-0.660122244	0.025521993	decrease
chr15	78709288	78709688	-0.732878905	0.025553362	decrease
chr9	121278851	121279251	-0.758079208	0.025586621	decrease
chr6	26568805	26569205	-0.758967052	0.025592005	decrease
chr17	75979296	75979696	-0.514858557	0.025644287	decrease
chr1	21783294	21783694	-0.664607789	0.025651326	decrease
chr19	4968553	4968953	-0.595731435	0.025652828	decrease
chr5	41924893	41925293	-0.768733251	0.0256567	decrease
chr19	9768459	9768859	-0.719382604	0.02567511	decrease
chr3	58463565	58463965	-0.727577696	0.02571272	decrease
chr1	228486986	228487386	-0.590448203	0.02572132	decrease
chr4	87422603	87423003	-0.701612016	0.025729753	decrease
chr1	48498248	48498648	-0.762221621	0.025762993	decrease
chr5	65723018	65723418	-0.747568903	0.02578671	decrease
chr12	4648861	4649261	-0.739965846	0.025789055	decrease
chr11	47553211	47553611	-0.741366762	0.025794851	decrease
chr7	152675843	152676243	-0.637899299	0.025800005	decrease
chrX	65534541	65534941	-0.687886615	0.025823682	decrease
chr2	69437267	69437667	-0.579978765	0.025832953	decrease
chr7	135714864	135715264	-0.5847403	0.025864386	decrease
chr17	7844626	7845026	-0.602300289	0.025872955	decrease
chr2	84459189	84459589	-0.632320958	0.025889316	decrease
chr7	80783728	80784128	-0.762156732	0.025895693	decrease
chr19	50333567	50333967	-0.648300792	0.025902181	decrease
chr6	79078365	79078765	-0.731853741	0.025911523	decrease
chr12	64608943	64609343	-0.683825883	0.025935899	decrease
chr1	150579290	150579690	-0.72936621	0.025944694	decrease
chr22	39523202	39523602	-0.539909345	0.025947496	decrease
chr4	139454182	139454582	-0.706030676	0.025963701	decrease
chrX	134796817	134797217	-0.770128881	0.025964832	decrease
chr11	114400290	114400690	-0.650849047	0.025965624	decrease
chrX	119852752	119853152	-0.544692914	0.025969556	decrease
chr16	1705581	1705981	-0.669079422	0.025978122	decrease
chr9	125752218	125752618	-0.740096422	0.02598481	decrease
chr2	156332746	156333146	-0.691341887	0.026028391	decrease
chr17	82153977	82154377	-0.611717482	0.026087906	decrease
chrX	47361850	47362250	-0.606212983	0.026128876	decrease
chr12	57217584	57217984	-0.691553367	0.026132201	decrease
chr2	19670385	19670785	-0.72564397	0.026151961	decrease
chr14	21103786	21104186	-0.595930536	0.026154278	decrease
chr4	7042773	7043173	-0.756833277	0.026157987	decrease
chr5	134586994	134587394	-0.662690893	0.026174893	decrease
chr7	128862893	128863293	-0.626281757	0.026191902	decrease
chr17	49707902	49708302	-0.620476359	0.026194199	decrease
chr12	72271728	72272128	-0.685164146	0.026214095	decrease
chr17	44070516	44070916	-0.700767929	0.026214101	decrease
chr6	34425858	34426258	-0.722651306	0.026218417	decrease
chr12	55940166	55940566	-0.667671928	0.026254785	decrease
chr1	54199959	54200359	-0.564609097	0.026259794	decrease
chr19	56595588	56595988	-0.759853432	0.026275486	decrease
chr6	7542221	7542621	-0.72455636	0.026304581	decrease
chr17	28599119	28599519	-0.659040302	0.026307863	decrease
chr13	93227821	93228221	-0.547582717	0.026308468	decrease
chr22	20129157	20129557	-0.581747568	0.026355596	decrease
chr17	45247488	45247888	-0.641537117	0.026357993	decrease
chr17	68292130	68292530	-0.756378025	0.026358618	decrease
chr1	6784988	6785388	-0.705980656	0.026368546	decrease
chrX	153245356	153245756	-0.743299453	0.026389802	decrease
chr8	92965744	92966144	-0.618854977	0.026403584	decrease
chr2	85615872	85616272	-0.683739901	0.026459946	decrease
chr1	175029643	175030043	-0.685758041	0.026468807	decrease
chr5	59275821	59276221	-0.719683452	0.026469061	decrease
chr8	119832567	119832967	-0.701611694	0.026500563	decrease
chr12	54324781	54325181	-0.678648909	0.026514583	decrease
chr1	229508053	229508453	-0.595569063	0.026546087	decrease
chr6	158999767	159000167	-0.560084901	0.02655691	decrease
chr14	24367122	24367522	-0.472217354	0.026567536	decrease
chr9	37913708	37914108	-0.748575518	0.02657025	decrease
chr5	179821616	179822016	-0.606148111	0.026604481	decrease
chr8	144373391	144373791	-0.611846776	0.026607236	decrease
chr1	237041649	237042049	-0.757542918	0.026633295	decrease
chr14	92794114	92794514	-0.624688084	0.02664071	decrease
chr5	140718877	140719277	-0.543729814	0.026642672	decrease
chr12	46371496	46371896	-0.699000835	0.026671824	decrease
chrX	154398026	154398426	-0.506480985	0.02667649	decrease

chr12	51082893	51083293	-0.766382272	0.026699026	decrease
chr20	38435467	38435867	-0.709513235	0.026719304	decrease
chr13	27620505	27620905	-0.643334958	0.026730652	decrease
chr20	32820200	32820600	-0.7242362	0.026738502	decrease
chr17	44186588	44186988	-0.63220761	0.026755868	decrease
chr9	68779948	68780348	-0.65437663	0.026767431	decrease
chr12	2812542	2812942	-0.558940658	0.026768008	decrease
chr16	88857450	88857850	-0.692818239	0.026774087	decrease
chr12	62260017	62260417	-0.591819847	0.026802246	decrease
chr20	31951755	31952155	-0.742514434	0.026815768	decrease
chr8	106657510	106657910	-0.674288271	0.026818581	decrease
chr12	76630613	76631013	-0.749438362	0.026910473	decrease
chr9	34989226	34989626	-0.688749762	0.026912938	decrease
chr11	3797574	3797974	-0.591907431	0.026937497	decrease
chr7	135728240	135728640	-0.671195499	0.02697479	decrease
chr4	174225229	174225629	-0.777644007	0.026980071	decrease
chr7	101856856	101857256	-0.623725161	0.026993178	decrease
chr7	139483515	139483915	-0.664672743	0.027006483	decrease
chr7	83266952	83267352	-0.77355143	0.027008613	decrease
chr11	45804767	45805167	-0.672894256	0.027010573	decrease
chr5	113214603	113215003	-0.757556437	0.027021042	decrease
chr7	128404316	128404716	-0.679945401	0.02703421	decrease
chr16	2223839	2224239	-0.686464374	0.027068311	decrease
chr3	27711972	27712372	-0.754651689	0.027073483	decrease
chr12	6554220	6554620	-0.581629332	0.027089798	decrease
chr14	49852624	49853024	-0.685396935	0.027096218	decrease
chr19	1383497	1383897	-0.745533017	0.02710273	decrease
chr19	7535180	7535580	-0.612061646	0.027126303	decrease
chr13	62358000	62358400	-0.693039243	0.027199722	decrease
chrX	63350941	63351341	-0.655516294	0.02720532	decrease
chr17	3724540	3724940	-0.735312521	0.027208043	decrease
chr19	37467115	37467515	-0.694485289	0.027253997	decrease
chr5	111453793	111454193	-0.757792452	0.027254369	decrease
chr9	122940666	122941066	-0.632828605	0.027267968	decrease
chr2	96857414	96857814	-0.703530775	0.027270852	decrease
chr6	8064134	8064534	-0.647381205	0.02730249	decrease
chr8	123156946	123157346	-0.582256167	0.02730669	decrease
chr5	134845633	134846033	-0.730575782	0.027323789	decrease
chr17	82534352	82534752	-0.69167334	0.027377799	decrease
chr18	29309443	29309843	-0.717919766	0.027397598	decrease
chr3	15098891	15099291	-0.706015772	0.027407325	decrease
chr20	9838401	9838801	-0.74206673	0.027432908	decrease
chr1	45011529	45011929	-0.604254665	0.027434664	decrease
chrX	77786038	77786438	-0.742287744	0.027459683	decrease
chr19	18203932	18204332	-0.570711095	0.027461486	decrease
chr1	32072831	32073231	-0.581558731	0.027465563	decrease
chr11	6390110	6390510	-0.67784538	0.027473226	decrease
chr2	24920693	24921093	-0.745976422	0.027477624	decrease
chr4	17614412	17614812	-0.64334658	0.027481824	decrease
chr3	9843736	9844136	-0.654155829	0.027486967	decrease
chr21	26845290	26845690	-0.605777974	0.027488413	decrease
chr4	72568993	72569393	-0.69690915	0.027514956	decrease
chr17	7887880	7888280	-0.65519298	0.027515419	decrease
chr6	125789699	125790099	-0.686401437	0.027595468	decrease
chr9	110256552	110256952	-0.715486211	0.027596811	decrease
chr7	75528047	75528447	-0.703889925	0.027600531	decrease
chrX	109536749	109537149	-0.683924276	0.027608972	decrease
chrX	54808538	54808938	-0.578170927	0.027640672	decrease
chr19	17932572	17932972	-0.645509167	0.027641681	decrease
chr2	190319717	190320117	-0.680010264	0.027673235	decrease
chr15	43776157	43776557	-0.739842078	0.027692869	decrease
chr22	39529073	39529473	-0.494329195	0.027706059	decrease
chr14	32201350	32201750	-0.596024679	0.027712406	decrease
chr22	18954563	18954963	-0.737140954	0.027740329	decrease
chr10	84196445	84196845	-0.72549459	0.027742844	decrease
chr1	229624964	229625364	-0.640731382	0.027760777	decrease
chr5	134738346	134738746	-0.618926928	0.027775222	decrease
chr12	132083738	132084138	-0.665555045	0.027777553	decrease
chr15	41416618	41417018	-0.752333151	0.027779185	decrease
chr17	75034822	75035222	-0.556023561	0.027795642	decrease
chr14	28768027	28768427	-0.693383683	0.027815674	decrease
chr3	156674084	156674484	-0.6777461	0.027847873	decrease
chr3	138187135	138187535	-0.686664196	0.027890007	decrease
chr6	27472719	27473119	-0.657352294	0.027923198	decrease
chr3	160755122	160755522	-0.652371269	0.027932052	decrease
chr17	39154200	39154600	-0.567520532	0.027935884	decrease

chr15	90902283	90902683	-0.670012841	0.027956572	decrease
chr6	156687734	156688134	-0.702746211	0.027957032	decrease
chr22	30426361	30426761	-0.671161338	0.027972092	decrease
chr11	67630375	67630775	-0.684012459	0.027980121	decrease
chr19	49533781	49534181	-0.694732578	0.02798048	decrease
chr1	228391883	228392283	-0.626426624	0.027986034	decrease
chr11	59550322	59550722	-0.50032676	0.027987648	decrease
chr3	101512121	101512521	-0.745113708	0.027997695	decrease
chr7	74173464	74173864	-0.741141094	0.028015239	decrease
chr4	141220929	141221329	-0.735162059	0.028030816	decrease
chr4	112144984	112145384	-0.750190206	0.028033824	decrease
chr12	55829614	55830014	-0.612462164	0.028049315	decrease
chr1	233328913	233329313	-0.696696272	0.02808063	decrease
chr6	93342156	93342556	-0.771853462	0.028118853	decrease
chr21	42653096	42653496	-0.765162536	0.02812054	decrease
chr19	47745431	47745831	-0.756660441	0.028131358	decrease
chr1	155135484	155135884	-0.612805352	0.028153905	decrease
chr15	89243795	89244195	-0.618576349	0.028164629	decrease
chrX	70289951	70290351	-0.696843975	0.028165743	decrease
chr18	36129425	36129825	-0.48256502	0.028167741	decrease
chr6	24666679	24667079	-0.671984651	0.028170698	decrease
chr19	38384782	38385182	-0.688418503	0.028177791	decrease
chr14	48809506	48809906	-0.716588895	0.028212915	decrease
chr15	64841298	64841698	-0.759380455	0.028229545	decrease
chr15	74906937	74907337	-0.647719428	0.028261554	decrease
chr3	51539238	51539638	-0.724987579	0.028278434	decrease
chr5	95834804	95835204	-0.730213297	0.028298961	decrease
chr2	85889130	85889530	-0.739319528	0.028305382	decrease
chr7	114085550	114085950	-0.670682468	0.028310227	decrease
chr11	134275791	134276191	-0.701402702	0.028316648	decrease
chr1	225882246	225882646	-0.54603556	0.028317919	decrease
chr10	21533649	21534049	-0.686108476	0.028334677	decrease
chr17	44123709	44124109	-0.682169093	0.028353512	decrease
chr10	75394796	75395196	-0.755983597	0.028378302	decrease
chr21	43866780	43867180	-0.681334587	0.028392986	decrease
chr11	107255188	107255588	-0.747531574	0.028394781	decrease
chrUn_KI270438v1	109235	109635	0.440376978	0.0283971	increase
chr19	16496039	16496439	-0.619010565	0.028411466	decrease
chr6	125791172	125791572	-0.705756467	0.028459979	decrease
chr17	8222101	8222501	-0.540532758	0.028483228	decrease
chr13	30301324	30301724	-0.696080096	0.02851937	decrease
chrX	44542791	44543191	-0.69802586	0.028522056	decrease
chr12	48119178	48119578	-0.691338618	0.028541484	decrease
chr13	67200543	67200943	-0.681836386	0.028545351	decrease
chr2	62705275	62705675	-0.64063912	0.028557302	decrease
chr13	66803244	66803644	-0.614230295	0.028557943	decrease
chrX	154750356	154750756	-0.749227159	0.028562825	decrease
chr10	97633504	97633904	-0.573936707	0.028573897	decrease
chr16	67926291	67926691	-0.688194818	0.028574901	decrease
chr18	35041363	35041763	-0.581248531	0.028597739	decrease
chr12	110613819	110614219	-0.693288759	0.028650112	decrease
chr17	75516148	75516548	-0.613212613	0.028652219	decrease
chr1	109112721	109113121	-0.651878093	0.028665552	decrease
chr6	1802521	1802921	-0.753576556	0.028666233	decrease
chr19	4584031	4584431	-0.725678219	0.028678877	decrease
chr8	144423440	144423840	-0.644407844	0.028681625	decrease
chr10	55631167	55631567	-0.748190578	0.028683498	decrease
chr1	28517831	28518231	-0.626601022	0.028708788	decrease
chr6	27838123	27838523	-0.599851638	0.028716155	decrease
chr17	82717855	82718255	-0.69102398	0.028722713	decrease
chr19	47697930	47698330	-0.719206731	0.028735209	decrease
chr7	2232910	2233310	-0.681768183	0.028737144	decrease
chr20	38748127	38748527	-0.719961461	0.028789912	decrease
chr17	13018055	13018455	-0.74027691	0.028804399	decrease
chr2	61764122	61764522	-0.559539804	0.028815425	decrease
chr10	27155028	27155428	-0.609509402	0.028817881	decrease
chr7	149496356	149496756	-0.603114567	0.028822339	decrease
chr6	35259112	35259512	-0.642645738	0.02885139	decrease
chr12	46133980	46134380	-0.746523447	0.028855725	decrease
chr17	47941358	47941758	-0.647019565	0.028856767	decrease
chr8	10839798	10840198	-0.724937973	0.028862134	decrease
chr11	86302117	86302517	-0.63422165	0.028883086	decrease
chr6	52200018	52200418	-0.696099951	0.028893402	decrease
chr12	100138402	100138802	-0.735377865	0.028899105	decrease
chr22	42661800	42662200	-0.74808084	0.028901677	decrease
chr5	96663015	96663415	-0.725027939	0.028911901	decrease

chr7	65982240	65982640	-0.679661602	0.028946218	decrease
chrX	49235352	49235752	-0.699749436	0.028947981	decrease
chr19	39412133	39412533	-0.56607593	0.028972361	decrease
chr16	56730210	56730610	-0.711633888	0.028987875	decrease
chr3	66220687	66221087	-0.584982533	0.029010916	decrease
chr8	33472944	33473344	-0.5667177	0.029019822	decrease
chr5	112861200	112861600	-0.683909248	0.029022219	decrease
chrX	129957361	129957761	-0.703947327	0.029031618	decrease
chr22	38506313	38506713	-0.733202305	0.029057486	decrease
chr11	63838734	63839134	-0.698132192	0.029061812	decrease
chr1	149812183	149812583	-0.703359397	0.029072947	decrease
chr1	198158701	198159101	-0.657398131	0.029089257	decrease
chr21	42923993	42924393	-0.659901628	0.02909104	decrease
chr19	48933487	48933887	-0.72442536	0.029096286	decrease
chr11	64675650	64676050	-0.745938681	0.029104103	decrease
chr6	37257722	37258122	-0.623732226	0.029142278	decrease
chr1	28510260	28510660	-0.622054988	0.029184371	decrease
chr1	52055224	52055624	-0.582832268	0.029191988	decrease
chr7	128739122	128739522	-0.614837988	0.029194768	decrease
chr5	128538811	128539211	-0.725441079	0.029227025	decrease
chrX	123784990	123785390	-0.712830563	0.029236508	decrease
chr2	61888514	61888914	-0.634656287	0.029263837	decrease
chr3	79019336	79019736	-0.602584602	0.029353158	decrease
chr11	72814639	72815039	-0.656400552	0.029373824	decrease
chr14	24242297	24242697	-0.583503675	0.029426017	decrease
chr8	61714632	61715032	-0.664379942	0.029445184	decrease
chr1	225845736	225846136	-0.514987791	0.029472639	decrease
chr5	83251648	83252048	-0.749883108	0.029477417	decrease
chr16	69726441	69726841	-0.653541975	0.029504042	decrease
chr3	119468928	119469328	-0.728634053	0.029504316	decrease
chr4	126427893	126428293	-0.696526974	0.029510495	decrease
chr20	35619380	35619780	-0.605217252	0.029532388	decrease
chr6	5891935	5892335	-0.705279823	0.029546329	decrease
chr5	141636863	141637263	-0.712431588	0.029546988	decrease
chr9	92765403	92765803	-0.65409135	0.029566284	decrease
chr11	36149760	36150160	-0.682251013	0.029572235	decrease
chr3	110888845	110889245	-0.750510033	0.029591604	decrease
chr12	27022366	27022766	-0.675394809	0.029594913	decrease
chr4	163494457	163494857	-0.543494103	0.029612162	decrease
chr7	158673280	158673680	-0.750229718	0.029614331	decrease
chr16	3191428	3191828	-0.56724176	0.029621091	decrease
chr16	3157099	3157499	-0.555347937	0.029675368	decrease
chr16	3023962	3024362	-0.518906479	0.029681567	decrease
chr9	124852860	124853260	-0.655771074	0.029701022	decrease
chr12	53727400	53727800	-0.635554607	0.02974789	decrease
chr20	13784761	13785161	-0.67569415	0.02974911	decrease
chr13	27425920	27426320	-0.640504223	0.029806918	decrease
chr16	74666686	74667086	-0.673960461	0.029808734	decrease
chr11	46239014	46239414	-0.719845572	0.029830516	decrease
chr2	223815173	223815573	-0.69017678	0.029845523	decrease
chr12	8949713	8950113	-0.614767579	0.029859365	decrease
chrX	40175431	40175831	-0.673481602	0.029870877	decrease
chr9	33076513	33076913	-0.629024628	0.029871975	decrease
chr1	228131076	228131476	-0.604846786	0.029876323	decrease
chr1	145859003	145859403	-0.594749506	0.029896349	decrease
chr5	120160101	120160501	-0.749261522	0.029901014	decrease
chr3	139389601	139390001	-0.573811981	0.029911056	decrease
chr1	84604398	84604798	-0.683150019	0.029955671	decrease
chr15	90717305	90717705	-0.599076102	0.029994134	decrease
chr3	185937678	185938078	-0.670076105	0.030019602	decrease
chr14	76913443	76913843	-0.737216563	0.030024	decrease
chr22	35350693	35351093	-0.609094524	0.030046496	decrease
chr10	19816834	19817234	-0.713178292	0.030054517	decrease
chr17	82339217	82339617	-0.643578987	0.030069381	decrease
chr18	56651073	56651473	-0.679299384	0.03007206	decrease
chr5	134226207	134226607	-0.701194601	0.030074691	decrease
chr2	74454792	74455192	-0.625286577	0.030129448	decrease
chr1	151189854	151190254	-0.62698141	0.030135851	decrease
chr8	60653534	60653934	-0.699259129	0.030150983	decrease
chr1	161420940	161421340	-0.745296027	0.030160619	decrease
chr2	223957163	223957563	-0.756087056	0.030186489	decrease
chr19	1876214	1876614	-0.68005154	0.030194164	decrease
chr7	55951711	55952111	-0.664169601	0.030230005	decrease
chr1	101282302	101282891	-0.706943044	0.0303166	decrease
chr5	79237396	79237796	-0.694107344	0.030319027	decrease
chr2	217434209	217434609	-0.705638241	0.030321188	decrease

chr11	64723186	64723586	-0.730663498	0.03033683	decrease
chr14	20682393	20682793	-0.551691763	0.030345857	decrease
chr11	66002064	66002464	-0.608030192	0.030348695	decrease
chr16	4957862	4958262	-0.641751434	0.030358117	decrease
chr19	49850620	49851020	-0.614617356	0.0303757	decrease
chr2	62505873	62506273	-0.718969877	0.030389251	decrease
chr6	8102234	8102634	-0.619948914	0.030406782	decrease
chr9	131373444	131373844	-0.603484652	0.030419248	decrease
chr14	30270638	30271038	-0.742407825	0.030432906	decrease
chr3	105868969	105869369	-0.667478235	0.030470383	decrease
chr5	100903095	100903495	-0.695304095	0.030480288	decrease
chr1	100266075	100266475	-0.614036831	0.03051167	decrease
chr15	69452412	69452812	-0.556502118	0.030531454	decrease
chr5	115626140	115626540	-0.661481057	0.030535948	decrease
chr15	72967803	72968203	-0.716986342	0.030547606	decrease
chr11	66480206	66480606	-0.602274473	0.030571885	decrease
chr14	49892707	49893107	-0.656053529	0.030634362	decrease
chr18	48538672	48539072	-0.749849306	0.03069214	decrease
chr11	65570000	65570400	-0.61248674	0.03070929	decrease
chr1	160291632	160292032	-0.716531692	0.030719351	decrease
chr6	49462963	49463363	-0.657568157	0.030726728	decrease
chr11	62880885	62881285	-0.623425286	0.030728929	decrease
chr7	150964666	150965066	-0.61249348	0.030795818	decrease
chr6	124823556	124823956	-0.700429751	0.03081595	decrease
chr8	93916339	93916739	-0.700832537	0.030864188	decrease
chr22	38353356	38353756	-0.632665466	0.030870543	decrease
chr1	44739548	44739948	-0.573367261	0.03089688	decrease
chr7	73715648	73716048	-0.643773199	0.030908367	decrease
chr2	84926004	84926404	-0.628980128	0.030923864	decrease
chr16	22374742	22375142	-0.690280447	0.03095084	decrease
chr12	202123	202523	-0.740683775	0.030962459	decrease
chr1	151399161	151399561	-0.699827052	0.030970108	decrease
chr5	91380684	91381084	-0.687808906	0.030979637	decrease
chr14	88824341	88824741	-0.562586772	0.030983182	decrease
chr2	112764407	112764807	-0.669858345	0.031014117	decrease
chr2	219433866	219434266	-0.712509236	0.031017872	decrease
chr14	24094476	24094876	-0.679854142	0.031031128	decrease
chr11	118997766	118998166	-0.614579476	0.031047166	decrease
chr6	6687021	6687421	-0.634714834	0.031051159	decrease
chr15	36579425	36579825	-0.744172951	0.031053858	decrease
chr14	22871391	22871791	-0.727956321	0.031061299	decrease
chr19	6199482	6199882	-0.664868653	0.031063778	decrease
chr17	29389977	29390377	-0.671786311	0.03106616	decrease
chr11	85664908	85665308	-0.678047281	0.031088364	decrease
chr21	28885043	28885443	-0.65229852	0.031094739	decrease
chr7	29146398	29146798	-0.652855483	0.03110755	decrease
chr11	117986002	117986402	-0.733832148	0.03111077	decrease
chr1	71054191	71054591	-0.733233619	0.031134836	decrease
chr7	76178014	76178414	-0.67648957	0.031144146	decrease
chr11	57568160	57568560	-0.72907573	0.03116998	decrease
chr3	49539879	49540279	-0.676991552	0.031176718	decrease
chr21	39388391	39388791	-0.744748607	0.031177851	decrease
chr7	118214389	118214789	-0.718806572	0.031179474	decrease
chr1	111755565	111755965	-0.633022057	0.031185958	decrease
chr3	179562662	179563062	-0.662670337	0.031187712	decrease
chr6	2989162	2989562	-0.676790709	0.031188321	decrease
chr17	79793856	79794256	-0.648504904	0.031211146	decrease
chr1	212477231	212477631	-0.666860435	0.031256155	decrease
chr17	75620110	75620510	-0.631432607	0.031259219	decrease
chr3	42600312	42600712	-0.598748019	0.031265629	decrease
chr22	36388037	36388437	-0.600733507	0.031271695	decrease
chr3	157160032	157160432	-0.617083527	0.031273455	decrease
chr7	97117469	97117869	-0.638106469	0.031290819	decrease
chr13	39602650	39603050	-0.740509247	0.031292194	decrease
chr5	111092303	111092703	-0.699288533	0.031304057	decrease
chr6	85678562	85679322	-0.585212637	0.031323589	decrease
chr14	100192908	100193308	-0.596215305	0.031323829	decrease
chr10	68527462	68527862	-0.698826229	0.03134686	decrease
chr14	50667298	50667698	-0.741342686	0.031347523	decrease
chr21	39348274	39348674	-0.753133365	0.031369492	decrease
chr9	128218705	128219105	-0.670132304	0.031397718	decrease
chr15	85296661	85297061	-0.697913584	0.031409946	decrease
chr17	63889352	63889752	-0.713532709	0.031428409	decrease
chr1	16904867	16905267	-0.554688994	0.03144421	decrease
chr5	93045068	93045468	-0.73144834	0.031455004	decrease
chr1	39799610	39800010	-0.66898082	0.031466835	decrease

chr16	30430051	30430451	-0.524706533	0.031525406	decrease
chr5	133051699	133052099	-0.595377001	0.031555139	decrease
chr12	121016248	121016648	-0.586340138	0.031609786	decrease
chr16	3172091	3172491	-0.577316347	0.031614159	decrease
chr6	10413826	10414226	-0.656387578	0.031628113	decrease
chr9	72305140	72305540	-0.737953885	0.03164717	decrease
chr10	72228243	72228643	-0.730377032	0.031649094	decrease
chr1	101025735	101026135	-0.589955478	0.031668095	decrease
chr1	150272177	150272577	-0.710292939	0.031671528	decrease
chr15	75023419	75023819	-0.618488285	0.031677178	decrease
chr3	44761735	44762135	-0.700893147	0.031714083	decrease
chr16	77190696	77191096	-0.578272886	0.031787184	decrease
chr2	241102263	241102663	-0.657224461	0.031801744	decrease
chr11	17208063	17208463	-0.640625176	0.031822938	decrease
chr12	43551994	43552394	-0.677055513	0.03184061	decrease
chr6	121437444	121437844	-0.731627947	0.031841293	decrease
chr15	92818842	92819242	-0.708421347	0.031850011	decrease
chr7	155380214	155380614	-0.706500306	0.031896055	decrease
chr16	56951161	56951561	-0.699700149	0.031911822	decrease
chr17	76398228	76398628	-0.646337489	0.03192527	decrease
chr10	28532720	28533120	-0.716933046	0.031938788	decrease
chr11	9575117	9575517	-0.699652469	0.031972189	decrease
chr22	39519870	39520270	-0.507964562	0.032025903	decrease
chr3	136818804	136819204	-0.639929363	0.032026678	decrease
chr3	56682527	56682927	-0.744920433	0.032034694	decrease
chr5	141617877	141618277	-0.714077754	0.032078194	decrease
chr6	168025509	168025909	-0.726344334	0.032081474	decrease
chr4	136395679	136396079	-0.746968256	0.032085222	decrease
chr12	89525761	89526161	-0.709437178	0.032096664	decrease
chr12	49188843	49189243	-0.618596166	0.032109707	decrease
chr19	45523675	45524075	-0.695953392	0.032116766	decrease
chr18	33804112	33804512	-0.734993397	0.032132288	decrease
chr5	149960628	149961028	-0.647262838	0.032139238	decrease
chr3	170189239	170189639	-0.749840027	0.032165715	decrease
chr9	113541312	113541712	-0.744113145	0.032171563	decrease
chr11	62646761	62647161	-0.639594107	0.032181893	decrease
chr17	21275400	21275800	-0.691551041	0.032182481	decrease
chr1	150515569	150515969	-0.572916557	0.032195945	decrease
chr3	56684001	56684401	-0.736514235	0.032196681	decrease
chr17	29929346	29929746	-0.688801819	0.032213936	decrease
chr7	151057866	151058266	-0.470908793	0.032223741	decrease
chr3	126835896	126836296	-0.718785761	0.03225195	decrease
chr16	70251933	70252333	-0.638911258	0.032269042	decrease
chr16	30805702	30806102	-0.580425103	0.032272591	decrease
chr17	1769783	1770183	-0.692910304	0.032279869	decrease
chr12	65170350	65170750	-0.715545639	0.03228128	decrease
chr10	59906525	59906925	-0.677544236	0.032301691	decrease
chr19	17830509	17830909	-0.689025183	0.032329901	decrease
chr3	150384838	150385238	-0.698211928	0.032334337	decrease
chr12	50167339	50167739	-0.65056777	0.03234821	decrease
chr5	43017658	43018058	-0.645473561	0.032358244	decrease
chr3	194769162	194769562	-0.692591581	0.032365778	decrease
chr9	76333547	76333947	-0.737232095	0.032401321	decrease
chr16	30927944	30928344	-0.56758779	0.032418351	decrease
chr12	54017874	54018274	-0.569345003	0.032426334	decrease
chr17	78782441	78782841	-0.645266009	0.032451973	decrease
chr15	88904819	88905219	-0.670225949	0.032490968	decrease
chr8	78515980	78516380	-0.631505346	0.032527511	decrease
chr15	67863514	67863914	-0.730120596	0.032536257	decrease
chr16	11742873	11743273	-0.60999824	0.032544048	decrease
chr9	114587417	114587817	-0.538930676	0.032549135	decrease
chr3	142999461	142999861	-0.673834092	0.032583445	decrease
chr15	72783818	72784218	-0.645479564	0.032619214	decrease
chr21	26125838	26126238	-0.725743528	0.032632619	decrease
chr17	60142657	60143057	-0.688180714	0.032634775	decrease
chr12	107912623	107913023	-0.701398047	0.032645073	decrease
chr14	100376228	100376628	-0.599318449	0.032673354	decrease
chr8	98045501	98045901	-0.685699173	0.032707352	decrease
chr4	18021914	18022314	-0.639099285	0.03271556	decrease
chr8	94474933	94475333	-0.565735045	0.032719159	decrease
chr22	36239463	36239863	-0.667127248	0.032724888	decrease
chr19	2151625	2152025	-0.753422642	0.032807965	decrease
chr2	3581931	3582331	-0.667646719	0.032808083	decrease
chr14	45084023	45084423	-0.678078892	0.032811969	decrease
chr10	63268951	63269351	-0.681246818	0.032812366	decrease
chr5	55226577	55226977	-0.711814739	0.032853388	decrease

chr14	34462821	34463221	-0.72850829	0.032925264	decrease
chr4	109559831	109560231	-0.72146439	0.032944415	decrease
chr5	116574743	116575143	-0.543396909	0.032946047	decrease
chr10	93437228	93437628	-0.646632979	0.032958607	decrease
chr5	110739106	110739506	-0.678582903	0.032974927	decrease
chr17	55750880	55751280	-0.642062459	0.033016731	decrease
chr12	123633574	123633974	-0.53969195	0.033053811	decrease
chr6	127516544	127516944	-0.630160281	0.033072769	decrease
chr19	57819363	57819763	-0.657013378	0.033086532	decrease
chrX	107676742	107677142	-0.594143536	0.033138853	decrease
chr3	48472940	48473340	-0.634489897	0.033140577	decrease
chr4	99950470	99950870	-0.600573522	0.033231345	decrease
chr9	104747669	104748069	-0.690205715	0.033237434	decrease
chr5	172735969	172736369	-0.726411361	0.03323746	decrease
chr1	33349950	33350350	-0.698606246	0.033243271	decrease
chr1	228866974	228867374	-0.600519644	0.033266134	decrease
chr5	154682479	154682879	-0.665156591	0.03333755	decrease
chr1	235093600	235094000	-0.65360502	0.03336189	decrease
chr8	38996457	38996857	-0.615702869	0.033364269	decrease
chr12	53985383	53985783	-0.679805191	0.033380278	decrease
chr2	127365325	127365725	-0.732857977	0.033418284	decrease
chr8	22561964	22562364	-0.72316639	0.033453314	decrease
chr4	174268235	174268635	-0.710835862	0.033476281	decrease
chr3	130746873	130747273	-0.680385107	0.033477795	decrease
chr10	22316600	22317000	-0.654056078	0.033485164	decrease
chr1	3857130	3857530	-0.650923453	0.033499425	decrease
chr10	60778351	60778751	-0.645191562	0.033568531	decrease
chr16	67841947	67842347	-0.58836496	0.0335867	decrease
chr10	93438575	93438975	-0.650751132	0.033612748	decrease
chr1	224183646	224184046	-0.610081526	0.03363269	decrease
chr2	99141268	99141668	-0.671079172	0.033637801	decrease
chr7	25854752	25855152	-0.684286368	0.033691012	decrease
chr9	129489021	129489421	-0.671787385	0.033702526	decrease
chr6	170584664	170585064	-0.627383411	0.033707285	decrease
chr10	78407048	78407448	-0.672801641	0.033712981	decrease
chr17	81295626	81296026	-0.677531654	0.033744578	decrease
chr1	42846296	42846696	-0.626926522	0.033757179	decrease
chr4	20700136	20700536	-0.716535996	0.033789321	decrease
chr18	11980935	11981335	-0.652741044	0.03379232	decrease
chrX	63560761	63561161	-0.716834962	0.033792876	decrease
chr1	150010447	150010847	-0.67573711	0.03380052	decrease
chr11	12800199	12800599	-0.727537278	0.033803879	decrease
chr5	60945330	60945730	-0.639503999	0.033817469	decrease
chr15	75801926	75802326	-0.681683744	0.033819916	decrease
chr19	51926995	51927395	-0.63723919	0.033824813	decrease
chr6	63635009	63635409	-0.69778611	0.033863289	decrease
chr17	29622514	29622914	-0.717848318	0.033881082	decrease
chr3	28059194	28059594	-0.74299776	0.033888962	decrease
chr3	55620463	55620863	-0.698813033	0.033894441	decrease
chr1	11273122	11273522	-0.685195034	0.033920459	decrease
chr1	85582331	85582731	-0.640375241	0.033958881	decrease
chr19	11035821	11036221	-0.649882352	0.033973341	decrease
chr6	96924331	96924731	-0.69572607	0.033987213	decrease
chr7	150369732	150370132	-0.69795153	0.03401762	decrease
chr6	158799128	158799528	-0.703457836	0.034026233	decrease
chr21	26124608	26125008	-0.732574954	0.034088937	decrease
chr21	43810853	43811253	-0.52028355	0.034106366	decrease
chr1	74733253	74733653	-0.573160146	0.034118477	decrease
chr1	205631094	205631494	-0.686041731	0.034133106	decrease
chr11	1309603	1310003	-0.721196439	0.034140441	decrease
chr19	11435177	11435577	-0.509047449	0.034141059	decrease
chr1	226308910	226309310	-0.703214029	0.034150152	decrease
chr8	38012951	38013351	-0.717540814	0.034175981	decrease
chr3	10115406	10115806	-0.628510991	0.034182754	decrease
chr15	96327106	96327506	-0.673121026	0.034185331	decrease
chr1	239386264	239386664	-0.732563647	0.034194902	decrease
chr9	92115211	92115611	-0.625990211	0.034217593	decrease
chr4	153204756	153205156	-0.682228215	0.034239001	decrease
chr12	47779172	47779572	-0.658854468	0.03431494	decrease
chr12	1690599	1690999	-0.7151634	0.034319973	decrease
chr22	37643232	37643632	-0.59779277	0.034372273	decrease
chr1	111140202	111140602	-0.636125968	0.034394872	decrease
chr4	158810592	158810992	-0.579235216	0.034422398	decrease
chr5	132737327	132737727	-0.685283975	0.034445104	decrease
chr1	153977684	153978084	-0.675799649	0.034462602	decrease
chr11	8328834	8329234	-0.669900164	0.03446914	decrease

chr3	174441306	174441706	-0.717857269	0.034493935	decrease
chr1	198848898	198849298	-0.7274809	0.034520622	decrease
chr10	125662048	125662448	-0.69657809	0.034526126	decrease
chr11	120335429	120335829	-0.725247568	0.034528169	decrease
chr20	37693950	37694350	-0.697515003	0.034531672	decrease
chr16	68237432	68237832	-0.589816716	0.034585707	decrease
chrX	46545311	46545711	-0.702916793	0.034590008	decrease
chr9	22005528	22005928	-0.644684734	0.034628551	decrease
chr12	6470683	6471083	-0.593924083	0.034747359	decrease
chr8	143829362	143829762	-0.619475292	0.034858306	decrease
chr16	89732457	89732857	-0.58894632	0.034861045	decrease
chr8	686954	687354	-0.731967949	0.034863272	decrease
chr15	58749792	58750192	-0.726581895	0.034893923	decrease
chr7	100691753	100692153	-0.646243123	0.034919761	decrease
chr2	119759697	119760097	-0.63355287	0.034934966	decrease
chr1	201482001	201482401	-0.675914154	0.034937198	decrease
chr1	159900459	159900859	-0.716838516	0.034957141	decrease
chr19	58519717	58520117	-0.554042625	0.034970754	decrease
chr5	43313342	43313742	-0.604105245	0.035012677	decrease
chr12	46268652	46269052	-0.657783109	0.035014806	decrease
chr16	30407238	30407638	-0.611583576	0.035022132	decrease
chr9	15553008	15553408	-0.681462571	0.035036937	decrease
chr2	202588752	202589152	-0.7212786	0.035060238	decrease
chr15	73964731	73965131	-0.723006946	0.03506376	decrease
chr11	72822269	72822669	-0.650624456	0.035067794	decrease
chr3	17730397	17730797	-0.72019124	0.03508006	decrease
chrX	129843868	129844268	-0.626285278	0.035107615	decrease
chr3	53881797	53882197	-0.656561546	0.035123312	decrease
chrX	15737933	15738333	-0.62319988	0.035141086	decrease
chrX	47836379	47836779	-0.63783368	0.035143808	decrease
chr2	112481180	112481580	-0.673744552	0.035178106	decrease
chr18	22559768	22560168	-0.708759158	0.035191079	decrease
chr12	75334443	75334843	-0.670183246	0.035213686	decrease
chr8	125431695	125432095	-0.635977119	0.035216742	decrease
chr3	100260702	100261102	-0.637813647	0.035220158	decrease
chr7	30771345	30771745	-0.729986774	0.035253648	decrease
chr2	222298942	222299342	-0.639478475	0.03526551	decrease
chr17	7204896	7205296	-0.643654396	0.035270076	decrease
chr11	94973454	94973854	-0.679363191	0.035295365	decrease
chr12	122749763	122750163	-0.667548699	0.035316076	decrease
chr11	118792557	118792957	-0.684988463	0.03533059	decrease
chr5	141849923	141850323	-0.700632096	0.035370091	decrease
chr16	2047392	2047792	-0.586241071	0.035374795	decrease
chr1	234600712	234601112	-0.637273207	0.03538068	decrease
chr11	78574763	78575163	-0.625928001	0.035383751	decrease
chr7	5418689	5419089	-0.656803373	0.035428421	decrease
chr7	102363542	102363942	-0.649193466	0.035437036	decrease
chr10	5692713	5693113	-0.672383583	0.035445915	decrease
chr1	161119375	161119775	-0.659362312	0.035448522	decrease
chr17	73232222	73232622	-0.527802431	0.03545515	decrease
chr9	125473191	125473591	-0.707141785	0.035478258	decrease
chr1	77998385	77998785	-0.719806799	0.035480349	decrease
chr4	112515464	112515864	-0.730896694	0.035487041	decrease
chr13	21176498	21176898	-0.672417115	0.035605545	decrease
chr12	132133906	132134306	-0.610008575	0.035670696	decrease
chr2	231786713	231787113	-0.709977308	0.035672049	decrease
chr7	149196365	149196765	-0.609722827	0.035682872	decrease
chr21	29024580	29024980	-0.566773864	0.035712716	decrease
chr11	46346982	46347382	-0.722007413	0.03576372	decrease
chr6	157108303	157108703	-0.674950302	0.0357767	decrease
chr22	50244570	50244970	-0.546475285	0.035794822	decrease
chr3	24521238	24521638	-0.699268438	0.035827898	decrease
chr2	209496057	209496457	-0.620277699	0.035828739	decrease
chr17	82520930	82521330	-0.692066095	0.035835555	decrease
chr15	90930094	90930494	-0.578817689	0.035889837	decrease
chr13	71797343	71797743	-0.686565098	0.035916671	decrease
chr2	231243155	231243555	-0.711783266	0.035924783	decrease
chr2	169694326	169694726	-0.658735877	0.035942672	decrease
chr3	100492113	100492513	-0.650898116	0.035959819	decrease
chr10	126247571	126247971	-0.695504403	0.035966909	decrease
chr4	170089231	170089631	-0.679362928	0.035969531	decrease
chr20	63740901	63741301	-0.615606607	0.036004709	decrease
chr12	55927291	55927691	-0.676772991	0.036015329	decrease
chr3	25448928	25449328	-0.730806174	0.036019399	decrease
chr6	24403076	24403476	-0.689458045	0.03603825	decrease
chr19	35702228	35702628	-0.605750434	0.036048794	decrease

chr13	21140057	21140457	-0.729494634	0.036068717	decrease
chr2	219597855	219598255	-0.603902787	0.036177908	decrease
chr5	81971920	81972320	-0.737210933	0.036182127	decrease
chr20	2470728	2471128	-0.603162906	0.036192738	decrease
chr6	146913977	146914377	-0.697590016	0.036225836	decrease
chr12	121500040	121500440	-0.671912485	0.036241997	decrease
chr7	66493502	66493902	-0.5888544	0.036252226	decrease
chr6	158560845	158561245	-0.695097257	0.036318546	decrease
chr1	104671825	104672225	-0.649514431	0.036371573	decrease
chr16	67783046	67783446	-0.654595796	0.036387303	decrease
chr6	80106401	80106801	-0.601438771	0.036389955	decrease
chr3	195894748	195895148	-0.708562187	0.036408107	decrease
chr17	17913816	17914216	-0.661016314	0.036418098	decrease
chr8	91070059	91070459	-0.60102895	0.036422876	decrease
chr2	68467175	68467575	-0.700778913	0.03643434	decrease
chr4	159104604	159105004	-0.654585417	0.036440104	decrease
chr1	227730381	227730781	-0.535022941	0.036450993	decrease
chr21	36319852	36320252	-0.582411773	0.036477896	decrease
chr5	113487507	113487907	-0.680862392	0.036483726	decrease
chrX	106802342	106802742	-0.590638221	0.0365275	decrease
chr1	161227201	161227601	-0.607787061	0.036595691	decrease
chr11	16738437	16738837	-0.636736276	0.0366181	decrease
chr14	73058299	73058699	-0.649081067	0.036624204	decrease
chr12	93591436	93591836	-0.70630512	0.036624641	decrease
chr20	38926051	38926451	-0.700854305	0.036668838	decrease
chr19	9621080	9621480	-0.720787649	0.036685952	decrease
chr15	70892304	70892704	-0.594304166	0.036707717	decrease
chr19	8307389	8307789	-0.627763797	0.036715529	decrease
chr19	41310300	41310700	-0.638164104	0.036725591	decrease
chr1	234498969	234499369	-0.652736664	0.036741408	decrease
chr12	31659185	31659585	-0.655166036	0.036751293	decrease
chr13	33285471	33285871	-0.676926663	0.03686388	decrease
chr1	113979188	113979588	-0.684632815	0.036865522	decrease
chr9	97922225	97922625	-0.597604831	0.036913704	decrease
chr1	109960049	109960449	-0.693492699	0.03695639	decrease
chr17	51121538	51121938	-0.64578013	0.036963302	decrease
chr9	79256841	79257241	-0.557551518	0.036988086	decrease
chr1	50348797	50349197	-0.718501193	0.036993346	decrease
chr19	42220031	42220431	-0.558835883	0.036997767	decrease
chr5	113488470	113488870	-0.68169864	0.037013054	decrease
chr9	108934516	108934916	-0.680498842	0.037019807	decrease
chr7	148700603	148701003	-0.636851955	0.037034029	decrease
chrX	134460970	134461370	-0.719269693	0.037036772	decrease
chr16	89443323	89443723	-0.657631115	0.037039224	decrease
chr11	61871149	61871549	-0.685571316	0.037060924	decrease
chr1	81803326	81803726	-0.652277372	0.037076094	decrease
chr6	27495406	27495806	-0.686508769	0.037097193	decrease
chr6	163965706	163966106	-0.714774007	0.037128174	decrease
chr6	32666424	32666824	-0.664679832	0.037137237	decrease
chr8	81732411	81732811	-0.67654766	0.037138819	decrease
chr17	16217107	16217507	-0.665044519	0.037150377	decrease
chr9	125746081	125746481	-0.558626717	0.037167037	decrease
chr12	103956999	103957399	-0.586858208	0.037189527	decrease
chr19	4139871	4140271	-0.687308486	0.03723809	decrease
chrX	103356349	103356749	-0.666839943	0.03724349	decrease
chr1	244451907	244452307	-0.546872918	0.037246286	decrease
chr19	43754835	43755235	-0.585723573	0.037254947	decrease
chr6	138572307	138572707	-0.636544896	0.037268685	decrease
chr13	110715411	110715811	-0.653953304	0.037285236	decrease
chr5	178075940	178076340	-0.616876788	0.037291192	decrease
chr9	35790309	35790709	-0.697763648	0.037295425	decrease
chr22	46578331	46578731	-0.695989934	0.037307402	decrease
chr15	72230892	72231292	-0.618156751	0.037317631	decrease
chr11	877521	877921	-0.623060142	0.037322779	decrease
chr4	140287321	140287721	0.715909258	0.037327681	increase
chr1	59214756	59215156	-0.653895876	0.037335416	decrease
chr19	37210551	37210951	-0.709070581	0.037344217	decrease
chr17	64145730	64146130	-0.583037636	0.037346631	decrease
chr17	41930187	41930587	-0.609037112	0.037347322	decrease
chr14	105473872	105474272	-0.648798395	0.037349199	decrease
chr7	35800738	35801138	-0.640699362	0.037356575	decrease
chr2	187577070	187577470	-0.726377116	0.037378165	decrease
chr4	83536015	83536415	-0.694677202	0.037388025	decrease
chr19	12400955	12401355	-0.699501269	0.037408903	decrease
chr11	66592811	66593211	-0.609335832	0.037443099	decrease
chr14	67819483	67819883	-0.605842462	0.037456523	decrease

chr16	85461922	85462322	-0.655453567	0.037460216	decrease
chr12	122423370	122423770	-0.66980503	0.037498637	decrease
chrX	154097852	154098252	-0.691853054	0.037562034	decrease
chr16	30558132	30558532	-0.569982806	0.037564345	decrease
chr3	197949849	197950249	-0.635894661	0.037571191	decrease
chr20	36575003	36575403	-0.624717109	0.037591537	decrease
chr20	9067696	9068096	-0.605427938	0.037600035	decrease
chr17	21457220	21457620	-0.656194339	0.037600118	decrease
chr7	146635548	146635948	-0.708297463	0.037612044	decrease
chr3	101561920	101562320	-0.703361295	0.037618776	decrease
chr14	73125107	73125507	-0.694148093	0.037661762	decrease
chr5	151252775	151253175	-0.642604807	0.037686173	decrease
chr17	8127003	8127403	-0.623142104	0.037747173	decrease
chr5	73216180	73216580	-0.727814119	0.037795728	decrease
chr1	145891873	145892273	-0.583105492	0.037801028	decrease
chr11	125568969	125569369	-0.712776304	0.037810059	decrease
chr1	151146703	151147103	-0.583107002	0.037841626	decrease
chr11	18721287	18721687	-0.614009484	0.037872868	decrease
chr7	139133540	139133940	-0.620097047	0.037921956	decrease
chr15	100341950	100342350	-0.652160145	0.037929281	decrease
chr1	203273150	203273550	-0.616768116	0.038036548	decrease
chr20	37178754	37179154	-0.540640372	0.038051489	decrease
chr15	41557810	41558210	-0.719944765	0.038115345	decrease
chr10	32980444	32980844	-0.689572049	0.038175506	decrease
chr7	120950864	120951264	-0.665975905	0.038179902	decrease
chr20	62392729	62393129	-0.631098649	0.038202118	decrease
chr8	88327727	88328127	-0.626725514	0.038245048	decrease
chr13	40664213	40664613	-0.682207728	0.038273855	decrease
chr22	43812284	43812684	-0.661017975	0.038287582	decrease
chr8	42052158	42052558	-0.686504609	0.038300824	decrease
chr11	76381168	76381568	-0.690282058	0.038308761	decrease
chr16	1839540	1839940	-0.683023263	0.03831111	decrease
chr13	79376655	79377055	-0.693424025	0.038345688	decrease
chr9	93468392	93468792	-0.710488245	0.038351734	decrease
chr22	39496229	39496629	-0.616799652	0.038383351	decrease
chr6	47309993	47310393	-0.723099372	0.038386753	decrease
chr1	94854388	94854788	-0.66517715	0.038408839	decrease
chr19	30004101	30004501	-0.699577471	0.038410583	decrease
chr18	12377472	12377872	-0.613182362	0.038443712	decrease
chr13	43879159	43879559	-0.721467585	0.038444637	decrease
chr4	3494293	3494693	-0.621280684	0.038463557	decrease
chr4	25160264	25160664	-0.699373736	0.038502168	decrease
chr2	158967655	158968055	-0.636158034	0.038523317	decrease
chr19	17309309	17309709	-0.557537344	0.038526948	decrease
chr13	64723731	64724131	-0.628585203	0.038542147	decrease
chr15	82750284	82750684	-0.594759201	0.038543934	decrease
chrX	100636489	100636889	-0.602961041	0.038552406	decrease
chr13	71979470	71979870	-0.699721915	0.038589827	decrease
chr7	151759594	151759994	-0.661337931	0.038607818	decrease
chr19	5977981	5978381	-0.650950947	0.038627524	decrease
chr1	224457086	224457486	-0.626099545	0.038635012	decrease
chr6	132798924	132799324	-0.681254597	0.038684741	decrease
chr3	158723343	158723743	-0.677635108	0.038685374	decrease
chr19	48629757	48630157	-0.64144507	0.038759334	decrease
chr16	29790433	29790833	-0.548338372	0.038768825	decrease
chr12	110269965	110270365	-0.69512159	0.038790106	decrease
chr11	64924142	64924542	-0.555298296	0.038800841	decrease
chr3	88149841	88150241	-0.641452748	0.038809217	decrease
chr9	38392625	38393025	-0.616004333	0.038810682	decrease
chr9	122913100	122913500	-0.654738625	0.03883717	decrease
chr8	144852866	144853266	-0.596668712	0.038839182	decrease
chr12	108856500	108856900	-0.66172849	0.038844606	decrease
chr5	126594975	126595375	-0.603188535	0.038859249	decrease
chr2	208254957	208255357	-0.604314335	0.038862326	decrease
chr10	78043418	78043818	-0.614656125	0.038882836	decrease
chr5	122076768	122077168	-0.639714493	0.038891486	decrease
chr21	34129141	34129541	-0.583826812	0.03889601	decrease
chr10	58513362	58513762	-0.676535121	0.038917165	decrease
chr2	46298930	46299330	-0.66377059	0.03892082	decrease
chr5	90558461	90558861	-0.531344536	0.038936059	decrease
chr1	180502649	180503049	-0.619241587	0.03894593	decrease
chr1	112715734	112716134	-0.587862009	0.038987363	decrease
chr12	47963226	47963626	-0.698497415	0.038989499	decrease
chr12	56104182	56104582	-0.531904889	0.039013946	decrease
chr11	65487660	65488060	-0.54685522	0.039015292	decrease
chr11	17598822	17599222	-0.711051469	0.03904042	decrease

chr10	26697041	26697441	-0.715724173	0.039047735	decrease
chr13	99498586	99498986	-0.585360991	0.039056603	decrease
chr10	50067597	50067997	-0.686266356	0.039057085	decrease
chr1	53326110	53326510	-0.573748628	0.03906459	decrease
chr5	69093733	69094133	-0.687705414	0.039066305	decrease
chr9	128322433	128322833	-0.610656746	0.039125549	decrease
chr20	35539928	35540328	-0.682901072	0.039160968	decrease
chrX	120629761	120630161	-0.64844394	0.039163516	decrease
chr7	17940362	17940762	-0.6417276	0.039258904	decrease
chr9	38362969	38363369	-0.693786209	0.039280436	decrease
chr4	185925929	185926329	-0.686426687	0.039293306	decrease
chr2	210002393	210002793	-0.618765236	0.039315516	decrease
chr7	30027875	30028275	-0.676117554	0.039360369	decrease
chr16	58129417	58129817	-0.591871028	0.03936426	decrease
chr19	57351229	57351629	-0.656658128	0.039386065	decrease
chr19	51394266	51394666	-0.695080666	0.039402577	decrease
chr11	65858319	65858719	-0.558670422	0.039418093	decrease
chr13	69828565	69828965	-0.605666221	0.039466916	decrease
chr1	63746122	63746522	-0.641380502	0.039471714	decrease
chr9	19408887	19409287	-0.697053257	0.039479926	decrease
chr10	90930459	90930859	-0.661907435	0.039498861	decrease
chr8	144315820	144316220	-0.561915994	0.039516218	decrease
chr12	50056085	50056485	-0.669356241	0.039518723	decrease
chr4	75990640	75991040	-0.684239229	0.039526864	decrease
chr4	8428299	8428699	-0.640195808	0.039530369	decrease
chr17	3668461	3668861	-0.616485664	0.039551938	decrease
chr11	47118972	47119372	-0.686330079	0.039561395	decrease
chrX	14029343	14029743	-0.69232687	0.039592613	decrease
chr2	85327998	85328398	-0.668961331	0.03959715	decrease
chr13	75173693	75174093	-0.60959357	0.03960431	decrease
chrX	101623182	101623582	-0.699360212	0.039626713	decrease
chr11	64743637	64744037	-0.607499695	0.039639706	decrease
chr10	120127923	120128323	-0.684537222	0.039669623	decrease
chr12	47715397	47715797	-0.693916113	0.039671631	decrease
chr7	6577369	6577769	-0.616391391	0.039678684	decrease
chr4	103075977	103076377	-0.658501014	0.039690975	decrease
chr4	114616079	114616479	-0.703630653	0.039741392	decrease
chr9	105244073	105244473	-0.697484009	0.039758234	decrease
chr14	21384154	21384554	-0.703445799	0.039770446	decrease
chr18	47150315	47150715	-0.69786767	0.039794406	decrease
chr2	152095855	152096255	-0.655510395	0.039946284	decrease
chr1	211579709	211580109	-0.675753093	0.03996742	decrease
chr6	98947039	98947439	-0.692525941	0.039973051	decrease
chr2	112054329	112054729	-0.61978345	0.039979301	decrease
chr7	106661010	106661410	-0.706891784	0.039980017	decrease
chr2	44361557	44361957	-0.622235701	0.039988652	decrease
chr5	157995038	157995438	-0.717461962	0.04001606	decrease
chr2	172098091	172098491	-0.674404158	0.040047318	decrease
chr2	169584400	169584800	-0.644619353	0.040051494	decrease
chr15	84928110	84928510	-0.677681395	0.040054639	decrease
chr9	127979157	127979557	-0.671396907	0.040056384	decrease
chr19	48513708	48514108	-0.641864053	0.040102601	decrease
chr7	73725748	73726148	-0.618309366	0.040119743	decrease
chr15	60478875	60479275	-0.68114467	0.040128832	decrease
chr17	7214691	7215091	-0.484435894	0.040147451	decrease
chr17	59786633	59787033	-0.708103754	0.040149869	decrease
chr9	78296911	78297311	-0.586698006	0.040154471	decrease
chr9	35103198	35103598	-0.578301014	0.040164562	decrease
chr1	94417900	94418300	-0.54918844	0.040170925	decrease
chr19	47600249	47600649	-0.607010779	0.040197441	decrease
chr2	210171404	210171804	-0.666847435	0.040205038	decrease
chr11	62862536	62862936	-0.609191116	0.040228772	decrease
chr1	223837331	223837731	-0.647372042	0.040237283	decrease
chr12	1576330	1576730	-0.635338087	0.040250465	decrease
chrX	118588774	118589174	-0.680266347	0.040266262	decrease
chr1	29283154	29283554	-0.629557499	0.040299046	decrease
chr17	48024929	48025329	-0.683761487	0.040316969	decrease
chr19	43619563	43619963	-0.675719421	0.040360257	decrease
chr21	46635552	46635952	-0.723527681	0.040364899	decrease
chr6	137589408	137589808	-0.682095062	0.040366582	decrease
chr19	35693609	35694009	-0.679487959	0.040399464	decrease
chr19	14496180	14496580	-0.608582826	0.040415672	decrease
chr2	46916016	46916416	-0.652495485	0.040424205	decrease
chr2	173354507	173354907	-0.674483206	0.040484978	decrease
chr1	215569484	215569884	-0.665910952	0.040492192	decrease
chr7	144835977	144836377	-0.677852526	0.040494462	decrease

chrX	149630197	149630597	-0.632700821	0.040538053	decrease
chr1	226272325	226272725	-0.507869057	0.040538764	decrease
chrX	150363736	150364136	-0.704069571	0.040565041	decrease
chr11	63681349	63681749	-0.65736623	0.040589724	decrease
chr22	37817748	37818148	-0.6696028	0.040589973	decrease
chr2	75200124	75200524	-0.69940171	0.040610876	decrease
chr11	12287732	12288132	-0.66682964	0.04061409	decrease
chr7	148941383	148941783	-0.593177319	0.040633512	decrease
chr6	27823842	27824242	-0.6125557	0.040641313	decrease
chr19	40810755	40811155	-0.650121004	0.040662118	decrease
chr21	36567241	36567641	-0.627732	0.040686218	decrease
chr12	92144190	92144590	-0.58805324	0.04072438	decrease
chr12	122266445	122266845	-0.589145783	0.040736615	decrease
chr3	98593696	98594096	-0.621264165	0.040752066	decrease
chr13	37047029	37047429	-0.676016872	0.04079044	decrease
chr9	21974877	21975277	-0.688413427	0.040825622	decrease
chr8	38503127	38503527	-0.711232932	0.040838438	decrease
chr2	219509634	219510034	-0.658023365	0.040852991	decrease
chr5	34915263	34915663	-0.546986092	0.040882836	decrease
chr13	29850338	29850738	-0.709346725	0.040893659	decrease
chr13	77919341	77919741	-0.588843449	0.040907163	decrease
chr12	21527656	21528056	-0.699173422	0.040918228	decrease
chr3	40505995	40506395	-0.705309861	0.040922167	decrease
chr3	51991052	51991452	-0.60001242	0.040943022	decrease
chr1	109426357	109426757	-0.543874326	0.040956097	decrease
chr19	29694514	29694914	-0.662764447	0.040976983	decrease
chr15	67521584	67521984	-0.648760905	0.040980057	decrease
chr12	122225831	122226231	-0.476733056	0.040995512	decrease
chr19	1259464	1259864	-0.574828925	0.041007733	decrease
chr13	79791418	79791818	-0.692486675	0.041022212	decrease
chr5	119299607	119300007	-0.663884957	0.041028414	decrease
chr14	64540295	64540695	-0.633396137	0.041067297	decrease
chr14	36835138	36835538	-0.651849492	0.041075135	decrease
chr20	31879743	31880143	-0.705575386	0.041149402	decrease
chr3	159763976	159764376	-0.672168964	0.041151777	decrease
chr7	146116665	146117065	-0.701758977	0.041185616	decrease
chr17	80455077	80455477	-0.657292034	0.041205514	decrease
chr1	47192776	47193176	-0.686524363	0.041245078	decrease
chr8	46617174	46617574	-0.664280164	0.041263372	decrease
chr3	79767556	79767956	-0.701590641	0.041267375	decrease
chr5	147782567	147782967	-0.646825226	0.041268062	decrease
chr10	120978979	120979379	-0.608320079	0.041278429	decrease
chr8	37762340	37762740	-0.688675556	0.041326352	decrease
chr3	10320852	10321252	-0.624923428	0.041326434	decrease
chr2	96532075	96532475	-0.601736423	0.041343587	decrease
chr5	54310369	54310769	-0.682975063	0.041425011	decrease
chr19	56393286	56393686	-0.676284377	0.041487975	decrease
chr7	157273528	157273928	-0.570956173	0.041498197	decrease
chr5	149320885	149321285	-0.685812116	0.041517144	decrease
chr17	48830885	48831285	-0.603103592	0.041558648	decrease
chr1	109643275	109643675	-0.656618253	0.041566277	decrease
chr9	112717705	112718105	-0.593870874	0.041590336	decrease
chr22	37601575	37601975	-0.696083333	0.041594138	decrease
chr9	95312983	95313383	-0.64368287	0.04164443	decrease
chr1	202810751	202811151	-0.626251916	0.041650545	decrease
chr4	139301009	139301409	-0.575509515	0.041659845	decrease
chr6	83430168	83430568	-0.670543599	0.041692146	decrease
chr11	124800429	124800829	-0.635388439	0.041692701	decrease
chr19	19192423	19192823	-0.538497352	0.041697057	decrease
chr4	112817893	112818293	-0.625865574	0.041724478	decrease
chr11	92838801	92839201	-0.683905834	0.041773854	decrease
chr11	126115856	126116256	-0.610742586	0.041816841	decrease
chr6	46170659	46171059	-0.656297772	0.041829087	decrease
chr5	147946676	147947076	-0.638060287	0.041839967	decrease
chr14	102304935	102305335	-0.64331229	0.041858888	decrease
chr17	70168725	70169125	-0.704268545	0.041875553	decrease
chr12	132827881	132828281	0.690241034	0.041915711	increase
chr12	93442233	93442633	-0.629939199	0.041979242	decrease
chr13	110706340	110706740	-0.598379283	0.041987909	decrease
chr5	34007848	34008248	-0.633965029	0.041999475	decrease
chr7	66741119	66741519	-0.642602446	0.04204113	decrease
chr14	103562919	103563319	-0.702999159	0.042047984	decrease
chr2	131153938	131154338	-0.669428411	0.042065425	decrease
chr11	70398113	70398513	-0.64173973	0.042130437	decrease
chr17	48101136	48101536	-0.662957531	0.042152097	decrease
chr12	30753528	30753928	-0.698481561	0.042196607	decrease

chr14	34700110	34700510	-0.679824523	0.042211896	decrease
chr2	113204034	113204434	-0.666257489	0.042225177	decrease
chr1	92080331	92080731	-0.58448283	0.04222584	decrease
chr2	64524144	64524544	-0.676258535	0.042226293	decrease
chr11	134034043	134034443	-0.582934842	0.042226517	decrease
chr7	39949383	39949783	-0.575935792	0.042252112	decrease
chr9	23826196	23826596	-0.665230589	0.042263663	decrease
chr19	55476863	55477263	-0.629740668	0.042273241	decrease
chr17	7650222	7650622	-0.678145168	0.042295009	decrease
chr1	81032799	81033199	-0.6267702	0.042298004	decrease
chr16	77409386	77409786	-0.688108204	0.042365028	decrease
chr3	53163122	53163522	-0.613966872	0.042371875	decrease
chrX	102659939	102660339	-0.701556826	0.042406034	decrease
chr3	126703821	126704221	-0.581234886	0.042451785	decrease
chr1	52366171	52366571	-0.602906789	0.042505021	decrease
chr9	34620324	34620724	-0.663726432	0.04251569	decrease
chr19	1095260	1095660	-0.580543431	0.042535118	decrease
chr2	196639464	196639864	-0.572964261	0.042535568	decrease
chrX	15493292	15493692	-0.681401091	0.042563908	decrease
chr12	132828797	132829197	-0.69152516	0.042619856	decrease
chr10	100878439	100878839	-0.682215906	0.042622546	decrease
chr21	42850831	42851231	-0.567752065	0.042622621	decrease
chr14	81219249	81219649	-0.643791442	0.042633394	decrease
chr13	28658812	28659212	-0.622754856	0.042643757	decrease
chr7	149285170	149285570	-0.655961424	0.042687354	decrease
chr12	75511468	75511868	-0.640877461	0.04269186	decrease
chr3	9396799	9397199	-0.504425251	0.042714224	decrease
chr1	54407581	54407981	-0.626183712	0.04272401	decrease
chr14	74251636	74252036	-0.665156603	0.042735827	decrease
chr5	131312461	131312861	-0.641615575	0.042740087	decrease
chr10	122980311	122980711	-0.620529869	0.042740543	decrease
chr2	127294138	127294538	-0.667143694	0.042753351	decrease
chr2	37670947	37671347	-0.69688752	0.042765929	decrease
chr13	63189963	63190363	-0.704244628	0.042768401	decrease
chr7	35694870	35695270	-0.647060681	0.042817707	decrease
chrX	40145437	40145837	-0.677628441	0.042829745	decrease
chr10	100518780	100519180	-0.674980112	0.042842049	decrease
chr1	36188025	36188425	-0.596780194	0.042867171	decrease
chr17	81508439	81508839	-0.480014708	0.042873361	decrease
chr11	65134576	65134976	-0.501941511	0.042918244	decrease
chr15	74835369	74835769	-0.595771257	0.042946747	decrease
chr22	31977099	31977499	-0.703271021	0.043000786	decrease
chr1	93878816	93879216	-0.583667231	0.043014591	decrease
chr16	10743802	10744202	-0.682046232	0.043071371	decrease
chr19	1652895	1653295	-0.670483167	0.043142063	decrease
chr3	99817801	99818201	-0.598054507	0.0431452	decrease
chr19	43575442	43575842	-0.624512064	0.043149646	decrease
chr11	65712270	65712670	-0.533236629	0.043157453	decrease
chr1	212595942	212596342	-0.668556947	0.043161998	decrease
chr1	235107958	235108358	-0.577538062	0.043163711	decrease
chrX	153226393	153226793	-0.663017463	0.043177469	decrease
chr10	50622553	50622953	-0.623712759	0.04320003	decrease
chr14	105300979	105301379	-0.586643399	0.043206626	decrease
chrX	123961452	123961852	-0.57317774	0.043246166	decrease
chr3	126982499	126982899	-0.64775272	0.043269302	decrease
chr16	28846313	28846713	-0.525214744	0.043272458	decrease
chr22	28882730	28883130	-0.651677924	0.043279471	decrease
chr20	44966406	44966806	-0.652984149	0.043356158	decrease
chr19	52923178	52923578	-0.665593789	0.043420878	decrease
chr10	102462946	102463346	-0.622829295	0.043432841	decrease
chr6	56955453	56955853	-0.652828908	0.043481773	decrease
chr1	209784345	209784745	-0.621531704	0.043509376	decrease
chr9	137459602	137460002	-0.604217459	0.043510071	decrease
chr4	19823794	19824194	-0.691027499	0.043553991	decrease
chr2	38751233	38751633	-0.566136987	0.043571287	decrease
chr1	28505621	28506021	-0.552450727	0.043588041	decrease
chr4	153222688	153223088	-0.687017829	0.043605178	decrease
chr1	204073020	204073420	-0.684125888	0.043606685	decrease
chr2	221573107	221573507	-0.652138524	0.043609794	decrease
chr17	77374293	77374693	-0.55468842	0.04362844	decrease
chr16	4271451	4271851	-0.611670891	0.04363185	decrease
chr19	48872465	48872865	-0.604878217	0.043648445	decrease
chr4	97037792	97038192	-0.689944492	0.043666624	decrease
chr19	17075179	17075579	-0.592003251	0.043674111	decrease
chr13	68005109	68005509	0.675930145	0.043691122	increase
chr8	65842350	65842750	-0.698092197	0.043706514	decrease

chr1	212558996	212559396	-0.682384218	0.043716149	decrease
chr15	90319907	90320307	-0.650807095	0.043905485	decrease
chr12	132762300	132762700	-0.621392639	0.043938106	decrease
chr10	91923498	91923898	-0.680388996	0.043941074	decrease
chr9	87725816	87726216	-0.615972959	0.043978939	decrease
chr5	136133306	136133706	-0.707451427	0.043985709	decrease
chr5	43192733	43193133	-0.69207185	0.04404727	decrease
chr8	79762773	79763173	-0.638846735	0.044064356	decrease
chr19	42295997	42296397	-0.563238839	0.044108963	decrease
chr2	216412391	216412791	-0.552084849	0.044120359	decrease
chr2	182716082	182716482	-0.670404591	0.044125085	decrease
chr12	93677562	93677962	-0.566847489	0.044139662	decrease
chr8	89901979	89902379	-0.681553813	0.044154397	decrease
chr19	3852711	3853111	-0.647182315	0.044175757	decrease
chr1	167926905	167927305	-0.662136955	0.044183267	decrease
chr21	41612471	41612871	-0.674512771	0.044235964	decrease
chr14	75259043	75259443	-0.570619899	0.044262604	decrease
chr20	10034933	10035333	-0.689157089	0.044270133	decrease
chr21	32612327	32612727	-0.528566552	0.044279037	decrease
chr12	6336373	6336773	-0.529280508	0.044330838	decrease
chr11	11841818	11842218	-0.610793411	0.044332673	decrease
chr10	104338426	104338826	-0.663535438	0.044339384	decrease
chr12	79935027	79935427	-0.547434436	0.044357058	decrease
chr12	56158961	56159361	-0.593832367	0.044366133	decrease
chr7	97024321	97024721	-0.622020216	0.044369898	decrease
chr1	148264766	148265166	-0.613149411	0.044371185	decrease
chr6	153002808	153003208	-0.56378495	0.044374607	decrease
chr6	125906351	125906751	-0.620073278	0.04439008	decrease
chr19	38831519	38831919	-0.62556298	0.044435015	decrease
chr20	45891202	45891602	-0.627093361	0.044439577	decrease
chr15	22838204	22838604	-0.621568231	0.044459985	decrease
chr12	93467447	93467847	-0.626065659	0.044479764	decrease
chr16	4693240	4693640	-0.574535329	0.044479773	decrease
chr14	20332897	20333297	-0.63972746	0.044541895	decrease
chr12	67067832	67068232	-0.697715037	0.044569145	decrease
chr5	19885487	19885887	-0.700353908	0.044600696	decrease
chr3	45841759	45842159	-0.673477138	0.044689008	decrease
chr10	73110811	73111211	-0.62022554	0.044692708	decrease
chr14	49620377	49620777	-0.574039452	0.044772856	decrease
chr15	84570842	84571242	-0.518443874	0.044781774	decrease
chr4	152536178	152536578	-0.591868449	0.044791998	decrease
chr19	17606244	17606644	-0.692942377	0.044800056	decrease
chr1	37690336	37690736	-0.582271343	0.04481513	decrease
chr17	81002161	81002561	-0.639925925	0.044858214	decrease
chr5	112160628	112161028	-0.636758075	0.044868625	decrease
chr10	96129760	96130160	-0.575971903	0.044899768	decrease
chr6	166032783	166033183	-0.670716393	0.044902505	decrease
chr6	113970009	113970409	-0.598450185	0.044949098	decrease
chr10	50739732	50740132	-0.646282805	0.045020594	decrease
chr15	65384973	65385373	-0.501899753	0.045035798	decrease
chr10	91409522	91409922	-0.596518506	0.045043101	decrease
chr17	5468655	5469055	-0.608151404	0.045096377	decrease
chr11	57761488	57761888	-0.663964974	0.045123389	decrease
chr5	74767091	74767491	-0.562598165	0.04515064	decrease
chr19	53980460	53980860	-0.502215243	0.04515125	decrease
chr1	156160762	156161162	-0.677443131	0.045171749	decrease
chr19	4124052	4124452	-0.681541392	0.045189892	decrease
chr19	7870895	7871295	-0.571994179	0.045199978	decrease
chr1	15835360	15835760	-0.637954062	0.045229086	decrease
chr6	44387478	44387878	-0.539337613	0.045232683	decrease
chr15	80989406	80989806	-0.630001688	0.045234859	decrease
chr21	34030024	34030424	-0.655834105	0.045272595	decrease
chr2	180034148	180034548	-0.642639171	0.045288992	decrease
chrX	103342943	103343343	-0.677482383	0.045368977	decrease
chr5	179732430	179732830	-0.559350567	0.045400819	decrease
chr8	124474330	124474730	-0.575026669	0.045425932	decrease
chr12	51238924	51239324	-0.596027764	0.045428639	decrease
chr7	72924521	72924921	-0.676267328	0.045431736	decrease
chr19	35557907	35558307	-0.672485525	0.045442582	decrease
chr20	32357930	32358330	-0.518133048	0.045472945	decrease
chr12	49043830	49044230	-0.630441228	0.045482927	decrease
chr6	106629336	106629736	-0.633074289	0.045494897	decrease
chr18	267661	268061	-0.657507539	0.045546681	decrease
chr1	32448413	32448813	-0.521979578	0.045554555	decrease
chr1	151175835	151176235	-0.624195643	0.045596394	decrease
chr19	2427589	2427989	-0.494420172	0.045621886	decrease

chr2	71282370	71282770	-0.672545097	0.045636058	decrease
chr15	67840030	67840430	-0.636893509	0.045680425	decrease
chr17	27686276	27686676	-0.690169228	0.045690655	decrease
chr10	102055609	102056009	-0.678977186	0.045713171	decrease
chr11	329474	329874	-0.676615371	0.045734695	decrease
chr22	29838139	29838539	-0.590473595	0.04573748	decrease
chr16	85753004	85753404	-0.657208847	0.045777259	decrease
chrX	49073714	49074114	-0.632161798	0.045790666	decrease
chr5	126600539	126600939	-0.683892967	0.045802253	decrease
chr21	32569656	32570056	-0.579950425	0.045817172	decrease
chr6	125780492	125780892	-0.574571512	0.045840864	decrease
chr13	44141824	44142224	-0.62330015	0.045852521	decrease
chr3	50299307	50299707	-0.489337412	0.045871323	decrease
chr10	22436910	22437310	-0.67229312	0.045882659	decrease
chr3	53254753	53255153	-0.589826675	0.04595715	decrease
chrX	110004210	110004610	-0.644223657	0.045990489	decrease
chr19	4172768	4173168	-0.660185339	0.046000014	decrease
chr10	43265835	43266235	-0.689389027	0.046018093	decrease
chr5	81301480	81301880	-0.562768729	0.046020667	decrease
chr2	96146976	96147376	-0.65614896	0.046020829	decrease
chrX	104156719	104157119	-0.543256747	0.046067497	decrease
chr1	185044986	185045386	-0.689744723	0.046100681	decrease
chr17	80035897	80036297	-0.662517884	0.046129108	decrease
chr17	21551077	21551477	-0.602535065	0.04614196	decrease
chr17	7014408	7014808	-0.59261134	0.046197169	decrease
chr7	149872793	149873193	-0.644714986	0.046204815	decrease
chr1	91500725	91501125	-0.507229765	0.04622368	decrease
chr8	56074492	56074892	-0.635258996	0.046240768	decrease
chr1	45491146	45491546	-0.566105283	0.046244564	decrease
chr12	10363988	10364388	-0.596939511	0.04633095	decrease
chr5	138213441	138213841	-0.554968312	0.046344163	decrease
chr21	32728024	32728424	-0.606634326	0.046344728	decrease
chr9	136849298	136849698	-0.59894011	0.04636386	decrease
chr19	48624103	48624503	-0.513882456	0.046372543	decrease
chr1	52703002	52703402	-0.586418973	0.046376119	decrease
chr7	149065460	149065860	-0.552278468	0.046380614	decrease
chr11	791007	791407	-0.572797195	0.046404575	decrease
chr11	123063234	123063634	-0.63148927	0.046435127	decrease
chr9	105901315	105901715	-0.692239135	0.046445268	decrease
chr3	25783672	25784072	-0.675645243	0.046458933	decrease
chr15	35545604	35546004	-0.693888998	0.046470051	decrease
chr1	225480789	225481189	-0.588746139	0.046489569	decrease
chr2	215311735	215312135	-0.692035237	0.046491428	decrease
chr12	69484495	69484895	-0.644125344	0.046504799	decrease
chr16	9091243	9091643	-0.603612787	0.046507622	decrease
chr10	21516009	21516409	-0.660200326	0.046553964	decrease
chr16	67806614	67807014	-0.511285132	0.046568824	decrease
chr1	155609848	155610248	-0.580296035	0.046582068	decrease
chr1	86396002	86396402	-0.643044084	0.046613117	decrease
chr18	62187058	62187458	-0.636095562	0.046615302	decrease
chr1	77779749	77780149	-0.60822165	0.046646922	decrease
chr1	120942446	120942846	-0.695887873	0.046656689	decrease
chr1	160101218	160101618	-0.642093586	0.0466659	decrease
chr8	144290342	144290742	-0.654439004	0.046674035	decrease
chr3	48151650	48152050	-0.630354707	0.046677889	decrease
chr1	43368099	43368499	-0.656747502	0.046691218	decrease
chr1	167716360	167716760	-0.605488176	0.046715347	decrease
chr19	21141922	21142322	-0.645604188	0.046716699	decrease
chr2	233566408	233566808	-0.640261961	0.046727584	decrease
chr12	56221772	56222172	-0.503667506	0.046736619	decrease
chr2	42168971	42169371	-0.687372012	0.046755195	decrease
chr6	20401334	20401734	-0.556179616	0.046762069	decrease
chr11	33700821	33701221	-0.634386526	0.046770812	decrease
chr17	81230622	81231022	-0.619229184	0.046805783	decrease
chr11	114035838	114036238	-0.684239421	0.046812457	decrease
chr21	32393058	32393458	-0.569957536	0.046833858	decrease
chr3	38496076	38496476	-0.625962419	0.046858327	decrease
chr9	134024720	134025120	-0.676679907	0.0468797	decrease
chr19	8324381	8324781	-0.561861828	0.046891711	decrease
chr15	82262535	82262935	-0.558910771	0.046896475	decrease
chr13	72839700	72840100	-0.676880195	0.046906184	decrease
chr5	134145953	134146353	-0.678607977	0.046911832	decrease
chr16	85610583	85610983	-0.651040542	0.046912775	decrease
chr9	133352025	133352425	-0.571103344	0.046921344	decrease
chr4	1746147	1746547	-0.546980077	0.046921464	decrease
chr11	27464346	27464746	-0.663191714	0.046950078	decrease

chr12	53998192	53998592	-0.643140978	0.046958972	decrease
chr1	43349346	43349746	-0.650584505	0.047009224	decrease
chr19	11559201	11559601	-0.65730447	0.047010019	decrease
chr12	133037152	133037552	-0.695980555	0.047107046	decrease
chr7	130373518	130373918	-0.635901885	0.047173255	decrease
chr22	41763042	41763442	-0.673683246	0.047176382	decrease
chr18	31931936	31932336	-0.61814786	0.047186678	decrease
chr19	58305128	58305528	-0.570763899	0.047207221	decrease
chr12	57520289	57520689	-0.482880075	0.047211863	decrease
chr3	142888220	142888620	-0.675798712	0.047229053	decrease
chr7	22854311	22854711	-0.694816376	0.047255124	decrease
chr13	58949542	58949942	-0.697580594	0.047270423	decrease
chr17	44947431	44947831	-0.612052039	0.047300572	decrease
chr1	244155329	244155729	-0.662685521	0.047319647	decrease
chr18	76492464	76492864	-0.652097804	0.047373879	decrease
chr6	43769708	43770108	-0.63963155	0.04737465	decrease
chr11	8033280	8033680	-0.644513648	0.047405848	decrease
chr1	109239662	109240062	-0.628614203	0.04741976	decrease
chr19	21396946	21397346	-0.67303595	0.047420046	decrease
chr17	39451061	39451461	-0.606314687	0.047496082	decrease
chr15	76345579	76345979	-0.674036608	0.047537497	decrease
chr11	65524491	65524891	-0.666793543	0.047565525	decrease
chr11	65471804	65472204	-0.606919532	0.047600067	decrease
chr19	42387142	42387542	-0.592551611	0.047613648	decrease
chr14	96888594	96888994	-0.587343067	0.047644785	decrease
chr16	30775294	30775694	-0.469382507	0.047698414	decrease
chr12	120195123	120195523	-0.533862715	0.047734046	decrease
chr5	81851668	81852068	-0.651710933	0.047734777	decrease
chr3	48535885	48536285	-0.678982889	0.047744782	decrease
chr12	29380717	29381117	-0.602929877	0.047746725	decrease
chr11	102747036	102747436	-0.681153608	0.04778868	decrease
chr2	171922487	171922887	-0.625204924	0.047843761	decrease
chr17	35567916	35568316	-0.564731865	0.047893862	decrease
chr3	47380890	47381290	-0.514718065	0.047924738	decrease
chr7	30504705	30505105	-0.521174632	0.047933	decrease
chr22	17368573	17368973	-0.620242603	0.04795673	decrease
chr7	88220045	88220445	-0.650380256	0.047975353	decrease
chr16	29995962	29996362	-0.463767029	0.048016046	decrease
chr4	52712080	52712480	-0.559218909	0.048057785	decrease
chrX	137566414	137566814	-0.682051382	0.0481019	decrease
chrX	101390751	101391151	-0.516174873	0.048121901	decrease
chr4	140427349	140427749	-0.636179228	0.048146042	decrease
chr5	151080261	151080661	-0.659202422	0.048165582	decrease
chr13	64650124	64650524	0.68651208	0.048174132	increase
chr7	151876324	151876724	-0.589327482	0.048176383	decrease
chr7	157387015	157387415	-0.631485831	0.048193801	decrease
chr17	42763453	42763853	-0.673264761	0.048205257	decrease
chr10	100286505	100286905	-0.59893259	0.048212511	decrease
chr1	110312598	110312998	-0.627929428	0.048219399	decrease
chr6	89819391	89819791	-0.60101576	0.048230725	decrease
chrX	135051865	135052265	-0.542664121	0.048249022	decrease
chr4	97633793	97634193	-0.666695183	0.048250183	decrease
chr16	3182695	3183095	-0.642928037	0.048267628	decrease
chr1	2231676	2232076	-0.558126367	0.048268739	decrease
chr13	67736694	67737094	-0.57248507	0.048273077	decrease
chr17	2030397	2030797	-0.583490301	0.048285109	decrease
chr17	5486438	5486838	-0.585392574	0.048337595	decrease
chr3	16264650	16265050	-0.52282832	0.048352134	decrease
chr3	186825536	186825936	-0.670740396	0.048352817	decrease
chr6	87155266	87155666	-0.495686607	0.048381508	decrease
chr9	137445374	137445774	-0.627223466	0.048399929	decrease
chr15	65517379	65517779	-0.498025324	0.048462818	decrease
chr14	73950122	73950522	-0.480660725	0.048473673	decrease
chr12	48681949	48682349	-0.560964056	0.048475403	decrease
chr6	133240593	133240993	-0.634100211	0.048499061	decrease
chr8	42152607	42153007	-0.612898202	0.048537566	decrease
chr4	57163668	57164068	-0.643552978	0.048571992	decrease
chr13	39655583	39655983	-0.67370832	0.048641021	decrease
chr1	212791539	212791939	-0.601606731	0.048649562	decrease
chr1	59296587	59296987	-0.575248283	0.048696481	decrease
chr12	121686917	121687317	-0.552346347	0.048751844	decrease
chr9	130712622	130713022	-0.59209388	0.048758338	decrease
chr11	65479162	65479562	-0.658452348	0.048782777	decrease
chr22	39502121	39502521	-0.55217011	0.048796868	decrease
chr18	23586556	23586956	-0.638463959	0.048857415	decrease
chr17	3048827	3049227	-0.618989138	0.048892939	decrease

chr7	100612110	100612510	-0.521930105	0.048902838	decrease
chr7	44573950	44574350	-0.569313227	0.048929236	decrease
chr14	73712749	73713149	-0.637806242	0.048934589	decrease
chr7	64307225	64307625	-0.599162515	0.048934777	decrease
chr5	103369387	103369787	-0.675988827	0.04899525	decrease
chr2	64751007	64751407	-0.683158694	0.049032998	decrease
chr22	29306313	29306713	-0.551864268	0.049041416	decrease
chr16	2128411	2128811	-0.653098546	0.049049149	decrease
chr6	82101407	82101807	-0.676210279	0.04907779	decrease
chr4	113762016	113762416	-0.536754702	0.049080675	decrease
chr9	36995992	36996392	-0.685614128	0.049091401	decrease
chr1	233241263	233241663	-0.630419525	0.049097628	decrease
chr18	35993863	35994263	-0.670018408	0.049105931	decrease
chr3	111071309	111071709	-0.635722736	0.04912973	decrease
chr11	36289383	36289783	-0.646502492	0.049130982	decrease
chr6	99520940	99521340	-0.590697051	0.049165537	decrease
chr7	84150405	84150805	-0.644518453	0.049169818	decrease
chr3	169966263	169966663	-0.571115182	0.04917251	decrease
chr11	43358899	43359299	-0.61903387	0.049184859	decrease
chr1	190478797	190479197	-0.623817858	0.049192762	decrease
chr9	34523265	34523665	-0.660078493	0.049261145	decrease
chr16	90077233	90077633	-0.571792837	0.049267181	decrease
chr5	473828	474228	-0.675382216	0.049268349	decrease
chr7	143901880	143902280	-0.568473563	0.049272892	decrease
chr6	64538665	64539065	-0.660907442	0.04929527	decrease
chr6	154734096	154734496	-0.531631374	0.049305894	decrease
chr17	28426144	28426544	-0.618737261	0.049331689	decrease
chrX	40735665	40736065	-0.66976265	0.049345415	decrease
chr17	58692536	58692936	-0.613693875	0.04935298	decrease
chr22	32204824	32205224	-0.572262693	0.049366748	decrease
chr14	67695069	67695469	-0.614805851	0.049377843	decrease
chr9	130824995	130825395	-0.64093779	0.049396555	decrease
chr15	59372599	59372999	-0.63636962	0.049425585	decrease
chr8	22400824	22401224	-0.667627738	0.04951959	decrease
chr15	88246719	88247119	-0.635915715	0.049559626	decrease
chr9	122905169	122905569	-0.638248362	0.049561779	decrease
chr6	99588599	99588999	-0.637741286	0.049563409	decrease
chr13	77027363	77027763	-0.519306902	0.049578321	decrease
chr10	17201312	17201712	-0.544015261	0.049582971	decrease
chrX	123780148	123780548	-0.621620219	0.04959319	decrease
chr4	65998809	65999209	-0.609314133	0.049605811	decrease
chr9	133336237	133336637	-0.636974326	0.049648092	decrease
chr2	24049266	24049666	-0.670368254	0.049653059	decrease
chr4	102345189	102345589	-0.555056552	0.049658509	decrease
chr1	54272976	54273376	-0.54847233	0.049663985	decrease
chr15	74950479	74950879	-0.5884566	0.049669162	decrease
chr1	116855742	116856142	-0.662976166	0.049672502	decrease
chr2	46541682	46542082	-0.643133756	0.04969203	decrease
chr19	38361851	38362251	-0.580667936	0.049694572	decrease
chr1	23169147	23169547	-0.647193613	0.049724438	decrease
chr1	236406476	236406876	-0.63950735	0.049739476	decrease
chr12	20369476	20369876	-0.618345769	0.049741083	decrease
chr19	17865556	17865956	-0.518805181	0.049780979	decrease
chr6	33632625	33633025	-0.625106363	0.049790844	decrease
chr17	81794142	81794542	-0.650142078	0.049799804	decrease
chr11	101914888	101915288	-0.602647714	0.049817121	decrease
chr10	63465547	63465947	-0.642532372	0.0498366	decrease
chr16	660716	661116	-0.568081236	0.049863737	decrease
chr1	110209502	110209902	-0.640069526	0.049878021	decrease
chr1	190475003	190475403	-0.626422587	0.049928786	decrease
chr2	10122526	10122926	-0.608687903	0.049982053	decrease
chr12	110450053	110450453	-0.614418063	0.049982141	decrease
chr9	12776118	12776518	-0.658130368	0.050038688	same
chr19	12784970	12785370	-0.597150014	0.050064634	same
chr3	52287686	52288086	-0.572610937	0.050095504	same
chr11	68314208	68314608	-0.667082187	0.050110467	same
chr19	3035635	3036035	-0.566332613	0.050141007	same
chr8	17495983	17496383	-0.683248282	0.050194849	same
chr19	40598395	40598795	-0.647096851	0.050196282	same
chr4	125314804	125315204	-0.555744188	0.050263569	same
chr2	128233374	128233774	-0.655604923	0.050275124	same
chr21	46228228	46228628	-0.631521387	0.050287105	same
chr7	54774228	54774628	-0.656722308	0.050314307	same
chr6	161586882	161587282	-0.632653408	0.050314611	same
chr1	27725562	27725962	-0.558784017	0.050319986	same
chr22	19942461	19942861	-0.603542409	0.050383001	same

chr12	48903971	48904371	-0.674497831	0.050415035	same
chr22	41286219	41286619	-0.517916541	0.050417659	same
chr3	100992440	100993093	-0.665057079	0.050424745	same
chr20	18137675	18138075	-0.654648986	0.050433726	same
chr13	80339661	80340061	-0.512008199	0.050444933	same
chr13	19633395	19633795	-0.628787548	0.050472359	same
chr2	70141164	70141564	-0.548781434	0.050507276	same
chr1	162497914	162498314	-0.679157709	0.050554657	same
chr17	28711315	28711715	-0.570611809	0.050598441	same
chr7	87039901	87040301	-0.592173373	0.050602184	same
chr2	37376642	37377042	-0.642381087	0.050608699	same
chr11	33802507	33802907	-0.511190608	0.050614581	same
chr16	68023051	68023451	-0.608799226	0.050617967	same
chr8	80485472	80485872	-0.640776347	0.050625701	same
chr20	30579879	30580279	-0.684119673	0.050692612	same
chr13	52850535	52850935	-0.607404086	0.050783969	same
chr6	97282943	97283343	-0.595867825	0.050785398	same
chr15	65530433	65530833	-0.643818292	0.050822704	same
chr1	78884872	78885272	-0.615927002	0.050845048	same
chr9	127870068	127870468	-0.625139577	0.050854955	same
chr12	6753316	6753716	-0.553311561	0.05086603	same
chr8	41828228	41828628	-0.62783579	0.050881933	same
chr1	116418115	116418515	-0.626676109	0.05089114	same
chr3	142578534	142578934	-0.598640144	0.050976021	same
chr11	62855879	62856279	-0.485374972	0.050987497	same
chr15	95557148	95557548	-0.574552661	0.050996238	same
chr10	89251751	89252151	-0.594625724	0.051000448	same
chr3	94062821	94063221	-0.562937359	0.05100975	same
chr11	5684771	5685171	-0.654053622	0.051022149	same
chr1	147670091	147670491	-0.586412895	0.051022608	same
chr12	120087119	120087519	-0.53580962	0.051058033	same
chr14	74018706	74019106	-0.644432998	0.051060165	same
chr17	1455085	1455485	-0.627612265	0.051103892	same
chr1	156281748	156282148	-0.587690923	0.051291057	same
chr20	50509874	50510274	-0.635252756	0.051301734	same
chr19	40090630	40091030	-0.613494806	0.051346318	same
chrX	110317814	110318214	-0.656180043	0.051387959	same
chr17	74960500	74960900	-0.577131164	0.051394523	same
chr3	129249284	129249684	-0.512436762	0.051398919	same
chr19	49363401	49363801	-0.473341064	0.051408145	same
chr8	123416797	123417197	-0.632479806	0.051427754	same
chr4	174829185	174829585	-0.644818233	0.05156991	same
chr19	14571830	14572230	-0.5721036	0.051570881	same
chr14	47901275	47901675	-0.666674355	0.051614849	same
chr7	127585420	127585820	-0.601895414	0.051627688	same
chr4	75673249	75673649	-0.558845492	0.051667198	same
chr4	39698162	39698562	-0.665827815	0.05170274	same
chr14	36661759	36662159	-0.584125397	0.05171001	same
chr5	119452328	119452728	-0.624124254	0.051761695	same
chr4	83455638	83456038	-0.539580336	0.051764712	same
chr1	63173129	63173529	-0.65055775	0.051770117	same
chr6	27334611	27335011	-0.647414214	0.051787257	same
chr6	28137181	28137581	-0.6207613	0.051815521	same
chr16	22321318	22321718	-0.625366167	0.051827164	same
chr8	97775190	97775590	-0.637768752	0.05183451	same
chr9	35696358	35696758	-0.648496201	0.051838387	same
chr1	173714949	173715349	-0.563170265	0.051844306	same
chr18	28684425	28684825	-0.669535787	0.051856488	same
chr1	147225152	147225552	-0.649398513	0.051872294	same
chr12	53054168	53054568	-0.53395227	0.051886092	same
chr12	68746043	68746443	-0.58636312	0.051895625	same
chr6	111873371	111873771	-0.622762519	0.051920612	same
chr1	198930068	198930468	-0.675781014	0.051946455	same
chr19	49301295	49301695	-0.630621328	0.051980334	same
chr1	201170732	201171132	-0.511888008	0.051983447	same
chr9	130941334	130941734	-0.648781333	0.052019264	same
chr7	106285271	106285671	-0.589619153	0.052041004	same
chr15	41603904	41604304	-0.653742721	0.05211041	same
chr17	28318968	28319368	-0.536819443	0.052129743	same
chr7	26187556	26187956	-0.58943069	0.05213863	same
chr16	66603989	66604389	-0.620224262	0.052173382	same
chr2	3650852	3651252	-0.626683066	0.052204807	same
chr4	41935123	41935523	-0.579863013	0.052272304	same
chr21	33931346	33931746	-0.627130703	0.052296041	same
chr2	95121302	95121702	-0.66674602	0.052298502	same
chr19	45817548	45817948	-0.59260389	0.05238292	same

chr2	133293643	133294043	-0.614680205	0.052386366	same
chr5	77492001	77492401	-0.646364276	0.052391442	same
chr13	40777277	40777677	-0.661341376	0.052435688	same
chr16	89701719	89702119	-0.543501538	0.05243625	same
chr1	112674360	112674760	-0.596319999	0.052495302	same
chr10	11773942	11774342	-0.646497378	0.052569487	same
chr1	27234243	27234643	-0.619956085	0.052602816	same
chr6	138773469	138773869	-0.531353037	0.052611407	same
chr9	124617484	124617884	-0.65776909	0.052618542	same
chr12	55716111	55716511	-0.580196685	0.05264416	same
chr22	21641727	21642127	-0.569926948	0.052654718	same
chr15	77420264	77420664	-0.542820342	0.052709312	same
chr8	74984458	74984858	-0.642523958	0.052710327	same
chr5	115169619	115170019	-0.651303762	0.052714444	same
chr15	77907973	77908373	-0.632360389	0.052753222	same
chr1	155859294	155859694	-0.58690913	0.052776003	same
chr1	51517790	51518190	-0.631639129	0.052848803	same
chr3	49029156	49029556	-0.514225574	0.052849737	same
chr11	9364142	9364542	-0.652327592	0.052878437	same
chr19	55503843	55504243	-0.618638052	0.052989317	same
chr5	69166906	69167306	-0.591352986	0.052999542	same
chr19	45455533	45455933	0.631011925	0.053020566	same
chrX	103831597	103831997	-0.608678202	0.053025527	same
chr15	84747799	84748199	-0.612132392	0.05302657	same
chr5	34657488	34657888	-0.64418552	0.053030143	same
chr2	171433779	171434179	-0.577134859	0.053075343	same
chr1	53945945	53946345	-0.661188406	0.053078196	same
chr1	45686673	45687073	-0.531869909	0.053082817	same
chr12	123365430	123365830	-0.549758384	0.05309635	same
chr17	44014580	44014980	-0.632793445	0.053183816	same
chr2	73779930	73780330	-0.617388414	0.053204629	same
chr12	48001169	48001569	-0.617538531	0.053220755	same
chr3	107697700	107698100	-0.617915913	0.053264456	same
chr22	21624150	21624550	-0.579816337	0.053390918	same
chr2	157838841	157839241	-0.612786114	0.053394805	same
chr10	42782626	42783026	-0.549340527	0.053434813	same
chr2	231714350	231714750	-0.493567693	0.05346306	same
chr17	7220868	7221268	-0.503082356	0.053502971	same
chr11	115504594	115504994	-0.64068849	0.053580441	same
chr16	68328439	68328839	-0.573662072	0.053588515	same
chr14	49867865	49868265	-0.655560004	0.053634661	same
chr2	48315521	48315921	-0.593580142	0.053634955	same
chr2	60881482	60881882	-0.609511201	0.053705631	same
chr5	36241963	36242363	-0.606617011	0.0537335	same
chr17	16438629	16439029	-0.464075595	0.053752494	same
chr6	89281834	89282234	-0.656221348	0.053774948	same
chr1	151836542	151836942	-0.633213673	0.053779294	same
chr3	48439832	48440232	-0.541719675	0.053803639	same
chr13	66760676	66761076	-0.607164384	0.053863927	same
chr2	164910447	164910847	-0.661277129	0.053906546	same
chr15	90903365	90903765	-0.627025428	0.053908981	same
chr7	2631347	2631747	-0.610810489	0.053934254	same
chr19	4036343	4036743	-0.506157527	0.053934685	same
chr1	85259331	85259731	-0.566983917	0.053990969	same
chr16	69424315	69424715	-0.630120197	0.054001472	same
chr2	20411329	20411729	-0.6412799	0.05400906	same
chr16	2136098	2136498	-0.619331003	0.054011833	same
chr12	10212283	10212683	-0.555575801	0.054014468	same
chr1	230978869	230979269	-0.522530526	0.054028347	same
chr3	8589754	8590154	-0.619048155	0.0540613	same
chr12	49622956	49623356	-0.656823558	0.054100464	same
chr7	129948983	129949383	-0.619615031	0.05410487	same
chr9	128921893	128922293	-0.60667629	0.054172452	same
chr8	66774714	66775114	-0.609488859	0.054197394	same
chr2	176104507	176104907	-0.597295814	0.054199725	same
chr2	197706721	197707121	-0.627717245	0.054235669	same
chr8	67064191	67064591	-0.671005491	0.054254662	same
chr13	49247821	49248221	-0.574068803	0.05426673	same
chr4	48830848	48831248	-0.587119367	0.054322659	same
chr19	55647906	55648306	-0.637621168	0.054356546	same
chr19	38506954	38507354	-0.569895688	0.05436756	same
chr9	104763840	104764240	-0.66936162	0.054379696	same
chr1	107965601	107966001	-0.5249697	0.054398045	same
chr4	65670389	65670789	-0.55335903	0.054423688	same
chr8	58552891	58553291	-0.583725467	0.054436855	same
chr3	49412123	49412523	-0.504877647	0.054460595	same

chr5	149272206	149272606	-0.629519925	0.054468823	same
chr9	99821202	99821602	-0.580299215	0.054481979	same
chr20	34955720	34956120	-0.525212404	0.054555897	same
chr12	120709806	120710206	-0.617156441	0.054568691	same
chr11	6469050	6469450	-0.577491115	0.054583519	same
chr2	108533870	108534270	-0.558947171	0.05460152	same
chr6	37222421	37222821	-0.53473323	0.054608726	same
chr4	121711516	121711916	-0.65199653	0.054664748	same
chr3	112990797	112991197	-0.595206781	0.054709054	same
chr20	3470525	3470925	-0.642711112	0.054717213	same
chr2	85935788	85936188	-0.615800167	0.054756015	same
chr9	122828069	122828469	-0.593488786	0.054771416	same
chr7	138161643	138162043	-0.664253996	0.054810901	same
chr22	50675463	50675863	-0.584122734	0.054846946	same
chr17	781959	782359	-0.558252108	0.054874312	same
chr19	3285022	3285422	-0.560590097	0.054877276	same
chr7	139499989	139500389	-0.587441056	0.054897401	same
chr22	23218391	23218791	-0.630779337	0.054947366	same
chr22	37019829	37020229	-0.619179809	0.054949448	same
chr19	46990895	46991295	-0.640653906	0.055028949	same
chr15	101334136	101334536	-0.650392668	0.055033073	same
chr3	9249461	9249861	-0.56926328	0.055099723	same
chr2	204510989	204511389	-0.673010273	0.055110206	same
chr10	67849394	67849794	-0.616475052	0.05513619	same
chr19	36687212	36687612	-0.590081063	0.055191765	same
chr17	75154453	75154853	-0.519217742	0.055249664	same
chrX	120560886	120561286	-0.562890163	0.055258705	same
chr11	61792732	61793132	-0.53284063	0.055260432	same
chr7	79452128	79452528	-0.570910906	0.055344868	same
chr2	158969835	158970235	-0.634830175	0.055363484	same
chr21	43938947	43939347	-0.536500369	0.05537761	same
chr13	51844956	51845356	-0.616857484	0.055383944	same
chr2	178478676	178479076	-0.590057913	0.055416785	same
chr6	12008429	12008829	-0.645565995	0.055424122	same
chr9	33211845	33212245	-0.637213018	0.055430394	same
chr8	22588937	22589337	-0.654248786	0.055459002	same
chr7	30594192	30594592	-0.587875803	0.055470779	same
chr2	241702655	241703055	-0.650031079	0.055479982	same
chr16	84504454	84504854	-0.587581903	0.055499825	same
chr11	60841645	60842045	-0.596138729	0.055548309	same
chr16	75565833	75566233	-0.591791288	0.055570684	same
chr5	150700751	150701151	-0.605970776	0.055598374	same
chr1	29230648	29231048	-0.592886057	0.055598915	same
chr2	241665282	241665682	-0.595841931	0.05561872	same
chr6	75761835	75762235	-0.65091613	0.055651201	same
chr1	152013597	152013997	-0.630158448	0.055784587	same
chr12	130878569	130878969	-0.584055646	0.055807372	same
chr16	682853	683253	-0.484810468	0.055807962	same
chr2	38947197	38947597	0.669098165	0.055827797	same
chr15	40755164	40755564	-0.602581443	0.055901869	same
chr11	36377308	36377708	-0.627645097	0.055964755	same
chr19	37317677	37318077	-0.665491091	0.055966372	same
chr19	49451615	49452015	-0.54646573	0.055982133	same
chr4	78056998	78057398	-0.55707324	0.055999668	same
chr16	1708668	1709068	-0.568625666	0.05602354	same
chr9	98848402	98848802	-0.664314859	0.056041221	same
chr8	38231212	38231612	-0.629166998	0.056081473	same
chr9	127224316	127224716	-0.571167311	0.056081481	same
chr19	35628600	35629000	-0.531712119	0.056093101	same
chr4	120066826	120067226	-0.509798751	0.056096261	same
chr19	48103145	48103545	-0.659061323	0.056097212	same
chr10	132434784	132435184	-0.589501106	0.056099572	same
chr5	93607478	93607878	-0.649671798	0.056122491	same
chr9	124419409	124419809	-0.655125821	0.056129475	same
chrX	127873577	127873977	-0.66511747	0.0561681	same
chr1	74245351	74245751	-0.666590025	0.056181146	same
chrX	65667618	65668018	-0.638533079	0.05620522	same
chr1	113930389	113930789	-0.587059022	0.056206348	same
chrX	115259677	115260077	-0.60735019	0.05623355	same
chr8	141127883	141128283	-0.651486841	0.056246937	same
chr6	159692361	159692761	-0.603189765	0.056276701	same
chr15	78726846	78727246	-0.626641167	0.056277296	same
chr19	13833406	13834147	-0.589418638	0.056282126	same
chr4	94758658	94759058	-0.656578981	0.056284865	same
chr17	48816864	48817264	-0.652741243	0.056294557	same
chr15	51622180	51622580	-0.653311362	0.056331784	same

chr15	70763504	70763904	-0.559105879	0.05635027	same
chr7	99325543	99325943	-0.645208722	0.056356133	same
chr21	39349330	39349730	-0.60093539	0.056378588	same
chr17	44006988	44007388	-0.559754058	0.056382548	same
chr19	42396882	42397282	-0.503298536	0.05638592	same
chr16	29595430	29595830	-0.580222886	0.056397103	same
chr16	14285884	14286284	-0.620329647	0.056409671	same
chr19	6393226	6393626	-0.538668129	0.056453035	same
chr15	77910496	77910896	-0.523732779	0.05652784	same
chr6	125748407	125748807	-0.592227731	0.056554339	same
chr4	56544697	56545097	-0.632447397	0.056595674	same
chr2	176157635	176158035	-0.659034889	0.056645381	same
chr21	43659158	43659558	-0.640650218	0.056646444	same
chr17	75904911	75905311	-0.528234	0.056667684	same
chr1	23528309	23528709	-0.644902281	0.056728111	same
chr11	66466436	66466836	-0.644224026	0.056796619	same
chr19	15555973	15556373	-0.553793786	0.056828634	same
chr5	143087078	143087478	-0.583250304	0.056832816	same
chr19	44644416	44644816	-0.611468335	0.056842538	same
chr3	89114338	89114738	-0.599408523	0.056852548	same
chr1	162562101	162562501	-0.624306595	0.056949781	same
chr7	157397898	157398298	-0.539244837	0.056957501	same
chr20	34112320	34112720	-0.611306308	0.056973033	same
chr15	92882213	92882613	-0.583427079	0.057005412	same
chr2	740240	740640	-0.64916164	0.057069602	same
chr19	37594578	37594978	-0.552357342	0.057146019	same
chr20	50929413	50929813	-0.594634356	0.05716324	same
chr8	101623897	101624297	-0.628108082	0.057169063	same
chr16	1959041	1959441	-0.552762491	0.057169075	same
chr1	26233991	26234391	-0.567338971	0.057223725	same
chr15	55407911	55408311	-0.584038729	0.057228332	same
chr15	78075887	78076287	-0.462678753	0.057315768	same
chr10	99731895	99732295	-0.543790344	0.057330561	same
chr19	1479202	1479602	-0.614543335	0.057348818	same
chr1	61392641	61393041	-0.618485426	0.057499739	same
chr1	32413669	32414069	-0.637239897	0.057534127	same
chr15	91022157	91022557	-0.556220654	0.057558659	same
chr22	37849315	37849715	-0.529990516	0.057687018	same
chr14	74945977	74946377	-0.646207922	0.057692877	same
chr6	69796794	69797194	-0.585076736	0.057745866	same
chr3	127697488	127697888	-0.609348685	0.057780638	same
chr3	184156597	184156997	-0.631345264	0.057785088	same
chr10	80078707	80079107	-0.570760703	0.057785849	same
chr9	130700916	130701316	-0.656325904	0.05778611	same
chr13	30306767	30307167	-0.573813086	0.057824254	same
chr7	116723979	116724379	-0.623207075	0.057834167	same
chr15	41543887	41544287	-0.612687964	0.057837607	same
chrX	47144773	47145173	-0.525557062	0.057867094	same
chr8	140510683	140511083	-0.590156177	0.057870089	same
chr1	51519373	51519773	-0.660149824	0.057914907	same
chr2	127025109	127025509	-0.590699457	0.05795715	same
chr11	94767944	94768344	-0.628995891	0.057962007	same
chr6	34249184	34249584	-0.585440859	0.057968825	same
chr17	78186974	78187374	-0.502188386	0.05796969	same
chr4	173879178	173879578	-0.660008258	0.057979688	same
chr15	84654355	84654755	-0.596373002	0.057985354	same
chr1	234612230	234612630	-0.593579673	0.058003546	same
chr19	29606158	29606558	-0.596646905	0.058020647	same
chr14	96363006	96363406	-0.633943621	0.058056191	same
chr3	172710925	172711325	-0.587026467	0.058059561	same
chr19	5622711	5623111	-0.623477259	0.058101007	same
chr14	64503330	64503730	-0.598172689	0.058139572	same
chr10	95907844	95908244	-0.618847187	0.058186294	same
chr5	75717282	75717682	-0.535335536	0.058204323	same
chr19	36528510	36528910	-0.543654282	0.058220972	same
chr13	67416185	67416585	-0.56102994	0.058307338	same
chr15	88467324	88467724	-0.526565757	0.058349699	same
chr1	6475572	6475972	-0.644156634	0.058364178	same
chr14	36320657	36321057	-0.59674933	0.058382339	same
chr19	38336392	38336792	-0.583279317	0.058386844	same
chr2	131673203	131673603	-0.600993875	0.058405669	same
chr11	92225694	92226094	-0.62972618	0.058442699	same
chr22	43882931	43883331	-0.607925326	0.058529789	same
chr21	25734947	25735347	-0.451235733	0.058539701	same
chr6	35919733	35920133	-0.632433415	0.058549262	same
chr2	104853676	104854076	-0.626621903	0.058676132	same

chr16	3364900	3365300	-0.622571244	0.058730515	same
chr6	8435375	8435775	-0.632726422	0.058765524	same
chr15	66707213	66707613	-0.613713723	0.058830042	same
chr22	42070640	42071040	-0.479597109	0.058845885	same
chr1	237012854	237013254	-0.629500634	0.05894391	same
chr1	185733633	185734033	-0.602056439	0.058950982	same
chr15	78299130	78299530	-0.519288995	0.058961907	same
chr20	14146315	14146715	-0.628973602	0.059016176	same
chr1	232594334	232594734	-0.6370279	0.059019821	same
chr5	6378249	6378649	-0.638076071	0.059026014	same
chrX	72307014	72307414	-0.594398298	0.059039629	same
chr6	73520965	73521365	-0.571981599	0.059049418	same
chr6	38702984	38703384	-0.594133279	0.059050457	same
chr8	54022295	54022695	-0.635118803	0.059129997	same
chr9	137035172	137035572	-0.587281998	0.059133201	same
chr7	99499914	99500314	-0.613702727	0.059151463	same
chr4	185202275	185202675	-0.638241162	0.059159606	same
chr1	58699859	58700259	-0.531611024	0.059192446	same
chr2	65662523	65662923	-0.588319541	0.059193528	same
chr5	96808155	96808555	-0.573259011	0.059231791	same
chr20	347745	348145	-0.548708677	0.059293422	same
chr6	71420731	71421131	-0.620187061	0.059382062	same
chr19	38387822	38388222	-0.528099493	0.059409683	same
chr17	50178295	50178695	-0.558727176	0.059447674	same
chr11	67614405	67614805	-0.626756322	0.059527682	same
chr17	30731055	30731455	-0.647655219	0.059531171	same
chr5	120464937	120465337	-0.609448242	0.059536505	same
chr6	36977248	36977648	-0.647842127	0.059564375	same
chr19	35154857	35155257	-0.627959306	0.059577634	same
chr5	140174842	140175242	-0.589412854	0.059598645	same
chr17	81710587	81710987	-0.487607601	0.059609814	same
chr2	39790306	39790706	-0.600185652	0.059643846	same
chr16	28880111	28880511	-0.601539728	0.059657359	same
chr12	8081948	8082348	-0.586326531	0.059672131	same
chr4	176001297	176001697	-0.631161729	0.059690296	same
chr19	48501615	48502015	-0.544609773	0.059694244	same
chr13	78593963	78594363	-0.619905886	0.059721952	same
chr12	74539156	74539556	-0.652503066	0.059723513	same
chr9	4792408	4792808	-0.546754787	0.059752541	same
chr1	114581443	114581843	-0.586108941	0.059759224	same
chr12	1593292	1593692	-0.598744182	0.059780786	same
chr17	82592653	82593053	-0.575376823	0.059808643	same
chr9	128684583	128684983	-0.544213398	0.059815664	same
chr16	79600521	79601227	-0.624696954	0.059904893	same
chr10	71377245	71377645	-0.612914531	0.059924885	same
chr3	50246316	50246716	-0.556971548	0.059935394	same
chr12	113162214	113162614	-0.59998509	0.059949002	same
chr12	131104554	131104954	-0.638684834	0.059982546	same
chr1	210329519	210329919	-0.602616453	0.059982575	same
chr11	7512955	7513355	-0.639902125	0.059992838	same
chr4	162164074	162164474	-0.632859743	0.060019163	same
chr3	70290559	70290959	-0.651521806	0.060026581	same
chr1	204493804	204494204	-0.614229825	0.060053758	same
chr14	76904696	76905096	-0.631442406	0.060087552	same
chr9	129496094	129496494	-0.613587101	0.060088127	same
chr9	127567538	127567938	-0.601428529	0.060090795	same
chr1	235083800	235084200	-0.565969331	0.060108352	same
chr5	171342414	171342814	-0.594447619	0.060194511	same
chr5	171340401	171340801	-0.616805432	0.060212995	same
chr10	8044977	8045377	-0.595802276	0.060230904	same
chr6	19247010	19247410	-0.640791095	0.060234897	same
chr1	212414765	212415165	-0.549208791	0.060409005	same
chr12	48157495	48157895	-0.531886729	0.060431616	same
chrX	48834441	48834841	-0.626464217	0.060480205	same
chr16	22436650	22437050	-0.651727757	0.060505478	same
chr19	45326114	45326514	-0.592854294	0.06054371	same
chr7	99616619	99617019	-0.508417804	0.060559154	same
chr11	65535480	65535880	-0.586160956	0.060583776	same
chr12	122965915	122966315	-0.570318709	0.060603601	same
chr22	23145142	23145542	-0.583950134	0.060640635	same
chr13	45341312	45341712	-0.51744154	0.06069922	same
chr13	66843084	66843484	-0.620172457	0.060784839	same
chr1	169794660	169795060	-0.5231007	0.060791483	same
chr1	111346299	111346699	-0.577119341	0.060896346	same
chr8	144463835	144464235	-0.493856529	0.060896721	same
chr1	89719652	89720052	-0.636831125	0.060929414	same

chr17	4143325	4143725	-0.528462287	0.060960519	same
chrX	17737538	17737938	-0.631854761	0.060972085	same
chr10	73096748	73097148	-0.516672461	0.061046773	same
chr7	39910693	39911093	-0.639674295	0.061050858	same
chr8	144413273	144413673	-0.542698595	0.06106327	same
chr9	114487652	114488052	-0.638049846	0.061121269	same
chr7	66099938	66100338	-0.617438309	0.061124892	same
chr9	19049019	19049419	-0.64075112	0.061126459	same
chr13	52448111	52448511	-0.606367374	0.061147659	same
chr14	51651372	51651772	-0.598107382	0.061148353	same
chr16	8621628	8622028	-0.640832813	0.061186945	same
chr1	42456388	42456788	-0.623560568	0.061209917	same
chrX	71146268	71146668	-0.592766234	0.061226547	same
chr19	19138226	19138626	-0.54208467	0.061259901	same
chr17	64938808	64939208	-0.597094388	0.061290073	same
chr2	158456509	158456909	-0.613408634	0.061296709	same
chr21	45541039	45541439	-0.573801921	0.061302317	same
chr14	60980919	60981319	-0.556395743	0.061304184	same
chr2	74421695	74422095	-0.600290031	0.061315421	same
chr15	66886059	66886459	-0.579619469	0.06134639	same
chr6	43059593	43059993	-0.605037456	0.06135664	same
chr16	28553902	28554302	-0.617381676	0.061370668	same
chr10	73413742	73414142	-0.551060269	0.061411791	same
chr22	30423676	30424076	-0.463365281	0.061412792	same
chr2	121649392	121649792	-0.498211033	0.061445399	same
chr6	15245497	15245897	-0.53009988	0.061468594	same
chr10	124419006	124419406	-0.572655803	0.061473374	same
chr14	23006818	23007218	-0.511787319	0.061531227	same
chr1	46603981	46604381	-0.506907491	0.061572365	same
chr16	3235102	3235502	-0.599872012	0.061573985	same
chr1	156162664	156163064	-0.61140544	0.061574372	same
chr17	59220493	59220893	-0.612395624	0.061613635	same
chr1	35641468	35641868	-0.535805091	0.061647931	same
chr4	90958368	90958768	-0.63353775	0.061688829	same
chr1	220272242	220272642	-0.562360869	0.061761021	same
chr12	124518522	124518922	-0.522456845	0.061773429	same
chr11	125625983	125626383	-0.469806975	0.061773854	same
chr12	48021742	48022142	-0.622129162	0.0617771	same
chr6	146028309	146028709	-0.638352249	0.061817639	same
chrX	102516458	102516858	-0.642735282	0.061833763	same
chr11	63671544	63671944	-0.588242257	0.061854021	same
chr7	27740052	27740452	-0.567843596	0.061890491	same
chr5	112420146	112420546	-0.649293391	0.061900116	same
chr1	184930402	184930802	-0.621757885	0.061917245	same
chr9	6758650	6759050	-0.574814491	0.061975412	same
chr9	137086608	137087008	-0.518019841	0.062076265	same
chr19	49496030	49496430	-0.419553229	0.062135173	same
chr19	38544020	38544420	-0.567747902	0.062144386	same
chr18	57621781	57622181	-0.615192392	0.062181731	same
chr3	120349339	120349739	-0.57980862	0.062199368	same
chr1	16352383	16352783	-0.62473393	0.06227252	same
chr11	118790426	118790826	-0.603708203	0.062275681	same
chr4	110963771	110964171	-0.647862226	0.062294727	same
chr1	157992013	157992413	-0.618170411	0.062375587	same
chr7	26858290	26858690	-0.619492608	0.062385687	same
chr22	44685154	44685554	-0.536888746	0.062389537	same
chr4	6222747	6223147	-0.579385696	0.062390439	same
chr2	68252651	68253051	-0.645650481	0.062418458	same
chrX	47658865	47659265	-0.512673682	0.062530144	same
chr1	153658569	153658969	-0.520137936	0.062554696	same
chrX	107450892	107451292	-0.617095982	0.062618686	same
chr4	56465692	56466092	-0.590166128	0.062621135	same
chr5	163461349	163461749	-0.556916162	0.06263099	same
chr10	52314166	52314566	-0.53779482	0.0626651	same
chrX	130339752	130340152	-0.621416234	0.062714954	same
chrX	73892505	73892905	-0.641435613	0.062777549	same
chr7	151969452	151969852	-0.518870083	0.062969749	same
chr1	161045672	161046072	-0.52199185	0.063003494	same
chr5	144170431	144170831	-0.598282616	0.063065907	same
chr4	7761699	7762099	-0.632399725	0.063077111	same
chr10	80205566	80205966	-0.586159433	0.063105117	same
chr4	146521799	146522199	-0.514898194	0.063157079	same
chr6	4647575	4647975	-0.59622108	0.063199303	same
chr3	113515006	113515406	-0.548056908	0.063219506	same
chr22	43089431	43089831	-0.634099176	0.063232719	same
chrX	154096314	154096991	-0.571740043	0.063257715	same

chr3	158106618	158107018	-0.590943247	0.063272455	same
chr7	157228676	157229076	-0.579972639	0.063305248	same
chr4	48269833	48270233	-0.60049915	0.063317076	same
chr13	66944585	66944985	-0.579941475	0.063395783	same
chr5	116572901	116573301	-0.506518534	0.063396399	same
chr7	136115309	136115709	-0.595534346	0.063422092	same
chr14	61332018	61332418	-0.612152826	0.063501027	same
chr2	219521488	219521888	-0.613375665	0.06353434	same
chr20	54207776	54208176	-0.641746853	0.063561707	same
chr15	73376095	73376495	-0.620114921	0.063589554	same
chr10	17617496	17617896	-0.641235976	0.063591535	same
chr7	146639585	146639985	-0.641869585	0.0636155	same
chr19	40798639	40799039	-0.540538399	0.063628937	same
chr19	17762607	17763007	-0.62926052	0.063665739	same
chr15	66619240	66619640	-0.609802013	0.063706005	same
chr16	2912630	2913030	-0.617436688	0.06371404	same
chr1	206612200	206612600	-0.553181419	0.063717679	same
chr17	17835952	17836352	-0.591686283	0.063725078	same
chr9	132669797	132670197	-0.545155259	0.063768439	same
chr5	88268601	88269001	-0.573055283	0.063800717	same
chr6	24359812	24360212	-0.618302419	0.063843811	same
chr16	28210191	28210591	-0.607234954	0.063853117	same
chr15	40160797	40161197	-0.629662625	0.063861605	same
chr9	120868964	120869364	-0.598218514	0.063886973	same
chr3	120567509	120567909	-0.631497109	0.063912063	same
chr11	108122310	108122710	-0.606631334	0.063914254	same
chr17	28725187	28725587	-0.464987854	0.063927415	same
chr1	161192730	161193130	-0.608910597	0.063950484	same
chr9	132944421	132944821	-0.577079044	0.063976066	same
chr1	61318382	61318782	-0.625302224	0.063983221	same
chr19	24033340	24033740	-0.572050714	0.064009004	same
chr22	29603190	29603590	-0.54356779	0.064023198	same
chr12	116506374	116506774	-0.644232957	0.064024142	same
chr19	36378835	36379235	-0.633521451	0.064060238	same
chr22	39490224	39490624	-0.57403903	0.064160687	same
chr15	65862630	65863030	-0.551719636	0.064183246	same
chr12	50763703	50764103	-0.592696617	0.064232073	same
chr13	79528493	79528893	-0.463662019	0.064256808	same
chr19	45398254	45398654	-0.599388859	0.064279481	same
chr7	83648715	83649115	-0.62623204	0.064286293	same
chrX	120603837	120604237	-0.545707492	0.064296513	same
chr11	64566645	64567045	-0.618727894	0.064301052	same
chr3	10026021	10026421	-0.575917787	0.064316025	same
chr5	177309555	177309955	-0.492771534	0.064316605	same
chr2	38666014	38666414	-0.555461897	0.064366923	same
chr6	169722839	169723239	-0.623149404	0.064401122	same
chr2	172465499	172465899	-0.626695921	0.064427657	same
chr1	154326211	154326611	-0.54293621	0.064476663	same
chr17	62416804	62417204	-0.639944917	0.064488397	same
chr15	78308249	78308649	-0.599775802	0.064525346	same
chr4	88277456	88277856	-0.626764811	0.06455777	same
chr19	18784956	18785527	-0.566054546	0.064634621	same
chr10	43203852	43204252	-0.596363162	0.064757214	same
chrX	129905780	129906180	-0.575368935	0.064762568	same
chr16	2682175	2682575	-0.607566594	0.06481204	same
chr11	76445256	76445656	-0.562138449	0.064835753	same
chr12	27710370	27710770	-0.557008651	0.06486284	same
chr9	37422357	37422757	-0.522579726	0.06489236	same
chr12	3300408	3300808	-0.602091664	0.064905614	same
chr17	29369924	29370324	-0.63291806	0.064907666	same
chr19	35900299	35900699	-0.60018231	0.064914755	same
chr6	98824026	98824426	-0.627338801	0.064917297	same
chr14	20625214	20625614	-0.560400547	0.064929001	same
chr11	12110101	12110501	-0.627542307	0.065082535	same
chr11	77819733	77820133	-0.563504002	0.065172456	same
chr2	185864642	185865042	-0.628104226	0.065179085	same
chr16	46831412	46831812	-0.587364976	0.065194782	same
chr13	42041406	42041806	-0.619985208	0.065198547	same
chr9	105558053	105558453	-0.573202412	0.065210093	same
chr7	157839713	157840113	-0.62519067	0.065257852	same
chr4	74157767	74158167	-0.602370028	0.065315408	same
chr3	113745275	113745675	-0.518058618	0.065360965	same
chr17	69394635	69395035	-0.624051357	0.065375756	same
chr22	45317697	45318097	-0.610973219	0.06540515	same
chr15	40594164	40594564	-0.594807426	0.065414588	same
chr16	3175371	3175771	-0.591855329	0.065415913	same

chr3	185586047	185586447	-0.563091977	0.065430818	same
chr11	18322207	18322607	-0.494465761	0.065435007	same
chr20	45935215	45935615	-0.584776729	0.065449375	same
chr1	108200311	108200711	-0.614525147	0.065454523	same
chr1	151198354	151198754	-0.533248119	0.065473316	same
chr13	41919377	41919777	-0.636285323	0.065520791	same
chr3	16482892	16483292	-0.607057426	0.065653801	same
chr12	70243209	70243609	-0.642083177	0.065687591	same
chrX	108438141	108438541	-0.553226255	0.065688083	same
chr16	53703697	53704097	-0.545951864	0.065695594	same
chr2	96858377	96858777	-0.572342038	0.065775966	same
chr15	89554133	89554533	-0.611565311	0.065779197	same
chr6	57800054	57800454	-0.566551662	0.065824924	same
chr1	17054001	17054401	-0.55888813	0.065839564	same
chr22	39319709	39320109	-0.487911419	0.065840692	same
chr17	76029392	76029792	-0.487742057	0.065912897	same
chrX	107628251	107628651	-0.48176691	0.065916528	same
chr18	32922907	32923307	-0.622249792	0.066057221	same
chr20	34876025	34876425	-0.521186897	0.066094551	same
chr6	133569324	133569724	-0.630838663	0.066117388	same
chr2	150485864	150486264	-0.60754781	0.06616369	same
chr14	22929515	22929915	-0.519831979	0.066166114	same
chr6	42746830	42747230	-0.550997445	0.066168097	same
chr9	128438649	128439049	-0.573955383	0.066187467	same
chr3	69086083	69086483	-0.59555877	0.066215066	same
chr3	57557059	57557459	-0.581456079	0.066240621	same
chr11	28107967	28108367	-0.555453216	0.066290536	same
chr7	112452348	112452748	-0.597640499	0.06633103	same
chrX	63754950	63755350	-0.598733484	0.066334572	same
chr4	74445209	74445609	-0.636397222	0.066366066	same
chr8	99893428	99893828	-0.601912985	0.066422602	same
chr6	138988324	138988724	-0.59030476	0.066424989	same
chr14	92039847	92040247	-0.547669965	0.066438485	same
chr8	11736392	11736792	-0.598595961	0.066470546	same
chr5	173055902	173056302	-0.548886461	0.066545151	same
chr1	109984327	109984727	-0.553465761	0.066655101	same
chr19	10380595	10380995	-0.544766845	0.066701401	same
chr11	27506580	27506980	-0.533608956	0.06670433	same
chr17	28728689	28729089	-0.518680529	0.066708022	same
chr13	31209345	31209745	-0.611125827	0.066720099	same
chr14	96979494	96979894	-0.602514838	0.0667403	same
chr9	92293617	92294017	-0.548405203	0.066823088	same
chr11	59556928	59557328	-0.487469518	0.066847254	same
chr2	40451028	40451428	-0.595931447	0.066862259	same
chrX	73214423	73214823	-0.620942641	0.066893621	same
chr4	66565146	66565546	-0.64444125	0.066917406	same
chr2	127415775	127416363	-0.568342206	0.06693083	same
chr5	151081081	151081481	-0.563918692	0.067059824	same
chr14	92105857	92106257	-0.588883289	0.067112123	same
chr4	151408644	151409044	-0.608030996	0.067154006	same
chr19	49476205	49476605	-0.534500397	0.067202542	same
chr6	27237303	27237703	-0.587342938	0.067217491	same
chr9	83707909	83708309	-0.60569179	0.067277835	same
chr8	140098866	140099266	-0.59330109	0.067288425	same
chr2	54558482	54558882	-0.603637882	0.067293795	same
chr21	34615302	34615702	-0.631793261	0.067306916	same
chr9	128771951	128772351	-0.600268662	0.067526086	same
chr3	131331517	131331917	-0.601076926	0.067544173	same
chr11	78139517	78139917	-0.557453319	0.067546793	same
chr5	85663288	85663688	-0.6013446	0.067597515	same
chr22	19730461	19730861	-0.544749623	0.067601934	same
chr15	66294197	66294597	-0.632367714	0.067660183	same
chr1	99765701	99766101	-0.588883099	0.067665108	same
chr19	55156463	55156863	-0.569029208	0.067699125	same
chr12	50924620	50925020	-0.599273811	0.067706155	same
chr1	225950951	225951351	-0.547353868	0.067719752	same
chr7	2808007	2808407	-0.570109853	0.06773255	same
chr22	41688684	41689084	-0.58566841	0.067737896	same
chr9	92808918	92809318	-0.604301472	0.067800816	same
chr16	73055346	73055746	-0.593151474	0.067820144	same
chr5	217920	218320	-0.595895297	0.067826851	same
chr17	42833140	42833540	-0.550097434	0.067869402	same
chr16	89816644	89817044	-0.60129659	0.067962174	same
chr19	32405342	32405742	-0.615973122	0.068087413	same
chr1	167935096	167935496	-0.546367177	0.068109459	same
chr11	58524305	58524705	-0.635227593	0.068188097	same

chr15	66592972	66593372	-0.613041268	0.068196455	same
chr20	33489668	33490068	-0.617654471	0.068243172	same
chr19	17209786	17210186	-0.589402224	0.068259649	same
chr19	40348375	40348775	-0.595101717	0.068292524	same
chr16	84145220	84145620	-0.605238093	0.068335145	same
chr6	80004242	80004642	-0.528787864	0.068450561	same
chr16	3690506	3690906	-0.566134034	0.068593687	same
chr17	18039040	18039440	-0.531629653	0.068609098	same
chr5	134466337	134466737	-0.611986377	0.068652065	same
chr15	81324169	81324569	-0.518627887	0.068662452	same
chr7	129829950	129830350	-0.607970877	0.068663116	same
chr7	42888337	42888737	-0.542528045	0.068719823	same
chr12	120711440	120711840	-0.550903089	0.068728491	same
chr19	19663698	19664098	-0.560646035	0.06875149	same
chr1	27772993	27773393	-0.514337055	0.068759734	same
chr4	174255910	174256310	-0.573968423	0.068761021	same
chr9	74952480	74952880	-0.625008161	0.068783367	same
chr12	55996828	55997228	-0.590539494	0.068822377	same
chr8	134832378	134832778	-0.541463347	0.06885537	same
chr11	130103261	130103661	-0.605489239	0.068907704	same
chr6	125986405	125986805	-0.466011713	0.068912068	same
chr2	96335954	96336354	-0.546720018	0.068935776	same
chr2	199911087	199911487	-0.604110764	0.068961267	same
chr1	23868358	23868758	-0.629159659	0.069004418	same
chr5	149618723	149619123	-0.622529894	0.069006069	same
chr4	159175651	159176051	-0.615062687	0.069027598	same
chr1	43358727	43359127	-0.534467724	0.069043862	same
chr17	80101999	80102399	-0.542616072	0.069075992	same
chr19	58380385	58380785	-0.575239943	0.069109595	same
chr9	127943576	127943976	-0.556374342	0.069188343	same
chr12	54038803	54039203	-0.576568176	0.069247123	same
chr1	156140139	156140539	-0.589205016	0.069250358	same
chr2	72115994	72116394	0.634177745	0.069251503	same
chr19	18007218	18007618	-0.603212944	0.069254525	same
chr2	112275291	112275691	-0.611470079	0.069283224	same
chr19	47348999	47349399	-0.523638827	0.069293074	same
chr8	67061983	67062383	-0.537513745	0.069315842	same
chr3	53891846	53892246	-0.519110619	0.069404427	same
chr7	149146959	149147359	-0.583301351	0.069510451	same
chr8	54101951	54102351	-0.62885826	0.06951199	same
chr4	24584432	24584832	-0.590236939	0.0696287	same
chr8	76672692	76673092	-0.602616623	0.069634728	same
chr13	50119104	50119504	-0.620451114	0.06964331	same
chr11	8219053	8219453	-0.605984515	0.069652795	same
chr11	86940446	86940846	-0.620634428	0.069657487	same
chr14	30621924	30622324	-0.493353947	0.069789246	same
chr9	87140789	87141189	-0.621696324	0.069811415	same
chr2	99488578	99488978	-0.609045306	0.069864568	same
chr20	17568830	17569230	-0.613888858	0.069869584	same
chr6	139250472	139250872	-0.614816193	0.069902866	same
chr1	222711469	222711869	-0.54759932	0.069933343	same
chr7	6590554	6590954	-0.59597642	0.069968234	same
chr3	52770912	52771312	-0.570702255	0.069968379	same
chr6	139152405	139152805	-0.585756411	0.069974587	same
chr17	50270190	50270590	-0.591850616	0.070022951	same
chr1	19210350	19210750	-0.575747904	0.070102	same
chr11	65888308	65888708	-0.487149143	0.070245148	same
chr1	40531590	40531990	-0.569551207	0.070259891	same
chr4	987174	987574	-0.635518156	0.070306877	same
chr19	42217283	42217683	-0.55135597	0.070335956	same
chr4	148442423	148442823	-0.563103563	0.070361657	same
chr11	107017380	107017780	-0.613257948	0.070411844	same
chr14	69191450	69191850	-0.544229174	0.070429459	same
chr10	68231146	68231546	-0.563953208	0.070453647	same
chr17	57849937	57850337	-0.565209987	0.070540386	same
chr3	137035634	137036034	-0.615875977	0.070592587	same
chr22	31212139	31212539	-0.603508513	0.070661028	same
chr20	43514126	43514526	-0.42839809	0.070677564	same
chr16	87493080	87493480	-0.601580755	0.070712094	same
chr19	17282678	17283078	-0.570984851	0.070783255	same
chr12	121894413	121894813	-0.613911758	0.07078459	same
chr5	178859606	178860006	-0.600058587	0.070792276	same
chr11	18410465	18410865	-0.608447287	0.070864249	same
chr12	48995471	48995871	-0.546063281	0.07095337	same
chr14	70988389	70988789	-0.614734151	0.070955619	same
chr22	25110836	25111236	-0.528897629	0.070973201	same

chr5	95731864	95732264	-0.628779041	0.070973631	same
chr18	35983608	35984008	0.616404177	0.070983905	same
chr4	173168285	173168685	-0.612165258	0.070998744	same
chr17	82077007	82077407	-0.480471824	0.071012342	same
chr6	16277522	16277922	-0.597778434	0.071022573	same
chr17	16386564	16386964	-0.627940111	0.071056277	same
chr15	50424034	50424434	-0.608394321	0.0710856	same
chr3	169661694	169662094	-0.570618526	0.07120229	same
chr5	177444452	177444852	-0.475209069	0.071215026	same
chr7	36152762	36153162	-0.492882367	0.071260196	same
chr17	57988481	57988881	-0.591814676	0.071274	same
chrX	51893264	51893664	-0.562383677	0.071277846	same
chr4	127632891	127633291	-0.58682275	0.071341654	same
chr3	51389254	51389654	-0.534730397	0.071365221	same
chr1	168354372	168354772	-0.605477766	0.071409262	same
chr19	37217830	37218230	-0.459337937	0.071443534	same
chr5	74354943	74355343	-0.622272373	0.071473703	same
chr22	38317918	38318318	-0.471159741	0.071519993	same
chr4	95044324	95044724	-0.624585159	0.071527645	same
chr4	119627666	119628066	-0.60213043	0.071540876	same
chr20	51639328	51639728	-0.619732647	0.071604388	same
chr6	27602299	27602699	-0.54242813	0.071613028	same
chr16	57757052	57757452	-0.539860245	0.071620538	same
chr4	104490751	104491151	-0.598453228	0.071620567	same
chrX	133415542	133415942	-0.58071452	0.07162354	same
chr19	55400788	55401188	-0.558535611	0.071654496	same
chr3	187738601	187739001	-0.598739774	0.071709848	same
chr13	21298138	21298538	-0.606304631	0.071754079	same
chrX	107206192	107206592	-0.516642729	0.071798307	same
chr19	44914949	44915349	-0.54454232	0.071833493	same
chr3	171809714	171810114	-0.550875442	0.071834366	same
chr14	88551731	88552131	-0.5986632	0.071836585	same
chr5	142088923	142089323	-0.595114525	0.071840755	same
chr17	30906272	30906672	-0.528937669	0.07188663	same
chr19	17841937	17842337	-0.55901851	0.071910505	same
chr17	30116538	30116938	-0.619846647	0.071926332	same
chr15	82108527	82108927	-0.596909237	0.071932255	same
chr1	145859932	145860332	-0.57460439	0.071999016	same
chr14	69727110	69727510	-0.613569933	0.072021402	same
chr15	98779451	98779851	-0.5770564	0.072038328	same
chr20	3173871	3174271	-0.532966068	0.072066452	same
chr17	78736609	78737009	-0.523820454	0.072183616	same
chr6	149922801	149923201	-0.565452075	0.07219122	same
chr1	50873568	50873968	-0.623162959	0.072198172	same
chr6	168035579	168035979	-0.588683128	0.07220953	same
chr3	138174586	138174986	-0.573357562	0.072254546	same
chr14	64764366	64764766	-0.578882219	0.07225654	same
chr5	137889313	137889713	-0.594508061	0.072302448	same
chr16	57244696	57245096	-0.586082939	0.072302732	same
chr7	39754188	39754588	-0.620756874	0.072303084	same
chr1	237806820	237807220	-0.611320343	0.072307513	same
chr6	57046511	57046911	-0.603386682	0.072320001	same
chr11	8964509	8964909	-0.47702858	0.07232223	same
chr4	155675983	155676383	-0.588277944	0.072434137	same
chr1	230268719	230269119	-0.48311211	0.072438686	same
chr17	3794911	3795311	-0.609037268	0.072449635	same
chr1	1399808	1400208	-0.547926539	0.072475672	same
chr1	68232568	68232968	-0.594912163	0.072477553	same
chr15	75336213	75336613	-0.52812453	0.072482468	same
chr17	28571320	28571720	-0.519311553	0.072492811	same
chr13	26971977	26972377	-0.617558937	0.072640168	same
chr2	134450725	134451125	-0.600526817	0.072728823	same
chr8	22010282	22010682	-0.616792029	0.07274243	same
chr19	34677318	34677718	-0.548730723	0.072774012	same
chr10	67764119	67764519	-0.497457265	0.072793051	same
chr6	43053513	43053913	-0.531134511	0.072831987	same
chr12	43758679	43759079	-0.550345437	0.072841646	same
chr10	23440460	23440860	-0.618052878	0.072847117	same
chr7	72879811	72880211	-0.614739722	0.072869559	same
chr9	133459911	133460311	-0.553279406	0.072876239	same
chr14	68979414	68979814	-0.614059235	0.072914755	same
chr1	6580647	6581047	-0.612805745	0.072916053	same
chr21	21048273	21048673	-0.612051873	0.072945337	same
chr19	1665658	1666058	-0.62307828	0.072973561	same
chr1	108560915	108561315	-0.594227605	0.072997447	same
chr9	127715675	127716075	-0.487295236	0.073002978	same

chr17	409964	410364	-0.54561567	0.073028448	same
chr1	26275628	26276028	-0.608979948	0.073030537	same
chr11	34357597	34357997	-0.622040002	0.073067702	same
chr14	99253639	99254039	-0.571039281	0.073071785	same
chr5	173615760	173616160	-0.609669543	0.073091308	same
chr2	241009907	241010307	-0.613894804	0.073147756	same
chr7	4683626	4684026	-0.605195414	0.073180792	same
chr17	48546781	48547181	-0.592525349	0.073195741	same
chr7	73556841	73557241	-0.584547387	0.073201394	same
chr10	11758006	11758406	-0.590021007	0.073222943	same
chr4	133148901	133149301	-0.583703613	0.073267667	same
chr16	589316	589716	-0.448458593	0.07327634	same
chr12	120356624	120357024	-0.568760052	0.073295401	same
chr15	34969542	34969942	-0.568901726	0.073390528	same
chr3	186109130	186109530	-0.603930664	0.073425941	same
chr7	130205193	130205593	-0.513607993	0.073449282	same
chr15	69394487	69394887	-0.580279611	0.073486166	same
chr19	5790645	5791045	-0.43164207	0.073491954	same
chr16	25257218	25257618	-0.618329419	0.073504642	same
chr6	109441183	109441583	-0.594618151	0.073516521	same
chr20	63658365	63658765	-0.489470267	0.073517975	same
chr1	156601270	156601670	-0.497368548	0.073545919	same
chr5	88667338	88667738	-0.621419229	0.073593824	same
chrX	115958007	115958407	-0.525505133	0.073609081	same
chr7	151113606	151114006	-0.510959674	0.073612915	same
chr3	158801801	158802201	-0.566703502	0.073615215	same
chr22	29245393	29245793	-0.600590392	0.073622998	same
chr7	39623291	39623691	-0.588625648	0.073625716	same
chr17	38867860	38868260	-0.570461583	0.07366658	same
chr19	10461273	10461673	-0.574299127	0.073696231	same
chrX	71254410	71254810	-0.521915573	0.073697644	same
chr8	43055987	43056387	-0.584778417	0.073766749	same
chr10	109884404	109884804	-0.606921943	0.073791589	same
chr15	68054738	68055138	-0.588895167	0.073836229	same
chr2	42906767	42907167	-0.597905229	0.073862483	same
chr1	1537884	1538284	-0.568827463	0.073979324	same
chr10	110871500	110871900	-0.570023492	0.07413828	same
chr1	153945210	153945610	-0.54789395	0.074139601	same
chr19	12676228	12676628	-0.509838427	0.074199084	same
chr12	56224175	56224575	-0.545945917	0.074296881	same
chr8	120124075	120124475	-0.55740027	0.074305163	same
chr4	139280044	139280444	-0.565763087	0.074373935	same
chr10	113854320	113854720	-0.586086652	0.074389912	same
chr2	39124001	39124401	-0.51595815	0.07440556	same
chr2	169824246	169824646	-0.58590431	0.074437486	same
chr16	30335168	30335568	-0.53088175	0.074450146	same
chr10	103396474	103396874	-0.522737344	0.07446177	same
chr14	24391990	24392390	-0.606139355	0.074478895	same
chr3	138115096	138115496	-0.558532799	0.074545793	same
chr3	58585021	58585421	-0.617848789	0.074615457	same
chr6	37499899	37500299	-0.528458648	0.074656875	same
chr1	219173938	219174338	-0.608769128	0.074720111	same
chr6	1597174	1597574	-0.58479111	0.074805457	same
chr9	128159803	128160203	-0.504612563	0.074824734	same
chr7	66930319	66930719	-0.592269943	0.074857908	same
chr1	206856658	206857058	-0.568073969	0.074908284	same
chr14	69610652	69611052	-0.568043583	0.074962268	same
chr19	55491943	55492343	-0.571854622	0.074978764	same
chr10	44978514	44978914	-0.584039123	0.074982402	same
chr14	89955113	89955513	-0.518138369	0.075053219	same
chr3	49013990	49014390	-0.48457436	0.075138496	same
chr6	46652678	46653078	-0.588533286	0.075161971	same
chr16	72008486	72008886	-0.502342357	0.075192237	same
chr5	44808601	44809001	-0.612231447	0.075374759	same
chr15	85380108	85380508	-0.561330202	0.075447775	same
chr13	20773627	20774027	-0.584750978	0.07551835	same
chr19	48746978	48747378	-0.55215923	0.075728231	same
chr10	73772056	73772456	-0.599388418	0.075744083	same
chr8	87945610	87946010	-0.610154481	0.075779129	same
chr8	90984701	90985101	-0.594938967	0.07578088	same
chr21	39314412	39314812	-0.553707539	0.075799976	same
chr19	57411050	57411450	-0.5343391	0.075809913	same
chr12	114914294	114914694	-0.555150654	0.075811249	same
chr20	33810959	33811359	-0.594804017	0.075863395	same
chr2	96801054	96801454	-0.558430258	0.075870777	same
chr20	44747465	44747865	-0.596069995	0.075904425	same

chr1	91886408	91886808	-0.567173425	0.075928015	same
chr6	73654051	73654451	-0.560859465	0.075948688	same
chr7	33129205	33129605	-0.496013764	0.075978192	same
chr3	113696638	113697038	-0.579963705	0.076046393	same
chr13	72221252	72221652	-0.52464858	0.076091295	same
chr2	95402499	95402899	-0.559856536	0.076120052	same
chr8	109539974	109540374	-0.561401306	0.07613684	same
chr6	138404811	138405211	-0.577014368	0.076153689	same
chr6	154593078	154593478	-0.561456339	0.076323118	same
chr1	65254822	65255222	-0.566538729	0.076344671	same
chr2	222709262	222709662	-0.538421504	0.07635388	same
chr3	48847039	48847439	-0.609837034	0.076354638	same
chr3	52428438	52428838	-0.569067715	0.07637029	same
chr6	159981098	159981498	-0.569722035	0.07639312	same
chr22	31561151	31561551	-0.595330498	0.07640695	same
chr15	74938235	74938635	-0.581474223	0.076433683	same
chr1	121519129	121519529	-0.614299273	0.07644109	same
chr14	91113978	91114378	-0.565512743	0.076537423	same
chr19	45038920	45039320	-0.538501779	0.076586064	same
chr4	40057462	40057862	-0.583761969	0.076591472	same
chr11	117181144	117181544	-0.538128937	0.076601542	same
chr22	20937174	20937574	-0.606390966	0.076606997	same
chr8	122780337	122780737	-0.579922287	0.076620757	same
chr6	97979086	97979486	-0.593382814	0.07662971	same
chr21	44428302	44428702	-0.590989272	0.076633069	same
chr19	37469228	37469628	-0.567388904	0.076638743	same
chr19	47427647	47428047	-0.558993667	0.076653975	same
chr17	81869946	81870346	-0.420481033	0.076731	same
chr1	70528649	70529049	-0.549910261	0.076764409	same
chr11	59754855	59755255	-0.567324461	0.076804725	same
chr1	76074707	76075107	-0.55436757	0.076872747	same
chr1	101130820	101131220	-0.588627373	0.076922659	same
chr21	25607423	25607823	-0.567360729	0.076997468	same
chr12	82686304	82686704	-0.538270613	0.077017581	same
chr11	19712368	19712768	-0.559958654	0.077050153	same
chr3	14124826	14125226	-0.520699626	0.077072213	same
chr3	37860531	37860931	-0.573182365	0.077082138	same
chr2	43595940	43596340	-0.493371028	0.07709264	same
chr19	703582	703982	-0.580398862	0.077184501	same
chr6	41936124	41936524	-0.57507643	0.077187292	same
chr10	102230168	102230568	-0.573325274	0.077191436	same
chr12	88578995	88579395	-0.577857646	0.077247664	same
chr1	30718115	30718515	-0.504652334	0.077257276	same
chr5	180069974	180070374	-0.564861923	0.077294984	same
chr17	62965715	62966115	-0.564044568	0.077334758	same
chr17	81552991	81553391	-0.457835001	0.07735332	same
chr7	66654971	66655371	-0.608723642	0.077362049	same
chr8	109539345	109539745	-0.597273872	0.077365191	same
chr2	96739779	96740179	-0.574511499	0.077379089	same
chr5	146041340	146041740	-0.59085759	0.077446181	same
chr14	77616677	77617077	-0.532193418	0.07750726	same
chr16	1475466	1475866	-0.597218915	0.07752295	same
chr5	52840172	52840572	-0.607157306	0.077535154	same
chr4	61372741	61373141	-0.54119822	0.077614608	same
chr6	168147163	168147563	-0.567847105	0.07762814	same
chr9	36192156	36192556	-0.589976826	0.077700299	same
chr4	139551009	139551409	-0.585087545	0.077708827	same
chr4	122827460	122827860	-0.6081613	0.077710974	same
chr5	36606541	36606941	-0.564242448	0.077753437	same
chr7	116218036	116218436	-0.567107383	0.077822731	same
chr18	31684249	31684649	-0.595860559	0.077841137	same
chr8	117191677	117192077	-0.530397189	0.077858365	same
chr18	62522586	62522986	-0.599251227	0.077886244	same
chr1	62131213	62131613	-0.527451189	0.077891179	same
chr2	169364130	169364530	-0.564687676	0.077988286	same
chr19	48976040	48976440	-0.572179301	0.077990146	same
chr17	21511446	21511846	-0.553765413	0.078004693	same
chr16	67160685	67161085	-0.538943653	0.078032763	same
chr2	177634688	177635088	-0.599932463	0.078037567	same
chr1	103525610	103526010	-0.540549259	0.078048282	same
chr8	17246486	17246886	-0.594831108	0.078057842	same
chr19	10552638	10553038	-0.523795961	0.078117485	same
chr2	69307209	69307609	-0.571414956	0.078181211	same
chr13	71799100	71799500	-0.579755785	0.07819799	same
chr5	103562451	103562851	-0.578191186	0.078199105	same
chr13	51542353	51542753	-0.586953527	0.078219137	same

chr7	65006447	65006847	-0.556593935	0.078288596	same
chr2	43995936	43996336	-0.475336253	0.078304735	same
chr11	10540941	10541341	-0.534389813	0.078306556	same
chr1	163069329	163069729	-0.612376171	0.078328624	same
chr15	84588110	84588510	-0.539673114	0.078379736	same
chr6	75334694	75335094	-0.608140396	0.07838877	same
chr13	85142444	85142844	-0.592349628	0.078396886	same
chr16	31142110	31142510	-0.499770603	0.078426747	same
chr16	28277548	28277948	-0.584267352	0.078429167	same
chr13	30421325	30421725	-0.590559098	0.078438501	same
chr4	152779616	152780016	-0.471785271	0.078458976	same
chr7	154985633	154986033	0.5964112	0.078492571	same
chr6	170178252	170178652	-0.614738617	0.078561891	same
chr4	6709620	6710020	-0.557398388	0.07872069	same
chr16	71882667	71883067	-0.602748624	0.078763902	same
chr9	94669192	94669592	-0.598604101	0.078790021	same
chr14	91284312	91284712	-0.600603289	0.078791089	same
chr5	56909860	56910260	-0.572743054	0.078809858	same
chr2	10536968	10537368	-0.568845634	0.078825009	same
chr13	49997025	49997425	-0.564128147	0.078828086	same
chr19	50050694	50051094	-0.580159587	0.078830166	same
chr6	158869538	158869938	-0.566784544	0.078874537	same
chr8	84182155	84182555	-0.609609666	0.078923627	same
chr1	200409781	200410181	-0.589249618	0.078927799	same
chr2	163348252	163348652	-0.575481375	0.078960645	same
chr20	56521018	56521418	-0.591635839	0.078962538	same
chr12	110501903	110502303	-0.520059537	0.079005709	same
chr11	73760877	73761277	-0.572029883	0.079035627	same
chr15	45587126	45587526	-0.598579619	0.07911366	same
chr4	177441887	177442287	-0.51165404	0.079171285	same
chr11	67482933	67483333	-0.516042631	0.079191256	same
chr14	49853765	49854165	-0.591014367	0.079213647	same
chr10	5666555	5666955	-0.586105133	0.079243777	same
chr2	232432542	232432942	-0.588514352	0.079271976	same
chr8	94895655	94896055	-0.51742855	0.079277792	same
chr17	28726655	28727055	-0.47230158	0.079279147	same
chr12	88035447	88035847	-0.597315325	0.079316335	same
chr16	74695935	74696335	-0.575402001	0.079320366	same
chr16	31404369	31404769	-0.571303111	0.079559437	same
chr9	133417996	133418396	-0.511150061	0.07956412	same
chr2	85887905	85888305	-0.597491776	0.079628763	same
chr5	65035388	65035788	-0.58459414	0.079656198	same
chr9	126611879	126612279	-0.606995091	0.079656385	same
chr14	104725422	104725822	-0.54517875	0.079701542	same
chr12	42145043	42145443	-0.594493417	0.079720746	same
chr1	109619771	109620171	-0.552164139	0.079728412	same
chr1	113147027	113147427	-0.569691708	0.079781857	same
chr7	30026689	30027089	-0.546073698	0.079864999	same
chr12	4320865	4321265	-0.577531497	0.079866108	same
chr1	214332988	214333388	-0.592366852	0.079876176	same
chr1	211513983	211514383	-0.530145562	0.079878801	same
chr19	46638675	46639075	-0.498166398	0.079880916	same
chr5	52989381	52989781	-0.589329127	0.07988183	same
chr10	96089759	96090159	-0.526035191	0.079936297	same
chr12	88141943	88142343	-0.439181568	0.079942183	same
chr2	110678120	110678520	-0.605140297	0.07996928	same
chr20	59042336	59042736	-0.586098399	0.080017267	same
chr5	141619023	141619423	-0.532062095	0.080079523	same
chr4	73222833	73223233	-0.568861206	0.080144726	same
chr14	22614202	22614602	-0.550130957	0.080172279	same
chr3	53270190	53270590	-0.582323487	0.080281614	same
chr4	85474937	85475337	-0.520465024	0.080419534	same
chr5	146533145	146533545	-0.58988424	0.080499563	same
chr1	148388485	148388885	-0.472642529	0.080502007	same
chr17	50372847	50373247	-0.560143264	0.080564241	same
chr12	41365650	41366050	-0.603651215	0.080591092	same
chr10	72364382	72364782	-0.554010263	0.080625762	same
chr9	113620308	113620708	-0.58013951	0.080629358	same
chr1	183281694	183282094	-0.592906266	0.080648777	same
chr17	61543509	61543909	-0.598165596	0.080668894	same
chr5	34929184	34929584	-0.554728944	0.080682065	same
chr13	66528726	66529126	-0.574229318	0.080711311	same
chr2	166376010	166376410	-0.574263371	0.080759951	same
chrX	39755380	39755780	-0.582990185	0.080781271	same
chr6	47478025	47478425	-0.551469016	0.080799601	same
chr15	74540706	74541106	-0.524213621	0.08083477	same

chr19	38316028	38316428	-0.588315714	0.08091684	same
chr3	101779147	101779547	-0.512971307	0.08097364	same
chr11	60775462	60775862	-0.560948743	0.081038659	same
chr1	177170740	177171140	-0.582723559	0.081106042	same
chr17	44503400	44503800	-0.575965002	0.081131076	same
chrX	123777616	123778016	-0.512786236	0.081152562	same
chr17	50087628	50088028	-0.580519833	0.081164733	same
chr6	26575516	26575916	-0.506024381	0.081220287	same
chr3	120907802	120908202	-0.523156497	0.081229045	same
chrX	135520501	135520901	-0.503031934	0.081260174	same
chr18	74147988	74148388	-0.546800375	0.081274536	same
chr7	120987818	120988218	-0.537469152	0.081322361	same
chr17_GL000205v2_random	44344	44744	-0.598760303	0.081344961	same
chr1	150926016	150926416	-0.48083085	0.081376845	same
chr17	75105675	75106075	-0.563091758	0.081385235	same
chr13	44436865	44437265	-0.569575841	0.081527257	same
chr13	50529159	50529559	-0.545233509	0.081538925	same
chr13	63100740	63101140	-0.597379906	0.081552983	same
chrX	131841383	131841783	-0.595281876	0.081574976	same
chr7	135211629	135212029	-0.515149228	0.08159632	same
chr12	6614009	6614409	-0.542523363	0.081613468	same
chr7	149623279	149623679	-0.50440231	0.081618195	same
chr10	44316452	44316852	-0.589001326	0.081648459	same
chr1	225627315	225627715	-0.560447041	0.081730455	same
chr4	139606588	139606988	-0.59379637	0.081744428	same
chr3	5122598	5122998	-0.579286664	0.081768398	same
chr16	20900479	20900879	-0.490572456	0.081786558	same
chr7	66075853	66076253	-0.599967114	0.081827284	same
chr5	88883907	88884307	-0.606585548	0.081843339	same
chr19	39996828	39997228	-0.51803905	0.081946057	same
chr2	58812851	58813251	-0.584252219	0.081967108	same
chr8	120444969	120445369	-0.580327344	0.081986181	same
chr6	143678373	143678773	-0.609566593	0.082001392	same
chr11	8688683	8689083	-0.472219488	0.082078545	same
chr19	49746097	49746497	-0.551763226	0.082093277	same
chr9	79684354	79684754	-0.568034888	0.082136536	same
chrX	48476143	48476543	-0.553315165	0.082152426	same
chr11	86790861	86791261	-0.605741212	0.08221433	same
chr10	42552514	42552914	-0.537164606	0.08222008	same
chr1	206635343	206635743	-0.5466079	0.08223756	same
chr7	73701325	73701725	-0.535673559	0.082252218	same
chr10	101442091	101442491	-0.592036366	0.082309807	same
chr2	85354389	85354789	-0.476155254	0.082320421	same
chr7	149005820	149006220	-0.522776712	0.082328019	same
chr17	66192157	66192557	-0.589872514	0.082353305	same
chr8	92095417	92095817	-0.601386537	0.08241239	same
chr2	10689654	10690054	-0.559333026	0.082420498	same
chr11	7986962	7987362	-0.565809437	0.08242444	same
chr2	136118217	136118617	-0.57042325	0.082425742	same
chr5	139293714	139294114	-0.529616726	0.082446564	same
chr17	7035863	7036263	-0.569354763	0.082458456	same
chr4	82421807	82422207	-0.553892402	0.082586713	same
chr11	124875744	124876144	-0.579182829	0.082616025	same
chr7	143345410	143345810	-0.527741703	0.08263671	same
chr2	118142466	118142866	-0.584237104	0.082644083	same
chr1	151511364	151511764	-0.565353635	0.082652397	same
chr19	11504973	11505373	-0.565885925	0.082802516	same
chr16	2428887	2429287	-0.576318108	0.082891943	same
chr1	99970178	99970578	-0.548445659	0.082910692	same
chr1	53725297	53725697	-0.598824419	0.082979428	same
chr20	2873121	2873521	-0.563932273	0.082992943	same
chr10	15850442	15850842	-0.541282186	0.082996377	same
chrX	68433323	68433723	-0.504437709	0.083024285	same
chr21	16419203	16419603	-0.578602837	0.083034162	same
chr5	32174354	32174754	-0.517880527	0.083092016	same
chr14	20971285	20971685	-0.509327664	0.083092927	same
chr13	79435594	79435994	-0.531079515	0.083126381	same
chr1	205376347	205376747	-0.573157384	0.083166584	same
chr19	3276139	3276539	-0.585528851	0.083217419	same
chr12	42729187	42729587	-0.606698593	0.08324112	same
chr9	37485864	37486264	-0.52867754	0.083271852	same
chr1	45750388	45750788	-0.507625558	0.083341994	same
chr12	122064072	122064472	-0.41180293	0.083354738	same
chr4	25914146	25914546	-0.561554054	0.083360278	same
chr12	132586490	132586890	-0.576459081	0.083432642	same
chr1	63441548	63441948	-0.589545676	0.083467037	same

chr14	20652994	20653394	-0.554891836	0.08347012	same
chr1	215581809	215582209	-0.569036559	0.083557909	same
chr1	37553991	37554391	-0.589160025	0.083568911	same
chr19	43901529	43901929	-0.576451661	0.083585447	same
chr13	35630767	35631167	-0.597656065	0.083601684	same
chr17	31859358	31859758	-0.609749648	0.083619732	same
chr7	75486195	75486595	-0.586612058	0.083623712	same
chr12	71839470	71839870	-0.563149768	0.083685953	same
chr18	2571169	2571569	-0.567591365	0.083809875	same
chr1	11980111	11980511	-0.608901766	0.083978289	same
chr2	224039819	224040219	-0.606580685	0.083997839	same
chr11	34051608	34052008	-0.598069276	0.084035142	same
chr4	48016544	48016944	-0.588156191	0.084047696	same
chr1	145918747	145919147	-0.505942856	0.084058001	same
chr8	28890030	28890430	-0.503310466	0.084079653	same
chr10	62817258	62817658	-0.592154727	0.084109842	same
chrX	48917311	48917711	-0.551660645	0.084115325	same
chr10	86349365	86349765	-0.567676814	0.084142408	same
chr12	10613510	10613910	-0.579575565	0.084192458	same
chr5	160009135	160009535	-0.599395772	0.084373655	same
chr2	177224559	177224959	-0.549906889	0.084390746	same
chr5	118988528	118988928	-0.55081713	0.08445367	same
chr3	48739966	48740366	-0.491294834	0.084525602	same
chr3	139534204	139534604	-0.562530743	0.084536869	same
chr19	7903313	7903713	-0.528759661	0.084543661	same
chr13	63427268	63427668	-0.518064885	0.084588196	same
chrX	71068382	71068782	-0.518745097	0.084638829	same
chr5	59127730	59128130	-0.547589127	0.084676252	same
chr6	108294869	108295269	-0.483052193	0.084784108	same
chr16	30187127	30187527	-0.529378204	0.084860309	same
chr15	96053114	96053514	-0.586827228	0.084935844	same
chr5	174751565	174751965	-0.594152666	0.084936439	same
chr1	44793572	44793972	-0.473618556	0.084939918	same
chr11	121655688	121656088	-0.578153026	0.085012801	same
chr19	45125772	45126172	-0.595329978	0.085042304	same
chr15	70078753	70079153	-0.573458781	0.085043689	same
chr14	21437241	21437641	-0.4910209	0.085242028	same
chr5	178130194	178130594	-0.552975011	0.085256185	same
chrX	106611814	106612214	-0.504053154	0.085258866	same
chr5	173144684	173145084	-0.527812549	0.08526344	same
chr5	150001520	150001920	-0.498229886	0.085283605	same
chr21	33605734	33606134	-0.575561313	0.085289162	same
chr17	75046657	75047057	-0.434417441	0.085332507	same
chr3	12994738	12995138	-0.584618887	0.085340352	same
chr19	44845637	44846037	-0.534839574	0.085363375	same
chr10	45594282	45594682	-0.604268467	0.085399052	same
chr9	33523558	33523958	-0.578062491	0.085458951	same
chr5	178164283	178164683	-0.576390727	0.085480915	same
chr4	80335652	80336052	-0.559384975	0.085482673	same
chr19	19360853	19361253	-0.519945568	0.085580821	same
chr11	130448584	130448984	-0.579022329	0.085607949	same
chr13	57629583	57629983	-0.566191473	0.085616612	same
chr2	32356599	32356999	-0.57115684	0.08562519	same
chr6	56542601	56543001	-0.478657172	0.085650839	same
chr2	43773989	43774389	-0.586844947	0.085665101	same
chr3	142724645	142725045	-0.561918948	0.085667181	same
chr14	99683788	99684188	-0.524579622	0.085684613	same
chr6	53359310	53359710	-0.534639365	0.085699777	same
chr20	63973722	63974122	-0.50777658	0.085743812	same
chr6	85660163	85660563	-0.54989191	0.085757193	same
chr18	24397205	24397605	-0.59122101	0.085779712	same
chr17	42535863	42536263	-0.588631878	0.08578023	same
chr15	99733536	99733936	-0.58335962	0.085791089	same
chr16	88747973	88748373	-0.547013346	0.085810662	same
chr1	244919184	244919584	-0.580035059	0.085850179	same
chr3	89944545	89944945	-0.590286032	0.085853999	same
chr17	3890628	3891028	-0.504935758	0.085895096	same
chr6	90218408	90218808	-0.515343414	0.0859569	same
chr5	150000211	150000611	-0.481597549	0.086001939	same
chr16	66880280	66880680	-0.525655548	0.086030322	same
chr5	126875157	126875557	-0.547134644	0.086082109	same
chr6	13574933	13575333	-0.498000249	0.086157283	same
chr22	31970446	31970846	-0.543584251	0.086177183	same
chr2	177618667	177619067	-0.492472179	0.086193893	same
chr15	101615999	101616399	-0.533546452	0.086282479	same
chrX	74382549	74382949	-0.548716272	0.086290886	same

chr1	92831801	92832201	-0.426038964	0.086310049	same
chr3	163891737	163892137	-0.597480343	0.086442075	same
chr17	73191453	73191853	-0.534438397	0.086485042	same
chr11	68904005	68904405	-0.571206101	0.086523303	same
chr1	225653646	225654046	-0.501441111	0.086596994	same
chrX	19343502	19343902	-0.556996613	0.086689471	same
chr7	42237270	42237670	-0.570388568	0.086689536	same
chr4	102760499	102760899	-0.491549971	0.086691652	same
chr5	154038601	154039001	-0.51397097	0.086693673	same
chr18	35616579	35616979	-0.542517296	0.086712767	same
chr7	120856951	120857351	-0.581291801	0.086749582	same
chr13	48532705	48533105	-0.517169753	0.086767298	same
chr11	74592340	74592740	-0.574305492	0.086813117	same
chr19	11694902	11695302	-0.542086046	0.086866765	same
chr22	20857658	20858058	-0.553701327	0.086933039	same
chr11	115581780	115582180	-0.586343535	0.086984408	same
chr10	26524240	26524640	-0.586661173	0.086986783	same
chr10	103969395	103969795	-0.586321317	0.087026723	same
chr1	157505563	157505963	-0.58737897	0.087047047	same
chr2	130372568	130372968	-0.577630997	0.087092488	same
chr17	81023656	81024056	-0.517681592	0.087101902	same
chr13	84870160	84870560	-0.56884314	0.087182752	same
chr3	173397967	173398367	-0.575854736	0.087205991	same
chr20	33653329	33653729	-0.52246294	0.087220963	same
chrX	72309115	72309515	-0.525413382	0.087250061	same
chr3	51384718	51385118	-0.461643677	0.087255686	same
chr11	125111488	125111888	-0.486402752	0.087319402	same
chr8	144476656	144477056	-0.532314576	0.087358129	same
chr16	70523485	70523885	-0.459364419	0.087361729	same
chr8	94436983	94437383	-0.555217614	0.087399678	same
chr4	171812381	171812781	-0.586329491	0.087406926	same
chr17	1456371	1456771	-0.519954279	0.087490372	same
chr3	14948187	14948587	-0.491759802	0.087511267	same
chr12	64451736	64452136	-0.541734376	0.087710255	same
chr4	8580465	8580865	-0.571312641	0.087725346	same
chr16	23678396	23678796	-0.586824577	0.087739829	same
chr1	158087448	158087848	-0.559766193	0.08777821	same
chr16	30445516	30445916	-0.570511537	0.08779184	same
chr10	92592900	92593300	-0.510098214	0.08783765	same
chr8	124888762	124889162	-0.582053147	0.087907309	same
chr9	128191601	128192001	-0.583276632	0.087913518	same
chr17	78378259	78378659	-0.593929435	0.087959827	same
chr10	14953995	14954395	-0.497224706	0.087967821	same
chr12	57229616	57230016	-0.498794193	0.087974862	same
chr4	102620469	102620869	-0.567352313	0.08797974	same
chr5	102155177	102155577	-0.586832087	0.088026437	same
chr10	99659142	99659542	-0.519550852	0.088033695	same
chr12	56449038	56449438	-0.481689289	0.088036193	same
chr22	31399361	31399761	-0.487222518	0.088039518	same
chr1	26470488	26470888	-0.558399987	0.08806083	same
chrX	112840618	112841018	-0.4400684	0.088224851	same
chr11	65261776	65262176	-0.569280987	0.088336707	same
chr15	48963329	48963729	-0.578534781	0.088389751	same
chr2	61439400	61439800	-0.580795757	0.08839233	same
chr6	43013850	43014250	-0.485501567	0.088405896	same
chr15	49620860	49621260	-0.583602393	0.088411043	same
chr19	1837704	1838104	-0.491556297	0.088446858	same
chr1	65469569	65469969	-0.532426648	0.088484922	same
chr2	27489631	27490031	-0.471033551	0.088488079	same
chr8	98825943	98826343	-0.563760734	0.088570502	same
chr14	81400654	81401054	-0.589976639	0.088601388	same
chr3	187745427	187745827	-0.525755283	0.088606791	same
chr3	169668986	169669386	-0.554893642	0.088616687	same
chr11	65551007	65551407	-0.492071266	0.088650011	same
chr11	14891911	14892311	-0.393223537	0.088654554	same
chr19	18522190	18522590	-0.44220363	0.08867293	same
chr6	68085533	68085933	-0.591021506	0.088689563	same
chr19	55606304	55606704	-0.544999199	0.088767223	same
chr15	43330370	43330770	-0.544377827	0.088817555	same
chr5	157743292	157743692	-0.574354582	0.088864775	same
chr7	12687003	12687403	-0.554516682	0.088869167	same
chr21	33588776	33589176	-0.551219611	0.088940527	same
chr20	34363129	34363529	-0.509478252	0.088949575	same
chr2	104702306	104702706	-0.595379623	0.088983574	same
chr4	52925777	52926177	-0.532703774	0.08904649	same
chr19	5695298	5695698	-0.450375248	0.089047904	same

chr16	58638376	58638776	-0.574365416	0.08907631	same
chr12	89709566	89709966	-0.560083655	0.08912449	same
chr15	67543285	67543685	-0.541134374	0.089137468	same
chr17	39927690	39928090	-0.529545572	0.089141906	same
chr2	74380123	74380523	-0.555565909	0.089146754	same
chr20	47501611	47502011	-0.577133521	0.089173348	same
chr11	47152972	47153372	-0.558888921	0.089177082	same
chr10	12068601	12069001	-0.498555527	0.089253991	same
chrX	115968696	115969096	-0.518977398	0.089260531	same
chr1	161726043	161726443	-0.530862296	0.089270607	same
chr9	4297483	4297883	-0.567585453	0.08927949	same
chr16	87490906	87491306	-0.54802424	0.089314436	same
chr11	73779425	73779825	-0.473553616	0.089394614	same
chr13	19863434	19863834	-0.517378393	0.089408812	same
chrX	103376130	103376530	-0.483463812	0.089434067	same
chr1	210074682	210075082	-0.577350413	0.089520019	same
chr5	179762311	179762711	-0.532863419	0.08957819	same
chr1	89524129	89524529	-0.489581346	0.089652079	same
chr4	37686468	37686868	-0.533850396	0.089712964	same
chr9	95580946	95581346	-0.591148292	0.089730858	same
chr11	47214388	47214788	-0.527924015	0.089776263	same
chr9	113657866	113658266	-0.569254277	0.089780825	same
chr5	62403715	62404115	-0.49017458	0.089809598	same
chr3	184812068	184812468	-0.560122227	0.089903215	same
chr4	8440348	8440748	-0.548973048	0.089935633	same
chr15	77083882	77084282	-0.521546307	0.089942295	same
chr4	83334808	83335208	-0.548830986	0.08995968	same
chr5	112737599	112737999	-0.513113752	0.089968959	same
chr4	3781013	3781413	-0.574678636	0.089983079	same
chr16	5071889	5072289	-0.525818156	0.089998889	same
chr22	50526792	50527192	-0.483303751	0.090010619	same
chr13	65967081	65967481	-0.575537299	0.090020604	same
chr4	2009791	2010191	-0.563962276	0.090106741	same
chr15	66356406	66356806	-0.518271352	0.090141397	same
chr4	89055349	89055749	-0.583753085	0.090293987	same
chr1	207797324	207797724	-0.553755673	0.090358306	same
chr2	99154827	99155227	-0.530072134	0.090414801	same
chr16	88770848	88771248	-0.427613219	0.090420758	same
chr14	104827468	104827868	-0.549720516	0.090445371	same
chr1	17239413	17239813	-0.557400788	0.090470748	same
chr15	63504364	63504764	-0.529682804	0.090477749	same
chr14	99946894	99947294	-0.568912407	0.090487642	same
chr3	53927352	53927752	-0.580268321	0.090493189	same
chr4	76213191	76213591	-0.559814136	0.090629743	same
chr22	43152385	43152785	-0.548603431	0.090634851	same
chr22	19448094	19448494	-0.454677734	0.090689077	same
chr7	128343566	128343966	-0.550350435	0.090765069	same
chr19	13747471	13747871	-0.560591159	0.090816098	same
chr8	47841778	47842178	0.577170323	0.090835123	same
chr1	117368351	117368751	-0.536136143	0.090920942	same
chr13	100088685	100089085	-0.560295191	0.09098741	same
chr16	25049903	25050303	-0.542508405	0.090991958	same
chr15	70701923	70702323	-0.559691317	0.091024595	same
chr15	68306362	68306762	-0.547589211	0.091069225	same
chr4	2478362	2479126	-0.559947087	0.091087782	same
chr19	45730420	45730820	-0.531781418	0.091098308	same
chr12	50283703	50284103	-0.585211839	0.091119722	same
chr3	38650032	38650432	-0.552002162	0.091152085	same
chr9	125904356	125904756	-0.576155587	0.091177431	same
chr4	75630397	75630797	-0.487819793	0.091199904	same
chr5	172697118	172697518	-0.554259127	0.091305729	same
chrX	102713214	102713614	-0.543520115	0.091328616	same
chr13	73922564	73922964	0.570520209	0.091330768	same
chr22	41197597	41197997	-0.532708803	0.091366911	same
chr19	9827537	9827937	-0.523725657	0.09146454	same
chr8	100150309	100150709	-0.519178103	0.091491	same
chr15	43777354	43777754	-0.576130013	0.091651021	same
chr3	98039417	98039817	-0.582517703	0.091665939	same
chr4	107989363	107989763	-0.532653559	0.091692772	same
chr1	117758837	117759237	-0.532927725	0.091712788	same
chr5	43007983	43008383	-0.569627374	0.091725938	same
chr3	139344091	139344491	-0.483167878	0.091809765	same
chr1	246566312	246566712	-0.493697642	0.091830955	same
chr13	23570294	23570694	-0.581378989	0.091873839	same
chr10	68955815	68956215	-0.449189943	0.091885526	same
chr7	2242527	2242927	-0.511332357	0.09199989	same

chr1	223090043	223090443	-0.522234702	0.092088032	same
chr1	151156139	151156539	-0.542159183	0.092146676	same
chr7	87345224	87345624	-0.465813405	0.092205651	same
chr12	24069877	24070277	-0.573532949	0.092222565	same
chr20	3408188	3408588	-0.4952899	0.09223523	same
chr12	123671014	123671414	-0.533696547	0.092279599	same
chr6	93105006	93105406	-0.586716833	0.092310906	same
chr20	45857243	45857643	-0.484504807	0.0923491	same
chr5	76818597	76818997	-0.502216911	0.092350858	same
chr1	172727909	172728309	-0.583665474	0.092387907	same
chr4	30836960	30837360	-0.585363162	0.092417763	same
chr2	45610692	45611092	-0.533599751	0.092418502	same
chr1	150187402	150187802	-0.578591013	0.092420102	same
chr5	43105553	43105953	-0.535831752	0.092421753	same
chr2	174486522	174486922	-0.52976243	0.092504258	same
chr4	158782839	158783239	-0.552278722	0.092521953	same
chr15	43824391	43824791	-0.566186235	0.092536439	same
chr1	231420599	231420999	-0.519759302	0.0926218	same
chr11	102268418	102268818	-0.573061209	0.092648494	same
chr1	109655961	109656361	-0.523504072	0.092659268	same
chr17	74775961	74776361	-0.505385025	0.092699319	same
chr22	24156830	24157230	-0.473146104	0.092900524	same
chr19	4414594	4414994	-0.537222032	0.093061943	same
chr3	11643575	11643975	-0.537394725	0.093138598	same
chr7	33886207	33886607	-0.585873925	0.09315399	same
chr18	46104175	46104575	-0.555998855	0.093156228	same
chr6	28080246	28080646	-0.534726308	0.093193146	same
chr7	42932218	42932618	-0.518784319	0.093227182	same
chr12	106074848	106075248	-0.570085242	0.093239441	same
chr6	55913642	55914042	-0.584524013	0.093284741	same
chr12	130838332	130838732	-0.576225918	0.093296694	same
chr3	120623101	120623501	-0.520212105	0.093328523	same
chr9	2843877	2844277	-0.520979595	0.093386869	same
chr19	12775610	12776010	-0.579923001	0.093412055	same
chr22	41800392	41800792	-0.508023432	0.093441281	same
chr19	18373955	18374355	-0.562331742	0.093483015	same
chr5	65480469	65480869	-0.56347431	0.093517064	same
chr4	6269475	6269875	-0.580281405	0.093573367	same
chr17	4710023	4710423	-0.473114267	0.093579438	same
chr2	232605912	232606312	-0.523266438	0.093584407	same
chr6	166097819	166098219	-0.529069815	0.093619395	same
chr1	151254545	151254945	-0.517503476	0.093664747	same
chr14	22766723	22767123	-0.534570516	0.093748965	same
chr3	52144374	52144774	-0.548263401	0.093775704	same
chrX	49155905	49156305	-0.480580543	0.093776732	same
chr12	57591132	57591532	-0.540888536	0.093802085	same
chr5	160421769	160422169	-0.54394959	0.093913803	same
chr4	155666897	155667297	-0.550465594	0.093921835	same
chr7	2661188	2661588	-0.544649513	0.093941341	same
chr2	219206421	219206821	-0.52333672	0.0939433	same
chr7	14836543	14836943	-0.570366153	0.093949687	same
chrX	154459430	154459830	-0.535985989	0.093966793	same
chr11	66051971	66052371	-0.521815909	0.094011706	same
chr12	62466729	62467129	-0.570653772	0.094071839	same
chr9	35162058	35162458	-0.526974801	0.094118311	same
chr1	67686487	67686887	-0.447892581	0.094127905	same
chr1	28248450	28248850	-0.475472369	0.094134806	same
chr20	5610017	5610417	-0.551298281	0.094158167	same
chr1	941139	941539	-0.468055399	0.094161014	same
chr2	219387334	219387734	-0.542366516	0.094169015	same
chrX	76173065	76173465	-0.522664144	0.094186517	same
chr15	82809250	82809650	-0.497273349	0.094187288	same
chr11	18588347	18588747	-0.56086552	0.094212351	same
chr12	6493396	6493796	-0.50295283	0.09422323	same
chr11	17276300	17276700	-0.538612021	0.094283221	same
chr3	50337429	50337829	-0.440223683	0.094299307	same
chr19	10870107	10870507	-0.550610682	0.094319607	same
chr19	9785726	9786126	-0.553256202	0.094354067	same
chr3	66038943	66039343	-0.565062102	0.094363544	same
chr11	16613735	16614135	-0.51119091	0.094405466	same
chr2	55620018	55620418	-0.50491756	0.094432685	same
chr15	85840648	85841048	-0.563426945	0.094481455	same
chr2	229780867	229781267	-0.579509617	0.094650825	same
chr7	20330008	20330408	-0.546259682	0.094695504	same
chrX	102599231	102599631	-0.484743257	0.094696125	same
chr12	50025295	50025695	-0.524309548	0.094724535	same

chr2	127526536	127526936	-0.491519849	0.09480019	same
chr1	74324604	74325004	-0.570423234	0.094868723	same
chr16	89687141	89687541	-0.519697667	0.094878597	same
chr22	41446940	41447340	-0.446098906	0.094930721	same
chr11	27019072	27019472	-0.567650082	0.09494047	same
chr21	33553090	33553490	-0.545902968	0.094957186	same
chr1	30782677	30783077	-0.483616075	0.095042169	same
chr12	44876433	44876833	-0.548308962	0.09510674	same
chr1	155044981	155045381	-0.51794845	0.095128461	same
chr1	51728582	51728982	-0.580239832	0.095132872	same
chr19	45902767	45903167	-0.562351009	0.095163435	same
chr15	67779642	67780042	-0.552051528	0.095271298	same
chr5	177592143	177592543	-0.538679418	0.095295345	same
chr3	158570933	158571333	-0.541812934	0.095337375	same
chr19	6412140	6412540	-0.471915824	0.095395365	same
chr16	31034128	31034528	-0.444019515	0.095485616	same
chr1	201946375	201946775	-0.478666736	0.09551797	same
chr9	33403238	33403638	-0.560604086	0.095523393	same
chr5	58460408	58460808	-0.573138576	0.095581558	same
chrX	123732700	123733100	-0.515877902	0.095607164	same
chr15	45198582	45198982	-0.45028617	0.095630334	same
chr6	104737570	104737970	-0.58276373	0.095652895	same
chr20	543672	544072	-0.538061661	0.095796371	same
chr9	7165116	7165516	-0.474299959	0.095796489	same
chr14	102139431	102139831	-0.492084589	0.095844498	same
chr17	44511644	44512044	-0.570885149	0.095858603	same
chr11	65588662	65589062	-0.491524178	0.095868792	same
chr20	62793885	62794285	-0.526974599	0.095890362	same
chr6	138966011	138966411	-0.541678884	0.095892273	same
chr2	42810540	42810940	-0.529436501	0.095894599	same
chr1	8878711	8879111	-0.541095531	0.095898729	same
chr19	29844793	29845193	-0.505683796	0.095930612	same
chr6	158817265	158817665	-0.566902702	0.09602111	same
chr12	125017628	125018028	-0.567572176	0.096034565	same
chr17	63961489	63961889	-0.553983867	0.096046377	same
chr1	37989773	37990173	-0.550382162	0.096063486	same
chr2	128028033	128028433	-0.581400503	0.096108413	same
chr19	8608903	8609303	-0.564191404	0.096119601	same
chr1	45339999	45340399	-0.480294859	0.096157226	same
chr5	171418822	171419222	-0.478722981	0.096237276	same
chr14	75293929	75294329	-0.561400698	0.096260078	same
chr3	66501183	66501583	-0.553468755	0.096350458	same
chr10	112446811	112447211	-0.516944289	0.096457977	same
chr1	213154203	213154603	-0.581358957	0.096471867	same
chr6	117906797	117907197	-0.567357381	0.096477768	same
chr1	33472434	33472834	-0.47869636	0.096481248	same
chr2	55519451	55519851	-0.466294386	0.096563232	same
chr12	56127959	56128359	-0.414032172	0.096696928	same
chr19	4041946	4042346	-0.533905634	0.096721224	same
chr11	63802998	63803398	-0.523152556	0.096728879	same
chr11	64803690	64804090	-0.497704131	0.096829301	same
chr15	77071121	77071521	-0.550453443	0.096839124	same
chr1	170074455	170074855	-0.437153951	0.096856129	same
chr12	6825679	6826079	-0.515576501	0.096892463	same
chr1	161421969	161422369	-0.511456233	0.096900152	same
chr7	151227166	151227566	-0.416565584	0.096945452	same
chr6	156780394	156780794	-0.555340446	0.09696545	same
chr8	71356691	71357091	-0.560602863	0.097076541	same
chr12	66302462	66302862	-0.515124138	0.097275063	same
chrX	108736483	108736883	-0.475609661	0.097343323	same
chr4	13627778	13628178	-0.566511351	0.097351278	same
chr19	45478685	45479085	-0.488455237	0.097355429	same
chr11	73567123	73567523	-0.583357546	0.097366568	same
chr1	153881058	153881458	-0.520760802	0.097401199	same
chr16	48365677	48366077	-0.540617623	0.097465508	same
chr15	40694730	40695130	-0.516228816	0.097483077	same
chr10	88387099	88387499	-0.565036082	0.097503809	same
chr14	80941388	80941788	-0.480682539	0.097532725	same
chr7	152759315	152759715	-0.560053037	0.097591901	same
chr12	25648413	25648813	-0.565695178	0.097609483	same
chr8	124271822	124272222	-0.528773334	0.097654475	same
chr2	39437637	39438037	-0.563060719	0.097679725	same
chr10	70816382	70816782	-0.539413179	0.097692632	same
chr20	34303057	34303457	-0.512097835	0.097787991	same
chr15	100349949	100350349	-0.539910089	0.097818458	same
chr3	69084694	69085094	-0.546557399	0.097838562	same

chr22	36455148	36455548	-0.545682548	0.097879071	same
chr1	201042789	201043189	-0.540671502	0.097909399	same
chr17	31095518	31095918	-0.570071539	0.097926133	same
chr12	120446226	120446626	-0.478808679	0.097954635	same
chr15	75903770	75904170	-0.475244001	0.09799991	same
chr14	105352257	105352657	0.563459563	0.098039513	same
chr17	19648586	19648986	-0.4479283	0.098153672	same
chr2	238234358	238234758	-0.516451878	0.098402151	same
chr3	119677194	119677594	-0.543337423	0.098473144	same
chr16	2023259	2023659	-0.469657374	0.098482885	same
chr16	10385906	10386306	-0.568638142	0.098496317	same
chr1	162631198	162631598	-0.540326574	0.098501111	same
chr13	63954831	63955231	-0.534875433	0.098509083	same
chr2	74120329	74120729	-0.567745976	0.098541808	same
chr3	160870088	160870488	-0.553494354	0.098699839	same
chr17	44774947	44775347	-0.494917072	0.098724154	same
chr12	109097583	109097983	-0.51401947	0.09879677	same
chr6	167826464	167826864	0.537900492	0.09879681	same
chr12	53940505	53940905	-0.549919894	0.098822056	same
chr1	227563514	227563914	-0.391047047	0.098866907	same
chr11	126303864	126304264	-0.472429932	0.098955254	same
chr3	57227218	57227618	-0.54851556	0.098986857	same
chr1	87718338	87718738	-0.53149312	0.098992343	same
chr10	119892642	119893042	-0.551135861	0.099057412	same
chr10	133419217	133419617	0.516051673	0.099065173	same
chr19	3505753	3506153	-0.465498503	0.099144373	same
chr19	7551180	7551580	-0.55281929	0.099223957	same
chr1	152036713	152037113	-0.54618545	0.099227396	same
chr1	213988008	213988408	-0.513456531	0.099278342	same
chr10	100228811	100229211	-0.506399677	0.099377627	same
chr6	4018478	4018878	-0.47669669	0.099403511	same
chr3	195909779	195910179	-0.549371539	0.099430248	same
chr5	72816906	72817306	-0.564722934	0.099479099	same
chr11	33997130	33997530	-0.558548044	0.099548158	same
chr2	120012964	120013364	-0.566978742	0.099591611	same
chr2	190881920	190882320	-0.556058556	0.099613381	same
chr9	113410324	113410724	-0.472970962	0.099631225	same
chr3	184316613	184317013	-0.535841306	0.099721091	same
chr17	49968858	49969258	-0.567360742	0.099755992	same
chr13	29847929	29848329	-0.574569778	0.099764135	same
chr22	24555327	24555727	-0.420462859	0.099776895	same
chr3	52278677	52279077	-0.537003674	0.099800991	same
chr1	85581054	85581735	-0.476655346	0.099802652	same
chr6	111871636	111872036	-0.548444168	0.100009968	same
chr7	151460006	151460406	-0.462326466	0.100015062	same
chr5	154941285	154941685	-0.547838328	0.100042307	same
chr4	113978781	113979181	-0.575358936	0.100102212	same
chrX	119667330	119667730	-0.528415302	0.100210401	same
chr7	155003847	155004247	-0.529432657	0.100217063	same
chr11	13462857	13463257	-0.549844741	0.100228246	same
chr1	156677361	156677761	-0.560970741	0.100393899	same
chr1	193070006	193070406	-0.553158504	0.100402764	same
chr3	49007377	49007777	-0.395918569	0.100441814	same
chr19	19385447	19385847	-0.44801	0.100449156	same
chr6	25992721	25993121	-0.512836117	0.100471146	same
chr2	180125330	180125730	-0.56346446	0.100472198	same
chr1	154324764	154325164	-0.459976711	0.100489068	same
chrX	69158518	69158918	-0.539102812	0.100508483	same
chr9	74952996	74953396	-0.575291668	0.100535538	same
chr1	176206035	176206435	-0.572140003	0.100538268	same
chr22	17084627	17085027	-0.537196534	0.100593279	same
chr16	84738515	84738915	-0.516893526	0.100668256	same
chr7	40134436	40134836	-0.514846612	0.100773281	same
chr17	8435642	8436042	-0.536165819	0.100790636	same
chr13	61415069	61415469	-0.541334596	0.100825816	same
chr9	129885269	129885669	-0.538891766	0.100843078	same
chr2	97645681	97646081	-0.522640319	0.100889903	same
chr19	57477287	57477687	-0.542700274	0.100921776	same
chr6	75349320	75349720	-0.563396165	0.10094432	same
chr5	149345084	149345484	-0.490837116	0.100953511	same
chr17_GL000205v2_random	56513	56913	-0.503296339	0.100992121	same
chr1	36397450	36397850	-0.474556407	0.101029648	same
chr17	50297485	50297885	-0.569446984	0.101086673	same
chr13	76473423	76473823	-0.552249422	0.101255822	same
chr19	12484534	12484934	-0.529772596	0.101310281	same
chr6	34756820	34757220	-0.527522824	0.101331657	same

chr6	108261070	108261470	-0.548905594	0.101483752	same
chr15	101652480	101652880	-0.548855873	0.10149726	same
chr11	77637482	77637882	-0.511082887	0.101501704	same
chrX	154526951	154527351	-0.494488204	0.101548385	same
chr1	203026349	203026749	-0.52188777	0.101585169	same
chr2	27628742	27629142	-0.537699651	0.101615633	same
chr5	172770944	172771344	-0.506291473	0.1016414	same
chr1	67700697	67701097	-0.432804466	0.101708544	same
chr11	9529139	9529539	-0.548352149	0.101754171	same
chr6	27177940	27178340	-0.510013775	0.101822142	same
chr12	121663801	121664201	-0.480283999	0.101900776	same
chr11	57657398	57657798	-0.441623906	0.101908418	same
chr4	108620415	108620815	-0.559023968	0.101915132	same
chr3	48446353	48446753	-0.559496553	0.101961652	same
chr10	71235711	71236111	-0.4801926	0.101996786	same
chr17	30708331	30708731	-0.364263317	0.102336859	same
chr11	124739543	124739943	-0.528484024	0.102348125	same
chr5	73498174	73498574	-0.498190317	0.102365532	same
chr15	91049834	91050234	-0.533650624	0.102380862	same
chr19	53977871	53978271	-0.513987314	0.102490359	same
chr1	39609557	39609957	-0.527256612	0.102491027	same
chr12	55729371	55729771	-0.508507368	0.102508447	same
chr6	39059524	39059924	-0.55649012	0.102526233	same
chr1	248873528	248873928	-0.47318705	0.102558607	same
chr22	41597252	41597652	-0.497465309	0.102600071	same
chr19	52007957	52008357	-0.509690902	0.102634861	same
chr5	176647130	176647530	-0.516042746	0.102652824	same
chr19	35510161	35510561	-0.511192627	0.1027896	same
chr1	119647517	119647917	-0.495716195	0.102817987	same
chr19	40623752	40624152	-0.462246417	0.102818761	same
chr3	38453409	38453809	-0.543123652	0.102915109	same
chr11	8594208	8594608	-0.549345082	0.102915278	same
chr8	123396360	123396760	-0.557667225	0.10294783	same
chr2	112254751	112255151	-0.490093608	0.102973424	same
chr8	100950320	100950720	-0.483168413	0.103009835	same
chr3	121545706	121546106	-0.506867259	0.10302466	same
chr3	49786662	49787062	-0.518410027	0.103036759	same
chr6	35474201	35474601	-0.405563028	0.103080139	same
chr17	42107807	42108207	-0.523344984	0.103247196	same
chr4	121950781	121951181	-0.553820232	0.103272668	same
chr7	155482055	155482455	-0.462029565	0.103293947	same
chr12	113185296	113185696	-0.462114277	0.103296483	same
chr9	78030345	78030745	-0.540155356	0.103471651	same
chr6	3118226	3118626	-0.487487099	0.10352146	same
chr3	155027106	155027506	-0.555819652	0.103640139	same
chr17	43388412	43388812	-0.471607722	0.103650279	same
chr15	73636306	73636706	-0.517428387	0.103784838	same
chrX	152926210	152926610	-0.5329521	0.103802012	same
chr1	28437902	28438302	-0.515612525	0.103804373	same
chr7	2757993	2758393	-0.528817317	0.103862704	same
chr6	27810112	27810512	-0.51587689	0.103912092	same
chr1	59147065	59147465	-0.540798555	0.103994001	same
chr1	28581799	28582199	-0.437468839	0.104024283	same
chr13	61880868	61881268	-0.459235229	0.104044472	same
chr15	60393077	60393477	-0.549094414	0.10410178	same
chr18	25351904	25352304	-0.54499108	0.104141338	same
chr12	14770393	14770793	-0.486569373	0.10428177	same
chr18	48901807	48902207	0.554426989	0.104286278	same
chr19	50608091	50608491	-0.533238999	0.104291325	same
chr17	7455466	7455866	-0.489625457	0.104301557	same
chr4	70901934	70902334	-0.501770827	0.104341736	same
chr11	236393	236793	-0.540465202	0.10434783	same
chr10	72298178	72298578	-0.543672733	0.104348416	same
chr1	921226	921626	-0.508208241	0.104349211	same
chr16	18926441	18926841	-0.479488236	0.104360147	same
chr8	144478869	144479269	-0.478830865	0.104467644	same
chr17	77471070	77471470	-0.513631366	0.104521051	same
chr3	23836596	23836996	-0.540097907	0.104534484	same
chr3	138118783	138119183	-0.512444404	0.104566829	same
chr19	1590718	1591118	-0.483725678	0.104579585	same
chr1	48093805	48094205	-0.54472558	0.104590067	same
chr11	43880613	43881013	-0.528068066	0.104606112	same
chr1	214280306	214280706	-0.522242093	0.104623562	same
chr19	18469505	18470177	-0.457982547	0.104632635	same
chr5	43020175	43020575	-0.541803518	0.104642377	same
chr5	124656007	124656407	-0.504468309	0.104679275	same

chr8	134905669	134906069	-0.55091285	0.104711732	same
chr11	65013637	65014037	-0.506445002	0.104748066	same
chr3	55486237	55486637	-0.526000808	0.104828262	same
chr13	77592949	77593349	-0.52475674	0.104841824	same
chr16	29679188	29679588	-0.558141275	0.104868308	same
chr6	10747497	10747897	-0.507063623	0.104888546	same
chr9	129110826	129111226	-0.520162323	0.104896473	same
chr16	21300833	21301233	-0.490122994	0.104935766	same
chr8	8227997	8228397	-0.571937334	0.104952988	same
chr3	51941869	51942269	-0.448865115	0.104972572	same
chr7	144384876	144385276	-0.532885533	0.10502503	same
chr1	176207377	176207777	-0.510028388	0.10505008	same
chr7	32490145	32490545	-0.531954161	0.10517523	same
chr17	21043318	21043718	-0.470047763	0.105308154	same
chr12	120235265	120235665	-0.507337721	0.10531333	same
chr1	155172768	155173168	-0.462929182	0.10535816	same
chr14	102509969	102510369	-0.544033768	0.105395182	same
chr7	38178072	38178472	-0.515632539	0.105401236	same
chr20	16573296	16573696	-0.455742185	0.105583162	same
chr6	116279613	116280013	-0.436781286	0.105596007	same
chr2	238060515	238060915	-0.571251424	0.105677553	same
chr10	113855563	113855963	-0.528671708	0.105715052	same
chr7	149195155	149195555	-0.459484224	0.105746111	same
chrX	48597750	48598150	-0.462681005	0.105791475	same
chr9	129569657	129570057	-0.531678495	0.105798751	same
chr9	131383308	131383708	-0.485352875	0.105828872	same
chr5	134875009	134875409	-0.529262689	0.105851431	same
chr9	32552249	32552649	-0.511665776	0.105945854	same
chr1	239803724	239804124	-0.564321318	0.105949496	same
chr19	49866965	49867365	-0.389169672	0.106007067	same
chr4	95640100	95640500	-0.549149606	0.106050247	same
chr5	79990912	79991312	-0.515907161	0.106116991	same
chr16	2970279	2970679	-0.447056759	0.106196328	same
chr6	53545064	53545464	-0.512818156	0.106260216	same
chr11	128061368	128061768	-0.558102103	0.106331121	same
chr16	87383796	87384196	-0.443260094	0.106371848	same
chr8	78666053	78666453	-0.50277656	0.106409622	same
chr1	26306427	26306827	-0.536892112	0.106430815	same
chr6	110958502	110958902	-0.480782048	0.106435196	same
chr10	84139433	84139833	-0.482879204	0.106565494	same
chr11	43311915	43312315	-0.512800151	0.106664415	same
chr21	33894897	33895297	-0.49921676	0.106683521	same
chr17	42824008	42824408	-0.499124656	0.106702176	same
chr16	68084535	68084935	-0.46319196	0.106732199	same
chr2	197640441	197640841	-0.54819543	0.106755686	same
chr5	88141128	88141528	-0.523652542	0.106786551	same
chr7	138979579	138979979	-0.523962533	0.106836659	same
chr6	159252928	159253328	-0.532369356	0.106849474	same
chr1	211579039	211579439	-0.532925473	0.10685131	same
chr4	112285781	112286181	-0.545627748	0.106855881	same
chr1	144418861	144419261	-0.553668567	0.106875303	same
chr17	35089500	35089900	-0.540768151	0.106894146	same
chr17	75012608	75013008	-0.437431289	0.10690004	same
chr15	41774101	41774501	-0.439051498	0.106965484	same
chr3	183635532	183635932	-0.544480332	0.106997563	same
chr7	149178957	149179357	-0.534409272	0.1070794	same
chr16	1611686	1612086	-0.432316103	0.107104394	same
chr22	38057159	38057559	-0.513907201	0.107126543	same
chr19	35612903	35613303	-0.513848599	0.107126635	same
chr7	103074503	103074903	-0.518232058	0.107195833	same
chr22	50498352	50498752	-0.483501958	0.107282898	same
chr12	123458372	123458772	-0.460606802	0.10735924	same
chr5	35617719	35618119	-0.509930274	0.107413445	same
chr7	73578685	73579085	-0.494468353	0.107461005	same
chr1	1063748	1064148	-0.526189438	0.107549694	same
chr19	49019441	49019841	-0.46536908	0.107644135	same
chr8	104325729	104326129	-0.528412657	0.107758518	same
chr19	18275410	18275810	-0.49330362	0.107811469	same
chr1	116569856	116570256	-0.548186476	0.107822336	same
chr4	103198096	103198496	-0.490568471	0.107846595	same
chr8	80874428	80874828	-0.518114648	0.107876773	same
chr4	99088867	99089267	-0.512205407	0.107955782	same
chr21	43771242	43771642	-0.492340767	0.108026756	same
chr19	52942144	52942544	-0.553719052	0.108055333	same
chr9	33375161	33375561	-0.526120956	0.108100459	same
chr7	28854036	28854436	-0.518696368	0.108111088	same

chr22	18077792	18078192	-0.523848175	0.108197827	same
chr11	61684261	61684661	-0.549380787	0.108206138	same
chr20	35542069	35542469	-0.478816287	0.108336953	same
chr1	43119813	43120213	-0.540256314	0.108403017	same
chr6	27631127	27631527	-0.525665203	0.10841361	same
chr20	327279	327679	-0.493904865	0.108445238	same
chr16	57422152	57422552	-0.558228666	0.10845909	same
chr21	33716067	33716467	-0.526225853	0.108506353	same
chr7	24980303	24980703	-0.513148043	0.108525764	same
chr1	997123	997523	-0.461850465	0.108555346	same
chr7	146654053	146654453	-0.528340363	0.108679135	same
chrX	115561002	115561402	-0.406794293	0.108747228	same
chr7	64903122	64903522	-0.528912548	0.108756762	same
chr5	1167831	1168231	-0.541687807	0.108827013	same
chr6	144063646	144064046	-0.533603727	0.108863643	same
chr2	201125471	201125871	-0.53993031	0.108880314	same
chr4	47836839	47837239	-0.553750241	0.108959569	same
chr11	130069140	130069540	-0.500638752	0.109006783	same
chr15	44195316	44195716	-0.556251473	0.109016795	same
chrX	155612580	155612980	-0.484154061	0.109030871	same
chrX	11111424	11111824	-0.535877645	0.109034464	same
chr9	128724067	128724467	-0.438746988	0.109039521	same
chr19	10106161	10106561	-0.481686643	0.109113325	same
chr1	159916154	159916554	-0.46113978	0.109120428	same
chr1	205229852	205230252	-0.528091092	0.109180048	same
chr8	85107149	85107549	-0.517827503	0.109237502	same
chr15	72375943	72376343	-0.425411294	0.109237521	same
chr16	50806336	50806736	-0.546005382	0.109242874	same
chr19	1411117	1411517	-0.485850235	0.109247897	same
chr6	13813567	13813967	-0.506321616	0.109277066	same
chr13	79246056	79246456	-0.516655624	0.109295869	same
chr12	106986816	106987216	-0.459987121	0.109329893	same
chr1	57623429	57623829	-0.531388086	0.109387147	same
chr1	228500939	228501339	-0.457991161	0.109400559	same
chr17	58218872	58219272	-0.515233607	0.109491137	same
chr3	52197955	52198355	-0.507502162	0.109516282	same
chr12	53251302	53251702	-0.518862282	0.109590578	same
chr5	151447551	151447951	-0.523977169	0.10959854	same
chr1	244439493	244439893	-0.502180038	0.109653187	same
chr19	12933717	12934117	-0.448468082	0.109657854	same
chr6	158405446	158405846	-0.506702391	0.109664215	same
chr12	43805538	43805938	-0.535470614	0.109700935	same
chr22	37561628	37562028	-0.473685221	0.109767622	same
chrX	53686412	53686812	-0.509774536	0.109781031	same
chr9	21995032	21995432	-0.529496818	0.109920146	same
chr12	54259406	54259806	-0.51158916	0.109924958	same
chr3	120742276	120742676	-0.447175593	0.109932003	same
chr6	28281535	28281935	-0.554005008	0.10995159	same
chr3	13451598	13451998	-0.494631195	0.110211528	same
chr3	14651419	14651819	-0.414518832	0.110219747	same
chr2	153476908	153477308	-0.550378944	0.110239175	same
chr8	69994602	69995002	-0.538120526	0.110297602	same
chr2	108636230	108636630	-0.511360738	0.110305112	same
chr4	143335781	143336181	-0.522535471	0.110332332	same
chr17	39461497	39461897	-0.500956653	0.110352211	same
chr10	70170529	70170929	-0.486278439	0.110397908	same
chr7	107563702	107564102	-0.538541034	0.110415011	same
chr7	56106312	56106712	-0.499674031	0.11044217	same
chr8	48284058	48284458	-0.523547633	0.110477247	same
chr12	93377445	93377845	-0.526869525	0.11061773	same
chr1	168137249	168137649	-0.543490359	0.110619928	same
chr16	71461949	71462349	-0.545409328	0.110705083	same
chr4	107736867	107737267	-0.552432005	0.110759906	same
chr17	59093446	59093846	-0.515026141	0.110842704	same
chr17	32483365	32483765	-0.496716759	0.110845722	same
chr9	33391216	33391616	-0.533930804	0.110855142	same
chr17	45148250	45148650	-0.406469149	0.110886545	same
chr13	76991792	76992192	-0.473757014	0.110940608	same
chr22	23183086	23183486	-0.54748512	0.110971884	same
chr7	122886974	122887374	-0.544327101	0.111017819	same
chr7	140231106	140231506	-0.53043708	0.111048906	same
chr1	44786181	44786581	-0.488143764	0.111260367	same
chr11	65182105	65182505	-0.509306456	0.111370669	same
chr8	54135069	54135469	-0.510022346	0.111460533	same
chr10	125691018	125691418	-0.534390539	0.111482355	same
chr4	139651414	139651814	-0.54801591	0.111489969	same

chr11	6315750	6316150	-0.55056396	0.111565365	same
chr4	167234419	167234819	-0.523600835	0.111721027	same
chr8	22108585	22108985	-0.545153141	0.111742905	same
chr8	124973112	124973512	-0.508825614	0.111770078	same
chr7	35253742	35254142	-0.515114359	0.111782856	same
chr10	84613495	84613895	-0.533538463	0.111840429	same
chr7	139370810	139371210	-0.529246671	0.111907067	same
chr8	23529762	23530162	-0.518459885	0.111909384	same
chr3	11718528	11718928	-0.539504558	0.111912365	same
chr10	100913065	100913465	-0.475024132	0.112011453	same
chr11	72091004	72091404	-0.526302735	0.112040082	same
chr20	35092852	35093252	-0.516483418	0.112054814	same
chr16	72664667	72665067	-0.49111615	0.112117455	same
chr13	99087650	99088050	-0.528897937	0.112195119	same
chr17	50560105	50560505	-0.532434248	0.112240384	same
chr19	36573072	36573472	-0.465746432	0.112336414	same
chr12	14384964	14385364	-0.478528643	0.112487058	same
chr7	35003690	35004090	-0.553492173	0.11252288	same
chr11	61161638	61162038	-0.532359767	0.112559016	same
chr11	63872038	63872438	-0.506463695	0.112597671	same
chr2	74148476	74148876	-0.467220576	0.112699811	same
chr7	151350162	151350562	-0.484230117	0.112707625	same
chr3	28575192	28575592	-0.530927748	0.11276397	same
chrX	151397358	151397758	-0.53100111	0.112878424	same
chr3	123961129	123961529	-0.489500141	0.112901706	same
chr1	27012908	27013308	-0.476932713	0.112903005	same
chr17	40035321	40035721	-0.521691384	0.112973354	same
chr21	17819380	17819780	-0.498072016	0.112983855	same
chr22	19171291	19171691	-0.460227548	0.112986821	same
chr20	44310791	44311191	-0.532086014	0.11306851	same
chr3	12832809	12833209	-0.498249318	0.113073778	same
chr12	6764092	6764492	-0.476654027	0.113192984	same
chr12	124734467	124734867	-0.49252001	0.113261451	same
chr3	184363312	184363712	-0.48268624	0.11335821	same
chr1	149390217	149390617	-0.540152021	0.113562329	same
chr1	224481879	224482279	-0.509025813	0.113581134	same
chrX	72068317	72068717	-0.539774092	0.113666861	same
chr4	171813707	171814107	-0.550953624	0.113752347	same
chr11	46593642	46594042	-0.515471755	0.113766561	same
chr7	101217766	101218166	-0.488914329	0.11380599	same
chr18	23505413	23505813	-0.4719205	0.113820703	same
chrX	49101178	49101578	-0.461329614	0.11388219	same
chr11	73375945	73376345	-0.501253667	0.113918775	same
chr12	52032642	52033042	-0.519628687	0.113948519	same
chr4	147767889	147768289	-0.550468222	0.113994517	same
chr5	134632453	134632853	-0.494102521	0.114121281	same
chr6	42984025	42984425	-0.462198529	0.114165924	same
chr8	48514194	48514594	-0.480512916	0.114182404	same
chr19	37779413	37779813	-0.53659873	0.114215695	same
chr6	57089936	57090336	-0.487803813	0.114236221	same
chr10	16817351	16817751	-0.408672831	0.114387724	same
chr3	17742761	17743161	-0.48959011	0.114437661	same
chr8	79783606	79784006	-0.494574757	0.114443132	same
chr3	195907893	195908293	-0.513725353	0.114468741	same
chr10	27241610	27242010	-0.51263173	0.11449423	same
chr1	84690537	84690937	-0.52586466	0.114499424	same
chr1	202143522	202143922	-0.510678222	0.114502266	same
chr9	94165850	94166250	-0.495080358	0.114511113	same
chr6	70413252	70413652	-0.495984664	0.114672313	same
chr16	66552225	66552625	-0.499167146	0.114690954	same
chr11	124644419	124644819	-0.496007357	0.114736314	same
chr22	19432301	19432701	-0.455195583	0.11484234	same
chr6	26055635	26056035	-0.468819904	0.114856973	same
chr18	24271242	24271642	-0.5273884	0.114921685	same
chr2	199452625	199453025	-0.48977889	0.114922953	same
chr1	32222324	32222724	-0.493158401	0.114986635	same
chr19	57302407	57302807	-0.525432923	0.115024767	same
chr18	35950094	35950494	-0.513054611	0.115042429	same
chr1	235972011	235972411	-0.472964594	0.115047219	same
chr22	36409866	36410266	-0.481747469	0.115048042	same
chr1	210236743	210237143	-0.480192679	0.115078385	same
chr5	151157665	151158065	-0.470528672	0.115100479	same
chr8	42843087	42843487	-0.476060882	0.115119253	same
chr1	26788168	26788568	-0.530926137	0.115315565	same
chr8	144078467	144078867	-0.446306461	0.115404371	same
chr17	752285	752685	-0.555857445	0.115411607	same

chr19	15452180	15452580	-0.491284521	0.115419851	same
chr2	196799359	196799759	-0.509360207	0.115421614	same
chr6	166998773	166999173	-0.524205099	0.115429127	same
chr5	95846865	95847265	-0.547058526	0.115455653	same
chr4	110623024	110623424	-0.461582073	0.115499173	same
chr9	128340150	128340550	-0.530794999	0.115509696	same
chr3	194350344	194350744	-0.535512379	0.115647435	same
chr1	211630295	211630695	-0.477399105	0.115655396	same
chr6	142232329	142232729	-0.499165871	0.115704904	same
chr8	81089306	81089706	-0.524326206	0.115742061	same
chr2	186570532	186570932	-0.526875105	0.115772213	same
chr9	100352899	100353299	-0.528706552	0.115775873	same
chr4	105146875	105147275	-0.54378501	0.115785762	same
chr3	160885874	160886274	-0.499441335	0.11579537	same
chr3	12664187	12664587	-0.518082119	0.115825237	same
chr11	68030475	68030875	-0.442115269	0.115903653	same
chr20	45812325	45812725	-0.466528033	0.116018049	same
chr9	114612306	114612706	-0.515828183	0.116064507	same
chr17	82236209	82236609	-0.447257225	0.116073138	same
chr11	68460190	68460590	-0.415496216	0.11619189	same
chr15	90994596	90994996	-0.435669257	0.116423737	same
chr19	33080865	33081265	-0.515956373	0.116444223	same
chr21	32300223	32300623	-0.40289348	0.116452029	same
chr3	9933647	9934047	-0.463314858	0.11646728	same
chr8	144522051	144522451	-0.456199079	0.116482777	same
chr20	44476125	44476525	-0.544014072	0.11652506	same
chrX	115940859	115941259	-0.463656216	0.116532157	same
chr6	43021018	43021418	-0.510610686	0.116550499	same
chr7	901293	901693	-0.478206333	0.116641639	same
chr12	71503954	71504354	-0.541272112	0.116705092	same
chr11	113974927	113975327	-0.481082971	0.116706481	same
chr11	86955700	86956100	-0.52670535	0.11675098	same
chr6	117265520	117265920	-0.536483864	0.116938776	same
chr10	22229190	22229590	-0.54390702	0.11693938	same
chr7	149090397	149090797	-0.476532008	0.11697395	same
chr3	96813889	96814289	-0.516152006	0.116975174	same
chr6	42453591	42453991	-0.5480459	0.117034699	same
chr10	95693668	95694068	-0.454243324	0.117073383	same
chr1	204807070	204807470	-0.553040946	0.117078945	same
chr9	77018587	77018987	-0.540587336	0.117162138	same
chr7	43869231	43869631	-0.51305481	0.117183737	same
chr1	93845906	93846306	-0.496381105	0.117228946	same
chr4	6917485	6917885	0.527969751	0.117296843	same
chr7	148803221	148803621	-0.497107078	0.11731825	same
chr3	38346434	38346834	-0.523100385	0.117445209	same
chr21	38989790	38990190	-0.504437865	0.117647126	same
chr16	48244140	48244540	-0.542615908	0.117656949	same
chr9	92877831	92878231	-0.464724743	0.117689945	same
chr13	27022542	27022942	-0.529178076	0.117738646	same
chr12	76559886	76560286	-0.516516481	0.117749781	same
chr20	3795919	3796319	-0.483176245	0.1177714	same
chr12	106084018	106084418	-0.520611707	0.117852227	same
chr11	15370965	15371365	-0.539109604	0.117957437	same
chr3	142149452	142149852	-0.50453317	0.117983694	same
chr6	126339849	126340249	-0.487632295	0.118025146	same
chr17	7329534	7329934	-0.377796791	0.118102782	same
chr11	61776545	61776945	-0.493778563	0.118123403	same
chr1	155140198	155140598	-0.494720398	0.118141417	same
chr4	138241888	138242288	-0.530086921	0.118158513	same
chr1	222643976	222644376	-0.434069026	0.118181744	same
chr9	135500272	135500672	-0.508160426	0.118226405	same
chr4	153467560	153467960	-0.521870169	0.118241956	same
chr11	62680845	62681245	-0.475478871	0.118244669	same
chr3	146544401	146544801	-0.513040052	0.118247021	same
chr10	44857656	44858056	-0.532281994	0.118260948	same
chr4	88378940	88379340	-0.528574002	0.118317317	same
chr19	12836191	12836591	-0.481023284	0.118452356	same
chr19	53431778	53432178	-0.539430377	0.118491938	same
chr1	99685669	99686069	-0.522079457	0.118542681	same
chr17	74296449	74296849	-0.538233346	0.118565729	same
chr15	89647202	89647602	-0.432239149	0.118579774	same
chr2	120923735	120924135	-0.532368389	0.118587092	same
chr3	128879431	128879831	-0.434405536	0.118608581	same
chr1	119714578	119714978	-0.499138111	0.118614561	same
chr2	46500089	46500489	-0.508353358	0.118615595	same
chr10	69392328	69392728	-0.530170337	0.118650582	same

chr17	81200872	81201272	-0.46926466	0.118685768	same
chr12	131829191	131829591	-0.523373876	0.118760406	same
chr19	52897483	52897883	-0.506945315	0.118834323	same
chr11	102317221	102317621	-0.528605443	0.118883938	same
chr1	147890136	147890536	-0.516890153	0.118937565	same
chr9	133030476	133030876	-0.458267589	0.118939763	same
chrX	152958818	152959218	-0.506609854	0.118972116	same
chr6	26596086	26596486	-0.537127963	0.119002959	same
chr19	48900086	48900486	-0.464191052	0.119003248	same
chr12	111841572	111841972	-0.491767372	0.119049361	same
chr8	65840856	65841256	-0.514827639	0.119242605	same
chr21	26169150	26169550	-0.489013782	0.119307264	same
chrX	84729779	84730179	-0.529619942	0.11941956	same
chr12	101698762	101699162	-0.537653344	0.119443102	same
chr16	87777705	87778105	-0.475693526	0.119512918	same
chr11	47407809	47408209	-0.514266305	0.119575874	same
chr19	53942174	53942574	-0.526916293	0.119661278	same
chr1	247104114	247104514	-0.506924032	0.119669033	same
chr6	45378022	45378422	-0.458800695	0.119883569	same
chr7	151897239	151897639	-0.484554143	0.119945724	same
chr1	223738644	223739044	-0.493876144	0.119968629	same
chr10	55630205	55630605	-0.533230498	0.1200112	same
chr13	32585863	32586263	-0.537579986	0.120031698	same
chr3	196942055	196942455	-0.512891669	0.120073471	same
chr1	21176858	21177258	-0.511312807	0.120114503	same
chr7	96075702	96076102	-0.528984225	0.120211069	same
chr1	228010464	228010864	-0.456640213	0.120250217	same
chr2	170930143	170930543	-0.50826819	0.120289357	same
chr8	70668851	70669251	-0.468144027	0.120349048	same
chr21	41678821	41679221	-0.523526307	0.120350825	same
chr2	156435901	156436301	-0.522349874	0.120385156	same
chr1	9428725	9429125	-0.504176604	0.120424281	same
chr6	24583547	24583947	-0.540679139	0.120509889	same
chr9	79865151	79865551	-0.510145313	0.120646295	same
chr17	78289803	78290203	-0.506429054	0.120661164	same
chrX	150568226	150568626	-0.504471315	0.120663831	same
chr2	54115577	54115977	-0.515843356	0.120704219	same
chr6	17393221	17393621	-0.531586488	0.120738552	same
chr9	114154413	114154813	-0.510331507	0.120740196	same
chr7	114653342	114653742	-0.522544975	0.120879323	same
chr2	60642372	60642772	-0.53323979	0.120884359	same
chr1	162024176	162024576	-0.510389309	0.120998812	same
chr11	11849150	11849550	0.523131373	0.121009189	same
chr16	3611077	3611477	-0.527917025	0.121030353	same
chr19	53879498	53879898	-0.505147312	0.121065356	same
chr5	96661836	96662236	-0.492173919	0.12116313	same
chr2	69893917	69894317	-0.534037537	0.121303688	same
chr16	84754252	84754652	-0.521974665	0.121342081	same
chr16	28321598	28321998	-0.496865797	0.121361108	same
chr5	37379039	37379439	-0.508679415	0.121403115	same
chr12	2960283	2960683	-0.509481419	0.121412394	same
chr1	19596517	19596917	-0.5351487	0.12144398	same
chr12	53159390	53159790	-0.48757268	0.121463927	same
chr22	50544747	50545147	-0.491323067	0.121574203	same
chr3	48918463	48918863	-0.451548074	0.121611807	same
chr12	53451775	53452175	-0.506519196	0.121617677	same
chr5	132526628	132527028	-0.507406174	0.121705984	same
chr15	89049337	89049737	-0.519585346	0.121719168	same
chr14	61762175	61762575	-0.44980512	0.121873878	same
chr1	181104611	181105011	-0.47840425	0.122033791	same
chr1	205775134	205775534	-0.48687597	0.122085678	same
chr19	13093596	13093996	-0.530147514	0.122103619	same
chr13	65864372	65864772	0.513272166	0.122162532	same
chr4	127782201	127782601	-0.476894019	0.122198423	same
chr10	5623046	5623446	-0.518778557	0.122308016	same
chr8	60619776	60620176	-0.531660046	0.122308164	same
chr4	139556279	139556679	-0.494844996	0.122411733	same
chr12	128823979	128824379	-0.500154725	0.12242759	same
chr16	89289387	89289787	-0.425145067	0.122552146	same
chr22	38201446	38201846	-0.484928958	0.122593954	same
chr3	49340144	49340544	-0.446971802	0.12261047	same
chr11	33774638	33775038	-0.520650808	0.12264631	same
chr9	127816892	127817292	-0.472284617	0.122716759	same
chr1	112369777	112370177	-0.50359962	0.122720683	same
chr22	40370350	40370750	-0.517558207	0.122786503	same
chr18	34224509	34224909	-0.461723129	0.12282187	same

chr2	27771665	27772065	-0.468796995	0.12289285	same
chr17	7403950	7404350	-0.424763163	0.122911042	same
chr6	141815038	141815438	-0.512203544	0.122952237	same
chr16	2150033	2150433	-0.481616503	0.123035504	same
chr20	407599	407999	-0.474532691	0.123052426	same
chr6	108560356	108560756	-0.499512214	0.123063237	same
chr1	151613019	151613419	-0.485667051	0.12307269	same
chrX	151082190	151082590	-0.494631507	0.123184496	same
chr1	40708934	40709334	-0.49912226	0.123201374	same
chr6	129501289	129501689	-0.49244377	0.123217778	same
chr4	129093278	129093678	-0.436741331	0.123229559	same
chr1	212950250	212950650	-0.44922891	0.123292664	same
chr11	67428049	67428449	-0.491383017	0.123317839	same
chr19	49347662	49348062	-0.500484571	0.123335854	same
chr5	125275157	125275557	-0.511788107	0.123374435	same
chr19	43207193	43207593	-0.405234528	0.123476494	same
chr10	18651559	18651959	-0.537101624	0.123512471	same
chr4	185396367	185396767	-0.502530394	0.123575441	same
chr14	24232542	24232942	-0.479296607	0.123605206	same
chr2	131093508	131093908	-0.516472971	0.123806321	same
chr18	24426466	24426866	-0.506506695	0.123926131	same
chr7	130070055	130070455	-0.477421106	0.12416961	same
chr1	231407905	231408305	-0.500771318	0.124193773	same
chr11	74398588	74398988	-0.487175315	0.124236795	same
chr12	49109144	49109544	-0.478172195	0.124254433	same
chr8	17922810	17923210	-0.489637268	0.124283286	same
chr10	118046934	118047334	-0.495150956	0.124293268	same
chr9	79648938	79649338	-0.483335233	0.12430882	same
chr2	127220663	127221063	-0.526388543	0.124369446	same
chr11	102346765	102347165	-0.474302462	0.124424416	same
chr9	4605928	4606328	-0.530411592	0.124507261	same
chr17	71398970	71399370	-0.530403738	0.12450857	same
chr2	231052860	231053260	-0.434855785	0.124776953	same
chr22	21045593	21045993	-0.485360782	0.124983544	same
chr21	37338921	37339321	-0.47149965	0.12504667	same
chr1	94237641	94238041	-0.526977506	0.125078099	same
chr15	84631259	84631659	-0.470883466	0.125107702	same
chr1	55277388	55277788	-0.477298449	0.125111949	same
chr1	32464781	32465181	-0.439344181	0.125190838	same
chr6	28489907	28490307	-0.53085717	0.125238633	same
chr9	137297810	137298210	-0.528150096	0.125285865	same
chr1	227999235	227999635	-0.464143329	0.125297823	same
chr17	51153406	51153806	-0.471901538	0.125383299	same
chr22	46335341	46335741	-0.493702206	0.125520289	same
chr11	112086472	112086872	-0.471928892	0.125537047	same
chr10	21518419	21518819	-0.52665749	0.125563832	same
chr16	66608355	66608755	-0.486198716	0.125689759	same
chr11	124673419	124673819	-0.505457222	0.1258145	same
chr14	50944705	50945105	-0.503905739	0.12581766	same
chr3	138915767	138916167	-0.489507382	0.125880888	same
chr22	44732604	44733004	0.49577691	0.12591325	same
chr8	100559435	100559835	-0.485514542	0.12616946	same
chr11	62788955	62789355	-0.433688267	0.126183157	same
chr17	44198468	44198868	-0.498210374	0.126330489	same
chr13	85363518	85363918	-0.485530526	0.126336691	same
chr17	57084696	57085096	-0.479084047	0.126339785	same
chr1	161132847	161133247	-0.445573448	0.126389926	same
chr6	12816249	12816649	-0.508128377	0.126422324	same
chr12	49336922	49337322	-0.485208807	0.126466539	same
chr19	6587093	6587493	-0.494150473	0.126495141	same
chr1	151008343	151008743	-0.467398675	0.126497698	same
chr12	6778936	6779336	-0.443071077	0.126699567	same
chr1	174159574	174159974	-0.455243311	0.12673096	same
chr11	65040769	65041169	-0.454377559	0.12673509	same
chr9	37753717	37754117	-0.516543099	0.126802132	same
chr5	43064645	43065045	-0.492923797	0.126851929	same
chr1	179814842	179815242	-0.463953031	0.126866281	same
chr19	48983566	48983966	-0.491771984	0.126920884	same
chr17	76014068	76014468	-0.47583981	0.126922555	same
chr10	75405164	75405564	-0.456058447	0.126989839	same
chr3	101827612	101828012	-0.521521669	0.126996099	same
chr18	39841384	39841784	-0.50637955	0.127031784	same
chr2	65359500	65359900	-0.512732117	0.127032593	same
chrX	119565218	119565618	-0.497753906	0.127045964	same
chr16	3500906	3501306	-0.49483391	0.127219081	same
chr8	122137227	122137627	-0.50852392	0.127256992	same

chr4	52722202	52722602	-0.426515034	0.127265381	same
chr19	43935007	43935407	-0.478456244	0.127336928	same
chr16	25015352	25015752	-0.497394061	0.127344213	same
chr2	11829933	11830333	-0.526917222	0.127357232	same
chr7	149126006	149126406	-0.430257421	0.127474476	same
chr19	49208263	49208663	-0.519381525	0.12756876	same
chr6	44224065	44224465	-0.491617251	0.127663023	same
chr8	38105437	38105837	-0.48702363	0.127789601	same
chr4	102737106	102737506	-0.51426719	0.127865978	same
chr4	84126957	84127357	-0.517821786	0.127879507	same
chr13	85508491	85508891	0.496225988	0.127925785	same
chr12	56206527	56206927	-0.520093481	0.128005199	same
chr1	44029747	44030147	-0.512960803	0.128051021	same
chr1	1371939	1372339	-0.444076546	0.128093342	same
chr5	99020432	99020832	-0.513185712	0.128172104	same
chr17	4909001	4909401	-0.45646712	0.128200257	same
chr2	55419530	55419930	-0.494564643	0.128233514	same
chr10	63132985	63133385	-0.495549253	0.128262449	same
chr7	102404418	102404818	-0.468170442	0.128277155	same
chr20	62921561	62921961	-0.475820654	0.128297675	same
chr19	39341755	39342155	-0.477699974	0.128305192	same
chr2	131104759	131105159	-0.493482814	0.128314026	same
chr16	67245970	67246370	-0.44837742	0.128406546	same
chr4	95548088	95548488	-0.450583411	0.128430902	same
chr6	47034627	47035027	-0.528835848	0.12843143	same
chr19	45469648	45470048	-0.400168788	0.12844441	same
chr11	34222903	34223303	-0.500559882	0.128479902	same
chr3	127575113	127575513	-0.469203085	0.128485795	same
chr5	87412732	87413132	-0.450188742	0.128490277	same
chrX	118116346	118116746	-0.529601952	0.128557367	same
chr2	97664127	97664527	-0.471544133	0.128571716	same
chrX	154412015	154412415	-0.4745978	0.128592548	same
chr12	130870851	130871251	-0.437498023	0.128643861	same
chr13	95301576	95301976	-0.516853736	0.128712016	same
chr6	3251619	3252019	-0.498742748	0.128716556	same
chr15	64825734	64826134	-0.498925528	0.128776343	same
chr5	159262968	159263368	-0.509113623	0.128824852	same
chr1	169367820	169368220	-0.425674235	0.128855353	same
chr6	27188626	27189026	-0.521401486	0.128897855	same
chr2	63841087	63841487	-0.432715387	0.129032438	same
chr13	96053148	96053548	-0.519989774	0.129150942	same
chr11	75803067	75803467	-0.509536655	0.129278964	same
chrX	119512561	119512961	-0.485799428	0.129350513	same
chr17	34981395	34981795	-0.487926765	0.129351872	same
chr2	32375444	32375844	-0.531098297	0.129426103	same
chr9	134641191	134641591	-0.520419883	0.12947577	same
chr16	89357050	89357450	-0.4649646	0.129556721	same
chr5	1174250	1174650	-0.509522851	0.129595643	same
chr19	34428031	34428431	-0.505483382	0.129655363	same
chr1	44631498	44631898	-0.478204667	0.129658339	same
chr1	36464362	36464762	-0.460751927	0.129661209	same
chr3	184712059	184712459	-0.512767659	0.129666505	same
chr4	139656794	139657194	-0.504065951	0.129703989	same
chr17	28812466	28812866	-0.484643658	0.129799034	same
chr19	19490200	19490600	-0.461872422	0.129911643	same
chr5	140639357	140639757	-0.473390544	0.129956357	same
chr9	114405672	114406072	-0.508590085	0.129969175	same
chr9	127898659	127899059	-0.478701968	0.130033096	same
chr22	20298825	20299225	-0.490022383	0.130107547	same
chr19	38618749	38619149	-0.477589764	0.130143397	same
chr11	71972612	71973012	-0.516616516	0.130169128	same
chr2	207770033	207770433	-0.5071277	0.130179524	same
chr1	225916243	225916643	-0.484898406	0.130199008	same
chr5	97193106	97193506	-0.514400965	0.130221496	same
chr1	32014559	32014959	-0.473096972	0.130240213	same
chr7	64665970	64666370	-0.53375576	0.130251847	same
chr7	4641481	4641881	-0.532867181	0.130263612	same
chr4	1940054	1940454	-0.493478847	0.130280993	same
chr12	13000376	13000776	-0.506270544	0.130312617	same
chr15	65611210	65611610	-0.413051145	0.130422254	same
chr16	70381249	70381649	-0.408032431	0.130447291	same
chr1	147599205	147599605	-0.425644327	0.130458743	same
chr19	17873822	17874222	-0.503314254	0.13046005	same
chr5	59317221	59317621	-0.489768465	0.130483075	same
chr3	133413441	133413841	-0.504516002	0.130520362	same
chr2	240232933	240233333	-0.506164061	0.130598878	same

chr11	65637754	65638154	-0.477676865	0.130619458	same
chr13	62847514	62847914	-0.425359464	0.130636839	same
chr3	113746786	113747186	-0.429954955	0.130638503	same
chrX	55160919	55161319	-0.468159276	0.130710904	same
chr3	15064136	15064536	-0.497532868	0.13074951	same
chr12	2853494	2853894	-0.513586742	0.130823235	same
chr1	230068014	230068414	-0.501408066	0.130938256	same
chr21	32506913	32507313	-0.516986876	0.131020204	same
chr10	72204447	72204847	-0.516822688	0.131033328	same
chr7	74206712	74207112	-0.481658129	0.131049378	same
chr19	43084234	43084634	-0.439958615	0.131098792	same
chrX	149909817	149910217	-0.515515749	0.131207992	same
chr7	123534633	123535033	-0.460737855	0.131278522	same
chr5	175378417	175378817	-0.488682046	0.131284378	same
chr1	26169879	26170279	-0.490480109	0.131336576	same
chr17	76557566	76557966	-0.410191152	0.131468013	same
chr1	149886613	149887013	-0.406676893	0.131496328	same
chr1	28296883	28297283	-0.4973401	0.131501094	same
chr4	185573818	185574218	-0.528094994	0.131589735	same
chr6	63573927	63574327	-0.482218092	0.13171074	same
chr17	42459051	42459451	-0.498833013	0.131783285	same
chr1	114716578	114716978	-0.450370841	0.131829544	same
chr19	50476447	50476847	-0.430631027	0.131987523	same
chr2	208025369	208025769	-0.495897072	0.132002823	same
chr5	59768423	59768823	-0.49679514	0.13203592	same
chr16	30534293	30534693	-0.474773765	0.132053976	same
chr3	81761205	81761605	-0.438643078	0.132142304	same
chr13	25301316	25301716	-0.499485374	0.132208337	same
chr17	43907550	43907950	-0.454699439	0.132220822	same
chr13	67840943	67841343	-0.471576976	0.132222193	same
chr16	87549581	87549981	-0.517248462	0.132229273	same
chr17	80649897	80650297	-0.480406179	0.132253078	same
chr7	27200633	27201033	-0.481256068	0.132336957	same
chr21	33801703	33802103	-0.495263361	0.132346719	same
chr14	103500020	103500420	-0.498053821	0.132466196	same
chrX	103585682	103586082	-0.509747109	0.132552659	same
chr9	136226102	136226502	-0.484755651	0.132592246	same
chrX	139931216	139931616	-0.486863017	0.132645534	same
chr2	201451228	201451628	-0.511011935	0.13265177	same
chr10	69515736	69516136	-0.515659839	0.132671721	same
chr10	112140287	112140687	-0.504953646	0.132695147	same
chrX	47541067	47541467	-0.525513521	0.132732182	same
chr12	111369129	111369529	-0.509588724	0.132753827	same
chr19	8268851	8269251	-0.473654075	0.132850316	same
chr2	74390943	74391343	-0.495939392	0.132872254	same
chr4	1899287	1899687	-0.462079929	0.13306703	same
chr13	71879319	71879719	-0.529138838	0.133134729	same
chr19	50414532	50414932	-0.457558256	0.133169128	same
chr6	163745912	163746312	-0.493262684	0.133240444	same
chr2	131682328	131682728	-0.429099342	0.133249939	same
chr1	165698119	165698519	-0.457329831	0.133289287	same
chr11	7665701	7666101	-0.504869355	0.133298384	same
chr6	26553487	26553887	-0.458798033	0.133298909	same
chr13	63486276	63486676	0.489115257	0.13331509	same
chr17	7687889	7688289	-0.385873021	0.133427474	same
chr20	25196313	25196713	-0.465141416	0.133452931	same
chr1	62391479	62391879	-0.495887077	0.133491123	same
chr7	135977287	135977687	-0.40116757	0.133691498	same
chr12	121888588	121888988	-0.407255485	0.133703251	same
chr22	20269232	20269632	-0.49885498	0.133846681	same
chr14	77181451	77181851	-0.480095419	0.133894817	same
chr2	61176882	61177282	-0.493888309	0.134012144	same
chr22	28663093	28663493	-0.501154827	0.13404496	same
chr3	53489645	53490045	-0.499079297	0.134116267	same
chr2	17540231	17540631	-0.480333354	0.134124096	same
chr20	23421474	23421874	-0.523273011	0.134174709	same
chr1	161225501	161225901	-0.404151111	0.134181358	same
chr1	25430240	25430640	-0.467833738	0.134210898	same
chr9	34675063	34675463	-0.479606415	0.134224287	same
chr19	23273864	23274264	-0.498284037	0.134298665	same
chr11	65169885	65170285	-0.449840792	0.134307216	same
chr3	15051647	15052047	-0.484338359	0.134397166	same
chr6	143450749	143451149	-0.439225591	0.134402685	same
chr2	38432422	38432822	-0.512792046	0.134446378	same
chr15	89760811	89761211	-0.488493263	0.134466069	same
chr1	247007790	247008190	-0.478453837	0.134475723	same

chr7	43838630	43839030	-0.517314308	0.134490349	same
chr16	75647545	75647945	-0.471271397	0.134500313	same
chr12	56152220	56152620	-0.474615543	0.134569912	same
chr2	238287732	238288132	-0.469058188	0.134607748	same
chrX	135521806	135522206	-0.521787633	0.134641925	same
chr4	121696753	121697153	-0.509729328	0.134668577	same
chr12	6891260	6891660	-0.491780615	0.13471417	same
chrX	52935033	52935433	-0.43392611	0.134788913	same
chr14	89939490	89939890	-0.517158343	0.134879618	same
chr15	67968149	67968549	-0.510081151	0.134899564	same
chr7	85186243	85186643	-0.504070941	0.135066451	same
chr5	16934141	16934541	-0.500085029	0.135078568	same
chr6	85643819	85644219	-0.474347269	0.135122853	same
chr6	28267009	28267409	-0.504065113	0.13528179	same
chr1	212665360	212665760	-0.427635052	0.135283985	same
chr7	141014747	141015147	-0.385264437	0.135377092	same
chr19	50368776	50369176	-0.47681965	0.135380955	same
chrX	134914799	134915199	-0.46707169	0.135408797	same
chr13	62067977	62068377	-0.47340774	0.135582826	same
chr16	69339590	69339990	-0.470431766	0.135670484	same
chr1	160098948	160099348	-0.486075358	0.135822049	same
chr3	42071813	42072213	-0.51415566	0.136027209	same
chr7	91264133	91264533	-0.44286247	0.136042631	same
chr3	4493045	4493445	-0.402143808	0.13608236	same
chr8	102412697	102413097	-0.478899749	0.136150053	same
chr2	178072627	178073027	-0.484468409	0.136159134	same
chr1	119327580	119327980	-0.464579293	0.136771853	same
chr17	46940694	46941094	-0.489592206	0.136880525	same
chr13	72851863	72852263	-0.508047113	0.136897755	same
chr11	64181623	64182023	-0.430231067	0.136975713	same
chr13	26253750	26254150	-0.509579147	0.136987809	same
chr11	130095923	130096323	-0.502078471	0.137009721	same
chr12	132685302	132685702	-0.442398926	0.137054504	same
chrX	48676451	48676851	-0.466036334	0.13729002	same
chr1	3900726	3901126	-0.433690506	0.137329861	same
chr6	108079963	108080661	-0.499062984	0.137365808	same
chr12	76348225	76348625	-0.486868052	0.137385531	same
chr5	56123643	56124043	-0.515308038	0.137405782	same
chr10	102117576	102117976	-0.46126185	0.137422744	same
chr3	132722805	132723205	-0.516837098	0.137435581	same
chr3	19946905	19947305	-0.423361968	0.137488869	same
chr3	52239269	52239669	-0.450634312	0.137502942	same
chr1	202888756	202889156	-0.425557497	0.137503841	same
chr1	59831257	59831657	-0.509161573	0.137541623	same
chr1	209747701	209748101	-0.38507787	0.137637272	same
chr5	107669245	107669645	-0.465708084	0.137647952	same
chr16	56318257	56318657	-0.489141084	0.137656983	same
chr19	1045112	1045512	-0.457981414	0.137690758	same
chr12	50400785	50401185	-0.46225035	0.137717572	same
chr14	105077134	105077534	-0.499943972	0.137823354	same
chr14	60734702	60735102	-0.491486501	0.137829486	same
chr6	101393063	101393463	-0.506132594	0.137846538	same
chr7	6484131	6484531	-0.505912616	0.137883722	same
chr5	141968939	141969339	-0.435788238	0.137941881	same
chr3	128153048	128153448	-0.515496077	0.138129014	same
chr16	23510564	23510964	-0.49559714	0.138161895	same
chr12	121352160	121352560	-0.385650451	0.138172855	same
chr9	86127234	86127634	-0.456042896	0.13826201	same
chr10	38010765	38011165	-0.477331919	0.138397534	same
chr6	27792112	27792512	-0.444632241	0.138423323	same
chr5	139765611	139766011	-0.437084528	0.138565335	same
chr8	73878549	73878949	-0.463066161	0.138610079	same
chr1	16440412	16440812	-0.484429192	0.138637982	same
chr19	12938237	12938637	-0.384074007	0.138650841	same
chr9	112784392	112784792	-0.479909061	0.138661672	same
chr1	226626307	226626707	-0.418236119	0.138676278	same
chr14	101965507	101965907	-0.490659534	0.138680981	same
chr12	67647388	67647788	-0.495416287	0.138695431	same
chr10	28939414	28939814	-0.502405965	0.13874291	same
chr10	94402013	94402413	-0.494682324	0.138838547	same
chr16	1408129	1408529	-0.448341064	0.138862609	same
chr6	43174342	43174742	-0.422043529	0.138862964	same
chr1	180154397	180154797	-0.512420496	0.138918943	same
chr1	34852826	34853226	-0.502120728	0.138926908	same
chr15	68819171	68819571	-0.450094255	0.138931361	same
chr2	9737043	9737443	-0.519093924	0.139019569	same

chr1	16896488	16896888	-0.464552174	0.139032463	same
chr17	61863261	61863661	-0.419026947	0.139065615	same
chr17	58157036	58157436	-0.506667717	0.139179745	same
chr1	116372813	116373213	-0.477067297	0.139263771	same
chr12	64758983	64759383	-0.462118514	0.139304897	same
chr1	9539008	9539408	-0.485391864	0.139380796	same
chr17	50707257	50707657	-0.509625206	0.139394735	same
chr3	79017620	79018020	-0.490683833	0.139443809	same
chr2	127388153	127388553	-0.489851136	0.139537835	same
chr3	16665264	16665664	-0.511762755	0.139545262	same
chr22	19218270	19218670	-0.506842517	0.139613163	same
chr20	18567450	18567850	-0.497024173	0.139619719	same
chr6	41318717	41319117	-0.51466323	0.139633877	same
chr6	44246846	44247246	-0.397603776	0.139692941	same
chr6	26537480	26537880	-0.389130519	0.139700085	same
chr1	61049712	61050112	-0.496158122	0.139704994	same
chr12	31591462	31591862	-0.513963169	0.139732941	same
chr16	2155336	2155736	-0.424346535	0.139745405	same
chr12	51424718	51425118	-0.501565974	0.139762103	same
chr5	52800013	52800413	-0.436742563	0.13979658	same
chr5	81270473	81270873	-0.510602546	0.139854668	same
chr5	96716774	96717174	-0.501628122	0.139888811	same
chr17	44138135	44138535	-0.433488943	0.139934576	same
chr7	90154204	90154604	-0.509183798	0.139967512	same
chrX	53441750	53442150	-0.48394218	0.140171297	same
chr16	2223252	2223652	-0.430148812	0.140238325	same
chr16	28950470	28950870	-0.456470093	0.140291425	same
chr1	201408208	201408608	-0.467700671	0.140345013	same
chr12	10722034	10722434	-0.485024792	0.140395593	same
chrUn_KI270466v1	638	1038	0.278616567	0.140528133	same
chr18	22683321	22683721	-0.494448979	0.140531935	same
chr17	29148615	29149015	-0.490465866	0.140579388	same
chr21	44845805	44846205	-0.434204605	0.140599434	same
chr6	127267311	127267711	-0.475085191	0.140688523	same
chr2	78417323	78417723	-0.500009546	0.140692954	same
chr12	62602414	62602814	-0.466232287	0.140732591	same
chr19	51594268	51594668	-0.480252619	0.140824517	same
chr15	40405558	40405958	-0.482960504	0.140825579	same
chr16	235288	235688	-0.492096555	0.140834667	same
chr16	30894290	30894690	-0.426560485	0.140894854	same
chr3	20186040	20186440	-0.449466914	0.140914946	same
chr6	44133509	44133909	-0.455888121	0.140929598	same
chr13	76243179	76243579	-0.473459524	0.141076404	same
chr1	203487536	203487936	-0.450408079	0.141137756	same
chr17	66508833	66509233	-0.488676431	0.141308947	same
chr1	38786297	38786697	-0.472610814	0.141334118	same
chr19	13163453	13163853	-0.488397667	0.141446808	same
chr1	31745495	31745895	-0.463833892	0.141526054	same
chr1	26321378	26321778	-0.501254181	0.141553485	same
chr17	67293613	67294013	-0.511104412	0.141579798	same
chr6	127343258	127343658	-0.475104047	0.141581084	same
chr9	112486433	112486833	-0.490743745	0.141596484	same
chr12	45215700	45216100	-0.493534361	0.141623192	same
chr1	239912380	239912780	-0.502352613	0.141668129	same
chr4	113760687	113761087	-0.469665731	0.141689552	same
chr10	7818931	7819331	-0.488328931	0.141731332	same
chr19	4267191	4267591	-0.456822924	0.141876574	same
chr5	122585713	122586113	-0.497960649	0.141884847	same
chr1	152047965	152048365	-0.41257401	0.141972039	same
chr6	90586933	90587333	-0.43820092	0.142038704	same
chr6	169638879	169639279	-0.476406967	0.142102151	same
chr1	95167807	95168207	-0.50251825	0.142203289	same
chr5	177403800	177404200	-0.474609133	0.142247765	same
chr12	79045151	79045551	-0.492519913	0.14236697	same
chr20	17939148	17939548	-0.427862716	0.142378769	same
chr8	56211717	56212117	-0.478377322	0.142438141	same
chr22	26512303	26512703	-0.458273338	0.142547935	same
chr6	105176247	105176647	-0.495905349	0.14254995	same
chr1	228083843	228084243	-0.410976986	0.14256591	same
chr3	197960180	197960580	-0.431069148	0.142591252	same
chr10	43348451	43348851	-0.470327968	0.142707723	same
chr8	93740828	93741228	-0.450845763	0.142758439	same
chr11	96243335	96243735	-0.441403129	0.142778525	same
chr16	31094457	31094857	-0.439003058	0.142789358	same
chr4	3047548	3047948	-0.499160695	0.142847009	same
chr1	203327708	203328108	-0.457052438	0.142933834	same

chr19	52139281	52139681	-0.461961898	0.14293511	same
chr9	113056678	113057078	-0.459300964	0.142939948	same
chr10	75210445	75210845	-0.441934413	0.142959709	same
chr20	49610804	49611204	-0.490119775	0.142968756	same
chr1	8525743	8526143	-0.468159087	0.143001594	same
chr5	188746	189146	-0.473643519	0.143003704	same
chr3	108222199	108222599	-0.443377933	0.143040329	same
chr12	57739174	57739574	-0.450782266	0.143303181	same
chr16	1983479	1983879	-0.412287557	0.143303826	same
chr5	32585641	32586041	-0.492405392	0.14331008	same
chr1	28247070	28247470	-0.387259868	0.143315051	same
chr6	166381991	166382391	-0.452670754	0.143366006	same
chr5	31854717	31855117	-0.498218444	0.143420262	same
chr8	23084085	23084485	-0.486282427	0.143440489	same
chr2	73680097	73680726	-0.489961236	0.143466389	same
chr22	46739026	46739426	-0.439134682	0.143575636	same
chr6	26065972	26066372	-0.498645356	0.143580202	same
chr2	120252356	120252756	-0.465451345	0.143632771	same
chr1	174021590	174021990	-0.47274656	0.143683399	same
chr4	4541932	4542332	-0.455411536	0.143699712	same
chr6	111552162	111552562	-0.419454786	0.143775306	same
chr1	39183760	39184160	-0.489787828	0.143799066	same
chr1	38859929	38860329	-0.487541957	0.143942982	same
chr9	79362211	79362611	-0.499080522	0.143956923	same
chr6	37423807	37424207	-0.460599117	0.144134844	same
chr17	35242589	35242989	-0.482605226	0.144243534	same
chr15	89654929	89655329	-0.44972368	0.144366087	same
chr12	21437726	21438126	-0.471074104	0.144367412	same
chr6	693100	693500	-0.466052292	0.144398936	same
chr1	43389453	43389853	-0.505676149	0.144401747	same
chrUn_GL000195v1	68495	68895	-0.500780056	0.144414225	same
chr4	55841863	55842263	-0.475962265	0.144497326	same
chr22	30634879	30635279	-0.458631076	0.144534807	same
chr11	123511109	123511509	-0.495019059	0.144548735	same
chr2	74178904	74179304	-0.425259744	0.144574011	same
chr11	57499746	57500146	-0.4541585	0.144586203	same
chr12	26126080	26126480	-0.491475083	0.144599855	same
chr1	234876328	234876728	-0.479439878	0.144660658	same
chr8	23019291	23019691	-0.480885594	0.144670257	same
chr6	112253863	112254263	-0.447480141	0.144774062	same
chr14	75069266	75069666	-0.509273752	0.144858842	same
chr1	99969591	99969991	-0.460564351	0.144862636	same
chr1	210311215	210311615	-0.468060382	0.144878457	same
chr19	1040756	1041156	-0.440644967	0.144886274	same
chr1	150876612	150877012	-0.412004583	0.144908891	same
chr7	135259621	135260021	-0.487627136	0.145048034	same
chr3	105366496	105366896	-0.394175699	0.145049337	same
chr19_KI270866v1_alt	41879	42279	-0.452254324	0.145090354	same
chr5	154610446	154610846	-0.495867139	0.14522668	same
chr10	98414914	98415314	-0.474056203	0.14522975	same
chr2	96305489	96305889	-0.436356743	0.145271864	same
chr22	19953487	19953887	-0.456930115	0.145310204	same
chr1	241640811	241641211	-0.455429801	0.145388565	same
chr10	15167608	15168008	-0.488308717	0.145475932	same
chr19	19515976	19516376	-0.415679716	0.145483913	same
chr15	68278599	68278999	-0.449659008	0.145664503	same
chr16	58661270	58661670	-0.500678414	0.145703172	same
chr20	63291083	63291483	-0.401575655	0.145766348	same
chr13	52194235	52194635	-0.461458958	0.145785818	same
chr2	55112181	55112581	-0.484180417	0.145925867	same
chr6	43247146	43247546	-0.465133828	0.145949361	same
chr8	100860371	100860771	-0.506734112	0.145974273	same
chr4	149915532	149915932	-0.473239465	0.145980101	same
chr1	109283049	109283449	-0.441117061	0.146046355	same
chr8	52714522	52714922	-0.440090476	0.146303483	same
chr15	73950714	73951114	-0.482909283	0.14638741	same
chr6	166997485	166997885	-0.481245596	0.146420813	same
chr16	30941895	30942295	-0.371385961	0.146665691	same
chr21	33127255	33127655	-0.463470048	0.146717398	same
chr6	108677085	108677485	-0.501333038	0.146812258	same
chr6	2764776	2765176	-0.48900251	0.14681556	same
chr10	5685326	5685726	-0.476824138	0.146848668	same
chr18	56638643	56639043	-0.496884693	0.146888489	same
chr7	140200303	140200703	-0.483690115	0.146946857	same
chr12	27703780	27704180	-0.482622032	0.146973647	same
chr6	10521063	10521463	-0.463207976	0.14700593	same

chr14	68548115	68548515	-0.441972949	0.14703312	same
chr12	569841	570241	-0.460487623	0.147043839	same
chr8	103501521	103501921	-0.498828721	0.147076013	same
chr9	137607016	137607416	-0.439480245	0.147100842	same
chr10	35126642	35127042	-0.507168508	0.147164973	same
chr17	28743471	28743871	-0.409250222	0.14751503	same
chr22	40177417	40177817	-0.491005372	0.147528844	same
chr16	2501238	2501638	-0.380624566	0.147583961	same
chr8	124425744	124426144	-0.482787822	0.14763537	same
chr17	82671132	82671532	0.48884442	0.147850774	same
chr6	116945880	116946280	-0.456943336	0.147918189	same
chr14	90396565	90396965	-0.497550196	0.147975556	same
chr15	76311246	76311646	-0.393119059	0.14799367	same
chr11	9759176	9759576	-0.477870148	0.148056705	same
chr5	179811118	179811518	-0.422808476	0.148155772	same
chrX	135973677	135974077	-0.431308461	0.148390676	same
chr11	124622612	124623012	-0.443242952	0.148425539	same
chr6	42217701	42218101	-0.47935688	0.14842602	same
chr8	108942445	108942845	-0.503192306	0.148489231	same
chr14	31207831	31208231	-0.487738411	0.148496499	same
chr4	4289890	4290290	-0.448316305	0.14851883	same
chr13	78660256	78660656	-0.40472754	0.148537264	same
chr4	169612347	169612747	-0.437528517	0.148540957	same
chr5	111072993	111073393	-0.455851562	0.148682844	same
chrX	129925481	129925881	-0.49515499	0.148714902	same
chr1	205679447	205679847	-0.471756469	0.148756047	same
chr1	228084556	228084956	-0.424361168	0.148776852	same
chr1	213051267	213051667	-0.432649609	0.149034188	same
chr3	44992694	44993094	-0.496616647	0.149077237	same
chr2	209476738	209477138	-0.505712152	0.149155644	same
chr8	94009084	94009484	-0.491380099	0.149156751	same
chr7	6374101	6374501	-0.478715141	0.14915872	same
chr1	150268528	150268928	-0.458506038	0.1491852	same
chr1	226134105	226134505	-0.407607868	0.149237627	same
chr4	17576762	17577162	-0.472751799	0.149286554	same
chr1	15526669	15527069	-0.42482714	0.149298826	same
chr17	70169437	70169837	-0.474737762	0.149351525	same
chr19	12801406	12801806	-0.43392386	0.149424641	same
chr7	76379863	76380263	-0.440983324	0.149427688	same
chr7	38335486	38336154	-0.47744006	0.149535035	same
chr1	33007280	33007680	-0.461490983	0.149551496	same
chr12	6662945	6663345	-0.413112648	0.14958557	same
chr2	104873557	104873957	-0.494570283	0.149644075	same
chr7	103380941	103381341	0.472298606	0.149737707	same
chr1	226064797	226065197	-0.397650134	0.149819186	same
chr17	28645153	28645553	-0.468298257	0.14983503	same
chr11	83071696	83072096	-0.408463982	0.150002659	same
chrX	53434316	53434716	-0.481417044	0.15007635	same
chrX	47482466	47482866	-0.456586617	0.150083489	same
chr16	4768593	4768993	-0.457192215	0.15009649	same
chr16	56519938	56520338	-0.444823605	0.150125101	same
chr12	123075619	123076019	-0.452648455	0.150290371	same
chr1	151611709	151612109	-0.487422082	0.150319095	same
chr9	37511133	37511533	-0.500180057	0.150359452	same
chr17	42980026	42980426	-0.478035915	0.150405072	same
chr14	72926146	72926546	-0.448214051	0.15045541	same
chr6	69866509	69866909	-0.458838182	0.150564988	same
chr18	76495428	76495828	-0.498383236	0.150659074	same
chr18	54357996	54358396	-0.495561426	0.150785571	same
chr22	19291808	19292208	-0.483094261	0.150829868	same
chr12	49828428	49828828	-0.431902832	0.150848996	same
chr15	70833991	70834391	-0.501121821	0.150936282	same
chr2	26503250	26503650	-0.476291468	0.151197544	same
chr4	128265482	128265882	-0.416138496	0.151264338	same
chr3	12984343	12984743	-0.475059979	0.151277552	same
chr1	84287308	84287708	-0.503553247	0.15132069	same
chr15	68559747	68560147	-0.459751483	0.151453538	same
chr16	2752105	2752505	-0.392593312	0.151475732	same
chr20	5113083	5113483	-0.415522272	0.151545306	same
chr20	34824491	34824891	-0.461017389	0.151582808	same
chr11	67302691	67303091	-0.483554222	0.151618879	same
chr4	94452499	94452899	-0.485665735	0.151622351	same
chr16	30935171	30935571	-0.419669664	0.151624671	same
chr14	53151632	53152032	-0.477404117	0.151644689	same
chr19	3762541	3762941	-0.387983592	0.151694809	same
chr5	173186996	173187396	-0.48625031	0.151869602	same

chr1	110053728	110054128	-0.466120644	0.151932598	same
chr14	55581038	55581438	-0.473402247	0.151937472	same
chrX	135021940	135022340	-0.420311696	0.151980911	same
chr2	206084163	206084563	-0.470710281	0.152001481	same
chr12	100266765	100267165	-0.406433917	0.152134402	same
chr8	22140966	22141366	-0.457993748	0.152135775	same
chr18	22466184	22466584	-0.496451508	0.152196226	same
chr4	3106420	3106820	-0.493575299	0.15222723	same
chr21	46458005	46458405	-0.455419434	0.152245168	same
chr17	35142110	35142510	-0.485885348	0.15230164	same
chr19	19063522	19063922	-0.393542119	0.152521172	same
chr7	4631606	4632006	-0.484985795	0.152587909	same
chr6	27146594	27146994	-0.374090288	0.152682407	same
chr9	21802236	21802636	-0.479491172	0.152698246	same
chr17	42527238	42527638	-0.472701733	0.152750248	same
chr4	8917632	8918032	-0.496933677	0.152762747	same
chr3	47781966	47782366	-0.434518508	0.152827526	same
chr11	74949023	74949423	-0.417829583	0.152835881	same
chr20	37415366	37415766	-0.483847879	0.152905052	same
chr10	72215883	72216283	-0.427956901	0.152905221	same
chr1	231603750	231604150	-0.451143142	0.152948861	same
chr11	62653341	62653741	-0.485209547	0.153007785	same
chr14	20999012	20999715	-0.468287239	0.15308297	same
chr4	76948777	76949177	-0.489523697	0.153134499	same
chr4	88523734	88524134	-0.492459971	0.153145733	same
chr15	89206888	89207288	-0.434414692	0.153237713	same
chr10	89645395	89645795	-0.451063623	0.153279662	same
chr9	121686784	121687184	-0.49196545	0.153378534	same
chr12	123141969	123142369	-0.475930261	0.153405488	same
chr19	51986477	51986877	-0.480751079	0.153637807	same
chr13	77679140	77679540	-0.483122259	0.153639317	same
chrX	38638877	38639277	-0.491553726	0.153712697	same
chr2	170771208	170771608	-0.483959375	0.153875652	same
chr10	102854310	102854710	-0.438370821	0.1539788	same
chr19	7616565	7616965	-0.488361793	0.154049526	same
chr1	84583146	84583546	-0.497890582	0.154301873	same
chr12	95003345	95003745	-0.459724976	0.154402483	same
chr13	41132168	41132568	-0.45115843	0.154425623	same
chr19	57633092	57633492	-0.461944224	0.15450511	same
chr8	143334907	143335307	-0.494597179	0.154616639	same
chr3	23629256	23629656	-0.491539273	0.154617091	same
chrX	119604984	119605384	-0.468566656	0.1546174	same
chr6	20211963	20212363	-0.474514328	0.154705661	same
chr16	19555327	19555727	-0.359264363	0.154716728	same
chr19	42282870	42283270	-0.465946316	0.154791143	same
chr1	146038798	146039198	-0.416024436	0.154936461	same
chr21	46286230	46286630	-0.443125651	0.154976433	same
chr15	40807301	40807701	-0.410254358	0.155033834	same
chr6	150068894	150069294	-0.444803038	0.155063415	same
chr12	54161578	54161978	-0.498145145	0.155112855	same
chr14	34461240	34461640	-0.481190748	0.155183914	same
chr2	165573914	165574314	-0.484930702	0.155394011	same
chr11	6926419	6926819	-0.43778116	0.155395629	same
chrX	48696194	48696594	-0.431977145	0.155428699	same
chr5	157858573	157858973	-0.481234084	0.155432345	same
chr10	119209240	119209640	-0.478084374	0.155435987	same
chr16	74743808	74744208	-0.459789708	0.15548256	same
chr9	35826047	35826447	-0.47811611	0.155651509	same
chr12	46383840	46384240	-0.481820064	0.155653484	same
chr7	123527739	123528139	-0.48805831	0.155685635	same
chr6	34392572	34392972	-0.489886638	0.155722473	same
chr2	15560394	15560794	-0.458985968	0.1558354	same
chr6	94680844	94681244	-0.48931484	0.156094152	same
chr5	84383627	84384027	-0.458899429	0.156150649	same
chr22	21665897	21666297	-0.400127193	0.156411191	same
chr11	36509916	36510316	-0.451835704	0.156587309	same
chr3	185498652	185499052	-0.479687386	0.156650212	same
chr12	68332022	68332422	-0.46309019	0.156952227	same
chr21	44802266	44802666	-0.473669527	0.157079886	same
chr1	110197010	110197410	-0.454396483	0.15714732	same
chr7	151518196	151518596	-0.425590935	0.157205083	same
chr13	79422111	79422511	-0.474931481	0.157232827	same
chr7	42856721	42857121	-0.48862573	0.157245178	same
chr1	46247047	46247447	-0.404547806	0.1573162	same
chr15	59105166	59105566	-0.457565338	0.15737783	same
chr1	179002554	179002954	-0.439416686	0.157393369	same

chr10	67884177	67884577	-0.428339614	0.157516367	same
chr15	83067277	83067677	-0.478871923	0.157543461	same
chr13	97222196	97222596	-0.473729652	0.157684157	same
chr1	208245048	208245448	-0.458519148	0.157746774	same
chr2	238256570	238256970	-0.469501877	0.157942565	same
chr15	90293359	90293759	-0.454584475	0.158143091	same
chrX	49002116	49002516	-0.413471465	0.158172828	same
chr1	179081625	179082025	-0.399869298	0.15819206	same
chr1	83102751	83103151	-0.464742275	0.15819403	same
chr4	72593815	72594215	-0.481665316	0.158248555	same
chr10	98134538	98134938	-0.434017131	0.158314228	same
chr2	176002040	176002440	-0.406144592	0.158346849	same
chr10	72273507	72273907	-0.352550595	0.158361457	same
chr13	76886622	76887022	-0.414487167	0.158373593	same
chr20	35698143	35698543	-0.427841322	0.158468741	same
chr5	119063623	119064023	-0.485752627	0.158471863	same
chr4	76306702	76307102	-0.459164327	0.158471912	same
chr2	73177425	73177825	-0.453455154	0.158527542	same
chr3	180602113	180602513	-0.455683233	0.158583506	same
chr5	83077138	83077538	-0.464476739	0.158606524	same
chrX	103152016	103152416	-0.462416976	0.158682657	same
chr2	88469441	88469841	-0.429348105	0.158712329	same
chr7	22083010	22083410	-0.446624598	0.158749462	same
chr21	43740049	43740449	-0.444915247	0.158770105	same
chr15	66386441	66386841	-0.416520225	0.158838192	same
chrX	153667689	153668089	-0.44625623	0.158858292	same
chr1	42166135	42166535	0.444499154	0.158966655	same
chr2	202238497	202238897	-0.439577187	0.158984123	same
chr12	1605422	1605822	-0.458038774	0.15901578	same
chr16	67247316	67247716	-0.395924657	0.159017948	same
chr4	15653959	15654359	-0.471212481	0.15903483	same
chr14	57268684	57269084	-0.42782984	0.159042654	same
chr9	129023731	129024131	-0.461529211	0.159153176	same
chr20	36773752	36774152	-0.371366964	0.159165923	same
chr7	87629034	87629434	-0.446634662	0.159189512	same
chrX	109732716	109733116	-0.470733301	0.159334616	same
chr19	37719537	37719937	-0.45935405	0.159396672	same
chr11	14907614	14908014	-0.477169781	0.159399137	same
chr8	105508606	105509006	-0.485923577	0.159405951	same
chr13	68405483	68405883	0.489463213	0.159406848	same
chr9	94726506	94726906	-0.456117738	0.159408719	same
chr1	25247452	25247852	-0.428806681	0.159418423	same
chr10	62268423	62268823	-0.466551815	0.159454734	same
chr2	171999874	172000274	-0.491854122	0.159475367	same
chr19	45952845	45953245	-0.469759576	0.159535534	same
chr10	51574525	51574925	-0.478469387	0.159568731	same
chr19	12881254	12881654	-0.414435889	0.159637414	same
chr2	241507583	241507983	-0.429946597	0.159754047	same
chr17	57892635	57893035	-0.459367253	0.159815027	same
chr19	1065393	1065793	0.452275873	0.159877225	same
chr1	61077709	61078109	-0.390828589	0.159884277	same
chr11	44565170	44565570	-0.446228816	0.160105365	same
chr1	110003808	110004208	-0.457904049	0.160128219	same
chr19	12957133	12957533	-0.458271782	0.160169864	same
chr5	111223754	111224154	-0.466668487	0.160204944	same
chr10	43205094	43205494	-0.436627129	0.160295346	same
chr6	27157831	27158231	-0.4633172	0.1603465	same
chr12	110583261	110583661	-0.473181903	0.160413578	same
chr9	94030436	94030836	-0.491580647	0.160444762	same
chr5	84382370	84382770	-0.455968562	0.160486089	same
chr1	156291428	156291828	-0.411845318	0.160510107	same
chr15	90157941	90158341	-0.441581771	0.160582678	same
chr19	16572575	16572975	-0.435251364	0.160615374	same
chr15	66842132	66842532	-0.474008957	0.160644365	same
chr6	27288427	27288827	-0.452177733	0.160668952	same
chr3	160577657	160578057	-0.482302973	0.160694991	same
chr12	51047838	51048238	-0.369450361	0.160722444	same
chr10	75508683	75509083	-0.429208224	0.160762338	same
chr2	201233416	201233816	-0.479391785	0.160904562	same
chr2	30146501	30146901	-0.412242045	0.161008281	same
chr11	65552094	65552494	-0.4242487	0.161166415	same
chr5	271035	271435	-0.399282642	0.161202448	same
chr16	31105468	31105868	-0.457020887	0.161207225	same
chrX	134549835	134550235	-0.457447054	0.161286111	same
chr5	138464981	138465381	-0.47040728	0.161317057	same
chr12	57846018	57846418	-0.456340047	0.161323551	same

chr19	35066954	35067354	-0.475058623	0.161349245	same
chr12	41288083	41288483	-0.48084783	0.161405663	same
chr1	51617110	51617510	-0.452925272	0.161414023	same
chr14	24296971	24297371	-0.425464003	0.161439292	same
chr9	92670344	92670744	-0.455602834	0.161598577	same
chr6	408698	409098	-0.473389342	0.161678336	same
chr19	35931475	35931875	-0.405491472	0.161720675	same
chr5	160438740	160439140	-0.462891491	0.161733848	same
chr1	240112238	240112638	-0.471452743	0.161808083	same
chr11	72025255	72025655	-0.470938958	0.161900258	same
chr19	47229099	47229499	-0.405728073	0.162057749	same
chr10	99430610	99431010	-0.376675428	0.162066069	same
chr3	121642904	121643304	-0.480526857	0.162078865	same
chr21	42974010	42974410	-0.446360488	0.162130383	same
chr6	116100676	116101076	-0.492058553	0.162272704	same
chr19	57363038	57363438	-0.435487654	0.162295761	same
chr12	3237003	3237403	-0.483232066	0.162297355	same
chr19	618701	619101	-0.444421509	0.162332623	same
chr19	47765730	47766130	-0.451342515	0.162343714	same
chr16	21599009	21599409	-0.477348229	0.162368078	same
chr1	111619314	111619714	-0.480940226	0.162458485	same
chr19	48110418	48110818	-0.455060541	0.162540262	same
chr9	110047889	110048289	-0.46888454	0.162630357	same
chr1	40097321	40097721	-0.443542338	0.162640108	same
chr3	86991458	86991858	-0.472680187	0.163076464	same
chr6	53794159	53794559	-0.384199558	0.163396564	same
chr6	44100171	44100571	-0.461438472	0.163406197	same
chr11	111926793	111927193	-0.461654788	0.163443813	same
chr7	85071114	85071514	-0.473313921	0.163467901	same
chr10	100185916	100186316	-0.432671531	0.163578497	same
chr1	67500525	67500925	-0.472542072	0.163609319	same
chr12	949432	949832	-0.406362143	0.163610728	same
chr1	145465339	145465739	-0.476497542	0.163679198	same
chr12	66130792	66131192	-0.466302573	0.163768868	same
chr5	94111387	94111787	-0.393139418	0.163777742	same
chr2	73926767	73927167	-0.483741165	0.163815948	same
chr12	29782784	29783184	-0.469412319	0.163865615	same
chr2	26012228	26012628	-0.480165902	0.163992669	same
chr13	64210221	64210621	-0.475770274	0.164001022	same
chr12	123711846	123712246	-0.415304727	0.164016144	same
chr8	37675279	37675679	-0.442589048	0.164018537	same
chr3	47282560	47282960	-0.410384735	0.164092263	same
chr17	68462808	68463208	-0.458199703	0.164191371	same
chr4	2535452	2535852	-0.455062076	0.164200516	same
chr12	118016603	118017003	-0.443350551	0.164210268	same
chr12	121095716	121096116	-0.478780376	0.164228714	same
chr11	70270182	70270582	-0.417927905	0.164235676	same
chrX	132488976	132489376	-0.474681754	0.164312621	same
chr1	45802930	45803330	-0.463707389	0.164409043	same
chr6	112163203	112163603	-0.453831481	0.164640574	same
chr15	63189046	63189446	-0.476085188	0.164706187	same
chrX	127878139	127878539	-0.433247032	0.164799055	same
chr4	155426916	155427316	-0.456757336	0.164914636	same
chr10	117375989	117376389	-0.432479853	0.164927373	same
chr19	10701528	10701928	-0.375775921	0.164989965	same
chr14	74414364	74414764	-0.470179007	0.164990488	same
chr13	75357908	75358308	-0.454690174	0.165006207	same
chrX	119887659	119888059	-0.412049675	0.165096494	same
chr19	48646361	48646761	-0.47106691	0.165277079	same
chr5	17379268	17379668	-0.472952305	0.165356057	same
chr11	76721860	76722260	-0.449094177	0.165477998	same
chr5	93582810	93583210	-0.427659472	0.165537831	same
chr1	37595627	37596027	-0.429227898	0.165644505	same
chr16	58198305	58198705	-0.476497072	0.165666429	same
chr2	105037883	105038283	-0.359581572	0.16587564	same
chr6	158744837	158745237	-0.475048187	0.16591855	same
chr6	107493138	107493538	-0.464893487	0.165998872	same
chr9	131477919	131478319	-0.465600429	0.166054843	same
chr1	184664025	184664425	-0.444518097	0.166072432	same
chr1	197683889	197684289	-0.473848431	0.166208165	same
chr1	68111912	68112312	-0.45204622	0.166237149	same
chr3	9422983	9423383	-0.481810182	0.166241656	same
chr15	71114914	71115314	-0.483500004	0.166266675	same
chr19	15378139	15378539	-0.425169036	0.166324976	same
chr9	5947937	5948337	-0.471862869	0.166335009	same
chr7_KI270803v1_alt	327391	327791	-0.476126385	0.166419587	same

chrX	155216203	155216603	-0.435267417	0.166441831	same
chr2	130391384	130391784	-0.445180642	0.166533996	same
chr15	41402149	41402549	-0.474431495	0.166536159	same
chr16	3400842	3401242	-0.416399209	0.166549563	same
chr1	35808930	35809330	-0.471002541	0.166576778	same
chr5	1222277	1222677	-0.457075906	0.166692406	same
chr5	145936444	145936844	-0.44318174	0.166803422	same
chr12	27780987	27781387	-0.48456912	0.166809867	same
chr16	636594	636994	-0.463008808	0.166877294	same
chr6	5996065	5996465	-0.441517306	0.166907256	same
chr7	28062215	28062615	-0.474590072	0.166927836	same
chr13	64397649	64398049	-0.45729641	0.167221169	same
chr10	30359702	30360102	-0.449876027	0.16726853	same
chr6	52517755	52518155	-0.462242024	0.167338335	same
chr17	75667027	75667427	-0.425230924	0.16755045	same
chr14	22853085	22853485	-0.432190536	0.167575156	same
chr21	16280227	16280627	-0.46945621	0.16760294	same
chr20	57265504	57265904	-0.451383419	0.167620887	same
chr16	71845631	71846031	-0.454786725	0.167660398	same
chr8	125091426	125091826	-0.462879276	0.167766902	same
chr14	71319906	71320306	-0.463398758	0.167883613	same
chr17	29591487	29591887	-0.369633479	0.167920098	same
chr11	90243355	90243755	-0.473656087	0.168000266	same
chr17	32444360	32444760	-0.426160592	0.16801023	same
chr14	67816315	67816715	-0.400511967	0.16825212	same
chr13	79441306	79441706	-0.37991382	0.168417949	same
chr7	87627957	87628357	-0.408558752	0.168494375	same
chr1	214261664	214262064	-0.450172914	0.168521561	same
chr12	66234165	66234565	-0.472902892	0.16864545	same
chr1	110106090	110106490	-0.448800737	0.168666461	same
chr11	47259345	47259745	-0.43723705	0.16874309	same
chr16	46883906	46884306	-0.387804329	0.168747853	same
chr15	78113303	78113703	-0.434292639	0.168824079	same
chr5	82932728	82933128	-0.45866243	0.168854683	same
chr11	44894052	44894452	-0.468336963	0.16891487	same
chr1	235511780	235512180	-0.417847895	0.168936372	same
chr1	146938382	146938782	-0.475971762	0.169171281	same
chrUn_KI270744v1	110920	111320	0.421621454	0.169172869	same
chr19	44051670	44052070	-0.425008871	0.169224675	same
chr13	63517209	63517609	-0.415605558	0.169281004	same
chr17	4703890	4704662	-0.402483747	0.16931348	same
chr9	94554352	94554752	-0.426454816	0.169328052	same
chr16	81403693	81404093	-0.462155653	0.169369856	same
chr19	6022170	6022570	-0.471023295	0.169382056	same
chr19	12840505	12840905	-0.380263425	0.169396074	same
chr22	23627590	23627990	-0.461690607	0.169428591	same
chr11	12067061	12067461	-0.463698484	0.169757065	same
chr2	74199172	74199572	-0.441377459	0.169776199	same
chr20	44909978	44910378	-0.476872503	0.170077429	same
chr9	137899846	137900246	-0.455312722	0.170080488	same
chr1	85298081	85298481	-0.421808424	0.170140989	same
chr2	177552648	177553048	-0.402799107	0.170146105	same
chr2	61426288	61426688	-0.470537921	0.170254094	same
chr20	25696708	25697108	-0.483998646	0.170324871	same
chr2	10448633	10449033	-0.453644189	0.170341719	same
chrX	13734394	13734794	-0.426518211	0.170469336	same
chrX	46903739	46904139	-0.45140365	0.170765375	same
chr7	39566285	39566685	-0.453034813	0.170886994	same
chr1	111739529	111739929	-0.37646781	0.170898826	same
chr9	4489680	4490080	-0.470027066	0.170909621	same
chr8	28092770	28093170	-0.476570132	0.170924902	same
chr6	96521835	96522235	-0.400331515	0.170991498	same
chr5	180493901	180494301	-0.429678374	0.171054961	same
chr11	65719079	65719479	-0.444719854	0.17107204	same
chrX	54529894	54530294	-0.433880969	0.171213867	same
chr19	2785163	2785563	-0.428309865	0.171344989	same
chr13	19959972	19960372	-0.455744705	0.171360277	same
chr12	6872134	6872534	-0.340375063	0.171465734	same
chr5	139670602	139671002	-0.468248473	0.17150134	same
chr4	154550287	154550687	-0.407318101	0.171605728	same
chr18	74291951	74292351	-0.444213735	0.171685386	same
chr11	33736779	33737179	-0.44436927	0.171687747	same
chr19	39480404	39480804	-0.423655319	0.171759414	same
chr12	131710828	131711228	-0.449207623	0.171844233	same
chr4	73259058	73259458	-0.388210159	0.171873383	same
chr22	37933797	37934197	-0.375182849	0.171884163	same

chr16	57984875	57985275	-0.431942316	0.171897688	same
chr17	17860104	17860504	-0.432890451	0.171974107	same
chr2	60756318	60756718	-0.446898417	0.171997772	same
chr1	204128739	204129139	-0.472914796	0.172020082	same
chr7	92424406	92424806	-0.43761442	0.172083167	same
chr1	236452111	236452511	-0.44502468	0.172123938	same
chr4	85796746	85797146	-0.466443099	0.172140253	same
chr6	116501007	116501407	-0.472772029	0.172146726	same
chr11	65078179	65078579	-0.433885582	0.172286257	same
chr20	18288124	18288524	-0.41016104	0.172442823	same
chr4	119454524	119454924	-0.43699457	0.172532407	same
chr6	27772370	27772770	-0.41112152	0.172646257	same
chr19	48445714	48446114	-0.37498336	0.17267754	same
chr22	23717111	23717511	-0.40383942	0.172816982	same
chr2	165953720	165954120	-0.479406726	0.172817096	same
chr1	231806583	231806983	-0.416439735	0.172864395	same
chr19	11346257	11346657	-0.39245637	0.172889082	same
chr13	66974245	66974645	-0.399070397	0.172910348	same
chr16	67109505	67109905	-0.437499305	0.172952767	same
chr8	144792469	144792869	-0.470666101	0.172955567	same
chr1	25819609	25820009	-0.449025266	0.173049452	same
chr3	53130319	53130719	-0.405119989	0.173103849	same
chr4	54539601	54540001	-0.462842929	0.173134125	same
chr5	172771954	172772354	-0.409751326	0.173182833	same
chr13	93233985	93234385	-0.447142265	0.173208574	same
chr12	114656193	114656593	-0.43052814	0.173337305	same
chrX	103624260	103624660	-0.418568178	0.173352342	same
chr1	234543945	234544345	-0.422499347	0.173397822	same
chr10	32346552	32346952	-0.461437525	0.173516734	same
chr6	154613294	154613694	-0.461244333	0.173582603	same
chr4	2469628	2470028	-0.429156737	0.17367455	same
chr3	51982738	51983138	-0.423974606	0.173830181	same
chr18	23437344	23437744	-0.473520989	0.173833509	same
chr5	159100375	159100775	-0.444915868	0.173900942	same
chr1	154272258	154272658	-0.401513643	0.173997174	same
chr2	219160426	219160826	-0.384128739	0.174013415	same
chr1	179025523	179025923	-0.458957663	0.174014604	same
chr6	106325358	106325758	-0.403411129	0.174022503	same
chr11	73983085	73983485	-0.412556423	0.174043438	same
chr7	70631158	70631558	-0.463874825	0.174074624	same
chr14	101476879	101477494	-0.446244797	0.17410342	same
chr17	3683091	3683491	-0.447109052	0.174147402	same
chrX	47129211	47129611	-0.397842293	0.174175064	same
chrX	102720473	102720873	-0.414889426	0.174200565	same
chr2	133435153	133435553	-0.416555487	0.174219554	same
chr7	89583972	89584372	-0.46048854	0.174227458	same
chr11	308351	308751	-0.468316909	0.174330309	same
chr7	151736523	151736923	-0.424891063	0.174335446	same
chr14	105162376	105162776	-0.408256927	0.174431302	same
chr17	62703589	62703989	-0.456896356	0.17447369	same
chr2	3574867	3575267	-0.4262529	0.174510446	same
chr1	53606724	53607124	-0.458584531	0.174536762	same
chr17	9644894	9645294	-0.438643231	0.174556878	same
chr19	41706805	41707205	-0.429656691	0.174618294	same
chr2	42025086	42025486	-0.462285926	0.174849148	same
chrX	65034913	65035313	-0.458274595	0.174903263	same
chr2	72916515	72916915	-0.43301497	0.17495699	same
chr13	51452883	51453283	-0.410678464	0.175268287	same
chr11	9568971	9569371	-0.438891226	0.175370547	same
chr14	20663172	20663572	-0.382640346	0.175538291	same
chr1	182789182	182789582	-0.431231866	0.175655519	same
chr21	42411355	42411755	-0.450153313	0.175701156	same
chr19	23686782	23687182	-0.428266105	0.175994511	same
chr1	16367231	16367631	-0.440582217	0.175995634	same
chr4	89837383	89837783	-0.454343419	0.176046955	same
chr1	178093313	178093713	-0.421650079	0.176410157	same
chr15	64153628	64154028	-0.434883864	0.176450615	same
chr10	102394089	102394489	-0.383754557	0.176460955	same
chr11	65507322	65507722	0.405769209	0.176570911	same
chr16	3443572	3443972	-0.447085112	0.176599125	same
chr22	28678308	28678708	-0.469243435	0.176627124	same
chr3	133662053	133662453	-0.460901809	0.17664388	same
chr17	42221775	42222175	-0.458784973	0.176805749	same
chr7	66681835	66682235	-0.406628798	0.176984976	same
chr17	43906372	43906772	-0.455578548	0.177033762	same
chr12	52213534	52213934	-0.435853328	0.177125784	same

chr11	14969728	14970128	-0.471966314	0.17715657	same
chr12	121773154	121773554	-0.415220502	0.177180157	same
chr12	71754407	71754807	-0.4431808	0.177234384	same
chr6	165309210	165309610	-0.399675939	0.177254883	same
chr20	3115381	3115781	-0.456268596	0.177327266	same
chr8	48009005	48009405	-0.460506055	0.177489618	same
chr15	84035164	84035564	-0.449012449	0.177501085	same
chr15	42548646	42549046	-0.431213488	0.177525443	same
chr18	23503044	23503444	-0.409914219	0.177668806	same
chr6	27478906	27479306	-0.447679032	0.177902399	same
chr12	116257039	116257439	-0.462811754	0.178011004	same
chr11	66328189	66328589	-0.433424527	0.178107033	same
chr2	64144395	64144795	-0.419411618	0.178142197	same
chr20	32028802	32029202	-0.445781873	0.178156447	same
chr1	173868084	173868484	-0.424813161	0.17825012	same
chr1	40040232	40040632	-0.380886492	0.178319887	same
chr13	62394746	62395146	-0.459990782	0.178349502	same
chr10	104353342	104353742	-0.444397864	0.178362796	same
chr1	63773742	63774142	-0.422176243	0.178377791	same
chr20	44018131	44018531	-0.407580422	0.178385588	same
chr15	83011513	83011913	-0.362542319	0.178400159	same
chr9	6645666	6646066	-0.444410133	0.178478575	same
chr1	109397928	109398328	-0.450261247	0.178504865	same
chr13	64847823	64848223	0.455082698	0.178514958	same
chr1	227762305	227762705	-0.391116817	0.178526483	same
chr15	41283770	41284170	-0.462952393	0.178691897	same
chr5	151786439	151786839	-0.461643905	0.178861873	same
chr10	15169062	15169462	-0.439490104	0.178943239	same
chr12	53371089	53371489	-0.412030392	0.179113966	same
chr22	25333771	25334171	-0.439339871	0.179300563	same
chr21	25801415	25801815	-0.456047658	0.179466659	same
chr19	3538218	3538618	-0.433415262	0.179472158	same
chr6	16761846	16762246	-0.44316734	0.179535233	same
chr15	88624116	88624516	-0.424933156	0.179600922	same
chr11	47523724	47524124	-0.457107507	0.179668407	same
chr13	50386947	50387347	-0.431909701	0.179690663	same
chr22	31107356	31107756	-0.446830132	0.179877568	same
chr6	17601037	17601437	-0.443326008	0.179977089	same
chr7	5570054	5570454	-0.460951346	0.18013375	same
chr1	36306919	36307319	-0.427379787	0.180138622	same
chr2	37196376	37196776	-0.431542531	0.180209031	same
chr1	204889544	204889944	-0.444006867	0.180226342	same
chr10	46525021	46525421	-0.416940367	0.180236065	same
chr1	118185091	118185491	-0.42856231	0.180302737	same
chr1	150697126	150697526	-0.413045088	0.180405517	same
chr20	59835784	59836184	-0.445902348	0.180409124	same
chrX	103310894	103311294	-0.4469418	0.18045163	same
chr22	20320107	20320507	-0.417752073	0.180768184	same
chr2	214809811	214810211	-0.471533777	0.180838379	same
chr19	37655454	37655854	-0.472352651	0.180859316	same
chr3	180989431	180989831	-0.41387841	0.18109695	same
chr10	58385244	58385644	-0.359435374	0.181167755	same
chr17	49677916	49678316	-0.39882492	0.181231314	same
chr17	4796051	4796451	-0.419276596	0.181252354	same
chr16	71808769	71809169	-0.430567626	0.181361782	same
chr21	29300064	29300464	-0.402735962	0.181369776	same
chr6	116325893	116326293	-0.456780641	0.181421774	same
chr1	153990597	153990997	-0.41094557	0.181424076	same
chr11	86924727	86925127	-0.408928124	0.181618614	same
chr6	26172149	26172549	-0.410508782	0.181688306	same
chr6	2999661	3000061	-0.41797971	0.181724097	same
chr8	94894931	94895331	-0.464929141	0.181783064	same
chr11	121236544	121236944	-0.411839617	0.181809583	same
chr11	74748470	74748870	-0.445407693	0.181816704	same
chr1	171742110	171742510	-0.425715253	0.181878269	same
chr3	193593168	193593568	-0.414138798	0.182087311	same
chr3	32177620	32178020	-0.411373653	0.182277445	same
chr11	129695782	129696182	-0.457458741	0.182292673	same
chr19	39824343	39824743	-0.426938485	0.182306466	same
chr4	44678178	44678578	-0.44526843	0.182327955	same
chr6	124981638	124982038	-0.454328989	0.182329996	same
chr16	234425	234825	-0.455213788	0.182375809	same
chr6	165663705	165664105	-0.424954626	0.182553349	same
chr20	31613025	31613425	-0.4452492	0.182635008	same
chr2	206159367	206159767	-0.376091552	0.182644829	same
chr8	143902610	143903010	-0.444919006	0.182669239	same

chr19	45442997	45443397	-0.414095433	0.182719192	same
chr10	24724214	24724614	-0.428248073	0.182767734	same
chr1	171644926	171645326	-0.410492252	0.182792699	same
chr17	56780405	56780805	-0.451464711	0.182922839	same
chr7	2451501	2451901	-0.398412611	0.182988802	same
chr3	112526554	112526954	0.454712983	0.183041935	same
chr16	56682040	56682440	-0.419438469	0.183178212	same
chr21	38805308	38805708	-0.450978756	0.183249315	same
chr18	34977821	34978221	-0.382495524	0.18329556	same
chr3	43769600	43770000	-0.407986257	0.183457268	same
chr16	58000949	58001349	-0.46908208	0.183473748	same
chr6	111605652	111606052	-0.417639274	0.183656766	same
chr19	33521936	33522336	-0.441875439	0.183679846	same
chr9	137200825	137201225	-0.398876066	0.183749612	same
chr8	52713176	52713576	-0.428180565	0.183749999	same
chr15	77543426	77543826	-0.409885856	0.183762212	same
chr16	3129761	3130161	-0.446553554	0.183813318	same
chr11	65497281	65497681	-0.398302059	0.183856626	same
chr16	58121920	58122320	-0.437603695	0.183867653	same
chr1	31715327	31715727	-0.313988571	0.183948528	same
chr8	129939726	129940126	-0.400031968	0.183993632	same
chr2	37231472	37231872	-0.384779228	0.183995167	same
chr20	3846895	3847295	-0.439700198	0.184006713	same
chr11	77047445	77047845	-0.458753403	0.184051081	same
chr7	149625478	149625878	-0.372703597	0.184138704	same
chr3	141077004	141077404	-0.44261628	0.184175038	same
chr6	109250842	109251242	-0.451041004	0.184241454	same
chr5	6745509	6745909	-0.455471846	0.184345759	same
chr5	43039703	43040103	-0.391916169	0.184368314	same
chr1	211619858	211620258	-0.442472239	0.184388266	same
chr18	36306138	36306538	-0.418633705	0.184422883	same
chr10	79445721	79446121	-0.41572876	0.184450832	same
chr2	73828871	73829271	-0.434677918	0.184544003	same
chr3	141231287	141231687	-0.437084533	0.184782864	same
chr2	69741264	69741664	-0.447622855	0.184940184	same
chr6	162727774	162728174	-0.41474808	0.184974094	same
chr4	2041490	2041890	-0.425587812	0.184986948	same
chr14	50321135	50321535	-0.452387838	0.185018381	same
chr6	44234222	44234622	-0.403877421	0.18525592	same
chr17	76547325	76547725	-0.408154182	0.185298183	same
chr3	84958584	84958984	-0.443865018	0.185344494	same
chr15	91032965	91033365	-0.446560404	0.185587395	same
chr17_GL000205v2_random	37527	37927	0.42277871	0.185728947	same
chr6	166342490	166342890	-0.416975485	0.185754288	same
chr6	1607841	1608241	-0.416312186	0.185763626	same
chr3	138935930	138936330	-0.406421946	0.185764095	same
chr14	105489526	105489926	-0.455087085	0.185805718	same
chr2	119366903	119367303	-0.355749098	0.185830165	same
chr7	157338798	157339198	-0.394823785	0.185843146	same
chr11	62664954	62665354	-0.35833142	0.186117791	same
chr10	122954049	122954449	-0.434564587	0.186243732	same
chr19	41873022	41873422	-0.392748268	0.186384505	same
chr10	132572229	132572629	-0.412564282	0.186712142	same
chr12	55681606	55682006	-0.421414163	0.186781205	same
chr11	9613545	9613945	-0.363222594	0.187043114	same
chr13	62561910	62562310	-0.44524124	0.18705252	same
chr2	181456930	181457330	-0.450500686	0.187217276	same
chr16	31033064	31033464	-0.399011873	0.187226626	same
chr2	20584579	20584979	0.391978139	0.187249956	same
chr3	29683554	29683954	-0.455646482	0.187272114	same
chr3	141370232	141370632	-0.421627754	0.187312467	same
chr12	111597526	111597926	-0.422585352	0.187320046	same
chrX	154490769	154491169	-0.398481459	0.18734084	same
chr14	24160992	24161392	-0.373737662	0.187413452	same
chr12	4562021	4562421	-0.45545124	0.187417578	same
chr15	89893805	89894205	-0.386394305	0.187476521	same
chr11	1408614	1409014	-0.434052954	0.187799344	same
chr1	31238576	31238976	-0.454182317	0.187810749	same
chr17	7013177	7013577	-0.414027128	0.187821148	same
chr3	13479802	13480202	-0.441349503	0.18792715	same
chr15	44288583	44288983	-0.459889021	0.188027747	same
chr14	58773211	58773611	-0.428158685	0.188328496	same
chr19	3557638	3558038	-0.400608459	0.188333941	same
chr7	155205346	155205746	-0.322120926	0.188349769	same
chr4	105708444	105708844	-0.40022093	0.188378959	same
chr1	244461174	244461574	-0.370737284	0.188388146	same

chrX	153922968	153923368	-0.41111418	0.188419571	same
chr19	48634940	48635340	-0.406168857	0.188498993	same
chr7	99506247	99506647	-0.419067269	0.188568931	same
chrX	153794083	153794483	-0.349469948	0.188596167	same
chr17	42101639	42102039	-0.430092327	0.188646896	same
chr11	57325065	57325465	-0.377166612	0.188710742	same
chr6	83859605	83860005	-0.417677582	0.18877285	same
chrX	152941621	152942021	-0.407735643	0.188791992	same
chr1	24642822	24643222	-0.439501239	0.188917245	same
chr17	41918363	41918763	-0.401881682	0.188919054	same
chrX	54440214	54440614	-0.421307367	0.188936076	same
chr3	51974191	51974591	-0.383678812	0.188995951	same
chr11	62560751	62561151	-0.392064166	0.189117691	same
chr8	53880202	53880602	-0.404826671	0.189163033	same
chr1	225988072	225988472	-0.429462682	0.18931636	same
chr1	165598025	165598425	-0.430245171	0.189365168	same
chrX	38803908	38804308	-0.382768634	0.189454417	same
chr14	67820437	67820837	-0.424088055	0.18946857	same
chr15	83436330	83436730	-0.443674077	0.189495269	same
chr5	139403764	139404164	-0.36040545	0.189534453	same
chr1	205227518	205227918	-0.437950277	0.189545055	same
chr11	45847074	45847474	-0.404875946	0.18960909	same
chr1	110319281	110319681	-0.42981466	0.189628303	same
chr1	222825784	222826184	-0.407785273	0.189670493	same
chr11	73393408	73393808	-0.403105953	0.189676398	same
chr10	133329552	133329952	-0.411089758	0.189690869	same
chr19	44094483	44094883	-0.439885486	0.189728554	same
chr19	49546842	49547242	-0.441939838	0.189760959	same
chr10	125707982	125708382	-0.419934561	0.189765508	same
chr18	23690296	23690696	-0.444670855	0.189877115	same
chr1	148151985	148152385	-0.461048925	0.190100451	same
chr21	36385024	36385424	-0.429265483	0.190121282	same
chr19	43504141	43504541	-0.405027997	0.190330873	same
chr9	127245114	127245514	-0.390696782	0.190361411	same
chr5	100535299	100535699	-0.416252579	0.190542269	same
chr22	17910848	17911248	-0.449155038	0.19066672	same
chr14	54565252	54565652	-0.447459216	0.190873911	same
chr2	25042905	25043305	-0.434712913	0.190990269	same
chr17	13602156	13602556	-0.433477282	0.191084666	same
chr9	96566845	96567245	-0.454929965	0.191088782	same
chr11	6612300	6612700	-0.384127977	0.19112885	same
chr9	131057753	131058153	-0.440382202	0.19116373	same
chr15	90934989	90935389	-0.324335573	0.191230848	same
chr17	75524262	75524662	-0.414034141	0.191277959	same
chr16	28925313	28925713	-0.404630333	0.191305016	same
chr14	52730097	52730497	-0.367498937	0.191309417	same
chr11	117045349	117045749	-0.451080857	0.191335307	same
chr2	173965443	173965843	-0.42742508	0.191403079	same
chr18	24076243	24076643	-0.41910888	0.19142127	same
chr22	39088230	39088630	-0.434992862	0.191495721	same
chr3	51705249	51705649	-0.413105954	0.19151542	same
chr11	125141821	125142221	-0.443061427	0.191566376	same
chr9	105782695	105783095	-0.412402378	0.191590393	same
chr2	182915365	182915765	-0.425499369	0.191670564	same
chr19	34733822	34734222	-0.391062804	0.191710908	same
chr1	1919239	1919639	-0.456408621	0.191875648	same
chr13	42333989	42334389	-0.447595862	0.191951542	same
chr10	80444851	80445251	-0.451745233	0.191965307	same
chr12	120495869	120496269	-0.454255518	0.192023639	same
chr3	49929646	49930046	-0.373591243	0.192296149	same
chr7	70092490	70092890	-0.443298488	0.192322202	same
chr4	77158047	77158447	-0.400967451	0.192389413	same
chr20	33401123	33401523	-0.40209885	0.192390146	same
chr2	11343155	11343555	-0.428854154	0.192398957	same
chr1	29181877	29182277	-0.38751069	0.192491266	same
chr11	20387181	20387581	-0.39829627	0.192544695	same
chr12	96907749	96908149	-0.426436206	0.192619276	same
chr14	58239680	58240080	-0.430491813	0.192681742	same
chr2	74198276	74198676	-0.39229838	0.192768504	same
chr8	73136102	73136502	-0.411712617	0.192776901	same
chr12	12611696	12612096	-0.400543552	0.193011464	same
chr4	3480630	3481030	-0.424639293	0.193047503	same
chr1	160078551	160078951	-0.435440021	0.193062606	same
chr5	98927275	98927675	-0.442810161	0.193079113	same
chr17	3636383	3636783	-0.384000853	0.193177519	same
chrX	76148762	76149162	-0.418005675	0.193293412	same

chr17	50346866	50347266	-0.416227324	0.19335818	same
chr1	16216304	16216704	-0.406909001	0.193583437	same
chr20	25750793	25751193	-0.449930403	0.193631821	same
chr20	4014073	4014473	-0.447484859	0.193632651	same
chr10	102432363	102432763	-0.368630226	0.193733967	same
chr16	1943180	1943580	-0.360058493	0.193790822	same
chr12	107092278	107092678	-0.40909389	0.193881743	same
chr1	109532555	109532955	-0.398043869	0.193972365	same
chr19	18328582	18328982	-0.444533976	0.194028823	same
chr1	108876639	108877039	-0.426970951	0.194063469	same
chr3	47522292	47522692	-0.409878082	0.194135909	same
chr11	17352523	17352923	-0.418159954	0.194210125	same
chr9	34329311	34329711	-0.344110324	0.194251834	same
chr11	6362879	6363279	-0.453043462	0.194279343	same
chr9	132355202	132355602	-0.435892418	0.194356237	same
chr5	154190190	154190590	-0.441161239	0.194438596	same
chr1	81920290	81920690	-0.434988284	0.194452484	same
chr1	179877698	179878098	-0.362313541	0.194575378	same
chr11	118572607	118573007	-0.403648423	0.194615272	same
chr1	78491092	78491492	-0.438680041	0.194638174	same
chr3	114498666	114499066	-0.441467619	0.194684194	same
chr5	139560881	139561281	-0.388905087	0.194703446	same
chr15	41660355	41660755	-0.41565897	0.194734854	same
chr11	65911858	65912258	-0.416443362	0.194739713	same
chr7	19708752	19709152	-0.411516815	0.194757823	same
chr8	111236250	111236650	-0.44420191	0.19508124	same
chr13	50357428	50357828	-0.448501649	0.195588947	same
chr12	38316691	38317091	-0.442510355	0.195592767	same
chr7	37916906	37917306	-0.446706887	0.195787878	same
chr7	1176200	1176600	-0.40770599	0.195870584	same
chr4	158824505	158824905	-0.442311306	0.195875229	same
chr12	30695854	30696254	-0.448405229	0.195876559	same
chr14	57391490	57391890	-0.438938376	0.195895677	same
chr7	158976733	158977133	-0.422159146	0.195912556	same
chr1	224004664	224005064	-0.4390499	0.195917015	same
chr11	110092112	110092512	-0.422040043	0.195954714	same
chr2	27582874	27583274	-0.395805509	0.195966362	same
chr2	152336506	152336906	-0.427756051	0.196051577	same
chr5	178212584	178212984	-0.340758081	0.19615037	same
chr15	64703343	64703743	-0.43880431	0.196166375	same
chr4	602437	602837	-0.447098603	0.19616791	same
chr6	134052212	134052612	-0.395854444	0.196174374	same
chr2	218979882	218980282	-0.420919075	0.196447344	same
chr14	71628548	71628948	-0.4485506	0.19660405	same
chr1	28643029	28643429	-0.340892399	0.196611957	same
chr20	62900304	62900704	-0.359309099	0.196623105	same
chr19	11090004	11090404	-0.407202797	0.196632258	same
chr22	17876399	17876799	-0.448149053	0.196640026	same
chr7	44490600	44491000	-0.429938945	0.197023075	same
chr12	53938162	53938562	-0.421523873	0.197034625	same
chr1	70420158	70420558	-0.427413445	0.197046557	same
chr5	55712259	55712659	-0.408814948	0.197103401	same
chr17	50719375	50719775	-0.417854047	0.197127623	same
chr6	160519729	160520129	-0.428599354	0.197254853	same
chr2	17518259	17518659	-0.422056756	0.197367281	same
chr19	10424171	10424571	-0.374276133	0.197410676	same
chr1	62724157	62724557	-0.427899354	0.197473218	same
chr1	156025957	156026357	-0.408250703	0.197678883	same
chr13	77135870	77136270	-0.436686196	0.197700015	same
chr5	179559449	179559849	-0.332117764	0.197705877	same
chr9	35096028	35096428	-0.405601591	0.197888143	same
chr1	1504954	1505354	-0.384134652	0.197910778	same
chr20	35269382	35269782	-0.437504562	0.198220326	same
chr17	7579311	7579711	-0.303607869	0.198326276	same
chr16	85010455	85010855	-0.431987939	0.198480978	same
chr15	90706468	90706868	-0.432621915	0.198650817	same
chr3	160399227	160399627	-0.40650937	0.198694739	same
chr11	34358783	34359183	-0.422038376	0.198701163	same
chr1	89762907	89763307	-0.410980753	0.198765866	same
chrX	71181582	71181982	-0.443420489	0.198826652	same
chr3	180679579	180679979	-0.440179116	0.198998194	same
chr18	11851363	11851763	-0.432858771	0.199091766	same
chr3	168248931	168249331	-0.436532283	0.199275248	same
chr1	206726109	206726509	-0.428742779	0.199301475	same
chr8	113046469	113046869	-0.446394853	0.199410998	same
chr7	92178886	92179286	-0.434574514	0.199439717	same

chr3	131381500	131381900	-0.348716246	0.199730785	same
chr1	45303777	45304177	-0.382643086	0.199750796	same
chr10	15856571	15856971	-0.428085908	0.199804571	same
chr4	151015879	151016279	-0.420589468	0.199864593	same
chr20	35954314	35954714	-0.375468936	0.200176441	same
chr1	18956773	18957173	-0.437347005	0.200235948	same
chrX	131752926	131753326	-0.430189019	0.200479045	same
chr17	59707500	59707900	-0.370272701	0.200895449	same
chr2	237085598	237085998	-0.395431255	0.200950514	same
chr5	51399048	51399448	-0.421708893	0.201020794	same
chr8	96235342	96235742	-0.394074756	0.201178707	same
chr3	53347277	53347677	-0.403883696	0.201392172	same
chr5	140591643	140592043	-0.432510828	0.201529929	same
chr12	112125380	112125780	-0.419613511	0.201569754	same
chr6	111560044	111560444	-0.434542634	0.201582415	same
chr3	11155243	11155643	-0.431017929	0.201587572	same
chr19	55447380	55447780	-0.406936356	0.201767667	same
chr10	91799152	91799552	-0.432109414	0.201851496	same
chr2	205680149	205680549	-0.398502458	0.201907449	same
chr20	58651011	58651411	-0.402745831	0.201977349	same
chr7	5495034	5495434	-0.431528592	0.202048324	same
chr12	109573238	109573638	-0.330068182	0.202191518	same
chr2	98086600	98087000	-0.433679738	0.202254774	same
chr5	172453184	172453584	-0.429614964	0.202256617	same
chr16	3189193	3189593	-0.350168209	0.202355063	same
chr13	64982418	64982818	-0.412279998	0.202404279	same
chr12	10153439	10153839	-0.437179228	0.202452515	same
chrX	84501998	84502398	-0.430640968	0.202538473	same
chr16	30598000	30598400	-0.421877687	0.202589754	same
chr20	38110047	38110447	-0.432193486	0.202784491	same
chr14	90383131	90383531	-0.339965401	0.203067894	same
chr6	127121139	127121539	-0.433202073	0.203263144	same
chr11	65427221	65427621	-0.292533418	0.203281069	same
chr1	233113243	233113643	-0.39904416	0.203431706	same
chr6	43089763	43090163	-0.412257175	0.203441876	same
chr8	131903399	131903799	-0.425700869	0.203468671	same
chr16	12067794	12068194	-0.436336028	0.203656535	same
chr17	47492471	47492871	-0.435307608	0.203685096	same
chr22	31753185	31753585	-0.394227558	0.203727994	same
chr21	36069810	36070210	-0.404639112	0.203751245	same
chr1	93680591	93680991	-0.406946699	0.2037858	same
chr4	183987636	183988036	-0.425335449	0.203811408	same
chr9	136758203	136758603	-0.401799791	0.203820186	same
chr5	430909	431309	-0.394549878	0.203910985	same
chr19	8390013	8390413	-0.384939713	0.203954944	same
chr14	101948147	101948547	-0.312178716	0.203970895	same
chr1	205321542	205321942	-0.407310613	0.203988023	same
chrX	130401556	130401956	-0.432534	0.204000794	same
chr10	118754848	118755248	-0.441260535	0.204047286	same
chr1	181033807	181034207	-0.435679414	0.204051294	same
chr20	36612525	36612925	-0.424255231	0.20429643	same
chr17	75238799	75239199	-0.348254759	0.204347505	same
chr9	935321	935721	-0.432117413	0.204348506	same
chr1	53838240	53838640	-0.355094256	0.20441956	same
chr2	178112321	178112721	-0.398610549	0.204483838	same
chr22	45309451	45309851	-0.420185519	0.204498928	same
chr17	30477328	30477728	-0.419284266	0.204503996	same
chr17	56592636	56593036	-0.439761654	0.204505368	same
chr1	16727336	16727736	-0.365355561	0.204625344	same
chr4	69034362	69034762	-0.431449348	0.204649068	same
chr1	214440221	214440621	-0.405851718	0.204704712	same
chr2	191020364	191020764	-0.423348031	0.204727779	same
chr6	158242300	158242700	-0.417814769	0.204812207	same
chrX	130702905	130703305	-0.40615921	0.205061866	same
chr2	26017768	26018168	-0.425194827	0.205061989	same
chr2	118186261	118186661	-0.400925512	0.205189836	same
chr11	77126841	77127241	-0.419794476	0.205206485	same
chr5	164188708	164189108	-0.362399286	0.205253482	same
chr1	54715490	54715890	-0.372460455	0.205426155	same
chr7	139109127	139109527	-0.372009977	0.205505267	same
chr19	2034826	2035226	-0.402341145	0.205550058	same
chr1	244371857	244372257	-0.419392021	0.205697866	same
chr6	138105735	138106135	-0.418405182	0.205730708	same
chr16	2663707	2664107	-0.415890357	0.205750211	same
chr21	37009935	37010335	-0.419985893	0.205751772	same
chr6	7338974	7339374	-0.43639751	0.205791422	same

chr18	35583166	35583566	-0.395912499	0.205814899	same
chr1	45018712	45019112	-0.404821728	0.205828897	same
chr1	161766355	161766755	-0.396809695	0.20593356	same
chr14	51093784	51094184	-0.420462374	0.205935193	same
chr2	71277533	71277933	-0.366471714	0.205938922	same
chr12	106302443	106302843	-0.420546039	0.205998602	same
chr11	34105170	34105570	-0.326100692	0.206207292	same
chr1	109075873	109076273	-0.37478368	0.206535377	same
chr10	78974646	78975046	-0.383373234	0.206537953	same
chr2	27409611	27410011	-0.423159687	0.206571774	same
chr7	158524404	158524804	-0.40480379	0.206763769	same
chr11	46948426	46948826	-0.430526846	0.206814546	same
chr18	9913533	9913933	-0.412736484	0.207129543	same
chr20	26010051	26010451	-0.384622624	0.207159134	same
chr4	70839788	70840188	-0.418808905	0.207317866	same
chr16	66977572	66977972	-0.384948849	0.207387746	same
chr1	151992254	151992654	-0.380594753	0.207468146	same
chr6	46129747	46130147	-0.409451054	0.207541664	same
chr10	102743726	102744126	-0.357966578	0.207628053	same
chr19	10603838	10604238	-0.411350508	0.207756811	same
chr7	100705587	100705987	-0.345629356	0.207819934	same
chr16	11835833	11836233	-0.408472596	0.208057682	same
chr13	66558662	66559062	-0.400655963	0.208130908	same
chrX	119516070	119516470	-0.409612316	0.208222488	same
chr11	128999103	128999503	-0.402079804	0.208317401	same
chr5	138536685	138537085	-0.37172906	0.208382367	same
chr20	3767913	3768313	-0.350530586	0.208408004	same
chr1	150293801	150294201	-0.37205125	0.208487882	same
chr9	113221218	113221618	-0.386772531	0.208573854	same
chr12	51951212	51951612	-0.401342697	0.208650053	same
chr5	893390	893790	-0.403955706	0.208656639	same
chr15	99306658	99307058	-0.421672042	0.208658545	same
chr20	50165858	50166258	-0.424606105	0.20866589	same
chr19	8002043	8002443	-0.397000404	0.208713986	same
chr1	75764216	75764616	-0.425395115	0.208728896	same
chr10	46228789	46229189	-0.401934133	0.208742317	same
chr9	128204109	128204509	-0.403163395	0.208757389	same
chr5	10607390	10607790	-0.428420001	0.208771529	same
chr2	127050275	127050675	-0.404902428	0.208884667	same
chr13	91375217	91375617	-0.403234686	0.208920132	same
chr2	33475834	33476234	-0.417590119	0.208993128	same
chr7	82444287	82444687	-0.434488086	0.209037513	same
chr1	92030184	92030584	-0.403097674	0.209055331	same
chr19	32675103	32675503	-0.439429358	0.209128872	same
chr7	101169557	101169957	-0.425651813	0.209326126	same
chr1	220990796	220991196	-0.37878286	0.209415829	same
chr12	121803145	121803545	-0.398393654	0.209574267	same
chr13	66405740	66406140	-0.427144881	0.209619694	same
chr8	74350310	74350710	-0.340574728	0.209684293	same
chr9	136362922	136363322	-0.324373528	0.20968942	same
chr11	27471236	27471636	-0.429318473	0.209746053	same
chr7	90245117	90245517	-0.397063322	0.209801849	same
chr9	127904748	127905148	-0.423821447	0.209993746	same
chr8	71361700	71362100	-0.41828191	0.210403594	same
chr5	10627707	10628107	-0.388364575	0.210588393	same
chr8	108042822	108043222	-0.417609193	0.210697059	same
chr11	57457433	57457833	-0.421987676	0.210705468	same
chr19	57240376	57240776	-0.375349573	0.210945783	same
chr2	218186357	218186757	-0.415369919	0.211194166	same
chr2	42663208	42663608	-0.426891827	0.211212251	same
chr1	234927169	234927569	-0.404263237	0.211228218	same
chr3	116190222	116190622	-0.427867239	0.211335799	same
chr2	177265059	177265459	-0.386769187	0.211377306	same
chrX	46573317	46573717	-0.398525182	0.211378179	same
chr13	91398804	91399204	-0.382258178	0.211507144	same
chr1	32612110	32612510	-0.371430798	0.211580106	same
chr2	144461303	144461703	-0.423106298	0.211580502	same
chr11	73141143	73141543	-0.405603115	0.211614086	same
chr16	730337	730737	0.384013452	0.211651369	same
chr19	55116373	55116773	-0.396973165	0.211919045	same
chr2	63050642	63051042	-0.429757972	0.21212986	same
chr1	165444956	165445356	-0.413226287	0.212192043	same
chr20	31514517	31514917	-0.41117472	0.212228098	same
chr6	157106404	157106804	-0.423634094	0.212366783	same
chr13	62887151	62887551	-0.39692956	0.212454954	same
chr22	25447738	25448138	-0.35661884	0.212486982	same

chr6	158506591	158506991	-0.409966043	0.212536459	same
chr10	655725	656125	-0.415413685	0.212552549	same
chr5	149381639	149382039	-0.425437017	0.212643605	same
chr17	75240437	75240837	-0.371267535	0.212841877	same
chr16	29951712	29952112	-0.408849114	0.212894137	same
chr4	143381527	143381927	-0.399573844	0.212913963	same
chr8	56993917	56994317	-0.393646141	0.212948432	same
chr14	51240006	51240406	-0.373935393	0.212985966	same
chr13	26983099	26983499	-0.417081715	0.21299938	same
chr20	3437546	3437946	-0.422976321	0.213008785	same
chr22	23521386	23521786	-0.409507421	0.213040317	same
chr12	24948890	24949290	-0.413032719	0.213162631	same
chr20	2664074	2664474	-0.350350855	0.213277048	same
chr9	85743475	85743875	-0.366296242	0.213311412	same
chr9	101449127	101449527	-0.426602026	0.213573621	same
chr12	132163782	132164182	-0.40220426	0.213717131	same
chr19	38374386	38374786	-0.387348687	0.213737627	same
chr22	40346331	40346731	-0.368960427	0.213887378	same
chr14	24431531	24431931	-0.394023489	0.213910565	same
chr16	84138875	84139275	-0.393645253	0.214000453	same
chr11	63974304	63974704	-0.350829963	0.214030994	same
chr8	42391475	42391875	-0.425788694	0.214215444	same
chr1	216723235	216723635	-0.420920146	0.214245439	same
chr1	236085793	236086193	-0.386816096	0.21430454	same
chr17_KI270729v1_random	2864	3264	0.346167363	0.214333837	same
chrX	72019521	72019921	-0.424899376	0.214348384	same
chr10	124741237	124741637	-0.406380413	0.21435303	same
chr19	4567478	4567878	-0.391981717	0.214375177	same
chr13	71831668	71832068	-0.386159035	0.214749689	same
chr6	76441879	76442279	-0.419093458	0.214880096	same
chr15	66387592	66387992	-0.40064945	0.214887851	same
chr8	123072492	123072892	-0.362285043	0.214906506	same
chr17	17204794	17205194	-0.412232271	0.215068544	same
chr4	80266489	80266889	-0.396711628	0.215222334	same
chr17	49952364	49952764	-0.402149065	0.215390905	same
chr5	171494497	171494897	-0.400607186	0.215787456	same
chr11	10735586	10735986	-0.408949712	0.215858237	same
chr16	75111081	75111481	-0.384165764	0.215867262	same
chr9	126704940	126705340	-0.417123412	0.216025242	same
chr2	27380929	27381329	-0.395883006	0.216144106	same
chr16	21820385	21820785	-0.397105968	0.216275217	same
chr2	105337152	105337552	-0.403636168	0.216316754	same
chr1	168225925	168226325	-0.411622068	0.216433559	same
chr11	64173487	64173887	-0.393510071	0.216656187	same
chr13	65783269	65783669	0.417804216	0.216777173	same
chr3	53196849	53197249	-0.369139061	0.216784214	same
chr5	88661094	88661494	-0.396322748	0.216787725	same
chrX	55717665	55718065	-0.378816815	0.216857327	same
chr16	667299	667699	-0.374980531	0.217162994	same
chr11	43680443	43680843	-0.335588842	0.217260979	same
chr10	87658908	87659308	-0.410384818	0.217275804	same
chr8	67342386	67342786	-0.424272664	0.217312019	same
chr2	181892325	181892725	-0.382949279	0.217343054	same
chr15	90973156	90973556	-0.417688097	0.2174063	same
chr2	68319547	68319947	-0.417080792	0.217473495	same
chr9	6015486	6015886	-0.398106936	0.217497891	same
chr17	41919342	41919742	-0.378378771	0.217546992	same
chr12	55738246	55738646	-0.36606017	0.217718553	same
chr8	53692901	53693301	-0.405168452	0.217830723	same
chrX	23907635	23908035	-0.401455138	0.217979893	same
chr1	27830602	27831002	-0.357525247	0.218153895	same
chr1	9942927	9943327	-0.394706591	0.218202224	same
chr19	53333561	53333961	-0.381796666	0.21827266	same
chr10	97418834	97419234	-0.397730798	0.218363192	same
chr17	8248693	8249093	-0.363103457	0.218397309	same
chrX	132022992	132023392	-0.377771966	0.218410602	same
chr10	80203659	80204059	-0.37233794	0.218420782	same
chr4	54091899	54092299	-0.422998979	0.218558901	same
chr5	75510611	75511011	-0.413487736	0.218617768	same
chr19	58409236	58409636	-0.359295966	0.218770579	same
chr4	138816821	138817221	-0.391985196	0.218849211	same
chr11	6481501	6481901	-0.365665658	0.218937028	same
chr1	52697972	52698372	-0.418764821	0.219018297	same
chr1	56764937	56765337	-0.422909416	0.219049856	same
chr20	46363985	46364385	-0.403912486	0.219172126	same
chr6	33711534	33711934	-0.399679153	0.219189817	same

chr7	38341218	38341618	-0.429656248	0.219246829	same
chr9	97238354	97238754	-0.372714194	0.219299579	same
chr20	50818446	50818846	-0.411475764	0.219311878	same
chr17	58447360	58447760	-0.384843156	0.219376548	same
chr6	155152294	155152694	-0.411511812	0.219506721	same
chr13	77751611	77752011	-0.372332926	0.219587487	same
chr9	88076252	88076652	-0.402085323	0.219720475	same
chr14	51989656	51990056	-0.420058908	0.219912359	same
chr11	72722292	72722692	-0.38005193	0.220078738	same
chr9	136354420	136354820	-0.353006676	0.220108948	same
chrX	154401191	154401591	-0.325334547	0.220341789	same
chr6	143511617	143512017	-0.375816479	0.220352324	same
chr1	171840928	171841328	-0.413162494	0.220566552	same
chr7	152859888	152860288	-0.395056496	0.220632106	same
chr14	73458533	73458933	-0.371751046	0.220719106	same
chr13	79225776	79226176	-0.375575146	0.220726353	same
chr18	33282518	33282918	0.424018339	0.220841543	same
chr5	317177	317577	-0.386127372	0.220857925	same
chr9	34126333	34126733	-0.388913864	0.220937597	same
chr7	155645022	155645422	-0.411177828	0.221095382	same
chr1	229272030	229272430	-0.400870577	0.221249163	same
chr17	74514259	74514659	-0.393603852	0.221291265	same
chr18	49021688	49022088	-0.422556567	0.221408002	same
chr11	62124304	62124704	-0.378752127	0.221428511	same
chr19	38596194	38596594	-0.384394774	0.221446952	same
chr20	32485132	32485532	-0.399841704	0.221462857	same
chr2	221309777	221310177	-0.418759456	0.221664384	same
chr6	5260151	5260551	-0.411656435	0.221850767	same
chr8	27837168	27837568	-0.412468675	0.221897965	same
chr1	26035554	26035954	-0.408702661	0.221975789	same
chr15	74800041	74800441	-0.355918617	0.22208852	same
chr22	46036050	46036450	-0.398633675	0.222174028	same
chr22	40711493	40711893	-0.406925547	0.222325791	same
chr2	218659035	218659435	-0.361892626	0.222358974	same
chr9	97232411	97232811	-0.412679548	0.222368562	same
chr14	104708723	104709123	-0.352925225	0.222439407	same
chr20	45972075	45972475	-0.384590195	0.22263635	same
chr5	159712779	159713179	-0.413049062	0.222697529	same
chr5	176416655	176417055	-0.409936964	0.222702209	same
chr14	105491701	105492101	-0.381826963	0.222703571	same
chr13	40770953	40771353	-0.381862022	0.222829915	same
chr9	114107805	114108205	-0.414922157	0.222835254	same
chr15	78860236	78860636	-0.364315389	0.222962173	same
chr20	462549	462949	-0.393093686	0.223089547	same
chr13	72023326	72023726	-0.416497206	0.223193268	same
chr16	58488019	58488419	-0.413159371	0.22321036	same
chr10	43420650	43421050	-0.400634034	0.223494629	same
chr11	108467253	108467653	-0.36115476	0.223926302	same
chr2	15939727	15940127	-0.419196126	0.223928004	same
chr19	3434938	3435338	-0.368116107	0.22405823	same
chr7	5073232	5073632	-0.412823719	0.224187061	same
chr1	84668074	84668474	-0.41632689	0.224197307	same
chr5	174520822	174521222	-0.409417584	0.224211207	same
chr5	145975532	145975932	-0.415824153	0.224261402	same
chr1	160401400	160401800	-0.370934006	0.224263857	same
chr4	107034084	107034484	-0.408140405	0.224309096	same
chr14	100476388	100476788	-0.409400411	0.224336181	same
chr1	28669413	28669813	-0.383419652	0.224396008	same
chr12	12813029	12813429	-0.378384563	0.224404786	same
chr2	200525996	200526396	-0.390184643	0.224460297	same
chr18	37537914	37538314	-0.385866996	0.224771847	same
chr20	45791556	45791956	-0.414063621	0.224775864	same
chr19	1855826	1856226	-0.385419378	0.224858068	same
chr2	85546148	85546548	-0.357913759	0.224895039	same
chr15	80152997	80153397	-0.369105634	0.225003014	same
chr1	225366638	225367038	-0.385222375	0.225186902	same
chr6	11270162	11270562	-0.405178015	0.225268618	same
chr16	47141772	47142172	-0.389395983	0.225306549	same
chr16	50741722	50742122	-0.368480459	0.225340488	same
chr1	89866149	89866549	-0.411716909	0.225580799	same
chr12	14394478	14394878	-0.39073858	0.225586733	same
chr6	75243495	75243895	-0.36873248	0.225676465	same
chr4	168585417	168585817	-0.407646013	0.225753862	same
chr3	111978713	111979113	-0.359030653	0.225915981	same
chr2	216012351	216012751	-0.402428032	0.22596621	same
chr3	93979943	93980343	-0.322371518	0.226041201	same

chr11	75562563	75562963	-0.349550203	0.226075328	same
chr15	74783037	74783437	-0.357838487	0.226215617	same
chr1	68342565	68342965	-0.398959374	0.226241795	same
chr9	93453182	93453582	-0.383042906	0.226305436	same
chr12	23950402	23950802	-0.406788568	0.226401205	same
chr13	62454786	62455186	-0.415108206	0.22651823	same
chr20	35875398	35875798	-0.3949934	0.226615163	same
chr10	100997805	100998205	-0.347619822	0.226761351	same
chr10	102461315	102461715	-0.386348324	0.22689373	same
chr6	152301833	152302233	-0.34586946	0.226941275	same
chr16	67846444	67846844	-0.328765399	0.227004071	same
chr6	43427420	43427820	-0.337987478	0.227061509	same
chr1	90831897	90832297	-0.418376349	0.227136676	same
chr12	65823254	65823654	-0.404762806	0.227182319	same
chr13	92719843	92720243	-0.401084896	0.227210514	same
chr2	206530307	206530707	-0.418563462	0.227220969	same
chr20	36199585	36199985	-0.384362779	0.227298479	same
chr19	5031360	5031760	-0.378397089	0.227373594	same
chrX	103214817	103215217	-0.380184951	0.227740491	same
chr10	27240236	27240636	-0.402121353	0.227754331	same
chr12	42588773	42589173	-0.411803258	0.227900616	same
chr6	52724484	52724884	-0.410904871	0.228092148	same
chr1	111592906	111593306	-0.411359904	0.228113432	same
chr1	51296700	51297100	-0.393680064	0.228172623	same
chr12	4273693	4274093	-0.38161037	0.228227715	same
chr12	7190026	7190426	-0.418248891	0.228422878	same
chr17	76711000	76711400	-0.382467838	0.22855321	same
chr2	181656778	181657178	-0.410559247	0.228776044	same
chr15	44663496	44663896	-0.376209659	0.228993436	same
chr22	21849874	21850274	-0.403137569	0.228997554	same
chr10	61897066	61897466	-0.378694028	0.229067322	same
chr10	101067435	101067835	-0.405964903	0.229067747	same
chr11	64284337	64284737	0.406976781	0.229082106	same
chr11	34174429	34174829	-0.37533688	0.229158241	same
chr10	75294517	75294917	0.41777607	0.229282647	same
chr7	43582551	43582951	-0.410585304	0.229339997	same
chr1	159829208	159829608	-0.37295522	0.22963839	same
chr3	114152525	114152925	-0.394831495	0.229751879	same
chr3	49554068	49554468	-0.389278911	0.230038002	same
chrX	102768982	102769382	-0.316020642	0.230070409	same
chr13	28718883	28719283	-0.392150595	0.230247412	same
chr2	203533690	203534090	-0.402809518	0.230248466	same
chr13	87124059	87124459	-0.313445728	0.230308066	same
chr1	64955334	64955734	-0.391998358	0.230374374	same
chr19	19430876	19431276	-0.375334627	0.230460722	same
chr17	67778968	67779368	-0.380478292	0.230723216	same
chr1	112358909	112359309	-0.404537238	0.230723582	same
chrX	110002067	110002467	-0.393747891	0.230738487	same
chr7	155408917	155409317	-0.417778151	0.230742505	same
chr13	91482321	91482721	-0.383956475	0.230978908	same
chr4	152352647	152353047	-0.372354802	0.23108147	same
chr19	21329064	21329464	-0.381408252	0.231100753	same
chr17	48908036	48908436	-0.371616771	0.231203592	same
chr19	40403836	40404236	-0.378057229	0.231271058	same
chr9	125200428	125200828	-0.320227844	0.231355606	same
chr2	105398673	105399073	-0.408890205	0.231440242	same
chr2	55048789	55049189	-0.370687609	0.23153363	same
chr17	40054446	40054846	-0.394495014	0.231641384	same
chr6	26305152	26305552	-0.372685823	0.231776031	same
chr19	45300703	45301103	-0.363169707	0.231870624	same
chr13	73056393	73056793	-0.385408247	0.232074425	same
chrX	119880774	119881174	-0.347811444	0.23208638	same
chr5	140700241	140700641	-0.360477937	0.232173731	same
chr7	149662652	149663052	-0.34866079	0.232232541	same
chr4	111883615	111884015	-0.413962266	0.232449791	same
chrX	85003520	85003920	-0.371300345	0.232465439	same
chr16	4668612	4669012	-0.379416954	0.232586258	same
chr22	28917768	28918168	-0.409507548	0.232663475	same
chr22	35399896	35400296	-0.333314226	0.232712108	same
chr4	153345036	153345436	-0.395442723	0.232752752	same
chr15	90351872	90352272	-0.362086126	0.232788649	same
chr2	99481957	99482357	-0.390062511	0.232827787	same
chr11	124740315	124740715	-0.381419693	0.232903249	same
chr20	18496766	18497166	-0.388271804	0.232941085	same
chr16	77722390	77722790	-0.400266935	0.232952053	same
chr1	234212859	234213259	-0.371098349	0.23310908	same

chr3	87227104	87227504	-0.360604554	0.233279402	same
chr6	100881091	100881491	-0.396122727	0.233474601	same
chr13	62512372	62512772	-0.358367642	0.233497619	same
chr5	169583648	169584048	-0.386137434	0.233667587	same
chr10	114881850	114882250	-0.412452973	0.233703827	same
chr2	177852003	177852403	-0.398648087	0.23381237	same
chr6	87589898	87590298	-0.347668802	0.233836004	same
chr15	40038880	40039280	-0.410225065	0.233846534	same
chr6	43933534	43933934	-0.396663289	0.23411881	same
chr1	32540283	32540683	-0.369945882	0.234414664	same
chr1	64823974	64824374	-0.405245473	0.234434779	same
chr12	110048275	110048675	-0.38145034	0.234493083	same
chr10	21502106	21502506	-0.415138171	0.234657984	same
chr3	140941815	140942215	-0.397312889	0.234731379	same
chr22	24259378	24259778	-0.408060148	0.234849732	same
chr11	66678032	66678432	-0.315440117	0.234863328	same
chr16	106929	107329	-0.404686941	0.234924193	same
chr6	27547435	27547835	-0.38218131	0.234970228	same
chr11	110098495	110098895	-0.385861163	0.235059081	same
chr11	435210	435610	-0.351410273	0.23506854	same
chr4	155376498	155376898	-0.34478152	0.23512133	same
chr1	75134447	75134847	-0.397485095	0.235122428	same
chrX	15790319	15790719	-0.395646361	0.235214469	same
chr1	219405611	219406011	-0.408235212	0.235247093	same
chr11	65418887	65419287	-0.299781459	0.235268665	same
chr22	17121484	17121884	-0.338765093	0.235323725	same
chr10	113385144	113385544	-0.393652488	0.235344093	same
chr7	1579592	1579992	-0.395870206	0.235350074	same
chr5	151758561	151758961	-0.365332153	0.23549495	same
chr20	49915478	49915878	-0.384393805	0.235563319	same
chr2	85418309	85418709	-0.409321634	0.235697498	same
chr17	8210296	8210696	-0.351922425	0.235958981	same
chr1	62082594	62082994	0.393463035	0.23598318	same
chr10	46106678	46107078	-0.397276853	0.236027832	same
chr20	44521786	44522186	-0.392128151	0.236246437	same
chr15	89630706	89631106	-0.366926132	0.236267898	same
chr8	102655199	102655599	-0.383481875	0.236347048	same
chr15	55196801	55197201	-0.381531218	0.236371405	same
chr1	7961361	7961761	-0.346668384	0.236405521	same
chr10	103661546	103661946	-0.402559577	0.236484174	same
chr22	42959945	42960345	-0.386946621	0.236491337	same
chr11	69294156	69294556	-0.402667847	0.236577257	same
chr22	21014138	21014538	-0.349349619	0.236621218	same
chr1	207050968	207051368	-0.365351514	0.236758004	same
chr3	72996397	72996797	-0.416003549	0.236805507	same
chr1	109493994	109494394	-0.338974439	0.236811945	same
chr19	8506138	8506538	-0.346615607	0.236895706	same
chr14	102524673	102525073	-0.327543331	0.236913997	same
chr2	206765316	206765716	-0.336243219	0.237052114	same
chr13	79973557	79973957	-0.385083465	0.23711706	same
chr19	10321700	10322100	-0.358593333	0.237205486	same
chr1	155187463	155187863	0.394014485	0.237546387	same
chr21	37700695	37701095	-0.391122725	0.237846451	same
chr8	42270428	42270828	-0.390013547	0.237883618	same
chr20	62875509	62875909	-0.374231475	0.237954051	same
chr11	66975865	66976265	-0.401408735	0.237997211	same
chr14	52832386	52832786	-0.385800196	0.238057098	same
chr22	41697267	41697667	-0.379588229	0.238093104	same
chr7	93591221	93591621	-0.364825086	0.238191946	same
chr15	78395368	78395768	-0.389338172	0.238277962	same
chr3	142828729	142829129	-0.405394775	0.238440704	same
chr4	26583892	26584292	-0.385678585	0.238738307	same
chr12	88580300	88580700	-0.361485805	0.238773221	same
chr11	67606519	67606919	-0.365577872	0.238789051	same
chr12	27523776	27524176	-0.341441172	0.238808548	same
chr16	31873816	31874216	-0.397954278	0.238919409	same
chr14	51581213	51581613	-0.397319327	0.238956833	same
chr2	80054122	80054522	-0.397405519	0.23897186	same
chr5	160399727	160400127	-0.356552288	0.239093221	same
chr12	53268197	53268597	-0.374451908	0.23912006	same
chr7	75994352	75994752	-0.389333501	0.239203813	same
chr5	92641294	92641694	-0.390012926	0.239300878	same
chr13	52455064	52455464	-0.366675237	0.239335796	same
chr15	85134557	85134957	-0.404601069	0.239437282	same
chr2	178413890	178414290	-0.364092008	0.23955945	same
chr17	17780969	17781369	-0.388098145	0.239802366	same

chr5	10601544	10601944	-0.400681593	0.240044015	same
chr4	7102966	7103366	-0.391558406	0.240065612	same
chr19	57559563	57559963	-0.39694796	0.240139974	same
chr16	71895042	71895442	-0.375984133	0.240160509	same
chr5	37717719	37718119	-0.410486974	0.240226856	same
chr15	38251509	38251909	-0.392088052	0.240597895	same
chr2	121902371	121902771	-0.37156996	0.240663494	same
chr2	3377831	3378231	-0.320354955	0.240763763	same
chr17	41801672	41802072	-0.349060882	0.240780837	same
chr1	45508401	45508801	-0.355822569	0.240896739	same
chr1	27914551	27914951	-0.324545974	0.241099894	same
chr7	116526221	116526621	-0.374538122	0.241234507	same
chr12	56688425	56688825	-0.334791784	0.241245054	same
chr2	96788696	96789096	-0.38369735	0.241660309	same
chr3	130893504	130893904	-0.41089587	0.24175444	same
chr15	65185184	65185584	-0.402055085	0.24191124	same
chr7	158647307	158647707	-0.398438991	0.24199644	same
chr1	81126971	81127371	-0.380957635	0.242008161	same
chr3	16885480	16885880	-0.3880977	0.242014492	same
chr15	76330207	76330607	-0.396610471	0.242068933	same
chr14	39114210	39114610	-0.354612483	0.242190997	same
chrUn_GL000195v1	137598	137998	-0.351912211	0.242314547	same
chr17	50094791	50095191	-0.382276753	0.242536052	same
chr7	32892312	32892712	-0.396378643	0.242536116	same
chr22	50756888	50757288	-0.365470911	0.242624995	same
chr3	13895368	13895768	-0.356571462	0.24280289	same
chr3	39152187	39152587	-0.322037729	0.242980902	same
chr4	39638847	39639247	-0.36616994	0.243171824	same
chr5	42908715	42909115	-0.374987318	0.243193791	same
chr20	16729845	16730245	-0.368141881	0.243259733	same
chr13	65843830	65844230	-0.368221261	0.243412529	same
chr9	36257883	36258283	-0.379346231	0.243588484	same
chr19	37370794	37371194	-0.370635182	0.243630419	same
chr20	50635910	50636310	-0.383134607	0.244093659	same
chr14	35413113	35413513	-0.402841261	0.244162768	same
chr11	108648136	108648536	-0.403873229	0.244258059	same
chr16	89240897	89241297	-0.335972799	0.244725969	same
chr12	66066322	66066722	-0.386198272	0.244871114	same
chr3	32392526	32392926	-0.399616825	0.244890959	same
chr6	27687874	27688274	-0.348823679	0.244933784	same
chr10	14878099	14878499	-0.346230074	0.245046384	same
chr12	123601627	123602027	-0.37525834	0.245178948	same
chr2	108448792	108449192	-0.339989627	0.245392201	same
chr16	8863814	8864214	-0.348201262	0.245506661	same
chr2	44044297	44044697	-0.393097816	0.245537079	same
chr12	122880752	122881152	-0.350782865	0.245593533	same
chr12	6326430	6326830	-0.375895921	0.245619389	same
chr12	123583150	123583550	-0.375208216	0.245904372	same
chr7	155008002	155008402	-0.389008557	0.246019106	same
chr3	51500146	51500546	-0.328825801	0.246054861	same
chr19	38976525	38976925	-0.38453058	0.246121779	same
chr3	72847709	72848109	-0.39830742	0.246303346	same
chr11	67464390	67464790	-0.367606659	0.246575681	same
chr18	23516004	23516404	-0.389510896	0.246630562	same
chr1	145425570	145425970	-0.360727001	0.246789464	same
chr2	231094437	231094837	-0.399137495	0.246929601	same
chr17	43913060	43913460	-0.359633597	0.24700339	same
chr6	27388618	27389018	-0.364715568	0.247109222	same
chr15	64461487	64461887	-0.37979874	0.247182745	same
chr2	24490247	24490647	-0.364243945	0.247283461	same
chr8	66613417	66613817	-0.39175105	0.24735595	same
chr13	87912501	87912901	-0.377503676	0.247389828	same
chr12	76558777	76559177	-0.382943334	0.247472562	same
chr8	15561643	15562043	-0.382016131	0.247492815	same
chr17	77140338	77140738	-0.331972162	0.247688376	same
chr17	2484670	2485070	-0.334235851	0.247847512	same
chr12	53299572	53299972	-0.369684336	0.247902693	same
chr2	728409	728809	-0.398676678	0.248641329	same
chr2	215619226	215619626	-0.390755518	0.248718728	same
chr21	45292107	45292507	-0.404579234	0.248773057	same
chrX	78139323	78139723	-0.385606989	0.248902933	same
chr1	234605969	234606369	-0.332439275	0.249012917	same
chrX	119482480	119482880	-0.371221314	0.249049515	same
chr17	79042632	79043032	-0.379657616	0.249052961	same
chr11	66312404	66312804	-0.405450962	0.249064233	same
chr22	31080845	31081245	-0.357705037	0.249109762	same

chr19	814664	815064	-0.319662483	0.249256848	same
chr12	116712674	116713074	-0.372667502	0.249344609	same
chr5	74640519	74640919	-0.369796734	0.249349017	same
chr2	75608806	75609206	-0.394106295	0.249554395	same
chr12	101279914	101280314	-0.365934988	0.249674106	same
chr14	54488428	54488828	-0.396857358	0.249766368	same
chr3	32239759	32240159	-0.358445552	0.249793663	same
chr11	26332081	26332481	-0.403582593	0.249794358	same
chr1	1746314	1746714	-0.387346094	0.2501021	same
chr6	112043387	112043787	-0.344665337	0.250264929	same
chr1	155989243	155989643	-0.356202178	0.250320398	same
chr5	172293340	172293740	-0.382250463	0.250693264	same
chr13	36346500	36346900	-0.385383372	0.250773207	same
chr20	35883942	35884342	-0.350747565	0.251140225	same
chr11	2434049	2434449	0.386629908	0.251249681	same
chr5	171441701	171442101	-0.378587691	0.251264211	same
chr2	240025424	240025824	-0.371767746	0.251280262	same
chr7	1183217	1183617	-0.397323297	0.251354295	same
chr5	913050	913450	-0.377897021	0.251377424	same
chr11	14499741	14500141	-0.386416345	0.251511406	same
chr21	43720254	43720654	-0.350718542	0.251620402	same
chr16	30355441	30355841	-0.290193796	0.251876594	same
chr1	181166693	181167093	-0.386727282	0.251895522	same
chr1	182604251	182604651	-0.299522925	0.252456705	same
chr6	1873010	1873410	-0.389279164	0.252563273	same
chr17	60079309	60079709	-0.388577497	0.252603714	same
chr3	12868202	12868602	-0.369884471	0.252619782	same
chr13	51080466	51080866	-0.390221501	0.253117825	same
chr16	29691762	29692162	-0.391013996	0.253230214	same
chr6	150753581	150753981	-0.34954107	0.253293222	same
chr19	8396738	8397138	-0.386622923	0.253309551	same
chr16	561940	562340	0.363065135	0.253350717	same
chr6	161891437	161891837	-0.384192967	0.253380196	same
chr10	120966174	120966574	-0.346915597	0.253449654	same
chr17	48107355	48107755	-0.332806063	0.253623553	same
chr13	66927105	66927505	-0.33319994	0.253720858	same
chrX	72277120	72277520	-0.343597605	0.253764683	same
chr1	59393278	59393678	-0.373388097	0.253845607	same
chr3	187139484	187139884	0.381421688	0.253957231	same
chr1	196608691	196609091	-0.300999747	0.254117276	same
chr21	45210818	45211218	0.392850674	0.254141361	same
chr13	78777720	78778120	-0.361657925	0.254457917	same
chr17	49408094	49408494	-0.392812404	0.254463171	same
chr11	47578644	47579044	-0.348171388	0.254674882	same
chr18	33670071	33670471	-0.346159834	0.254778091	same
chr16	67667991	67668391	-0.344629283	0.254838424	same
chr1	244047790	244048190	-0.328894784	0.254854474	same
chrX	47556128	47556528	-0.373407644	0.255095788	same
chr16	89374715	89375115	-0.323819061	0.255396593	same
chr9	96875699	96876099	-0.355316528	0.255536031	same
chr17	62423636	62424036	-0.352890974	0.25556303	same
chr19	47778355	47778755	-0.335634679	0.255597492	same
chr13	71703338	71703738	-0.393173874	0.255692562	same
chr17	29569087	29569487	-0.360929508	0.255693257	same
chrX	153599320	153599720	-0.3799168	0.255724742	same
chr1	155324330	155324730	-0.267838635	0.255746712	same
chr3	135965226	135965626	-0.362676386	0.255775568	same
chr11	74988546	74988946	-0.377925684	0.255932942	same
chr11	112961134	112961534	-0.369575081	0.255943604	same
chr1	172449753	172450153	-0.37141586	0.256243738	same
chr16	2238697	2239097	0.373403131	0.256487631	same
chr4	84966739	84967139	-0.374750483	0.256651796	same
chr19	48203823	48204223	-0.388772254	0.256865815	same
chr2	66079990	66080390	-0.389385074	0.257026905	same
chr11	59668170	59668570	-0.384315228	0.257037967	same
chr8	134713223	134713623	-0.387978795	0.257102809	same
chr3	43622086	43622486	-0.331068254	0.25716872	same
chr6	117675078	117675478	-0.300299388	0.257186463	same
chr12	120437827	120438227	-0.311430665	0.257326821	same
chr9	899739	900139	-0.382383877	0.257385592	same
chr6	43369667	43370416	-0.348857627	0.257528456	same
chr10	104274793	104275193	-0.373821133	0.257568369	same
chr6	27251156	27251556	-0.366014856	0.257573383	same
chr2	227862884	227863284	-0.38993521	0.257892758	same
chr4	176319499	176319899	-0.390447857	0.257917984	same
chr16	4616003	4616403	-0.389023168	0.257982619	same

chr14	33393665	33394065	-0.385941659	0.258163502	same
chr1	145824094	145824494	-0.37454919	0.258299527	same
chr8	41529140	41529540	-0.373839014	0.258342739	same
chr10	95655874	95656274	-0.376045234	0.258381626	same
chr13	23888945	23889345	-0.369727768	0.258396731	same
chr6	16964768	16965346	-0.379710305	0.258474753	same
chr17	42744737	42745137	-0.38153755	0.258633414	same
chr11	61430059	61430459	-0.362349154	0.258773156	same
chr22	29907894	29908294	-0.350244733	0.258785933	same
chr19	55643143	55643543	-0.359523211	0.259031093	same
chr1	227953756	227954156	-0.322401454	0.259109444	same
chr12	92785161	92785561	-0.383338232	0.259127254	same
chr16	86555162	86555562	-0.375771965	0.25921253	same
chrX	70434113	70434513	-0.359744486	0.259412402	same
chr7	69781304	69781704	-0.367654621	0.259414129	same
chr1	205787013	205787413	-0.390302889	0.259643394	same
chr17	69414589	69414989	-0.381358631	0.259688327	same
chr2	233353644	233354044	-0.38338744	0.259877991	same
chr11	57340430	57340830	-0.379223112	0.259966643	same
chr16	56608532	56608932	0.385965158	0.260209288	same
chrX	73828800	73829200	-0.373929815	0.260231171	same
chr9	114348818	114349218	-0.376323377	0.260357134	same
chr17	14622648	14623048	-0.391201456	0.260468465	same
chr6	142301800	142302200	-0.381886702	0.260511492	same
chr10	72173835	72174235	-0.344693049	0.260562638	same
chr10	5684429	5684829	-0.3548001	0.260564421	same
chr17	47949155	47949555	-0.3506879	0.260707629	same
chr1	65527373	65527773	-0.385068544	0.260800589	same
chr1	156368059	156368459	0.364437425	0.260803144	same
chr6	28443138	28443538	-0.362926418	0.26084254	same
chr12	120317430	120317830	-0.308307286	0.260945184	same
chr12	104219660	104220060	-0.373526819	0.261065924	same
chrX	20141770	20142170	-0.342940387	0.261090341	same
chr2	44955800	44956200	-0.384826741	0.261111168	same
chr10	120851206	120851606	-0.32179538	0.261123117	same
chr1	161021047	161021447	-0.349682146	0.261222551	same
chr22	38271981	38272381	-0.333738192	0.261329495	same
chr7	80919284	80919684	-0.389359842	0.261432662	same
chr3	194003479	194003879	-0.37173994	0.261591171	same
chr6	26473886	26474286	-0.330340641	0.261767917	same
chr6	34196297	34196697	-0.373900441	0.26178691	same
chr1	229549994	229550394	-0.362929468	0.26216115	same
chr1	241519640	241520040	-0.3902912	0.262229891	same
chr1	58162906	58163306	-0.361457266	0.262249894	same
chr16	56931989	56932389	-0.321524741	0.262263315	same
chr8	38031000	38031400	-0.363873211	0.262370743	same
chr6	137866374	137866774	-0.36566877	0.262421307	same
chr2	120879792	120880192	-0.371680535	0.262448108	same
chr1	201154117	201154517	-0.336888686	0.262652188	same
chr12	122952389	122952789	-0.36705834	0.262687615	same
chr6	118800627	118801027	-0.375606198	0.262736753	same
chr19	6278852	6279252	-0.384693086	0.262964682	same
chr19	928009	928409	-0.374154056	0.263016048	same
chrX	54841226	54841626	-0.373990083	0.263168495	same
chr2	32039722	32040122	-0.364283207	0.263227284	same
chr18	36572153	36572553	-0.37699478	0.263264871	same
chr17	56834631	56835031	-0.368508284	0.263307358	same
chr9	128552094	128552494	-0.369800248	0.263343682	same
chr1	204431408	204431808	-0.382433797	0.263400339	same
chr1	58815587	58815987	-0.379832417	0.263548476	same
chr6	123841338	123841738	-0.334139579	0.263599007	same
chr5	139955555	139955955	-0.365410016	0.263647889	same
chr19	8321234	8321634	-0.313903566	0.263662221	same
chr12	110403726	110404126	-0.328325915	0.263766373	same
chr8	68325168	68325568	-0.381482843	0.2640086	same
chr17	61927462	61927862	-0.317219131	0.264067515	same
chr6	151325212	151325612	-0.382311668	0.264200901	same
chr19	6753671	6754071	-0.367723362	0.264320775	same
chr4	13654205	13654605	-0.372424245	0.264666547	same
chr16	12260664	12261064	-0.378403414	0.265046645	same
chr11	62771215	62771615	-0.336278159	0.265093423	same
chr1	231627289	231627689	-0.320284295	0.265220152	same
chr6	41787191	41787591	-0.280490279	0.265982186	same
chr9	129103153	129103553	-0.346418607	0.266023914	same
chr11	47415518	47415918	-0.33731929	0.266067673	same
chr20	35664785	35665185	-0.315358797	0.266072683	same

chr3	48919553	48919953	-0.36349945	0.266077624	same
chr17	81683636	81684036	-0.304160287	0.266115867	same
chr6	70668217	70668617	-0.373989922	0.266161345	same
chr2	121596532	121596932	-0.364525676	0.266164937	same
chr17	81601422	81601822	-0.361334259	0.266197659	same
chr1	151195907	151196307	-0.354617247	0.266275282	same
chr7	92448046	92448446	-0.37917885	0.266311829	same
chr7	140913211	140913611	-0.377623213	0.266361034	same
chr1	226095525	226095925	-0.343966571	0.266404129	same
chr8	60672542	60672942	-0.378839815	0.266408282	same
chr17	8288393	8288793	-0.322838153	0.266439361	same
chr16	28974332	28974732	-0.357476742	0.266460006	same
chr1	110035106	110035506	-0.346936715	0.266493782	same
chr17	74430558	74430958	-0.380887448	0.266643212	same
chr6	100464692	100465092	-0.366795965	0.266653715	same
chr7	106009572	106009972	-0.370472563	0.266659331	same
chr7	87933788	87934188	-0.386000426	0.266815563	same
chr20	23351163	23351563	-0.360679781	0.26681575	same
chr1	32886667	32887067	-0.35574511	0.266843352	same
chr2	55268995	55269395	-0.34227413	0.266950883	same
chr18	37564986	37565386	-0.373026858	0.267067723	same
chr18	48952535	48952935	-0.346044231	0.267574657	same
chr6	143745042	143745442	-0.357174648	0.267640535	same
chr17	3935183	3935583	-0.365851181	0.267696885	same
chr1	148263217	148263617	-0.306943415	0.267721615	same
chr17	1515595	1515995	-0.346954414	0.267722907	same
chr17	57956572	57956972	-0.353702265	0.267780876	same
chr17	67585129	67585529	-0.380417742	0.267808181	same
chr1	33127262	33127662	-0.304434574	0.267871568	same
chr3	40309450	40309850	-0.326647953	0.268051692	same
chr1	167658909	167659309	-0.380862611	0.268124258	same
chr12	121295638	121296038	-0.337145597	0.268349623	same
chr7	140541594	140541994	-0.367247277	0.268453162	same
chr7	134891325	134891725	-0.318804501	0.268823142	same
chrX	110145829	110146229	-0.360594825	0.269018729	same
chr15	83446908	83447308	-0.34744802	0.269225047	same
chr8	94120229	94120629	-0.373860249	0.26951607	same
chr6	4020951	4021351	-0.345972105	0.269606918	same
chr14	68191566	68191966	-0.369987908	0.269612019	same
chr20	17682507	17682907	-0.358764852	0.269667987	same
chr18	28177236	28177636	-0.388434328	0.270025953	same
chr3	112086329	112086729	-0.315739543	0.270063724	same
chr20	63980705	63981105	-0.317319153	0.270251716	same
chr20	3159506	3159906	-0.364874555	0.27035707	same
chr6	27176669	27177069	-0.381859301	0.27058229	same
chr1	26921600	26922000	-0.36695798	0.270601085	same
chr17	3913795	3914195	-0.343842194	0.270814172	same
chr19	17419648	17420048	-0.363813399	0.270858233	same
chr1	27858181	27858581	-0.356732782	0.270935875	same
chr16	29290852	29291252	-0.352185271	0.271081504	same
chr7	149803776	149804176	-0.341750581	0.271168194	same
chr7	129825538	129825938	-0.378315784	0.271244179	same
chr6	52670874	52671274	-0.361074114	0.271434564	same
chr1	110407432	110407832	-0.347793596	0.271438664	same
chr4	19752718	19753118	-0.377744801	0.271498438	same
chr1	119675430	119675830	-0.365957654	0.271593688	same
chr19	37172659	37173059	-0.374454675	0.271631095	same
chr1	148345623	148346023	0.36999548	0.271644493	same
chr19	47289577	47289977	-0.331896976	0.27172719	same
chr1	10210132	10210532	-0.357010961	0.271835248	same
chr12	69585424	69585824	-0.335548902	0.271916204	same
chr1	205141518	205141918	-0.354970999	0.27194362	same
chr20	21125888	21126288	-0.386154536	0.272063217	same
chr1	231590536	231590936	-0.358372502	0.272090248	same
chr7	50721800	50722200	-0.368962039	0.272274867	same
chr12	132615038	132615438	-0.323259259	0.272302559	same
chr1	90718282	90718682	-0.362070784	0.272497728	same
chr10	97196548	97196948	-0.372546998	0.272871838	same
chr5	122774964	122775364	-0.379286759	0.273176217	same
chrX	102900383	102900783	-0.321152252	0.27319307	same
chr20	2245831	2246231	-0.376680232	0.273344689	same
chr17	66191328	66191728	-0.349888549	0.273395141	same
chr18	34222812	34223212	-0.341168715	0.273504135	same
chr11	4182030	4182430	-0.352059248	0.27367274	same
chr19	16472068	16472468	-0.349283576	0.273788738	same
chr1	33633195	33633595	-0.343668357	0.273886596	same

chr19	43652144	43652544	-0.363394144	0.274009696	same
chr5	116084428	116084828	-0.377498474	0.27425521	same
chr7	50450106	50450506	-0.360270005	0.274380263	same
chr1	230310457	230310857	-0.362780577	0.274432835	same
chr19	50909202	50909602	-0.372752194	0.274501143	same
chr4	118591659	118592059	-0.351720989	0.274516417	same
chr6	148746277	148746677	-0.337876261	0.274652464	same
chr17	75091722	75092122	-0.338242918	0.274674123	same
chr5	179804110	179804510	-0.335732131	0.274900435	same
chr17	48970105	48970505	-0.371544525	0.274911341	same
chr22	19122530	19122930	-0.345912789	0.275092669	same
chr15	89971466	89971866	-0.339552513	0.275178421	same
chr10	56361230	56361630	-0.347857694	0.275184046	same
chr2	231514270	231514670	-0.325708506	0.275222224	same
chr3	123652394	123652794	-0.375967848	0.275296453	same
chr1	11262449	11262849	-0.361223287	0.275337567	same
chr3	186783457	186783857	-0.373369709	0.275444899	same
chr12	85317921	85318321	-0.357170386	0.275607724	same
chr9	33676662	33677062	-0.378393787	0.275738638	same
chr15	40323127	40323527	-0.357721746	0.275855652	same
chr19	48738522	48738922	-0.359355083	0.275873366	same
chr2	218909015	218909415	-0.318818457	0.275993189	same
chr4	89056905	89057305	-0.362307934	0.276016628	same
chr8	94553253	94553653	-0.315465887	0.276121166	same
chr19	44164736	44165136	-0.329537523	0.276211978	same
chr14	55411723	55412123	-0.344952575	0.276258963	same
chr19	48809629	48810029	-0.352320887	0.276324739	same
chr12	132500566	132500966	-0.307360665	0.276416818	same
chr19	1232449	1232849	-0.338152012	0.276553941	same
chr9	136306400	136306800	-0.373497034	0.27664852	same
chr1	116911412	116911812	-0.363957557	0.276671359	same
chr6	93088777	93089177	-0.345861276	0.276709553	same
chr16	88452645	88453045	-0.332177325	0.276715201	same
chr10	72260536	72260936	-0.324544759	0.276809472	same
chr11	13873373	13873773	-0.360632643	0.276845786	same
chr12	55966652	55967052	-0.318853137	0.276965582	same
chrX	153689746	153690146	-0.320000905	0.276968645	same
chr15	101501807	101502207	-0.302461888	0.277003495	same
chr8	11467385	11467785	-0.369418205	0.277203167	same
chrX	107118563	107118963	-0.352147387	0.277458265	same
chrX	49163937	49164337	-0.348859789	0.27752032	same
chr7	73564230	73564630	-0.372966411	0.277542723	same
chr6	104958627	104959027	-0.37441892	0.277548383	same
chr4	80197773	80198173	-0.370493831	0.277908378	same
chr8	125152468	125152868	-0.376892731	0.27801559	same
chr20	59032382	59032782	-0.352889013	0.278089574	same
chr8	78444962	78445362	-0.345840715	0.278095947	same
chr5	86617904	86618304	-0.366720325	0.278248641	same
chr5	132491316	132491716	-0.371410803	0.278295237	same
chr13	63636336	63636736	-0.352377317	0.278335224	same
chr2	46616718	46617118	-0.320377335	0.278491078	same
chr12	15688348	15688748	-0.350635252	0.278493818	same
chr6	133502888	133503288	-0.373196861	0.278618373	same
chr11	72698707	72699107	0.363196732	0.278865034	same
chr10	73358458	73358858	-0.360283053	0.279063075	same
chr15	70111284	70111684	-0.361258771	0.27942806	same
chr17	42798475	42798875	-0.338010818	0.279453447	same
chr17	42677590	42677990	-0.307746127	0.279495374	same
chr5	36066716	36067116	-0.354918742	0.279517191	same
chr19	2097825	2098225	0.326184268	0.279521438	same
chr3	45995560	45995960	-0.307223129	0.279555527	same
chr16	53503239	53503639	-0.376811094	0.279681385	same
chr3	48500901	48501301	-0.338266282	0.280263551	same
chr19	2977022	2977422	-0.359971524	0.280388387	same
chr1	203440825	203441225	-0.363564241	0.280593536	same
chr1	225426635	225427035	-0.312900625	0.28094579	same
chr7	157340028	157340428	-0.32041609	0.28103237	same
chr19	40690764	40691164	-0.331992644	0.281147764	same
chrX	53281189	53281589	-0.351640283	0.281208024	same
chr15	98650432	98650832	-0.359998101	0.281233863	same
chr3	14548125	14548525	0.350635964	0.28128465	same
chr15	26082153	26082553	-0.361868412	0.281365693	same
chr2	100004935	100005335	-0.365297567	0.281417551	same
chrX	115690904	115691304	-0.365438332	0.281483013	same
chr16	398062	398462	-0.337631153	0.281573053	same
chr3	71226768	71227168	-0.371380389	0.281682939	same

chr14	95157519	95157919	-0.339420575	0.281924703	same
chr11	103091937	103092337	-0.328748924	0.281955632	same
chr18	57630123	57630523	-0.367175336	0.28196418	same
chr4	103019541	103019941	-0.33962693	0.282056926	same
chr8	98865923	98866323	-0.355580221	0.282070438	same
chr13	65774736	65775136	0.355175438	0.282157354	same
chr6	43773244	43773644	-0.317677647	0.282331813	same
chr1	8439159	8439559	-0.377238904	0.282344408	same
chr12	54288873	54289273	-0.336793958	0.282441261	same
chr7	100158715	100159115	-0.365926118	0.282625737	same
chr5	133968228	133968628	-0.341597598	0.282665422	same
chr1	28232582	28232982	-0.331154126	0.282669688	same
chr1	72283799	72284199	-0.350630408	0.282670389	same
chr11	10669450	10669850	-0.361796792	0.28278788	same
chr12	51218391	51218791	0.365327929	0.282812946	same
chr1	79044974	79045374	-0.347075032	0.282831859	same
chr14	24108659	24109059	-0.363551588	0.282833446	same
chr6	142071789	142072189	-0.371945959	0.282834651	same
chr1	112705926	112706326	-0.31182179	0.283100944	same
chr4	155667935	155668335	-0.371683878	0.283134261	same
chr2	195694591	195694991	0.37023624	0.283251867	same
chr19	990066	990466	-0.323642061	0.283374956	same
chr19	36128027	36128427	-0.354537696	0.283467627	same
chr12	9648049	9648449	-0.344747167	0.283517336	same
chr4	82284851	82285251	-0.319747779	0.283610333	same
chr5	138493407	138493807	0.332147604	0.283681011	same
chr6	27091796	27092196	-0.363683764	0.283681164	same
chrX	119943421	119943821	-0.289906952	0.283797914	same
chr9	121286972	121287372	0.357802705	0.283803526	same
chr12	119804253	119804653	0.359692445	0.283974158	same
chr1	23980328	23980728	-0.321427464	0.283992572	same
chr6	136644242	136644642	-0.333488723	0.284231048	same
chr12	8915018	8915418	-0.353048834	0.284366263	same
chr3	33645195	33645595	0.360648304	0.284426472	same
chr6	53061610	53062010	-0.321991747	0.284635768	same
chr9	101486258	101486658	-0.315002728	0.284697803	same
chr7	79453868	79454268	-0.368322846	0.284725166	same
chr18	51029810	51030210	-0.361027399	0.284795489	same
chr12	20368188	20368588	-0.363620603	0.284916069	same
chr5	67004257	67004657	-0.363086249	0.284989384	same
chr13	40921465	40921865	-0.34423117	0.285025168	same
chr20	43657365	43657765	-0.312602222	0.285078903	same
chr1	59759241	59759641	-0.346226869	0.285156016	same
chr20	1118629	1119029	-0.361212456	0.285351672	same
chr6	26188727	26189127	-0.289766226	0.285380858	same
chr16	89453920	89454320	-0.346600733	0.285664935	same
chr14	69574551	69574951	-0.370853009	0.285668374	same
chr3	173395386	173395786	-0.32529051	0.28568925	same
chr1	161314246	161314646	-0.310139857	0.285768211	same
chr5	32329445	32329845	-0.35922835	0.286051714	same
chr9	133478335	133478735	-0.358756435	0.286139365	same
chr2	230762211	230762611	-0.366976245	0.286141444	same
chr4	98260850	98261250	-0.335903884	0.286262612	same
chrX	130304971	130305371	-0.360828359	0.286396552	same
chr9	75087770	75088170	-0.345593861	0.286398516	same
chr5	27472039	27472439	-0.359775996	0.286412833	same
chr2	218963003	218963403	-0.354985071	0.28659374	same
chr5	79508453	79508853	-0.350840761	0.28670677	same
chrX	80810020	80810420	-0.359596317	0.286753912	same
chr6	28491517	28491917	-0.362083819	0.287064064	same
chr10	14185588	14185988	-0.341829584	0.287110247	same
chr12	66169523	66169923	-0.303770543	0.28713483	same
chr9_KI270718v1_random	37569	37969	-0.327115338	0.287241562	same
chrX	106966519	106966919	-0.347429255	0.287343261	same
chr2	72862482	72862882	-0.357523869	0.287455932	same
chr1	93909701	93910101	-0.353006831	0.28756375	same
chr11	119084822	119085222	-0.29393708	0.287621804	same
chr11	122580888	122581288	-0.343693682	0.287787963	same
chr1	116374795	116375195	-0.325208185	0.28784918	same
chr2	212191226	212191626	-0.347109104	0.287931664	same
chr9	16705133	16705533	-0.360008497	0.287973215	same
chrX	131633406	131633806	-0.360814944	0.288198751	same
chr3	61560984	61561384	-0.349499657	0.28833164	same
chr15	69161738	69162138	-0.34096338	0.288343368	same
chr10	102835055	102835455	-0.350210765	0.288471196	same
chr10	132164788	132165188	-0.343013066	0.288528205	same

chr19	51887724	51888124	-0.353659122	0.288552629	same
chr7	128783307	128783707	-0.323985243	0.288585204	same
chr10	118341903	118342303	-0.350003687	0.288631155	same
chr12	56257364	56257764	-0.331730649	0.288818928	same
chr6	122610774	122611174	-0.342357648	0.289017033	same
chr3	10263974	10264374	-0.356518671	0.289150323	same
chr1	229149015	229149415	-0.26627207	0.289178889	same
chr11	119533942	119534342	-0.342877937	0.289329535	same
chr5	132963604	132964004	-0.371574035	0.289340202	same
chr2	53587117	53587517	-0.350289446	0.289670978	same
chr2	65435607	65436007	-0.302391492	0.28977318	same
chr13	84126889	84127660	0.343782144	0.28980972	same
chr2	23926212	23926612	-0.341294403	0.28997936	same
chr17	68436003	68436403	-0.349056897	0.290108046	same
chr3	42653543	42653943	-0.338780777	0.290139811	same
chr3	121111766	121112166	-0.363868998	0.290199038	same
chr7	1067820	1068220	-0.360285819	0.290218126	same
chr1	161524111	161524511	-0.348137597	0.29024114	same
chr16	31508008	31508408	-0.344942673	0.290391614	same
chr13	65818831	65819231	-0.339319743	0.290441309	same
chr4	55397139	55397539	-0.354182447	0.290560932	same
chr17	75368213	75368613	-0.320361622	0.290645896	same
chr10	110304892	110305292	-0.358932334	0.290802712	same
chr7	33722556	33722956	-0.336916304	0.290938058	same
chr1	99264356	99264756	-0.345922981	0.290968019	same
chr12	72520090	72520490	-0.350016796	0.291137036	same
chr4	87220502	87220902	-0.334449398	0.291251166	same
chr1	212284969	212285369	-0.267640691	0.291500402	same
chr7	87876208	87876608	-0.324962856	0.291581936	same
chr1	145979216	145979616	-0.322988017	0.291656941	same
chr21	34887884	34888284	-0.343566117	0.291670657	same
chr12	50075262	50075662	-0.33017325	0.29167266	same
chr8	90645074	90645474	-0.354984394	0.291689464	same
chr21	42840610	42841010	-0.277917715	0.291927322	same
chr18	597011	597411	-0.362573167	0.292373829	same
chr19	16584777	16585177	-0.352815821	0.292570222	same
chr1	31394768	31395168	-0.343852167	0.292638629	same
chr10	114526612	114527012	-0.333653295	0.292850706	same
chr6	138971658	138972058	-0.35887584	0.292894544	same
chr15	74489661	74490061	-0.344298686	0.292960336	same
chr5	141960613	141961013	-0.35967259	0.292973777	same
chr17	28903066	28903466	-0.298068425	0.292995323	same
chr15	99123616	99124016	-0.301444576	0.293296181	same
chr5	174419152	174419552	-0.356105476	0.29354795	same
chr5	180213418	180213818	-0.330881839	0.293593767	same
chr19	57947424	57947824	-0.357933356	0.293628837	same
chr2	58973881	58974281	-0.352489463	0.294001122	same
chr9	100411690	100412090	-0.341157554	0.294049354	same
chr20	62873783	62874183	-0.335995059	0.29411075	same
chr18	32724232	32724632	0.324637767	0.294175785	same
chr17	63675825	63676225	-0.314861147	0.294286878	same
chr8	140791042	140791442	-0.342363147	0.294293928	same
chr1	26531137	26531537	-0.33402321	0.294497178	same
chr3	15738288	15738688	-0.327248238	0.294915222	same
chr9	5886216	5886616	-0.342365657	0.294921535	same
chr7	6706931	6707331	-0.341454069	0.294982019	same
chr6	35687747	35688147	-0.344617588	0.295035123	same
chr14	24179454	24179854	-0.316399479	0.295381302	same
chr3	37862676	37863076	-0.324318019	0.295453445	same
chr9	83956406	83956806	-0.316316792	0.295492283	same
chr1	15616863	15617263	-0.358077297	0.2955005	same
chr17	7915466	7915866	-0.324823447	0.295635027	same
chr3	129383230	129383630	-0.327485528	0.295661429	same
chr19	55178903	55179303	-0.300442255	0.29575245	same
chr1	84505441	84505841	-0.361725003	0.295845432	same
chr1	6205722	6206122	-0.321029887	0.295921063	same
chr16	67393179	67393579	-0.350803804	0.29597894	same
chr13	32031286	32031686	-0.364743301	0.296112243	same
chrX	18425031	18425431	-0.343404426	0.296125407	same
chr7	133252971	133253371	-0.34135531	0.29615756	same
chr19	6633124	6633524	-0.358589047	0.296240918	same
chr4	139243662	139244062	-0.356437555	0.296491916	same
chr3	155744838	155745238	-0.273881691	0.297233494	same
chr3	9769210	9769610	-0.341347027	0.297251295	same
chr18	22346654	22347054	-0.356208126	0.297288206	same
chr11	118907253	118907653	-0.347357576	0.297415633	same

chr2	127591347	127591747	-0.341509955	0.29745224	same
chr12	121388578	121388978	-0.345850034	0.297460757	same
chr10	68153645	68154045	-0.322771503	0.297492349	same
chr19	10112974	10113374	-0.28662251	0.297536843	same
chr12	121297261	121297661	-0.350684974	0.297612333	same
chr12	112116440	112116840	-0.321919123	0.297612471	same
chr20	35771600	35772000	-0.359646121	0.297628861	same
chr22	31749875	31750275	-0.302830697	0.297941917	same
chr4	110636724	110637124	-0.354139529	0.298095518	same
chr7	38345625	38346025	-0.353191826	0.298287421	same
chrX	103533704	103534104	-0.342171614	0.298338578	same
chr8	37453014	37453414	-0.326913671	0.298502989	same
chr1	241855233	241855633	-0.320397494	0.298548127	same
chr1	231049407	231049807	-0.356076312	0.298633541	same
chr9	98796586	98796986	-0.353122793	0.298634859	same
chr1	1906328	1906728	-0.345224041	0.298827759	same
chr11	65477540	65477940	-0.336025783	0.298985656	same
chr11	123654570	123654970	-0.324307465	0.298999313	same
chr19	8578878	8579278	-0.359327912	0.299097587	same
chr2	85679103	85679503	-0.343072059	0.299116104	same
chr1	101236325	101236725	-0.312567772	0.299200601	same
chr4	1693454	1693854	-0.327473157	0.29926221	same
chr16	21502214	21502614	-0.343282874	0.299299288	same
chr12	49132034	49132434	-0.315246068	0.299373228	same
chr1	20661512	20661912	-0.311198829	0.29943014	same
chr15	89758305	89758705	0.357996628	0.299562195	same
chr10	5888551	5888951	-0.321738672	0.299612473	same
chr6	137705302	137705702	-0.344772659	0.299697575	same
chr11	35821971	35822371	-0.331633524	0.299790384	same
chr14	81533711	81534111	-0.318512456	0.299871477	same
chr19	17998504	17998904	-0.356653028	0.2999094	same
chr1	161566014	161566414	-0.339144813	0.299913219	same
chr6	149060375	149060775	-0.333058134	0.299914326	same
chr5	132007978	132008378	-0.352077713	0.300105562	same
chr6	87328830	87329230	-0.358501321	0.300189302	same
chr4	8267114	8267514	-0.323684025	0.300296112	same
chr7	105581169	105581569	-0.316910739	0.300341015	same
chr3	143973242	143973642	-0.355349111	0.300391168	same
chr1	185170268	185170668	-0.347446235	0.300549449	same
chr12	132275060	132275460	-0.281576442	0.300750028	same
chr11	10750873	10751273	-0.303772562	0.300769904	same
chr6	73513915	73514315	-0.274896849	0.301077314	same
chrX	119693137	119693537	-0.312942257	0.30110921	same
chr14	75002608	75003008	-0.298762377	0.301144148	same
chr13	78066092	78066492	-0.357850599	0.301310788	same
chr6	27020070	27020470	-0.318331805	0.301607201	same
chr2	170928819	170929219	-0.345407586	0.301607272	same
chr9	127771197	127771597	-0.295502123	0.30164248	same
chr11	78416640	78417040	-0.316014863	0.302065091	same
chr20	52104638	52105038	-0.359876702	0.302125055	same
chr1	85714394	85714794	0.345340897	0.302745922	same
chr9	109440801	109441201	-0.342616789	0.30291776	same
chr3	107927655	107928055	-0.327989732	0.303133075	same
chr7	44019139	44019539	-0.321587325	0.303205054	same
chr1	201720240	201720640	-0.331424344	0.303261095	same
chr1	42462921	42463321	-0.335962881	0.303725295	same
chr4	47914288	47914688	-0.325238584	0.303933909	same
chr7	98294868	98295268	-0.339124102	0.304160945	same
chr1	235845767	235846167	-0.33807687	0.304205694	same
chr19	19732923	19733323	-0.335432868	0.304299213	same
chr12	27968964	27969364	-0.345384953	0.304322612	same
chr14	77615728	77616128	-0.358759142	0.30438785	same
chr15	40564813	40565213	-0.313449489	0.304389608	same
chr1	240611874	240612274	-0.311595694	0.304403394	same
chr4_GL000008v2_random	2556	2956	0.322921871	0.304556312	same
chr12	54052471	54052871	-0.316419125	0.304634046	same
chr11	107565180	107565580	-0.34113273	0.30483357	same
chr10	112183674	112184074	-0.342951764	0.305034803	same
chr1	156591900	156592300	-0.321892553	0.305211209	same
chr21	33818000	33818400	-0.325886782	0.305249081	same
chr9	133121648	133122048	-0.332822624	0.305274749	same
chr4	6640393	6640793	-0.307693983	0.305498275	same
chr5	139341712	139342112	-0.266003763	0.305636027	same
chr14	52791776	52792176	-0.347098906	0.305683555	same
chr2	139529426	139529826	-0.35619582	0.305802584	same
chr12	69359559	69359959	-0.309571747	0.306017164	same

chr1	67367847	67368247	-0.353295464	0.306143027	same
chr12	101407596	101407996	-0.330829737	0.306156223	same
chr12	121713646	121714046	-0.301036869	0.306179684	same
chr4	176238443	176238843	-0.337743116	0.306272128	same
chr1	229159745	229160145	0.333623191	0.306389211	same
chr3	42497028	42497428	-0.293854206	0.306396398	same
chr13	66164934	66165334	-0.325668578	0.30658287	same
chr22	20206057	20206457	-0.298650557	0.306799202	same
chr2	77593028	77593428	-0.355197157	0.306830449	same
chr6	3455450	3455850	-0.337589568	0.306846349	same
chr1	178542039	178542439	-0.324514709	0.306923318	same
chr22	43955263	43955663	-0.313501172	0.306979267	same
chr11	95923912	95924312	-0.324581355	0.307154599	same
chr7	155745647	155746047	-0.342389146	0.307271771	same
chr11	62761485	62761885	-0.356965834	0.307312336	same
chr6	107459421	107459821	-0.298570234	0.307354675	same
chr8	74599784	74600184	0.34406703	0.307512531	same
chr4	84298821	84299221	-0.347547293	0.307595425	same
chr10	96513835	96514235	-0.360128422	0.307631017	same
chr2	38742712	38743112	-0.316371147	0.307688017	same
chr6	35342083	35342483	-0.344095631	0.307787429	same
chr2	27217678	27218078	-0.325079542	0.30783149	same
chr5	181160217	181160617	-0.307429287	0.307940689	same
chr2	64873594	64873994	-0.351691853	0.30828635	same
chr9	81371011	81371411	-0.341435742	0.308424159	same
chr7	100450589	100450989	-0.311717745	0.308476666	same
chr4	70902802	70903202	-0.340220047	0.308610501	same
chr3	185363011	185363411	-0.353840189	0.308876964	same
chr6	26312356	26312756	-0.293559411	0.309089089	same
chr6	10838082	10838482	-0.325431983	0.309430585	same
chr1	61213548	61213948	-0.335943552	0.309638395	same
chrX	104310058	104310458	-0.318632419	0.309729898	same
chr2	67397432	67397832	-0.34775046	0.309800932	same
chr2	108620881	108621281	-0.350240351	0.310142558	same
chr12	7960522	7960922	-0.344522206	0.310158094	same
chr3	10873482	10873882	0.324906996	0.310173035	same
chr1	174040628	174041028	-0.349548538	0.310217685	same
chr7	155263563	155263963	0.330929837	0.310336028	same
chr16	67428164	67428564	-0.298474839	0.310365383	same
chr13	79423758	79424158	-0.341770236	0.310424951	same
chr10	119872883	119873283	-0.350419554	0.310472473	same
chr19	4452852	4453252	0.335496067	0.310663638	same
chr13	73014538	73014938	-0.348277157	0.310723411	same
chr1	43597070	43597470	-0.302193214	0.310840355	same
chr3	58306553	58306953	-0.318431514	0.310917501	same
chr9	74887053	74887812	-0.32559602	0.310933187	same
chr12	70367008	70367408	-0.321548478	0.310989485	same
chr1	212367828	212368228	-0.323931473	0.311034768	same
chr1	200738971	200739371	-0.320363476	0.311302861	same
chr3	179347479	179347879	-0.327703379	0.31132793	same
chr21	31343809	31344209	-0.325664564	0.311332059	same
chr17	8176146	8176546	-0.298545566	0.311344051	same
chr6	158704152	158704552	-0.327581353	0.311409688	same
chr7	140443855	140444255	-0.325401365	0.311601087	same
chr14	74294408	74294808	-0.332176664	0.311679979	same
chr9	134125457	134125857	0.347208875	0.311820604	same
chr3	113168340	113168740	0.322525477	0.312074709	same
chr15	88620925	88621325	-0.312540248	0.312224636	same
chr3	33095911	33096311	-0.344908539	0.312574852	same
chr1	155976840	155977240	-0.312842492	0.312696566	same
chr17	42017346	42017746	-0.289795548	0.313137419	same
chr6	158040241	158040641	0.280242868	0.313170416	same
chr7	23528540	23528940	-0.337228996	0.313474764	same
chr1	228781276	228781676	-0.267190939	0.31356081	same
chr22	20697425	20697825	-0.328594297	0.313577999	same
chr18	31658277	31658677	-0.321332216	0.31358798	same
chr9	75028304	75028704	-0.322076772	0.3136658	same
chr13	91592523	91592923	0.333524175	0.314103508	same
chr3	33114624	33115024	-0.328101043	0.314231627	same
chr20	30377169	30377569	-0.34544599	0.314264228	same
chr17	7280834	7281234	-0.288105669	0.314304597	same
chr19	55146818	55147218	-0.348374697	0.31430841	same
chr2	219245181	219245581	-0.30397446	0.314705331	same
chr12	131017350	131017750	0.337300927	0.314708673	same
chr7	155509614	155510014	-0.291060835	0.314831636	same
chr11	68009993	68010393	-0.334584797	0.314832678	same

chr21	42663152	42663552	0.322140749	0.314925077	same
chrX	69616166	69616566	-0.341890197	0.314938957	same
chr16	50203466	50203866	-0.340972024	0.314959522	same
chr6	85593890	85594290	-0.297500507	0.314997293	same
chr15	101578386	101578786	-0.292269565	0.315113559	same
chr9	36765771	36766171	-0.341626308	0.315132756	same
chr20	21303612	21304012	-0.339858627	0.315264247	same
chr15	63157171	63157571	-0.340206593	0.315270398	same
chr8	144200260	144200660	-0.345034974	0.315432481	same
chr10	42475523	42475923	-0.325390346	0.31549183	same
chr11	65096140	65096540	-0.317516049	0.315541921	same
chr1	26900178	26900578	-0.338866188	0.315658689	same
chr7	130152576	130152976	-0.337822962	0.315938706	same
chr1	218345047	218345447	-0.319899765	0.315987284	same
chr10	133144058	133144458	-0.33430622	0.31598775	same
chr19	57435251	57435651	-0.280154242	0.316145634	same
chr14	53953220	53953620	-0.342316863	0.316166431	same
chr19	46413326	46413726	-0.300837584	0.316384858	same
chr2	27322858	27323258	-0.273159844	0.31639708	same
chr9	35646667	35647067	-0.310726276	0.316425709	same
chr9	136842315	136842715	-0.249122178	0.316471471	same
chr11	102111874	102112274	-0.322765044	0.316694058	same
chr6	41545797	41546197	-0.321502654	0.316711643	same
chr9	5437623	5438023	-0.30877347	0.316835691	same
chr1	145960533	145960933	-0.320377566	0.31694688	same
chr1	32062712	32063112	-0.321686071	0.317016586	same
chr12	55646040	55646440	-0.322807544	0.317050203	same
chr17	32141958	32142358	-0.329216522	0.317242717	same
chr2	218834368	218834768	0.345923984	0.317271209	same
chr16	81614869	81615269	-0.33240268	0.317304931	same
chr14	103697742	103698142	-0.270476704	0.317401402	same
chr10	73252442	73252842	-0.288884843	0.317445273	same
chr6	149864377	149864777	-0.332005374	0.317451349	same
chr12	57342257	57342657	-0.340643237	0.317526664	same
chr11	120186902	120187302	-0.332059599	0.317613929	same
chr3	138082171	138082571	0.343894637	0.317645011	same
chr13	52309811	52310211	-0.34443629	0.317655006	same
chr14	24215566	24215966	-0.272862781	0.318208712	same
chr17	714673	715073	-0.321265961	0.318209747	same
chr9	93508241	93508641	-0.340152335	0.318249199	same
chr19	47863551	47863951	-0.347699951	0.318322963	same
chr3	48609474	48609874	-0.280987447	0.318325322	same
chr5	150217965	150218365	-0.327739622	0.31844275	same
chr13	99606080	99606480	-0.340338622	0.318647896	same
chr4	173369517	173369917	-0.298065499	0.318724212	same
chr12	76869205	76869605	-0.335446779	0.318820337	same
chr2	15591564	15591964	-0.286453333	0.318956136	same
chr19	14518127	14518527	-0.335018176	0.318980566	same
chr6	27865186	27865586	-0.317059472	0.319468745	same
chr3	47825387	47825787	-0.322454828	0.319506423	same
chr1	161972135	161972535	0.341422997	0.319691861	same
chr1	16513973	16514373	-0.326055617	0.31985981	same
chr4	171629757	171630157	-0.339695747	0.320028669	same
chr9	94259172	94259572	-0.305868577	0.320153203	same
chr20	58850713	58851113	-0.33360351	0.320430917	same
chr13	74210823	74211223	0.33359219	0.320498991	same
chr4	499092	499492	-0.320968458	0.320749365	same
chr19	47699415	47699815	-0.340119342	0.320860881	same
chrX	115898749	115899149	-0.308603312	0.321079237	same
chr17	2336347	2336747	-0.295401248	0.321081964	same
chr17	18682178	18682578	-0.345880762	0.321120646	same
chr3	50088759	50089159	-0.292485878	0.321135849	same
chr10	63866338	63866738	-0.340221534	0.321281225	same
chr9	25677472	25677872	-0.341339084	0.321299922	same
chr5	72308053	72308453	-0.293864752	0.321311375	same
chr5	415544	415944	-0.31923768	0.321390153	same
chr12	7933285	7933685	-0.334255787	0.321504492	same
chr20	37094984	37095384	-0.339605223	0.322220809	same
chr7	48035496	48035896	-0.337665171	0.322352507	same
chr16	87394239	87394639	-0.328660146	0.322530379	same
chrX	119468628	119469028	-0.307021385	0.322716788	same
chr2	152025938	152026338	-0.335779037	0.322832798	same
chr3	180912295	180912695	-0.331937035	0.322834752	same
chr14	35808515	35808915	-0.327596755	0.323069853	same
chr16	75464521	75464921	-0.309191401	0.323199226	same
chr1	209884634	209885034	0.341658311	0.323267512	same

chr6	142944414	142944814	-0.33371684	0.323344945	same
chr19	47706824	47707224	-0.336812818	0.323387921	same
chr2	70947763	70948163	-0.303728087	0.323425182	same
chr14	77033362	77033762	-0.32333638	0.323450737	same
chr18	33560856	33561256	0.336808108	0.32364459	same
chr3	142225263	142225663	-0.33700173	0.323769574	same
chr2	109029756	109030156	-0.343365571	0.323881375	same
chr16	87502693	87503093	-0.331056872	0.323890428	same
chr6	157273817	157274217	-0.319957503	0.32392104	same
chr13	73803685	73804085	0.315709547	0.323980317	same
chr12	130839316	130839716	-0.304900913	0.324010614	same
chr14	73851692	73852092	-0.300902771	0.324211174	same
chr16	75148356	75148756	-0.312514878	0.324213163	same
chr7	33904466	33904866	-0.339435337	0.324269299	same
chr19	13794905	13795305	-0.335906376	0.324429189	same
chr6	168157613	168158013	-0.300610357	0.324756438	same
chr9	125177090	125177490	-0.32523738	0.324921913	same
chr12	121513867	121514267	-0.29901276	0.324966213	same
chr14	74882669	74883069	-0.322002598	0.32499443	same
chr3	5018230	5018630	-0.335834305	0.325015826	same
chr9	100350312	100350712	0.323697745	0.325018473	same
chr1	93448834	93449234	-0.340171237	0.325221917	same
chr6	143060375	143060775	-0.322676922	0.325401955	same
chr6	87796425	87796825	-0.340039575	0.325425553	same
chr1	145885854	145886254	-0.304611262	0.325900931	same
chr8	97595120	97595520	0.33675675	0.325924061	same
chr12	54379598	54379998	-0.300852146	0.325937332	same
chr10	18627570	18627970	-0.313282349	0.326174338	same
chr15	37101474	37101874	-0.33067351	0.326218764	same
chr8	63124675	63125075	-0.316006607	0.32626344	same
chr1	198876382	198876782	-0.33905718	0.326679919	same
chr12	117127132	117127532	-0.263757967	0.326712676	same
chr7	146345775	146346175	-0.326384739	0.326717765	same
chr19	14450183	14450854	-0.285129935	0.32675904	same
chr1	19251426	19251826	-0.328006174	0.327091488	same
chr17	74850769	74851169	-0.296577879	0.32711836	same
chr4	88880962	88881362	-0.33247431	0.327161524	same
chr3	8963269	8963669	-0.323209482	0.327364459	same
chr1	46348056	46348456	-0.335499517	0.327400263	same
chr9	33290444	33290844	-0.265445511	0.327475659	same
chr11	107858923	107859323	-0.32530065	0.32768616	same
chr11	8192670	8193070	-0.314873559	0.32787937	same
chr13	68272973	68273373	-0.314136563	0.327905337	same
chr1	93345420	93345820	-0.317464041	0.327949863	same
chr7	148966839	148967239	-0.268677069	0.327952896	same
chr1	169106235	169106635	-0.320987272	0.328102178	same
chr3	47703676	47704076	0.333822017	0.328148881	same
chr13	65353354	65353754	-0.328625039	0.328235367	same
chr3	40477049	40477449	-0.31649397	0.328244672	same
chr11	117327786	117328186	-0.311321901	0.328494709	same
chr1	227767332	227767732	0.282560087	0.328551526	same
chr17	82660179	82660579	-0.320240876	0.328733499	same
chr7	100603688	100604088	-0.324251689	0.32874909	same
chr1	45570173	45570573	-0.318343483	0.328769283	same
chr11	129143440	129143840	-0.331060623	0.328847934	same
chr6	28349947	28350347	-0.329119159	0.328958496	same
chr2	118014044	118014444	-0.316240916	0.329087289	same
chr13	77737481	77737881	-0.265615428	0.329251887	same
chr1	205211353	205211753	-0.310780805	0.329328799	same
chr7	44885657	44886057	-0.306377823	0.32955722	same
chr6	161838589	161838989	-0.337221752	0.329822687	same
chr10	62804387	62804787	-0.286573253	0.329890081	same
chr17	75270356	75270756	-0.283729778	0.329943047	same
chr13	63801043	63801443	0.320976986	0.330075053	same
chr21	34510675	34511075	-0.332408172	0.330087497	same
chr17	42562132	42562532	-0.302022346	0.330159034	same
chr10	92290828	92291228	-0.312899411	0.330171725	same
chr6	136526477	136526877	-0.310621732	0.330205132	same
chr17	75601209	75601609	-0.275670315	0.330287862	same
chr19	35248457	35248857	-0.304435647	0.330306114	same
chr10	103918055	103918455	-0.302472207	0.330445473	same
chr16	2842491	2842891	-0.299541475	0.330563656	same
chr10	133231872	133232272	-0.334362111	0.330602927	same
chr17	76688585	76688985	-0.295231797	0.330610893	same
chrX	119574289	119574689	-0.283382341	0.330654333	same
chr19	49403879	49404279	-0.320969629	0.330704807	same

chr16	28212207	28212607	-0.32582047	0.330895076	same
chr10	31032077	31032477	-0.309350992	0.330968308	same
chr19	58183045	58183445	-0.336725979	0.330971314	same
chr6	26026646	26027046	-0.301066371	0.3309985	same
chr12	108823102	108823502	-0.309656159	0.331280178	same
chr17	48492806	48493206	-0.33426046	0.331465568	same
chr22	30289719	30290119	-0.296815837	0.331564294	same
chrX	77910326	77910726	0.336488967	0.331678402	same
chr17	74970650	74971050	-0.311603469	0.331814148	same
chr8	100847242	100847642	-0.308492028	0.332087913	same
chr12	46267369	46267769	-0.308285032	0.332205949	same
chr3	107241034	107241434	-0.326223516	0.3322485	same
chr19	48881563	48881963	-0.32927051	0.3323084	same
chr7	51366965	51367365	-0.311508818	0.332676369	same
chr17	68413640	68414040	-0.325989744	0.333031714	same
chr18	59358982	59359382	-0.317943411	0.333091605	same
chr12	56751887	56752287	-0.301574161	0.333154015	same
chr5	125043848	125044248	-0.333482419	0.333266785	same
chr17	16589157	16589557	0.334128378	0.333455341	same
chr6	27605576	27605976	-0.324737631	0.333504509	same
chr9	128818167	128818567	-0.267055315	0.333572679	same
chr4	61201816	61202216	-0.317438369	0.3337012	same
chr15	65401122	65401522	-0.318315194	0.33370313	same
chr6	138499043	138499443	-0.307605875	0.333814574	same
chr17	44708796	44709196	-0.317086176	0.33382781	same
chr3	14431945	14432345	0.325701577	0.334158079	same
chr7	27347066	27347466	-0.331218953	0.334227946	same
chr3	100709201	100709601	-0.318108293	0.334455978	same
chr17	28842668	28843068	-0.290576765	0.334557505	same
chr3	20041378	20041778	-0.30512965	0.334559028	same
chr2	95076840	95077240	-0.317783823	0.334630517	same
chr6	44128325	44128725	-0.299644004	0.334809553	same
chr1	1290156	1290556	0.311253193	0.334929446	same
chr19	50922363	50922763	-0.327833328	0.334998196	same
chr18	25261710	25262110	-0.330421752	0.335139455	same
chr5	181161123	181161523	-0.312093506	0.335317767	same
chr6	28124418	28124818	-0.311790911	0.335387239	same
chr1	15795166	15795566	-0.320493479	0.336010627	same
chr22	42553890	42554290	-0.313612906	0.336102881	same
chr3	146160524	146160924	-0.312376566	0.336169782	same
chr11	14359303	14359703	-0.317935411	0.336273791	same
chr2	203014519	203014919	-0.272679953	0.336505924	same
chr6	39229107	39229507	-0.332248567	0.336570585	same
chr13	63449341	63449741	-0.305132953	0.336582069	same
chr7	76166567	76166967	-0.301988785	0.336962937	same
chr14	104816386	104816786	-0.31850921	0.337175853	same
chr2	27890220	27890620	-0.294963474	0.337369482	same
chr11	67353354	67353754	0.285201709	0.337402691	same
chr19	5828241	5828641	-0.310082855	0.337764816	same
chr3	184354162	184354562	-0.322868743	0.337850172	same
chr11	17077690	17078090	-0.302335966	0.337998034	same
chr10	92591469	92591869	-0.311032704	0.338080701	same
chr12	24378454	24378854	-0.324941682	0.338104701	same
chr16	1343116	1343516	-0.25256047	0.338243167	same
chr7	43824656	43825056	-0.308873893	0.338558755	same
chrX	56995336	56995736	-0.314327854	0.338635467	same
chr18	23453088	23453488	-0.26207032	0.33919421	same
chr3	169812637	169813037	-0.312942866	0.339354341	same
chr12	56079374	56079774	-0.292551948	0.339526192	same
chr13	43787664	43788064	-0.328227556	0.33957582	same
chr7	157298647	157299047	-0.288460081	0.339618245	same
chr13	61903189	61903589	-0.287727748	0.33964256	same
chr19	57814590	57814990	-0.304925118	0.339790583	same
chr22	41941359	41941759	-0.269927723	0.339853822	same
chr19	49056138	49056538	-0.297882572	0.340094086	same
chr15	43122853	43123253	-0.318147378	0.340293874	same
chr21	45513641	45514041	-0.293319119	0.340419562	same
chr15	75921425	75921825	-0.329048357	0.340505029	same
chr1	225485380	225485780	0.287005312	0.340566097	same
chr2	59080398	59080798	-0.322135495	0.340642205	same
chr17	16535630	16536030	-0.293408314	0.340804586	same
chr22	31496526	31496926	-0.307591356	0.340853056	same
chr9	2570839	2571239	-0.313546196	0.34112222	same
chr9	123678214	123678978	-0.299381489	0.341694312	same
chrX	11759223	11759623	-0.315693026	0.341772915	same
chr12	49347241	49347641	-0.333222358	0.34177459	same

chr3	32947639	32948039	-0.323171311	0.341797325	same
chr6	79946387	79946787	-0.28269147	0.34196697	same
chr19	18323378	18323778	-0.321844468	0.341998059	same
chr6	137853609	137854009	-0.316372131	0.34202619	same
chr3	129279412	129279812	-0.327167514	0.342028221	same
chr3	173585438	173585838	-0.32763369	0.342082342	same
chr2	183032420	183032820	-0.312139373	0.342234694	same
chr22	41444372	41444772	-0.255738029	0.342306453	same
chr1	161117635	161118035	-0.277968789	0.342469383	same
chr4	106213548	106213948	-0.316307926	0.34299048	same
chr10	102479020	102479420	-0.283789922	0.343000088	same
chr10	86755703	86756103	-0.31670094	0.343187164	same
chr4	186726092	186726492	-0.324879704	0.3434168	same
chr5	129905958	129906358	-0.32742147	0.343527624	same
chr6	143537724	143538124	-0.320432386	0.343761901	same
chr7	151039197	151039597	-0.251789031	0.34395451	same
chr9	125189576	125189976	-0.311610982	0.344069602	same
chr12	132085167	132085567	-0.296093143	0.344177219	same
chr18	32140055	32140455	-0.299634112	0.344387607	same
chr14	74493014	74493414	-0.306260383	0.344826543	same
chr13	79338452	79338852	-0.320971602	0.344839442	same
chr7	5563251	5563651	-0.28977647	0.345116221	same
chr19	46766828	46767228	-0.324524308	0.345148645	same
chr16	21935006	21935406	-0.305035928	0.345223807	same
chr3	132659593	132659993	-0.281046006	0.345420121	same
chr16	58392451	58392851	-0.29864258	0.345458258	same
chr7	66844786	66845186	-0.281089714	0.345494213	same
chr1	17406154	17406554	-0.304503495	0.345694935	same
chr12	52003922	52004322	-0.317941969	0.345952286	same
chr3	75906485	75906885	-0.321936154	0.34603294	same
chr19	13921454	13921854	-0.324955315	0.346248297	same
chr14	64501678	64502078	-0.289502175	0.346381852	same
chr7	128409978	128410378	-0.307298429	0.346384814	same
chr6	100196547	100196947	-0.327647033	0.346452804	same
chr6	151389687	151390087	-0.29401381	0.346539941	same
chr8	144509762	144510162	-0.230315442	0.34661125	same
chr13	74306536	74306936	-0.301675292	0.346837374	same
chr5	102296262	102296662	-0.301355873	0.347080485	same
chr3	42042915	42043315	-0.317825493	0.347111056	same
chr12	64221963	64222363	-0.309102046	0.34726005	same
chr16	30953707	30954107	-0.228696987	0.347383278	same
chr6	158853176	158853576	-0.268339719	0.347445221	same
chr1	189149643	189150043	-0.306371767	0.347503903	same
chr19	48584115	48584515	-0.307262158	0.347582987	same
chr13	63351192	63351592	0.308094008	0.34760664	same
chr9	129139314	129139714	-0.300827095	0.347620113	same
chr21	36419925	36420325	-0.293745566	0.347807615	same
chr5	164422296	164422696	-0.324914454	0.347873604	same
chr1	109483414	109483814	-0.286509437	0.348155188	same
chr21	36260392	36260792	-0.306971038	0.348173944	same
chr10	133280559	133280959	-0.319308373	0.348209997	same
chr15	45279201	45279601	-0.310674566	0.348904897	same
chr6	42226699	42227099	-0.313615114	0.349085054	same
chr14	67414304	67414704	0.315582068	0.349829679	same
chr3	29393798	29394198	-0.317081065	0.349897034	same
chr17	48678413	48678813	-0.320686166	0.350013586	same
chr2	66567009	66567409	-0.315568936	0.350054087	same
chr6	107115980	107116380	-0.317718335	0.350107976	same
chr3	126081861	126082261	-0.31559209	0.350218295	same
chr14	73457637	73458037	-0.31958102	0.350306525	same
chr1	59926595	59926995	-0.29243023	0.350363658	same
chr5	173753389	173753789	-0.315511235	0.350402554	same
chr6	27295770	27296170	-0.314849284	0.350472772	same
chr16	14325951	14326351	-0.317234408	0.350473956	same
chr1	64349040	64349440	0.299016848	0.350489971	same
chrX	15335401	15335801	-0.289773454	0.350527304	same
chr22	43185879	43186279	-0.294488801	0.350911641	same
chr16	4613550	4613950	-0.260252413	0.350914646	same
chr17	39687773	39688173	-0.297353253	0.351005649	same
chr1	109740240	109740640	-0.289795489	0.35109598	same
chr10	103193254	103193654	-0.322072577	0.351190711	same
chr20	39050226	39050626	-0.318495327	0.351316676	same
chr20	62156772	62157172	-0.28716036	0.35136983	same
chr6	37018094	37018494	-0.281947971	0.35150251	same
chr1	173476369	173476769	-0.3136942	0.351613217	same
chr22	39328217	39328617	0.320770515	0.35204818	same

chr13	65784088	65784488	-0.315447839	0.35216419	same
chr4	75573538	75573938	-0.307358304	0.35234875	same
chr10	46030332	46030732	-0.321818235	0.352364173	same
chr2	132668403	132668803	-0.321626464	0.352413526	same
chr14	54773466	54773866	-0.304120767	0.352715455	same
chr16	69112725	69113125	-0.302382532	0.352942871	same
chr3	69691207	69691607	-0.317941395	0.35301333	same
chr17	63432656	63433056	-0.307087659	0.353053497	same
chr1	58250274	58250674	-0.322684559	0.353422328	same
chr15	74382460	74382860	-0.311133014	0.353435048	same
chr17	44919878	44920278	-0.28741514	0.353508514	same
chr17	77088623	77089023	-0.285399828	0.353708829	same
chr1	84298865	84299265	-0.294398554	0.353741819	same
chr21	36990184	36990584	-0.301112055	0.353744127	same
chr1	197775985	197776385	-0.300698733	0.353860924	same
chr11	73289313	73289713	-0.28836436	0.353923781	same
chr6	3226863	3227263	-0.309400161	0.354395771	same
chr2	202032907	202033307	-0.29233486	0.3544162	same
chr17	76292309	76292709	-0.297552326	0.354572789	same
chr10	86585488	86585888	-0.303657909	0.35471226	same
chrX	153651669	153652069	-0.292859024	0.354964486	same
chr7	149854766	149855166	-0.281911255	0.355188537	same
chr19	57916488	57916888	-0.294184993	0.355236904	same
chr3	125990309	125990709	-0.319774259	0.355525944	same
chr22	21001820	21002220	-0.240142745	0.355539433	same
chr19	21358811	21359211	-0.30271309	0.35572894	same
chr15	88586502	88586902	-0.306045694	0.355755318	same
chr15	89017478	89017878	-0.318329899	0.35578787	same
chr1	177946011	177946411	-0.319001901	0.355956785	same
chr1	232037721	232038121	0.292671753	0.356125857	same
chr1	64184049	64184449	-0.279253911	0.35620874	same
chr8	142623912	142624312	-0.307446752	0.35631533	same
chr17	8016416	8016816	-0.314139232	0.356952404	same
chr10	12350503	12350903	-0.31634361	0.357039222	same
chr14	101809880	101810280	-0.289371486	0.357052326	same
chr11	31812468	31812868	-0.284213529	0.357157619	same
chrX	41442222	41442622	-0.302089894	0.357201557	same
chr7	135260386	135260786	-0.30198007	0.357495772	same
chr11	47642354	47642754	-0.293754376	0.358011048	same
chr2	46643000	46643400	-0.280559709	0.358051302	same
chr19	55280056	55280456	-0.275515523	0.358261762	same
chr17	14309183	14309583	-0.295769594	0.35826178	same
chrX	86148138	86148538	-0.282583565	0.358898138	same
chr15	76931230	76931630	-0.309769412	0.359126593	same
chr13	67323906	67324306	-0.284069746	0.359140061	same
chr19	48837024	48837424	-0.290855685	0.359320717	same
chr12	122087620	122088020	-0.300865723	0.35942649	same
chr17	55731898	55732298	-0.31387576	0.359444614	same
chr20	41316916	41317316	-0.308248569	0.359681067	same
chr1	214281936	214282336	-0.299781492	0.359828535	same
chr11	46369501	46369901	-0.263196004	0.359924138	same
chr19	56538387	56538787	-0.30601592	0.359944592	same
chr16	88737458	88737858	0.288078921	0.360203904	same
chr15	98428326	98428726	-0.277052396	0.360218353	same
chr7	151616431	151616831	-0.306502976	0.360317358	same
chr19	11424992	11425392	0.30372745	0.360352935	same
chr4	126583470	126583870	-0.311172664	0.360450697	same
chr15	86165473	86165873	-0.316575169	0.360552694	same
chr7	65373692	65374092	-0.277523095	0.36147702	same
chr14	70359391	70359791	-0.29993086	0.361709857	same
chr6	57274024	57274424	-0.318333464	0.361711995	same
chr7	74079191	74079591	-0.261608037	0.361763254	same
chr14	52949928	52950328	-0.309337629	0.362051596	same
chr17	81675743	81676143	-0.272618717	0.362221474	same
chr7	118183957	118184357	-0.301416781	0.362521463	same
chr14	74954129	74954529	-0.304516114	0.362668712	same
chr16	53323	53723	-0.260308156	0.362701791	same
chr13	69924310	69924710	0.311912642	0.362732736	same
chr19	12712116	12712516	-0.293264586	0.362891339	same
chr18	48997958	48998358	-0.299226909	0.363105976	same
chr1	223531899	223532299	-0.309842138	0.363120837	same
chr4	123398691	123399091	-0.290128481	0.363462186	same
chr2	155966011	155966411	-0.300449233	0.363962826	same
chr6	117908227	117908627	-0.311359035	0.363964054	same
chrX	75188624	75189024	-0.305379458	0.363999667	same
chr11	62851422	62851822	-0.272684431	0.364024483	same

chr13	45418132	45418532	-0.269263084	0.364108448	same
chr4	86560834	86561234	-0.313988846	0.364140515	same
chr17	81976656	81977056	-0.277031279	0.364200377	same
chr9	4662784	4663184	-0.299024795	0.364218957	same
chr1	223133601	223134001	-0.276092938	0.364292206	same
chr1	202135487	202135887	-0.293002975	0.364373113	same
chr12	121443308	121443708	-0.267946432	0.364407302	same
chr6	52839551	52839951	-0.303260916	0.364528799	same
chr13	65472466	65472866	-0.280386425	0.364541022	same
chr19	16185405	16185805	-0.289708304	0.364740913	same
chr3	71542657	71543057	-0.306042436	0.365610323	same
[truncated: 3,850,638 more chars]
